# Cell-free supernatant of *Lactobacillus gasseri* 1A-TV shows a promising activity to eradicate carbapenem-resistant *Klebsiella pneumoniae* colonization

**DOI:** 10.3389/fcimb.2024.1471107

**Published:** 2024-12-03

**Authors:** Gaia Vertillo Aluisio, Maria Lina Mezzatesta, Viviana Cafiso, Renata Scuderi, Stefania Stefani, Maria Santagati

**Affiliations:** Medical Molecular Microbiology and Antibiotic Resistance Laboratory (MMARLab), Department of Biomedical and Biotechnological Sciences (BIOMETEC), University of Catania, Catania, Italy

**Keywords:** *Lactobacillus gasseri*, ESBL, cell-free supernatant, carbapenem-resistant *K. pneumoniae*, antimicrobial activity

## Abstract

**Background:**

The use of beneficial bacteria like *Lactobacillus* spp. is a potential innovative approach to fight antibiotic-resistant pathogens. *Klebsiella pneumoniae* is one of the most concerning multi drug-resistant (MDR) pathogens, and its ability to colonize the human gut is considered to be the main reason for recurrent infections in critically ill patients.

**Methods:**

In this study, *Lactobacillus gasseri* 1A-TV, already described for its probiotic activity, was characterized at the genomic level. Moreover, its cell-free supernatant (CFS) was tested for antimicrobial activity against extended-spectrum β-lactamase (ESBL)- and carbapenemase (KPC)-producing *K. pneumoniae* clinical isolates.

**Results:**

Whole-genome sequencing showed that the *L. gasseri* 1A-TV genome was of 2,018,898 bp in size with 34.9% GC content, containing 1,937 putative protein coding sequences, 55 tRNA, and 4 rRNA detected by RAST and classified in 20 functional groups by Cluster of Orthologous Genes (COG). BAGEL4 (BActeriocin GEnome minimal tooL) and the antiSMASH 7.0 pipeline identified two bacteriocin biosynthetic gene clusters (BBGCs), namely, BBGC1 that comprises two class IIc bacteriocins including gassericin A-like bacteriocin, and BBGC2 carrying the class III bacteriocin helveticin J. Strikingly, 1A-TV CFS inhibited the growth of all *K. pneumoniae* isolates only after 8 h of incubation, showing a bactericidal effect at 24 h and interfering, even at lower concentrations, with the biofilm production of biofilm-producer strains independently of a bactericidal effect. NMR analysis of CFS identified and quantified several metabolites involved in carbohydrate metabolism and amino acid metabolism, and organic acids like ethanol, lactate, acetate, and succinate. Finally, *in vitro* assays of 1A-TV showed significant co-aggregation effects against carbapenem-resistant *K. pneumoniae*, namely, strains 1, 2, 3, and 7.

**Conclusions:**

Our findings highlight the antimicrobial activity of 1A-TV as a probiotic candidate or its CFS as a natural bioproduct active against MDR *K. pneumoniae* strains, underlining the importance of novel therapeutic strategies for prevention and control of ESBL- and carbapenemase-producing *K. pneumoniae* colonization.

## 1 Introduction

The global burden of antimicrobial resistance has become a major health concern (Aslam et al., 2018; Antimicrobial Resistance Collaborators, 2022) and *Klebsiella pneumoniae* is one of the most concerning multidrug -resistant (MDR) pathogens involved in hospital-acquired infections thanks to its wide resistome and mobilome. Colonization and carriage of carbapenem-resistant *K. pneumoniae* (CRKP) are considered to be the main reason for recurrent infections in critically ill patients (Kontopoulou et al., 2019); therefore, decolonization of CRKP could reduce the secondary and recurrent infections. *K. pneumoniae* biofilm formation on catheters and internal devices of hospitalized patients can also be another agent of colonization in different districts, invasive infections, and treatment failure (Wang et al., 2020). In fact, extended-spectrum β-lactamase (ESBL)- and carbapenemase (KPC)-producing *K. pneumoniae* has a greater ability to produce biofilms compared to non-ESBL- and KPC-producing strains, conferring a further advantage to the MDR strains in terms of persistence in the host and resistance of the effect of the antibiotics (Sabença et al., 2023).

In light of the above, several studies have reported beneficial effects exerted by novel strategies based on probiotics as preventive therapy against MDR pathogens, in order to guarantee a better outcome to hospitalized and immunocompromised patients. The use of probiotics could be valuable to re-establish a healthy microbiota and ultimately homeostasis (Davison and Wischmeyer, 2019); in fact, dysbiosis of the microbiota, often exacerbated by antibiotic treatment, is one of the causes of MDR pathogen colonization (Alagna et al., 2020), whereas a balanced state is necessary to prevent the onset of infections and other non-infectious pathological conditions (Gomaa, 2020). Moreover, the health-promoting effects are due to not only the viability of probiotics but also certain biomolecules produced by probiotics such as postbiotics, which could be useful in preventing the risks of using live bacteria while still exerting their beneficial effects to the host (Aggarwal et al., 2022). Recently, the International Scientific Association of Probiotics and Prebiotics (ISAPP) defined postbiotics as “preparation of inanimate microorganisms and/or their components that confers a health benefit on the host” (Salminen et al., 2021). Postbiotics are metabolic and/or structural microbial compounds such as cell-free supernatants (CFSs), bacterial lysates, vitamins, cell wall fragments, peptides, and teichoic acid that have the ability to confer health-promoting effects to the host.

In this context, lactobacilli belong to the lactic acid bacteria (LAB) group are promising candidates to human health; thus, they are often employed for their beneficial effects (Pace et al., 2015; Seddik et al., 2017). *Lactobacillus* antimicrobial activity is exerted through different mechanisms, such as the stimulation of host immune system, competition for binding sites and nutrients with pathogens, and the production of inhibitory compounds like organic acids, hydrogen peroxide, and bacteriocins. The antagonistic activity of lactobacilli has been demonstrated to act against a wide spectrum of pathogens, suggesting a promising future for alternative strategies in fighting infection (Chen et al., 2021; Scillato et al., 2021).

In our previous study, we firstly characterized the strain *Lactobacillus gasseri* 1A-TV for its excellent ability to interfere and co-aggregate with urogenital pathogens and the 1A-TV CFS also exerted a strong antimicrobial activity, supported by the production of D- and L-lactic acid, hydrogen peroxide, and bacteriocin production (Scillato et al., 2021).

Considering the potential use of *L. gasseri* 1A-TV in the prevention and/or treatment of infections associated with MDR *K. pneumoniae*, this study is focused on the antimicrobial effects exerted by 1A-TV CFS as a postbiotic against ESBL- and KPC-producing *K. pneumoniae*.

In order to address this, we used the whole-genome sequencing of *L. gasseri* 1A-TV combined with a genome mining tool to identify biosynthetic gene clusters (BGCs); likewise, we investigated the antimicrobial activity of 1A-TV against ESBL- and KPC-producing *K. pneumoniae* by using killing curve assay and we evaluated its anti-biofilm and co-aggregating ability versus the pathogen strains.

## 2 Materials and methods

### 2.1 Bacterial strains and culture conditions

*L. gasseri* 1A-TV was grown in de Man Rogosa and Sharpe (MRS) broth and agar plates (Oxoid, Basingstoke, UK) at 37°C for 48 h in anaerobic conditions, using the GasPak™ EZ Anaerobe Pouch System (BD, New Jersey, USA).

In this study, we used seven *K. pneumoniae* clinical strains from our microbial bank at the MMARLab, previously characterized for their MDR profiles, and selected for their clinical and epidemiological characteristics (**Table 1**) (Gona et al., 2011, Gona et al., 2019; Mezzatesta et al., 2011). A non-resistant strain was added as control, Kbp7. They comprised the following: (i) Two KPC-producing strains belonging to ST258, isolated from hospitalized patients in two different hospitals in Catania as previously described (Mezzatesta et al., 2011); these strains were isolated from central venous catheter for patient 2 (renamed Kbp1) and from bloodstream for patient 8 (Kbp4 in this study). (ii) Two co-producing NDM-OXA-48 KP belonging to ST101, strain KP730 and KP723 as previously reported (Gona et al., 2019), renamed Kbp2 and Kbp3, respectively; both were isolated from patients of the neonatal intensive care unit of the University Hospital of Catania and belonged to two different epidemiological clusters by the Core Genome MLST (Kbp2 cluster A, Kbp3 cluster C). (iii) Two ESBL-producing strains ST307, isolated from patients 2 and 6 as previously reported (Gona et al., 2011) and named Kbp5 and Kbp6, respectively; Kbp 5 and Kbp6 were responsible for severe urinary tract infections in the renal transplant unit. MLST for *K. pneumoniae* isolates was performed as previously described (Mezzatesta et al., 2011), and STs were assigned by the MLST Pasteur website (http://www.pasteur.fr/mlst). In addition, minimum inhibitory concentrations (MICs) of amikacin and colistin were performed by the microdilution method interpreted according to EUCAST guidelines v.14.0. (https://www.eucast.org). Fosfomycin MIC was performed by the agar dilution method described by CLSI. All ESBL- and KPC-producing *K. pneumonia*e were also resistant to colistin. All strains were grown on MacConkey agar (Oxoid, Basingstoke, UK) and aerobically incubated at 37°C overnight.

**Table 1:** Molecular characterization and antibiotic resistance profile of *K. pneumoniae* clinical isolates described in this study.

*Indicator strains ID*	*Specimen*	*ST*	*Carbapenemase gene*	*ESBL gene*	*Other resistances*	*Reference*
***Kbp1***	CVC	258	*bla*_KPC-3_	*bla*_SHV-11_, *bla*_TEM-1_, *bla*_oxa-9_	AK, CIP, COL, FOS	Mezzatesta et al., 2011
***Kbp2***	Urine	101	*bla*_NDM-1_ and *bla*_oxa-48_	*bla*_CTX-M-15_, *bla*_SHV-28_, *bla*_TEM-1_, *bla*_oxa-9_	AK, GM, CIP, COL, FOS, RIF	Gona et al., 2019
***Kbp3***	Gastric aspirate	101	*bla*_NDM-1_ and *bla*_oxa-48_	*bla*_CTX-M-15_, *bla*_SHV-28_, *bla*_TEM-1_, *bla*_oxa-9_	AK, GM, CIP, COL, FOS, RIF	Gona et al., 2019
***Kbp4***	Bloodstream	258	*bla*_KPC-3_	*bla*_SHV-11_, *bla*_TEM-1_, *bla*_oxa-9_	AK, CIP, COL, FOS	Mezzatesta et al., 2011
***Kbp5***	Urine	307	-	*bla*_TEM-1_, *bla_CTX_*_-M-15_	COL, FOS	Gona et al., 2011
***Kbp6***	Bloodstream	307	-	*bla*_TEM-1_, *bla_CTX_*_-M-15_	COL, FOS	Gona et al., 2011
***Kbp7***	Feces	-	-	-	-	-

AK, amikacin; COL, colistin; CIP, ciprofloxacin; FOS, fosfomycin; GM, gentamicin; RIF, rifampicin; CVC, central venous catheter.

### 2.2 Whole-genome sequencing, assembly, and annotation

*L. gasseri* 1A-TV genomic DNA was extracted using the PureLink™ Genomic DNA Mini Kit (Thermo Fisher Scientific, USA) according to the manufacturer’s instructions, then it was quantified by a Qubit 2.0 fluorometer (dsDNA HS assay, Invitrogen, USA) and quality assessed by a Nanodrop2000 Spectrophotometer (Thermo Fisher Scientific, USA). Genomic libraries were prepared with a Nextera XT DNA Library Prep Kit (Illumina, USA) and sequenced by the Illumina MiSeq platform following the manufacturer’s protocol (Ravi et al., 2018). The raw data were provided as paired-end reads (2 × 250 Read Length) with a 258× coverage and their quality was checked using Trimmomatic (Bolger et al., 2014), while the genome was assembled by SPAdes v3.14.0 through the MEGA annotator pipeline (Bankevich et al., 2012). The genome annotation was performed with Prokka v. 1.13 (Seemann, 2014) (**Supplementary Data Sheet S1**), whereas rRNA and tRNA gene predictions were performed with RNAmmer v1.2 (Lagesen et al., 2007) and tRNAscan-SE v1.21, respectively (Lowe and Eddy, 1997). Rapid annotation using subsystem technology (RAST) was also applied for microbial genome annotation to identify different subsystems in the genome defined as “a set of functional roles” connected to specific genes of the available annotated genome (Aziz et al., 2008). Furthermore, the database of Cluster of Orthologous Genes (COG) was used for a summary of functional annotation (Galperin et al., 2021).

### 2.3 Genomic data accession number

*The L. gasseri* 1A-TV genomic reads have been deposited in the National Center for Biotechnology Information (NCBI) SRA database under SUB14624849, with BioProject accession number PRJNA1140194.

### 2.4 *In silico* prediction of bacteriocin biosynthetic gene clusters

Microbial genome mining was obtained using the antiSMASH 7.0 pipeline (antibiotic and Secondary Metabolite Analysis SHell) to predict classic and novel BGCs coding for secondary metabolites (Blin et al., 2023) and BAGEL4 (BActeriocin GEnome minimal tooL) to identify ribosomal synthesized and post-translationally modified peptides (RiPPs) (van Heel et al., 2018). The amino acid sequences of the core peptides identified within the BGCs were further analyzed by the CD search tool.

### 2.5 Determination of antimicrobial activity

The inhibitory activity of *L. gasseri* 1A-TV was firstly tested against seven *K. pneumoniae* strains shown in **Table 1**, using a deferred antagonism test, an assay typically used to investigate the production of bacteriocins and then quantified by the agar spot test (Siroli et al., 2017; Scillato et al., 2021). For the agar spot assay, 5 µL of 1A-TV broth cultures was spotted on the surface of MRS soft agar plates (1.2%) and incubated anaerobically for 48 h at 37°C. An overnight culture of indicator strains (100 μL; approximately 10^7^ CFU/mL) was mixed with Brain Heart Infusion soft agar (0.7%) (Oxoid, Basingstoke, UK) and poured over the plate where lactobacilli were spotted, as previously described (Scillato et al., 2021). Antagonistic activity was assessed by measuring diameters of inhibition zones and expressed as follows: no inhibition (−); diameter between 1 and 3 mm (+); diameter between 3 and 6 mm (++); diameter between 6 and 10 mm (+++); diameter > 10 mm (++++).

### 2.6 Auto-aggregation and co-aggregation assays

Auto-aggregation and co-aggregation activities were determined using our previous protocol with some modifications (Scillato et al., 2021; Vertillo Aluisio et al., 2022). Overnight cultures of *L. gasseri* 1A-TV and *K. pneumoniae* were centrifuged at 3,000 *g* for 15 min at 4°C, and the pellets were washed twice and resuspended in phosphate buffer saline (PBS) (Sigma-Aldrich) to obtain a final concentration of 10^7^ CFU/mL. For auto-aggregation assay, 4 mL of each suspension was incubated at room temperature with a shaker at 200 rpm, whereas for the co-aggregation assay, 2 mL of 1A-TV and 2 mL of each indicator suspension were mixed and incubated in the same conditions. The optical density of bacterial suspensions was measured at 600 nm at T_0_ and after 5 h of incubation (T_5_). Suspensions were dispensed in a 96-well plate at T_0_ and T_5_, and OD_600_ readings were attained with a microplate reader (BioTek Synergy™ HTX). For T_5_ readings, each sample was centrifuged at 650 g for 2 min to precipitate aggregated bacteria before dispending the 96-well plate.

Auto-aggregation percentage was then calculated as:


$$\text{Auto‐aggregation}\%=(1-\frac{{\text{AT}}_{5}}{{\text{AT}}_{0}})\times 100$$

Co-aggregation percentage was calculated as:


$$\text{Co‐aggregation}\%=(\frac{{\text{AT}}_{0}-{\text{AT}}_{5}}{{\text{AT}}_{5}})\times 100$$

where AT_0_ is the absorbance value at time 0, and AT_5_ is the absorbance value after 5 h of incubation.

The co-aggregation percentage of *L. gasseri* 1A-TV with *K. pneumoniae* has been considered significant when it is higher than auto-aggregation percentage of each pathogenic strain. Each assay was performed in triplicate.

### 2.7 Cell-free supernatant preparation

CFS of *L. gasseri* 1A-TV was prepared as previously described (Scillato et al., 2021). 1A-TV was inoculated in MRS broth at 37°C for 48 h, then the broth culture was centrifuged at 7,000 *g* for 30 min at 4°C and the supernatant was filtered with a 0. 22-µm membrane. pH values of CFS were measured by a pH meter (pH 50+DHS Benchtop pH Meter).

### 2.8 CFS ^1^H-NMR analysis

^1^H-NMR analysis was performed to determine the metabolic profile of *L. gasseri* 1A-TV CFS. 1A-TV CFS (630 μL; OD_600_ ≈ 1.6) was added to 70 μL of a buffer solution, pH 7.4 [1.5 M K_2_HPO_4_, 100% (v/v) D_2_O, NaN_3_ 2 mM, TMSP 5.8 mM]. The final mix (600 μL) was used for the acquisition of the NMR spectra. This analysis was performed using a Bruker IVDr 600 MHz (Bruker BioSpin) operating at the Larmor proton frequency of 600.13 MHz, equipped with a 2H PATXI H/C/decoupling probe 5 mm N and an automatic refrigerated sample changer (SampleJet). The temperature was adjusted to 300 ± 0.1 K with a BTO 2000 thermocouple. The signals were assigned by comparing their chemical shift and multiplicity with the Chenomx software data bank (Chenomx Inc., Canada, ver 8.02). To estimate the reproducibility, 1A-TV CFS NMR analysis was performed three times and MRS free was used as control.

### 2.9 *In vitro* antimicrobial activity of *L. gasseri* 1A-TV CFS

A time-killing assay was performed to evaluate the *in vitro* inhibitory activity of *L. gasseri* 1A-TV CFS against *K. pneumoniae* strains as previously described (Scillato et al., 2021). The kinetic growth of indicator strains in the presence of CFS was tested at different time intervals: 0, 4, 6, 8, and 24 h; viable microbial cell counts (CFU/mL) on Mueller Hinton (MH) agar (Oxoid, Basingstoke, UK) were determined according to the Clinical and Laboratory Standard Institute guidelines (7.17.2 Time-Kill Assay 7.17 tests to assess bactericidal activity, 2024). For the assay, *K. pneumoniae* indicators were used at 3 × 10^5^ CFU/mL in 10 mL of broth containing 5 mL of cation-adjusted Mueller Hinton broth (BD, New Jersey, USA) and 5 mL of CFS (1:1 ratio). The control growth of indicator strains was assessed by inoculating 10 mL of fresh medium containing MH-MRS broth (1:1). The CFU/mL values of each indicator strain were recorded as log_10_ reduction of the total count of CFU/mL in the original inoculum and the bactericidal activity was defined as a reduction of at least 99.9% (≥ 3 log_10_) (approved guideline M26-A, CLSI). This experiment was repeated in triplicate.

### 2.10 Measurement of bactericidal activity of CFS

The sensitivity of the CFS to enzymatic activity was assayed by trypsin (E. C.3.4.21.4, type II) and proteinase K (E. C. 3.4.21.64) at pH 7.5 (100 mM Tris-HCl buffer). Aliquots of the CFS were incubated (1:1 v/v) with enzyme solutions (1 mg/mL) and their respective controls at 37°C for 2 h under aerobic conditions (Scillato et al., 2021). After proteolytic enzyme treatments, the antibacterial activity of the CFS was performed by antagonism experiments in 96-well plates using the indicator strains at 3 × 10^5^ CFU/mL. After incubation for 24 h at 37°C, the bactericidal effects were estimated by a turbidimetric method with Microplate Reader (BioTek Synergy™ H1) system using OD_600_ for bacterial strains. All experiments were repeated three times.

### 2.11 *K. pneumoniae* biofilm formation

The crystal violet staining method was used to test biofilm production of *K. pneumoniae* indicators as previously described with some modifications (Raras et al., 2019; El-Mokhtar et al., 2020). *K. pneumoniae* ATCC 700603 was used as control for the biofilm production (Ochońska et al., 2021).

Briefly, overnight cultures of *K. pneumoniae* in LB broth were diluted 1:100 and dispensed in a 96-well plate (200 µL per well). After 24 h of incubation at 37°C, the plate was washed thrice with 200 μL of distilled water and air-dried. Then, 100 μL of 0.2% crystal violet was added to each well, incubated for 20 min, and then discarded. The plate was washed thrice with distilled water, air-dried, then 200 μL of 95% ethanol (Sigma-Aldrich) was added to each well. After 30 min of incubation, the intensity of crystal violet was measured at 570 nm using a microplate reader (BioTek Synergy™ HTX). Based on OD_570_ values, the isolates with OD_570_< 0.120 were considered as weak biofilm producers, those with OD_570_ 0.120–0.240 had intermediate biofilm formation ability, and isolates with OD_570_ > 0.240 were classified as strong biofilm producers, as previously described (Maldonado et al., 2007).

### 2.12 Biofilm inhibition assay

To investigate the effect of *L. gasseri* 1A-TV CFS on *K. pneumoniae* biofilm production, only strong biofilm producers were used as indicators. The assay was performed in a 96-well plate as aforementioned with the addition of *L. gasseri* 1A-TV CFS at scalar concentrations starting from a 1:1 ratio (50% of CFS), then the plate was incubated for 24 h at 37°C. To estimate inhibition of biofilm formation with CFS treatment, the following formula was applied: =


$$\text{Percentage of biofilm reduction}=\frac{\left(\text{C }–\text{ B})\;-\;(\text{T}-\text{B}\right)}{\left(\text{C}-\text{B}\right)}\times 100$$

where C denotes the OD_570_ values of wells without CFS treatment, T represents the OD_570_ values of treated wells, and B indicates the OD_570_ values of negative control, as previously described (El-Mokhtar et al., 2020).

To evaluate whether the antibiofilm effect was simultaneously associated with the bactericidal activity of 1A-TV CFS, the same assay was performed in another 96-well plate without crystal violet staining to obtain viable microbial cell counts (CFU/mL). After 24 h of incubation, the cultures were removed from each well, serially diluted and plated on MH agar for determination of viable microbial cell counts. All experiments were performed in at least three independent assays.

### 2.13 Statistical analysis

Statistical analyses were performed by using GraphPad Prism 8 software (GraphPad Software Inc., San Diego, CA, USA), and results were expressed as mean ± standard deviation (SD) of three independent experiments.

For the co-aggregation assays, ANOVA with Fisher’s least significant difference (LSD) test was applied to determine significant differences. For time-killing assays, the two-way ANOVA test with the Geisser–Greenhouse correction was applied to compare growth curves in treated and untreated conditions. For biofilm inhibition assays, two-way ANOVA with multiple comparisons was used. In all cases, *p*-value was expressed as follows: * *p*< 0.05, ** *p*< 0.01, and *** *p*< 0.001.

## 3 Results

### 3.1 Genome annotation and genome mining for biosynthetic gene clusters

**Figure 1:**
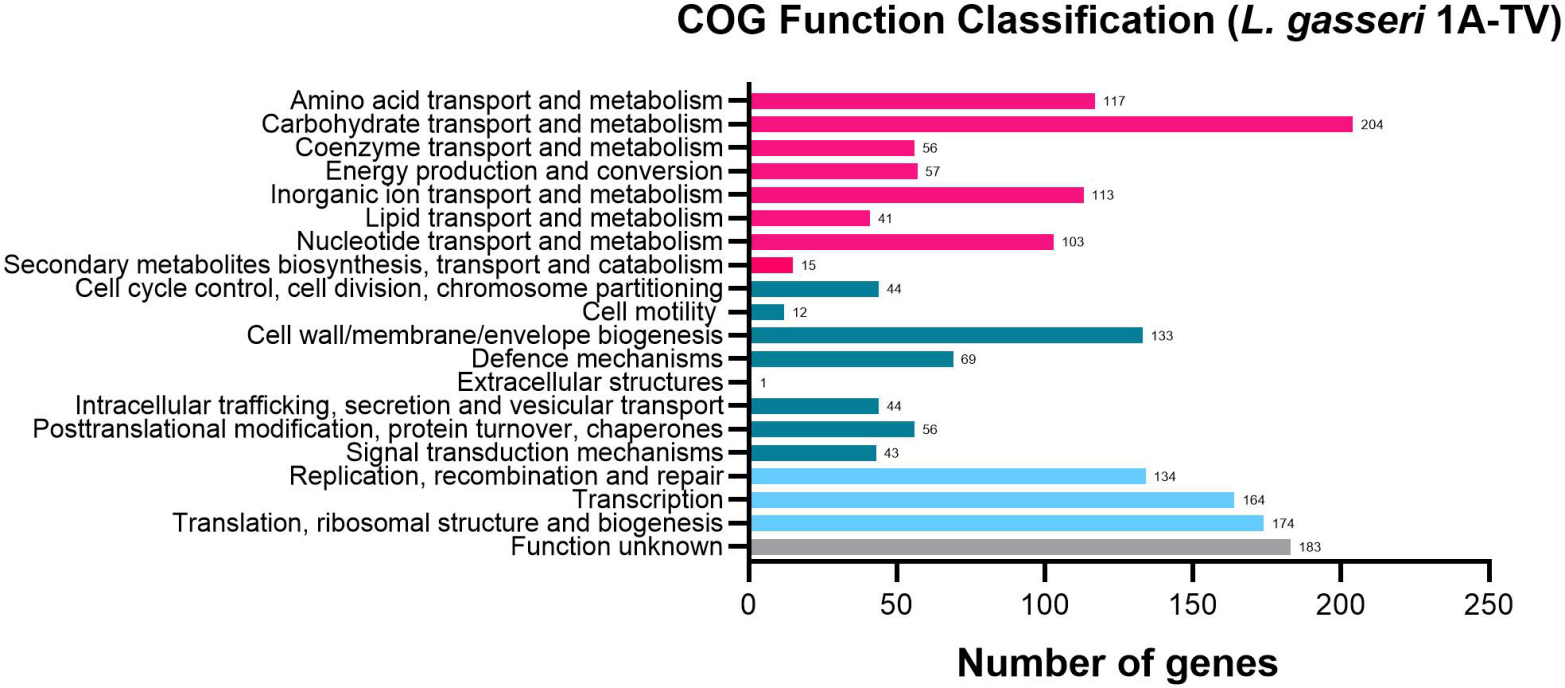
1A-TV genome analyzed by Cluster of Orthologous Genes (COG) and divided into functional categories: in red, the genes linked to metabolism; in dark green, the cellular functions; and in light blue, the genes involved in information through clusters of orthologous genes (https://www.ncbi.nlm.nih.gov/research/cog-project/).

**Figure 2:**
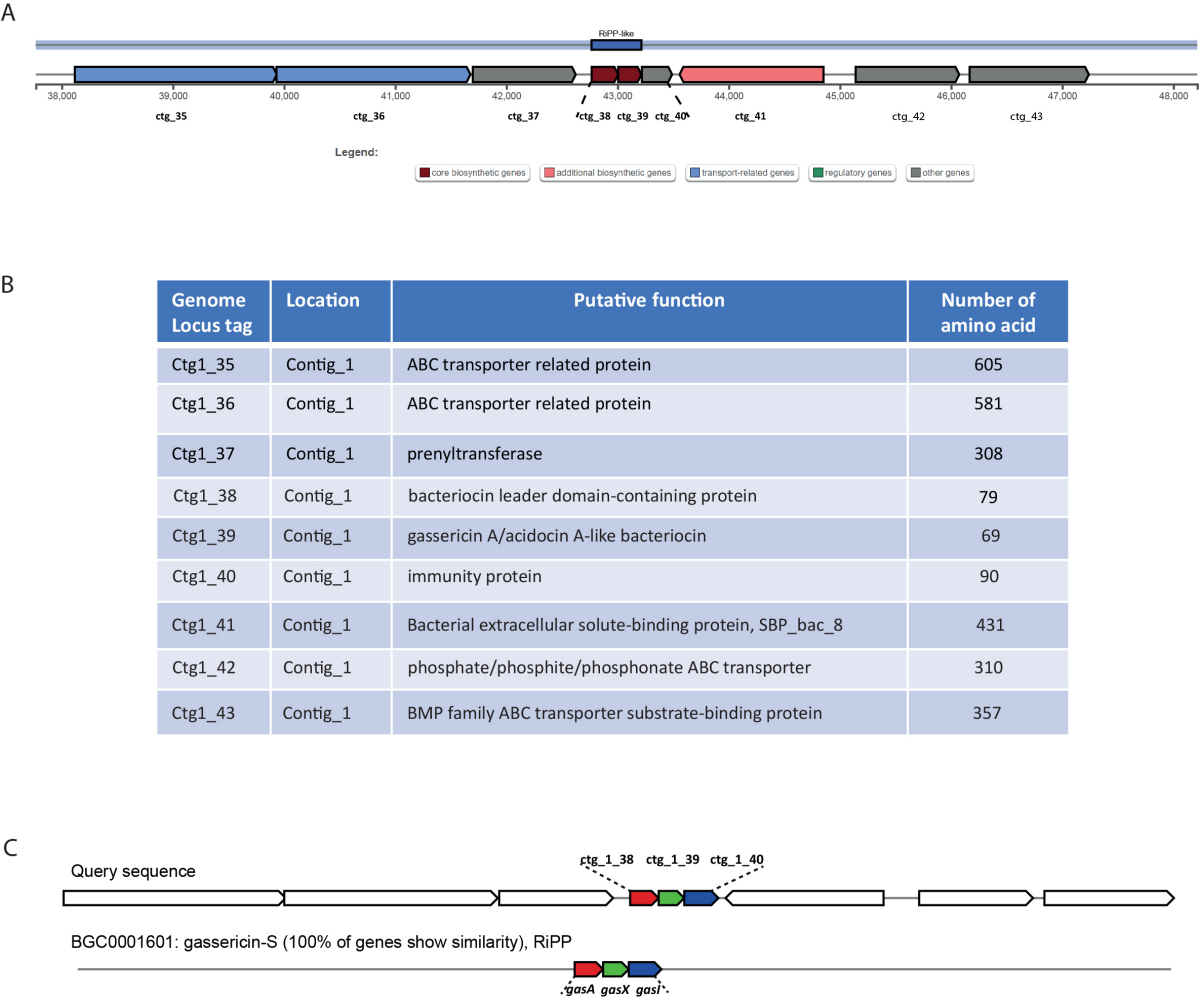
**(A)** Genetic map of the bacteriocin cluster BBGC1 identified by antiSMASH. The bacteriocin cluster comprises nine putative proteins, of which six are involved in the bacteriocin biosynthetic process, ctg1_38 and ctg1_39 encoding for two bacteriocins belonging to class IIc. **(B)** Genetic organization of cluster BBGC1, including nine locus tags with their putative function. **(C)** Comparison between 1A-TV BBGC1 and gassericin-S BGC1 in *Lactobacillus gasseri* carrying *gasA*, *gasX*, and *gasI* genes.

The whole-genome sequencing of *L. gasseri* 1A-TV showed a single chromosome of 2,018,898 bp in size with a 34.9% GC content, composed of 14 contigs with an N50 value of 352,766. The genome carried 1,951 coding sequences (CDSs), 55 tRNA, and 4 rRNA by RAST annotation. The COG database assigned 1,763 protein-coding genes to 20 different functional categories (**Figure 1**), such as clustering in transport and metabolism of different macromolecules, energy production and conversion, secondary metabolite biosynthesis, and cell functions. Genome mining using both antiSMASH v.7.0 and BAGEL4 identified two putative bacteriocin biosynthetic gene clusters (BBGCs), namely, BBGC1 (contig_1 RiPP-like) located in contig 1 (from 37,767 to 48,215 nt) that contained two bacteriocin belonging to IIc class, and BBGC2 located in contig 2 (from 10.357 to 31348 nt) carrying the bacteriocin helveticin J of class III. In particular, antiSMASH v.7.0 identified nine putative proteins in the bacteriocin cluster BBGC1, of which six were involved in the bacteriocin biosynthetic process: (i) two class IIc bacteriocins, namely, bacteriocin leader domain-containing protein (ctg1_38; PF10439) and gassericin A-like bacteriocin (ctg1_39, PF10439); (ii) two ABC transporter-related protein (ctg1_35 and ctg1_36; PF00005); (iii) immunity protein (ctg1_40); and (iv) one bacterial extracellular solute-binding protein (ctg_141, PF13416) (**Figures 2A, B**; **Supplementary Data Sheet S2**). Furthermore, the antiSMASH cluster blast showed identical genetic organization of gassericin-S BGC from *L. gasseri* (BGC0001601) including two bacteriocins (*gasA* and *gasX*) and immunity protein (*gasI*) as shown in **Figure 2C** (Kasuga et al., 2019). BBGC2 was detected only by BAGEL4 and identified the bacteriocin helveticin J as the core peptide and two bacteriocin ABC transporters (PF00005), which may be involved in the transport of the bacteriocin across the membrane (**Supplementary Data Sheet S3**).

### 3.2 Anti-*Klebsiella* activity of *L. gasseri* 1A-TV

**Figure 3:**
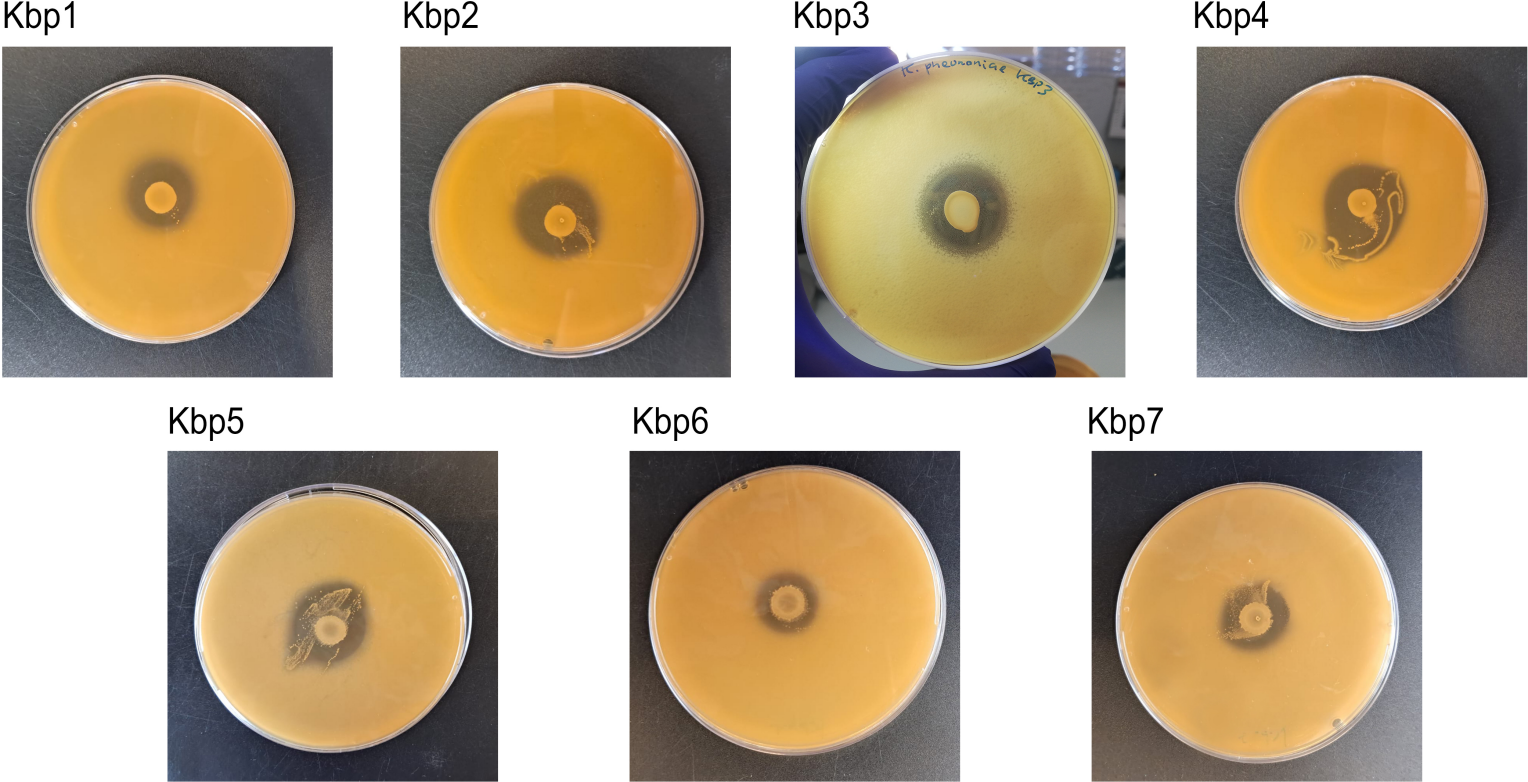
Agar spot test for each strain of *K. pneumoniae*. Strong antagonistic activity of 1A-TV was confirmed for all strains, with a diameter of inhibition zone > 10 mm.

The effect of antagonistic activity of *L. gasseri* 1A-TV against seven MDR *K. pneumoniae* strains evaluated by the deferred antagonism test (**Supplementary Figure 1**) and quantified by the agar spot test demonstrated a total inhibition of the seven indicator strains with diameters of inhibition zones > 10 mm (++++) for all seven indicator strains (**Figure 3**).

### 3.3 Auto-aggregation and co-aggregation ability

**Figure 4:**
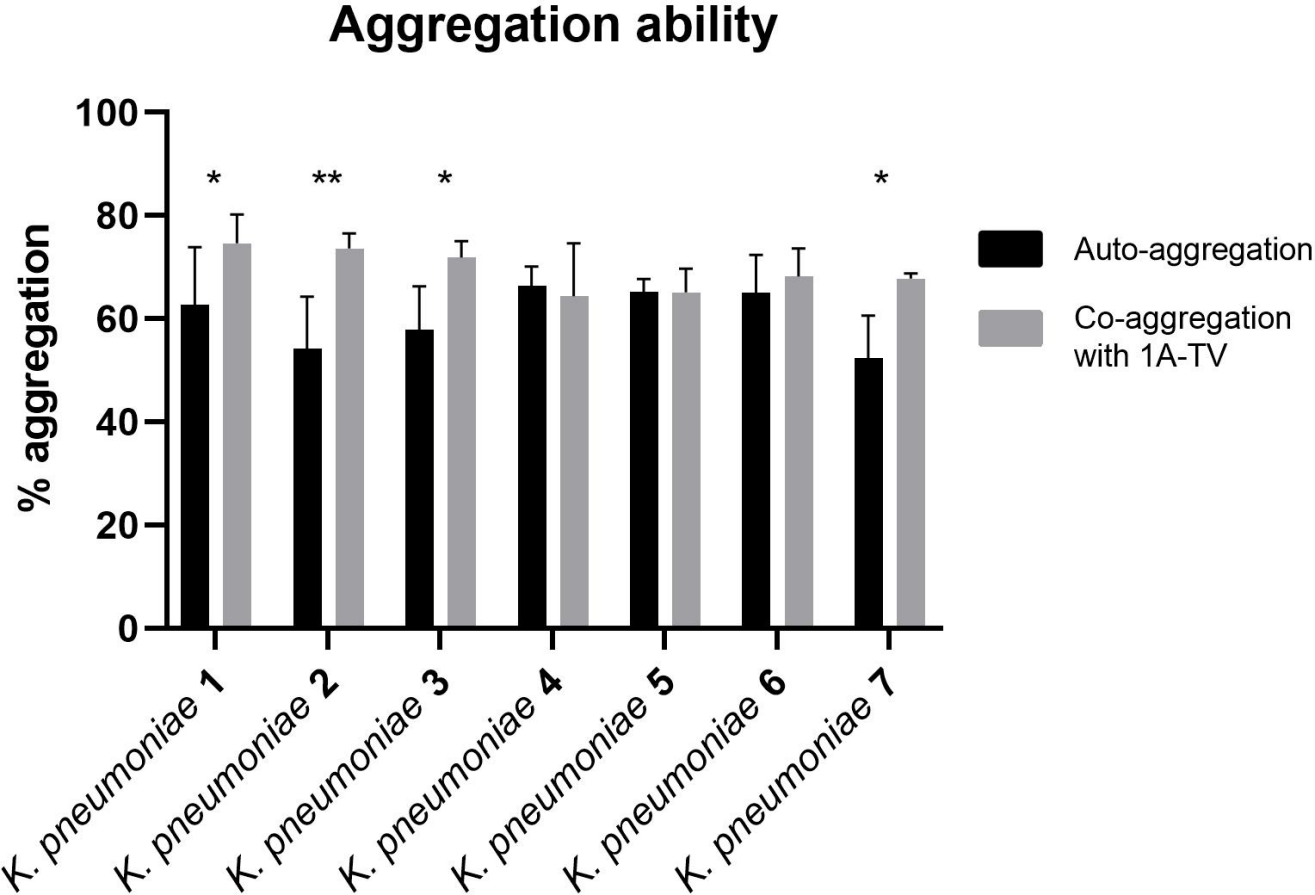
Auto-aggregation and co-aggregation of *K. pneumoniae* strains with *L. gasseri* 1A-TV. Co-aggregation values were considered statistically significant for strain Kbp1, Kbp2, Kbp3, and Kbp7, where ns was assigned for *p* -value > 0.05, * for *p*-value ≤ 0.05, and ** for *p* -value ≤ 0.01.

The auto-aggregation and co-aggregation levels of the indicator strains are shown in **Figure 4**. A good ability for auto-aggregation is shown by each strain, with aggregation values ranging between 52% and 66%. Moreover, all *K. pneumoniae* strains co-aggregated with *L. gasseri*, but only for *K. pneumoniae* 1, 2, 3, and 7 was it statistically significant (*p*< 0.05), with co-aggregation values of 74.6%, 73.6%, 71.9%, and 67.8%, respectively (**Supplementary Table S1**).

### 3.4 Metabolic profile of *L. gasseri* 1A-TV CFS

The NMR spectra of CFS *L. gasseri* 1A-TV identified 25 molecules, mainly metabolites belonging to the families of organic acids, ketones, alcohols, amino acids, monosaccharides, and nucleosides, and concentrations were expressed subtracting the original quantity present in the MRS medium for each component (**Supplementary Table S2**). Although the 1A-TV metabolic profile included many metabolites involved in the carbohydrate metabolism and amino acid metabolism, organic acids showed the highest concentrations, including ethanol (55.68 mM), lactate (46.62 mM), and acetate (16.61 mM), followed by succinate (7.88 mM) and amino acids like alanine (0.53 mM), valine (0.13 mM), and isoleucine (0.01 mM). Lower levels of certain components compared to the original medium included statistically significant variations for citrate (−2.29 mM), malate (−1.90 mM), and threonine (−0.73 mM) levels.

### 3.5 Inhibition of planktonic *K. pneumoniae* by 1A-TV CFS and bactericidal effect

**Figure 5:**
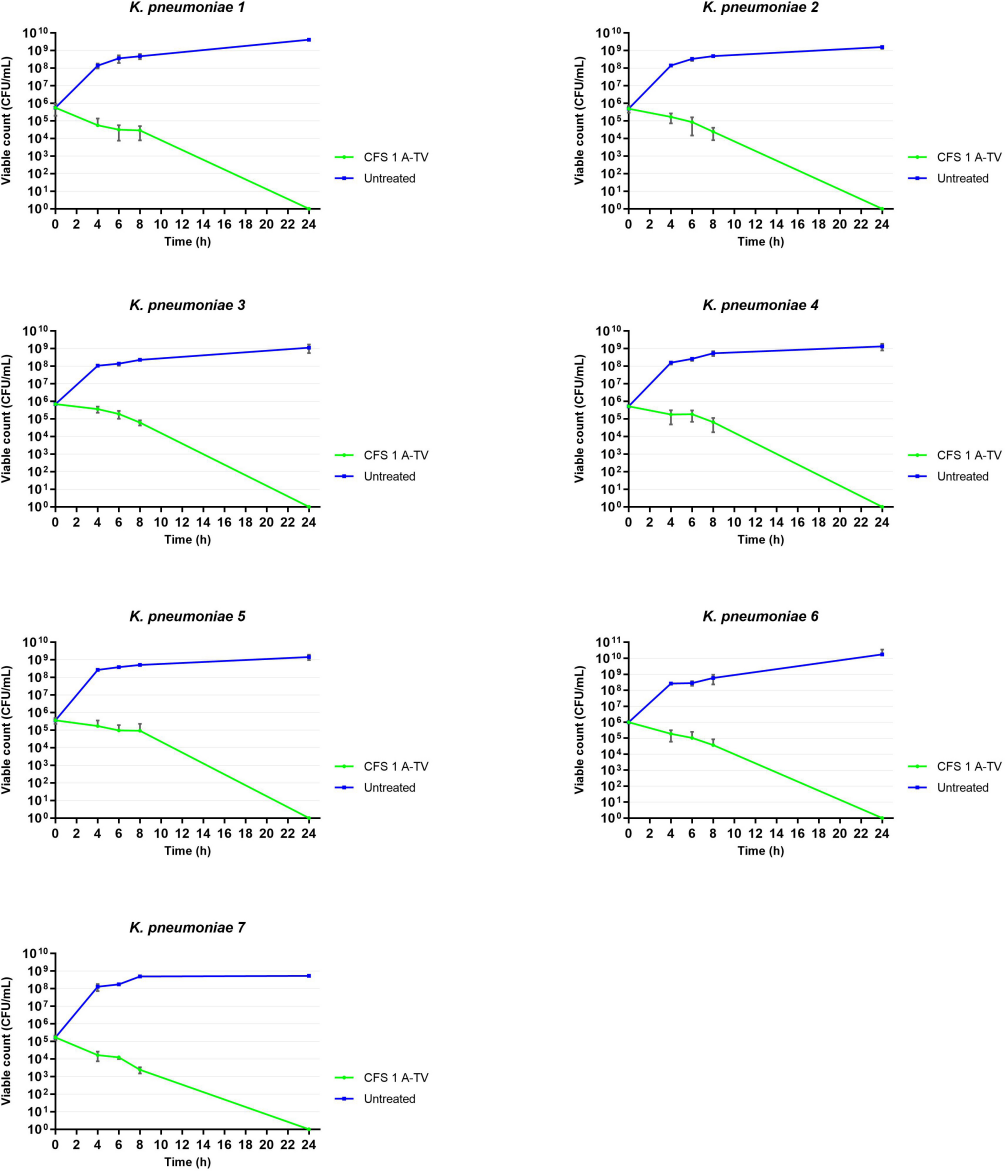
Time-killing curves showed the ability of *L. gasseri* 1A-TV CFS to inhibit the growth of all indicators. After 24 h of incubation, CFS showed bactericidal activity reducing the inoculum by more than three logs. Statistical analysis showed that differences between treated and untreated *K. pneumoniae* were significative for all strains: *p*< 0.001 for strains 1, 2, 5, and 7, and *p*< 0.01 for strains 3, 4, and 6.

The results of time-killing assays are summed up in **Figure 5**. After 4, 6, and 8 h of incubation, 1A-TV CFS showed a bacteriostatic effect on all ESBL- and KPC-producing strains, with a reduction in bacterial growth of about 1 log_10_ compared to the initial inoculum. After 24 h of incubation, 1A-TV CFS exerted an extraordinary bactericidal effect against all indicator strains, as the bactericidal activity is defined as a reduction of at least 99.9% (≥ 3 log_10_) of the total count (CFU/mL) of the original inoculum (**Supplementary Table S3**). Statistical analysis showed that differences between treated and untreated *K. pneumoniae* were significant for all strains: *p*< 0.001 for strains 1, 2, 5, and 7, and *p*< 0.01 for strains 3, 4, and 6. Furthermore, proteolytic treatment of CFS with proteinase K and trypsin determined a decrease of the antibacterial activity compared to the CFS without proteolytic treatment (**Table 2**).

**Table 2:** Effect of proteolytic enzymes on the antagonistic activity of CFS against *K. pneumoniae*.

*Strain*	*CFS*	*CFS treated with proteinase K*	*CFS treated with trypsin*
*Kbp1*	+++	+	+
*Kbp2*	+++	+	+
*Kbp3*	+++	+	+
*Kbp4*	+++	+	+
*Kbp5*	+++	+	+
*Kbp6*	+++	+	+
*Kbp7*	+++	+	+

After the treatment, the antibacterial activity of the CFS was expressed as total (+++), good (++), partial (+), and no inhibition (−).

### 3.6 Inhibition of biofilm formation of ESBL- and KPC-producing *K. pneumoniae* by CFS

**Figure 6:**
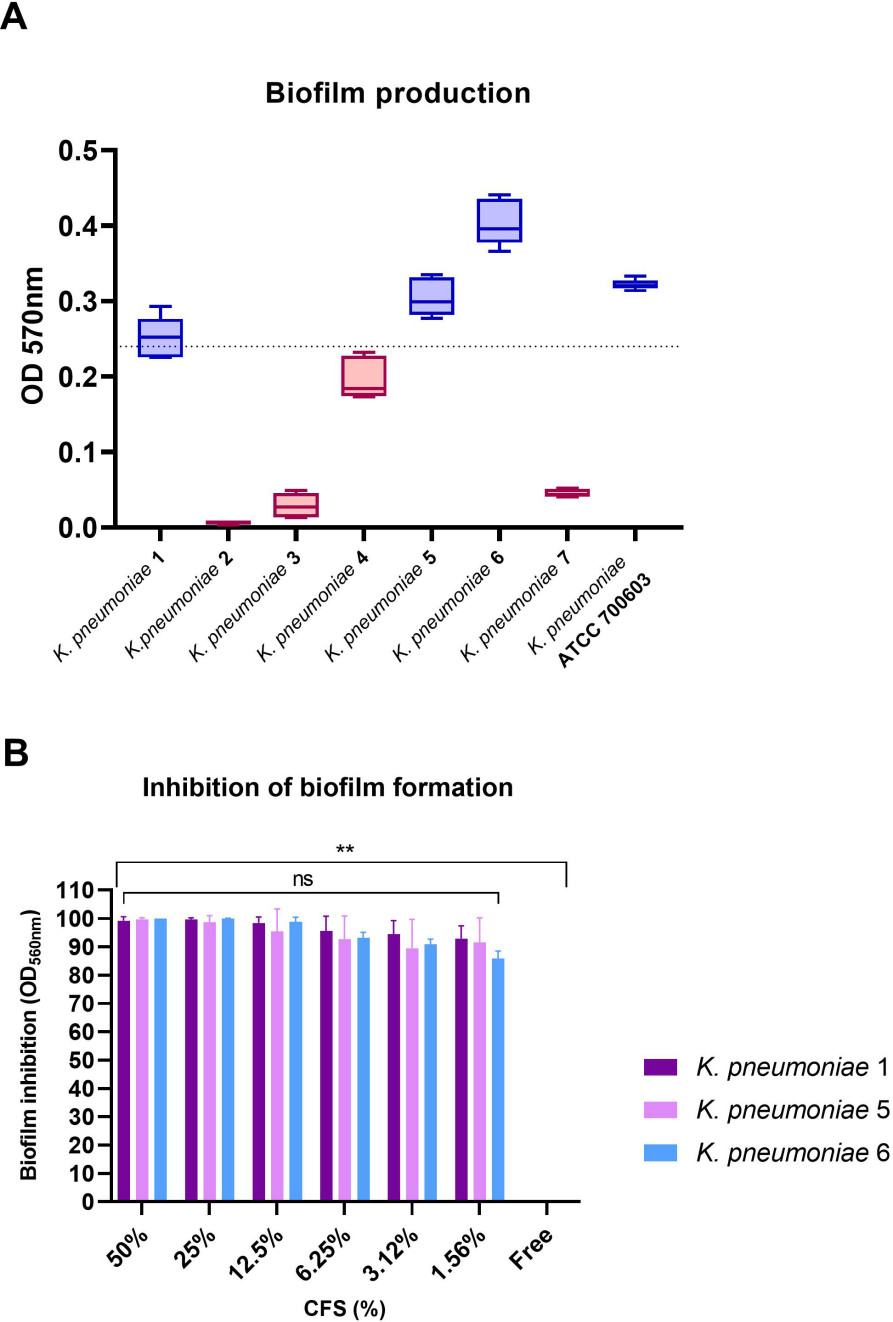
**(A)** Biofilm formation by *K. pneumoniae* indicator strains. Strains with values above baseline (OD_570_ > 0.240) were considered strong biofilm producers. **(B)** Inhibition of biofilm formation for biofilm producers at scalar concentrations of CFS. No statistical differences were found between CFS at different concentrations, while comparing the free control with all strains and all CFS concentrations showed statistical significance in all cases (**p< 0.01). ns, not significant.

To evaluate the ability of 1-ATV CFS to interfere with the *K. pneumoniae* biofilm formation, the total biomass was evaluated by colorimetric assay using crystal violet. Only *K. pneumoniae* 1 (ST258, *bla*_KPC-3_, *bla*_SHV-11_, *bla*_TEM-1_, *bla*_oxa_-positive), *K. pneumoniae* 5 (ST307, *bla*_TEM-_ *_1_*, *bla_CTX_*_-M-15_-positive), and *K. pneumoniae* 6 (ST307, *bla*_TEM-_ *_1_*, *bla_CTX_*_-M-15_-positive) were considered strong biofilm producers with values above the threshold line (OD_570_ > 0.240) and were selected for biofilm inhibition testing (**Figure 6A**).

As shown in **Figure 6B**, *L. gasseri* 1A-TV CSF was able to determine a high percentage of biofilm inhibition (from 100% to 95% inhibition) for all *K. pneumoniae* strains tested at different concentrations, and only at lower concentrations (from 6.25% to 1.56%) was inhibition just slightly reduced with high values from 93% to 86% compared to the free control (**Supplementary Table S4**). Importantly, no statistical difference was found among the scalar concentrations of CFS. When scalar concentrations of CFS and free control were compared, a statistical difference was found (*p*< 0.01) for all strains and all concentrations analyzed.

Moreover, viability was assessed after treatment with CFS at scalar concentrations (**Supplementary Figure 2**): At higher concentrations, CFS inhibited *K. pneumoniae* growth, despite the higher inoculum compared to time-killing assays, and at concentrations from 12.5% to 1.56% of CFS, all strains had a viable count ranging between 10^8^ and 10^11^ CFU/mL, but biofilm formation was still inhibited (**Supplementary Table S5**).

## 4 Discussion

The emergence of CRKP represents a serious threat to public health, especially for critically ill and immunocompromised patients, and its fecal carriage can represent a risk factor for the onset of infections; therefore, intestinal clearance of CRKP could prevent CRKP infections. Several studies have reported beneficial effects exerted by novel strategies based on probiotics such as lactobacilli against MDR pathogens. The wide spectrum of valuable properties yielded by probiotics is associated with their ability to reinforce the dynamic equilibrium of the resident microbiota, which is an important tool to counteract “dysbiosis” in the host, maintain mucosal integrity, and prevent infection (Stavropoulou and Bezirtzoglou, 2020). In particular, antagonistic mechanisms carried out by probiotics can comprise colonization of the host epithelium, interference of pathogen adhesion, and direct co-aggregation with pathogens, all hindering host mucosa colonization; likewise, release of inhibitory substances and metabolites can interfere with growth and biofilm formation of pathogens (Sanders et al., 2019).

In this study, we evaluated the antimicrobial activity of *L. gasseri* 1ATV against six clinical isolates selected for their multidrug resistance profiles and for the presence of carbapenemase and ESBL genes, including two ESBL-producing strains (ST 307), two KPC-producing strains (ST 258), and two co-producing NDM-OXA-48 KP (ST 101); moreover, all strains were also resistant to colistin and fosfomycin. Interestingly, four CPKP strains belong to nosocomial high-risk clones ST101 and ST258, which were associated with infections reported worldwide (Shaidullina et al., 2023). Only a few studies have characterized the activity of alternative strategies like probiotic *Lactobacillus* spp. against similar carbapenemase-producing *K. pneumoniae* strains (Chen et al., 2019, Chen et al., 2021; Yan et al., 2021), and even less have focused on the antimicrobial activity against biofilm formation by carbapenemase-producing *K. pneumoniae* (Chatupheeraphat et al., 2023). To the best of our knowledge, this is the first study to explore the antibacterial activity of *L. gasseri* against ESBL- and carbapenemase-producing *K. pneumoniae* strains, combined with genome mining tools to obtain new insights into its anti-CRKP effects. First, *L. gasseri* 1A-TV exhibited excellent antimicrobial activity against all strains by using the classic methods of deferred antagonism and agar spot assays, and these results were confirmed using 1A-TV CFS. In particular, when the 1A-TV CFS activity was evaluated by time-killing tests, an extraordinary bactericidal effect was observed after 24 h of incubation against all isolates, and the antimicrobial effects decreased when the CFS was treated with either proteinase K or trypsin.

Since there is a renewed interest in alternative strategies, involving metabolic by-products of probiotic strains that could retain their bioactivity without using viable cells in patients (Vallejo-Cordoba et al., 2020), these results are very promising for future perspectives. Moreover, several studies have demonstrated the antimicrobial activity of lactobacilli CFS against a wide spectrum of pathogens, including ESBL-producing *K. pneumoniae* and *P. aeruginosa* (El-Mokhtar et al., 2020).

Genome mining by antiSMASH and BAGEL4 revealed three bacteriocin genes in two different BBGCs. In BBGC1 two bacteriocins were detected and categorized as class IIc, which comprises a wide range of bactericidal bacteriocins usually produced by the Firmicutes phylum. Interestingly, one was a gassericinA/acidocin-like bacteriocin, a circular bacteriocin with a wide spectrum against food-borne pathogens like *Listeria monocytogenes* and *Staphylococcus aureus* (Pandey et al., 2013). In BBGC2, a third bacteriocin was detected, namely, helveticin J belonging to class III, as previously described (Scillato et al., 2021), that has a bactericidal mode of action against sensitive indicators as well (Joerger and Klaenhammer, 1986). The data obtained by genome mining could suggest their potential role in the antagonistic activity of *L. gasseri* 1A-TV, considering that bacteriocins are believed to contribute to interspecies competition with other bacteria, including pathogens; therefore, the production of bacteriocins could represent an important antimicrobial factor (Soltani et al., 2021). Further studies need to be performed to elucidate the actual involvement of bacteriocins in the antimicrobial activity of *L. gasseri* 1A-TV CFS.

We also evaluated the metabolic profiles of 1A-TV CFS by ^1^H-NMR analysis to measure what metabolites are produced and consumed by this strain, which included organic acids, amino acids, and metabolites of the carbohydrate pathways. 1A-TV is mainly a producer of ethanol, lactic acid, and, in minor concentration, acetic acid, with a similar ratio of lactate/acetate to other *Lactobacillus* spp (Parlindungan et al., 2021). Yet, when compared to other strains with inhibitory activity, like *L. plantarum* and *L. sakei* as previously reported (Parlindungan et al., 2021), the concentration of organic acids in *L. gasseri* 1A-TV CFS is half or even less. In particular, acetic acid produced by *L. plantarum* has been reported to be essential for its anticolonization effect against CRKP in mice, and it was normally produced as higher concentrations by this strain (50 mmol/L) than by 1A-TV (Yan et al., 2021). The lower concentration of organic acids in 1A-TV CFS might reinforce that the strong antimicrobial activity of this strain is particularly given by the bacteriocins it produces.

In this study, we investigated two other aspects that contribute to antibacterial effects like co-aggregation and anti-biofilm activities. In fact, multiple studies have highlighted the protective role of probiotics that, by binding pathogens into co-aggregates, can exert their inhibitory activity: co-aggregation can expose pathogens to higher concentrations of inhibitory substances and metabolites and it can also hinder biofilm formation, which is an important feature for infection and resistance to host defenses and antibiotics (Monteagudo-Mera et al., 2019). In particular, 1A-TV showed high levels of co-aggregation with all strains, with statistically significant values for *K. pneumoniae* 1, 2, 3, and 7 strains, with higher co-aggregation values compared to *Lactobacillus delbrueckii* vs. *K. pneumoniae* (Bnfaga et al., 2023) that might depend on strain- or species-specific aggregating factors of *L. gasseri* 1A-TV.

Another feature of lactobacilli CFS is the strong anti-biofilm activity that could be due to the presence of bio-surfactants and exopolysaccharides produced by these strains (Raras et al., 2019; El-Mokhtar et al., 2020). The anti-biofilm activity of *L. gasseri* 1A-TV CFS against *K. pneumoniae* strong biofilm producers (strain 1, *bla*_KPC-3_, *bla*_SHV-11_, *bla*_TEM-1_, *bla*_oxa_-positive; strain 5, *bla*_TEM-_ *_1_*, *bla_CTX_*_-M-15_-positive; and strain 6, *bla*_TEM-_ *_1_*, *bla_CTX_*_-M-15_-positive) is noteworthy because biofilm formation is a strategy of some organisms to persist in severe environments by promoting bacterial resistance to antimicrobial agents, the immune system, and stress conditions (Piperaki et al., 2017; Chatupheeraphat et al., 2023). Furthermore, the majority of clinical isolates of *K. pneumoniae* are able to form biofilm (approximately 80%), even those that are ESBL or carbapenemase producers, which can lead to persistence of colonization and infection, and it can also promote the spread of antibiotic resistance (Sabença et al., 2023).

This study has potential limitations. First, despite using NMR analysis to identify and quantify different metabolites released in CFS including organic acids, amino acids, and carbohydrate metabolism, the full characterization of the bioactives present in the CFS was not clearly defined. Thus, further studies by mass spectrometry are already underway to thoroughly investigate the bioactive molecules present in CFS and to investigate their activity in antimicrobial effects. A second limitation of this study is the inclusion of only a small number of *K. pneumoniae* strains, which were selected because they were well-characterized at the phenotype and genotype level.

In conclusion, this work supports the efficacy of the *L. gasseri* 1A-TV strain in adjuvant and preventive strategies against ESBL- and carbapenemase-producing *K. pneumoniae*, elucidating the inhibitory effect of CFS on bacterial growth and biofilm production. Even more, the use of metabolites and products of probiotic strains, like CFS, could be the answer to the debate of using probiotics as live microorganisms that could acquire virulence or antimicrobial resistance genes, becoming a potential vehicle for the diffusion of resistance determinants to other bacteria (Imperial and Ibana, 2016). Thus, the use of active natural bioproducts opens new perspectives aimed at the prevention and control of colonization by ESBL- and carbapenemase-producing *K. pneumoniae*.

## Data Availability

The datasets presented in this study can be found in online repositories. The names of the repository/repositories and accession number(s) can be found in the article/**Supplementary Material.**
