# Supplementary material for: Cell-free supernatant of Lactobacillus gasseri 1A-TV shows a promising activity to eradicate carbapenem-resistant Klebsiella pneumoniae colonization
Source: Front Cell Infect Microbiol. 2024 Dec 3;14:1471107. doi: 10.3389/fcimb.2024.1471107 (PMC11613640; doi:10.3389/fcimb.2024.1471107)
Supplement: Supplementary file 2 [file DataSheet2.pdf]

```
LOCUS       Contig_1               10449 bp    DNA     linear   UNK 01-
JAN-1980
DEFINITION  Contig_1 Contig_1 undefined product 1:593801 forward.
ACCESSION   Contig_1
VERSION     Contig_1
KEYWORDS    .
SOURCE
  ORGANISM
            .
COMMENT     ##antiSMASH-Data-START##
            Version      :: 7.1.0
            Run date     :: 2024-01-07 18:16:49
            NOTE: This is a single region extracted from a larger record!
            Orig. start  :: 37766
            Orig. end    :: 48215
            ##antiSMASH-Data-END##
FEATURES             Location/Qualifiers
     protocluster    1..10449
                     /aStool="rule-based-clusters"
                     /category="RiPP"
                     /contig_edge="False"
                     /core_location="[5000:5449]"
                     /cutoff="20000"
                     /detection_rule="(strepbact or Antimicrobial14 or
                     Bacteriocin_IId or BacteriocIIc_cy or Bacteriocin_II
or
                     Bacteriocin_IIi or Lactococcin or Antimicrobial17 or
                     Lactococcin_972 or Bacteriocin_IIc or LcnG-beta or
Cloacin
                     or Linocin_M18 or TIGR03651 or TIGR03693 or
TIGR03601 or
                     TIGR03795 or TIGR03975 or DUF692 or TIGR01193 or
(YcaO or
                     TIGR03604))"
                     /neighbourhood="5000"
                     /product="RiPP-like"
                     /protocluster_number="1"
                     /tool="antismash"
     proto_core      5001..5449
                     /aStool="rule-based-clusters"
                     /tool="antismash"
                     /cutoff="20000"
                     /detection_rule="(strepbact or Antimicrobial14 or
                     Bacteriocin_IId or BacteriocIIc_cy or Bacteriocin_II
or
                     Bacteriocin_IIi or Lactococcin or Antimicrobial17 or
                     Lactococcin_972 or Bacteriocin_IIc or LcnG-beta or
Cloacin
                     or Linocin_M18 or TIGR03651 or TIGR03693 or
TIGR03601 or
                     TIGR03795 or TIGR03975 or DUF692 or TIGR01193 or
(YcaO or
                     TIGR03604))"
                     /neighbourhood="5000"
```

```
                     /product="RiPP-like"
                     /protocluster_number="1"
     cand_cluster    1..10449
                     /candidate_cluster_number="1"
                     /contig_edge="False"
                     /detection_rules="(strepbact or Antimicrobial14 or
                     Bacteriocin_IId or BacteriocIIc_cy or Bacteriocin_II
or
                     Bacteriocin_IIi or Lactococcin or Antimicrobial17 or
                     Lactococcin_972 or Bacteriocin_IIc or LcnG-beta or
Cloacin
                     or Linocin_M18 or TIGR03651 or TIGR03693 or
TIGR03601 or
                     TIGR03795 or TIGR03975 or DUF692 or TIGR01193 or
(YcaO or
                     TIGR03604))"
                     /kind="single"
                     /product="RiPP-like"
                     /protoclusters="1"
                     /tool="antismash"
     region          1..10449
                     /candidate_cluster_numbers="1"
                     /contig_edge="False"
                     /product="RiPP-like"
                     /region_number="1"
                     /rules="(strepbact or Antimicrobial14 or
Bacteriocin_IId or
                     BacteriocIIc_cy or Bacteriocin_II or Bacteriocin_IIi
or
                     Lactococcin or Antimicrobial17 or Lactococcin_972 or
                     Bacteriocin_IIc or LcnG-beta or Cloacin or
Linocin_M18 or
                     TIGR03651 or TIGR03693 or TIGR03601 or TIGR03795 or
                     TIGR03975 or DUF692 or TIGR01193 or (YcaO or
TIGR03604))"
                     /tool="antismash"
     CDS             352..2169
                     /gene_functions="transport (smcogs) SMCOG1288:ABC
                     transporter related protein (Score: 250.1; E-value:
                     7.4e-76)"
                     /gene_kind="transport"
                     /locus_tag="ctg1_35"
                     /transl_table=11

/translation="MIDKRLFKLPKAKIMLAMLAGLMFLQAFAILGQGIFLARAIVGSW

KRQPFTEIAQDVLLFLIFYLLRQGINWFQKWYMNRYANQTTTLLRQQLLNKTYDGGIAL

VSRIGTGNLVSTLLDGMDEISNYLSLIFPKLIALAIVPWVILIYIFTLNALSGWILLLV

FPLLILFMIILGTAAQSKASKQYAGYVKLQNHFVDALRGLSTLKVLGLARKYGNIVYKN

SENYRKKTMGVLRVAILSTFTLDFFTTLSIAMIAMFLGIGLINGNLVLYPSLVILILSP
```

```
EYFLPIRDFGNDFHATLNGKNALGQIFDILAFPTTPQEDQLSSFTWNNDSTLVAKNLSF

NYSHVDQSKFTVNKNAKMSGVVKKKTMHKTSQHTADELRNINLNLTGFQKVGIIGLTGA

GKTTLMRILAGFLTPHLKDDNFTINGQTLAQLNQKNWQNQITYIPQDPYMFAASIKDNL

TFYNPNASQEEIDAALKATDLDNFVASLKDGLNTKIGENGRGISGGQKQRIALARAFLA

KDRKILFFDEPTAHLDIETEYELKQPMKKLMENHLVFFTTHRLHWLNDMDWCLVIENGE
                     IVEQGTPADLAKNGTAFKKLTKPLKEDLL"
     aSDomain        388..2061
                     /aSDomain="TIGR02857"
                     /aSTool="tigrfam"
                     /description="CydD: thiol reductant ABC exporter,
CydD
                     subunit"
                     /detection="hmmscan"
                     /domain_id="TIGRFam_ctg1_35_0001"
                     /evalue="5.60E-153"
                     /identifier="TIGR02857"
                     /label="ctg1_35"
                     /locus_tag="ctg1_35"
                     /protein_end="570"
                     /protein_start="12"
                     /score="508.6"
                     /tool="antismash"

/translation="KIMLAMLAGLMFLQAFAILGQGIFLARAIVGSWKRQPFTEIAQDV

LLFLIFYLLRQGINWFQKWYMNRYANQTTTLLRQQLLNKTYDGGIALVSRIGTGNLVST

LLDGMDEISNYLSLIFPKLIALAIVPWVILIYIFTLNALSGWILLLVFPLLILFMIILG

TAAQSKASKQYAGYVKLQNHFVDALRGLSTLKVLGLARKYGNIVYKNSENYRKKTMGVL

RVAILSTFTLDFFTTLSIAMIAMFLGIGLINGNLVLYPSLVILILSPEYFLPIRDFGND

FHATLNGKNALGQIFDILAFPTTPQEDQLSSFTWNNDSTLVAKNLSFNYSHVDQSKFTV

NKNAKMSGVVKKKTMHKTSQHTADELRNINLNLTGFQKVGIIGLTGAGKTTLMRILAGF

LTPHLKDDNFTINGQTLAQLNQKNWQNQITYIPQDPYMFAASIKDNLTFYNPNASQEEI

DAALKATDLDNFVASLKDGLNTKIGENGRGISGGQKQRIALARAFLAKDRKILFFDEPT
                     AHLDIETEYELKQPMKKLMENHLVFFTTHRLHWLNDMDWCL"
     PFAM_domain     424..1188
                     /aSDomain="ABC_membrane"
                     /aSTool="clusterhmmer"
                     /database="35.0"
                     /db_xref="PF00664.26"
                     /db_xref="GO:0005524"
                     /db_xref="GO:0016020"
                     /db_xref="GO:0055085"
```

```
                     /db_xref="GO:0140359"
                     /description="ABC transporter transmembrane region"
                     /detection="hmmscan"
                     /domain_id="clusterhmmer_ctg1_35_0001"
                     /evalue="7.10E-24"
                     /gene_ontologies="GO:0005524: ATP binding"
                     /gene_ontologies="GO:0016020: membrane"
                     /gene_ontologies="GO:0055085: transmembrane
transport"
                     /gene_ontologies="GO:0140359: ABC-type transporter
                     activity"
                     /label="ctg1_35"
                     /locus_tag="ctg1_35"
                     /protein_end="279"
                     /protein_start="24"
                     /score="85.0"
                     /tool="antismash"

/translation="LQAFAILGQGIFLARAIVGSWKRQPFTEIAQDVLLFLIFYLLRQG

INWFQKWYMNRYANQTTTLLRQQLLNKTYDGGIALVSRIGTGNLVSTLLDGMDEISNYL

SLIFPKLIALAIVPWVILIYIFTLNALSGWILLLVFPLLILFMIILGTAAQSKASKQYA

GYVKLQNHFVDALRGLSTLKVLGLARKYGNIVYKNSENYRKKTMGVLRVAILSTFTLDF
                     FTTLSIAMIAMFLGIGLINGNLVLYPSLVILIL"
     PFAM_domain     1483..1941
                     /aSDomain="ABC_tran"
                     /aSTool="clusterhmmer"
                     /database="35.0"
                     /db_xref="PF00005.30"
                     /db_xref="GO:0005524"
                     /description="ABC transporter"
                     /detection="hmmscan"
                     /domain_id="clusterhmmer_ctg1_35_0002"
                     /evalue="2.90E-27"
                     /gene_ontologies="GO:0005524: ATP binding"
                     /label="ctg1_35"
                     /locus_tag="ctg1_35"
                     /protein_end="530"
                     /protein_start="377"
                     /score="96.0"
                     /tool="antismash"

/translation="LRNINLNLTGFQKVGIIGLTGAGKTTLMRILAGFLTPHLKDDNFT

INGQTLAQLNQKNWQNQITYIPQDPYMFAASIKDNLTFYNPNASQEEIDAALKATDLDN
                     FVASLKDGLNTKIGENGRGISGGQKQRIALARAFLAKDRKILFFDEPTA"
     CDS             2166..3911
                     /gene_functions="transport (smcogs) SMCOG1288:ABC
                     transporter related protein (Score: 229.7; E-value:
                     1.2e-69)"
                     /gene_kind="transport"
                     /locus_tag="ctg1_36"
```

```
                     /transl_table=11

/translation="MMNKKFSWKNDHWIKPYLAQYKWNFLLAIFLGVIMFFCGGALMFY

AGYTIDKAATRPENILMIYVPIVLMRAVGIGRPLFRYLERLVSHNWILRVTSSLRRRLF

YIAERNTSAVGSSFQTGSILSLLTDDIGHLQNLYLRTIFPAILSYLVGFFVVILLGLFS

WPLAGAIAILLVAELVLVPFFSLLKQAAVRTKEKEEKAQLYTEFTDQVLGAGDWKISGR

RDAFFNQTKETLKSLGQHEKKSGKFDWARDFGLEFIFGLMAVALLYFTNQTLTNNQEAA

NYVGAVVLALFPLSDAFIPVGQGIEEWHTYSDSVKHLNELKVPENRLPVQQYLDPTFAG

TLRVSDISFTYPKEDYPIIQNFSLTLKRGQKAALIGPSGAGKSTILQLILGDLKPNQGT

VTLDRLNVLKLQRERSKLFSVLNQEPFLFNTTIYENLKMANPDATTDQMMEILEKVQLA

DFVNSLPKGLDTEVAEAGARFSGGQKERLALARVLLQDTPIVLLDEPTVGLDSLTEQKL

LNLVFEVLKNKTVVWVTHHLQGVKYMNEVLFFKDGKVTMQGNPHELFKHNEHFHQLYLM
                     DQGLI"
     aSDomain        2199..3776
                     /aSDomain="TIGR02868"
                     /aSTool="tigrfam"
                     /description="CydC: thiol reductant ABC exporter,
CydC
                     subunit"
                     /detection="hmmscan"
                     /domain_id="TIGRFam_ctg1_36_0002"
                     /evalue="1.90E-139"
                     /identifier="TIGR02868"
                     /label="ctg1_36"
                     /locus_tag="ctg1_36"
                     /protein_end="537"
                     /protein_start="11"
                     /score="463.6"
                     /tool="antismash"

/translation="HWIKPYLAQYKWNFLLAIFLGVIMFFCGGALMFYAGYTIDKAATR

PENILMIYVPIVLMRAVGIGRPLFRYLERLVSHNWILRVTSSLRRRLFYIAERNTSAVG

SSFQTGSILSLLTDDIGHLQNLYLRTIFPAILSYLVGFFVVILLGLFSWPLAGAIAILL

VAELVLVPFFSLLKQAAVRTKEKEEKAQLYTEFTDQVLGAGDWKISGRRDAFFNQTKET

LKSLGQHEKKSGKFDWARDFGLEFIFGLMAVALLYFTNQTLTNNQEAANYVGAVVLALF

PLSDAFIPVGQGIEEWHTYSDSVKHLNELKVPENRLPVQQYLDPTFAGTLRVSDISFTY

PKEDYPIIQNFSLTLKRGQKAALIGPSGAGKSTILQLILGDLKPNQGTVTLDRLNVLKL

QRERSKLFSVLNQEPFLFNTTIYENLKMANPDATTDQMMEILEKVQLADFVNSLPKGLD
```

```
TEVAEAGARFSGGQKERLALARVLLQDTPIVLLDEPTVGLDSLTEQKLLNLVFEVLKNK
                     TVVWVTHHL"
     PFAM_domain     2367..2966
                     /aSDomain="ABC_membrane"
                     /aSTool="clusterhmmer"
                     /database="35.0"
                     /db_xref="PF00664.26"
                     /db_xref="GO:0005524"
                     /db_xref="GO:0016020"
                     /db_xref="GO:0055085"
                     /db_xref="GO:0140359"
                     /description="ABC transporter transmembrane region"
                     /detection="hmmscan"
                     /domain_id="clusterhmmer_ctg1_36_0003"
                     /evalue="9.10E-07"
                     /gene_ontologies="GO:0005524: ATP binding"
                     /gene_ontologies="GO:0016020: membrane"
                     /gene_ontologies="GO:0055085: transmembrane
transport"
                     /gene_ontologies="GO:0140359: ABC-type transporter
                     activity"
                     /label="ctg1_36"
                     /locus_tag="ctg1_36"
                     /protein_end="267"
                     /protein_start="67"
                     /score="28.9"
                     /tool="antismash"

/translation="VLMRAVGIGRPLFRYLERLVSHNWILRVTSSLRRRLFYIAERNTS

AVGSSFQTGSILSLLTDDIGHLQNLYLRTIFPAILSYLVGFFVVILLGLFSWPLAGAIA

ILLVAELVLVPFFSLLKQAAVRTKEKEEKAQLYTEFTDQVLGAGDWKISGRRDAFFNQT
                     KETLKSLGQHEKKSGKFDWARDFGLEFIFGLMAVALL"
     PFAM_domain     3243..3683
                     /aSDomain="ABC_tran"
                     /aSTool="clusterhmmer"
                     /database="35.0"
                     /db_xref="PF00005.30"
                     /db_xref="GO:0005524"
                     /description="ABC transporter"
                     /detection="hmmscan"
                     /domain_id="clusterhmmer_ctg1_36_0004"
                     /evalue="8.10E-32"
                     /gene_ontologies="GO:0005524: ATP binding"
                     /label="ctg1_36"
                     /locus_tag="ctg1_36"
                     /protein_end="506"
                     /protein_start="359"
                     /score="110.7"
                     /tool="antismash"

/translation="QNFSLTLKRGQKAALIGPSGAGKSTILQLILGDLKPNQGTVTLDR
```

```
LNVLKLQRERSKLFSVLNQEPFLFNTTIYENLKMANPDATTDQMMEILEKVQLADFVNS
                     LPKGLDTEVAEAGARFSGGQKERLALARVLLQDTPIVLLDEPT"
     CDS             3932..4858
                     /locus_tag="ctg1_37"
                     /transl_table=11

/translation="MSAEAKQGYYELVEPRTLFNSIIPVLLGMMYTEYNFQFFRIFPTI

EMCIATIVLQIFMNVNDGYWDYKREKAAKTGDHKKNPIGKYHLNPKHVLAFVWVLFIIS

AVCAILIGLQTNIYIWIVGIICYAIAISYSTGSHTISAGPFGEIAACFAMGFGIFLVMV

YINVCSKVSFNWNFIYPIILAAGIPEICNFTLMLGNNLCDHDADIANGRHTLVSYIGIK

GGLYLFVFNYLLGFFLTGWAIWIGVLPWSVALILICIPTIYKNMRFLWKIQTKPKSFPK
                     VVQNTQVLFITEAVGFFIGLILNLRIR"
     PFAM_domain     3995..4747
                     /aSDomain="UbiA"
                     /aSTool="clusterhmmer"
                     /database="35.0"
                     /db_xref="PF01040.21"
                     /db_xref="GO:0016020"
                     /db_xref="GO:0016765"
                     /description="UbiA prenyltransferase family"
                     /detection="hmmscan"
                     /domain_id="clusterhmmer_ctg1_37_0005"
                     /evalue="1.80E-19"
                     /gene_ontologies="GO:0016020: membrane"
                     /gene_ontologies="GO:0016765: transferase activity,
                     transferring alkyl or aryl (other than methyl)
groups"
                     /label="ctg1_37"
                     /locus_tag="ctg1_37"
                     /protein_end="272"
                     /protein_start="21"
                     /score="70.1"
                     /tool="antismash"

/translation="IIPVLLGMMYTEYNFQFFRIFPTIEMCIATIVLQIFMNVNDGYWD

YKREKAAKTGDHKKNPIGKYHLNPKHVLAFVWVLFIISAVCAILIGLQTNIYIWIVGII

CYAIAISYSTGSHTISAGPFGEIAACFAMGFGIFLVMVYINVCSKVSFNWNFIYPIILA

AGIPEICNFTLMLGNNLCDHDADIANGRHTLVSYIGIKGGLYLFVFNYLLGFFLTGWAI
                     WIGVLPWSVALILICIPTIYKNMRFLWKI"
     aSDomain        5001..5045
                     /aSDomain="TIGR01847"
                     /aSTool="tigrfam"
                     /description="bacteriocin_sig: bacteriocin-type
signal
                     sequence"
                     /detection="hmmscan"
```

```
                     /domain_id="TIGRFam_ctg1_38_0003"
                     /evalue="6.00E-03"
                     /identifier="TIGR01847"
                     /label="ctg1_38"
                     /locus_tag="ctg1_38"
                     /protein_end="15"
                     /protein_start="0"
                     /score="15.2"
                     /tool="antismash"
                     /translation="MKVLNECQLQTVVGG"
     CDS             5001..5240
                     /gene_functions="biosynthetic (rule-based-clusters)
                     RiPP-like: Bacteriocin_IIc"
                     /gene_kind="biosynthetic"
                     /locus_tag="ctg1_38"
                     /sec_met_domain="Bacteriocin_IIc (E-value: 8.8e-15,
                     bitscore: 50.0, seeds: 35, tool: rule-based-
clusters)"
                     /transl_table=11

/translation="MKVLNECQLQTVVGGKNWSVAKCGGTIGTNIAIGAWRGARAGSFF
                     GQPVSVGAGALIGASAGAIGGSVQCVGWLAGGGR"
     CDS             5240..5449
                     /gene_functions="biosynthetic (rule-based-clusters)
                     RiPP-like: Bacteriocin_IIc"
                     /gene_kind="biosynthetic"
                     /locus_tag="ctg1_39"
                     /sec_met_domain="Bacteriocin_IIc (E-value: 2.4e-08,
                     bitscore: 29.4, seeds: 35, tool: rule-based-
clusters)"
                     /transl_table=11

/translation="MIEKVSKNELSRIYGGNNVNWGSVAGSCGKGAVMGIYFGNPILGC
                     ANGAATSLVLQTASGIYKNYQKKR"
     CDS             5453..5725
                     /locus_tag="ctg1_40"
                     /transl_table=11

/translation="MTGIWIVIISIIVLSIIITNIVALIQTLLHKKNEKYYFDKSFGAY
                     GDKNNPKYLFNNVEHHDFFHVFYQYFICSYIPFIDVIFWFLGSIW"
     CDS             complement(5792..7087)
                     /gene_functions="biosynthetic-additional (smcogs)
                     SMCOG1143:sugar-binding lipoprotein (Score: 175.9;
E-value:
                     2.6e-53)"
                     /gene_kind="biosynthetic-additional"
                     /locus_tag="ctg1_41"
                     /transl_table=11

/translation="MNLYKKIALGLVAGLALIATGCSKGNKEDSSAANIPTKITKKTTV

TFWHGMNGGQRAELEKLTAEFEKKNPNIKIKLENQGSYIDLQAKVNSTLQSPNNLPTIT

QAYPGWLYNAVKSNMLVDLSPYINNSKLGWGSAAKSNIRTELLDGAKIQGKQYGIPFNK
```

```
SIEVLTYNKSMFEKYGIKKVPTTMKELAQVSETIYKKSNHQVVGAGFDSLANYYTLGMK

NEGINLTDKVNFTGKDSKKVIDYYAQGMKKGYFTTAGSAHYLSGPFANEKVAMYIGTSA

GEGYVKKAVGDKFTYDVAARPGKYTMQQGTDIYMFKKATAMQRTAAFKYMQFLTSKSTQ

LKWANATGYIPVNNSVVKSKEYKENTKTKLPAKLEGAMKHLYSIPVAKNSNAAYSQLDP
                     IMAKILYAAKNGQNVDNQIKAGKAKFDAAWKQ"
     PFAM_domain     complement(5990..6907)
                     /aSDomain="SBP_bac_8"
                     /aSTool="clusterhmmer"
                     /database="35.0"
                     /db_xref="PF13416.9"
                     /description="Bacterial extracellular solute-binding
                     protein"
                     /detection="hmmscan"
                     /domain_id="clusterhmmer_ctg1_41_0006"
                     /evalue="1.10E-47"
                     /label="ctg1_41"
                     /locus_tag="ctg1_41"
                     /protein_end="366"
                     /protein_start="60"
                     /score="163.4"
                     /tool="antismash"

/translation="KLTAEFEKKNPNIKIKLENQGSYIDLQAKVNSTLQSPNNLPTITQ

AYPGWLYNAVKSNMLVDLSPYINNSKLGWGSAAKSNIRTELLDGAKIQGKQYGIPFNKS

IEVLTYNKSMFEKYGIKKVPTTMKELAQVSETIYKKSNHQVVGAGFDSLANYYTLGMKN

EGINLTDKVNFTGKDSKKVIDYYAQGMKKGYFTTAGSAHYLSGPFANEKVAMYIGTSAG

EGYVKKAVGDKFTYDVAARPGKYTMQQGTDIYMFKKATAMQRTAAFKYMQFLTSKSTQL
                     KWANATGYIPVNNSVVKSKEYKENT"
     CDS             7376..8308
                     /locus_tag="ctg1_42"
                     /transl_table=11

/translation="MKIKKFLIGAAMVLLAATSAACSNSKSASKSSDGYTPKELNVQFV

PSVQASKLEAKAKPLQGLLEKQLHMPVHVTVSTDNSALVEAMASKKVDVGFLPPDAYVL

AHKRGVADVLLQAQRYGYDEPSGKQNHKLMDSYRSMIVVKKGSKIKSWKDLKGKTIAVQ

DPTSTSGYVLPIAELHKKGLNVPKDCKLVQVKGHDQAVLSVYNGDADAAFVFSDARPLA

AKDAPAVMKDVVPIYFTKYIPNDTVSVRSGMSKSFKKKLAKAFKDIAKTKKGKKILENI
                     YQHYGYVDSKDSNFNIVREYEAEAKKAQK"
     aSDomain        7382..8179
                     /aSDomain="TIGR01098"
                     /aSTool="tigrfam"
```

```
                     /description="3A0109s03R:
phosphate/phosphite/phosphonate
                     ABC transporter, periplasmic binding protein"
                     /detection="hmmscan"
                     /domain_id="TIGRFam_ctg1_42_0004"
                     /evalue="2.80E-86"
                     /identifier="TIGR01098"
                     /label="ctg1_42"
                     /locus_tag="ctg1_42"
                     /protein_end="268"
                     /protein_start="2"
                     /score="287.1"
                     /tool="antismash"

/translation="IKKFLIGAAMVLLAATSAACSNSKSASKSSDGYTPKELNVQFVPS

VQASKLEAKAKPLQGLLEKQLHMPVHVTVSTDNSALVEAMASKKVDVGFLPPDAYVLAH

KRGVADVLLQAQRYGYDEPSGKQNHKLMDSYRSMIVVKKGSKIKSWKDLKGKTIAVQDP

TSTSGYVLPIAELHKKGLNVPKDCKLVQVKGHDQAVLSVYNGDADAAFVFSDARPLAAK
                     DAPAVMKDVVPIYFTKYIPNDTVSVRSGMSKSFKKKLAKAFKDI"
     PFAM_domain     7505..8257
                     /aSDomain="Phosphonate-bd"
                     /aSTool="clusterhmmer"
                     /database="35.0"
                     /db_xref="PF12974.10"
                     /description="ABC transporter, phosphonate,
periplasmic
                     substrate-binding protein"
                     /detection="hmmscan"
                     /domain_id="clusterhmmer_ctg1_42_0007"
                     /evalue="1.30E-63"
                     /label="ctg1_42"
                     /locus_tag="ctg1_42"
                     /protein_end="294"
                     /protein_start="43"
                     /score="214.9"
                     /tool="antismash"

/translation="FVPSVQASKLEAKAKPLQGLLEKQLHMPVHVTVSTDNSALVEAMA

SKKVDVGFLPPDAYVLAHKRGVADVLLQAQRYGYDEPSGKQNHKLMDSYRSMIVVKKGS

KIKSWKDLKGKTIAVQDPTSTSGYVLPIAELHKKGLNVPKDCKLVQVKGHDQAVLSVYN

GDADAAFVFSDARPLAAKDAPAVMKDVVPIYFTKYIPNDTVSVRSGMSKSFKKKLAKAF
                     KDIAKTKKGKKILENIYQHYGYVDSKDSN"
     CDS             8401..9474
                     /locus_tag="ctg1_43"
                     /transl_table=11

/translation="MKLNKKFSLLLIASTIFLSLSACSKPTHEEQTNKKLANHIALISD
```

TNGIKDNSLNQSAWQGVKNYGTKNNLVEGNKGYNYFVPKDPQDYQATINKVLDNDFSTI

IGVGYTLKPNIVKAAKNNPKKNFVLIDTHAAKIKNLASISFKNQEGAYLAGVAAAYTTK

NHTVGLILGQNSKEMDSFKAGFTQGVTASSRKLHKHIKVMSKTVGNFSDINKAHDIAQE

MYNQKADIIFQAAGKSGQGVFKAAKEVNQTNPVGQKVWVIGSDEDQTKLGNYQAKGGQP

SNFTLTSVIKSADVAVEDLANQTAKGNFPGGKNLNYGLKNKGISLVQGNLSYHTWIKVQ
KAKQKIIDGKIKIMANR"
PFAM_domain 8533..9468
/aSDomain="Bmp"
/aSTool="clusterhmmer"
/database="35.0"
/db_xref="PF02608.17"
/db_xref="GO:0005886"
/description="ABC transporter substrate-binding
protein
PnrA-like"
/detection="hmmscan"
/domain_id="clusterhmmer_ctg1_43_0008"
/evalue="2.30E-57"
/gene_ontologies="GO:0005886: plasma membrane"
/label="ctg1_43"
/locus_tag="ctg1_43"
/protein_end="356"
/protein_start="44"
/score="194.4"
/tool="antismash"

/translation="DTNGIKDNSLNQSAWQGVKNYGTKNNLVEGNKGYNYFVPKDPQDY

QATINKVLDNDFSTIIGVGYTLKPNIVKAAKNNPKKNFVLIDTHAAKIKNLASISFKNQ

EGAYLAGVAAAYTTKNHTVGLILGQNSKEMDSFKAGFTQGVTASSRKLHKHIKVMSKTV

GNFSDINKAHDIAQEMYNQKADIIFQAAGKSGQGVFKAAKEVNQTNPVGQKVWVIGSDE

DQTKLGNYQAKGGQPSNFTLTSVIKSADVAVEDLANQTAKGNFPGGKNLNYGLKNKGIS
LVQGNLSYHTWIKVQKAKQKIIDGKIKIMAN"
ORIGIN
1 ccagtttctt caacaatcat tactttaatt attgttgccc taactgcttg
gtcagatatt
61 tctttgatta agaacaaaca ggtagcagct tttgtaacaa gtggtttaac
tttagtggct
121 gttgttggac tcttgttcaa tggacttttc ccacgcgttt taattgcaac
tgattcagca
181 cactcactct tgattaagac tgctgctaac tctaaattga cacttgaggt
aatgacaatt
241 gttgctctta ttctcttgcc aattgccttg gtttacatca tctggtcata
tgtagtcttt
301 agacggcgga ttaaggtaaa tcaaaacgcc taatctagaa caggaataaa
aatgattgat

361 aaacgacttt tcaaattacc aaaagccaaa atcatgctcg caatgcttgc aggactaatg
421 ttcttgcaag catttgctat tttaggacaa ggtatttttc ttgcccgagc aattgttggc
481 tcttggaaac ggcaaccttt tactgaaata gcacaagatg tgttactttt tttgatcttc
541 tatttattac ggcaaggtat caactggttt caaaaatggt acatgaaccg ttatgcaaat
601 caaacaacta cacttttgcg ccagcaacta cttaataaaa cctatgatgg cggaattgca
661 ctagtatcaa gaattggaac tggtaactta gtttctactt tactagatgg aatggatgag
721 atttctaact atctatcttt gatttttccc aaattaattg cacttgcaat tgttccttgg
781 gttattttga tctatatctt taccttaaat gccctatctg gctggatttt attgctagtc
841 tttcctctac taatcttatt tatgattatt ttagggacag cagcacaaag taaagctagt
901 aagcaatacg caggctatgt taagttgcaa aatcattttg tcgatgcctt gcgtggctta
961 agtactttaa aagttttagg acttgctaga aaatatggca atattgttta caagaacagc
1021 gaaaactatc gtaaaaaaac aatgggcgtt ttaagagtgg ctatcttgtc tacttttaca
1081 cttgatttct ttacaacttt atcaattgcg atgattgcca tgtttcttgg aattggctta
1141 atcaatggca atttagttct ctatccttcg ctagttattt tgatcttatc acccgaatat
1201 tttttaccta ttcgtgattt tggtaatgac tttcatgcaa cattgaacgg taaaaacgcg
1261 cttggacaaa ttttcgatat tttagctttt cctactacac cgcaagagga tcagttatcc
1321 agtttcacgt ggaacaacga tagcacttta gtagctaaga atctttcttt taactacagt
1381 catgttgatc aaagtaagtt cactgttaat aaaaatgcta aaatgagcgg cgtcgtaaaa
1441 aagaaaacga tgcataaaac aagccaacat accgctgatg aattacgtaa tatcaatttg
1501 aaccttactg gttttcaaaa agttggcatt atcgggctaa ctggagctgg caagacaact
1561 ttaatgcgaa tcttagctgg ttttcttact ccgcacctta aagacgataa cttcactatc
1621 aatggacaaa ctcttgctca acttaatcaa aagaattggc aaaatcaaat tacttacatt
1681 ccccaagatc cttacatgtt cgctgctagt atcaaagaca atctaacttt ctataaccca
1741 aatgccagcc aggaagagat tgatgcagct ttaaaggcaa ctgacttaga taacttcgtg
1801 gccagcttaa aagatggttt gaatactaaa attggcgaaa atggacgtgg catttctggt
1861 ggtcaaaaac agcgaattgc cttggcacgc gcatttttag caaaagaccg caagatttta
1921 ttcttcgatg aaccaactgc tcaccttgac attgaaactg aatacgaatt aaagcaacca

1981 atgaaaaaat taatggaaaa tcacctagtt ttctttacaa ctcaccgtct
gcattggtta
2041 aatgacatgg attggtgctt agttattgaa aatggagaaa tcgtcgaaca
aggtactcct
2101 gcagatttag caaaaaatgg aactgccttt aagaaactca ctaagccact
gaaagaagac
2161 ctactatgat gaataaaaaa ttttcttgga aaaatgatca ctggattaaa
ccatacctag
2221 cccaatataa atggaacttt ctcttagcaa tttttctagg cgtaatcatg
ttcttttgcg
2281 gtggtgccct tatgttttat gctggctata ccatcgataa agccgctaca
cgtccagaaa
2341 acattctaat gatttatgta ccaatagttt taatgcgggc tgttggtatt
ggtcgtcctt
2401 tatttagata tttagaacgc ttagtatccc acaactggat tttacgtgtt
actagttctt
2461 taagacgaag actgttttac attgccgaaa gaaatacttc agctgttggt
tcaagctttc
2521 aaacaggaag tattttatcg cttttaactg atgacattgg tcacttgcaa
aatctttatc
2581 taagaactat tttccctgca atcttgagtt acttagttgg tttctttgta
gtaattttgc
2641 tggggctctt ctcctggcct ttagctggag caatcgcaat tttattagtt
gcggaattag
2701 tattggttcc cttcttctcc ctcttaaaac aagctgctgt tcgcactaaa
gagaaagaag
2761 aaaaagcaca actttacact gaatttactg accaagtttt aggagctggg
gactggaaaa
2821 tttctggtag acgggatgcc ttttttaatc aaactaaaga gactttaaaa
tctttaggcc
2881 aacatgaaaa gaagtctgga aagtttgatt gggctagaga cttcggtctt
gagttcatct
2941 ttggcttgat ggcagttgcg cttctttatt tcacgaacca aactcttacc
aataatcaag
3001 aagcagcaaa ctatgttgga gcagttgtct tagctctctt tcctttatca
gatgccttta
3061 ttccagttgg tcaaggaatt gaagaatggc atacatatag cgattctgta
aaacacttaa
3121 atgaattaaa ggtaccagag aatcgtttgc ctgttcagca atatctcgat
cctacttttg
3181 caggtacttt aagagtatcg gatatttcat tcacttatcc taaagaagac
tatccaatta
3241 ttcaaaattt ctctcttact ctaaaaagag gacaaaaagc tgctttaatc
ggcccatcag
3301 gtgctggtaa aagtactata ttgcaattaa tcttaggtga tttaaaacct
aaccaggaaa
3361 ctgtgacact cgaccgttta aacgtactta aacttcaaag agaacgctca
aagttattct
3421 ctgttctaaa tcaagaacca tttttgttta atacaactat ttatgaaaat
ctcaaaatgg
3481 ctaacccaga tgccactaca gatcaaatga tggaaatttt agaaaaggtc
cagctagctg
3541 atttcgttaa ttcacttcct aaagggttag acacagaagt agctgaagct
ggagcgcgtt

3601 tttcaggtgg tcagaaagaa cgtttagcat tagcacgtgt acttttacaa
gacacaccaa
3661 tcgtcctctt agacgagccc actgttggtt tagattctct tacagaacaa
aaacttttaa
3721 atttagtgtt tgaagtttta aaaaataaga cagtagtttg ggtaacgcac
catctacaag
3781 gtgtaaaata tatgaacgaa gttctcttct ttaaagatgg caaagttaca
atgcagggaa
3841 atccgcatga actttttaag cataatgaac atttccacca actttattta
atggatcaag
3901 gactaatcta aaaaatagaa agaagcttac tatgtctgct gaagctaagc
aaggctacta
3961 tgaattagtt gaaccacgta cactatttaa ttcaattatt cccgtattat
tagggatgat
4021 gtatacagaa tataattttc agtttttcag aatttttcct acaattgaaa
tgtgtattgc
4081 cacgattgtt ctacaaattt ttatgaacgt gaatgatggc tattgggatt
ataaacgtga
4141 aaaagctgcc aaaactggcg atcataagaa gaatcccatt ggtaagtatc
accttaatcc
4201 taaacacgtt ttagcttttg tttgggtatt attcatcatt tcagctgttt
gcgctattct
4261 gattggactt caaactaata tctatatttg gattgttgga attatttgtt
atgctattgc
4321 tatttcttat tcaacgggtt cacacacaat ttctgctgga ccgtttggag
agattgcagc
4381 ttgttttgca atgggctttg gtattttctt ggtaatggtt tacatcaatg
tttgttctaa
4441 ggtctccttt aattggaact ttatttatcc aattattctg gctgctggaa
tacctgaaat
4501 ttgtaacttt actttaatgc taggtaacaa tttatgtgat catgacgccg
atattgcaaa
4561 tggtcgccac acactagttt catacattgg aatcaaaggc ggattatatt
tatttgtatt
4621 caattattta cttggtttct ttttaacagg ttgggcaatt tggattggtg
tccttccttg
4681 gagtgtagct ttaattttaa tttgcattcc aaccatctat aagaacatgc
gctttctttg
4741 gaaaattcag acgaagccaa aatcatttcc taaagttgtt caaaatacgc
aagtattgtt
4801 tattactgaa gcggttggtt tctttatcgg tttgatttta aatcttagaa
ttagataact
4861 aaaaaaggtc gaaacttcat cttagtatga agtttcgacc ctttttagtt
atattttaag
4921 ctaaattagt cacttttcct cttaagattt atttttttag taaactataa
aaaaatataa
4981 agaaaaggat ataaacaaac atgaaagtat taaacgagtg tcaactacaa
accgtcgttg
5041 gtggaaaaaa ttggtcagtt gcaaagtgcg gaggaacaat tggtactaat
atagctattg
5101 gtgcatggag aggtgcacgg gctggatcct tttttggtca gccagtttct
gtaggagcag
5161 gtgcactaat cggtgcaagt gctggtgcaa ttggcggatc agtacaatgt
gtgggctggt

5221 tagctggagg tggaagataa tgatcgaaaa agtttctaaa aatgaactaa gccggatata
5281 tggtggaaac aacgtaaatt ggggtagtgt tgcaggatca tgtggtaaag gtgcagtaat
5341 gggaatatat ttcgggaatc ccatattagg gtgcgctaac ggagctgcaa catcattggt
5401 tctacaaact gctagtggaa tatataaaaa ttatcaaaaa aagagatagt atatgacggg
5461 aatatggata gtaattatta gtattattgt gctttcaata attatcacca atatagtagc
5521 tctaatacag acactattac ataagaaaaa tgaaaaatat tattttgata aatcttttgg
5581 agcctatggc gataaaaata atccaaaata tctgtttaat aatgttgaac atcatgattt
5641 tttccatgta ttttatcagt attttatttg ctcatatatt ccatttatag atgtcatttt
5701 ttggttctta ggttcaattt ggtaataatc aaaaaaggtc gaaacttcat cttagtacga
5761 agtttcgacc tttttttatt tcaaattatt tttattgctt ccacgctgca tcaaattttg
5821 ctttacctgc tttaatttga ttatcaacat tttgaccatt cttagctgca tacaaaatct
5881 tagccataat tggatctaat tgactgtagg cagcgtttga gttcttagct actggaatac
5941 tgtacaagtg tttcatagcc ccttctaact tagctggaag cttagtcttg gtgttttctt
6001 tgtattcttt agatttcaca acagaattat taactggaat atagccagtt gcatttgccc
6061 atttaagttg agtagattta gaagttaaaa attgcatata tttaaatgcg gcagttcttt
6121 gcattgctgt tgctttttta aacatgtaaa tatcagtacc ttgctgcata gtatatttac
6181 cagggcgtgc agctacatca taagtaaact tatcaccaac tgccttttta acgtagcctt
6241 cacctgctga tgttccaata tacattgcta ccttttcatt agcaaatgga cctgataaat
6301 agtgtgctga accagcagtt gtaaagtagc ccttcttcat accttgagca tagtaatcaa
6361 taactttctt ggaatctttg ccagtaaagt taactttatc agttaaatta attccttcat
6421 ttttcatacc caaggtatag taattagcta aagaatcaaa gcctgcacca acaacttgat
6481 ggttactctt cttataaatg gtttctgaaa cttgtgctaa ttccttcata gttgtaggaa
6541 ctttttttaat accatatttt tcaaacatgg acttgttata ggttaaaact tcaatagatt
6601 tattaaatgg aataccgtat tgttttcctt gaattttagc tccatccaat aattcagttc
6661 taatatttga cttagcagca cttccccaac cgagcttact gttattaatg tatggggaaa
6721 gatcaaccaa catattactc ttaacagcat tatagagcca accaggatat gcttgcgtaa
6781 ttgttggtaa attatttggt gattgtaatg ttgaatttac cttagcttgt aaatcaatat

6841 aagaaccttg attttcaagt ttgattttaa tattaggatt tttcttttca aattctgcag
6901 ttaatttttc taattctgca cgctgaccac cattcattcc atgccaaaag gttaccgtag
6961 tttttttggt aattttagtt ggaatatttg ctgctgaact atcttcttta tttccttttg
7021 aacatccagt agcaattaat gccaaccctg caactaaacc taaagcaatc tttttatata
7081 aattcatcaa aatcctccag taatttcaaa attgaagcac tatttattgt atcaaagtaa
7141 aaatcatata caagatgttg tagtataaaa ataagatttt ttattttgaa atttttcata
7201 ttttaaattc tgcgtcaaac gcgcattata tatagctttt gtatatataa aatacgtata
7261 tatccgtaca ttataaagaa taatgtttat aattattcat attttgcctt gaattacgat
7321 taatagtctg ctatctttaa atcgtcaact tcattaaata gttctggagg aaaaaatgaa
7381 gataaagaaa tttttgattg gcgccgctat ggttttactt gctgcaacaa gtgcagcttg
7441 ctcgaacagt aaatctgctt ctaaatcaag tgatggttat actcctaagg agttaaacgt
7501 tcaattcgtt cctagtgtcc aagcttctaa gcttgaagct aaagctaaac cattacaagg
7561 tttacttgaa aagcaattgc acatgcctgt tcacgtaact gtttcaactg ataattccgc
7621 tttggttgaa gcaatggcat ctaagaaagt tgatgtcggt ttcttaccac ctgatgctta
7681 tgttttagct cacaagcgtg gtgtagctga cgttttactt caagcacaac gttatggcta
7741 tgacgaacca agcggtaagc aaaatcataa attaatggac agctaccgtt caatgatcgt
7801 tgttaaaaaa ggttctaaga ttaaatcttg gaaagatctt aaaggtaaga caattgccgt
7861 tcaagaccca acctctactt ctggttacgt tcttccaatc gccgaacttc acaagaaggg
7921 cttaaacgta ccaaaggatt gtaagttagt tcaagttaag ggacacgatc aagctgtttt
7981 atcagtttat aacggagacg ctgatgctgc ctttgtcttc tcagacgctc gcccacttgc
8041 tgcaaaagat gcaccagccg taatgaaaga tgttgttcca atctacttta ccaagtacat
8101 tccaaacgat actgtttcag ttagaagtgg tatgtccaag tcatttaaga agaaacttgc
8161 taaagcattc aaagatattg ccaagactaa gaaaggcaag aagatccttg aaaacatcta
8221 ccaacactat ggttatgtag attctaaaga ttctaacttc aacattgttc gtgaatacga
8281 agctgaagct aaaaaagctc aaaaataagt ttaatatttt taaaggccac cctactttga
8341 cttgtaggat ggcctttttc tcgtttaaaa ttagaataga tttttaatcg aggtttttaa
8401 atgaaactaa ataaaaaatt ttctctacta ctaatagcaa gtactatttt tttgagccta

8461 agtgcttgca gcaaaccaac tcatgaagag caaacaaata aaaagctagc caatcacatt
8521 gctttaatct cagataccaa tggaattaaa gataattctc taaatcaatc tgcctggcaa
8581 ggcgttaaga attacggtac taagaataac cttgttgaag gaaataaagg ctacaattat
8641 ttcgttccta aagatcccca agattatcaa gcaactatca acaaagttct agataatgat
8701 ttttcaacaa tcattggcgt tggttataca cttaagccta atattgttaa agccgcgaag
8761 aataatccta aaaagaactt cgttcttatt gatactcatg ctgctaagat taaaaaccta
8821 gctagcatct cctttaaaaa tcaagaagga gcttatttag caggcgttgc agctgcctac
8881 accactaaaa accacacagt gggacttatt ttagggcaaa acagtaaaga gatggattcc
8941 tttaaagccg gttttactca aggagttaca gccagcagtc gtaagcttca taagcatatt
9001 aaagtgatga gcaaaactgt cggtaatttt tcagatatta ataaagcgca tgatattgcc
9061 caggaaatgt acaatcagaa agctgatata atcttccaag ctgcgggtaa aagcggtcaa
9121 ggagtcttta aagctgccaa agaagtcaat caaaccaatc ccgtaggaca aaaagtctgg
9181 gttattggtt cagatgaaga tcaaaccaaa cttggaaatt accaagcaaa aggcggtcaa
9241 ccttctaact ttacccttac ctctgtaatc aaaagcgcag atgtagctgt tgaagatctt
9301 gctaatcaaa ctgctaaagg taatttccct ggtggaaaga acttaaacta tggtcttaaa
9361 aacaagggta tttctctagt acaaggtaat ttatcttatc atacctggat caaagttcag
9421 aaagctaagc agaaaattat tgatggtaaa atcaagatca tggctaatcg ttaaaagatc
9481 ggtagcatca aaattaatac aaactaaaaa gtcacattaa cctatgccaa aaagttactt
9541 gtggcttttt tctttgcttt attttctaca ttactatcta cattacaaaa cacactataa
9601 tatagttgta tagatacaaa gaaagtgtgt ttaagatggc taatgaaagt aaaatagtga
9661 ctcttaaaaa tggttatcat gtatggactc gtaaagaagg acacggtcct attaagattt
9721 tgcttctgca cggcggtcct ggcatggatc atgaatatct tgagcctttt tctaattaca
9781 ttaaaatgca tcccaatata gagattatct actatgatca attgggatca tatttctcag
9841 atcaacctaa tgatccaaag ttatggacaa ttccgcgttt tatagaagaa atagaagaag
9901 tccgtaaagc atgggattta gatcagtttt atttatacgg tcaatcattt ggtggtttat
9961 ttgctttaga atatgccgct tctaaatacg gcaaacatgt gaaagcccta attgattcaa
10021 acatggttga ttcatatcaa gactacgcaa aatatattaa caagtaccgc gaatcgatgg

10081 atcctgctga tgtttcatat atgaagaaac aagaagaacg acacaactat gatgatccac
10141 attatcaaga actattgatg aaattatatc accgctgcat ttgcagaata tctccatggc
10201 ccgatgccgt gcaaagaact tttgatcata tgaatgagca agtttatgtc accctacaag
10261 gaccaacgga atttaacatt actggttcat gtaaaaattg gacaattcgg gatcgtttat
10321 ccaaaattac gatgcctact cttgtattag gaggaaagta tgacagcatg aatccagctg
10381 atattaaagc tttagccgat agattaccta atggtacagc tcatatatgt cctaatggaa
10441 gtcactttt
//
