# Supplementary material for: Cell-free supernatant of Lactobacillus gasseri 1A-TV shows a promising activity to eradicate carbapenem-resistant Klebsiella pneumoniae colonization
Source: Front Cell Infect Microbiol. 2024 Dec 3;14:1471107. doi: 10.3389/fcimb.2024.1471107 (PMC11613640; doi:10.3389/fcimb.2024.1471107)
Supplement: Supplementary file 3 [file DataSheet3.pdf]

```
LOCUS       Contig_2.9.AOI_01           20981 bp    DNA     linear
FEATURES             Location/Qualifiers
     source          1..20981
                     /organism="unkown"
                     /mol_type="genomic DNA"
                     /strain="unkown"
     gene            complement(9923..10018)
                     /gene="sORF_3"
                     /locus_tag="sORF_3"
     CDS             complement(9923..10018)
                     /gene="sORF_3"
                     /locus_tag="sORF_3"
                     /product=""
                     /note=""
                     /translation="LSFFSNHNWTNPFFIYPSLNFIMSDLNPDNK"
     gene            9998..10993
                     /gene="6.3;Bacteriocin_helveticin_J"
                     /locus_tag="orf00018"
     CDS             9998..10993
                     /gene="6.3;Bacteriocin_helveticin_J"
                     /locus_tag="orf00018"
                     /product="6.3;Bacteriocin_helveticin_J"
                     /note="Evalue=5.88e-62 match=45.525%;"
                     /translation="MIGKETQIRLVNKLENIHHVVVQASALDGSNVFVLQLLHRQSDV
                     IVYQTPNDSETVTFNEDHPILYLKGPNSAGTAGGHTQTWVQSGEDNKWFVGTKPKKHG
                     NTYWTTQIARVTVPGYQTQIFTSNTELPRLSYLNRAGSGYGDGSVAYPGKDLVRVEAA
                     VSPNKQYFLIASIDINHTGHFAIYNLDEVNKKLDQAEEKAEDVNIQSLNCLGAFNVPH
                     FNDQKIISIQGYGIDDNKNIYISSQPSPHTTFLGFPKQGKPREIVKIPWGISDPSKWS
                     VVNLDNSLKLDALNFCTEFEGIQVTSDCLYLTVAYHQRNSDLTTLMNRIYQVEKF"
     gene            complement(6995..7102)
                     /gene="sORF_4"
                     /locus_tag="sORF_4"
     CDS             complement(6995..7102)
                     /gene="sORF_4"
                     /locus_tag="sORF_4"
                     /product=""
                     /note=""
                     /translation="MKLLLILGVSAMAVLAANAAIVNKPVPVPVKVRRK"
     gene            11622..12560
                     /gene="orf00020"
                     /locus_tag="orf00020"
     CDS             11622..12560
                     /gene="orf00020"
                     /locus_tag="orf00020"
                     /product="Uncharacterized transporter MTH_1382 OS=Methanot
                     hermobacter thermautotrophicus (strain ATCC 29096 / DSM 10
                     53 / JCM 10044 / NBRC 100330 / Delta H) OX=187420 GN=MTH_1
                     382 PE=3 SV=1"
                     /note="Species='-'<br>match=24.126%|Evalue=1.05e-11<br>Uni
                     Ref:<a href=http://www.uniprot.org/uniprot/sp|O27435|Y1382
                     _METTH>sp|O27435|Y1382_METTH</a>"
                     /translation="MSLLLPTKQVVIMFILMIVGWICYQVKFLHEQTVKDLSKVLLYV
                     VSPCLIINSFRQSFSAARLLQFGLVFLLVIALFIFKIITSEFIFGKNLVKDRQKRTVL
                     RYAGTYTNAGFMGVPLVQAILGTKGVFFAVPYLIAYNIFMWTHGIRMFTQKKQSFRES
                     FRQAVINPNIIAAVIGMILFITQVKLPDVVSDPMNYIANLNTPLSMIVIGTNLGSINL
                     KADWQDKLAWSGVFVRNLFFPIVILGILYALPLPAIAKMTTLIMATCPVAGVVVLFSL
                     LSNFDVKFPTKLMCLSTLGAIITIPLVIFLATLIGL"
     gene            4797..5714
                     /gene="orf00008"
                     /locus_tag="orf00008"
     CDS             4797..5714
                     /gene="orf00008"
                     /locus_tag="orf00008"
```

```
                     /product="Fructose-bisphosphate aldolase OS=Streptococcus
                     pyogenes serotype M18 (strain MGAS8232) OX=186103 GN=fba P
                     E=3 SV=2"
                     /note="Species='-'<br>match=56.055%|Evalue=5.62e-112<br>Un
                     iRef:<a href=http://www.uniprot.org/uniprot/sp|P68906|ALF_
                     STRP8>sp|P68906|ALF_STRP8</a>"
                     /translation="MAYLVNGNDIFKAARENHYAVGAYNTNNLEWTRALLRGAKETRT
                     PLLIQVSTGAAKYMGGYKTVKDLVLNEMDNMDIDVPVILNLDHGDYESAKECIALGYS
                     SVMFDGHNLPTDENLAKTKEIVKLAHERGISVEAEIGKIGENQGADGGELASVEDAKT
                     FVAAGVDKLACGIGNIHGVYPEGWKGLNFDRLKEIAEAVPGEPLVLHGGSGIPQDQIE
                     KAIKLGIAKININTEFQLAFQAATRKYIEDKMDLDKGNKGYDPRKLLRAGTDAITDSM
                     KEMISWMGTAPIDSKESSVKFDEASLNEE"
     gene            9792..9974
                     /gene="sORF_2"
                     /locus_tag="sORF_2"
     CDS             9792..9974
                     /gene="sORF_2"
                     /locus_tag="sORF_2"
                     /product=""
                     /note=""
                     /translation="VVPQFLVNLFNFNTYKTFNRENYIFNLNICLSSHPNQIYVSSLL
                     YLLSGFKSLIIKLREG"
     gene            1775..2728
                     /gene="orf00004"
                     /locus_tag="orf00004"
     CDS             1775..2728
                     /gene="orf00004"
                     /locus_tag="orf00004"
                     /product="D-alanyl-D-alanine carboxypeptidase DacA OS=Baci
                     llus subtilis (strain 168) OX=224308 GN=dacA PE=1 SV=3"
                     /note="Species='-'<br>match=43.478%|Evalue=8.94e-62<br>Uni
                     Ref:<a href=http://www.uniprot.org/uniprot/sp|P08750|DACA_
                     BACSU>sp|P08750|DACA_BACSU</a>"
                     /translation="VQRKKLGDEMRHAKKKSRKKFLWIIGIAIIIICFFLWKNDHGPN
                     GMPSNYHADQVNLNVKAAVAIDAKNENVIYAKNANQSLPIASMTKLLTVYLTLKAIKE
                     KKISWDTTVSPTQEIINLSSNPDYAGVPLSLGQKYTVRELYDAALIKSANNAARMLAI
                     AVSGSETNFLNQMRQQANKWKLYNVKLVTVDGLPEKNKNFLGMTTTIENKMSANDMAI
                     IARKLVTDYPEVLSTTKVAKSDFRNTLMTNSNKMLSGLSDYDPNYPVDGLKTGTTDGA
                     GACFTCTMNKNNKRVITVILGAQNDNERFSETKKLLNYSFN"
     gene            11164..11622
                     /gene="orf00019"
                     /locus_tag="orf00019"
     CDS             11164..11622
                     /gene="orf00019"
                     /locus_tag="orf00019"
                     /product="Probable GMP synthase [glutamine-hydrolyzing] OS
                     =Helicobacter hepaticus (strain ATCC 51449 / 3B1) OX=23527
                     9 GN=guaA PE=3 SV=1"
                     /note="Species='-'<br>match=48.966%|Evalue=2.69e-42<br>Uni
                     Ref:<a href=http://www.uniprot.org/uniprot/sp|Q7VG78|GUAA_
                     HELHP>sp|Q7VG78|GUAA_HELHP</a>"
                     /translation="MLVLESFQSGLSWETILNKRRNFRKAFANFDYHKVAEFNQDDFE
                     RLMQDKGIVRNRLKINAAINNAKILVKLEKENRTFENFLTEFIPKPIMHHPQKMEDIP
                     ASDGLSTQISKEMKKLGFKFVGPVTVYSFLQAVGLINDHLENCSFKNGGR"
     gene            complement(13404..14159)
                     /gene="orf00023"
                     /locus_tag="orf00023"
     CDS             complement(13404..14159)
                     /gene="orf00023"
                     /locus_tag="orf00023"
                     /product="Glycerol uptake facilitator protein-like 5 OS=La
                     ctobacillus plantarum (strain ATCC BAA-793 / NCIMB 8826 /
                     WCFS1) OX=220668 GN=glpF5 PE=3 SV=1"
```

```
                     /note="Species='-'<br>match=66.800%|Evalue=7.43e-122<br>Un
                     iRef:<a href=http://www.uniprot.org/uniprot/sp|F9UUB3|GLPF
                     5_LACPL>sp|F9UUB3|GLPF5_LACPL</a>"
                     /translation="MEHSWMLKYFAEFFGTLILVLFGNGSVANSFLKGTTGNAPDGKA
                     NGGWILVAFSFGFGVMLPAMLFGSISGNHINPAVTVAQAAAGNFPWSQVAPYIICQLL
                     GAICGQLLVLAMYWPHFKESTDPDIVFSCFATSDCTNSKWNGFISEVVGTAVLMFVAI
                     GLYKGMFFKQAVDIANIGVGFLITALVMALGGPTGPALNPARDFGPRLVYSLLPIPNK
                     KGGAHWSYGWIPVIAPTLGAIIGIFLYKIPFGN"
     gene            6575..6907
                     /gene="orf00012"
                     /locus_tag="orf00012"
     CDS             6575..6907
                     /gene="orf00012"
                     /locus_tag="orf00012"
                     /product=""
                     /note=""
                     /translation="MSLTVNLYYTGKNGSARKFAEEMESSGVADRIRQEPGNEKYDYF
                     IPMNDPEMILLIDSWKNQKSLDAHHASPMMKELADLREKYDLHMRVERYISDENGMPD
                     TDQKFIRK"
     gene            12582..13364
                     /gene="orf00021"
                     /locus_tag="orf00021"
     CDS             12582..13364
                     /gene="orf00021"
                     /locus_tag="orf00021"
                     /product="Putative membrane peptidase YdiL OS=Bacillus sub
                     tilis (strain 168) OX=224308 GN=ydiL PE=3 SV=1"
                     /note="Species='-'<br>match=25.000%|Evalue=5.81e-05<br>Uni
                     Ref:<a href=http://www.uniprot.org/uniprot/sp|O05525|YDIL_
                     BACSU>sp|O05525|YDIL_BACSU</a>"
                     /translation="MKKDSYLAISKTNASIKWMLIASIIYLVVYSLAGRLEFQSQSIW
                     MQILYLIIVCVLAITFKKFYKTDKVFIKLPKEGKVKIITIIFCTLVILLYALLGSVVS
                     WAEVLNSMPEMMWGSICVALAAGIGEEVLCRVLLFNLFAKIFENKKYVLVWASLASSI
                     LFGLFHLINLTHGAAINATMQQVFYATAIGLIFSYIHIFTNRIWLCIVMHFLLDLQPN
                     IGTMEAQASPWGLILLIFGTAMIVSLFSIYVFNRRANKVFEH"
     gene            complement(2898..2996)
                     /gene="sORF_5"
                     /locus_tag="sORF_5"
     CDS             complement(2898..2996)
                     /gene="sORF_5"
                     /locus_tag="sORF_5"
                     /product=""
                     /note=""
                     /translation="LHKKSLFLANLERDEKVSRGTTLLDTKYPLIN"
     gene            15944..17395
                     /gene="orf00026"
                     /locus_tag="orf00026"
     CDS             15944..17395
                     /gene="orf00026"
                     /locus_tag="orf00026"
                     /product="Multidrug resistance protein 3 OS=Bacillus subti
                     lis (strain 168) OX=224308 GN=bmr3 PE=1 SV=1"
                     /note="Species='-'<br>match=30.396%|Evalue=2.66e-48<br>Uni
                     Ref:<a href=http://www.uniprot.org/uniprot/sp|P96712|BMR3_
                     BACSU>sp|P96712|BMR3_BACSU</a>"
                     /translation="MIKKQVTMVTFAMVLANVMAGLDSTIINTAIPAIIADLHGIQFM
                     GWIIAIMLLGMSVSTPIWTKVGEKIGNKATFELSLLFFVLGSLFQGLADNMYFFLLAR
                     ALMGIGAGGMGSLPYIMAGFIFDNIKARTKILGYLGAAFSVAAIVGPLVGGYLVDSLS
                     WHWVFYINIPIGLLAILLSLMYYHEGKIRKTPKFDILGSFLIIVGLTLLLLGIQLLGL
                     TKPWSVASLIIAGLVLLVLFFMHEGGHPNPVVPISMFKNRALVGDFLLFIFSWGAFLA
                     INTYLPMWAQGLLGTTALIGGMTLIPNSLVDVVGTLVVNPLKARFNNRTLLSMGLICI
                     LISSLGLALAPQSTNIWWLAIIGTFSGFGVGFIFVLLQIKVQVDASEKNMAPATSLSY
                     LIRILAQTVMAAVYGVIMNVQLAKGVAENSGITMGMLNKLSDASSAKSLPQNLLPTMR
```

```
                     NIFHLGLQEIMWCATALLVIAIGLNFIFNKNSK"
     gene            6974..7084
                     /gene="sORF_1"
                     /locus_tag="sORF_1"
     CDS             6974..7084
                     /gene="sORF_1"
                     /locus_tag="sORF_1"
                     /product=""
                     /note=""
                     /translation="MKNRIIDLFPTNLYRNWNWFIYNSGICSKYCHSANS"
     gene            19283..20815
                     /gene="ABC"
                     /locus_tag="orf00028"
     CDS             19283..20815
                     /gene="ABC"
                     /locus_tag="orf00028"
                     /product="ABC transporter bacteriocin "
                     /note="Species=Lactobacillus acidophilus | | T<br>match=44
                     .400%|Evalue=2.39e-143<br>UniRef:<a href=http://www.unipro
                     t.org/uniprot/Q5FMU6_LACAC>Q5FMU6_LACAC</a>"
                     /translation="MISLLILEFLTSGLIIGVSYINTYQITAIKNRKWQQFIFLITLS
                     LILFIISYAGLNVCQYWIEKQIQQYNHQIRFKIVNHYFYDNKTHSTAQVQNRLTNDLN
                     LIKDSKLAVYTDIPYYLAQIIFASIGLLLFHWSLLIVVLILGTLSFYLPKLLRPAMQA
                     AALKLSQANKQYLDTAEKWLDGLSELKKFSVGSQLSRIMDNASDTLENANIARTGTMQ
                     ELAVLNKATSALLQFALLAVTAILVTNHIVIFGVIVTVESFSSYINVSVKMLATELGQ
                     IHSVDRLSSEVNADTAVVEHTGDLQSPASLITKNVSVEFSNGKIIKFPDLNIKTGEKI
                     LLTGDSGSGKTTLFKVLLGKIKLHTGMINFKNKYGDDINVDKAKIGYIPQDPILFPDS
                     IENNITMFNSKLKNKVLDYVKDVSFESDIRKMPLGLKTELNLQKLNISGGQRQKIVLA
                     RACVHDDDFILIDEGTSAIDKKATLDILKKLLDGPYTVIFIAHNFNEEMNSLFDREIH
                     LT"
     gene            8634..9272
                     /gene="orf00015"
                     /locus_tag="orf00015"
     CDS             8634..9272
                     /gene="orf00015"
                     /locus_tag="orf00015"
                     /product="Uncharacterized sugar epimerase YhfK OS=Bacillus
                      subtilis (strain 168) OX=224308 GN=yhfK PE=2 SV=1"
                     /note="Species='-'<br>match=34.862%|Evalue=1.41e-20<br>Uni
                     Ref:<a href=http://www.uniprot.org/uniprot/sp|O07609|YHFK_
                     BACSU>sp|O07609|YHFK_BACSU</a>"
                     /translation="MIMAKIFIFGGSGRVATDLIKNLVADGNTITAAARHPENIIKLD
                     GVTAEKLDLHADVDDIAKQVKGFDAIYFTAGSRGKDLIQTDAMGAIKTMMAAEKAGVK
                     RYIMLSSMLSLDINSWKKIPSLEDYLAAKFFADTYLMDSTNLEYTILQPGSLIEETGT
                     GKIQLNVEATDSNPIPDVAKTLAAILKYPNTIGKVIPMSSGKTPIDDALKEI"
     gene            complement(9331..9789)
                     /gene="orf00016"
                     /locus_tag="orf00016"
     CDS             complement(9331..9789)
                     /gene="orf00016"
                     /locus_tag="orf00016"
                     /product="Protein YtsP OS=Bacillus subtilis (strain 168) O
                     X=224308 GN=ytsP PE=3 SV=2"
                     /note="Species='-'<br>match=52.083%|Evalue=9.09e-47<br>Uni
                     Ref:<a href=http://www.uniprot.org/uniprot/sp|O34553|YTSP_
                     BACSU>sp|O34553|YTSP_BACSU</a>"
                     /translation="MSATTESNYQLLVKQAEALVDGESDWIANTANISALLFNSLDNV
                     NFAGVYRYENNELILGPFQGKPACVHIAVGKGVCGTTAKERQTQIVKNVHEFAGHIAC
                     DSDSNSEIVIPIFKKNGELWGVFDFDSTKIANFDELDQKYLEAISNVFKF"
     gene            14381..15838
                     /gene="orf00024"
                     /locus_tag="orf00024"
     CDS             14381..15838
```

```
                     /gene="orf00024"
                     /locus_tag="orf00024"
                     /product="Multidrug resistance protein 3 OS=Bacillus subti
                     lis (strain 168) OX=224308 GN=bmr3 PE=1 SV=1"
                     /note="Species='-'<br>match=31.556%|Evalue=4.84e-49<br>Uni
                     Ref:<a href=http://www.uniprot.org/uniprot/sp|P96712|BMR3_
                     BACSU>sp|P96712|BMR3_BACSU</a>"
                     /translation="MVTVALMLGNVMAGLDGTITNTAIPAIVSALHGIQFMGWIVAIY
                     LLGMSVSIPIWTKIGEKITNKLAFEIALGLFVLGSTLEGLAPNIYFFLVARMIMGIGG
                     GGMGSLPYIIAGYVFPNIKKRTQILGYLTASFNGAAILGPLVGGWLIDALSWHWVFYI
                     NIPIGLVALLIALVYYKPVTPKSAPVFDLRGAFLLVSGLIMFLMGIQLLGLTATWIVV
                     GLILLSLVLLIFFFLHEAKAENPIIPLSIFRNRDLNGDLILFATTWGAFIAVNTYLPM
                     WAQALLGMSALMGGMTLIPNSVVEIIASQTVATIQEKIRTFTLVMIGIVTMMISSGGL
                     FLANNHTPLWVLIVVGAFSGIGVGFIFVALQVKVQIDAGMKYMATATSTSYLIRILAQ
                     TVMAAVYGVIMNLALASGINSHSNITMKMMNELSDAKSAKLLPQHLLPEMREIFHSGI
                     HEIMAVSFILLLIATVFNFYFNFKQPNKKIKNYSK"
     gene            1088..1765
                     /gene="orf00003"
                     /locus_tag="orf00003"
     CDS             1088..1765
                     /gene="orf00003"
                     /locus_tag="orf00003"
                     /product="Putative L,D-transpeptidase YciB OS=Bacillus sub
                     tilis (strain 168) OX=224308 GN=yciB PE=2 SV=1"
                     /note="Species='-'<br>match=49.265%|Evalue=2.81e-44<br>Uni
                     Ref:<a href=http://www.uniprot.org/uniprot/sp|C0SP99|YCIB_
                     BACSU>sp|C0SP99|YCIB_BACSU</a>"
                     /translation="MKKISKYQNSVLIIGTILTILVFAIILFPHRNVSLASPNKIVNS
                     KVTKKVAQKVKKVTHKDSNLPYPDPKDLQPAGSWKVKSESKAHPDLVRLNNLWIRVSI
                     KGNRVYIMDGNKPVYTMLASCGVYHNGKSATPTGTYAVEAERGATFFNQSLQVGARTY
                     ISWHGHGTYLFHSVPTDGNNKIMKNEAKKLGKTQASHGCIRLSIPDSKWLYEKLPVGT
                     KVVVKDE"
     gene            17651..19237
                     /gene="ABC"
                     /locus_tag="orf00027"
     CDS             17651..19237
                     /gene="ABC"
                     /locus_tag="orf00027"
                     /product="Bacteriocin ABC transporter "
                     /note="Species=Lactobacillus helveticus (strain DPC 4571)
                     | | T<br>match=39.223%|Evalue=7.15e-125<br>UniRef:<a href=
                     http://www.uniprot.org/uniprot/A8YV79_LACH4>A8YV79_LACH4</
                     a>"
                     /translation="MSFQKLIKTNLLLFIVIIVLEILFAAGSATSSYIIQFAYNQLVK
                     NILLVFLLIIASSVFLSFLSYILSSLATYLFSKQTQKYIHSIRHKLISKYYHDKAPKV
                     AEMENELNSNLQVLTKNYADQSLSIIQSGFLLVTSISSLLLMNWMLTLLAIILSVITL
                     YIPRFTRQKASNATQKVVSRNSKYLLAIEEWFNGLEELRKYTAFNKLDLVMQKVSQNL
                     ETSFVKRKKVISVADFLNGCANSFSQIAITLLAAILFFNHQVTFGVIIAAGNFSSMIL
                     NCLLTITTSLTRIQSVQGLNKQIIESQKLASSHKETNTDEIYSISTHNLSISFKNGET
                     IYFPDIHIERGEKVLLSGDSGTGKSTLFKLILNKLQPTNGQVIFENKYGKEVSPDYAE
                     IGYIPQDGKLFPTSIINNIIMFDNKLKKLVKKAVENNDLSKDIASMPNGLETEIDLDT
                     NNFSGGQKQKIILARNEIHNFSIILADEATSAIDSKSSYRILKNLVSSNKTVIVVAHN
                     LTPAIEKLFSRKISLVNNKR"
     gene            2991..4685
                     /gene="orf00006"
                     /locus_tag="orf00006"
     CDS             2991..4685
                     /gene="orf00006"
                     /locus_tag="orf00006"
                     /product="Arginine--tRNA ligase OS=Lactobacillus johnsonii
                      (strain CNCM I-12250 / La1 / NCC 533) OX=257314 GN=argS P
                     E=3 SV=1"
                     /note="Species='-'<br>match=97.491%|Evalue=0.0<br>UniRef:<
```

```
                     a href=http://www.uniprot.org/uniprot/sp|Q74KR5|SYR_LACJO>
                     sp|Q74KR5|SYR_LACJO</a>"
                     /translation="MQEVLSVDFKQKVVDLVSEQVDLPKEKIAMLIERPKNAKMGDYA
                     FPAFALAKIEHKNPALIAKDIAEKISDDNFTSIQAVGPYVNFAIDHAKLVNATLNDVL
                     TEKDHFGDQQLGEGNVPIDMSSPNIAKPMSMGHLRSTVIGNSIAKTLEKVGYTPIKIN
                     YLGDYGTQFGKLITAYRLWGNEEDVKKDPITNLFHYYVKFHEEAEKDPKLDDEGRAAF
                     KKLENGDEEEIKLWKWFREVSLQEFNRIYKELGVTFDSYNGEAFFNDKMQPVVDELRE
                     KGLLEESRGAQVVNLGEDENPALILKSDGSSLYMTRDLAAALYRKKEYDFVMSLYVAG
                     GEQSGHFKQLKQVLKKMGYDWADNIHHIPFGLITQGGKKLSTRKGNVVFLDKVLKDAV
                     SLAEQQIEEKNPNLANKDQVAHDVGVGAVVFHDLKNDRMDNFDFDLEEVVRFEGDTGP
                     YVQYTNARAQSILRKANKEISMDNLSLNDDWSFAVAKALADFPAIVAKASEKFEPSII
                     AKYALDLSKKFNKYYANVRILDEDDQLNARLALVQATSIVLTEALRLLGVNAPKEM"
     gene            6151..6420
                     /gene="orf00011"
                     /locus_tag="orf00011"
     CDS             6151..6420
                     /gene="orf00011"
                     /locus_tag="orf00011"
                     /product="Probable GMP synthase [glutamine-hydrolyzing] OS
                     =Helicobacter hepaticus (strain ATCC 51449 / 3B1) OX=23527
                     9 GN=guaA PE=3 SV=1"
                     /note="Species='-'<br>match=36.765%|Evalue=1.65e-06<br>Uni
                     Ref:<a href=http://www.uniprot.org/uniprot/sp|Q7VG78|GUAA_
                     HELHP>sp|Q7VG78|GUAA_HELHP</a>"
                     /translation="MKILKIKDLDQEIENKLLVIWESSVRATHNFLSDPEINNIKKYV
                     PQALKGVAHLVIAYNDANESVAFMGINDQKLERLFVAANLTASII"
     gene            7399..7881
                     /gene="orf00013"
                     /locus_tag="orf00013"
     CDS             7399..7881
                     /gene="orf00013"
                     /locus_tag="orf00013"
                     /product="Hypoxanthine-guanine phosphoribosyltransferase O
                     S=Streptococcus mutans serotype c (strain ATCC 700610 / UA
                     159) OX=210007 GN=hpt PE=3 SV=1"
                     /note="Species='-'<br>match=49.686%|Evalue=1.33e-50<br>Uni
                     Ref:<a href=http://www.uniprot.org/uniprot/sp|Q8DWM8|HPRT_
                     STRMU>sp|Q8DWM8|HPRT_STRMU</a>"
                     /translation="MDEMAAELNVKYKDEEPIVVPVLNGAMIFASDMIKRLNFKLTID
                     PIKASSYAGTQSTGEVKITQDIKSDVKDRPVIFMEDIIDTGRTLQALSEVMKGRGAKS
                     VEVVAMLDKPETRVVDFHADYYGFKAPDEFLVGYGLDYNGLYRNLPYVGILKHEVYAK
                     "
     gene            5784..6101
                     /gene="orf00010"
                     /locus_tag="orf00010"
     CDS             5784..6101
                     /gene="orf00010"
                     /locus_tag="orf00010"
                     /product=""
                     /note=""
                     /translation="MRVEFYEKGCKSFFKKYNKQKDTIVEFVEDTIDKEVASGMTKVK
                     LATRKRIKGRNIYEFRLNVGTIGSIRIAFSIFDKKAIVYFISKNIQKSAFSKDFEKII
                     SKL"
     gene            237..1082
                     /gene="orf00002"
                     /locus_tag="orf00002"
     CDS             237..1082
                     /gene="orf00002"
                     /locus_tag="orf00002"
                     /product="ADP-dependent (S)-NAD(P)H-hydrate dehydratase OS
                     =Enterococcus faecalis (strain ATCC 700802 / V583) OX=2261
                     85 GN=nnrD PE=1 SV=1"
                     /note="Species='-'<br>match=47.535%|Evalue=4.56e-85<br>Uni
```

```
                     Ref:<a href=http://www.uniprot.org/uniprot/sp|Q833Y3|NNRD_
                     ENTFA>sp|Q833Y3|NNRD_ENTFA</a>"
                     /translation="MKSISKELMSEVIKKRESATHKGNYGRVLLIGGNKKYGGALIMS
                     AEGALNSGAGLTTVATDSVNISALHTRDPEIMALDWDKRDELKNLIVGSNVVVCGMGL
                     GLDDQARDILALIRDSISLKQVLILDASALDLISQQKDLLPVNSKLVIFTPHQMEWQR
                     LSKIKIADQNDQSNQAFLNELVPRKNAILVLKSNHTKVYDQAGNIYQNPFGNPGMAIG
                     GMGDTLAGIIGGFCGQYTPNLKTVAGAVGIHSITADEIAKKHYIVRPTQLSALIPAMM
                     RKYEK"
     gene            7896..8564
                     /gene="orf00014"
                     /locus_tag="orf00014"
     CDS             7896..8564
                     /gene="orf00014"
                     /locus_tag="orf00014"
                     /product="Putative glutamine amidotransferase-like protein
                      YfeJ OS=Salmonella typhimurium (strain LT2 / SGSC1412 / A
                     TCC 700720) OX=99287 GN=yfeJ PE=4 SV=2"
                     /note="Species='-'<br>match=30.601%|Evalue=5.66e-19<br>Uni
                     Ref:<a href=http://www.uniprot.org/uniprot/sp|P40194|YFEJ_
                     SALTY>sp|P40194|YFEJ_SALTY</a>"
                     /translation="MRVNVLQHTPNEGPGSIKTWADQHHYDFYVYHPETFGKLPTVEE
                     TDLLIIILGGPMSPNDDLIWIKQERKLIKAMLDAHKPMFGACLGGQQIAKTVGAKILDA
                     PHKEVGWAPVYLKDQTIPNLPEKLTALHWHQQMFEIPEGAKLLFSSDLVKNQGFLLGD
                     NVIGLQFHFEPEEDNVREIAINDVDYPLENNDLHQTGEEIIAHGVPKENQKVMFKLLD
                     FITK"
ORIGIN
        1 aactgatttc cttaagccat ttagtgatag cgaactagga aaatatgaaa
       51 attcgatttc tgaagagtta aaggacaatt ttgagcatta tgctttaggc
      101 gttcaaacag atgcacgaga aacagcagct gataaattga caaaatatgc
      151 tgttgaactt gcagaaaagg aaaataacga taaattataa agctgaagct
      201 catcgatttg atgggctttt ttggataaga taaaaatgaa aagtatttca
      251 aaagaactaa tgtctgaggt aattaaaaag cgtgaaagtg ccacacataa
      301 gggtaattat gggcgagtac tattaattgg tggtaataaa aaatatggtg
      351 gtgctctcat tatgtcagct gaaggagcac taaacagtgg tgctggtttg
      401 actacggtgg cgacagattc agtaaatatt agtgccctac ataccagaga
      451 tcctgaaatt atggctcttg attgggataa aagagatgaa ttaaaaaatc
      501 taattgtggg ctctaatgta gtagtttgtg ggatgggact tggtttagat
      551 gaccaggcac gagatatttt ggctcttatt agagatagca ttagcttaaa
      601 gcaagtattg attttagatg caagtgcact tgatttaatt agccagcaaa
      651 aagatttatt gccagtaaac tcaaaattag taatttttac acctcaccag
      701 atggagtggc aaagattgag caaaataaaa attgctgacc aaaatgatca
      751 atcgaatcaa gctttttttaa atgaattagt ccccaggaaa aatgctattt
      801 tagtattaaa atctaatcat actaaagttt atgatcaagc aggaaatatt
      851 tatcagaatc cttttggcaa tccaggaatg gcgattggcg gaatgggaga
      901 cacgttagct ggaatcattg gtggtttctg cggtcaatat acacctaatt
      951 taaagactgt cgcaggtgct gtgggcattc attcaataac tgctgatgaa
     1001 attgcgaaga aacactatat tgttcgaccg acacaactgt cagcccttat
     1051 tcctgcaatg atgagaaaat atgagaagtg aataaaatga aaaagataag
     1101 taaatatcaa aactctgttt taattattgg aacaatacta actattttag
     1151 tatttgcaat tattttattt cctcatcgta atgtatcgct ggcaagtcca
     1201 aataaaatag taaacagtaa agtaacaaaa aaagtagccc aaaaagtaaa
     1251 aaaagttaca cataaagatt ctaatttacc atatccggat cctaaagatt
     1301 tacagccagc tggtagctgg aaagttaaaa gtgaaagtaa ggcacatcct
     1351 gatctggtaa gactaaataa tttgtggata agagtttcaa ttaagggaaa
     1401 tcgtgtttat attatggatg gtaacaagcc agtttacaca atgcttgcat
     1451 cttgcggggt gtatcataat ggtaaatctg caacgccaac tggaacttat
     1501 gctgtcgaag ctgaaagggg agcaacattt tttaaccaga gtctccaggt
     1551 tggcgcacgt acttacatta gctggcacgg ccatggaact tatctctttc
     1601 attcggtccc aacagatggt aataataaaa tcatgaaaaa tgaagcaaag
     1651 aaacttggta aaacccaagc ctcgcatggt tgcattcgct tgagtattcc
     1701 tgattctaaa tggctatatg aaaaattacc tgttggcact aaggttgtag
     1751 taaaggatga gtagactaaa tatgtgcagc gaaagaaatt aggtgacgaa
     1801 atgaggcatg caaaaaagaa atctagaaaa aaatttctgt ggataatagg
```

1851 tatagcaata attattattt gctttttttct gtggaaaaat gatcatggtc
1901 caaatgggat gccgtctaat tatcatgccg atcaagtgaa cttaaatgtt
1951 aaagctgccg tagcaattga tgccaaaaat gaaaacgtca tttatgccaa
2001 aaatgccaat caaagtttgc caattgcttc aatgactaag ttgttaactg
2051 tttacttaac cttaaaagca attaaagaga agaaaatttc ttgggataca
2101 actgttagtc ctactcaaga aatcattaat ttaagttcta atcctgatta
2151 tgcaggtgtg ccacttagtt taggtcaaaa atacactgtt agagaacttt
2201 atgatgcggc attgataaag tcagctaata atgcagctcg tatgctggcg
2251 attgcagtta gcggaagtga gactaatttt ttaaatcaaa tgcgacagca
2301 agccaataaa tggaaacttt ataatgttaa attggtgaca gttgatgggt
2351 taccggagaa aaataaaaac ttcttaggga tgacaacgac aattgaaaat
2401 aaaatgagtg ctaatgatat ggcaattatt gcgagaaaat tagttacaga
2451 ttatcctgaa gttttaagta cgactaaagt agcaaagtcc gattttagaa
2501 atacattaat gactaatagt aataaaatgc taagtggact ttcagactac
2551 gatcctaatt atcctgtcga tgggttaaaa acaggaacaa ctgatggagc
2601 aggcgcttgc tttacttgta caatgaataa aaataataag agagtaatta
2651 cagtgatttt gggtgctcaa aatgataacg aacgcttttc agaaaccaaa
2701 aagttactta attattcttt taattaaaat ttagctatga attcgtttta
2751 atattgcact ttggctaaga ttgctgatat aatcttcatg ataaagagtt
2801 aaaagtagta atattaatct attttcagcg agtctatggt tggtggaagt
2851 taggcaatag agtgatatga aggcgtgttt aactacaatt ttaaaatcaa
2901 ttaataagcg gatactttgt atcaagtaga gtggtaccgc gggaaacttt
2951 ctcgtctctt tctagattag ctaggaagag actttttttta tgcaagaggt
3001 gttatcagtg gattttaaac aaaaagtcgt agatttagtt agtgaacagg
3051 tagatttgcc taaagaaaaa attgcaatgt taattgaaag accgaagaat
3101 gctaagatgg gagattatgc ttttccagca tttgctttgg ctaagattga
3151 acacaagaat ccagctttga ttgcaaaaga cattgcagag aaaattagtg
3201 acgataattt cactagtatt caggcagttg gaccatatgt taactttgcg
3251 attgatcatg ctaaacttgt taatgcaact ttaaatgatg ttttgacaga
3301 aaaagaccat tttggtgatc aacaattagg cgaaggtaat gtaccaattg
3351 atatgtcttc tcctaacatt gctaagccaa tgtcaatggg gcatttacgt
3401 tctactgtta ttggtaattc aattgctaaa actttggaaa aagttggtta
3451 cactccaatt aagattaatt accttggcga ctatggtaca caatttggta
3501 agttgattac agcgtatcgt ttatggggca atgaagaaga tgttaagaaa
3551 gatccaatta ctaatctttt ccattattac gttaaattcc atgaagaagc
3601 tgaaaaagat cctaaattag atgatgaagg acgggctgca tttaagaaac
3651 ttgaaaatgg tgacgaagaa gagattaaat tgtggaaatg gttccgcgaa
3701 gtttccttgc aagaatttaa ccgtatttat aaagaattag gtgtaacttt
3751 tgattcatac aacggtgaag ctttctttaa cgataagatg caaccagtag
3801 ttgacgaatt aagagaaaaa ggattactag aagaatctcg cggagctcaa
3851 gtcgttaact taggtgaaga tgagaatcca gcattaatct taaaatctga
3901 tggctctagt ctttatatga ctcgtgattt agctgctgca ttatatcgta
3951 aaaaagagta tgactttgtt atgtctcttt atgttgctgg tggtgaacaa
4001 agcggtcact ttaagcaatt aaagcaagtt ttgaagaaaa tgggatatga
4051 ctgggctgat aatattcacc acattccatt tggtctaatt acgcagggcg
4101 gtaagaagtt atcaacaaga aaaggtaatg ttgtcttctt agataaagta
4151 ttgaaagatg cggtttcttt agcagaacaa caaatcgaag aaaagaatcc
4201 taacttagct aataaagacc aggtagctca tgatgttggt gtgggtgcag
4251 tagtattcca cgacttaaag aatgatagaa tggataattt tgactttgac
4301 ttagaagaag ttgttcgttt cgaaggggat actggtccat acgttcaata
4351 tactaatgct agagcacaaa gtatcttgcg taaggcaaat aaagaaatct
4401 caatggataa tttgagctta aatgatgatt ggtcatttgc agttgcgaaa
4451 gctttagctg atttcccagc tattgtagca aaagcttcag aaaaatttga
4501 accatcaatc attgccaaat atgcgttaga tttaagtaag aaatttaata
4551 aatactatgc aaatgtgaga attttagatg aagacgatca gttaaatgct
4601 cgtcttgcac tagttcaagc aacttcaatt gttttaactg aagctttgag
4651 acttttaggc gtaaatgcac ctaaagaaat gtaatattta tcaaaataaa
4701 aatattcact gaattttttt gagctaaagt atgttaattt ttgcactaat
4751 gggttaaact aatgatgtgt taaaaattta ggaggtattt ttttaatggc
4801 ttatttagta aatggtaatg acattttcaa agctgctcgt gaaaaccact
4851 atgctgtagg tgcatacaac actaacaacc ttgaatggac tcgcgcactt
4901 ttaagaggtg ctaaggaaac tagaactcct ttattgattc aagtttctac
4951 tggtgctgct aagtacatgg gtggttacaa gactgttaag gatttagttc

5001 ttaacgaaat ggacaacatg gatatcgatg ttccagttat tttgaacttg
5051 gaccacggtg actatgaatc tgctaaggaa tgtatcgcac ttggttactc
5101 atcagttatg tttgatggtc acaacttacc aactgacgaa aacttagcta
5151 agactaagga aatcgttaag ttagctcacg aaagaggtat ctctgttgaa
5201 gctgaaatcg gtaagattgg tgaaaaccaa ggtgccgatg gcggtgaatt
5251 agcatctgtt gaagacgcta agactttcgt tgctgctggt gttgacaagc
5301 ttgcttgtgg tattggtaac atccacggtg tttacccaga aggctggaaa
5351 ggcttgaact tcgatcgttt gaaggaaatc gcagaagctg taccaggtga
5401 accacttgtt cttcacggtg gttctggtat tcctcaagac caaattgaaa
5451 aggcaattaa attaggtatt gctaagatca acattaacac tgaattccaa
5501 ttagcattcc aagctgcaac tcgtaagtac atcgaagaca agatggactt
5551 agacaagggc aacaagggtt acgacccacg taagcttttg agagctggta
5601 ctgacgcaat tactgattct atgaaggaaa tgatttcatg gatgggtact
5651 gcaccaatcg actcaaagga atcatcagtt aagtttgacg aagcttcatt
5701 aaacgaagaa taatcaacta attctattaa aaaagacctc agtttttctg
5751 gggtcttttt tgtttaggtg agataaaaga taatgcgtgt cgaattttac
5801 gaaaagggtt gtaaaagttt ctttaaaaaa tataataagc aaaaagatac
5851 cattgtagaa tttgttgaag acacaattga caaagaagtg gctagcggga
5901 tgacaaaggt taaattggcg accagaaaaa gaataaaggg tagaaatatt
5951 tatgaatttc gtcttaatgt tggtacaata ggttcgattc gaattgcatt
6001 ttcaattttt gataaaaaag caatagttta ctttattagt aagaacattc
6051 aaaaatcagc gtttagtaaa gattttgaga aaataatttc taagttatag
6101 taaaatactt ttaacgcaat acatatttaa aaagtaagaa tttgttacta
6151 tgaaaatttt gaaaattaaa gatctagatc aagaaataga aaataaatta
6201 ttagtaattt gggaaagtag cgtcagagct acgcacaatt ttttatcaga
6251 tccagaaatc aacaatatta agaaatatgt tcctcaggct ctaaaaggag
6301 tagcgcactt agtaattgca tacaatgatg caaatgaatc tgttgccttt
6351 atgggaatca atgatcaaaa gttagaaagg ctttttgttg ctgctaatct
6401 tacggcatca ataatttaga tattaatgag cttgccgtta atgagcaaaa
6451 tccaggagct cgtggttttt atgaacatat gggatttaaa gcagtagaaa
6501 gatctgagtt tgatgatcag ggtaatcctt acccaatttt aattatgaaa
6551 agaatttaga aagaaggatt tagatgtctt taacagttaa cctttattac
6601 actggaaaaa atggaagtgc ccgcaagttt gctgaagaaa tggaaagtag
6651 cggtgtggct gatcgtatta gacaagagcc aggcaatgaa aaatacgatt
6701 atttcattcc tatgaatgac ccagaaatga tacttttgat tgatagctgg
6751 aagaaccaaa aatctttaga tgctcatcat gcttcgccaa tgatgaaaga
6801 attagcagat ttacgtgaaa agtatgatct acatatgcgt gttgaacgtt
6851 atatttctga tgagaatggg atgccagata cggatcaaaa gtttattaga
6901 aaatagtaac agaaaaactc gttgatctat caatcaacga gtttttgcgt
6951 tcaatactaa aaatagagga aaatgaagaa tagaattata gatttatttc
7001 cgacgaacct ttacaggaac tggaactggt ttatttacaa tagcggcatt
7051 tgcagcaagt actgccatag cgctaactcc tagaattaat aataatttca
7101 ttaaaccatt cctttcttta tttgattgat atccatctta aagtgaagtt
7151 gtgaattttt tgtgacttat atttaagaga aaatctaaaa aactataaat
7201 aattttattc cctttataaa tacgaacaat tttgcaatat taagtaaaaa
7251 taattaataa ataagtgtaa aaagactaaa aaaatctctt tatttatgtt
7301 aaaatttcat cattgacgat tttaagtaaa gaggaattac agaatgaata
7351 acgatatcga acgtatattg tgtactcaaa gcgacttaga tagtcgtatg
7401 gatgaaatgg ctgcggaatt aaatgtgaag tataaggacg aagagccgat
7451 tgtcgtgccc gtcttaaatg gagccatgat ttttgctagt gacatgatta
7501 agcgattgaa ttttaagttg actattgatc cgattaaggc ctctagttat
7551 gctggtacgc aatcaactgg agaagtaaag attacgcaag atattaagtc
7601 agatgtaaaa gatcgtccag taatttttat ggaagatatt attgatactg
7651 gtcggacttt acaagcactc agtgaagtca tgaaaggtcg tggagctaag
7701 agcgttgaag ttgttgcaat gcttgataag ccagaaacac gtgtagtaga
7751 ttttcatgct gattattacg gttttaaggc accagacgag tttttagttg
7801 gctatggctt agattataat gggttgtaca gaaaccttcc ttatgtggga
7851 attttaaagc atgaggttta tgctaaataa gggggggata agtaatgcgt
7901 gtaaatgttt tacaacatac tccaaatgaa ggacctggtt caattaaaac
7951 ttgggctgat caacatcact atgatttcta tgtttatcat cctgaaactt
8001 ttggtaaact gccaactgtg gaagaaacag atttattaat aattcttggc
8051 ggaccaatga gccctaatga tgatttaatt tggattaagc aagagagaaa
8101 attaattaaa gcaatgcttg atgcgcataa accaatgttt ggtgcatgtc

8151 ttggtggaca acaaattgca aaaactgtgg gcgctaagat tttagatgct
8201 ccccataaag aagttggatg ggcaccggtt tacttgaagg atcagacaat
8251 tcctaactta ccggaaaaat taacagctct tcattggcac cagcaaatgt
8301 ttgaaattcc agaaggagct aagttattgt tctcaagtga tttagtaaag
8351 aatcaaggct tttacttgg tgataatgtg attggtttac aatttcattt
8401 tgaaccagaa gaagataatg tgagagaaat tgcaattaat gatgttgatt
8451 atccgttaga gaataatgat ttacatcaaa ctggtgaaga aattattgct
8501 catggggtac cgaaagaaaa ccagaaagta atgtttaaat tacttgattt
8551 cattactaaa taaaaagtgt aaattctatg ctactaataa aaagtaagct
8601 aatataaagc tgtaagtttt tcgaggagga aaatgattat ggcgaagatt
8651 tttatttttg gtggttctgg aagagttgct acagacttaa ttaaaaactt
8701 agtagcagat ggcaatacta taacagcagc tgcacgccat ccagaaaata
8751 ttattaaact tgatggtgtt acggcagaaa aattagattt acatgctgat
8801 gttgatgata ttgccaagca agtaaaaggt tttgacgcaa tttactttac
8851 tgctggttca cgtggtaaag atcttattca gacagatgcc atgggtgcaa
8901 ttaagacaat gatggctgct gaaaaggcag gcgttaagcg ctacattatg
8951 cttagttcga tgcttagttt agatattaat tcatggaaga agattccaag
9001 cttagaggat tatttagcag ctaagttctt tgcagatact tatttgatgg
9051 attctacaaa tttggaatat actattttac aaccaggcag tcttattgaa
9101 gaaactggaa caggtaaaat tcaacttaat gttgaagcga cagattcgaa
9151 tccaatccct gatgttgcca aaacattagc agcaattctg aaatatccga
9201 atacaatcgg caaggttatt ccaatgagta gtggtaagac accaattgat
9251 gatgctttga aagaaatcta ataaagagaa aaaagactcc attatgggag
9301 tctttttgtg tgttaaggaa atatgctatt taaaatttaa agacattaga
9351 aattgcttcc aaatattttt gatctaactc atcaaaatta gcaattttag
9401 ttgaatcaaa gtcaaacaca ccccataatt caccattttt cttaaaaata
9451 ggaataacaa tttcagaatt tgaatcacta tcacaagcaa tatgtcctgc
9501 aaactcatgt acatttttta caatttgagt ttgcctttct tttgcagttg
9551 ttccgcaaac tcctttacca acagcaatat gaacgcatgc tggttttccc
9601 tgaaatggac caaggattaa ttcattattt tcatagcgat aaacaccagc
9651 gaagttaaca ttatcaagtg aattaaataa gagtgcagaa atatttgcag
9701 tgttagcaat ccaatcactt tctccgtcta ctagagcttc agcttgtttt
9751 actaatagtt ggtaattgct ttctgttgtt gctgacataa gtggtacctc
9801 aatttttagt aaatttattt aattttaaca cttataaaac atttaataga
9851 gaaaattata tttttaattt aaacatttgt ctttcttcac atcctaatca
9901 gatttatgtt agcagtttac tttatttgtt gtctggattc aaatcgctca
9951 taataaaatt gagagaaggg taaatgaaga aaggatttgt ccaattatga
10001 ttggaaaaga aactcaaata cgtttagtaa ataaattaga aaatatacac
10051 cacgttgttg ttcaagcttc agcactcgat ggaagtaatg tatttgtcct
10101 acaattactt catagacaga gcgatgttat tgtttatcaa actccaaatg
10151 atagcgaaac tgtgaccttt aatgaggatc atccaatttt atacttgaaa
10201 ggacccaatt cagctggtac agcgggtgga catactcaaa cttgggtaca
10251 aagtggagaa gacaataaat ggttcgttgg aactaaacct aaaaagcatg
10301 gtaatactta ttggacaacg caaattgcgc gagtaacagt tcctggctat
10351 caaactcaaa tttttaccag taatacggag ttgccaagac tttcttatct
10401 taatcgcgca ggttcaggct atggcgatgg aagtgtagct tatcctggca
10451 aagatttagt tagagtagaa gcagctgttt caccgaataa acaatatttc
10501 ttaattgcaa gtattgacat caatcacaca ggtcattttg ctatctataa
10551 tcttgatgaa gttaataaaa aactagatca agcagaagaa aaagctgaag
10601 atgttaatat tcagagcttg aattgtttag gtgcatttaa tgtcccgcat
10651 tttaatgatc aaaagattat ttcaattcaa ggctacggaa ttgatgataa
10701 taagaatatt tacatttcta gtcagcctag cccgcataca actttttttag
10751 gatttccaaa acaaggcaaa ccacgtgaaa ttgttaaaat tccctgggga
10801 atatccgatc caagtaaatg gtcagtggta aatttagaca acagtttaaa
10851 attagatgca ctaaactttt gtactgaatt tgaaggaata caagtaacaa
10901 gtgattgtct ttacttaacc gttgcctatc accaacgaaa cagtgatttg
10951 actactttga tgaatcgaat atatcaggtt gaaaaatttt agaaaatgga
11001 cagactattg agtttgtcta ttttgttata attgataaaa atgaggtgta
11051 aaaattgaaa ctaagtcgtt gtagttgggg aaacagtaaa aattctctgt
11101 atcaaaagta tcatgatcaa gaatggggaa agcttaattt agattcaact
11151 tatttatatg aaatgctagt tttagaaagt tttcagtcag gattatcatg
11201 ggagacaatt ttaaataaac gaagaaattt tagaaaagcc tttgcaaatt
11251 tcgattatca taaagtggct gaattcaatc aggatgattt tgaacgattg

11301 atgcaagata aaggaattgt acgcaatcgt ttaaagataa atgcagcaat
11351 taataatgca aaaatattgg ttaaactaga aaaagaaaat cggacttttg
11401 aaaattttct aactgaattt attcctaagc caataatgca tcatccacaa
11451 aagatggaag atattcctgc ttcggatggt ttatctacgc aaatttcaaa
11501 agaaatgaaa aaacttggct ttaaatttgt aggaccagta actgtgtact
11551 ctttcttaca agcggttgga ttaattaatg atcacttaga aaattgtagt
11601 tttaagaatg gaggacgttg atgtctttat tactgccgac aaaacaagta
11651 gtaatcatgt tcattttgat gattgtcggc tggatttgtt atcaagttaa
11701 gtttctacat gaacaaacgg ttaaggatct aagtaaggtc cttttatatg
11751 tagtatctcc ttgtttgatt attaactcat ttcgacagtc cttttcggct
11801 gctagattat tacaatttgg gctagtgttt ttactagtaa ttgctttatt
11851 tatatttaaa attattacta gtgagtttat ttttggtaaa aatttagtca
11901 aagatcgtca gaaaagaaca gtattgcgat atgcaggcac ctataccaat
11951 gcaggcttca tgggagttcc attagtacag gcaattctag gaactaaagg
12001 agtctttttt gcagttcctt atttaattgc ttataacatt ttcatgtgga
12051 cacatggaat tagaatgttc acacagaaaa aacagtcatt tagagaaagt
12101 tttcggcaag ccgtgattaa ccctaatatt attgcggcgg ttattgggat
12151 gattttattt ataacgcaag ttaaattacc tgatgtagtt tcagatccaa
12201 tgaattatat tgctaattta aatactccct taagtatgat cgtaatcggt
12251 acaaatcttg gttccattaa tttaaaagca gattggcaag ataagctagc
12301 ttggagcgga gtgtttgtta gaaacctatt ttttccaata gtcattttag
12351 ggatcttata tgccttacca ttaccggcaa ttgccaaaat gactacgtta
12401 attatggcta cttgtccagt tgcaggtgtt gtagttctct ttagtttact
12451 tagtaatttt gatgtgaaat ttccaacgaa attaatgtgt ttatcgactt
12501 taggagcgat tattactatt cctttagtga ttttttttagc tacgttgata
12551 ggcttataag ctaaagggtg aggaaagata atgaaaaaag atagttattt
12601 agcaatcagt aaaacaaacg catcaattaa atggatgcta attgcgtcta
12651 taatttatct agtggtttat tccctagctg ggagattaga atttcagtct
12701 caatcgattt ggatgcaaat cttgtattta atcattgtct gcgttttggc
12751 aataacgttt aaaaagttct ataagacaga caaagttttt attaaattgc
12801 ctaaagaagg aaaagtaaag atcatcacaa ttattttttg tactctggtg
12851 atcttactct atgctctatt gggtagtgtg gtaagttggg cggaagtttt
12901 aaattcaatg cctgaaatga tgtggggatc catttgcgtt gctttggctg
12951 caggaattgg cgaagaagtt ttatgccgag ttttactatt taatttgttt
13001 gctaaaattt ttgaaaataa aaaatatgtt ttagtttggg cttctttagc
13051 ttcgtcaatc ttattcggat tatttcattt gattaattta acgcatggag
13101 cagctattaa tgctactatg caacaagtat tttacgcaac tgctattgga
13151 ttaatatttt catatattca tatttttact aatcgaattt ggttgtgtat
13201 tgtgatgcac tttttattag atttacagcc caatattgga acgatggaag
13251 cacaggcatc tccatggggg ttaattttat taatctttgg tacagcaatg
13301 attgtttctt tattttctat ttatgtattt aatagaagag ctaataaggt
13351 gtttgaacat taaaaaaagc agatcaactg atctgctttt ttacatactt
13401 aattaatttc caaaaggaat tttgtaaaga aaaattccga taattgcacc
13451 aagagtagga gcgataacag gaatccagcc ataactccag tgtgcgccac
13501 ccttttttatt agggataggt aataatgagt aaacaagtct tggaccaaaa
13551 tcacgtgctg ggttaagagc tggaccagtt ggaccaccaa gagccataac
13601 taatgcagtg attaagaatc ctacaccgat attagcaata tcaacagcct
13651 gtttgaaaaa catacccttg taaagtccga ttgcaacaaa cattaaaact
13701 gcagtaccaa caacttctga aataaaaccg ttccatttgc tgttagtaca
13751 atcgctagta gcaaaacaag aaaatacaat gtcaggatca gtagattctt
13801 taaagtgtgg ccaatacatt gctagaacaa gaagctgacc acaaattgca
13851 cctagtaatt ggcaaattat ataaggagcg acttgtgacc atggaaagtt
13901 accagcagct gcttgagcaa cagtaacggc cggattaata tggttaccag
13951 aaattgaacc aaatagcatg gcaggaagca taacaccaaa gccaaaactg
14001 aaggcaacca aaatccaacc accattagcc ttaccgtcag gtgcattacc
14051 agttgttcct tttaagaaag agttggcaac tgaaccatta ccaaataata
14101 ctaaaattaa agtaccgaaa aattcggcaa aatacttgag catccaacta
14151 tgttccatag atactttttc ctctcttgaa aaaataagtg aaaaataaac
14201 acagctactg attataccta aagttctaga aaaatcctag atattttttta
14251 agattcttgc atttttgagc aaaaagtttg cataatataa agataaatgt
14301 tttagctcta attacttgat gtaataaggg ctctttttat tgttgaggaa
14351 gtgctttaat gaacaagaaa cagatcagga tggtaacagt ggccttgatg
14401 ctgggaaacg taatggctgg actagatgga acgatcacta atactgcaat

14451 tccagcaatt gtatcggctt tacacggaat tcaatttatg ggttggattg
14501 tagcaatcta tcttttagga atgtctgttt caataccaat ttggaccaaa
14551 attggtgaaa aaatcactaa taaattagct tttgaaattg cattaggtct
14601 gtttgtttta gggtcaactt tagaagggtt agcaccaaac atctactttt
14651 tcttagtggc aagaatgatc atgggcattg gtggcggtgg tatgggatca
14701 ctaccttata ttatcgctgg ctatgttttt ccaaatatta aaaagagaac
14751 acaaatttta ggttacttga ctgcaagttt taatggtgca gcaattctgg
14801 gacctttagt gggtggctgg ctaattgatg ccctatcttg gcactgggtt
14851 ttctatatta atattccaat cggattagtt gctttactaa tcgccctagt
14901 ttactacaaa ccggtgacac caaaatctgc accagtcttt gacttaagag
14951 gagcattttt attagttagt ggtttgatta tgtttctgat ggggattcaa
15001 ctcttaggtt taactgcaac ttggattgta gtcggattaa tattattgag
15051 cttagttctt ttaattttct tcttcttaca cgaagcaaag gctgaaaatc
15101 caattattcc attatcaatc tttagaaaca gagatttaaa tggtgatcta
15151 attctatttg caactacctg ggtgccttc attgccgtta acacttattt
15201 accaatgtgg gcacaggcgc ttttagggat gtcagcgtta atgggtggaa
15251 tgactttaat tcctaactca gttgttgaaa ttattgcttc acaaactgtt
15301 gctacaattc aagaaaaaat tagaactttt actttggtaa tgattggaat
15351 tgtcactatg atgatctctt ctggtggatt atttttagca aataatcaca
15401 ctccgttatg ggtattaatt gtagtcggtg ctttttctgg aattggtgtt
15451 ggttttatct tcgtagcact ccaagtaaaa gttcaaattg acgctggaat
15501 gaaatatatg gcgactgcta cttcgacatc ttacttaatt agaatcttag
15551 cgcaaacagt aatggcagca gtatatggtg tgattatgaa tttggcttta
15601 gctagcggaa tcaattcgca tagcaatatt acgatgaaga tgatgaatga
15651 attgagtgat gcaaaatctg ctaaattatt accacaacac cttttgccag
15701 agatgcgtga aatttttcat agtggtatcc acgaaataat ggcagtttct
15751 tttattctat tgttgattgc tactgttttt aatttctatt tcaacttcaa
15801 acagccaaat aaaaaaatta aaaattattc aaaatagttc ttgtttttaa
15851 gtaagtgagc aagtatattt tagatagatt attagccttg attattgcga
15901 aataattggg gctttttatt ttttagaaaa gtaggtgtag acatgataaa
15951 aaagcaagta acgatggtga cgtttgccat ggttttagca aatgtaatgg
16001 caggattaga tagtacgatt attaataccg cgattcccgc cattattgcg
16051 gacttacatg gaatccaatt tatgggctgg attattgcaa ttatgctctt
16101 agggatgtca gtttcaaccc caatttggac caaagtcggt gaaaaaattg
16151 gtaataaagc cacctttgaa ttatcgttac tctttttgt cttaggctct
16201 ctttttcaag gattggctga taatatgtat ttcttccttt tggcacgtgc
16251 tttaatgggg atcggtgctg gaggaatggg atcgctgcct tatattatgg
16301 ctgggtttat ttttgataat attaaggcaa gaaccaagat tttaggttat
16351 ttaggtgctg cttttagcgt tgcagcgatt gtgggaccac tagtcggtgg
16401 ctatttagta gattctcttt cctggcactg ggtcttttat attaatattc
16451 caattggtct tttagcaatt ttattatcgt taatgtatta tcacgaaggc
16501 aagatcagaa aaacgcctaa gtttgatata ctgggttctt ttttaatcat
16551 tgttggatta actttgcttt tattagggat tcagttgctt ggtctaacta
16601 agccatggag tgtagcaagt ttaattattg ctggtttagt gttattagtt
16651 ttattcttta tgcatgaagg agggcacccc aatcctgtag taccgattag
16701 tatgtttaaa aatcgagctt tagtcggtga tttttactc ttcattttta
16751 gctggggtgc atttttagca attaatacat atttaccgat gtgggcgcaa
16801 ggtttgcttg gcacaactgc gttaattggt ggaatgacct taattccgaa
16851 ttccttagtt gatgtggtag gaactttagt ggttaatccc ttaaaggcgc
16901 gttttaataa tcgaacacta ctttcgatgg gattaatttg tattttgatt
16951 tcgtccttag gactagcact ggcaccgcaa agtactaata tttggtggtt
17001 ggcaattatt ggaacttttt ctggctttgg ggtggggttt atttttgtat
17051 tgcttcaaat taaagttcaa gttgatgcta gtgagaaaaa catggcgcct
17101 gcaacatcac tttcatattt aattagaatt ctggctcaaa ctgtcatggc
17151 tgctgtttat ggtgtgatta tgaatgtaca attggccaag ggagttgctg
17201 aaaattcagg tataacgatg gggatgctta ataagttaag cgatgcttca
17251 tctgctaaaa gcttgccaca gaatttatta ccaacaatga gaaatatttt
17301 tcatttagga ttgcaagaaa ttatgtggtg tgccactgct ttgcttgtaa
17351 ttgcgattgg attgaatttt atttttaata aaaatagtaa ataagaaaaa
17401 agctggtaga cgaatcctaa agttggaatt gtctatcagc tttttttgta
17451 gtattaaatc gctgtagtct aaacactact tagtgctggc actaataaat
17501 taaagaaata ttgacatttt aataaaaaaa taatattatt gcggtaattt
17551 ctaaatggtt atctccaact aacagatatg tatatttatc agttccctta

17601 gaaaaagcgg acgctattaa aaataattat aggatctaga tgattaaaaa
17651 tgagttttca aaaattaatt aaaactaatt tgctcttatt cattgtaata
17701 attgttttag agattttgtt tgcagcagga tctgcaacaa gttcgtatat
17751 tatacaattc gcttataatc aactagttaa aaatattctt ctagtttttt
17801 tgctaattat tgccagtagt gtctttcttt cattcctttc ttatattttg
17851 agctctttag ctacatatct ttttagtaag caaacgcaaa aatatattca
17901 cagtattagg cataaattaa taagcaaata ctatcatgac aaggcgccta
17951 aagtagctga aatggaaaat gaattaaata gcaatttaca agtgctgact
18001 aaaaattatg ccgatcagtc tttaagtatt atccaatcag gtttttttatt
18051 ggttacgtca attagcagcc ttcttctaat gaattggatg ctgacattgt
18101 tagcaataat tctatcagtt attacgcttt atattccccg ctttacaaga
18151 cagaaagctt ctaatgcaac gcaaaaagta gttagtagaa atagtaaata
18201 cttgttagct attgaagaat ggtttaatgg cctagaagaa ttaagaaaat
18251 atactgcttt taataaatta gacttagtca tgcaaaaagt tagtcagaat
18301 ctagaaacaa gtttcgtaaa aagaaaaaag gtaatttctg ttgctgattt
18351 ccttaatgga tgtgctaatt ctttttctca aattgcgatt actctgttag
18401 ccgcaatttt gtttttaat catcaagtaa cttttggagt aataatagca
18451 gcaggtaact tctcctctat gattttaaat tgtttattga caattactac
18501 atcgctaaca agaattcagt cagttcaagg tcttaacaaa caaattattg
18551 aatcccagaa gttagcctct tctcataaag aaactaatac tgatgaaata
18601 tactcaatta gtacgcataa tttaagtatt tcatttaaaa atggagaaac
18651 gatctatttc ccagatattc atattgaaag aggagaaaaa gtgttgctta
18701 gtggcgacag tggtactggt aagtctacgc tatttaagtt aattcttaat
18751 aaattacaac caactaatgg acaagttatt tttgaaaata aatatggtaa
18801 agaagttagt cctgattatg ctgagatagg atatattcct caagatggga
18851 agctttttcc tacttctatt atcaacaata ttattatgtt cgataataaa
18901 ttgaaaaaac tggttaaaaa agcagttgaa aataatgatt taagtaaaga
18951 tatagctagt atgccaaatg gacttgaaac ggaaattgat ttagatacga
19001 ataattttc tggaggtcaa aaacagaaaa ttattttagc tcgtaatgag
19051 atacataatt tttctattat tttagctgat gaggcaacca gtgcgataga
19101 ttcaaaaagt agctatcgaa ttctaaaaaa cttagtatca tcgaataaaa
19151 cagttattgt agtagctcat aatttaactc cagccattga aaaactattt
19201 agtagaaaga ttagccttgt gaataataag aggtgactat aagtgtcttt
19251 taaagatcta tatgctgtta atagaataag aatgatatcg ctattaattc
19301 tagaattcct aacttctggt ttaataattg gggtcagcta tattaataca
19351 tatcaaataa cagccattaa gaaccgaaaa tggcaacaat ttattttctt
19401 gattactcta tctttaattt tgtttattat aagctacgct ggtcttaatg
19451 tttgtcagta ttggattgaa aaacaaattc agcaatacaa tcatcaaata
19501 agatttaaaa tagtaaatca ttatttttat gataataaaa cgcatagtac
19551 tgcccaagtt cagaatcgat taactaacga cctgaactta ataaaagatt
19601 caaagcttgc agtatatacg gatattccat actatttagc gcaaattatc
19651 tttgcatcaa ttggcttgct gctgtttcat tggagcctat taattgttgt
19701 tttgatatta ggaacgctta gtttttactt gcctaaactt cttcgtcccg
19751 ccatgcaagc agctgctcta aagttatctc aagcaaataa gcaatatttg
19801 gatacagctg aaaaatggtt agatggtcta tcagagctca aaaagttttc
19851 agtaggttca cagctgtcta ggattatgga taatgcttcc gatacattag
19901 aaaacgcaaa catagcgaga acaggtacta tgcaagagtt agccgtatta
19951 aataaggcta cttccgcctt gttacaattt gctttattag ccgttacggc
20001 tatattagtt acaaaccata tcgttatatt tggtgtaata gtaactgttg
20051 aaagctttag ctcttatatc aatgtatcag ttaagatgct tgcaactgaa
20101 ttaggacaga ttcactcagt agaccgttta agcagtgaag ttaatgcgga
20151 cacagctgta gttgagcata cgggcgactt acaatctcca gctagtttaa
20201 ttacaaaaaa tgtgtcagta gaattttcta acggtaaaat aataaaattt
20251 cctgatttaa atataaaaac tggtgagaaa attctgttga ctggtgactc
20301 aggatccgga aaaactactt tatttaaagt acttcttggt aagattaaac
20351 ttcataccgg tatgatcaat tttaaaaata aatatggtga tgatattaac
20401 gtcgataagg ctaagatagg atatatccca caagatccga ttttatttcc
20451 tgatagtatc gaaaataata ttacgatgtt taattcaaaa ttgaaaaata
20501 aagtgctaga ttatgttaaa gacgtttctt ttgaatctga tattagaaaa
20551 atgccgctag gtctaaagac agaattaaat ttgcaaaaat taaatatttc
20601 tggaggtcag cgacaaaaaa ttgtgttagc tcgggcttgc gttcacgatg
20651 acgattttat tttgattgat gaggggacta gcgctataga taaaaaagca
20701 acgcttgata tcttaaagaa gttattagat ggcccataca ctgtgatatt

```
20751 tatagctcat aattttaatg aagaaatgaa cagtctcttt gatagagaaa
20801 tacacttaac ttagatgcgt tttttgtatt attaattatt tgcttttttct
20851 atttttccaa ctgcaattgg aagagtaaca tgtggaccat attttactac
20901 atttatattg aaaggaacca ttgttgtgcc tgacactagc tcagtttctg
20951 gtaaatcaac aaatccatct ccatttaagt
//
```
