# Supplementary material for: Cell-free supernatant of Lactobacillus gasseri 1A-TV shows a promising activity to eradicate carbapenem-resistant Klebsiella pneumoniae colonization
Source: Front Cell Infect Microbiol. 2024 Dec 3;14:1471107. doi: 10.3389/fcimb.2024.1471107 (PMC11613640; doi:10.3389/fcimb.2024.1471107)
Supplement: Supplementary file 4 [file DataSheet4.docx]

**Supplementary Material**

**Supplementary Table S1.** Percentage values and statistical significance for the auto-aggregation and co-aggregation abilities of the examined strains, where ns was assigned for p. value > 0.05, * for p. value ≤ 0.05, and ** for p. value ≤ 0.01.

| **Selected strains** | **Auto-aggregation (%)** | **Co-aggregation (%)** | **Significance** |
| --- | --- | --- | --- |
| *K. pneumoniae 1* | 62.7 ± 11.2 | 74.6 ± 5.6 | * |
| *K. pneumoniae 2* | 54.3 ± 10 | 73.6 ± 2.9 | ** |
| *K. pneumoniae 3* | 57.9 ± 8.4 | 71.9 ± 3.1 | * |
| *K. pneumoniae 4* | 66.4 ± 3.7 | 64.4 ± 10.2 | ns |
| *K. pneumoniae 5* | 65.3 ± 2.4 | 65.1 ± 4.6 | ns |
| *K. pneumoniae 6* | 65.1 ± 7.3 | 68.2 ± 5.4 | ns |
| *K. pneumoniae 7* | 52.4 ± 8.2 | 67.8 ± 1 | * |

**Supplementary Table S2.** Quantification of metabolites identified by ^1^H-NMR in cell-free supernatant of *Lactobacillus gasseri* 1A-TV, grown in MRS. Metabolites expressed as mM, referred to the quantities of substances found in the samples after subtracting the quantities of the components present in the MRS culture medium.

| **Molecule (mM)** | |
| --- | --- |
| **ethanol** | 55.68 |
| **lactate** | 46.62 |
| **acetate** | 16.61 |
| **glucose** | -38.17 |
| **pyruvate** | -0.25 |
| **formate** | -0.16 |
| **acetone** | -0.10 |
| **valine** | 0.13 |
| **isoleucine** | 0.01 |
| **alanine** | 0.53 |
| **acetone** | -0.10 |
| **pyroglutamate** | -0.30 |
| **methionine** | -0.02 |
| **choline** | -0.01 |
| **threonine** | -0.73 |
| **tyrosine** | -0.05 |
| **phenylalanine** | -0.10 |
| **uracil** | -0.04 |
| **tryptophan** | -0.02 |
| **uridine** | -0.04 |
| **leucine** | -0.39 |
| **adenosine** | -0.07 |
| **succinate** | 7.88 |
| **malate** | -1.90 |
| **citrate** | -2.29 |

**Supplementary Table S3.** Viable count of CFS-treated and untreated K. pneumoniae strains for time-killing assays.

| **Viable count (CFU/mL)** | | | | | | |
| --- | --- | --- | --- | --- | --- | --- |
|  | CFS | T_0_ | T_4_ | T_6_ | T_8_ | T_24_ |
| ***Kbp1*** | CFS-treated | 5,63E+05 | 5,67E+04 | 3,20E+04 | 2,92E+04 | 0,00E+00 |
|  | Untreated | 5,63E+05 | 1,41E+08 | 3,66E+08 | 4,80E+08 | 4,20E+09 |
| ***Kbp2*** | CFS-treated | 4,97E+05 | 1,70E+05 | 8,69E+04 | 2,45E+04 | 0,00E+00 |
|  | Untreated | 4,97E+05 | 1,42E+08 | 3,36E+08 | 4,87E+08 | 1,61E+09 |
| ***Kbp3*** | CFS-treated | 7,03E+05 | 3,67E+05 | 1,96E+05 | 6,31E+04 | 0,00E+00 |
|  | Untreated | 7,03E+05 | 1,07E+08 | 1,39E+08 | 2,29E+08 | 1,14E+09 |
| ***Kbp4*** | CFS-treated | 5,23E+05 | 1,80E+05 | 1,87E+05 | 6,59E+04 | 0,00E+00 |
|  | Untreated | 5,23E+05 | 1,60E+08 | 2,63E+08 | 5,47E+08 | 1,35E+09 |
| ***Kbp5*** | CFS-treated | 3,63E+05 | 1,74E+05 | 9,63E+04 | 9,33E+04 | 0,00E+00 |
|  | Untreated | 3,63E+05 | 2,67E+08 | 3,90E+08 | 5,16E+08 | 1,47E+09 |
| ***Kbp6*** | CFS-treated | 1,01E+06 | 1,87E+05 | 1,05E+05 | 3,69E+04 | 0,00E+00 |
|  | Untreated | 1,01E+06 | 2,65E+08 | 2,81E+08 | 5,80E+08 | 1,75E+10 |
| ***Kbp7*** | CFS-treated | 1,69E+05 | 1,68E+04 | 1,26E+04 | 2,43E+03 | 0,00E+00 |
|  | Untreated | 1,69E+05 | 1,28E+08 | 1,77E+08 | 4,93E+08 | 5,33E+08 |

**Supplementary Table S4*.*** Inhibition of biofilm formation by CFS at scalar concentrations for biofilm producer strains, expressed in percentage values.

| **CFS percentage** | **Kbp1** | **Kbp5** | **Kbp6** |
| --- | --- | --- | --- |
| 50% | 99% ± 0,013 | 99,67% ± 0,005 | 100,00% ± 0 |
| 25% | 99,67% ± 0,005 | 98,68% ± 0,022 | 99,98% ± 0 |
| 12.5% | 98,42% ± 0,021 | 95,44% ± 0,078 | 98,78% ± 0,013 |
| 6.25% | 95,58% ± 0,052 | 92,72% ± 0,081 | 93,02% ± 0,015 |
| 3.12% | 94,47% ± 0,047 | 89,47% ± 0,102 | 90,88% ± 0,014 |
| 1.56% | 92,89% ± 0,045 | 91,65% ± 0,085 | 86,01% ± 0,020 |

**Supplementary Table S5.** Viable counts at scalar concentrations of CFS for each strain.

| **CFS percentage** | **Kbp1** | **Kbp5** | **Kbp6** |
| --- | --- | --- | --- |
| 50% | 1,21E+03 | 8,30E+02 | 9,20E+03 |
| 25% | 3,75E+05 | 2,36E+06 | 1,90E+06 |
| 12.5% | 496E+08 | 4,70E+08 | 9,58E+08 |
| 6.25% | 1,41E+09 | 5,16E+09 | 4,66E+09 |
| 3.12% | 8,83E+09 | 2,29E+10 | 1,62E+10 |
| 1.56% | 8,13E+09 | 2,68E+11 | 1,03E+11 |
| free | 1,60E+10 | 4,44E+11 | 2,89E+11 |

***
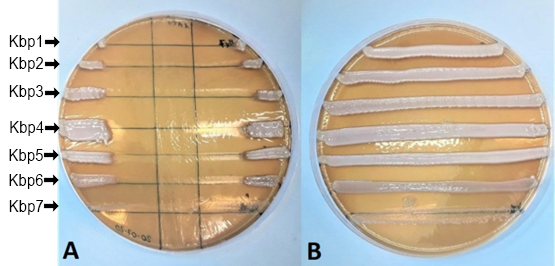
***

**Supplementary Figure 1. A**) Deferred antagonism assay: *K. pneumoniae* growth on an agar plate previously inoculated with *L. gasseri* 1A-TV; **B**) *K. pneumoniae* control growth without 1A-TV pre-treatment.

***
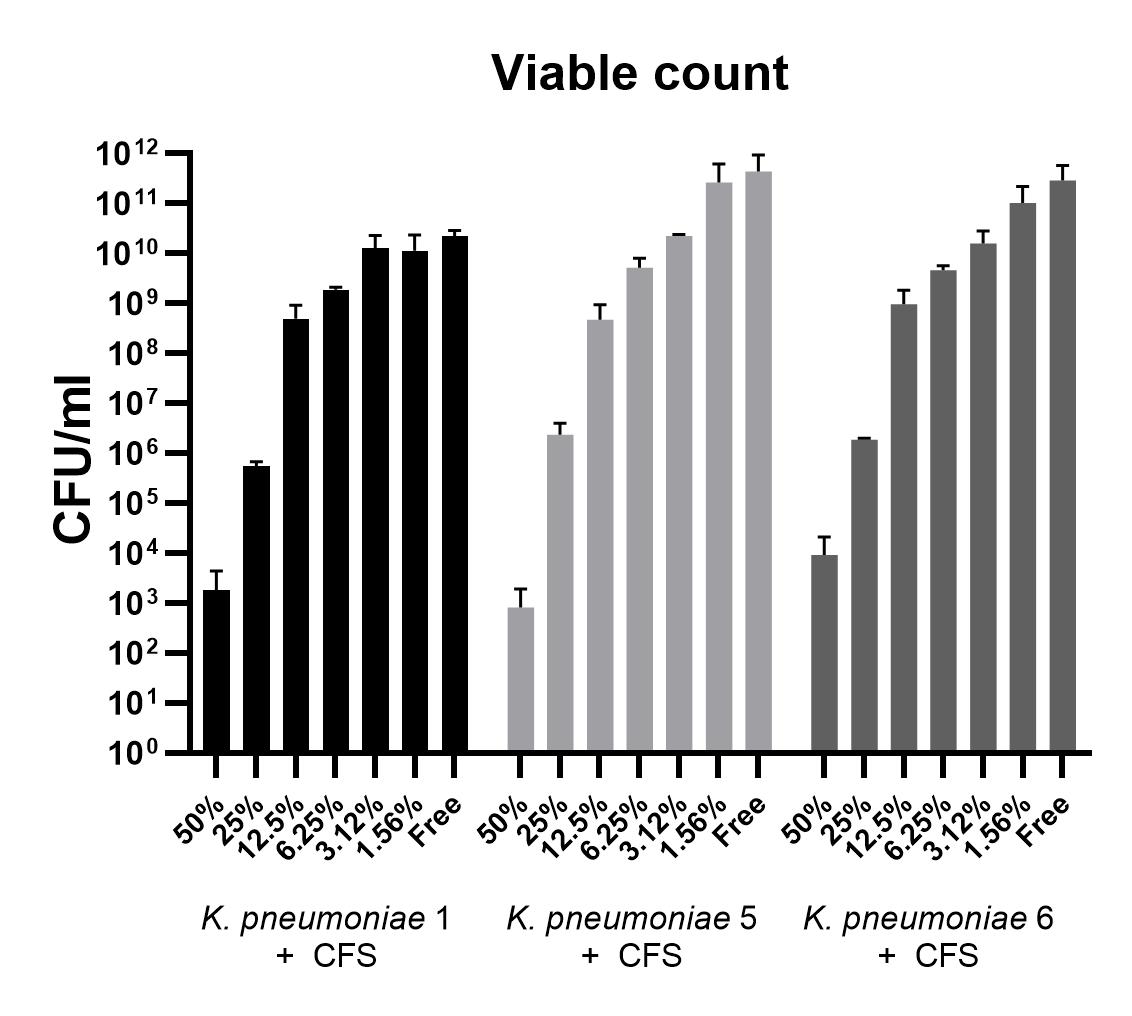
***

**Supplementary Figure 2.** Viable count of biofilm producers after CFS treatment overnight.

**Supplementary Data Sheet S1.** Genome annotation with Prokka v 1.13.

**Supplementary Data Sheet S2.** Genetic organization of Bacteriocin Biosynthetic Gene Cluster BBGC1.

**Supplementary Data Sheet S3.** Genetic organization of Bacteriocin Biosynthetic Gene Cluster BBGC2.
