# Supplementary material for: Cell-free supernatant of Lactobacillus gasseri 1A-TV shows a promising activity to eradicate carbapenem-resistant Klebsiella pneumoniae colonization
Source: Front Cell Infect Microbiol. 2024 Dec 3;14:1471107. doi: 10.3389/fcimb.2024.1471107 (PMC11613640; doi:10.3389/fcimb.2024.1471107)
Supplement: Supplementary file 1 [file DataSheet1.pdf]

```
LOCUS       Contig_1              593801 bp    DNA     linear       18-
OCT-2022
DEFINITION  Genus species strain strain.
ACCESSION
VERSION
KEYWORDS    .
SOURCE      Genus species
  ORGANISM  Genus species
            Unclassified.
COMMENT     Annotated using prokka 1.12 from
            https://github.com/tseemann/prokka.
FEATURES             Location/Qualifiers
     source          1..593801
                     /organism="Genus species"
                     /mol_type="genomic DNA"
                     /strain="strain"
     CDS             complement(319..1719)
                     /gene="yhdG_1"
                     /locus_tag="OCPHGMPK_00001"
                     /inference="ab initio prediction:Prodigal:2.6"
                     /inference="similar to AA sequence:UniProtKB:O07576"
                     /codon_start=1
                     /transl_table=11
                     /product="putative amino acid permease YhdG"

/translation="MKNFFKKVPVNTFLKADSRLTRHLNARDLVALGIGAVIGTGIFI

LPGHEAAQHAGPAVAISFLLAAIVSGMVGMAYAEFSSAMPVAGSAYSFGSVIYGEIVG

WIIGWGLLLEYFLAVSAEATGFASYFNNNILAPIGIHLPKALEAGPMEGGVINLSAVL

IVLIVALILYQGANLSKRVENIAVIIKVAIIILFIVIGLFYIKAENYVPFYPKKFQTP

PLGMGGISTAAATVFFAFIGFDALAANSAETKDPAKNVVKGIMGTVIIAVLLYVSFSL

VLTGMVNYKRLNVDDPAAYALKVVGLTAWNKLITVGALVGIFTAMITMLLGGSRLLYA

LGRDGLLPDSMGSVHAKKMIPDHAVIVSTIVAAIFAGLVPLMELASLINAGTLIAFAL

ISFGIIPLRHRKNIVNNGFKMPFYPVLPIISGLLSVYFIWMLPKMTKIMVGIWLIAGI
                     VVYVTYGVRHSKLQKQ"
     CDS             complement(1851..3968)
                     /gene="clpE_1"
                     /locus_tag="OCPHGMPK_00002"
                     /inference="ab initio prediction:Prodigal:2.6"
                     /inference="similar to AA sequence:UniProtKB:Q9S5Z2"
                     /codon_start=1
                     /transl_table=11
                     /product="ATP-dependent Clp protease ATP-binding
subunit
                     ClpE"

/translation="MAYFDNGNYNFDDLFNELNRDFFNDGRMTNGQQPMRYSAGQAPQ
```

QGAQNGQQQKEKPIGVDLTEQAKKGKYDPVIGRTAVIDQVIEILSRRKKNNPVLTGPA

GVGKTSVVEGLAQRIVDGDVPEKLKNAHIIELNINELVAGTSLRGSFEEKLKKIIDKA

KGDKNVILFIDELHNIVGAGSTDSENNSGDAANILKPALASGEIRVIGATTTSEYQQI

EKDPALARRFQPVQVPEPTIEDAVQILKGLAPRYEKYHNVKYDPEALKLAAELSNRYI

NDRHLPDKAIDLMDEAGAKKSLGMDQKDETSIKNKIHALEGKKAEAAKNEKYDEAAKL

KKEIEDLKKDLKTAGKASELKVTPEDMYSLIQTKTGIPMSEINANEAQKNANLAADLK

KVVIDQDKAIDIITDAIARKQVFKDSNRPTGSFLLTGPTGVGKTELAKQLAIKLFGKA

DALVRLDMSEYQDQMAVNKLIGSAPGYVGYGEGGQLTEKIRHQPYSLILLDEIEKANP

QVFNALLQIMDDGRLTDAQGRTISFKDTIIIMTSNAGYSDKLLKGDDQDALRKALEVY

FRPEFLNRLDAIVPFNSLTSEDMLKILDLYLAKMEKSLAKRNIKLHVTADAKKYLAKK

GYDKEFGARPLRRVVEQDLETPAAKLIVSKPDTKEVKFTIDDKHLYLNGEVLEDVLFD
KETREQEKKDVKEAEKADKKENK"
CDS complement(4222..5136)
/locus_tag="OCPHGMPK_00003"
/inference="ab initio prediction:Prodigal:2.6"
/codon_start=1
/transl_table=11
/product="hypothetical protein"

/translation="MLNKKRVSKNRLLLLGTFIFLIVLSIPTFFWIKSANHDLAERRK

SQMSPVIMIPGSSATTERFNELVNLLNKDTFKKHSLLKIQVKKDGTLKYSGKINRNDN

EPFIVVGFENNHDGYANIKKQAGWFDEAFAQLSQQYKFNNFKAFGHSNGGLIWTYWLE

NYYDDYSDEIRIKKLMTLGTPYNFNESNIDHKTEMLNDFIKNRKKIPQNLDVYSVSGG

QNYESDGLVPESSVAAGKYIFQNQVKHYTTMTVTGADAQHSSLPQNKQVVQLIEQYLL
ESTIGDNTQGNGQRPRGSRKKQDQKLNN"
CDS complement(5126..6445)
/gene="lacE_1"
/locus_tag="OCPHGMPK_00004"
/inference="ab initio prediction:Prodigal:2.6"
/inference="similar to AA sequence:UniProtKB:Q99S77"
/codon_start=1
/transl_table=11
/product="PTS system lactose-specific EIICB
component"

/translation="MEKIIFKTIVRLRRLAFFRVADRTLMMLMPLAVVGSIFQFLWQS

VFSPTSLISNIFYFDKWLPDHLFNAAWYSCQGITSVIFGTFGLFTAYFSAQYTARLYQ

```
KDAQMAGVSGMLALLLCAYRFRDTRDFQLSFNWRFLNINSFLFALLIGFGTGLIFRFL

GVEYHHRHTESAQQIKQRAFDSFRPMLATWVIGLIVGILTSLVHVRVVATNFYQFFQN

QGQNNLNLGVFIPLLLLALLLNWLGIGQPLASLTAGSDSATNVANLNYALTHGSSWNV

PNPYIGNSLYQSYGKFGGSGLTLVLLIGILIFIKKSSIVRVARWSFIPTLFGSNQGAF

LGIPIMLNPLFLFPYVILPVMNMLLAASMIAVHLVPASAYNVLSGTPGPLVAFIATNG
                     TWQALVFSMLLFALDILLYLPIIKMSKDVQDEIDLLNDKEAGYKHVK"
     CDS             6613..6849
                     /locus_tag="OCPHGMPK_00005"
                     /inference="ab initio prediction:Prodigal:2.6"
                     /codon_start=1
                     /transl_table=11
                     /product="hypothetical protein"

/translation="MAKRSFSINDETDRLLNYYLNRSEKDWDAVINEAIKYYICDHLT
                     QKQVHEAIKHTGKDAFPADEVLRNFNSSWMNGDA"
     CDS             6967..8166
                     /locus_tag="OCPHGMPK_00006"
                     /inference="ab initio prediction:Prodigal:2.6"
                     /codon_start=1
                     /transl_table=11
                     /product="hypothetical protein"

/translation="MLKKHALDPIKLIWLFAGSLIINTGVSFIWPLTTIYIHNYLHET

LTIAGIVLFINSAFTMVGNALGGFLFDKWHPYQTLLTGVSISTLSTFLLVLFHGWPAY

PILLITLGLGNGIVVTGLNSIATLIRSRNASYVFNVLYFTQNLGLVFGSLMVGFILPF

GITYIFLLAFIMFAFLSIVVFLEYRGLNQAHAAKGKKVAESEYQEKIPYGAKKAIFSI

LICVLAAWIAYEQWNSNISSYMLGLHMSVRLYSLLWTLNAVLIVLIQPLLTYFDDWLT

QHLHGRLYIGFSLFGLAFLLLIGANHYFSFVLAMAVLTCGEILAFPAVSTFVNDRASN

KDKGKYQGIVQSVTSAGRALGPLIGALVIDNFSYLVLFIFCTVLVLISVLLFAMINTY
                     NKKKIAK"
     CDS             complement(8405..9331)
                     /gene="pbpX_1"
                     /locus_tag="OCPHGMPK_00007"
                     /inference="ab initio prediction:Prodigal:2.6"
                     /inference="similar to AA sequence:UniProtKB:O31773"
                     /codon_start=1
                     /transl_table=11
                     /product="Putative penicillin-binding protein PbpX"

/translation="MAIKNNKVILNESTGYANISKKVRNTPKTAFFINSVNKVFTGTL

VMKQVERKKLKLSDKLSKFYPQVPHANQITIEQLLTMEAGLQGKDESNYGTPVFKNNQ
```

```
AGIKYDIKHNVIFDKRHYNQRVYSSINYILLSGILEKVTHRSYENLVKDTYIKKLGLS

ETEFYWDIPKNKQIKVAIPYTKSSQGYLVPHFISADKVHGDLGAGCLVMSNKDLYRAT

SAILNGEIIKPSSVQKVYTPSDPAKYNAGFYNFPDFHSSNGSGDGYTTYYRISNDTRD
                     VLVIQSNYPVKDYFKVRQMCNDLMENLIKATS"
     CDS             complement(9358..10659)
                     /locus_tag="OCPHGMPK_00008"
                     /inference="ab initio prediction:Prodigal:2.6"
                     /codon_start=1
                     /transl_table=11
                     /product="hypothetical protein"

/translation="MSNKRKLWQIFGPVLCAFILLVILFLIPWQNGEKSDNALFKASV

SQSQTVFKGQSIKQAAFEKNYVPFYGSSELSRMDALHPSVLAYKYKRNYRPFLLGGPG

SQSLTHFFTMQETVNQLSNKKAVFIISPQWFTKQGQNPAAFGMYFSQLQAVDWILGAK

DSVATRYAARRLLDMPAGTASETTKYALMTLASGQKLSKYQTLYLKARRRMLANEDNF

FSSIGMTNNIPKIQKEAGKLPGTYAYDNLRQFANEQGKQGTTNNKFGISNGFFDKKLN

NKHLKKLKDSQRSFDYTKSPEYADFELVLNELAHEHVNVLFIIPPVNEKWAKYTGLSQ

TMYQECVAKITKQLTSQGFNNIADLSKDGGKKFFMEDTIHLGWNGWLKVDQYVKPFME
                     EKNHPVNYKLDSYYFTKAWGNKSDVKMPNTKSKVATDIKKN"
     CDS             complement(10652..10891)
                     /gene="dltC"
                     /locus_tag="OCPHGMPK_00009"
                     /EC_number="6.1.1.13"
                     /inference="ab initio prediction:Prodigal:2.6"
                     /inference="similar to AA sequence:UniProtKB:P55153"
                     /codon_start=1
                     /transl_table=11
                     /product="D-alanine--poly(phosphoribitol) ligase
subunit
                     2"

/translation="MDTKQAVLDILNELTGEDLSDQMDENIFENGLLDSMATVQMLLE
                     LQDKCGVTAPVSEFHREDWDTPNKIIAKVESLRNE"
     CDS             complement(10922..12145)
                     /gene="patA_1"
                     /locus_tag="OCPHGMPK_00010"
                     /EC_number="2.3.1.-"
                     /inference="ab initio prediction:Prodigal:2.6"
                     /inference="similar to AA sequence:UniProtKB:O25526"
                     /codon_start=1
                     /transl_table=11
                     /product="Peptidoglycan O-acetyltransferase"

/translation="MINIQPYSNPRYFVILFIALLPLIIGLYHGRRFKTYEAFVSLLF
```

LFLMFNGKDWIQGVQLIGYIIFQFLITFAYQKYVNSGKNKTSVFCLAVILAILPLAAV

KIAPLAHNKTADLIGFFGISYLTFKTVQVIMELRDKTIKKVDPITYARFLLFFPTISS

GPIDRYRRFKEDYDKAPERDKYIENLKFGTRYIFQGFLYKFIIGYVFGTLWLLQLARD

ALFYGQANGGLRLSWALLGYMYCYSMYLFFDFAGYSLFAVGTSYFMGIRTPMNFNKPF

ISQNIKDFWNRWHMTLSFWFRDFIFMRFTFFAMKHKLVKNRIRLSQLAYLVDFLIMGF

WHGLTWYYIVYGIYHALAIIINDIWLRFKRKHRKQIPHNKFTKWFAIFLTFNVVCFSF
LIFSGFLSTLWFGWK"
CDS complement(12142..13650)
/gene="dltA"
/locus_tag="OCPHGMPK_00011"
/EC_number="6.1.1.13"
/inference="ab initio prediction:Prodigal:2.6"
/inference="similar to AA sequence:UniProtKB:P35854"
/codon_start=1
/transl_table=11
/product="D-alanine--poly(phosphoribitol) ligase
subunit
1"

/translation="MIKDVIKTIDQIATEDPKRIAYDYLGETNTYGDLKARSDAYAAK

ISELDLPEKAPVMIWGGQTFEMIASFLGAVKAGHAYIPIASYSNSERLLMIQEVSESP

LVIAIDKLPIEMDNIKVLTPEEVSDNHAEIEQSKFVSGDDNFYIIFTSGTTGKPKGVQ

ISHDNLLSFVNWELNDFSLPDQPSFLAQAPYSFDLSVMSLYPALTAGGKLVVLPHDVT

ENLGQLFKTLPQLKFNVWVSTPSFVEMSFLDPTFDAKHHPELTHFLFCGEELSHKTAK

MLKMKFPDSHIFNTYGPTETTVAVTQVEITDDILSKYDRLPIGVSKEDTKISIDTSKG

EEKDQGEVIIEGPSVSKGYLNNPEKTKAAFFKDGEHSSYRTGDLGFLDGDMLFYRGRI

DFQVKFNGYRIELEEINFYLAKNPLVRYGVVAPKYNKDHKVQQLVAVVELADGVREKY

DDAELTKKLRESLSQDIMPYMLPQRFIYRDEMPISQNGKVDIKQVIKEVNNA"
CDS complement(13671..13829)
/locus_tag="OCPHGMPK_00012"
/inference="ab initio prediction:Prodigal:2.6"
/codon_start=1
/transl_table=11
/product="hypothetical protein"

/translation="MAENKDKQNNTKKKEIGLFIAKTLFYFAVLVVLVYLYSYSGIGA
AKFIYKDF"
CDS complement(13967..14254)
/locus_tag="OCPHGMPK_00013"
/inference="ab initio prediction:Prodigal:2.6"

```
                     /codon_start=1
                     /transl_table=11
                     /product="hypothetical protein"

/translation="MVKKIAFWVRLAGWSGLISGSSVLMLYQYSHSSLFLINLITIVL
                     FSAYALATANDKKWENPDWLLKVILVVLVFVSILPTIFLGIGYFIERKRNQ"
     CDS             complement(14256..15407)
                     /locus_tag="OCPHGMPK_00014"
                     /inference="ab initio prediction:Prodigal:2.6"
                     /codon_start=1
                     /transl_table=11
                     /product="hypothetical protein"

/translation="MKRFLKILFKLILILAIIAGASYGGYYGYQQYQKREQAKATFTS

RPDVEKAKDGTSISPGHHNLAYFKKLLNEKYPDVYSAAYETPRASKIGNSVVIPGQVV

TPSYDFNKKKITDTDSMTPQGLTVAGKYLLITAYDSTHNHRSVIYCLDKKTGKYLKTI

QVSGSPHLGGIAYDPTAKNIWVTGSQDDSSALMSFSLKKLEKYSYNKKKQPIQYDNVI

ALPTIERASCVTYYDNQLFVGFFNTDGQGQIASYPIARSGNFKGTITSDQIKAVTGQV

SWALGSGSASMDRQIQGIAFYQNWIILSQSYGSKDSKLYFFPISALNYLDEGNAEKVV
                     VMPPYLEQIYVQNGQLLMLFESGAKAYARDQIMIVDRILSANINALLGG"
     CDS             complement(15764..16075)
                     /locus_tag="OCPHGMPK_00015"
                     /inference="ab initio prediction:Prodigal:2.6"
                     /codon_start=1
                     /transl_table=11
                     /product="hypothetical protein"

/translation="MEYQEYISLLEAFRLSLTAENDPNVVQIVDQINETIANLKTGKY

HAVALTSQLFDVVSLVENLMAFNDLNLNTFQSKVWNKLRDATYQRYTKDGNLLNFIPF
                     P"
     CDS             complement(16344..16517)
                     /locus_tag="OCPHGMPK_00016"
                     /inference="ab initio prediction:Prodigal:2.6"
                     /codon_start=1
                     /transl_table=11
                     /product="hypothetical protein"

/translation="MKIKSWMLMLIAMGFLFSPVAMVYILEGKCEQIVTFFIKKVKFV
                     KKAGINYKLAPQL"
     CDS             16705..17193
                     /gene="ykuL"
                     /locus_tag="OCPHGMPK_00017"
                     /inference="ab initio prediction:Prodigal:2.6"
                     /inference="similar to AA sequence:UniProtKB:O31698"
                     /codon_start=1
                     /transl_table=11
                     /product="CBS domain-containing protein YkuL"
```

/translation="MFSPSIQSLIEEKAGSFIIPASRIAFVEDENPLYHAFLILTKMK

YSKIPVLDKNQKVVGLISLSMITDKMLTPDNIVIEPLSDLKVKDVMQTDFETINFAHT

NLETQLHLLVDNPFIPVVNDNNIFQGLLTRREWIKAFNYVTHSIDDKYDITKKSDPSE
AS"
CDS 17272..19149
/gene="zosA_1"
/locus_tag="OCPHGMPK_00018"
/EC_number="3.6.3.-"
/inference="ab initio prediction:Prodigal:2.6"
/inference="similar to AA sequence:UniProtKB:O31688"
/codon_start=1
/transl_table=11
/product="Zinc-transporting ATPase"

/translation="MRHQWKFFTILGIGVIAAILDFLCGAPKIGTWPISGILIDIFGI

FMAITMLREMIHTLESGRWGVDILAIIAVVSTMIVGDYWAAWMILIMLTGGDSLEDYA

TSQADKELRSLLQNSPRIADKLVNGKIEEVKVDDLKIGDIVLIKPGSQVPVDGEIIKG

NSSFDQSSLTGESVPVDKKVGDDLMSGSINGDAAVEMKVTKAAKDSEYQSIVALVKSS

EAKPAKFVKMADRYAVPFTIISLIIGIAAMITSAVTNPALGWQGHFLRFAQVMVVASP

CPLLIAAPVAMVSGMSSMSRSHIIVKSGTTLEKLSRTLTFAFDKTGTLTENQLVIDQV

VLAKDSSISKEELQGLAASVEQQSSHVIATSLVKSTDKNLIKPVTNLKEATAKGVSGE

VDGKLVKVGKLAYVDPDQEKITVQSTAVFVSIDNKFAGYITFQDQIKKNTPETIARLR

RQGIKQIMMLTGDRRSVADKVATEAGIRESEVHADLLPAQKIQAIRDVKPNLRPVAMV

GDGVNDAPSLMAADVGIAMGAKGATAASESADAVIMVNDISKVNDAVAISKHTMKVAH

VDIITAICIVILIELIAFTGIIPAFWGAILQEVVDLITILLALLAKTKPTNPKQTGLE
K"
CDS 19316..20476
/gene="napA"
/locus_tag="OCPHGMPK_00019"
/inference="ab initio prediction:Prodigal:2.6"
/inference="similar to AA sequence:UniProtKB:P26235"
/codon_start=1
/transl_table=11
/product="Na(+)/H(+) antiporter"

/translation="MHFLGELILILLATTLLGQLFSRLNMPAVIGQLLSGILLGPAIL

GLVKPNEIISLFSEFGVILLMFLAGLESNLELLKKYFNLSFTVAGIGVILPVFFMGIA

SYLFGMNFLEALFIGIVFAATSVSISVVVLQEANQIHTRAGTAILGAAVVDDILAVIV

LSLFTTFSHEGGKSGLTNNFFLNLLIEVIYFVVVWIIFKFIAPYFMKLAEKIEVDYAV

VIGSLVLALSMAWAADFVGLSAVVGAFFGGLAIRQTPQYKEVQSSVSAIGYSIFIPVF

FVNIGLSMTFASFIKDIWFIIVMTILAVVSKFWAGKYSSELFGFTKNEGNIVGAGMVS

RGEVALIVAQIGITHHLFPEDIYSSLILVIIITTVLSPFILNYYIKRGIKNH"

```
     CDS             complement(20536..21189)
                     /locus_tag="OCPHGMPK_00020"
                     /inference="ab initio prediction:Prodigal:2.6"
                     /codon_start=1
                     /transl_table=11
                     /product="hypothetical protein"
```

/translation="MNWKKTLFSIGIIVIILVGGLTMFNVANQKVVNQDYSLSKFTKV

DIDIPTGDVVLKAGNKYHVSYSGLEKNVPKVEVKDSKLKISFKNQITHIRLFNFKQLN

SKIVIEMPKEQLESLNVDNSNGNFSADYLAVKIGDIDLSNGNIDIKDLKTKDGISFDT

SRGNIRVESSNATGYDLDTSLGKITISGKKHGDSFEKNSDSRNVLEADTSLGSISVN"

```
     CDS             complement(21190..22758)
                     /gene="mdlB"
                     /locus_tag="OCPHGMPK_00021"
                     /EC_number="7.6.2.2"
                     /inference="ab initio prediction:Prodigal:2.6"
                     /inference="similar to AA sequence:UniProtKB:P0AAG5"
                     /codon_start=1
                     /transl_table=11
                     /product="Multidrug resistance-like ATP-binding
protein
                     MdlB"
```

/translation="MLFKYSNKFRFILMIIFGLIASFQNIIMANVVQILTNIATNKNW

GKITQFLIIIIAALVVTLIASLVFNRLKTNTIKETNTYLRTHILGGMLEESKDENNDS

LGFLTNDFKLLETNRFNAQIEIIMQIFSLVLALGYALAVNWLLTLLFLVGSFIPMVVS

NVFQKPIQESSEKWTSANSKYVNQTKNFLAGVETLHLYDGQNQAVAKNKQTISKLEQA

LSNMNLLNLDTNSWINFVGNIVTFLMPFLFGIYLVVKGQTSLGALFAIVQLANSFINP

ILFILDDRSKLSTTKKIVEKVNDFLDKEKDAENRKVVSLQDLQVEDLILKRDGKQLAT

GIDLNVNLGKKIAVIGPSGAGKSTLLQFLLYGKYGKAEAIRLNDKRVKAGTFPDLFSY

ASQAPVIFADSLLFNLTLGKNISREKVEEVCDNLDLSELVKEKGFDYQLGEAADKLSG

GQLARIELARAILMKRPVLLLDEINASLDKKTSDIIHKYLLASKLTFIEVIHHYEKDE

```
                     LKQYDEILDLKEHI"
     CDS             22869..23729
                     /locus_tag="OCPHGMPK_00022"
```

```
                     /inference="ab initio prediction:Prodigal:2.6"
                     /codon_start=1
                     /transl_table=11
                     /product="hypothetical protein"

/translation="MYGHEFKELRLEQGITQKEACSGICSESKLSRWENDQVEVEFST

AMKLLNRIHITSHEFMGWSKFSPRPEIDPELSEATEKENIPFLKRVTQKQLTKYHKNH

NKFDLFMAANLCNQLFIIEHKNYLPPIDQKKLFAIFSKVNLWSQYYISAFGASIFLFN

PKQIYGSSMQIIRNIDRLKEAETSFNLEIIMGSLSDGILRLISLNELSYAQKLVKELK

KIELPQYLMFFTLTLTYLQKAIDYMKNGDDKPILTLISEVLDLGCSVQATTYLDMFKY
                     LKAQRKKYNF"
     CDS             complement(23857..24687)
                     /locus_tag="OCPHGMPK_00023"
                     /inference="ab initio prediction:Prodigal:2.6"
                     /codon_start=1
                     /transl_table=11
                     /product="hypothetical protein"

/translation="MTIGDLLRQYRINQNKTLQEFAGNIIDRSYYGKVERNIHQISAE

NLINLLRYNQIDAIEFIETLDPNYENYQSQIQIQKKIMEEAYYQVDKDKLKKVKLMIT

ESKLSEKDKEIQNLIADGLLELLNPDKPNQKIRDEIKKKIFEIPNFNSTKFMLYCNSM

RFYSLADNEAIVRKIIEKYQVRESMLVKKSLLSLAINVLVLSIEENKFENIDFYSDFA

NKVPTNPDLFFYKSAMTFFTYFIQYKKNQDKSALQYCDAIIKSFCLVGMPEYGEELRI
                     "
     CDS             complement(25027..25269)
                     /locus_tag="OCPHGMPK_00024"
                     /inference="ab initio prediction:Prodigal:2.6"
                     /codon_start=1
                     /transl_table=11
                     /product="hypothetical protein"

/translation="MFWMFLSLAIIGFILCLLVRQHIIKYGFPFAYCLIGIIVSSFVC
                     LTQFGNHDTNFDWYGYLVIVFIVETWYLYHYFLLRS"
     CDS             complement(25289..25936)
                     /gene="pcp"
                     /locus_tag="OCPHGMPK_00025"
                     /EC_number="3.4.19.3"
                     /inference="ab initio prediction:Prodigal:2.6"
                     /inference="similar to AA sequence:UniProtKB:Q5XDD4"
                     /codon_start=1
                     /transl_table=11
                     /product="Pyrrolidone-carboxylate peptidase"

/translation="MKILVTGFDPFGGDKINPAIEAVKRLPDEIKGAEVVKLEIPTVF

NKSAEVVKEAIKKENPDYVLNVGQAGGRFGLTPERVAININDGRIPDNEGYQPLGEPI
```

```
HKDGETAYFTQLPIKAEAKAIRDAGLPASVSNTAGTYVCNHIMYQVQYMRDKEFPNIK

AGFIHIPFLPEQVVNRPNTPSMALDDIVKGLTAALSAIVERDGKGDIEAIEGANH"
     CDS             complement(25955..26878)
                     /locus_tag="OCPHGMPK_00026"
                     /inference="ab initio prediction:Prodigal:2.6"
                     /codon_start=1
                     /transl_table=11
                     /product="hypothetical protein"

/translation="MQNTINFLLAILYALIGLCMAVAGIETLRDKANKARIGTAVFWF

FLAIIFAFGDWIPAVVSGAMVLVIGILALFKQIQVGSLPKLDPKTAQEGAKRLGAKVF

LPSIVLALTSILIAQFTPLGGQVGIGIGAVVSLILAIILTKAPTKQVFKDSQRMVRSV

GAPGVLPQLLAMLGVVFTTCGVGKLTANMISHVFPAGNHFLGVALYCIAMALFTYIMG

NAFAAFAVITAAIGIPFVIAQGANPVVVAAIGMTSGYCGTLVTPMAANFNTLPVALLE
                     MKDQMGVIKQQAPIAVILLIVQIGLMYFLAF"
     CDS             complement(26880..27554)
                     /locus_tag="OCPHGMPK_00027"
                     /inference="ab initio prediction:Prodigal:2.6"
                     /codon_start=1
                     /transl_table=11
                     /product="hypothetical protein"

/translation="MEYLKLLGILIIVVGFVLKWDTTAVVVIAAIVTGLFSGMDFVKL

LEVIGKSFVDNRMVSLFFLTLPMIGLVESHGLRDYAVNAIKKLKKLTAGKILNIYLLI

RELAGVFGISLQGQVQFVRPLIAPMVIAAAEDQDKKKLTESEIDLIKGRSAAVDNFGN

FFAQNLFVASGGVLLISSTMKSLGYKVDPVGIVLNTIPIAIITFVLVFAYNLYFDRQM
                     KKWEGQ"
     CDS             27718..29169
                     /gene="gatC_1"
                     /locus_tag="OCPHGMPK_00028"
                     /inference="ab initio prediction:Prodigal:2.6"
                     /inference="similar to AA sequence:UniProtKB:P69831"
                     /codon_start=1
                     /transl_table=11
                     /product="PTS system galactitol-specific EIIC
component"

/translation="MNTINFFFNSFGTTIVIPTTIFIIALFVGYDPKKSLLYGLYAGI

GLTGFSWIINQFTPIVIKIVYKMVNNTGIKSPIVDIGWEAGAVASFGSAIGLTFFIFG

LLVEIILFATRITKVFMPSNLWNNFVFMLWGSLAFYVTHDWWLSLGLSFFMLLYTLLF

AEIQADRWSTYYKSKNITVCSAQNIVQTIPAILLDPLWNRLGFNRTTLTPTTLKHKLG
```

IFGESPILGAALGLFISLIANIKELNQVSAWEQIFQFTIQLSAVITIFPLIATVFNMA

FTPLAEQIDKSRKQKQKSNSGIEINPIHDKKRWFLAVDDSVGYGESATIISGIVLIPI

MLILAFLLPGNKTLPIVDLVLLPFMVESIVAITNGNILKVIATSIVWFSLGLYTASWL

GPIYTHAIAHYGVTIPSGIILVTSFSLMAQPFNALIFTAFISRNPFWISLCIIIYLIS
LLILRRYRPQIWTYLNKMAAKNIQIKHKSSFQK"
CDS 29285..30739
/gene="gatC_2"
/locus_tag="OCPHGMPK_00029"
/inference="ab initio prediction:Prodigal:2.6"
/inference="similar to AA sequence:UniProtKB:P69831"
/codon_start=1
/transl_table=11
/product="PTS system galactitol-specific EIIC
component"

/translation="MLNNIHTFFNAFGATVIVPVMIFIIALFLKVKPKKAMMCALYAG

VGLTGFSWIINEFTPVVTKIVRQMVDNTGIKLPIVDIGWQAGSLASFGSPVGLTFFVF

GLIAEFILFAVGVTKVFMPSNLWNNFGFMIWGTLAFYVTHNWWISLGLSFFMLLYTLL

LAEIQADRWSRYYKIENTTVCAAQNIVQTVPAILLDPFWNMLGFNKANLTPAAFKNKF

GVFGEPTTLGAILGIVIGILGNLKDLATIKAWGQIFQFAIQLSAVMTIFPLVTNVFSK

AFTPLAKQIDQTRKQNANQRPEVDPVHDKKRWFLAVDDGVGYGESATIISGIILIPIM

VVIAFLLPGNKTLPVVDLISLPFMVESIVAITDGNILKVIANGVIWFSLGLYAASWLG

SIYTGAISQYGAAIPAGIVLVTSFNLMARPLNALVFAAFISQNPLWISLCIIVYLVLL
YILRKYRSQIWIYLNKMAAKNVHAKEDLKSSPKD"
CDS 30786..31295
/gene="yagU"
/locus_tag="OCPHGMPK_00030"
/inference="ab initio prediction:Prodigal:2.6"
/inference="similar to AA sequence:UniProtKB:P0AAA1"
/codon_start=1
/transl_table=11
/product="Inner membrane protein YagU"

/translation="MSKFSWKNVLIAGTAAGVISGLVKLGWENILPPRTPERNKTNPP

QKLLEQMGVPAKLTHATYTYSGEKLPWVSYLVHFGFSISFATAYAALLEKKVKWLTVD

QGVPFGLAVWIAFHLVIMPLMKTVPSPKDQPLEEHVSEALGHIAWMWTNNEIAEVVLE
QLRHNKKEH"
CDS complement(31312..31959)
/locus_tag="OCPHGMPK_00031"
/inference="ab initio prediction:Prodigal:2.6"
/codon_start=1

/transl_table=11
/product="hypothetical protein"

/translation="MKTFLRFLGNLAVIIIAFVFYTVLQLGYAQKKVNLLFAIILAVL

TVAVIYWMYWIYKRELKQENDWFFNAKPHWDVKRIIIAVAAFFALVIFQVAYIRLIGG

NTVSQNQAELDEVRKVASPMFNILLVVVAPICEEFIFRALFFNTFFPADNNLNKWVGV

VASGFVFAYGHDPMFSKFIYLYWVMGMILAWTYVETKDIRYSILTHMLNNILSIL"
CDS 32040..33161
/locus_tag="OCPHGMPK_00032"
/inference="ab initio prediction:Prodigal:2.6"
/codon_start=1
/transl_table=11
/product="hypothetical protein"

/translation="MQSQLMQSLHQFLNFTMHHRVTITTLLMFMFLLLFLFSWLIEPR

RLINGLIFTAFGLTFLTWAAILIFSQHNVILTSSFSFIALAFLFGIFFLVTFSWIFFL

WNAYFVWKYENHSLPNLLTLIIGLFLVALWTLYRLGVFHKLPGWLHSLVAGAALIAFY

LLFVMYNFLLNLVLYQIVPRRYKQDYLIVLGAGLIDGKKVSRLLGSRIDRAIAFSNKQ

YDKGHKRPKLIMSGGQGKDEDLSEAEAMKDYAVKRGYDPDLILLEDKSTNTYQNMVYS

KAVAIKDYGNEKFKVKFFTNNYHLFRAGLYAKMADLKANGIGATTRFYFLPNGTIREF
AGVFIMHKKRHFVIIGLIAILFIIQAIFALLGWSKYIIA"
CDS 33174..35513
/gene="yknY"
/locus_tag="OCPHGMPK_00033"
/EC_number="3.6.3.-"
/inference="ab initio prediction:Prodigal:2.6"
/inference="similar to AA sequence:UniProtKB:O31711"
/codon_start=1
/transl_table=11
/product="putative ABC transporter ATP-binding
protein
YknY"

/translation="MLQLLHLKKTYHVGDTVTHALNDVTINFRNSEFVAILGPSGSGK

TTLLNVIGGLDHYDSGDIIINGKSTKNFSQTDWDAYRNNSVGFIFQSYNLISHLSIIE

NVELGMTLSGVSSNERRKKAVSALERVGLGDHLKKRPNQLSGGQMQRVAIARAIANDP

DILLCDEPTGALDTKTSESIMKLIKELSHDKLVIMVTHNPELAEEYASRIVHFQDGKI

LSDSNPFEPKKEAKDTFKLKKTKMSYWNALKLSFTNIMTKKGRTFLTAFASSIGIIGI

AIVLALSHGFQKQINDTQSKTLAKFPISISQTATDMNAATSRNESDKNVKNKGYLVAS

KPDNEKNTHENKITQSYINYVKKINPDYANNISFIRGTQLNLLTNDNGKIKHVEFSNV

NNSGSAIASAQLQGMNSVGINTSVFPKTLDSKQGTFLKDNYQILAGSWPKSNNDVVLV

LNNKNQANINALKNLGISIKDGQKLELNKIVGRTFKVINNNDYYQELPTGNFVPQKAS

KSMYDSSNLTLKVTAVIRGKNNSQMALLDNGIAYSDGLTQEIIKQNENSNIVKAQKNS

STNVMTNQPMNKNQKEQFIASLGGSSIPSGILIYPNSFKSKDKVLDYLDKYNKGKAKK

NQIIYTDMSGTVTKLTGGLLDGITDVLVAFAGISLVTSMIMIGILTYTSVLERTKEIG

VLKALGARKRDITRVFDAETFILGLFSGILGIFIAYLCTFPINAVLYAITNMSNVAQL
DPTQALILVIISTILTMLGGHIPARMAAKKDAAIALRSE"
CDS 35686..37077
/gene="cydA"
/locus_tag="OCPHGMPK_00034"
/EC_number="1.10.3.-"
/inference="ab initio prediction:Prodigal:2.6"
/inference="similar to AA sequence:UniProtKB:Q09049"
/codon_start=1
/transl_table=11
/product="Cytochrome bd ubiquinol oxidase subunit 1"

/translation="MLSGVTMLDLARFQFAMTTVFHFFFVPFSIGMGFIVSIMETLYV

VKKDEVYKKMAQFWGKIYLLSFAVGVVTGIIQEFQFGMNWSDYSRFMGDIFGAPLAIE

ALLAFFMESTFIGLWMFTWDKFKPGLHCALVWISFIGSMLSAIWILTANAFMQHPVGY

VIKNGKAQMASFWALIANEQLWASFPHVVIGAIVTASFVVIGMAAFGLLRKRDVNFHK

KSMRVALWIALFASIAEIGAGDYQTQVEIHEQPMKFAATEGVYETTGDHAPWDIVANL

NTKEHKNEGAISIPDVLTILTYHKTSGSIKGMNQINKELHAKYDKKFGKDMSYYVPVK

TLFYSFRIMAGFGALFALLSILGLIWTRPKKNTIENKRWFLWILGISTFLPFVANTCG

WFITELGRFPWTVYGLFTIADSISASTTTGELWFTNIMYFLIFSTLGGVMIYYCKRQL
DMGVAGALERGAY"
CDS 37077..38099
/gene="cydB"
/locus_tag="OCPHGMPK_00035"
/EC_number="7.1.1.7"
/inference="ab initio prediction:Prodigal:2.6"
/inference="similar to AA sequence:UniProtKB:P0ABK2"
/codon_start=1
/transl_table=11
/product="Cytochrome bd-I ubiquinol oxidase subunit
2"

/translation="MIDGIDFLQLLWFLLIGLLFMIFYFTEGFDYGVGMATKFLARND

QEKHLMMETIGPHWDGNEVWLITAGGAMFASFSDWYASLFSGYYILLFFTLFALIIRG

VSFEFSAHAETEHGFRIWTGALFWGSLLAPFFLTMMFGSLVQGVPIDAQGNMSLSFWN

IVNPLSIVAGVAGVLLCLIHGINFLRLRIDDPELSERAASLNEKLYLIAYLGEVVFAL

LVFFQTDFFKLRPVSSTIITLIIVALTAWSDISLIKNKQVAAFVTSGLTLVAVVGLLF

NGLFPRVLIATDSAHSLLIKTAANSKLTLEVMTIVALILLPIALVYIIWSYVVFRRRI
KVNQNA"
CDS 38118..39935
/gene="cydD"
/locus_tag="OCPHGMPK_00036"
/inference="ab initio prediction:Prodigal:2.6"
/inference="similar to AA sequence:UniProtKB:P29018"
/codon_start=1
/transl_table=11
/product="ATP-binding/permease protein CydD"

/translation="MIDKRLFKLPKAKIMLAMLAGLMFLQAFAILGQGIFLARAIVGS

WKRQPFTEIAQDVLLFLIFYLLRQGINWFQKWYMNRYANQTTTLLRQQLLNKTYDGGI

ALVSRIGTGNLVSTLLDGMDEISNYLSLIFPKLIALAIVPWVILIYIFTLNALSGWIL

LLVFPLLILFMIILGTAAQSKASKQYAGYVKLQNHFVDALRGLSTLKVLGLARKYGNI

VYKNSENYRKKTMGVLRVAILSTFTLDFFTTLSIAMIAMFLGIGLINGNLVLYPSLVI

LILSPEYFLPIRDFGNDFHATLNGKNALGQIFDILAFPTTPQEDQLSSFTWNNDSTLV

AKNLSFNYSHVDQSKFTVNKNAKMSGVVKKKTMHKTSQHTADELRNINLNLTGFQKVG

IIGLTGAGKTTLMRILAGFLTPHLKDDNFTINGQTLAQLNQKNWQNQITYIPQDPYMF

AASIKDNLTFYNPNASQEEIDAALKATDLDNFVASLKDGLNTKIGENGRGISGGQKQR

IALARAFLAKDRKILFFDEPTAHLDIETEYELKQPMKKLMENHLVFFTTHRLHWLNDM
DWCLVIENGEIVEQGTPADLAKNGTAFKKLTKPLKEDLL"
CDS 39932..41677
/locus_tag="OCPHGMPK_00037"
/inference="ab initio prediction:Prodigal:2.6"
/inference="similar to AA sequence:UniProtKB:Q57538"
/codon_start=1
/transl_table=11
/product="putative ABC transporter ATP-binding
protein"

/translation="MMNKKFSWKNDHWIKPYLAQYKWNFLLAIFLGVIMFFCGGALMF

YAGYTIDKAATRPENILMIYVPIVLMRAVGIGRPLFRYLERLVSHNWILRVTSSLRRR

LFYIAERNTSAVGSSFQTGSILSLLTDDIGHLQNLYLRTIFPAILSYLVGFFVVILLG

LFSWPLAGAIAILLVAELVLVPFFSLLKQAAVRTKEKEEKAQLYTEFTDQVLGAGDWK

```
ISGRRDAFFNQTKETLKSLGQHEKKSGKFDWARDFGLEFIFGLMAVALLYFTNQTLTN

NQEAANYVGAVVLALFPLSDAFIPVGQGIEEWHTYSDSVKHLNELKVPENRLPVQQYL

DPTFAGTLRVSDISFTYPKEDYPIIQNFSLTLKRGQKAALIGPSGAGKSTILQLILGD

LKPNQGTVTLDRLNVLKLQRERSKLFSVLNQEPFLFNTTIYENLKMANPDATTDQMME

ILEKVQLADFVNSLPKGLDTEVAEAGARFSGGQKERLALARVLLQDTPIVLLDEPTVG

LDSLTEQKLLNLVFEVLKNKTVVWVTHHLQGVKYMNEVLFFKDGKVTMQGNPHELFKH
                     NEHFHQLYLMDQGLI"
     CDS             41698..42624
                     /gene="menA_1"
                     /locus_tag="OCPHGMPK_00038"
                     /EC_number="2.5.1.74"
                     /inference="ab initio prediction:Prodigal:2.6"
                     /inference="protein motif:HAMAP:MF_01937"
                     /codon_start=1
                     /transl_table=11
                     /product="1,4-dihydroxy-2-naphthoate
                     octaprenyltransferase"

/translation="MSAEAKQGYYELVEPRTLFNSIIPVLLGMMYTEYNFQFFRIFPT

IEMCIATIVLQIFMNVNDGYWDYKREKAAKTGDHKKNPIGKYHLNPKHVLAFVWVLFI

ISAVCAILIGLQTNIYIWIVGIICYAIAISYSTGSHTISAGPFGEIAACFAMGFGIFL

VMVYINVCSKVSFNWNFIYPIILAAGIPEICNFTLMLGNNLCDHDADIANGRHTLVSY

IGIKGGLYLFVFNYLLGFFLTGWAIWIGVLPWSVALILICIPTIYKNMRFLWKIQTKP
                     KSFPKVVQNTQVLFITEAVGFFIGLILNLRIR"
     CDS             42767..43006
                     /locus_tag="OCPHGMPK_00039"
                     /inference="ab initio prediction:Prodigal:2.6"
                     /codon_start=1
                     /transl_table=11
                     /product="hypothetical protein"

/translation="MKVLNECQLQTVVGGKNWSVAKCGGTIGTNIAIGAWRGARAGSF
                     FGQPVSVGAGALIGASAGAIGGSVQCVGWLAGGGR"
     CDS             43006..43215
                     /locus_tag="OCPHGMPK_00040"
                     /inference="ab initio prediction:Prodigal:2.6"
                     /codon_start=1
                     /transl_table=11
                     /product="hypothetical protein"

/translation="MIEKVSKNELSRIYGGNNVNWGSVAGSCGKGAVMGIYFGNPILG
                     CANGAATSLVLQTASGIYKNYQKKR"
     CDS             43219..43491
                     /locus_tag="OCPHGMPK_00041"
```

```
                     /inference="ab initio prediction:Prodigal:2.6"
                     /codon_start=1
                     /transl_table=11
                     /product="hypothetical protein"

/translation="MTGIWIVIISIIVLSIIITNIVALIQTLLHKKNEKYYFDKSFGA
                     YGDKNNPKYLFNNVEHHDFFHVFYQYFICSYIPFIDVIFWFLGSIW"
     CDS             complement(43558..44853)
                     /gene="ugpB"
                     /locus_tag="OCPHGMPK_00042"
                     /inference="ab initio prediction:Prodigal:2.6"
                     /inference="similar to AA sequence:UniProtKB:P0AG80"
                     /codon_start=1
                     /transl_table=11
                     /product="sn-glycerol-3-phosphate-binding
periplasmic
                     protein UgpB"

/translation="MNLYKKIALGLVAGLALIATGCSKGNKEDSSAANIPTKITKKTT

VTFWHGMNGGQRAELEKLTAEFEKKNPNIKIKLENQGSYIDLQAKVNSTLQSPNNLPT

ITQAYPGWLYNAVKSNMLVDLSPYINNSKLGWGSAAKSNIRTELLDGAKIQGKQYGIP

FNKSIEVLTYNKSMFEKYGIKKVPTTMKELAQVSETIYKKSNHQVVGAGFDSLANYYT

LGMKNEGINLTDKVNFTGKDSKKVIDYYAQGMKKGYFTTAGSAHYLSGPFANEKVAMY

IGTSAGEGYVKKAVGDKFTYDVAARPGKYTMQQGTDIYMFKKATAMQRTAAFKYMQFL

TSKSTQLKWANATGYIPVNNSVVKSKEYKENTKTKLPAKLEGAMKHLYSIPVAKNSNA
                     AYSQLDPIMAKILYAAKNGQNVDNQIKAGKAKFDAAWKQ"
     CDS             45142..46074
                     /gene="phnD_1"
                     /locus_tag="OCPHGMPK_00043"
                     /inference="ab initio prediction:Prodigal:2.6"
                     /inference="similar to AA sequence:UniProtKB:A0QQ71"
                     /codon_start=1
                     /transl_table=11
                     /product="Phosphate-import protein PhnD"

/translation="MKIKKFLIGAAMVLLAATSAACSNSKSASKSSDGYTPKELNVQF

VPSVQASKLEAKAKPLQGLLEKQLHMPVHVTVSTDNSALVEAMASKKVDVGFLPPDAY

VLAHKRGVADVLLQAQRYGYDEPSGKQNHKLMDSYRSMIVVKKGSKIKSWKDLKGKTI

AVQDPTSTSGYVLPIAELHKKGLNVPKDCKLVQVKGHDQAVLSVYNGDADAAFVFSDA

RPLAAKDAPAVMKDVVPIYFTKYIPNDTVSVRSGMSKSFKKKLAKAFKDIAKTKKGKK
                     ILENIYQHYGYVDSKDSNFNIVREYEAEAKKAQK"
     CDS             46167..47240
                     /gene="tmpC"
                     /locus_tag="OCPHGMPK_00044"
```

/inference="ab initio prediction:Prodigal:2.6"
/inference="similar to AA sequence:UniProtKB:P29724"
/codon_start=1
/transl_table=11
/product="Membrane lipoprotein TmpC"

/translation="MKLNKKFSLLLIASTIFLSLSACSKPTHEEQTNKKLANHIALIS

DTNGIKDNSLNQSAWQGVKNYGTKNNLVEGNKGYNYFVPKDPQDYQATINKVLDNDFS

TIIGVGYTLKPNIVKAAKNNPKKNFVLIDTHAAKIKNLASISFKNQEGAYLAGVAAAY

TTKNHTVGLILGQNSKEMDSFKAGFTQGVTASSRKLHKHIKVMSKTVGNFSDINKAHD

IAQEMYNQKADIIFQAAGKSGQGVFKAAKEVNQTNPVGQKVWVIGSDEDQTKLGNYQA

KGGQPSNFTLTSVIKSADVAVEDLANQTAKGNFPGGKNLNYGLKNKGISLVQGNLSYH
TWIKVQKAKQKIIDGKIKIMANR"
CDS 47402..48286
/gene="fpaP_1"
/locus_tag="OCPHGMPK_00045"
/EC_number="3.4.11.5"
/inference="ab initio prediction:Prodigal:2.6"
/inference="similar to AA sequence:UniProtKB:O05420"
/codon_start=1
/transl_table=11
/product="Proline iminopeptidase"

/translation="MANESKIVTLKNGYHVWTRKEGHGPIKILLLHGGPGMDHEYLEP

FSNYIKMHPNIEIIYYDQLGSYFSDQPNDPKLWTIPRFIEEIEEVRKAWDLDQFYLYG

QSFGGLFALEYAASKYGKHVKALIDSNMVDSYQDYAKYINKYRESMDPADVSYMKKQE

ERHNYDDPHYQELLMKLYHRCICRISPWPDAVQRTFDHMNEQVYVTLQGPTEFNITGS

CKNWTIRDRLSKITMPTLVLGGKYDSMNPADIKALADRLPNGTAHICPNGSHFSIFDD
QKDYFNAISSFIRTVENK"
CDS 48565..50502
/gene="nagZ"
/locus_tag="OCPHGMPK_00046"
/EC_number="3.2.1.52"
/inference="ab initio prediction:Prodigal:2.6"
/inference="similar to AA sequence:UniProtKB:P40406"
/codon_start=1
/transl_table=11
/product="Beta-hexosaminidase"

/translation="MKIKRAFTSVIAGFSIAVGCLAINNLSANAATTQNGVTNDQINQ

YVNNATLEQKVGQMYVSRTPQDPNQVKNDVSKYNLGGLIVYDADMKGLTQQQFKDKMK

SFQDSAQTPLLVGVDQEGGLVSRLTHSGLVQQNGNQFAFPRKQYEEGGMKEVVDDAEK

NATLLRSLGINWNYAPDADYSTKPGTFMWDRTFGQGYQQTADYLSKVVPAWQHDNLVA

STLKHFPGYGDAADTHTGFAENSKSLTELENQDMVPFKAGMKAGADSVMVTHVIYNKI

DPEYPASLSPKINSLIRNNCNYNGVIVTDALEMGAITDFAKEHGNASVDVLAVKAGND

MIMTTDYATGIKEIVSAVKAGEIPESQINASVTRILQLKNKLGLLKPENLMFRKPTNN

NNNNNNTSTIINPGNTDHKNPSVISGNNSITISGITYNNDKKVAFINGKVSDPAAEGS

MIMTATNANTGKKITTAVVGGKGVFAIELPMTNEVQNVKITSDDDSYAAVTTKVEAIK

ADKPVVNTDQNNKQNSSTEKQTNKQNNSVKDEVNITLNTEKQANSAKKNTQVVSKNIN

SEKTTVTNPVYKSAIKTNYQGKNVITTSHDSAAHAVAQKSNNNAKVLPKTGTKENIVA
LFGLAITSTTALFGLALRKKN"

```
     CDS             50777..51649
                     /gene="sorC"
                     /locus_tag="OCPHGMPK_00047"
                     /inference="ab initio prediction:Prodigal:2.6"
                     /inference="similar to AA sequence:UniProtKB:Q9RGG3"
                     /codon_start=1
                     /transl_table=11
                     /product="PTS system sorbose-specific EIIC
```

component"

/translation="MKISVIAIIFLSLYVGLGQLDALSLKTGLYSPVFGGAVTGLVLG

DLKTGLIVGATLQLATLGVATYGGATVPDFLSGSVMGTAYAIISGRGAAYGIGLAIPI

GLLLTQMDILARLANTYFQNHATKEAEKVNYTAVERDNLYGIISWVLSRAIPVFIGLT

FGATVVKAINNWIPAWLMNGLKTAGIILPAMGIAILMRYLPLKRYYPYFILGFALMAY

FAKQFSLLGLALVGFSLAAIYVMQKQKNNKSGKAQNIEKKDSNSKKEIKGIQNPNEAI
EASRVDAEEVEFDA"

```
     CDS             51642..52490
                     /gene="manZ_1"
                     /locus_tag="OCPHGMPK_00048"
                     /inference="ab initio prediction:Prodigal:2.6"
                     /inference="similar to AA sequence:UniProtKB:P69805"
                     /codon_start=1
                     /transl_table=11
                     /product="PTS system mannose-specific EIID
```

component"

/translation="MPKKNIQVKMKNLTKGDLVNIFWRNTLGLQLGWNYPRMQGLGYA

YTVMPALKRLYKNKKDKMKQALKLEMEFFNTTPQMSHLIVGADVALQDELGMDDNNEK

AIAGLKTGLMGPFAGVGDTVFVAIYNAIIYSITAYLALSGQPLGLLIPVVGCLAITWI

RWKFFNIGLSQGQKLAFAFSDKMNMLTEGASVLGLTVVGGMIPALINYKVDLTYKVGK

VTMSVQSMLDKIMPALLPLAIALFSYWILGKKHMNSTRLIFVLIGLGMILGNLQPICN
WIGSIF"
CDS 52503..52982
/gene="sorB_1"
/locus_tag="OCPHGMPK_00049"
/EC_number="2.7.1.206"
/inference="ab initio prediction:Prodigal:2.6"
/inference="similar to AA sequence:UniProtKB:Q9RGG4"
/codon_start=1
/transl_table=11
/product="PTS system sorbose-specific EIIB
component"

/translation="MSICAARVDERMIHGQVAMVWTNVVGANRILVANDEVVKDDLKI

EGLKLAKPAGIKLSICSIQRAIENLKNNKYGEDDVFLITRNIPDMADIINGGVDLKEF

NVGNISSKDGSRQIKKSVNVTPKDVDIIKNLINKGVKITAQMVPNEPDSSIMTYIEK"
CDS 52994..54025
/gene="pbpE_1"
/locus_tag="OCPHGMPK_00050"
/inference="ab initio prediction:Prodigal:2.6"
/inference="similar to AA sequence:UniProtKB:P32959"
/codon_start=1
/transl_table=11
/product="Penicillin-binding protein 4*"

/translation="MQQNTVDKIVKVINQSIQNNDIYGASYSLIGKNENLRTYVGYQG

EDNDRIPISSGMMYDLASVSKVIGTTTRIFQLLANRQLSLDSEVGEYLSDISYPKIKI

KSLLLHESGLQSDFDNVHSMAKEELIKKVKSAPLIYTPESKTVYSDLNYILLGWIIEN

IDQIPLNEDLKSHIFQPLDMNNTTYSPNSIPSSQFIPTEYQKDRGGIIRGKVHDYKAY

LLDGISGHAGLFSTLNDLSQFAQVFLNGGKYKGIQVFPKIMYTLLKNKKYRHNDRTLG

WELWDSNKNMLWHSGFTGTSIALNLDSHEAFICLTNRIYPTRKKTGWIQERRESLSYF
FNQKEEIKK"
CDS 54022..55077
/gene="ykfB"
/locus_tag="OCPHGMPK_00051"
/EC_number="5.1.1.20"
/inference="ab initio prediction:Prodigal:2.6"
/inference="similar to AA sequence:UniProtKB:O34508"
/codon_start=1
/transl_table=11
/product="L-Ala-D/L-Glu epimerase"

/translation="MSIYIKKITCSKKTVPLKRPFITALHKVTEIEGLKIVVELSDGQ

TGTGTATPNEKVTGDTLQSSLDIVNQVIKPALINQDFKNWNKILTTLQTCIVHNSAAK

AAMDIALHQLKAKENNESLVNLLGSEKGKLTTDYTISIGSDDHMVSEAKALVKDGFTA

IKIKLGNNSVSHDIKTIQKISKAVGPDISLRIDCNQAWNYKDTLKASRTWAQDNLNID

FIEQPVLKDAISDLTLLSENSPYPIMADESVASYQDAINLIQHHACDYINIKLMKTGG

LSEAIKINDLAQACGIKTMIGCMIEPVESIAAAAAFAVANKNVKFIDLDSIFMATQDP

DLSKYLEIKNNEIILKD"

CDS 55082..55813

/locus_tag="OCPHGMPK_00052"

/inference="ab initio prediction:Prodigal:2.6"

/codon_start=1

/transl_table=11

/product="hypothetical protein"

/translation="MQLTQIQEQIENNPWKTAITIRKNNHTIFENSNANVIFKSASLI

KLGIALYIQEKKPGTIANTLTLSSNDIVGGAGIINRLSIKSWKISDLLDLMLSVSDNT

ATNALLNYYNINTINDYLQRNFQNISLGRFLMKKSDKENTCTSNSMMDVFEKLLAGNN

EVNHVVLDALKHQEVRNKLVAYSNPKYEVFNKTGELLHEQHDIARFRANTDVIDCCVL

TNYQSQKDYENILSMMQKIGKILTH"

CDS 55829..56722

/gene="murQ"

/locus_tag="OCPHGMPK_00053"

/EC_number="4.2.1.126"

/inference="ab initio prediction:Prodigal:2.6"

/inference="similar to AA sequence:UniProtKB:Q45582"

/codon_start=1

/transl_table=11

/product="N-acetylmuramic acid 6-phosphate etherase"

/translation="MEIKNLTTEQRNPATMHIDSMSTIDMVKTINQEDQKVAVAVGTQ

DDKIARAIDEAAKRYSKGGRLIYIGAGTSGRLGVLDAAELVPTYGIKPERAIGLIAGG

KGAMYVAVEGAEDSQDLAKRDLKDLKLNKNDIVLGLAASGRTPYVIGGLDYAKAIGAL

TISIACVKDSKIGHHADIAIEAVVGPEAITGSTRMKAGTAQKMILNMISTGVMIKQGK

VYENVMIDVKPTNSKLIDRACRIIHTTTGVSTQKAKDTLKEANNDVGLAIVMLKTNCS

LNQAKSLLETKNGNVAEVLNK"

CDS 56796..57557

/locus_tag="OCPHGMPK_00054"

/inference="ab initio prediction:Prodigal:2.6"

/codon_start=1

/transl_table=11

/product="hypothetical protein"

/translation="MSPFFLFIYLIFGGILGGLLSTIASMASLATYPVLLSVGIPPVY

ANTTNDAALIWTGVGSTASSLKELKGHWKQVSFYSIFTIIGSALGCMLLIQFPGKIFE

KIVPFCIAFSGLMILFSGEIHLGNESDHPNRLVQALSLLCLLIAGAYAGYFGAASGVL

MLVILNAISDDTFLTVNAMKNIIGALSNLVALIIYMFTEKIYWSAAIPLAIGALIGSY
FGPRIMRHIPVKIIRLGIAVLALIQAAYFAYTAYK"
CDS 57649..58533
/locus_tag="OCPHGMPK_00055"
/inference="ab initio prediction:Prodigal:2.6"
/codon_start=1
/transl_table=11
/product="hypothetical protein"

/translation="MVKKIEPWFGFTEDKVFGMVMENKDFCKYLLEIIIPDLKIEKID

WLDKQVEINNFERKNEAKEVRLDVLVTDHEGRVFNIEMQTTDQDDIGRRMRYYLSRLD

LRYTLNKGKTYRNLKDAFIIFLCNFKPKKDDKFYESYHTYSDQDRSKQLQDGVTKIII

NSQVSAEGQSEDLKALAKLMNNEPVKLNKHFDYAQRRIKEINEDPETREKIMLYETRM

LEREQAAAKIAGKVAYAEGRKDGVEQGRAEGKIDVAKVILENQIDNGSTLEQATKFVK
DLKIISDKDLEKLVKIYK"
CDS complement(58625..59464)
/gene="dkgB_1"
/locus_tag="OCPHGMPK_00056"
/EC_number="1.1.1.346"
/inference="ab initio prediction:Prodigal:2.6"
/inference="similar to AA sequence:UniProtKB:P30863"
/codon_start=1
/transl_table=11
/product="2,5-diketo-D-gluconic acid reductase B"

/translation="MEKSFQENNLSGLGMGTWGIGEDASKKKDEIAALRYGLDNGIKV

IDTAEMYGEGKSEKLIGEAIQGYDRSQIFLISKFYPYHASPELERQSLENSLKRLKTD

YLDLYLLHWRGDKRLSETVRGLKELQNEGLIRHWGVSNFDVADLEELFSVPGGKDCFA

NEDLYNLSERGVEYDVLPWQKKHGVGFIGYSPFNSGKGDSIRITNNLKIVARAHNVTP

HQIMLAWTLRHGNVLSIPKAGTVKHMKENIAAQDIKLTSDEIKLIDNDFPQPTEKTPL
AMI"
CDS 59594..60313
/gene="nagB"
/locus_tag="OCPHGMPK_00057"
/EC_number="3.5.99.6"
/inference="ab initio prediction:Prodigal:2.6"
/inference="similar to AA sequence:UniProtKB:Q8DV70"
/codon_start=1
/transl_table=11
/product="Glucosamine-6-phosphate deaminase"

/translation="MKIIVTKDNIEGGTKAFEIIKKGMENGDKVLGLATGSSPIPMYD

DMCDSDLDFSDMTSINLDEYYGLKPDNDQSYHYFMQKHLFDKKPFKHSYIPNGMAKDI

DEEVDRYNDIIAANPIDIQILGIGRNGHIAFNEPGTPFGSLTHKVQLTESTIKANSRF

FDNEDEVPRQAICMGIKSIMQSKKIVLLAFGESKQDAVKALVEGPVTEEVPASILQDH
PDVTVICDEVAAAKLDPKYRK"
CDS 60499..61146
/locus_tag="OCPHGMPK_00058"
/EC_number="2.7.1.76"
/inference="ab initio prediction:Prodigal:2.6"
/inference="similar to AA sequence:UniProtKB:Q74HC3"
/codon_start=1
/transl_table=11
/product="Deoxyadenosine kinase"

/translation="MTVIVLSGPIGAGKSSLTSLLAEHLGTQAFYEGVDNNPILPLYY

KDMAHYTFLLNTYLLNHRLAQINQAIRDHNSVSDRSIYEDALFFKMNVDSGIADPTEF

KIYDSLLENMMEQAPGNPSKKPDLLIYIHVSLDTMLHRIQKRGRTFEQLSTDPGLKDY

YARLLSYYEPWYEKYNASPKMMIDGDKYDFVADEDARKEVINTIDQKLTDLGNLN"
CDS 61168..61842
/locus_tag="OCPHGMPK_00059"
/EC_number="2.7.1.113"
/inference="ab initio prediction:Prodigal:2.6"
/inference="similar to AA sequence:UniProtKB:Q74HC2"
/codon_start=1
/transl_table=11
/product="Deoxyguanosine kinase"

/translation="MTVIVLSGPIGAGKSSLTGILSKYLGTNPFYESVDDNPVLPLFY

ENPKKYAFLLQVYFLNTRFQSIKSALTDDNNVLDRSIYEDALFFQMNADIGRATKEEV

DTYYELLHNMMSELDRMPKKNPDLLVHIDVSYDTMLKRIQKRGRNYEQLSYDPTLEDY

YKRLLRYYKPWYEKYDYSPKMTIDGDKFDFMASEEDRQEVLNQIVAKLKEMGKLDENW
KPNLVK"
CDS complement(62003..63241)
/locus_tag="OCPHGMPK_00060"
/EC_number="1.13.12.4"
/inference="ab initio prediction:Prodigal:2.6"
/inference="similar to AA sequence:UniProtKB:P21795"
/codon_start=1
/transl_table=11
/product="Lactate 2-monooxygenase"

/translation="MTTYYKGFPQSTREEKLHMVNLDELENEAKYVIPEAAYYYIASG

AENEWTWRNNTQAFNHFQIVPRALTGMQDPELNTEFLGMKLKTPVMICPIACHGIANA

EAEIDTAKGAKAAGALFGMSTYANKSVQDVQNAVGDSPRFMQLYLSKNWDFNKMVIEE

SVKAGFTGFFLTVDALVSGYREANLRTNFTYPVPLAFFNEWTGGKGEGQSVAEMYASS

AQNIGPDDIRKIKDIADVPVIVKGVECAEDAMLAIGAGADGIVVSNHGGREVDGAPAT

IDVLPEIAKAVKSCDRPVPIILDGGVRRGSHVFKALALGADLVGIGRPFLYGLALGGA

QGVQSVIEQLNKELLIDMQLTGCKTIDDIKHAKIDHLDYTADWGISSTSKSVMKPYPV
TKENQLTGEAADAVSGASRH"
CDS 63481..64791
/gene="pbuG_1"
/locus_tag="OCPHGMPK_00061"
/inference="ab initio prediction:Prodigal:2.6"
/inference="similar to AA sequence:UniProtKB:O34987"
/codon_start=1
/transl_table=11
/product="Guanine/hypoxanthine permease PbuG"

/translation="MNFIKNYFHLEKLGTNVRTEFIAGLTTFISMSYILFVNPSVLGA

SGMNTGAVFTATALAAALGTAIMGIVANYPIGEAPALGINAFFAYTICIGMKVSWETA

LASVFVASIIFILITLFKLREKIIDSIPSDLKFAISSGIGLFIAFLGLQNGGLIVANK

STLVGLGSLHNPLVWITIFGLIVTVILMILGVPGAIFIGMIAASIFGICTGQIAVPHK

FISLAPSLAPTFGQAVFHVKDINSLQMWVVVLTFLLVTFFDTAGTLIGLAQQAGFMKN

NKMPRVGKALAADSTAMMFGSIFGTSPVGAFVESSAGIAVGGRSGLTAVFVAIFFLIS

MIFSPLLGVFTTQVTAPALIIVGVLMAQNTAHIHWNKLEIAVPAFLILLGMPLTYSIS
DGLALGMITYPICMVSAKRGKEVSPMMWVLFVVFIIFLWVLNFK"
CDS 65021..65203
/locus_tag="OCPHGMPK_00062"
/inference="ab initio prediction:Prodigal:2.6"
/codon_start=1
/transl_table=11
/product="hypothetical protein"

/translation="MIITGKTIFKIVYILSIIFSITYIVWNTLQHNPLDPTYLLVTVI
SIVAMTLVFIKINKEE"
CDS complement(65725..66432)
/gene="tipA"
/locus_tag="OCPHGMPK_00063"
/inference="ab initio prediction:Prodigal:2.6"
/inference="similar to AA sequence:UniProtKB:P0A4T9"
/codon_start=1
/transl_table=11
/product="HTH-type transcriptional activator TipA"

/translation="MQEKYSIGEVAAMLEVSTRTLRFYDEKGLVKPAYTEENGYRFYE

KEQIRQIELILFLKDLGFSLKQIKTLIQDERGSKSLELLLKQQYQENKQKINELTAKQ

KQIEHLQKIGVLSAALTNFSDITSIMRKENKMTVLRRKMWLYIIMWIVVELIGISLMY

ALRLNASIAIVIAVLGAIMFSKYYYDRVEYVCPNCGDVFIPSFLIFNLAPHTPKFRKL
TCPKCEKRSYCLEICRD"
CDS 66737..68047
/gene="pbuG_2"
/locus_tag="OCPHGMPK_00064"
/inference="ab initio prediction:Prodigal:2.6"
/inference="similar to AA sequence:UniProtKB:O34987"
/codon_start=1
/transl_table=11
/product="Guanine/hypoxanthine permease PbuG"

/translation="MNSIGKFFHLNENHTSFKTEFLAGLTTFVSMSYILFVNPAVLGA

SGMNKGALFTATALSAAFTCIVMGLVANYPIASAPTLGLNAFFTYTVCLGMHVKWQTA

LAAVFVASILFILLTVFKVREMIIDAIPSDIKYAISAGIGLFIAFIGLQGGKLISKSD

STLVTVGALNNPLVWITIFGLVVTIFLMIARVPGAIFIGMIVAAVFGIAIGQIPMPKA

FISGVPSLSPIFGQAVFHISDINTVQMWIVVFTFLLVTFFDTTGTLIGLVQQAGLMKD

NKMPRAGEALAADSSGMLVGSVLGTSPVGAFVESSAGIAVGGRTGLTAVWVGIFFLIS

TIFSPILSVFTTQVTAPALIIVGVLMAENLAHVHWTDLEIAIPCFLIALGMPLTYSIS
DGLGWGLIIYPVSMLAAKRFKEITPMMWILFFVFVIYFVVLNVK"
CDS 68058..68357
/gene="hisE"
/locus_tag="OCPHGMPK_00065"
/EC_number="3.6.1.31"
/inference="ab initio prediction:Prodigal:2.6"
/inference="protein motif:HAMAP:MF_01020"
/codon_start=1
/transl_table=11
/product="Phosphoribosyl-ATP pyrophosphatase"

/translation="MKKLVRDKIPEFATYASYRQLKPDEREDALKNKIVEEANEVKAA

PDDQNLLEELADVYTVLEAFLDFKNISKEDLLKQVEAKKAEKGGFTKFLLMNTDK"
CDS 68620..69933
/gene="pbuG_3"
/locus_tag="OCPHGMPK_00066"
/inference="ab initio prediction:Prodigal:2.6"
/inference="similar to AA sequence:UniProtKB:O34987"
/codon_start=1
/transl_table=11
/product="Guanine/hypoxanthine permease PbuG"

/translation="MQFLDKVFHLEDAHTNVKRELIAALTTFVSLSYILFVNPNILSA

AGINKGAAFTVTAIATAIGCFLMGFIANYPIALAPTLGSAAFFSYNVVIGMHINWQTA

LASVLVASILFILITVLKLREKVVDAIPQDLKYAISAGIGLFIAFIGLQNGKLIVDNK

SSLVGLGSFNNPAVWITLFGLILTVILMAAKVPGSIFIGMIVTAIFGMVIGQIPMPHG

IVSGAPSIAPTFGQAVFHLKDINTPQLFMVVLTFLLVTFFDTAGTLIGMTQQAGMVDK

NGKIPRIGKAFLSDSLAMVEGSVLGTAPLGTSVESSAGIAMGGRTGLTAIFVGILFLI

SMIFSPLLAVIPTTVTAPALIIVGVLMAGNLKYIHWDNFEIAFPSFLVVVGMPLTYSI
SDGLALGMIAYPITMIASKRYKEVSPMMYILFVIFIIFFLITNMG"
CDS 70073..70486
/gene="def_1"
/locus_tag="OCPHGMPK_00067"
/EC_number="3.5.1.88"
/inference="ab initio prediction:Prodigal:2.6"
/inference="similar to AA sequence:UniProtKB:P96113"
/codon_start=1
/transl_table=11
/product="Peptide deformylase"

/translation="MSVKPIIHDELSLKFKSTLATKQDLAAAEDLKDTLIANNGKAAG

LAANMIGVQKRIIALFVGPLPIVMLNPIIVKKTDKYLAYEGCLSLEGERPTERYKKIT
VKYQNENFETRQQEFSDFVAEVIQHEVDHCNGILI"
CDS complement(70548..71852)
/gene="licC_1"
/locus_tag="OCPHGMPK_00068"
/inference="ab initio prediction:Prodigal:2.6"
/inference="similar to AA sequence:UniProtKB:P46317"
/codon_start=1
/transl_table=11
/product="Lichenan permease IIC component"

/translation="MNNGFTKWVNANLVPPVLKFVDLKAIRALKDGILYTLPLLMIGS

LFTLIANFPYQPVTDFLTKTGWIDPLNQINGATFSIIAIVAVLGVSYQYAKIEKVEPF

ASAVIALSAFLVLTKSSAVTPNGKFTITNVIPKDWTSGQGMIAAIIVGLLVGWSYSYM

IKHKWTVKMPDTVPSGVSNSFAALIPAAVIISVTFLIYALIHFVWNTTFIEGVYKIIQ

TPLQGMTDSIWGVLAYCFVATFFWWFGVHGGTIVTSIFYPILLANLTANQKILDAGKA

LTTANGAHVFTDQFTAILINMTGTGATIGLVIYMFFFAKSQQTKQLGRLAIVPSIFNI

NEPVIYGTPIVMNPFLFIPFVLTPVVIGLLDYSLMSAGVLPLFSGVQVPWTTPPIISG
FILGGWKLALAQVANIIISCLIYFPFIRKVDNINYEKEKAAK"
CDS complement(71869..73323)
/gene="bglA_1"
/locus_tag="OCPHGMPK_00069"
/EC_number="3.2.1.86"
/inference="ab initio prediction:Prodigal:2.6"
/inference="similar to AA sequence:UniProtKB:Q46829"

```
                     /codon_start=1
                     /transl_table=11
                     /product="6-phospho-beta-glucosidase BglA"

/translation="MEGSMLIMPFKKDFLWGGAVAANQIEGAWNEDGKGISIADVVTS

GNVHNPREITSELEPNKYYPMHQASDFYHHYKEDIELLSEMHFKCFRLSIAWTRIFPN

GDDKEPNYKGLEFYKKVFKECKKYGIEPVVTLSHFEMPLNLVKKYGGWCNRKVIDFYI

HFAETCFFFYKGLVKYWMTFNEINNQTDYNSELSMLADSGILIKGLSEKERENKMYQA

AHYELVASAKAVQIGHGVDSNNKIGCMINMTPQYPFSCKPEDIFQAQKAMQRRYWFAD

VQVFGKYPNHIEAYLKQNNFRKDITEEDKLSLIDGQVDYIGLSYYNSNTIKAKSNNLN

FKFVGSELNVENPYLKKSEWGWPIDALGFRYSLNWLEDHYHKPLFVVENGIGMHDSVT

KNKAIHDNYRIEYLREHVLAMRDAVEKDGIDIMGYTPWGCIDLVSMGTGQMSKRYGFV
                     YVDSNDKGEGSFNRLKKDSFYWYKDVIDSNGEKI"
     CDS             complement(73323..74765)
                     /gene="gtfA"
                     /locus_tag="OCPHGMPK_00070"
                     /EC_number="2.4.1.7"
                     /inference="ab initio prediction:Prodigal:2.6"
                     /inference="similar to AA sequence:UniProtKB:P10249"
                     /codon_start=1
                     /transl_table=11
                     /product="Sucrose phosphorylase"

/translation="MKLQNKTMLITYADSLGNNLNDLSEVMEQHFKGAIGGIHILPIF

PSTGDRGFSPTRYDEIDPKFGTWKDFEKLGEKYYLMLDFMINHISKHSKYYQDFQKNG

ENSKYKDLFLDWNKFWPKGRPTKADIDQIYKRKDKAPYQEITFADGSKSKLWNTFGDE

QIDLDVRTKVTQEFIKDSVRTLAKHKASLIRLDAFAYAIKKLDTNDFFVEPEIWELLD

QVKNDLKGTDAKILPEIHEHYTMPMKVAKHGYYIYDFALPMVLLYSLYSGKSDRLAAW

LRKCPMKQFTTLDTHDGIGVVDAKDVLTDDELEYTTNELYKVGANVSRKYSSAEYHNL

DIYQINTTYYSALGNDDKKYFLARLLQVFAPGIPQIYYVGLLAGKNDLKLLEETKEGR

NINRHYYSKEEIAKEVKRPVVQSLIKLLRFRNEEAAFDLDGSIEVETPSENVIVIRRS
                     NKDQSRTAEATVNLKDLSYNVKVNDQEVRF"
     CDS             74935..75885
                     /gene="degA"
                     /locus_tag="OCPHGMPK_00071"
                     /inference="ab initio prediction:Prodigal:2.6"
                     /inference="similar to AA sequence:UniProtKB:P37947"
                     /codon_start=1
                     /transl_table=11
                     /product="HTH-type transcriptional regulator DegA"
```

/translation="MRVSITDVAKKAQVSIATVSRVINQNDYVAPETRKIVEAAIKKT

GYHLPQHHLANHTKNKIIEVILPSVTNPLYAKFYQEIERNLAAENYSSLLKIDNDPTH

TLEPYLTALKKGKIAGIITSSNLPISDQNASDLLPIVSFDRHLSKVTVLKCNNLDGGY

KIAQKVLSLGKKNILILSGNRADLYPLNDRLKGMLSVFNHYKVNVKTTYIDFESSMMV

KKIQIAQIIDSPKFDAICCTDDVTALLVKQHTDEMNYHPLITGFDGTDFIHSFFPELI
TVKQPIEDITKLMCEILIKKIHHPSKHFEPEYIFPVTLLD"
CDS complement(75940..76485)
/locus_tag="OCPHGMPK_00072"
/inference="ab initio prediction:Prodigal:2.6"
/codon_start=1
/transl_table=11
/product="hypothetical protein"

/translation="MRPDLIVKNRKLNRLLGVAGIILSVIVIFLALRNLGWLYRLVGL

CAIIYYLVDIEQGLEESHAITRLGRIILVVIGILYIGLSMVCVFWNVDLLLRLFGLAG

ATYLGFKSYQLVRSGIFNKKLKLTDSLIAVFLGLFPAILLIAYAFWYFWLILLAFTLL
YNLVHLIVRIKIERGPTNREL"
CDS complement(76506..77066)
/locus_tag="OCPHGMPK_00073"
/inference="ab initio prediction:Prodigal:2.6"
/codon_start=1
/transl_table=11
/product="hypothetical protein"

/translation="MDDKRWIELAKRKKQDKYFGFISLLIVVMMVFFTVKNIGWLLTF

IVVAVILYFWIDVEQILEDSDAISSRTVKIVFIVLGTMYELVSIIFAFVNLDYLLVAF

GIISAGYLGFKTNAVLRKDLKNKNLKFADSMIATFLGIVVASLIVAFTPRDILWLFLL
IFALAYNLLHLIVRIKVEHDGTNRQL"
CDS 77225..77659
/gene="copY"
/locus_tag="OCPHGMPK_00074"
/inference="ab initio prediction:Prodigal:2.6"
/inference="similar to AA sequence:UniProtKB:Q47839"
/codon_start=1
/transl_table=11
/product="Transcriptional repressor CopY"

/translation="MNEKNLSPISDSEWEVMRIVWTLGETHTNQILKELQAKKNWSDS

TIKTLIRRLVQKGWLTAKHEGRRYTYTATVSQTDMMYNEAKSLLNRMCDMHKGEVILK
LLEDSPISKGDLMKMQEEISQKEKTAPDMVPCNCLKTGSTIC"
CDS 77672..78049
/locus_tag="OCPHGMPK_00075"
/inference="ab initio prediction:Prodigal:2.6"
/codon_start=1

```
                     /transl_table=11
                     /product="hypothetical protein"

/translation="MNTSQIIVLIIGIILIGFIVWWFFGKHKEATGSGTIVNDEQTAT

IVVNGGYSPSTVVLKKGIPAQVNFDMRDSTACLSHVVFEQLGVNKDLTKQKITTVNIP
                     TDKASTYNFACGMDMFHGKVIVK"
     CDS             78068..78355
                     /locus_tag="OCPHGMPK_00076"
                     /inference="ab initio prediction:Prodigal:2.6"
                     /codon_start=1
                     /transl_table=11
                     /product="hypothetical protein"

/translation="MSIFSKKQTKKVVVNAENHGYKPDVITFKEGKPAQVKFIPSDNM
                     GCMNEIVFKELGIDKVLDGKNEIIVDIPTDKPGTYNFACGMDMFHGKVVVK"
     CDS             78355..80289
                     /gene="copA"
                     /locus_tag="OCPHGMPK_00077"
                     /EC_number="7.2.2.8"
                     /inference="ab initio prediction:Prodigal:2.6"
                     /inference="similar to AA sequence:UniProtKB:Q2FV64"
                     /codon_start=1
                     /transl_table=11
                     /product="Copper-exporting P-type ATPase A"

/translation="MKLSNIKRFWISFILSIPMLIQMFAMPFHWMMPGYNWIALITTT

IIMAISALPYWKSAIAAFKKHSANMNTLVATGTAVAYFYSIFAMITNRPVYFESAAFV

TVFVLLGDAMEEKMHDNASNALGKLMGLQAKDAEVQRDGKFVKIPLDQVKVGDIIRVK

PGEKIPVDGEILEGVTTLDESMVTGESMPVVKKVGDTVVGSTINSNGTITFKATKVGS

DTMLAQIVDLVKKAQTSHAPIQNLTDKISNIFVPAVMIIAILTFIIWYSFLGATAVEA

MLFAVSVIVIACPCALGLATPTALMVGTARSAKMGVLIKNGEVLQEVSDLDTVVFDKT

GTITVGKPEVTDVVGNQKQVLTIAASLEESSEHPLATAIVKKAKAEKLTIEKVDDFEA

IEGKGVKANYNGQTAFVGSDRLLADVNISQEMTQKATDLQNEAKTVVYIGLDGEIIGL

VAIQDVPKPSSREAIKELRERGLKTIMLTGDNKNVAEAIAKQVGIDQVIAGVLPNEKA

MHIKKLQDQGKKVAFVGDGINDAPALSTADVGIAMGSGTDIAIDSGGIVLVQNDLRGV

VRALDISKKTFNRIKLNLFWALIYNTIGIPIAAGLFVGLGFTLSPELAGLAMAFSSVS
                     VVGSSLMLNKTKIAGETAKA"
     CDS             complement(80344..81210)
                     /locus_tag="OCPHGMPK_00078"
                     /inference="ab initio prediction:Prodigal:2.6"
                     /codon_start=1
                     /transl_table=11
                     /product="hypothetical protein"
```

/translation="MTVGELLKEYRVKQNKSQKEFAAEIVSQSYYSKVEKNVHRITAD

DLLLLLTHNAIPVKNFFEKLEIDPHQQQLNQVNAIFEEITKANYSDNSLAQIKKIKEK

AAKLDVEADMKKNMMLIIDGVLESEKDDPDTFDFNLRNKLKEKIFLMPEYDTYKLSVY

ANFVDLYDFDSNRQIIKQIVKALKKKKVERQDEILFAIILNVLKQGIEEQKYNETNEL

ISLGHQIKVIPEFFLYREMLTFYEELIAYHTDRKETHLEQCNLILESFEWGGMSAFGK
EMIKFFDKYKEQ"

```
     CDS             81309..83255
                     /gene="pepO_1"
                     /locus_tag="OCPHGMPK_00079"
                     /EC_number="3.4.24.-"
                     /inference="ab initio prediction:Prodigal:2.6"
                     /inference="similar to AA sequence:UniProtKB:Q07744"
                     /codon_start=1
                     /transl_table=11
                     /product="Neutral endopeptidase"
```

/translation="MKSFKIRGGLGDSAVVNPDVKLQDNLYLAINSKWLEHAEIPADR

PMIGGFTELDIKIKKQLLQDFDNFASNDKALPKIANLDKAVRLYQLALDFKQRDADGA

RPIQADLQKLTSLKNFSDFDKHAVNLVKGALAFPFTINTDVDMKNTAKNALYFDSPAT

FLPDAGNYQDSSADELLAIFEKQSVDLLKLAGFNENDSKKYAANAIKFDKLLAKYKKS

QEELNDIAGIYNPYSLSDFKAAFSNLNIEQLLNDLLPNLPEKVIVIEPKFLQHINKLL

NPDNFEELKSWLVVTFINQYATYLSQEFREAAFTYDHAVYGAKELKSQAQHAYGIANN

ILGEVIGLYYGQTYFGPKAKADVEQMIRKILATYKARLQTNDWLTPFTQKQALKKLNA

ITLKIGYPDKLSDLYDQIQVSSDKSLYENIIAANQTKIIHNFNQLNEPIDRTLWGADL

PANEINAGYNPTNNDITFPAGILQKPFYSLDNTLSENLGGIGAVIGHEITHAFDPNGS

KFDEVGNIRNWWSDEDYAKFNELSQSEVTLFDGIICGGIKTNGNLTVGENVADLGGLT

AAVETAKKENGNLQEVFQSWAKIWRGKMTPQVRKTLIAYDPHAPRELRTNVTAQSIDD
FYTAFDIKPGDGMWLDPEKRVHIW"

```
     CDS             complement(83571..85049)
                     /gene="ycaM_1"
                     /locus_tag="OCPHGMPK_00080"
                     /inference="ab initio prediction:Prodigal:2.6"
                     /inference="similar to AA sequence:UniProtKB:P75835"
                     /codon_start=1
                     /transl_table=11
                     /product="Inner membrane transporter YcaM"
```

/translation="MDKPDVLNDLEPNKKHYMSWPVIALIDFVTIISFENIFYPFQNQ

GLSVVISWIFLLFTYVIPYALISSQMSLTFDNQDGGLASWVRRSTNDTLGYWTSWMYW

VQSVPYIVDVSNSVIVSFSWMIFGNNSLDKKMSTFWFGILTFVIILIFILLENKLRNS

LEILSLIGGGAMFIMSMLFVLLAAWSVMHGHHIATQPFNWGAFKPSFSLNYFSTTGLL

IFAMSGAELAAPYVQQMKNPKRDFPKAMWMLAIMTGFLTIFGTLALAMFFNAHHIPHD

FKMNGPYYAFQLLGESLGVGKVLMYIFAVVQAIFMMAQLAVLLDASSRVFAGDVGSKF

MPKWLTKKNKNGRPIHSYTFTVGLSLFLLLLTGTLPNINTIYNWLLNVNGIISPYKTC

WVFFAFVAMRMHQDKYHSDYVFIKNKAGALTVGGWCLLFTFVCATLGFIPQNVAVGTK
AFTHQLWMNIITVVVLFGLGFVLPWLRKREAKREQAMGEKLD"
CDS 85293..87392
/gene="odcI"
/locus_tag="OCPHGMPK_00081"
/EC_number="4.1.1.17"
/inference="ab initio prediction:Prodigal:2.6"
/inference="similar to AA sequence:UniProtKB:P43099"
/codon_start=1
/transl_table=11
/product="Inducible ornithine decarboxylase"

/translation="MNFLKIALSNKLKNNDFNSWQTTNLNNNPQASELAAIVLAEGDQ

NSLKTAQDLQKTSGLGIPIIKVSHETISNNEKEKIIDAANKYTKKMVPGFLTDLVNFA

EDHPVSFTTPGHHNGLYYEKHPAGVVFNRFFGKNLMFADTSDTVPELGDTMTHEGTPL

TAEQKAAETYHADKVYFCTNGTTSANSICASALLTKDDLVLFDRNNHKSLYNSALVMS

GAKPVYIPTDRNALGLIGEMDPNFLSEEKIRAEVAKVDPEKAKAKRPFRLAIIQSETY

DGLFYDAKWMIDKIGKLCDYILFDCAWGGFEQFVPIMNHLSPLNLDFGPDDPGILVTQ

SLHKQQAGMGQASQILKKDAHIKGQKRYVDHKHFNHAYLKFVTSSYSYPLYASLTVNS

YLTSGEGNKKWWDKILRLGIEWRKELIRKSKLFSPLVIDNFENISTDDLATNEKYWNL

EPNNLWHGFSKIATGQAMIDPLKITVVTPGIDVKNAKYEAVGIPGPVVAEFLMEKRII

RAKDDLYSLLFLLTPGDTKAELDTLLNAFLEFEQYYNEDAPLEKVLPKLTKVYGDRYK

GYTLKQLCQEMHNYYRGNKTFTLQQELFAKPNMQNYPMTPEHADYLFMKNESELVNLD

DVKGRIAAEGALPYPPGVFIVAPGEKWSDIDQKYFEVLVGAIERFPGFVPEIQGVYWD
QKADGKIRVQAEVLKEN"
CDS 87396..88664
/locus_tag="OCPHGMPK_00082"
/inference="ab initio prediction:Prodigal:2.6"
/codon_start=1
/transl_table=11

/product="hypothetical protein"

/translation="MVLSWKENLPTELKEKVDKVDKMIEPRLEEIDEQVFYNQQRVLG

LFRKHRVGEEDLVPSTGYGYDDVGRDKLEAMYADYFKTDDALVRPQFSSATHAIAVGL

FSMLRPGDTLYYLTGMPYDTIQEVIGLAGNKPGNMKEWGIDFKHTDLLDNGEVDYEQA

RKDLQDPKIKVVTIQRSLGYAVRASFTMEKIKKMLKFIKEVRPDVKIFVDNCYGEFSE

TEEPTFYGADMMAGSLFKNAGAGIVKAGAFLVGKKDLIEGAGSRLNVPGSGKGEGATW

GYLRDMYQGFFMAPHTTGEALKGMIYTAALCEEMGMKVAPKWNDPRTDIVQTVTFGEP

DSMVKFCAAIQHYSPMNSFVDPIPYHQDGYEDDVVMASGSFTEGSTIELSSDGPLRAP
YRLYIQGGLSYAHDKIAITHAVEETFYKNN"
CDS complement(88731..89171)
/gene="yafP_1"
/locus_tag="OCPHGMPK_00083"
/EC_number="2.3.1.-"
/inference="ab initio prediction:Prodigal:2.6"
/inference="similar to AA sequence:UniProtKB:Q47158"
/codon_start=1
/transl_table=11
/product="putative N-acetyltransferase YafP"

/translation="MFQIKIRLVMSEEVIIRKYKNTNYVQLCKVHDEARMQELIVGNA

VDLYAPLERAVYKEELFSETIYVAEVDSHVVGFVAFRKNELGYIYVLREYQGKGIGGK
LLDTAIPYLKRPAFLEVFSTNIRAKRLYGSRGFVEETKEKMILY"
CDS 89409..90752
/gene="ygcS"
/locus_tag="OCPHGMPK_00084"
/inference="ab initio prediction:Prodigal:2.6"
/inference="similar to AA sequence:UniProtKB:Q46909"
/codon_start=1
/transl_table=11
/product="Inner membrane metabolite transport
protein
YgcS"

/translation="MKKENQNNTNNIKEKKNYKKAPLMPIHLRVFMAVLLGQIACGFA

LGISGTALSNASKYIQINDLWTGLIGAGSLIGLAGSILIGRLSDKIGRRKLLMLNMYI

LGTLSLLQLLTNNLPTLFIIRILIGLMIAVDYTVGNALLTEWLPKGEDSKRQSHLLIY

WTIGFIASYITGTFISGFGSHTWQIILATGAIPAFIAAIFRSIFPLPASPSWLASRGK

VKTANKVIKKHMGRKWGIPKKLKKAKPVKEVSWSILFSGKYLRRTLVGGLFYACQAFA

FFGISIFLPILLQSMHVTDQKVSGIIYNGGMFIGVILGILLFNRISRRAFLISNFLLS

GILIGFLAIDKNLNSNIQLTIFCIFAIILSSGLVLDYPYPTELFDIKIRGTGVGTCIT

```
ISRFGAAAGTFLLPILTNQGGASLSMLICAIVLLAAFIICLIWAPETSPKFKQNN"
     CDS             90806..91204
                     /locus_tag="OCPHGMPK_00085"
                     /inference="ab initio prediction:Prodigal:2.6"
                     /codon_start=1
                     /transl_table=11
                     /product="hypothetical protein"

/translation="MVKMTISQDAINFLKNRGYDKHEIILIVDGGGGDYSIEGGSCNI

GMDYSLISLDTMKHDSRYSVKIENNADFEIYTSDYDLAFLGDNLVLDIHDTTLRLRND
                     SGILTGAVKIAKASFLAEQAKKGIISQKNC"
     CDS             91417..93294
                     /gene="mutS2_1"
                     /locus_tag="OCPHGMPK_00086"
                     /EC_number="3.1.-.-"
                     /inference="ab initio prediction:Prodigal:2.6"
                     /inference="similar to AA sequence:UniProtKB:P65496"
                     /codon_start=1
                     /transl_table=11
                     /product="Endonuclease MutS2"

/translation="MNNTDEIEFSRVRKLVSHYAVSPEAKDKILVQPLATKLVKATAL

LDETAEMVWILNHGLHIPFISSDALTPILNKVKKGFILNPAELEQVADFIRVTRLLVR

FFNKQRNNTPIIASYCDTLTILDTLESEIYNAIERGQVADKADKDLAKLRQRRAKLST

TIQDTLQKYLQNKKYQTMLQDYQIIQKDNHYTLPIKASFKHTFGGSIIDHSNNGNTVF

IEPTKIVHLTQEKATVENQIYLIESQILGDLSAKIYQELSQLEANLNIIIDLDCILAR

AKYSQSINGKRPLLNEQNQLQLNEMRHPLLSNPVPLSLNLDSSKRGLIITGPNAGGKT

VTLKTAGLAIAMAEFGLFLPSSGICSIPVMTAIYTSIGDHQDLDNSLSTFSAEMKQMS

YIVQHATKYSLILLDELGSGTDPNEGAALAIALLQTLQLKGSLILATTHYSAIKDYST

KHPTFITAAMDFDLKTLEPTYKLLLHQIGTSRALWIAEKNGMPQEVLTQANNFLNTGK

FPLNQSKIKFANNQKKIKSTVSFKKGDRLKVPEYEQDVLFYQNSDLANQIIIFVNKTF

KTIPIKGAKLVRKAKDLYPEGYNLDLLFIQDWKEYKLNRDLNRGSKKAWKKLKKIDKL
                     R"
     CDS             complement(93351..94031)
                     /gene="aadR_1"
                     /locus_tag="OCPHGMPK_00087"
                     /inference="ab initio prediction:Prodigal:2.6"
                     /inference="similar to AA sequence:UniProtKB:Q01980"
                     /codon_start=1
                     /transl_table=11
                     /product="Transcriptional activatory protein AadR"
```

/translation="MIYIKKEVCKMTKHSPLACLSNATLFKDLPESLRKKLVTISTHQ

EYFPKGSLIRQPFDGKDGLLVMDKGKAKVYSLNEDGKETILGLLNKGDSEGQEHLFST

SARENFIQATTDAWVCSMTRNDFQKLVRQTPDLALSLLNDFGQKLVTAEKNTVRRNSM

DAKQRIMAYLNDLSKKQGTNKVKLDLKKKDLAGYLGITPETLSRKLKELAKENKIQVD
KKIITIAK"
CDS 94145..94702
/locus_tag="OCPHGMPK_00088"
/inference="ab initio prediction:Prodigal:2.6"
/codon_start=1
/transl_table=11
/product="hypothetical protein"

/translation="MTKYIAHINPLNAEKIGCQPHGTAEFTENGDQLHIKVEMFDTPK

NIEHWEHFHGFPDGKVAQAATMAQDVNHDGFVDLPETEPVSGTTMVPLDADPAKMNIP

NNTYPVADSNGYYEYEIDVPLTELQENFKKAFSSTDLQLDKRVVYVHGVPESIKLPDT
VKGCVMDYDTHTTLPIAAGKIEKED"
CDS complement(95086..95931)
/locus_tag="OCPHGMPK_00089"
/inference="ab initio prediction:Prodigal:2.6"
/codon_start=1
/transl_table=11
/product="hypothetical protein"

/translation="MIGSLLKEYRLKQNKSQRKFIGSIVTQSYYSKVEKNISQITADN

LIGLLQYNNISVQEFFNNFSQKSDDSYRQTKELENMMIEAYYTNNIEQMHNIKKIIHE

STLSEYDKNYQTLMTNGFLALMNPKLNSEKLTSTIKNKIFDIPSYTQDKLMLYCDFMR

FYSLSDNEIITRNILKQYQSTNDSNIQELILAIVCNILIFSIENDKFENVSYFLNKIQ

NIKTTPQLLFYKIDIEFFKNIIEIKNSNDAGKINKCKNFIKTLQDAGMEEYSNELRKF
IKDNF"
CDS 96030..97289
/locus_tag="OCPHGMPK_00090"
/inference="ab initio prediction:Prodigal:2.6"
/codon_start=1
/transl_table=11
/product="hypothetical protein"

/translation="MNIFLTNKNYRRFSIASFLSSAGDILFYLAFMTYASKLKNYSLA

LSLIAISESSPKLFTIFGGYFADKTRHKFKNIFLSAIIRCVLYGIVGLLFISNVNQWS

LVIIVVIINLISDTFGAYSSGLVSPLVVDLVGKDHFGEAEGFTNGVNEVINIAAQFIG

SALLLIMSYSSLAFLNAISFLLAGILFASVGLRQKSIESLKSHEINTQSFLVTMKTSF

```
KQVKKQAGLLEVVLIVAVLNGLLSSMGSLIPIMIAAHKTAMVISRYSFTIALMGVVVA

CGAALGSMFGTQLFKKFTIFSMTILATLIALMTTFAIFATSIYLVLGLYFLLALTASA

TSIKLTQWLVTSVEHKILASTVGLLNTILIASAPLMTTILTTVSGATNIRYALILLVV
                     LEVITFLIELKVSHKVKAASIISSIKE"
     CDS             97561..98112
                     /gene="yecD"
                     /locus_tag="OCPHGMPK_00091"
                     /EC_number="3.-.-.-"
                     /inference="ab initio prediction:Prodigal:2.6"
                     /inference="similar to AA sequence:UniProtKB:P0ADI7"
                     /codon_start=1
                     /transl_table=11
                     /product="Isochorismatase family protein YecD"

/translation="MTQEALLIIDYTNDFVSDKGSLTCGKPAQELENEIVNLADSFLK

QNKWVIIPTDLHFPGNKYHPETKLFPPHNLPNTWGRQLYGKLQTWYDANKTNDHVIFM

DKTRYSAFAGTNLDLILRERKIDTLHLTGVCTDICVLHTAMDAYNRCYNLVIHQDGVA
                     SFDENGHKWALNHFKTCLGATIL"
     CDS             complement(98173..99693)
                     /gene="clcA_1"
                     /locus_tag="OCPHGMPK_00092"
                     /inference="ab initio prediction:Prodigal:2.6"
                     /inference="similar to AA sequence:UniProtKB:Q8ZRP8"
                     /codon_start=1
                     /transl_table=11
                     /product="H(+)/Cl(-) exchange transporter ClcA"

/translation="MLQALCVGLIAGVVVGFFRYGIGKTEAFWLDLFKKSHQNPLWFI

VIVAGLALTGIIAGYFVKQYPHVGGSGIPEVKLQMQGKLTLQWWPILWRKLIGGIMVI

GTGLFLGPEGPSLQLGSTIGQGVGQGFKQTKTNSRVLLATGAASGLSAAFGAPLSGAL

FVLEEIFHNFSPRVWMNALVGAIASNFVVSNLFGQKPALALAYNHSFPMPLYWHLVLL

GLILGLLGHLYKLGLFNFKKLYAKITFIPRWLHGLIPLVILMPIMYYLPLITGPGNHL

ILSLPKSIAYSSWNLVGMLAIFYVIRIAFSIVSYDSGLPSGIFLPILTMGALIGATYG

LFMVQLGLLPQKLVINLIIFAMAGYFAAIIRAPFTAIILITEMVGSLLHLMPLAVVAF

IALLVDQLLGGRPIYDSLAAAMEPKSSEKGLCGEEDQISIPVYESSKLVDEKIEDVKW

PDDTLIKVIHRGSQDIIPHGDTVIAAGDLLVLAVDQNRRGQVYDAIKKLQGVELDG"
     CDS             complement(99817..101205)
                     /gene="gnd"
                     /locus_tag="OCPHGMPK_00093"
                     /EC_number="1.1.1.44"
                     /inference="ab initio prediction:Prodigal:2.6"
```

/inference="similar to AA sequence:UniProtKB:P96789"
/codon_start=1
/transl_table=11
/product="6-phosphogluconate dehydrogenase,
decarboxylating"

/translation="MQQFGVIGLSVMGKNLALNVKNHGYSVSGFSIDKPEVDALAKYE

DKDLKPTYTWEEFVNSLEKPRKILIQIMAGKPVDETLQKLLPLLDKGDILIDGGNSNF

HDTNRRYEEMEKHGIHFIGMGVSGGEEGALNGPALMPGGDEKAYEEVSPILEAMAAKT

PTGQPCVGYIGPAGSGHYVKMVHNGIEYGIMQIISEVYDVLRTVGHYSNDEMSEIFKN

WDKGALQAYLIEITAKVLKQKDNLTDDHIIDHILNEASYKGTGNWMLEDAIRLGAPIT

VIAEAVLARFMSKATLRDDPEPKIDPEKAPKDLVDQLAKALYLAMAVSYGQGFEQLTM

AAKAYNWRLHYSTIAQNWEAGCIIRSQMLHDIKFAYNADKDLPNLFKAPFFDKVKKEN

ISALREVVKIAADAGVPTPTLSAALNYLESIFNPRLPQNMIQGQRDYFGAHTYQRNDR
PGTFHTEWYEEK"
CDS complement(101350..103158)
/gene="pox5"
/locus_tag="OCPHGMPK_00094"
/EC_number="1.2.3.3"
/inference="ab initio prediction:Prodigal:2.6"
/inference="similar to AA sequence:UniProtKB:P37063"
/codon_start=1
/transl_table=11
/product="Pyruvate oxidase"

/translation="MTKISGSDAMFQVLYDWGIDHIYGFPGGSFDSTMNAIHNWRDKI

KFIEVRHEEAGALAASAEYKISGKVGVCFGSAGPGAVHLMNGLYDAKYDKTPMVAIVA

NVPTSRQDIDFFQAFDEKPWFDPVAVWNHQAKTAESIPVLMDEAIRQAYQRKGPAVLI

LPKDFGWDKIEDNFRNNAAAHKVVPNFPAPRKEQVDKALELIKNAENPIVYFGHGAED

ASAELKEFSDKFKMPLVSSVLGKGIVEDEFPAYMGSIGRVGAKPSNDIQTHADLVVWV

GNNSPFSVFFFNPNAKVIQIDINSERLGKRHAVTVPMLADAKKTLRALIEAGESRPES

PLYKAALADRENWEAWLESFNDSDEIPMRVEPIFDVINKKAADDAVFAVDVGNVNINF

DRLMHLHGDQKWTTSGLYATMGYGAPASLAAATIYPNREVWNLAGDGGFAMMNQELLT

QARYNMHVINVVFTNETLGYIEAEQVDESHQPLSGVKLPDNDWAMSAEGMNVKGFTVR

NKREFEDAVTAAQQMEGPVLIDCKITHDMPYSTELNTLDDPAFVAKYDAQALKPFSYF
AGKYGVEADAASGASEHTEAEPVEAEEDASSGASRH"
CDS complement(103411..105306)
/gene="mnmG"

/locus_tag="OCPHGMPK_00095"
/inference="ab initio prediction:Prodigal:2.6"
/inference="similar to AA sequence:UniProtKB:P64230"
/codon_start=1
/transl_table=11
/product="tRNA uridine 5-carboxymethylaminomethyl modification enzyme MnmG"

/translation="MKTYESNEYDVIVVGAGHAGVEAALAAARMGEKTLLLTINLDMV

AFMPCNPSVGGPAKGTVVREIDALGGEMGKNIDATYIQMRMLNTGKGPAVRALRAQAD

KWQYHEHMKDTIENEPNLTLRQAVADELIVEDGVCKGLITNTGAKYHAKSVVLTTGTA

ARGKIIIGELTYSSGPNNSLPSIKLPENLEKLGFKLRRFKTGTPPRVDGNTVDYSKTQ

EEPGDKEPRHFSYTSKDSDYLKDQMSCYMTYTNTVTHDIIRANLDRAPMFSGVIKGVG

PRYCPSIEDKVVRFADKDRHQIFLEPEGRNTKEIYVGDFSTSMPEEVQLKMLHSVAGL

EKAELMRPGYAIEYDVIEPWQLKHTLETKNIEHLFTAGQMNGTSGYEEAAGQGLIAGI

NAALSAQNKPGFTLQRDEAYIGVLIDDLVTKGTNEPYRLLTSRAEYRLLLRHDNADLR

LTEKGHKLGLISEERYAKFEAKKQAISRAMAAIKKVTIHPTDEVQEYLASVKQDRLNA

GVSGADFLKRPRVTFDAVEKLSGQTLATDRYVKEQVEIALKYEGYIKKEKTLVDRLHR

LESKKIPVDIDYNAIPSLATEARQKFEKIRPESIAQAERISGVNPADLAILTAYIQQG
RIAKIKK"

CDS complement(105314..106699)
/gene="mnmE"
/locus_tag="OCPHGMPK_00096"
/EC_number="3.6.-.-"
/inference="ab initio prediction:Prodigal:2.6"
/inference="similar to AA sequence:UniProtKB:Q8YN91"
/codon_start=1
/transl_table=11
/product="tRNA modification GTPase MnmE"

/translation="MAQVLTQFDTIAAISTPIGEGGISIVRLSGEDAVAIANKLFKGA

DLTKVPTHTIHYGHIVDPKTQEVVDETMVSVLRAPKTFTREDMVEINCHGGMIVTNDI

LQLLLANGARMADPGEFTKRAFMNGRIDLTQAESVMDIVRAKTDKSRQVAMTQLAGGL

LEKIKTMRQELLDTMAHEEVNIDYPEYDMDDLTSQEMKKKAQEVSKQIEQLLQTAQEG

KIIRNGLATAIVGRPNVGKSSLLNYLTQDDKAIVTDIAGTTRDTLEEYVSVKGVPLKL

IDTAGIHHTEDKVEKIGVERSKKAIKEADLVLLLLDASQDLTAEDKRLLDLTANKKRI

IILNKQDLGTKISQEMIKNLTDNPIIVTSILKQKNMDALENAIEKLFFSGIENSQNQI

LVTNQRQAGLLAKAKQSLEDVISGINDAMPLDLVQIDLKNAWDTLGEITGESAPDELI
                     TQLFSQFCLGK"
     CDS   complement(106808..107683)
                     /gene="misCA"
                     /locus_tag="OCPHGMPK_00097"
                     /inference="ab initio prediction:Prodigal:2.6"
                     /inference="similar to AA sequence:UniProtKB:Q01625"
                     /codon_start=1
                     /transl_table=11
                     /product="Membrane protein insertase MisCA"

/translation="MRNHLSKKNWKLILLGLGMLTLALVVTGCASTNGSSVAPISHTS

GNWWDRYIVYYISQFILWIASLVRDSYGWAIIIFTIIVRIILLPLNAISIKSMAKQQK

VQPQMDALRKKYPGKDVESRQKLQEETSKLYKEAGINPYTGCLPMLIQLPVMYALYQA

IWRTPQLQNGRFLWMDLGRPDPYYIMPVLAAAFTFISTYISQMSTPQSSQNGMTKGMT

YLMPLIIGVSAIGIQSAISLYWVISNLFQVVQTFILQNPFKYQRELEEQKKVERERER
                     KVRRVYKRLGRKKQK"
     CDS   complement(107685..108053)
                     /gene="rnpA"
                     /locus_tag="OCPHGMPK_00098"
                     /EC_number="3.1.26.5"
                     /inference="ab initio prediction:Prodigal:2.6"
                     /inference="similar to AA sequence:UniProtKB:Q2FUQ1"
                     /codon_start=1
                     /transl_table=11
                     /product="Ribonuclease P protein component"

/translation="MRKSYRVKTERDFQKVFKEGQSMANRGFVVYTLPKEKQKHFRVG

ISVGKKVGHTAVARNRLKRFIRATLTELKPEIRSDLDFLVIARPYARDFDMERTKKNL
                     IHVLRLAKVLSNEETETEEK"
     CDS   complement(108102..108242)
                     /gene="rpmH"
                     /locus_tag="OCPHGMPK_00099"
                     /inference="ab initio prediction:Prodigal:2.6"
                     /inference="similar to AA sequence:UniProtKB:Q2FUQ0"
                     /codon_start=1
                     /transl_table=11
                     /product="50S ribosomal protein L34"

/translation="MSTKRTYQPKKRHRSRVHGFMKRMATANGRKVLARRRKKGRKVL
                     SA"
     CDS   108726..110090
                     /gene="dnaA"
                     /locus_tag="OCPHGMPK_00100"
                     /inference="ab initio prediction:Prodigal:2.6"
                     /inference="similar to AA sequence:UniProtKB:P05648"
                     /codon_start=1
                     /transl_table=11

```
                     /product="Chromosomal replication initiator protein
DnaA"

/translation="MFDLDKFWQFFNAEMKKSYSTVAYNAWFKNTKPISFNKKTKEMI

IAVESPVAKGYWEKNLASQLIQEAYAYADMEIQPKFEVAGKEGPERLVTPKPRIKTNQ

EILEDRRDEFAQDLQLNSKYTFDTFVQGEGNKLAAGAALAVADNPGSFYNPLFIFGGV

GLGKTHLMQAIGHQMLAEKPHAKVVYIQSETFVNDFINSIKNKTQAEFRNKYRNCDLL

LVDDIQFFSKKEGIQEEFFHTFETLYNDQKQIVMTSDRLPTEIPELSERLVSRFAWGL

QVEITPPDLETRIAILRKKAETDGLAIDDSTLDYIASQVDTNIRELEGALVKVQAHAT

IEREDINVDLAKEALADLKLVQKNRGLQISKIQEVVANYFQTSTTELKGKKRVKQIVV

PRQIAMYLSRELTDSSLPKIGQEFGGKDHTTVMHACDKISRALKTDAEIKAAVYDLKA
                     MLEH"
     CDS             110275..111405
                     /gene="dnaN"
                     /locus_tag="OCPHGMPK_00101"
                     /EC_number="2.7.7.7"
                     /inference="ab initio prediction:Prodigal:2.6"
                     /inference="similar to AA sequence:UniProtKB:P99103"
                     /codon_start=1
                     /transl_table=11
                     /product="DNA polymerase III subunit beta"

/translation="MQFTINRNLFLENLNNVMRAISSRATIPILSGIKLNLTDEMLTL

TGSDTDISIEIQIPVNDDLIVQSTGSIVLPARFFSEIVKKLPGKDFSFEVKESFQTKI

VSENTEFMINGLDANNYPHLPEISTDASFKISGKTFREIINETVFAVATQESRPTLTG

VNFIFNNSSIKAVATDSHRLSQRQISLENGPQTSTDLIIPGKSLVELARIIGESDPEI

KVNPGENQVLFEVGNIAFYSRLLDGQYPDTDRLIPTESTTSVEFELPVLARSLERASL

LTHESRNNVVKMTLDVQNQLVKLQGDSPEIGNVEEEIGFKNLEGEGLTISFNPDYLRE
                     ALRASITDSIIMNFTQPLRPFTVVPAKQDVNFTQLITPVRTF"
     CDS             111683..111832
                     /locus_tag="OCPHGMPK_00102"
                     /inference="ab initio prediction:Prodigal:2.6"
                     /codon_start=1
                     /transl_table=11
                     /product="hypothetical protein"

/translation="MISSGGQAKWYLKENPVKLNGELEDRRGKKIHAGDVLNLAGEEY
                     EFVSE"
     CDS             111836..112960
                     /gene="recF"
                     /locus_tag="OCPHGMPK_00103"
                     /inference="ab initio prediction:Prodigal:2.6"
```

/inference="similar to AA sequence:UniProtKB:P9WHI9"
/codon_start=1
/transl_table=11
/product="DNA replication and repair protein RecF"

/translation="MYLANFELKDFRNFKELKTNFDPHVNIFIGPNAQGKTNLLEAIY

FLALTRSHRTNSDKELIRFGSKFAGLQGRVHKSQLQVELKLRLTANGKKAWVNRLEQK

RLSAYVGQMNAILFSPEDLALVKGAPSVRRRFMDLEFGQINSEYLYFSSQYRQVLQQR

NNYLKQLSIKKANDQVFLDVLSDQLAGIAAEIISRRIKYIKKLNSYAQAAHREISGQA

EKLQIFYRPSVKEIIPEDDVETIYQKVITSYKKNRSNEIRKGTTLSGPHRDDLEFLIN

DKNAHDFASQGQQRTISLSVKLAEIQLVHELTQEYPILLLDDVMSELDHRRQSRLLNY
IHGKTQTFITTTDLEGISWEIVKEPKVYHISAGTISAKES"
CDS 112961..114928
/gene="gyrB"
/locus_tag="OCPHGMPK_00104"
/EC_number="5.6.2.2"
/inference="ab initio prediction:Prodigal:2.6"
/inference="similar to AA sequence:UniProtKB:Q839Z1"
/codon_start=1
/transl_table=11
/product="DNA gyrase subunit B"

/translation="MAEDKSKAALNKAKDFEKRADTYNASQIQVLGGLEAVRKRPGMY

IGSTSSQGLHHLVWEIIDNSIDERLAGFATKIEITVNEDGSVTVQDDGRGIPVDIQAK

TGRPALETVFTVLHAGGKFGGGGYKVSGGLHGVGASVVNALSTKLDVTVMRDGKKYAI

SFDHGRVVGEMKQIGTVPLEEHGTIVHFYPDPDIFTETTTFDDKILKNRIRELAFLNK

GLKLTFTDKRKESAETDVYHFEGGIKEYVAFLNKGQEVLFDEPIYVEGNYEGIDVEVA

LQYTNGYKTTLMTFANNIHTYEGGMHEAGFKTALTRVVNDYAHKAKILKDKDDNLSGE

DIREGITAVVSVKHPNPQFEGQTKTKLGNSDARTAVDRAFSETFSRFLMENPSVGRKI

VEKGQLAERARTAAKRAREVTRKKSGLEIANLPGKLADNTSNDPSISELFIVEGNSAG

GSAKQGRSRLTQAILPIRGKILNVEKASMDRILANQEIRSLFTALGTGFGADFDVSKA

RYHKLIIMTDADVDGAHIRTLLLTLFYNYMRPMIEKGYVYIARPPLYQVRQGKIEKYL

DTDEELHDYLGTLQPSPKPVVQRYKGLGEMDPEQLWETTMNPENRRLDRVSPEYAEDA
DKIFDLLMGNEVGPRREFIEKNAKYVENLDA"
CDS 114939..117428
/gene="gyrA"
/locus_tag="OCPHGMPK_00105"
/EC_number="5.6.2.2"
/inference="ab initio prediction:Prodigal:2.6"

/inference="similar to AA sequence:UniProtKB:P05653"
/codon_start=1
/transl_table=11
/product="DNA gyrase subunit A"

/translation="MANENQPQDHRIRNVDLTHTMRSSFLDYAMSVIVARALPDVRDG

LKPVQRRILYGMHELGVTPDKQYKKSARIVGEVMGKFHPHGDSSIYLAMAHMAQDFSY

RYMLVDGHGNFGSVDGDEPAAMRYTEAKMSKIAVEMLRDINKETIDWQRNYDDTENEP

VVLPARIPNLLVNGASGIAVGMTTNIPPHNLAEVISGIHMLMDNPDVTTKDLMKVIPG

PDFPTGGIVMGRGGIYRAYETGKGNIVVRAKTNIETEKSGRERIVVSELPYLVNKAEL

VKKIADLAREKTIDGITGVRDESDQTGMRITIDIRRDASASVVLNNLFKETQLQANFG

MNMVAIVNGAPHFLTLKQMLEYYLHHQEDVITRRTKFELKKAEARAHILAGLRIALDH

IDEIIRIIRGSQNSDIAKAQLIQNFGLDDRQSQAILDMRLVRLTGLERDKIEAEYQDL

QAKIADYKDILAKPERIDEIIYNELLDIQKRFGDDRRTKIAEGEAVSIEDEDLIEQKD

VLLTLTHNGYIKRMPADEFKVQNRGGRGIKGMGVQDDDFINHLIFSSTHDFLLFFTNL

GKVYSKKAYEIPEFGRNAKGLPIVNLLELDKGEKIQAVINVPEGADDNYLFFVTKMGT

VKRTKVSEFQNIRRSGLIALTLRDGDELNNVLTTDGKQNILIGTHLGYAVTFNENDVR

SMGRTAAGVRGINLRDNDYVVGSDILEPNSEVLVISEKGYGKRTAASEYPVKGRGGKG

IKTANITEKNGPLAGVTVVNGDEDIMLITSAGVMIRFDVDDVSQTGRATLGVRLIKVD
DGAQVASITAVPKANDEDEKSTDEEEPVEEN"
CDS 117615..117911
/gene="rpsF"
/locus_tag="OCPHGMPK_00106"
/inference="ab initio prediction:Prodigal:2.6"
/inference="similar to AA sequence:UniProtKB:Q2G113"
/codon_start=1
/transl_table=11
/product="30S ribosomal protein S6"

/translation="MAKTKYEVTYIIKPDIDEDSKKALIDRFDKVVTDNGAEELESKD

WGKRRFAYEIEKYREGTYHIMTFVAENSEPVDEFGRLSRIDNQVLRSMTVKLDK"
CDS 117945..118463
/gene="ssb"
/locus_tag="OCPHGMPK_00107"
/inference="ab initio prediction:Prodigal:2.6"
/inference="similar to AA sequence:UniProtKB:P66854"
/codon_start=1
/transl_table=11
/product="Single-stranded DNA-binding protein"

```
/translation="MINNVVLVGRLTRDPDLRTTGSGISVATFTLAVDRQYTNSQGER

GADFVSCVIWRKAAENFANFTSKGSLVGIQGRIQTRTYDDKDGKRVYVTEVIVDNFSL

LESRRDRENRQTNGGNFAPQGGNAPSTNNFGGSSAPSTNNAPAGGESNKPQDPFADSG
                     STIDISDDDLPF"
     CDS             118488..118724
                     /gene="rpsR"
                     /locus_tag="OCPHGMPK_00108"
                     /inference="ab initio prediction:Prodigal:2.6"
                     /inference="similar to AA sequence:UniProtKB:P10806"
                     /codon_start=1
                     /transl_table=11
                     /product="30S ribosomal protein S18"

/translation="MAQQRRGGRRRRKVDFIAANHIDYIDYKDVDLLKRFISERGKIL
                     PRRVTGTSAKNQRKLTVAIKRARVMGLLPFVAED"
     CDS             118819..119736
                     /locus_tag="OCPHGMPK_00109"
                     /inference="ab initio prediction:Prodigal:2.6"
                     /codon_start=1
                     /transl_table=11
                     /product="hypothetical protein"

/translation="MIIKQKLAGNIDFDYKWYDIYNCDDHDIRLLKDDFDLTSEIISY

ITDLHERPHFDHDYITNSDLLVYDVPVWPTADADHFTTLPIKFLMVGHTLFTVHSPDT

TYMIEEFRQKPDEHIHSEKELIFAILFAVTKYFQRALSQLNSQRLVLDNHLSERIHNK

DLQELSQVEKSLVYLSSSIRTNLMMLESLKNKKSGLHMNASEEEMCDDIIIEVQQSSQ

MIKIYSEVTEEISKTSNNILNNNLNNTMQFLTVWSLLLTIPTIVTGFFGMNVDLPIFH
                     NPFDWVIITVGSVIVMAILLWYLRRHNML"
     CDS             119842..121860
                     /gene="gdpP"
                     /locus_tag="OCPHGMPK_00110"
                     /EC_number="3.1.4.-"
                     /inference="ab initio prediction:Prodigal:2.6"
                     /inference="similar to AA sequence:UniProtKB:P37484"
                     /codon_start=1
                     /transl_table=11
                     /product="Cyclic-di-AMP phosphodiesterase GdpP"

/translation="MKGFLRKLELPEFVKDTRLTASLVIVLVLSLLGAIIGMLISPLF

GLAMVLLFILTVIFAVYGIYVLAGNTNNYAANLSYRIKRGEQEAMIKMPLGIMLYDKD

RQIQWINPYLQMYLHDKDIIGSSISSVDKELAKYIDDAIKSNSKQNKIIKWGDRKFEM

VVQDDLGVVYLLDITRYANIEEKYKQERLAIGLIFIDNYDELSQAMGDQNLTNMSSYV

QTTLSKYAGQFNSYLKRIDEDHFILLTHMHDLAKMEDNKFSILDKVRTESSRKNMPLT
```

LSIGIAFGSESLNEIADQAQSNLDLALGRGGDQVVVKQSGHEARFYGGKSNPMEKRTR

VRARMVSQALVELFKGVDHVFVQGHRNPDLDAIGSAIGIVKIARIHGVKASVVLDVDH

VNYDVGRLIAKMQAAGIDKDVFISPKDALEAATDESLLVLTDHSKYSITYDPELYKRL

KNRLIIIDHHRRGEEFPENPMLVYIEPYASSTCELVTEMIEYQPQGGEGVLTDLEASA

MLAGITVDSKEFSLRTGTRTFDAASYLRSIGADTQVVSELLKENIDNYLQRSHLVSTI

DMIEPNMALLMGEDNKIYDPIITAQAADTALSLEHVDASFALTRRSKDVIGISARSMG
NVNVQVIMEKLGGGGHLSNAATQIKGVTVEEAKDKLLAAINEYLKENE"
CDS 121872..122327
/gene="rplI"
/locus_tag="OCPHGMPK_00111"
/inference="ab initio prediction:Prodigal:2.6"
/inference="similar to AA sequence:UniProtKB:P02417"
/codon_start=1
/transl_table=11
/product="50S ribosomal protein L9"

/translation="MKVIFIKDMKGKGKRGEVKNVPDGYAQNFLIKNGYAKEATSSNL

NTLKRVQKAEKDAYEAEKAAAEDIKQKLEDDKTIVNFKSKAGTDSRLFGSISGKKIVE
GLEKQYGIKVDKRKLELPEPIKSLGYTNVPVKLFKGVEAVIRVHIMEQD"
CDS 122330..123703
/gene="dnaC"
/locus_tag="OCPHGMPK_00112"
/EC_number="3.6.4.12"
/inference="ab initio prediction:Prodigal:2.6"
/inference="similar to AA sequence:UniProtKB:P37469"
/codon_start=1
/transl_table=11
/product="Replicative DNA helicase"

/translation="MDNVVSQQIPHDSEAEKAVLGAVFLDPEAIIDASDILQPDDFYE

HANRIVFQAMLNISDREEVIDPVTLQDELKKNNQVDDIGGIAYVTELSMATPTAAHVT

YYAKIVKRKAILRNLISTSQRIIQNAIEGSDDVTDILDDAESQIMGVSQDNASGGFKS

IHDVLNTTMEEINSIPDDGNTVTGLPSGFSELDKMTTGFHDDELIIILAARPGVGKTAF

ALNVAQFVGLKTDKTVAMFSLEMGAEQLVQRMLASEGLIDSQHLRTGQLTDEEWRKLV

VAAGSLDNTSIYIDDTPGIKMSEIRAKARRLAKEKGNLGLIVIDYLQLIEGPRSESRQ

QEVSAISRQLKKLAKELHIPVIALSQLSRSVEQRQDKRPVLSDIRESGSIEQDADIVA

FLYRDDYYRDERDEDDEGEVEAEEDNGEVEVIIEKNRSGTRGTVKLMFSKPYNRFSNL
DYTHNEA"
CDS 123796..124680
/gene="gmuE"

/locus_tag="OCPHGMPK_00113"
/EC_number="2.7.1.4"
/inference="ab initio prediction:Prodigal:2.6"
/inference="similar to AA sequence:UniProtKB:O05510"
/codon_start=1
/transl_table=11
/product="Putative fructokinase"

/translation="MVENKYVGSIEAGGTKFILAVQDTESGEVVAKKRIPTTDAKETL

AKSIEFFKEHPVSALGIGTFGPIDINPNSRTFGYILDTPKRGWSGTNVKGTFEKELDI

PVVMTTDVNASCYGEYIARGRDNSKTYFYVTIGTGIGAGAVQAGKFIGLNNHPEMGHM

LVTPYPGDNYTGKCPFHGNKCVEGMAAGPSLEGRTGIPGEKLPRDHKVFTYVSYYVAQ

LLFNAYMTMRPDVMVVGGSVLNDQDLVQVRGFFKEFNNNYVATPDVDELIVRPAVADN
GSATLGDFELAKEALKNK"
CDS complement(124758..125900)
/locus_tag="OCPHGMPK_00114"
/inference="ab initio prediction:Prodigal:2.6"
/codon_start=1
/transl_table=11
/product="hypothetical protein"

/translation="MGKILDYVRWRGDLTLDREPFNSLDAGLFAAFAYLPFDSSVKGH

TLEAATKRLIQKKTLIHSDKLEAELVNLSDRYKNMRFLDWSHLSQKKPPVQFTAMTIE

LDSETILVAYRGTDGSMVGLNEDVDMSYQPEIFGQSVAADYLDKMAQAYPDKKIYTVG

HSKGGNFAAYALYAASPSLQDRVIKAYSFDGPGFMKEMYNQIEFQRVIPKMMTYVPEG

SIFGMMLDHPERVIVVKSRMKHLMKQHNPLHWEVARNSFVMAPGLSNASRIIRSTFIS

WNTKIPREKREAFWLAFFTALESQKITEASQLTTNKIRGAIQFSHAYLALDPDARLIA
NEIISDITATARQYINLPFLSHNEHLEPLGNDSSKGPIVDDSYDGS"
CDS complement(126029..128047)
/locus_tag="OCPHGMPK_00115"
/inference="ab initio prediction:Prodigal:2.6"
/codon_start=1
/transl_table=11
/product="hypothetical protein"

/translation="MSYIYTLRYVFDPRTNTPEKDRKLLDFVQKAKIDDVAFIINGEE

LNHSHLTKEETQVWLDAIIPLQKQLAKLDVTTSLNPWTTIMHSDRGFSVNPEIGFNTF

VDINGNQAKDMACPADPTWRKYLADRYAQYASIHPRRLWMEDDFRHYNHTPLKLMCFC

DYHMKLYQEKLGKTESRAGFVKNMLKPGEPTIERKIYLDQARKEMIENEHLIEQAVHK

VSPNTDLGQMTSFPDWHAIEGRDWPSLFDAQAGPGHPRVARPHLPSYNEVAPLIYGRK

```
FEEYSRTTAAFLGPRAELYPELENSMWTPQVKSNTFIAFQIITTALLGAKGIMLNIFD

MMGNGVNEDWGYDKMLADIKPFASALASHRLNMQKLRGIKVLVDQDSAYTAHTTFGKE

PEELLPHEKNWASLLASFGFATTILPVQNDLAIENQTIAIAGQLLRNLSNEQITNLIT

KNTVLLDGESIQVLLDRKLGNLLHIQSAKWHPCKSGYQSFEQADGTTVQGVKNPRITM

LQHTGNYLQLDYQTDSNVKIWSSAYNSIDQKLGNFRAVIDDHIIVLPMDQDPKHGWES

QFSSYKQGLMQQIMASVEPIDYLINMPHVKLNIQENGKIVWLANFNLDGYQEINWHLS
                     KPLPSNTATLIRRRDNSVEKEAVKIEVTDNVASIKAPLSPLETIQLIF"
     CDS             complement(128154..128855)
                     /locus_tag="OCPHGMPK_00116"
                     /inference="ab initio prediction:Prodigal:2.6"
                     /codon_start=1
                     /transl_table=11
                     /product="hypothetical protein"

/translation="MVSLTSLSTELDWLLRILVAALCGTAIGYERATQRKSAGIRTHM

VVAVASALFMLVSKYGFFDLLSLHDISLDPSRIAAQIVTGISFIGAGTILVRREQISG

LTTAAGVWATAAIGMAVGAGMYVIGIVSTVFLFIIQIIFHDDTFINFIILHVRFNVQI

EANNHPDILNELKKELSLNEVEQISIKIIDVTSEHIVVNVDGVIRNRKDENDIILALE
                     KDQNIRRINSTHGAK"
     tRNA            128993..129065
                     /locus_tag="OCPHGMPK_00117"
                     /product="tRNA-Thr"
                     /inference="COORDINATES:profile:Aragorn:1.2"
                     /note="tRNA-Thr(agt)"
     CDS             complement(129323..129949)
                     /gene="ppaX"
                     /locus_tag="OCPHGMPK_00118"
                     /EC_number="3.6.1.1"
                     /inference="ab initio prediction:Prodigal:2.6"
                     /inference="similar to AA sequence:UniProtKB:Q9JMQ2"
                     /codon_start=1
                     /transl_table=11
                     /product="Pyrophosphatase PpaX"

/translation="MIKTILFDVDGTIVDTQKVVLSTFKEVIKEYLNANVNINDLAFA

LGIPGRDAIKHFTNNKVLINKMAKSWSEKASNRVNEDTLFNNIPETIKSLSQEGITLG

IVTSKTKQEYINEVSHFGLDDYFDVIVTASDTKNHKPNPEPLNFAISKLGVDRTETLY
                     VGDTKYDMLTANAAKVKFALANWGAHEELPEADYQLNNPINLEEIVKG"
     CDS             130323..130517
                     /gene="sinR"
                     /locus_tag="OCPHGMPK_00119"
                     /inference="ab initio prediction:Prodigal:2.6"
                     /inference="similar to AA sequence:UniProtKB:P06533"
```

```
                     /codon_start=1
                     /transl_table=11
                     /product="HTH-type transcriptional regulator SinR"

/translation="MNRVKEYRKQKGLSQMALAKRIDVARQTINLIENDKYNPSLDLC
                     LKLAHELDTDLNTLFWDGDV"
     CDS             130520..131038
                     /locus_tag="OCPHGMPK_00120"
                     /inference="ab initio prediction:Prodigal:2.6"
                     /codon_start=1
                     /transl_table=11
                     /product="hypothetical protein"

/translation="MKAKETRFEKYMKFWYGISGPLDEQKRQVMNQIGNRAFFIFFLG

MDVAFLISLVLFSMYKADVAYLFLSGSFFVMLMAVNLYIMQQSRKARLTDNEVEKDEL

PEAKKAAWTKGMISGAIFGILMLILEFLTSYSDGSWTWIMIPTCLISGVFFGIFMSLI
                     AVHRIKVVKDED"
     CDS             131172..132125
                     /gene="ydhF"
                     /locus_tag="OCPHGMPK_00121"
                     /EC_number="1.-.-.-"
                     /inference="ab initio prediction:Prodigal:2.6"
                     /inference="similar to AA sequence:UniProtKB:P76187"
                     /codon_start=1
                     /transl_table=11
                     /product="Oxidoreductase YdhF"

/translation="MKTTKLGKTSLDIPAIALGVMRMGTRTPEEGAAAINAAYKAGVN

FIDSADIYGRGNSEKVFGKALKLADVNREDLFIQSKTGIVVGSSGSHGSLAYGSRYEF

TKEHILDAVDGILKRMDINYLDCLVLHRPDILMDLEGIKQAFDILQATGKVRFFGVSN

FNPAQFKMLQNAVSQPLMFDQVQFGLMHTGMVDFGLHTNMTDDASINHDGSLLEFLRM

NHITLQAWSPFQYGTFAGTFINNPAFPELNKKLQEIGDKYSVGKNAVAAAWILKWPGQ
                     VQVIMGTMTPAHIVDSAKGADIELTNQEWYDLYFAAGHDLP"
     tRNA            132181..132254
                     /locus_tag="OCPHGMPK_00122"
                     /product="tRNA-Thr"
                     /inference="COORDINATES:profile:Aragorn:1.2"
                     /note="tRNA-Thr(agt)"
     CDS             132369..133532
                     /locus_tag="OCPHGMPK_00123"
                     /inference="ab initio prediction:Prodigal:2.6"
                     /codon_start=1
                     /transl_table=11
                     /product="hypothetical protein"

/translation="MKLSQNKETLDQIAPLVEYAFLKNNDIREDPNFLSRYNHSMGFG

EYKDNHLASYIMVNEFKSRIFAKKVKMGGVGYVASYPENRGQGDINRLMKEIILELHD
```

QNYAISDLAPFSETFYRRYGYENTIYEKMYQIRPEYLRFFKPIKEGKVVRGRWSDSSL

KEAVIKLYQAKLNQGEQRNTVDRADWWWDRLDTYYPGRLICVYFDKFQQPQGYMFYRI

VERNFRVEEMYYQTPQAAQALLSIIASHSSSDLKYYVKMPEESLLGEFFPEQEGITIK

TIPYMMTRIIDMKQVLEALRLVNNKKLTIEVTDDDIIVENNGIWFVNDAAEETRVRKV

ETDPDYTASLTNWTKVLLGHLTLKQAVQLGFVKENHPVNTDFVKGEVSFYDYF"
     CDS   133684..134331
                     /gene="dapB"
                     /locus_tag="OCPHGMPK_00124"
                     /EC_number="1.17.1.8"
                     /inference="ab initio prediction:Prodigal:2.6"
                     /inference="protein motif:HAMAP:MF_00102"
                     /codon_start=1
                     /transl_table=11
                     /product="4-hydroxy-tetrahydrodipicolinate
reductase"

/translation="MKVGIIGASGMAGSAIYKLASQNKDLEVTGIVRDKDKDKRVLGK

DAKLLIGDVLTMDDSLLQEFNVIVDAFGTTPERARDQVKLATKLCGLAKNKDIRVIFI

LGAGSLRTGIDNHLVVEDIKKLTGSENWINTPKEQLNELNYLKQVEDVDWLGISPALT

FEAGPEAAGYMLGNDTLLYNDKNESKVTSGTMARLVVNEIVHPEHHKERITLVDE"
     CDS   complement(134385..135074)
                     /locus_tag="OCPHGMPK_00125"
                     /inference="ab initio prediction:Prodigal:2.6"
                     /codon_start=1
                     /transl_table=11
                     /product="hypothetical protein"

/translation="MTRAQLKQAAKDQLRGNWGWAICLTIFAWLVNAVIMDLNRWIWT

GKDFTYTVLRFNKDNLIQGYKPAYNLSEIIVGLITGLILWGVAYTILDFVETGKMEPW

YSGIFSAYSNGRFKNSLFTLFMTNLFVSLWTILFVIPGFIKGYSYAMTPYILKDKFSA

GLTDIGATEAITESRKLMDGHKMDLFILDLSFIGWGLLGLITCGIGFIWITPYYRQTK
                     ANFYRSLVANN"
     CDS   135214..135921
                     /gene="yfnB"
                     /locus_tag="OCPHGMPK_00126"
                     /EC_number="3.-.-.-"
                     /inference="ab initio prediction:Prodigal:2.6"
                     /inference="similar to AA sequence:UniProtKB:O06480"
                     /codon_start=1
                     /transl_table=11
                     /product="Putative HAD-hydrolase YfnB"

/translation="MKYKQLIFDVDDTLIDFAATEDSSLHALFKSHKLPLSSDLQKQY

```
HTYNQGLWRRLELGEITYDELSEMTFHDFIKENFNLDVDGNEWMNEYRSYFGEAHQLL

PGVEDTLKFAKKQGYKLTVLSNGEKFMQRHRLELAGIKDYFDLIVTSEEAHYSKPNPH

AFDYFFSRTEIGPNETLFFGDGLQSDILGAEKYGFDSIWYNHRHRKDTIGLHPLFEVE
                     TYPEFVKIMQNDFQKAY"
     CDS             complement(135957..136796)
                     /locus_tag="OCPHGMPK_00127"
                     /inference="ab initio prediction:Prodigal:2.6"
                     /inference="similar to AA sequence:UniProtKB:P67372"
                     /codon_start=1
                     /transl_table=11
                     /product="DegV domain-containing
protein/M5005_Spy1226"

/translation="MPIKIITDSTANITADQIDGIEHITVPLSLHVDGKLWMDTPDID

LDKFLHTLKHTKSKSTSACPNVNDWLKSFEGSDEIFVITLTSALSGSYNSATQAAKIY

HEKNPKAKILVFDSRSAGPQLRLLAEKIASLANAGKSFEEIVKATEEYQHQLDLMFAL

QDLTNLGNNGRIPPAVAKIAGLLKLFVIGTANNKGEFELLGKARGAKKARRKLLDEMV

KAGYKGGRVQIDHVQNEGSALSMKEIILDNFPDAKVTIGECGALCSFYAEDGGLMVGF
                     EKE"
     CDS             complement(136908..137093)
                     /locus_tag="OCPHGMPK_00128"
                     /inference="ab initio prediction:Prodigal:2.6"
                     /codon_start=1
                     /transl_table=11
                     /product="hypothetical protein"

/translation="MFSILLALLIIWLFWKLGIGMLKIVGFLIIVGLLFAFVSYLVLP
                     LLALIALGGLLGLVFSR"
     CDS             137458..139380
                     /gene="tetM"
                     /locus_tag="OCPHGMPK_00129"
                     /inference="ab initio prediction:Prodigal:2.6"
                     /inference="similar to AA sequence:UniProtKB:P21598"
                     /codon_start=1
                     /transl_table=11
                     /product="Tetracycline resistance protein TetM from
                     transposon Tn916"

/translation="MKKLTIGILAHVDAGKTTLSEGLLYQSGTLRKLGAVDKGTAYLD

SDNLEKKRGITIFSHMAHIQNENSELLLLDTPGHIDFAQEMEETLGVLDYAILVVSAS

EGVTAYTQTLWNLLKNHKIPTFIFVNKMDTLKADKEKVLHDLSILDDSCVEFGDEGSD

FYEKIATADEAILEEYLESGQIKDEEIKDLIFQRRIFPVYFGAALKLKGVAEFLQGLD

KWTKKIEYPEKFASRIFKISHDEKGERLTWLKVTGGELRAKTELMPDEKVNEIRLYNG
```

TKYQVVPSAQAGEIAAISGLKTTYPGQGLGFERDQTNFTMQPVLTYAVQTVPDKMHAT

LTALRQLEDENPQLHVKWDKQAEEISIDVMGKIQLEILQQILHDRFGLKAEFAQGKIL

YQESIKDSVEGVGHFEPLRHYAEVHLLLKPGKLGSGLVFKNECSLEVLPKKWQDQVMD

SLANKEHLGVLTGSPLTDMEITLVGGRGSNVHTVGGDFREATYRALRQGLMELKAQNQ

VSLLEPWYQFTLRINREQVGRAINDIEKKGGKFEIGESDHDVVTITGQAPVAQMQDYA

TEVRNYTHGSGQLECLFAGYRKCKNSAEVIESINYDPVSDIYNTPNSVFCSHGAGHTV
VWNEVPDYAQYPYLQS"
CDS complement(139415..140341)
/gene="corA"
/locus_tag="OCPHGMPK_00130"
/inference="ab initio prediction:Prodigal:2.6"
/inference="similar to AA sequence:UniProtKB:Q9WZ31"
/codon_start=1
/transl_table=11
/product="Cobalt/magnesium transport protein CorA"

/translation="MLTVYDKNGYITSDNLLSAKLVILVNPTSEELTNIEKTIGCDAH

VFTKQTSATEVSHFNVLPKCKIENARIFVSFNFDAGQTQVEDAIYPTIAIFNSQQLIL

VLQKKPAPILENQKAIAKLSVEDLLIKQLLYQNHCFNEELTKIKQQLDQLDHAARTTT

KTKSLKEATDLTRTLVYFNHTMDDQASSIEALTDFLKKQHSANSIFIIDLLLSQRRIT

KKIKVYRDLLESINGLFSAMMDNHLNNLMKYLDSIALIISIPALISGIWGMNVGGLPG
KDNEFGFLWVILFGAALAIISALLLRKKNYLK"
CDS 140632..140796
/locus_tag="OCPHGMPK_00131"
/inference="ab initio prediction:Prodigal:2.6"
/codon_start=1
/transl_table=11
/product="hypothetical protein"

/translation="MMKDKHGIKDKVAGKFKEVEGKVTGDKARELEGKAQQAKGKVKS
KATKVKEDLE"
CDS 140831..141097
/locus_tag="OCPHGMPK_00132"
/inference="ab initio prediction:Prodigal:2.6"
/codon_start=1
/transl_table=11
/product="hypothetical protein"

/translation="MLHWIWVLIVGGVIGLIAGAITHKGGSMNWIANIIAGLVGSSLG
EALLGAWGPQVAGMAIVPSLLGAIILVILVSWIMTMLFGPRASE"
CDS 141233..142060
/gene="exoA_1"
/locus_tag="OCPHGMPK_00133"
/EC_number="3.1.11.2"

```
                     /inference="ab initio prediction:Prodigal:2.6"
                     /inference="similar to AA sequence:UniProtKB:P37454"
                     /codon_start=1
                     /transl_table=11
                     /product="Exodeoxyribonuclease"

/translation="MRLISWNIDSLNAALTSDSARAKLTRGVLNTIKEKDPDIIAIQE

TKLRSTGPTKKHQEILAEMFPDYDYVWRSSEEPARKGYAGTMFLYKNTLTPEVTKPVI

GAPEPMDHEGRILTLEFDNYYVTQVYTPNSGNELKRLDDRQVWDEKYTEYLQKLDQVK

PVIASGDYNVAHTPIDLKHPENNHHNAGFTDEERQDFDKLLKLGFTDTFRKIHGNVEG

VYSWWAQRVRTSKANNSGWRIDYYIVSNRIANQVTKSEMLDTGERKDHCPIMLEINL"
     CDS             142094..144967
                     /gene="radD"
                     /locus_tag="OCPHGMPK_00134"
                     /EC_number="3.6.4.12"
                     /inference="ab initio prediction:Prodigal:2.6"
                     /inference="similar to AA sequence:UniProtKB:P33919"
                     /codon_start=1
                     /transl_table=11
                     /product="Putative DNA repair helicase RadD"

/translation="MIFFFSLGMEVKAVDNVLKDAILNGLYNKNENKGNEFISPQLVN

NDDQSKIWLTLRQELMSCTSFTWAVAFISENMLVPFKLVMSELAKKGVNGTLITGTYL

GFNSPKVFKELMKIPNLTVRLSEKTGFHAKGYIFNHEDYQTILIGSVNFTRSALLKNC

EWGLKVTSHENGQLVEEVNDQIKNNLSNSLILTSSWIKEYEQNWQPIKSNPHLLLRKS

KKIAPNQMQKEALANLDALVKEQAKRALVVSATGTGKTYLGAFAVKEYKPKKFLYLVH

REQIAKKALESFYQVIGGKKSDYGLLTGNKHDFDKKYLFGTVQTVSQEQILGQLNPEE

FDYILIDEAHRVAAPTYQKILNHFIPKFLLGMTATPERMDKQNIYEIFDYHLAYEIRL

KDALNEQMLTPFHYVGVEDYTVGNQIITETSKLKDLVAEKRVEYVLKQLNYYGYCGEK

AKGLVFCSRQEEARELAKLFSQAGHPSRALTNEDSQKRRAEVTQQLENGELEYIFTVD

LFNEGIDIPSLNQIVMLRNTQSSIIFIQQLGRGLRKFPGKDYVTVIDFLGNYKNNYLI

PIALNGDKSRDKDQAIRESKLPQVIDVSTITFTEIASQRILASLEKIKLDSMHELRQS

YEDLKEKIGKVPLLCDFYQYGTTAPTVFAKNHALAHYGAFLKKMGEEVNLDNYQNAVL

SFVTKELLFGKRPHELLLLESCLNKNISKADFIKLLKNKNCYVSKDVLESVEKILSLE

FFDVKQGKATKKEQYGIYPLIEKENGEYHLSNQLFQALNDNQQFKILFKDVLKTGLEL

SKEYDSQSQFTLYKQYDRKDVCRLLNWPKDVSAPMYGYRVGEKETPIFITYQKDSKKK
```

RNARYQNTLENGHSLRWYTRTPRHLDSDEVQRLLYTDEMKLHLFVKRSDAAGKEFYYL
GTADIQKDSVKEEKIGLKQKSAVGMNLVLNHPLKQSIYNLLFS"
CDS 145062..145949
/locus_tag="OCPHGMPK_00135"
/inference="ab initio prediction:Prodigal:2.6"
/codon_start=1
/transl_table=11
/product="hypothetical protein"

/translation="MKNKLVFVLALLVIVLTGCTTQDKPNQADAAPKYYVEKRQTPKT

NLNVIPTLFFHGGLSNYKGEENMVKAAQKAGVTNSIIRANVDANGKVKLIGTISKNAI

NPIVEVNYRNNVQLDFKEHGRYARNVVQTLQNEYGIKKVNMVGHSLGNISIMYYLLQT

AHDPEMPKLNKQVSIGGHFDGLDFKQLPIAIRQPSNLHVDAEGKPNKTNSTYREMMKL

RTLYPNKQTSVLNIIGNIGGNSDGIVKNASSLSLEYLVEPMAKSYRVVKIVGKNAEHG
QLTYNKQVEKEIIKFLWLQ"
CDS complement(145993..146244)
/locus_tag="OCPHGMPK_00136"
/inference="ab initio prediction:Prodigal:2.6"
/codon_start=1
/transl_table=11
/product="hypothetical protein"

/translation="MKAVSVMKKLWWGANIYWVILLIYGGIKLFTYNFDTSELGETAS
YALIIMVLISASVLIIEFQAAWGMWLHNFDKSNQHENKI"
CDS 146360..147409
/locus_tag="OCPHGMPK_00137"
/inference="ab initio prediction:Prodigal:2.6"
/codon_start=1
/transl_table=11
/product="hypothetical protein"

/translation="MGQAWENFKKNTNVHRLATLCLIILVLYAARSMMNTILLTFIFT

YLIVHLIRFTQKKLPNLPSQVIVVLAYLLVIAIIYFAITIYVPILIKQIVKMSHSLMK

FYQSDNMDWLTQYLNHYISDSEITTQAKHGVTILVHALTNVGTLTIAFFMSLIMSFFY

TIELDSMNEFSHTFLNSRHLSWLFKDIAYFGNKFVNTFGVVLEAQFFIALCNTAMTMI

CLIIMKMPQIIALGLMVFILSLIPVAGVIISLIPLSFVAYSVGGIRYVIYIFIVIMII

HAIEAYILNPKFMSSKTELPIFYTFVVLLAGEHFLGTWGLIVGVPIFTFLLDILGVKS
VKTKKPKILKIENKK"
CDS 147651..148982
/gene="garB"
/locus_tag="OCPHGMPK_00138"
/EC_number="1.8.1.16"
/inference="ab initio prediction:Prodigal:2.6"
/inference="similar to AA sequence:UniProtKB:D0VWY5"

/codon_start=1
/transl_table=11
/product="Glutathione amide reductase"

/translation="MKKYDYIILGTGPAAYQFIKLLATQHKSILVIESGLFGGTCPNV

GCEPKIYLDGAVQAALLSKQLEGQGIEQAAAMNWSQLMKEKKERFTSWPDETKEIIGK

TCDVISGSAHFINRQTIAVNQKYFQGNKIIIATGRRPHELSIPGAKFLHDSSDVLSLN

KVPEHATFIGAGYVAMELATFLAAAGSQVTILVRGEQVLRHFYQKYSSDLVNRMMKRG

IQFRFVTEATRITQLSDKYVVELNQGSSLVTDYVVNASGRTPNIEKLDLSAAQIDYSP

KGIEVDRHLQTNVNNIYAIGDVTSQDVPNLTTVAEFQARYLFNSLEKGLSQPIDYPAI

GTGVFAFPQLAQAGVNPDSVSEDRDNFEIVEYELSQSSLYAGQREKGLLTVVYDKANY
IVGVSEISTSAVNDVNYFVPIIGLRIRKNEWHRNVLPIYPALADKIEGILR"
CDS complement(149028..150182)
/gene="guaB"
/locus_tag="OCPHGMPK_00139"
/EC_number="1.1.1.205"
/inference="ab initio prediction:Prodigal:2.6"
/inference="similar to AA sequence:UniProtKB:Q5X9A3"
/codon_start=1
/transl_table=11
/product="Inosine-5'-monophosphate dehydrogenase"

/translation="MSLWETKFAKKGLTFDDVLLIPAESHVLPNEVKLDTKLAPNLQL

HIPLISAGMDTVTEGDMAIAMAENGGLGVIHKNLSIEAQVEEVKKAKTKAVDPNLSHP

AVDDQGRLLAAAAVGVTSDTFERAEALLEAGADAIVIDTAHGHSAGVLRKIKEIRDHF

PKATLIAGNVATGEGTAALFDAGVDVVKVGIGPGSICTTRIVAGVGVPQITAIYDAAN

VAQKYGKKIIADGGIKYSGDVVKALAAGGNAVMLGSMFSGTTEAPGTIFTNEGKKFKS

YRGMGSVGAMSQQHGSSDRYFQGGVNEANKLVPEGVEALVPYKGDVSNIIYQIDGGLR
AGMGYVGAGTIEELIENSQFVQITNAGLRESHPHDVQMAKEAPNYGGSDL"
CDS complement(150256..150831)
/locus_tag="OCPHGMPK_00140"
/inference="ab initio prediction:Prodigal:2.6"
/codon_start=1
/transl_table=11
/product="hypothetical protein"

/translation="MKLLLTGDSIIARSENHSIPELNFQLQKILSCDIYNTAISGINS

GGLALSLPSLVFNQPKCNYLIIILVGTNDLATHKQVPLRQFEKNLDLIASSIIWLYYPE

KVIFITPPAVDENKQRVRSNRLVMEYSNIVKKVAGKYKFSVIDLASKMQENINFPEIF
NGKKNDGLHFGVNGYKLLANLIVKKINQISN"
CDS complement(150907..151920)

/locus_tag="OCPHGMPK_00141"
/EC_number="1.1.1.28"
/inference="ab initio prediction:Prodigal:2.6"
/inference="similar to AA sequence:UniProtKB:P30901"
/codon_start=1
/transl_table=11
/product="D-lactate dehydrogenase"

/translation="MTKIFAYAIRKDEEPFLNEWKDAHKDVEVEYTDKLLTPETAKLA

KGADGVVVYQQLDYTPETLQALADAGVTKMSLRNVGVDNIDMDKAKELGFEITNVPVY

SPDAIAEHAAIQAARVLRQDKRMDEKMAKRDLRWAPTIGREVRDQVVGVVGTGHIGQV

FMRIMEGFGAKVIAYDIFKNPELEKKGYYVDSLDDLYKQADVISLHVPDVPANVHMIN

DKSIAEMKDGVVIVNCSRGPLVDTDAVIRGLDSGKIFGFVMDTYEGEVGVFNKDWEGK

EFPDARLADLIDRPNVLVTPHTAFYTTHAVRNMVVKAFDNNLKLINGEKPDSPVALDK
NKF"
CDS 152179..154800
/locus_tag="OCPHGMPK_00142"
/inference="ab initio prediction:Prodigal:2.6"
/codon_start=1
/transl_table=11
/product="hypothetical protein"

/translation="MSVVVKYIDLDNNSAELASSEELNGKAGERINYHTAAEVQKLTA

AGYVLINNSFDPNNEVHFFDETSQEYKLTFKHSQEEVTAKNLKYDCQLNDVQLKGKQV

VHYTGLEKDIPDNVVEVTFNRKIIYDKVTKQKVSLSAWQPEKYYLPLVATPAVLDYTP

DKAVIGGEAVTVQKPKHEYVVTYLPNKKNARRQKAEIKFIDIDDSNQELASSGELKGK

PGKEISYSTTEILKELTDKGYEVVTNDFDSNDQKPVFGNSRDYIQTFIIALRHKKQAV

NSDHLSSEIDHRLYEKEVHRRITFSGLNGQQLDDIVQTAVLKRSLTMDMVTKKIIPGE

YTSQWQSTETYPSVAVPVVAGYHTKTKEVVACPVIEKDISEEVKYYANGYLIPVDANH

NKLSEIDKKQLITNSIDPTKAIFPKLELENVVLKPIKEVKIEDPGRDYEVPYLLVHKY

IAVDEKHPKKEVSPAYYRRIVTARVHYQGAGDLTPTDAEQTVTWTRTVTYDEVSREII

DNGMYTTKWLADKDIFEATPTPVIKGFCADIGLIGEHPVTETDLMATVTYAPVGKMIP

IDEHGNEIKNAMRSPYINDPYDPVRVLFTEEVPEVQGYAPVNNTISVNDPFSDTKVTY

TLKPRYIPVNSEHPYRPIKPTMYSVPVKETVKYQGAGEQTPTARLQAARWTRTLTADE

NTSELIDNGKYTTAWKVDKNHYAAVKTPVIDGYHADKNIIEAKEVKKADLNFSVVYQA

NGRIVPVDAKDNSLVGVEQPSYVTDPEDATKVLPKQGVPRILNYVPEQEVITVGDAGE

DTRVKYYTFSEWSELKAKKKAEQKHKNLKTKKPIENKDEQEKKEKVEEKPLKSAEEEK
KSEKKDTGLKAMFSWLK"

CDS 155197..166254
/locus_tag="OCPHGMPK_00143"
/inference="ab initio prediction:Prodigal:2.6"
/codon_start=1
/transl_table=11
/product="hypothetical protein"

/translation="MLSKNNYQERLRKMDDKQERFSIRKFSVGAASVLVGTAILSMQN

VQTVRADATTDTEKGTTDVTSKNDEQNKQKAYNQVVSEDQNKSSKTTGTTMEGQDSKV

ASFSASKNEGTFAETSSEDKAASTTDATESKTSDTTKATEDNKVDAVADKSTEDKANT

DATQESSDSKSTENKTTDAQKVESKVATTKATTDTANSVKSASTTSTDKTTSVNTTTF

NTNQSSTAALFSASALSESKALAATPRASSATTNTQAKNNNYKLVTSASALQQAINSG

VAGVNIDRSIDASNVDLAITNTFAIVGINDAAVLNLGQKSLNNSGNLTLQDITINGAV

SGNGTVNIKGNVTSNVNENNSLIKGATVDEANAALNDQTGTGKIGTQGTSWASGSSNQ

NGWAVKGWNYANFSGSKVNVAVDANLTINRSAIGDGIHLANNGTVNVADGGQLTINMN

TNNDLNTTARYHNAGIFAVGNGNLTTGYKSVVTLNTSIGQGIAMTGMRPYVTDTDVFG

GYSARDRGDGSGQINLGQYSTLNFTGRDGVILGNNSNFNVGDSANVHFENKGRGVALD

LAANSNINIDDHAVTYFHSVGKTTTNALGNTVGASGSFSGYNYIGVNEGGNITVGQFA

TFRVILEGRGNNNYDDVVSLDSQNSNTNAAFTSKTGAIVDIRDDNTNFYAELISFPLG

TSNTRIDIHDPLMLNLQRYSSGGPTTGWMPIGGDMINTTSNQYTANLIYMSGSKGVFS

VDGTDYVVYQKIKSDGSKQIWLNVNGVNIPMSGFQTKDIWNNQANPDVSIKGTDLTSG

IRANQVHNYDGTPLTGNDAPYYGISTQRASQQIWFPHKTQMEVVGSHTNTIKYVYEDG

TPVLDENGNQIVKTQNLNLTRKLTLDITDDKIEEIQKYALTHNADQTLEYIKNAQSVS

EDSGWVYTDAQGNTVTDPYATVVSPVEDGYTASIKSSNVPGITEGADGTSVTAKLQYK

EDLVQNGELSNNYKQNGLSAILPDNYETVVVYGMPQEVTNTLKFYDDTTKSYISTVTD

QTVTGKENDDVNFKDGASIVKSLEDQGYKFVNVTDGTPDDTNATVLSGDTFSDVDFGK

FGKDGKTFVVHLTHKVVPVTPDTPNVPSNSKVSKDDLTKTATRTIHYVENDQNGAELK

ESTVQTVNYTGTAYVDVVTGQMVNAKADGEDAQGNTTYVVDTDNKKQPLIAWTTDNDG

KFAQVTPDASIKKGDDTWTTGVKSVDEKNAPDVSTITGKTTNEDVYVPYTLSQKTYTG

TKETTSVTRVISYLDNETKQPVSDAVKQTATLSRTRIKDEKDNVIGYGSVSEDGHSYT

LNNDWTIDSNGWVAQVSPDETAKGYKKTPHFEDGKDASTVAADAPSITDPQDVTVNVF

YDHDTTPVTPDKPGHGLTHDDLNKDVTRTINYVDTTGAAVNGAPDGKSTYTQTAHFTR

TAIVDKVNDKLLGYEINGDGSVDITPDAGDFAWKSTDANLPAVTSKTPSEVGYDSVDT

PVVQATTVAYNSEPINVTVTYSKNAQQGSFQIHYIDEDNNNAILHQDTVSGKIGDSVT

YSTADQIKLWESKGYVLDHDGYTTQTTINEDNNGKTYIVSFKHGRKNGTTETIVPTET

IHFQYADGTKAADDVYGNAGDFKFTRTPIIDTVTGQVVDPGTWNKESYTFDDGQKNVK

VINGYVADKVTYGNRTATPNDLNVEDTITYRKISNIIPVDENGNQIPGTTPVDYKNDP

SDPTKVTPDEESPKVPSGWTISPDQPEGVTPNTTTNTAKVTPVDPTEPTKVVYTRDEE

PEDLQYATVKVIYHDDTTGQDLESWSSDTNGNKKEVGTDTGYTQADINRVVQGYEAKG

YYYVTTDGTLPTTIPADGATIVVHLAHNQIPVGPDTPDKHGVDPDQVKKVYTSTLYYQ

DSEGKTLSPDQQQTSTWTRTVTVDAVTNQIVNGGKYDTNWTLQDANDQYSNFTVPVVE

GYVARKITNNGATVTTVVAGQTKVQQNLEDTVVYDKVGKLVPVGPDGKTPIPDAPTPS

YPNDPTDPTKVIPNEPVPDVPGYTPVDPTPITPEDPTKDTPIPYTKDPVKAGLTVQYI

DQDNNNSVIKSDAVNGNIGGKVDYSTASSITDFENKGYVLVTDGFTGQAGDEFTTENN

GQVYKVVFKHGTRPVTPENPADPNKPVDPDHPDTPTPSNPNLSKTDLQKTITRTVEYK

YADGTQAHEPVKQELTFTGKGTIDLVTGNLVTVDKDGNITSQNGKITWNHDSQEFEAV

PAIDHDDYYISSINQSNSTASVDGQTGAVGTETVTPNSQNGNIVITLTRNPDVPVAAQ

GSINYIDDTTGQTIESANFSGNVGQKINYTTAGSIKNWEAKGYNLVSNNFKDGEEVFT

DGKNAFEVHLVHATTPVTPENPGKPGEPVNPTNPDDPHKYPDNYVPQELAKTVTRDVT

YVYADGSQAEAPVHQEVKFTGSGYLDLVTGEYVTVDNNGRITGKGQINWTPESANFDA

TKSIDTSKYQIVGIKENNTTANVDQTTGVVAGETVTQNGNNSSIVITLANKPAPVVEK

GSITVRVHDLTDNVDLPQYGKESGEQEVGTSFTYDKSAVITELINKGYKLVNGGEDVP

SEVAKGAKTITILVEHDTVPVTPENPSKPGEPINPNDPDGPKWPEGTDENSVKRTGTQ

TIHYEGAGDKTPSDDVQTFDFTKKMLVDKVTGKIIDSGEWNVTSHTFGYKDTPVIDGY

HADKRNAGGSVVTPDDLNKKVVVTYKPNGKIIPTDPSGNPIPNVPTPTYPTDPTDPTK

VVPDEPVPDIPGMTPSTPTVTPEDPGKDTPVPYTPVVPAKDQVAQVIYRDVQDGTNKQ

LATSGDLTGKSGSEINYSTADQIKELISQGYVLKNDGFPAGVVFDNDDSKNQVFYVDF

IHGQVPVNPDNPHEGIDPSQYEKTVKEKVHYVGAGDKTPADNVQNSKWTRTLTVDTVT

GKVVENGQYTTDWSIAKGEKTVYDQVNTPVIDGYHADKREVPATAVTQDDIEVTVTYK

PNGKIIPTDPSGNPIPNVPTLTYPTDPTDPTKVVPNEPVPDIPGMTPSTPTVTPEDPG

KDTSVPYNPIVPAKDQAAIVSYVDADNGNAEITNSGNLTGKAGDKIDYSTKATIASLE

NKGYVLVNDGFPADATFDNDDNTTQRFVVVLKHGTVPVTPENPGKSGEPINPNDPDGP

KWPEGTDENSVKRTGTQTIHYVGAGDKTPSDDVQTFDFTRNMVVDKVTGKVIDGGSWN

VTSHTFGYKDTPVIDGYHADKRNAGGTVVTPDDLNKIVTVNYSQNGKIIPTDPSGTPI

SNVPTPTYPTDPTDPTKVVPDQPVPKVPDMTPSTPTVTPEDPGKDTPVPYNPVKTLDK

VTTVEGKQIVHFVDGDNGNTPLRDPNTQTHEFKITNGVPDESSHTFTLVDVPVIPGYV

AEVKSAGGKTVTPDTPLAEVTVVYHKVGKIVPVDPNGTPIPNVPTPSYTNDPTDPTKV

VPDEPVPAITGKTPDKTSVTPVDPTKDTPVVYKDNEVPATPNSQKAVVNFIDVNTGKL

IKTSGILSGRPGEDINKLYSSAEVIKQLEEAGYEVVYNAFDGDGVTKYFDDDDNTTQQ

FTVALKLKEKAKTPDPVVPAPETPAKEPEAPAEKVSRPEQSVKQNVSVPTPQKPVEKK
TNNKKEVLPQTGADHNEAASILGAVAAAIGMTSLIGAKRRKKDDK"

CDS 166422..169376
/locus_tag="OCPHGMPK_00144"
/inference="ab initio prediction:Prodigal:2.6"
/codon_start=1
/transl_table=11
/product="hypothetical protein"

/translation="MNSLSEKAASVVIKYTDLDNNLAELSNSGSLTGNIGEIINYSTT

DEIKELTKQGYVLVNNPFDNKEKAPVFSGDQDSYMITFKHGRERVTADDLKYGCKLED

LQVKGTQTVHYVGAGSRTPRDEVSTITFNQILVYDQVTGKKIGSKGWEKVEQSFPVVA

APSILGYIPDQVLVGGKAVTADNPNREYTITYQVNEHISNKEQKAEVKYLDIDSNNEE

IVESELLTGKPNTKINYSTIDQLKKLGEKGYEVVSNGFDANGDVQFFDTSDEYVQTFI

VTLKHKQVLVNAENPLDGIDETEYHKTSKRVISYAGAEDKTPEEVVQLVNWNRNLTVD

AATKRVIADGKYTTDWKPEQESYSAISVPVVSGYHTRIKEIPEEKVRLANITEKIRYV

KNGYVIPIDKNGQKIDSLPKLHFVSDKDDPTLVSLPENSLEDEKYEPEEIDLTGIDPA

KDFEAKYLLKHKYVTINKDNSHFDVNPGSYRRTATAIVRYEGAGDKNPKDSIQTVQWN

RNITYDEVTKEILEDGKYTTDWKPDKEYFEAVDTPVISGFTADIGVVAKHDVTQSDLF

ATVKYQKNGAIIPVDEDGKEIAKAKSIPFLNDLTDPTRVLATEEVPEIKGYRRTEESV

LIKDPAEDIKVIYILKPHYVLVDSEHPYRAVKPYNYSIPVKETIHYVGANEETPADRV

QGARWRRSLTVNDNNGKVIEDGKYTTDWSVDKKEYSAAVTPVVDGYHADQYQVKAREI

DKEDIDVEVKYQRNGQIVPVNSKGEKIEYADCPTYITDPTDATKVLMEQPVPRLLNYM

AQDSSIVVKDPSRDTKVTYYTFTEIKELSSAQNLKTEIQSIDGKNESSNVVSLPVSGK

RRKAIVTFVDLNNNAIQIASSGVLSGNVGDKITDLYNTSKQVEELKKKGYEVVYNGFD

PKGASKYFEEDQRRVATFTVAVKKVKQVKPKKEMQAEKTSKIEEKPKAANSDKKKKQD
HKVLKHIFPWMK"

```
     CDS             169496..170929
                     /gene="gadC"
                     /locus_tag="OCPHGMPK_00145"
                     /inference="ab initio prediction:Prodigal:2.6"
                     /inference="similar to AA sequence:UniProtKB:O30417"
                     /codon_start=1
                     /transl_table=11
                     /product="Glutamate/gamma-aminobutyrate antiporter"
```

/translation="MSDKSESLTKLEEDEKYNRLTALKLFAMTSSMVISVNELAPFGK

TGPTAVFYLLLAGLIWFVPITQMAGEMASVDGWDKGGIFTWVKGSLGDRAGWTAMFYQ

WIHITVGMNTMMYVIIGALSITFNTPWFNTTPSIRFLLMMVIIWSITIVEMVGVKKIG

HIAEWLFALGIALPVILLIVTFFIYLIQGRPLYMHLNWDNIIPHHLTGTTLVAFVPFV

LAFCGGEASAPHAKYLENPKTYSTVMLALAITAISFDLLGSTAIGMSVPKDQIQNSTG

FVYTFGKILDSIGLPGDLIKKFIGVLLAAGIIGELGNWLAGPSQGMFEAAKEGYMPKF

FAKSTNRDVPMRLIVLQTLIVTASAVLITFTSGKNSDFAFNVSLAATTAQYLMVYILM

LISYIVLKIKHGNLKRSYYMTKNNALGITIAIIALVITVVAFFISFIPAQGTPTYLRG
VYVWTMIGLCMIVTILPLVIYHYHRHF"

```
     CDS             171114..172040
                     /gene="cynR"
                     /locus_tag="OCPHGMPK_00146"
                     /inference="ab initio prediction:Prodigal:2.6"
                     /inference="similar to AA sequence:UniProtKB:P27111"
                     /codon_start=1
                     /transl_table=11
                     /product="HTH-type transcriptional regulator CynR"
```

/translation="MNNPEEIQHFIDVLIKEDSFVKAAKKLYISQPYLTQLIKRIESH

LGTPIINRDKKTYSLTQAGLLYYQYLENVSYDKQQLNKKIAKYTHPNKEIIKIGILES

LGTYLLPEILPDFLKQNPQVEIQLFENFPRENEKNLLSGNIDCYIGQTPEAIDSSLDI

VSNGGEKYYIVISPASPYYQAGKFILDPHELDLKELLQEPFVLSVPGSAIRHQVNGVF

QRFHLEPKVILESKSIITATSLAIHGMGLTISTASIIKRIGETPINLFPISHDLINVV
FFIASRREKTKSKALKNLIREFKNKNLQAIIR"
CDS 172168..174018
/gene="urdA_1"
/locus_tag="OCPHGMPK_00147"
/EC_number="1.3.99.33"
/inference="ab initio prediction:Prodigal:2.6"
/inference="similar to AA sequence:UniProtKB:Q8CVD0"
/codon_start=1
/transl_table=11
/product="Urocanate reductase"

/translation="MKSGKYEVRAKGHGASFMPMEVTLSEDKIEDIKVDAKGETKGVA

DEVFRRLPEEIVKNQTLNVDTVSGATISSHGVIDGVAAAISEAGGDPDEWKKRAKPAE

QREKDETYTTDVAIVGAGGAGLAAAARSIQHNKKVIVLEKFPQIGGNTSRAGGPMNAA

EPDWQKQFKALAGEKETLEELAATPVEKIDPEYQTDFKELQKQIKKYVASGANYLFDS

KLLHEIQTYLGGKRTDLKGNEIHGNYALVKELVDNALDSVHWLADLGVDFDRSQVTMP

VGALWRRGHKPVEPMGYAFIHVLGDWVKEHGGTILTDTRAKHLIIEDGKVCGVIAKRP

DGSKITIHAKAVILTAGGFGANTPMVQKYNTYWKHIDDNIATTNSPAITGDGIGLGKE

AGADLVGMGFIQMMPVSDPKTGELFTGLQTPPENYIMVNQKGKRFVNEFAERDVLTKA

AIDNGGLFYLIADDKIKDTAYNTTQESIDAQVKAGTLFRADSLEELAKQIGMDADTLV

DTIKKYNSYVDAGKDPDFEKSAFNLKCEVAPFYATPRKPAIHHTMGGLKIDTGAHVLD
KTGKQISGLYAAGEVAGGIHAGNRLGGNSLADIFTFGRIAANSAIDELEK"
CDS 174110..175474
/gene="brnQ_1"
/locus_tag="OCPHGMPK_00148"
/inference="ab initio prediction:Prodigal:2.6"
/inference="similar to AA sequence:UniProtKB:P0AD99"
/codon_start=1
/transl_table=11
/product="Branched-chain amino acid transport system
2
carrier protein"

/translation="MREAQEKKLTTKQYLIVASMIFALFFGAGNLIFPLHLGQLAGKN

WGPAAIGFSITGVVLPLLSLLAVAITRSNGVYQIGLPVGKIFALSFMTLMQLAIGPLF

AAPRNATVSYTVGIAPLLPKQFHGIGLIVFTIIFFAIVYVIAYNESDILSSLGKVLNP

IFLILLFIVFVIAFARPLGNPDMATTTKEYMNGAIVKGFLEGYNTMDALAGLAFGVTV

VTAIKELVNNDEKKTAKMTAKSGLIAVIAIGVIYTLLIVIGAMSLGHFKIASDGGILF

SEIVKYYAGIFGQALLAVLIFLACLTTAVGVLAAFALDFSAHYSKISYKGWLTIGCLG

SLATANLGLEKIIHWSLPVLMFLYPLAIVLIILSVCSPLFKDDMVVYKITMLLTLVPA

IFDLITNLPAPISGTQFYKVVSQIRLQYLPLANIGLSWVVPTILGLVISVLIHFWRRK
ANKI"
CDS 175540..176253
/gene="menG"
/locus_tag="OCPHGMPK_00149"
/EC_number="2.1.1.163"
/inference="ab initio prediction:Prodigal:2.6"
/inference="similar to AA sequence:UniProtKB:P67062"
/codon_start=1
/transl_table=11
/product="Demethylmenaquinone methyltransferase"

/translation="MSLTNKVPEKDVHDLFTRVALHYDQMNNLISLGTQKGWRKKFLK

ELKVAPGEFALDLCCGTGDITIALAKQVGPSGNVIGLDFNQEMLDLAEQKIRQQDLQK

EIQLKQGDAMELPYPDQSFDIVTIGFGLRNVPDANQVLKEIYRVLKPTGKVGVLETSQ

PTNPLVKLGWKSYFKLFPSFAKLLGANVSDYQYLSHTTAKFVSAEHLKAMLIKNGFKN
VHVDKLNLGAGAIHIGIKK"
CDS 176296..177210
/gene="fpaP_2"
/locus_tag="OCPHGMPK_00150"
/EC_number="3.4.11.5"
/inference="ab initio prediction:Prodigal:2.6"
/inference="similar to AA sequence:UniProtKB:O05420"
/codon_start=1
/transl_table=11
/product="Proline iminopeptidase"

/translation="MKTGTKIITLDNGYHLWTNTQGEGDIHLLALHGGPGGNHEYWED

AADQLKKQGLNVQVTMYDQLGSLYSDQPDYSDPEIAQKYLTYEYFLDEVDEVREKLGL

DNFYLIGQSWGGLLVQEYAVKYGKHLKGAIISSMVDEIDEYVDSVNRRRQEVLPQTEI

DFMHECEKNNDYDNQRYQEDVQILNINFVDRKQPSKLYHLKDLGGSAVYNVFQGDNEF

VITGKLKDWHFRDQLKNIKVPTLITFGENETMPISTAKIMQKEIPNSRLVTTPNGGHH
HMVDNPDVYYKHLADFIREVENGNFKGE"
CDS 177345..177947
/locus_tag="OCPHGMPK_00151"
/inference="ab initio prediction:Prodigal:2.6"
/codon_start=1
/transl_table=11

/product="hypothetical protein"

/translation="MKRKISKLLAATALIGTLGISVVACNNNDSTSKTKQSSVKKTVK

DKASNTKNTARRSKIKLSQTEAINKFDKKYSDKKLKEIDLKLDGNKYFYEITGFDKDK

EYEMTINAISGKEVKSSSEKLDLDERLQKGLDLDKVISRDQASEIAEKEVKNSTAKEW
TLKMDQDKVIWDVTVESGSSKHEVEIDAISKKVIKSEKDD"
CDS complement(178008..178958)
/gene="cbh_1"
/locus_tag="OCPHGMPK_00152"
/EC_number="3.5.1.24"
/inference="ab initio prediction:Prodigal:2.6"
/inference="similar to AA sequence:UniProtKB:P54965"
/codon_start=1
/transl_table=11
/product="Choloylglycine hydrolase"

/translation="MCTGLRFTDDQGNLYFGRNLDVGQDYGEGVIITPRNYPLPYKFL

DNTTTKKAVIGMGIVVDGYPSYFDCFNEDGLGIAGLNFPHFAKFSDGPIDGKINLASY

EIMLWVTQNFTKVSDVKEALKNVNLVNEAINSSFAVAPLHWIISDKDEAIIVEVSKQY

GMKVFDDKLGILTNSPDFNWHLTNLGNYTGLDPHDATAQSWNGQKVAPWGVGTGSLGL

PGDSIPADRFVKAAYLNVNYPTVKGEKANVAKFFNILKSVAMIKGSVVNKQGSNEYTV
YTACYSAATKTYYCNFENDFELKTYKLDDKTMNADKLITY"
CDS complement(178976..180331)
/locus_tag="OCPHGMPK_00153"
/inference="ab initio prediction:Prodigal:2.6"
/codon_start=1
/transl_table=11
/product="hypothetical protein"

/translation="MSTDVATKDKVVSKGYKYFMVFLCMLTQAIPYGIAQNIQPLFIH

PLVNTFHFTLASYTLIFTFGAVFASVASPFIGKALEKVNFRLMYLIGIGLSAIAYVIF

GISTKLPGFYIAAIICMIGSTFYSGQGVPWVINHWFPAKGRGAALGIAFCGGSIGNIF

LQPATQAILKHFMTGNTKTGHLTSMAPFFIFAVALLVIGIVIACFIRTPKKDEIVISD

AELAESKKEAELAKAKEFKGWTSKQVLQMKWFWIFSLGFLIIGLGLASLNEDYAAFLD

TKLSLTDVGLIGSMYGVGCLIGNVSGGFLFDKFGTAKSMTYAGCMYVLSILMMVLISF

QPYGAHVSKIAGIAYAIFCGLAVFSYMSGPAFMAKDLFGSRDQGVMLGYVGLAYAIGY

ATGAPLFGIIKGKASFTVAWYFMIAFVAIGFIILVFAVIQIKRSQKKYIIQQETKTTT
E"
CDS complement(180356..181702)
/locus_tag="OCPHGMPK_00154"
/inference="ab initio prediction:Prodigal:2.6"

```
                     /codon_start=1
                     /transl_table=11
                     /product="hypothetical protein"

/translation="MSNDRQKVVSKGYKYFMVFLCMLTQAVPYGIAQLIQPLFVHPLV

NTFHFTLASYTLIFTFGAVVGSLVSPLVGKALQKVNFKILYLIGICLSAGAYVIFEIS

TKLPGFYLAGIICMVGSTFYSGQGVPWIINHWFPFKGRGVALGLAFCGGSIGDIFLQP

ITQEILKHFMTGNTKTGHLTSMAPFFIFAIALLIVGLIITAFIRVPKKDEILASAQEV

EQNRHEAAQKQAHEFQGWSGKQVLHMKWFWIFSIGFLIIGLGLASLNEDYAAFLDTKL

SLTEVGMIGSVFGLAGIIGNISGGYLFDKFGTAKSMAYAGIMLIIAILMMIFISLHPY

GDRVNFYAGMGWAFTSGLSVFSYMSGPAFMSKSLFGAKAQGVNLGYISLAYAVGFAIG

APLFGVIKGATSFTTAWCCTTFFVAIGFILLIFAAIKIKQIQKNIVVSKPNIILDK"
     CDS             complement(181889..182749)
                     /locus_tag="OCPHGMPK_00155"
                     /inference="ab initio prediction:Prodigal:2.6"
                     /codon_start=1
                     /transl_table=11
                     /product="hypothetical protein"

/translation="MIIGENRAKVIENIKAAVKKGKMHAKVELGDPILTKSERIKLVN

DYWEQQKKFSHKLKNIIAQGIINIDTLILMAHTQIKNKHRAQGPKYGAIVTSNHFKQT

DSLPIKKLANKCHKKLYIVIEDSNLKLPGFFGFLMNNINAIPIVQSYQYLGREFPKHL

QQVFDKKGWVLIYPEQEMWYNYRKPRPLQKGAYYYAAKANVPIISCFVEIQDLPEMEK

NSSEFHKTRYILHVLPTIYPIDRLTVDQNAKRMRDIDYLQKKKAYEKAYGKKLSYSFD
                     DDDIAGYVKK"
     CDS             complement(182746..183696)
                     /gene="gspA_1"
                     /locus_tag="OCPHGMPK_00156"
                     /inference="ab initio prediction:Prodigal:2.6"
                     /inference="similar to AA sequence:UniProtKB:P25148"
                     /codon_start=1
                     /transl_table=11
                     /product="General stress protein A"

/translation="MTIPVFYSISDDFTKYAAVSLNSLVKNASPQNDYTVTFLNQDLS

EKHQKQLAAFANDYVHVKFFHIDEKLVKPIQNRKENYLRADFFTMSIFYRLFIPDLFP

QYDKAIYIDSDTVVVDDIAKLYNNDLDNKLFAACTDSSIQYVDKMVKYIKEVLSLDPK

KYINSGMLVLNSKAFRDEHFIDHFMHLLERYHFDCIAPDQDYLNEIGDGRILHLDPRW

DAMPNENTEPISKPGLIHYNLFFKPWHFKDVQYNDYFWKYASSTPFYEELKGELDNYT
```

```
                     DQERAEDRAKLDHMLEKEASTALDPNNWAQVKRKGERVKL"
     CDS             complement(183709..184659)
                     /gene="gspA_2"
                     /locus_tag="OCPHGMPK_00157"
                     /inference="ab initio prediction:Prodigal:2.6"
                     /inference="similar to AA sequence:UniProtKB:P25148"
                     /codon_start=1
                     /transl_table=11
                     /product="General stress protein A"

/translation="MTIPVFYTISDNYTPYAAVSIQSLIDHVDPNKDYTITLLVQNIS

DMHKKDLEDLSTKNVHVNIFHIDDEMVAPIHNSEENYLRAQFFTMSIFYRLFIPNLFP

QYDKAVYLDADTIICTDIAELYNTEIGDNMFASVPDMSIRFIKPLQVYIKECQGIFPP

EKYINNGVILFNMKAFRDKKFVDKFYSLIEKYHFDNIDPDQAYMNEICEDKIYHLPLE

WDAMPNEHMDEIKNPKIVHYNLFFKPWHFADVQYGKYFWDVAKKSPYYEELKAQLASF
                     TDEDRKKARTDLDWMIKKVDEIKDEDVTWAKVKKTTSVKI"
     CDS             complement(184691..185515)
                     /locus_tag="OCPHGMPK_00158"
                     /inference="ab initio prediction:Prodigal:2.6"
                     /codon_start=1
                     /transl_table=11
                     /product="hypothetical protein"

/translation="MNIMFCGDSHAEDGILITTLSLLKNTSTPLHFYILTMHAEGYTA

FNQQAFRLIKKLLKEKDPNNTAELIDCTELFKKEPPRANMETRFTPYAMLRLFADQLP

QIPDRVLYLDDDIIVRKDISDFYNQDLTNIELVGVLDFWGRFFFHNIHTHKPFDYLNS

GVLLLNMSEIKKTQLFAKVREKMSDKKMLLPDQSALNKLATEKKIAPRCYNEQYRLRP

DTKIQHFTTSFRFKPYFHTLTVKPWDIERVHSVLKLHEYDDLLKQYTELRSQLKRA"
     CDS             complement(185518..186297)
                     /locus_tag="OCPHGMPK_00159"
                     /inference="ab initio prediction:Prodigal:2.6"
                     /codon_start=1
                     /transl_table=11
                     /product="hypothetical protein"

/translation="MNTKTSQRNVIYYNSLTDDIVKSKQQDFTLPSNYQIIKRTPLNY

LMRFLATGFAYLFTYGVMHVKVIGRDKLFKYKHQSYFVYGNHTQMVNDAFMPLTLFGW

KDYYAIANQANWGIPIIGKILLPYGGLPVGKNIKQAIHLLKAVNTLTKEDAHIIIYPE

AHVWPYYTDIRPFPATSFNFPVQTNKPSFVMTTTYQKRKFSKRPRITVYIDGPFFPDT
                     SLPKKLQQKKLHDQVFTQLKDRSKKLSNYEYYRYQQRKIGG"
     tRNA            complement(186362..186436)
                     /locus_tag="OCPHGMPK_00160"
                     /product="tRNA-Lys"
```

```
                     /inference="COORDINATES:profile:Aragorn:1.2"
                     /note="tRNA-Lys(ctt)"
     tRNA            complement(186559..186633)
                     /locus_tag="OCPHGMPK_00161"
                     /product="tRNA-Lys"
                     /inference="COORDINATES:profile:Aragorn:1.2"
                     /note="tRNA-Lys(ctt)"
     CDS             186759..187466
                     /gene="walR"
                     /locus_tag="OCPHGMPK_00162"
                     /inference="ab initio prediction:Prodigal:2.6"
                     /inference="similar to AA sequence:UniProtKB:P37478"
                     /codon_start=1
                     /transl_table=11
                     /product="Transcriptional regulatory protein WalR"

/translation="MPKKILVVDDEKPISDIIKFNLTKEGFDVDTAYDGEEAVKKVDE

YDPDLMILDLMLPKKDGLEVAREVRQTHDMPIIMVTAKDTEIDKVLGLEMGADDYVTK

PFSNRELVARVKANLRRRDLTQKATEDDEDKNITISNLVIMPEAYMVEKNGEKIELTH

REFELLHYLAQHMGQVMTREHLLQTVWGYDYFGDVRTVDVTVRRLREKIEDNPSSPTI
                     LVTRRGVGYYVKNPSDE"
     CDS             187513..189363
                     /gene="walK"
                     /locus_tag="OCPHGMPK_00163"
                     /EC_number="2.7.13.3"
                     /inference="ab initio prediction:Prodigal:2.6"
                     /inference="similar to AA sequence:UniProtKB:Q45614"
                     /codon_start=1
                     /transl_table=11
                     /product="Sensor histidine kinase WalK"

/translation="MKKIKSVLNSINFKIAVIFMLLLLATIEVVGASFTRQLEQNSIQ

NFESSIQVPNIITNQISSQLSRANSKKANQQLSQIISNYNLGDISQLMVVDNKGVIRA

VSNVNDQNRIGQRTSNVDIKSVLSNGKQVSKVINDNGNYMVQISPLTSANGTNTPVGA

IYVRASLQGVFNNLRQVSIYFLIASLIAAVLGAIVALVISRAITRPIEEMRKQALRVA

NGDYSGHVRVYAQDELGQLAEAFNTLSVRIERTQEISDSERRRLDNVLTHMTDGVIAT

DRHGNITIINETALDFLGKTEKDVIGKPITNLLGLKDVTIQDLLSTQQELVVRVNDNT

RDEMILHANFSLIQRVTGFVSGLVCVLHDITQQQKNEREQQQFVSNVSHELRTPLTSL

RAYVEALNDGAWKDPNIAPQFLHVIQDETERMIRMINDLLSLSRMDRGVARMDLEWVN

LNDFVNHVLNRFDMMLKSDSDKMHKKKYTIKREFPHQALWVEIDTDKMMQVIDNIMNN

AIKYSPDGGVITVRLLQAQKHVILSISDQGLGIPRKDLNKIFDRFYRVDKARSRKQGG
                     TGLGLAISKEIVEAHHGRIWADSAEGAGSTFYISLPYEAISEEGENWDEV"
```

```
     CDS             189353..190681
                     /locus_tag="OCPHGMPK_00164"
                     /inference="ab initio prediction:Prodigal:2.6"
                     /codon_start=1
                     /transl_table=11
                     /product="hypothetical protein"

/translation="MRFKDKFSRITLRISLIVMVVLSIILSAIIWGSDARFSRIEETS

NQTQTKDLGQRSLRDIYLPTQTFYFKDKQMYQVYDTKNNLPLEFSKLTQSVKPLLPIR

VWSSQSKYEKLLKNPNYVQLTYPDQITISLFLTNVRKADSREFNRFFVPARSSKYIYL

GNDENYTIYRVRLSDVSFDNLVEHIQSAKTQMPVTLQKVHDDYLPFYDKNLSLPTYSY

LTKEESDSYFVYRLLGSNNPTQHSSGESITYSNGVYERLIAAKHTHNYEYIDYQQDQV

PKTISRKLNDSLYFVRKIGLSEPDLRFFDADNNTVIYQNYVEEYPIFLPGKYKMRAQV

KFASNGMTINFNSLDLQIPIPTSGEKKTLIPTNEAMDELYQKGYHQKDIERIVIGYTA
                     KSDNSKNKKLVDLEPTYYVKINKQWKTLDEWLNINNQVVNSGKEGLVDGL"
     CDS             190671..191492
                     /gene="yycI"
                     /locus_tag="OCPHGMPK_00165"
                     /inference="ab initio prediction:Prodigal:2.6"
                     /inference="similar to AA sequence:UniProtKB:Q45612"
                     /codon_start=1
                     /transl_table=11
                     /product="Two-component system WalR/WalK regulatory
                     protein YycI"

/translation="MDFKRIEWIFLVVFIGINIFLGIELWQTPTLLSAGSTPAQTDIK

SETSADQITIPKVNDKQEDGYYLAAKVDNSWTKKAAAQVNQQVETNASENSISVNLSK

PIALSKDSKKALKEIIHFKNNSQNVYEGKDYVYLSELSEGDDYVFNQKTKYGEVFAAT

ARLHIIVKNNQIVSYSQRYVSNLNPVRERQNTISSKAAVNSLYTYSELPNNSKIIWLK

QVYTKLITVRESEIYIPTWVAAIENNNSHTMTLKRVNAFTGTIIQNNIAADDSKE"
     CDS             191499..192296
                     /gene="yycJ"
                     /locus_tag="OCPHGMPK_00166"
                     /EC_number="3.-.-.-"
                     /inference="ab initio prediction:Prodigal:2.6"
                     /inference="similar to AA sequence:UniProtKB:C0SP91"
                     /codon_start=1
                     /transl_table=11
                     /product="Putative metallo-hydrolase YycJ"

/translation="MKVSVLSSGSAGNSTLIETPQHKILMDAGLSGKKTKDLLAQVGV

DIKDIDMVFISHDHTDHSGGLGVLMRRYPKISAYANSGTWNYLIESNKIGKLPVEQIN
```

```
TFESGITKTFGDLDVTNFATSHDAAQPQYYVFTSGGKRFACLTDTGYVSSTVKGEIKD

ADGYLMEFNYDDMMLRNGPYSWSLKQRIMSDVGHLSNDQAADTLLDVVSPKTKHIFLA
                     HRSQHNNTQYLAHETAEDLLVAGDANLPADVKIIDTNPMQAGSLTKI"
     CDS             192383..193603
                     /gene="htrA"
                     /locus_tag="OCPHGMPK_00167"
                     /EC_number="3.4.21.107"
                     /inference="ab initio prediction:Prodigal:2.6"
                     /inference="similar to AA sequence:UniProtKB:Q9LA06"
                     /codon_start=1
                     /transl_table=11
                     /product="Serine protease Do-like HtrA"

/translation="MAENNTKHPTKHNALIKTGIVGVVAGLLGGGVAYAGLSQVNGQN

APQTSIVPTTKVEKSSSKNSSQMTNAFNTVKKSVVSVVNLKRQSSSSSSDPFGIFGSD

SSSSSKKNSKSDLETYSEGSGVIYMKSNGKGYIVTNNHVVSGSDEIQVILSNGKKVTA

KKVGTDSETDLAVLTIDGKYVTQTAQFGSSKNLEPGQQVIAVGSPLGSEYATSVTQGI

ISAKNRTVDVTNSAGQVTNQATVIQTDAAINPGNSGGPLVNMSGQVIGINSMKLSSSS

DGTAVEGMGFAIPSDEVVSIINQLVKNGKITRPKLGIRIVSVDELTEYGRKKLGLPDS

VKSGVYVASVTKNGSADKAGIKSHDVITKIDGKAVDSVVSLHTALYTHKVGDTVTLQV
                     VRDGKSQDIKVTLS"
     CDS             194117..194596
                     /gene="rlmH"
                     /locus_tag="OCPHGMPK_00168"
                     /EC_number="2.1.1.177"
                     /inference="ab initio prediction:Prodigal:2.6"
                     /inference="similar to AA sequence:UniProtKB:Q45601"
                     /codon_start=1
                     /transl_table=11
                     /product="Ribosomal RNA large subunit
methyltransferase H"

/translation="MNIKIVCVGKLKEKYFKDGIAEYVKRMGRFAKIKIVQVPDEKAP

EKLSPAEMEQVKEIEGKRILDKIKDKEYVYVTAIKGKERTSEDFAKELADLTTYGHSD

ITFVIGGSLGTSDAVNKRADDLISFGKFTMPHQLMRLVLVEQIYRAFMINSGSPYHK"
     CDS             complement(194597..195250)
                     /locus_tag="OCPHGMPK_00169"
                     /inference="ab initio prediction:Prodigal:2.6"
                     /codon_start=1
                     /transl_table=11
                     /product="hypothetical protein"

/translation="MNPGLKLFLILIISLEISLIPNLIANIIIIVASLSYLTFKHLSL
```

KKLLLIFLIPLFAALVVFITIYYFTPGHSLYQASVLFTRIYAYIFLGATFTETTSVLA

LARSLEQNFKLPSKFAYGTLAAFNVIPKIHAEVNRIRLVGDMRHYHLSFYSPILYFKA

ILAAISWSNSLAEGMISHGYREDKARSIIVPITLTKKDWLIFSSILILLQPILFFIK"

```
     CDS             complement(195265..196617)
                     /gene="ykoD_1"
                     /locus_tag="OCPHGMPK_00170"
                     /EC_number="3.6.3.-"
                     /inference="ab initio prediction:Prodigal:2.6"
                     /inference="similar to AA sequence:UniProtKB:O34362"
                     /codon_start=1
                     /transl_table=11
                     /product="Putative HMP/thiamine import ATP-binding
protein
                     YkoD"
```

/translation="MSSKQIKLNQLSFQYQNQPIFKNLNLIINQGDFVLLTGPTGSGK

STLLKLISGLDPTFNGKITAGSLKQPFTNWGMVFQDPSRQFTMATPREELIFALENKL

VDRTSALKRINYASTKTNISSLLDQPFLQLSGGEKQRVALAVLIAMKSDLFLLDEPFA

NCDSHNRQFLLTCLATLHKEGKTILISDHNFSGYEKLASMVLAIKDKNIKTITLPDNT

LKNQALSFSLPDLKQNPVYFLKSFSLSFPKKNLISPTNLKIYPGRATLLTGENGSGKT

SLFKALTKIIPYQGQLIFESKDIKKWHRRPYLAKVGQIFQNPDDQFLNVTVKEELDFS

LKYNRNLSLNKIKLEHLLTKLNLQNLDEQVVYSLSGGQKRKLQILVMLMAYPEVLLLD

EPFSGLDQESVSAVIYLLKKYFLDSNHTLLVISHQLEQVQHLCDYHLTIKDQKLFYTK

```
                     "
     CDS             complement(196598..197155)
                     /gene="ykoE"
                     /locus_tag="OCPHGMPK_00171"
                     /inference="ab initio prediction:Prodigal:2.6"
                     /inference="similar to AA sequence:UniProtKB:O34738"
                     /codon_start=1
                     /transl_table=11
                     /product="Putative HMP/thiamine permease protein
YkoE"
```

/translation="MKKLHVRDIILIALIAIIFGLIYLASDGLYNVLTALLTPVGYGM

LANDIMMGIWCMAGPLAGFLVRLPGAAFLGEFLGSCVELLAGEQWGAVNLISGAVQGL

GCELGFTFTSYHRYNWFTLFLSATTTTIVTYLYDFIKNGYSYFSTFNMIFYFVVRWLS

```
                     MLLFTCVLVKLIINLLVKAHVVQTN"
     CDS             197394..198323
                     /gene="btr_1"
                     /locus_tag="OCPHGMPK_00172"
                     /inference="ab initio prediction:Prodigal:2.6"
```

```
                     /inference="similar to AA sequence:UniProtKB:P40408"
                     /codon_start=1
                     /transl_table=11
                     /product="HTH-type transcriptional activator Btr"

/translation="MATATLKSYLSSLMQNTQGTEIEIRTKNLLLPYSLYLEKVHYDF

TEYQSNTSQLIYCLAGKCEVEINNKTFYLKAGNILLIEKYTDYTIKVSDKNAIIVKFK

LDNDFSWRKQLEQVDANTPNEQRLSTLFLEKLNQDRAFLFTTTSVMWGSQNLKGIIQD

YLNNVAFNGTIGLELLKIIILRNLREQNFKSNEVKESTFKDETLDQYIDQHYNDISLA

QAAKYFGFNRNYFSTMVKEKTGKSFVEHVDERRMKEARRLLAKPNISLKEIIETIGYS
                     SKSFFYKKFKHYYGMTPAEMRKRLFREAHINLK"
     CDS             complement(198361..199251)
                     /gene="htpX_1"
                     /locus_tag="OCPHGMPK_00173"
                     /EC_number="3.4.24.-"
                     /inference="ab initio prediction:Prodigal:2.6"
                     /inference="similar to AA sequence:UniProtKB:O30795"
                     /codon_start=1
                     /transl_table=11
                     /product="Protease HtpX"

/translation="MLYQQIARNKRKTAFLLVIFVIILALVGGGLGYLINGEPLSGIV

IALIGSLIYLFIVLQNPGNLVMSMNHGREIHEEDDPELWHIVEDMALAGQVPMPRVFI

INDESPNAFATGRDPKHSFVAVTTGLRKRLNRSELEGVLGHEISHIRNYDILVSTIGV

ALAAVISFISSFASRIWWWGGNSDRDDDDSSPLEIIFKIVAIVFTLILGPLAAALAQM

ALSRNREYLADASSVELTRNPQGLISALEKISNSEPMKDPDPSSAGLYIENPLHKRGL
                     SSLFDTHPPTADRIKRLENM"
     CDS             complement(199260..199817)
                     /locus_tag="OCPHGMPK_00174"
                     /inference="ab initio prediction:Prodigal:2.6"
                     /codon_start=1
                     /transl_table=11
                     /product="hypothetical protein"

/translation="MTLTWIIIIILILLVAVYIGIYNGLQKAKVHADEAWSQIDVQLK

RRNDLIPNLVETVKGYAKHESGTLEKVVALRNQLVNIPTSDHEEAIKLSNQITDSLKS

IFALAENYPDLKANQNFMSLQEELTNTENKIAYSRQLYNSTVAMFNEKLLTFPSNLVA
                     KIHGFKNMDYLQTPTEEKAVPKVKF"
     CDS             complement(199933..201039)
                     /gene="ybiR"
                     /locus_tag="OCPHGMPK_00175"
                     /inference="ab initio prediction:Prodigal:2.6"
                     /inference="similar to AA sequence:UniProtKB:P75788"
                     /codon_start=1
```

/transl_table=11
/product="Inner membrane protein YbiR"

/translation="MTVIKNIAKDRILQITVVITIISLFFARPRIEDINFHTLYSILA

MLTIIQIFSYLHVLDVLAYKLTASARSTRRLTAIFTILSIISAMFLTNDITVLTLIPL

YLTIARRHHLPEILPVTLIGMGANIGAAFTPWGNPHNIFVVNRYNVSPIKFFSWSIPL

LLVSLIIVLVFIFFVKDKPIPTVPLEDIRISIRPMLLTIAVSIFFFFGVFNIVPAYVP

AIVAVILALIINPSIMLHVDYALLLTFTCFFIFISDIQQIPFIVTLISKTMFSEHSVF

LTSIISSQFISNVPSTILIGKFTNFAEALFLGSNIGGFGSIVGSMANMLVFKSFVENG
TVSKRKFFWVFSVLEFAGLIILTILGWIVLDFVI"
CDS 201216..201626
/locus_tag="OCPHGMPK_00176"
/inference="ab initio prediction:Prodigal:2.6"
/codon_start=1
/transl_table=11
/product="hypothetical protein"

/translation="MVDSKNLELKIKDSAKDVLEKKIKPGQVVLLALNDGSNGYSKLG

GTCTIGANFQLVLLDHPDPEFSIEVKNNMGLDMYTSDKELAFLDDGLVLNARNATLSL
SSNEGIIDGGVTISEFKGEKLSADEMKKLGGKIC"
CDS 201705..201845
/locus_tag="OCPHGMPK_00177"
/inference="ab initio prediction:Prodigal:2.6"
/codon_start=1
/transl_table=11
/product="hypothetical protein"

/translation="MRKGENYNTGVTPNLRPKNKKNSKARVKRAAEVVAFLNKAAKDE
NK"
CDS 202018..202878
/locus_tag="OCPHGMPK_00178"
/inference="ab initio prediction:Prodigal:2.6"
/codon_start=1
/transl_table=11
/product="hypothetical protein"

/translation="MNNISRKGVEVKAIFALVLPSIFLGLASNFSRNIFLGVMLLLIL

GAVIGLSAPTIVSTWLILILTVLGISALTLGYVAIDFYGKALLLVSFPLETYLTSQIK

ECIFRWQIFKKNQSSAYRYLKHYDQNVKLQTTYNAEKLYKKIKKILTEKNYLPLWCDF

TIIDWEHDQQFAQFNLEDHDEILRQIATVLKKSRLVDENLYYLGNGQFLIISNTIAPG

TLMVLNDELKAELKGLQYKEYQPAFKMATQHITEKDLLEYPDFKAILKRLKRKLETDL
VVEYLKGVEV"
CDS 202875..203021
/locus_tag="OCPHGMPK_00179"

```
                     /inference="ab initio prediction:Prodigal:2.6"
                     /codon_start=1
                     /transl_table=11
                     /product="hypothetical protein"

/translation="MSNWFFFLVILITLTFLVAFFAMFRNLWTIYHQTEHTVRHLGRK
                     HNVK"
     CDS             203011..204318
                     /locus_tag="OCPHGMPK_00180"
                     /EC_number="2.4.1.336"
                     /inference="ab initio prediction:Prodigal:2.6"
                     /inference="similar to AA sequence:UniProtKB:Q8YMK0"
                     /codon_start=1
                     /transl_table=11
                     /product="Beta-monoglucosyldiacylglycerol synthase"

/translation="MLNDFLAVSTLVSIWFSLLASLVTLYGATCFWLKHSKKVISITP

LKRYPTITIVVPAHNEEVVIANTTKGILNLNYPASKIELLLYADNCQDKTAAEMRKAT

DLPQYRYRNVQIIERHGSGGKAGVLNDALKIAQGEYICVYDADALPEQNALYFLVKKV

LENPQRYMATFGRNKTRNAKQNFLTKCINQEVIVSQRLQHVGVWNLFKIGRIPGTNFI

INTEYVKKIGGWQSGALTEDTEISFRIMEDGYLIALAYNLEAFEQEPEHLRDYYYQRL

RWAKGNYQVVMNNFKHLFDKSNWRVKLETFYLTCTFFWFNLAVILSDIIFFVDLGCII

ARFFNPEIPIIFAMNSNVLLMQLLLINWLLMILLYVSQINLALATQYGQATSDQIWLA
                     LVSYFTYSQLFIAISLQAVCSVIGDKLFRRDGTKWVKTKRFAD"
     CDS             204328..205446
                     /locus_tag="OCPHGMPK_00181"
                     /EC_number="3.2.1.4"
                     /inference="ab initio prediction:Prodigal:2.6"
                     /inference="similar to AA sequence:UniProtKB:P29019"
                     /codon_start=1
                     /transl_table=11
                     /product="Endoglucanase"

/translation="MKVKNLILILGTIIAYLGIIGYVRMSNDRTLEERYYHEWREDYI

KNKNQNEQYVNAAGEKGPSFALSEAQGYGMLLAAKAGEKHLGSRIDFQKLDNYYLAHR

LTNSNLMSWRQKDRKNIWRDNPVSASDGDIMIAQALLHANRVWPGHGYKSQAVNLIND

IKRFEINQKAKMVTVGNWANKDSRFYNVLRTSDVMPKAFEEFYQATGDREWLTIKSKM

LNYLQKLSKEHDTGLVPDFAWVSNNYVKPAKANEVATNDDGHYSANACRVPMLLAESN

DKNAAQVVKKMLKFFKKKGTSAGFTLKGKKLHYYQSASFSAPIFVAAHKYRNQGYDTL
                     VEQEKYIFLRSLSKDNYYDATLTTLAALNTNELTGLEK"
     CDS             205561..206205
                     /gene="yfbR"
                     /locus_tag="OCPHGMPK_00182"
```

/EC_number="3.1.3.89"
/inference="ab initio prediction:Prodigal:2.6"
/inference="similar to AA sequence:UniProtKB:P76491"
/codon_start=1
/transl_table=11
/product="5'-deoxynucleotidase YfbR"

/translation="MGLNAYIQGFNSLESIDRAPGYFKYQHHSVADHSFRTAELAQMM

GDIEEVVGKQKINWKALYEKSLNHDYTERFIGDIKTPVKYATPQLRKMIGDVEETMTA

KFIKDEIPKEFQKIYTKRLSEGKDDTLEGKILSICDKLDLLYEAYGEIELGNPNPVFM

QMFKESLETIKKFDDLTCVQYFIKKILPDLFKGDFAGKDKMQRIAFSILLMGEE"
CDS 206207..207136
/gene="cas9"
/locus_tag="OCPHGMPK_00183"
/EC_number="3.1.-.-"
/inference="ab initio prediction:Prodigal:2.6"
/inference="protein motif:HAMAP:MF_01480"
/codon_start=1
/transl_table=11
/product="CRISPR-associated endonuclease Cas9"

/translation="MKLQCASCGLRLDSLHFKQEGLMNPKLTSICDICLTRNLDAEDY

ENLVIENISQVMLYGFVGKKNTGNANPEALNQYLVGSDHRRQYESFIQDYDGNEVALQ

QLPRFKFNQAIIKSEDKEIDYIPNSNVDFAVNMKNMGIEVDFSLNEVDFVDRERIRHK

YNYRCQYCGRRGTSVDHKDPVSLSHDNSFDNLILSCSECNRIKSNMPYKLFIKLNNQL

SVVNKKLVKYEDTLANLKEEFQQAKRDLAGQVHLKGIVNDPELNAMRKQNKKLQDAID
SLQSDYDALRKLRKTYFETGWKLAQEKENSEII"
CDS complement(207180..207938)
/gene="glnQ_1"
/locus_tag="OCPHGMPK_00184"
/EC_number="3.6.3.-"
/inference="ab initio prediction:Prodigal:2.6"
/inference="similar to AA sequence:UniProtKB:O34677"
/codon_start=1
/transl_table=11
/product="Glutamine transport ATP-binding protein
GlnQ"

/translation="MNNNNEENILQVQHLQKKYGDHTVLKDISFDINKGEVMTIIGPS

GGGKSTMLRCINLLEEPSNGKILFHGNNILDSHFDRNKFRSKVGMVFQQFNLFNNKNV

LQNCMIGQEMVLGRSKEEAKKIAIENLKKVGMEPFLTAKPQQLSGGQQQRVAIARAIS

MDPEILLFDEPTSALDPEMVGEVLNTIQLLAKTGLTMVIVTHEMGFARDVSDQVIFMS
DGVITEQGTPQEIFNNPQEEKTKKFLRNFRSNEL"
CDS complement(207928..209541)

```
                     /gene="yecS_1"
                     /locus_tag="OCPHGMPK_00185"
                     /inference="ab initio prediction:Prodigal:2.6"
                     /inference="similar to AA sequence:UniProtKB:P0AFT2"
                     /codon_start=1
                     /transl_table=11
                     /product="L-cystine transport system permease
protein
                     YecS"

/translation="MKSKIRWFSALLALILSLVIGFSSNSTQTEAAKKEAPLKIGMEA

NYPPYNWTQTTDANGAVPIDGSKQYANGYDVQIAKIIGKKLHRKVVVEKTEWDGLLPA

LTSGKIDLIIAGMSPTAERRKAINFSEPYRKSTFVVITSKTGKFAQAKGLNDFKGAKL

TAQQGTLHYDLIKQLHGAKREPAMRSFSAMRQSLQSGTIDGYVAEDIEYQSYKAVNPN

IVAVNLNKMQGFKVAHDDSITSIGVKKGNTELLNQVNSILNGISKKKRDQLMADAVKE

QPQAGSSKKENWFMSIMHRYGSMILGGVGMTLLLALVGTIVGFFIGLLVGIVRTIPTP

KSTGKKWGLKIVDWILAVYIEVFRGTPMMVQAAVIYYGIAQLWHLNLNRTVAALVIVS

INTGAYLAEVIRGGINATPKGQFEAASAIGMTHSQQMWHIILPQAIRNCLPSITNEFI

VNIKDTSVLSIISVSELFFVGTTVASQTFQFFQTYFIISMIYLILTFSITRIFNFIEK
                     KLEGPKNYNLMANQLQVEDPKETGFEHEQ"
     CDS             complement(209878..210504)
                     /locus_tag="OCPHGMPK_00186"
                     /EC_number="3.2.2.-"
                     /inference="ab initio prediction:Prodigal:2.6"
                     /inference="similar to AA sequence:UniProtKB:P9WJP7"
                     /codon_start=1
                     /transl_table=11
                     /product="Putative 3-methyladenine DNA glycosylase"

/translation="MNYEDFFTNRSTSEISKDLLGRTLSFNNGNEILSGTIVEAEAYV

GVKDRAAHSYGGRRSQANEGLYRPGGSLYIYSQRQYFFFDVACQAAGEPQGVLIRAID

PLTGIDTMIKNRNGKTGPLLTNGPGKMMQALGITSRKWDLARLEDSPFDIDINHKRKI
                     EEITALPRVGINQSDPKWAKKKLRFIVAGNPYVSDIKKKDVKKNHGFI"
     CDS             210579..210941
                     /locus_tag="OCPHGMPK_00187"
                     /inference="ab initio prediction:Prodigal:2.6"
                     /codon_start=1
                     /transl_table=11
                     /product="hypothetical protein"

/translation="MNEIKVVRIYDHEQPAGYRILVDRLWPRGMSKVKAHLDEWDKEI

GPTNELRKWFNHEDDKFPEFKTKYIAQLKANPVTPNFIKNVKEKLAQEDVIFLYGAKN
                     KKHNQAVVLKDFIDSQLG"
```

```
     CDS             complement(210938..211720)
                     /gene="alx"
                     /locus_tag="OCPHGMPK_00188"
                     /inference="ab initio prediction:Prodigal:2.6"
                     /inference="similar to AA sequence:UniProtKB:P42601"
                     /codon_start=1
                     /transl_table=11
                     /product="Inner membrane protein alx"

/translation="MQLIKLYQPFFDANNWAHVLTSGQDWMMILTLILMECLLSVDNA

VVLAAQTQVLPTKDEQRKSLVYGLWGAYLFRFIVIGIGTYLINFWEIKLLGGLYLLYL

VYKYFYDVRHPAQVAKKEAAKKEAHKKKNSKTRKHHLSLFWRTVISIESMDIVFSIDS

VLAALAMSNNPVVVLVGGMIGILCMRGVAEVIIKLMEIIPELQTMAYVLITIIAVKLL
                     VSLPPLNFKLPDAIFACIVFGTVILTIIFHYVRQNLHGKKMP"
     CDS             complement(211707..212237)
                     /gene="adaB"
                     /locus_tag="OCPHGMPK_00189"
                     /EC_number="2.1.1.63"
                     /inference="ab initio prediction:Prodigal:2.6"
                     /inference="similar to AA sequence:UniProtKB:P19220"
                     /codon_start=1
                     /transl_table=11
                     /product="Methylated-DNA--protein-cysteine
                     methyltransferase, inducible"

/translation="MPLAQAKVFYYDFVEIKPWTYELVISNLGLAFVGSKNSNIESSI

LGFYPNHILVQDSKKLAPYVQQLKEYFAGTRRSFTVPIDYSSFGTPFQNQVVKMIENI

PYGTTISYNDLASAINGSTSVRTVAHAVALNPSLIFIPSHRVILASDKVGSYRMGEKE
                     KQRLIKLEEGYLNAID"
     CDS             complement(212353..213066)
                     /gene="cobB_1"
                     /locus_tag="OCPHGMPK_00190"
                     /EC_number="3.5.1.-"
                     /inference="ab initio prediction:Prodigal:2.6"
                     /inference="similar to AA sequence:UniProtKB:P66815"
                     /codon_start=1
                     /transl_table=11
                     /product="NAD-dependent protein deacetylase"

/translation="MDLDNQIAALQADLNNAHHVTYLTGAGVSTPSHIPDYRSKNGIY

NGISESPEQILSEDTLFHEPAKFHHFVMENMYFPNAQPNIIHQKIAASCNKNGTLITQ

NVDGLDKKAGNKHVIEFHGNLYNIFCTKCHEKISYEEYKESYLHAKDHGIIRPGIVLY

GEPINEMVLTESIKAIQNSDLVIIAGTSFVVYPFAQLLAYRQAAAKVWVINNTPVPAP
                     KEVLTIIENAEKVFDKLYI"
     CDS             213190..213666
                     /locus_tag="OCPHGMPK_00191"
```

```
                     /inference="ab initio prediction:Prodigal:2.6"
                     /codon_start=1
                     /transl_table=11
                     /product="hypothetical protein"

/translation="MSLLYIAFDILLIGYIFYSWYWQANIDYKARFRSSSVIWALIFL

LIGFYLDYFTDPTLLMNVFIATFLLMSIIDGVSGLAKKRLVVSGYFKRTVKYSDIAHV

TLIAVPNPKKPTVMAIFQTNNRQAYYLRFSQQISDVIANIRKYLGSNVGIEVQSMM"
     CDS             complement(213751..215142)
                     /gene="cls_1"
                     /locus_tag="OCPHGMPK_00192"
                     /EC_number="2.7.8.-"
                     /inference="ab initio prediction:Prodigal:2.6"
                     /inference="similar to AA sequence:UniProtKB:P63801"
                     /codon_start=1
                     /transl_table=11
                     /product="Cardiolipin synthase"

/translation="MFHRKRSVATSWAWLIILLIFPVLGFILYGFIGRGLSQENLFAI

NNQKHIGLNKVNKMIPRIPKDTGPTDTSKEAKILIDYFNFKYDSPLSKNNKISFYTDG

EEKFKALFNDINQAKETINVEYYSFMNDDLGNKFLNLLIKKAREGVKIRLIYDPWGSP

GASKAWFKPLTDLGGEVAAFITSQNMIKKYRMNYHLHRKIVVVDGRISWTGGFNVGDQ

YVSKSKKFGYWRDTHLRIVGSASLLLQERFVMDWNASVTNKNETISFNEKLFPKIEEN

KLTEDDVATQIVSDGPDTSTPYLRNGMIRMMMMARKTLWIQTPYLVPDDPMIATWVIA

ARSGVDVRIMIPSMPDHPFIYRATQWYANYLLHEGIKIYVYNNGFIHAKTVVVDERFS

AVGSMNQDFRSYSLNFETDAVFYDKNITRELNQIFKKDLTNCTELTLDITNNWSRYLR
                     FKQAFSRLLSPIL"
     CDS             complement(215226..215933)
                     /locus_tag="OCPHGMPK_00193"
                     /inference="ab initio prediction:Prodigal:2.6"
                     /codon_start=1
                     /transl_table=11
                     /product="hypothetical protein"

/translation="MYYMPFFYDPYFWMIILGVAISGWASMNVNSTFNRYDQVRNQGN

YTGKVAARYILDQAGLYEVEIREVAGNLTDNYNPTNKVLSLSQSVYNSSSIAAVGVAA

HEVGHALQDASHYAPMRMRSALVPIVSLGSNLSMPLILLGLVLGMNQTLIRIGIWCFA

LVLLFQVVTLPVEFNASHRALRMLNNGAILSPQEMPMARKVLFAAALTYVAAVLTSML
                     QLFRLLLIFGGHNNDNN"
     CDS             complement(215957..216883)
                     /locus_tag="OCPHGMPK_00194"
                     /inference="ab initio prediction:Prodigal:2.6"
```

/codon_start=1
/transl_table=11
/product="hypothetical protein"

/translation="MFFIQKRYSEEVEKNNKHKNKEPSIARMTFLGLMATFTPTRWLL

RMHKRKVEDSSTTEAATSTITQTPIFYVHGFRGGDYTTNKMVHAACKAKGTDKFLKVT

IDPFGNFILEGTWTADPQPIVQLVFKDRIAGVYASSYYLRTALSYLSKRYHFKKYSAV

AHSLGAPSVVKVEMKTSLRKNFPHLDHCALIAGPFDGVMYLGDLPNVNRLNEKGRPIL

MSISYLGMLFNQRRFNPNISVLNIYGNILDETNTDRFISVVSAKSIRYILAPGAHTFQ
EVEVRGDAAEHSVLHDNPFVINIINKFLKLTD"
CDS 216967..217722
/gene="rnhA"
/locus_tag="OCPHGMPK_00195"
/EC_number="3.1.26.4"
/inference="ab initio prediction:Prodigal:2.6"
/inference="similar to AA sequence:UniProtKB:P0A7Y4"
/codon_start=1
/transl_table=11
/product="Ribonuclease HI"

/translation="MKYYAVKKGRTPGVYRTWEDAKKQVDGFSGAEYKSFEKITDATE

YLDWNKETQPDIVKEDSLSNAIKKIQAANITHQSKKQKVKKTSSNNSADYFATIYTDG

GSRNTGVHKGGHVNKTDRAAWAYLIEWQDGRTYGSGGEFGATNNKMELLGLINALEKL

LELKFNDKHLLFVLDSQYVLNGINKHWIEGWKKRGWKRSNGPLINATEWKRLDSLLGH
FPDSTFSWTKGHADNEGNVFVDHELNRYMDKMK"
CDS complement(217881..218849)
/locus_tag="OCPHGMPK_00196"
/inference="ab initio prediction:Prodigal:2.6"
/codon_start=1
/transl_table=11
/product="hypothetical protein"

/translation="MKKILTALVAILTSGILGLTAISNTKTVKANENDTPVVTLGTSL

TSSQKQGTINTLTQSLNGSNYTTITITGADLVKYLNPSGYDFTNNSGVWSSAMIQKTS

SGNGINVHILPYNGHNNITTITANQYKNAALTAGISDADIYVTSATPIDGSGALAGVY

AAYAKNGNSLNQNQINAAQTEMNTLSKITSQNKGKDGYSDAQLNNAIAGAKKEMAKQG

QNISDSQIRDIVNNQININHLGDTITNNQKEQIINVLIKIKNSGALKDKNFQQQAGQL
ADQIQSGAKNIFSKFNTPETRNWFQKLIDSIVSWFRSIFGGVIILN"
CDS 218977..219807
/gene="epsH_1"
/locus_tag="OCPHGMPK_00197"
/EC_number="2.4.-.-"
/inference="ab initio prediction:Prodigal:2.6"

```
                     /inference="similar to AA sequence:UniProtKB:P71057"
                     /codon_start=1
                     /transl_table=11
                     /product="Putative glycosyltransferase EpsH"

/translation="MLRQPMLTVIMPVYNMEKYLGRALEALAKQKDKNFKLLIVNDGS

KDKTREVAEKYRDRFLYFDIINKKNGGLSDARNVGMAHVTTPYFTFHDGDDWVDPGYT

AYFVRAFDEHPDVDIVSCGFWIDSEKRHESHPVGSKQSGGLIDKREAYLKMTNVFGSP

VKGYTWNKGYKISVIKKYHLRFVEDLAFMEDQIFNVKYVSVTNGFYYSSTPYYHYWQR

ADSMVHDLNPKKIPDNFRANYRVWHQIIKSLMAEREAQKENKKIAKKASLSDLDHQDL
                     "
     CDS             219832..220494
                     /locus_tag="OCPHGMPK_00198"
                     /EC_number="3.3.2.10"
                     /inference="ab initio prediction:Prodigal:2.6"
                     /inference="similar to AA sequence:UniProtKB:O52866"
                     /codon_start=1
                     /transl_table=11
                     /product="Soluble epoxide hydrolase"

/translation="MKVKVNGVEIYYQKLGKGHPLILLHGHHQDGGIFDKLVAPLSLY

YTVVVPDMRGHGLSEGEASEHYQTEVEDLRAFISALKLEKPYILGFGSGGLVALSLAA

QAPELVSKIIVAGTYVNGNGVNAKHIAANTIRGFFKGDRDSRVALRESHIPVETLKRI

KTPTLCVVGEKDWVKVEHVRWYSQLLPNCRLVLMPRQTHYSYVVHSLKLLDLIKDFCK
                     ES"
     CDS             220583..220945
                     /locus_tag="OCPHGMPK_00199"
                     /inference="ab initio prediction:Prodigal:2.6"
                     /codon_start=1
                     /transl_table=11
                     /product="hypothetical protein"

/translation="MTKIVVQGMNGEMSINDEKITIKHTAPLFPGKREVILDIHSLQA

VVYKKAHILINGFLKLVPKGDNPMIYQTASLHQMGKDELAIVLRAFENTNPKDAEDFY
                     NYVVGRLDQIKAAENNQL"
     CDS             221029..222018
                     /gene="prs_1"
                     /locus_tag="OCPHGMPK_00200"
                     /EC_number="2.7.6.1"
                     /inference="ab initio prediction:Prodigal:2.6"
                     /inference="similar to AA sequence:UniProtKB:P14193"
                     /codon_start=1
                     /transl_table=11
                     /product="Ribose-phosphate pyrophosphokinase"

/translation="MNKEELHEMLHPMKLIGLGGNQALAERIAAALDKPLLETAVQHF
```

SDGEIQVNITESVRGCDVYVIQSIQDPVNENFMELMIVLDALHRASAHSVNVVIPYMA

YSRQDTKNRSREPITAKLLANLLQLTDIDDLIAVDLHASQIQGFYNIPVDHLHAMPIL

AQYFLDNGIASKDDDDVVVVSPDHSGAKLARTFGSYFDAPIAIVDQRGARYDAEAHDM

IGDVKDKTVIIVDDLIDTGSRIASSTKSVLAAGAKKVYVAATHALLSQNAIDVLNELP

VEQIVVTDTIKHKKYPDRMVRLSVARLLAKGIDYIYNDKSIHRIFDEQNRIQG"

```
     CDS             222174..223118
                     /locus_tag="OCPHGMPK_00201"
                     /inference="ab initio prediction:Prodigal:2.6"
                     /codon_start=1
                     /transl_table=11
                     /product="hypothetical protein"
```

/translation="MKKVFQAQLYLNILIAVIILINYHTVKDWIYLGILALAVVVSKN

KRISQLINIVLIPMIFIDQVRNLSDILIQHFSQLTLLIFWIYAVGTIIVLIPVTIVEY

GKIKKPIWRLIASVWMINFVIMFCYLLTLKNVNPDGFLVSLNKSGLVYALAILVYVYF

AVKSWGYEFCFNLPTFKGKKLQLLSFILIFGIAIWLSFFQTFSRFAQRWQELFWNWDF

SLLDPTESVRLKNAWSVFLYSIEAGIGEEAARYINLVLLLVIFKSKKRQINGAVLGSA

```
                     ILFALPHIGNAFASELKQTPLATAFQVIDTFGFGWRQC"
     CDS             223112..223375
                     /locus_tag="OCPHGMPK_00202"
                     /inference="ab initio prediction:Prodigal:2.6"
                     /codon_start=1
                     /transl_table=11
                     /product="hypothetical protein"
```

/translation="MLILYSGKLWPTMVIHALYDILTFSETPLTQDGVGIFGGNIGSL

```
                     IHVIISLVLWVSFAAFLLIKNRKLIKQNVQILTQVQKTDLTIS"
     CDS             223508..224857
                     /gene="cycA_1"
                     /locus_tag="OCPHGMPK_00203"
                     /inference="ab initio prediction:Prodigal:2.6"
                     /inference="similar to AA sequence:UniProtKB:P0AAE0"
                     /codon_start=1
                     /transl_table=11
                     /product="D-serine/D-alanine/glycine transporter"
```

/translation="MDEKEKLKLKRSLKSRHVTMIAIGGAIGTGLFLGSGTAIQSAGP

SIILSYLIVGIISFFMMRALGELILTEPDQHSFIESVKKYLGNRMEFVAGWTYWLCWL

SLAMADLTATGIYLKYWFKDLPQWVGPLVIVVLLMLVNMVNVGLFGELESWFSMIKVL

AILALIITGAILLVMHTKIDSRTVSLTNLVNYGGFFPTGSWGFLKSFQMVVFAFVGIE

IVGLTAGETANPDKDIPKAINTLPVRIGLFYVGSMIAIMAVYPWNKIVTTTSPFVQVF

GAIGIPAAAGILNFVVLTAAMSATNSAIFSTSRSLYALSRGNNAPKRFGKLSRKAVPN

NALTFSSLILFIVVILNYIMPAQVFDIISSVSTINFIMVWIIIMWTHLKYRAENKGKL

GSFRMPGFPITSWVTIIFYLVILIIILALIPATRLPLLISIIFILALSLSYTFLERKN"

```
     CDS             225032..225691
                     /gene="slmA"
                     /locus_tag="OCPHGMPK_00204"
                     /inference="ab initio prediction:Prodigal:2.6"
                     /inference="protein motif:HAMAP:MF_01839"
                     /codon_start=1
                     /transl_table=11
                     /product="Nucleoid occlusion factor SlmA"
```

/translation="MARQKNLARRHEILRNTFMLLKKRGMENVSLQMIADKSGISKSL

LQSYYPHKNLLITEIVTNFMTAVLKSLNATDFKNLNTYSKMKIFIYLILEQGIQDEGV

ERVVKSILSDGDSLDRWSQILDDWLKHEGVKHDLGNDRQVQIGLNFIVTAGGGLYVKR

TEFDLDADQISDIMVKTFMSTFLSIDESKISQTLAEAKSALQKYELTTLTQAINKMFD

```
                     N"
     CDS             225712..226551
                     /locus_tag="OCPHGMPK_00205"
                     /inference="ab initio prediction:Prodigal:2.6"
                     /codon_start=1
                     /transl_table=11
                     /product="hypothetical protein"
```

/translation="MAVRTRSELRRIDQAQTVHDKRSFGIAIFTIVVLFFSLVTFMNP

IFMRSQIAKESNGVVAERYINQKFDNFAAAIGADRSSNNSTNNLLTVEQTRPIANAMI

DYTLGIHLFRAENSSLASQIRKVILTKIDDNSSMEAKSVQEKLKKNKESGLYAIITSF

GLANATLAANVELVFLILNILVIIFVGILAYQQIKRLKEIVSTGRLINMFAAGGMRAS

LGLIVIFGLLAFIPTIFNVEGLILNIGYFLEIASGIFLELVIVGVVLFVISTVTWQLT

```
                     SAK"
     CDS             complement(226880..228406)
                     /locus_tag="OCPHGMPK_00206"
                     /inference="ab initio prediction:Prodigal:2.6"
                     /codon_start=1
                     /transl_table=11
                     /product="hypothetical protein"
```

/translation="MNFTKKATAFLVAITLSATTSTVTISEALAATFTKDEIQEVHRI

QNQYSRLPKQTFNSGNLYASSPHLTAPFSPGSVTNSYINSQLDYINFYRGLFDLPSIS

TNKTDNDNAQITASVMAAIKANPFTNQHGLPSETRPNYISDTYWTIAKNVSASSNLNF

NVSNQSAGDVITDLLTDTYNLDGSDTGHRAWLLSSRLTTTGIGAAYGENSYRYSVQQV

```
AYSSDGYKAAAKSAVAYPNSGVFPIELLQGNNIAWSLYLSDKTTSGIPKITVTDLDTG

EVSQATNVNNFSNKAYGYFKTIITYFPGDIKLVSGHEYNVNIDNVYQYSFKLFNQVAA

NQPKLKTSNDNTKTKNGEKSSEKISSSQNIKDSSDKTTRKILNQVADPDSSTTIKSAL

LLQAEKLRDSLNKKRQMNTVIFGRSYQDGYSYYNLGNDQWFHNFYVYNNPDFTAGVVN

INNQSFDTNIYTSPYPNLRKRTANHVTSGKSYAYGQSITTDHITWYYLGKNQWIRQNN
                     "
     CDS             228565..229650
                     /gene="ddlA"
                     /locus_tag="OCPHGMPK_00207"
                     /EC_number="6.3.2.4"
                     /inference="ab initio prediction:Prodigal:2.6"
                     /inference="similar to AA sequence:UniProtKB:P0A1F0"
                     /codon_start=1
                     /transl_table=11
                     /product="D-alanine--D-alanine ligase A"

/translation="MTDKIKVGLIFGGNSSEYEVSIMSAHNIYEEIDTNKFDVYPMWI

TNDGYLADDADSRRVLDNPKMEVANPHKVANISNIVELKDRPEIDVFFPIVHGNLGED

GCLQGLFRVLDKPFVGDDVLAAAVTMDKEMTKILAQRAGVPVAKWIAVKRFEYNDPDN

EKLDYEYVASQLGSDLFVKPSNQGSSVGVSHVTNEKEYKVALAEAFKYDDKVLVEETV

HGTEVETAVLGNDKPIVAGVGQIINAKDSFYTYENKYDDDSTSTLEIPAKLPEGIVEK

VRKNALKVFQATECSGLARIDSMLRTEDKEVVLTEVNALPGFTNISMYPKLFEEVGIP
                     YTDLITKLIDYAMERYDHKKTLLHKHD"
     CDS             229680..230399
                     /locus_tag="OCPHGMPK_00208"
                     /inference="ab initio prediction:Prodigal:2.6"
                     /codon_start=1
                     /transl_table=11
                     /product="hypothetical protein"

/translation="MASDHNFEELEAPAKTAEELHISVATLRKYSLIVEKVTGNKDYY

ERTKQKSRLYSAKDIEDLKAFKALSKKADLTLQEAARQVFAVSDKKAEEAKKAAKMQK

EVATKTATVDADQVVKLLTMLQNTIASQNQAIQDLQKQVARVEAQNKKLIDSSHQLAE

PESTDSQKEVQANKDQEAADKITEEQKSEQEAHEEILRKARENEEKRRKQKVEENIHR
                     TLAQMQIHPKKKHHWWDRFFN"
     CDS             complement(230433..232004)
                     /gene="nhaK"
                     /locus_tag="OCPHGMPK_00209"
                     /inference="ab initio prediction:Prodigal:2.6"
                     /inference="similar to AA sequence:UniProtKB:O32212"
                     /codon_start=1
```

/transl_table=11
/product="Sodium, potassium, lithium and
rubidium/H(+)
antiporter"

/translation="MELMISTFTLAIAAAISIIIAQAIDKVSVNYISMIIGIIIGLIP

FLNQQIASFDSEIFMELIVAPLLFFEGQKTRIHNVGRRIKEIIGLTIIMVLLALIVSG

FSIHLLVDVSLPLAFIIGSISTPTDATASEAVTNGLRIPRRVTSALKAESLFNDASGI

ILLNMSLLWFANGYINYGQTIQDFCISSIGGAILGFAVAWVIIIFRQTLVRSRFNSLN

AQNLIYILTPLALYALAEHLHVSGIIAVVVAGLLHNAESQQSLLLNSRQVHMSQDLQN

LISDIFNSMVFIILGLMMVRISRNKLFNGDLAKWIIVGIIVYLANLLVRYLYGRLIIR

YDRRESIVFSLGGIHGAVTLALALTISVDFLGSQRYNLVIMSEAVLIIILSMLVPTIVF

QFILPHNVSDEEAHIVMDKIRSEMVKRALVAVHKMYLPQRVKRHVIYTLLNQKRVVKT

REYMRVLLKTIDQPNLSKSEQYLQRLAFFRAFAIEREYLEMIGQKEGKYRTYILNLYN
DVLLAESLIIEPEDE"
CDS 232113..233219
/locus_tag="OCPHGMPK_00210"
/inference="ab initio prediction:Prodigal:2.6"
/codon_start=1
/transl_table=11
/product="hypothetical protein"

/translation="MKKNQKVWLSLTTLIVGIVFTLITYFSTTFASKPIAVITDNHIQ

DTLINKTKDNYENKDEVRQQILHLKIISGKYRGKRFVVTNTYSPSQAVSQKYRPHQRV

IVSFIKEKPKLVEPKRDWVVVLSMFLTISLIIILITGKQASLLLISMILNSIIFYFVIK

NDIKENGTKIFLVYGIAAILFTFISLIIVQGFNQKMLVTLVATLLGVFVSFGIFYLVM

RLTHERGIDYEAVDYATQDPRALFLSQTILGVLGAVMDEATDIVSSLYALAKHKVDLT

FKELFLSGRTLGQEIMGPLINVLVLIFMAEALPMTILYLRDNNTLVYTFKYTLSLGVI
QSLSSAIGIVLTVIFATLASSVFLKNKKIEEAAK"
CDS 233216..233977
/locus_tag="OCPHGMPK_00211"
/inference="ab initio prediction:Prodigal:2.6"
/codon_start=1
/transl_table=11
/product="hypothetical protein"

/translation="MTTLTALLIIILLILMIIVGGKTGLKSYLSVVINACLIIILVALLI

SWGVNIILVGIIFIPLKLLTIIYLGTHDYTVAKNAFLTALCVSLIVMLIIIILLESLAQ

TQGFGDQAGEELIGLSLNVGISFAQIAILVAIFSMLGAIAEASVAMSAGLLELKRHDP

NITQKQLIKSGNEVGSDVLGTAMNTILFGLFGSFLPIFIWYMRLNYSLFEILNDKLFV
DEFLIIVYSFIGVLLTVPLTTIFLAHTLTHNELKK"
CDS 233999..234817
/gene="axeA1_1"
/locus_tag="OCPHGMPK_00212"
/EC_number="3.1.1.72"
/inference="ab initio prediction:Prodigal:2.6"
/inference="similar to AA sequence:UniProtKB:D5EV35"
/codon_start=1
/transl_table=11
/product="Acetylxylan esterase"

/translation="MKVEYLTQKNFNNREFKITYYGLQDNPQLLQKKRPLILVFPGGS

FDHLSLREGEPVALAYAAHGFNAAVVSYNLTTDPGKIYPDAALSGLKAVSYFRENSEK

LGIDQERITTIGFSAGGHVVSSMNVMAESEKCQAKYDFNHDQVAPNATILGYPLINIK

DIGFPLPADANQKMPTAKELLDTALGVTPDTPPTFIFQAVDDPVVLIDNTIEYITALR

KKKVHFEAHLFNKGGHGFSLATPEVAVGGREPDSHLAHWFKLSLEWLKDRKIYI"
CDS complement(234917..237976)
/locus_tag="OCPHGMPK_00213"
/EC_number="2.4.1.161"
/inference="ab initio prediction:Prodigal:2.6"
/inference="similar to AA sequence:UniProtKB:B3PEE6"
/codon_start=1
/transl_table=11
/product="Oligosaccharide 4-alpha-D-
glucosyltransferase"

/translation="MSEITNNQQPQQLSRLVQVAKGTRYYDLQFADGKRARLYIIAKG

IFRLIIDPAAEFKPLSPSLTITAGNFSLRPFEESQLLVTDETFTIKSGQFSLRLQKNP

AIFSIFDDQLHRYRMMQSSPIELGADYSSEFLRQNKNEFYYGGGMQNGSFSHKGKIIE

IKNTNLTGPGAVACPEGFFWSNAGFAELRNTWKNGIYDFKGENGNTAILTHQTPIFDN

FYLLGDSPAEILRQYYKITGSPLFLPKYALGLGYLGNYLDTCWSVAKPQENNAIKFED

GNTYKIAHADNEIVAKSSLNGEEQYQFSARAMIERYRSHHFPLSWFVPNYNSKQTLNE

TELANLVDFAKEQNIKLGFYGALPANAPANFLKFKTEAPNLANDKIMQDFAIASEPLV

DKNQTNRPWAMASNGWSAVQTLATTANEGVGGEWEQIATEIASFLSMSLSGQPNIGSA

IDGKEGGGNAQVNIRDLQWKVFTPLLYSIDNYGNIQKTPFAFNAKMTRITRAYLKLRE

EITPYLYTLTRAAQDGMPIVRPLFLSFPHEKVNYTNQVKHEFMLGNNLLIAPITNGRE

DPSGASLKDNLYLPDHRTMWIDLFTGKKLIGGRIYDKLHYPLWHLPVYVRGGAILQES

LRSALVYPQNKSSAILYEDDNQTNDYEKGNAATTQITSSVNGSKLHITVYPTEGSYEG

LEEKQATILTIMADRYPGKVSLKLNDQVIPLPEYNDLDSFNKSEEGIFYQQDYVPCKE

FGQFTGQLQPALKVKMPAVDIHDNKFELTIENYSYGSEVEQHAIIDSAMASPRSAAIL

TDRLTSKSITITWTNPTNFVGKNIMADIEVNGIVHTNIDGDTFTFHELEPNTRYRFRI

RNKFGNKVSDWSEYFGTHTKRDQMDYAISNVKVTSTAESENDSPVKNLTDLKLASEWL

SKDQISKDSPLDLTFNFNHIYKLSRMVYVPRSRDRIGHVLRVQVAISKDGENFSDFSK
EINWPNDAKNKVIGLRDVVAKAIKLRILATSDNLASGKEVLFFMAKE"
CDS 238135..238854
/gene="yvoA_1"
/locus_tag="OCPHGMPK_00214"
/inference="ab initio prediction:Prodigal:2.6"
/inference="similar to AA sequence:UniProtKB:O34817"
/codon_start=1
/transl_table=11
/product="HTH-type transcriptional repressor YvoA"

/translation="MKKPLYQQVILDLEKKIKTEMKPNNRLPSERQLLKEYGVSRNTI

RLALNNLEERGIIYRLHGKGTFVSTIYLDQTNLGSMYSFSEQMSEAGHKPTTQNRTLE

LVVPDAAVANQLNLKAQEKAYKLVRLRLADGVPLMYGETYLPESIFPDLKMSDLNENH

LYTVMKQKYNEQSVLAFEDVQAVNLNERDSKILGVKPGDASLKIFRRAINDKNVPIEF
TISLARGDRFIYRSRQYNHLV"
CDS 238875..240041
/gene="agaS"
/locus_tag="OCPHGMPK_00215"
/EC_number="3.5.99.-"
/inference="ab initio prediction:Prodigal:2.6"
/inference="similar to AA sequence:UniProtKB:A0KYQ7"
/codon_start=1
/transl_table=11
/product="D-galactosamine-6-phosphate deaminase
AgaS"

/translation="MFTKSDAELKKMGADITTREIQQQPSLWKETFKIYQEKKQEIDK

FLAKINQKFNKVKVIFTGAGTSAYVGNTVMPYLRKHADRTKYDFEAIDTTKIVSTPED

YLEAETPTILVSFARSGNSPESVATVELAKQLVTNLYQIAITCAPEGHLAQDLKDDPN

GLVLLMPAKSLDQGFAMTGSFSCMSLATLLVFDTRSDAEKETIVNEIAEMGESVVDRE

AEIQKLVDTDFDRITYIGSGALGGLAEEARLKILELTAGKVAALFDTSMGLRHGPKSF

LDKKTIVFDFVSNNSYTRKYDLDILDEIKADEIVPLVMGVGQLKKGQDFDGKFFAFSG

KELPDAYLALPDIMFGQTIALLTSVKVGNTPDTPSPTGTVNRVVKGVTIHKFIK"

CDS 240103..241260
/gene="nagA"
/locus_tag="OCPHGMPK_00216"
/EC_number="3.5.1.25"
/inference="ab initio prediction:Prodigal:2.6"
/inference="similar to AA sequence:UniProtKB:Q84F86"
/codon_start=1
/transl_table=11
/product="N-acetylglucosamine-6-phosphate deacetylase"

/translation="MSYYIHAGKFFLENKTENGGYLEVQDDGNFGLYYREDEKPSHSL

IKEYGDQWIAPGYVDTHIHGLLDEDTMKSDWEGVNKISEGLLQAGVTSWLPTTITASD

KELTETCEKFAAHKGEETGAKIQGLHFEGPFFTPKHGGAENPKYMTDPSIMLLKKWRK

ASDNMLIKISMAPERKNARQFVREAVKMGLVVALGHSDSDFEDAIACVEAGASVFTHT

FNGMNGLSQHSPNIIGAAFSSRLTTDELICDGHHVEEPAVRALVNARGPEHIALITDC

MQAGLMPDGDYVLGELPVYVKDGMARLKDTNNLAGSILLLKDAVKNVVDWNVATPEDA
VMMASYVPAKSCNLLDKCGVIKPDHPADFVVLNHDMTVSETYLNGESRYKA"

CDS 241400..241888
/gene="levE"
/locus_tag="OCPHGMPK_00217"
/EC_number="2.7.1.202"
/inference="ab initio prediction:Prodigal:2.6"
/inference="similar to AA sequence:UniProtKB:P26380"
/codon_start=1
/transl_table=11
/product="PTS system fructose-specific EIIB component"

/translation="MNIVGARIDGRLVHGQVANLWTPKLQADRIMVVDEDAAKSDIQK

SGLRMATPLTTRLSVLPAQVAADHLIHERYGNQRIFIVAKKPAAFLDLLNLGLKLDTL

NVGTMSQTDTTKQVTKQINVEEKDVEDFKKIADKGVKITAQLTPSDDSHDFMKLMNEK
IK"

CDS 241900..242817
/gene="sorA_1"
/locus_tag="OCPHGMPK_00218"
/inference="ab initio prediction:Prodigal:2.6"
/inference="similar to AA sequence:UniProtKB:P37082"
/codon_start=1
/transl_table=11
/product="PTS system sorbose-specific EIIC component"

/translation="MAWWQILLLTLYAGYEILDELQIYSSLNTPVGAGLIAGLIMGDL

KTGLIIGGAMQLTVLGVGTFGGASKIDATTGTVLATAFSVSIKGMSPQVAISSIAVPV

AAIMVQLDVLARFANTYFAHRIDHLIEENNYKGIERNFLYGAIPWALSRAIPVFIALA

FGRGLVQTIANSLNGNLKWLGTGLTVAGAVLPAVGFAILLRYLPVKKHIAYLIMGFTF

TTLFSVLFTSIQTLGTGLQAANKSFTATFNGLPMLAMALIGLAFAILHYKDEISGKGG
SGKGGTKEAAATTESTNVASEGEITDDEL"
CDS 242804..243622
/gene="manZ_2"
/locus_tag="OCPHGMPK_00219"
/inference="ab initio prediction:Prodigal:2.6"
/inference="similar to AA sequence:UniProtKB:P69805"
/codon_start=1
/transl_table=11
/product="PTS system mannose-specific EIID
component"

/translation="MMNSKPKYKLTDKDFNQINRRSLFGFQLGWNYERMQNTGYLFLI

LPQLRKIYGDNTPELQEMMKTHVQFFNTSNFFNTIITGIDLAIEENEGVEGKDTVTGL

KVGLMGPFAAIGDSIFAALIPTIFGALAASMASQGNPVGVFIWIAAQIAICFFRWKQL

RFAYDKGVTLVTEMRDRLNALTDAATVLGVFMVGALVATMVNVKFAWVPQIGKVTMDI

QNNLDMIIPKLLPALIVGFVYWLLGKKKMTSTKAILIVLVLSVVLGGIGVLAKI"
CDS 243646..244044
/gene="manX_1"
/locus_tag="OCPHGMPK_00220"
/EC_number="2.7.1.191"
/inference="ab initio prediction:Prodigal:2.6"
/inference="similar to AA sequence:UniProtKB:Q5XAF5"
/codon_start=1
/transl_table=11
/product="PTS system mannose-specific EIIAB
component"

/translation="MAKELVLISHGKMAEEVKKSAELIMGPQEHIHAVCLLPEEGPED

FEKKFQDTINGIPEEDLTVFADLMGGTPANTVSRLIMGGQNIHLVAGMNLAMVIDWLN
SQMIGNDSDAVNAGKAGIVDINQMLASMKK"
CDS 244237..244431
/locus_tag="OCPHGMPK_00221"
/inference="ab initio prediction:Prodigal:2.6"
/codon_start=1
/transl_table=11
/product="hypothetical protein"

/translation="MTKIIIINSQVSAEGQSEDLKALAKLMNNEPVNLNKYFDYAQRR
IKEINEDPEMREKTVLNKEK"
CDS 244461..244562
/locus_tag="OCPHGMPK_00222"
/inference="ab initio prediction:Prodigal:2.6"
/codon_start=1

```
                     /transl_table=11
                     /product="hypothetical protein"
                     /translation="MDNGSTLEQATEFVRNLKLISDEELNKLIALYK"
     CDS             244799..245875
                     /locus_tag="OCPHGMPK_00223"
                     /inference="ab initio prediction:Prodigal:2.6"
                     /codon_start=1
                     /transl_table=11
                     /product="hypothetical protein"

/translation="MPKRSEIHSNKLKSQKRNIIIGAIIAVFVLILGGWGLYQHGRNS

AESTVIGGNGRLTNAEIKKIPRKQLAAWAILYADKKYGKKWGEAADDLRSGHLTIKSY

SKYRFGDYEISATDGSTLYVINDQVGYLVSKDNKEITYVGNKSSYSNRSVLSQIYPEI

KSENTEKNVNIWNDNLSIVAGKSDENVNDQSSKTSTKSKKTTSENKLWSSNQNDELIS

YMDEFGEKMHQSYEHYIDGKDDLVTLAGEHFPSDINENTYPFKLSSGDHDSRDPKDMD

SIKLKWNPQVTSKDNDSDTYNVVAIFNHNGKSAEDHITYLFCFYHGEPVALVDQTTNG
                     GYVVVHPTANQDLTSHFEEIANND"
     CDS             245896..246909
                     /locus_tag="OCPHGMPK_00224"
                     /inference="ab initio prediction:Prodigal:2.6"
                     /codon_start=1
                     /transl_table=11
                     /product="hypothetical protein"

/translation="MKNRVFIFLLVGSAVLAMAGCQSRYKQEKGSNEQAKIIKQSITN

KNVSIVGSYRDDTDGAAIAFNSDHTGRYVYADPKNSDTDDRLTWKKTGKNTYIIRLND

NDVNSALTAKLTGSTLVISGDDDWNTETFNKTKNKLNLDDFLSSKKVESSTSSKAKNN

VKNSKSTNSVPGDEGLFDMPNELRGTWYLANESDGSVSKLIIDKNTITGEGFIEKLHK

MNKNFDFTSYAQNHPSYQDMTKDWARVVVLSENGESKVNVRGWLQSAGAGEYYNLATE

HGQKVLVIGGGAEAWVDGIGWRNKTDAEKYGKEKFSDLHYREDISLENNDDSDSAATN
                     DED"
     CDS             complement(247003..247479)
                     /gene="ntd"
                     /locus_tag="OCPHGMPK_00225"
                     /EC_number="2.4.2.6"
                     /inference="ab initio prediction:Prodigal:2.6"
                     /inference="similar to AA sequence:UniProtKB:Q9R5V5"
                     /codon_start=1
                     /transl_table=11
                     /product="Nucleoside deoxyribosyltransferase"

/translation="MTKQKTVYFGAGWFTETQNKAYKDAMSALNANPTIDLENSYVPL

QNQYKDIRVDEHPEYLHDKEWAQATYNGDLVGIKTSDIMLGVYVPKEEDVGLGMELGY
```

```
AMSQGKYVLLVIPDELYGESINLMSWGVADNVIKMSELATFDFNRPRYNFYDGAVY"
     CDS             247564..248124
                     /locus_tag="OCPHGMPK_00226"
                     /inference="ab initio prediction:Prodigal:2.6"
                     /codon_start=1
                     /transl_table=11
                     /product="hypothetical protein"

/translation="MFLKSVLYNETRNFCLGKAEPTTNTKKSLFDPEAVFVTDLTDRN

EIFKTVSQKLYDLGYVKEDFLGDIIEREDKYPTGISMRPLSRELPDVAVPHTEGNYVS

TQLIVPIALRNSATFNNMVNPTEKLKVKFLFLILNNDPDMHSTILAKIMDFLAGTSVD
                     DLNELFNSDSNEEIYDFLETNFETEK"
     CDS             complement(248171..248896)
                     /gene="yvoA_2"
                     /locus_tag="OCPHGMPK_00227"
                     /inference="ab initio prediction:Prodigal:2.6"
                     /inference="similar to AA sequence:UniProtKB:O34817"
                     /codon_start=1
                     /transl_table=11
                     /product="HTH-type transcriptional repressor YvoA"

/translation="MDIPKYLQIENELKKEIISGKFKYGDKFYSEAQLKEKFNVSSIT

VIRSVKDLVKAGYLVRHQGKGTFISRSRKDKLVRLSDNEVFSDKQEEDRVKVVKLQED

NQAKMLKKLGLPKYESYYYIERLRLIDDTPFMVHRSFIPAKFINKNLAKDPNNYHSIY

ETLQKDKQLYLFDEPFTETDTIVKANSEISQQLKIAKDYPVVRQVKKTILSSTGEVVE
                     LVVGYKRCDYFSLSFSSTDYPGV"
     CDS             complement(248903..249706)
                     /gene="iphP_1"
                     /locus_tag="OCPHGMPK_00228"
                     /EC_number="3.1.3.48"
                     /inference="ab initio prediction:Prodigal:2.6"
                     /inference="similar to AA sequence:UniProtKB:Q05918"
                     /codon_start=1
                     /transl_table=11
                     /product="Tyrosine-protein phosphatase"

/translation="MSENYETKLIGIQNGRNFRELGGYKTVSGQTIKYNKLLRTGNLA

DLSSSDLDLLQRHGVKYDVDFRTEKEKTEHPDRVPEGAKYIFDPVFSDDLTNASKGIF

ALEENAEKDPEFGFKHMFFAYEDMINGQTAQKAYRKFFDLLLANDQEKNALLFHCTAG

KDRTGFGALLVLSALGVPFTTIKYDYTLTNVTTKDFVDGMLKQAAADGSSKRVLQSIK
                     DIQSVYPEYLDHAIHVLNQQYGGINEYLRSTMKLSSRDIMDLRRIYLKD"
     CDS             249880..250374
                     /locus_tag="OCPHGMPK_00229"
                     /inference="ab initio prediction:Prodigal:2.6"
                     /inference="similar to AA sequence:UniProtKB:Q7A551"
```

/codon_start=1
/transl_table=11
/product="Putative universal stress protein"

/translation="MVKDYNNILVPVDGSETAERAFDKAVKIALDNNAHLDVLNVIDT

RQFMGEMQDTLISGDTIYQMTQDSEEYLKSLKEWAKEHFNFTDVSYHIRYGSPKRIIA

VDFIKDHHNDLIIMGATGMNAVERMLMGSVTAYVNQHALSDVLIIKTDIDNKKVAKPK
KKYF"

CDS 250556..250702
/locus_tag="OCPHGMPK_00230"
/inference="ab initio prediction:Prodigal:2.6"
/codon_start=1
/transl_table=11
/product="hypothetical protein"

/translation="MMVNFDYNIIKRNQDSVARSWAGSFWESVFIGVGAFVGFLATFK
TQCD"

CDS 250777..251706
/gene="phnD_2"
/locus_tag="OCPHGMPK_00231"
/inference="ab initio prediction:Prodigal:2.6"
/inference="similar to AA sequence:UniProtKB:A0QQ71"
/codon_start=1
/transl_table=11
/product="Phosphate-import protein PhnD"

/translation="MKKFKKVLVGLFAILLAVVATACSNKSNSSKDGYTPKSLTIQFV

PSQAANKLEARAKPLEKMLSKKLGIPVHVSMSTDYNTVVEAMKSKKVDVGFLPPDGYV

LAHKQGAANVLLQAQRYGVKQPGGQATKNLVNSYRAEILVKKGSKIKSWKDLKGKSIS

VQNPTSSAGYVFPVAELEEKGLNVPKDCKLVTVTGHDQAVLNVLNGDTDAAFVFEDAR

NIVKKDNPKIMDQVVPIYFTKPIPNDTISIRSDMSKSFQKKLAKAFIEVGQSKEGKKI
IESVYSHEGYTKSKDSNFDIVRKYDKIIAKDKK"

CDS 251814..252599
/gene="glnQ_2"
/locus_tag="OCPHGMPK_00232"
/inference="ab initio prediction:Prodigal:2.6"
/inference="similar to AA sequence:UniProtKB:P27675"
/codon_start=1
/transl_table=11
/product="Glutamine transport ATP-binding protein
GlnQ"

/translation="MAATNQPMIQLKDVSKIYDNGTVGLKDINLNIDKGEFVVVVGLS

GAGKSTLLRSINRLQDVSKGDILIDSKSITSAKGKDLREIRRDIGMIFQSFNLVKRSS

VLRNVLTGRVAYYPTWKTTFNLFTKEDKQKAYEALQRVDLADKVYTRADQLSGGQQQR

```
VAIARVLTQNPKIILADEPTASLDPQTSRRVMHDLKMLNEEYGMTVVANLHSVELAKE
                     FGDRVIGVRAGQIVYDGKMSETSQAVFDDIYNGGNGKKGEEDA"
     CDS             252592..253395
                     /gene="phnE_1"
                     /locus_tag="OCPHGMPK_00233"
                     /inference="ab initio prediction:Prodigal:2.6"
                     /inference="similar to AA sequence:UniProtKB:A0QQ68"
                     /codon_start=1
                     /transl_table=11
                     /product="Phosphate-import permease protein PhnE"

/translation="MPKSKEQHLAELPKKKFKIMNLVWVVIMVLGLFLSVNVTNTHIS

VLFNNFSQFTDIFVQMCHPDWGYAATAVPLLIETIKMAILGTVIGSAIAFVYSLLIAR

NIVKNKAVTGILRVIMNIVRTIPDLLLGAIFVAIVGIGPVAGVLALSVFTFGVVVKLF

YEAIETIDPGPIEALTAVGANKLQIIHFAVMPQIITYFISYVLYAFEINVRASTVLGY
                     IGAGGIGLYLQQTLQVFDYAKTGLIILIIIVVVVLIDYVSTKSREALMK"
     CDS             253395..254207
                     /gene="phnE_2"
                     /locus_tag="OCPHGMPK_00234"
                     /inference="ab initio prediction:Prodigal:2.6"
                     /inference="similar to AA sequence:UniProtKB:A0QQ68"
                     /codon_start=1
                     /transl_table=11
                     /product="Phosphate-import permease protein PhnE"

/translation="MDTTMKKLPPKPIDTKKKIKHWVIAIVTVALIIWSFVGMNFGGI

KATAGQIAGAILAGIFHPDWSYVYNGSGEDLISQLWETICIAFLGTFISAVISLPFAF

WAANTRHKKWYTSRSGKIVLAVIRSFPEIVLALMFIKAVGPGSAAGVLALGFHSVGML

AKLFSEAIENLEEGANEAVTAAGGSKFNIIMFATMPNLMPALISNTLYRFDVSIRSAS

ILGLVGAGGIGYPLIIALQYRQWNRVGVILVGIIVMVVIIDWISGWIRKKLV"
     CDS             254360..254842
                     /locus_tag="OCPHGMPK_00235"
                     /inference="ab initio prediction:Prodigal:2.6"
                     /codon_start=1
                     /transl_table=11
                     /product="hypothetical protein"

/translation="MDNLTTMTLADDIALSQEKIINGQQDLNTKRIYNEINSLGILDK

PIEEYFDMTQDEYYNSESDGKLTLLHLDQPLSELSDRILTNHVDGYVDKDEINLTYNH

EDPLQDGSYDRATDLHVLLYSLKVIGAVRAIAPDDLKKVLSKDAVLSLGLAAAALAKN
                     "
     CDS             254895..256289
                     /gene="cpdA_1"
                     /locus_tag="OCPHGMPK_00236"
```

/EC_number="3.1.4.53"
/inference="ab initio prediction:Prodigal:2.6"
/inference="protein motif:HAMAP:MF_00905"
/codon_start=1
/transl_table=11
/product="3',5'-cyclic adenosine monophosphate phosphodiesterase CpdA"

/translation="MKIINKTKGMIDKFSGMTRKMMRKKNNVPFDGMQKYMTVGEYNV

GAPEGTPHGEEYKLIVYKDTDNVLHQALRSVDASDLAPAYVRKFDKRLNRYTAFVNKR

TGRRYIVEDQLDQLVDYVKKRSRKGYNSINIGVLTDTHYKDTDSIDFYGHNGLRHVKE

FNYLEDKDILDLKAHLGDWMDGSDPGLVGEAELISLSRTFRSKKTNFAVIKGNHDEND

KFDEHHDLRASFPENEFEDIMWPSMYAQDGIHYITRKHGVAYFDKDDVRIITVNTSDL

PYELDDQGKKKYDTKLSLAIREDQMQEIIEILENSNGKTIIFMSHANPITRKGTNALK

FNGRSLHELMVAFNQHEKGYLNSRDVPPEFTLANSFDFTKIKNAKIVAYFCGHRHTED

QYRINGIQYIFFNCSALMGPNHALTTQYNKRWNRQMDHANEAAGYIVNVDIKRHLLQV
FGYGAASKRRFYRI"

CDS 256350..258299
/gene="asnB"
/locus_tag="OCPHGMPK_00237"
/EC_number="6.3.5.4"
/inference="ab initio prediction:Prodigal:2.6"
/inference="similar to AA sequence:UniProtKB:P54420"
/codon_start=1
/transl_table=11
/product="Asparagine synthetase [glutamine-
hydrolyzing] 1"

/translation="MCGICAFFDPELKDKDCAIQGMMDTIKHRGPSSDGKYTNDQVAL

GFRRLSIIDLRGGSQPIFNEDKSRAIIFNGEIYNFKPLRKELIDAGHTFTTKADTEVL

LHGYEEWGMDGLLKRVRGMFAFVIWDDNTKTLYGARDFFGIKPMYYSDQNGKLIVGSE

LKSFLAYPGFKKELNTEAVKPYLMNQYNDLKETFFKGVYRFPAGHWFEYKDGKMKTHQ

YWDAKYVENSLSFEETLDKINEDLKETVDLYRNADVPVGAFLSEGIDSSYLTSLLDPE

DVFSVSFDDSTYNEASKAKALSDLHGWKFFADKVDADEAMRDFPEMQYHMDEPDANPS

IIPLWYLCKLARKHVTVALSGEGADELFAGYVNYGMHTHNDVIKVFTAGLKKLPKKSR

VRLAHKIKKMPNFPGKVHMYTNLAEPSEFYVGQSVIYDMDYPTIFTSKDANNILRDKY

KNDLTVNGIYQEDFKKVKNIDEVKQMQYIDLHHFMLNDIEQKADKISMAHSLELRVPY

LDKKIAELANSIPTKYLVNRHDTKYALRKASEKVLPEEWAQRPKLGFPTPIKQWLKEP

RFYKQVRELFTEDFVNDIFDQKKIVKLLDDNYKGDGSARRQIWAIYTFLVWYKLFFVD
YDETVKKYQHVQPEVAELIESGRLV"
CDS 258329..259660
/locus_tag="OCPHGMPK_00238"
/EC_number="6.3.1.12"
/inference="ab initio prediction:Prodigal:2.6"
/inference="similar to AA sequence:UniProtKB:H8L902"
/codon_start=1
/transl_table=11
/product="D-aspartate ligase"

/translation="MVQAKFTPILLGSDINVYGMARSFNEAYGIKVQAWAASQLAATR

YSKIVDVEVHEGFEEDPGFMNVMKQKIEEYKNHPEPVILIACGDGYAELLAKHKDELK

DTFVVPYIDYDLLEKLISKEGFYEIAEKYGLPYPHTKIVTMDDYKAENYLNLPFDYPI

ELKPEDPVSWLNCQFEGRKKAFTIHDETELVDIVGKIYTNGYTEDLILQDFIPGDDSN

MRVLNAYVDKDHKVKMMCLGHPLLEDPTPQSIGNYMAILPAFSQKLYDTVQSFLEKLN

YTGMANFDIKYDPRDGEYKFFEINLRQGRSSFYVTLNGYNLAKWYVDDYVEDNLKDKP

TVYGNKDGANYMLWLGVPKKIFKEYAYDNGSKRLAEKLIDEGHYGTTVFYDKDRSFKR
WLLMRYMFHNYYARYKKYYEVNKGQYFEEEAKKLKKQALRDGQQENHENEI"
CDS 259770..260159
/locus_tag="OCPHGMPK_00239"
/inference="ab initio prediction:Prodigal:2.6"
/codon_start=1
/transl_table=11
/product="hypothetical protein"

/translation="MIDWGLMALCIVTMLLGFFELYRTFRFYKWDKKTKEMPTAPYVI

YFGTFFSGVLIVVSAMFMMGNTSLTLPKIFYIILGIILVVVAVLMYRRGHQMAKKLGK
DDSNIAVWQTYLISTVILITGLINFLR"
CDS 260389..262599
/gene="nrdD"
/locus_tag="OCPHGMPK_00240"
/EC_number="1.17.4.2"
/inference="ab initio prediction:Prodigal:2.6"
/inference="similar to AA sequence:UniProtKB:P28903"
/codon_start=1
/transl_table=11
/product="Anaerobic ribonucleoside-triphosphate
reductase"

/translation="MLQQDAQIKSITQMTLPETAIKRDGSIYPFALYKIEMVLDNLHL

TEHEADILPSILKKLNGVKQTSTKDIADAFVQTLTELGFDDEANAYVDYRREDEANFE

KQTETTNRLDRLVHHDPTIVNENANKDSRVFSTQRDLTAGVVGKTIGLTMMPEHIAKA

HLRGDIHWHDLDYTPLSPLTNCCLIDFKEMLSHGFTIGNANIESPHSIETATAQMSQI

IANVASSQYGGCSADRVDEVLAPYAEKNYHKNINEAREFFDDEEKIKAFAIKRTKKDI

YDAMQALEYEINTLFSSQGQTPFTTLGFGLGTSWIEREIQKAILKIRIEGLGKDKRTA

IFPKLVFTLKKGLNLHPGDPNYDIKELALECSTKRMYPDIVSYDMIKKITGSFKAPMG

CRSFLQGWKDPETGKEVNSGRMNLGVVTLNLPRIAMESKGDKDLFWQIFREKVQTAHE

ALQIKAKRCTEAVPDNAPILYEYGAFGKRLNAEDNVNDLFKNGRCTVSLGYIGLYEVG

TVFYGPNWEHNEEAHQFTINIVKELHDYCAKWEKEDPNHYHYSVYSTPSESLTDRFCR

LDTKKFGKVKDITDKEYYTNSFHYDVRKHPTPFEKLAFEAPYPFYAAGGFIHYCEYPN

LKQNPKALEAVWDWAYDKVGYLGTNTPIDQCYKCGFKGEFKATAKGFECPQCGNHDPE

TCDCVKRTCGYLGNPLKRPMVHGRHEEIVHRVKHLNLGMEEKMAKDEVRKNDEY"
     CDS   262599..263312
                     /gene="nrdG"
                     /locus_tag="OCPHGMPK_00241"
                     /EC_number="1.97.1.-"
                     /inference="ab initio prediction:Prodigal:2.6"
                     /inference="similar to AA sequence:UniProtKB:P0A9N8"
                     /codon_start=1
                     /transl_table=11
                     /product="Anaerobic ribonucleoside-triphosphate
                     reductase-activating protein"

/translation="MPEKDKNSQEGPDVRGNLIKVNVGGDTIFVDSNQYKPQVEHAKK

LKQQHRKPKNPKPQEWISEEYSKHKIADYKPFNFVDGEGVRCSLYVSGCLFDCPGCYN

LAAQNFNYGRPYTQELEDKIIEDMSQSYVQGLTLLGGEPFLNTWVCLKLINRVRKEFG

HTKDIWSWSGYTWDELQKETPDKKEMLSKIDILVDGRFMDDLKDLTLQFRGSSNQRII
                     DVQKSLKAKKVIIWDKLVR"
     CDS   263368..263961
                     /locus_tag="OCPHGMPK_00242"
                     /inference="ab initio prediction:Prodigal:2.6"
                     /codon_start=1
                     /transl_table=11
                     /product="hypothetical protein"

/translation="MQEKNLKLVDKIMTALQKVEDPELLVDVVNLGLIYGIDIEGDHA

TIKMTLTIVGCPLSTYLQNAIEKAVLSVPEIKTCDVKLVWYPVWSPERMTTAAKQQLG

MLDNEQALDQEEEIEDTEEKQKIVDFSVPIKKLAEEYPDFVQIMYDCGFTRIKIPGLL
                     STVGRVMTIPLGAQAMKIDLNKIKQAFENKGYKVIDK"
     CDS   263958..265154
                     /locus_tag="OCPHGMPK_00243"

/inference="ab initio prediction:Prodigal:2.6"
/codon_start=1
/transl_table=11
/product="hypothetical protein"

/translation="MKELAKQRQEAILKILNFIQNGGELEEAKKMFQAAFDQVDVAEI

TAAERELIAQGLDPRKIQYLCNVHADVFKGNIKENKESPEFETPGHPVHTFKLENIVI

KSLINDALLPDLAKWEAGSSDALPKLKQELKDLAKIHYHYARKETSMFPLMTKYGITA

PPKVMWGVDDKIRKLIGQANMLVNQKNVDPSEVASVIKSAAHEVLEMIFKEEKIMLPM

LDEVANEEDWGNVKNEEEQIGYTLIQKPMNWKPKIKPKAAGPISLDKLSSLALNFAEG

SLNLEQLSAILDLLPFAIAFVDENDKVAYFGGGVSIFPHSKNAIGNSVYSCHLPESVP

RVKKIFDDFHQGKKDKYEFWFEPRHMGRYLYLQYLAVRKDGKYLGCLEVAQDVTEIRS
WTEEKR"
CDS 265504..274044
/locus_tag="OCPHGMPK_00244"
/inference="ab initio prediction:Prodigal:2.6"
/codon_start=1
/transl_table=11
/product="hypothetical protein"

/translation="MLFNKKDFDSKQRFSIRKLSVGVCSVLLSTLFLTFNEGQTVHAS

TDVNAADATTTSVSSNTTNNSSDNNTDKKDDSKVEASSLATAEKHAPIPIAAEIKSNK

SEDQTNSASAETDTQVSKSKQAQSQTTINIVKATATQASTSSKDSSDTTSSKDNSAQN

TVEKNRSTSTSKYTPVLTSETSVSTKTNQSNSNSATQEKSNIKPVTPHADSTEHIAPE

TYTMDSDSPAYVVAGSDLTNLASKFISNADALTQSGATFSWDGNVPTATQQDAIDGNT

ISGTIRVTYKDGTSTTIDIESYVEARSQLQPSTFYYVNRVNDTPDFNTADANGNDFST

ILNNTYVYDPTAFSYKILGTLDTSSLGIHWANVQVTDNNTFAGDPDAPKVIGGLYVVK

VPYVVQGLKLRDDIPTDDNGNPVINAQLATMPKSTSTTPTTPEVAFDPAQGNSTNFPA

GLWGQYFYQDYALAYALGIKVQVAEDDLVNNGKWKNKPTDLVNTKTNHFAITFNKLSN

ATQQEVDVNYVSAPHAEDMYIYNATGKTYMTGRPENANKIKELLADGYMNDKVWVTLA

DGTKVYASELEYIENRNNFVVGGARSGKYNINFNASFNQKNSAGQPLIFSNNIFSAAS

KEFNDELNKWYQKTNNPTVANYAAPTATTWDKVSTNGISLAPTIKASDDPHVQTLTNG

PVTFDSEALEKLVKNPSTGAEVKKVQGTWIVNMTPNPILDNNTKWPEGTKLTWAGNDG

STKLTFDKAGESKTGNVTITLPSGSSYTVKDITVISKANVIAKSETVDYGTTLTAADL

VTNKNVFPEGTTYQFVDNSEPTWGTAGSYNNVKITATYKDASGASVTTPVANCAVAIN

DSRSIKVLVGSDVPSVDSVLNLPSTWEEHTASWTTSPNTNSTNEGVITIHYPASNLDQ

QIKVYVTVIPKTTAVDGQNFFTNGEKYDGTTGSIANGNNQGAILTQANGQAIDYTAYS

GTNTAGTQTSYNTKTTSYTPTYSLSGLQTNSDGSLVSGAQTATVRVSVPKGTLGAKVD

TDGNYYYEVNAKVNITQPVTFEFVDEYKNDQVVGQTYSQEFIPGVATNLNFNMAMPDG

YELGNGASIPSQYTLTAFSQTTLVVQVPIHQKMHFYITFHDEDSNTNLGTVDVSGSAA

AGNGGYYPNINTKLTFPAGANAGDYYSVSTSGVPDGVQLLGSYGVPLTDPKADWTTPN

YQWENTFSVLKALTGATMTINLKHKIQETQEQQTRKPTVNYIKAKVNADGTYTPDGSA

FTSAVLDVYYSRTKSKDLVTKKVTYTPWLWDTKQGQNGYHVESGKWTSLPQQWAAVVA

DVPTLDGYTAATITDKSGQLANQFVFPTWNNSDQDTSDINKESIAYTANAPIYEARPV

HTVLYIPNETQTRTVTENYVYADAGDLTGEEVFSPYKLEIFYKKTGSLNVSTNTITYS

GWSWDTDAGDSDTPGFHVISGQGNWTGLGSNGQPSGSSLGINVPTKDGYTAIISAYPA

AIYSSTVFGNPGVNSGTGYTNDTDSTWYWRNSLTTYYVPNSKLSKTVTRTINITQPGK

ETETTVQKSTITRTAHLNVNDSGIAYNDWGTDSMYWYALNVPKVGGYTMTVTKTVNGV

TTPIQLVNGQIPAETVNGTSQDTTINITWGAVATATLTGTGTSTYNGQAITNAELNNA

LKVNVTGPTAGAGTYTLQAGDVEFSTDGTNWTTALPINAGNYQVRLTAQGKNGIIGKY

GNNSIIWTQNGQSTITSNATYMINKLADNAVMANAQSGNYEMTYNGAAPSTIDPSKFV

FTTTVNGQTVTLDATGLNSSDFTWADGNAPVNVGTYKVVLTDAGFAKLNANNPNFDLT

NSGNGTFTINQANASATLSGSASREYNGQAVSVDDLNSTSTGNNITLTLHYPKDGNAN

YSTTVKLNTGYFTWNTPNGQAPVNANDQPYTISLNSDAIEGIIKQAVGSGQNGVSNVK

FANNAISGSASYKITPLETAAELSNITDGNYTKVYDAQATNQIDASKLHITANLGGQN

VDLDTTGLTGSDYQWVDASGTALTYNPKNVGTYYIKLSDTAFAKLQAQNPNFQLTSND

GLGVYTITPTTANITFGGTNSRVYNGQGTTTAEINSNGQIKIHFTFPGSVSDASYTLK

DRDYYLVDQAGAGYDGAQIINVGTYTIKLTQQGMQNVIDTIHQLSGSGDSGYNINVDS

ATQLGNLTSTFNIVPSQNMISVSGTQTETYNGSPINVVYNADGTNSVKVSIAQTSGNT

TGAVASLTDVTLDSGDFAIVNGPAENAGSYQVKLTDAGLAKIQNAVGDNYHISIDPNA

FGTLVIKKYKASAVFSGDPSYTYTGTPVSADDYLGKYSITLNEPNNPTYNLVAGDIEF

NVDGNWTSQAPVKVGTYQVRLSQSGWNHIKAINSDNVEWAATASAGKGSYTIDQAKVT

ADLSGNNSMIYNGSHVTTADLYTEGSTIKVAINGTGIANLPKSFTLEDGDYTWSTSDN

QSPVNVGNYTIKLTQAGINKIQSQIDQVVGAGNVVLTTTADDGGSASFEIKQAVAANV

QLYGRENTIYNGNPVSFDPTKTETANNFGFHNVENLTVPTFTASDFTWYDGQGNKLSS

APTNAGTYYLKLNTQGEKAFADANPNYTFEENGKSTISGEIPYVVTPAELTIGVSGSA

SKVFNDKNAEITQEQINNGDIKLVWGNSTTEPTDLGKFTLTPSDLEVVDANGKHAIHA

NAGIGADGQPVKGNPYKVQLTADAIKRIKQLSGAANYKISQSAKSGEYFIYEHKAELT

LTGNQTTVYGTAMPFSPSKYQLAFTNWLSTDLPVPNLTWKDNKLLNNDNDTGLTWTVG
DLYVEGYPDGGVPTNVGT"
CDS 274237..280248
/locus_tag="OCPHGMPK_00245"
/inference="ab initio prediction:Prodigal:2.6"
/codon_start=1
/transl_table=11
/product="hypothetical protein"

/translation="MKYGESTNINVGDYSISITAPVHNETVDNNAEQIYTNVTLAAGD

LEFVTVPTNVGTYQVKLSAQGLQKLKDLTGSSNYDWTQAADAKANFFVEQMPVTIAVS

GQSSVTYGTQDWLNAIKVNPSGYVLTITTENGAALSYHTVDGDLVFNQTPGNVGEYQV

ELSAQGLANIEEKLGTNYSYPQTAADVTAKGTFTVKQGEVTVTLNGSDGKTYNAVPTL

SSGLNLDKYNVTYSATVYSADGKAQTLTLTANDLQIIGDATNVGTYQVKLSEIGQEKL

KALTGNQGANYKWAFNTNADYVVKAATASAELSGSNQKTFDGTAVTTAEVNSNGQILV

HLTYPGSNVQSTYTLQDGDYIWETEDGQIISAPTNTGTYTIKLNKQAILVHLQAALNQ

QAGLGDNDQSNVTVSADKLSGQASFKINPQTLTDVTISAPDQSKTYDAQAADLDVNGI

TITANGIVANNPLVNPGISASDFTWYDETGNKLESAPVDVGTYQARLNASTLAELQNT

NPNYQFSSVAGLINYTINPAPATAKISGSADRDYNAQTTSVSDVMNNIKWKATGLVTD

QELNLNGLTANSYAWYSKDADGNYVAMTGNPVNAGTYYLRLTKDAIAQVKADNSNYNF

TSVDGEFTYTINVVNGTATLRGSSSKTYDGQAATTAEVNSTNGDIIVNFAFPGSSAQS

TYALQAGDYIWENKDGQEISAPTNAGTYTIKLSADGITNLQNAINQYAGQGNVTLDAQ

DLLGAAVYTIKQKALNVILGNNSTGTDGKTYDGQPAVINTQAENFGVFTTSGLVNGED

LNAANLTNDDYEWVDANGKAISAPTNAGTYYISLTANGLKKLQADNPNYAISESGQFT

YVISPAEENVTISGSQESTSTSIDNANFTVHAPAGITVPTGMTYEFATGVPSESGVYV

IKLTPESIAALEKTNPNYKLNISSDARFTLDATLSIEFEDTQDGNKQVGKTITKTGVA

NSTINDLKLVVPDNYELAPDQELPTSYTFGKTLNQNLYIKLVHKLNELNPTDPSTNPD

PTNKNWFRENGLVKDVTRTINYEGLSTAQLAQIPETQKAQTVEFTRTAKYDLVTGKIV

VNSEGSWTAVDGKDIFAGFTPFAFAGYTAAPAKVEQVKVTGNDKNSQITVTYTANNQT

GKISYVDSDGKEVGQTTISGKTGETVKVTPEAPTGWQIVSGQDIPETVVATPTGVPTV

VVKVTHSTITVTPETPEKDIPTGPVPGDPSKNYEKMESLTASPTRTIVVTDPSGRTTK

VTQIVNFTRTATFDEVTGEITYSDWTSSEPAEWSEYTAPEVAGYTATQMVKSQAVSAE

TKNETVSISYTANNQTGKISYVDSQGNEVGQTALTGKTDQNVAVTPEAPTGWIIVAGQ

DIPKSVTATATGIPTVTVRVEHSTTIVTPETPEKDIPTGPVPGDPSKDYEKMESLTAS

PTRTIVVSDPSGKTTEVTQTVNFTRMATFDEVTGEITYSNWASSEPAEWSEYTAPEVA

GYTATSSVSAKPVTAKTADETVSISYTANNQTGKISYVDGDGKEVGQTTISGQTGETV

NVTPQVPSGWKIVPGQDIPKTITISENGIPTVTVKVEHGTVIVTPDTPEKDIPTGPVP

GDLSKNYEKMESLTATPTRTIVVTDPSGKTTKVTQTVNFTRTATFDEVTGRVTYSDWK

LQNSNASNHAAQWDSYTPHVITHYVPSVAEVPAKVVNSHTANSQVEITYAPASESQVI

RYVDQNGKEISTQIVSGKYGLDTTFTAQLPNNWQAANTIPTSIKIGENGGLTTIIVEA

KTEKVQQSKTVTETIHYHTANGKQLFADKEMEVNFFRTGVKNLVTGEITWNNNWNKDKE

SLNEVLSPKVSGYTASPAKVAAQTVTPNSEDLVFNVIYTKNSQTYPTIPENKPNKPQE

ENVSKQETKTQDKLIHEYGYKKRADGRLVDHTGHVYPASSKVKENGAIYSEKGELLSV
GSRRKHELPQTGLHDNSLIAAIGSLLAGISIFGLLGGRKKKDDDK"
CDS 280360..281577
/gene="mdtH"
/locus_tag="OCPHGMPK_00246"
/inference="ab initio prediction:Prodigal:2.6"
/inference="protein motif:HAMAP:MF_01529"
/codon_start=1
/transl_table=11
/product="Multidrug resistance protein MdtH"

/translation="MHGKQNIQPDDEIKLHWLLLGELFTWIGASFIWPLTSVYLNKRL

HVSLAMIGIVLLFNCLANMLGSFVAGWAYDHFNPYYLIIAGAGLDALVLFGMAANHTW

PIYWVWMTLTGLLGGWNGALINSIATSIKSKPGRYVFNTIYFAQNLGVVLGTLIVGYI

YDYSVTILFIIAASLFVIVCINSIINYRPIIKFHLERKAQQNTNSKNKITPMPSANLK

LTIGFFTTLAVIWLMYMNWESNLSVYMVSLGIPFHLYSLLWTINASIIVIVQAFLSRF

PNTFKNLFHQVVFGVTMFAISFVTLIFAKDYAHFVFSMVTLTLGESTAMPAMPAYVND

LSPVESKGKYQGLTISTSAIGRAFGPLFGGLVIDKFGYINFFIVAAAGIFMMLAIIVP
LHAQLKDKLKIFR"
CDS 281696..282691
/gene="lacD"
/locus_tag="OCPHGMPK_00247"
/EC_number="4.1.2.40"
/inference="ab initio prediction:Prodigal:2.6"
/inference="similar to AA sequence:UniProtKB:P0A011"
/codon_start=1
/transl_table=11
/product="Tagatose 1,6-diphosphate aldolase"

/translation="MRKISPEVAKHMDNLSNEAGVISALAIDQRGSLKRMLAEAANKP

ADETTIVDFKEAVSKELTPYASAILTDPEYGLPATKVRDKNCGLLLSYEKTGYDTTEP

GRMCDLIADQSALRIKNDGADAVKFLLYYDPDESDEINDKKKAFVERVGAETKANGLP

FFLELLTYDGKMDDVKSEEYAKVKPEKVFKTIEEFSKPQYDVTVLKVEIPFNIKYVEG

FNGDNNVVYTQEEAKKLLKKQSDLTDLPYIFLSAGVTSEEFIAEIKMAEEADADFNGV

LCGRATWKPAIKPFAAEGEKQGREWLSTEGKKNIENLNDALKGAKSWKDKLEVED"
CDS 282859..285762
/gene="smc_1"
/locus_tag="OCPHGMPK_00248"
/inference="ab initio prediction:Prodigal:2.6"
/inference="protein motif:HAMAP:MF_01894"
/codon_start=1
/transl_table=11
/product="Chromosome partition protein Smc"

/translation="MKGLKYTSLAVAALTATILASTNNGKVQAASTNENDTVTVAKSD

QENAQAAVNEAQNSVNNAQAAVDSKKSALTDAQAKAKAPDNAYSAQQAKVNDSQKNLS

AKQAIAKQAQDKLNSVTEISKKATPENIQKAQKSIAAQQDAIKNAKQNVSDANVDLNK

AQAQLATAKQKQADAQNVVNAKESAKQTADKNVAKAEADVKNSGLDEAKQDVEKVQTT

LNDIKKTNANNESILTTNKSALTENNQKIATVNNKIATANQAVVDAQNNVNTKQQALA

DAKKALKDLQDQVAKDQASGAAGFFKSIADNKDNSESLREDARTAYDIVTGKPNAYQD

ITVRWVNQAVELGQEKDSTSFSNMEKALGYYEHWMTYRDKYGLSHPSISLTAVAIAML

SSDFQHYSDEFNHPILLTAKYGPFYEDEEDISAGDVNPIDNWMSEKEDIDNYIKDHPD

AAAYSFESSHPLTEDDWARDVDFWSNKPVDIGHYTSMIKPDANYVGLAGNEYEIEPFM

DNADSMDEIEFDPINGMDFNSYQNLVHSYLQNIKQEDQINTLKANVETASQNLTAAQN

AVQAAQNNVKSLQANLAELNNGSTKINKAIADTQDALAKGRENLEFEQKQLDQANKNL

AAVQAKVGAKAKALDDAKAAQAKAADALAQAQNVLAGATAAVKSAQTTVDNAQGNVNA

KNKDLKAAQASLESLKQTLSSLENAKANLAAAQAALTTANNDVLTADKDVKAQELILS

SLGEAKGKSDAQVASAEKDLEKAESDLANEQSKLAAAKAKLAELNKKANNNTGKSDSK

PEINNQKPAKPATKPGSESVNTEKPEPDFSGNNKSDSKKEQNSTDNVKSDVKSENKSN

KTKEESKSSSSVKIVDNGDDSAASVVLTGSDTAVKVFGEKTVNGTTYYKIGANHWVKA

DKAHVVSIGGQATTKHLPQTGAKNEIIAAISGLTVASVGIASAMGMSRKKKNN"

```
     CDS             complement(285856..286614)
                     /gene="glvR"
                     /locus_tag="OCPHGMPK_00249"
                     /inference="ab initio prediction:Prodigal:2.6"
                     /inference="similar to AA sequence:UniProtKB:P54717"
                     /codon_start=1
                     /transl_table=11
                     /product="HTH-type transcriptional regulator GlvR"
```

/translation="MQNNDLSNAELHLWEIVQNNPQKIAKMSIVKLSQFAHVSTATIV

RTMQKMGYSGYTSYRESLKLQDRSNSNFDVLNDADDKIKSVITKNEIEMNNTLHNLSY

SNIEDSISMTKQAKVVYIFARGLSESIGQEIMVKLQLTGKYVEFHADPNIIKTASKRI

KQDALVIFISLNGNTPELVDAAKSLSHHDVPIITFTTNPNGQLASLSTLTFMGYMSKT

NYFPDYEVRSRLPLQIMTRIFSDAYSVRTGFARQ"

```
     CDS             286739..287665
                     /gene="lacC_1"
                     /locus_tag="OCPHGMPK_00250"
                     /EC_number="2.7.1.144"
                     /inference="ab initio prediction:Prodigal:2.6"
                     /inference="similar to AA sequence:UniProtKB:Q833W9"
                     /codon_start=1
                     /transl_table=11
                     /product="Tagatose-6-phosphate kinase"
```

/translation="MIYTITLNPAIDLVIITKKLESNTVNRTENFELQPNGKGVNVSF

ILKKIGIKNMATGIGGGFTLDYITAGLEEKGIKTKFLKVKEPTRINVFTRVLDQNVEY

KEVNPGPEVGQEVQDKFLKYLKDTLKADDTLIISGSFSRGIKAEYLVEIAKITAEKKV

KLVIDSSSKVVLDTLEYQPYLLKPNDQELASFFDLNEKLDQKKIIELARKLISAGCQN

VLVSLGENGAALINKDHAYFGNAPKIQALNTAGAGDTMLGTFIGEKEKDKSDTAALKS

```
                     AIAAGSDTASRSGLTDFKLEKYLNEIQVSEIK"
     CDS             287684..289615
                     /gene="fruA_1"
                     /locus_tag="OCPHGMPK_00251"
                     /inference="ab initio prediction:Prodigal:2.6"
                     /inference="similar to AA sequence:UniProtKB:P71012"
                     /codon_start=1
                     /transl_table=11
                     /product="PTS system fructose-specific EIIABC
component"

/translation="MAQYDLVGATGCATGVAHTFMAEEALEKAAQKLGYKIKIETHGQ

TGVEHELTPEEIKEAKGVVIASDVDVDPDRFAGKRVVIVPVAKGIKEPETLIREALSL

DTPIYKPGQTAKSSTGKSSAGAEKLGTKIYTSLMNGVSHMLPFVVAGGVLIAVSFFWG

IYSADPKSSQYNQFAYMLNTIGSTTMGLMVPILGAFIAEALAKRSGLVVGFAAGMITS

VGGGGFLGAIIGGYLAAVVVLLLQKLMKPLPDKEFRGLKSIFLLPVLGVFITGAIMFW

LNGPIKAINTGMMSWLKGFENSSPILLGIIIGVMCASDFGGPINKAAYVTGTALLAQG

NYYFMAGVSAACIAPPLATGFAVLLNKKAYSKNERTAGYVNFLLGSTHITEGAIPFAA

KHPLWNIPAFMVGSAIAAALTYVSRIQVPAPHGGFIILPLVNKPFLWVLWIVVGALVS

GILLALIAGRFAKTEPQFEDGPDIDVFGEEENVNKPVAEKETTDYNPKDILDTQNIKI

NVDVPDKDSALKYLANLAVKNGLASDSEEVYDKYLAREKESPTGMTDGFAIPHAQSTA

IKQSAMLVLKLKNPIDWNSLDGQKIDTVISFLIPAKDSKTHLQYLSNTAKLLTHKDFI
                     EKLKQANTPEGIKKLFDKE"
     CDS             complement(289633..290568)
                     /locus_tag="OCPHGMPK_00252"
                     /inference="ab initio prediction:Prodigal:2.6"
                     /codon_start=1
                     /transl_table=11
                     /product="hypothetical protein"

/translation="MKRNNLLLQTYSKKLFSLTSILFTVFLCIFSNNIVYAEDTNINS

KIQSSSTHERSVQKLYAKVYDQDTDKYLGRFLLATQVGYTNSPQRMFDADGINPVHID

GYAFVGNPSAMIPVNWKNKSGVTEIKWTMVKTNSETYKKTQQAIDNIKNNENSNKTAN

PKLNEDGLQVDDEGNVNLHDLYHSYMSFLGHPLPSDRAGYNPSSHALYLFQKYLGYDP

NKTKEENYKHSTNYQSSLNKSTSSYSNASRSNSSKKTTHNSSTKKATRNIKHSTKKDK
                     KKPKLNSSLKPTLLGMSLGLIGFLIFLIIKRLKLK"
     CDS             290730..292679
                     /gene="pepO_2"
                     /locus_tag="OCPHGMPK_00253"
                     /EC_number="3.4.24.-"
```

                     /inference="ab initio prediction:Prodigal:2.6"
                     /inference="similar to AA sequence:UniProtKB:Q07744"
                     /codon_start=1
                     /transl_table=11
                     /product="Neutral endopeptidase"

/translation="MALFNVRGGAGDITKPDLNARPQDNLYLAVNSEWIEKAKIPSDR

SRISSFDGIDLNIEKSLMQDFADFADGKKKLPEVPNFEKAVELYKIANNFDKRNEDGA

KPIKADLEEITSLRNLADLNLRAADFYKNAFDLPISVSVDADMKDTKVHVVYFGGPGL

FLPDTTTYDNPAAADLLKVLQDQSENLLKMAGVSEADAKKYVEDALKFDKKLSKVLKS

TEEWADYPAMYNPMSLAEFEAKFDSFKIDNFLTSLFAQKPEKVIVTEPRFLDHVEELI

NEDNFDEIKGWMVVKFINGVAAYLSQDFREAAFPFSQALSGQPELSSGTKQAYRLANG

MFSEVVGVYYGRTYFGAEAKADVEDMIHKMIDVYEKRISENDWLSEDTKKKAIVKLRA

LILKIGYPEKIEKIYDRLQVTPESEGGSLYSNESAAAVESVKYNLEKLNEPVDRTVWL

MPGNLVNACYDPQRNDITFPAAILQKPFYDLKQSRSLNYGGIGVVIAHEISHAFDNNG

AKFDEFGNLKNWWTDEDFAEFKKRTQDEIDLFNGIEYGPVTLNGKQIVSENIADQGGL

TAAVEANKGENGNMKEVFENFARVWANKQLPESIKTQVSVDVHAPGPERANVQSQCQE
                     EFYKAFDVKPEDGMWLDPEKRVVIW"
     CDS   292824..294545
                     /locus_tag="OCPHGMPK_00254"
                     /inference="ab initio prediction:Prodigal:2.6"
                     /codon_start=1
                     /transl_table=11
                     /product="hypothetical protein"

/translation="MLEKIKTNKFCQFIWPYLLIALATTIVISPQLISHTILASDDTA

DTLFHYSRFYDAGMQLKTGIFSIFQTNFTFQQSGRIINAIYGPLFAYFNGILVLIAGN

WFNYQVILAYVISLLGAGSMYYLLQKVGINKLLATVMGIIYINIGMIPAFINRSSFNG

WGQALMPLVVLCGVRMIQDREKPINWIQLMLVMSLLVQVHVLSTLMAALLLLPFFIYS

LIINQNSKKIWIPFIKAVVGTIVLSANVIGAFLVLMPNNHLAANTEFDLSRGGLRPHV

LWHNEYGTAFAYILPIFVLLIIAQFVYVVMHYKKDKLNTFVTIWGTVLLGLSSFVFPW

GLVQSIFPSLKSYFQFPFRLTVVAYPLILLGMTLTVNHLLKQGVKPRAVGISLVVVLL

LEAALPNIITNHIFTVACGKDAQVQTVAKASNSDQMLKILKWHYLPDYLPTKDKSIDQ

NAAYLYKINVVDQYQKFNHKVAKNGDLILSWKGKKDKDIVLPVVLYKQSELTVNSKKF

TGAKNVIGNPIISQKSGLNRAVLHFIAPAWFYVVSAISLIACLSVLVFYLWKKFINNK

NNKIKVA"
CDS 294605..294736
/locus_tag="OCPHGMPK_00255"
/inference="ab initio prediction:Prodigal:2.6"
/codon_start=1
/transl_table=11
/product="hypothetical protein"

/translation="MVLLTININLFFDYYMAIAVLLCYDLIVIIETAFYGFRGGKIS"
CDS 294733..296391
/gene="pckA"
/locus_tag="OCPHGMPK_00256"
/EC_number="4.1.1.49"
/inference="ab initio prediction:Prodigal:2.6"
/inference="similar to AA sequence:UniProtKB:Q5SLL5"
/codon_start=1
/transl_table=11
/product="Phosphoenolpyruvate carboxykinase (ATP)"

/translation="MSTQERYSHDELRKANKKFSRIRSTIESAFYGNNVHEVTSVAEA

YNLAKKQSGVIETDLPILHTKELGLPQGAKQLVYNHGKILGRTASARHFVDDPKEDPE

ALAGNLREDIYKGHDQKFLKATVLVGLDPSFTVKAHIMMPEDQAFNLLSYILNFHFFD

EEAEKIYKNSKVYDEGDIYFYFDPNTQDEDYPKGFGVFDAPHNCAAVFGLRYFGELKK

GTLTLAWAMAHRNGYTACHGGEKSFHFKDKDDKVFAFYGLSGSGKSTLTHEMYDGKYD

ITVLHDDAFIISREDGSSVALEPSYFDKTHDYPAGHHETKYYTTIMNCAVTQDEDGKK

VIVTEDLRNNNGRVIKTRYTSPHRVDYEDAPITALFWIMKDGSLPPILKVDDPVLATT

MGLTLATKRTSAENLPKGFDMNTLVIEPFADPFRAYPVSGDYSDFKELFTKRGASCYI

LNTDAFMGKDIPKEVTKKLVEDLANDSIKDSDWKPFGNFKGVSYLPIEGYEVHLEDPE
YQKTLAKRMQDRLDWLNKYDEEHPTQPIQEEAKKTLEGIIKELN"
CDS complement(296485..298536)
/gene="kup_1"
/locus_tag="OCPHGMPK_00257"
/inference="ab initio prediction:Prodigal:2.6"
/inference="protein motif:HAMAP:MF_01522"
/codon_start=1
/transl_table=11
/product="Low affinity potassium transport system
protein
kup"

/translation="MNEKTRKNISAAGLLIAIGIVYGDIGTSPLYVMKSIVAGNGGIA

HVNRDFIVGSISLVLWTVTLLTTLQTVFIALKATNHGEGGIFALYTLVRKRAKWLVLP

ALIGGAAILADGTLTPAVTVTTAIEGLKGIEFGHGNVPVSTQGAVIAITVVILLILFS

IQRMGTSIIGKAFGPIMFIWFTFLGVMGLMNMMGDLSILQALNPYYAIKLLFSPYNKV

GIFILGSIFLATTGAEALYSDVGHVGKGNIIGSWPYVFVCLSLNYFGQGVWILNNPNY

NAGNGDFNPFFEIIPQNIRLAAIVLATIAAVIASQALITGSFTLVAEASGLKFLPRMN

IMYPSTKKGQIYIPSVNKMICIATIAIVLFFQTSAHMEAAYGLSITISMLMTTILLYE

WLAMKGIRTIWNWIFLIFFGFLDIMFMLASLSKFTHGGYVSLIIAGFIGIIMYVWFYG

NKIRDKRESRNAYVRLDEYVDMLTNLSHDEDYPTFATNLVYMAKVKYNKFIKREILYS

ILDKRPKRARAYWFVTVNVTNEPFTAEYAVNTYGTKNVINVQLFLGFKQQTSVNVYLR

QIVHDLIADGTIESQPQEYTTTPGRDVGDFSFVIVNDVISPQTQLRSYEKFLVEARVW

LQNLSSNPASWFGLDYADTVIERVPLILGDQRRQHITRIAPKKFEDIKKRLRAEGELK
E"
CDS complement(298688..300685)
/gene="kup_2"
/locus_tag="OCPHGMPK_00258"
/inference="ab initio prediction:Prodigal:2.6"
/inference="protein motif:HAMAP:MF_01522"
/codon_start=1
/transl_table=11
/product="Low affinity potassium transport system
protein
kup"

/translation="MNSNLRKKVTLAGLLVSIGIVYGDIGTSPLYVMKAIVNENGGIA

HVSREYIVGSISLILWTITLLTTVKYVLIALKATNHGEGGIFSLYALVRKKAKWLVIP

ALVGGAALLADGTLTPAVTVTTAIEGLKNMKFGNDIPVPNQNSVIMITIVILLFLFSI

QRMGTSVIGKTFGPIMLVWFTFLGLTGIMNLSHDWSLLEALNPLLAIKILFSPANKVG

VLILGAVFLATTGAEALYSDVGHVGKGNIMGSWPYVFICLALNYLGQGVWILENPNYH

AGATDFNPFFEALPSQWKFFAIILATLAAIIASQALITGSFTLVSEASGLKFLPRMKI

IYPSTEQGQLFIPSINKMLCAATIGIVFLFRTSEHMEAAYGLAITVTMLMTTVLLFEY

LSLRKVNFSLRLIFLLLFGAIETMFLISSLAKFLHGGYVTVIIAAFIGAIMYVWYFGN

KVRDKREAENAYVRLDEYTTMLSNLSHDESIPLYATNLVYMAKVKYNKFIKRDMLYSI

LDKRPKRAHAYWFVTVNVTNEPFTAEYAVNTYGTKNVINVQLYLGFKQQKVNVYLRQ

IVHDLIKDGTIESQPQEYTTTPGRDVGDFKFVIVNDVISPQTQLSSYEKWLVESRVWL
QNLSSNPAVWFGLEYADTVIERVPLILGSQDVKSIHRTKLR"
CDS complement(300829..301248)
/locus_tag="OCPHGMPK_00259"

```
                     /inference="ab initio prediction:Prodigal:2.6"
                     /codon_start=1
                     /transl_table=11
                     /product="hypothetical protein"

/translation="MTEDKKNTEETQEKEEFEVVMPEANRVEMPATEFKEQPDYLKTF

ANFYISKFDESDLEIMDVYDGNHDVIEINTYLTNNMAFSRQNLVKHVLNIHAERFMDM
                     LNNIQKQTGVDPQNMKTYEDWNKWYTDRRNEIKQTLS"
     CDS             complement(301335..302372)
                     /locus_tag="OCPHGMPK_00260"
                     /inference="ab initio prediction:Prodigal:2.6"
                     /codon_start=1
                     /transl_table=11
                     /product="hypothetical protein"

/translation="MKRSKIGVAIMAFFITLFAVGCSNSSNSSSNNGSDKYTTYMSQG

KDAASSQKYEAAEEKFEDAHDVKATSESKAYADQAGAMKDAQEEIREYEFKDALGELD

KAANKTNGYSVMTKQAAKLYKTINKVQDHITHDINPLYKQAKLSYRDGNYQATVESCD

QILDLSYINGKYYKKVKKNVKHLRKVANAKLNGEDVNDDIDTDNLDANDDDEATTSHK

STSSKSNSSNSSNTNTSSSSNQSNSNTANTQNNSAASKQSSHATTSNSNANNSSNTNS

SSNSNSGMMIDGQTVTGRVIAQTRNQLSSLGVDTSSWSDQKVVNFMRSAANNGHTTID
                     SYTKQDVENFQ"
     CDS             302559..302768
                     /locus_tag="OCPHGMPK_00261"
                     /inference="ab initio prediction:Prodigal:2.6"
                     /codon_start=1
                     /transl_table=11
                     /product="hypothetical protein"

/translation="MEFVLKDQSVQFTFAGEKYSNGKKNRILKNVKKQASAEDVMKVG
                     KALSKLQKDDGIKSARLISYSDIQA"
     CDS             302814..303053
                     /locus_tag="OCPHGMPK_00262"
                     /inference="ab initio prediction:Prodigal:2.6"
                     /codon_start=1
                     /transl_table=11
                     /product="hypothetical protein"

/translation="MTTTKTLRLTFKNAKNKKVNLSLPDAAENLTEEEVKTAMNEICE
                     ANLFVKEEVDQYQAPLSAQYVERTVTSVFDDSEKN"
     CDS             303257..305140
                     /locus_tag="OCPHGMPK_00263"
                     /EC_number="3.2.1.17"
                     /inference="ab initio prediction:Prodigal:2.6"
                     /inference="similar to AA sequence:UniProtKB:P39046"
                     /codon_start=1
                     /transl_table=11
                     /product="Muramidase-2"
```

/translation="MKKRTFTGIATAALITTAGISVTNNLKPENPLKTGEGTVQAATY

QQEFLNKAIPAATTASSKYGTYTSVMLAQAAVESAWGQSGLAQSPNNNLFGIKGSYNG

QSVNMNTGEYGSNGYYTTNAGFRKYPSYTESFEDNGSLLRNQMGNYYSGTWVENSKNY

AQATQNGLQGKYATAPNYAQTLNSVIAANGFDKYDPVTQVVNENRTVAQTTPIMSAPV

DASVGTQVGTARTGQNVNVTKYITYNNGVKRAYIGTGWINALAFSPITTNTTTKQNTA

ANTNNQASQAVKTPVAQTQQVKSQAPAAPVKTATVKVKSAAEVKTPVQATTLNVKTEN

KAAQPVKQLAVSLAVAPKKVEVKKNVVKAEPAKTTPVKTETTKTENKEVKTATPVVKT

TDTVKKVETPAVKPVESVKKESTPVVKTAPVTVTKEAAKTTEAPVVKPNKVEVKEIKT

TPVTTSAKTVVKPVQSYQSAVTTAANNNYGTQWISTNTAPKVASTVLIKVTKTVDVLS

APDGQKLDQQVEAGSEFVVIASKYYNGNLYYEISNGKWIMAKYTTQEAQITAKSGVLT

INSKPDYGVPVWRVPGQDQIAGKFLKDGSSWRYFRVANVQGQTWYDLGGNQWVSAKSV
LVR"
CDS 305197..307269
/locus_tag="OCPHGMPK_00264"
/inference="ab initio prediction:Prodigal:2.6"
/codon_start=1
/transl_table=11
/product="hypothetical protein"

/translation="MRSTNKKFISALACASAMTALAIADPMGITKTHQVVQAATDNVP

AKSAGVDVSSWQGTNLTQQAKSGAKFAVVKVSESTNYQNPHAQGQINNAEQNNMMPMG

YHYAHFGADSNRAVQEGNYAVNSAQQAGLPQGSYLAADWEQDAHNNTNGGREASANAI

TSFMDTVHNGGYNPMLYSSEWLLKAKVDTNKVTQKYPNSLWVASYKTMGRQDQPDYNY

FPSMNNVAIWQYTQNWRGQNVDGNVNVLPLSNKSNNASQANTNTLNVHTNGQSSSQAP

SNPFVNTSNKAVNTNWVKQEGTFTTGGAINLRTGASTNSSIIAQLPANSEVKYDAYST

RGNYTWLRQPRANNQYGYLVGRANGQDWGTFKVAPVTNHVTKPTTNTVKPANKPSQPV

HVNTNNNVNNNWTKQNGVFITGGAINLRTGASTNSKVIALLPTNTEIKYDAYRTEGQY

TWLRQPRANGQYGYLVGRDNGRAWGTFKAGSAVATKPATNNKPAQKPATNNSDWTKQN

GVFITGGAINLRIGASTNSKVIALLPTNTEIKYDAYRTTGQYTWLRQPRANGQYGYLV

GRNNGNAWGTFKAGSAAASKPATNNKPAQNTNKPSQPVHTNTSNNVNSNWTKQNGVFI

TGGAINLRTGASTNSKVIALLPTNTEIKYDAYRTEGQYTWLRQPRANGQYGYLVGRLN
NQAWGTYR"

```
     CDS             307372..308187
                     /gene="lacC_2"
                     /locus_tag="OCPHGMPK_00265"
                     /EC_number="2.7.1.144"
                     /inference="ab initio prediction:Prodigal:2.6"
                     /inference="similar to AA sequence:UniProtKB:Q833W9"
                     /codon_start=1
                     /transl_table=11
                     /product="Tagatose-6-phosphate kinase"

/translation="MIYTVTLDPSESVVGKGINISLVLKKLGIESIATGIVNHKNVEQ

VAEELDKAEIENQFIEQKRGIKITAKNQQKLLDYLKVLKAGDILVIAGGFAKGIDPVY

LTDLAKVADKKAAGLVVDVPYENVLDILPMNPLLIKPNETELKHWFEKDNQKVTTKEL

INMAHDLVVKGAQHVLLSLGANGAAIVNMMDALMAHAPEIEEVVDSTGSGDALLGTFL

AGMLKGYMPVKNLSDAIAAGSDTARSDWLTDFRTTPALQKQVIARRIAFEEAE"
     CDS             308277..310103
                     /gene="kefC"
                     /locus_tag="OCPHGMPK_00266"
                     /inference="ab initio prediction:Prodigal:2.6"
                     /inference="similar to AA sequence:UniProtKB:P03819"
                     /codon_start=1
                     /transl_table=11
                     /product="Glutathione-regulated potassium-efflux
system
                     protein KefC"

/translation="MDLSLFIVSLAALVIPIIMARFKINAIPTAVAEIVTGIILGKSL

LNTIKITSSVSLLSNLGVILLMFLSGMEINFDLFKKKDLPDTKQSSINPARIAVFSFA

MITAMALILAEALKLIGLFSDVMLAMIIFMTVALGVVIATLKEKEILSRPIGQTILLT

AVLGEVIPLLLLTIYASINGGNAGKLWLIILLFLVAILLLKRFKQPYQWFNRISKSTT

QLDIRLAFFLIFTLVTVAERVGAENILGAFLAGMVMKLLEPSEATMDKLTSIGYGFFI

PIFFITTGVKLDLKTLITNPNALMLIPVLVLFLLLSKLPIFLVYVRSFNKRNSIAGTF

LIMTTITIVLPTLEVARKLNAITETQSDAFILAAVVVCILGPILFNSIFKLTKEDKIK

QRVVIMGTNVMTVPVAQELHDNWYDVLLVTHKQENYDTYKSKVANLKLISSLEETCLE

KAGVFNCDILVAGFIEDELNRKIARMAKEHGVQRVIASQERPKPETIKNLTNEKIEIY

NLFNVQTSVLRALIESPSILRILTDTKNGLFEVTVRNHKYAGQKLMNLDFIEQMTVSR
                     IWRNGTWLVPHGNTIIETGDHLIFTAKGEDAERVREELGRKN"
     CDS             complement(310143..311105)
                     /gene="afr_1"
                     /locus_tag="OCPHGMPK_00267"
                     /EC_number="1.1.1.292"
```

```
                     /inference="ab initio prediction:Prodigal:2.6"
                     /inference="similar to AA sequence:UniProtKB:Q2I8V6"
                     /codon_start=1
                     /transl_table=11
                     /product="1,5-anhydro-D-fructose reductase"

/translation="MKLGIIGSGKIVHDFLTIADQISDLELTALATTKRSHQIGLDLQ

KKYQITKLYDNNADLFNDPDVDTVYVAVPNSLHFSISKEALEAGKNVICEKPFVATVD

EARELKVIADQKKLIIIEAITNIYLENFKFIEENLDKIAPIHVVNLNYTQYSSRYDAF

LAGDIQPAFDPKKDGGALMDLGIYNLHIIIKLFGKPESINYFPTMQKNIDTSGILHLS

YSDKQASSIAAKDSFSPNVSTIEGEKGALIIYGHPNEIPKVGMQLKGQEVKVINNNKY
                     SHRMVAEFIEFTKIIGQHKFKEADHAFVHSLNTLSVLQEAKSQR"
     CDS             complement(311117..311899)
                     /locus_tag="OCPHGMPK_00268"
                     /inference="ab initio prediction:Prodigal:2.6"
                     /codon_start=1
                     /transl_table=11
                     /product="hypothetical protein"

/translation="MQKNKAKNKKIIFPNNQGGKIAALILAIIILVVIIWGAIFAFGK

PSPKAALTKAADTAIKTARVDVKTTNSKGKMYSNYVVGPNNTIHITMKSIPKSDTNSE

LWSNDHYVYHRDNNEAWNYIKQNAIFNEVYSGYKKLYTAHNFKQFSDDAFKQLKLKSN

GFNGYVIAYHGDDQDVIKSMQKATSVASTSDPQSSNVKRIELRVKVNRQKELTDLYYK
                     VTYYSKKEGTLSLHLYDVNQVKKLKIPTSVTKNAKKLNIKSN"
     CDS             complement(311921..312748)
                     /locus_tag="OCPHGMPK_00269"
                     /inference="ab initio prediction:Prodigal:2.6"
                     /codon_start=1
                     /transl_table=11
                     /product="hypothetical protein"

/translation="MKKKFGLLLLLGAILVSITTACSKNTSKSKTNPEPSASSLINAK

FNSSLASGHFTQTINSDEMSQKSKSEGLFKDKGDVTNLTYTLTQKKKSQTEQMWLTKK

DMYLLLEQNKGHWIKNSVNADTFDPDQVKERFDPATFKKINKALAKKATVQKKNGNYE

ISFNGTDSNLWNAVNPLVIDAMNTPGSQNMQVARLVKSAQVQNLKITYLVDPATKKIE

SMTFKAQYTAGGKYNFTWNLTYDQLGQHADLAVPADIQKNAIDAEEIKKAQAEQNNQ"
     CDS             complement(312832..313254)
                     /locus_tag="OCPHGMPK_00270"
                     /inference="ab initio prediction:Prodigal:2.6"
                     /codon_start=1
                     /transl_table=11
                     /product="hypothetical protein"
```

/translation="MDNINLDGGHLSLEQLNAIFRTIPQEMDVLDENDRIVWSSMNKN

RLFKRTEKDIGKTVFEVHPGHSQRHVKEVLNQMHAGNRKNISIMITKDEQPINISFYS
LHNEDGKYIGCIEVTQAVKNLQVKGSKLRNILNVLKKH"
CDS 313414..314106
/gene="gpmA_1"
/locus_tag="OCPHGMPK_00271"
/EC_number="5.4.2.11"
/inference="ab initio prediction:Prodigal:2.6"
/inference="similar to AA sequence:UniProtKB:Q5XB88"
/codon_start=1
/transl_table=11
/product="2,3-bisphosphoglycerate-dependent
phosphoglycerate mutase"

/translation="MSKLVLIRHGQSEWNLSNQFTGWVDVNLSEKGVEEAKKAGRLIK

EHGLEFDQAYTSLLTRAIKTLHYALEESDQLWIPETKTWRLNERHYGALQGLNKKATA

EKYGDEQVHIWRRSYDVLPPAIDDDNEFSQAHDRRYANLDPHIVPKAENLHVCLDRVM

PFWEDHIAPDLLDGKNVIIAAHGNSLRALTKYIENISDDDIMNLEMKTGEPVVYTFDD
KLDVVNKEKLDD"
CDS 314289..315365
/locus_tag="OCPHGMPK_00272"
/inference="ab initio prediction:Prodigal:2.6"
/codon_start=1
/transl_table=11
/product="hypothetical protein"

/translation="MDTSYREGFFLHKFMKKHDISLRYIDQLALFKMRGVDGISLSVH

RAEEEKRKLLNKQLKTLSTIGYYNLKNYANPYFDSSEGKYKNLSFDKLVARYYRDKHL

KQAVLHAIEDIETTLNTKIASVLGERYGAYGYTDFRLWCQRNSVNKFLRGKYMDKFAI

QKEQINFLKELQNIVRKSYSEDLQDYIKNTEKIYPPIWLVMNELTLGTSIHLYKLMSK

SNQRRISSYFGCKTDELVSWLESINLIRNICCHNGILADFKLKTRAKVPKEYKSSSEL

KTVLLKIKPEVYTNRLAFQLCIIVKLMSKINNNYTYRDLRNSVKKLLDESTPATYYGF
QNQEAIQKLFKVKILKDNSSLILY"
CDS complement(315515..315772)
/gene="rpsN2"
/locus_tag="OCPHGMPK_00273"
/inference="ab initio prediction:Prodigal:2.6"
/inference="similar to AA sequence:UniProtKB:O31587"
/codon_start=1
/transl_table=11
/product="Alternate 30S ribosomal protein S14"

/translation="MAKKSKIVKAAKQRELIKKYYELKEAGDVEALAKLPLDAHPTHY
HNRDLHDGRPHGYMRKFGMSRLRFKELAHKGQLPGVRKASW"

```
     CDS             315948..316385
                     /locus_tag="OCPHGMPK_00274"
                     /inference="ab initio prediction:Prodigal:2.6"
                     /codon_start=1
                     /transl_table=11
                     /product="hypothetical protein"

/translation="MGIFKRILHNEDLRQLIIYVLIGVLGLGVDFGIFALLTHFKMQV

EVANFISSSCGLINNFFWNSFLNFKVHDKLLIRFVSYYLVGQITTLFTTLCLFIFVTQ
                     LGYNQLIVKAVSTFIATLIQFVINKLLTFRKIKTTKSKVDVRK"
     CDS             316405..317337
                     /gene="yfdH"
                     /locus_tag="OCPHGMPK_00275"
                     /EC_number="2.4.1.-"
                     /inference="ab initio prediction:Prodigal:2.6"
                     /inference="similar to AA sequence:UniProtKB:P77293"
                     /codon_start=1
                     /transl_table=11
                     /product="Prophage bactoprenol glucosyl transferase"

/translation="MKKLSIIVPCYNEEESVPLFYPAVNKVMDTIPDLEPEYWFINDG

SKDNTLKEIKELRKKDPEHVHFVSFSRNFGKESALYAGLQAATGDYVVVMDVDLQDPP

KFLPQMYDLIKTGEYDCIGTRRVDRTGEAKFKSFLSDMFYKVVNKISDTEIVPGARDY

RMMTRQMVNAVLDMPEYNRFSKGIFSWVGFKTKYLDYHNVERVAGESDWNTWKLFKYA

MDGIADFSQAPLNLAVWIGTGSFVLSIIGLIIVIIRRALYPGSSIFGWASMVCIILLL
                     GGLQLLCIGILGKYIGRVYIQVKNRPIYIIKEKK"
     CDS             317440..318153
                     /locus_tag="OCPHGMPK_00276"
                     /inference="ab initio prediction:Prodigal:2.6"
                     /codon_start=1
                     /transl_table=11
                     /product="hypothetical protein"

/translation="MKNFSQNTWIKIALSILGVLAAILVYVPNYAAVDQGLKGRCAIF

FIIAVVLLWLPMELKSSIFALNFYYELGLILIIVFLMTNQMAMNFVIGLVIAFLLLTW

IGMVIFKNNLVMKQKNSELLIIRLVMMLALAYLAYISGFAALMGAEIFTVQGQTWLLI

LGFLLCVYYLVLAGSIWQPWFRRWTSWIMILAAVVLHMIFASATSLNIIFLPIIGEII
                     MLISVWRSNRILMKKKRIS"
     CDS             318226..319089
                     /gene="glcU_1"
                     /locus_tag="OCPHGMPK_00277"
                     /inference="ab initio prediction:Prodigal:2.6"
                     /inference="similar to AA sequence:UniProtKB:P40420"
                     /codon_start=1
                     /transl_table=11
                     /product="Glucose uptake protein GlcU"
```

/translation="MKYVYLFLPAIGWGLMPLVIASVKNSTVYNQIVGTVAASFIFGA

IVMAIMHPAMSWSLFLLSALGGACWVIGQVGQYISYEKIGVSETMPISTGLQLIGVPL

VGVLAFGEWASPQAKFYGFIGILVLIIGVVLTSLTDRGTSEGNKSNQISTIILLVLTS

LGYITSSSIPKVLHGSSISIFFGQTFGMLVAVFIYTLVTKNLHVWKEKSTVQSGGAGI

LYAIAALAYILSVQDNGVNMAFVISQLCVVISTLGGLIFLHEKKTRNGLIFTIAGLVL
IIGGAMLTTLF"
CDS 319374..320255
/gene="galM_1"
/locus_tag="OCPHGMPK_00278"
/EC_number="5.1.3.3"
/inference="ab initio prediction:Prodigal:2.6"
/inference="similar to AA sequence:UniProtKB:P39840"
/codon_start=1
/transl_table=11
/product="Aldose 1-epimerase"

/translation="MLTIENNQLKVEINEVGAQLTHVVDKTTNADYIWNGSEWERHAP

ILFPAIGRSNDNKYILNGKTYEMKQHGFARDYPWTVVDKGDDRVSLTLTENEETLAVY

PFHFSLMVTYTLEANQLKVEFLVKNNSEETMPFGLGFHPGFNVPIAGDELKFSDYELT

LSPEVTKLTQFQIDPIPFRNGKVIPVPNAQKGTLPLSYSEFDDGLIIINNPGLTGIEL

SSAKSEHKISLTLEDFPYVALWTMTDPEAKFLCMEPFAGLPDIKSSELTDWMEKEGNN
FLAPDESSHFSTTITLE"
CDS 320388..321497
/gene="brpA"
/locus_tag="OCPHGMPK_00279"
/inference="ab initio prediction:Prodigal:2.6"
/inference="similar to AA sequence:UniProtKB:Q8DVR0"
/codon_start=1
/transl_table=11
/product="Biofilm regulatory protein A"

/translation="MDNKPKEPVLSRVELNKEKHSKKKPIRIIIAVILVLVLGLGAYA

GSVYLKAKNAFDKTYDPKTAVKQDSFSGKEPFNILLLGTDTGAFGRKEVRGNSDTMIL

VTVNPAKKKLSLMSIPRDTMARMIGTENFSVHKINAAYNIDGAKMAMKTTSKVLNVPI

KYYISMNMGGMRKIVDGVGGVTVTPPLTFTYDGYTFTKGKKVHLNGSQALAYSRMRYD

DPRGDYGRQLRQREVIMSVLEHAMSFSTLKNLDSILGSVSTSLRTNLTFDSMVKISKN

YRKCTQNMSSDYLHGVGAMIGDASYQVMSDRELQRTSNIVRNDLGLDSMNIDNNETYQ
NSMNSQFDWTSGDSNQVYYIYDPYTDELWNGDRAY"
CDS 321497..322117
/gene="plsY_1"

/locus_tag="OCPHGMPK_00280"
/EC_number="2.3.1.-"
/inference="ab initio prediction:Prodigal:2.6"
/inference="similar to AA sequence:UniProtKB:P0A4Q0"
/codon_start=1
/transl_table=11
/product="Glycerol-3-phosphate acyltransferase"

/translation="MNELISLVIGYFLGNILFAMIVAKIFLHKDPTKYGSGNPGTANI

GAVFGKKWGILTCIGDLAKTLAALLIVYFLYHGNRLDLSFAGIGVVLGHSFPFWNHFK

GGKGVAVTALWIVFFDWRAGLIALLIGLFLVIIMKNLTIPPLVYMLGFSIFTWLNFGW
EQGLIFLIATLIMIFQFRKDIVDFFTGHGKRVDVLVTIKKKLGIYK"
CDS 322114..325062
/locus_tag="OCPHGMPK_00281"
/inference="ab initio prediction:Prodigal:2.6"
/codon_start=1
/transl_table=11
/product="hypothetical protein"

/translation="MKSALRKTIQWILLLCLLLGILIQTLGFWNYNPTSVSTKTRIGM

VISLIQLIVVVWYGMSYGNKEYSFKEAVKNWLEGVVTLIIFYLVFVISLPQFFSAWNL

WGIFFPVLTSTSALFSGIIISLFFQPFIFRLQEKLNTKQNVLLLTAITVLIFALSAGN

SLLTSYSIFGLYLAVPFAWGMLISKIKASKKVVLGLVVATIILLPAVYYLTIKLMPIQ

TPQGFIFSQMNMSWNTSLLMAPSSPLMILFVVAGALLFRSSMLGVSHRLFSILIPAII

FGTTSYGMSLWKEKLQLLLAPVSKKVTVLLILSLLVASFIINFVFVKFLLSNKHVQRF

LNKFDENNLDGLIKLLEAGVDFLKRHSKSIILFAFLMFLSVIGFYTVRDIQSASDFWA

ALVFIFTSKFGTLVLSSIFLFAIYEIFYVITTRFWVSASIPTVLALGIAIADGIKMDL

REEPVYPNEISEIVNWKTLIPMIGVQTLIYILVGIALLIAIIVYLELKHPHNLRRKKK

SWLVLIGSLLILITPVWFNDENSAIYYISKGFDNNPDFRNPPDSTANNGAVLTFLDFI

KVPIMEKVDGYSEHAIKQITKKYEKEAIAINKTRKNKLSDQTIVFNLSESFVDPKEFP

GVKISDNVRDPMKYIRSLMSQTTSGKMLSAGYGGGTGNMEYESLTGFNMGNFSSALTP

YTQVTSRYNFYPTIGMNFPYSSAIHPFNGTYYGRIDNYRRFKFNKFAYLGSKYKIYDK

KSLGTSPYLSDETAYQNGLRQIKSRKNGQFINLISMQNHMPYGDYYSPNEYKDNVSGS

SLADDNVKTSFAAYTKGVEYTDKAVKKFIKEIDEINKPITLVFYGDHYPSIIDQSLLS

KYPIKMHSITYFIYSNKYAREHGAKNKIVPDKYVATSSFIPMALEQTNSKVTAYQALL

TRIYKDLPAMTINYSSSDGFELVDQNGKKVSEKKLTKKQKELLKDYQLIQYDMSAGKG

```
                     YTLDVKGFYK"
     CDS             325321..326583
                     /gene="tyrS"
                     /locus_tag="OCPHGMPK_00282"
                     /EC_number="6.1.1.1"
                     /inference="ab initio prediction:Prodigal:2.6"
                     /inference="similar to AA sequence:UniProtKB:A6QHR2"
                     /codon_start=1
                     /transl_table=11
                     /product="Tyrosine--tRNA ligase"

/translation="MAKFNILEDLKWRGAINQETDEEGLRKYLAEHDDLALYCGTDPT

GDSLHIGHLIPFMILKRFQMAGYHPVILIGGGTGSIGDPSGRKTERVLQTAEQVKHNE

EKLTAQMKKLFGTENFEIKNNADWLGKLNLIDFLRDYGKFFQVNNMINKDVVASRLEN

GISFTEFSYQILQAIDFYHLNKDNGVQLQIGGSDQWGNITAGIDLIHKLEGQDHPAFG

LTIPLMLKADGTKFGKSAGGAVWLDPEKTSPYEFYQFWINQDDRDVVKYLKYFTFLSR

EEIEDLAEKTEKEPWKRAAQKRLAEEVTKFVHGEEGLKEAQMITEALFSGNVKSLSVP

QIEQALKNAPSAEATHEAKNIVEFLVETKIEPSKRQAREDVKNGAIYVNGERQNDIDF
                     IIEPDSDFDGKYVIIRKGKRKYTLVKIK"
     tRNA            326653..326725
                     /locus_tag="OCPHGMPK_00283"
                     /product="tRNA-Thr"
                     /inference="COORDINATES:profile:Aragorn:1.2"
                     /note="tRNA-Thr(ggt)"
     CDS             326852..328165
                     /gene="pepE_1"
                     /locus_tag="OCPHGMPK_00284"
                     /EC_number="3.4.22.-"
                     /inference="ab initio prediction:Prodigal:2.6"
                     /inference="similar to AA sequence:UniProtKB:P94870"
                     /codon_start=1
                     /transl_table=11
                     /product="Aminopeptidase E"

/translation="MAHKLTLEEIEKFQQDYQQNKQNKIAELAVVNNGVQKASFNSEG

IRDLNRTFSIEIPTDNVTDQKQSGRCWLFAALNVLRHKFAKQYHAKNFTFSQSYLFFW

DRIERANIFFNHILETADKPVGDRTVHFYLQAPDTDGGQWHMAISLIRKYGLVPTYAQ

DESFTANNTAAFNQALNMKLREDGLVLRKLAQEGKNDEIEQKRQEFLSEVYRMAVIAF

GQPVQKFDLEFKDDDGKYKLDQNLSPLDFFHNYFEDDLDDYVVLFNAPDHEYDKLYAF

PFEDNVEGGSPIRFLNTKIENLKEAAIKQLKDGETIWFGCDVGKQSDRQKGILAADLY

ETDTVFNIETKLSKKERLETGASGSTHAMTFVGVDVVDGKPRQWKVENSWGTKVGEKG
                     YFVMDDKWFDEYLFKVVVKKQYLPEKLVKIAEGEATPVPCWDSMA"
```

```
     CDS             328182..328835
                     /locus_tag="OCPHGMPK_00285"
                     /inference="ab initio prediction:Prodigal:2.6"
                     /codon_start=1
                     /transl_table=11
                     /product="hypothetical protein"

/translation="MDIELVKQFTQDHLKGEKTGHDYYHGQRVANLATKMYLSDYPDA

HADSRVVAIIQTGSYLHDTIDEKICDDPSKVIAEIKELLPKVGFTELEAQDILFTIQH

MSFSANIEHHYKLPLSGQYVQDADRIESLGAIGIARAFTYGGKHGNKIYDPEIKPEKL

VSHDQYRNHVETTINHFYEKLFDLEGLMNTPAAKKEAHRRTEYMREFVQEFMDEWNV"
     CDS             328899..329282
                     /locus_tag="OCPHGMPK_00286"
                     /inference="ab initio prediction:Prodigal:2.6"
                     /codon_start=1
                     /transl_table=11
                     /product="hypothetical protein"

/translation="MSYIADYTNSYDIHGQRITITAPARFDENTGKVIADLELDDHAA

KLALEKYRRKFGIISPTDLKNLRHHWNFSKEKFAEVLGWNVATIALYEAGAVPSISDN
                     RLLKILIKNPKLMEKFIEDSEKEEL"
     CDS             complement(329340..330650)
                     /gene="pepE_2"
                     /locus_tag="OCPHGMPK_00287"
                     /EC_number="3.4.22.-"
                     /inference="ab initio prediction:Prodigal:2.6"
                     /inference="similar to AA sequence:UniProtKB:P94870"
                     /codon_start=1
                     /transl_table=11
                     /product="Aminopeptidase E"

/translation="MSHELTLQEIDQFRNDFDNSRNEVVSRAAMRSGVLEASFNPSVT

NRLNDVFSVEVETDNVTNQMQSGRCWLFATLNTLRHDFGKKYKAKNFTLSQAYNFFWD

KIERANIFYDAIIDSADKPLDDRTVKAYMNFAGSDGGQWAMAASLVKKYGVVPTNAMP

ESFNTNHTAGLADALARKERKDALVLRKLVQAGKTEEVEKKRKEFLSEIYRMTAIAVG

EPPKTFDLEYRDDDKKLHLDKNLTPVEFFNKYWDVNFDDYVCLTNAPDHEYGKLYSLP

FEDNVNGGLPITFLNVPIEYLKEAAVKQLKDGESVWFGNDVLKEMDRKTGYLDTELYK

TDELFDVDTYMTKAERLATGEGEVSHAMTLVGVDLDKGEIRKWKVENSWSEKSGRKGY
                     FTMSDKWFDEFVYEVVVRKEFLTDDQKKLAESKPTPLPAWDSLA"
     CDS             330812..331432
                     /locus_tag="OCPHGMPK_00288"
                     /inference="ab initio prediction:Prodigal:2.6"
                     /codon_start=1
                     /transl_table=11
```

/product="hypothetical protein"

/translation="MKKNIFVSGITLMTMLLAVGCSNSANQSSTNNEAKSSKTSHKKS

HKAKKHTTKKDKTDVDTDESETTNSDKKSDKSSENSSNTTSKKTNKNKSTDSNNSSDS

TGSTNSNNASNSAASNTQTNGNSQNASSSHNASDAMFNKMVSDTIKEHGYSSNYGPDN
FTLIDGGNGQYSLAEKSTGTIVGHYSVKNGELYKQDILTGNDVKVK"
CDS 331432..331893
/gene="greA_1"
/locus_tag="OCPHGMPK_00289"
/inference="ab initio prediction:Prodigal:2.6"
/inference="similar to AA sequence:UniProtKB:P80240"
/codon_start=1
/transl_table=11
/product="Transcription elongation factor GreA"

/translation="MVYYQKMTPEGYQEIKDEIARLKKDRPRRIKILQEARSMGDLSE

NSEYTTAKMELGHLQSRLRYLDKQLRYSEIIQKDKSGTIDLGSNVKLLFEGDDDAEEY
RIVGRMEADLAKGKISFDSPLGQALMKQKAGATVTVEAPAGSYDVKIIAVN"
CDS complement(331940..332389)
/locus_tag="OCPHGMPK_00290"
/inference="ab initio prediction:Prodigal:2.6"
/inference="similar to AA sequence:UniProtKB:P80485"
/codon_start=1
/transl_table=11
/product="Acid shock protein"

/translation="MEGSFIMANEMMKNRNNDNMMDQLSDWFSFPKDFFDDSSIKNIM

QSDVAETDKDYIVKVDMPGMDKKDIKVSYKDGILNVSGSRDSFDNLDDKNGNLIHRER
SVGHIQRSYRIPDVDSKEISAKDIDGVLTITLPKLTEEDKENTITIE"
CDS complement(332521..333969)
/gene="bglA_2"
/locus_tag="OCPHGMPK_00291"
/EC_number="3.2.1.86"
/inference="ab initio prediction:Prodigal:2.6"
/inference="similar to AA sequence:UniProtKB:P42973"
/codon_start=1
/transl_table=11
/product="Aryl-phospho-beta-D-glucosidase BglA"

/translation="MTGYTMPKGFLWGGAVAAHQLEGAWNEDGKGMSVADVMTVGSAT

KPREITDGVIPGKNYPNHDAIDFYHHYKGDIKLMAEMGFKAFRTSIAWTRIFPKGNEK

EPNEAGLKFYDDLFDECHKYGIEPVITLSHFEIPYHLVKEYGGFTNRKLIDYFVRFAR

VCFKRYKNKVKYWMTFNEIDNQSSFNNDFLMATNSGILFKNGMGDKEKEAAMYQAAHY

ELVASALAVKEGHKINPDFQIGCMINYSPVRPLTPSSDDVLLADKFEQRRDFFSDVHV

NGEYPNAVEDYIERNGYRPDITEEDKIALKEGTVDYVGFSYYQSTTVSSKKVKPDELT

DLQEAIVENPTLQRSDWGWEIDPEGLRISLNHLTDRYHKPLFIVENGLGAYDKREADG

SVHDPYRIDYLRKHIEQMEKAVVLDGVDLMGYLPWGCIDLVSAGTGQMDKRYGFIYVD
KNDKGEGTLERSKKDSFDWYKKVIESNGKDLD"
CDS 334168..334665
/locus_tag="OCPHGMPK_00292"
/inference="ab initio prediction:Prodigal:2.6"
/codon_start=1
/transl_table=11
/product="hypothetical protein"

/translation="MTKKDQSLEQKKHDMSVRSINFSRYLMIRYFSAAFLFTNLFWLV

FAICYKDIVASIISGLLFVLLLVASVEQVSKWNVKNTDLKFTKLYYQLQLGANVIFAI

GCYLPFGKLLFPFMTTNDVANVIVTILVIGMLGSILILRRISNIQNGRDKYARAIKTF
ESNRQ"
CDS 334688..336040
/gene="gmuC_1"
/locus_tag="OCPHGMPK_00293"
/inference="ab initio prediction:Prodigal:2.6"
/inference="similar to AA sequence:UniProtKB:O05507"
/codon_start=1
/transl_table=11
/product="PTS system oligo-beta-mannoside-specific
EIIC
component"

/translation="MSDTAQPSFKDKMMKILGKFSGSRFVRAIMGAGYSIIAFSIIGS

MFLVLTVLTQVITAKGFVDFYNNTFGRFNNIYTVIYNATMGIIAIYFAGSFAYNYADI

YRKEENLLLDPLNAVFLTLMGLFITVPQLVWKGGNTVFVNILKKDNVIAGGYGVSGSG

LTRIGATGIFTGLIVAWLTVQIYRFCIKHNWRIKMPASVPSGVANSFTALIPGFVIAI

VIALIDVILIVLGTDIFQLLFIPFSFVSNIANTWWGVLIIFFLIHFLWWFGIHGATII

SSFYQAIVLSNMAANASGAHYVFAGEFSNAFVIMGGSGATLGMALWMAFASRSKQLRE

LGKLEAVPAVFNINEPLLFGLPIVYNVRLFVPFLLAPMTCSMVAYAAIATHLVPKIIV

QQPWPIPVGLGGMMATASWQGAVLALVNAVVAFLIWYPFIKHYDNELLKKEQAGAEKA
"
CDS 336116..336814
/gene="yydK"
/locus_tag="OCPHGMPK_00294"
/inference="ab initio prediction:Prodigal:2.6"
/inference="similar to AA sequence:UniProtKB:Q45591"
/codon_start=1
/transl_table=11
/product="putative HTH-type transcriptional
regulator

```
                     YydK"

/translation="MKKYELVADKIKNYINKEQLHHGDKLPTITDLMKKYQVGKSTIL

QAITLLTQRGIVYKVQGSGVFVRPTGQDGYMSLTDNAGFAHVLKKPTTEVIEFSEKKA

PKPISEHLHSDEECYFIKRLRKQDGKPFVLEESYYRKSIVPFMSKEIAEGSIFDYIRE

GLKKEIRFSDKYMRVRKLTADEAKYLELKEGDPCLEVYDTFYLANGTAFDSSKLVYNY
                     KNSKFYDQSSDDII"
     CDS             complement(337395..338825)
                     /gene="bglH_1"
                     /locus_tag="OCPHGMPK_00295"
                     /EC_number="3.2.1.86"
                     /inference="ab initio prediction:Prodigal:2.6"
                     /inference="similar to AA sequence:UniProtKB:P40740"
                     /codon_start=1
                     /transl_table=11
                     /product="Aryl-phospho-beta-D-glucosidase BglH"

/translation="MTKTYSMPKDFYWGGASAANQYEGGYNEGGKGLNAIDVLTNGSA

TEPRKVTWKTNDGKTGVTPMTWGKEFKLPDGAKPALLDNYYYPSHEGTDFYHHYKEDI

KYMAEMGFNMFRLSLNWSRILPNGDDQEPNEEGLAFYDKVFDECAKYGIEPLVTLSHY

ETPLSLINRFGGWKDRKMIDIFVHYADIVMNHYKGKVKYWLTFNEINAMDMAPYMGGG

LIDGSKQNRAQGAHNQFVASSKVVKLAHEIDKNNQVGQMLAYSAYYPYTSDPADQLLV

MKAKQEMLFYSDVQTGGRYPEYRLKEYERDGIKLDDRPEDYELIKEYPADFLSFSCYT

SNVLTTHEADAKANGNMSAGGVKNPYLDYNAWGWATDPDVLRIALNDLWDRYHKPLFI

VENGLGWGDELTEDHKVHDDYRINYLRAQIKSMEEAVNEDGIPLMGYTMWSAIDLVSN
                     GTGEMKKRYGFVYVDRDDMGNGSLKR"
     CDS             complement(338892..339617)
                     /gene="gmuR_1"
                     /locus_tag="OCPHGMPK_00296"
                     /inference="ab initio prediction:Prodigal:2.6"
                     /inference="similar to AA sequence:UniProtKB:O05509"
                     /codon_start=1
                     /transl_table=11
                     /product="HTH-type transcriptional regulator GmuR"

/translation="MTAEAKYLKVARILQKRIEDGVYKTQAPLPDQKTLADELQVSRL

TVKKALDGLQRKGLVYKESGLGTFVLGPVPIQDKFDSPANAFSGLANLLGSDEVTSDI

IEFNVEFPSEDIQHYLKLKSSDPVYNIRRLRRLEGKPLILEHTFMPVHLVPNLTEDIL

HNSIYNYLHHDLKLKFGVAYRKIKAAKADDWDQKYLKAKKDDPILELEQIVWLNNGQP
                     VEYSTSRNRYDERNYVVLETNSF"
     CDS             complement(339617..339943)
```

```
                     /gene="celD"
                     /locus_tag="OCPHGMPK_00297"
                     /EC_number="2.7.1.205"
                     /inference="ab initio prediction:Prodigal:2.6"
                     /inference="similar to AA sequence:UniProtKB:Q45402"
                     /codon_start=1
                     /transl_table=11
                     /product="PTS system cellobiose-specific EIIA
component"

/translation="MAQENSDNMQVVMGIIMQAGNAKAAAMQAIQAAKKGDFDKADEF

IKQANEGLVNAHNVQTDMLTQEAQGNHVKVDLYMVHAQDHLMTAITFIDLAKEVVAVY
                     KKMAEK"
     CDS             complement(340032..340280)
                     /locus_tag="OCPHGMPK_00298"
                     /inference="ab initio prediction:Prodigal:2.6"
                     /codon_start=1
                     /transl_table=11
                     /product="hypothetical protein"

/translation="MNDEVKIVNEFDRDGHHFKIGVNADGQVSIYIDDETKAHHGYHF
                     PGIIQIPKGLELDGKLMLQLPIDCDAAIDQGIQELKQK"
     CDS             complement(340286..340609)
                     /gene="licB"
                     /locus_tag="OCPHGMPK_00299"
                     /EC_number="2.7.1.-"
                     /inference="ab initio prediction:Prodigal:2.6"
                     /inference="similar to AA sequence:UniProtKB:P46318"
                     /codon_start=1
                     /transl_table=11
                     /product="Lichenan-specific phosphotransferase
enzyme IIB
                     component"

/translation="MADKTIMLACSAGMSTSLLVSKMQNAAKEKGKDYKIFATAASGI

QDEIDKEHPDVLMLGPQVQYMEDSVKKITDAAGIPLTVINMQDYGMMNGEHVLETAQE
                     LMGDK"
     CDS             complement(340756..341307)
                     /gene="licC_2"
                     /locus_tag="OCPHGMPK_00300"
                     /inference="ab initio prediction:Prodigal:2.6"
                     /inference="similar to AA sequence:UniProtKB:P46317"
                     /codon_start=1
                     /transl_table=11
                     /product="Lichenan permease IIC component"

/translation="MLQANSFDNAQLYKAGKLTIANGAHVVTNKFYNNFINLTGSGIT

IGLIIFILIAAKSAQLRSIGKVELVPGIFNINEPFLFGLPIVMNPFLAIPFFLTPVVV

AISTYFVIKTGIVPPLNGFACPWTMPAVISGFLIGGWKMAIWQACTLVISTLIYCPFA
                     KKYDNILVKREAAALKKDEAEGK"
```

```
     CDS             complement(341285..342184)
                     /gene="gmuC_2"
                     /locus_tag="OCPHGMPK_00301"
                     /inference="ab initio prediction:Prodigal:2.6"
                     /inference="similar to AA sequence:UniProtKB:O05507"
                     /codon_start=1
                     /transl_table=11
                     /product="PTS system oligo-beta-mannoside-specific
EIIC
                     component"

/translation="MNRKNRALVFSLINTSCPPVMKFVNTKAIQALQNGMIYTLPFIL

IGSIFLILGNIPIKSVADAINASGWGAFFNQAYTTTFSIMAMWASVGIAYIYVKNEGY

EPLAPGLTSLAAFLMLQTLTIDSPLKNAMSKGIDGQMTAKAVTENIDKLPHALQSFLE

SPVTGVFNITWLGGDGMIAAIIVGLLVGWIYSAIMKKGWTIKLPEQVPAAVFNQFTAM

IPSGIILIGTMLIYAGFKLTTGSDFLQWTYQTLQIPLQGISDSLGGAIAIGFLVPFFW
                     FFGVHGGLIVRFSCWSYASSKLI"
     CDS             complement(342188..343567)
                     /gene="bglH_2"
                     /locus_tag="OCPHGMPK_00302"
                     /EC_number="3.2.1.86"
                     /inference="ab initio prediction:Prodigal:2.6"
                     /inference="similar to AA sequence:UniProtKB:P40740"
                     /codon_start=1
                     /transl_table=11
                     /product="Aryl-phospho-beta-D-glucosidase BglH"

/translation="MNKQKMPKDFFWGNSVSSMQTEGAWNEDGKGLSVYDVRPATENT

TDWHTAIDEYHRYDEDLDLMKEMNMNMYRIQISWSRVCPEGDGEFNPKGIDFYDRLIN

AMLKRGIEPMICLYHFDMPLHLAKEYNGFMSRHVVDAFVRFGKKMIDHFSDRVKYWIV

FNEHNLYFQDEVFNISGYEKGNKSLDDMYTIFHHTMISHIRLANYIHEKYDDVKIGGM

LAYQQIYPETSKATDVWAAKQAQEFLNFNIYDADTGRGYSPEVMQYAKDHDINWDITD

EDKEEMEKAQADFLAFSYYSSWTLSADKIPKDAAPNRYMNYGGVENKYLKTNDWGWTI

DPLGFRNAITTMYNHYKIPVFPIENGIGLKETWDGEHMIEDDERIAYHEEHIKAMKDA

MFLDGVKVLGYLGWGLIDIPSSHADMEKRYGAVYVNRSNHDLKDLKRVPKKSFYWFQK
                     VLKDNGDEI"
     CDS             complement(343769..344629)
                     /gene="bglK_1"
                     /locus_tag="OCPHGMPK_00303"
                     /EC_number="2.7.1.85"
                     /inference="ab initio prediction:Prodigal:2.6"
                     /inference="similar to AA sequence:UniProtKB:Q93LQ8"
                     /codon_start=1
```

```
                     /transl_table=11
                     /product="Beta-glucoside kinase"

/translation="MNLIAIDIGGTTIKIATWINKKLKMIFTIDTPNDLETFYNELSN

AVNEIKASNKIDGVAISSPGAVNKKTGVIEGASALPYIHNFKIVPELEKRFGLPVSIE

NDANCAALAELVDGSAKGCSSMAFLVIGTGVGGSIIINNQIWHGAHLYGGEFGFMIID

GQQLSALASPVSMAKRYNEKTGKDFDGKTVFELADTDDLVAQEERGKLIHALATAIYN

IQHSFDPEKIIIGGGISQNQELIPLLNDEISKIRNKLNIATVKPILDICTLKNEANLR
                     GAVADFEKEH"
     CDS             344776..345438
                     /locus_tag="OCPHGMPK_00304"
                     /inference="ab initio prediction:Prodigal:2.6"
                     /codon_start=1
                     /transl_table=11
                     /product="hypothetical protein"

/translation="MNTPSSRQGNLTRYAVYLVLYLIVIGVKKLTLLSDRVNKIGYFF

FLIFALFTLYFYIRQFNREQRFFEKKPSMPLLADYGFIVGVSILVILGRIFISYLQAK

GAVPLMSFQVIYQKGECNLLFWFFIFSIGILIPAMQVYLTTGFFFNYGFRAQNMTSGI

VGIICSGFFFGILNFQTSFALFIINSLYGMLLAWSYLYSQRIMMPIYLAILNGIFMVI
                     LA"
     tRNA            complement(345478..345548)
                     /locus_tag="OCPHGMPK_00305"
                     /product="tRNA-Gly"
                     /inference="COORDINATES:profile:Aragorn:1.2"
                     /note="tRNA-Gly(ccc)"
     CDS             complement(345576..346187)
                     /gene="yedK"
                     /locus_tag="OCPHGMPK_00306"
                     /EC_number="3.4.-.-"
                     /inference="ab initio prediction:Prodigal:2.6"
                     /inference="similar to AA sequence:UniProtKB:P76318"
                     /codon_start=1
                     /transl_table=11
                     /product="Putative SOS response-associated peptidase
YedK"

/translation="MCSHYAMPSLKEIKTYLTTDLKLPLIVPKNIEEKFSAGTQIYPK

SSSLVMLYSNNELKLVEKKWGYPSPFQKNRVLYNARIERFYEEKPSMWDNSFARQRCI

ILANEFWESSKQTYLASNNQTYHEHSSFTAKEEPMTLIAGIYQGDDFSMVTTAANSTV
                     LPVHERMPLVLKPSELRRWLFQNFTSLLNRSEYNLVRHQLPHR"
     CDS             346320..347348
                     /gene="trpS2"
                     /locus_tag="OCPHGMPK_00307"
                     /EC_number="6.1.1.2"
```

```
                     /inference="ab initio prediction:Prodigal:2.6"
                     /inference="similar to AA sequence:UniProtKB:Q9RVD6"
                     /codon_start=1
                     /transl_table=11
                     /product="Tryptophan--tRNA ligase 2"

/translation="MTKEILLTGDRPTGKLHIGHYIGSLKNRVKLQNEGKYEPYIMIA

DTQALTDNARDPEKIKNSLIQVALDYLAVGLDPEKSTIYVQSQIPALFELTAYYMDLV

TVARLERNPTVKTEIKQKNFKDSIPVGFLNYPVSQAADITAFKATVVPVGDDQEPMLE

QTREIVRTFNRVYNTDILVEPKGYFPPKGQGRLPGLDGNAKMSKSLGNAIYLSDDAKT

VQKKVMSMYTDPNHIHVEDPGQVEGNTVFTYLDVFAPDKDKVAELKEQYQKGGLGDVK

IKRYLNKVLEEELGPIRERREKFAQNPDAVYEMLLEGSKKANKVANQTLKEVREAIGL
                     NYFDRLNK"
     CDS             347368..347856
                     /gene="tadA_1"
                     /locus_tag="OCPHGMPK_00308"
                     /EC_number="3.5.4.33"
                     /inference="ab initio prediction:Prodigal:2.6"
                     /inference="protein motif:HAMAP:MF_00972"
                     /codon_start=1
                     /transl_table=11
                     /product="tRNA-specific adenosine deaminase"

/translation="MTRDRIPWKQYFMMQALVIAQRSTCDRALVGSVLVKDDRMIATG

YNGSVSGQPHCDDVGHLMVDGHCVRTIHSEMNALIQCAKNGISTEGTEIYVTYFPCFN

CSKCLVQAGIKKINYYYDYHDNPLALKLLREKGVAIEQVTLDHKYVQSLADRLEKADN
                     EK"
     CDS             347846..348433
                     /locus_tag="OCPHGMPK_00309"
                     /inference="ab initio prediction:Prodigal:2.6"
                     /codon_start=1
                     /transl_table=11
                     /product="hypothetical protein"

/translation="MKNKIIGLVSLLICLLTLTASTKSFVSDPQGLFDNQTKELVNQK

NESYQKTKTKPEIRLLSLKDSNDLNRIQPLKNQIIIAVALQNKKKNVQIIVGKNYQDP

LTQSKCGNIVRYAGNKLRSEKAATFNFGIRLVFNACATLVAQKDKLSYDKNSLTQDEL
                     QKIDHPQSVNLVWGLVIAALISFGLVLFKSFRKKK"
     CDS             348742..349062
                     /locus_tag="OCPHGMPK_00310"
                     /inference="ab initio prediction:Prodigal:2.6"
                     /codon_start=1
                     /transl_table=11
                     /product="hypothetical protein"
```

/translation="MFKINTSGTRNTTDTKLVRRIACEDKFATLQRLKTSISGLQSKD

ARQRLAKYGSNEIIIKHDKTKLHYLLKAFITPFSLVLLFLAIVAIFLNSSQHTLNLSP
AILF"
CDS 349095..349730
/gene="mgtB"
/locus_tag="OCPHGMPK_00311"
/EC_number="7.2.2.14"
/inference="ab initio prediction:Prodigal:2.6"
/inference="similar to AA sequence:UniProtKB:P22036"
/codon_start=1
/transl_table=11
/product="Magnesium-transporting ATPase, P-type 1"

/translation="MQNIRINTNIQNLINRISRTTQVMRDCEPHQLLTRDLTIGDVVL

LKAGDIVPADVKLLKSNDLECSLLGEEDTVKKSATNYTGKNNNLDYANILYAGTIILS

GSGIGVIFATGKETVFGKVVQNLSKINLENHLFNFSMKKLTKIFLTLILVIVPVVLLI
NEIMRGNWENALIFAIAITIGITAEVMPTAIVNNLVKANIDMPRIRATYDN"
CDS 349851..351827
/gene="metG"
/locus_tag="OCPHGMPK_00312"
/EC_number="6.1.1.10"
/inference="ab initio prediction:Prodigal:2.6"
/inference="similar to AA sequence:UniProtKB:P67579"
/codon_start=1
/transl_table=11
/product="Methionine--tRNA ligase"

/translation="MAEKKTFYITTPIYYPSGKLTIGNAYTTIAADTMTRYKKSRGFD

TFFLTGTDEHGLKIEQKAEAQGIKPQEFVDKMAASYKELWKMLDVSYDRFIRTTDKDH

VKAVQKIFEKLLKQGDIYLGEYTGWYSVEDEEYFTESQLAEVYKDDNGNVIGGKAPSG

HEVRLVKEPSYFFRMSKYADRLLQYYKDHPDFILPHSREKEMINNFIKPGLEDLSVTR

TTVDWGIPVPSDPKHVVYVWIDALSNYITALGYGSDDDSLFKKYWPADVHLVGKEIVR

FHTIYWPIMLMALNLPLPKQIFGHGWVLMKDGKMSKSKGNAVYPEMIVKRYGLDALRY

YLMRAIPFGSDGIFTPEDFVERVNFDLANDLGNLLNRTVSMINQYQDGQVAPVAAEQD

EFAQDLIKTANETIAEYQKHMDALHFSQALDSVWKLISRANKYIDETTPWTLNKEGKS

EELSKVMSNLAESLRLVAILIAPVMTQSPVKMFEQLGLDWNNEEQKKLEFGGFDWNVK

VTEKPTPIFPRLKNDVEVKYIKEEMKKAKPKKKTRSQQKEEKHITIDDFDKVKIQVGQ

ILSVEPVKGSSKLLMFKLDFGDGNERQILSGIRKFYPDASELLDKKVLAVTNLKPRKM
LGHESQGMLLSSEKDGQVKLAIVGDEHEVGAELG"
CDS 351827..352597

/gene="dtd3"
/locus_tag="OCPHGMPK_00313"
/EC_number="3.1.1.96"
/inference="ab initio prediction:Prodigal:2.6"
/inference="similar to AA sequence:UniProtKB:P73335"
/codon_start=1
/transl_table=11
/product="D-aminoacyl-tRNA deacylase"

/translation="MKIFDSHTHLNDVPFRGKEEIYLDRARELGVVKSAVAGQDPEFN

ERAVDLSQRFDNLYAIVGYCPDVAKDYDQKSEDKLVEQLKKPKTVALGEIGLDYYWDE

SPRDVQRKVFAQQLDLAHSLKMPVNIHTRDAFEDTYQVLKDSHVGEYGGIIHNFNGDP

DWLKKFLDLGMMVSFSGVVSFTKATDVHASAKVVPWDRFLIETDAPYLTPKPYRGKQN
ETGYVRYVAEAIAKLRDTSVEEVAKHTFENTMRAYGIK"
CDS 352584..353147
/gene="rnmV"
/locus_tag="OCPHGMPK_00314"
/EC_number="3.1.26.8"
/inference="ab initio prediction:Prodigal:2.6"
/inference="similar to AA sequence:UniProtKB:P37547"
/codon_start=1
/transl_table=11
/product="Ribonuclease M5"

/translation="MESNNQKQFNAVVVVEGRDDTKRLKQFFPGIETIETNGSEVSDQ

TLAEIKKLAKTREIIIFTDPDYNGERIRRLVTNAAPNAKQAFITRKEGEPTKRGNSLG

VEHASKEALVRALGDLHEVQAVESDITKEKYDDLGLAASPEARLLREKVGIKLGIGYG
NSKQFLKRLKMFGITYKELKRAVEDVK"
CDS 353137..354027
/gene="rsmA"
/locus_tag="OCPHGMPK_00315"
/EC_number="2.1.1.182"
/inference="ab initio prediction:Prodigal:2.6"
/inference="similar to AA sequence:UniProtKB:P66662"
/codon_start=1
/transl_table=11
/product="Ribosomal RNA small subunit
methyltransferase A"

/translation="MSNSMPIASPVRTQAIVNRYFMHAKKNLGQNFLVDLAAIKGIVE

AADIQPGDQVIEIGPGIGSLTEQLLLAGAKVLAYEVDQDLPEILKNELPQKVAGEELS

NRFKLVMKDVLKANFTKDSDGFLDLNKSVKIVANLPYYITTPIIFNLIKSNLAFSSLT

LMMQKEVAERLVAKPKTKEYGPLTIAVQSRMNVKLAEEVKSTSFMPRPKVDSAVVVLT

PLTEKPDIDDYFFFDHVVKMCFAQRRKTLANNLKSLVKDKDMREKMINDLGLDLRVRP
EELTLNQFVELAHLLKDQQA"

```
     CDS             354104..354343
                     /locus_tag="OCPHGMPK_00316"
                     /inference="ab initio prediction:Prodigal:2.6"
                     /codon_start=1
                     /transl_table=11
                     /product="hypothetical protein"

/translation="MPTTLMAIKHNLDSHLGESLVVVAQAGRKKVTRRKGRLTKTYRA
                     VFVVDLDQDQNNFERVSYSYTDLLTKNIELEFDEM"
     CDS             354464..355300
                     /gene="purR_1"
                     /locus_tag="OCPHGMPK_00317"
                     /inference="ab initio prediction:Prodigal:2.6"
                     /inference="similar to AA sequence:UniProtKB:P37551"
                     /codon_start=1
                     /transl_table=11
                     /product="Pur operon repressor"

/translation="MKRSERLVDMVKYLLARPHTLIALPFFADRYGAAKSSISEDLAI

LRQTLASDQNGILETVAGAAGGVRYIPFVGKKEATNYLHDLADRIEDPDRILPGNFVY

LSDILGSPQDLQQIGQLIATKYAYSNVDYVMTIETKGIAIAQAVSRFLNVPFVMVRRR

PKITEGSTISVNYVASSSERVEKMELAKRLLPEGSNVLIVDDFMKGGGTLTGMEELVK

EFKGTVAGMCVLCETKYASQKVVDDYQSLIKITEVDRTKKLIKVRPGNFLEQTDFNRF
                     PK"
     CDS             355348..356733
                     /gene="glmU"
                     /locus_tag="OCPHGMPK_00318"
                     /inference="ab initio prediction:Prodigal:2.6"
                     /inference="similar to AA sequence:UniProtKB:Q97R46"
                     /codon_start=1
                     /transl_table=11
                     /product="Bifunctional protein GlmU"

/translation="MNKYVVILAAGKGTRMKSQLYKVLHKVCGKTMVEHVVDAAKGTN

PDKIITVVGNGADSVKDVLAGQSDFAFQKQQLGTGDAVLAASDLLANLEGSTLVATGD

TPLFTAETFNNLFKKHEESGNSATVLTAKAPNPFGYGRIIRDEDGNVLRIVEQKDGTP

EELAVDEINTGVFCFDNKELFKALKQVGNDNAQGEYYLTDVLEIMRKAGHKVGAYEMP

DFSESLGVNDRIALAQATKIMQRRINEEHMRNGVSFIDPDTAYIDSDVKIGNDTVIEG

NVVIKGNTEIGSDCYITNSSRIVDSKIGNHVTITSSTLQEAQMDDNTDIGPNSHLRPK

AIIRKGAHIGNFVEIKKAEIGENSKVGHLTYVGDATLGKDINIGCGTIFSNYDGVKKF

HTNVGDHSFIGAGATIIAPVNIADHAFVAADSTITKDVERYDMAIARGRQTNKPDYWH
                     KLPLSKDKEWE"
     CDS             356843..357814
```

/gene="prs_2"
/locus_tag="OCPHGMPK_00319"
/EC_number="2.7.6.1"
/inference="ab initio prediction:Prodigal:2.6"
/inference="similar to AA sequence:UniProtKB:P14193"
/codon_start=1
/transl_table=11
/product="Ribose-phosphate pyrophosphokinase"

/translation="MSQLDKEIKIFALNSNKPLAEKIADKVGVKLGKSDVKRFSDGEI

QINIDESVRGKDVYLVQSTSAPVNDNLMELLIMIDAVKRASARSVNLVMPYYGYARQD

RKTRAREPITAKLVADMLQKAGADRVLSLDLHAPQIQGFFDIPVDNLMGAPLLADYFL

SHELEKDAVVVSPDHGGVTRARKLAEFLKTPIAIVDKRRPKANVAEVMNIIGDVEGKR

AIIIDDMIDTAGTITLASQALMDAGATEVYASATHAVLSGPAIERLNNSPIKKLILTD
SINQPEEKDLSKAEIVSVGPLMGEAIKLIQEHKPVSPLFNTRFQSHN"
CDS 357970..358914
/gene="cytR_1"
/locus_tag="OCPHGMPK_00320"
/inference="ab initio prediction:Prodigal:2.6"
/inference="similar to AA sequence:UniProtKB:P0ACN7"
/codon_start=1
/transl_table=11
/product="HTH-type transcriptional repressor CytR"

/translation="MTTLSDVAKKASVSKMTVSRVINHPQQVTPELRKIVEQAMEQLN

YHPNSVASALAHHRTNVVKLVILEDIDTTEPYYMNLLFGIASGLGKKHYALQLATDSE

VGGGDGYIITGGRANDAKWLDQLNKPFVLFGENRYGYDFIDTDNKLGEQIATQYALDK

NYQSIVFIGIDEKEPFEYSREAGYINTLQKHNMIPKIFRIQNHSSLAEKLIISNWKKF

APNTCFICASDRIAVGVVRAIQRKNGNIPRDFGIIGFDGVFLDQVSNPKLTTIKQNLF
KLGELLAEMILQKIQQGGAQQGEVLIEPELIKSESTRI"
CDS 359018..360676
/gene="malL_1"
/locus_tag="OCPHGMPK_00321"
/EC_number="3.2.1.10"
/inference="ab initio prediction:Prodigal:2.6"
/inference="similar to AA sequence:UniProtKB:P21332"
/codon_start=1
/transl_table=11
/product="Oligo-1,6-glucosidase"

/translation="MRHWYDQAIIYQIYPKSFQDSNGDGIGDLNGIRKRIPYLKELGV

NAVWLNPVFVSPQVDNGYDVSNYFAIDPKMGTMEDMDNLIKEMHEAGIHVIMDFVLNH

TSDQHPWFKDAIKNPDSIYRDYYIFAGQNNQRPNNWGSFFGGSVWEKDPAGTGQFYFH

LFDKRMPDLNWKNPEVRHAMSEISKYWLEKGIDGLRLDAFIHIAKADLRQNFPVNSKA

EEPVVAEPFFANLPQVQKWMRPFCEEIKQDYPDALLLGEAASANVNLAVDYTNKDNHL

MDSVITFRYFTEDQSQVDPSFSAQYQPKSLDLVKFKQNQAVWQQTLSGVSKPTLYWNN

HDMARIATRIAKNDIQAQSLAMLMYLQNGLPIIYYGEELGMKNLEFEDVTQFEDETVK

EFIESARKAGKTSQEALLMVSKTHKLPARGPMPWNNAKNNGFSDGKPWLKGKKVDHAN

AADEIADSHSMFNFYRKLISLKKEALFEDGGYYLLPTSNDSYVYERNLGDQKALVAVS
LSKDAIELDVDPEFKQEKLVAGNYQLKDGKLKLAPYAGVVLTKD"
CDS 360687..362411
/gene="bbmA"
/locus_tag="OCPHGMPK_00322"
/EC_number="3.2.1.-"
/inference="ab initio prediction:Prodigal:2.6"
/inference="similar to AA sequence:UniProtKB:Q9R9H8"
/codon_start=1
/transl_table=11
/product="Intracellular maltogenic amylase"

/translation="MQLAALRHRTESEDCTVIDHSHVKIRLHTAKNDVKKVIVHYIDN

YLPPADAATLEMKKAGSGQVNDYWTATLTAPYHRIKYTFEVMGNDGSHVIFGDRSIEK

FSDEKLREDGLYFKVPYFHDIDRIKTPAWLKDIVWYQIFPERFANGDKSNDPVNVKPW

NPEDHPGREDFYGGDLQGVLDHLDDLQKLGITGLYFCPIFKASSNHKYDTIDYLQVDP

AFGDKDLFAKVVNEAHKRGMKVMLDAVFNHLGDQSMQWQDVVKNGANSRFKDWFHINS

YPVEPYRDPSKGEKNPPYETFAFEKHMPKLNTANPEVQDFLLEIATYWVKYFDIDAWR

LDVANEVDHHFWKRFHDELIKIKPDFYIVGEIWHSARPWLQGDQFTGVMNYPYTLQIE

DHFFKHNLDAKELSEHLTDQLMMYPDIVDQAMMNMLDSHDTARILTLAKGNQDLALQA

IAYEFAQPGIPCIYYGTEMGMSGDNDPDCRKPMDWSKLNGPVWKRVHELVKFRLKHRD

TLNQGDIKLTVTENGLLKVERIGKETINAYYNTSAHDVKIDKKPALSQNYEDGILAPD
GFLVEVKA"
CDS 362565..364835
/gene="malP"
/locus_tag="OCPHGMPK_00323"
/EC_number="2.4.1.8"
/inference="ab initio prediction:Prodigal:2.6"
/inference="similar to AA sequence:UniProtKB:E6ENP7"
/codon_start=1
/transl_table=11
/product="Maltose phosphorylase"

/translation="MKRIFEVDPWKVTTHKFSKEDKRLQESITAIGNDYMGMRGNFEE

```
GYSGDTLQGTYLAGVWFPDKTVVGWWKNGYPKYFGKTPNAPSFIGIGITVNGEKLDLA

KVKFSDFELSLDMHQGLLSRSFVYEGKDVKVKFEFERFLHIIQKEAALIKVKATVLEG

HAKIDFDSTLDGTVVNEDSNYDERFWVPLGEDKDRRTIQVKTKPNPYEVPQFTVLLKQ

ALRHNGEALQGEVSSQEAKLSEKISVELDQDQSYELEKDVIVVTSRDVEEKDQADVAE

NLMSKLQAKSFAENLAEHTATWEKRWETSDVEIAGDAAAQQGIRFNILQLFMTYYGED

ERLNVGPKGFTGEKYGGATYWDTEAFIVPMYLAITKPSVTKALLEYRHNQLPGAYHNA

KEQGLPGALFPMVTFNGIECHNEWEITFEEIHRNADIPHAIAMYTEYTGDDSYVKNEG

MDVLVGTARFWAARVHWSKWRNKYVMHGVTGPNEYENNVNNNWFTSTMARWLLKYTLE

RLPLATKEAQERVHVTEEEKEKWQDIVDKIYLPEDKDLGIFLQQDDFLDKEIRPVSEI

EDQRPINQHWSWDKILRSPFIKQADVLQGIYFFPENYTKEQKEKNFDFYEPLTVHESS

LSPSIHSVLAAELGKKDKAVELYERTARLDLDNYNNDTVDGLHITSMSGSWLAIVQGF

AGMRYDHDQLKFKPFIPDNWNHYSFKINYRGRLIEVYVDHQETKISLLKGENLEILVN
                     DQKVMLKEGETECLKA"
     CDS             364820..365482
                     /gene="yvdM_1"
                     /locus_tag="OCPHGMPK_00324"
                     /EC_number="5.4.2.6"
                     /inference="ab initio prediction:Prodigal:2.6"
                     /inference="similar to AA sequence:UniProtKB:O06995"
                     /codon_start=1
                     /transl_table=11
                     /product="Beta-phosphoglucomutase"

/translation="MLKGLIFDLDGVLTNSAVYHLTAWNNLAKELGINLTDEQLNSLR

GISRMDSLNLILKYGGQENKYSEAEKEKFAAEKNTKFVEQVEKMTPADILPGIPELLS

DAKKQNLKMVIASASKNAPKILTKLGIMDEFDGIVDPATLHRGKPDPEIYEKAQKLLG

LNADEVISFEDAQAGVQSIKSAGQFAVGIGDKEVLKEADYVVPTTKELKLSEIESVFN
                     KK"
     CDS             365503..366606
                     /gene="sugC_1"
                     /locus_tag="OCPHGMPK_00325"
                     /EC_number="3.6.3.-"
                     /inference="ab initio prediction:Prodigal:2.6"
                     /inference="similar to AA sequence:UniProtKB:P9WQI3"
                     /codon_start=1
                     /transl_table=11
                     /product="Trehalose import ATP-binding protein SugC"

/translation="MVEVDLNHIYKKYEGNDKYSVNDFDLHIKDKEFIVFVGPSGCGK
```

STTLRMVAGLEDISKGTLEIDHKVMNDVAPKDRNIAMVFQNYALYPHMSIYDNMAFGL

KLRHYKKDEIDKRVKHAAEILGLSEYLDKKPSELSGGQRQRVALGRAIVRDAPIFLMD

EPLSNLDAKLRVTMRAEIAKLHQNLGTTTIYVTHDQTEAMTLADRVVVMSVGQVQQIG

TPLEVYNKPVNMFVAGFIGSPQMNFFNVHFKGNRISDGKGLNIEIPEGKAKMLKDKGY

EDKDLVFGIRPEDIHSEEAFLETWPNQTVKSTVVVSELLGSTIQLYQKVDGTEFVAIV
NARDYHTPGDKVEMGFDVNKAHFFDKDTTNAIR"
CDS 366717..367934
/gene="cycB"
/locus_tag="OCPHGMPK_00326"
/inference="ab initio prediction:Prodigal:2.6"
/inference="similar to AA sequence:UniProtKB:O07009"
/codon_start=1
/transl_table=11
/product="Cyclodextrin-binding protein"

/translation="MKLWKKLALGSVVALSAISLTACGKSSNSNESKSSDKQLTLWVD

TARVSWYKGVVKDFEKEHPNIKVKVTQNPNGSANAKTDVGKDPSKAADVFGVPNDQLG

QMADSGYINPLSPSDTKEIKKNSVPEAYKGAEWKGKLYAYPYAEQAQTVYYDKSKLSP

EDVKSWKTMTSKGVVATDFTNAYVMWPVMFSAGTKLFGDSGEEVKGSTFDSQNGVNAM

KWYAEQKNNKGVMQTSNALNQLKKGHAQAILDGPWNAANIKKILGKNLGVAPYPTIEL

GGKTAQMEAFLGIECFAVNSHTSNAKNAAILAKYLSNKENQLIVHKNAGEIPVNKAAQ

NTTEVKNDPVAKAVIQMAQPGYSVLQPKLPQMSIFWNDSAPLISGAYDGKIKPSQYKT
KLAKLQKSISKKE"
CDS 367971..369326
/gene="malF"
/locus_tag="OCPHGMPK_00327"
/inference="ab initio prediction:Prodigal:2.6"
/inference="similar to AA sequence:UniProtKB:P02916"
/codon_start=1
/transl_table=11
/product="Maltose transport system permease protein
MalF"

/translation="MFKKKHATPAVTLKETWKDGDTATKLTFFIMGLNAIKHRQWLKG

FSLLFSEIVFLIWFFLSGIHSIAGLSNLGAIKTKRVIFDKAQGVYVTQQPDNSVLILL

FGILALFIVAGFIYLYIINLKSNKHTFLLEKRHEHIPTNREEIASLFDQRLHATLMTI

PIVLIIILFTVLPTVFMISMAFTNYDRQHSIGFSWTGFQAFGNVLGGDLAGTFFPVLGW

TLIWAVAATATTFFFGVLLALLIESKGIKHKNLWRTIFVIVYAVPQFVSLLMMAQFLD

YQGPLNTLLMNWGWISHPIHFIDNQASPLVARITVIVVNMWIGIPVSMLTSTAIIQNL

PQDQIEAARIDGASPVQIFNHITFPQILFVMSPALIQQFIGNINNFNVIYLLTGGAPM

NNNYNGAGSTDLLVTWLYNLTFGQEQRYNASAVLGILIFIISATFSLIAYRHTNAYKE
G"
CDS 369329..370186
/gene="malG"
/locus_tag="OCPHGMPK_00328"
/inference="ab initio prediction:Prodigal:2.6"
/inference="similar to AA sequence:UniProtKB:P68183"
/codon_start=1
/transl_table=11
/product="Maltose transport system permease protein
MalG"

/translation="MKSYHNQRKIALIFRYVLLAILAVLWIFPIIWIILASFSYNNTG

FVSTIWPDKFTWQNYIGIFTNSQYPFVNWVLNTLFVAVISATLSTFVTIAVAYVLSRL

RFRFRKPFLQIALVLGMFPGFMSMIALYYILKALNMLNLGGLILVYVGGAGLGFYIAK

GFFDTMPRAIDEAAEIDGATKWQVFIHIGLPLSKPMIVYTALTSFMAPWVDFIFSGII

LSTSGNPKTYTVAYGLYNMVHSSKGMMAQFFTQFIAGCVVIAIPITILFIVMQKFYVN
GITAGADKG"
CDS 370257..371873
/gene="dexB"
/locus_tag="OCPHGMPK_00329"
/EC_number="3.2.1.70"
/inference="ab initio prediction:Prodigal:2.6"
/inference="similar to AA sequence:UniProtKB:Q99040"
/codon_start=1
/transl_table=11
/product="Glucan 1,6-alpha-glucosidase"

/translation="MTPWWKKAVVYQIYPKSFQDSNGDGIGDIPGIVSRLEYLEKLGI

DAIWLSPVYLSPGIDNGYDISDYQKIDPQYGTMKDMDNLIKEAKKHHIRIIMDLVVNH

TSDQHPWFVEAKKSKDNPYRDFYIWRDSVDGHEPNDLKSAFSGSAWKFDEKTGQYYLH

FFADQQPDLNWKNPKLRNKIYDMMNYWIDKGIGGFRMDVIELIGKEPDQRIRENGPML

HPYLKEMNEHTFARQDMMTVGETWNATPKIAEEYSDPARHELSMVFQFENQNLDQEPG

KEKWDLRPLDLGELKKVLVKWQTEIDFNHAWNSLFWENHDIPRVISRWGNDQEYRVQC

AKMFAIILHLMHGTPYIYNGEEIGMTNCPVKSIAEVEDIESINMYHERLEQGYKKADL

IKSINAKGRDNARRPMQWSNEENAGFTSGKPWLKVNPNYQKINVKAALADPGSIFYTY

QKLIKLRHENPVVVDGDFELVENTGDSVLAFWRKLGNEKWLVVANLSGEKQGFNLDSD

```
                     FKEVLISNYEKRASLKNIVLKPYEAFAVKA"
     CDS             complement(371920..373284)
                     /locus_tag="OCPHGMPK_00330"
                     /inference="ab initio prediction:Prodigal:2.6"
                     /inference="protein motif:HAMAP:MF_01212"
                     /codon_start=1
                     /transl_table=11
                     /product="Deoxyguanosinetriphosphate
                     triphosphohydrolase-like protein"

/translation="MEKFQSKKLDHEKVLRDPVHNYIHVKDKVILDIINSKEFQRLRR

IKQLGPASYVFQGATHTRFEHNLGVYELTRRICDIFEEKYTSKEPGDGLWDPNERLLA

ECAALLHDIGHGPYSHTFEHLFGTNHEKMGQQIITDKNTEVNQALRQVSPNFPELVAS

VIAKTYSNPQVVKLISSQADADRMDYLLRDAYFTGVTYGSFDLTRILEVIRPYRDGIC

FTDKGIHAVEDYIISRYQMYQQVYFHRVNRSMEVILHHLLERAQIIYEAGKLQVTPQL

EAFLKGNWTLEDYLNLDDGVMETNFLLWTNSGDQILSDLSSRYLYRHPLESVKINEDT

KSLLPKLKNLIKQAGFDPNYYTATNSAFDEPYDAYKPTGKNAHSPIEIMQADGSLVEL

SELSPLVKSLNGTLQGDERFFFPKVMVKETDEPQIFDPIYQEFQKYIRNNTLRYLRRP
                     NKKK"
     CDS             373397..373819
                     /locus_tag="OCPHGMPK_00331"
                     /inference="ab initio prediction:Prodigal:2.6"
                     /codon_start=1
                     /transl_table=11
                     /product="hypothetical protein"

/translation="MNKEVEIKITSEIHQEDDSAVFEREGLGSLERIGNNWRLKYNEK

NKEGDVEVKLLIKPNELVMQRGDIKGDHTLMKFEPGEKQKCKVMAAGKQMDLVSLTNK
                     LDFSKISPGKMQLKVEYDLFSGLYLVGNYAIKIEVTDL"
     CDS             373860..374402
                     /gene="rpoE"
                     /locus_tag="OCPHGMPK_00332"
                     /inference="ab initio prediction:Prodigal:2.6"
                     /inference="similar to AA sequence:UniProtKB:Q5XA09"
                     /codon_start=1
                     /transl_table=11
                     /product="putative DNA-directed RNA polymerase
subunit
                     delta"

/translation="MGLDDFKGQNKDELSMIEVARAILQDSGKRMAFADIVNAVQNFL

GKSDEEIRERLPQFYTDMNTDGEFISMGDNVWALRSWFPYESVDEEVNHPEDEDDVPR

KKHHKKVNAFIGDSDDDDIIDYDSDDPEDEDLDVDDDDNNEDDYSDDDLDDADDNELD
                     DGIEGQLSELHQDDLDDDDD"
```

```
     CDS             374533..376155
                     /gene="pyrG"
                     /locus_tag="OCPHGMPK_00333"
                     /EC_number="6.3.4.2"
                     /inference="ab initio prediction:Prodigal:2.6"
                     /inference="similar to AA sequence:UniProtKB:O87761"
                     /codon_start=1
                     /transl_table=11
                     /product="CTP synthase"

/translation="MTKYIFVTGGVVSSLGKGISASSLGRLLKNRGLKVTMQKFDPYI

NIDPGTMNPYQHGEVYVTDDGTEADLDLGHYERIVDVRTSKYSNVTTGKIYQEVLDKE

RRGDYHGATVQVIPHITDMIKKKIMRAALTTDSDVIISEIGGTVGDIESTPFMEAIRQ

MRREVGEENVMYIHCTLVPYLHAAHEMKTKPTQHSVAELRSIGIQPNMLVLRAEKPVP

QDLKNKISTFTDVPVDRIIESIDAPSLFDLPLAFQAQGMDQKVCDFLHLESPKPEADM

EAWKKLDERAKNLKHETTITLVGKYVELEDAYISVTDALQHAGYLYDTKIKVNKVQAE

DITEDNIAEIMEGSDGLIVPGGFGTRGLEGMITSIKYAREHDIPFLGICLGMQMASVE

FARDVLGLKDANSAEADPETKNNIIDIMADKRDEENIGGTLRLGLYPATLKKGTKTRA

AYDDQDVIQERHRHRYEFNNDYREAFEKAGMVFSGVSPDNHLVEIIEIPDKKFFIAAQ
                     YHPEFLSRPQRPEGLFKAFIGAASGLPAQHFN"
     CDS             376289..377554
                     /gene="murA1"
                     /locus_tag="OCPHGMPK_00334"
                     /EC_number="2.5.1.7"
                     /inference="ab initio prediction:Prodigal:2.6"
                     /inference="similar to AA
sequence:UniProtKB:A0A0H2ZNL3"
                     /codon_start=1
                     /transl_table=11
                     /product="UDP-N-acetylglucosamine
                     1-carboxyvinyltransferase"

/translation="MKQMIIHGGKPLKGDVWIGGAKNSTVALIPASILSRTPVTLEGV

PRIADVDNLMDLLSEMDVKCDFHETTLRINPDDIKRSPLPAGKIKSLRASYYFMGALL

GRFGKAVVGFPGGDDIGPRPIDQHIKGFEALGATVENENDQIIITAPKTGLRGAKIHL

KMPSVGATMNIIMASVMAKGQTIIENAAKEPEIIDLATFLNNMGAVIRGAGTDVIRIE

GVEELKAQTPHTIIPDRIEAGTYVALAACIGDGIRIHNIIEEHLDSYLAKVEEMGVVI

DADEDSLYVYPAGDLKMVQVRTDVYPGFATDLQQPITPLLLTAKTGEGVVIDQIYPKR

VGHIPELQKMGANIHVEDNIILVHPTHHLHGAHVSAGEIRAGACLMLAGLMADGETII
                     SNAGNILRGYDRIEQKLRQLGAEVSVIDV"
```

```
     CDS             377765..379348
                     /gene="hlyB"
                     /locus_tag="OCPHGMPK_00335"
                     /inference="ab initio prediction:Prodigal:2.6"
                     /inference="similar to AA sequence:UniProtKB:P08716"
                     /codon_start=1
                     /transl_table=11
                     /product="Alpha-hemolysin translocation ATP-binding
                     protein HlyB"

/translation="MIKSFFKSNYKYTFIFIVSTVIGGALVAYGSYGLTPATDSLKSL

DTKKFILWIVVITIASAGGRVLQFSAGYLFAKQEQEYFHSLRKKLINHYYESTSIDKL

SNMQNRLTNDLKIISDQALDPVSNVVLCVVDLVFSAYVVLTFNFWLLCLILVLAVIML

YLPDLLSKQLEKATNEVSSNYSKYIDTIEKWLAGLAVLQRYRSRTKLSAALARSSSGL

EKATVIRDKKSVELRNLTYTANMVAQGAVLLATGLLVLNHQLTIGTLMSIANFSSLIF

SQLVNVTSQIGSIKSTRSLADSVMKEIKELPNSEKKEDSLTNFDTLSTHGLVLQYPNG

EKIKYPDIEIKAGEKVLLSGDSGTGKSTLFKMILNEIKPTQGKIVFKDKLGKILNPDY

SQIGYIPQDPVVFPGTIEDNITMFNSRLNKKAANWAQKVQLATDFEKFPDGIKTVVDL

DKNNLSGGQRQKIILARTEVYNSKIILIDEGTSAIDSVATEQILKNILKEPATVVFIA
                     HNLTDEMVQLFDRRINLSK"
     CDS             complement(379404..380732)
                     /gene="pucK"
                     /locus_tag="OCPHGMPK_00336"
                     /inference="ab initio prediction:Prodigal:2.6"
                     /inference="similar to AA sequence:UniProtKB:O32140"
                     /codon_start=1
                     /transl_table=11
                     /product="Uric acid permease PucK"

/translation="MKHMQVNHKKAALLGLQHLLAMYSGAIAVPLLIGTALKFNSTQM

TYLVSIDIFMCGLATALQLLRNRYFGIGLPVVLGCAIQAVAPLQMIGKKFTIGTMYGA

IIVAGIFVFLVAGYFSKIKKLFPPVVTGSLITVIGLSLIPVSIQNLGGGNSAAKSFGD

PKNLLTGFITVAIILALQVWGKGFIKSIAVLVGLIAGTLIASMLGMVSLTPVAQASWF

HLPQPFYFGMPQFEWSSSLTMIIIALVSMVESTGVFFAIGDLLHKDISSDDLKKGYRA

EGLAQILGGIFNTFPYTTFSQNVGLLELSGITTKRPIYWASGFLMLMGLLPKFGALVT

IIPDSVLGGAMLVMFTMIAVQGMRMLKRVDFDDTRNILIVAISIGLGLGVTVYPQVFQ
                     SLPETVQLFLGNGIVVASLSATILNLLFKGKSGLEEQHIPAAPEKIEVQH"
     CDS             complement(380733..381311)
                     /gene="xpt"
                     /locus_tag="OCPHGMPK_00337"
```

/EC_number="2.4.2.22"
/inference="ab initio prediction:Prodigal:2.6"
/inference="similar to AA sequence:UniProtKB:P42085"
/codon_start=1
/transl_table=11
/product="Xanthine phosphoribosyltransferase"

/translation="MKLLEERIRKDGEVLDGDVLKINSFLNHQVDPELMMQVGAEFKR

LFENEKITKVLTCEASGIAPGIMAAYQLGVPMVFARKKKPSTLNDAVYWADVFSYTKK

VNNKICVEKKFLSSDDHLLIIDDFLAHGEAVKGMLNIARQADASVAGVGVVVAKEFQG
GSDWVKEHGYRLEALARIANFENNQVHFVGEK"
CDS complement(381527..382303)
/locus_tag="OCPHGMPK_00338"
/inference="ab initio prediction:Prodigal:2.6"
/codon_start=1
/transl_table=11
/product="hypothetical protein"

/translation="MSNENYISYSATQDFLSARRRSSTLIASGVMLCIFSPIVLLILI

SLTRLDILTWSINFATGIGVIVLLTFIATAVALFIAGNRWLKVHENYEYEDCNLSNNV

REKIIKENKVYENQHMIFKIIGITFCILSAIPLMSGALFVDALASSRLDDLMTGFSSA

TLLLVGIGVFFLVKTNIIHDSFNIILQLDDYTSEKKAGKKLIEKYATIYWMIISFIYL
AYSFLSNNWNQSWIIWPLAGIAYGIFEAVMSLKKKKAVSE"
CDS 382488..384041
/gene="guaA"
/locus_tag="OCPHGMPK_00339"
/EC_number="6.3.5.2"
/inference="ab initio prediction:Prodigal:2.6"
/inference="similar to AA sequence:UniProtKB:P99105"
/codon_start=1
/transl_table=11
/product="GMP synthase [glutamine-hydrolyzing]"

/translation="MAKTNLNDFDKIIVLDFGSQYNQLITRRIRDFGIYSELLPHDLS

IEKIKKMAPKGIIFSGGPNSVYDEGALKVDPEIFKLGIPILGICYGMQLMSYDLGGNV

EKADNSEYGRADIEVTDPNAVLFEGLPKEQYVWMSHGDLVTKAPEGFKVTAKSKNCPI

SAIANDEKKFYGIQFHAEVRNSEYGLDILKNFAFNVCDAKANWTMDDFIDMQVDEIRE

KVGDKKVILGLSGGVDSSVTATLLHKAIGDQLTAIFVDHGMLRKDEGDQVMQALNKDL

GVNIIRVNAQKRFLGKLKGVTDPEQKRKIIGKEFIEVFNEEAKKLKDVDFLAQGTLYT

DVIESGTNTAQTIKSHHNVGGLPKDMHFELIEPLRKLFKDEVRELGEKLGIPHDLVWR

QPFPGPGLGIRVLGEVTEDKLKIVRESDAILRDEIKKAGLQEDVWQYFTVLPGIRSVG

```
VMGDGRTYDYTIGIRAVTSIDGMTADFAKIPWDVLSKISTRIVDECDHINRVVYDITS
                     KPPSTIEWE"
     CDS             384587..385114
                     /locus_tag="OCPHGMPK_00340"
                     /inference="ab initio prediction:Prodigal:2.6"
                     /codon_start=1
                     /transl_table=11
                     /product="hypothetical protein"

/translation="MGGAIALLTVGLTGCANNTSSNSSNNQATSSKSSKTNQSSSHEL

TITDIVGTYVNKDKTAAVVLQNGHTLWYYQTKNHNDGSKNDSGNWHYEGHNKFSITMS

HRDDNNQEVDTKGSLVLDGKTLTMTENNNSAKQVFTKTSNEEKEYDEIFNPGKKVDLD
                     DPSTSANKNDQSSNN"
     CDS             complement(385172..386176)
                     /gene="immR_1"
                     /locus_tag="OCPHGMPK_00341"
                     /inference="ab initio prediction:Prodigal:2.6"
                     /inference="similar to AA sequence:UniProtKB:P96631"
                     /codon_start=1
                     /transl_table=11
                     /product="HTH-type transcriptional regulator ImmR"

/translation="MRLGQKIADLRKKNNLSQESLAEKMNVSRQAVSKWESEQSIPDI

EKIVNLSELFGVTTDYLLKSGEPSFELKNEDINDKDKLPVLSDELVKKYLTASQKSSK

LRALAIALIIFSPVWICFACILSIFFDHDSHYETPLTFTISLSGYVAAIITIAIALGL

FIYSLLNMREFKQIKKQHFDLTEERKKLEFTIKLFHQNNDKYVVFASILAVLSIIGPM

INWFSNFSSSSIGFIITWGITFIIFSGASYLFTFYLFQRNYLSILIKHKKHLPIKLHR

LFVYGSWIYAFCVLGIFYITTRLPYGLYIPFFNPTLFIDFSIIVYCLFTYFFIKEKAE
                     "
     CDS             386361..386516
                     /locus_tag="OCPHGMPK_00342"
                     /inference="ab initio prediction:Prodigal:2.6"
                     /codon_start=1
                     /transl_table=11
                     /product="hypothetical protein"

/translation="MDFLKGKENNKTAKVFKAGNSEAIRLTKQDTAIFKVKPEYQVIK
                     EVGCRLH"
     CDS             386501..386608
                     /locus_tag="OCPHGMPK_00343"
                     /inference="ab initio prediction:Prodigal:2.6"
                     /codon_start=1
                     /transl_table=11
                     /product="hypothetical protein"
                     /translation="MSITLKKPEVSDDVKKKVSEILNEDADLIQALKDL"
     CDS             387104..387631
```

```
                     /gene="betI"
                     /locus_tag="OCPHGMPK_00344"
                     /inference="ab initio prediction:Prodigal:2.6"
                     /inference="protein motif:HAMAP:MF_00768"
                     /codon_start=1
                     /transl_table=11
                     /product="HTH-type transcriptional regulator BetI"

/translation="MNTQDKLVGKTITVIDKYGYQNLSLRKLTATLGLTTGAFYKHFK

SKDALYEKVAIELSRNFIDKISLPDNATAQERLLIIAKNFCQYVDQHSNLAEFLFFNP

TTLKAFSTKRSKYDFLNKMKVLVNELNPKKNVSNQELFIQIWSFIQGYAALVNNKIVE
                     YDEDLVKGTLDEFLQ"
     CDS             387647..388228
                     /gene="azoR_1"
                     /locus_tag="OCPHGMPK_00345"
                     /EC_number="1.7.-.-"
                     /inference="ab initio prediction:Prodigal:2.6"
                     /inference="protein motif:HAMAP:MF_01216"
                     /codon_start=1
                     /transl_table=11
                     /product="FMN-dependent NADH-azoreductase"

/translation="MDCLIIYCHPYDKSFNHAVLENVENNLKQNNKDYKLIDLYKDGF

VPIYDLEEMRLFHTGQTHDPLVKKYLSWLKETPEVIFITPFWWNGIPGMLKGFIDKVM

KEGPGLSHTVTKTGIHGELTNVKHTYIFTTSTSPTFYLKLFLGNGIKRIFVNKTLKQL
                     GMQDRHWYNLGGISNSSIEKRKKYLKKIAKINL"
     CDS             388738..388941
                     /locus_tag="OCPHGMPK_00346"
                     /inference="ab initio prediction:Prodigal:2.6"
                     /codon_start=1
                     /transl_table=11
                     /product="hypothetical protein"

/translation="MEYIKKLFADPKMYNEVEFGKKIHEENVLLKLMQEILPEEHVVQ
                     PDFVFPKIRIIEIFLLKLLMEKL"
     CDS             388941..389198
                     /locus_tag="OCPHGMPK_00347"
                     /inference="ab initio prediction:Prodigal:2.6"
                     /codon_start=1
                     /transl_table=11
                     /product="hypothetical protein"

/translation="MIGLLVADKETWPSGLNYLQNMLKDEVYRFMRNELLLCRTYFIL
                     LTPVDPWKNGREKYTISFRNEQSEELTEMLKSKLVIVCPEN"
     CDS             389256..389546
                     /locus_tag="OCPHGMPK_00348"
                     /inference="ab initio prediction:Prodigal:2.6"
                     /codon_start=1
                     /transl_table=11
                     /product="hypothetical protein"
```

```
/translation="MILLMRKGERMLYPYIELPDETIITHSEILENNSVLINFEQPYD

DGFREARIELPSYKWLYNDGFSNEQIKFFVQFAHKNAAVIYKYARLGGIDNA"
     CDS             complement(389908..390819)
                     /gene="menA_2"
                     /locus_tag="OCPHGMPK_00349"
                     /EC_number="2.5.1.74"
                     /inference="ab initio prediction:Prodigal:2.6"
                     /inference="protein motif:HAMAP:MF_01937"
                     /codon_start=1
                     /transl_table=11
                     /product="1,4-dihydroxy-2-naphthoate
                     octaprenyltransferase"

/translation="MKKYSIIQKFNALVQIQTIIISALPYIIGSLMASYYYHHFNLSY

CLWLFLAVISFHLAVNGHNQYTDYLRYKKNHVTSYNNILEKFNISRKWARNVIILLTL

VSATIGIILSFKVGWIILLIGFFSYLIGYCYSGGPYPILKTPFGEPASGITMGYNITL

LGIYVNIYNIHPFDSFFWAKAIIVACPAIFVIANVMLANNICDVEEDVKIGRKTLPFY

IGRKNALIVLCIYYVLAYIFLILSVVLHYLPTLALGGLLTIPLVYRSTRTFVKDPHKE
                     TTFKGILTNVLLVLVSEIIFSLIGLVW"
     CDS             complement(390930..391274)
                     /gene="ogt_1"
                     /locus_tag="OCPHGMPK_00350"
                     /EC_number="2.1.1.63"
                     /inference="ab initio prediction:Prodigal:2.6"
                     /inference="similar to AA sequence:UniProtKB:P9WJW5"
                     /codon_start=1
                     /transl_table=11
                     /product="Methylated-DNA--protein-cysteine
                     methyltransferase"

/translation="MVKYFFQGKEPNFIPQLHLTGTDFQKRVWQALLKIPYGTTTTYK

DTAMKAAQDKKHLPIRATGGAVGRNHICIIVPCHRVVGVNHNLTGYGGGIDKKIKLLQ
                     LEGVDISQFKKP"
     CDS             complement(391524..392036)
                     /locus_tag="OCPHGMPK_00351"
                     /inference="ab initio prediction:Prodigal:2.6"
                     /codon_start=1
                     /transl_table=11
                     /product="hypothetical protein"

/translation="MKDNYTIDHDMYLMNHKNGDVLKVTVDNKDKSLKNLQLYRQDGL

GNYYYDKNSQLKHQDNLATKKDIVKASKEIGAHNTHHIDKWHHKVTPVYNAEHKITDF

EPKLALEHHAKIKNAKRYLRHLNHHHADFLFLANKGLLRQPLNYLKDAIYTDRNKEMV
                     IAHKVHKIAK"
     CDS             complement(392243..393601)
```

/gene="frc"
/locus_tag="OCPHGMPK_00352"
/EC_number="2.8.3.16"
/inference="ab initio prediction:Prodigal:2.6"
/inference="similar to AA sequence:UniProtKB:P69902"
/codon_start=1
/transl_table=11
/product="Formyl-CoA:oxalate CoA-transferase"

/translation="MSENEKNEQNEYAPLKGIKIVDWTQVQSGPSCTQLLAWLGADVI

KIERTNTGDPTRNELLDIKDSWSLYYLQLNANKRSLTLDIKTPEGKKIMYDLAKKADV

FVENIAPGSADRNGFGWEKLHSINPRLIYASLKGFNEGSRFENVKAFEPVAQAAGGSA

SATGWNKGANNVPTQSAAALGDSNTGMHLAIGILAALLQREHTGEGTFVYQSMQDAVL

NLCRIKLRDQLMLDNLGALPHYAVYPDWKWGKAIPRAENTEGGQVIGWTYKAKGWETD

PNAYVYIVIQNENKKWDKMCEAMGHPEWEKDPRFVDWQHRQLHKEELYPLIEAYTKQY

DKYTLTKELGAAGVPVGPVLDWHELENDPDLNSDGTIVTIDQGGNRGKFKTIGLPFSL

SNYKPAYDRAPDLGENNKEILTELGYDADQIEDLVNKGVISKAKGPKNPRVHVIIDGK
EH"
CDS complement(393607..395343)
/gene="oxc"
/locus_tag="OCPHGMPK_00353"
/EC_number="4.1.1.8"
/inference="ab initio prediction:Prodigal:2.6"
/inference="similar to AA sequence:UniProtKB:P40149"
/codon_start=1
/transl_table=11
/product="Oxalyl-CoA decarboxylase"

/translation="MVDDSLNRTGASLLIDALQKNGINNLYGVVGIPITDLARLAELR

GMKYYGFRREDSAVDAAAAAGFLTKKPGVAMTVSAPGFLNGLTALAQATKNCFPLIMI

SGSSERHIIDLAQGDYEGLDQYNVAKPFCKKAYRVDRAQDVGLAVARAIRTAVSGRPG

GVYLDLPADTIAQLNDDLGEKNMGVYKLVDPAPLQEPSDDAINRAVNIIKQAKKPLII

LGKGAAYDRTEKQVQELINKTDIPFLPMSMAKGLVPDDDKHSAAAARSLSLKNADVVI

LIGARLNWMLSYGDAPQFNPDAKFIQLDIDATQFDSAQKISAPLQGDLTSILNKLVPA

LTATGYKVPSDWLDAIAQDTAKNDAKFAKRIKAGETNPEFGYYGAIEPINDYFQEHPD

TYLVSEGANTLDIGRDMIGMKLPRHRLDTGTWGVMGVGMGYAIATAVETGKHVVALDG

DSAFGFDGMDVETMCRYNLPITVVVINNGGIYNGVDNVVPDQLGPTTLDPTGRYDLIA

KAFGGDNYYVTNYQEMKDTFATAVDSGRPSIINVQIDPSMGKESGHIGNLNPELNLKP

```
                     LEEAEQKKIDEE"
     CDS             395702..396883
                     /locus_tag="OCPHGMPK_00354"
                     /inference="ab initio prediction:Prodigal:2.6"
                     /codon_start=1
                     /transl_table=11
                     /product="hypothetical protein"

/translation="MISILVNDIIPILVIMLLGYLCGKFYFFDNDQRQGLNKLVLDIA

LPAALFISIVKATRKMFAQDIVLTLISIVGVVGLFMLSYYLDKLLFHRTTQQAAVCAL

IAGSPTIGFLGFAVLDPIYGNNATTNLVIGIVSIVVNAITIPLGLALINKGQTLTKKK

AKANPNNKGTKVEVEDIKGKGLNKTKKKVVVTIPDGVPVTDAEAKALKEKGIEREIDL

ARAEIADHADEPHKPMNPTLKSVINAIKQPVAAAPLIAVILVLIGIRIPTSWAPTFDL

IAKANAGVAVLAAGLALSTVKFSIDKEVIWNTFFRLFLTPAVIVAAAYLCGMGSDPTK

ISMLCLATGLPPAFSGIIISSRYNIYVKEGASSVAVSTVFFAVSCIFWIWLLPILAHM
                     F"
     CDS             396947..397222
                     /locus_tag="OCPHGMPK_00355"
                     /inference="ab initio prediction:Prodigal:2.6"
                     /codon_start=1
                     /transl_table=11
                     /product="hypothetical protein"

/translation="MIDNKNLLKLLRIELRKMSNGNKLKFLTYKKDRSVLVEKNGDNF
                     RIVEDGYRKIVWDDLTEAEVLKRVKRLQKIEFPRSNKLYLARERQAL"
     CDS             complement(397242..397958)
                     /locus_tag="OCPHGMPK_00356"
                     /inference="ab initio prediction:Prodigal:2.6"
                     /codon_start=1
                     /transl_table=11
                     /product="hypothetical protein"

/translation="MKNISKKFGLISLLFSLAMILSACSIIISNHDDTKPEIKTTISN

QGFNKLEISVALADISVKYGDKYQVEYEGPKNLKPTVSRKNGILTIKQSATGIHHVNG

DPAITITVPKQLLKNLTLSTNDGDIEVENIATTSPISLETNDGDININNLKAPSGKID

TNDGDITINKLSNQNNFKVNTDDGDIKITQSNFTGYKLNVSDGDINVKGRSSNDFFVE
                     NQNSSNVLTASSQDGDIKIK"
     CDS             398045..398497
                     /locus_tag="OCPHGMPK_00357"
                     /inference="ab initio prediction:Prodigal:2.6"
                     /codon_start=1
                     /transl_table=11
                     /product="hypothetical protein"

/translation="MKKLPPIQKILEAYTAIVDNHVNLTENEAKVTSSNGAKTYTVSW
```

```
ENDVYHSNDNATYWQGYAGYPVIAVLMLQGKLPFNQKLADNFKAVNWNEINLSFKRNY
                     AKAATEVIEEKGLNENEVMAELEVVYENLKKLPITIKRGSLRPPKARK"
     CDS             complement(398523..398966)
                     /locus_tag="OCPHGMPK_00358"
                     /inference="ab initio prediction:Prodigal:2.6"
                     /codon_start=1
                     /transl_table=11
                     /product="hypothetical protein"

/translation="MQGTYECVKVIGTFHRYGVQQSVDSNGTFYYRPVVYMKDCKDLN

NIDLKIPDYLLFNLTKGFKSLGILNPGIKLKINCRRYLKEERNAGYGYPTNIVIANDR
                     PDFPDDPNVLTGMIMVKNQGNPEAEKDPINTDLIQIYKNWLANKE"
     CDS             complement(399188..400603)
                     /gene="pepDA_1"
                     /locus_tag="OCPHGMPK_00359"
                     /EC_number="3.4.-.-"
                     /inference="ab initio prediction:Prodigal:2.6"
                     /inference="similar to AA sequence:UniProtKB:Q48558"
                     /codon_start=1
                     /transl_table=11
                     /product="Dipeptidase A"

/translation="MKQTECTTILVGKDATIDGSTMIARSEDGGRTIIPERFDVVMPK

DQPKHYESVISHQKIDDKDLLPNPLRYTSAPDASGENGIWAAAGINSDNIAMTATETI

TTNSRIQGIDPLLEEGGLGEEDFVTLTLPYIHSAYEGVERVGYLLEKYGTYEMNGMAF

SDKNEIWYLETIGGHHWAARRIPDNAYVIAPNRLNIDEFYFDDDDFASSKDLKDLIEE

YHLNPDLEGYNLRHIFGSSTIKDAHYNNPRAWYIHNYFDPDFGGEPSDQDQPFICHAN

RKISIEDIKWAESSHYQDTPFDAYGDQGSAEQKKTFRPIGINRNFETHILQIRNDVPE

KIAGVQWLAFGPNTFNSMVPFYTNITTTPASFQTGPKFDLNKIFWLNKLTAQLGDTNF

RVYGELEADFEQKSLAQCHKIQHETDKEAEKYSGDELQDKLNEANQKMADTVYNNTVE
                     LLGNMVNEGHGLMTLKYDLLD"
     CDS             400753..401988
                     /gene="glyA"
                     /locus_tag="OCPHGMPK_00360"
                     /EC_number="2.1.2.1"
                     /inference="ab initio prediction:Prodigal:2.6"
                     /inference="similar to AA sequence:UniProtKB:Q5SI56"
                     /codon_start=1
                     /transl_table=11
                     /product="Serine hydroxymethyltransferase"

/translation="MNYGEKAPALWDAIKSEEKRQEDTIELIASENIVSDAVREAQGS

VLTNKYAEGYPGKRYYGGCQYIDQVEQLAIDYAKKLFNAEYANVQPHSGSQANMTVYN
```

ALLKPGDTILGMGMDAGGHLTHGSKVNFSGKIFNSVSYDLNPETEELDFEKIRQIALE

NKPKLIIAGASAYSRIIDWQKFREIADEVGAYLMVDMAHIAGLVATGAHPSPIPVADV

VTTTTHKTLRGPRGGMILSNNKKLGKKIDSALFPGTQGGPLEHVIAAKAQAFYEDLQP

QFSTYIEQVVKNAQAMADEFKKSENIRVVSGGTDNHLMIIDITKTGVTGKDAQNLLDS

VNITTNKESIPGDTRSPFVTSGLRIGTPAITSRGFKEDDAREVARIIIKVLDSPEDKD
VLTNAKSSVKSLVEKHQIK"
CDS 402059..404290
/gene="agaSK_1"
/locus_tag="OCPHGMPK_00361"
/inference="ab initio prediction:Prodigal:2.6"
/inference="similar to AA sequence:UniProtKB:G4T4R7"
/codon_start=1
/transl_table=11
/product="Bifunctional alpha-galactosidase/sucrose
kinase
AgaSK"

/translation="MIDVIDNKYFYLHNKEVAYLFDLLPNQQLEHLYYGEDLGKLSKN

DLEYMSLHPNKASGTVKFSKKIKNLTLADRMQEFPTYGTSDFREGAVSIENEDAYLYP

EFKYDHFEITTGKRRSLDFPTSYGNDSENLIVYLIDTEHQLELQLSYSIFENLSAIVR

KAAIINHGQQEIKLTNLQSTVLDLPNDSYDFLQLSGAWTKERHIIRRPLTQGITKIES

LRGASSHQENPFIALVDKDVTNDGGNIYASNLIYSGNFISQIEVDEWGQSRLMTGINP

ATFTWRLSRNQVFSTPEAVLFYSKHGFNGLMKQTHNFVTKHIIDQKWQRRDRPIVINN

WEATYFEFNEAKLLKLAKEANKLGIECFVLDDGWFGKRDSDNSSLGNWTVDKRKFPQG

LDHFSAQVHKMGMKFGLWFEPEMVSPDTELYNMHPDWIVRHPYNRFSVGRSQYVLDFA

NPEVVDNIYRQMEKVIETAQVDYIKWDMNRNITEAYSPYLNKSGYSQGEFFHRYIQGV

YKLYAKLLTSFPDLLIEGCASGGGRFDLGILYYSPQIWPSDDSDAAERLDIMSGTLLA

YPLSTFSNHVSAVPNDQVKRITSLKFRQNVACFGPLGYELDLGKLSTEEKNQIKNWIR

WYKENRKLLVNGTFDQLLKFDPSQNTYAWSVSNSNKQIVGFYRKLARPNETLDQYLPL

KHLNDQQKYQINSDQMVSGRVLQKFGLREPYQFNASNPDTAQISGDFQSYLYQIKTID
RKI"
CDS 404549..405541
/locus_tag="OCPHGMPK_00362"
/inference="ab initio prediction:Prodigal:2.6"
/codon_start=1
/transl_table=11
/product="hypothetical protein"

```
/translation="MKRMYGLIAILIAFLAVAFFTENRKQETERKVPQVGVLTLMHHP

ALDEIYRGFKAELKAAGYVDGKNIKIDYQNANNDQSNLRTMATKLTDEQSKVLVGITT

PAAQALANSTSKTPIVLGAVTNPKASGLVKNERHPESNVTGVSDAAPIKQQLDLIKKF

MPNLKTLGVIYTSSDSSAVSGFREIEQECKRRKINLRVFSIANSNDLNQVSEQMFRQV

DAAIVPTDNTIAGAMETLAKNGNAAKKPIFPAAATMVKQGGLATYSVNQYKLGQMTGK

MTIAILKGKRVSSLPVERVQKGEPVVNLKEAKELNLQVPQRFLKECQRNGVVYR"
     CDS             405538..406437
                     /gene="lsrC"
                     /locus_tag="OCPHGMPK_00363"
                     /inference="ab initio prediction:Prodigal:2.6"
                     /inference="similar to AA sequence:UniProtKB:Q8ZKQ3"
                     /codon_start=1
                     /transl_table=11
                     /product="Autoinducer 2 import system permease
protein
                     LsrC"

/translation="MNLIVSAIGQGLLWALLGLGLYLTFRILNFADMTVEGTFPLGAA

VAVANLAHGMSPYLATFLALLAGMIAGLVTGLLYTKGKIPILLAGILTMTAIYSINLR

IMGGSNISLIGKKTLLNNEFLKSLPQYFNSVVLGVIVVVIITGILVFFLNTDFGQAFI

ATGDNPKMAKSLGIYTDSMIIIGLMLSNGIVALCGALIAQNNGYADINMGIGTIVIAL

ASIIIGEVAFGELTLNQRLVAVTLGSIIYRLILLAVLQLGFSANDLNLISSIVLALCM
                     MLPQLEKIVHLKKPIERGVKKHD"
     CDS             406430..407194
                     /gene="thiQ"
                     /locus_tag="OCPHGMPK_00364"
                     /EC_number="3.6.3.-"
                     /inference="ab initio prediction:Prodigal:2.6"
                     /inference="similar to AA sequence:UniProtKB:P31548"
                     /codon_start=1
                     /transl_table=11
                     /product="Thiamine import ATP-binding protein ThiQ"

/translation="MTKPILELKDVKTTVKTADGEIVPILKGIDLKIKAGDFITIIGT

NGAGKSTLLNTIAGSLRPDSGFLLHNGHDITKMSEEKRTQFIGRVFQDPKMGTAPRMT

VAENLLLATKRGKRRFLKIRKLKQNLPRFKKLAAVMNNGLENRLNTFVEGLSGGQRQA

LSFLMATIERPDILLLDEHTAALDPHTSENLLAVTDQQIKENKLTALMITHHMEDALK
                     YGNRLLVLKDGKVKADINEQEKQNLKVSDLYKYFED"
     CDS             407292..408893
                     /gene="yveA"
                     /locus_tag="OCPHGMPK_00365"
```

/inference="ab initio prediction:Prodigal:2.6"
/inference="similar to AA sequence:UniProtKB:O07002"
/codon_start=1
/transl_table=11
/product="Aspartate-proton symporter"

/translation="MNNKGHKISLFSAIMLALNSLIGSGWLFGSGSAAKIAGPAAILS

WIIGAVIIIAIALTYVELGAMFPESGGMSKYAQYSHGPMLGFIAAWANWISLVTLVPM

EAVAAVQYMSSWPWKWANWTHHFMQGGNITTPGLLVVFAFMIIFTLINFWSIKIMTHF

TNLISVFKVLLPTITIIMLFASSFHPANFGHDVKTFMPYGSRAIFEAASGAGIIMSYD

AFQTIINIGGELKNPRKNIIRGVLISMLVTAAIYILLQIAFIGAVDPAIIAKNSWQGI

NFASPFADIAILLGINWLVILLYMDAFVSPFGTGVAFVATASRALAAMTHTKHLPAWL

GRLNRRYLVPRFAMVADLILAMILVSVFRNWNLLATVITCATLIAYLTGPVTTITLRK

IAPDLDRPYKPVYMRWFAPLTFVLTSLAIYWTMWPTTIQVILVIVLGIPIYFYYEVKY

QKRNFKSQFKHAAWLLGYLVFISIMSYCGSDGFGGQNWISYPWDFLVISIASLLFYKF
AINSGLEKIDATAIKVNEKIKLEED"
CDS 409011..409262
/gene="rpmE2"
/locus_tag="OCPHGMPK_00366"
/inference="ab initio prediction:Prodigal:2.6"
/inference="similar to AA sequence:UniProtKB:Q8DTN5"
/codon_start=1
/transl_table=11
/product="50S ribosomal protein L31 type B"

/translation="MRKGIHPDYQEVVFMDSATGAKFVAGSTLKPEETIEFEGKTYPL
VRVEISSDSHPFYTGKQKFAQADGRIEKFNKKYGISSKN"
CDS 409428..410795
/gene="murF_1"
/locus_tag="OCPHGMPK_00367"
/EC_number="6.3.2.10"
/inference="ab initio prediction:Prodigal:2.6"
/inference="similar to AA sequence:UniProtKB:P11880"
/codon_start=1
/transl_table=11
/product="UDP-N-acetylmuramoyl-tripeptide--D-alanyl-
D-

alanine ligase"

/translation="MKMQLAEIAKALNSSVNMGEDKVITSVVFDSRKATPNSLFIPLA

GVRDGHDFVSNAMANGAEATLWKKGHEGKPTDVPVIEVEDPLTALQSLAKYYLGKVNP

TVVGITGSNGKTTTKDMVAAVLAKRFNVHKTQANFNNEIGVPVTILEMKPSTEILVLE

MGMDRPGQLHHLSELVRPDVCVITMIGEAHIEFFGTRDKIADAKMEITDFLKEDGKFI

YNGDEPLLEERAAKIKQDKSTFGFREEDTVFATTFRSYMHHATFEVNDSEQQFKIPMI

GKHNVSNALAAISVGRHFGENDEQIAQALANFTPTANRMQWKKGDVGEAIMDDVYNSN

PTAVKAVTTSFGQVMVKDGGRRIAVLGDMLELGDESAELHASLANALDPAIINELYLY

GPEMKNLYDALVDKYPAANLHYYPQDEMNHMIDDLKNDIKPDDIVMLKGSHGMHLEKV
LDRLM"
CDS 410811..412268
/gene="cshA"
/locus_tag="OCPHGMPK_00368"
/EC_number="3.6.4.13"
/inference="ab initio prediction:Prodigal:2.6"
/inference="similar to AA sequence:UniProtKB:Q8Y8N0"
/codon_start=1
/transl_table=11
/product="ATP-dependent RNA helicase CshA"

/translation="MKFSEMNLKPEILKAIKRSGFEEATPIQEKTIPLVLEGKDVIGQ

AQTGTGKTAAFGLPILQNLDKQHDSIQAIVIEPTRELAIQTQEELFRLGRDEKARVQV

VYGGADIRRQIRALKQTPAILVGTPGRLLDHLKRGTIDISKVKTIVLDEADEMLDMGF

IQDIESILNYASSKHQTLLFSATMPKPILRIGEKFMHDPEIVKIKGKELTANLIDQYF

VRAKENEKFDILCRLIDVQNPDLAVIFGRTKRRVDELTRGLQARGYNAAGIHGDLSQA

KRMSVLKRFRKGKLDILVATDVAARGLDISGVSHVYNYDIPQDPDSYVHRIGRTGRAG

QNGMSVTFVTPNEIGYMRTIEQLTHKKMMPLKPPTDEEAFKGQLSAANKKVTELLDGD

LSKYTAEASQLLDDYSAVDLVAALLKNLSKDAESVKVKITPEKPLPFKSKHGNNNRHF
KRNFKRGGDRNERYHRKPNARRGGRKHDFVIKKKD"
CDS 412341..412700
/gene="acpS"
/locus_tag="OCPHGMPK_00369"
/EC_number="2.7.8.7"
/inference="ab initio prediction:Prodigal:2.6"
/inference="similar to AA sequence:UniProtKB:P0A2W6"
/codon_start=1
/transl_table=11
/product="Holo-[acyl-carrier-protein] synthase"

/translation="MIRGFGIDSVEVERMKKIVEKGDKFAKRVLTPKEFTQYQQLKGK

RKVEYLGGRFSLKESFSKALGTGLGKYVGFQDIETLWDDLGHPVMTSTKFSGNIFASI
THDDHEIITGVVLEELN"
CDS 412700..413830
/gene="alr"
/locus_tag="OCPHGMPK_00370"
/EC_number="5.1.1.1"
/inference="ab initio prediction:Prodigal:2.6"

/inference="similar to AA sequence:UniProtKB:Q9RLU5"
/codon_start=1
/transl_table=11
/product="Alanine racemase"

/translation="MVPGIHRPAVVKVNLGAIKRNIENEMQHLEPGQKMLAVVKANGY

GHGAVEVAKLADQVGAAGFCVAILDEALELRRADITKTILVLGVVSPEYAPIAAANDI

SLTVPNLEWLKEAEKYLENSDLQLKIHLGIDSGMGRIGFNEDKDFIAANKFLKNNDNF

YIEGMFAHFASADSSDETYFKHQCEKFNHMKSLLTVKPKWIHVDNTAASIFHDGIKSD

LVRFGIGIYGLNPSSNPNSTDLKSKIALDPALSFESELTHVKTIHQGDGVGYGSTFVA

NKDTIIGTVPVGYADGFIRKFQGFKVKVGNAYCPIVGRICMDQFMVEMPKEMPIGTKV
VIISNNPDDPNNIKAAADYVDTIHYEVACLLNDRLPRVYYED"
CDS complement(413893..414573)
/locus_tag="OCPHGMPK_00371"
/inference="ab initio prediction:Prodigal:2.6"
/codon_start=1
/transl_table=11
/product="hypothetical protein"

/translation="MLIKSLVYKKDYLTTVNEKATLAEALKILEDSGFRCVPILDDTG

TIFRGNIYKMHIYRHKSQGGDMNLPVTYLLKNATKTIKVNSPFFRVFFNIKDLPYIAV

LDEENHFYGILTHARLLDMLSDAWNIKNGSYVLTILSDNDRGNLVKMAKIISKYSNIA

GCLTLDVKTGELVRRNLFTLPIGVSRETMTAIVNRLEKKGFVVPEIEDLQSGLTIRSA
EQPGELKD"
CDS complement(414716..415687)
/gene="ldh_1"
/locus_tag="OCPHGMPK_00372"
/EC_number="1.1.1.27"
/inference="ab initio prediction:Prodigal:2.6"
/inference="similar to AA sequence:UniProtKB:O32765"
/codon_start=1
/transl_table=11
/product="L-lactate dehydrogenase"

/translation="MSEEKIHKIILVGDGAVGSTYAFSLVQQGIAQELGIVDIVKERT

QGDAIDLADATPWIAPKTIYSAEYSDAKDADLVVISAGAPQKPGETRLDLVNKNLKIL

SSIVEPIVESGFNGIFLVAANPVDILTHATWRMSGFPKDRVIGSGTSLDTGRLQKVIG

EMEHVDPRSVNAYMLGEHGDTEFPVWSYNNVGGVKVSDWVKAHPEVGENKLEAIHKEV

ADMAYDIINKKGATFYGIGTALAFITKAILNNEHRVLPLSVPMDGEYGLHDLHIGTPA
VVGRHGLEQVIEMPLNADEQAKMEASAKQLKEVMDKAFKETGVKVRQ"
CDS 415868..416425
/gene="pth"

/locus_tag="OCPHGMPK_00373"
/EC_number="3.1.1.29"
/inference="ab initio prediction:Prodigal:2.6"
/inference="similar to AA sequence:UniProtKB:Q6YP15"
/codon_start=1
/transl_table=11
/product="Peptidyl-tRNA hydrolase"

/translation="MKLIAGLGNPGKKYDQTKHNTGFMALDHYLNKNNLDLDKDKFEG

LWTKQKINGEDVIFLEPQTFMNDSGKSIAQVANFFKIAPEDILVIHDDMDMPIGKIRI

RANGKSGGHNGIKSIMACLGTNNFNRLKIGIRHPQKESVVSWVLSPFNDEQQKLMDAA
FDVSENIINDFIKGKNAQYLMNQYN"
CDS 416427..419924
/gene="mfd"
/locus_tag="OCPHGMPK_00374"
/EC_number="3.6.4.-"
/inference="ab initio prediction:Prodigal:2.6"
/inference="similar to AA sequence:UniProtKB:Q7A7B2"
/codon_start=1
/transl_table=11
/product="Transcription-repair-coupling factor"

/translation="MNITELLNKDKDLNNFINKVPKEKRSLLTGVNSVAFSAVLMQML

STWQRPLILVEDSEDKAQMLLDELGNLLPDNMVFSFPVDATIATQTAIASPDELSQRL

QTLKFLTEKRAGIVVVTPQALQYKLSDPRDFTKAKQVFKPEAEFDLDELTAWFTQAGY

RRENIVARPGEFARRGDILDVYPLDQENPFRIEFFGDEVDTVKEFDAATQRSLEEKDI

VSIGPALDRVFSSRNLHEAVKKIKQDMKESIAKEENVKNHFVKAIDLLEADGLPENYA

FLVDYLLPKSFNLIDYLDKNGLLLFDDWQAIKKNVADVDAQNEAFISEEIKAGAMLNS

QKLRHNFEQVVQKVKQAQVIFSLFQKGMGRLKFDQILNFTTREVQQFFSQMPLIKSEL

KRFANEENTVILQADSATRAKQITQNLVDYGVDLPIVKSDQILIKKAQITVGDFAHGF

ALPSLNLVYLTERELFNQRPHHRKRIKTLENAQRLRNYNELKPGDYVVHVNHGIGRFE

GIKTLDVDGKKRDYITITYQHHDQLFVPADQLGLVQKYVASEGKVPHINKLGSAEWAK

TKKRVQSKVEDIADDLIELYAKRESEKGFAFSPDDDLQKEFEDAFPYPETPDQLRSIR

EIKADMESPKPMDRLLVGDVGFGKTEVALRAAFKAIRDGKQVAFLAPTTILAQQHFET

MQDRFKNFPVNCALLSRFQTPAEVKEIIEGVKNGQIDMVVGTHRLLSKDVKFKDLGLL

IVDEEQRFGVKHKERLKELKANIDVLTLTATPIPRTLHMSMVGVRDLSVMETPPTNRY

PIQTYVMEEMPSVVREAVLREMKRNGQVFFLHNRIDDIDKVVSRLEELIPEAKIEYIH

GRMSENQMEDIMYRFSRNEFDILVTTTIIETGVDMPNVNTMIVENADHYGLSQLYQLR

GRIGRSARLAYAYFLYQPNKVLTEIGEKRLSAIRDFTELGSGFKIAMRDLSIRGAGNM

LGKQQHGFIDSVGYDLYAQMLDDAIKKRKGKKVAVKSNAEVQFNLEAYIPDDYIADQE

QKIEFYKKIKGINDQKEAESIADELIDRFGDYPLAVENLLNVSKVKADADLAQVVAIN

QKSDDKLQVIFNNRASEELEGPNIFKALEHVSLRARINMDPQKRMNVMLELKKKMSTR
QLFNELHVFLAAASDIVQR"
CDS 419938..420180
/locus_tag="OCPHGMPK_00375"
/inference="ab initio prediction:Prodigal:2.6"
/codon_start=1
/transl_table=11
/product="hypothetical protein"

/translation="MRIDKFLKVSRLVKRRTVAKEMADQGRVKVNDRVVKSSYDVKVG
DVIEIGFGTKQLKARVLDLRETTKKAEASDLYELID"
CDS 420269..420646
/gene="ftsL_1"
/locus_tag="OCPHGMPK_00376"
/inference="ab initio prediction:Prodigal:2.6"
/inference="protein motif:HAMAP:MF_00910"
/codon_start=1
/transl_table=11
/product="Cell division protein FtsL"

/translation="MQRGPRLYNALSDEERLARLRRKAARKEKEVHRVRRNRIVACFA

IAILIFGLQLIMVHVQTGRINSQIQTNKTELNQVNKTNKKLKAQVADLKDPDYVAKVI
RYKFYYSKSGESIYTLPDKEEDY"
CDS 420646..421002
/gene="pnp"
/locus_tag="OCPHGMPK_00377"
/EC_number="2.7.7.8"
/inference="ab initio prediction:Prodigal:2.6"
/inference="protein motif:HAMAP:MF_01595"
/codon_start=1
/transl_table=11
/product="Polyribonucleotide nucleotidyltransferase"

/translation="MKFPIGLRLNAKVNNITKLGIFVTLPHHHHGLIFHKDFGDKWES

ARNKYSIGDEIRVVIINNREGKLSLSLSQVNNPDLIDPTNEFKDSKDFTPLAKLVESS
TKKIADLKNSIELSDQ"
CDS 421024..422319
/gene="tilS"
/locus_tag="OCPHGMPK_00378"
/EC_number="6.3.4.19"
/inference="ab initio prediction:Prodigal:2.6"
/inference="similar to AA sequence:UniProtKB:P37563"
/codon_start=1

/transl_table=11
/product="tRNA(Ile)-lysidine synthase"

/translation="MTEFTNFFTKNNIEIKNKKFLLAASGGPDSVALFHMLVNFLPDP

SEQLIVAHLDHCLRKDSYLESELLQRLTTAFKVKLIEKSWPLALHPQTGIEARAREFR

YAFLANTGKKYQVDYLLTAHHGDDLIENILLKFIRSGDVAEMNSLQVVGNFNSMKLLR

PLIKYSKDQLLEYDKQNDLDYIEDETNFEDDTLRNRLRHHVVPLLKKETNHLVENAYR

FSESAALLSDCQSSFFESLILPVSFGKALQGRKSDLIDLNKNQLAAFFDYLIYKKWHQ

RVHFDEIRWSKNATFNKENFQLIFYQDYYYLINRNQLTPIDVNKKKIKLDEKFSLNGK

EYMISNDKKLGNLVGYFYGAKSDFLEVGSLPQGSKLRLADGRQTKAKKKFAENGIPLI
LRPYCLTIWQGKNPVYVENVYQNQEYNPDFIRYNVYIYL"
CDS 422403..424529
/gene="ftsH"
/locus_tag="OCPHGMPK_00379"
/EC_number="3.4.24.-"
/inference="ab initio prediction:Prodigal:2.6"
/inference="similar to AA sequence:UniProtKB:Q88Z31"
/codon_start=1
/transl_table=11
/product="ATP-dependent zinc metalloprotease FtsH"

/translation="MKNRRNRLLSNGLFYIIVFVILLAGINWAVGGSGSNGSTENIRY

SQLVKDLKAGKVKSINVQPSNGVYTVTGSYKKAQKSKNQNNTGFGLIPSSQSSSEVTR

FSTTMLQNDSMVKTVSDLAQKSGASISGQGESQSGTWISTIVMLVPTLIFIVMLWMMM

NQGGQGRGGGMMNFGKSHVKPQDPSKNKVRFSDVAGEEEEKQELVEIVEFLKNPAKYT

KLGARIPAGVLLEGPPGTGKTLLARAVAGEAGVPFYSISGSDFVEMFVGVGASRVRDL

FETAKKNAPSIIFIDEIDAVGRKRGNGVSGGHDEREQTLNQLLVEMDGFEGDEGVIVM

AATNRSDVLDPALLRPGRFDRKVLVGRPDVKGREAILKVHAKNKPLAPDVDLKEVARQ

TPGFVGADLENVLNEAALVAARRNKTEITASDIDEAEDRVIAGPAKKDTVISPEERKR

VAYHEAGHAIVGLVLSDSRTVRKVTIVPRGRAGGYNIMLPKEDENIITKKQLMEQVAG

LMGGRAGEEVVVGDKSTGASNDFEQATNIARGMVTQYGMTDVGMTELESPSMQSPYGT

KPYSEATAAKIDEAVKEILDEGHKQAVDIIKSHRETHKIIAEALLKYETLNEKQILSL

FKTGKMPENDENEFPSESKASTYEEAKAAMEKKDQQREETENKDDKEDVHPLPDDHKN
IDDIPLNPPSEKKDSDEHNSDENEDR"
CDS 424600..427311
/locus_tag="OCPHGMPK_00380"
/inference="ab initio prediction:Prodigal:2.6"

/codon_start=1
/transl_table=11
/product="hypothetical protein"

/translation="MVLRMKYLSKKDKKILKLSLISCLTTIVLFFLLSTFTDCNPIFG

GVLYGGDLPNQYLSFFQYYRHLVLGNWASAGYTFSNGLGGDMAGNIAYYVLSPLNFIV

FLFPANKINIAVYVIILLKLGLMSGTFTWLILKWFNFKYQAYAIFLGIAYSLSGYSVA

YAGNVMWFDGLILLPLITYALVRGIKTNKWLAYSILLACAIIFNYYIGYMICIFMVII

FLAYTINNFKDIRLFFHQFLNFAISSIISGLISAIVLLPTLFNLSSNKLAQSDFNSDF

NIKLLITGGKTVSRLFIGDTYNDWPPIFVGTLAAIIFIIYFIDSRNSVKARITNLIIG

IMFVLSIIVRPLYLFWHGGQQTIAYPYRFSFLIVFWILLLAAKELSYQDFKRKDRIIA

TTIYLLLSLMTVYIRRRIGPNNFYEWIAVALVLLFGLLIYFSNKNFVRIILILVGLVE

LSGNAYTGLSHLGMKSDYYPRYVAENNEIISKIPAADKTGRIAKNYELNNDRGEGYTF

NYRGVEEFSSNNDSRISSLMTDLGFSTFRYFYYYQTGTVVTDAIFNVKSFINSSLTNQ

SISPEYVNYGLRDDLKTRPVILKQGDKTVYRNETLPFAFAGNLSNKLKFKDENPVYNQ

NLVLNSLTQSKSNVLDYSTKKARITTNNLSVKYGTVKYHVIKKHKKITKRTEQKYFTI

KRYDKERPGTMSFTYDNLKPNQVGYIRFSKNLMQLVLPLNNYQWNKNPDYRPPFSLTI

NGKQVQLQEYTDQLIGVQADKNGKVDIKMTIDGKGGKFILKYPKFVNIDFSALHDKVK

KARNREMKFTSFKDGYVAGKVTIDKSQNLVTTIPYSKGWQAKVDGKPVKINRTLGVFI
GLKMKPGTHQITLKYRTPGVLVGAILSIIGIISLIVFTLFLKKNKKD"
CDS 427532..428407
/gene="hslO"
/locus_tag="OCPHGMPK_00381"
/inference="ab initio prediction:Prodigal:2.6"
/inference="similar to AA sequence:UniProtKB:P37565"
/codon_start=1
/transl_table=11
/product="33 kDa chaperonin"

/translation="MKDYLVKAIDKTKNLRLITVNAKGLVGEAQKRHDTWSASSAVLG

RTLIGGLLLSAALLKDKDELTVRLLGNGPVGATVVTAKADLTIKGYIQNPHIALPPKK

DGHIDVARAVGEGWLEVTKDQGLKEPYTGQVPIVSGEIAEDFTYYLAKSEQIPSAVGL

SVFVEPNNSIGAAGGFVLQALPGATDEQLAQVEKRIKALPNLSTLFLDGMTPENLAER

ILGTDCKILAKEDVAFSCDCSKEKYSKILATLKPGQLKEMIEKDHGAELVCRFCKEKY
HFTEDELKDVLKKVN"
CDS 428467..429474

```
                     /gene="dus"
                     /locus_tag="OCPHGMPK_00382"
                     /EC_number="1.3.1.-"
                     /inference="ab initio prediction:Prodigal:2.6"
                     /inference="similar to AA sequence:UniProtKB:P9WNS7"
                     /codon_start=1
                     /transl_table=11
                     /product="putative tRNA-dihydrouridine synthase"

/translation="MKNDSWKIRDVEIPNRVVVAPMAGISNAAFRVVCKEFGAGLVVC

EMISDHGIIYRNKKTLEMLTVDPREHPMSIQIFGGSEETLVEAAHYVDTHTAADIINI

NMGCPVPKVTKTDAGARWLLDPNKIYQMVHAVVRNVNKPVTVKMRTGWDQKHIFAVEN

ALAAQEAGASAVAMHGRTRKQMYMGEADWETLKDVADALTIPFVGNGDVTTPEKAKSM

LEDVGADAVMVGRAALGNPWIIKDMVHYLDTGEKLRPQTVEEKVATAKEQLNGLIDLK

GEKIAVPEFRRQAAYYLKGIPRSARTRAKINDVWTKQEVFDLLDDFVEKYEARQKQIN
                     R"
     CDS             429489..431030
                     /gene="lysS"
                     /locus_tag="OCPHGMPK_00383"
                     /EC_number="6.1.1.6"
                     /inference="ab initio prediction:Prodigal:2.6"
                     /inference="similar to AA sequence:UniProtKB:Q97RS9"
                     /codon_start=1
                     /transl_table=11
                     /product="Lysine--tRNA ligase"

/translation="MAKNEMNDQLIVRREKMDEMRENGIEPFGVRKFDRQDLARTLNE

KYSKEDKDELNADMPMTKIAGRMLAKRGKGKVGFADLYDRTGKIQIYVRKDIVGEDNY

KIFKKSDIGDFLGIDGEVMKTDTGELTIRATHVTFLAKALRPLPNKWDGLKDVEQIYR

QRYLDLITNHESYMRFVHRTQIIQAIRNYLNNQDFLEVETPVLHNIPGGAEARPFITH

HNALDIDLYMRIALELPLKRLIVGGMERVYEIGRVFRNEGVDTRHNPEFTELETYAAY

WDFHDVMDEAEGIIRAAAKVVSPDGKINYQGTDIDLGKPFRRVHMVDLIKEKTGVDFW

KEMTDEEAKKIAEEHGIHTEPFWKVGHVINAFFDEFCEKTIVDPTFVYGHPVEISPLA

KKNADDPRFTDRFEIFILGMEYGNAFSELNDPVDQRHRFEEQMEEREAGNDEADMIDE

DYIRAMEFGMPPTGGLGIGIDRLVMLLTDAPAIRDVLLFPTMRPEKVEDVDAEVQEDL
                     KKKNK"
     tRNA            431131..431205
                     /locus_tag="OCPHGMPK_00384"
                     /product="tRNA-Gly"
                     /inference="COORDINATES:profile:Aragorn:1.2"
                     /note="tRNA-Gly(gcc)"
```

```
     tRNA            431245..431320
                     /locus_tag="OCPHGMPK_00385"
                     /product="tRNA-Pro"
                     /inference="COORDINATES:profile:Aragorn:1.2"
                     /note="tRNA-Pro(cgg)"
     CDS             431483..433951
                     /gene="clpC"
                     /locus_tag="OCPHGMPK_00386"
                     /inference="ab initio prediction:Prodigal:2.6"
                     /inference="similar to AA sequence:UniProtKB:P37571"
                     /codon_start=1
                     /transl_table=11
                     /product="Negative regulator of genetic competence
                     ClpC/MecB"

/translation="MEKSYSDSAKNVLEIAKEQAQNFHHRIIGTEHVLLALVIEANGD

AGKLLRERNVTPTLVREEIERYTGYGSSPKATYMEMSPRLSLVLNFAKQKSDELGTPQ

IETKHILLGLLASDQILASLILKNIGVEPRDLSQDINDSFMDDSGEANNIFDVSSATR

TKKGKSLTPNLDKVSVNLNKRAREGAIDPVIGRDKEIKRVIQILSRRTKNNPVLVGEP

GVGKTAVAQGIASAIVNREVPDNLAKKRVMALDMGSLIAGTKYRGEFEDRMKKILKEI

QRDGSVILFVDEMHTLIGAGGAEGAIDASNILKPSLARGDIQMIGATTFDEYQKYIEK

DQALARRFQQVKIGEPSKEETVDILKGLRPKYEKFHNVKIEDEAINDAVEFSTRYIAN

RFLPDKAIDLIDEASAAVKIQAVGKADPRLTKLDTQINDVIHRKEEAAENQNFVQAAK

LRDEESKLTQDRDKLIAKVENKNSKKSIVDSNKIAQIVSEWTGVPVTRMKKSETKRLA

HLESILHERVIGQDKAISAVSRAIRRSRSGIKDENRPIGSFLFLGPTGVGKTELAKAL

AAAVFGSERNIIRVDMSEYMDQVATSKLIGSAPGYVGYEEGGQLSERVRRNPYSVILL

DEVEKAHPDVFNLLLQVLDEGFLTDSKGRKVDFRNTIIIMTSNLGSRSLQEDKTVGFA

ADNEDKNKLQQEKVTAAVKQFFRPEFLNRIDETVVFDSLTKKQLREIVSLMTSHLITR

LARKEVTLKISPAALDVLAKDGFDPEMGARPLRRAIQHELEDVIAEDLISEKVKAGQT
                     VKVGAHQGKLKFTIVDESKPLVKN"
     CDS             434176..437790
                     /gene="rpoB"
                     /locus_tag="OCPHGMPK_00387"
                     /EC_number="2.7.7.6"
                     /inference="ab initio prediction:Prodigal:2.6"
                     /inference="similar to AA sequence:UniProtKB:P37870"
                     /codon_start=1
                     /transl_table=11
                     /product="DNA-directed RNA polymerase subunit beta"

/translation="MNYGSHRTRRSFSRIKEVLKLPNLTDVQTQSYKWFLNEGIREMF
```

DDIMPISDFSGKLSLEFVDYKLLKPKYTLEEARDHDANYSAPLHVTLKLTNHETGEIK

TQDVFFGEFPLMTDSGTFVINGAERVIVSQLVRSPGVYYHSDFDKNGRQIFGATVIPN

RGAWLEYETDAKDLAYVRIDRTRKLPLTVLIRALGFGSDSEVADMFGESDSLRFTLEK

DIHKNPADSRVAEALKDIYERLRPGEPKTTDSSRSLLYARFFDPRRYDLAPVGRYKIN

KKLSLKNRLLRQTLAETLADPDTGEIIAKKGDVVTHEILDKLSPYLDRDDFKMVTYEP

SKEGVLPDPVTVQEIKVYSKVDPERVIKLMSNGHIADDVKHLTPADVLASINYFFNLQ

DNIGTTDDIDHLGNRRIRRVGELLQNQFRIGLARMERVVRERMSIQDISTVTPQQLIN

IRPVVASVKEFFGSSQLSQFMDQNNPLGELTHKRRMSALGPGGLSRDRAGYEVRDVHY

THYGRLCPIETPEGPNIGLINSLATYAIVNKYGFIETPYRRVSWDTHKVTDKIDYLTA

DVEDNYIIAGANAPLNEDGSFKDKIVLARHKEDNLEVTPDKIDYMDVIPKQVVSVTSA

CIPFLENDDSNRALMGANHQRQAVPLINPHAPIVGTGMEYRAAHDSGDALVAKAPGVV

EYVDANEIRIRRDDDTLDKYVLEKFRRSNATKNYNQTPAVKQGERVVADEVIADGPAM

ENGELALGQNPIIAFLTWNMYNYEDAVMISERMVKDDVYTSIHIEDYESEARDTKLGP

EEITREIPNVGEDALKDLDEEGIVRIGAEVQDGDILVGKVTPKGVTELSAEERLLHAI

FGEKAREVRDTSLRVPHGGGGIVQNVQVFTREAGDELPPGVNKMVRVYIVQKRKIQVG

DKMSGRHGNKGTIALVCPEEDMPYLPDGRPVDICLNPMGVPSRMNIGQVLELHLGIAA

KQLGIHVATPVFDGASEDDMWNMVREAGIGKDGKTVLYDGRTGEPFHNRVSVGIMYYL

KLTHMVDDKIHARSIGPYSLVTQQPLGGKAQFGGQRFGEMEVWALEAYGAAYTLQEIL

TYKSDDVVGRVKAYEAIVKGERIPKPGVPESFRVLVKELQSLGLDIKVLDMDHKEIEL

RDMDDDSNDHFNIDTLSKLAEQQEKKKLAEEAAKKDDKSAKPVDQSDSSTSSDDKVSK
"

```
     CDS             437806..441477
                     /gene="rpoC"
                     /locus_tag="OCPHGMPK_00388"
                     /EC_number="2.7.7.6"
                     /inference="ab initio prediction:Prodigal:2.6"
                     /inference="similar to AA sequence:UniProtKB:P37871"
                     /codon_start=1
                     /transl_table=11
                     /product="DNA-directed RNA polymerase subunit beta'"
```

/translation="MIDVNKFESMQIGLASPNKIRSWSYGEVKKPETINYRTLKPEKD

GLFDERIFGPTKDWSCACGKYKGIRYRGIVCDRCGVEVTSAKVRRERMGHIELAAPVS

HIWYFKGIPSRMGLVLDISPRALEEVIYFAAYIVIDAGDTDLEDKQLLTEAEYREKKA

KFGNRFEAKMGAEAVKELLEQVDIDKEVHDLKEELKTATGQKRTRAIRRLDILDAFKN

SGNKPSWMVMDCIPVIPPDLRPMVQLDGGRFATSDLNDLYRRVINRNNRLKRLLDLNA

PRIIVQNEKRMLQEAVDALIDNGRRGRPVVGPGNRPLKSLSHMLKGKQGRFRQNLLGK

RVDYSGRSVIDVSPKLKFYQCGVPRPMALELFKPFVMHELVKRGLASNIKNAKRKIDR

EDDDIWDILEDVIKERPVLLNRAPTLHRLGIQAFEPVLVPGKSIRLHPLACEAYNADF

DGDQMAIHVPLSDEAVAESRLLMLAAHHILAPKDGKPIVTPSQDIVLGNYWLTQAERG

REGEGMIFDSPAEAAIAYANGDIHYHTRIGLAADSMPEKPWPKGYEHGIFVTTYGKLV

FNQIFPKDFFYINDPTQENLTHPVDERYFLQPGEDIHEKLDNMKLGKAFKKGFLSDSI

AQIYKDYKVQRTSDFLDDLKELGYTVCTTSGLTIGVEDIPTITDKDDIVAEARKKVDV

VSKQYRRGLITDEERHDRVISIWNNCKDIVQNEIAQIHAPRNPITIMADSGARGNISN

FTQLAGMRGLMAAPNGGMMEIPVTSNFREGLSVLEMFMSTHGARKGMTDTALKTANSG

YLTRRLVDVAQDVIIREEDCGTDRGLTVHAITEGDEMIEPLFDRLVGRYTSKSVYDPE

THEVICPADVLMDEDMAHKIVDAGVTEVTIRSVFTCNTQHGVCKKCYGMNLATGDDVE

VGEAVGTVAAQSIGEPGTQLTMRNFHNGGVAGAADITQGLPRVQELFEARNPKGRATI

SEVTGEITSIEEDPAEHTRQITVKGQTDTRTYDVPYTASVAVAEGDHVVRGDKLTLGS

IDPKELIRVRDALTTEKYILSEIQKAYRMQGVEIADKHVEVMARQMLQKVRILDPGET

DILPGELMDIGEFKARNREVIISGGIPATAQSVILGITKAALETNSFLSAASFQETTR

VLTDASIRGKNDPLLGLKENVIIGKIIPAGTGMPVYREMEPKVDIPEDEKKKSVYSIA
DIEKKLAAADAEKNDGSAD"

```
     CDS             complement(441545..442222)
                     /locus_tag="OCPHGMPK_00389"
                     /inference="ab initio prediction:Prodigal:2.6"
                     /codon_start=1
                     /transl_table=11
                     /product="hypothetical protein"
```

/translation="MSNSFIYILNFIIGSIIGSHLLVIVQRFETEDFITDKSHCDNCQ

IPLTLLNQIPIFSFLRYQGKCFFCHAYIPIETFYCEILGGLAFMPLNPLTNQISYFFI

IFIFLISIFDYFDQSFPVQILIPLIAVVGFKISYFISDYTPIEWMLISFVILSFLIMN

LKKKFGYGDTIIFIILIFYYNFYIAEYLFLLSSIFLIITHLIARNKKQYPFIPFIFLS
IIFYNFI"

```
     CDS             442390..442797
                     /gene="rpsL"
                     /locus_tag="OCPHGMPK_00390"
                     /inference="ab initio prediction:Prodigal:2.6"
                     /inference="similar to AA sequence:UniProtKB:P0A0H0"
                     /codon_start=1
                     /transl_table=11
                     /product="30S ribosomal protein S12"

/translation="MPTINQLVRKGRHSKTTKSDSPALNYAYNSMKKKMNYNPAPQMR

GVATRVGTMTPKKPNSALRKYARVRLSNLIEVTAYIPGIGHNLQEHSVVLIRGGRVKD
                     LPGVRYHIIRGALDTAGVDGRKQGRSKYGAKKG"
     CDS             442823..443293
                     /gene="rpsG"
                     /locus_tag="OCPHGMPK_00391"
                     /inference="ab initio prediction:Prodigal:2.6"
                     /inference="similar to AA sequence:UniProtKB:P22744"
                     /codon_start=1
                     /transl_table=11
                     /product="30S ribosomal protein S7"

/translation="MPRKGNVAKRDVLADPVYNSKLVTKLINHLMIDGKKAKASSILY

DAFGIIQDKTGKEPVEVFEEAMNNVMPVLEVKARRIGGSNYQIPVEVRPERRTTLGLR

WLVSYARLRNEHTMDERLANEIMDAANNTGSAVKKREDVHRMAEANRAFAHYRF"
     CDS             443323..445419
                     /gene="fusA"
                     /locus_tag="OCPHGMPK_00392"
                     /inference="ab initio prediction:Prodigal:2.6"
                     /inference="similar to AA sequence:UniProtKB:P80868"
                     /codon_start=1
                     /transl_table=11
                     /product="Elongation factor G"

/translation="MANKREFPLDKTRNIGIMAHIDAGKTTTTERILYYTGKIHKIGE

THEGDSQMDWMDEEKERGITITSAATTAQWKDYRINIIDTPGHVDFTIEVERSLRVLD

GAVTVLDAQAGVEPQTENVWRQAETYGVPRIVFVNKMDKIGADFDKSVKSLHERLNAN

AHAVQMPIGSADTFEGVIDLINMVADVYDEDKLGSKWDTIPVPDEYKEEALKRRNELI

EAVADVDDGIMDKYLGGEEISNDELKAAIRKATLNLEFFPVYAGSAFKNKGVQMMLDG

VIDYLPSPLDVKPYVAHDPKTGDEVELMADDKKPFAALAFKIATDPFVGRLTFIRVYT

GSLQSGSYVLNASKNARERVGRLLQMHANSRTEIPEVFSGDIAGAIGLKNTTTGDSLT

DPDHPLILESLQVPDPVIQVSVEPKSKADRDKMDVALQKLTEEDPTFRAETNPETGQT

LISGMGELHLDIMVERMKREFNVEATIGEPQVAYRETFTKEAKAQGKFVRQSGGKGQY
```

```
GDVWIDFTPNEEGKGYEFEDAIVGGVVPREFIPSVDQGLQEAMKNGVLAGYPLIDVKA

KLYDGSYHEVDSSEAAFKVAASLALRNAAAKAGAVILEPIMKVQVTTPEEYLGDVMGS

ITARRGSMEGMEDRAGAKVINSFVPLSEMFGYATTLRSSTQGRGTFTMVFDHYSPTPK
                     SIQAEIIKKRGGEDAE"
     CDS             445690..445998
                     /gene="rpsJ"
                     /locus_tag="OCPHGMPK_00393"
                     /inference="ab initio prediction:Prodigal:2.6"
                     /inference="similar to AA sequence:UniProtKB:P21471"
                     /codon_start=1
                     /transl_table=11
                     /product="30S ribosomal protein S10"

/translation="MASQQIRIRLKSYEHGILDESAAKIVATAERTGAQISGPVPLPT

ERTLFTVLRSPHKNKDSREQFEIRTHKRLIDILNPTPKTVDSLMKLDLPSGVDIEIKL
                     "
     CDS             446025..446654
                     /gene="rplC"
                     /locus_tag="OCPHGMPK_00394"
                     /inference="ab initio prediction:Prodigal:2.6"
                     /inference="similar to AA sequence:UniProtKB:P42920"
                     /codon_start=1
                     /transl_table=11
                     /product="50S ribosomal protein L3"

/translation="MTKGILGRKVGMTQIFTKNGILVPVTVIEATPNVVLQVKTNESD

GYEAVQVGYQDMREVLSNKPAKGHAAKAKTSPKRFIREIRDVELKDYEVGSEITVDSF

SEGDVVDVTGTTRGHGTQGNIKRWGQSRGPETHGSRYHRIPGSMGSIINRVPKGKKLP
                     GHMGGKKVTVQNLVIEKVVPEKNVLLIKGNVPGAKNSLIFVKSAAKAAK"
     CDS             446669..447286
                     /gene="rplD"
                     /locus_tag="OCPHGMPK_00395"
                     /inference="ab initio prediction:Prodigal:2.6"
                     /inference="similar to AA sequence:UniProtKB:P28601"
                     /codon_start=1
                     /transl_table=11
                     /product="50S ribosomal protein L4"

/translation="MANLEIIDQKGKSAGNVDLNEEIFGIEPNESVVFDAIIRQRAGK

RQGTSAVKNRSAVRGGGKKPWRQKGTGRARQGSIRAPQWRGGGTVFGPTPRSYKMDMP

RKARRLAMKSVLSQKVADKDLIILDQLTLEAPKTKELKAILDNANVSGKVLVVSDDKN
                     VQLSGKNLPKVKVVPVNGLNVVDAVDYQKLVLTQDAIKRIEEVLA"
     CDS             447286..447582
                     /gene="rplW"
                     /locus_tag="OCPHGMPK_00396"
                     /inference="ab initio prediction:Prodigal:2.6"
```

/inference="similar to AA sequence:UniProtKB:P42924"
/codon_start=1
/transl_table=11
/product="50S ribosomal protein L23"

/translation="MDARDIILRPVVTEKSMNLMDDKKYTFDVLVSATKTQVRNAVEE

IFDVKVKNVNIMNVRGKEKRVGRYTGKTARRRKAIVTLTEDSNDIKIFNDNKEN"
CDS 447602..448438
/gene="rplB"
/locus_tag="OCPHGMPK_00397"
/inference="ab initio prediction:Prodigal:2.6"
/inference="similar to AA sequence:UniProtKB:P60430"
/codon_start=1
/transl_table=11
/product="50S ribosomal protein L2"

/translation="MAIIKYKPTTNGRRNMTSSDFAEITKKKPEKTLLESQSHTAGRN

SYGHITVRHRGGGHKQKYRIIDFKRNKDDVKAVVKAIEYDPNRTANIALLHYTDGIKA

YILAPKGLKVGTVVESGPNADIKPGNALPLSAIPAGTEIHNIELKPGKGGQLVRSAGT

VAQVLGNDGKYTLVRLQSGEVRKILSTCRATIGSVGNEQHSLIQLGKAGRKRWLGKRP

QSRGSVMNPNDHPHGGGEGKAPVGRPQPMTPWGKKSRGIKTRNSKARSEKLIIRHRKG
NK"
CDS 448460..448747
/gene="rpsS"
/locus_tag="OCPHGMPK_00398"
/inference="ab initio prediction:Prodigal:2.6"
/inference="similar to AA sequence:UniProtKB:Q2FW10"
/codon_start=1
/transl_table=11
/product="30S ribosomal protein S19"

/translation="MSRSIKKGPFADASLLKKIEAQEGSEKKQVIKTWSRRSTIFPSF
VGFTIAVYDGRKHVPVYITEDMVGHKLGEFVPTRTFRGHKVADKATTTVGK"
CDS 448768..449121
/gene="rplV"
/locus_tag="OCPHGMPK_00399"
/inference="ab initio prediction:Prodigal:2.6"
/inference="similar to AA sequence:UniProtKB:P42060"
/codon_start=1
/transl_table=11
/product="50S ribosomal protein L22"

/translation="MAEQISSAKAEARTVRIAPRKARLVVDLIRGKSVAEALAILQFT

PRAASPIVEKVLKSAIANAEHNYDLESANLYVSEAYVNEGATLKRFRPRAKGMASPIN
KRTSHVVVVVSEKND"
CDS 449136..449804
/gene="rpsC"
/locus_tag="OCPHGMPK_00400"

```
                     /inference="ab initio prediction:Prodigal:2.6"
                     /inference="similar to AA sequence:UniProtKB:P21465"
                     /codon_start=1
                     /transl_table=11
                     /product="30S ribosomal protein S3"

/translation="MGQKINPNGFRLGVNRDWEAKWYADKNYADTLNEDLRIRKFISE

KLADASVSTVEIERAANRINISIHTAKPGMVIGKGGKEVEALRKQLSALTKKNVHINI

VEIKKPDLDAKLVGEGIARQLEARIVFRRATRQATQRSMRSGAKGIKVQTAGRLNGAD

MARREWHTEGSVPLHTLRADIDYAWVEAATTYGQIGVKVWINRGEILPQRKNKPSKKA
                     KGGN"
     CDS             449807..450244
                     /gene="rplP"
                     /locus_tag="OCPHGMPK_00401"
                     /inference="ab initio prediction:Prodigal:2.6"
                     /inference="similar to AA sequence:UniProtKB:P0A475"
                     /codon_start=1
                     /transl_table=11
                     /product="50S ribosomal protein L16"

/translation="MLVPKRVKHRREFRGKMRGEAKGGKTIAFGEYGLEAVESHWITN

RQIEAARIAMTRFMKRGGRVWIRIFPQKSYTAKGVGVRMGSGKGAPAGWVAVVKRGKI
                     MFEIGGVSEDVAREALRLASNKLPIKTKFVKKSSEVGGESNEG"
     CDS             450234..450431
                     /gene="rpmC"
                     /locus_tag="OCPHGMPK_00402"
                     /inference="ab initio prediction:Prodigal:2.6"
                     /inference="similar to AA sequence:UniProtKB:P04457"
                     /codon_start=1
                     /transl_table=11
                     /product="50S ribosomal protein L29"

/translation="MKAKDIRALTTDQMLEKEKQYKEELFNLRFQQATGQLENTARLR
                     QVRKNIARIKTILSEKELSKN"
     CDS             450454..450720
                     /gene="rpsQ"
                     /locus_tag="OCPHGMPK_00403"
                     /inference="ab initio prediction:Prodigal:2.6"
                     /inference="similar to AA sequence:UniProtKB:P12874"
                     /codon_start=1
                     /transl_table=11
                     /product="30S ribosomal protein S17"

/translation="MSETNERNNRHVYQGRVVSDKMDKTITVVVDTYKNHPVYKKRIK
                     YSKKYYAHDENNEAKIGDTVRIMETRPLSHAKRYRLTKIVKKSI"
     CDS             450754..451122
                     /gene="rplN"
                     /locus_tag="OCPHGMPK_00404"
                     /inference="ab initio prediction:Prodigal:2.6"
                     /inference="similar to AA sequence:UniProtKB:P12875"
```

```
                     /codon_start=1
                     /transl_table=11
                     /product="50S ribosomal protein L14"

/translation="MIQHESRLKVADNSGARELLVIKVLGGSKRKTGNIGDIVVAAVK

QATPGGVVKKGDVVKAVIVRTKSGARREDGSYIKFDENAAVVINADKSPRGTRIFGPV
                     ARELREHDFMKIVSLAPEVL"
     CDS             451143..451382
                     /gene="rplX"
                     /locus_tag="OCPHGMPK_00405"
                     /inference="ab initio prediction:Prodigal:2.6"
                     /inference="similar to AA sequence:UniProtKB:P04455"
                     /codon_start=1
                     /transl_table=11
                     /product="50S ribosomal protein L24"

/translation="MFVKTGDKVKVIAGKDKGKEGTVLSVNAKTNRIVVKGVNKIKKH
                     EKPSQANANGGVVEKEGSIHASNVKVIAKKEDNNK"
     CDS             451397..451939
                     /gene="rplE"
                     /locus_tag="OCPHGMPK_00406"
                     /inference="ab initio prediction:Prodigal:2.6"
                     /inference="similar to AA sequence:UniProtKB:P12877"
                     /codon_start=1
                     /transl_table=11
                     /product="50S ribosomal protein L5"

/translation="MANSLVEKYSNEIAPAMNKKFNYDSVMEIPKIDKIVLNMGVGDA

VSNAKNLDEAVEELTLISGQKPLITKAKKSIANFRLREGMSIGAKVTLRGDRMYDFLY

KLINVSLPRVRDFRGISSRSFDGRGNYTLGIKEQLIFPEIDYDKVNRVRGLDVVIVTT
                     ANTDEEARELLTEFGMPFAK"
     CDS             451954..452139
                     /gene="rpsZ"
                     /locus_tag="OCPHGMPK_00407"
                     /inference="ab initio prediction:Prodigal:2.6"
                     /inference="similar to AA sequence:UniProtKB:Q2FW19"
                     /codon_start=1
                     /transl_table=11
                     /product="30S ribosomal protein S14 type Z"

/translation="MAKTSQIVRNHRPAKFSSREYTRCERCGRPHSVYRKFKLCRICL
                     KDLAHKGQIPGVKKASW"
     CDS             452164..452562
                     /gene="rpsH"
                     /locus_tag="OCPHGMPK_00408"
                     /inference="ab initio prediction:Prodigal:2.6"
                     /inference="similar to AA sequence:UniProtKB:P12879"
                     /codon_start=1
                     /transl_table=11
                     /product="30S ribosomal protein S8"
```

```
/translation="MVMTDPIADYLTRIRNANMAKHSSVEIPASSMKKSLSEILKNEG

FIRDYQVEDDNKQGMIKIFLKYGPNNERVISGLKRISKPGLRNYVSAENLPKVLNGLG
                     IAIISTSAGVITDKEAREKNVGGEVIAYVW"
     CDS             452587..453117
                     /gene="rplF"
                     /locus_tag="OCPHGMPK_00409"
                     /inference="ab initio prediction:Prodigal:2.6"
                     /inference="similar to AA sequence:UniProtKB:Q2FW21"
                     /codon_start=1
                     /transl_table=11
                     /product="50S ribosomal protein L6"

/translation="MSRIGLKVIEVPEKVTVTKNGDDITVKGPKGELTRYFDPRITFE

QKDGEIHFSRSNEADKALHGTERANLASMIEGVTDGYVKKLTLVGVGYRAVAQGKKLT

LNVGYSHPVVFEAPEGVTVKTPSATSIEIEGISKQVVGQFAAEIRDVRPPEPYKGKGI
                     RYEDEYVRRKEGKTGK"
     CDS             453145..453504
                     /gene="rplR"
                     /locus_tag="OCPHGMPK_00410"
                     /inference="ab initio prediction:Prodigal:2.6"
                     /inference="similar to AA sequence:UniProtKB:Q2FW22"
                     /codon_start=1
                     /transl_table=11
                     /product="50S ribosomal protein L18"

/translation="MISKPDKNKLRLKRHKRIRGKISGTAERPRLSVFRSNKNIYAQL

IDDVEGVTLASASTNDKNISAEGSKMEQAAEVGKALAETAAKKNIKSVVFDRSGYLYH
                     GRVQALADAARENGLEF"
     CDS             453522..454046
                     /gene="rpsE"
                     /locus_tag="OCPHGMPK_00411"
                     /inference="ab initio prediction:Prodigal:2.6"
                     /inference="similar to AA sequence:UniProtKB:P21467"
                     /codon_start=1
                     /transl_table=11
                     /product="30S ribosomal protein S5"

/translation="MANRNDSRRDSRKDRKKDDIEDQLVAINRITKVVKGGRRMRFAA

VVIVGDRKGHVGFGTGKAQEVPEAIRKAVEAGKKRMIKVPTVGTTIPHEVMGHYGSGN

IMLKPAEAGSGVAAGGAVRIIMDLAGISDVTSKSLGSNTPINVIRATMDGLSKLKTRE
                     DVLKLRESAKSLED"
     CDS             454059..454241
                     /gene="rpmD"
                     /locus_tag="OCPHGMPK_00412"
                     /inference="ab initio prediction:Prodigal:2.6"
                     /inference="similar to AA sequence:UniProtKB:P0A0G2"
                     /codon_start=1
```

/transl_table=11
/product="50S ribosomal protein L30"

/translation="MDLKVTLIKSVAHRLPKQRKIVKALGLGKVNSTVVLPDNAATRG
ALLKIAHLISVEEVNK"
CDS 454267..454707
/gene="rplO"
/locus_tag="OCPHGMPK_00413"
/inference="ab initio prediction:Prodigal:2.6"
/inference="similar to AA sequence:UniProtKB:P19946"
/codon_start=1
/transl_table=11
/product="50S ribosomal protein L15"

/translation="MKLNELKPNEGSRRNRKRVGRGTSSGYGKTAGRGQKGQLARTGG

KTRLGFEGGQMPLFRRMPKRGFKNVNRKEYAIINLNDLNRFDDGSEVTIDTLKSSGLV
KKELAGVKLLANGELKVKLTVKVNKASEAAKKAVEAAGGTVEVI"
CDS 454707..456002
/gene="secY"
/locus_tag="OCPHGMPK_00414"
/inference="ab initio prediction:Prodigal:2.6"
/inference="protein motif:HAMAP:MF_01465"
/codon_start=1
/transl_table=11
/product="Protein translocase subunit SecY"

/translation="MFSTLKNAFKDKDIRSKIFFTLFILLLYRIGANITVPGVNAKAI

TRVAQTGLVPMLDTVSGGGLDTYSIFSLGVSPYITAQIVIQLLQMDIVPKLVEWGKQG

EVGRRKTNQVTRYLTLVVAFVQSLGITLGFNVLTEMGLVKTQTPQTYIEIAIIMTAGT

MLLTWLGDEITDKGLGNGVSVIIFAGIIARLPNGIYQLFKDFIINNSASDRWQGILFF

IAIIIAILLVTQFVTWFQQADLRVPIQYTRRAATSGSESFLPLKVNVSGVIPVIFASS

FIITPATILMAFQRSHGNEQWFKICNQIFSLQTTPGALIYTLLIIILFTFFYAFVQVNP

EKLAENLQKQGAYIPSVWPGKDTQKYVSKILIRLSTVGAVFLGLVALLPQLATNIFGL
PSSIGLGGTSLLIVIGVVLELARQVDGLLMKREYVGFIR"
CDS 456014..456667
/gene="adk"
/locus_tag="OCPHGMPK_00415"
/EC_number="2.7.4.3"
/inference="ab initio prediction:Prodigal:2.6"
/inference="similar to AA sequence:UniProtKB:P27142"
/codon_start=1
/transl_table=11
/product="Adenylate kinase"

/translation="MINLILLGLPGAGKGTASESIVDKYHLAHISTGDMFREAMANKT

PVGLEAKSYIDKGDLVPDEVTAKLVEERLKQPDTKNGFILDGFPRTTVQAELLEGITK

RLEKPLTNVIAIDVDEDVLIKRLSARYICKNCGATYNKISNPTKVEGTCDRCGGHEFF

QREDDKPEVVKNRLEVNKKMNTPLRDFYEEKGILSTVNGEQTPEKVFEDIDKILSKD"
     CDS   456741..456962
                     /gene="infA"
                     /locus_tag="OCPHGMPK_00416"
                     /inference="ab initio prediction:Prodigal:2.6"
                     /inference="similar to AA sequence:UniProtKB:P20458"
                     /codon_start=1
                     /transl_table=11
                     /product="Translation initiation factor IF-1"

/translation="MAKEDVIEVEGKVVDTLPNAMFKVELENGATILAHVSGKIRMHY
                     IRILPGDRVTVELSPYDLTKGRITYRFIK"
     CDS   456977..457093
                     /gene="rpmJ"
                     /locus_tag="OCPHGMPK_00417"
                     /inference="ab initio prediction:Prodigal:2.6"
                     /inference="similar to AA sequence:UniProtKB:Q2FW29"
                     /codon_start=1
                     /transl_table=11
                     /product="50S ribosomal protein L36"

/translation="MKVRPSVKPMCEHCKIIKRHGRVMVICSANPKHKQRQG"
     CDS   457127..457474
                     /gene="rpsM"
                     /locus_tag="OCPHGMPK_00418"
                     /inference="ab initio prediction:Prodigal:2.6"
                     /inference="similar to AA sequence:UniProtKB:P20282"
                     /codon_start=1
                     /transl_table=11
                     /product="30S ribosomal protein S13"

/translation="MARIAGVDLPRNKRVVVALTYIYGIGESTAKKICKDAGISEDIR

TNDLTPEDQEKLRSEVDKYRVEGDLRREVSLNIKRLVEIGSYRGIRHRRGLPVRGQNT
                     KNNARTRKGSKRK"
     CDS   457499..457888
                     /gene="rpsK"
                     /locus_tag="OCPHGMPK_00419"
                     /inference="ab initio prediction:Prodigal:2.6"
                     /inference="similar to AA sequence:UniProtKB:Q2FW31"
                     /codon_start=1
                     /transl_table=11
                     /product="30S ribosomal protein S11"

/translation="MAAKKTARKRRVKKHVESGVAHIHSTFNNTLVMITDVQGNAVAW

SSAGALGFKGSRKSTPFAAQMAAEAAAKSAMDQGMKHVEVSVKGPGAGREAAIRALQA
                     AGLEITAIRDVTPVPHNGSRPPKRRRV"
     CDS   457934..458872
                     /gene="rpoA"
                     /locus_tag="OCPHGMPK_00420"

```
                     /EC_number="2.7.7.6"
                     /inference="ab initio prediction:Prodigal:2.6"
                     /inference="similar to AA sequence:UniProtKB:P20429"
                     /codon_start=1
                     /transl_table=11
                     /product="DNA-directed RNA polymerase subunit alpha"

/translation="MIEFEKPNITVVDQEEAYGKFVVEPLERGFGTTLGNSLRRVLLT

SIPGTALSYIQIDGVLHEFSTIPGVREDVTKIILNLKKLELKSLSDEEKIAEIDVTGP

AIVTAADLKVDSDIEVLNPDQYICSIADGGHLHMNVAIKNGRGYVPASENKTDDMPIG

VIPVDSLFSPIKKVNYQVESARVGKRDDYDKLTLEIWTDGSITPNDALSFAAKILVEH

FKVFMSTDMDAQFDDVMVEKEDDKNEKKLEMTIEELDLSVRSYNCLKRAGINTVQELT
                     DKSEADMMRVRNLGRKSLEEVKNKLAELGLSLRQDD"
     CDS             458899..459282
                     /gene="rplQ"
                     /locus_tag="OCPHGMPK_00421"
                     /inference="ab initio prediction:Prodigal:2.6"
                     /inference="similar to AA sequence:UniProtKB:P20277"
                     /codon_start=1
                     /transl_table=11
                     /product="50S ribosomal protein L17"

/translation="MAYRKLGRDSAHRKAMLREMTTQLIMNERIVTTETRAKEIRKTT

EKMITLGKRGDLSARRKAAAFVRNEIADIHEEKDAVVVKSALQKLFSDIAPRYKDRNG
                     GYTRMYKLANPRKGDAAPMVIIELV"
     CDS             459462..460310
                     /gene="ecfA1"
                     /locus_tag="OCPHGMPK_00422"
                     /EC_number="3.6.3.-"
                     /inference="ab initio prediction:Prodigal:2.6"
                     /inference="similar to AA sequence:UniProtKB:Q1GBJ0"
                     /codon_start=1
                     /transl_table=11
                     /product="Energy-coupling factor transporter ATP-
binding
                     protein EcfA1"

/translation="MKDNIVTVEHLSFTYKDSEEPAVKDVSFSIPKGTWTTLVGHNGS

GKSTIARLLNGILLPDDNPNTLINIDGITLTEKTMWDIRDRVGIVFQNPDNQFVGATV

EDDVAFGLENRQVPRTKMKSIVQDVLNQVEMIAFQESEPQYLSGGQKQRVAIAGILAI

GPKLIIILDESTSMLDPAGKSKILRLIKKLQKENELTIFSITHDINEAVHADQILVLNK

GSLLASGSPGDIFKDDSLIRSAGLDLPLFYKIKNKLINEGIGIPQEVNTEEKLVNYLC
                     QLNSKM"
     CDS             460286..461152
                     /gene="ecfA2"
```

/locus_tag="OCPHGMPK_00423"
/EC_number="3.6.3.-"
/inference="ab initio prediction:Prodigal:2.6"
/inference="similar to AA sequence:UniProtKB:Q1GBI9"
/codon_start=1
/transl_table=11
/product="Energy-coupling factor transporter ATP-
binding
protein EcfA2"

/translation="MSIEFKNVDYIYAPETPFQTQGLADISFKIKSGSFVAIAGHTGS

GKSTLMQHFDGLLLPSSGAVNIDDKTITSNTSSKSLKEIRKKVGLVFQFPESQLFEET

VLKDVMFGPLNFGFSEQKAKEQAIEWIEKVGLSEQVMNKSPFELSGGQMRRVAIAGVM

AYEPDILCLDEPAAGLDPEGQKQMFDIFKNYQQEGHTVILISHNMDDISQYADDMLVL

EHGRLIKHASPKEVFSDPNWVKEHFLDEPATSKFARELEKGGFQFSEMPLTIDSLVNK
IANELKAKGGNE"
CDS 461149..461946
/gene="ecfT_1"
/locus_tag="OCPHGMPK_00424"
/inference="ab initio prediction:Prodigal:2.6"
/inference="similar to AA sequence:UniProtKB:Q035B4"
/codon_start=1
/transl_table=11
/product="Energy-coupling factor transporter
transmembrane
protein EcfT"

/translation="MSKIIIGRYIPGNSVVYKMDPRGEIIATFLFIVIIFLANNWLSY

LFLSFFTLLAVGATKLKPKVFWDGVKPLIWLILFTSVLQLFFTTGGKIYWHWGIFNVS

EYGIQNSIFIFIRFVMIILISTVLTLTTTSLEIADGMEWILRPLGYLKVPVAQIALVM

SIALRFVPTLLDQAVRIMNAQRARGADFNSGGLIKRIHAIIPILIPLFISSLTIAIDL
ATAMEARGYREGTKRTRYRVLKWSKYDWINLGYFALLTLILLLTRTY"
CDS 461950..462741
/gene="truA"
/locus_tag="OCPHGMPK_00425"
/EC_number="5.4.99.12"
/inference="ab initio prediction:Prodigal:2.6"
/inference="similar to AA sequence:UniProtKB:P07649"
/codon_start=1
/transl_table=11
/product="tRNA pseudouridine synthase A"

/translation="MTTRYKLTMAYDGHLFHGFQIQPKERTVQGVIEEALKKMTKGKR

VVVHGSGRTDAGVHAKGQVIHFDYPGKEIPAKNMMLALNALMPIDTVFFDSEIVNSDF

HVQYSTKGKWYRYIVDQHRFTDPFNRFYTGHYPYPLDIDLMQKAAKDLIGKHDFTSFA

ASGGQIVDKVRTIYYVNITSDPDNKKIIFDFIGNGFLYNMVRILVGTLLEIGNKKRPV
DDIPRVLAAKDRQEVRTTAPASGLYLQHVFYEEVPKKYRLDLKKD"
CDS 462844..463287
/gene="rplM"
/locus_tag="OCPHGMPK_00426"
/inference="ab initio prediction:Prodigal:2.6"
/inference="similar to AA sequence:UniProtKB:Q2FW38"
/codon_start=1
/transl_table=11
/product="50S ribosomal protein L13"

/translation="MRTTPLAKTSDIKRKWYVIDATDVSLGRLSTAVASILRGKNKPQ

YTPNVDTGDYVIVVNAGKLKLTGKKATDKIYYHHSDYRGGLRATPAGELLAKNPVRLV
ELSVKGMLPKNTLGHQEFMKMHVYAGEDHEHAAQKPEKLDINELI"
CDS 463301..463696
/gene="rpsI"
/locus_tag="OCPHGMPK_00427"
/inference="ab initio prediction:Prodigal:2.6"
/inference="similar to AA sequence:UniProtKB:Q2FW39"
/codon_start=1
/transl_table=11
/product="30S ribosomal protein S9"

/translation="MAQQAAYAGTGRRKDSVARVRLVPGNGQITINKEDVTKYIPYPN

LVQDMKQPLALTETEGQYNVEVNVNGGGFSGQAGAIRLGIARALLEVDPDFRGPLKKA
GMLTRDPRMKERKKPGLKKARKASQFSKR"
CDS 463833..464537
/gene="yxlF"
/locus_tag="OCPHGMPK_00428"
/EC_number="3.6.3.-"
/inference="ab initio prediction:Prodigal:2.6"
/inference="similar to AA sequence:UniProtKB:P94374"
/codon_start=1
/transl_table=11
/product="putative ABC transporter ATP-binding
protein
YxlF"

/translation="MTDLLTTNNLRKVFKDQVVLEGIDLHVPRNQVYCLLGPNGAGKT

TLMKILTGMISKTSGQVIFDNHEWSRKDLKNIGSLIENAPLYGNLTAQENLKVITKLR

GVDDSVISNILNTIGLTNTGHKLSKDFSLGMKERLGIGLALVGEPKLLILDEPTNGLD

PVGIQQLRNLIAEFKNKDITIIISSHMLSEVEHLADTIGIIGGGKLLLEKPYDHQENL
EKLFNDTLEKAGVLNV"
CDS 464530..465258
/locus_tag="OCPHGMPK_00429"
/inference="ab initio prediction:Prodigal:2.6"
/codon_start=1
/transl_table=11

/product="hypothetical protein"

/translation="MFNYCSIEILKTKRTIIRKLVWIFPLLVSIITFLFFASTGYVVQ

SIINQWSFIWVNLFLALEIGLIDRHERNSTDYKMILSSPDDLFQYELGRILHDVLLSL

ITTCTLGILVILLSFLMPMKVSLGACLLALFGIFAATLWEIPLYSWLSRVTNLYVSVG

LAFAGSLIGIWVDNLSLGKLFPFTWSAIMPVSLIQMHVNGLLVEDTDKIPNNSWTLLA
SLILFIVLSGLSAAAFKKQVVKNA"
CDS 465251..465985
/locus_tag="OCPHGMPK_00430"
/inference="ab initio prediction:Prodigal:2.6"
/codon_start=1
/transl_table=11
/product="hypothetical protein"

/translation="MLKQVVEAENIKIRRSLLLYLHLIVLFLFPVLFGLYYGSRKQVI

VPANMIIGFYEILAIASPIIISVVVCLVFDREEKAGNFKNWLTEPISKGWMIQAQLSY

YWIWYVIEIMGTSLIFSGVLFGIYQIRFSLIKIALTSIAFSLCGIVQYELTQIIALRW

GIGDALILGFFGTVISLLSITSLLDFVWPIIPWAWQIRLTTFWQTNIDLGLVKLSLIE
YLIPLMLTCLIIFWGKRYFDNWQGKN"
CDS 466086..466952
/gene="qorB"
/locus_tag="OCPHGMPK_00431"
/EC_number="1.6.5.2"
/inference="ab initio prediction:Prodigal:2.6"
/inference="similar to AA sequence:UniProtKB:P39315"
/codon_start=1
/transl_table=11
/product="Quinone oxidoreductase 2"

/translation="MKYLITGATGNLGEKVTRWLRTMTPENNIRVGIHNLKKANKFDD

LDVEKVKLDYFDLDTLEKAVSGVDLVIYIPSITYDLQRRITEFENVLQAVKKAKAKLI

FVSFFADQENNPFVMSPFYAYVPRRLASSKVEYSIVKNSLYADPLVPYLEELIKRKNI

IYPVGAQPLSFITRNDSAHAIACLAMQAIMRDQGQSYLLSMDKNYNMVELSYIMSRIT

DHKIGYAPVTITEFAKIYAQEGDGKELASMYAGGAKGLLSATSDDFHRLTGREPEEME
HFLRNGYQIARL"
CDS complement(467205..467789)
/locus_tag="OCPHGMPK_00432"
/inference="ab initio prediction:Prodigal:2.6"
/codon_start=1
/transl_table=11
/product="hypothetical protein"

/translation="MTKVAVVFADGCEEVEGLSVVDVLRRLNIDCDMVGLDKKEIDGD

```
HHILLTCDKVVDDSLLDYDLVAFPGGRTGALNLRNNKKLADLMIQRNKAGKWDAAMCA

APIALGHYGLLEGANYTCYPGFEKQIEEECPNGHFSTDITVVDQEHKIITSRGPATAW
                     AYAYTIARTLGHDTSDLEKGMLYDYLAKNINASL"
     CDS             468065..468754
                     /gene="gpmA_2"
                     /locus_tag="OCPHGMPK_00433"
                     /EC_number="5.4.2.11"
                     /inference="ab initio prediction:Prodigal:2.6"
                     /inference="similar to AA sequence:UniProtKB:O51602"
                     /codon_start=1
                     /transl_table=11
                     /product="2,3-bisphosphoglycerate-dependent
                     phosphoglycerate mutase"

/translation="MATEVYLVRHGETMFNQLNKVQGWADSPLTVKGINDLKVTAANL

SQVHFDKMYSSDLKRAIDTVHLIADTNEVSEIGKIKKLPAFREVFFGTFEGDDIDETW

RKVAKAGGMKPTSDVVKIIQTLGIHDFREATKKADPRHLAEDAEALDDRMDQAISQLA

QETKGLGRVLVVSHGDFIKTLGIKYWDQASGDHDIPFPDNGSVTRGIIDDNGKFRIIN
                     YGVKNTDIPNL"
     CDS             complement(468781..469104)
                     /gene="czrA"
                     /locus_tag="OCPHGMPK_00434"
                     /inference="ab initio prediction:Prodigal:2.6"
                     /inference="similar to AA sequence:UniProtKB:O31844"
                     /codon_start=1
                     /transl_table=11
                     /product="HTH-type transcriptional repressor CzrA"

/translation="MSLNTSLVNEAAKIYKVLSNSRRISILFFLRNSSKEVDVSTIAT

TLELAQPVVSKQLGILEKYNLVKHHKHGTHVFYFLNDPDVLSMIDAMIEHVEHTAKEH
                     PSNLY"
     CDS             469202..470086
                     /gene="czcD"
                     /locus_tag="OCPHGMPK_00435"
                     /inference="ab initio prediction:Prodigal:2.6"
                     /inference="similar to AA sequence:UniProtKB:O07084"
                     /codon_start=1
                     /transl_table=11
                     /product="Cadmium, cobalt and zinc/H(+)-K(+)
antiporter"

/translation="MKDKSSLRYFWVTLLNIIITIAEFIGGAISGSLALLSDAVHNLS

DVGSIVLAFVANLISKRKRNTSKTFGYDRAEILAAFTNGIILIVISIYLFIEGIQRFS

HPEPIRGTIMLIVSLIGLAANVISMFVVMKEAKTSLNAKATFLNMLSDAITSVAVVLG

AIVISIWKIYWIDPVLTIAASVFLLKEAYEVTIKAANILMETNPSIDLNKVNELVLSC
```

PHVNNIHHVHVWQYSDNVTMLDAHINVDKNLDAVQLEKIYTEIAKKLKSLGINHVTLQ
AECTRGIDDKMIFADKED"
CDS 470152..470409
/locus_tag="OCPHGMPK_00436"
/inference="ab initio prediction:Prodigal:2.6"
/codon_start=1
/transl_table=11
/product="hypothetical protein"

/translation="MKFDTDDLKASLKNYQQKASDQFKDFAENDWPDIKDHVTEKGQQ
AGDYLKKNGGKVYQNSVKAFKKGVRDLDQWVNGTSVHKDDK"
CDS 470411..470596
/locus_tag="OCPHGMPK_00437"
/inference="ab initio prediction:Prodigal:2.6"
/codon_start=1
/transl_table=11
/product="hypothetical protein"

/translation="MKVNIWVALIVLVVGLWNLYYAYNRYQEKKITKRGSKADAISFA
ILGTIFTIGGIIMLFMH"
CDS 470615..471292
/gene="gpmA_3"
/locus_tag="OCPHGMPK_00438"
/EC_number="5.4.2.11"
/inference="ab initio prediction:Prodigal:2.6"
/inference="similar to AA sequence:UniProtKB:Q5XB88"
/codon_start=1
/transl_table=11
/product="2,3-bisphosphoglycerate-dependent
phosphoglycerate mutase"

/translation="MVKLVLVRHGESIANRDNVYTGWNDVPLSKKGIEQAKDAGLKVA

KIPDFVPTHIHTSVLSRAIMTANIIADVCSFLYLPITKTWRLNERHYGALRGINKDVS

KKIFGKEQVLKWRRGFDSVPPLLTQPVQDRRYQKYDMRLMPQGESLHQTQERLMPYFW

DHIAPELMSGHDQLIVAHGSSLRALIKKIEGISNEDIVKVEVPNAEPIVYTFDSDLHI
IKKEILH"
CDS 471402..472337
/locus_tag="OCPHGMPK_00439"
/inference="ab initio prediction:Prodigal:2.6"
/codon_start=1
/transl_table=11
/product="hypothetical protein"

/translation="MRKIFLLRGAPGSGKSSFIARHHLQPYAISRDEIRLLLADLTVY

YEESTDHLHQVIPRHVTVRTEQMVDNLVQHKMAYGETVIVDGTHITPDKIEHFRPWVE

KYRYELFVVDLMQNNSLESLLRRNQTRMHYDWVKPDVIKMMYEQYEANPEVPSWAYSI

LPNGMERALSQREKNLDHYSHVVCIPDKVKPEDFPHVHISNFYFSFNDEFTRKYGTYR

NVITLAKTQDEVIEQFRLPYFVFKFHHKHFLISAYPIRNEMLDPIRKVKGVWSYSTGL
YNVADFVKEFPENEHQHVHQFNLSKIDPTRLLHIW"
CDS complement(472404..473753)
/gene="pepC"
/locus_tag="OCPHGMPK_00440"
/EC_number="3.4.22.40"
/inference="ab initio prediction:Prodigal:2.6"
/inference="similar to AA sequence:UniProtKB:Q04723"
/codon_start=1
/transl_table=11
/product="Aminopeptidase C"

/translation="MSKEITNDLINDFENDFNSSKANKIAARAAQENGIFKASQNLQT

KIDLDPTFSIEIDTGKVANQKQSGRCWMFSALNTMRHSIQKNFKIKDFELSQNYTNFW

DKFEKSNWFFENVIATADKDLGDRKVAFLFATPQQDGGQWDMLCGIIEKYGIVPKKVY

PETANATNSSALNDTLNTLLRKDGLELRKMVQAGKSEAEVQERKNEMLREVYRILAIS

LGVPPKQFDFEYKDDDGNYHRDADLSPQDFFKKYVNWDLSEYISTINAPTKDKPFHKV

FSVEYLGNVEGGRQVRHLNLPVDEMKDLIIKQLKNGEVVWFGSNVVKDSERQAGLLDT

ELYKRDDLFDVNFDMSKADMLDSGESMMDHAMVITGVDLVDGKPTKWKIENSWGEKPG

FKGYFVMSDKWFDKFVYQAVINKKYLSDDLKKAFDEGSKAPIQLLPWDPMGALAKNY"
CDS 473948..474499
/gene="dut"
/locus_tag="OCPHGMPK_00441"
/EC_number="3.6.1.23"
/inference="ab initio prediction:Prodigal:2.6"
/inference="similar to AA sequence:UniProtKB:Q2YRG4"
/codon_start=1
/transl_table=11
/product="Deoxyuridine 5'-triphosphate
nucleotidohydrolase"

/translation="MKKRGFEIVSKYHNAGLHLPQRQTIASAGYDFECAQDIVLPSIW

CMNFVRIFRLIRNSHELNERDLELAEKVLKPFLVPTGIKAYMPEDEVLILANRSSNTY

KRNVTLPNGIGVIDSDYYNNEKNEGEIYFQMINYGVRPLKIKKGERIGQGIFMPYLKA
DNDEPVQRQRLGGFGSSGTRNEE"
CDS 474499..475878
/gene="radA"
/locus_tag="OCPHGMPK_00442"
/EC_number="3.6.4.-"
/inference="ab initio prediction:Prodigal:2.6"
/inference="similar to AA sequence:UniProtKB:P37572"
/codon_start=1
/transl_table=11
/product="DNA repair protein RadA"

/translation="MAKVKTRYKCRNCGYISASYLGRCPNCGAWNQFEEETQEVKKVS

TKATASRLMTKIGNNDPVKLNEVKAEKEKRIVTPFEELNRVLGGGIVPGSLVLIGGDP

GIGKSTLMLQITGALAKEHSVLYVSGEESASQIKMRADSLGVSNSGILLYPETNMQNI

REQIDEIKPDFLVIDSIQTMNEPSLDSMVGSASQVREVTSELMKIAKNDQITTFVIGH

VTKEGAIAGPKIMEHMVDTVLYFEGDGHHSYRILRSVKNRFGAANEIGMFEMKNEGLT

EVNNPSAIFLDERLPNSTGSAVVVSLEGTRPLLADIQALVTPTAFGYAKRTTSGLDFN

RVALLLAVLEKRGNLMLQNQDAFLTATGGIKLNEPAIDLAICMAVASSYKNKEISSTD

CFVGEVGLTGEIRRVNQIEARVKEAAKVGFKRIFIPKHNLTTELKNNSEIEVIGVASL
PQALKLVFN"
CDS 475960..477459
/gene="gltX"
/locus_tag="OCPHGMPK_00443"
/EC_number="6.1.1.17"
/inference="ab initio prediction:Prodigal:2.6"
/inference="similar to AA sequence:UniProtKB:Q97NG1"
/codon_start=1
/transl_table=11
/product="Glutamate--tRNA ligase"

/translation="MAKQKIRVRYAPSPTGHLHIGNARTALFNYLFARHNKGTMVLRI

EDTDQKRNVEGGSKSQMENLHWLGIDWDEGPDKGGDFGPYRQSERKDIYNKYIKQLID

EGKAYYSYKTEEELEAQREEQRAMGIAPHYTYEYEGMTADEIKQAQADAEAKGLKPVV

RIHIPENRTYAWDDMVKGKVSFESDTIGGDFVIQKRDGMPTYNFAVVIDDHLMEITHV

LRGDDHVANTPKQLVVYEALGWEPPKFGHMTLIINSETGKKLSKRDESVLQFIEQYRD

LGYLPEAMFNFITLLGWSPVGESEIFSQRELIKSFDPKRLSKSPAAFDQKKLEWINNQ

YVKKADRDVLLDLALNNLQEAGLVGKEISPEKMEWVRQLVNIYAVQMSYTKQIVDITK

IFFEEAPELSDAEIEEIKKDDARPVIEEFKKQLNAIPRFTAVQVMNAIQATRHETGVK
GRKLFMPIRIAATRSMVGPGIGEAIELMGKEKVNKHIDLTLKQLSDLGL"
CDS 477831..478307
/locus_tag="OCPHGMPK_00444"
/inference="ab initio prediction:Prodigal:2.6"
/codon_start=1
/transl_table=11
/product="hypothetical protein"

/translation="MLELNEPATIKNTRKFFEKDLERYETVCAGRYSVQGLNFENIKV

NSSNNTSAVMNRYNQIITYSQRLACLKPAIDNCTNEPNKPYKRVLELRYLKHISAWNV

```
ANNIGYSHTQYGVILNRAFLQFADVYLLEQLKQGVKDILDLHVYHGVTNCYTMLET"
     CDS             complement(479597..480130)
                     /locus_tag="OCPHGMPK_00445"
                     /inference="ab initio prediction:Prodigal:2.6"
                     /codon_start=1
                     /transl_table=11
                     /product="hypothetical protein"

/translation="MTDERKWVQFTVDKNTFKKGLTPTEAIILKAIETLDRGQGCFAT

NAYFAEYFNLHPVTVSKNINSLKDKGFITVVLKRQNTNKTKRIIKTIKMSHYTEQSQV

IGVINYINGMFKEEHDFEPIKPTTEIKKAIQQKIKEYRSQKELIQYLKIHRDNFLSTH
                     GVSLWLTGQLTKEQLNM"
     CDS             complement(480168..480488)
                     /locus_tag="OCPHGMPK_00446"
                     /inference="ab initio prediction:Prodigal:2.6"
                     /codon_start=1
                     /transl_table=11
                     /product="hypothetical protein"

/translation="MEENNLINMSIQEVREKLIAKKENFKLNYAELSAMANVSRGTLT

NFINGNDCISSKTFFKIIEWLSLEIMTFTTMKNGQNTTIFLQEVPHVQHINKYTPQPI
                     EWIK"
     CDS             complement(480599..480913)
                     /locus_tag="OCPHGMPK_00447"
                     /inference="ab initio prediction:Prodigal:2.6"
                     /codon_start=1
                     /transl_table=11
                     /product="hypothetical protein"

/translation="MKVILDPEERQKIQSMVIEAVHDGIKQATNFKPYLNRKEVAKYF

GVADSTISYWATLGMPIAVIDGRKLYGKQSITEWLKSKEQPKKEQNKKPLTIAVINGK
                     KK"
     CDS             complement(480916..481212)
                     /locus_tag="OCPHGMPK_00448"
                     /inference="ab initio prediction:Prodigal:2.6"
                     /codon_start=1
                     /transl_table=11
                     /product="hypothetical protein"

/translation="MNKILFIKHGKNLAENIGGLHAYVEETDQASISFPLTVICNGKA

HSYSKKEIELAIFINSEKNKIIVWRSEYGLNEMDLTNDKVNTLGSLLNSLFCEV"
     CDS             complement(481217..481600)
                     /locus_tag="OCPHGMPK_00449"
                     /inference="ab initio prediction:Prodigal:2.6"
                     /codon_start=1
                     /transl_table=11
                     /product="hypothetical protein"
```

/translation="MKEETETMKPTNKKLTKFEKQVLKCIPSGRKHPITSNKLAIINN

TTKRNITYTIQKLRIKGVLIGSSRLKDKSGYYRIQTMNEFFEVVGMLNSERNTLSKTI
SAMMNTFNSGTTLSGQQDLFEFDED"
CDS complement(481551..481838)
/locus_tag="OCPHGMPK_00450"
/inference="ab initio prediction:Prodigal:2.6"
/codon_start=1
/transl_table=11
/product="hypothetical protein"

/translation="MKYTDHEFNKHDDNYSNKLNYLKESIKFAENESNYRIDGTLYPT
STIWRDVAKTALELADLADWFDRYYENDAKETREFYERRNRNNETNQQEIN"
CDS complement(481857..482021)
/locus_tag="OCPHGMPK_00451"
/inference="ab initio prediction:Prodigal:2.6"
/codon_start=1
/transl_table=11
/product="hypothetical protein"

/translation="MKRIYKAFKDWLYLPDGNEAELFNIFLFVVLGLAALVAVVTFTR
TLGMFLALIK"
CDS complement(482005..482217)
/locus_tag="OCPHGMPK_00452"
/inference="ab initio prediction:Prodigal:2.6"
/codon_start=1
/transl_table=11
/product="hypothetical protein"

/translation="MPNTEYFNFKQAMRYLGIQSYTGLKDLINAGLPISIVGKSKKIK
KTDIDKFMKVHTVVTTTGKNKHEENL"
CDS 482354..483070
/locus_tag="OCPHGMPK_00453"
/inference="ab initio prediction:Prodigal:2.6"
/codon_start=1
/transl_table=11
/product="hypothetical protein"

/translation="MNRIKQLREQKGLSQRDFIKSFNLFLKENANKYDGKPGIKAVSF

ATGSRWENGLNKPTSSMWQALADFFGVYVPYLQGAYSKVEILKVLQEYYLRYYIGDYS

TDDIEDLIYTDIGDVVDDFVISKKIKPWNIKKENVLLSKEEVSSTKFWWEHFQVVFDH

IAIIWLLTKPSLNATKRDVADALIDALSGEQNNMLLTRRMKFIDKYLYFMKGKTIKSI
YDFEHPHSLDGKNHYIDEIH"
CDS 483478..484638
/gene="intQ"
/locus_tag="OCPHGMPK_00454"
/inference="ab initio prediction:Prodigal:2.6"
/inference="similar to AA sequence:UniProtKB:P76168"
/codon_start=1
/transl_table=11

/product="Putative defective protein IntQ"

/translation="MKQNKDITRYKTQNGKKRYRFKVYVGKDEETGNSIQARKQGFKS

YDDALKSYFEIKDKVAKGTYTGNENKKHKFSELYEMWLENYKNTVKVSTLSNTKLFFR

LHILPELGNLYIEKITTFKCQKIVNKWFKELPKTYKVYTNLASNIFKFAVNNDLIAKN

PMKSVIKPKLQVEKKPFTNFYSKNELNIFLKEAKECKGLKVYMLFRLLAYTGMRRGEA

LALKWSDIDFSNQTIKINKTVTTAEHAALIIGTPKTKASYRTIAIDRQTIKYLSGWRK

EQRKQLFQLGFNALSDDQLIFSNGDNTLIRQATVRKWDQSISKHAGLKYITVHGFRHT

HASLLFEAGTPMQDVKERLGHSSITTTMNIYTHVTKSAKKKTADNFAQFMEG"
CDS 484778..486190
/gene="cysS"
/locus_tag="OCPHGMPK_00455"
/EC_number="6.1.1.16"
/inference="ab initio prediction:Prodigal:2.6"
/inference="similar to AA sequence:UniProtKB:Q06752"
/codon_start=1
/transl_table=11
/product="Cysteine--tRNA ligase"

/translation="MKVFNTLTRQKEEFKPLVAGKVSMYVCGPTVYNYIHIGNARSVI

AFDTIRRYFEYRGYDVKYVSNFTDVDDKMINEARKEHTTVGELANRYIKAFMEDTEAL

NIEPATLHPRATHEIKEIIDFVQDLIDKGFAYEVDGDVYYRAKKFPHYGQLSDQNIAE

LEEGASEHINEEEQNKKEDPIDFALWKAQKEDDEIAWDSPWGKGRPGWHIECSVMSTK

YLGETIDIHGGGQDLEFPHHENEIAQSEAKTGKKFVNYWMHNGFVTVGKKQEKMSKSL

HNFVTVHDILKEIDPQVLRFYMSSVQYRRPINYSEDGLKQAETVLKRYQNTLRNLDAR

LADEQEPLEDNTLRDNLTQAKAEFIEAMDDDFNVQNALSIMYDLASNLNTHLQKDQVD

KPALKKAKKLLLDWLEIFGVSFNEKQADDEDEIEAMVAKRDEARKNKNWAESDRLRDE
LQAKGIVIEDTPQGTRWHRA"
CDS 486183..486626
/gene="mrnC"
/locus_tag="OCPHGMPK_00456"
/EC_number="3.1.26.-"
/inference="ab initio prediction:Prodigal:2.6"
/inference="similar to AA sequence:UniProtKB:Q81J58"
/codon_start=1
/transl_table=11
/product="Mini-ribonuclease 3"

/translation="MLKPKVLTEKKTNPDALGPLTLAYLGDGVYEVYIRRHLVKGGIV

KPQMLQRYATHYVSAKAQAALITKMQDLNMLDENELAAFKRGRNAKSHTHAKNTSINT

YKLSTGFEAMFGYLDLLGKKERVDELANWCIDQVEQGGLDNYEFE"
CDS 486613..487368
/locus_tag="OCPHGMPK_00457"
/EC_number="2.1.1.-"
/inference="ab initio prediction:Prodigal:2.6"
/inference="similar to AA sequence:UniProtKB:Q7A794"
/codon_start=1
/transl_table=11
/product="Putative TrmH family tRNA/rRNA
methyltransferase"

/translation="MSSNNDEFVYGRHAGIDFLKTQDANLINKVFLQNGVQEEFANQV

YQLARKKNLVVQNAPKNKLDQMVNHENHQGLVLTVAPFEYADLDKLLDKFDQDGKDPF

ILMLDSIEDPHNLGSILRTCDATGVDAVIIPKRRATGLTSVVAKTSTGAIDYVPVARV

NNLVQTTKMLKKRGYWIFGTAMKGEDYRKWNANGKTVLVIGNEGKGISPLLLKQMDQT
LTIPMVGHVQSLNASVATGVLLYGMFNSRHPEK"
CDS 487551..488414
/gene="licT_1"
/locus_tag="OCPHGMPK_00458"
/inference="ab initio prediction:Prodigal:2.6"
/inference="similar to AA sequence:UniProtKB:P39805"
/codon_start=1
/transl_table=11
/product="Transcription antiterminator LicT"

/translation="MLLIKRVFNNNSALVELGNNREAVVVGNGIAFKKTEGKEIDTSK

VENIFYLENSATKRTIFSLLKNTPVDIAVTTMHVVDHAHRKYHYELFDYFYLSLSEHI

LGVYHRILDNTYQASQIPDIKDEYPLENKIAQESVKIIERDLKINLPVSERKNIMLHL

INAKIPEKKQFNREKNMINIEEMLHIVQVILDQHNIYRLASNNDEYERFMIHLQYLFR

RLEQGEKFRSSDITKKVKDELISEYPESFAVVKEIDEQLKQDFQWKISDEEKLYLIVH
IQRIYEKSSKY"
CDS 488424..488783
/gene="lacF_1"
/locus_tag="OCPHGMPK_00459"
/EC_number="2.7.1.207"
/inference="ab initio prediction:Prodigal:2.6"
/inference="similar to AA sequence:UniProtKB:P11502"
/codon_start=1
/transl_table=11
/product="PTS system lactose-specific EIIA
component"

/translation="MVSKEEISMVGFTIVAYAGDAKTDLLNALKLAREGKFEKAEKLI

EEANNSLVDAHNAQTKLMSQEANGEELETTFIMSHGQDTLMTTMLLRDEVKYFIDLYK
QNLALRAEIEQLKEEMN"
CDS 488802..490505

/gene="lacE_2"
/locus_tag="OCPHGMPK_00460"
/inference="ab initio prediction:Prodigal:2.6"
/inference="similar to AA sequence:UniProtKB:P24400"
/codon_start=1
/transl_table=11
/product="PTS system lactose-specific EIICB
component"

/translation="MDGLVKRIEKAQPFFRKVATNQYLRAIRDGFIALMPIIIFSSFF

LLIANVPQIWGFVWPKKVATALNLFYNLSMGFLSLMAASTVAKALTGSLNLKLPKTNQ

IPVAGVQYTAQISFAFVAIDTLLNKGNLYLATDMIGTKGLICAFLVAFIVPNIYYFCF

KHNVTITLPDVVPQNIAQAFKNIIPFALATTFFWAFTLIFRALTNMNLAAWIIKSLTP

LFTAADGYVGLAIVYGAMAFFWFIGIQGPSIVEPAVTAIYLTNVEANLRAFNSGNIQG

ANHILSQGIQMFVATLGGTGATLVVTFMFAFLSKSKQLKAVGRASTIPVIFGVNEPIL

FGAPLILNPIFFIPFIFAPILNVWIFKIFVDVLHMPSFIYNLPWTTPPTLGLWMGTGF

NILALLLGILLLVVDSLLYYPFFKAYDASMLAQEEKKEELEEKDDKKSVVTSAEPKFN

ESKIPLGKTKDQQALNVLVLCAGGGTSGILAKSLNKLAKEDKLPLNAAAAAFGSHHDL

ISGMDVVILAPQMDTMKDELAKECKQNNARMITTTGKQYIYMTQHANECLKLLINDIN
K"
CDS 490516..491943
/gene="lacG_1"
/locus_tag="OCPHGMPK_00461"
/EC_number="3.2.1.85"
/inference="ab initio prediction:Prodigal:2.6"
/inference="similar to AA sequence:UniProtKB:P11546"
/codon_start=1
/transl_table=11
/product="6-phospho-beta-galactosidase"

/translation="MSTLLKRVKKLPKNFVWGAATAAYQVEGNTNLDGKGKIMWDDYL

KSQGNFSPNPACDFYHRYPEDLKLAHNLGLNAVRISIAWTRIFPNGYVTNKPNEAGVK

YYHRLFHECLKNNLVPYVTLHHFDSPKTLFDQGDWLNREMINEFVKYADFCFQEFAEV

DNWFTINELISLAMCQYIQGTFPPNKLFDVSDAIQAQHNELVAHAKVVNLFKAKGYKG

RIGLIHVIQPVYPATNSEADRHAADLRDAFMNRFLLDGTLLGFYSDRTQHLIKEILVA

NNAELKILPEDLEILKQASKQNDMLGINYYQNQTVASYEGPSETYHNGNGKKGSSIFA

FHGVGKDVRNPAIPTTDWDWNIYPEGLYDVLKRIEHDYPDYPVIYITENGMGAKEAWD

SSKQYLDDDYRIDFIDQHLAAILKARNEGVNVQGYFLWSLQDQFSWSNGYNKRYGLIY

```
                     VDFASQDRHLKRSALWFKALSKTMQ"
     CDS             492089..492646
                     /locus_tag="OCPHGMPK_00462"
                     /inference="ab initio prediction:Prodigal:2.6"
                     /codon_start=1
                     /transl_table=11
                     /product="hypothetical protein"

/translation="MEKVNEVYLIRRVKEKQDMKALHELLDRYRPLINGAKMNFFIRN

FDNDDWEQEALIMCYQSCCTYDEDRADNFGAYYRTKFYNHLRSLLRYELAQKRTAFTK

SISYDNVVQKNLIKEEVEEMHITPRKEMYQKFIKKLSDKEVIALKVFLGELTIEEASQ
                     KTKYSRYQLLQAKARCKAKMQKELF"
     CDS             492714..492932
                     /locus_tag="OCPHGMPK_00463"
                     /inference="ab initio prediction:Prodigal:2.6"
                     /codon_start=1
                     /transl_table=11
                     /product="hypothetical protein"

/translation="MADEVIKTELLDRHMKEVFDWSDSDMPVRDALWDYFMEKNGRDT
                     MKTESDMLPFLKDSDDKIEAFVNENLKK"
     CDS             493055..494170
                     /gene="xylB_1"
                     /locus_tag="OCPHGMPK_00464"
                     /EC_number="2.7.1.17"
                     /inference="ab initio prediction:Prodigal:2.6"
                     /inference="similar to AA sequence:UniProtKB:P35850"
                     /codon_start=1
                     /transl_table=11
                     /product="Xylulose kinase"

/translation="MDYIIGMDIGTTASKGVLYQENGKKIVESTIPYPLIQEKVDQAE

EDPQVIFDAVQKIIFDLSKKASGKVKAISWSSQMHSLIGLGKNKQLLTNSITWADNRS

REVVTAAEKSGLANSIYQRTGMPPHPMAPVYKLLWIQEEMPELFAQVQKWIGIKEYII

WRLTGKMMTDTTMAAGTGLLNLNNLSWDEDLLSQITLEKEKLPILDKPETVVGGIIPN

YIQKLGLTDQTKIILGASDGYLSTIGVGVLNDKSFALNIGTSGAVRVIASKAIVDSEN

RFFCYPVDKKHYLLGGPVNNGGIVFEWARKTIFGPDQTAEDFINVAESVPAGSNGLIF
                     HPYLGGERAPIWNAQARGSFIGLTRNHTKPQMARTVF"
     CDS             494191..494565
                     /gene="xylB_2"
                     /locus_tag="OCPHGMPK_00465"
                     /EC_number="2.7.1.17"
                     /inference="ab initio prediction:Prodigal:2.6"
                     /inference="similar to AA sequence:UniProtKB:P35850"
                     /codon_start=1
                     /transl_table=11
                     /product="Xylulose kinase"
```

```
/translation="MGAARGLREKIGEPEALRVTGGFVKSDFVRQLIADIFNLPVIII

KNDQSGTLAAMFLAQVGMKREANLEKIVKKIDESNVYFPNPKNVAVYQDIIPIYREVE
                     HDLDQSYEKISSFQEKYPRLFE"
     CDS             complement(494612..495751)
                     /gene="garK"
                     /locus_tag="OCPHGMPK_00466"
                     /EC_number="2.7.1.165"
                     /inference="ab initio prediction:Prodigal:2.6"
                     /inference="similar to AA sequence:UniProtKB:P23524"
                     /codon_start=1
                     /transl_table=11
                     /product="Glycerate 2-kinase"

/translation="MKIIIAPDSFKGSLTAKEVANSIYTGLKRALPSAEYIEIPMADG

GEGTMQALVDATNGKIFTKSVTGPLNTSVTAHYGILGDKETAVIEMAEAAGLQYVNDQ

TRNPLITTTYGVGELIKTVLDTGIRKIIVGLGGSCTNDGGAGMAQALGVKLLNLQGQE

FPLGGGNLNHLVRIDNSKIDPRLKDTEIILASDVTNPLTGKNGASSVFGPQKGATPEM

VKELDQNLHHFAAVVRRDVKQNYENTPGAGAAGGLGFGLLTFTNATIQSGVEAVLEMT

NFKEKTKNADFIFTGEGATDFQTKFGKTPYGVAIAAKEVAPTSTVICLTGNIGKKVNE
                     LYGPIDAIFATESGAKDLKQAIKDSQHDLAQISENIGRLIKNIYL"
     CDS             complement(495852..497612)
                     /gene="dxs_1"
                     /locus_tag="OCPHGMPK_00467"
                     /EC_number="2.2.1.7"
                     /inference="ab initio prediction:Prodigal:2.6"
                     /inference="similar to AA sequence:UniProtKB:P9WNS3"
                     /codon_start=1
                     /transl_table=11
                     /product="1-deoxy-D-xylulose-5-phosphate synthase"

/translation="MNKHPEFLLNKINSPKDLKKLDLKELEKLASEIRTLILEKDAAE

GGHLGPDLGIVEATIAYHYVFDAPKDKIIWDVSHQTYPHKMLTGRAYAWLDPDKYEDV

TPYSNPDESPYDYYAVGHTSTSVALATGMARARDMMGKHENITALIGDGSLTGGLAFE

GFNNAADEKHNLIIVVNDNQMSIDNNVGGVVTALKKLRESNGQAEDNPFTAMGLDYKY

VGQGNDIKSMIDAFKAVKDIDHPIVLHINTLKGKGYEPAVENEASHHWVLPFDLKTDK

TTVPAPKTPNASTVILDLLKKHIENQENILAINAAIPGVFGLGAIKKNYPKNYKDVGI

AEQESVAFAAGAVKEGITPVLFENSTFLQRAYDQLSHDVAANDLPVVMIVAGGGITAA

SKTHLGIFDNTMVANWPNWEYLAPTTLNELKSMLEWAVKQRKHPVAIKSPVNPLPEGN

VVSEDYSTIHYDVKSGSKVAIIGLGDFYQLGEKVVDLLKKDNINATLINPKSAAHLDT
```

DTLDDLQKDHELVVTLEDNSIDGGFGQKVASYYGPTKMMVKNYGAKKEYTDNEPKGSI
                     YKRDHLMPEQIASEVLEIIK"
     CDS   complement(497711..498958)
                     /locus_tag="OCPHGMPK_00468"
                     /inference="ab initio prediction:Prodigal:2.6"
                     /codon_start=1
                     /transl_table=11
                     /product="hypothetical protein"

/translation="MNKKQNTSHFFAWWVFIASCLISLVGFGLIVNTIGLFFEPISKS

FHVGRASVALMTTLQNAAAAITLLFAGKIMTKLNLRWLLTFCFATIGLSMLSLSVAQS

LTHFYIAWIIIGICQPIAITLSIPVLLGNWFNEKLGTVMGIALGVSAFGGTIFNPIIG

DIITNFGWRGGFICEGLLILGILVPAAFFLREKPNEKHHAYGKVKNIPEKVLETTGIS

LKAALKTPVFYALAFAMLALQFVSGSVQHISGHITSQGLPLTIGASVVSGVMLGAAAG

KISIGFMLDRFNTRLTLLIYSLFGLLGWSGEIFFKTSLPLIASAFILGLGQGVCLVAL

PYLIREEFGAKDYSTILSVINMTGAFAMSASVYLNGLLFDTTGSYNFGWAINSAAFAL
                     SFIALFLTLKKQATSQELEESID"
     CDS   complement(499054..500070)
                     /gene="hepT"
                     /locus_tag="OCPHGMPK_00469"
                     /EC_number="2.5.1.30"
                     /inference="ab initio prediction:Prodigal:2.6"
                     /inference="similar to AA sequence:UniProtKB:P31114"
                     /codon_start=1
                     /transl_table=11
                     /product="Heptaprenyl diphosphate synthase component
2"

/translation="MLEKRNNIKNKFTFWDSFPQVQKDMIEINQIILKHINSVPGLLG

QALEDTFAVPGKMLRPACVMLFGAFGPDATQKHDDLQKIATSIETLHNATLIHDDIID

ESSMRHGRPSIQTKYGKHIAVYAGDYLFALSLTLLSENAQSLHSVIADGKTMQNILVG

ETEQYNNAYNINISEKEYLNQIKGKTGVLFGLTCFIGAYESGIKVTKALKAKQFGEYL

GQAFQLRDDILDYTTTSSTFKKPVLLDVKDGIYSGPLIFALQNDNNNHLHELVKMGKD

LTQDQLHEIENLVNHLGGVERAQKLADQYTEKALASLKKHWPDNENRALIEQLTGRLL
                     KRQY"
     CDS   500516..500806
                     /locus_tag="OCPHGMPK_00470"
                     /inference="ab initio prediction:Prodigal:2.6"
                     /codon_start=1
                     /transl_table=11
                     /product="hypothetical protein"

```
/translation="MKLKKSLLTIGVVVTLGLSASVVSSNKVSAAEVGNTETTTFEIK

NNVQSFNQEHLFLESKAGGWLNHIAHTGYWGGGPLGGGESPTNPIKEYYPWR"
     CDS             500957..502132
                     /locus_tag="OCPHGMPK_00471"
                     /inference="ab initio prediction:Prodigal:2.6"
                     /codon_start=1
                     /transl_table=11
                     /product="hypothetical protein"

/translation="MKKISYSKILTIQIVLNIVFTLLIFNQIGIFFFIFQEIILILVL

FLKKKNIYLSVVFLPFIFMYTYGLILYTIAQTNLKLTLPIYIVYFTGMLLILIPVVKE

KFGKIKQELIRLISLIWLVLISIHSVGIFPIVTREQNELLYAINASGILYAFVTFICV

YILLKLWGYDFHFNLHVKNTGMRYYGVLLLSLLFIIWYTFFSIFLGLAQSSNEIFNNW

NVSLINPDYSAKYINVVEIVLNSLDPAIYEEVERYGYIIILLVIFKSKKYQMQGAIFI

SALLFSLSHIGNFFTTDMSFNDVISQTIVAFGLGCFLAVMLLYTGKVWINILIHFSFD

FLVFSITDIGYLTVSIFNKNNGWLLKSSIELCILLFATFIFAKFNKKILKMNIEELI"
     CDS             502306..502455
                     /gene="rpmG2"
                     /locus_tag="OCPHGMPK_00472"
                     /inference="ab initio prediction:Prodigal:2.6"
                     /inference="similar to AA sequence:UniProtKB:Q2FY22"
                     /codon_start=1
                     /transl_table=11
                     /product="50S ribosomal protein L33 2"

/translation="MAVKKAALACTECGSRNYSIAASKNRTQRLELKKFCKHCGKQTL
                     HKETR"
     CDS             502463..502633
                     /gene="secE"
                     /locus_tag="OCPHGMPK_00473"
                     /inference="ab initio prediction:Prodigal:2.6"
                     /inference="protein motif:HAMAP:MF_00422"
                     /codon_start=1
                     /transl_table=11
                     /product="Protein translocase subunit SecE"

/translation="MFKFFKSVNQTMAKVSWPTWKQNRRDTGVVVISSILFGAYLGLL
                     DLLFSYLTQLFL"
     CDS             502729..503280
                     /gene="nusG"
                     /locus_tag="OCPHGMPK_00474"
                     /inference="ab initio prediction:Prodigal:2.6"
                     /inference="similar to AA sequence:UniProtKB:Q06795"
                     /codon_start=1
                     /transl_table=11
                     /product="Transcription termination/antitermination
```

protein NusG"

/translation="MVETSEKKWYVLHTYSGYEEKVKKDLLSRAQSMGMQNYIFRVIV

PEEQKTETVRGKKQEVDEKVFPGYVLVEMVMTDESWYVVRNTPNVTGFVGSHGGGSKP

SPLYDEEIERILKSQGQPAKEQKADYEVGETVTIIDGAFSGMSGKITEINPEKDKLYV
SIDMFGRETNAELDYNQVKEFDE"
CDS 503411..503836
/gene="rplK"
/locus_tag="OCPHGMPK_00475"
/inference="ab initio prediction:Prodigal:2.6"
/inference="similar to AA sequence:UniProtKB:P0A0F2"
/codon_start=1
/transl_table=11
/product="50S ribosomal protein L11"

/translation="MAKKVINVVKLQIPAGAATPAPPVGPALGQAGINIVGFTKDFNA

RTADQKGMIIPVVITVYEDRSFEFITKTPPAAVLLKKAAKVDKGSGEPNTKKVAKVTK
DQVKEIAETKMKDLNAADVEAAMRMIEGTARSMGFTVED"
CDS 503934..504626
/gene="rplA"
/locus_tag="OCPHGMPK_00476"
/inference="ab initio prediction:Prodigal:2.6"
/inference="similar to AA sequence:UniProtKB:Q99W68"
/codon_start=1
/transl_table=11
/product="50S ribosomal protein L1"

/translation="MAKHGKRYLEAAKKVDSTKFYSVDEAMKLAKETSYANFDATIEV

AYNLSVDPKQADQQIRGALVLPNGTGKSKKVVVFAEGPQADQAKEAGADEVGSDDLVE

KVQNGYLDFDVVIATPMMMAKVGRLGRILGPKGLMPNPKTGTVTMDVAKAVENQKAGQ

VEYRVDKQGLIHAPIGKASFDADKLAQNFDALRDVILRARPASAKGQYIKSVAVSATF
GPGIHLDPLNLD"
CDS 504757..505401
/gene="yecS_2"
/locus_tag="OCPHGMPK_00477"
/inference="ab initio prediction:Prodigal:2.6"
/inference="similar to AA sequence:UniProtKB:P0AFT2"
/codon_start=1
/transl_table=11
/product="L-cystine transport system permease
protein
YecS"

/translation="MLNYIFSILPSLFSGAWLTIEIFFWTTVVSIPLGLLVAFGLHSN

LKLVKAILKFYVWIIRGTPLLLQLIFIFYGLPTVGIVFPRYEAALVAFIINYAAYFAE

IFRGGLQAVPVGQFEAAQVLGLSRWQTINRIVRPQVVKIVLPSIGNELINLVKDSSLV

YVIGLGDLLRAGNVATARDVSLVPLVLVGCVYLAMTAVLTALLRVVEKKSNVWK"
     CDS   505388..506035
                     /gene="artM_1"
                     /locus_tag="OCPHGMPK_00478"
                     /inference="ab initio prediction:Prodigal:2.6"
                     /inference="similar to AA sequence:UniProtKB:P54537"
                     /codon_start=1
                     /transl_table=11
                     /product="Arginine transport ATP-binding protein
ArtM"

/translation="MSGNKVLELKKISKSFGTRKIIDKLDLSLDQGDILSIIGPSGAG

KTTLLRLIAGLESADSGKFIHNGKEFDPTDRNNHLVGMVFQDYNLFPNLSVMDNITLA

PIMVNKMSKSDAEKAAEPVLKQLDLEKFKNLYPYQLSGGQKQRVAIARALQMKPEILC

YDEPTSALDPELVGKVKEIFLKLKQTGMTQVVITHDHQFGKAVADKILKVEPIEK"
     CDS   506068..506823
                     /gene="artJ"
                     /locus_tag="OCPHGMPK_00479"
                     /inference="ab initio prediction:Prodigal:2.6"
                     /inference="similar to AA sequence:UniProtKB:P30860"
                     /codon_start=1
                     /transl_table=11
                     /product="ABC transporter arginine-binding protein
1"

/translation="MIVITALGLYGCQRQESSWQKIKERGTLVIGVDDSFVPMDFRQK

NGKLVGFDVDLATAVCKKIGLKPDFQTIDWSMKETELRNGTIDVIWNGYTKTKARAKK

VAFSRPYLENDQILVTRKKDKIDNYRQMKGKVLGVQTGSSGADDLDNYPNVLKKRVKS

IVLYDTYNNAFIDLKAGRITGLLIDGVYANYYLAHSKYQKEFKKEKLPYPSEKFSVGI
                     NKKDKILKDKINQALEHLEKTGELEKIRQKWFN"
     CDS   507023..507523
                     /gene="rplJ"
                     /locus_tag="OCPHGMPK_00480"
                     /inference="ab initio prediction:Prodigal:2.6"
                     /inference="similar to AA sequence:UniProtKB:Q5XCB5"
                     /codon_start=1
                     /transl_table=11
                     /product="50S ribosomal protein L10"

/translation="MSKAIIAKKEKLVDDFAAELKEAKAILVIDYLGLTVEEVTNLRK

DLRDSNVKMKVIKNTYLKRAAEKAGIEGLDDTFVGPTAVVYTADADDITEPARIVSKY

EDDIDALSIKGGMLEGKVASQEEIKKLAAIPGREGLLSMLVSVLQAPVRNFAYAVKAV
                     ADSKDE"
     CDS   507567..507929
                     /gene="rplL"

/locus_tag="OCPHGMPK_00481"
/inference="ab initio prediction:Prodigal:2.6"
/inference="similar to AA sequence:UniProtKB:P02395"
/codon_start=1
/transl_table=11
/product="50S ribosomal protein L7/L12"

/translation="MALDTEKIIEDLKGASILELNDLVKAIEDEFGVSAAAPVAAAGA

AGAGAEKTEFDVELTDVGQEKVKVIKVVRDITGLGLKDSKDLVDGAPKNVKEGVSEDE
ANDIKAKLEEVGATVTVK"
CDS 507959..508843
/gene="kcsA"
/locus_tag="OCPHGMPK_00482"
/inference="ab initio prediction:Prodigal:2.6"
/inference="similar to AA sequence:UniProtKB:P0A333"
/codon_start=1
/transl_table=11
/product="pH-gated potassium channel KcsA"

/translation="MRTLFYGVLIFVYNKIMLKKNFYKWFKATLAIVSIVLIVLDFAA

IIDINGTNSKWFWLNNIILIILAIDYFYRLYLAKDRRAFFRNNIFELVAIIPVGIAFN

WMKFAQLGDIGLYFRLLRLIRLAGLVGMLKDILHTHGILYVIYFSIAFLMLGSVAISI

TEHVSLDQAFWWAITTASTVGYGDISRHTISPQSLLGKLVILAMILIGVGVMGIVTSS

LTAYLMRRNEGKVSIKDKDAELTLILDKLDRLEQQNKLLAQQTQNLQEQIQELKDSHT
PSEWNKFKDWIEKRKDKN"
CDS complement(508879..509103)
/locus_tag="OCPHGMPK_00483"
/inference="ab initio prediction:Prodigal:2.6"
/codon_start=1
/transl_table=11
/product="hypothetical protein"

/translation="MLVSIPIFILAIIVGIFCLWMGFRIKGNQAVFGRVLSTNSQTWA
RFWLTAGWVIIIIAGCYLLFMIILHFYLTM"
CDS 509209..509820
/gene="rsmC"
/locus_tag="OCPHGMPK_00484"
/EC_number="2.1.1.172"
/inference="ab initio prediction:Prodigal:2.6"
/inference="similar to AA sequence:UniProtKB:P39406"
/codon_start=1
/transl_table=11
/product="Ribosomal RNA small subunit
methyltransferase C"

/translation="MTKKNQMYFAANPDAKHDEHLVDYHIDDIDLKFTTDAGVFSKLR

IDYGSGVLIKTMKDLTFPKAGILDVGTGYGPMGLFAAKFWPDQEVDMVDVNERALDLA

RRNAKFNQINNVNIYQSNIYEQVDKKYGLVITNPPIRAGKKVVDQILSEAKEHLVENG
ILLVVIQKKQGAPSAKKLMTKVYGNCEILARDKGYYILMSKNN"
CDS 509897..510079
/locus_tag="OCPHGMPK_00485"
/inference="ab initio prediction:Prodigal:2.6"
/codon_start=1
/transl_table=11
/product="hypothetical protein"

/translation="MKLLINIILVLIVLAGFGGMIYLSVNGNRFLGAAGLVMLYFVGS
IGYFVNCYTIKKRGRI"
CDS 510271..510723
/gene="tadA_2"
/locus_tag="OCPHGMPK_00486"
/EC_number="3.5.4.33"
/inference="ab initio prediction:Prodigal:2.6"
/inference="similar to AA sequence:UniProtKB:P21335"
/codon_start=1
/transl_table=11
/product="tRNA-specific adenosine deaminase"

/translation="MQLAFAQAKKAEDQGEVPIGAIVVDKDGNVIGEGYNRRELDEDA

TQHAEMIAIREACKNLGSWRLVDCSLFITLEPCPMCSGAIINSRLAEVYYGAFDPKAG
AAGSVIDLFKVEKFNHHPQVFGGLFKDQAAQMLKDFFREIRRKQKQDK"
CDS 510900..512708
/gene="dnaX_1"
/locus_tag="OCPHGMPK_00487"
/EC_number="2.7.7.7"
/inference="ab initio prediction:Prodigal:2.6"
/inference="similar to AA sequence:UniProtKB:P06710"
/codon_start=1
/transl_table=11
/product="DNA polymerase III subunit tau"

/translation="MAYQALYRKWRPRTFDGVVGQTAITDTLKNAIKRGKISHAFLFA

GPRGTGKTSCAKIFAKALNCTNLQDGEPCNECENCLAADQGTMNDIIEIDAASNNGVD

EIRDIRDKVKYAPTQGKYKVYIIDEVHMLSMGAFNALLKTLEEPPEHVVFILATTELQ

KVPATIISRTQRYNFKRIDQHDLIARMTYILGQEKIAFEEKAIEVIAQVADGGMRDSL

SILDQILSYDQDKVKYDDALKITGYAAQEKIEQVLLDLLKGETSEAFEIVHNLLQDGA

STKNILDELISLTVKGMLVIKSNKEATFLTKDYAAELAKIPEEKFYNLVQAANTALND

LRYTNQQQIPLDVFVVQVTQGVGGNVPVEQGDSVSSTVVQKLKNEIDNLSKQVAELKN

RPVQVTNRSSSDFSGTIKTETNSRPKQVAAQTVNTDQKPKRRKASANNQAAQENNRRQ

VYHVLENATKEDLNTVKDIWPDLLSTLKVSQRAVMEVLNPVAASHEQIVLKCKYELWF

ERANQDGDLISQLEAEIAKLTKHNYSIVLVADSSWLKVRHDFVASHKEELLAKKFQKI
EKSEQETNENQVDPQAKKEVIDKAKELFGDLAQIKD"
CDS 512720..513049
/locus_tag="OCPHGMPK_00488"
/inference="ab initio prediction:Prodigal:2.6"
/inference="similar to AA sequence:UniProtKB:A3DHB8"
/codon_start=1
/transl_table=11
/product="Nucleoid-associated protein"

/translation="MSRRPNFGGMGMGNMQGLIKQAKKMQQQMEAEQANLATQELVGK

SADDMVVATFSGDRQLKDLKIDKEAIDPDDPDMLQDLVIDAVNKGIKAVDDATQASMG
KYTKGLM"
CDS 513052..513651
/gene="recR"
/locus_tag="OCPHGMPK_00489"
/inference="ab initio prediction:Prodigal:2.6"
/inference="similar to AA sequence:UniProtKB:P0CB76"
/codon_start=1
/transl_table=11
/product="Recombination protein RecR"

/translation="MQYPLPIARLIDNYMKLPGIGEKTATRLAFYTMDMPEDDVEDFS

KSLMQVKENLHSCSICGNITESDPCEICRDANRDRSTIMVVEQPKDVMAFEEMGEYNG

LYHVLHGVLSPMDGVGPEEINIKSLITRLQKQDEVKEVILALNSSPEGEATAMYLAKL
IKPAGLKVTRLAAGLAVGSDIEYANSITLKRAVQGRTDL"
CDS 513648..513884
/locus_tag="OCPHGMPK_00490"
/inference="ab initio prediction:Prodigal:2.6"
/codon_start=1
/transl_table=11
/product="hypothetical protein"

/translation="MIGKRHKVKKAGDERLLKVVAQLQRQLAEQKVFDQTTIDYSFDN
RVLNKILHAKFIFLYEEARRRNTKSSSSSSVITR"
CDS 513992..514633
/gene="tmk"
/locus_tag="OCPHGMPK_00491"
/EC_number="2.7.4.9"
/inference="ab initio prediction:Prodigal:2.6"
/inference="similar to AA sequence:UniProtKB:Q97R91"
/codon_start=1
/transl_table=11
/product="Thymidylate kinase"

/translation="MKGYLITFEGPDGAGKTTVINELIKQLPKEIQKKTIITREPGGS

KISENIRTIILDPANKEMDDRTEALLYAAQRSQHVSEVIRPALAAGKIVLSDRFIDSS

LAYQGVGRNLGIEAVKQINDFGTGGLEPDLTIFLDLDPATGLARIEKERAGQEDRLEQ

```
EKLAFHKKVYAGYIDLLKRYPDRIKKVDANLPIEEVAANSVKIIRKQLPDIFM"
     CDS             514647..514973
                     /locus_tag="OCPHGMPK_00492"
                     /inference="ab initio prediction:Prodigal:2.6"
                     /codon_start=1
                     /transl_table=11
                     /product="hypothetical protein"

/translation="MKLVLAIVQDKDADALAREFIQNNVRATKLATSGGFLKAGNTTF

IVGIEDDRVNEVLEIIKKSSHTREQYVSNMNMDAAGTSMLGKPVQVTVGGATVFVLPV
                     EEFKHF"
     CDS             514973..515830
                     /gene="dnaX_2"
                     /locus_tag="OCPHGMPK_00493"
                     /EC_number="2.7.7.7"
                     /inference="ab initio prediction:Prodigal:2.6"
                     /inference="similar to AA sequence:UniProtKB:P9WNT9"
                     /codon_start=1
                     /transl_table=11
                     /product="DNA polymerase III subunit gamma/tau"

/translation="MIDLKNIGQKQTDLLRDAYLNKKVAHSYLFVDPMQKKGLNTAYW

LACLFNCTGENKPDGTCNNCQRILDGNHPDVFLVKLEGKQTLSIDQIRPLKEELAKSP

VEGKRRFFIIENAEKLTLAASNALLNLLEEPVAPVVTILITNNENQILPTVKSRTQIL

NFSDEKIDSKRAQLLEYGLTDEEIDDLGDTAKLEEESKYLFQELLEQNDLALVRVSQI

SGLATKPASQKFVFYQLKTLAMKSLAAGEKLRKSAFLLELLMTADKMRASNVSFHNTL
                     DYLVLSFER"
     CDS             515841..516179
                     /gene="yabA"
                     /locus_tag="OCPHGMPK_00494"
                     /inference="ab initio prediction:Prodigal:2.6"
                     /inference="similar to AA sequence:UniProtKB:P37542"
                     /codon_start=1
                     /transl_table=11
                     /product="Initiation-control protein YabA"

/translation="MDPFSQLSQLQQNLQAMTKTVAGLENDMLEVLKENTELKVENQL

LREKISKLDANKEPAESKSQAGLKSLRNIYDSGYHICNMYYGSHRESGEDCMFCLDIL
                     DNFVNHGQKK"
     CDS             516190..517047
                     /gene="rsmI"
                     /locus_tag="OCPHGMPK_00495"
                     /EC_number="2.1.1.198"
                     /inference="ab initio prediction:Prodigal:2.6"
                     /inference="similar to AA sequence:UniProtKB:P67087"
                     /codon_start=1
                     /transl_table=11
```

/product="Ribosomal RNA small subunit methyltransferase I"

/translation="MQAQSSFNEKDKGRLYLVPTPIGNLEDITLRARRILTEADYIAA

EDTRTSGILLEKIGVHNRMISFHKYNSKERARELIKLLKEGNTIAEISDAGMPVISDP

GYILVQECIKNDIPVVSLPGPSAFATALIASGFDAQPFTYYGFLPRKSSEQKVYFEMM

NQARATSIFYEAPHRLKKTLKTLAEVIKPDRKIVLARELTKIHEEYLRGTISEINEYF

TKNDPRGEFVVLVSPNDEKEKQLSWDELIKQVADRVEAGESKKDAIKLVAKANKVSKN
ELYDKYHQN"

```
     CDS             517065..517799
                     /locus_tag="OCPHGMPK_00496"
                     /inference="ab initio prediction:Prodigal:2.6"
                     /codon_start=1
                     /transl_table=11
                     /product="hypothetical protein"
```

/translation="MEKIFKEEHQVTYGDCDETGKIQLPKLIEHFMKVSNDQLTQGNA

GIHDLLKQNLGWVVVEYHLDLERLPEAGEKITVTTNGSGYNRFFEYRDFGIIDSTNKK

IVDVKSQWVILDLKNRKITEADSQMMEKFGNPYLKHMPRFKRLRPLKDYESSKTYAVR

YYDLDTNHHLTNSVYFDWMIDTLPREFLNSHTAKSVDISFKKEVQYGDQAVSELTLDQ
DMLTSYHLISNQGEISALAEISWREN"

```
     CDS             517850..518365
                     /gene="folT"
                     /locus_tag="OCPHGMPK_00497"
                     /inference="ab initio prediction:Prodigal:2.6"
                     /inference="similar to AA sequence:UniProtKB:A0Q1J7"
                     /codon_start=1
                     /transl_table=11
                     /product="Folate transporter FolT"
```

/translation="MKTGIKKIDLQGLVTLALFVAMDIVLEKISFGPSTVKVGLGFIG

SALLGYYFGPLWGGIGALISDLLRSAIFGVEGGFFPGFTLSAIVGVVIYALFLYQKPI

KWWRVIAATLLVTLVVNVLMNTYWLHLLYGLDLRAAFLQRILKELITPWIQIVVLYVI
LNAFQRVKIRR"

```
     CDS             518394..519119
                     /gene="tsaB"
                     /locus_tag="OCPHGMPK_00498"
                     /inference="ab initio prediction:Prodigal:2.6"
                     /inference="similar to AA sequence:UniProtKB:O05516"
                     /codon_start=1
                     /transl_table=11
                     /product="tRNA threonylcarbamoyladenosine
biosynthesis
                     protein TsaB"
```

/translation="MKILSITTATNHLSVALNDGDKIVEKNEEDHRNHSEHLDPLITE

LLKENNLTLKDIDRFAVAEGPGSYTGLRIGITTAKMFASILNKDLVGVSTLAALANGV

NDGVIVSELDARNKNFFAGVYRKVNGKLENLVLDGHYHLDDLINKVKELGLPEEVVFV

GSPIDKYQDEIVELLGSDNFKQAAGDNQIHAGEIGKLALNKEAIDPDKMVPKYLRRTQ
AEIDWHNKTGKAFGPDSDYVEEV"
CDS 519103..519660
/gene="rimI"
/locus_tag="OCPHGMPK_00499"
/EC_number="2.3.1.255"
/inference="ab initio prediction:Prodigal:2.6"
/inference="similar to AA sequence:UniProtKB:I6YG32"
/codon_start=1
/transl_table=11
/product="N-alpha-acetyltransferase RimI"

/translation="MLKKFSLFFHPAEIDLSFSPFALRLDNQTYQIMKASDENIADLL

GLEQEVYFGRTPWSRFSFKSELKKHANSLYLVVYQGSTLVGFVGMRMQAQEGHITNIA

VKPAFQKRGIGGFLLRTMIEIAQRNHAVQMTLEVRSDNYNAQSLYRKIGFRDNFIRKN
YYSAEHADAVSMIKKIADREQEEVN"
CDS 519657..520703
/gene="tsaD"
/locus_tag="OCPHGMPK_00500"
/EC_number="2.3.1.234"
/inference="ab initio prediction:Prodigal:2.6"
/inference="similar to AA sequence:UniProtKB:O05518"
/codon_start=1
/transl_table=11
/product="tRNA N6-adenosine
threonylcarbamoyltransferase"

/translation="MTKKDIRILAFESSCDETSTAVIKNGREIESLIVATQIKSHQRF

GGVVPEVASRHHIEVITQITKEALAEANATWDDIDAIAVTYGPGLVGALLIGVSAAKA

ASMATGIPLIGVDHIMGHIMAAQLKDEIEYPALALQVSGGHTEIVLMKDPIHFEIVGD

TRDDAAGEAYDKIGRVLGVNYPAGKTIDEWAHKGKDTFHFPRAMMEDDDYDFSFSGLK

SAFINTCHHADQIHEKLDKYDLAASFQASVVDVLSHKTIRAIKEYKPKTFILGGGVAA

NHGLRDRLAEEIEKLPADIKPKVILPDLKLCGDNAAMIGAAAYNLYKAGKFSDENLNA
DPSLELPYAESMLK"
CDS 520938..521561
/locus_tag="OCPHGMPK_00501"
/inference="ab initio prediction:Prodigal:2.6"
/codon_start=1
/transl_table=11
/product="hypothetical protein"

/translation="MKDEVQSPQEETPNTYWRWLVESWRYPLGHNPAEKWYGVVTLLG

EDIVLFIGLAVAGNYIANLFDMPLVHHGSGATFWAIVILFVVQIITILCSMVGHLFVF

GNTGGFWQYVNKVAQVTNWNFWIAICAFFFLILSLSSGLLGFGEGLVWIFYALFALGS
FLLVVCPDPGTEKMIHDPFWGAVIYLVMVAVVYAIIWGIALSILINL"
CDS complement(521652..523025)
/gene="clcA_2"
/locus_tag="OCPHGMPK_00502"
/inference="ab initio prediction:Prodigal:2.6"
/inference="similar to AA sequence:UniProtKB:Q8ZRP8"
/codon_start=1
/transl_table=11
/product="H(+)/Cl(-) exchange transporter ClcA"

/translation="MIKTAKQVLAKPFTSNFFFIFLQGIIIGIITGLIVGTFRWIIDH

TMKFLFFIYPLMRQKPIYLVPYIIATLLIVLILGKIIKPVLSNITGSGVPQVEAVMLN

ENKMNWWSILWRKFVGGLLAICPGLFLGREGPCIQMGAMVGQAFGENLFHADPDDLKR

LQGCGIAAGLSAAFSAPLAGVFFLVEEITFNFTPKEVLTALAAAMSSDLMTLCFFGTQ

PCLYLPLDKSLPLTSYWFVAIIGVVLGLLAYLYQYCLLSLKPVYAKITKIPAIYHSVI

PLLLVIPIGLWNANLLGGSHDFISSLFDPTFMKSIQTGALSLILLPLVWFAVRFIFSM

ISYGASVPGGIFMPILVLGALLGVVFAVLMIHFNIAPKRCYGIIIVTSMCAYFGTIEK

APFTALTLLTEMVGSVEQIFPMLITTFIAYFVLDLLGGKPIYAALRIQMNYHKLSNLP
KVKHAEE"
CDS complement(523135..523422)
/locus_tag="OCPHGMPK_00503"
/inference="ab initio prediction:Prodigal:2.6"
/codon_start=1
/transl_table=11
/product="hypothetical protein"

/translation="MKKNKYVTESFINAITKEVSILIAANDHMSIEKAENLFKESKVY
NFLCFPPDYFYEEDPQYFYEMYENLEHKGLMVDNNQLYLMQNPQLYKLPTD"
CDS complement(523409..523939)
/locus_tag="OCPHGMPK_00504"
/inference="ab initio prediction:Prodigal:2.6"
/codon_start=1
/transl_table=11
/product="hypothetical protein"

/translation="MKISIPNLLYHGSDVIVEKPDIHAGKPRHDFGKGFYLTTDINQA

AKWSKHKIKINSSKLKQNPYNILISKYVFDTNQFYSLRVKIFDHVSEDWPNYIIKNRK

NIDFALNTDYDLVIGPVVDGANSWEALALYSNGTISFEKAISRLKPENLTDQWAFKTQ
NSINCLKYGGVLYEEK"

```
     CDS             complement(524094..525086)
                     /gene="guaC"
                     /locus_tag="OCPHGMPK_00505"
                     /EC_number="1.7.1.7"
                     /inference="ab initio prediction:Prodigal:2.6"
                     /inference="similar to AA sequence:UniProtKB:P60563"
                     /codon_start=1
                     /transl_table=11
                     /product="GMP reductase"

/translation="MSNYFSMEAFDYDDIQLVPNKCIIKSRKEADTSVKFGSRTFKIP

VVPANMESVIDDDLAIWLAENGYYYVMHRFYPEKRADFIKMMHDKGLFASISVGIKDS

EYDFIDYLAKEKIIPEYITIDVAHGHSDYVIKMIKYIKDKLPDTFLTAGNIATPEAVR

ELENAGADATKVGVGPGRACITKLKTGFGTGGWQLAALRMCSKAARKPLIADGGIRHN

GDIAKSVRFGASMVMIGSLFAGHEESPGNLITIDGKRYKQYWGSASEVQKGAYRNVEG

KQMLVPYRGSIKDTLREMQEDLQSSISYAGGKDLSAIRVVDYVIVKSSIFDGDK"
     CDS             525277..526566
                     /gene="purA"
                     /locus_tag="OCPHGMPK_00506"
                     /EC_number="6.3.4.4"
                     /inference="ab initio prediction:Prodigal:2.6"
                     /inference="similar to AA sequence:UniProtKB:P65887"
                     /codon_start=1
                     /transl_table=11
                     /product="Adenylosuccinate synthetase"

/translation="MTAIAVVGSQWGDEGKGKITDFLSKDAAMAVRSNGGNNAGHTIE

IGDKTFKMRLIPSGIFAAKKGAVIGNGVVINPEVMFGELDNLEKEGIDISGLKISNRA

HVIMPYHILQDTYQEEAKGDKKIGTTKNGIGPCYMDKASRIGIRVCDLLERDTFEEKL

RTNLAEKNALFTKVYNKPALKFEDIFEKYLEYGQKMKKYVTDTSVVVNDALDKNEKVL

FEGAQGVMLDIDEGTYPYVTSSNTISGGIASGIGMGANRLNTVIGVCKAYTTRVGEGP

FPTELLDEVGDRIRETAHEYGTVTGRPRRVGWFDSVALRHAKRVAGINALSLNLLDVF

SGFDKIKIATAYELDGKKIDYYPASLKELYRCKPVYEELPAWDEDITNVKTWEDLPEN
                     AKKFLNRVSELVGVPLVTVSVGPDREQTIVLKNPWEM"
     CDS             526570..527865
                     /gene="purB"
                     /locus_tag="OCPHGMPK_00507"
                     /EC_number="4.3.2.2"
                     /inference="ab initio prediction:Prodigal:2.6"
                     /inference="similar to AA sequence:UniProtKB:P12047"
                     /codon_start=1
                     /transl_table=11
                     /product="Adenylosuccinate lyase"
```

/translation="MLERYTRPEMGKIWSLKNKYAAWLKVEIAATNAWAELGEVPAED

AKKIADNASFTVERVSELEAITHHDVVAFTRTVSESLGEEKKWVHFGLTSTDVVDTAQ

GYILKQADEIILKDLETLKETIADSARKYKYTVEMGRTHGVQAEPTTFGLKLARWYAE

INRDIERFKAAAKDVESGKISGAVGTFANVPPAVETSVLKQLGLTQQPVTSQVLPRDL

HAYYIATIALIATSIENWATEIRGLQRSEIHEVEEHFRAGQKGSSAMPHKHNPIGSEN

LCGMARVMRGFMVPAYEDVALWHERDISHSSAERVILPDATIGIDYMLNRFNRILTNL

DVFPETMLKNMDRTYGLIYSQRVLLKLIDEAGLSREKAYDMVQKLANKSWQEQTSFRK
LIEDSEVMNYLSEADLDDAFDYHYHLKHVDEIFEKCGLD"
CDS 527943..528392
/gene="ndx1"
/locus_tag="OCPHGMPK_00508"
/EC_number="3.6.1.61"
/inference="ab initio prediction:Prodigal:2.6"
/inference="similar to AA sequence:UniProtKB:Q75UV1"
/codon_start=1
/transl_table=11
/product="Diadenosine hexaphosphate hydrolase"

/translation="MKHEHSAGAIIWRKKNNEIQYLLIQSQPYKQFKSAWAFSKGHLE

AGETAQEAAKREIFEEVGLKAEFNFDFSESYSYQVTSEIEKTVTLFLAKYNPDQKIKR
QESEIRQTAWLNYEDAQKRIREQNFKEFSFEDLSSILEKANDYLNGH"
CDS 528508..528807
/locus_tag="OCPHGMPK_00509"
/inference="ab initio prediction:Prodigal:2.6"
/codon_start=1
/transl_table=11
/product="hypothetical protein"

/translation="MAHTYSYRADSKDHDEVKRILDNLGLDMSTSIKMYFKQIIRHNG

IPFSVTNSDTLTEDTKKALLLAEAKDMGLIEDDTPAFKDTNKLISYMKKRAKELE"
CDS 528807..529115
/locus_tag="OCPHGMPK_00510"
/inference="ab initio prediction:Prodigal:2.6"
/codon_start=1
/transl_table=11
/product="hypothetical protein"

/translation="MYEIKAEDTFIQDVNRWSKKVPELWDEIQAITSYMQETGEIPEE

YDPHLLTNEELNYVGYFEFHLFEGKLDLLVIHTKNRNKKVFRLVRLGSHNELFHSDLR
"
CDS 529215..530402
/gene="uctC"
/locus_tag="OCPHGMPK_00511"
/EC_number="2.8.3.19"

```
                     /inference="ab initio prediction:Prodigal:2.6"
                     /inference="similar to AA sequence:UniProtKB:A9X6P9"
                     /codon_start=1
                     /transl_table=11
                     /product="Acetyl-CoA:oxalate CoA-transferase"

/translation="MSEPNYAKLEANNLHLDKEKPYPLAGILVVDFTHVLSGPTCTRM

LADAGARVIHIERKTGDDTRHMGPYIADGSSEYFRICNAGKESIALDLKDPKDHALAE

KMIAKADVVVENFRPGVMARLGFDPEEMVKKYPKLIFASVSGFGQYGPWSKQAAYDTI

IQAVSGLMDATGEPNGKPTRVGTSVSDVVAGIMGYSAITTALVARERTGKGTTIDVSM

LDSTFSLMVQDLMLALGPHEVPHRIGNRHPDMYPFDTFDCQDQPIAICCGNDHLWGLL

SHALGHEEWMNQADFKTNDLREKNWQKVKNAMQDVLKTKPAAEWDKILHEAGVPAGLV

LNVDKTRRLDQIIERGMVKTLPDGNEVLGSPLKYGTWNSYGLQKDAPKLDEQGEQIRK
                     EFE"
     CDS             complement(530473..531300)
                     /gene="melR"
                     /locus_tag="OCPHGMPK_00512"
                     /inference="ab initio prediction:Prodigal:2.6"
                     /inference="similar to AA sequence:UniProtKB:P0ACH8"
                     /codon_start=1
                     /transl_table=11
                     /product="Melibiose operon regulatory protein"

/translation="MKITHEQILNKSIAPFKAFSFRSRDSSRIIPSHWHESTEILFVL

SGKMNTTINGKLQEINQNQFIIINPCVIHSTNALKNSHILCIQYPFTFLNLVTNGKFQ

QFFQFNSNLNSISSLTLSANIINLVKALETQDKDISINLSIYILGLQILQELIKKYLV

VTHYSTNKNIPFLNEFIDFVSKHYTENISLHMVADHFGYSVAYCSRLIRKNLDHSFSD

YLTSLRLEQSLRLSLQSNLNIEKIAEKSGFKCYRNLYNAFKKVYNCTPEEAFNQLKE"
     CDS             531456..533684
                     /locus_tag="OCPHGMPK_00513"
                     /EC_number="3.2.1.21"
                     /inference="ab initio prediction:Prodigal:2.6"
                     /inference="similar to AA sequence:UniProtKB:A7LXU3"
                     /codon_start=1
                     /transl_table=11
                     /product="Beta-glucosidase BoGH3B"

/translation="MKQEKIFTLLNEMSLDEKIGQLIQLSGEFFSASDISIGPIEKLG

ISKKMVNLCGSALNVVGAENVHKVQDQQMQKQPHHIPMLFMSDVIYGFKTIFPIPLGL

GSSWNPKLVKRAYSVAAEEASASGNQVAFAPMVDVVHDARWGRVLESPGEDPYLNSVY

SKNMVEGLQESLANKTGQVSCIKHYAAYGAVEAGREYNQVDMSLSNLYQNFLPPYKAG
```

VKAGAGMVMTALTTLNGVPSTADRWLLSDVLREKWKFNGIIISDYASIYELIKHGFAR

DSTDAALKAIDAGVDIDMKSPCYANGLHELVTNGALDERKINNAVLKVLNLKNQLGLF

ENPYFGASVEKEKNSILTPQKRKLARKLAQESLVLIQNKNNILPLKKDTKVALIGPYA

NSQKLIGMWAIHGNSKDTVTIADGLRQYTNKLTVNHGTDLCRDKALLKKLGFLTEKDI

NKIVSSPEKERLNIQNAINAAKAADTIVLTLGEETYEAGEAGAKTNLNIAPNQLKLLD

KLSQLNKPIILVLISGRPLVLTKVKDKADAILESWFPGTEGGDAIADILFGKVNPSGR

LTMSFPYASAQEPLYYNHLCTGRPENNSQHVGRFVSKYIDAPAKALYPFGYGLSYGKI

KYSNLTISSKRLFLDKPIKISMDIENLSDYDRKETIQLYIHQETASIVQPVKRLIDFT

KIAISGHSSQHVEFYINASQLSFYDNIGRSHKESSKIQVYVGTNGVQLLKGKFDFINK
RS"

CDS 533781..535745
/gene="bglF_1"
/locus_tag="OCPHGMPK_00514"
/inference="ab initio prediction:Prodigal:2.6"
/inference="similar to AA sequence:UniProtKB:P08722"
/codon_start=1
/transl_table=11
/product="PTS system beta-glucoside-specific EIIBCA component"

/translation="MSSKENKKIAEEVIAAIGGKENIVSLAHCATRLRIVVKDRKKVN

EKALDKIDKAKGYFFTAGQYQIIFGTGLVNDVYEAVKEEGVSTTSKDDLNDVAAKQSG

WFQRSIRMFADVFVPIIPALVATGLFMGLQGLLTQPALLKMMGMSAKSIPVQFTTYFN

ILTGTAFSFLPVLICWSAFRVFGGSPILGILLGLMLVNPALPSAWDVAQGKVAPLMFF

GFIPVAGYQASVLPAFILGFIGAKTEKWIHKHVISSLDLIVTPFATLLIGSILGLLCV

GPIFRIVEQGILHAAEWIVQLPFGLGGLIWGAVCQMIVVTGLHHALNLVEIQLLANTH

WNPVNPIGSAAIAAQAGAALAVALRIHSNKMKQIAYPSTISALLGITEPAIYGVNIKY

IKPFLFGCIGGGIGGFLSSILNLKATGMSITVIPGILLFLNQQLPLYILVCVVGFGVA

FGLTYVYYGKHAFDSNKNSKKEDIEKKQNTFTQEPSHVSDEVIDAPVSGKIEALNLVS

DKAFASGMMGKGIAIEPSSNNIVSPVNGTITVACPTGHAYGIKSDDGAEILIHLGIDT

VNLKGKGFNTVVKQGQKINKGELLGTYDYKEIGKEGYDNIVIVLITNSKDYAEVDPIA
SGNVKAQQKLIALTEPTMTEQVKGTVNELN"

CDS 535842..537371
/gene="scrB_1"
/locus_tag="OCPHGMPK_00515"

/EC_number="3.2.1.26"
/inference="ab initio prediction:Prodigal:2.6"
/inference="similar to AA sequence:UniProtKB:P13394"
/codon_start=1
/transl_table=11
/product="Sucrose-6-phosphate hydrolase"

/translation="MTNRREQFEHLVSNLKDIPKENLIKEKNSVKKSPYRQRIHIESL

FGMLGDPNGFSYFNGYYHLFHQWFPMKYSTNPNYFQQGWFHWKSRNLVDWIPVGEAMN

NDTVFDKYGVYSGSAIPINNKLFLMYNGNSWTNTDTDDWHRVPCQLGAYMNENDNVAK

LANPLIKGPISGYTSHFRDPKIFKKNGKYYSIIGAQTKAKTGTVLIYESQNLRIWNKV

GEVKTNFEKTGYMWECPDYFELDNKGILLFCPQGLKSKGSQFLNTFQACYAIGNKLDF

RNLYFEGKKFEEIDSGFDFYAPQTMIEPDGRRILSAWMSIMNSNSPLIKYHYDGCLIF

PRELSVQNDKLIQKPVSEIKKLYTTDYKGKRAISKNQEIVAGLVNCRDIKFDISTKDN

SAISIIDLFANRENTSHLRLVLNRMKNRFIVQRGKAGIEFEDEFGNERSCELKVANSV

SIRIIQDISSAEIFINNGENVFTMRVFVPKDQYHIFVKNVNGEVNVKYQIHQLRKMSS
E"
CDS complement(537438..539369)
/gene="ettA_1"
/locus_tag="OCPHGMPK_00516"
/inference="ab initio prediction:Prodigal:2.6"
/inference="similar to AA sequence:UniProtKB:P0A9W3"
/codon_start=1
/transl_table=11
/product="Energy-dependent translational throttle
protein
EttA"

/translation="MIIAQAQDLEQRFGGNIIFSNISFSVPDNARIGLVGPNGAGKTT

LLKIMTGQQEPTSGQFTINKGLKVGYIAQENGLDEDKTIWDEMLTVFNDLIEKNKKIT

KMQEQIADHPEDEDLLKRYDQLAYDFEQEGGFTYQAEIKSILNGFNFKENTWNKVIGT

LSGGEKTRLAFVKLLLQKPPVLLLDEPTNYLDLDTLDWLEAFLKNYQGAIITVSHDQY

FLDHLANQIFELNFGKLTTFKGNYSQYVKERELMDSQQEAAYEKQQEKIKKEEEFIQK

NLVRASTTKRAQSRRKVLDKMERIKPPKHKQKVRINFTSERPSGKEVLIAKDLTIGYP

DKVMVSDIDFQVNKNDRVAIIGPNGIGKSTLLKTIMKKLEPKGGSIKYGASLDIGYYD

QELQSLDPSKTVLDTIWDRHKTMPEKDVRSILASFLFTAEDIDKTVGQLSGGQKARLT

LTVLSLEKDNFLLMDEPTNHLDIEAKEVLEEALDNYDGTLLFVSHDRYFINELANKII

SVRDGHAKIYNGNYSYYLDEKAKQAAAAQEAEAQKAETETTPAASQNKGKLSYQEQKA

RDSQKRKLERAVSDAEAKIEKLEAEEKEIQTEMANPEIAASFEKLGPLQEKLSAVQKQ
LDQANNDWENALDALDEFE"
CDS 539535..540179
/gene="rex"
/locus_tag="OCPHGMPK_00517"
/inference="ab initio prediction:Prodigal:2.6"
/inference="similar to AA sequence:UniProtKB:O05521"
/codon_start=1
/transl_table=11
/product="Redox-sensing transcriptional repressor
Rex"

/translation="MDEIKIPKATARRLPLYYRYLIFLNDEGKEKVSSTELAEAVQVD

SASIRRDFSYFGALGKRGYGYDVKNLLNFFKKILNQDTLTNVALVGVGNMGHALLNYN

FKRTNNIRISAAFDINPEITGTIMSGVPVYDMSEMKKQLREQQITIAILCVPQTAAQK

TANEMFDAGIKGIMNFTPLRLSAPSSVRVQNVDLATELQTLIYFLDSDKDKNKK"
CDS 540317..540883
/gene="yvdD"
/locus_tag="OCPHGMPK_00518"
/inference="ab initio prediction:Prodigal:2.6"
/inference="similar to AA sequence:UniProtKB:O06986"
/codon_start=1
/transl_table=11
/product="LOG family protein YvdD"

/translation="MIKKMAVYCGARSGNRLEYAKAAYEFGKKMADHQIELVYGGGKY

GLMRAVADGVLENGGIVHGIITEELKDRGAAYDKVQDFRVVPSMDKRKDTMMNLADGM

VALPGSIGTLEEISQAISWTAIGDNAKPVAFYNYAGFYDYLDKLLKRMNQDDFLENIY
LDTVYFETDFDKILQFMEDYQAPAYREY"
CDS 541054..541512
/locus_tag="OCPHGMPK_00519"
/inference="ab initio prediction:Prodigal:2.6"
/inference="similar to AA sequence:UniProtKB:Q5HDJ2"
/codon_start=1
/transl_table=11
/product="putative HTH-type transcriptional
regulator"

/translation="MKVKLELDPDQQETEITIHAKSLTPEVERIYHQLQTDSEHPDQI

EGMMDNTSYYLSINNILFFETDAKQVMAHTTRNAYFVKYKLYELENLLSGQFMRVSKS
TILNLDQIYAVTKSISNCQIKFHDSYKTVYVSRRYYRDLNERLKERRALS"
CDS 541509..542267
/locus_tag="OCPHGMPK_00520"
/inference="ab initio prediction:Prodigal:2.6"
/codon_start=1

```
                     /transl_table=11
                     /product="hypothetical protein"

/translation="MRKASFGRILWGLGLLAAAAFLVLDQLHLMPLELGFWAIFWTVV

FGATLITSLINKSLGGSIFSIAFLLIIYAKPLHIERIVPWTVLLAAFLIWGGLRLIFK

KSWKPTVIINGEKVNANWSDLKAPHKFKAEHVFSDTSSSDNDDNIVISEKLSSTSRYV

HSQHLETVTVNVSMGDVNVYLDSAKPAGDEVLANINMSMGDINIYIPTSWRVDDQLEH
                     KFGDFTIDGDQPAEGPTLVLQGRANMGDVTIKRV"
     CDS             542793..543077
                     /gene="groS"
                     /locus_tag="OCPHGMPK_00521"
                     /inference="ab initio prediction:Prodigal:2.6"
                     /inference="similar to AA sequence:UniProtKB:O50304"
                     /codon_start=1
                     /transl_table=11
                     /product="10 kDa chaperonin"

/translation="MLQPIGDRVIVKVKDEEEEKVGGIVLASNAKEKPQMGEIIAVGN
                     GKRNANGDLIPMSVAKGETVFFDKYSGTNLKYEGEKYLVLRESDLLAVVK"
     CDS             543109..544740
                     /gene="groL"
                     /locus_tag="OCPHGMPK_00522"
                     /inference="ab initio prediction:Prodigal:2.6"
                     /inference="similar to AA sequence:UniProtKB:O50305"
                     /codon_start=1
                     /transl_table=11
                     /product="60 kDa chaperonin"

/translation="MAKDIKFSENARHSLLKGVDKLADTVKTTLGPKGRNVVLEKSYG

APDITNDGVTIAKSIDLEDHFENMGAKLVSEAAQKTNDIAGDGTTTATVLTQAIVREG

MKNVTAGANPVGIRRGIETATKAAVDELHKISHKVSTKDEIAQVASVSSASTEVGNLI

ADAMEKVGHDGVITIEESKGIDTELSVVEGMQFDRGYLSQYMVTDNDKMEADLDNPYI

LITDKKISNIQDILPLLQEIVQQGKSLLIIADDVDGEALPTLVLNKIRGTFNVVAVKA

PGFGDRRKAMLEDIAILTGGTVISSDLGLELKDTKIDQLGKAGKVTVTKDSTTIVEGA

GSKDAISERVDQIKKQIADTTSDFDREKLQERLAKLAGGVAVIKVGAATETELKERKY

RIEDALNATRAAVEEGYVAGGGTALVDVMKSIQGNVKGDSQDAQTGVNIVMKALGAPV

RQIAENAGKDGAVILDHLEHEDPEIGYNAATDKWENMVKAGIIDPTKVTRSALQNAAS
                     IAALLLTTEAVVADAPEDDKNQAPAAPNPGMGMGM"
     CDS             545032..546402
                     /gene="lip"
                     /locus_tag="OCPHGMPK_00523"
                     /EC_number="3.1.1.3"
                     /inference="ab initio prediction:Prodigal:2.6"
```

/inference="similar to AA sequence:UniProtKB:P0C0R3"
/codon_start=1
/transl_table=11
/product="Lipase"

/translation="MSSKMFGDKHNHYSIRKLSVGAASVLIGATIYLGGVNAQAVRAD

SVNEDATEQVEKKDEANVKAAEVKTTEQKQENNKTSVSATNENAKQNVAETTSDSKKV

ASNGDVNVIKNDVTTDEEAATKSSVQADKDVNANKLDTNTVSVNKLQRSSNVAGLTES

KAADENIEQKAEESPLGTRFMNHTNDFPGDATAIRTVTLNKPQSAGGSETITQTLGAF

GFSEVINESNTSNTTKVKLDFPLTPTLNGKPVLSSTRHFSTNLVFSNNKWTFASAEDE

ENYNKFLAFQPSDYKATLPAIEDSQIAVDGYTWKVTSATPDGAGLGAETLSPYAGISQ

NIVIDYTKEEHTVTYKFEDPSGNQVGSSVPVTGAAGSNQPVSLTLPAGYQLVSGSLPT

SVTIPTNSDKTISLPVKYQLTITLLGDNTINYAGPNWQSIAKNKDNVSASGYRVKFND
DEAGVN"

CDS 546675..551237
/gene="bca_1"
/locus_tag="OCPHGMPK_00524"
/inference="ab initio prediction:Prodigal:2.6"
/inference="similar to AA sequence:UniProtKB:Q02192"
/codon_start=1
/transl_table=11
/product="C protein alpha-antigen"

/translation="MGRISIYKHDGTITTIQLAPNDVEFWLNGQKVAKDQAKNVGEYD

LRLTDDFINKVKSADGNNGNNYEWTYGTHTPSGDTTYTAIYKIYQATGKAKLSGNNSK

LYDGNAVTTADVNKDGKITIDLTLPVYNPDDESQLLRTVPLGKYTLQDGDYTWNTADG

SAPTKGGSYTISLNKDKILSHLQDRLKTLAGVGTDPDSEDPDNPTPMDNVTVSAEDLA

GTANYDINTSVSYQFIDNDNNDSKVGAPVVVTGLNGESKDVSLVVPEGYELVDVLPTK

ATFGDINRTIRINLKHKKTSVDGRDHKDDNDIYRTVTRTITVNIEGQPTQVISQKVSL

YRIKTVDEALKAAGKPESEYTTYSAWTSNMSDGSTSFATVSVPANGNGYVRSITGGTI

EKDKVTEQSALNNGEPAKDITVTVNYAKAEQSTTVTYVDKDGNEIKMPDGQPVAGSHY

EISGKTGDTVNTDVKDKVPAGWEIIPGTKYPTTVTFGSTPTSPIKVKVEHKITKTDGR

NHKDKQDLYREVTRTILMNVPNATSQGKETETLSFYRIMTHDEVTGDNTYSEWKSDVA

GDKTAFDEFDVSKTNDGKEIAAGYTPTSRDVTLEAKNGDKFVPSQSALKNGVPADSFI

VEVAYTPNAQKTTVTYVDESGNEITNPDGSVIPGSHYDLTGVTNKKVDTNIQKNVPTN

WHITDPEVPATITFGADGHTPITVHVAHNTKPVDKNDVPDGYTKDNFVKEIKRSITAK

EPTGDVDLSQTTELTRTGTYDEVTKKVISYGNWTTGSFDEVTAPEVAGYTPSQAKVDG

ADVTVDSKFDPTVITYAPNAQKTTVTYVDKNGKEITNPDGGAITGSHYDVDGVTGQKD

IPTNIEKNVPTNWHITDKTIPGTISFGPNGHDPIKVHVAHNTKPADKDNVPDGYKRED

FDKTVTRTITAKEPSGDVDLSQHVELTRTGTYDEVTKEVTYNPWTTSSFVEVTAPEVA

GYTPSQAKVDGVKVTADSNFDPVVITYVEDPIGQDITVKKGDTPSPEDGVKNHGDLDK

VTDPKHPDAKTTYTWKKTPDTSVPGNVPATVIVHYPDGSNKPVDITVHVIDETPIPTK

DPDPIGQDITVKKGDTPSPEDGVKNHGDLDKVADPKHPDARTTYTWKKTPDTSVAGDV

PATVIVHYPDGSNKPVDITIHVVDDTPVVPTKNSDPIGQDITVKKGDTPDPEDGVKNH

GDLDKITDPKHPGTKTTYTWKKTPDTSVAGDVPATVIVHYPDGSNKPVDITVHVVDDT

PVVPTKNPDPIGQDITVKKGDTPDPEDGVKNHGDLDKITDPKHPGTKTTYTWKKTPDT

SVAGDVPATVVVHYPDGSEKTVDITVHVVDDTPVVPTKNPDPTGQDIHTPQGKVPTPE

SAITNKDKMPDGTKYTWKEIPDVNTLGKHPSVVVVTYPDGTAVEVKVNVFVDGTPEAK

KETKAPVVKKHVVEPTKVETRQKLVNNYVAPRAVEVQRAQAKGKRQLPQTGAKENIAS
EVLGMLSVGLGALTAGFASKRRKKNR"

CDS 551492..553255
/gene="yheI_1"
/locus_tag="OCPHGMPK_00525"
/EC_number="3.6.3.-"
/inference="ab initio prediction:Prodigal:2.6"
/inference="similar to AA sequence:UniProtKB:O07550"
/codon_start=1
/transl_table=11
/product="putative multidrug resistance ABC
transporter
ATP-binding/permease protein YheI"

/translation="MKHLHKLNQYFKELTRTYHLFFKSAPLVAACVLLLAPLQAVIPL

LAVAAGQKVIDQVTNHSPFMEMIIVWIVATAFQQFLPSLSTMVQGVLTDKLTGFINIS

LMKKSADLQSISIFDDSKYFDDLQMLRDDASWRPVNLIVFGVSVLQSFLTLAFMLIYL

ARYNWWLALLLLVVMVPQSISYYRIQQQSFETMVERSKNARYLHYYSGLLLDRRDAKE

VRLFNMFPKIIEKYINLFEQTKKDVNQIRKRQLATSSLFVVLTVGVFGYGFYWFTNPV

RTGALEVGVLLMFVSVIGYISTSMARVVEDSSLLYDSLLWIEKYFKFLEYQDNFENGS

QNFPDDFDDINVKNLSFTYPFSDTEILHNVSFSVKSGEKIAVVGENGSGKSTLVKLLM

RFYDPTNGKISVDNYDLKDFNIFDLHKNLSATFQDFSRFKLTLQENVITGYSFNKDRV

NNVLKASGLGDLLANDHLNLNTMLAKDFKNGTDLSGGQWQKVALARDLYADGKIEFLD

EPTAALDAKSEAEIYQRFLKENDKKTIFFVTHRLSAVRFADKVLFLDGGKISGFDTHT
NLLQTNPKYKEMYDLQKDAYL"

```
     CDS             553399..555972
                     /gene="mutS"
                     /locus_tag="OCPHGMPK_00526"
                     /inference="ab initio prediction:Prodigal:2.6"
                     /inference="similar to AA sequence:UniProtKB:P0A1Y0"
                     /codon_start=1
                     /transl_table=11
                     /product="DNA mismatch repair protein MutS"
```

/translation="MAKKDTTPMMKQYYEIKEQYPDAFLFYRVGDFYELFEDDAVKGA

QILELTLTHRSNKTKNPIPMAGVPHLAVDTYVNTLVEKGYKVALCEQLEDPKKAKGMV

KRGIIQLITPGTMMQERPDQAKDSNYLTSVISTNSGFGLAYSDLSTGETFSTHLTNFE

GVANELLSLQTREVVYNGHLTEANKDFLKKANITISEPVKVESEHAEISYVAQNLTDD

AEIKATKQLVAYLLSTQKRSLAHLQVAQSYEPTQYLQMSHTVQTNLELIKSAKTSKKM

GSLFWLLDKTSTAMGGRLLKSWIERPLLSVTEINRRQEMVQALLDDYFTREKVIDSLK

GVYDLERLTGRIAFGSVNAREMLQLAHSLGAIPDILNSLLETNNPHLQNFAKQIDPLK

GIHDLIVNTIVDNPPLLTTEGGLIREGVSDQLDRYRDAMNNGKKWLSEMESHEREVTG

INNLKVGYNKVFGYYIEVTNSNKDKVPTDRYTRKQTLTNAERYITPELKEHESLILEA

EAKSTGLEYDLFVKLREDVKKYIPALQKLAKQVASLDVLTDFATVSEQNNYVRPDYTV

DKQEINVVNGRHPVVEQVMTAGSYIPNDVKMDQDTDIFLITGPNMSGKSTYMRQMALI

AIMAQIGCFVPADSATLPIFDQIFTRIGAADDLISGQSTFMVEMSEANDALQHATKRS

LILFDEIGRGTATYDGMALAGAIVKYLHDKVGAKTLFATHYHELTDLDQTLKHLKNIH

VGATEENGKLIFLHKILPGPADQSYGIHVAQLAGLPRKVLREATTMLKRLERQGAGEL

QPASEQLDLFTAEEASEPAISDDEKDVLDDIQNVYLADKTPLQVMELVSQWQQELKDK
D"

```
     CDS             555978..557870
                     /gene="mutL"
                     /locus_tag="OCPHGMPK_00527"
                     /inference="ab initio prediction:Prodigal:2.6"
                     /inference="similar to AA sequence:UniProtKB:P49850"
                     /codon_start=1
                     /transl_table=11
                     /product="DNA mismatch repair protein MutL"
```

/translation="MSKIHELSPELTNQIAAGEVIERPASVVKELCENSLDAGSSRIR

IDFIDAGLKQITVQDNGSGIAKDQLDLAFTRHATSKIATERDLFNISTLGFRGEALAS

IAAVSHVEVITSSDNLGGVRAIFVGSEKKLQEDAASPKGTKISVSDLFFNTPARLKYL

RSERTEILKIVDIVNRLALGHPDVAFTLTNNGKILLKTNGRNDLRQDIANIYGRQLAE

KMEVLKGTSPDFKITGLLSDPNTTRSNRNFISLLLNGRYIKNYRLTQAIIAGYGSKLR

PRRYPIAVVNIELDPLLVDVNVHPTKQEVRLSKEQELERLLTTSISEALDQTSQINSG

LNNLLTPKKSTNIDQLKFNLNKDVVDTVRPVEFIPQVEADKSAEVHETAANFVSLDKV

RNDDKYVITASWDENVDKQVRLSPFDEEKDIQGKDDSLISSGDEILANNLPELIYLGQ

TKSYLVARHNEDLYLIDQIAAYRRLAYDQIFHDLNSENISQQGLLSPLILDFSNVDYL

KLKENLNNLQELGIFLEDFGQNSLILRTYPIWLQPDVEKNVRMILDLYLNQNEQDFTK

LKAQIAGEITMRQSTRRRNLNPVEAQELLKDLRNSSDPYQDFEGKIIIIQLSENDLNK
MFKKDE"
CDS 557876..558454
/gene="ruvA"
/locus_tag="OCPHGMPK_00528"
/EC_number="3.6.4.12"
/inference="ab initio prediction:Prodigal:2.6"
/inference="similar to AA sequence:UniProtKB:P0A809"
/codon_start=1
/transl_table=11
/product="Holliday junction ATP-dependent DNA
helicase
RuvA"

/translation="MFEYLKGIVAKIDPAYVVLDVNGIGYKILCPTPYSYQENQPATI

YVEQVVRDTGITLYGFLSLEDKELFLKLLSVSGIGPKSAVAIMAAEDTDSLASAIQNG

EVKYLTRFPGVGKKTASQIVLDLKGKLGDYVKKSTVAADLSPSLQDAVLALVALGYTQ
KEVDRITPKLAKLPENTADGYIKEALALLLKK"
CDS 558482..559501
/gene="ruvB"
/locus_tag="OCPHGMPK_00529"
/EC_number="3.6.4.12"
/inference="ab initio prediction:Prodigal:2.6"
/inference="similar to AA sequence:UniProtKB:Q97SR6"
/codon_start=1
/transl_table=11
/product="Holliday junction ATP-dependent DNA
helicase
RuvB"

/translation="MKDVNDEERIIGAESSEEDETIELSLRPQLLAQYIGQDKVKSEM

KIYIKAAKKRDEALDHVLLYGPPGLGKTTLAFVIANEMGVHLKSTSGPAIEKAGDLVA

LLSELNPGDVLFIDEIHRLAKPVEEVLYSAMEDFYIDIVVGEGQTTHAVHVPLPPFTL

IGATTRAGQLSAPLRDRFGIIEHMQYYSVDDLEKIIQRSSVVFNTKIDPEAAIELARR

SRGTPRVANRLLKRVRDFAEVKGEEAISLATTKHSLHLLEVDDEGLDQTDRKLLRMMI

ENYGGGPVGIKTIAANVGEDTDTIEEVYEPYLLQKGFITRTPRGRSVTQKAYLQLGYP
PKKAE"
CDS 559577..560014
/locus_tag="OCPHGMPK_00530"
/inference="ab initio prediction:Prodigal:2.6"
/codon_start=1
/transl_table=11
/product="hypothetical protein"

/translation="MNTFFLAQSAAGMNNMFMIIAMIAIFVFFYFSMIKPQKKQQQER

MKMMSELKKGDQVIMVDGLHGKVDSINDADKTVVIDADGIFLTFERMAIRRVLPTAAA
PAKDVEANEAKEEKVETEEKPTESSKTEETTSDKADNTNSEDK"
CDS 560122..561570
/gene="zwf"
/locus_tag="OCPHGMPK_00531"
/EC_number="1.1.1.49"
/inference="ab initio prediction:Prodigal:2.6"
/inference="similar to AA sequence:UniProtKB:P54547"
/codon_start=1
/transl_table=11
/product="Glucose-6-phosphate 1-dehydrogenase"

/translation="MENIPVVMIIFGGSGDLAHRKLYPALFNLYQKGLIHDHFAVIGT

ARRPWSHEYLQEQVVAAIKESNNSFDEKNAKEFASHFYYQSHDVTDVDHYIALKELAT

KLDKKYHAEGNRIFYMAMAPRFFGTIATHINDQKLVGSGFNRLVIEKPFGHDLASAEK

LNQEIGESFAEDSVYRIDHYLGKEMVQNIMPLRMTNPIVNNIWCKKYIANMQVTLAES

LGVGTRGGYYETSGALRDMVQNHIFQIITLLAMPEPKALDSDHIHEAKQELLDSLVIP

TPEMVKKHFSRGQYLASDDEVEYLKADQVAPDSKVETFVAGEVNFKKGPVAGVPIYFR

TGKKMKDKVSRIDIVLHHMNNLYGNAHSNNISIIIDPRSEIFFTINGKKITTEGLRRE

NLSYKFSKEEMDQVPDGYERLLHDVFVADRTNFTHWSELKQYWKFVDAVEAAWQDENK
DIKQLEQYPSGEFGTESSNHIFEKDTEHWIYR"
CDS 561570..562691
/gene="dinB_1"
/locus_tag="OCPHGMPK_00532"
/EC_number="2.7.7.7"
/inference="ab initio prediction:Prodigal:2.6"
/inference="similar to AA sequence:UniProtKB:Q47155"

/codon_start=1
/transl_table=11
/product="DNA polymerase IV"

/translation="MDLLPKNNIKRKIIHLDMDAFYASVEIRDNPALKNKAVLIGGDP

KKNRGHGVVATANYLARQYGAHSAMPTAKAVRLIPAEKLVIIQPHFEKYRAVSAEIHR

LMHEMTDRVESVSLDEAYLDVTENKLHLDDPVRIATILQEQIYQKVGLTSSFGVSYNK

FLAKMGSEYAKPFGRTVIKPETALDFLAKQKIEKFPGIGPKTQERLAEMGVYTGADLI

KIPTDVLIKKFNRMGYLIAQHAHGIDLREVVTDSERNRKSIGIERTFNQSLFDENEAL

TKLRAYSGELENKLKKRHFLANCVVLKIRDVNFKTITKRKKLKQGTNDKIVIYDTGRV
LFETEKGMLATGVRLLGLTVTDFEEHPVENLSLDIFENK"
CDS 562732..563697
/gene="nrnA"
/locus_tag="OCPHGMPK_00533"
/EC_number="3.1.-.-"
/inference="ab initio prediction:Prodigal:2.6"
/inference="similar to AA sequence:UniProtKB:Q8DTN6"
/codon_start=1
/transl_table=11
/product="putative bifunctional oligoribonuclease
and PAP
phosphatase NrnA"

/translation="MATFDEIYEKIKEYPTIILHRHTSPDPDAIGSQAGLARSLRLAF

PDKKILCAGENDEGDLIWINKMDEVTPEDYQGALVITTDTANTPRISNKNYDKGDLLI

KIDHHPDVDPYADMSYVDPDAPAASEIVFDFLKAENLPVTKEVAAALYAGIVGDTGRF

MYPETSAHTFDVAAELTKTGINITDIAREIADVTFDEAKLQALAMDKMEINPVGAAYT

ILMQDDLKKMGLTDDQANVAVSTPGRIKDVLAWNVFVEKPDGTFRVHYRSKGPVINHL
AEKHDGGGHALASGANAKDMDEVKQVFDEVVEVTKKYNEEHGTNK"
CDS 563672..565066
/gene="cshB"
/locus_tag="OCPHGMPK_00534"
/EC_number="3.6.4.13"
/inference="ab initio prediction:Prodigal:2.6"
/inference="similar to AA sequence:UniProtKB:Q8Y755"
/codon_start=1
/transl_table=11
/product="DEAD-box ATP-dependent RNA helicase CshB"

/translation="MKNMAQTNSIFQSEKIRPELRTGLEKINFSKPTKVQAAVIPALL

NNKNVVVQAATGSGKTHAYLLPLLNMIDENAPVTQAVVTAPSRELANQLYKVARQLRD

ASGLNISIEYLGGGNDRNRQIEKAENRAPQLIIATPGRLHDFASKKVINLDNVKAFII

DEADMTLDMGFLGQMDEIISKLDKKVVLGAFSATIPVKLENFLRKYMSKPDFIVIDNP

AIIAPTIQNDLIDVGSRDKKEILFKLLTMGQPYLALVFANTKKTVDELTDYLEEQGLK

VAKIHGGITERERKRIIRQVREGQYQYVVASDLAARGIDVPGVSLVVNYEIPKDLEFV

IHRIGRTGRNGLKGHAITLIYDEEMPQIEDLEKLGIHFDFKEIKNGELVERTHYHRRD

NRQARSHKLDNRMIGMVKKTKKKVKPGYKKKIKQAIQKDRQQKRKLEERHQIRKAKRR
RKREREQARGNFDN"

```
     CDS             565297..567945
                     /gene="alaS"
                     /locus_tag="OCPHGMPK_00535"
                     /EC_number="6.1.1.7"
                     /inference="ab initio prediction:Prodigal:2.6"
                     /inference="similar to AA sequence:UniProtKB:P67011"
                     /codon_start=1
                     /transl_table=11
                     /product="Alanine--tRNA ligase"
```

/translation="MKQLTSSQVRQMFLDFFKEHGHMVMPSASLIPQDDPTLLWINSG

VATMKKYFDGSVVPKNHRITSSQKSIRTNDIENVGKTARHQTFFEMLGNFSVGDYFKK

EVIPWAWEFLTSPKWLGLDPDKLYVTVYPKDTEAYHMWHDVVGLPEDHIVKLEDNFWD

IGEGPCGPDSEIFYDRGQENNDVAEDDPENFPGGENARYLEIWNIVFSQFNHLPNGKY

VDQPHKNIDTGMGLERVVSIIQDAPTNFETDLFMPIIKETEKLSDGKKYAANKEDDVA

FKIIADHVRAVSFAIADGALPSNSGRGYVLRRLIRRADLNGQRLGIKGAFLYKLVPVV

GEIMKSHYPEVVDQQAFIQKVIKNEEERFQVTLSSGLNLLDNIIAEAKKSDDKTVSGK

DAFKLFDTYGFPYELTFEAAQDAGLKVDKKGFDEEMKAQKERARKARGNLQSMGSQDV

TLMNIKDKSEFEYGTLEEKHAKLIDIVVDDKLVDKADGEHATLIFDKTPFYAERGGQV

ADHGEILNQNGELVARVTDVQHAPNDQNLHFVDIILPLEKGQEYVLKVDQKRRRGLKH

NHTATHLLHAALREVLGTHTHQAGSLVEPDYLRFDFTSLEPMTKKEIANVEKIVNEKI

WEEIPVKTTVTDPDTGLKMGALALFGEKYGDTVRVVQIDDFSTEFCGGTHCENTDQIG

MLKIVSESAVGAGTRRIIAVTGPEAYKYVTDRDEILKEVQDEVKATKAEDVTNKISSL

EEDLRASQKEAEQLKAQINKAKAGDLFNDVKQVKDLTVIAAQADVEGMNDLRELADNW

KSSAKSDVLVLAAEVNGKANMVISLNDKAIKAGLKAGDLIKTAAPIFGGGGGGRPNMA
QAGGKNPAGLKDAIAKVLQEVEEKQN"

```
     CDS             568000..568257
                     /locus_tag="OCPHGMPK_00536"
                     /inference="ab initio prediction:Prodigal:2.6"
```

```
                     /codon_start=1
                     /transl_table=11
                     /product="hypothetical protein"

/translation="MSSLDKTMHFDFNQNKGKNVYDTLQDVYNALEEKGYSPINQIVG
                     YLLSGDPAYIPRHNDARNLILKHERDEIIEELVKSYLGKNK"
     CDS             568257..568688
                     /gene="yrrK"
                     /locus_tag="OCPHGMPK_00537"
                     /EC_number="3.1.-.-"
                     /inference="ab initio prediction:Prodigal:2.6"
                     /inference="similar to AA sequence:UniProtKB:O34634"
                     /codon_start=1
                     /transl_table=11
                     /product="Putative pre-16S rRNA nuclease"

/translation="MRLLGLDVGSKTVGVAISDPLGITAQELETIKIDESKFSFGMRQ

IRKIVRKYDVEGFVLGLPKNMDGSSGHSVERSKQYGERLKEKFDLPVHYMDERLTTVQ
                     ADRILVQEAGVHDRVERKKVIDQMAAVLILQSYLEATRKDK"
     CDS             568690..569004
                     /locus_tag="OCPHGMPK_00538"
                     /inference="ab initio prediction:Prodigal:2.6"
                     /codon_start=1
                     /transl_table=11
                     /product="hypothetical protein"

/translation="MSEKINANQDNDRQITLVDDQGNEELFEILFTFTSEDYGKSYVL

LYPAAVSDDDDVEVQAFSYDADEDGDVTSSDLHEITDDDEWNMVQGVLNTFLSDDRLS
                     GE"
     CDS             569060..569602
                     /locus_tag="OCPHGMPK_00539"
                     /inference="ab initio prediction:Prodigal:2.6"
                     /codon_start=1
                     /transl_table=11
                     /product="hypothetical protein"

/translation="MISILILLIFIYCCYVGYRRGIVYEGLAAGGYVVGLIFATLLYK

PFSNFLNLWVPYPSASDRSSFAFFDKTTGLTLDKSFYAAIAFSIVLLLVCCIWRLVML

GFNQLRYITVDARLNTWGSIIIAFIVTQISVYLLLFILATIPNNSLQNMLGHSILASG
                     ILHFSPGISQIFIKLFITTI"
     CDS             569646..572012
                     /gene="mutS2_2"
                     /locus_tag="OCPHGMPK_00540"
                     /EC_number="3.1.-.-"
                     /inference="ab initio prediction:Prodigal:2.6"
                     /inference="similar to AA sequence:UniProtKB:P65496"
                     /codon_start=1
                     /transl_table=11
                     /product="Endonuclease MutS2"
```

/translation="MNSKIIEKLEYNRIIKQLSDLAITAPAKAQALKLMPSSDFDEVK

KSIDQTRVLSNILRVKGPMPITDFKDVRPSLKRLKVKANLNGEELGNIFLVLSLAKDV

GQFTSDLEEREIDTRPIEKYLKNLAVSEDLFKKLNQSIEYDGTVKDTASSKLMQLRHD

IQSNETDIKNHMNDYISGKHTQYLSENIVTIRDGRCVLPVKQEYKNKFGGVVHDQSAS

GQTLFVEPQAVLVLNNRQQNLLAQERQEIHRILIELSELAGTYQKEIKNNAVALAQLD

FLSAKSKLAKKMKATEPVLNQDHVIKLRKARHPLIDPKKVVPNNIELGTTFDTMLITG

PNTGGKTITLKTLGLLQLMAQAGLFITAEEGSQLTVFNEIYADIGDEQSIEQSLSTFS

SHMDQIIKIMNNVTEDDLVLIDELGAGTDPEEGASLAIAILDDLRQTQAKIAITTHYP

ELKLYGYNRERTTNASMEFDLKKLAPTYRLRIGIPGQSNALAIAHQLGMNEAVVDKAR

SLMNDEDSDINKMIERLTEQTKAAEQLHETLKQNVDQSITLKRQLQNGLDWYNQQVQK

QLEKSQEKADEMLAKKRKQAEKIINDLEEQRRAGGQVRTNKVIEAKGALNKLERENQN

LANNKVLQREKKRHDVSVGDNVKVLSYGQQGVITKKLGDHEFEVQIGILKVKVTDRDV

EKIAAQANQKKPEKSVRSSRGLRSSRASSELDLRGQRYEEALTNLDRYLDASLLAGLN
TVTIIHGIGTGAIRNGVQQYLKRNRHVKSYNYAPANQGGTGATIVNLQ"
CDS 572092..572403
/gene="trxA"
/locus_tag="OCPHGMPK_00541"
/inference="ab initio prediction:Prodigal:2.6"
/inference="similar to AA sequence:UniProtKB:P14949"
/codon_start=1
/transl_table=11
/product="Thioredoxin"

/translation="MVDEITDATFEDETSEGVVLTDFWATWCGPCKMQSPVIDQLSEE

MDDVKFTKMDVDQNQETARNLGIMAIPTLLIKKDGKIVDRLTGYTPKEKLEQILDQYT
D"
CDS complement(572489..572884)
/locus_tag="OCPHGMPK_00542"
/inference="ab initio prediction:Prodigal:2.6"
/codon_start=1
/transl_table=11
/product="hypothetical protein"

/translation="MEHTNEHLYFLNSLYRDFILPTILGSEDKEILYWAGKHVSRKYD

LSDIDDLIEFFDMAQFGTLKVLKDRKHTAVFELSGQVVTDRLDSQSDEFSLESGIIAE
CLERQNGTPTEASATITKKHVVQITAQSD"
CDS 572981..573781
/gene="murI"
/locus_tag="OCPHGMPK_00543"

/EC_number="5.1.1.3"
/inference="ab initio prediction:Prodigal:2.6"
/inference="similar to AA sequence:UniProtKB:P63640"
/codon_start=1
/transl_table=11
/product="Glutamate racemase"

/translation="MDNRPIGVLDSGLGGLTVLKKVIEKMPNESTIFIGDQANMPYGD

RSKEEIISLTRDSVNFLLSKDVKIIIFGCNTATAVAMSTIQKEVPLQIIGVVQSGALA

AARTTETKNVAVIGTKATVNSHSYLKEIQYRDPEIQVSEFAQPKLAPLAEEDPAEEIK

QAVVSESLAPLKNIDYDTLVLGCTHYPLLRDEIVAVVGQDKKIVDPADQVAQYTYNVL
RRDGLFAADDSNTTHEYYTTGEAKKFTEITRQWMNDETIVGHHVDAED"

CDS 573783..574406
/gene="rdgB"
/locus_tag="OCPHGMPK_00544"
/EC_number="3.6.1.9"
/inference="ab initio prediction:Prodigal:2.6"
/inference="similar to AA sequence:UniProtKB:P52061"
/codon_start=1
/transl_table=11
/product="dITP/XTP pyrophosphatase"

/translation="MDTLLFATNNKNKAKEVEEALQKNNFPIHVITNQDLTDPPHVLE

TGTTFLANAKLKAHQMAEFSNLPTLADDSGLSVDKLNGAPGVYSARYGGEAHNDALNN

AKLLAELGGVPKEERKATFHTTMVVSWPGKFEDDLVTEGEIRGEILTYPQGKGNFGYD
PLFFVPDKGKTFAEMTVDEKNAISHRGQALRKLLTELPAWWKKMENK"

CDS 574542..575720
/gene="ackA_1"
/locus_tag="OCPHGMPK_00545"
/EC_number="2.7.2.1"
/inference="ab initio prediction:Prodigal:2.6"
/inference="similar to AA sequence:UniProtKB:P37877"
/codon_start=1
/transl_table=11
/product="Acetate kinase"

/translation="MKKVLAINSGSSSFKYKLFSFPDEKVIASGMADRVGMENAVFKI

KLSNGREYIKNMPIHDQEEAVKLLIEDLKKFHVVEDLREITGIGHRIVNGGEIFKESV

RVGDKELQEIFDLGELAPLHNIPEANGIKAFLKIVPNVPQVAVFDTSYHQTLDPIHYL

YSIPYEYYEDYGIRKYGAHGISISYVAPRAAKMLKKNPNLVNLIVCHLGSGASVTAVK

RGKSYDTSMGISPLTGVTMGTRSGDFDPSALQRLMHKTGMNIDEAIDVLNYKSGLLGI

SGVSSDMRDLIESKDKRAKLARKIFINRVVRYVGAYAAELGRIDAVVFTAGVGEHDPG

IRAGIMSSLRYLGLEPDFKANRTDGEKFISKPKSRVKALIVPTNEELMIAREVVRVVQ

"
     CDS   complement(575763..576659)
                     /gene="ykuT"
                     /locus_tag="OCPHGMPK_00546"
                     /inference="ab initio prediction:Prodigal:2.6"
                     /inference="similar to AA sequence:UniProtKB:O34897"
                     /codon_start=1
                     /transl_table=11
                     /product="putative MscS family protein YkuT"

/translation="MNWPTLLKGTSTTKKTNNIDSLINWDNVLHTLLSRGIQIIITTI

IFFLIWHIGKRLLTKYLLKNPKFQERMTGRKRTLAQLGIALFQYTVLLFYLYSVLTLM

GIPVGTLLASVGLVSLALGMGAQGLVSDVITGMNILSEGEYNIGDTVKIGNYTGTVLS

FTLRNTRLKTTNGAVIYIPNRNITVVENLSHGSHSGWSMDINLNLSVENNLTEINHAV

DTVNAALKDKFKKQIKKGPQIIGIINQTGSSITYQIHFQVIANSQSTIKNAYLSAYIK
                     EFNQRGLKFSPMPTKQENSSGS"
     CDS   576786..577217
                     /locus_tag="OCPHGMPK_00547"
                     /inference="ab initio prediction:Prodigal:2.6"
                     /codon_start=1
                     /transl_table=11
                     /product="hypothetical protein"

/translation="MSISYGALAGLIAAVAFLILVLFTLPLIVRVTKTMKKVDATMDS

VNTAVDDLTKQTSVLMKQSEDLLEKSNALLADVNGKVTELEPVVKAAADLGESLSDIN
                     SSSRRMVERFSGMGIRGAGIGIFSSLVSRMFARRKRRRGED"
     CDS   577240..577581
                     /locus_tag="OCPHGMPK_00548"
                     /inference="ab initio prediction:Prodigal:2.6"
                     /codon_start=1
                     /transl_table=11
                     /product="hypothetical protein"

/translation="MRKLGGFLLGSVVGLGVGLIAGSLLLPEDATDDVKKKLAENEKL

QDLKEKYDKGTEAIKNQLASFPKSVEDDSELRDFDDIVIDDTNKDLGEDEKSDKDAVS
                     DLNNAEDTENN"
     CDS   complement(577673..578779)
                     /gene="pepQ"
                     /locus_tag="OCPHGMPK_00549"
                     /EC_number="3.4.13.9"
                     /inference="ab initio prediction:Prodigal:2.6"
                     /inference="similar to AA sequence:UniProtKB:Q9S6S1"
                     /codon_start=1
                     /transl_table=11
                     /product="Xaa-Pro dipeptidase"

/translation="MNLDKLQNWLISTNNDVAYISDPISINYFTGYSMDPHERIFALL

AFKDKPAFIFAPELNVEEAKNSAWDGDVYGYLDHENPWAKIADLVNQRVGKPNNIAIE

KSHLSVDRLEQLRIAFPDSSFTNNVSPFVAEARLRKTPEEVKQLKAAGAEADFAFQIG

FNALRNGVTERYVAGQIDYQLKLQKGVMHTSFETIVQAGTNAANPHLGPTMNKIEPNE

LVLFDLGTMHNGYASDSSRTVAYGEPTAKEKEIYEVDREAQQAAIEAAKPGITASELD

AVARDIITKAGYGEYFIHRLGHGIGMNVHEFPQIMEGNDVVLEEGMCFSIEPGIYIPN
VAGVRIEDCGVVTKNGFETFTKTSKELKYIPVKD"
CDS 578923..579918
/gene="ccpA_1"
/locus_tag="OCPHGMPK_00550"
/inference="ab initio prediction:Prodigal:2.6"
/inference="similar to AA sequence:UniProtKB:P99175"
/codon_start=1
/transl_table=11
/product="Catabolite control protein A"

/translation="MQKQEVTIYDVAREAKVSMATVSRVVNGNNNVRKETREKVLAVI

DRLHYQPNAVAQGLASKRTTTVGLIMPDLTNMHFAELSKGIDDIATMYKYNILLSSVG

DTLLNEDQVIQNLLNKQVDGVIYMSNLMSEKAQEIFKRTNTPVVLAGTADANQDFPSV

TIDFKAAEKEALGLLLKNGKKNLALVVGDGKAYVNKDNRFVAYKDFMEEHNLKNVHIY

EDAKTYEDGYKLFKQIKADKVDGIIATRDVTAAGIVNAAVHAGVKIPEGLEIISAAST

NVAKIVRPQLTTVQQPLYDIGAVAMRMLTKLMNDEELDDAHVILPYTLKKSRSTK"
CDS complement(579999..581633)
/gene="msbA_1"
/locus_tag="OCPHGMPK_00551"
/EC_number="3.6.3.-"
/inference="ab initio prediction:Prodigal:2.6"
/inference="similar to AA sequence:UniProtKB:P63359"
/codon_start=1
/transl_table=11
/product="Lipid A export ATP-binding/permease
protein
MsbA"

/translation="MELLKYFKKLKWEFLLVVFLIVVNAGFLTLAGISSANALSSVAK

LRAAEFFMWVAIMGGAYIIYAIVNCLVNVEQTRLSQNVDKLIRNSIATDLSKANYATF

HKQTVATYSSWLTNDITTINNFGITDFLMIVRQVSEIVFGMLTLAYFNISLVVTVVVL

TVIMGIVPNMFSKILAKRSLEYTHANERLVNTINDILNGFNTLFLANLPQTIVKKIDG

SSDDVKKHTLNYSKTAGITQAITNGLAFISQVIILGQTGWLILHHLTPVGTISGAQFF

ASTIFAELSGISFNWQEFKSIKPIMVKFKSVSNNESETHSISNWQLGKLNLKNVSYHY

TESDEPIFHNLNLDFILQNKYILTGDSAAGKSTILNLIGGLLRDYKGEINLGNMSYDQ

ISDHDLHQKISYLQQDPYIFSASLKWNLTLGKKISTQKINKVIKECGLEDLITKLPNG

IDTVLDDQGKQLSGGQKQRIAFAREILRDTPIYLLLDEATSALDKASSTQLERLILTKQ
DKTVIMVTHHLRNEIEQLANKVVNLNEIKNTASETI"
CDS complement(581800..584628)
/gene="pbpF_1"
/locus_tag="OCPHGMPK_00552"
/inference="ab initio prediction:Prodigal:2.6"
/inference="similar to AA sequence:UniProtKB:P38050"
/codon_start=1
/transl_table=11
/product="Penicillin-binding protein 1F"

/translation="MKNLKEKIIEFLTAGPEVKTLQSESDESQWKFYSGIVYLTLRRV

FHYLILIAVFSLFLLIGFGGGYALGIVRQQPIPTISELNQQINHAQNSATLYYAGNKK

IATVRSDTVTKKASESELTPLVKNAVTATEDENFYIHKGVLPKSLVRAVLSELTGVGV

QTGGSTLTQQLVKMQFLTSQTTWRRKVTEMFYAHKIEKHFSKEDILRSYLNAAPYGKN

NRGENIVGIKTAAQGIFGKSISELNLPQAAFIAGLPQSPSIYTPYRLNGKIKKDLDLA

MRRKDIVLFRMYRNGDISKKDYLAAKKYDLRADFLAPEKASKQKKQSGYLYNLVMNKS

TSLLAEKLIEQDGLKVSDVKQDTNRYNQYLTSATELLHQKGYHIKTTIRQPLYQTMQR

IVKQNKYGQDKTSRDFDSSTNKWVNTTEHVENGSVVIDNATGKVLAFSGGVDFKNSQI

NHAFDTYRSPGSSIKPYLVYGPAIEHKLISSQTALADFPTRFGNYIPTDYNSTVENRF

ISAQEALSKSYNLPAVNLYSKLVNDKNINLRQDMKKLGLNLSKSEFENLGLALGGTDY

GFSVADNASAFSNFYNNGKRADPYYIEEIRDPSGRVIYKHKQNPQKVFSTGTSYIMQK

MLHQVVTKGTASSLTGTLKFNYKNLIGKTGTSNDYRDIWFNGSTPGITISSWMGYDNF

YGHSYNLDSNSSETNLNLWANIANALYEEDPSIFKLNDAPSRPSSVYKNRVLARTGTL

PGTVSYDGDNIELSGKKTTALSLSPAPAATARFGIGGSTKDYNLFYDYLEGKNNDYGK

VLIYTGKTISKKKNINSLFAVADGSTSEDAKNYYGKNHEVYRESNNNSSSSTNRNVGA

NDRQARSTGQGGNNNGSSSSTSTRRNRSSSTGESSSSAESSNSESNNAANDVNSNISS
NEGSAANTNTSSETTEGSTATNNGPTAP"
CDS complement(584726..586123)
/gene="pepV_1"
/locus_tag="OCPHGMPK_00553"
/EC_number="3.4.13.-"
/inference="ab initio prediction:Prodigal:2.6"
/inference="similar to AA sequence:UniProtKB:P45494"

/codon_start=1
/transl_table=11
/product="Beta-Ala-Xaa dipeptidase"

/translation="MEELDYKKLAQEKKDAILNDLKELIAIDSSEDLNNTSKEYPVGP

GPVKAMKKFLSFAKRDGFDTENFDNYAGRINMGSGDKRVGIIGHMDVVPAGEGWKTDP

FKMTIKDGKIYGRGSADDKGPSLAAYYGMLILKEHGFKPKKKIDFVVGTNEETNWVGI

DYYLKHQPAPDVAFSPDAEFPIINGEQGIVTLKLDFKDDPNQGDVKLWTFQSGIATNV

IPQTAHAQLEGDIDGIKEKFNLFLKEHKLEGKAEMLSGRLSLTLTGHGAHASAPETGR

NAATYLALFLDSLNFDGQAKNFLHFLATVEHKDFNGKKLGIFHHDDLMGDLTSSPSMF

NFEGQNAYLLNNVRYPQGIEPEEMVKNINEKFGNILDARVDGSAEAPHYVPGDDPIVK

TLLSVYEKQTGKKGHEVIIGGGTYGRLFKHGVAFGAQPEGAPLVMHQPNEYMKVEDLI
NSIAIYAEAIYELTK"
CDS complement(586141..587523)
/gene="glpQ_1"
/locus_tag="OCPHGMPK_00554"
/EC_number="3.1.4.46"
/inference="ab initio prediction:Prodigal:2.6"
/inference="similar to AA sequence:UniProtKB:P37965"
/codon_start=1
/transl_table=11
/product="Glycerophosphodiester phosphodiesterase"

/translation="MKTSRHYFLALFFSLIFIFESGFLVIGHRGNPSKYPEETIQSDN

SAFADGADYVELDLHVSKDNVLVVSHDRDLSRVVGSSVIVSQNNFSYLNTLKQANGES

IISLDQLFDYYKDKPNTKFLLETKKTKHNSPKNMEELLAASIKKYHMQDRVMIHSFSA

PSLKTMSQLLPDVPRIFIVGSLQRINFEVLSYVNGINISSDLITQNPKLISQLHALHQ

KVFVWAEMNETPKLWNWLINNDVDGVVTNFPAKGYKYKLAKTGTKKYDVNKDGIYFGR

MPTAISENPYLKVTTSKELNFLDPVHVSSAVIASGQTFYQIGDKEFVPADLVSLDLQP

EWILPYWNLSIINPTQNPIFTYNKPDKQSGKKNRLMPRKHYKILGVSGGVKDLWLLTN

DGWVKSSKVLYYGLFNKNTFAYKNYQKLDPAYRQTNVALFPYLPVEKVPNKSFWSTYS
DVNAVIFNQR"
CDS 587624..587950
/locus_tag="OCPHGMPK_00555"
/inference="ab initio prediction:Prodigal:2.6"
/codon_start=1
/transl_table=11
/product="hypothetical protein"

/translation="MAYEQLDLIEEVTRNDGSKYYEISNIDQNGIAELAVDRGLIKKV

```
RILQLNLARTKALQLYEEYINKTYQLETLTNEDDWKDPQWVEWEKPKGKVLDAFNTVL
                     KANHIG"
     CDS             587964..589331
                     /gene="mggB"
                     /locus_tag="OCPHGMPK_00556"
                     /EC_number="3.1.3.-"
                     /inference="ab initio prediction:Prodigal:2.6"
                     /inference="similar to AA sequence:UniProtKB:A9BJC1"
                     /codon_start=1
                     /transl_table=11
                     /product="Mannosylglucosyl-3-phosphoglycerate
phosphatase"

/translation="MEKLRIVHTNDLHSHFEQFPKIKRYLHQAQNDKSVDQTFTFDAG

DFMDRSHPLSDATEGQANIKLMNEFNYDAITIGNNEGISNSHAVLEKLFDHANFPVIL

ANLREEDESMPKWCTSYKIFETKKKTRIAVVGLTAAYPMTYGPNHWHVKLISDTLDRL

LPTIKGQYDMLIIVSHIGLRMDRYIAYHYPEVNLIVGGHSHDYLPKGEKVNKTWITQT

GKWGQHVGDIAIQLNDNHQIVSIVPNTVKTADLPSLESDQAKIAAWRKQGEQMLQSRQ

IAKLPAKFNNDKMAAIQVSLDAISNFAGTDIAVLNSGLFLTPFHQGILTEADLQAALP

HPMHVVQTKLLGSDVWRMVMEMEKNRHFLSKFALKGMSFRGKIFGDLVYKGIQVDHAS

RTVYINGKEIDPEKEYTLALLDHYVLIPFFPTISIMGKNKFLFPDYLRTVIGNYLKEK
                     YPIDK"
     CDS             589343..589888
                     /locus_tag="OCPHGMPK_00557"
                     /inference="ab initio prediction:Prodigal:2.6"
                     /codon_start=1
                     /transl_table=11
                     /product="hypothetical protein"

/translation="MEYKEADKEQPLYHPLAHVIVNEDTIMINGRKYEILANVKDALD

IELLKEKYDPYLDQYDYLVGDISSEHLRLKGFYDEKDRVAIDKKANAIVDYLEEYCNP

GSAYFVLRLAQENQIKKVAFGRQNKRKNMSNNRYHSKRPYFKERRVHKTRIGGHKTAV
                     KFQRGKGSHKNRGFVIKKRKG"
     CDS             589891..590673
                     /gene="yutF"
                     /locus_tag="OCPHGMPK_00558"
                     /EC_number="3.1.3.-"
                     /inference="ab initio prediction:Prodigal:2.6"
                     /inference="similar to AA sequence:UniProtKB:O32125"
                     /codon_start=1
                     /transl_table=11
                     /product="Acid sugar phosphatase"

/translation="MEHKDYKCYLIDLDGTIYRGSDTIESGVRFIHRLQEKNIPHLFL
```

TNNSTRTPQMVVDKLRGHGVNTDIYHIYTPVLATESYLLAQNPDTAKIPVYIIGQTGL

VQGLLKNERFYYDDRNPKYVVVGMDTDLTYHKIRVATRSIRNGATFIGTNADKNLPSG

DELLPGNGAFCTMLEVATGVKPIYIGKPSSIIVASALKMLNAQGKDAILVGDNYDTDI
MAGVNCNIDSLLTLTGVTTKKQLAERDKQPTYVVENLDKWKL"
CDS 590670..591278
/locus_tag="OCPHGMPK_00559"
/inference="ab initio prediction:Prodigal:2.6"
/codon_start=1
/transl_table=11
/product="hypothetical protein"

/translation="MKKKPIVAVVYNCLMALATATLGAVVLSWPLLAVFVKIQKTNLI

VKTTLATVMKNYTQLLDYLLLPFNDKLQMSNFPTSSSAARHFYECKLLFELAIIVFVV

GLLILIFLKMRKRMSYIYISRTTALIFMILPVIILPFALMNFDEFFIAFHHLLFNNGD
WLFNPATDPIINVLTEEFFAGCFATGGIIYELYFSCFILSKK"
CDS complement(591286..591936)
/locus_tag="OCPHGMPK_00560"
/inference="ab initio prediction:Prodigal:2.6"
/inference="similar to AA sequence:UniProtKB:P9WP09"
/codon_start=1
/transl_table=11
/product="putative membrane protein"

/translation="MGLIDFILHIDDHLVTIVNMFGNWTYLILFAIIFIETGLVIFPF

LPGDSLIFACCAMAATPKYNLHFWVCYLIFIGAAVLGDTINYEIGHWSQAEGTKHSWF

NKLINQDKRKAAENFFDRHGGITIVIGRFIPFIRTFVPFVSGASKMHYRTFIIYNFIG

GFLWVTLFSALGFFFGNIPVVQEHFSLIVIAIILISVLPVLIIWLKKKLVLKKELR"
CDS complement(591963..592919)
/gene="yumC"
/locus_tag="OCPHGMPK_00561"
/EC_number="1.18.1.2"
/inference="ab initio prediction:Prodigal:2.6"
/inference="similar to AA sequence:UniProtKB:O05268"
/codon_start=1
/transl_table=11
/product="Ferredoxin--NADP reductase 2"

/translation="MEKYDLAIIGTGPIGLFAASFANLHGLKTISFDALDEVGGQINM

LYPQKNIKDIPAFPSIKGEKLVSQLLEQNTNTKFILSHKVKEISFLENENILIDKTYE

VKSLLIATGLGAFKPKTLPLSTTLDIQKHIHYSMQHPEIFANKKVAILGGGDSALDWA

LELATTSDIFLIHRRNEFRGLESSVNKLKSLKNVELLTPYLPKDVQLNNNRMELVLHK

VGASQDFVTKNVDEILVAYGFKSDNRQLRKWGIKLDHNLIAVSQQMHTNLPHVYAIGD

AITYPGRVPMIALGFGEAQIAISNIMQDLFPEKTMTLHSTSI"
CDS 593020..593385
/gene="yugI"
/locus_tag="OCPHGMPK_00562"
/inference="ab initio prediction:Prodigal:2.6"
/inference="similar to AA sequence:UniProtKB:P80870"
/codon_start=1
/transl_table=11
/product="General stress protein 13"

/translation="MQVKIGDIVHGRISGIQQYGIFVRLDSKVEGLIHISEIHGGYVK

DIGREYQVGETIKVQVIDIDPYSNQISLSRRAVLPEVKEARKKRVHFWTSKRVKKGFT
PLKEVLSTQIKEAKNRYSK"
ORIGIN
1 ttttatatc ccgcacactt tcagcgaaac tttgttcagt tttcaaggta
ctaccgttgc
61 cgtgtgacaa cttttatatt atatcatgcc tcttgagttt tgacaagtct
tttttcctct
121 ttctcatctc tctcaaatga cagctttata atattatcat ctttctgctc
tcttgacaag
181 cttctcgctc tgcttcttgc ttgcctcaaa cagcatgtat tactttacag
acttttctcc
241 ttttcgtcaa cccctttttt ctaaaaatta gcaatatttt tttagagtgc
taaaaaaggt
301 ctcattttgt gagacctttt attgtttttg taatttagaa tgtcttacac
catacgttac
361 atatacaact attccagcta ttaaccaaat tcctaccata atttttgtca
tttttggcag
421 catccagatg aaatatacgc tcagcaatcc agatattatt ggtaaaaccg
gataaaatgg
481 cattttgaat ccattattta caatattttt tctatgacga agcggaataa
ttccaaaaga
541 tattaaagca aaagcaatta aagtgccagc atttattaaa gacgctaatt
ccatcaatgg
601 gactaaccca gcaaaaatag ctgctactat agtagaaacg attacagcat
gatcaggaat
661 catcttttta gcatgtacac tccccattga gtcaggtaat aatccatctc
ttcctaaagc
721 atagagtaat cttgatccac ctagtagcat tgtaatcatt gccgtaaaaa
ttcctaccaa
781 cgccccaact gtgattaact tattccaagc agttaatccg acaactttta
gtgcatatgc
841 cgctggatcg tcaacattta atcgcttata gttgaccatc ccagtcaaaa
ctaaagaaaa
901 actaacataa agtaatactg caataattac tgttcccata attcctttaa
ctacattttt
961 agcaggatct ttagtttcag cactattagc tgctaaagcg tcaaagccaa
tgaaagcaaa
1021 gaatactgtc gctgctgctg tagaaattcc acccattcct aatggcggag
tttgaaattt
1081 cttaggataa aaaggaacat aattttctgc cttaatatag aaaagtccaa
tcacaataaa

1141 caaaatgata atagcaacct taataattac tgctatgttt tctacccgtt tcgataaatt
1201 agcgccttga tataaaatta gagctacaat caagacaatc aagactgcag aaagattaat
1261 tactccgccc tccattggtc ccgcttcaag agccttaggc aaatgaatac caattggcgc
1321 caaaatatta ttattaaaat aagaagcaaa acctgttgct tcggcagaaa cagccaagaa
1381 atattctaat agtagtcccc atccgattat ccaacccaca atttctccgt aaatcactga
1441 gccaaaagaa taagcagatc cagccactgg catagcagag gagaactcag catatgccat
1501 tccaaccata ccagaaacaa ttgccgctag caaaaacgaa atcgcaacag caggtccagc
1561 atgttgagca gcttcatgac caggtaaaat aaaaatgcct gttccaataa ctgccccaat
1621 tcccagtgca actaaatcac gtgcatttaa atgtctagtt aagcgagaat ccgcttttaa
1681 aaaagtattt actggaactt tcttaaagaa atttttcatt ccaattctcc ttaatttatt
1741 tttaattatt atacctgatt ttgtcgggaa ttctacccta taattaaaat taattaaaaa
1801 aagagacggt gaattaatcc accgtctctt ttcaagcaaa atagtattaa ctatttattt
1861 tctttttat cagcttttc agcttcttta acgtctttct tttcttgttc gcgtgtttct
1921 ttatcaaaca atacgtcttc aagtacttca ccatttaagt ataagtgctt atcgtcgatg
1981 gtgaacttaa cttccttagt atcaggctta gaaacaatta acttagcagc tggggtttca
2041 agatcttgtt caacaactct tctcaatgga cgagcaccga attctttatc atatcccttc
2101 ttagccaagt acttcttagc atcagcagta acatgaagtt tgatgtttct cttagcaagt
2161 gatttctcca tcttagctaa gtaaagatct aaaatcttaa gcatatcttc gctagtcaat
2221 gaattgaatg gcacaattgc atcaagacgg ttcaagaatt ctggtctgaa gtaaacttca
2281 agtgccttac gtaaagcatc ttgatcgtca cccttaagta acttatcaga gtatccagcg
2341 tttgaagtca tgatgatgat agtatccttg aaggaaattg ttctaccttg tgcgtcagtc
2401 aagcggccat catccataat ttgaagtaag gcattgaaga cttgtgggtt tgccttttca
2461 atttcgtcaa gcaagatcaa agaatatggt tggtgacgaa tcttttcagt caattgtccg
2521 ccttcaccat aaccaacata acctggtgct gaaccaatta acttgttaac agccatttgg
2581 tcttggtatt ctgacatatc caaacgtact aaggcatcag ccttaccgaa caacttaatt
2641 gctaactgct tagccaattc agtcttacca acaccagtag gaccagtaag taagaatgaa
2701 ccagttggac ggttagaatc cttaaatact tgcttacgag caattgcatc agtgatgata

2761 tcaatggcct tatcttggtc aatgacaacc ttcttcaagt cagcagctaa
gttagcgttc
2821 ttttgagctt catttgcatt aatttcactc attggaatac cagtcttagt
ttgaatcaaa
2881 gagtacatat cttctggtgt aactttaagt tcactagctt tacccgcagt
cttgagatct
2941 ttcttgaggt cttcaatttc tttcttaagc ttagcagctt catcatattt
ttcattttta
3001 gctgcttctg ccttttttacc ttctaaggca tgaatcttat tcttgattga
agtttcatcc
3061 ttttgatcca tccctaagga cttcttagca ccagcttcat ccattaaatc
aatggcctta
3121 tctggtaaat gacgatcatt gatgtaacga tttgaaagtt cagcagcaag
ctttaatgct
3181 tctggatcgt attttacatt gtgatacttc tcatatcttg gagctaagcc
tttcaaaatt
3241 tgaacagcat cttcaatcgt tggttctggt acttgaactg gttggaatct
tctagctaaa
3301 gctggatcct tttcgatttg ttggtactca ctagtagtag ttgcaccaat
cacacgaatt
3361 tcaccacttg ctaatgctgg tttcaaaata ttagcagcat ccccagaatt
gttttcggag
3421 tcagtagaac ctgccccaac aatattatgt aattcatcga taaagagaat
aacattcttg
3481 tcacccttag ctttatcaat aatcttcttt aatttttctt caaaactacc
acgaagagaa
3541 gtacctgcaa ctaactcatt aatgttcaat tcaataatgt gggcgttctt
aagtttttca
3601 ggaacgtctc catcaacgat tctttgagct aagccttcaa ctactgaagt
tttaccaaca
3661 ccagcaggtc cagttaaaac aggattgttc ttcttacgtc tacttaaaat
ttcaataact
3721 tggtcaataa cagctgtacg tccaatgaca ggatcatatt taccctttttt
agcttgttca
3781 gttaaatcaa caccaattgg cttttccttt tgttgttggc cattttgtgc
tccttgttga
3841 ggtgcttgac cagcactata tctcattggt tgttgaccat tggtcattct
tccatcattg
3901 aaaaagtctc tgtttaattc attaaacaaa tcatcaaaat tgtagtttcc
attgtcaaag
3961 tatgccatgc tttacacact cctcatattc tcaaaaattt tagcacttgt
aactaatgag
4021 tgctaactac tcacattttt tattataaca ggttatccac agaaagccaa
atattatatc
4081 atgtttttttc cacagattat tttaatttgt gaacaaaaga agcacaatac
gctattcaaa
4141 gtgcattgtg cttaaataat ttgtgaatta atatgtcaga tttgtttcta
aattatttgt
4201 ttttattatt tttccaatct tttaattatt taacttttgg tcttgctttt
ttcgacttcc
4261 tcttggtctt tgaccatttc cttgcgtgtt atctccaata gtgctttcaa
gcaagtattg
4321 ttcaattagc tgaactactt gtttattctg cggcaatgat gagtgctgag
catcagctcc

4381 agtaacagtc atcgtagtat aatgttttac ttgattttgg aaaatatatt
ttcctgcagc
4441 tacactactt tctggaacca acccatcaga ttcataattc tgtccgcctg
atacagaata
4501 tacatctaag ttctgtggta ttttctttcg attcttaata aaatcattta
acatttcagt
4561 cttatgatca atattacttt cattaaagtt atacggagta cctaaggtca
ttaatttctt
4621 gatccgaatt tcatcactat aatcgtcata ataattttct agccaataag
tccaaatcaa
4681 tcccccatta gaatgtccaa aggccttaaa attattaaat ttgtactgct
gactaagttg
4741 cgcaaatgct tcatcaaacc aaccggcttg tttttgata ttagcatagc
catcatgatt
4801 gttttcaaaa cctacaacaa taaaaggctc attatcatta cggttgattt
ttccactata
4861 tttcagcgtt ccatcctttt ttacttgaat tttcaataaa ctatgctttt
taaaagtatc
4921 tttattcagt aaattaacta attcattaaa tcgttcagtc gtagcactag
agcccggaat
4981 cataatgact ggtgacattt gggattttct tctttcagct aaatcatgat
tggcactttt
5041 gatccagaaa aaagtaggaa tggacaaaac tattaaaaaa ataaatgttc
ctagtaaaag
5101 taggcggttt tttgacaccc ttttttatt taacatgttt ataccccgcc
tccttatcat
5161 ttaacaaatc aatttcatcc tggacatcct tagacatttt aatgatcggt
aaatataaca
5221 aaatatccaa agcaaagagt aacatcgaaa atactaaagc ttgccacgtt
ccatttgtag
5281 caataaatgc tactaagggg cctggtgtgc cagataagac attataagcc
gaagctggaa
5341 ccaaatggac tgcaatcatg ctagcagcta aaagcatatt cataactggc
aaaattacat
5401 atggaaataa aaatagtgga ttaagcataa taggtatacc tagaaaagca
ccctgattag
5461 aaccgaataa tgtcggtata aagctccagc gtgccactct aacaatgctg
cttttttaa
5521 taaaaattaa aataccaatt aataaaacta aagttaaacc gctaccgcca
aattttccat
5581 aagattgata taaagaatta ccaatataag gattaggtac attccagctt
gaaccatgag
5641 ttaaagcata gttcagatta gcaacattag tcgcactatc gctacctgcc
gtcaaagatg
5701 ctaaaggttg accaattcct agccaattaa gtagaagtgc caagagcaat
aaaggaataa
5761 atactcctaa gtttaaatta ttctgacctt gattttggaa aaattgataa
aaattagttg
5821 ctactacacg cacatgcacc aaactagtta aaattccaac tattaagcca
attacccaag
5881 ttgcaagcat tggacggaaa gaatcaaagg ctctttgctt aatttgctgt
gcgctctctg
5941 tatgccgatg gtgatactca actcctaaaa atctaaaaat aagcccagta
ccaaaaccta

6001 ttaaaagggc aaataaaaag ctattaatat ttaaaaaacg ccaattaaaa gaaagctgaa
6061 aatctctcgt gtccctaaaa cgatacgcac acaaaagcaa cgctaacatt cctgaaactc
6121 cagccatttg agcatctttt tgataaagac gagcagtata ctgagctgaa aaataggcgg
6181 taaacaaacc aaaagtgcca aatattacac tggtaatgcc ttgacaggaa taccatgccg
6241 cattaaataa atggtcaggc aaccatttat caaaatagaa aatatttgat atcaaacttg
6301 taggtgaaaa aacactctgc cataaaaatt ggaaaatact tccgacaacg gccaaaggca
6361 ttagcatcat caaagttcga tctgctactc taaaaaacgc tagtctccga agtcgaacga
6421 tagttttaaa aattattttc tccatatttc tctaatcctt agttctttaa gtctaacctt
6481 tagtttaaat taaatataaa aagaaaccta tcatttttca ttaaaatatc gcaaaaaata
6541 caaaattcgg gaatcattga ttcgataaag aacatgatat aatgataaag aagataacaa
6601 gggagaaaaa ttatggctaa acgaagtttt tctattaatg atgaaactga ccgtctactc
6661 aattactact taaatcgttc cgaaaaggat tgggacgcag taattaatga agcaattaaa
6721 tattatatct gcgatcattt aacacaaaag caagttcatg aagcaataaa gcatacagga
6781 aaagatgctt ttccagcaga tgaagttctt cgtaatttta atagcagctg gatgaacggc
6841 gatgcttaat atggattgca taaaagacta tcttaggtca aaatgaaagt agcattatta
6901 aaaagaagct gacaaacgtc agcttctttt tatacgataa atcttaattt aaaaggagga
6961 acacccatgt taaaaaagca tgcgctagac cctattaagt taatttggct atttgctggt
7021 tctttaatca tcaataccgg cgttagtttt atttggccac taactacaat ttatattcac
7081 aattatcttc atgaaacgct tactatagct ggtattgtat tatttattaa ctcagctttt
7141 acaatggtag gaaatgcact aggtggcttt ttatttgata agtggcatcc atatcaaacg
7201 cttttaactg gtgtaagcat ttctacacta tccacttttt tattagtcct ctttcatggc
7261 tggccagcat atccaatctt gttaattact ctaggcttag ggaatgggat cgtagttact
7321 ggtctaaatt ctatcgctac tctaattagg agtcgaaatg cttcttatgt ttttaatgtt
7381 ttatatttta cacaaaacct aggactagta tttggctcgt taatggttgg atttatttta
7441 ccctttggta ttacttatat ttttctactt gcttttatta tgtttgcatt cttaagtatc
7501 gtggtttttc ttgaatatcg cggtttaaat caggctcatg ctgcaaaagg taaaaaagta
7561 gctgaatcag aatatcaaga aaaaattccc tatggtgcaa aaaaagctat tttctctatc

7621 ttaatttgtg ttctagcagc gtggattgca tatgagcaat ggaactcgaa catttcttcc
7681 tacatgttag gcttacatat gagtgtgcgt ttatacagct tgctttggac tttaaatgct
7741 gttttaatcg tactcattca acctctttta acttacttcg acgattggct aactcagcac
7801 ttacacggtc gtttatatat tggctttagt ctatttggac tagctttctt gttgctaatc
7861 ggagcaaatc actatttcag ctttgtctta gctatggctg tccttacttg cggagaaatt
7921 ttagcctttc ctgctgtctc cacttttgtt aatgatcggg caagtaataa agataaggga
7981 aaatatcaag gtattgtcca atcagttact tcagctggtc gtgctttggg accactaatt
8041 ggtgccttag taattgataa tttttcttac ttagttttat ttatcttttg taccgttcta
8101 gttttaattt ccgtccttct atttgcaatg attaatacct ataacaagaa aaagatcgct
8161 aaatagcgat cttttatttt atctaatttc ataagaaaag cctaattcca ccaacatcgg
8221 aatcaggctc acacagtgtt aggtaccgtt attctgacca aataaaaagc gacgttttcg
8281 ctaccgtaga aaacgcactt tgttacaacg gtacaggtaa atcttagcac agcaatctta
8341 cttacacaac cagttttagc gatgcttaaa tggcaaaaaa gagatggaaa acatgtttcc
8401 atctctatga agtagccttt atcaaatttt ccataagatc attacacatc tgtcttactt
8461 taaaataatc cttaaccgga taattagact gaatcactaa tacatccctt gtatcattag
8521 aaattcgata gtatgttgtg taaccatctc cagaaccatt tgaagaatga aaatcaggga
8581 aattgtaaaa accagcatta tacttagcag gatcagaagg agtatagact ttttgtactg
8641 aagaaggttt gattatttct ccatttaaaa tagcactagt agctcgatac aggtctttat
8701 tggacattac caagcatcct gctcctaagt ccccatgtac cttatcagct gagataaagt
8761 gcgggactaa gtatccctga gagctctttg tataagggat tgcaacctta atttgcttat
8821 tcttaggaat atcccaataa aactcagttt cagacagacc caattttta atataagtat
8881 ctttcacaag gttttcataa gaacgatgag ttactttttc caaaatacct gataacaaaa
8941 tgtaattaat tgacgaatat acacgttgat tatagtgccg tttatcaaaa attacattgt
9001 gcttgatgtc atacttaatc cctgcttggt tgtttttgaa gacaggagtt ccataatttg
9061 actcatcttt tccttgtaaa ccagcctcca tagttagaag ctgctcaatc gttatttgat
9121 tagcatgcgg aacttgagga taaaatttcg ataatttgtc actaagcttt agtttctttc
9181 gctcaacctg tttcataact aaagtaccag taaaaacttt attgactgaa ttaatgaaaa

9241 aagcagtctt cggcgtatta cgcacttttt tactgatatt agcatagcca gttgactcat
9301 taagaattac tttattattc ttaatcgcca agacgttacc aataattcct gcctgtgcta
9361 gttttttttt atatctgtcg ctaccttact tttagtattt ggcattttta cgtcagattt
9421 attaccccag gctttagtga agtagtaact atcaagtttg taattaaccg gatggttctt
9481 ttcttccata aatggcttaa catattgatc tacttttaac caaccgttcc agccaaggtg
9541 aatcgtatct tccatgaaga acttctttcc accatctttt gataaatcgg cgatattgtt
9601 aaagccctga gaagtaagtt gtttagtaat tttagcaacg cactcttgat acattgtttg
9661 agataagccg gtgtacttag cccatttttc attaacaggt ggaataataa acagaacatt
9721 cacatgttca tgagctaact catttaatac taattcaaag tcagcatatt ctggcgactt
9781 agtgtagtca aagcttcgct gggaatcttt taacttcttt aagtgcttat tatttagttt
9841 cttatcaaag aaaccattag aaataccgaa tttattgttc gtagtacctt gcttaccttg
9901 ttcattagcg aattgacgca aattatcata tgcgtaagta cctggtaatt taccagcttc
9961 tttttgaatc ttagggatat tgttagtcat ccctatagaa gaaaagaagt tatcctcatt
10021 agctagcatt cgacgtctag ccttcaagta taaggtttga tacttagaca gtttttgtcc
10081 tgaagctaat gtcattaagg catacttcgt agtttcacta gcagtgccag caggcatatc
10141 taataaacgt cttgcagcat agcgcgtagc aacactatct ttagcaccta aaatccagtc
10201 aacagcttga agctgagaaa agtacatgcc aaaagcagct ggattttgtc cctgtttagt
10261 aaaccactgc ggagaaataa taaatacagc cttcttatta gaaagttgat ttactgtttc
10321 ttgcatagtg aaaaagtggg ttaatgattg acttcccggt cctcccaata agaaaggtcg
10381 ataatttctc ttatatttat aagctaaaac acttgggtgc aaagcatcca ttcttgataa
10441 ttcactagag ccataaaatg gaacatagtt tttttcaaaa gcggcttgct taattgattg
10501 tcccttaaaa acagtctgag attgtgaaac tgaagcttta aataaagcat tatctgactt
10561 ttcaccattt tgccatggaa tcaaaaataa gatcactagc aaaatgaaag cacaaagaac
10621 tgggccaaaa atctgccaca gtttgcgttt attactcatt tcttaagctt tctacctttg
10681 caatgatttt atttggagtg tcccaatctt cacggtgaaa ttctgaaact ggtgcagtaa
10741 caccacattt atcttgtaat tcaagtaaca tttgaacagt tgccatggaa tctaacaaac
10801 cattttcaaa aatgttttca tccatttgat cacttaaatc ttcaccagtt aattcattta

10861 aaatatctaa aacagcttgc tttgtatcca taattaagaa aatcctttca aaaataataa
10921 attacttcca gccaaaccat aatgtactca agaaacctga gaaaattagg aaactgaaac
10981 aaacaacatt aaatgttaag aagatcgcaa accacttagt aaacttatta tgtggaatct
11041 gcttacggtg cttgcgctta aatcttagcc aaatatcgtt gatgataatt gccaaagcat
11101 ggtaaatacc atatacgata tagtaccacg ttaagccatg ccaaaagccc ataattaaga
11161 aatctactaa atatgcgagt tgcgataatc taattctatt ctttaccaat ttatgcttca
11221 ttgcaaagaa tgtaaaacgc ataaagataa agtcacggaa ccagaaagat agagtcatat
11281 gccatctatt ccagaaatct ttaatatttt gagaaataaa tggtttatta aagttcatcg
11341 gagttctaat tcccatgaaa tatgaagtac ctacagcaaa taaagaatat cctgcaaagt
11401 caaagaagag atacatactg tagcaataca tataaccaag aagagcccaa gataaacgta
11461 gaccaccatt ggcttgacca taaaatagtg catctctagc taattgcagc aaccacaaag
11521 taccaaagac ataaccaata ataaatttat acaagaaacc ttggaaaata tatcttgtcc
11581 caaattttag gttttcaata tatttgtctc tctctggcgc cttatcatag tcttccttaa
11641 atctccgata acgatcaata ggacctgagg aaatagttgg gaagaagagg aggaatcttg
11701 catacgtaat tggatcaact tttttgatag ttttatcgcg aagttccata ataacttgca
11761 ctgtcttaaa agttaaatat gaaattccga agaatccaat taaatcagca gtcttattgt
11821 gtgctaatgg tgcgatcttt accgctgcta aaggcaaaat tgctaagata acggctaaac
11881 aaaaaacaga ggtcttattc tttccagaat taacatactt ttgataagca aaagtaatta
11941 aaaattggaa aataatatat ccaatcagtt ggactccctg tatccagtcc ttaccattaa
12001 acatcaaaaa caagaatagt aatgaaacga aagcttcata agtcttaaag cgccgaccat
12061 gatataaacc aataattaat ggcaaaagcg caataaaaag aatgacgaag taacgcggat
12121 tactatatgg ctgaatgttg atcacgcgtt atttacctcc ttaattactt gtttaatatc
12181 taccttaccg ttttgagaaa ttggcatttc atcacgatag ataaagcgtt gaggaagcat
12241 atatggcata atatcttgac ttaaagactc acgtagcttt ttagtcaatt cagcatcatc
12301 gtacttttct cgcactccat cagctaattc aactacagca actagttgtt gaaccttgtg
12361 gtctttattg tacttaggag caacaacgcc ataacgcact aatgggttct tggcgagata
12421 gaagttaatt tcttctagtt caatacgata gccattgaac ttaacttgaa aatctattct

12481 tccgcgataa aagagcatat cgccatctaa aaatcctaaa tctccagtac ggtagcttga
12541 atgctctccg tctttaaaga aagcagcctt agtcttttct gggttattta aatagccttt
12601 tgaaacacta ggtccctcaa taataacttc gccttgatcc ttttcttcac cttttgaagt
12661 atcaattgag attttagtat cttctttaga aacaccaatt ggtaagcgat catacttgct
12721 caaaatatca tcagtaattt ctacttgagt aacagcaaca gttgtttctg tagggccgta
12781 agtgttgaaa atatgactat ctgggaattt catcttaagc atttttgctg ttttatgact
12841 taattcttca ccgcagaata aaaaatgagt taattctggg tggtgcttcg cgtcaaaagt
12901 tggatccaaa aagctcatct ctacaaatga tggtgttgaa acccagacat taaattttaa
12961 ttgtggcaaa gtcttaaata actgacctaa gttctcagtt acatcatgag gtaacactac
13021 taacttgcca cctgcagtta aagcaggata taagctcatg acagataagt caaaagagta
13081 tggagcttgt gctaagaaac taggttgatc tggaagtgaa aagtcattta actcccaatt
13141 tacaaagctc agcaagttat catggctaat ttgcacaccc ttaggtttac cggtagtacc
13201 tgaagtaaag ataatataga agttatcatc cccacttaca aatttacttt gctcaatctc
13261 tgcgtgattg tcagaaactt cctcaggagt taaaactttg atattatcca tttcaattgg
13321 aagcttatca attgcaatta ccaaaggtga ttcagaaact tcctgaatca ttaataaacg
13381 ttcactatta gaataactag caattggaat ataagcatgt cccgctttta cagcaccaag
13441 aaaactggcg atcatttcaa aggtttgacc accccaaatc attactgggg ctttttcggg
13501 aagatctaat tcgctaatct tagctgcata tgcatctgag cgagctttca agtctccata
13561 agtattagtt tcgccaagat aatcataggc gatccgcttt ggatcttcag tagcaatttg
13621 atcgattgtc ttgattacat ctttaatcat ttaatgcgac cctttcagtt ctagaaatct
13681 ttataaataa attttgcagc cccaatgccg ctatatgagt acaagtaaac tagcactaca
13741 agcactgcaa agtaaaagag agttttggca atgaataggc cgatctcttt tttctttgta
13801 ttattttgct tatctttatt ttctgccatg cttacacctc gtaatctttt ttctaaattt
13861 tacaacattg ttataaaaca ggttaacatt tttactattt cttagaaaaa tagcatgaac
13921 agaagtaatt ataccaaaaa tagccttagt tatggatagt tcactattat tgattacgtt
13981 ttctttcaat aaaataacca attcctaaga aaatagtcgg taaaatcgaa acaaacacta
14041 acactactaa gattaccttt aaaagccaat caggattttc ccacttttta tcattggcag

14101 ttgctaaagc ataggccgag aataaaacaa ttgtaataag gtttattaaa aacaagctgc
14161 tatgcgaata ttgatacagc attaaaacac tacttcctga tatcaagcca ctccagcctg
14221 ctaatcgtac ccaaaaagct atcttcttaa ccattttaac cccctaataa ggcattaata
14281 tttgctgata aaattcgatc gacaatcata atttgatcac gtgcataagc ctttgcacca
14341 gattcaaata acattaatag ctgaccattt tgaacataaa tttgttctaa atatggcggc
14401 ataactacaa ctttttctgc atttccttca tctagatagt ttaaagctga aattgggaaa
14461 aagtataact tagaatcctt gcttccataa gactgactca aaataatcca attttgataa
14521 aaggcaattc cttgaatctg tcgatccatt gaggcacttc cagaacctaa ggcccaagat
14581 acttgaccag ttactgcctt aatttggtca ctagtgattg ttcccttaaa gttccctgaa
14641 cgcgcaatcg gataactagc aatttgtcct tgaccatctg tattaaaaaa gccaacaaac
14701 aattgattat cataataagt tacacaagat gctctttcaa tcgttggcaa agcaatgaca
14761 ttgtcatatt gaataggttg ctttttctta ttataactgt atttctctag tttctttaat
14821 gaaaaagaca ttagggccga agaatcatct tgactacctg ttacccaaat atttttagca
14881 gttggatcat aagcaatacc acctaaatgc ggagatccag aaacctgaat agtcttcaaa
14941 tatttaccag ttttttatc taaacagtaa ataactgagc gatgattatg tgttgaatcg
15001 taagctgtaa ttaataagta cttccccgct acagttagtc cttgaggagt catcgaatct
15061 gtatctgtaa ttttcttttt attgaaatca tagcttggcg ttacaacttg tcctgggatt
15121 acgacactgt taccaatttt actagcacgt ggcgtttcat atgctgcact atagacatca
15181 ggatactttt cattcaataa cttcttaaag taagccaaat tatgatgacc tgggctaatg
15241 cttgtgccat cctttgcctt ttctacatca ggtctagaag taaacgttgc ttttgcctgt
15301 tctcgctttt gatattgctg atagccatag taaccgccat aactagcccc agcaataatt
15361 gccaaaatta agattagttt aaacagaatc tttaaaaatc ttttcattag ctttccctac
15421 ctagtaaaca acttccttaa ttcgatatta tacctgaaca gtccatttac tatatgaaaa
15481 taaaaaatta agcaagtata ttatatttgg ttctagtttg tactataata tattaattgt
15541 agtagtggat taataataat tcactgctat tagcattaac atccaacttt ttattttcat
15601 tcttactcct ccaagtagat tgaataataa taagatcagc ttaaaggctg attatctatc
15661 ttttgaagag acctacgcac ttcccagcgt aggtttcttt ctttttagcg cattaaaaaa

15721 gagaatcctg attatggatt ctcttttctt gtactgatga ttattaagga aatgggatga
15781 aattaagtaa gttgccgtct ttagtatatc tttgataggt ggcatccctt aatttattcc
15841 aaaccttact ttgaaaagta ttcaaattta agtcattaaa cgccattaaa ttttcaacta
15901 aactaacaac atcaaataat tgcgatgtta aagcgacagc gtgatatttc cctgttttca
15961 aattagcaat tgtttcattt atctgatcaa ctatttgaac aacgttagga tcattttcag
16021 ccgtcaaact taatcgaaat gcttcaagca agctgatata ttcttgatac tccattttga
16081 caataccaac tttctaatct agtaaaaaat attatgtttt gatcatgata aaagcggttt
16141 tatttatata taaattatac cataccaatt ttcaaaataa tgattttcca ctggcaatat
16201 tatgccaaga tacatagcat atttagcaat gaatcgactt aagctttctt cttaataaaa
16261 gacataccaa tcccattttt attaaaaata attgcataaa gtttccttca gatgaaaaaa
16321 tgatttataa agcacaatta ttactatagc tggggtgcaa gtttataatt tatcccagct
16381 tttttaacaa atttaacttt tttaataaaa aacgtcacaa tttgttcaca ttttccctct
16441 aatatataaa ccatagcaac gggggaaaac aaaaacccca tcgctattag cattaacatc
16501 caacttttta ttttcattct tactcctcca agtagattga atacatttat cagtctttga
16561 ctgatcctat acttatatat ctgaaatccg cgtctttccc agcgcggatt tttttgtggg
16621 cttatttcca agactttcct ataatagaag tgaaagggaa tacaaatttt agaaattaat
16681 tatttaagca taggaaggat tattatgttc tcaccatcaa ttcaatcctt gattgaagaa
16741 aaagcaggct catttatcat ccctgcctct agaattgcat tcgtagaaga tgaaaatcca
16801 ctctaccatg catttttaat tcttactaaa atgaaatact caaaaattcc agttttagat
16861 aagaatcaaa aagtagttgg tttaattagc ttatcaatga ttacagataa gatgctaacg
16921 cctgataata tcgttatcga acctttatca gacttgaaag tcaaagacgt aatgcaaaca
16981 gactttgaaa cgatcaactt tgcccatact aatcttgaaa cgcagctgca tcttttagtc
17041 gataatcctt ttattccagt tgtaaatgac aacaatattt ttcaaggatt acttacaaga
17101 agagaatgga ttaaggcgtt taattacgta actcattcaa ttgatgataa atatgacatt
17161 actaaaaaga gcgatccgag cgaagcaagc taaattgctt aaatttaatc tattaagtgg
17221 tatagtaata atgttttagt attattgtta atttagttgg aggtaaagct aatgagacat
17281 caatggaaat ttttcacaat tctcggaatt ggtgtcattg cagcaatttt agactttctg

17341 tgtggagctc ctaaaattgg tacatggcca atctcaggta tcttaattga
tatcttcgga
17401 atttttatgg caattacaat gctccgtgaa atgattcata cgcttgaatc
aggacgctgg
17461 ggtgtagata tcttagcaat tattgccgtg gtttcaacaa tgattgttgg
cgactattgg
17521 gcagcttgga tgattttaat catgcttacc ggtggtgact cacttgaaga
ttacgctacc
17581 agtcaagcag acaaggaact acgttcttta ttacaaaact caccaagaat
tgctgataag
17641 ttagtcaatg gcaagattga agaagttaaa gttgacgatt taaagattgg
cgacattgtc
17701 ttaattaagc caggtagtca agttcctgtt gatggagaaa tcattaaagg
taattctagt
17761 tttgaccaat cttcattaac tggtgaatca gttccagtag ataaaaaagt
cggcgatgac
17821 ttaatgtcag gatcaattaa tggggatgca gcagtagaaa tgaaagttac
taaagctgct
17881 aaagattctg aatatcaatc aatcgttgcc ttagttaagt cttcagaagc
aaaacctgct
17941 aaattcgtta aaatggctga ccgctacgct gtaccattta ccattatttc
tttaattatt
18001 ggtattgcag ctatgattac ttctgctgta actaatccag cactaggttg
gcaaggtcac
18061 ttcttacgtt ttgcacaagt tatggttgtt gcttcgcctt gtccattgtt
aattgctgcg
18121 ccagttgcaa tggtttcagg catgagttca atgtcgcgta gccacattat
tgtaaagtct
18181 ggtactacgc ttgaaaagtt atctcgtacc ctaacttttg cctttgataa
aaccggtact
18241 ttaaccgaaa accaactagt tattgaccag gtcgttttag caaaagatag
ctcaatttca
18301 aaagaagaat tacaaggttt ggcagctagc gttgaacaac aatcaagtca
cgttatcgca
18361 acttcattag taaaaagcac agataaaaac ttaattaaac cagttaccaa
cttaaaagag
18421 gctactgcta agggtgttag cggtgaagta gacggtaaac tagtaaaagt
tggtaaactt
18481 gcctacgttg atcctgatca agaaaaaatc acagttcaat caacagctgt
ctttgtttca
18541 attgacaata aatttgctgg atatattact ttccaagacc aaatcaaaaa
aaacacccca
18601 gaaacaatcg ctcgtcttcg ccgtcaaggt atcaagcaaa ttatgatgct
gactggtgac
18661 cgtagatctg tagccgacaa agttgctaca gaagccggaa ttagagaaag
cgaagttcac
18721 gctgatttgc ttcctgctca aaagattcag gctattagag atgttaaacc
taatctcaga
18781 ccagttgcca tggttggaga tggtgttaat gacgcaccta gtttaatggc
tgctgatgtt
18841 ggtatcgcta tgggtgctaa aggtgcaacc gctgctagcg aaagtgccga
tgcagttatt
18901 atggttaatg atatttctaa agttaacgat gctgtcgcaa tttctaaaca
tacgatgaaa

18961 gttgcccacg ttgatattat taccgcaatt tgtatcgtta tcttaattga attgattgcc
19021 tttaccggta tcattccagc attctggggt gcaattttac aagaagtagt cgacttaatt
19081 actatcttgc ttgctcttct tgctaaaact aagccaacca atcctaaaca aacaggatta
19141 gagaaataaa aaaactagtt tgcaaccaag aaagttttta gctaaactaa aatttagtta
19201 aatatttttc attagtggtt gggttttatt taaaactttc aagatttgct tatttgggtc
19261 aataagcgct tgaaagtttt ttattttgcc acaaagttat agaaagttga aagaaatgca
19321 ctttttagga gaattaattt taattctgct ggctactaca ttattaggac aactttttc
19381 tcgtcttaat atgccagcag ttattggtca attattatca ggaatcttat taggaccagc
19441 cattttaggt ctagttaagc caaacgaaat catttcgctc ttttcagagt ttggtgtaat
19501 tctgttgatg ttcttagctg ggctagaaag taacttagaa ttattgaaga aatatttcaa
19561 tctaagtttc actgttgctg gaattggcgt tatcctacca gttttcttca tgggaattgc
19621 aagctactta tttggaatga acttccttga agccctattt atcggaattg tatttgcggc
19681 tactagtgtt tcaatttcag ttgtcgtgtt gcaagaagct aatcaaattc atacccgagc
19741 tggtacagct attctaggag cagctgtggt tgatgatatc ttagcagtaa ttgtacttag
19801 tttatttact acctttagtc atgaaggcgg taaaagtggt ttaactaata atttctttct
19861 taatttatta attgaagtaa tctattttgt tgtagtctgg attatcttca aattcatcgc
19921 accatatttt atgaaattag cagaaaaaat tgaagtcgat tatgctgtag ttattggatc
19981 acttgtactt gccttatcaa tggcttgggc agctgacttt gtaggtttaa gtgcggttgt
20041 aggggccttt tttggtggtc ttgcaatcag acaaactccg caatataaag aagttcaatc
20101 ttcagttagt gcaattggat attcaatttt tattccagta ttttttgtaa atattggtct
20161 ttcaatgaca tttgctagtt ttattaaaga tatttggttc attattgtaa tgacaatttt
20221 agctgtcgta tctaagttct gggctggtaa atattcaagt gaattatttg gctttaccaa
20281 aaatgaggga aacatcgttg gtgctgggat ggtatcacgt ggtgaagtag ccttgattgt
20341 tgctcaaatc ggaattacgc accacctctt cccagaggat atttattcaa gtttaatcct
20401 ggtaattatt atcaccactg tcctctcacc atttatcttg aactactaca ttaaacgtgg
20461 cataaaaaat cactaaaata aaaacgaatt agccgttgat ttaatcaaca aatagctaat
20521 tcgttttttt atattctaat ttactgaaat actacccaaa gaagtgtctg cttctaaaac

20581 attcctagag tcactattct tttcaaagga atcaccatgt tttttaccac taattgtaat
20641 tttcccaaga gaagtatcta gatcataacc agtcgcatta ctgctctcaa ctctaatatt
20701 tcctcttgaa gtatcaaaac taattccatc tttagtcttc aaatctttaa tatcgatatt
20761 gccgtttgat aaatcaatat cgcctatttt cactgctaaa taatcagcac taaaattacc
20821 atttgaatta tcaacattta aactttctaa ttgttctttc ggcatttcaa tgacgatttt
20881 agaatttaat tgcttaaagt taaataaacg aatgtgggta atttgattct taaaactaat
20941 ttttaattta ctatctttaa cctcaacttt agggacattt ttttctaatc cagagtaact
21001 aacatgatat ttatttcctg cttttaaaac tacatctcct gttggaatat ctatatctac
21061 tttagtaaat ttagaaagtg aataatcctg attaacaact ttttgattag ctacattaaa
21121 cattgtcaat cctccaacta atataattac aataatgcca atactaaata atgttttctt
21181 ccaattcatc taaatatgtt ccttcaagtc aagtatttcg tcatattgct tcaattcatc
21241 tttctcatag tggtgaatca cttcaataaa agttaacttt gaagctaata aatatttatg
21301 aattatatca gaagttttct tatcaaggga agcattaatt tcatcaagta ataacactgg
21361 acgcttcatt aaaattgctc ttgctaactc aatccgtgct agttgtccac ctgataattt
21421 atcggctgct tctcctaatt gatagtcaaa gccttttttct ttaactaatt cgctcaagtc
21481 caagttatcg caaacttcct ctaccttttc ccgggaaatg tttttttccta aagtcaaatt
21541 aaacaataga ctatccgcaa aaataaccgg tgcttggctt gcatatgaga ataagtctgg
21601 aaatgtacca gcttttactc ttttatcatt taatctaatc gcctctgctt ttccatattt
21661 tccatatagt aagaactgca ataaagtcga ttttcctgct ccagatggtc caatgacagc
21721 aattttctta cctaggttga catttaagtc aatcccagta gctaattgct taccatctct
21781 ctttaagatc aaatcttcta cttgtaaatc ctgtaaactt acaacttttc tattttctgc
21841 atctttttct ttatctaaaa agtcattaac cttctcaaca atcttcttag tagtagacaa
21901 tttacttcta tcatctaaga taaataaaat tggatttata aatgaattgg ctaattgcac
21961 aattgcaaat aaagctccta aacttgtttg tccttttaca actaaataaa ttccaaatag
22021 aaatggcatc aaaaatgtaa caatatttcc aacaaaatta atccatgagt tcgtatcaag
22081 gtttaagaga ttcatattac tcaatgcttg ttctagctta gaaattgttt gtttattttt
22141 agcaaccgcc tgattttgac catcatataa atgtagcgtt tcaacacctg ctaagaagtt

22201 tttagtctga ttaacatact tactattagc actagtccac ttttcagaag attcttgaat
22261 aggcttttga aaaacattag aaacaaccat tggaataaaa gatccaacta ggaatagtaa
22321 tgtaagtagc caattaactg ctaaagcata gcctagtgct aatactaacg aaaaaatctg
22381 cataataatt tcaatttgag cattaaaacg attagtttca agtagtttaa aatcattagt
22441 taaaaatcct aaactatcat tattttcgtc ttttgactct tctagcattc cacccaaaat
22501 atgtgttctt aaataagtgt tagtttcctt aattgtatta gttttcaatc tgttaaatac
22561 taggcttgca attaaggtaa caactaatgc tgcaatgata ataatcaaaa attgtgtaat
22621 tttgccccag ttcttattag tagcaatatt agttagtatt tgcacaacat tggccataat
22681 aatattttga aaactagcaa ttaaaccaaa gataatcatc aatatgaatc taaatttatt
22741 tgaatactta aacagcatct tcttcatccc ctcatcgatt taaacttgtt gataagagta
22801 tatgatgaga ttatacttaa aaataaatct cttgcagatc tgcaagttag tttttggaga
22861 atgaaaaaat gtatggtcat gaattcaaag aattacgctt agaacaaggt attacgcaaa
22921 aagaggcttg cagcggaatc tgttctgaat caaaactatc acgctgggaa aatgatcaag
22981 tcgaggttga atttagtacg gcaatgaaat tactaaatag aattcacatt actagtcatg
23041 aatttatggg ttggtccaaa ttttcaccac gacccgaaat agatcctgag ctttctgaag
23101 ctactgaaaa agaaaatatt ccgtttctca aacgagttac acaaaaacaa ctaactaagt
23161 atcataaaaa tcataataag tttgacttat ttatggctgc taatctatgt aatcaacttt
23221 ttatcatcga acataaaaat tacttgcctc ctattgacca aaaaaagctt tttgcaattt
23281 tttcaaaagt caatttatgg agtcaatact atatttcagc gtttggtgca tcaatttttc
23341 tattcaatcc taagcaaatt tatggtagtt caatgcaaat cattcgtaat attgaccgtc
23401 ttaaagaagc tgaaactagt tttaacttag aaatcattat gggaagttta tcagatggca
23461 ttttacggct gatttcactt aatgaattgt cttatgctca gaaattagtc aaagaactta
23521 aaaaaatcga attaccacaa tatttaatgt tttttactct aaccttgact tatttgcaaa
23581 aggcaattga ttacatgaaa aatggtgatg acaaaccaat acttacatta atttcagaag
23641 ttttagattt agggtgcagt gtgcaagcca ccacctacct agatatgttt aagtatttaa
23701 aagctcagcg aaaaaaatac aatttttaac atagcgtcta cattcatttt ttaaacaatc
23761 ccaatatata gtcacgatca cttaacaggc ataataaaaa acgcagttta aaactgcgtt

23821 ctttgtacga tcaaatcata gcggcaacaa tccagactat attcgtagct cttccccata
23881 ctcaggcata cctacaagac aaaaactttt tattatagca tcgcaatatt gcaacgcaga
23941 cttatcttga ttctttttat attgaatgaa ataagtaaaa aaggtcatcg cacttttata
24001 aaagaaaaga tcaggattag ttggaacctt attagcaaaa tcactataaa aatcaatatt
24061 ctcaaattta ttttcttcga tagaaaggac taaaacatta atagctaaag aaagtagaga
24121 tttttaaca agcatagatt cacgcacttg atacttttca attatctttc taacaattgc
24181 ttcgttatca gccaaactat agaaacgcat agaattgcaa tacagcataa acttagtact
24241 attgaaattt ggaatttcaa aaattttctt cttaatctca tctctaattt tttgattagg
24301 tttatccgga tttagtaatt ctaaaagtcc atcagcaatc aaattttgaa tttctttatc
24361 tttttcagat aatttacttt ctgtaatcat taatttaacc ttttttagtt tatctttatc
24421 tacttgataa tatgcttctt ccattatctt tttttgaatt tgaatctgac tttgatagtt
24481 ttcataattt gggtctaaag tttcaataaa ttctatagca tcaatttgat tatatctaag
24541 cagattaatt aagttttccg ctgaaatttg atgtatattt ctttctactt ttccgtagta
24601 agatcgatca attatattac ccgcaaattc ttgcaaagtt ttattttggt taatacggta
24661 ttgcctaagc agatctccta ttgtcatttt taccttcacc cctaatcaaa aatttttatc
24721 actattttaa tgagatggac aaaaccaaac aagtacaaaa ttttggattc cgattaaaac
24781 gtatgtcgtt aataacgtag tcatcactga tgcagtacca taaggatcgt attaatcaag
24841 ccaattcctg aggctaaaat tttctgagca attgcattaa ctaaccattg ctgcatttta
24901 agtgaagcag tacttacaac tgcagccaat aaaaagtaaa acgataagat tataaaaaca
24961 acaaagaaaa aggccaaaca ccttacgtgc ttgaccttac ttaaaagagg ctaccttagc
25021 ctcttttcat gatcttaata aaaagtaatg atataaatac caagtttcaa ctataaacac
25081 aattactaga taaccatacc aatcgaaatt tgtatcatga ttaccaaatt gtgttaaaca
25141 tacaaaacta gaaacaataa ttccaattaa gcaataagca aatggaaaac catatttaat
25201 aatatgctgc ctaactagta agcatagaat gaatccaata atcgctaaac ttagaaacat
25261 ccaaaacata caataaatca cctacgcttt agtgatttgc gccttcaatt gcttcaatat
25321 cgcccttacc atcacgttca acaatagcgc taagagctgc agttaatcct ttaacaatgt
25381 catcaagcgc catggatgga gtattaggac ggtttacaac ttgctcaggt aagaatggaa

25441 tatgaataaa tcctgcctta atgttaggaa attctttatc tctcatatat tgaacttgat
25501 acataatatg attacataca taagtaccag cagtatttga aacgctagct ggtaagcctg
25561 catccctaat tgccttcgcc tctgccttaa ttggcaattg tgtaaagtag gcagtttctc
25621 catctttatg aattggttca ccaagtggtt ggtagccttc attatctgga attcgtccat
25681 cattgatatt aattgctact cgctctggtg ttaagccgaa gcgtccgcca gcttgaccaa
25741 catttaagac ataatcagga ttttcttttt ttattgcttc ttttacaact tcagctgact
25801 tattaaagac agttggaatt tctaacttaa caacttctgc acctttaatt tcatcaggca
25861 gtcttttaac tgcttcaatt gctggattaa ttttatctcc cccaaaggga tcaaaacctg
25921 ttactaaaat tttcataata ttatcctttc aagactaaaa tgccaaaaag tacatcaaac
25981 caatttgaac aatcaataat ataactgcga ttggggcctg ttgtttaata acgcccatct
26041 gatctttcat ttctaacagg gcaacgggta aggtgttaaa gttagcagcc attggcgtta
26101 ccaacgttcc gcagtatcct gatgtcattc caattgccgc aacaactact ggattggctc
26161 cttgagcaat tacaaatgga ataccaattg ctgctgtaat tacagcaaag gcagcaaagg
26221 cattacccat gatataagta aagagcgcca tcgcaataca gtataacgcc actcctaaga
26281 aatgatttcc agctggaaaa acatgactaa tcatattagc agttagctta ccaacaccgc
26341 aagtggtaaa gactactcca agcatagcta atagttgcgg taaaacgcca ggtgcaccaa
26401 cgcttcgtac cattctttgt gagtctttaa agacttgctt agtcggagcc ttagttaaaa
26461 taattgctaa gatcaatgaa acaacagcac caataccgat tccaacttga ccacccagcg
26521 gtgtaaactg cgcaatcaaa atagaagtta atgctaaaac tatacttggt aaaaatactt
26581 tagctcctaa acgcttcgca ccctcttgcg cggttttggg atctagctta ggcaagctac
26641 caacttgaat ttgcttaaac aaagctaaaa ttccaattac taaaaccatc gcacccgata
26701 caacagctgg aatccaatct ccaaaggcaa aaataattgc caagaagaac caaaaaaacgg
26761 cagttccaat tctcgcctta tttgccttat ctcttaaagt ttcaattcca gcaaccgcca
26821 tacataaacc aatcagggca taaaggatgg cgagtaaaaa gttaatcgta ttttgcattt
26881 tattgaccct cccatttctt catctgacgg tcaaagtaaa gattataagc aaagactaaa
26941 acaaaagtaa taatcgcaat tggaatcgta ttaagcacaa ttccaaccgg atcaacctta
27001 tagccaagcg acttcattgt tgaagaaatt aaaagcacac cacctgaagc cacaaacaag

27061 ttttgagcaa agaagttacc aaagttatca actgctgcac tacgaccctt aattaagtca
27121 atctctgact cagttaattt cttcttatct tggtcttcag cagcagcaat taccattggc
27181 gcaattaatg gacgaacgaa ttgaacttgt ccttgaagag aaattccaaa gacaccagct
27241 aattcacgaa ttaaaagata gatatttaaa atctttccag cagttaattt cttcaacttt
27301 ttaatcgcat taactgcata atctcttaac ccatgagatt caactagtcc aatcattggt
27361 aaagttaaga agaacaaact taccattcgg ttgtctacaa aactctttcc aattacttcc
27421 agcaacttaa caaaatccat tcctgagaat agtcccgtta caattgctgc gataactaca
27481 actgcggttg tgtcccactt taggacaaag ccgaccacaa ttattaatat tcctaacagc
27541 tttaaatatt ccatactacc ccttccttaa ataaaaaata gaaaaatact ataaaagtat
27601 tatacaaaat ataataaaaa aatacgaaga ttaacctgct cttagcatta agtaacttgc
27661 ttgacccccc catctggata tacttatgtt attagttaca agggatgatc accattgatg
27721 aatactatta atttttcttt taattctttt ggtacaacga tagttatacc aacaacaatt
27781 tttatcatcg ctttattcgt agggtatgat cctaagaaaa gcttgcttta tggtctttat
27841 gcaggaattg gtttgactgg tttttcatgg attatcaatc aatttactcc aatagttatc
27901 aaaattgttt ataaaatggt caacaacact ggaattaaaa gccccatcgt tgacataggc
27961 tgggaggctg gagcagttgc cagttttgga tcagctattg gtttaacatt ttttatattt
28021 ggactattgg tagaaataat cttatttgct acaagaatta caaaagtctt catgccatcc
28081 aacttatgga ataactttgt ttttatgctc tggggaagtc tggcattcta cgttactcat
28141 gattggtggc tttccttagg gctttcattc ttcatgcttc tctacacgct actttttgcc
28201 gaaattcaag cagaccgttg gtcaacttac tataaaagca aaaacattac tgtttgctcc
28261 gcacaaaata tagttcaaac aattcccgct attttacttg atccgctttg gaatcgacta
28321 ggttttaatc gaactactct cacaccaacc accttaaagc ataagctagg aatatttggt
28381 gaatctccaa ttctaggagc tgctttagga ctatttatca gcctaattgc aaatatcaaa
28441 gagctcaacc aagtcagtgc ttgggagcaa atctttcaat ttactattca attatcagct
28501 gtaattacca tttttcctct gattgcaaca gtctttaata tggcctttac tcctttggct
28561 gaacaaatag ataaaagtcg taagcaaaag caaaaatcca attctggcat tgaaataaat
28621 ccaattcatg ataaaaaaag atggtttctg gcagttgacg atagcgtagg atatggtgaa

28681 tcggctacta taatttcagg aatagtttta attccaatta tgcttatctt agctttcctt
28741 ttgcctggca acaaaacttt gccaatcgtt gatttggttt tacttccatt tatggtggaa
28801 tcaatcgtgg caataacaaa tggtaatatt ctaaaagtta tcgcaactag tatagtctgg
28861 tttagtttag gactttatac agctagctgg ttagggccaa tctatactca tgctattgct
28921 cattatggcg taacaattcc ctctggcatt attctagtca ctagctttag tttgatggct
28981 caacctttta atgcattgat ctttactgcc tttatttctc gcaatccttt ttggattagc
29041 ttatgtatta tcatttactt aatttcatta ttaattctta gacgataccg tccacaaatt
29101 tggacttact taaacaagat ggctgctaaa aatatccaga taaagcataa gtcatctttt
29161 caaaaataaa ataatttaaa cttactagca cactgatata cataattcta attattagcc
29221 ttctttttc ttcttttcta tactaagact ggatgcttga gatggaaaga aaagaggcaa
29281 actaatgcta aacaatattc atactttttt taatgccttc ggtgccacag tgattgttcc
29341 tgttatgatc tttatcattg ccttattttt aaaagtaaaa cctaaaaaag caatgatgtg
29401 cgcactctat gcaggagttg gcttaactgg attttcttgg atcattaatg agtttactcc
29461 tgtagtgaca aagattgtcc ggcaaatggt tgataatact ggaattaaat tgcctattgt
29521 cgatattggc tggcaggctg gttctcttgc aagttttgga tctccagttg gtttaacctt
29581 ttttgtattt ggactaattg cagaatttat tttgtttgct gttggcgtta ctaaagtttt
29641 tatgccctca aatctttgga acaattttgg tttcatgatt tggggtacat tagcttttta
29701 tgtaactcat aattggtgga tctcccttgg attatccttt ttcatgctgc tttatacctt
29761 attgttagct gaaatccaag cggaccgttg gtcaagatac tacaagattg aaaatacaac
29821 tgtttgtgct gcccaaaata tcgtccaaac agttccagct attctgcttg atccgttttg
29881 gaatatgctt ggcttcaata aggcaaatct aaccccagca gcctttaaaa acaaatttgg
29941 cgtcttcggt gagccaacca cattaggagc tatcctagga atagtaattg gtatcttagg
30001 caatcttaaa gatttagcta ccatcaaagc ttgggggcaa attttccaat ttgccattca
30061 attatcagct gtgatgacca tctttccttt agttactaat gtcttcagta aagcttttac
30121 tccgctggcc aagcagattg atcaaacaag aaagcaaaat gctaatcagc ggccagaagt
30181 agatccagtt catgataaaa agcgctggtt cttagcagtt gatgacggtg taggatatgg
30241 tgaatcagct acgattattt ctggcataat cttaatccca atcatggttg ttattgcttt

30301 tcttttacct ggaaacaaaa cgcttcctgt agttgattta atctcactac cgtttatggt
30361 tgaatcaatt gttgctatta ctgacggcaa tatcttaaaa gttattgcaa acggcgttat
30421 ctggtttagc ttaggtttat atgctgctag ttggcttggt tcaatttata cgggtgctat
30481 ttctcagtat ggcgctgcta tcccagccgg aattgtctta gtaactagtt ttaatttgat
30541 ggctcggcct cttaacgctt tagtctttgc cgcctttatt tcacaaaatc cgctctggat
30601 cagtttatgc atcattgttt atttagtcct gttatatatt ttaagaaaat atcgttcaca
30661 aatttggatc tacttaaaca agatggctgc taaaaatgtt catgcaaagg aagaccttaa
30721 atcatcccct aaggactgaa tttgttacaa taagaacaaa ggacctacaa aaggacgtga
30781 agataatgtc aaaatttagt tggaaaaatg tattaattgc aggtactgct gctggtgtta
30841 tttctggatt agttaagtta ggctgggaaa atattctccc tccaagaaca ccagagagaa
30901 ataaaaccaa tccaccacaa aaattacttg aacaaatggg tgtacccgct aaattaactc
30961 acgcaactta cacttattct ggtgaaaaat taccatgggt aagctatcta gttcactttg
31021 gatttctat ttcctttgca acagcctatg ctgccttgct cgaaaagaag gttaagtggc
31081 taactgttga tcaaggagtt ccatttggtt tagcagtttg gattgctttt cacttagtaa
31141 tcatgccatt aatgaagact gttccttctc ctaaagacca gccactagaa gaacatgtct
31201 cagaagcact aggccatatt gcttggatgt ggactaataa tgaaattgca gaagttgtct
31261 tagaacaatt aagacataat aaaaaggagc attagtgctc cttttctttt actataaaat
31321 tgagaggata ttatttagca tatgcgttaa gatcgaatat ctaatatcct ttgtttccac
31381 atatgtccat gcaaggatca ttcccattac ccagtagaga taaataaatt tactaaacat
31441 tggatcatga ccataagcaa aaacaaaacc acttgctaca acgccaaccc acttattaag
31501 gttgttatct gctgggaaga aagtattaaa gaatagcgct ctaaaaatga attcttcaca
31561 aattggagct actacaacaa gcaaaatatt aaacatcggc gatgctactt ttctaacctc
31621 gtcaagttct gcttggttct gagaaacagt atttccgcca atcaatctaa tataagctac
31681 ttgaaaaatt accagagcaa aaaaggcagc tactgcgata ataattctct tcacatccca
31741 gtgcggttta gcattaaaga accaatcatt ttcctgctta agctctcttt tataaatcca
31801 atacatccag taaatcaccg ctaccgttaa aacggctaga ataattgcaa ataataagtt
31861 cacctttttt tgtgcgtatc ctaattgcaa aactgtataa aacacaaagg caataataat

31921 tactgctaaa ttacctaaaa agcgtaaaaa agtcttcatt cgaacacttc ccctaattga
32041 tgcaatcaca actcatgcaa tctttacatc aatttcttaa ttttaccatg catcatagag
32101 taacaattac tactttattg atgtttatgt ttttattact atttttattt tcctggctaa
32161 ttgagccgcg tcgcttaatt aatggattaa tctttacagc atttgggctt acatttttga
32221 cttgggcagc aatattgatc ttttcacagc ataatgttat cttaacctct tcattttcat
32281 ttatagcctt ggctttttg tttgggattt ttttcctagt tacattctca tggattttct
32341 ttctatggaa tgcttatttt gtctggaaat atgaaaatca ctctcttcct aatcttctaa
32401 cattaattat tgggttattt ctagtagcat tatggactct atatcgttta ggcgtcttcc
32461 ataagctacc tggttggctt cattctttag ttgccggcgc agctttaatt gctttctatt
32521 tattgtttgt aatgtacaat ttcttactca acctagtctt atatcagatt gttccgcgac
32581 gctacaaaca agactactta atcgttttag gcgcaggtct tattgatggt aaaaaagttt
32641 cacgacttct tggctcaaga attgaccgtg caattgcctt ttctaataaa caatatgata
32701 aaggccacaa gcgccctaaa ttgatcatgt ctggtggaca aggtaaagac gaagatttgt
32761 cagaagcaga agcaatgaaa gattatgcag ttaaacgcgg ttatgaccct gatttaatct
32821 tacttgaaga caaatcaacc aatacttatc aaaatatggt ctactctaaa gctgttgcta
32881 tcaaagatta tggcaatgaa aagttcaagg taaaattttt tactaataac tatcatcttt
32941 ttagagccgg attgtatgcg aaaatggcgg atttaaaggc taacggtatc ggcgcaacaa
33001 ctagatttta tttcttgcct aatggtacta tccgtgaatt cgctggtgtc tttatcatgc
33061 ataagaagcg ccactttgtt attattgggt taattgcaat tctatttata attcaagcaa
33121 tattcgctct attaggttgg agcaaataca taattgctta gaggggaaga aacatgctac
33181 agcttttaca tttaaaaaag acatatcatg tcggagacac tgttacacat gctcttaatg
33241 acgttacaat caactttcgt aacagcgaat ttgtagccat tctaggtcca agtggctctg
33301 gaaaaacaac acttttaaac gtaatcggtg gcttagacca ttacgattct ggtgacatca
33361 tcattaacgg taagtctacc aagaactttt ctcaaactga ctgggatgcg tatcgtaata
33421 attctgtcgg ctttattttt caaagttata acctaatttc ccacttatca attattgaaa
33481 acgtagaatt aggtatgacg ctttctggcg taagtagcaa tgagcgtcgt aaaaaagctg

33541 tttctgcttt agagcgtgtt ggccttggcg atcatttaaa aaagcgtcct aaccaattat
33601 cgggtggaca aatgcaacgt gtcgctattg cccgggcaat agctaatgat ccggatattc
33661 ttttgtgtga cgaaccaact ggtgctttag atactaagac tagcgaaagt atcatgaagt
33721 taattaaaga attgagccat gataagttag tcatcatggt aactcacaac ccagaattag
33781 ccgaagaata tgctagtcga attgttcatt tccaagatgg taaaattctc agtgattcaa
33841 atccttttga accaaagaaa gaagcaaaag atacttttaa gctcaaaaag accaaaatgt
33901 cttactggaa tgctttaaaa ttaagtttta ctaacattat gactaagaaa ggtagaactt
33961 ttttaactgc gtttgcctca agtattggca ttatcggaat tgcaattgtc ttagccttgt
34021 ctcatggttt tcaaaagcaa attaacgaca cacagagcaa aacgttagcc aagtttccga
34081 tttctatttc acaaacagca accgatatga atgctgctac aagtcgaaac gaatcagata
34141 aaaacgtcaa aaataagggc tatttagttg cttctaagcc agacaatgag aagaatacgc
34201 acgaaaataa aattacccag tcttacatta attatgttaa aaagattaat cctgattatg
34261 ctaacaacat ttccttcatt cgtggcactc aacttaatct tttaaccaat gataatggaa
34321 aaattaagca tgtagaattc tctaacgtta acaactccgg ctcagctatt gctagtgcgc
34381 agcttcaggg aatgaatagc gttgggatta ataccagcgt ctttccaaaa acactcgatt
34441 caaagcaagg aacattttta aaagataatt accaaatact tgctggatca tggcctaagt
34501 caaacaatga tgtagttctc gttttaaata ataaaaatca agccaacatt aacgcactta
34561 agaatttagg tatttcaatt aaagatggtc aaaagttaga acttaataaa atcgttggtc
34621 gaacttttaa agtgatcaat aataacgatt actaccaaga actacctact ggcaactttg
34681 ttccgcaaaa agcttctaag tctatgtatg attcaagcaa cttaactttg aaggtaacgg
34741 cagtcattcg tggtaaaaat aatagccaaa tggctctcct agataatgga attgcctaca
34801 gcgatggctt gactcaagaa atcatcaaac aaaatgaaaa ttcaaacatt gttaaagctc
34861 aaaaaaatag ctcaactaac gtcatgacta atcagccaat gaacaaaaat caaaaagagc
34921 aattcattgc ttctctaggt ggctctagca tccctagcgg tattcttatt tatccaaatt
34981 cctttaagtc aaaagataaa gtacttgatt acttagacaa atacaataaa ggaaaggcga
35041 aaaagaacca gattatttat actgatatgt caggtacagt aactaaatta actggtggtt
35101 tgcttgatgg tatcaccgat gttttagttg cctttgctgg aatttcatta gtaacctcta

35161 tgattatgat cggtatttta acctacactt ctgtcttaga acgaactaag gaaatcggtg
35221 tgttaaaggc tcttggagct agaaagcgcg acattactcg tgtctttgat gcagaaacat
35281 ttattttagg actcttttct ggtattttag gtattttcat tgcttaccta tgtaccttcc
35341 caattaatgc tgttttatac gcaattacta atatgagtaa cgtggctcag cttgatccaa
35401 cgcaagcttt aatcttagta attatcagta ctattttaac tatgcttggt ggacacattc
35461 ctgctagaat ggcagctaag aaagatgccg caattgcatt aagaagtgaa taaaaaatac
35521 ttatttaaca ggacttcaac tttgaagccc tgttttttta ttctattttt aagctagcta
35581 aaatttataa acttattctt atatattatt gaaaacgtca tcaagaaagt atatgcttaa
35641 ggtaacggat gttatatatg tggatataga aataggtgaa tgaaaatgtt atcgggtgta
35701 acaatgttag atcttgcccg cttccaattc gcaatgacta cagtattcca cttcttcttt
35761 gttcccttct ctatcgggat gggattcatt gtatcaatca tggaaacact ctatgtcgtt
35821 aaaaaggacg aagtatataa aaagatggct cagttctggg gaaaaattta tctcttgagc
35881 tttgctgtcg ggtagttac aggtattatt caggaattcc agtttggaat gaactggtct
35941 gactactctc gttttatggg agatattttt ggtgctccat tagctattga agccttactt
36001 gctttcttta tggaatctac tttcattggc ttatggatgt ttacttggga taagtttaaa
36061 ccaggtctgc actgtgcttt agtttggatt tcctttattg gttctatgtt aagtgcaatt
36121 tggattttaa cggctaatgc ctttatgcag caccctgttg gctatgtcat taaaaacggt
36181 aaagcacaaa tggctagttt ctgggcttta attgcaaatg aacaactttg ggctagtttc
36241 ccacacgtag taatcggtgc gatcgttact gcctcatttg tagtaattgg tatggctgcc
36301 tttggtttac ttagaaaacg cgatgtcaac ttccacaaga agtcaatgcg cgttgccttg
36361 tggattgctt tatttgcctc aattgctgaa attggtgctg gtgactacca aactcaagtt
36421 gaaattcatg aacaaccaat gaagtttgct gcaactgaag gtgtttacga aactactggt
36481 gatcatgcac cttgggatat cgtagctaac ttaaatacta aagaacataa aaatgaaggt
36541 gcaatttcta ttcctgatgt tttaactatt cttacttacc ataagactag cggttctatt
36601 aaaggaatga accaaattaa taaggaatta catgctaaat atgacaagaa atttggcaag
36661 gacatgagct attatgttcc tgttaagacc ttgttctaca gcttcagaat tatggctggt
36721 tttggtgcct tatttgcact actttcaatc ctaggtttaa tttggactcg tccaaagaag

36781 aacacaattg aaaataagcg ttggttctta tggattttag gtatttctac
cttccttcca
36841 tttgttgcga atacttgtgg ttggttcatt acagaacttg gtcgtttccc
atggactgtt
36901 tatggactct tcacaattgc agactcaatt tctgcaagta caacaaccgg
cgagctttgg
36961 ttcacgaata ttatgtactt cctaatcttc tcaactcttg gcggagtaat
gatctattac
37021 tgcaaacgtc aacttgacat gggtgtagct ggtgcacttg aaaggggtgc
atactaatga
37081 tcgacggaat tgatttttta cagttgcttt ggttcttatt aatcggcctc
ttattcatga
37141 tcttctactt cactgaaggt tttgattatg gtgtaggaat ggcaactaag
tttttagcaa
37201 gaaatgatca agaaaagcat ctcatgatgg aaaccatcgg acctcactgg
gatggaaatg
37261 aagtatggtt aatcaccgct ggtggtgcta tgtttgcttc attttctgac
tggtacgcaa
37321 gtctatttag tggctattac attttgctat tctttacttt atttgctcta
atcatcagag
37381 gtgtgtcatt tgaattctct gctcatgcag aaactgaaca tggttttaga
atttggactg
37441 gcgctctatt ttggggcagc ttattagcac cattttttcct aacaatgatg
tttggtagct
37501 tggttcaagg tgtgccaatt gatgctcaag gaaatatgag cttaagtttc
tggaatattg
37561 ttaacccact atcaattgtt gctggtgtcg ctggtgtact tctttgctta
attcatggaa
37621 ttaacttctt aagattaaga attgatgatc cagaattgag cgagcgcgct
gctagtttaa
37681 atgaaaagct ttaccttata gcctacttag gtgaagttgt ctttgccttg
ctagtcttct
37741 tccaaactga tttctttaag ctaagaccag tttcttcaac aatcattact
ttaattattg
37801 ttgccctaac tgcttggtca gatatttctt tgattaagaa caaacaggta
gcagcttttg
37861 taacaagtgg tttaacttta gtggctgttg ttggactctt gttcaatgga
cttttcccac
37921 gcgttttaat tgcaactgat tcagcacact cactcttgat taagactgct
gctaactcta
37981 aattgacact tgaggtaatg acaattgttg ctcttattct cttgccaatt
gccttggttt
38041 acatcatctg gtcatatgta gtctttagac ggcggattaa ggtaaatcaa
aacgcctaat
38101 ctagaacagg aataaaaatg attgataaac gacttttcaa attaccaaaa
gccaaaatca
38161 tgctcgcaat gcttgcagga ctaatgttct tgcaagcatt tgctatttta
ggacaaggta
38221 tttttcttgc ccgagcaatt gttggctctt ggaaacggca accttttact
gaaatagcac
38281 aagatgtgtt acttttttg atcttctatt tattacggca aggtatcaac
tggtttcaaa
38341 aatggtacat gaaccgttat gcaaatcaaa caactacact tttgcgccag
caactactta

38401 ataaaaccta tgatggcgga attgcactag tatcaagaat tggaactggt aacttagttt
38461 ctactttact agatggaatg gatgagattt ctaactatct atctttgatt tttcccaaat
38521 taattgcact tgcaattgtt ccttgggtta ttttgatcta tatctttacc ttaaatgccc
38581 tatctggctg gattttattg ctagtctttc ctctactaat cttatttatg attattttag
38641 ggacagcagc acaaagtaaa gctagtaagc aatacgcagg ctatgttaag ttgcaaaatc
38701 attttgtcga tgccttgcgt ggcttaagta ctttaaaagt tttaggactt gctagaaaat
38761 atggcaatat tgtttacaag aacagcgaaa actatcgtaa aaaaacaatg ggcgttttaa
38821 gagtggctat cttgtctact tttacacttg atttctttac aactttatca attgcgatga
38881 ttgccatgtt tcttggaatt ggcttaatca atggcaattt agttctctat ccttcgctag
38941 ttattttgat cttatcaccc gaatattttt tacctattcg tgattttggt aatgactttc
39001 atgcaacatt gaacggtaaa aacgcgcttg gacaaatttt cgatatttta gcttttccta
39061 ctacaccgca agaggatcag ttatccagtt tcacgtggaa caacgatagc actttagtag
39121 ctaagaatct ttcttttaac tacagtcatg ttgatcaaag taagttcact gttaataaaa
39181 atgctaaaat gagcggcgtc gtaaaaaaga aaacgatgca taaaacaagc caacataccg
39241 ctgatgaatt acgtaatatc aatttgaacc ttactggttt tcaaaaagtt ggcattatcg
39301 ggctaactgg agctggcaag acaactttaa tgcgaatctt agctggtttt cttactccgc
39361 accttaaaga cgataacttc actatcaatg gacaaactct tgctcaactt aatcaaaaga
39421 attggcaaaa tcaaattact tacattcccc aagatcctta catgttcgct gctagtatca
39481 aagacaatct aactttctat aacccaaatg ccagccagga agagattgat gcagctttaa
39541 aggcaactga cttagataac ttcgtggcca gcttaaaaga tggtttgaat actaaaattg
39601 gcgaaaatgg acgtggcatt tctggtggtc aaaaacagcg aattgccttg gcacgcgcat
39661 ttttagcaaa agaccgcaag attttattct tcgatgaacc aactgctcac cttgacattg
39721 aaactgaata cgaattaaag caaccaatga aaaaattaat ggaaaatcac ctagttttct
39781 ttacaactca ccgtctgcat tggttaaatg acatggattg gtgcttagtt attgaaaatg
39841 gagaaatcgt cgaacaaggt actcctgcag atttagcaaa aaatggaact gcctttaaga
39901 aactcactaa gccactgaaa gaagacctac tatgatgaat aaaaaatttt cttggaaaaa
39961 tgatcactgg attaaaccat acctagccca atataaatgg aactttctct tagcaatttt

40021 tctaggcgta atcatgttct tttgcggtgg tgcccttatg ttttatgctg gctataccat
40081 cgataaagcc gctacacgtc cagaaaacat tctaatgatt tatgtaccaa tagttttaat
40141 gcgggctgtt ggtattggtc gtcctttatt tagatattta gaacgcttag tatcccacaa
40201 ctggatttta cgtgttacta gttctttaag acgaagactg ttttacattg ccgaaagaaa
40261 tacttcagct gttggttcaa gctttcaaac aggaagtatt ttatcgcttt taactgatga
40321 cattggtcac ttgcaaaatc tttatctaag aactattttc cctgcaatct tgagttactt
40381 agttggtttc tttgtagtaa ttttgctggg gctcttctcc tggcctttag ctggagcaat
40441 cgcaatttta ttagttgcgg aattagtatt ggttcccttc ttctccctct taaaacaagc
40501 tgctgttcgc actaaagaga aagaagaaaa agcacaactt tacactgaat ttactgacca
40561 agttttagga gctggggact ggaaaatttc tggtagacgg gatgcctttt ttaatcaaac
40621 taaagagact ttaaaatctt taggccaaca tgaaaagaag tctggaaagt ttgattgggc
40681 tagagacttc ggtcttgagt tcatctttgg cttgatggca gttgcgcttc tttatttcac
40741 gaaccaaact cttaccaata atcaagaagc agcaaactat gttggagcag ttgtcttagc
40801 tctctttcct ttatcagatg cctttattcc agttggtcaa ggaattgaag aatggcatac
40861 atatagcgat tctgtaaaac acttaaatga attaaaggta ccagagaatc gtttgcctgt
40921 tcagcaatat ctcgatccta cttttgcagg tactttaaga gtatcggata tttcattcac
40981 ttatcctaaa gaagactatc caattattca aaatttctct cttactctaa aaagaggaca
41041 aaaagctgct ttaatcggcc catcaggtgc tggtaaaagt actatattgc aattaatctt
41101 aggtgattta aaacctaacc agggaactgt gacactcgac cgtttaaacg tacttaaact
41161 tcaaagagaa cgctcaaagt tattctctgt tctaaatcaa gaaccatttt tgtttaatac
41221 aactatttat gaaaatctca aaatggctaa cccagatgcc actacagatc aaatgatgga
41281 aattttagaa aaggtccagc tagctgattt cgttaattca cttcctaaag ggttagacac
41341 agaagtagct gaagctggag cgcgtttttc aggtggtcag aaagaacgtt tagcattagc
41401 acgtgtactt ttacaagaca caccaatcgt cctcttagac gagcccactg ttggtttaga
41461 ttctcttaca gaacaaaaac ttttaaattt agtgtttgaa gttttaaaaa ataagacagt
41521 agtttgggta acgcaccatc tacaaggtgt aaaatatatg aacgaagttc tcttctttaa
41581 agatggcaaa gttacaatgc agggaaatcc gcatgaactt tttaagcata atgaacattt

41641 ccaccaactt tatttaatgg atcaaggact aatctaaaaa atagaaagaa
gcttactatg
41701 tctgctgaag ctaagcaagg ctactatgaa ttagttgaac cacgtacact
atttaattca
41761 attattcccg tattattagg gatgatgtat acagaatata attttcagtt
tttcagaatt
41821 tttcctacaa ttgaaatgtg tattgccacg attgttctac aaatttttat
gaacgtgaat
41881 gatggctatt gggattataa acgtgaaaaa gctgccaaaa ctggcgatca
taagaagaat
41941 cccattggta agtatcacct taatcctaaa cacgttttag cttttgtttg
ggtattattc
42001 atcatttcag ctgtttgcgc tattctgatt ggacttcaaa ctaatatcta
tatttggatt
42061 gttggaatta tttgttatgc tattgctatt tcttattcaa cgggttcaca
cacaatttct
42121 gctggaccgt ttggagagat tgcagcttgt tttgcaatgg gctttggtat
tttcttggta
42181 atggtttaca tcaatgtttg ttctaaggtc tcctttaatt ggaactttat
ttatccaatt
42241 attctggctg ctggaatacc tgaaatttgt aactttactt taatgctagg
taacaattta
42301 tgtgatcatg acgccgatat tgcaaatggt cgccacacac tagtttcata
cattggaatc
42361 aaaggcggat tatatttatt tgtattcaat tatttacttg gtttcttttt
aacaggttgg
42421 gcaatttgga ttggtgtcct tccttggagt gtagctttaa ttttaatttg
cattccaacc
42481 atctataaga acatgcgctt tctttggaaa attcagacga agccaaaatc
atttcctaaa
42541 gttgttcaaa atacgcaagt attgtttatt actgaagcgg ttggtttctt
tatcggtttg
42601 attttaaatc ttagaattag ataactaaaa aaggtcgaaa cttcatctta
gtatgaagtt
42661 tcgacccttt ttagttatat tttaagctaa attagtcact tttcctctta
agatttattt
42721 ttttagtaaa ctataaaaaa atataaagaa aaggatataa acaaacatga
aagtattaaa
42781 cgagtgtcaa ctacaaaccg tcgttggtgg aaaaaattgg tcagttgcaa
agtgcggagg
42841 aacaattggt actaatatag ctattggtgc atggagaggt gcacgggctg
gatccttttt
42901 tggtcagcca gtttctgtag gagcaggtgc actaatcggt gcaagtgctg
gtgcaattgg
42961 cggatcagta caatgtgtgg gctggttagc tggaggtgga agataatgat
cgaaaaagtt
43021 tctaaaaatg aactaagccg gatatatggt ggaaacaacg taaattgggg
tagtgttgca
43081 ggatcatgtg gtaaaggtgc agtaatggga atatatttcg ggaatcccat
attagggtgc
43141 gctaacggag ctgcaacatc attggttcta caaactgcta gtggaatata
taaaaattat
43201 caaaaaaaga gatagtatat gacgggaata tggatagtaa ttattagtat
tattgtgctt

43261 tcaataatta tcaccaatat agtagctcta atacagacac tattacataa
gaaaaatgaa
43321 aaatattatt ttgataaatc ttttggagcc tatggcgata aaaataatcc
aaaatatctg
43381 tttaataatg ttgaacatca tgatttttc catgtatttt atcagtattt
tatttgctca
43441 tatattccat ttatagatgt catttttgg ttcttaggtt caatttggta
ataatcaaaa
43501 aaggtcgaaa cttcatctta gtacgaagtt tcgacctttt tttatttcaa
attattttta
43561 ttgcttccac gctgcatcaa attttgcttt acctgcttta atttgattat
caacattttg
43621 accattctta gctgcataca aaatcttagc cataattgga tctaattgac
tgtaggcagc
43681 gtttgagttc ttagctactg gaatactgta caagtgtttc atagcccctt
ctaacttagc
43741 tggaagctta gtcttggtgt tttctttgta ttctttagat ttcacaacag
aattattaac
43801 tggaatatag ccagttgcat ttgcccattt aagttgagta gatttagaag
ttaaaaattg
43861 catatattta aatgcggcag ttctttgcat tgctgttgct tttttaaaca
tgtaaatatc
43921 agtaccttgc tgcatagtat atttaccagg gcgtgcagct acatcataag
taaacttatc
43981 accaactgcc tttttaacgt agccttcacc tgctgatgtt ccaatataca
ttgctacctt
44041 ttcattagca aatggacctg ataaatagtg tgctgaacca gcagttgtaa
agtagccctt
44101 cttcatacct tgagcatagt aatcaataac tttcttggaa tctttgccag
taaagttaac
44161 tttatcagtt aaattaattc cttcattttt catacccaag gtatagtaat
tagctaaaga
44221 atcaaagcct gcaccaacaa cttgatggtt actcttctta taaatggttt
ctgaaacttg
44281 tgctaattcc ttcatagttg taggaacttt tttaatacca tatttttcaa
acatggactt
44341 gttataggtt aaaacttcaa tagatttatt aaatggaata ccgtattgtt
ttccttgaat
44401 tttagctcca tccaataatt cagttctaat atttgactta gcagcacttc
cccaaccgag
44461 cttactgtta ttaatgtatg gggaaagatc aaccaacata ttactcttaa
cagcattata
44521 gagccaacca ggatatgctt gcgtaattgt tggtaaatta tttggtgatt
gtaatgttga
44581 atttacctta gcttgtaaat caatataaga accttgattt tcaagtttga
ttttaatatt
44641 aggattttc ttttcaaatt ctgcagttaa tttttctaat tctgcacgct
gaccaccatt
44701 cattccatgc caaaaggtta ccgtagtttt tttggtaatt ttagttggaa
tatttgctgc
44761 tgaactatct tctttatttc cttttgaaca tccagtagca attaatgcca
accctgcaac
44821 taaacctaaa gcaatctttt tatataaatt catcaaaatc ctccagtaat
ttcaaaattg

44881 aagcactatt tattgtatca aagtaaaaat catatacaag atgttgtagt ataaaaataa
44941 gatttttat tttgaaattt ttcatatttt aaattctgcg tcaaacgcgc attatatata
45001 gcttttgtat atataaaata cgtatatatc cgtacattat aaagaataat gtttataatt
45061 attcatattt tgccttgaat tacgattaat agtctgctat ctttaaatcg tcaacttcat
45121 taaatagttc tggaggaaaa aatgaagata aagaaatttt tgattggcgc cgctatggtt
45181 ttacttgctg caacaagtgc agcttgctcg aacagtaaat ctgcttctaa atcaagtgat
45241 ggttatactc ctaaggagtt aaacgttcaa ttcgttccta gtgtccaagc ttctaagctt
45301 gaagctaaag ctaaaccatt acaaggttta cttgaaaagc aattgcacat gcctgttcac
45361 gtaactgttt caactgataa ttccgctttg gttgaagcaa tggcatctaa gaaagttgat
45421 gtcggtttct taccacctga tgcttatgtt ttagctcaca agcgtggtgt agctgacgtt
45481 ttacttcaag cacaacgtta tggctatgac gaaccaagcg gtaagcaaaa tcataaatta
45541 atggacagct accgttcaat gatcgttgtt aaaaaaggtt ctaagattaa atcttggaaa
45601 gatcttaaag gtaagacaat tgccgttcaa gacccaacct ctacttctgg ttacgttctt
45661 ccaatcgccg aacttcacaa gaagggctta aacgtaccaa aggattgtaa gttagttcaa
45721 gttaagggac acgatcaagc tgttttatca gtttataacg gagacgctga tgctgccttt
45781 gtcttctcag acgctcgccc acttgctgca aaagatgcac cagccgtaat gaaagatgtt
45841 gttccaatct actttaccaa gtacattcca aacgatactg tttcagttag aagtggtatg
45901 tccaagtcat ttaagaagaa acttgctaaa gcattcaaag atattgccaa gactaagaaa
45961 ggcaagaaga tccttgaaaa catctaccaa cactatggtt atgtagattc taaagattct
46021 aacttcaaca ttgttcgtga atacgaagct gaagctaaaa aagctcaaaa ataagtttaa
46081 tatttttaaa ggccacccta ctttgacttg taggatggcc tttttctcgt ttaaaattag
46141 aatagatttt taatcgaggt ttttaaatga aactaaataa aaaattttct ctactactaa
46201 tagcaagtac tatttttttg agcctaagtg cttgcagcaa accaactcat gaagagcaaa
46261 caaataaaaa gctagccaat cacattgctt taatctcaga taccaatgga attaaagata
46321 attctctaaa tcaatctgcc tggcaaggcg ttaagaatta cggtactaag aataaccttg
46381 ttgaaggaaa taaaggctac aattatttcg ttcctaaaga tccccaagat tatcaagcaa
46441 ctatcaacaa agttctagat aatgattttt caacaatcat tggcgttggt tatacactta

46501 agcctaatat tgttaaagcc gcgaagaata atcctaaaaa gaacttcgtt cttattgata
46561 ctcatgctgc taagattaaa aacctagcta gcatctcctt taaaaatcaa gaaggagctt
46621 atttagcagg cgttgcagct gcctacacca ctaaaaacca cacagtggga cttattttag
46681 ggcaaaacag taaagagatg gattccttta aagccggttt tactcaagga gttacagcca
46741 gcagtcgtaa gcttcataag catattaaag tgatgagcaa aactgtcggt aatttttcag
46801 atattaataa agcgcatgat attgcccagg aaatgtacaa tcagaaagct gatataatct
46861 tccaagctgc gggtaaaagc ggtcaaggag tctttaaagc tgccaaagaa gtcaatcaaa
46921 ccaatcccgt aggacaaaaa gtctgggtta ttggttcaga tgaagatcaa accaaacttg
46981 gaaattacca agcaaaaggc ggtcaacctt ctaactttac ccttacctct gtaatcaaaa
47041 gcgcagatgt agctgttgaa gatcttgcta atcaaactgc taaaggtaat ttccctggtg
47101 gaaagaactt aaactatggt cttaaaaaca agggtatttc tctagtacaa ggtaatttat
47161 cttatcatac ctggatcaaa gttcagaaag ctaagcagaa aattattgat ggtaaaatca
47221 agatcatggc taatcgttaa aagatcggta gcatcaaaat taatacaaac taaaaagtca
47281 cattaaccta tgccaaaaag ttacttgtgg ctttttcttt tgctttattt tctacattac
47341 tatctacatt acaaaacaca ctataatata gttgtataga tacaaagaaa gtgtgtttaa
47401 gatggctaat gaaagtaaaa tagtgactct taaaaatggt tatcatgtat ggactcgtaa
47461 agaaggacac ggtcctatta agattttgct tctgcacggc ggtcctggca tggatcatga
47521 atatcttgag cctttttcta attacattaa aatgcatccc aatatagaga ttatctacta
47581 tgatcaattg ggatcatatt tctcagatca acctaatgat ccaaagttat ggacaattcc
47641 gcgttttata gaagaaatag aagaagtccg taaagcatgg gatttagatc agttttattt
47701 atacggtcaa tcatttggtg gtttatttgc tttagaatat gccgcttcta aatacggcaa
47761 acatgtgaaa gccctaattg attcaaacat ggttgattca tatcaagact acgcaaaata
47821 tattaacaag taccgcgaat cgatggatcc tgctgatgtt tcatatatga agaaacaaga
47881 agaacgacac aactatgatg atccacatta tcaagaacta ttgatgaaat tatatcaccg
47941 ctgcatttgc agaatatctc catggcccga tgccgtgcaa agaacttttg atcatatgaa
48001 tgagcaagtt tatgtcaccc tacaaggacc aacggaattt aacattactg gttcatgtaa
48061 aaattggaca attcgggatc gtttatccaa aattacgatg cctactcttg tattaggagg

48121 aaagtatgac agcatgaatc cagctgatat taaagcttta gccgatagat
tacctaatgg
48181 tacagctcat atatgtccta atggaagtca cttttcaatt tttgatgacc
aaaaagatta
48241 ctttaatgct ataagtagtt ttatcagaac agttgaaaat aaataagaaa
tacatatatt
48301 tttatcattg atatgatcta taccacattc tatactgaga gcgtaagact
tgatataaag
48361 tcaaacactt tctacattaa taaaaaagcc atattgcata aatacatgca
atatggcttt
48421 tttattttta taaacatata cccagcacca ttacatttta tacatttata
tataggaagt
48481 gattgacatg gtctagccca ctaactatac taaacgtgta agcgcttata
tgtatttagt
48541 gtaaaataat gagaggataa aataatgaaa attaaacgtg cttttacatc
agtaatagcc
48601 ggattttcaa tagctgttgg gtgtttggcg ataaacaatt tatctgctaa
tgctgcaact
48661 actcaaaacg gtgttactaa tgatcaaata aatcagtatg taaacaacgc
tactctagaa
48721 caaaaagtgg gacaaatgta tgtatcaaga accccacaag atccaaacca
agttaaaaat
48781 gatgtttcta aatataacct tggtggatta attgtttatg atgctgatat
gaagggactt
48841 actcaacaac aatttaaaga taaaatgaag agtttccaag actctgctca
aacaccttta
48901 ctagttggtg ttgatcaaga aggtggacta gtttcaagac ttactcacag
cggattagtc
48961 caacaaaatg gtaatcaatt tgcctttcca agaaaacaat acgaagaagg
tggcatgaaa
49021 gaagtagttg atgatgccga aaagaatgcc actcttttac gaagcttagg
tattaattgg
49081 aattatgcgc cggacgctga ctatagtact aagccaggaa catttatgtg
ggacagaact
49141 tttggacaag gatatcaaca aactgctgac tacctatcta aagttgttcc
tgcatggcaa
49201 catgataatc tcgtcgcatc aacattaaag cacttccctg gatatggtga
tgctgccgac
49261 acccatactg gatttgcaga aaacagcaaa tcattaacag aacttgaaaa
tcaagacatg
49321 gtcccattta aagctggtat gaaagctggc gcagattccg tcatggttac
tcacgtcata
49381 tataacaaga ttgatcctga atatccagct tccctttcac ctaaaattaa
ttctttaatt
49441 cgcaataatt gtaactataa cggtgttata gttaccgatg ccctagagat
gggagctatt
49501 acagacttcg ctaaagaaca cggtaatgct tcagtagacg tccttgcagt
taaagctgga
49561 aatgatatga tcatgaccac agattatgct actggtatca aggagattgt
atctgctgtt
49621 aaagctggcg aaattcctga atcacagatt aatgcatctg tcacaagaat
tttacaatta
49681 aagaataaac taggtctttt aaagcctgaa aacttaatgt tcaggaaacc
tacaaataat

49741 aataataata ataataatac aagtaccatt attaaccctg gaaatactga ccataagaac
49801 cctagcgtta tttcaggaaa taattctatt actattagtg gtattactta taacaatgat
49861 aaaaaagttg cctttattaa tggtaaagta tcagatcctg cagcagaagg ttctatgatt
49921 atgactgcta caaatgctaa tactggaaaa aagattacta ctgcagttgt tggtggaaaa
49981 ggcgtatttg ctattgaact tcctatgact aatgaagttc aaaatgtaaa aataacttca
50041 gatgatgatt catatgctgc agttactact aaggtagaag ctatcaaagc tgacaagcct
50101 gtagttaata ctgaccaaaa taataagcaa aatagctcta cagaaaaaca aacaaataag
50161 caaaacaatt cagttaaaga tgaagttaat atcactttaa atacagagaa acaagcaaac
50221 agcgctaaaa agaatactca agtagtatct aagaacatta attccgaaaa gactactgta
50281 accaatccgg tttataaatc agctataaaa actaattacc aaggtaaaaa tgttattact
50341 actagccacg attcagcagc ccatgctgtt gcacaaaagt caaataacaa cgctaaagtt
50401 ttaccaaaaa ctggtactaa agaaaatatt gtagcattat ttggattagc aataacttct
50461 actactgcat tatttggatt agcacttaga aaaaagaact aataatacaa aatatattaa
50521 ttaaacttaa aagagttgag tcaaaaaatg attcaactct ttttttacac tcttaagggc
50581 acaaatatat gaaaaagtta gaatactaat actatatagg cttagttttt tatatattca
50641 taacttatat ataaacttta tagctattta tgaaatatta ttttatttta tgcctatttt
50701 tagtgttttt acgaaatatt atttcaaaag tgctataatt aacttgaaag cgatattaga
50761 aagagaggga aatataatga aaatttctgt tatagcaata atttttctaa gtctttatgt
50821 aggcttaggt caattagatg ctctatcatt aaaaacaggt ctctactcac cagtattcgg
50881 aggtgcagtt acaggtttag tactaggtga cttaaaaact ggattaatcg ttggtgctac
50941 attacaattg gcaactttag gtgtagcaac ttatggcggt gctacagttc ctgactttct
51001 atctggttcc gttatgggaa cagcatacgc aataatttct ggtcgtggcg ctgcatacgg
51061 tataggtttg gcaattccta ttggtttact cctaactcaa atggatatct tagctcgttt
51121 agctaacacc tattttcaaa atcatgctac taaagaagca gaaaaagtta attacactgc
51181 agtagaacgt gataatcttt atggcattat ttcttgggta ttatcacgtg caattcctgt
51241 atttattggt ttaactttcg gtgccactgt tgttaaggct ataaataatt ggattccagc
51301 ttggttaatg aatggtttaa agacagcggg tattattcta ccagctatgg gtattgcaat

51361 attaatgaga tatctaccat taaagagata ctacccatac tttattctcg gttttgcttt
51421 aatggcatac tttgcaaaac aattctcatt acttggcctt gctttagtag gtttctccct
51481 tgcagcaatc tacgtaatgc aaaaacagaa gaacaataaa tctggaaaag ctcaaaatat
51541 agagaaaaaa gattctaact ccaagaagga aattaaagga atacaaaatc ctaatgaggc
51601 aattgaagca agtcgtgtag atgctgagga ggttgagttc gatgcctaaa aagaatattc
51661 aagttaaaat gaaaaattta actaaaggtg atttagtaaa catattttgg cgcaatactt
51721 taggcttaca acttggatgg aactatccaa gaatgcaagg cttaggctat gcatatacag
51781 tgatgccagc tttaaagaga ttatataaaa ataaaaaaga taaaatgaag caagccttaa
51841 aactagaaat ggaattcttc aataccacac cacaaatgtc tcatttaata gtaggtgccg
51901 atgtcgcctt acaagatgaa ttgggtatgg atgataataa tgaaaaagct atagcgggtt
51961 taaagaccgg tttaatggga cccttcgcag gtgttggtga taccgtcttt gtagcaatct
52021 ataacgctat tatctattca atcactgcct acttagcact atctggccaa ccattaggac
52081 ttttaattcc cgtagttgga tgtttagcta ttacttggat tagatggaaa ttctttaaca
52141 tcggattatc acaaggtcaa aaacttgctt ttgcattttc agacaaaatg aacatgctta
52201 ctgaaggagc ttcagtttta ggattaacag ttgtaggtgg tatgattcca gctcttatta
52261 attataaagt tgatctaaca tataaagtcg gaaaagtgac catgtcggtt caaagtatgc
52321 tggataagat tatgccagca ttattaccgt tagctattgc cctattctca tactggattc
52381 ttggtaaaaa gcatatgaat tcaaccagat taatcttcgt attaattgga ctaggtatga
52441 ttttaggaaa cttacaacct atttgtaact ggattggtag catttttttag aaggtgaaat
52501 aaatgagtat ttgtgcagca cgtgttgatg aaagaatgat acacggacaa gtagcaatgg
52561 tatggacaaa tgtggtcggt gctaaccgta tactagtagc caatgatgaa gtcgtaaaag
52621 atgatttaaa gattgaggga ttaaaattag caaaaccagc tggcatcaaa ctcagcattt
52681 gctctataca acgcgctata gaaaatctaa aaaataataa atatggtgaa gatgatgtct
52741 ttttgattac gcgtaatatt cccgatatgg ccgatatcat taatggtggt gttgatctaa
52801 aagagtttaa cgtaggtaat atttcttcta aagatggttc tcgacaaata aaaaagtcag
52861 ttaatgttac ccctaaagat gtagatatca ttaagaattt aattaacaaa ggcgtaaaaa
52921 tcactgctca aatggtacca aatgaacccg attcttccat catgacttat attgaaaaat

52981 agaggactaa attatgcagc aaaatacagt tgataaaatt gtaaaagtca taaatcagtc
53041 tatacaaaat aatgatatat atggtgcttc ctacagttta attggaaaaa atgaaaatct
53101 tagaacctat gttggatacc agggagaaga caatgaccgt attccgatat cttctggaat
53161 gatgtacgat ttggcgtctg ttagcaaagt tatcggaact accactagaa tttttcaact
53221 actagccaat aggcaactat ctcttgatag tgaagttgga gaatatttat ccgatatttc
53281 ttatcccaaa ataaaaataa aaagtttatt gttacatgaa agtggtttgc aatcagattt
53341 tgataatgtt catagcatgg caaaagaaga attaataaaa aaagttaagt ccgccccact
53401 tatatacact cccgagagta aaactgtata ctctgattta aattacatct tacttggatg
53461 gattattgag aatattgacc aaatccctct aaatgaagac ctaaaatcac atatctttca
53521 accgttagat atgaataata ccacttactc acctaacagt attccttcgt ctcaatttat
53581 acctactgaa taccaaaaag atcgtggtgg cataattcgt ggaaaagtgc atgactataa
53641 agcttatttg ttagacggta taagtggaca tgccggatta ttctcaactt taaatgatct
53701 atcgcaattt gcccaagtct ttctcaatgg tgggaaatac aaaggaatac aagtatttcc
53761 taaaataatg tataccctac tgaaaaataa aaaatataga cataatgatc gtacattagg
53821 ctgggaactt tgggattcaa ataaaaatat gctatggcat tctggtttta caggaacttc
53881 aattgctctt aatctagata gccatgaagc ttttatatgt cttactaaca gaatttaccc
53941 aacacgtaaa aaaacgggct ggattcaaga aagacgagag tcgttatctt atttctttaa
54001 tcagaaagaa gaaatcaaaa aatgagtata tatattaaaa aaattacgtg tagtaaaaaa
54061 acggtcccat taaagagacc gtttattact gccttacata aagtcactga aattgaagga
54121 ctgaagatcg ttgttgaatt aagtgatggt caaacaggta ctggaacagc tactcctaat
54181 gagaaagtta ccggtgacac attacaaagc tcgctagaca ttgttaatca agttattaaa
54241 cctgcattaa taaaccaaga cttcaaaaat tggaacaaaa ttttaaccac acttcaaacc
54301 tgtatcgttc ataattctgc cgcaaaggcg gcaatggaca ttgccttaca tcaattaaag
54361 gctaaagaaa acaatgaatc tctagttaac ctgcttggta gtgaaaaagg caaattaaca
54421 accgactata caattagtat cggttcagat gaccatatgg tttctgaagc caaagcctta
54481 gttaaagatg gctttactgc aatcaaaatc aagttaggca ataattctgt aagtcatgat
54541 attaaaacca ttcaaaaaat tagtaaagct gttggtcctg acatttcctt acgtattgat

54601 tgcaatcaag cctggaatta caaagacact ttaaaagcta gtcgaacttg ggcacaggat
54661 aatttaaaca ttgattttat cgaacagcct gttctaaaag atgcaatctc agatttaact
54721 ttattaagtg aaaattcgcc ttatccaatt atggctgacg aaagtgtcgc ttcatatcag
54781 gatgccatta acttaatcca gcatcacgct tgtgactaca tcaacattaa gttaatgaaa
54841 actggtggct tatcagaggc aatcaaaatt aatgatttag cccaagcttg tggtattaaa
54901 actatgattg gttgcatgat tgagccagtt gaaagtattg cggcagctgc tgcattcgct
54961 gttgcaaata aaaatgttaa atttattgat ctcgattcaa tttttatggc tacacaagat
55021 cccgatctta gtaagtattt agaaattaaa aataatgaaa ttattctaaa ggactaagac
55081 aatgcaactg acccaaattc aagaacaaat agaaaataat ccttggaaaa cagcaattac
55141 tattcgtaaa aataatcata cgatctttga aaattcaaac gctaatgtga tttttaagtc
55201 agcctcatta ataaaattag ggattgctct ttatattcag gaaaaaaagc ctggcacaat
55261 cgccaatact cttaccctat ctagtaatga tattgttgga ggagccggca ttataaatag
55321 actaagcatt aagtcatgga agattagcga tttattagac ctaatgttat ctgtttctga
55381 taatacagct actaacgctt tattgaatta ttacaacatc aatacaatta atgattattt
55441 acagcgaaat tttcaaaata tttctttagg tcgattttta atgaaaaaaa gtgataaaga
55501 aaatacttgt acatcaaatt ctatgatgga cgtttttgaa aaactacttg caggtaataa
55561 cgaagttaac catgtggttc tcgatgcctt aaagcaccaa gaagtaagaa ataaattagt
55621 agcttatagc aatcctaagt atgaagtctt taataaaact ggcgaactat tgcatgaaca
55681 gcatgatata gcacgattta gagcaaacac tgatgtgatt gactgctgcg tattaaccaa
55741 ttatcaaagt caaaaagatt atgagaatat tttaagtatg atgcaaaaga tcggaaaaat
55801 acttacacac tagaaagaag tatttacaat ggaaattaaa aacttaacta cggagcaacg
55861 aaaccccgcg actatgcaca ttgattccat gtcaactatc gacatggtca aaactattaa
55921 tcaagaagac caaaaagtcg ctgtagcagt gggaactcaa gatgataaaa ttgcccgggc
55981 aatagatgaa gcagctaagc gctatagcaa aggtggacgt ttaatctata ttggtgctgg
56041 aacaagcggc cgtcttggcg ttttagatgc tgccgaatta gttcctacct atggtatcaa
56101 accagaacgc gcaatcggat taattgctgg cggtaaaggt gcgatgtatg ttgctgtcga
56161 aggtgctgaa gattcacaag atctagcaaa aagagattta aaggatctta aactaaataa

56221 aaacgatatc gttttagggc ttgccgccag tggtcgcact ccatatgtaa ttggcggtct
56281 tgactatgct aaagccatag gtgctctaac tatttccatt gcctgtgtta aagactctaa
56341 gataggccat cacgccgata tagctatcga agctgttgtt ggacctgaag cgattaccgg
56401 ttcaactagg atgaaagccg gaactgctca aaagatgatt ctgaacatga tttctactgg
56461 tgtcatgata aaacaaggaa aagtctacga aaacgtcatg attgatgtta aacctactaa
56521 ttctaagcta attgatcgtg cctgtcgcat tattcacact actactggtg tatctaccca
56581 gaaagctaaa gatactctta aagaggctaa taatgatgtt ggcttagcaa tcgtaatgct
56641 taaaactaac tgcagtctca atcaagcaaa aagtttgtta gagacaaaaa atggaaatgt
56701 cgcagaagtt ttaaataagt agcttgctag ctaaacagaa ggggcttata atacctacca
56761 ttgcaatata tcaatttaca atgtgaggcg aaattatgag tcctttttc ttattcattt
56821 atctgatttt tggtgggatt ttaggtggcc ttctctcaac aattgcaagt atggcatccc
56881 ttgccactta tccagtcctc ctttcagttg gtattccccc agtttatgca aatactacta
56941 atgatgcagc cttaatctgg actggcgttg gatcaactgc atcttcgctt aaagaactaa
57001 agggtcactg gaaacaagtt agtttctatt caatttttac aattatcgga tcagctttag
57061 gctgtatgct tttaattcaa tttcccggga aaatatttga gaagatcgtt cccttctgta
57121 tcgctttttc cggcttaatg atttattca gtggagaaat tcatcttgga aatgaaagtg
57181 atcatcctaa tcgcttagtt caagctctat cacttttatg cttattgatt gctggagctt
57241 atgctgggta cttcggcgct gcgagcggcg ttttaatgtt agttatcttg aacgcaatta
57301 gtgatgatac ctttttaact gttaatgcta tgaaaaatat tattggcgca ctctccaatt
57361 tagttgcgct aattatctat atgtttaccg aaaaaattta ctggagcgct gctattcctt
57421 tggcaatcgg agcgttgatc ggtagttact ttggaccacg tatcatgcgc catattccag
57481 ttaagatcat tcgtttgggc attgccgttt tagccctcat tcaagctgct tacttcgctt
57541 atactgcgta taaataaaaa caaattttat ctaggaaaaa taaatttagt ttgcgtaata
57601 aacatatggg attacttcct caaatatttt tagagaggat ttactcatat ggtaaaaaag
57661 attgaaccct ggtttgggtt cacagaagat aaggttttcg gaatggtgat ggaaaacaaa
57721 gacttctgca agtatttgct tgaaattatt attccagatc tgaaaattga aaagatagac
57781 tggttagata agcaagttga gattaataat tttgagcgca agaatgaagc taaagaagtt

57841 agacttgatg ttttggttac agaccatgag ggacgcgtgt ttaacatcga aatgcaaaca
57901 acggatcaag atgatatcgg tagacgcatg cgttactatc tttcaagact tgatttacgc
57961 tatactttaa ataagggtaa gacttatcga aacttgaaag acgcctttat tatttttctc
58021 tgtaacttta aaccaaagaa agatgataaa ttttacgaat cttatcatac ttattctgat
58081 caagataggt caaaacaatt gcaggatggc gtgacaaaga ttatcattaa ttcacaagtt
58141 tcagctgagg gacaaagtga agacttaaag gctctagcta aactgatgaa taatgagcca
58201 gttaaattaa ataaacattt cgattacgcg caaagaagaa ttaaagaaat taacgaagat
58261 ccggaaacga gggagaagat tatgctttac gagacaagaa tgttagaacg tgaacaggct
58321 gctgctaaaa tagcagggaa agtagcatat gcagaaggta gaaaagacgg tgtagaacaa
58381 ggtagagcag aaggtaaaat tgacgttgct aaagtcattc ttgaaaacca aatagacaat
58441 ggcagtacgc tagaacaagc cacaaaattt gtcaaagatt taaagataat ctctgataaa
58501 gatctcgaaa aattggtcaa gatatataaa taaaattgtc aaaaaataat tcttaaaaca
58561 acaaaagatt gattaatctg cgttactaaa acgttggtta atcaatcttt ttattttttc
58621 taaactagat catcgcaagt ggcgtctttt cagtcggctg cgggaaatca ttatctatta
58681 attttatttc gtccgatgtt aatttgatat cttgagcagc aatattctcc ttcatatgct
58741 taaccgttcc cgctttagga atcgataaaa cattaccatg tcttaaagtc caagctaaca
58801 taatttggtg cggagttaca ttgtgcgcac gagctactat tttaagatta ttagtaatcc
58861 taatcgaatc gcccttacct gagttaaatg gagaataccc aataaaacca acgccatgct
58921 tttttgcca aggtaaaaca tcatattcta ccccgcgttc agaaaggtta tacaaatctt
58981 cattggcaaa acaatccttg ccgcctggga cactaaacaa ttcttctagg tcagctacat
59041 caaagttaga tacaccccag tggcgaatta agccttcatt ttgtaattct tttagtccgc
59101 gtactgtttc agataaacgc ttatcgccac gccaatgaag caaatacaag tctaaataat
59161 cagtttttag tcgttttaag ctattttcta agctctgacg ttctaattct ggagaagcat
59221 gatacggata aaattttgag atcaaaaaga tttgactgcg atcataacct tgaattgctt
59281 cgccaattaa tttttcactt tttccttcac catacatttc tgcagtatca attactttaa
59341 ttccattatc taaaccatag cgaagtgcag ctatttcgtc tttttctta gaagcgtctt
59401 cgccaattcc ccaagttccc atgcctaagc ctgacaagtt attttcttga aaactcttct

59461 ccatctcttc tcacctcacc taaattttac cgcagaaaaa aacaaattta
aaaatgttag
59521 cggtttaagt tgactagacc aattttatat tttataatgg actagaccaa
tgaatgagga
59581 ggtacacata attatgaaaa tcatcgtaac taaagataat attgaaggcg
gcactaaggc
59641 atttgaaatt attaaaaagg gaatggaaaa cggcgacaaa gttttaggcc
ttgcaactgg
59701 ctcttcccca attccaatgt atgacgatat gtgcgacagc gacttagact
tctctgacat
59761 gacaagtatt aacttagatg aatactacgg cttaaaacca gataatgatc
aaagctacca
59821 ctacttcatg caaaaacact tattcgataa gaagcctttc aagcattcat
atattccaaa
59881 tggaatggca aaagatattg acgaagaagt tgaccgttat aacgacatta
ttgctgctaa
59941 cccaattgac atccaaattt taggtattgg tagaaacgga cacattgcct
ttaacgaacc
60001 aggtactcca tttggcagct taactcacaa agttcaatta actgaaagta
ctattaaggc
60061 aaattcacgc ttctttgaca atgaagacga agttcctcgc caagctattt
gtatgggaat
60121 taagtcaatt atgcaaagta agaagattgt tttactagcc tttggcgaat
caaagcaaga
60181 tgcagttaaa gcattagttg aaggtccagt tactgaagaa gttcctgcat
ccattttgca
60241 agatcaccca gatgtaacag ttatttgtga cgaagtagct gcagcaaagc
ttgatccaaa
60301 atacagaaaa taatcctcat ttaagactgt ttactttggc gaattaatct
ttttagatta
60361 gttcgtcttt tctttttgta ttttttgat gtgttctctt gacttttttc
ttttatagca
60421 aattaaaaaa ccacttgttt tcataccaga tattgaatta cactattaaa
gcaaactata
60481 tcttgtagaa agaacgtgat gacagttatt gtattgagcg ggcctattgg
agccggaaaa
60541 tccagtttaa ccagtcttct tgcagagcat ttaggcaccc aagcatttta
tgaaggtgta
60601 gataataatc caattttgcc actttattat aaagacatgg cacattacac
ttttctctta
60661 aatacctatc ttctaaacca tcgtttggct caaattaacc aagctattcg
cgatcataac
60721 agtgtgtctg atcgttcaat ttatgaagat gccttatttt tcaaaatgaa
tgtcgatagt
60781 ggaatcgcag accctactga attcaagatc tacgatagtt tacttgagaa
tatgatggaa
60841 caagccccag gcaacccaag taagaaacct gatttgttaa tttatattca
tgtatctctt
60901 gataccatgc ttcaccgcat tcaaaagcgc ggtcgtacat ttgaacaatt
atcaactgat
60961 ccaggcttaa aagactacta tgctcgactt ttgtcttatt acgagccttg
gtatgaaaaa
61021 tataatgcat cccctaagat gatgattgat ggcgataaat atgactttgt
tgcagatgaa

61081 gatgcacgaa aagaagttat caacacaatt gatcaaaagc ttactgatct aggtaatcta
61141 aactagctaa cgaatagaag gaacgtgatg acagttattg tattaagtgg gcccattgga
61201 gccggaaaat ccagtctaac aggtattcta tctaagtatt tgggcactaa tcccttttat
61261 gaaagtgtag atgacaatcc tgttttgcca ttattctacg aaaaccctaa gaagtacgca
61321 tttttattgc aagtttattt cttgaatact cgcttccaaa gtatcaagtc agcattaact
61381 gatgataata acgtactcga ccgttctatc tacgaagatg cacttttctt ccaaatgaat
61441 gccgatattg gccgtgctac taaggaagaa gtagatactt attatgaact cttacacaat
61501 atgatgagcg aattagatcg gatgcctaag aagaatccag atctccttgt tcatattgat
61561 gtatcatatg acacaatgct taaacgtatt caaaaacgtg gtcgtaacta cgagcaatta
61621 agttatgacc caactctaga agactactac aagcgtttgc ttcgctatta taagccttgg
61681 tatgaaaagt atgattattc accaaaaatg actattgatg gtgataaatt tgacttcatg
61741 gcaagtgaag aggatcgtca agaagtctta aatcaaattg ttgctaagct caaagaaatg
61801 ggtaaacttg acgaaaattg gaaaccaaat ttagttaaat aaagtaaaaa catgttagaa
61861 tactttatag gaacagtgtt actatctgct ttataattta atgcgcagaa tagtgcttag
61921 gcggtcgtta ggccgttttt tttataccaa aaaactgcgg tccgtgttgg atcgcagttt
61981 ttcatatact cacatcatta aactaatgtc ttgaagcacc acttactgca tctgcagctt
62041 caccagttaa ttgattttcc ttagtgacag gatacggctt cataacactc ttactcgttg
62101 aagaaattcc ccaatcagca gtgtagtcaa gatgatcaat tttagcatgt ttaatatcgt
62161 caatcgtctt acaaccagtc aattgcatgt caatcaacaa ttccttattc aattgctcaa
62221 taactgactg aacaccttga gcaccaccaa gtgctaagcc gtataagaat ggacgaccaa
62281 taccaactaa gtcagcacct aatgccaatg ccttaaagac gtgtgaacca cgacgaacgc
62341 cgccatcaag gataattgga acggggcgat cacaactctt cacagccttc gcgatttcag
62401 gcaaaacatc aattgtagca ggagccccat caacttcacg acctccgtga ttagaaacta
62461 caattccatc tgcaccagca ccaattgcaa gcatcgcatc ttcagcacat tcaacgccct
62521 taacaattac aggcacatca gcaatgtcct taatcttacg aatatcatct ggcccaatat
62581 tttgagcaga tgaagcgtac atttcagcaa cgctttgtcc ctctcctttt ccaccagtcc
62641 attcattaaa gaaagcaagt ggaacaggat atgtaaagtt agtccgtaaa tttgcttcac

62701 gatagccact aactagcgca tctacagtta agaagaaacc ggtaaagccg gctttaactg
62761 attcttcaat taccatttta ttaaagtccc agttcttgct tagatataat tgcataaacc
62821 gtggggagtc gcctactgcg ttttgcacat cttgaacact cttatttgca taggtactca
62881 ttccaaacaa agcaccggca gcttttgcac ccttagcagt atcaatttcg gcttcggcat
62941 ttgcaatacc atggcaggca attggacaga tcataactgg tgtctttaac ttcatcccta
63001 aaaattcagt attcaattct ggatcctgca tcccagttaa agcacgaggc acaatttgaa
63061 aatggttaaa agcttgtgta ttattccgcc aagtccattc attttctgca ccactagcaa
63121 tgtagtaata agcagcttct ggtattacat attttgcttc gttttccagt tcatccaagt
63181 taaccatgtg tagtttttct tctctagtac tttgtgggaa ccccttatag taagttgtca
63241 tcgatatttc ctccaatcat taagtaggtt tctatacacc ttaattttag gacgatttca
63301 ttaatttatt aaataaaagc tattatgaaa taattcacta agtccgatat gctcactatc
63361 catgttcttt gtagcgcttt ctttagttaa aattcgtgtt tacatttatt tatcagaatg
63421 ctacactgta aaagttgctt atttaaagct aaaaagcgaa cattatgtct ggaggacaaa
63481 atgaatttta tcaaaaacta ctttcattta gaaaagttgg gaactaatgt tagaactgaa
63541 tttattgcag gtctaacaac ctttattagt atgtcttata ttctcttcgt taacccaagt
63601 gttcttggtg ctagcggcat gaatactggc gctgtattta ctgcaactgc tttagctgcg
63661 gctcttggta cagcaatcat gggtattgta gctaactatc cgattggtga agcacctgcc
63721 ttaggtatta acgccttctt tgcctatacc atatgtattg gcatgaaggt atcttgggaa
63781 actgccttag ccagtgtttt tgttgcatca attatcttta ttttaattac tttatttaaa
63841 ttaagagaaa agatcattga ctctatccct tcagacctta aatttgccat ttcatcaggt
63901 attggtcttt ttattgcctt cctaggttta caaaatggtg gtttaattgt tgctaacaaa
63961 tctactttag ttggtttagg atctcttcat aatccgcttg tttggattac aatttttggc
64021 ttaatcgtta ctgtaatctt aatgatttta ggcgttccag gtgcgatttt catcggaatg
64081 attgctgcct caatctttgg catttgtact ggtcaaattg ccgtaccaca caagtttatt
64141 tctttagcac caagtcttgc acctactttt ggtcaagcag ttttccacgt taaagacatt
64201 aattctctgc aaatgtgggt tgttgtttta actttcttac ttgttacatt ctttgatact
64261 gctggtactt taattggtct tgctcaacaa gctggcttta tgaagaacaa caaaatgcca

64321 cgtgttggta aagcattagc cgcagattca actgctatga tgtttggttc tatctttggt
64381 acttcacctg ttggagcctt tgttgaatca agtgctggta ttgcagttgg tggacgttca
64441 ggtttaactg cagtctttgt tgccatcttc ttcctgattt caatgatctt tagtccatta
64501 cttggggtgt tcactactca agtgactgcg ccagccctaa ttattgttgg agttttaatg
64561 gcacaaaaca ctgctcacat tcactggaac aagttagaaa ttgccgttcc tgcatttttg
64621 attttacttg gtatgccact tacctactca atttctgatg gtttagcatt agggatgatt
64681 acttacccaa tttgtatggt ttctgcaaag cgtggtaaag aagtcagccc aatgatgtgg
64741 gtactgtttg tcgtattcat catcttcttg tgggttttga attttaagta agctaaaatt
64801 cactaacaaa aataccgatt atcatccatc ttgataatcg gtatttttta ttggcgtgat
64861 ttttataaag tatacttaac aaaattacgt attttcgtaa agtatacttt acagcatcta
64921 taattaagcc gctcatagta taatataagt ataaaattaa cctttatttt ccccactcaa
64981 ctgtttgaca attattataa caacgacagg aggaataaaa atgatcatta ctggtaagac
65041 tatttttaag atagtctata tacttagcat aatatttagc attacttata tcgtctggaa
65101 cactctccag cataatccat tagatccaac ttacctttta gttaccgtca tcagtatcgt
65161 agcgatgact ttagttttta taaaaattaa caaagaagaa taaattcctt ccataacaaa
65221 gcccgtgcat cgcacgagct ttttaagtac cacttctcat ccagccgcaa aatactcaat
65281 attcatactt taaatacaaa attcgctagt ttagttagtc ttttttgcct gttcgtcttc
65341 ttttacaaaa aagctttcgc tataattatt ttcacagcaa gttttatgag cggcggttgt
65401 ttttcctctc ggaggtgagg cctatggtca atatactaca gaaagggttc aaccatgtcg
65461 gtttacgaaa gcatctcttt gatgcttttg tttggtacat ttgtgttagc tttgctaacc
65521 tatatagacc accacaaaaa ataaaaaacgc cgctctacct ataactttgg cgagttatag
65581 agcgtcgttt tcgtcatctt aacattaaaa gccaccgctc ttaaagcggg ctgtataagg
65641 agatccgcgg atctcctttt ttcatacttg attatatcat agtatttatt aataaatatc
65701 atttttagat ttgtacattt taccctaatc tctacaaatt tctaggcaat atgatctctt
65761 ttcacatttt ggacaagtta attttctaaa cttaggtgta tgcggggcta agttaaaaat
65821 taaaaatgac ggaataaaga catccccgca attaggacaa acatattcaa ctcggtcgta
65881 atagtatttt gaaaacatta ttgcccccag cacagcaatc acaattgcta tacttgcatt

65941 taatcgcaac gcatacatga gactaatgcc tatcagttca actactatcc acataattat
66001 atacagccac atttttcttc ttaaaactgt cattttgttt tcttttctca taatacttgt
66061 aatgtcagaa aaattggtta atgcggcaga taaaacacct attttttgta aatgttctat
66121 ctgtttttgc tttgcagtca attcgttgat tttttgtttg ttttcttgat actgctgctt
66181 taaaagcaac tcaagcgatt tacttccgcg ctcatcttga atgagcgttt ttatttgttt
66241 caacgaaaag ccaagatctt ttagaaacaa aattaattct atctgcctta tctgttcttt
66301 ttcataaaaa cgatagccat tctcttccgt ataagccggc ttaacaagcc ctttttcatc
66361 atagaatctt agagtacgcg tacttacttc cagcatcgca gctacttcac caattgaata
66421 tttttcttgc attactttca cctcctagct tcaagatatc aattaacgca aggtcaaagt
66481 caatatttaa attataaaat attatatttc ccgggccata atgttcggat tctgccaaaa
66541 atatgctatt ttaggtacta ttgtctgcct ctagattagt ttaatcgaat aattttgact
66601 attattcttg aaatagttaa tattttagat ataattttag ttgaagattc gcttttagat
66661 gactaagaac acaagtaaat cattttaaag cttctttttc tttaaaatac gaacattacc
66721 acatctggag ggttacatga attcaattgg taaatttttc catttaaatg aaaaccacac
66781 ttcatttaaa acagagtttt tagctggact gactactttt gtaagtatgt cttacatttt
66841 gtttgttaac ccagctgttt taggagcaag tggcatgaac aagggagcgc tgtttactgc
66901 aacagcctta tctgctgctt tcacgtgtat tgtaatggga ctagttgcca attaccctat
66961 tgcttctgca ccaaccttgg gacttaacgc attctttact tacaccgtct gcttaggaat
67021 gcacgttaaa tggcaaactg ctttagctgc tgtttttgtt gcttcaattc ttttcatctt
67081 actaactgta tttaaagtac gtgagatgat tattgatgct attcctagtg atattaaata
67141 tgcaatctca gctggtattg gtttatttat tgcctttatc ggtcttcaag gcggtaaatt
67201 aattagcaag agtgattcca ctttagttac tgttggtgct ttgaacaatc cacttgtttg
67261 gattacaatt tttggtttag ttgttactat tttcttaatg atcgccagag tccctggtgc
67321 catctttatc ggaatgatcg tggctgctgt ctttggcatc gccatcggtc aaattccaat
67381 gcctaaggcc tttatttcag gagttccaag tctttcacca atttttggtc aagctgtttt
67441 ccatattagt gatattaata ctgtccaaat gtggatcgtt gtcttcactt tcttactggt
67501 tactttcttt gatactactg gtactttaat cggacttgtt caacaagcag gcctcatgaa

67561 agacaacaaa atgccacgcg ccggtgaagc tctagcagct gactcatctg gtatgttagt
67621 tggttctgtt cttggtactt caccagttgg tgcctttgtt gaatcaagtg ccggtatcgc
67681 agttggtggt agaactggtt taactgctgt ctgggttggt attttcttct taatttcaac
67741 tatcttcagc ccaatcttaa gtgtctttac tactcaagta actgctcctg ctttaattat
67801 tgttggggta ttaatggctg aaaacttagc acacgttcac tggactgatc ttgaaattgc
67861 aattccatgt ttcttaatcg cacttggcat gcctcttacc tactcgattt cagatggtct
67921 tggctggggc ttaattattt acccagtatc aatgcttgct gctaagagat tcaaagaaat
67981 cacgccaatg atgtggattt tattctttgt cttcgtcatt tacttcgtag ttctaaatgt
68041 gaaataggat taggataatg aaaaagctag ttagagacaa aatacctgaa ttcgctacct
68101 atgcatctta tcgtcaacta aaacctgatg aaagagaaga tgccttgaaa aataaaatcg
68161 tcgaagaagc taatgaagtt aaagctgcac ctgacgatca aaatctcctt gaagaattag
68221 ctgatgttta cacagtatta gaagcatttt tagattttaa aaatattagt aaagaagact
68281 tactaaagca agtagaagca aagaaagctg aaaaaggtgg ctttaccaaa ttccttttga
68341 tgaatactga taaataatat ctccttaaac aagtagttca catgactact tgttttctat
68401 ttatctccat ttgcgtatac gataaatttc tgctagaata cttaacaatc aagttatcta
68461 tatatcgtcg aaataaggtt gacagtatct accctgagcc ataaattcag gactataggt
68521 atcagacgtc ataaatttgc tttaaaggac atcttttacc catggctttt ttgggtaaac
68581 tgtgtccttt tttcattttt attttatgtt ggagaaatga tgcaattttt agataaagtc
68641 tttcatttgg aggatgcaca tactaatgtc aaacgcgaat taatcgctgc tctcaccacg
68701 tttgtttcat tgtcatacat cctttttgtt aaccctaata ttttaagtgc agctggtatt
68761 aataaaggcg ctgctttcac tgttactgca atcgcaactg caattggttg tttcttaatg
68821 ggattcattg ctaactaccc aattgcctta gcaccaactc ttggtagtgc cgcctttttc
68881 tcatataacg tagttattgg aatgcatatc aattggcaaa ccgctttagc tagtgtgtta
68941 gttgcttcaa ttttgtttat tttaattacc gttttaaaat tacgtgaaaa agttgttgat
69001 gctattcctc aggacttaaa atatgcaatc tctgctggta ttggcctatt cattgctttt
69061 atcggcttac aaaatggtaa attaatcgtt gataataaat cttctctagt tggtttagga
69121 tcattcaata acccagctgt ttggattacc ttatttggtt taattttaac tgtcatctta

69181 atggcagcca aagttcctgg ttcaatcttc atcggaatga tcgtgactgc tatctttgga
69241 atggtaattg gtcaaattcc aatgccacat ggtattgttt caggtgctcc aagtattgcg
69301 cctacttttg gtcaagcagt ctttcattta aaagacatta atactccgca actatttatg
69361 gttgttttaa cattcttatt agttactttc tttgatactg ctggtacttt aattgggatg
69421 acgcaacaag ccgggatggt tgacaaaaac ggtaaaattc cacgtattgg taaagccttc
69481 ttgtcagatt cacttgctat ggttgaaggt tctgttcttg gtactgctcc acttggtact
69541 tctgttgaat caagtgcagg tatcgcaatg ggtggtagaa ctggtttaac tgcaattttc
69601 gttggtatct tattcttaat ttcaatgatt ttcagtcctc ttctagcagt aattccaact
69661 actgtaactg ctccagcttt aattatcgtt ggggtattaa tggcaggaaa ccttaaatac
69721 attcactggg ataactttga aatcgccttt ccatcattct tagttgttgt tggtatgcca
69781 ttaacatact caatctctga tggtttagct ttaggaatga ttgcttaccc aattacgatg
69841 attgcttcta agcgctacaa agaagtatca ccaatgatgt atattctatt tgtaatattc
69901 atcatcttct tccttattac aaatatggga taaaaataaa aatgagatcg tacgcgatct
69961 catttttat taccttggaa ataattcact agctttaccg taatatttat ttcataatgt
70021 tagatatatt tactagtacg acaagctagt caatcgagga gtatttggaa taatgtcagt
70081 taaacctatc attcatgatg aactttcttt aaaattcaaa tctacgctag ccaccaagca
70141 ggatctagca gcagctgaag atttgaaaga caccttaatt gctaataatg gcaaagctgc
70201 tggacttgca gcgaatatga tcggtgtaca aaagcgaatt attgctttat ttgttggtcc
70261 acttccaatt gtgatgctta atccaattat tgttaaaaaa actgataagt atttagctta
70321 tgagggatgt ctttctttag aaggagaacg tccgacagaa agatataaga aaattacagt
70381 gaaataccaa aatgaaaact ttgaaactcg tcagcaagaa ttttctgatt tcgtcgctga
70441 agtaattcaa catgaagttg atcattgtaa tggtattttg atttagaaaa aatgtcagta
70501 gcgggggat gctactgaca ttttcttttt attcttaata ctacagatta tttagcagct
70561 ttttcttttt catagtttat gttgtctact tttctaataa atgggaagta aatcaaacat
70621 gaaatgatta tatttgctac ttgagctaaa gctaatttcc atccacctaa aatgaaacca
70681 gaaattatag gtggagtagt ccaaggtact tgaactccac taaacagtgg caaaactcct
70741 gctgacatca ggctataatc taacaaacca attacaactg gcgttaaaac aaatgggata

70801 aataagaaag gattcataac aataggtgta ccataaatta ctggctcatt aatgttaaat
70861 atggagggta caatagccaa tctacctaat tgtttagttt gctgcgactt agcaaagaaa
70921 aacatataaa ttactaatcc aattgtagct cctgtcccag tcatattaat caaaattgcc
70981 gtaaattgat cagtaaatac atgagctcca tttgcagtag ttaatgcttt tccagcatct
71041 aagatttttt ggttagcagt taaattagct aataagattg ggtaaaaaat actagtaaca
71101 attgttccgc catggacacc aaaccaccaa aagaaagtag ccacaaaaca atatgcaagt
71161 actccccaaa tagaatcagt cattccttgc aaaggagttt gaattatctt gtatactccc
71221 tcaataaacg ttgtattcca tacaaagtga attaaagcat agattaaaaa cgttactgat
71281 ataatcactg ctgctggaat aagtgcagca aatgaatttg atactcctga tggaactgta
71341 tcaggcattt ttactgtcca cttgtgcttt atcatatatg aataagacca gccaactaat
71401 agaccaacaa tgattgcagc aatcattcct tgaccactag tccaatcctt aggaatcaca
71461 tttgtaattg taaattttcc attaggcgtc actgcactag attttgttaa tactaaaaat
71521 gctgataagg caataactgc ggatgcaaat ggttcaactt tttctatttt tgcatactga
71581 taactaacac ccaaaactgc aacaatcgca ataattgaga acgtagctcc attaatctga
71641 tttaatggat ctatccaacc tgttttcgta agaaaatcag taacgggctg ataagggaag
71701 ttagctatta gtgtaaataa agagcctatc attaataatg gtaatgtata taaaattcca
71761 tcttttaatg ctcgtattgc tttcaagtca acaaatttta aaactggtgg tacaagatta
71821 gcattgaccc actttgtaaa accattattc attttacatt cctccaattt atatcttttc
71881 accgtttgaa tcgatcacgt ctttatacca ataaaatgaa tctttcttta gtctattaaa
71941 tgatccctct cctttatcat tactatctac atatacaaat ccataacgtt tagacatttg
72001 gccagtgccc attgacacta aatctatgca tccccatggt gtatatccca taatatcgat
72061 accatctttc tctacggcat cacgcatagc caaaacatgt tctctcaaat attctattct
72121 gtaattatca tgtattgctt tattttttgt gacactatca tgcataccta taccattctc
72181 tactacaaat aatggcttat gataatgatc ttctagccaa tttagtgaat atctaaaacc
72241 taatgcatca atgggccatc cccactcact cttctttaaa tatggattct ccacattaag
72301 ttcagatcct acaaatttaa aattcaaatt atttgatttt gcttttatag tattggaatt
72361 ataataactt agcccaatgt aatctacttg tccatctatt aagcttaatt tatcttcctc

72421 agttatatct tttctaaaat tattttgttt taaataagct tcaatgtgat taggatattt
72481 tccaaagact tgcacatctg caaaccagta tcttcgttgc atagcttttt gcgcttgaaa
72541 tatatcttct ggtttgcagg aaaaaggata ttgtggggtc atatttatca tgcatcctat
72601 cttattattt gaatccaccc catgtcctat ttgtactgcc ttggcacttg caaccaactc
72661 gtaatgagct gcttggtaca ttttattctc tctttctttt tctgaaagtc ctttaattaa
72721 aatacccgaa tcagccagca tacttaattc actgttataa tcagtttgat tatttatttc
72781 attaaatgtc atccaatatt taactaatcc tttataaaag aaaaaacagg tttctgcgaa
72841 atgtatataa aaatctataa cttttctatt gcaccaacct ccatattttt tcaccagatt
72901 taaaggcatt tcaaagtgag aaagtgttac tactggttct attccatatt tcttacattc
72961 tttaaaaact tttttataaa attctagacc tttataattc ggttctttat catctccatt
73021 aggaaaaatt cgtgtccaag ctatagaaag tctaaagcat ttaaaatgca tctcactaag
73081 tagctcaata tcttctttat aatgatgata gaaatcacta gcttgatgca taggataata
73141 cttatttggt tctaattcag aagtgatttc tctgggatta tgtacattac cactagtaac
73201 tacgtcggca atacttatac cttttccatc ttcattccat gctccttcta tttggttagc
73261 agctacagct ccacccccata aaaaatcctt tttaaaaggc ataattaaca tacttccttc
73321 catcaaaacc taacttcttg atcattaacc ttaacgttgt agcttaaatc ctttaagtta
73381 accgtagctt ctgccgtgcg tgattggtcc ttattacttc ttcgaattac aatcacattt
73441 tcactaggcg tctcaacttc aattgaccca tctaaatcaa aggctgcttc ttcatttcta
73501 aacctaagca acttaatcaa cgattgaaca accggtctct taacttcctt ggcaatttct
73561 tctttagaat aataatgcct attaatattt cgaccttctt tagtctcctc aagtagtttc
73621 aaatcattct taccagctaa caatcccaca taatatattt gtggtattcc tggtgcaaaa
73681 acttgaagta agcgagctaa gaagtacttt ttatcatcgt ttccaagggc cgaataatat
73741 gttgtgttga tttgataaat atccaaattg tgatactcag cactcgaata ttttcggctt
73801 acattggctc caaccttgta cagctcatta gtcgtatact ctaattcatc atcagtcaaa
73861 acatctttgg catctactac tccaatccca tcatgtgtat ccaaagtagt aaattgcttc
73921 attgggcatt ttctaagcca ggcggccaaa cgatctgact ttccgctata gagcgaataa
73981 agcaaaacca ttggcaaagc aaaatcataa atataatatc catgctttgc taccttcatt

74041 ggcatcgtat agtgctcatg aatctcaggt aaaatcttag cgtctgttcc tttaagatca
74101 ttttttacct ggtctagcag ttcccaaatt tcaggttcta caaagaaatc attcgtatct
74161 aactttttaa tcgcatacgc aaaagcatct aacctaatca aacttgcttt gtgcttagcc
74221 aaagttcgta ctgaatcttt gataaactct tgcgttactt ttgttctaac atctaagtca
74281 atttgctcat ccccaaaagt attccataat ttcgacttgc ttccatctgc aaaagtaatc
74341 tcttgatatg gagccttgtc ctttctttta tagatctgat caatatcagc cttagtcggt
74401 cttccttttg gccagaactt attccaatct aaaaacagat ctttatattt actattttca
74461 ccgttcttct gaaaatcttg atagtactta gaatgcttag aaatatgatt aatcataaaa
74521 tcaagcatta aataatactt ttcaccaagt ttttcaaaat ctttccaagt gccaaacttg
74581 ggatctattt catcatatct agttggtgaa aatcctcgat ctcctgtcga aggaaaaatc
74641 ggcaaaatgt ggattccacc aatagcgcct ttaaaatgtt gctccatcac ttctgataga
74701 tcatttaaat tatttcccag gctatcagca taggtgatta acatcgtttt attttgcagt
74761 ttcaatcttt tattcccctc gctttttatt attaagcgct tacatttatt acacttatag
74821 aatatacata atttgtcttc ttgtagcagt tttcattttt ttacattatt actttctaag
74881 agcttataat ataagtgtat ttcactatgt aaattttcat atgagggaat aaatatgaga
74941 gtatcaatta ccgatgttgc taaaaaagca caagtatcaa ttgcgacagt ttcacgcgtt
75001 atcaatcaaa acgattatgt tgcaccagaa actagaaaaa tcgttgaagc agctattaaa
75061 aagaccggat atcacttacc ccagcatcat ctagctaatc atacaaaaaa caaaataatc
75121 gaggtcatcc ttccatctgt tactaatcca ctatatgcaa agttttatca agaaattgag
75181 cgaaatttag ctgccgaaaa ttattccagt ttattaaaaa tagacaatga tccgacacat
75241 acgttagagc catatttgac agccttaaaa aagggtaaaa tagctggaat tattacttct
75301 tcaaatcttc caatttccga tcaaaatgct agcgacttat tgccaatcgt ttcattcgac
75361 cgacatttaa gtaaagttac tgtcctcaaa tgcaataact tagatggtgg ctataaaata
75421 gcccaaaaag ttttaagttt agggaagaaa aatatcttaa ttttatctgg gaatcgggct
75481 gatttatatc ctcttaatga tcggcttaaa gggatgcttt cggtatttaa tcattataaa
75541 gttaatgtaa aaacaacata tattgatttc gagagttcaa tgatggttaa aaaaatccaa
75601 attgcacaaa ttatcgactc accaaaattt gatgcaattt gttgtactga tgacgttact

75661 gccttattag tcaaacaaca tacggatgaa atgaactatc atcccttaat tactggtttt
75721 gatggaacag attttattca tagtttcttt ccagaattaa tcactgttaa acaaccaatt
75781 gaagatatca caaaactaat gtgcgaaatt ttaattaaga aaattcatca tcctagcaaa
75841 cattttgagc cagagtatat ttttccagta acattattag attaaattgt tcacaaaaaa
75901 ggtagcacct tgattaaaaa atcgaagata ctaccttttt tataattcac gattcgttgg
75961 accacgttca attttaattc taacaatcaa atgaactaag ttataaagca gagtaaacgc
76021 tagtaaaatc aaccaaaaat accaaaatgc ataagcaatt aacaaaatag ctggaaaaag
76081 gcctaaaaaa actgcaatta aactatcagt tagtttcagt ttcttattaa aaattccact
76141 tctcactaat tgatatgact taaagcccaa gtatgtagct ccagctaatc caaatagtcg
76201 caataacaaa tcaacattcc aaaagacaca taccatgctt aatccgatgt acaagatccc
76261 gattacaacc agtatgattc gtcctaacct cgtaatagca tggctttctt ctaagccttg
76321 ttctatatca acaagataat aaataatcgc acataatcca actagcctat atagccaacc
76381 taaattcctt aacgctaaaa atatcacaat aacgcttaga ataattccgg ccacaccaag
76441 aagacggttc aattttctat tcttaacaat taagtctggc cgcatccaat cattcctatc
76501 taatcctata gctgtcggtt agtcccatca tgttcaacct taattctgac aatcaagtgc
76561 agtaaattat atgccaaagc aaagatcaac aggaacaacc agagaatatc tcttggagta
76621 aacgccacaa ttaaagaagc tactactatt cctaaaaaag ttgctatcat gctatcagca
76681 aacttcagat ttttattttt cagatcttta cgcaaaactg cattagtctt aaagcctaaa
76741 tatcctgcac ttataatgcc aaatgctaca agcaaataat ctagattaac aaaagcaaaa
76801 ataatgctaa ctagttcata cattgtaccc agaacaatga aaacgatttt gaccgtacga
76861 ctactaatcg catcgctatc ttccagtatc tgttcaacat caatccaaaa ataaagaata
76921 actgccacca caataaacgt gagtagccag ccaatgtttt ttacagtgaa aaagaccatc
76981 ataactacaa tcagcaaact gataaatcca aaatacttat cttgcttttt tctttttgct
77041 aattcaatcc atcttttatc gtccatctac tcaccttccc gttttagttg tctagatttt
77101 actatgaaat atttgcttta acaatatttt aatcatattt ttatacaaat tgtgtagttt
77161 tcattgttga caagtgtaaa cataaagata tatgattaca agtgtaaacg aaaggaaagt
77221 aaaaatgaac gaaaagaatt tatctcctat ttcagacagt gaatgggaag taatgcgtat

77281 tgtctggaca ttaggtgaaa ctcatacaaa tcaaatccta aaagaacttc aagcaaaaaa
77341 gaattggtca gactctacta tcaaaacact aattagacgt ttagttcaga aaggctggtt
77401 aactgccaag cacgaaggac gacgttatac ctacactgct actgttagtc agacagatat
77461 gatgtacaac gaagctaaat ccttattaaa ccgaatgtgt gacatgcata agggcgaagt
77521 gatcttaaaa cttttagagg actcacctat ttctaaaggc gatttaatga agatgcaaga
77581 agaaattagt caaaaagaaa aaacagcacc ggacatggtt ccatgcaact gcttaaaaac
77641 cggctctact atttgctagg aggacttaaa aatgaatact agtcaaatca tcgtcttaat
77701 tatcggaata attttaattg gctttattgt ttggtggttc tttggcaaac ataaggaagc
77761 tacaggaagt ggaactattg ttaatgacga acaaactgcg acaattgtag ttaacggtgg
77821 atactctcct tcaacagtag ttctaaagaa agggatacct gcacaagtta attttgatat
77881 gcgcgattct actgcctgtt tatcccacgt tgtctttgaa caattaggcg ttaataaaga
77941 tctaactaaa caaaaaatca ctactgtcaa tattccaacc gataaagcta gcacttataa
78001 ctttgcctgc ggaatggaca tgtttcatgg aaaagtaatt gttaaataaa agaaaggaac
78061 tactaaaatg agtatttttt caaaaaaaca aactaaaaaa gtcgttgtta atgcagaaaa
78121 tcatggctac aagccagatg taattacttt taaagaaggt aaacctgcac aagttaagtt
78181 catcccttca gataacatgg gttgcatgaa tgagattgtc ttcaaagaac ttgggattga
78241 taaagtcctt gatggtaaaa atgaaataat agttgatatt ccaactgata agccaggcac
78301 ttacaatttt gcctgcggaa tggatatgtt ccatggaaag gtcgtcgtta aataatgaaa
78361 ctatcgaata ttaaacgctt ctggatctca ttcatcctat ctattccaat gctcattcag
78421 atgtttgcaa tgccattcca ttggatgatg ccaggatata actggattgc gcttattaca
78481 acaacaatca ttatggcgat ttctgctctc ccttattgga aaagtgccat tgctgctttt
78541 aaaaagcata gtgctaatat gaatacctta gttgctacag gtacagcagt tgcttacttc
78601 tacagtatct tcgcaatgat tactaaccgt cctgtctact ttgaaagtgc cgcctttgtt
78661 actgtcttcg ttcttcttgg agacgctatg gaagaaaaaa tgcacgacaa cgcatcaaat
78721 gccttaggaa aattaatggg cttacaggca aaagatgctg aagttcaaag agatggcaag
78781 ttcgttaaaa ttccacttga tcaagttaaa gttggcgaca ttatccgcgt taagccgggc
78841 gaaaagattc cagttgatgg tgaaatctta gaaggtgtta caacgcttga cgaatcaatg

78901 gttactggtg aaagtatgcc agttgtcaaa aaagttggtg acaccgttgt aggttcaact
78961 attaacagta acggtaccat tactttcaaa gccactaaag ttggctccga tacaatgctc
79021 gctcaaatag ttgacttagt taaaaaagct caaactagtc acgctccaat tcaaaaccta
79081 acagataaga tatccaatat ctttgtccca gccgtaatga ttattgctat tttaactttc
79141 attatctggt attctttctt gggtgcaact gctgtagaag caatgctctt tgccgttagc
79201 gttattgtta ttgcctgccc atgtgcttta ggtttagcta ctccaaccgc cttaatggtc
79261 ggaactgctc gtagcgccaa aatgggtgtt ttaatcaaaa acggtgaagt attacaagaa
79321 gttagcgact tagatacagt cgtctttgat aaaactggta ctattactgt cggtaagccc
79381 gaggtaaccg atgttgtagg taatcaaaaa caagttttaa ccattgctgc tagcctagaa
79441 gaatcttctg aacacccgct tgctacagca attgtcaaaa aagcgaaagc ggaaaaatta
79501 acaattgaaa aagttgatga cttcgaagca attgaaggta aaggcgttaa ggctaactac
79561 aatggacaaa cagcctttgt tggtagtgat cgcttactag cagacgttaa tatttcccag
79621 gaaatgacac aaaaagcaac agatttacaa aatgaagcta aaacggttgt ctatattggt
79681 ttagatggag aaattattgg cttagtggcc attcaagatg taccaaagcc aagttcacgt
79741 gaagctatca aagagctaag agaacgcggc ttaaagacca ttatgcttac aggagacaat
79801 aaaaatgtag ctgaagctat tgctaaacaa gtaggtattg accaagtgat tgctggagtt
79861 ttacctaatg aaaaagcaat gcacattaag aaattacaag atcaaggtaa aaaagttgcc
79921 tttgtaggtg acggaattaa tgatgctcca gcactctcga ctgctgatgt tggaattgct
79981 atgggatcag gaactgatat tgccattgat tctggtggta ttgtcttagt tcaaaacgac
80041 ttgcgtggcg tagttcgcgc ccttgatatt tctaagaaga catttaacag aattaagttg
80101 aacctcttct gggctttaat ttacaacaca attggtattc caattgcagc tggattattt
80161 gttggcttag gatttactct tagtcctgaa ttagccggct tagctatggc attttcgtcc
80221 gtttctgtag ttggtagctc attaatgctt aataaaacaa aaattgctgg tgaaacagct
80281 aaagcataat tacataatca aaaagaccta gtatcaattt ataaatacta ggtctttttt
80341 tgtctattgt tctttatatt tatcaaaaaa ttttatcatc tctttgccaa aagctgacat
80401 cccaccccat tcaaaacttt ctaaaatcaa attacactgt tctaaatgtg tttcctttct
80461 atcagtatga taagcaatca attcttcata aaaagtaagc atttcacgat aaagaaaaaa

80521 ttcaggaata actttaattt gatggcctag actgataagt tcatttgttt cattatattt
80581 ctgctcttca attccctgct ttaatacatt cagtataatc gcaaataaaa tttcgtcttg
80641 tctttcgact ttcttctttt ttaatgcttt aacaatctgc ttaattattt gccgattact
80701 gtcaaaatca tacaaatcca caaaattcgc atagactgat aatttatagg tatcatattc
80761 tggcatcagg aatatctttt cttttagctt atttcttaaa ttaaagtcaa aagtatcggg
80821 atcgtccttt tctgattcta aaacaccatc aattatcagc atcatgtttt tcttcatatc
80881 agcttcgaca tctaatttag cagctttttc cttaattttt ttaatttgtg ctaacgaatt
80941 atccgaataa ttcgctttgg ttatttcttc aaaaatggca ttcacttgat ttaattgttg
81001 ctgatgtgga tcaatttcta gtttttcaaa aaaattttta actgggattg cattatgcgt
81061 caggagcaac aataaatcat cagcagtaat tcggtggaca ttcttttcaa ctttagaata
81121 gtaagactga ctaacaatct cagctgcaaa ctctttctga cttttatttt gttttacgcg
81181 atattcttta agtaattcac caacagtcat tttttcacct caaagtaatt tgaatacgac
81241 tattctagaa atgattttac tttcttgata ttattaaatc aacattaaat ttaaaggagt
81301 aatcagttat gaaaagcttt aaaattcgtg gcggacttgg cgactcagct gtagtaaacc
81361 ctgacgttaa attacaagac aacttatact tagctattaa ttcaaaatgg cttgaacatg
81421 cagaaattcc agctgaccgt cccatgattg gtggcttcac agagttagac atcaagatta
81481 aaaagcaact gttacaagat tttgataact ttgcttctaa cgacaaagca cttcctaaga
81541 ttgcaaatct agataaagct gttcggcttt atcagcttgc actggacttt aagcaaagag
81601 acgcagatgg agctagacct atccaagcag acttacaaaa actcacttct ttaaagaatt
81661 tttcagattt tgataaacat gctgttaatt tagttaaggg tgcacttgct tttcctttca
81721 ctattaacac tgatgttgat atgaaaaata cagctaagaa tgcactctat tttgactcac
81781 cagctacatt cttacctgat gctggaaact atcaagactc atctgctgat gagttattag
81841 ctatttttga aaaacaatca gtagaccttt taaaactagc tggctttaat gaaaatgata
81901 gtaaaaaata tgccgctaat gcgattaaat tcgataaact cttagccaaa tataaaaaat
81961 cacaagaaga attaaacgat attgctggta tttataatcc ctactcactc tctgacttta
82021 aagcagcatt ttctaacttg aatattgagc aattacttaa tgacctactt cctaatttac
82081 ctgaaaaggt aattgtaatt gaacctaaat ttttacagca cattaataaa cttctcaatc

82141 ccgataactt tgaggaactg aagagttggc ttgtagttac cttcattaat
caatatgcta
82201 cctacctttc tcaagaattt cgtgaagcag cttttactta tgatcatgca
gtatacggag
82261 caaaagaatt aaaaagtcaa gcccagcatg cttatgggat tgctaataat
atcctaggcg
82321 aagtcattgg tctctactat ggtcaaactt actttggacc aaaagctaaa
gcagacgtgg
82381 aacaaatgat tagaaaaatt cttgcaactt acaaagcaag acttcagact
aacgactggc
82441 ttacaccatt tacacaaaag caagctctaa aaaaactcaa tgcaattaca
ttaaaaattg
82501 gttatcctga taaacttagc gacctatatg atcaaattca agtatcatca
gataaatcat
82561 tatatgaaaa tattattgct gctaatcaaa ccaaaattat tcataatttt
aaccaattaa
82621 atgaacccat tgatcgaact ctttggggag ctgatcttcc agcaaatgag
atcaatgcag
82681 gttataatcc aacaaataat gatattactt tccctgcagg aatcttacaa
aagccttttt
82741 acagtctaga taatacacta agcgaaaatc ttggcggcat tggagctgtt
attggtcatg
82801 aaattactca tgctttcgat ccaaatggct ctaaatttga cgaagtcggt
aatattcgta
82861 attggtggag tgacgaggat tatgctaagt ttaatgaatt gtctcaatct
gaagtcactt
82921 tatttgatgg tatcatctgt ggcggaatta aaacaaatgg taatctaact
gtcggtgaaa
82981 atgttgctga tttaggtggc cttacagctg cagtagaaac tgcaaaaaaa
gaaaatggta
83041 atttgcaaga agtctttcaa agttgggcta aaatttggcg tggtaaaatg
acgcctcaag
83101 ttagaaaaac gcttattgcc tacgacccac atgcaccacg agaacttagg
acaaatgtta
83161 cggctcaatc tattgatgac ttttatacag cttttgatat taagcctggc
gatggcatgt
83221 ggcttgatcc tgaaaaacga gtacatattt ggtaattaaa agttttcatc
ataaagtaag
83281 tttttgtaag aagtctgata aatttacttt ataaagtaaa ttttgataaa
attaaggtaa
83341 aatttacttt acgatgtaaa gatatgaaac ctgagttaat taacagaccg
caatatttaa
83401 acagactaat tgattttatt gctacaaaag attgtaatta ctggtcgcta
tgaagacgat
83461 acaataattg atggtattcc tattattaac atcaaagatt ggttactaaa
ttaaaaacgc
83521 aaaaaagact gaatctagag atatcaaaat ctcgatccag tctttttag
ttagtctaat
83581 ttttcaccca ttgcttgttc acgcttagct tcacgttttc taagccatgg
taaaacgaat
83641 cctaaaccaa acaatactac aaccgtaata atgttcatcc ataattggtg
agtaaatgcc
83701 ttagtaccta ctgcaacgtt ttgaggaata aatcctaaag ttgcacagac
aaaggtaaag

83761 agtaagcacc aaccaccaac agttaaagca ccagccttat ttttaataaa gacataatct
83821 gagtgatact tatcttggtg cattctcatt gccacaaagg caaagaatac ccaacaagtc
83881 ttatatggtg aaataattcc gttaacattc aaaagccagt tataaatggt attaatgtta
83941 ggtaaagttc cagttaataa tagcaagaat aaactaagtc caacggtaaa tgtgtatgag
84001 tgaattggac ggccgttctt atttttctta gtaagccact taggcatgaa cttggagcca
84061 acatctccag caaagacacg acttgaagcg tctaataata cagcaagttg agccatcatg
84121 aagattgctt gtacaactgc aaagatatac attaacacct tacctacacc taaactttca
84181 ccaagcaatt ggaaagcgta gtaaggaccg ttcattttga agtcatgtgg aatgtggtgg
84241 gcattaaaga acattgctaa agctaatgtt ccaaaaatcg ttaagaatcc ggtcataatg
84301 gcaagcatcc acattgcctt aggaaagtcc ctcttcggat tcttcatttg ctgtacataa
84361 ggcgcagcaa gttcagcacc tgacatcgca aagatcaaaa gaccagtagt tgagaagtaa
84421 ttcaaactaa atgaaggctt aaatgcaccc cagttaaatg gctgagttgc aatatggtgt
84481 ccatgcatta ctgaccatgc agcaagcaag acaaagagca tagacataat aaacattgct
84541 cccccaccaa ttaaagaaag aatttctagt gaattacgaa gcttattttc tagcaaaata
84601 aagatcaaaa tgatcacaaa agttaagatt ccgaaccaga aagtcgacat cttcttgtcc
84661 aagctattgt taccaaagat catccagcta aaagaaacaa taactgagtt tgagacgtca
84721 acgatataag gcacactttg aacccagtac atccaagagg tccagtaacc taaagtgtcg
84781 tttgtacttc ttctgaccca agaagcaagc ccaccatctt gattatcaaa agtcaaactc
84841 atctgactac taatcaatgc gtaaggaata acataagtaa ataaaagaaa gatccaagaa
84901 attacaactg aaaggccttg gttttgaaat ggatagaaga tattttcaaa actaataatc
84961 gttacaaagt caatcaatgc aataacaggc caactcatat agtgcttttt attcggttct
85021 aaatcattta agacatctgg tttatccaat tttcaatttc ctccaaaatt aaaatattta
85081 tctaaatcca aggtcttaaa tttggaggct tcaaaactat cattaaagta ttcaaaatca
85141 cttgtagaaa tcctttataa atcgttataa tttatgccgt agtcttcttg caaataaaat
85201 tgcgtacaaa aacgttcgca caaaattata aggatttata aaaattttgc aagtctattt
85261 tagaaaatac agtaggaaaa caaggtgatt aaatgaattt tctaaaaatt gcattaagca
85321 ataaacttaa aaataatgat tttaattcat ggcaaactac taatttgaat aataatcctc

85381 aagctagtga acttgccgct attgttttag cagagggcga ccaaaatagc
cttaaaactg
85441 ctcaagatct acaaaaaact tctggccttg gaattcctat tattaaagtt
tcacatgaaa
85501 caatttctaa taatgaaaaa gaaaaaataa ttgatgcagc aaataaatat
acgaaaaaaa
85561 tggtgccagg ctttttaact gacctagtta attttgccga agatcatcca
gtaagcttca
85621 ctacgcctgg ccaccacaac ggcctctatt atgaaaagca tcccgctggt
gttgtcttca
85681 accgcttctt cggtaagaac ctaatgtttg cggatactag tgacactgtg
cccgaactag
85741 gcgacactat gacgcacgag ggaacaccct taactgctga acaaaaagct
gctgaaactt
85801 atcacgctga taaagtttat ttttgtacta atggtacaac tagtgctaac
tcaatctgtg
85861 ctagcgcact tcttacaaaa gatgacctag ttctttttga ccgcaacaac
cataaatcac
85921 tttataacag tgccctagtt atgagtggag ctaaaccagt atacattcca
actgaccgaa
85981 atgcacttgg tctaattgga gaaatggatc ctaacttttt aagtgaagaa
aaaattagag
86041 cagaagtcgc taaagttgat cctgaaaagg ctaaagctaa acgtccattt
agattagcaa
86101 ttattcaatc agaaacttat gatggacttt tctacgatgc taaatggatg
attgacaaga
86161 ttggcaagct ttgcgactac attttattcg attgtgcttg gggtggcttt
gaacaattcg
86221 tgccaattat gaatcactta tctcccctca atttggattt tgggccagac
gatccaggga
86281 tcttagtaac gcaatcactc cataagcaac aagcaggaat gggacaagct
tcccaaattt
86341 tgaaaaaaga tgctcacata aaaggtcaaa aacgctatgt tgatcataag
cactttaatc
86401 atgcttatct taaattcgtc acttccagct attcatatcc cctctatgct
tccttaacag
86461 ttaactcata cttaacaagt ggtgaaggca ataaaaaatg gtgggataaa
atattgcggc
86521 taggaattga atggcgtaaa gaactaatta gaaagtccaa attatttagc
ccactcgtaa
86581 ttgataattt tgaaaatatc agtactgatg acttagccac aaatgaaaaa
tactggaact
86641 tagaaccaaa taatctttgg catggcttta gtaaaattgc gaccggtcaa
gcaatgattg
86701 atccgttaaa gatcacagtt gtcactcctg gcattgatgt taaaaacgca
aaatatgaag
86761 ctgtaggaat tcctggtcca gtagtagcag agtttttgat ggagaagcga
attattcgcg
86821 caaaagatga tttatattca cttctcttct tacttacccc aggtgataca
aaagctgaat
86881 tagatacatt gctgaatgca tttttagaat ttgaacaata ctataatgaa
gatgcaccat
86941 tagaaaaagt attaccaaaa ttgactaaag tatacggcga tcgctacaag
ggttatactc

87001 taaagcaact ttgccaagaa atgcataatt attaccgggg aaataaaact
ttcactttgc
87061 aacaagaact atttgctaag ccaaatatgc aaaattaccc aatgacgcca
gaacatgctg
87121 actatctttt tatgaaaaat gaaagtgagc tagttaacct agatgatgtt
aagggcagaa
87181 ttgcggccga aggtgcactt ccttatccac ccggcgtctt tattgttgca
ccaggggaaa
87241 aatggagcga tattgatcaa aagtactttg aagttttagt aggagcaatt
gagcgcttcc
87301 ctggctttgt acctgaaatt caaggcgtct actgggatca aaaagcagat
ggtaaaataa
87361 gagtacaagc agaagtatta aaggagaatt aactaatggt tttatcttgg
aaagaaaact
87421 taccaacaga attaaaagaa aaagtcgata aagtcgacaa aatgattgaa
cctcgtttag
87481 aagaaattga cgagcaagtc ttttacaacc aacaacgtgt tttaggccta
tttagaaaac
87541 accgcgtagg agaagaagat ctagttcctt caactggcta tggttacgat
gatgttggtc
87601 gtgataaact tgaagcaatg tatgctgatt actttaaaac agacgacgct
ttggtccgcc
87661 cacaattttc ttcagcaaca catgcgattg ccgttggtct tttcagtatg
cttcgccctg
87721 gtgatactct ttactactta actggtatgc catatgacac aattcaagaa
gttattggct
87781 tagcaggcaa caaaccagga aatatgaaag aatggggcat tgacttcaag
catactgact
87841 tacttgataa tggcgaagta gactatgaac aagccagaaa agatttacaa
gatccaaaga
87901 ttaaagtcgt aactatccag cgttctcttg gttacgctgt acgtgcaagt
ttcaccatgg
87961 aaaaaatcaa gaaaatgctt aagtttatca aagaagttcg tccagacgta
aagatcttcg
88021 ttgataattg ctatggtgaa ttttctgaaa ctgaagaacc aactttctac
ggtgccgata
88081 tgatggcggg atctctattt aaaaatgctg gtgctggtat tgttaaagca
ggtgctttct
88141 tagttggtaa aaaagatcta attgaaggag caggatctcg ccttaatgtt
ccaggctctg
88201 gtaaaggtga aggcgctact tggggttatt taagagatat gtatcaaggc
ttctttatgg
88261 caccccacac tactggtgaa gccttaaaag gtatgattta taccgctgca
ctttgcgaag
88321 aaatggggat gaaagttgct cctaagtgga atgatccaag aactgatatt
gtccaaaccg
88381 taacttttgg tgaaccagat tcaatggtta aattctgtgc cgcaattcaa
cattattcac
88441 caatgaactc atttgttgat cctattccat accaccaaga tggttatgaa
gatgatgttg
88501 ttatggcttc tggtagcttt actgaaggtt ctacaatcga attatcttca
gacggtcctc
88561 ttcgtgcccc ttatcgcctt tacattcaag gtggtttatc ctatgctcat
gataagattg

88621 cgatcactca tgcagtagaa gaaactttct acaaaaataa ttaatcttaa tcaacatatc
88681 taaaacggtc agtgaaaaat cactgaccgt tttttatttt cccgacaaaa ttaatataaa
88741 atcattttct ctttggtttc ttctacaaat ccgcgactac cgtatagtct ttttgctctg
88801 atatttgtag aaaaaacttc taaaaatgct ggacgtttta ggtaaggaat tgcagtatct
88861 aaaagtttac ctcctatacc ctttccttga tattccctta acacgtaaat atagcctagt
88921 tcattcttcc taaaagcaac aaatcctact acatgactgt cgacttccgc tacataaatt
88981 gtttctgaaa atagttcttc tttgtatact gccctttcaa gtggagcata taaatcaact
89041 gcattaccca caattaattc ttgcattctc gcttcatcat gtactttaca taattgaaca
89101 tagtttgtat ttttatattt tctgataatt acttcttctg acataactaa cctaattttt
89161 atttgaaaca tcaggagaaa taaactcctg attaacgact aaattggcta gcttttctac
89221 gtaatctcat ttttttaccc gctttctgaa tttttagcaa tattagcaga taattaaatt
89281 agtgtcaata tttaagtaaa gattaatcta tctttgcatc aattaaaaat ctttgttagg
89341 gtggaatgta tactttaaat ttttaaggag gatttttttcg agttaatcta aatgatttaa
89401 ctcatgttat gaaaaaagaa aatcagaata atacaaataa tattaaagaa aaaaagaatt
89461 ataaaaaagc acctctcatg cctattcatt tacgagtatt tatggctgtc ttattaggtc
89521 aaatcgcctg tggctttgcc ttaggtattt ctggtacagc tctttctaac gctagtaaat
89581 atatccaaat taatgatctt tggactggat taattggtgc tggatcttta attggactag
89641 ctggcagtat tctaattggg cgtttatcag ataagattgg tagacggaaa ttattaatgc
89701 tcaatatgta tattttgggt actttatctc tactgcaatt actaactaat aatcttccca
89761 ctctttttat cattagaatt ttaattggat taatgattgc cgttgattat accgttggga
89821 acgctctttt aactgagtgg cttcctaaag gagaagacag caaaaggcaa agccacttgc
89881 ttatctactg gacaattgga tttattgctt catatataac aggtactttt attagcggct
89941 ttggctcaca tacttggcaa ataatcttag caactggggc cataccagct tttattgccg
90001 ctattttcag atctattttt cctctacctg cctccccaag ctggcttgca agccgtggta
90061 aagtcaaaac agctaataaa gttattaaaa agcatatggg acgtaaatgg ggcatcccta
90121 agaaactcaa aaaggctaag ccagtcaagg aagtttcctg gtcaatatta ttttccggga
90181 aatatttacg tcgtactcta gttggtggac tcttttatgc ttgccaagct tttgcattct

90241 ttggtatcag tatttttctt ccaattcttt tacaaagtat gcatgtcact gaccaaaagg
90301 tttctgggat tatctacaat ggtggtatgt ttatcggcgt cattttagga atcctacttt
90361 ttaacagaat tagtcgtcgc gcatttctca tcagcaattt tttactttca ggtattctaa
90421 tcggcttttt agctattgac aaaaacctta attcaaatat tcaacttact atcttttgta
90481 tttttgcaat tatcttatct tctggtttag tacttgatta cccatatcca actgaactat
90541 tcgatattaa gattcgtgga accggcgttg gaacttgtat tactataagc agatttggtg
90601 ccgcagcagg taccttttta ttgccaattt tgactaatca aggcggtgca agcttatcaa
90661 tgctaatctg cgcaattgtt cttttggctg cctttatcat ctgcttaatt tgggcacctg
90721 aaacctcacc aaaatttaag caaaataatt gattctgttc tacttttttc taaaataaaa
90781 atagaaagag tcaggaggga ttactatggt aaaaatgaca ataagccaag acgcaattaa
90841 ctttcttaaa aatcgtggtt atgataaaca tgaaataatt ttgattgttg atggtggtgg
90901 cggtgattat tcaattgaag gtggatcttg caatattggc atggattact ctctaatttc
90961 actagataca atgaaacatg attcacgcta ttcagtaaaa atagaaaata atgctgactt
91021 tgaaatttat acctccgatt atgatttagc ttttttaggc gataacttgg ttctagatat
91081 ccacgatact accttacgcc ttcgtaacga ttctggcatt ttaactggtg ctgttaagat
91141 tgctaaggca agctttcttg ccgagcaagc taaaaaaggt attataagtc aaaaaaattg
91201 ttaattaata gttttactct caaatataaa tttgagagtt tttatttacc ctaatttata
91261 atgatttttt agtaaaaatg ttatatcatt aaaatcaata atttagaaaa ggacatattg
91321 ctatatgatg ctaattgtat tttagtctca agggactaat ttactttccc ttgagaaaaa
91381 aattaaaatc tattttctta aggagaaaaa acaaaattga ataatactga cgaaatcgaa
91441 ttttcacgtg tacgtaaact tgtctctcac tatgctgttt cacccgaagc aaaagacaaa
91501 attctagttc agccacttgc cactaaatta gttaaagcaa ctgctttact tgatgagact
91561 gcagaaatgg tctggatttt aaatcatgga ttacacatac cctttattag ttcagatgcc
91621 ttgactccta ttttaaataa ggtaaaaaaa ggttttatct taaatccagc agaattagaa
91681 caagtagcag acttcattag agttacgcgt ctattagtgc gctttttttaa caagcaaaga
91741 aataatactc caattattgc aagttattgt gatactttaa ctattctaga cacactagaa
91801 agcgaaattt ataatgctat cgaacgcgga caagttgcag acaaagctga taaagatctt

91861 gctaaattga gacaaagacg tgctaaatta agtacgacta ttcaggatac gctacaaaaa
91921 tatttacaaa acaaaaaata tcaaacgatg ctgcaagatt atcagataat tcaaaaagat
91981 aatcactata ccctcccgat taaagcaagt tttaaacata cttttggtgg aagtattatc
92041 gatcattcta ataatgggaa tacagttttt atagaaccca ctaaaatcgt gcatcttacc
92101 caagaaaaag caactgttga aaatcagatt tatttaattg aatctcaaat tttaggtgat
92161 ttatcagcaa aaatttatca agaattatct caactagagg caaatcttaa tattattatc
92221 gacctagatt gtatcttagc tcgtgcaaaa tactctcaaa gtattaatgg taaacgacca
92281 ttgttaaatg aacaaaatca actccaatta aatgaaatga gacaccctct tttatctaat
92341 cctgttcctc tttcacttaa tttagattct tctaagcgtg gcttgataat cacaggtcct
92401 aatgcaggtg gaaagactgt cactctaaaa acagctggtt tggccattgc aatggcagaa
92461 tttggactat tcttaccaag ttcagggatt tgctcaattc cagtaatgac cgctatttat
92521 acttcaatcg gcgatcatca ggacttagat aactctctgt ctacattctc tgccgaaatg
92581 aagcaaatgt cttatattgt ccaacatgca acaaaataca gtctaatttt attagatgaa
92641 ttaggcagcg gtacagatcc aaatgaaggt gctgcccttg ctattgctct gctgcagaca
92701 ttacagctaa aaggcagtct aatcttagca accactcact atagtgcaat aaaagactat
92761 tctactaagc atcccacttt catcactgca gctatggact ttgatctaaa gacacttgag
92821 ccaacatata agttactgtt acatcaaata ggaaccagcc gtgctctatg gatagctgaa
92881 aagaatggga tgcctcaaga ggttttaacg caagccaata atttcttaaa tactggtaaa
92941 tttcctttaa atcaaagcaa aattaaattt gcaaataatc agaaaaaaat caaatcgaca
93001 gtttctttta aaaaaggtga tcgtcttaaa gtcccggagt acgaacaaga tgttttattc
93061 tatcaaaact cggatcttgc aaatcaaatc attatctttg ttaataaaac ttttaagaca
93121 ataccaatta agggagctaa gctagttaga aaggcaaaag atctttatcc agaaggttac
93181 aatcttgacc tattattcat acaagattgg aaagaatata aattaaacag agatttaaat
93241 cgtggctcta aaaaagcctg gaaaaaattg aaaaaaatag acaaacttag ataataatat
93301 gaacccagaa attcggacaa gaatttctgg gttttattta ccggctcctt ttactttgca
93361 attgtgataa tttttttatc gacttgaatt ttattttcct tagccaattc ttttaactta
93421 cgacttaaag tttctggcgt aatgcccaaa taaccagcta ggtctttttt cttaagatct

93481 agctttactt tatttgtacc ttgttttttt gacaaatcat tcaaatacgc cataattctt
93541 tgcttagcat ccattgaatt gcgacgcact gtatttttct ccgcagtgac taatttttga
93601 ccaaaatcat tgagtagact taaggctaga tctggcgttt gacgaactaa tttttgaaaa
93661 tcattccgtg tcatcgaaca cacccaagca tcagtagttg cctgaataaa attctcacga
93721 gcgctagtcg aaaataaatg ttcttggcct tcagaatcac ccttattcag caagcctaaa
93781 atcgtctctt taccatcttc attaagacta taaactttag cctttccctt atccatgaca
93841 agcagcccgt cttttccatc aaagggttgg cgaatcaaac tgcctttagg aaaatattct
93901 tgatgcgtcg aaatggtaac tagctttttt ctaagtgatt ctggtaaatc cttaaataaa
93961 gtggcattac tcaaacaagc aagtggcgaa tgttttgtca ttttacatac ttctttcttg
94021 atataaatca attttattat atcctatctt tttataatta acttgtaagc actgataaat
94081 gaattttcaa gcttttcgat ttatgataga gctgaagtgt tacttaaaag aaaggatcgt
94141 tattatgaca aaatatattg ctcacattaa cccattaaat gcagaaaaaa ttggttgtca
94201 gccccatggg acagctgaat ttaccgaaaa tggcgatcaa cttcacatta aagtagaaat
94261 gtttgataca ccaaagaata tcgaacactg ggaacatttc catggctttc ctgatggtaa
94321 agtcgctcag gctgcaacta tggctcaaga tgtcaaccac gacggctttg tcgatttacc
94381 tgaaactgaa ccagtatcag gaactacgat ggtgccactt gatgcggatc ctgccaaaat
94441 gaatattcct aacaatactt accctgtcgc tgacagcaat ggttattacg aatatgaaat
94501 tgatgtgcct ttgaccgaat tacaagaaaa cttcaaaaaa gctttcagca gcaccgatct
94561 tcaattagat aaacgcgtag tttatgtcca cggagttcct gagagcatta aattgccgga
94621 cactgttaag ggctgtgtaa tggattatga tactcacact accttgccaa ttgccgcagg
94681 aaagattgaa aaagaagatt aattagataa ttaaaattaa ttgaatattt attctaaaaa
94741 gctgttacta ataatagctt tttattttta tgtgtttcat tgagagagta ttaattttgt
94801 cgatacattc aagtacgtag ttgtgatctg atactaataa gactatcgaa cagatgcaag
94861 tttgcacgat cctaatgtat cgtcaccatc tgtttaataa gcatataaaa acgggtcatt
94921 tttactaatg acccgttttc tggttgtatt ttaaagttga ttaacccttg ataacaataa
94981 ttactacatc ttctggcttc atgatttggt cctccttttg tttaatcttt atgctactaa
95041 tcatatttag ctaccagaat cagatcacaa ttagtcatat ttaactcaaa aattatcttt

95101 aataaatttt ctgagttcat tgctgtattc ttccatgcct gcatcttgta
gagttttaat
95161 aaaattcttg cacttattta tttttcccgc atcattacta tttttaattt
caattatatt
95221 tttgaaaaac tcaatatcta ttttataaaa aagcaattgt ggcgtagttt
ttatattttg
95281 aatcttatta agaaagtaag agacgttttc aaatttatca ttttcaatag
aaaaaataag
95341 aatattgcat actatggcta aaataagttc ctgaatatta gagtcattgg
tggattgata
95401 ctgttttaaa atgttccgcg taattatttc attatctgat aagctataaa
atcgcataaa
95461 gtcgcagtaa agcattagct tgtcttgagt ataagatgga atgtcaaaaa
ttttattttt
95521 aatagtcgat gtgagctttt cactatttag ttttggattc attaatgcta
aaaatccatt
95581 agtcattaat gtttgatagt ttttatcata ttcgcttaaa gtactttcgt
gaataaattt
95641 cttaatatta tgcatctgct ctatattatt agtgtaataa gcttcaatca
tcatattttc
95701 caattctttt gtttggcggt atgaatcatc gctttttttga ctaaagttat
taaaaaattc
95761 ctgtacagaa atattgttat attgaagcaa gcctattaaa ttgtccgctg
taatttgact
95821 tatattcttt tctacttttg aataataaga ctgcgtaaca attgaaccaa
taaattttct
95881 ttggctcttg ttttgtttta aacgatattc tttcaaaaga ctgccaatca
tcatatttat
95941 ctctctttgc aatgagtcat atatgactta ttttactata tatatggcta
atttgctaag
96001 ctcaacctaa ttaaacgagg tgaatatgaa tgaatatatt tttaactaat
aaaaattatc
96061 gtagattttc aattgctagt tttctttcta gcgcaggtga tattcttttc
tacttggcat
96121 ttatgactta tgcaagtaaa ttaaaaaact attctttagc actttcatta
attgccattt
96181 cagagtcttc acctaaatta tttacaattt ttggcggcta ctttgcagac
aaaacgcgac
96241 ataaatttaa aaatatattt ttaagcgcca ttattcgttg tgttttatat
ggcatcgttg
96301 gactgctatt tatcagtaat gtcaatcaat ggagtttagt aattattgtt
gtaataatta
96361 atttgatttc agatacattt ggagcttatt ccagcggatt agtttcacca
ttagtcgttg
96421 atctagtagg aaaagatcat ttcggagaag cagagggttt tactaatggg
gttaatgaag
96481 ttattaatat agcagcacag tttataggct cagcattgct gctaattatg
tcatactcat
96541 cccttgcgtt tttgaatgct atttcatttt tacttgctgg aattttgttt
gcgagtgtgg
96601 gactgaggca aaaatctatt gagtcgctta aaagccatga aattaatact
caatcgtttt
96661 tggtaacaat gaagacgtct ttcaagcaag ttaaaaagca ggctggtctt
ttagaggttg

96721 tcttgattgt tgcagttttg aatggattgc taagttcaat gggatcatta attccaatta
96781 tgatagcagc tcataaaaca gcaatggtaa tttcaagata tagttttacg attgctttga
96841 tgggtgtggt tgtagcttgc ggagctgctt tgggaagtat gtttggtacg caattgttta
96901 aaaaattcac aattttttct atgacaattc tagccacttt aatcgcttta atgaccactt
96961 ttgcgatttt tgcaactagt atctatcttg ttttaggatt gtatttttta ttagcattaa
97021 cagccagcgc aacttcaatc aaattgacac aatggctagt aacgtctgtc gagcataaga
97081 ttttagcttc tactgtcggt ctattaaaca ctattttgat tgcgtctgct cctttgatga
97141 ctactatttt aacaactgtt tcaggtgcaa ccaatataag atatgccctt attttgcttg
97201 tagttttaga agtaattact tttttgatag aacttaaagt aagtcataag gtaaaagcag
97261 cctcaatcat atcttctata aaggagtgaa atatttattt ataccacttt tttatgtctc
97321 aacagttcaa gagattagaa cagtcacttt gcatgatttt tcataatttg ttgcggtggt
97381 gcaagtttag ccatgctggt ctgcagcatt gtattactcg caagtttcat tatctgctta
97441 atttgggcac cagaaacttc acccaaattc aaaaaagaat aactaatttt aaagtctcac
97501 ttgaggcttt ttcttttgtg gctcataata gccataatta tatttataaa ggagaatact
97561 atgactcaag aagcactctt aatcatcgat tacactaatg attttgtttc agacaaaggc
97621 agcttaactt gtggcaaacc tgctcaagaa cttgaaaatg aaattgtcaa tttagcagat
97681 tcatttttaa agcaaaataa atgggtaatt attcccactg acctacattt cccaggaaat
97741 aaatatcatc ccgaaactaa gttgtttcca ccgcataatt tacccaacac ttggggtcgg
97801 caattatatg gcaagctgca aacttggtat gatgccaaca aaacaaatga tcatgtcatc
97861 ttcatggata aaacgcgcta ttctgctttt gcaggcacta acttagatct tattttaaga
97921 gaaagaaaaa tcgatactct gcatctgact ggtgtctgca ccgatatctg cgtccttcat
97981 acagcaatgg atgcatataa tcgttgctat aatttagtaa ttcaccaaga tggtgttgca
98041 agttttgatg aaaatggaca taaatgggcc ttaaatcatt ttaaaacttg cctaggtgcg
98101 actatacttt aattccaaaa aataagccct ctagcttaaa tttgagctag agggctattt
98161 tgtatattta agctatccat ctaattcgac accttgcaat tttttaattg catcatagac
98221 ttgtccccgt cgattttgat caacagctag cactaataaa tcgccagctg caatcacagt
98281 gtctccgtga ggaataatat cttgactgcc gcgatgaata actttaatta aggtatcatc

98341 cggccattta acatcttcta tcttttcatc gactaattta ctgctttcat agactgggat
98401 tgaaatttga tcttcttcgc cgcacaatcc cttttcacta gattttggct ccatagcagc
98461 cgctaagcta tcataaattg gacggccgcc aagtaattgg tcaactaaca aagcaataaa
98521 tgctacaaca gctaacggca ttaaatgtaa cagcgatccg accatttcag taattaaaat
98581 aatcgctgta aatggtgctc gaataattgc tgcaaaataa ccagccatgg caaaaataat
98641 taaattaata actaattttt gtggcaataa tcccagctgc accataaata gaccgtaagt
98701 tgcgccaatt aaagcaccca ttgttaaaat tggtaaaaag atgccgctcg gcagacccga
98761 atcataagaa acaattgaaa atgcaattct aattacataa aaaatcgcca gcatccctac
98821 taaattccaa ctgctataag caattgactt aggcaaagac aaaatcaaat gattacctgg
98881 tccagtaatt agcggcaaat aatacataat cggcattaag atcactagtg gaatcaatcc
98941 gtgtagccag cgcggaataa aagtgatttt tgcataaagc tttttaaaat taaacaagcc
99001 caatttatat aagtgtccta aaagtcctaa aataagtcct aataacacta aatgccaata
99061 aagtggcatt gggaaggaat gattgtaagc taaagctaaa gctggttttt gaccaaataa
99121 gttagataca acaaagttcg aggcaatcgc acccaccaag gcattcatcc acacacgtgg
99181 cgaaaaatta tgaaaaattt cctctaaaac aaacaacgca ccacttaaag gggcaccaaa
99241 agcagctgat aaaccactgg cagcacctgt tgctagcaaa actcgtgaat ttgttttagt
99301 ttgcttaaag ccttggccga ctccttgacc aattgtggaa cctaattgaa gcgatggtcc
99361 ttctggtcct aaaaacaagc ctgttccaat aaccataatg ccgccgatta actttcgcca
99421 caaaatcggc caccattgca aagttaattt tccttgcatt tgtaatttaa cttccggaat
99481 accagaccca ccaacgtgcg gatattgttt aacaaaatat cctgcaatta tgccagttaa
99541 tgccagtcct gctacgatta caataaacca taatggattt tggtgcgact ttttaaataa
99601 atcgagccaa aaagcttcgg ttttaccaat tccatatcta aaaaatccca ctacaactcc
99661 agcaattaat cccacgcata atgcttgcag cattaatttg agattgcttt tttccccgta
99721 tctcatactt tcccgctttc tgattgatat aaatattatt ttaacgcaaa aagccactac
99781 taatgtagtg actttttagt ttcatgtgaa acattattat ttttcttcgt accattcagt
99841 atggaaagta cctggacgat catttctttg gtaagtgtga gcaccaaagt aatcacgttg
99901 accttgaatc atattttgag gcaaacgtgg gttaaagata gattctaagt agttcaaagc

99961 tgcgctaaga gttggagttg gtacacctgc atcagcagca attttaacaa
cttctcttaa
100021 agcactaata ttttctttct ttaccttatc gaaaaatgga gccttgaata
agtttggtaa
100081 gtctttatca gcattatagg caaacttaat atcatgaagc atttgtgaac
gaataatgca
100141 tccagcttcc caattttgag cgatagttga gtagtgcaag cgccaattat
aagcctttgc
100201 agccattgtt agttgctcaa atccttgtcc atatgaaaca gccattgcta
aatataaagc
100261 tttcgcaagt tgatcaacta aatcttttgg agccttttct ggatcaatct
ttggttcagg
100321 atcatctctc aaagttgctt tcgacataaa gcgtgcaaga acagcttccg
caattacagt
100381 aattggagcg ccaagacgaa ttgcatcttc aagcatccag ttaccagttc
ccttgtaaga
100441 ggcttcattt aaaatatgat caataatgtg gtcatcagtt aaattatctt
tttgctttaa
100501 tactttcgca gtaatttcaa ttaagtaagc ttgtagagct cccttatccc
aattcttgaa
100561 aatttcactc atttcatcat ttgagtaatg accaacagta cgtaaaacat
cgtatacttc
100621 agaaataatc tgcataatac cgtattcgat accattgtga accatcttta
catagtgtcc
100681 acttcctgct ggcccaatgt agccaacaca tggttgacca gttggagtct
tagcagccat
100741 tgcttcaaga attggtgata cttcttcata agccttttca tcgccacctg
gcattaaagc
100801 tgggccattt aaggcacctt cttcaccacc agatacaccc attccgataa
agtgaatgcc
100861 atgtttttcc atttcttcat aacgacgatt agtatcatgg aaattagagt
taccaccatc
100921 aattaaaata tcgcccttat ctaaaagagg caataatttc tgtaaagtct
catcaactgg
100981 tttaccagcc atgatttgaa ttaagatctt acgtggcttt tctaatgaat
ttacaaactc
101041 ttcccaagta taagtaggct ttaaatcttt atcttcatat tttgctaaag
catcaacctc
101101 tggtttatca attgaaaatc cagagacaga gtagccatga ttcttaacgt
ttaaggcaag
101161 gtttttaccc ataacggaca agccaataac cccaaattgt tgcatatttt
tgcctccaca
101221 tatttaataa aattcttcat ctactagtat actttttat tgcaaatttc
gcacaatttt
101281 gcttactttt tatcaaaaaa ataaagagtt caactatgtg aactctttat
ttaattattt
101341 cctaggaaat taatgacgtg atgcgcctga tgaagcatct tcctctgctt
cgactggttc
101401 tgcctcagtg tgttcagaag cacctgatgc ggcatctgct tcaacaccat
acttgccagc
101461 aaagtaacta aatggtttca aagcttgggc atcgtactta gcaacgaatg
ctggatcatc
101521 caaagtattc aactcagttg aatatggcat atcatgagta atcttacagt
caattagaac

101581 tgggccttcc atttgttgag cagctgttac tgcatcttca aattctctct tattgcgaac
101641 agtaaagccc tttacattca taccttcagc actcattgcc caatcattat caggaagctt
101701 tacacctgat aatggttgat gtgattcatc aacttgttca gcttcaatgt agcctaaagt
101761 ttcattagtg aagactacgt taattacatg catgttgtaa cgtgcttggg tcaaaagctc
101821 ttggttcatc atagcaaagc caccatcacc agctaggttc caaacttcac ggtttggata
101881 aatagttgct gcagcaagag aagctggagc accataaccc atagttgcat agagtcctga
101941 agtagtccat ttttgatcac cgtgcaagtg cattagacga tcaaagttaa tgtttacgtt
102001 accaacatct actgcaaata ctgcatcgtc agctgctttt ttgttgatga catcaaaaat
102061 tggttcaact cgcattggaa tttcatctga atcattaaag ctttcaagcc aagcttccca
102121 gttttcacgg tcagcaagag ctgccttata caatggagat tctggacggc tttcacctgc
102181 ttcaattaaa gcacgtaaag tcttcttagc atcagctaac attggaacag ttactgcatg
102241 gcgtttacct aatctctcag aattaatatc aatttgaatt acttttgcat tagggttaaa
102301 gaagaagact gagaatggtg aattattacc aacccaaaca actaaatcag catgagtttg
102361 aatatcgtta cttggcttag caccaacacg accaattgaa cccatatatg ctggaaattc
102421 atcttcaaca attccttttc ctagaactga tgaaactagt ggcatcttga atttatcaga
102481 aaattctttc agttccgcac tagcatcttc agcaccatga ccaaagtaaa caattggatt
102541 ttcagcattt ttaattaatt caagcgcctt atcaacctgt tccttacgag gtgctgggaa
102601 gttaggtaca actttatgcg ctgcagcatt gttgcggaaa ttatcctcga ttttatccca
102661 accaaagtcc ttaggtaaga ttaaaactgc tggacctttt ctttgataag cttgacgaat
102721 tgcttcatcc atcaaaactg gaattgattc agctgttttt gcttggtgat tccaaacagc
102781 aacaggatca aaccatggct tttcatcaaa agcttggaag aagtcaatat cttgacggct
102841 tgttggaacg ttagcaacaa tagcaaccat tggagtctta tcatattttg catcataaag
102901 tccattcatt aggtgaactg caccaggacc agcagaacca aagcaaactc caactttgcc
102961 actaatctta tattcagcag aagctgctaa agcaccagct tcttcatgtc ttacttcaat
103021 aaattttatc ttatctctcc agttatgaat tgcgttcatt gttgagtcaa atgaaccacc
103081 tgggaaaccg tatatatggt caattcccca gtcatacaaa acttggaaca ttgcatctga
103141 accactaatt ttcgtcatag tctataagca tctccttaat gatttttctt acaccttgat

103201 taaagcacat ttattaacta attgaaagcg tttgccataa ctgatatatc aatatttaga
103261 gcatactcaa aaataattat ttcacaaact atttaccttt ttgcttaact tatcggtaaa
103321 acacaatttt aaagtgtgat taagtgatca tactcaacaa aaaaagcaaa aaaactttta
103381 aaaatttttt tgctttgatt caattattat ttatttcttt attttagcaa ttcttccttg
103441 ttgaatataa gcagttaaaa ttgctaaatc agctggatta accccagaaa ttctttcagc
103501 ttgagcaata gattcaggac gaatttttc gaatttctga cgtgcttcag tagccaagct
103561 tggaattgca ttatagtcaa tatctactgg tattttcttt gactctaatc gatgtaaccg
103621 atcaaccaaa gtcttttctt ttttaatata gccttcatat ttcaatgcta tttccacttg
103681 ttctttgaca taacgatcag tagctaaagt ttgaccactt aatttttcaa cggcatcaaa
103741 agtgacgcgg ggacgcttca aaaagtctgc tccgctaact cctgcattca aacggtcctg
103801 cttgacgcta gccaaatatt cttgtacttc atcagttgga tgaattgtaa ctttttttaat
103861 agctgccatt gcccgggaaa tagcctgctt ttttgcttca aattttgcat agcgctcttc
103921 tgaaatcaag cctaacttat gccccttttc agtcaaacgt aagtcggcat tatcatgacg
103981 aagaagaaga cgatactctg ccctacttgt aagtagacga tatggctcat tagttccctt
104041 agttactaaa tcatcgatca agacaccaat ataagcttcg tctctttgta aagtaaagcc
104101 aggcttattt tgagcactca aagcagcatt gattccagca attaaacctt gtccagcagc
104161 ttcttcatag cctgaagttc cattcatttg gccagcagta aagagatgct caatattctt
104221 agtttctaac gtgtgcttta attgccaagg ctcaatgaca tcatattcaa tagcatatcc
104281 tggacgcatt aattctgctt tttctaaccc agcaacacta tgaagcatct ttaattgtac
104341 ttcttcaggc attgatgtag aaaagtctcc tacgtagatt tctttagtat tgcgtccctc
104401 tggttctaag aaaatttggt gacgatcttt atctgcaaag cgaactactt tgtcttcaat
104461 tgaaggacag taacgtggtc ctacgccttt aataactcca gaaaacattg gtgctcggtc
104521 aaggttagct ctaataatat cgtgagtgac agtattggta tatgtcatat agcaagacat
104581 ttgatctttt aagtagtcac tatctttact tgtgtaagaa aaatgtctcg gttccttatc
104641 gccaggctct tcctgagtct ttgaataatc aactgtatta ccatcaacac gtggaggagt
104701 accagtctta aaacgacgta atttaaagcc taatttttca agattttcag gcaatttaat
104761 tgatggcaaa gagttgttag gtcctgaaga ataagttaat tctccaataa taattttccc

104821 tcttgcagct gttccagtgg ttaaaactac gcttttagca tgatattttg caccagtatt
104881 agttattaac cctttgcata ctccgtcctc aacgattaat tcatctgcaa cagcttgccg
104941 caaagttaaa ttaggttcat tttcaatggt atctttcata tgttcatgat attgccactt
105001 atctgcctgt gcacgcaaag ctctaactgc aggccctttt cccgtattaa gcattctcat
105061 ctgaatgtaa gtcgcatcaa tattcttacc catttctccg ccaagtgcat caatttcacg
105121 cacaacggtt ccctttgctg ggccaccaac tgatggatta catggcataa atgccaccat
105181 atctaaatta atagttaaaa gaagggtttt ttctcccata cgagctgccg ctaaagccgc
105241 ttcaacccca gcatgtccag ctcccacaac aataacgtca tactcgtttg attcgtacgt
105301 cttcattttc atcctacttt ccaagacaga attgactaaa taactgtgta attaattcgt
105361 caggtgcact ttcaccagta atttcaccta aagtatccca agcatttttt agatcaattt
105421 gaaccaaatc taaaggcata gcatcattaa ttccgctaat tacatcctct aaagactgtt
105481 ttgccttagc taataatcca gcttgtcgtt gattcgttac caaaatttga ttttgagaat
105541 tttctattcc gctaaagaat aatttctcaa tagcattctc aagtgcatcc atattctttt
105601 gttttaagat cgacgttaca ataataggat tatcggtaag gtttttgatc atttcctggg
105661 aaattttcgt acctaagtcc tgtttattca aaataatgat ccgcttttta tttgcagtca
105721 aatctaataa tctcttatct tcagctgtta aatcttgact tgcgtcaagg agcaaaagaa
105781 ctaggtcagc ttccttaata gccttcttag aacgttcaac gccaattttc tcaactttat
105841 cttctgtgtg gtgaattcca gcggtatcaa tcagcttaag tggaacaccc ttaacagaaa
105901 catactcttc aagcgtatct ctagtagttc cagcaatatc ggttacaatc gctttatcat
105961 cttgagttaa atagttcaag agcgatgatt ttccaacgtt tggtctaccg acaattgcag
106021 ttgctaagcc attacggata attttgcctt cttgagctgt ttgaagtaac tgctcaattt
106081 gttttgaaac ttcttgtgct ttctttttca tttcctgaga agttaaatcg tccatatcat
106141 attctgggta atcaatatta acttcttcat gagccattgt atcgagcagc tcttggcgca
106201 tcgtcttgat tttttctagc aagccaccag ctagttgagt catcgcaact tggcgagatt
106261 tatcagtttt agctctaaca atgtccatta ctgattcagc ttgagttaaa tcaattcggc
106321 cattcataaa cgcacgttta gtgaattcac ctgggtctgc catcctggct ccattggcca
106381 aaagcaattg caaaatatcg ttggtaacaa tcattccccc atgacaattt atttctacca

106441 tgtcttctct agtaaaagtt ttcggcgcac gcaacacact gaccatcgtc
tcatcgacaa
106501 cttcctgcgt ttttggatca acaatatgtc catagtgaat tgtatgcgta
ggaactttag
106561 ttaaatctgc tcccttaaaa agcttattcg caatcgctac tgcatcttca
ccagatagac
106621 gaacaattga aattcctcct tcgccaatag gagtagaaat tgcagcgatt
gtatcaaatt
106681 gcgttaatac ttgtgccatt tttcacctac ataaataatt aaataaaaaa
agtgcccact
106741 ccaccactat aaaaacaatg atggataaag cactttgact actttatctt
aacaatttaa
106801 agataattta tttttgtttt ttacgtccta aacgtttgta tacacgacga
actttacgtt
106861 cacgttcacg ttctaccttc ttttgctctt ccaattcacg ttgatacttg
aatggatttt
106921 gcaaaatgaa ggtttgaact acttggaata agtttgaaat cacccagtaa
agtgagatcg
106981 cagattggat accaattgct gaaaccccaa tgataagagg cattaagtat
gtcattccct
107041 tagtcattcc attttgtgag gattgaggcg ttgacatttg actaatatac
gtacttataa
107101 aagtaaatgc agctgccaaa acaggcatga tgtaatatgg atcgggacgt
cccaaatcca
107161 tccacaaaaa ccgaccattt tgcaactgtg gagtccgcca aattgcttga
tacaaggcat
107221 acataactgg taactgaatt agcatcggca aacatccagt atacggatta
ataccggctt
107281 ctttatacaa cttactagtt tcttcttgca gcttttgacg tgactcaaca
tccttaccag
107341 gatacttttt acgcaatgca tccatctgtg gttgcacttt ttgttgctta
gccatgctct
107401 taatagaaat ggcattcaat ggtaataaga ttattcttac tataatagta
aagataatga
107461 ttgcccaacc ataggaatca cgaactaaac ttgcaatcca aagaataaat
tgggaaatat
107521 agtaaacaat ataccgatcc caccaattgc cactagtatg agaaatagga
gcaacacttg
107581 atccattagt agatgcacaa cctgtaacta ctaaagctaa ggttagcatt
cctagaccta
107641 atagaatcag tttccaattt ttcttagata aatgatttct cactctactt
ttcctcagtt
107701 tccgtctctt cgtttgataa gactttagct aaccttaaga catgaataag
attctttttg
107761 gttcgttcca tgtcaaaatc tcttgcataa ggcctagcga ttactaaaaa
gtctaaatca
107821 gaccttatct ctggcttaag ttctgtcagt gtagcacgaa tgaaccgttt
gaggcgatta
107881 cgtgctaccg ccgtatgacc cactttttc ccaacagaaa ttccaacgcg
aaaatgcttt
107941 tgtttttctt tgggcaaggt atagacaaca aagcctctat tagccattga
ctgtccttct
108001 ttaaaaactt tttgaaagtc tctctcagtt tttacacggt agctttttct
caatctagtt

108061 catccttact taaattaaaa aaaccactga gcttcagtgg cttaagcaga taagactttt
108121 ctacccttttt tacggcgtct tgctaaaacc ttacggccgt ttgctgtagc catacgcttc
108181 ataaaaccat gaacgcgtga acggtgacgc ttcttaggtt ggtaagttct cttagttgac
108241 attaatgttg cacctccgtt tttcaatgct taataagcac aatttctaaa atagaatagc
108301 atatttacgc ctaaaaacca agtctttatc aaaattttcc taaaaagaaa agcttttta
108361 aagttatttt taaattatat tttgtggata tcttggatat atatttttgc gtgctttttt
108421 atcaacaaga taattctcag agttatgcac aattcacacc ctgttcaaaa tataataaac
108481 accctgtgca taagtgaata cttctcggca gaaatactga tgtatcgcta agtttaaacc
108541 cacatgaatt tgtgaataat gcaaaagtga aattttattt caaagatcag tggattttta
108601 ttctctcttt tccacatact tgtgaatttg ttagttattt ttgttccttg tggaaaactt
108661 ttaaaattca tgctaaactt attttgactt aatctgtgga taatacagta tttggaggaa
108721 tttttttgtt tgatttagat aaattttggc aatttttcaa tgctgagatg aaaaaaagct
108781 acagcactgt tgcctataac gcttggttta aaaatactaa acctatttcc tttaacaaaa
108841 agacaaaaga aatgataatt gctgttgaat ctccagttgc aaaagggtat tgggaaaaga
108901 acttggcttc tcaactaatt caagaggcat atgcttatgc agacatggaa attcagccaa
108961 aatttgaagt agcaggtaaa gaaggacccg aacgtctagt tacgccaaag ccaagaatta
109021 aaactaatca agaaatatta gaggatcgcc gtgacgaatt tgctcaagat cttcaattaa
109081 atagtaagta cacttttgat acctttgttc aaggtgaagg aaataagctg gcagctggag
109141 ctgctttagc agttgccgat aatccaggaa gtttttataa tcctttattt atctttggtg
109201 gtgtcggttt aggtaagacg cacttaatgc aagctatcgg tcatcagatg ctagctgaaa
109261 agccccatgc aaaagtggtc tacattcaaa gtgaaacttt tgtaaatgac tttattaatt
109321 caataaaaaa caaaactcaa gctgaatttc gcaataaata tcgaaattgt gatctcctat
109381 tagtagatga tattcaattc ttctctaaaa aagaggggat tcaagaggaa tttttccata
109441 cttttgaaac tctttataat gatcaaaaac agattgttat gactagtgat cggttgccaa
109501 ctgaaattcc tgaattatca gagcgactgg tttctcgatt tgcctggggc ttacaagttg
109561 agattacgcc tcctgattta gaaactagaa ttgcaatcct acgtaaaaaa gctgaaacag
109621 atggcttggc gatcgatgac agtactcttg actacattgc ttcgcaagtt gataccaata

109681 ttcgagaact cgaaggtgct ttagttaaag ttcaagcaca tgcaacaatt gagcgggaag
109741 atattaatgt tgatttagca aaagaagctt tagcagacct aaaacttgtc caaaagaata
109801 gaggacttca aatttcaaaa atccaagaag tcgtcgcaaa ttacttccaa acttcaacta
109861 cagaactaaa aggaaaaaaa cgggtaaaac aaatcgtagt tccacgtcaa atagcaatgt
109921 atctatcgcg tgaattaacc gattctagct tgcctaaaat cggacaggaa tttggtggca
109981 aagaccatac cacagttatg catgcctgcg ataaaatttc tcgtgcctta aaaaccgatg
110041 ccgaaattaa agcagctgta tatgatttaa aagcaatgct tgaacactaa aagttaataa
110101 catgtggata agttttcact ttgtccacat ttattgaaca cacaaaagtc cgttttattc
110161 aggtatttgc ctactttccc acagattcca ctggccctac tactacttct aagtatttaa
110221 atatataatt aaataaataa gacaaagtac atataacttc acaggaggta aacgatgcag
110281 tttacaatta atcgtaattt attcctcgaa aatttaaata atgtaatgcg tgcaatttct
110341 tcacgtgcaa ctattcccat tttaagtggt ataaaactta accttactga cgaaatgcta
110401 actttaacag gtagcgatac tgatatttca atcgaaattc agattcctgt aaatgatgac
110461 ttaattgtcc aatctacagg ttcaattgtt ttacctgcac gtttctttag tgagatcgtt
110521 aaaaaattgc ctggtaaaga tttttcattt gaagtaaagg aaagcttcca aacaaaaatc
110581 gtttctgaaa ataccgaatt tatgatcaat ggattagatg ccaataacta tcctcattta
110641 cctgaaattt ctactgatgc atcatttaag atttcaggta aaacctttag agaaattatt
110701 aacgaaactg tttttgctgt tgctactcaa gaaagtcgac ctactttaac gggtgttaac
110761 ttcattttta ataattcttc aattaaggca gttgctactg acagtcatcg cttatctcaa
110821 cgtcaaattt ctttagaaaa tgggccacaa actagtactg atttgatcat tccaggaaaa
110881 agcttagtag aattagcgag aattattggt gaaagcgatc ctgaaattaa agtaaatcca
110941 ggtgaaaacc aagttctatt tgaagttgga aatattgcct tttattcccg cttacttgat
111001 ggtcaatatc cagatactga tcgcttaatt ccaactgaat ctactacttc tgttgaattt
111061 gaattgccag ttttagctag atctttagaa cgtgccagtc ttcttactca tgaaagtcga
111121 aacaatgtag taaaaatgac tcttgatgtt caaaatcaat tagttaagct tcaaggtgat
111181 tctccagaaa taggtaatgt ggaagaagaa attggattta agaaccttga aggtgaaggc
111241 ttgacaattt catttaaccc cgactattta agagaagctt tacgcgcatc aattactgat

111301 tccattatta tgaactttac gcagccactg agaccattta cagttgtgcc tgcaaagcaa
111361 gatgtcaact ttactcaatt gattacacca gtgagaacat tttaaattac tgaaaaagag
111421 ctacggaagt agctcttttt ttcttgttgt acttaaaagc taaatttagc ctggttaaac
111481 gaatttttct ctagttaagt atagtcatat taaattagaa taaaatggct taaaaaaggc
111541 tacaatacgt cataaaattg aatttcacat aaaaaaacgg tataataaca tggtatgaca
111601 aggtaaatgg ggtgatatta tccaaaaatt cacaattaag ggagaatata ttacgcttgc
111661 tcaattttta aaagaagaga gcgtgatatc ttctggtggt caagctaaat ggtatctaaa
111721 agaaaatcct gttaagttaa atggcgagct tgaagatcgt cgcggaaaga aaatacatgc
111781 aggggatgta ttgaacttag ctggagaaga gtatgaattt gtttcagagt agctaatgta
111841 tcttgcaaac tttgaattga aagactttag aaattttaaa gaattaaaaa caaattttga
111901 tcctcatgta aatattttca ttggtccaaa tgcccaagga aaaactaatt tacttgaggc
111961 aatttatttt ttagccctta ctcgatccca tcgcactaat agcgataaag aattaattcg
112021 ttttggcagc aaatttgccg gtttacaagg tagggttcat aaaagccaac ttcaagtaga
112081 gcttaaacta cgtttaacgg ctaatggaaa aaaggcctgg gtaaatcgct tggagcaaaa
112141 gaggctttct gcctatgtgg ggcaaatgaa tgcaatcttg ttttctccag aagatttagc
112201 tctagttaag ggagcaccat ctgttagacg gagatttatg gatttagaat ttgggcagat
112261 taattcagaa tatctatatt tttcaagtca gtatcgtcag gtactgcagc agagaaacaa
112321 ttatttaaag caattaagca tcaaaaaagc aaatgatcaa gtttttttag atgttttatc
112381 cgatcagctg gcaggaattg cagcagagat aatatcgcga agaataaaat acattaaaaa
112441 actcaattcc tacgctcaag ctgctcatcg tgagattagt ggtcaagctg aaaaattgca
112501 gatttttttac cgtccatcag ttaaggaaat tattccagag gatgatgtag agaccattta
112561 tcaaaaagtt attactagtt ataagaaaaa tcgttctaat gaaattcgaa agggtactac
112621 tctcagtggc cctcatcgag atgacttgga gtttttaatt aatgataaaa atgcccatga
112681 ttttgcatct cagggacagc agcgaacgat ttctttaagc gttaagttag ctgagattca
112741 attagtgcat gaattgacgc aagaatatcc gattctgctg ttagatgatg tgatgagtga
112801 gttagatcat cgaagacaga gtcgtttatt gaactatatt catggtaaaa cgcaaacatt
112861 tataacaacg acagatttag aaggtatttc ttgggaaatt gtgaaggaac ctaaggttta

112921 tcacatttct gctggaacga tcagtgcaaa ggagagttaa atggcagaag ataagagcaa
112981 agcagcatta aataaagcta aagactttga aaaacgtgct gatacttaca atgcaagtca
113041 aattcaagtt ttaggtggac ttgaagcagt tagaaaacgc ccaggtatgt acattggctc
113101 aactagtagc cagggtttac accaccttgt ttgggaaatt atcgataata gtattgatga
113161 acgattagca ggttttgcta ctaaaattga aattactgtc aatgaagatg gtagtgtaac
113221 cgttcaagac gatggacgtg gtattcctgt tgacattcaa gctaaaaccg gtcgtccagc
113281 tttagaaact gtatttactg ttttgcatgc tggtggtaaa ttcggcggtg gcggatacaa
113341 agtatctggt ggtcttcacg gtgttggtgc ttctgtagta aatgctcttt caactaagct
113401 tgatgttaca gttatgagag atggtaagaa atatgcgatt tcctttgatc atggccgtgt
113461 agtaggagaa atgaagcaga ttggaacagt tcctcttgaa gaacatggaa caattgtgca
113521 tttttatcct gatccagata tttttactga aactacaact tttgacgaca aaattttaaa
113581 gaacagaatt cgtgaacttg ctttcttaaa caaaggctta aagttaacct ttactgataa
113641 gcgtaaagaa agcgctgaaa ccgatgttta ccactttgag ggtggtatca aggaatatgt
113701 tgccttttta aataagggac aagaagtctt atttgatgaa ccaatttacg ttgaaggtaa
113761 ttacgaagga attgatgttg aagttgcctt gcaatatacc aatggttata agacaacttt
113821 aatgactttt gctaacaaca ttcacaccta tgaaggcgga atgcatgaag ctggatttaa
113881 gacagcttta actcgtgtag ttaatgatta tgctcataag gctaagatcc tgaaagacaa
113941 ggacgataac ctatctggtg aggatattcg tgaaggaatc acagctgttg tttctgttaa
114001 gcaccctaac cctcaattcg aaggtcagac gaagacaaaa ctaggaaatt cagatgcaag
114061 aactgcagtg gatcgagcat tttcagaaac atttagtcgc ttcttaatgg aaaatccttc
114121 tgtcggtcgt aagattgttg aaaaaggtca acttgctgaa agagcaagaa ctgctgccaa
114181 acgtgctcgt gaagttacac gtaagaaatc tggtcttgaa attgcaaact tgccaggaaa
114241 attagccgac aatactagta atgatccaag tatttctgaa ttattcattg tcgagggtaa
114301 ctcagctggt ggatcagcaa aacagggacg ttcacgttta actcaagcga ttttgccaat
114361 tagaggtaaa attttgaacg ttgaaaaagc ttcaatggat cgaattcttg ccaatcaaga
114421 aattagatcg ctctttacag ctttaggtac tggatttggt gctgattttg acgtgtcaaa
114481 ggcaagatat cacaagttaa ttattatgac ggatgccgat gttgacggtg cccacattag

114541 aactcttctt ttgactctct tctacaatta catgcgtcca atgattgaaa aaggctatgt
114601 ttatatcgct cgtccacctc tataccaagt tcgtcaaggt aagatagaga agtatttaga
114661 tacagatgaa gagttacatg attatctagg tacgcttcag ccaagtccta agcctgttgt
114721 tcaacgttat aagggattgg gagaaatgga tccagaacag ttatgggaaa cgactatgaa
114781 cccagaaaat cgtcgtttag acagagtaag tccagaatac gctgaagacg ctgataagat
114841 ctttgatctt ttgatgggga atgaagtagg tccaagacga gaatttattg aaaagaacgc
114901 taaatacgtt gagaatttag atgcttagga ggctttagat ggctaacgaa aatcaacctc
114961 aagatcacag aatacgtaat gttgatttga cccatacgat gcgtagctct ttcttagatt
115021 atgctatgtc agttattgtg gcacgtgcct tacctgacgt tcgggatggt ctaaagcctg
115081 tacaacgtcg tattctttac ggaatgcatg aattaggggt tacaccagac aaacagtaca
115141 aaaagagtgc cagaattgtt ggagaagtta tgggtaagtt tcacccacat ggtgactcct
115201 caatttattt agctatggct cacatggctc aagacttttc atatcgttat atgctagttg
115261 atggacacgg aaacttcggt tctgtcgatg gggatgaacc agccgcaatg cgttataccg
115321 aggcaaaaat gagtaaaatt gcggttgaaa tgcttcgtga tatcaataaa gaaacgattg
115381 actggcaacg taactatgat gatacggaaa atgaaccggt agttttacct gccagaattc
115441 ctaatttact tgtaaacggt gctagtggta ttgcagttgg tatgacaact aatattccgc
115501 cacataattt ggcggaagta attagtggga ttcatatgtt gatggataat cctgatgtta
115561 ctaccaaaga cttgatgaag gttattcctg gtcctgattt tccaactggt ggtattgtaa
115621 tgggccgtgg cggtatctac cgcgcttatg aaacaggtaa gggtaatatc gtcgttcgtg
115681 ctaagaccaa tattgaaaca gagaagagtg gccgtgaacg aatcgtagtt agtgaattac
115741 catacttagt aaataaggct gaactagtta agaaaattgc agatttagct cgtgaaaaaa
115801 caattgatgg tattaccgga gttcgtgatg aatcagacca gactggtatg agaattacga
115861 ttgatattcg ccgagatgca agtgcgagtg tggttttgaa taacttattt aaagaaaccc
115921 aattgcaggc aaacttcgga atgaatatgg ttgcaattgt aaacggcgct ccacacttct
115981 taactttgaa gcaaatgctt gaatactatc ttcaccatca agaagatgtt attactcgca
116041 gaactaagtt tgaattaaag aaggctgaag ctagagcaca tattttagct ggattgagaa
116101 ttgcactaga tcacattgat gaaatcatta ggattattcg tggctcccaa aacagcgata

116161 ttgctaaggc acagttgatt caaaactttg gattagatga tcgtcaatct caagctattt
116221 tagatatgcg tctggttcgt ttaacaggtt tagagcgaga caagattgaa gctgaatatc
116281 aagatttaca agcaaaaatt gcagattaca aagatattct tgctaagcca gaacgaatcg
116341 atgaaattat ctacaatgag ttgcttgata ttcaaaagcg ttttggtgat gatagacgga
116401 ctaagattgc agaaggcgaa gcagtttcaa ttgaagatga agatttaatt gaacaaaaag
116461 atgtactttt aacccttacc cataatggct acattaagcg gatgccagct gatgaattca
116521 aggtacaaaa ccgtggtgga cgcggaataa agggaatggg agttcaagat gatgacttta
116581 tcaatcactt gattttctca agtacccatg actttttgct attctttact aacttaggta
116641 aggtttactc aaagaaagct tatgaaattc cagaatttgg taggaacgcc aaaggactgc
116701 caattgttaa cttgctagaa cttgataagg gtgaaaagat tcaagcggtt attaacgttc
116761 ctgaaggtgc agatgataac tacctcttct ttgttactaa gatgggaaca gtcaagagaa
116821 caaaagttag tgaatttcaa aatatccgtc gtagtggctt gattgcctta acccttcgcg
116881 atggggatga actgaataat gttttaacta ctgacggtaa acaaaatatc cttattggaa
116941 cccatttagg ctatgcagtt acctttaatg aaaatgatgt tcgttcaatg ggaagaaccg
117001 cagcaggtgt acgcggaatt aatctaagag acaacgatta tgtagttgga tctgatattt
117061 tagaacctaa ttcagaagtg ttagtaattt ctgaaaaagg ttacggaaaa cggactgcag
117121 cttctgaata tccagttaaa ggacgtggtg gtaaaggaat taagactgct aacattactg
117181 aaaagaatgg tcctttagca ggtgtaacag tagttaatgg tgatgaagat atcatgttaa
117241 ttactagtgc tggcgttatg attcgtttcg acgttgatga tgtttctcaa actggtcgag
117301 ctactttagg tgtacgttta atcaaggttg atgatggtgc tcaagttgca agtattactg
117361 cagtaccaaa ggctaatgat gaagacgaaa aatcaactga tgaagaagag ccagtagaag
117421 aaaattaata aataaaaaaa ttaccaaatt tttgcgtaat aataattgag taatgtaact
117481 caggatcgcg tgaaaaatat ttggtaattt ttttgtttct gtgataaaat caaacattgt
117541 gagtaatatt attcattact ccttgttctt gattttaaca agaaccattt agtccaagag
117601 gaggtgcaat tattatggca aaaactaagt acgaagttac ttacatcatc aagcctgata
117661 ttgacgaaga ttcaaagaaa gcattgatcg atcgcttcga taaagttgtt actgacaacg
117721 gtgctgaaga acttgaatct aaagattggg gtaagagacg tttcgcatac gaaattgaaa

117781 aataccgtga aggtacttat cacattatga ctttcgttgc agaaaactct
gaaccagttg
117841 atgaatttgg tcgtctttct cgtattgata accaagtttt acgttcaatg
actgtaaaat
117901 tagataaata atttgttttc gtgaattagg aaggggacaa aagtatgatt
aataatgttg
117961 tacttgttgg ccgtttaaca cgtgatcctg atttacgtac aaccgggagt
ggaatctcag
118021 ttgctacgtt tactctagct gttgaccgtc aatacaccaa tagtcaaggt
gaacgtggtg
118081 ctgatttcgt cagctgtgta atttggcgta aagccgcaga gaattttgct
aactttactt
118141 caaagggttc actagttggt attcaaggtc ggattcaaac tagaacgtat
gatgataaag
118201 atggtaagag agtctacgtg actgaagtca tcgttgataa tttctcttta
ttagaatcac
118261 gtcgtgaccg tgagaatcgt cagactaatg gtggtaattt tgctccgcaa
ggaggaaatg
118321 ctccaagtac caataacttt ggtggatcaa gtgcaccaag cactaataat
gctcctgctg
118381 gtggagaaag caataaaccg caggatccat ttgcagattc aggtagcact
attgatattt
118441 cggatgatga tctcccattt taaatttaat tagaaaggat ctttgatatg
gctcaacaaa
118501 gaagaggtgg ccgtcgtcgt cgtaaggttg acttcattgc agctaaccac
attgactaca
118561 tcgactacaa ggatgttgat ttgttgaaac gttttatctc tgaaagaggt
aagatcttac
118621 cacgtcgtgt cactggcact agcgcaaaga atcaacgtaa gttgactgtt
gctattaaga
118681 gagctcgtgt tatgggctta ttgccatttg tcgcagaaga ctaataaaac
agatcaaaaa
118741 taataaaaga gtagcatatg ctactctttt tattttattt tttgcggtat
cataaaagaa
118801 gtaaatggag ggattgtcat gattattaag caaaaactag ccggaaatat
tgattttgac
118861 tataaatggt acgatatcta taactgtgat gatcatgata taagattatt
aaaggatgac
118921 tttgatttaa cttcagaaat tatttcttat atcaccgacc ttcacgaacg
tccccacttt
118981 gatcatgact acattactaa tagtgatttg ttagtttatg atgtgccagt
ttggccaaca
119041 gcagatgcag accatttcac tactttacca attaaatttt tgatggttgg
gcatactctt
119101 tttacagttc actctcctga cacaacttat atgattgaag aatttaggca
gaagccagat
119161 gaacatattc atagtgaaaa agaactgatt tttgcaattt tattcgcagt
tactaagtat
119221 tttcaaagag ccttgagtca gctgaacagt caaagactag tgttagataa
tcacttgagt
119281 gaaagaattc ataataaaga cctgcaagag ctatcacaag ttgaaaaaag
tttggtttat
119341 ttgtctagct caattaggac taatctaatg atgcttgaaa gcttaaaaaa
taaaaaatca

119401 ggtcttcata tgaatgcatc tgaagaagaa atgtgtgatg acattatcat
tgaagttcag
119461 cagtcctcgc aaatgatcaa gatttatagc gaagttactg aagaaatatc
taaaacgtct
119521 aataacattt tgaataataa cttgaacaac acgatgcagt tttgactgt
ctggtcactt
119581 cttttaacaa ttccaacgat cgtaacagga ttttttggga tgaatgtgga
cttacctatt
119641 tttcataatc catttgattg ggtaataatt actgttggtt cagttatcgt
aatggcaatt
119701 ttactttggt atttgcgtcg acacaacatg ctataatgct tgcttagctt
agatgatgag
119761 caagcatttt ttgatgtata atataaaatg atgtagtata tattttttat
gatattaatg
119821 ctataacagg aaggaatttc aatgaaaggc tttttacgca aattagaatt
gcctgaattt
119881 gttaaagata cgcgtttaac agcatcattg gttattgtcc tagttttatc
cttattggga
119941 gctattattg ggatgttaat tagtccgttg tttggattag caatggtgct
actgtttatc
120001 ttgacggtta tttttgcagt ctatggcatt tatgttctag ctggtaatac
aaacaactat
120061 gctgctaatc tttcatatcg catcaagcgt ggtgaacaag aggcaatgat
caaaatgcca
120121 cttggaatta tgctttatga taaggaccga cagattcaat ggattaatcc
atatctacag
120181 atgtatctgc atgataaaga tattattggc tcttcgatct cttcagttga
taaagagtta
120241 gctaaatata ttgatgatgc tattaagtct aatagtaagc aaaataaaat
tattaaatgg
120301 ggcgatcgca agtttgagat ggtggtccag gatgatctgg gtgtggttta
cttacttgat
120361 attacgcgtt acgcaaatat tgaagagaag tataaacaag aaagattggc
aattggctta
120421 atatttattg ataactatga cgaattaagc caagcaatgg gcgatcaaaa
cttaacaaat
120481 atgagttcat atgttcaaac tactctgagc aagtatgctg gtcaatttaa
ctcatatctt
120541 aagcgtatag atgaagatca ctttattttg ttaacgcata tgcacgattt
agccaaaatg
120601 gaagataata agttttctat tttggacaag gttagaactg aatcaagtag
aaagaatatg
120661 ccgctgaccc tatcgattgg gattgcgttt ggatctgaaa gtttaaatga
gattgccgac
120721 caagcacaat ctaatctaga tctagcctta ggacgtggag gcgaccaagt
tgttgtaaaa
120781 caatcaggcc atgaagcccg cttttacggt ggaaaatcta atccaatgga
aaaacgtacg
120841 cgagtgagag ctcgaatggt ttctcaagct ttggtagaat tatttaaagg
cgttgatcac
120901 gtctttgttc aaggacatcg aaatccagac ttagatgcta tcggttcagc
gattggaatt
120961 gttaagattg ctagaattca tggcgtgaaa gctagcgtcg tactagatgt
tgaccacgtt

121021 aattatgatg taggacggtt aattgctaaa atgcaagctg ctggaattga taaggatgta
121081 tttatttcac ctaaagatgc tcttgaagca gcaacggatg agtcgctttt agttttaaca
121141 gatcactcta agtatagtat tacttatgat ccagaacttt ataagcgctt gaagaatcgt
121201 ttaatcatta ttgaccatca ccgtcgaggg gaagaattcc cagaaaaccc aatgctcgta
121261 tatattgaac catatgcatc ttcgacgtgt gaattagtaa cagaaatgat tgaatatcaa
121321 ccgcaaggcg gcgagggagt cttaactgat ttagaagctt cagcaatgct agctggtatt
121381 acagttgact ctaaagaatt ctccttgaga actgggacta gaacttttga tgctgcaagc
121441 tacttgcgat caattggagc agatactcaa gtcgtatctg agcttttgaa ggaaaatatt
121501 gataattact tgcaaagaag tcatttagtt tctactattg acatgattga accaaatatg
121561 gctctcttaa tgggagaaga taataagatc tatgatccaa ttattactgc ccaagctgca
121621 gatacggctt tatctcttga gcatgtagat gctagttttg cattaactag acgtagtaaa
121681 gatgtaattg gaatttcggc tcgctcaatg ggaaatgtta acgttcaagt aattatggag
121741 aaattaggtg gcggtggtca cttatctaat gcagcaactc aaattaaggg cgtaacagtt
121801 gaagaagcta aagataagtt gctcgctgca atcaatgaat atcttaagga aaatgaatag
121861 gagtgaaatt catgaaggtt atttttatta aagacatgaa gggtaaaggt aagcgcggag
121921 aagttaagaa cgtaccagat ggttatgctc aaaacttctt gattaagaat ggttatgcaa
121981 aagaagctac tagctccaac ttaaatacct taaagcgggt tcaaaaagcg gaaaaagatg
122041 cttatgaagc agaaaaagca gcagctgaag acattaagca gaagcttgaa gatgacaaga
122101 caattgtgaa cttcaagtca aaggccggta ctgattctcg tttatttggt tctatttcag
122161 gtaaaaagat cgttgaaggt cttgaaaagc aatacggtat taaggttgat aagcgtaaat
122221 tagagctccc agagccaatt aaatctttag gatacacaaa cgtacctgtt aaactattta
122281 aaggcgtaga agctgtaatt cgcgttcaca ttatggagca agactaataa tggataatgt
122341 tgtttctcaa caaattcccc atgatagtga agcagaaaag gcggtcctag gggccgtctt
122401 tttagatcca gaagccatta ttgatgcttc agatatattg caacctgatg atttctatga
122461 gcatgcaaat agaattgtct ttcaggcaat gcttaatatt tctgatcgtg aggaagtaat
122521 tgacccagta actctgcaag atgagttaaa gaaaaataat caggtagatg atattggtgg
122581 gattgcatat gtaacggagt tgtcaatggc aacgccaacg gcagcccatg ttacttatta

122641 cgctaagatt gtaaaacgta aggcaattct cagaaattta atttctacta gtcaaagaat
122701 tattcaaaat gccattgagg gttcagatga tgtaactgat atcttagatg acgctgaaag
122761 ccaaataatg ggtgtctcac aggataatgc tagtggcggg tttaagtcta tccatgatgt
122821 cttaaacaca accatggagg aaattaactc tattcctgat gatggaaata cagttacggg
122881 tttgccatca ggattttctg aattagataa gatgacgact ggttttcacg atgatgagtt
122941 gattatattg gctgctcgtc ctggtgttgg taagactgcc tttgctttga atgtagcgca
123001 atttgttggt ttaaaaactg ataaaactgt tgctatgttt tcactagaaa tgggagctga
123061 gcaactagtt caaagaatgc tggcttcaga aggattgatt gattcacagc accttagaac
123121 tggtcaatta actgatgaag aatggcgtaa attagttgtt gcggcaggct ctttagacaa
123181 tacgagtatc tatatagatg acacgcctgg aattaaaatg agcgagattc gcgctaaagc
123241 acgtaggtta gctaaagaaa agggaaactt aggattaatt gtaatcgact acttgcagtt
123301 aattgaagga ccacgtagtg aatcacgtca gcaagaagtt tctgcaattt cacggcaatt
123361 aaagaaatta gccaaagagc tacatattcc agttatcgct ctatcgcagc tctctcgttc
123421 ggttgaacaa cgtcaagata agcgaccagt cttatctgat attcgtgaat ctggttcgat
123481 tgaacaggat gctgatattg ttgcctttct ttatcgtgat gattattatc gcgatgaacg
123541 ggatgaagat gatgaaggtg aagttgaggc ggaagaagat aatggtgaag tagaagtaat
123601 tattgaaaag aaccgttctg gtacgcgcgg aacagttaaa ttgatgttct ccaagccata
123661 taaccgtttt tcaaaccttg actatactca taatgaggct taagaaaata atttgtaagc
123721 gtttcattaa ttagctatac ttgaagagtt aaaagtctat ttttttaaat aattgaatga
123781 gaaggtgtaa gttgaatggt agaaaataaa tatgtaggta gtatcgaagc tggtggtact
123841 aagtttatct tggcagttca agatacagaa agcggcgaag tggtagcaaa gaagagaatt
123901 ccgacaactg atgcaaaaga aactttagca aagagcattg agttctttaa agaacatcca
123961 gtatctgctt tgggaatcgg aacttttggc ccaattgata tcaatccaaa ttcaagaaca
124021 tttggttaca tcttagatac gcctaagcgt ggctggtctg gaacaaatgt taaaggtact
124081 tttgaaaaag aattggacat tccagttgta atgactactg atgttaatgc atcttgttac
124141 ggtgaatata ttgcgcgtgg tcgagataat tctaagactt acttttatgt aacaattgga
124201 acgggaattg gtgctggtgc agttcaagct gggaagttta ttggcctaaa taatcaccca

124261 gaaatgggac atatgttagt aactccttat ccaggtgata attacaccgg aaaatgtcca
124321 ttccacggta ataagtgtgt tgaaggaatg gcagctggac cttcactaga aggcagaacc
124381 ggtattcctg gtgaaaaatt acctcgagac cataaagtat ttacttacgt gtcttactat
124441 gtagcacaat tattgtttaa tgcttatatg actatgcgtc ctgatgtaat ggttgttggt
124501 ggatcagttt taaatgatca agatttggta caagtgcgtg gtttctttaa agaattcaac
124561 aataactatg ttgcaactcc agacgtggat gaattaattg ttcgtccagc tgttgcagat
124621 aatggttctg caactttagg tgattttgaa ttagctaaag aagctttaaa gaataaataa
124681 gtaatttata ttaatgttta tactaaaaaa gcaacttcag ttttgaagtt gctttttgtt
124741 tcacatgaaa catatgatta actaccatcg taagagtcat caacaattgg acctttgctt
124801 gagtcattgc ctagtggttc taagtgttca ttatggctaa gaaaaggaag attgatgtac
124861 tgacgagcgg ttgccgtaat gtcagagata atctcattag caattaaacg tgcgtcagga
124921 tcaagagcaa gataggcatg gctaaattga attgcaccac gaattttatt agttgttagt
124981 tgactagctt cagtaatttt ttgtgactct aaagcagtaa agaaggctaa ccagaaagct
125041 tcacgttttt cacgtggaat cttggtattc caagagataa aagtacttct aataattctg
125101 cttgcattgc ttaaacctgg cgccattaca aaactatttc gtgctacttc ccaatgtaaa
125161 ggattatgtt gcttcattaa atgtttcatt ctacttttaa ctacgataac gcgttctgga
125221 tggtcaagca tcattccaaa aattgatcct tcaggaacat aagtcatcat ttttggaatt
125281 acgcgttgaa attcaatctg gttatacatt tctttcatga aaccaggacc atcgaagcta
125341 taagccttaa ttactctgtc ttgaaggcta ggagaagctg cataaagtgc ataagcggca
125401 aaatttccac cttttgaatg gcctactgtg tagattttct tatcaggata ggcttgggcc
125461 attttatcta gataatctgc tgctacgctt tgaccaaaga tttctggttg ataactcata
125521 tcgacgtctt catttagacc taccatcgaa ccatcagttc cgcggtaagc cactagaata
125581 gtttctgaat ctagctcaat tgtcattgca gtgaactgga cgggtggctt tttctgagaa
125641 aggtgtgacc aatcgagaaa acgcatattt ttatagcggt cacttaagtt tactagttct
125701 gcctctaatt tatctgagtg aattaaggtt ttcttttgta ttaagcgttt tgtagctgcc
125761 tcaagcgtat gacctttaac actggaatca aaaggaagat aagcaaaagc agcaaaaaga
125821 cctgcatcta aactattaaa tggttcccga tctaatgtta gatcaccccg ccagcgaaca

125881 taatcaagta ttttacccaa gataaaagtc ctttcgttaa cggatatagt
actttcattt
125941 taatgctttt ttgattaaag acaaaagagg acgcttagtg ctaattagaa
gcaacaaaaa
126001 agaagagcta agttgctctt cttagtgatt aaaatattaa ttggatagtt
tcaagaggag
126061 ataaaggagc cttaattgaa gcgacattat ctgttacctc aatttttact
gcttcttttt
126121 caacactatt atcgcgtcgt cgaattaaag tagctgtatt agaaggtaga
ggtttactta
126181 aatgccagtt gatctcttgg taaccatcta agttgaaatt ggctagccag
acaattttac
126241 cgttttcttg aatgtttaat ttgacgtgag gcatattgat taaatagtca
attggttcaa
126301 cgctagccat aatttgttgc attaatcctt gtttataaga tgaaaattgt
gattcccaac
126361 cgtgttttgg atcttgatcc attggtaata caattatgtg atcatcaata
acagccctga
126421 agtttccgag cttttggtcg atcgaattat aggctgaact ccaaatctta
acattgctat
126481 ctgtttgata gtctaactgt agatagttgc ctgtatgttg cagcatcgta
atacgagggt
126541 tcttaacgcc ttgaacagtt gtgccatcag cttgttcaaa tgattgatag
ccactcttgc
126601 atggatgcca tttagcactt tgaatatgaa gtagattacc tagctttctg
tctagtaaaa
126661 cctggatact ttcaccgtca agtagaactg tatttttagt gattagatta
gtgatttgtt
126721 catttgaaag gttgcgtaat aattgaccag caattgcaat agtttgattt
tcaattgcaa
126781 ggtcattttg aacgggaaga atagttgtcg caaaaccgaa acttgctaat
aagctagccc
126841 aattcttttc atgtggaagt agctcttctg gttcttttcc aaaagttgtg
tgagcagtat
126901 aagcagagtc ttgatctact aaaactttaa taccacgtaa tttttgcata
ttaaggcgat
126961 ggctagctag ggcagaggca aaaggcttaa tgtcagctaa cattttgtca
tagccccaat
127021 cttcatttac gccattgccc atcatgtcaa agatattaag cataatgcct
ttggctccga
127081 ggagtgctgt ggtaataatt tgaaatgcga taaaggtatt agattttact
tgcggggtcc
127141 acattgaatt ttccaattct gggtaaagct cagcccgagg tcctaagaag
gcagccgtag
127201 ttcgactata ttcttcaaat ttacgaccat aaattaaagg agcaacttcg
ttataggacg
127261 gtaagtgtgg acgtgctacg cgaggatgtc caggacctgc ttgggcgtca
aataaactag
127321 gccagtctct accttcaata gcatgccaat caggaaacga agtcatttgt
cctaaatcag
127381 tattagggct cactttgtga actgcttgtt caattaagtg ttcgttttcg
atcatttcct
127441 tacgtgcttg atcaagataa atttttcgtt caatagtagg ttcgccaggc
ttaagcatat

127501 ttttaacaaa tcctgcacgg ctttctgttt tacccaattt ttcttgatat
agcttcatat
127561 ggtagtcaca aaaacacatt aactttaatg gagtatggtt atagtgccga
aagtcatctt
127621 ccatccataa acgacgtgga tgaatcgaag catattgagc atacctatca
gctaaatact
127681 tacgccaagt tggatctgcg ggacaagcca tatctttagc ttgattgcca
ttaatatcga
127741 caaaagtatt aaagccaatc tcaggattaa cgctaaatcc gcgatcggag
tgcataatag
127801 ttgtccatgg atttagactc gtagtaacat caagcttagc taattgcttt
tgtaggggga
127861 taatcgcatc aagccagact tgtgtttctt ccttagttag atgactgtgg
tttaattctt
127921 cgccattaat aataaaagcc acgtcatcaa ttttggcttt ttgtacaaaa
tctaaaagtt
127981 ttctatcttt ttcaggcgta ttagtacgtg gatcaaaaac ataacgtaaa
gtataaatgt
128041 agctcataat aaatcctcaa tattctatac aattaatatt caaaaaagtg
tgtaacttag
128101 tatattaagt cacacacata tcttagcata ttttgaaaac ggtttctata
atttatttt
128161 gcgccgtgag tagaattaat tcggcgaata ttttggtctt tttcaagagc
tagaataata
128221 tcattttcgt cttttctatt tctaattact ccgtcgacat taacaacaat
atgttcagaa
128281 gttacgtcaa taattttgat agaaatttgt tcaacttcgt ttaaggatag
ttcttttttc
128341 aattcattaa gaatatctgg atgattatta gcttcaattt gaacattgaa
acgaacatga
128401 agaataataa agttaatgaa tgtatcatcg tgaaaaataa tttggataat
gaataaaaag
128461 actgtgctga caattccgat gacatacatt ccagcaccaa ctgccatacc
gattgcagca
128521 gttgcccaaa caccagctgc agtagttaaa ccagaaattt gttctcgtct
aactaggatt
128581 gtaccggcac caataaagga aatcccagtt acaatttgag ccgcaattct
tgatggatca
128641 agcgaaatat catgtaacga taataggtca aagaaaccat atttagaaac
cagcataaat
128701 agagcagagg cgacagcaac aaccatatga gttctgatac ctgcactttt
tctttgagtg
128761 gcacgttcat agccgattgc tgttccacaa agagcggcaa ctaaaattcg
tagtaaccaa
128821 tctaattcag ttgaaagtga tgttaaactt accataatta atcccttcta
tataacagat
128881 acgtctttat tgtagcccag atttagttgg ataatcattt aagtaaaaag
taaaaaaata
128941 tcttgtacag aaacagataa tatggttaaa tacataattg tcatataaac
aggctgactt
129001 agctcagttg gcagagcacg tcactagtaa tgatgaggtc gaaggttcga
atcctttagt
129061 cagcattttt tatgctcata tttaattaaa agctatcaaa tcatttctta
aaatactgat

129121 ttgatagctt tttctttgct ttgaactaga ataaaaaggt ttgggagcaa gcgttaagcc
129181 tgtttttttg tgtttagggt atttgaaaag gattatcttc agtattagag ataatccttt
129241 tttgtttgag ctattatatt ttttgacacg ttcaagatga gataaagtaa ttaatttatt
129301 agccaattgt agtactaaat aattatcctt taactatttc ttctaaatta attgggttat
129361 ttaattgata atctgcttca ggaagttctt catgtgctcc ccaattggct aaagcaaatt
129421 tcactttagc agcgttagca gttaacatat catatttggt atcacctaca taaagcgttt
129481 cagtacgatc tacaccaagt ttacttattg cgaaatttaa tggttcagga ttaggtttat
129541 gattcttagt atcacttgca gtaacaatta catcgaaata atcatctagt ccaaagtgac
129601 tgacttcatt aatatactct tgtttagttt ttgaagtaac aattcctaaa gtaattccct
129661 cttgagatag agatttgata gtttcgggga tattattaaa taatgtatct tcattaactc
129721 ggttactagc tttttcagac caacttttag ccattttatt gatcaatact ttattattag
129781 taaaatgctt aatggcatct cttccaggaa ttcccaatgc aaaggctaaa tcgttaatat
129841 taacatttgc atttaaatat tctttaataa cctctttgaa tgtggaaaga acaacctttt
129901 gtgtatcaac aatagtgcca tctacatcga ataaaattgt cttaatcata atttactcct
129961 tacttttaaa gcggttacag caattatatc aaaagcataa ttatctgaat atgaaaaaac
130021 tactaatttc cttaatagca atagcgttta taattggcat catttttaat taatacacta
130081 gagaactgaa ataagtttac gtaatacgcc ttgttttagt tttttattgc tgtggttcaa
130141 tacgtaattg gatggtcaat gatttaacta atggagtatc agaataaagc ttagtattta
130201 tagatctacc tgtttgttaa caaaatatgt catatataat tgacaatttg aaaataaata
130261 ttatacttaa agtataaaaa gtcacatata tatgacttat aagaatgata gagagaattg
130321 ccatgaatag agttaaagaa tatcgaaagc aaaaaggctt atcacagatg gcattagcta
130381 aaaggataga tgttgctagg caaacaataa atttaattga aaatgataaa tataatcctt
130441 cattagattt atgtttgaag ttagcgcatg agttagatac agatttaaat acattattct
130501 gggatggaga tgtttaaaaa tgaaagctaa agaaactaga ttcgaaaaat atatgaaatt
130561 ttggtacgga atttctggtc cacttgatga gcagaaacgt caagttatga atcaaattgg
130621 aaatagggca ttttttattt tctttttagg gatggatgta gcatttttaa tttctctggt
130681 tttgttttcg atgtataaag ctgatgtggc atatcttttc ctttctggta gctttttgt

130741 aatgctaatg gcagttaact tatacataat gcagcaatct agaaaggcga gattgacaga
130801 taatgaagta gagaaagacg aattacctga ggctaaaaaa gctgcatgga caaaaggaat
130861 gatatcggga gcaatctttg gcatattgat gttaatctta gaattcttaa ctagttatag
130921 cgatggttcg tggacctgga taatgattcc tacatgtttg atttcaggtg ttttcttcgg
130981 tatctttatg tcgctaattg cagttcatag aatcaaagta gtaaaagacg aagattaaag
131041 aattagccat ttttaatgtt acattgttgc attaaaaatg gctttttttg ttgaaaaaac
131101 ttcacgtagc ttataaatat ttgtatcatt taaatgtaat cgtttgcata aaaatagaaa
131161 agataaagaa aatgaaaaca acaaaattag gaaaaacaag tttagatatt ccagctattg
131221 ctttaggcgt tatgcggatg gggactagaa ctccagaaga aggagctgct gcaattaacg
131281 cagcttataa agctggtgta aattttattg attctgcgga tatttatgga cgcggaaatt
131341 cagaaaaggt ttttggtaag gcgttaaaat tagccgatgt taaccgtgaa gatttattta
131401 ttcaatcaaa gactggaatt gttgtaggat caagtggaag ccatggatca ttagcttatg
131461 gctcacgcta tgaatttact aaagagcaca ttttggatgc tgttgatggt attttgaaga
131521 gaatggatat taattactta gactgcctag tcttacacag accagatatt ttgatggatc
131581 tagagggaat taaacaagcc tttgatattt tacaagctac cggtaaagtt agattctttg
131641 gtgtatctaa ctttaatcct gctcaattta agatgcttca aaatgcagta agtcagcctt
131701 taatgtttga tcaagtgcaa tttggattaa tgcacacagg aatggttgac tttggcttgc
131761 atactaatat gactgatgat gcttctatta accacgatgg tagcttactg gagtttttac
131821 ggatgaatca tattactttg caagcgtggt cgccatttca atatggaact tttgctggta
131881 catttatcaa taatccagcc tttcctgaat taaataagaa attgcaggaa attggagata
131941 aatatagcgt tggtaaaaat gcagttgcgg cagcttggat tcttaaatgg cctggtcagg
132001 ttcaggttat catgggaact atgactccgg ctcatattgt cgattctgct aagggtgcag
132061 atattgagct gactaatcaa gaatggtatg atttatattt tgctgcggga cacgatttac
132121 cgtaaaaaag ttttgctttt tataaaatca tctgatatac tagtagacgt tgtgagaaat
132181 gctgacttag ctcagttggc agagcacgtc attagtaatg atgaggtcga aggttcgaat
132241 cctttagtca gcacatgtga aaagtcctaa cctgtcggtt aggacttttt ttattgcctt
132301 ttttaacttg atataataag aggtagaaat atattacgaa agataaatgc tagaaaaggt

132361 aaaagcagat gaaattaagt caaaataaag aaactttaga tcaaatagca ccattagtgg
132421 aatatgcttt tttaaaaaat aatgatatac gtgaggatcc caacttttta agtcgttata
132481 accatagtat gggttttggt gaatataagg ataaccatct tgctagctat attatggtaa
132541 atgaatttaa gagtagaatt tttgctaaaa aagttaaaat gggcggagtt ggttatgttg
132601 cttcctatcc cgaaaaccgt ggtcagggcg atattaatcg cttgatgaag gagattattt
132661 tagaattaca cgatcaaaat tacgcaattt ctgacttagc tccattttca gaaacttttt
132721 atcgtcgtta tggctatgaa aatactattt atgaaaaaat gtatcaaatt cggcctgaat
132781 atctacgttt ctttaagcct atcaaagaag gaaaagtagt tcgtggaaga tggagtgatt
132841 catccctaaa agaagcagta atcaagttat atcaggctaa actcaatcaa ggtgaacaaa
132901 gaaatacagt tgaccgtgca gactggtggt gggatagatt agatacgtat tatcccggta
132961 gattaatttg tgtttatttt gataaattcc aacaaccaca ggggtatatg ttttaccgaa
133021 ttgttgagcg taacttccga gtagaagaaa tgtattatca aactccgcaa gcagctcaag
133081 ccctgttaag cattattgca agccacagtt caagcgactt gaaatactat gtgaaaatgc
133141 ctgaagaaag tttgttagga gaattcttcc cggaacaaga aggaatcact ataaagacaa
133201 taccttacat gatgactaga atcattgata tgaaacaagt gttagaagca ttgcgtttag
133261 ttaataataa gaaactgact attgaggtca ctgacgatga tattattgtt gaaaataatg
133321 gtatctggtt cgttaacgat gctgctgaag aaactagggt aagaaaagtc gaaacagatc
133381 cagactatac agcttctcta actaactgga ccaaagtttt attaggccac ttaactttaa
133441 aacaggcagt tcaattaggt tttgttaaag aaaatcatcc agttaatact gattttgtta
133501 agggcgaagt ttcattttac gattactttt aattaaaaat agttttttgc tgttttacgg
133561 cagtacctct tataagttgg tttcttttat tgattttaaa tttttaaaac caaatattat
133621 cctacttttc ttttttcatg ctaagctaag gtaacagata aaacacagag aggagaaatt
133681 ataatgaaag taggtattat tggagcttca ggcatggcag gaagtgcaat ttataagttg
133741 gctagtcaaa ataaagactt agaagtaaca ggtattgtta gagataaaga taaagataaa
133801 agagtcttag gtaaggatgc taagctttta attggtgacg tcctaacaat ggatgatagt
133861 ttgcttcaag aatttaatgt gattgttgac gcttttggta ccacaccaga gagggcaaga
133921 gatcaagtaa aacttgctac caagttgtgc ggattagcta agaataaaga tattagagta

133981 atcttcattc taggagctgg tagtcttaga actggaattg ataaccactt agttgttgaa
134041 gatattaaga aattaactgg ctctgaaaac tggattaata cacccaaaga gcagttgaat
134101 gagttaaact acttaaagca agttgaagat gtagattggc ttggaatttc accagcctta
134161 acctttgaag caggtccaga agcagcaggt tatatgttag gtaacgatac gctgctctac
134221 aacgataaaa atgaatctaa agtaacttcg ggcacaatgg cacgcttagt tgtaaatgaa
134281 attgttcatc cagagcacca taaggaaaga attactttag ttgatgaata aaaaaataac
134341 cgctacaacg ctcaggcgct atagcggcta tttttatgta atatttaatt attagcaact
134401 aatgaacgat aaaagttagc tttagtttga cggtagtaag gagtaatcca aataaatcca
134461 attccgcaag tgattaaacc aagtaggccc caaccaatga aacttaagtc taagatgaat
134521 aagtccatct tgtgtccatc cattaactta cggctttcag tgatagcttc agtagctcca
134581 atatcagtta gaccagctga aaatttatct ttcaaaatat atggcgtcat ggcataagaa
134641 taacccttaa taaagccagg aataacaaat aaaatggtcc ataaactaac gaataaatta
134701 gtcatgaata gggtaaataa actattctta aagcggccat tgctataagc gctaaaaata
134761 ccagaatacc aaggctccat cttgcctgtt tcaacaaagt ctaagattgt gtaggcaact
134821 ccccataaaa ttaagccggt aatcaagcct acaataattt ctgaaaggtt ataggctggc
134881 ttatatcctt gaattaagtt gtctttatta aagcgtagaa cagtataagt aaaatctttt
134941 ccagtccaga tccagcggtt aagatccata ataactgcgt taactaacca agcaaagata
135001 gttaggcaaa tagcccagcc ccaattgcca cgtagttgat cttttgcagc ttgtttcaat
135061 tgtgcacgtg tcatagtatt acttcctcta acaatatatt aatttctgat cattataatc
135121 ttaccactaa tctttaagta atttgaaaaa ctaaattctg ccgtcttctg ctataatcaa
135181 aattactaac ttaaatagaa gaagggttgc tacttgaaat ataaacaatt aatttttgac
135241 gttgatgata ctctgatcga ctttgcggct acggaagatt catctttgca tgcgctattt
135301 aaatcacata agctgcctct ttcttctgat ttgcagaagc aatatcatac ctataatcaa
135361 gggctttggc gtagacttga gcttggtgag attacgtatg atgagctaag tgagatgact
135421 tttcatgatt tcattaaaga gaattttaat ttagatgtag atggtaatga atggatgaat
135481 gaataccgtt cttactttgg cgaagcacac cagttattac cgggtgtgga agacacgctt
135541 aagtttgcta agaagcaggg atacaagtta actgtattaa gtaacggtga aaagtttatg

135601 cagcgtcatc gcttagagtt agcaggaatt aaggattatt ttgatttgat
tgtaacgtct
135661 gaagaagctc attactctaa gccaaatcca catgcttttg attatttctt
cagtagaact
135721 gaaattggtc cgaatgaaac tttattcttc ggggatggtt tacagtcaga
tattttaggt
135781 gctgaaaagt acggctttga cagcatttgg tataaccatc gtcatagaaa
ggatacaatt
135841 ggtcttcatc ccttatttga agtcgaaaca tatcctgaat ttgttaaaat
aatgcagaac
135901 gattttcaaa aagcatatta aaaggagtac ctagagcact ccttttgtgt
tatacactat
135961 tctttttcaa agccaaccat taaaccgcca tcttcagcgt agaaactgca
gagggcacca
136021 cattcgccga tcgttacttt agcatctggg aagttatcta agataatctc
tttcatgctt
136081 agggcagagc cttcattttg aacgtgatca atttgaacac ggccaccttt
atagccggct
136141 ttgaccattt catctaagag ttttcttcta gctttcttag caccgcgtgc
cttacccaaa
136201 agttcaaatt cacctttatt gttggcagtt ccgataacga aaagcttcaa
taaaccagca
136261 atcttagcga ctgctggtgg gatacgacca ttattgccta gattagttaa
gtcttgcaag
136321 gcaaacatta aatcaagctg gtgttggtac tcttcagtgg ccttgacaat
ttcttcaaat
136381 gacttgcccg catttgctag actagcaatt ttctcagcca gtaatctcaa
ttgaggacca
136441 gctgagcgag aatcaaatac taagatctta gcttttggat tcttttcatg
ataaatctta
136501 gctgcttgag tagctgaatt ataactaccg gaaagggcgc tagttaaagt
aattacaaag
136561 atctcgtcgc taccttcaaa agatttaagc cagtcgttta cattgggaca
agcagaagta
136621 gacttgctct tagtgtgttt tagagtatga agaaacttat ctaaatcaat
atcaggcgta
136681 tccatccaaa gcttgccatc aacgtgaagc gataaaggaa ctgtaatgtg
ctcaattccg
136741 tcaatttgat cagctgtgat attcgcagtt gaatctgtga taatttttat
tggcatatag
136801 attacctcga attaaatata ttagcctatt ttctttcagt ctagagaaga
tagatagtaa
136861 agtcaaagaa aatgctagca aaaaagacag cactgaggct gtcttgatta
acgtgaaaat
136921 actaggccca gaagaccgcc aagtgcaatt aatgctaaaa gcggcaatac
taggtaagaa
136981 acgaaggcaa atagcaagcc aacgatgatt aggaaaccaa cgatttttag
catgccaatt
137041 cctagtttcc agaaaagcca aattattaat aaggctaata aaattgaaaa
catattctaa
137101 accttcttat aaaaaattaa actatatttt gatattaatc atattacgaa
attgagagaa
137161 agtgtagata agtttttatt aaggatagga gatagtaaaa tatactaaat
aaaccgcttt

137221 catattttgc aaaaacgtga tatattaatg gtgtgaaata gctcaagtag aagtcacgat
137281 taagattgtt agaagttata ttttttatta attaagtaga cgatactcta gctattgcaa
137341 ttcataaata taattcataa gtaaaaggaa gttctctttt gaacttcctt tttttgctgt
137401 aatgcttgct ataataatac taaaaagtac tatctataat ttttggaaaa taaactaatg
137461 aaaaaattaa caataggaat tctagcacac gttgatgcag gcaagactac tttatcagag
137521 ggcttgcttt atcaaagtgg tacgttaaga aaattaggcg cagttgataa aggaactgca
137581 tatttagaca gcgataattt agaaaagaaa cgcggaatta cgatcttttc tcatatggcg
137641 cacattcaaa atgaaaatag cgagttgctc ctcttagata ctcccggcca cattgacttt
137701 gcccaggaaa tggaagagac gcttggcgta ttagactatg caattttagt tgtgtcggct
137761 agtgaaggcg taacagctta tacgcagacg ctttggaact tattgaaaaa tcataagatt
137821 ccaactttta tttttgttaa taagatggat acattaaagg ctgataagga gaaggttctt
137881 catgacttaa gtatactcga tgacagctgt gttgagtttg gggatgaagg ttctgatttt
137941 tatgaaaaaa ttgctaccgc agatgaggca attttagaag aatatctaga atcgggtcag
138001 ataaaagacg aagaaatcaa agatttgatc tttcaaagaa gaattttccc agtgtatttt
138061 ggtgcagctt taaaattaaa aggcgtagct gaatttttac agggactaga taagtggact
138121 aagaaaatag agtatcctga gaaatttgct agtcgtattt ttaagatttc tcacgatgag
138181 aaaggcgaac gcttaacttg gcttaaagtt actggtggtg aattaagagc caagactgag
138241 ctaatgcctg atgaaaaggt taatgaaatt cgtctctata acggtactaa atatcaagta
138301 gttcctagtg ctcaagctgg cgaaattgct gctattagtg gcctgaaaac tacctatcct
138361 ggtcaaggct taggctttga aagagatcaa acgaacttta caatgcagcc agttctaact
138421 tatgcagttc agactgtacc ggataagatg catgcgacgc taacagctct aagacagcta
138481 gaagatgaaa atccgcaatt gcatgttaag tgggacaagc aagcagaaga gattagcatt
138541 gatgtgatgg gaaagattca gttagaaatt ctccagcaga ttttgcacga tcgctttggc
138601 ttaaaagctg aatttgctca aggcaaaatt ctttatcaag aaagcattaa agattcagtt
138661 gaaggagtag gacattttga acctttaaga cactatgcgg aagttcactt attgttaaaa
138721 cctggtaagt taggcagcgg cttagttttc aaaaatgagt gtagtctaga agtgctgcct
138781 aagaagtggc aagaccaagt aatggatagt ctagcaaata aagagcatct aggtgtttta

138841 accggttctc ctctaactga tatggaaatt acactagttg gcggtcgcgg tagcaatgta
138901 cacacagtgg gtggtgactt tagagaggca acctatcgtg cgctgcgaca aggtttaatg
138961 gaattaaaag cacaaaatca agtatcttta cttgagcctt ggtatcaatt tactttaagg
139021 atcaatcggg aacaggttgg tcgtgcaatt aatgatatcg agaaaaaagg cgggaaattt
139081 gaaatcggag aaagcgatca tgatgtcgtg acgattactg gtcaagcgcc agtagcacag
139141 atgcaagatt atgcaactga agttaggaac tacacgcacg gcagtggtca acttgagtgc
139201 ttatttgcag gctatcggaa atgtaaaaat agtgctgaag taattgagtc aattaattat
139261 gatcctgttt ctgacattta caacacccct aattctgttt tttgctctca tggcgcaggt
139321 catactgttg tttggaatga agtgccagat tatgctcaat atccgtattt acaatcttaa
139381 gattgcttac gatcgactaa aaagatagac aaaattattt caagtaattc tttttttcgca
139441 aaagcaaagc tgaaataatg gctaaggcag ctccaaataa gattacccat aaaaatccaa
139501 attcattatc cttacctggc aatccgccaa cattcatccc ccagatgcca ctaattaaag
139561 ccggaattga gataattaga gcaattgaat caagatattt catcaaattg tttaaatgat
139621 tatccatcat tgccgagaaa agtccattaa ttgattctaa aagatcgcgg taaactttta
139681 ttttttagt aatccggcgc tgagaaagta gtagatcaat gataaaaatt gagttggccg
139741 aatgctgctt tttaagaaag tcagttagag cttcaataga agaagcttgg tcatccattg
139801 tgtgattaaa atagaccaaa gttctggtta agtccgttgc ttctttcaaa gacttagttt
139861 tggtagtagt tcttgctgca tgatctaact gatcaagttg ctgctttatt ttagttaatt
139921 cttcattaaa gcaatgattt tgataaagta gttgcttaat taaaagatct tcaactgata
139981 gtttagcaat tgccttttga ttttctaaaa taggtgctgg cttttttttgt aaaactaaga
140041 ttagttgctg agaattgaag atcgcaattg tgggataaat cgcatcttca acttgagttt
140101 gaccagcgtc aaaattaaag gatacgaaaa ttctggcatt ttcaatttta catttgggca
140161 agacgttaaa atgtgatact tcagtagcgg acgtttgttt agtaaagacg tgcgcgtcac
140221 acccgattgt tttttctata ttggttaatt cttcagaagt aggattaact aaaataacaa
140281 gtttagctga taatagattg tctgatgtta tgtagccatt tttatcataa acagtaagca
140341 taagaaaacc tcctgactat tctttttttag ggtaaatata tcatagaagg cactataagt
140401 tagatttaac gccttacagt tgttaaaaat gaaaacgata tacgtacaga aaaattaatt

140461 tataattaat gtacatagca agttattaag gaaatattaa gcttgtcgtt ttggagatgc
140521 catagtatct ttatcgacta gtgcttgata tgattaggtt aatcagtcat gaggattgaa
140581 gatagctagc taaaacaatc ttttatgtgt tttaggaaag gatacctaat tatgatgaaa
140641 gataaacatg gaattaaaga caaagtagca ggtaaattta aagaagtaga aggaaaagtc
140701 actggcgata aggctcgtga attagaagga aaagctcaac aagctaaagg aaaagtaaaa
140761 tctaaggcta ctaaagtaaa agaagactta gaataaatta ctactaaatt agaaatagga
140821 ggcgatcccc atgcttcact ggatttgggt tttgattgtc ggaggcgtaa tcgggttaat
140881 tgcaggagcc attactcata agggtggctc aatgaactgg attgcaaata ttattgctgg
140941 gctagtaggc tcatctctag gcgaggcctt gcttggtgcc tggggtccgc aagtagcggg
141001 aatggcaatt gttccatcac tcttaggtgc tattattcta gttatcttgg tatcatggat
141061 tatgacgatg ctatttggcc ctagggctag cgaataaatc atatttaaca caattaaata
141121 atctctctca aaagagcagt cttagcaggc tgctcttttc tatggtcggc gttttgagtt
141181 ttcatagtat aatagatgtg atataaaaat ctaggtaaga aaagagataa taatgcgttt
141241 aatttcatgg aatattgatt cgctaaatgc tgctttaact agtgattcag cacgtgccaa
141301 gcttacacgt ggcgttttaa atacaattaa agaaaaagat cctgatatta ttgccattca
141361 agaaactaaa ttacggtcaa ctggtccgac taagaagcat caagaaattt tggccgaaat
141421 gtttcctgac tatgattatg tttggcgctc ttctgaagaa cctgcacgaa aaggttatgc
141481 tgggacaatg tttttatata aaaatacttt gactccagaa gtaactaagc cagtaattgg
141541 ggcgccagaa ccaatggatc atgaaggccg aattttaact ttagagtttg ataactatta
141601 tgttacccaa gtttatacgc caaattctgg aaatgaattg aagcgattag atgatcgtca
141661 ggtttgggat gaaaagtata cagagtattt acaaaaatta gatcaagtaa agccggtaat
141721 tgctagcggt gactataacg ttgctcatac accaattgat ttgaagcatc cagaaaataa
141781 tcatcataac gctggtttta ccgatgagga acggcaagac tttgataagt tgctcaagct
141841 tggctttact gatacattta gaaaaattca tggcaatgtc gaaggagttt attcttggtg
141901 ggctcaaaga gtgagaacca gcaaggctaa taactcgggc tggagaattg actattacat
141961 cgtgtctaat agaattgcta atcaggtaac taagtcagaa atgctcgata ctggtgagcg
142021 aaaagatcat tgtccaatta tgttagaaat taatctgtaa ttatccactt gtggataaat

142081 caagaagatc caaatgatct tcttttttag tttaggaatg gaagtgaaag ctgtggataa
142141 cgttttaaaa gatgcaattt tgaatggctt gtataacaag aatgaaaata aggggaatga
142201 gtttatcagt cctcagttag taaataatga tgatcaaagt aaaatttggc ttactttgcg
142261 gcaggaatta atgtcatgta cctcttttac ctgggccgtt gcttttattt cagagaatat
142321 gcttgttcct tttaagttag taatgtctga actagctaaa aaaggagtta acggtacttt
142381 aattacagga acttatcttg gctttaatag tcctaaagtt tttaaagagt taatgaagat
142441 tcctaatcta acggtacgct taagtgagaa aactggtttt catgcaaaag gctacatttt
142501 taatcatgaa gattatcaga cgattttaat tggaagtgta aattttacta gaagtgcctt
142561 attaaaaaat tgcgagtggg gattaaaagt aacttcgcat gaaaatgggc aattggtcga
142621 agaagttaac gatcaaatta aaaataattt aagtaatagc ttgatactta ctagttcttg
142681 gattaaggaa tacgagcaaa attggcaacc gatcaaaagt aatccgcatc ttctattaag
142741 aaaatccaag aaaatagcac ctaaccaaat gcaaaaagaa gctttagcga atttagatgc
142801 tttagtaaaa gaacaagcaa aacgtgccct tgttgtttca gcgacaggaa ctgggaaaac
142861 atatttgggt gcttttgcag ttaaagaata taagccaaaa aagtttcttt atttagttca
142921 ccgagaacaa atagccaaaa aggctttaga gagtttttat caagtaattg gtggcaagaa
142981 aagcgactat ggcttattaa ctggaaataa acatgatttt gataaaaaat atctttttgg
143041 aacggttcaa actgtaagtc aggaacaaat actcggacaa ttaaatccag aagaatttga
143101 ctatatttta attgatgaag ctcatcgagt ggcagcgcca acttatcaga agattttgaa
143161 tcattttata ccaaaatttt tattgggaat gacagcaacg cccgagcgaa tggataagca
143221 aaatatttat gaaatatttg attatcactt agcttacgaa attagactga aagatgcctt
143281 aaatgagcag atgctcacgc cctttcatta tgtgggcgtc gaagattata cagtgggaaa
143341 ccagattatt actgaaacta gtaaattaaa agacttagtt gctgaaaaac gagtagagta
143401 tgtacttaaa cagcttaatt actacggcta ttgcggagaa aaggcaaagg gcttagtatt
143461 ttgcagtcgg caagaagaag caagagagct ggcaaaatta tttagtcaag ctggtcatcc
143521 gtctcgtgcg cttactaatg aggatagtca aaaaaggcgt gctgaagtaa cgcagcagtt
143581 agaaaatggt gaattagagt atatttttac cgttgatcta tttaatgagg gaatcgatat
143641 tccgtcgctc aatcaaattg ttatgttacg aaatactcaa tcaagtatta tttttattca

143701 gcaattaggt cgaggcctgc gtaaatttcc gggaaaagat tatgtcacag ttattgattt
143761 cctgggaaat tataaaaata attatctaat tccaattgct ttaaatggtg ataaaagtcg
143821 tgacaaagat caagcaatta gagaaagtaa attaccacag gtaattgatg tttcaacaat
143881 tacctttacc gagattgctt ctcagagaat tttagcttcg cttgaaaaga taaaactaga
143941 cagtatgcat gaactaaggc agtcgtatga agatttgaaa gaaaagattg gtaaagtgcc
144001 actcttatgc gatttttatc aatatggtac gactgcgcca acggtttttg ctaaaaatca
144061 tgctctagca cattatggtg ctttttaaa gaagatggga gaagaggtaa atttagataa
144121 ttatcagaat gcagttctat cttttgtaac caaggagttg ctattcggaa agagaccgca
144181 tgagttgcta ctcttagaaa gctgcttaaa taaaaacatc agtaaagctg attttattaa
144241 attacttaag aacaaaaatt gttatgtcag taaagatgta cttgagtcgg ttgagaaaat
144301 tctgtcgctt gaatttttg atgttaagca gggaaaagct accaagaaag agcaatatgg
144361 aatttatcct ttaattgaaa aagaaaatgg agaatatcat ttatccaatc aattatttca
144421 ggcattaaat gataatcagc aatttaaaat actatttaaa gatgtgctaa agacaggttt
144481 agaactgtca aaagaatatg attcacagtc tcaatttact ctgtataagc aatatgatcg
144541 taaagatgtc tgccgtttat taaattggcc taaggatgtt tcggctccaa tgtatgggta
144601 tcgtgtggga gaaaaagaga caccaatttt tatcacttat caaaaagact ctaagaaaaa
144661 gcgtaatgct cgctatcaaa atactttaga aaatggtcat agtttacgtt ggtataccg
144721 cacaccgcgt catttagatt cagatgaagt gcagcgcttg ttatatactg atgagatgaa
144781 attacaccta tttgtgaagc gaagtgatgc agcaggaaaa gaattttatt atttaggaac
144841 agcagatatc caaaaagact cagtcaaaga agaaaaaatt ggacttaagc aaaagagtgc
144901 agttggtatg aacttagttc tcaatcatcc tttaaagcaa tcaatttaca atttgctttt
144961 tagttaggaa attaaacata gtttaggata aattataaaa atacataata attatttgat
145021 aaattttaaa ttgcaaaact ggaggaagag agaaaataag attgaaaaat aaactagttt
145081 ttgtactggc tttattagtg attgttttga ctggttgtac tactcaagac aaaccaaatc
145141 aggcagatgc ggcacctaag tactatgttg aaaagcgtca aacacctaag acaaacttga
145201 atgtgatacc aactctattt ttccatggtg gcttaagcaa ttacaagggc gaagaaaaca
145261 tggtaaaagc agctcaaaaa gcaggagtta ccaattcaat aattagagct aatgtagatg

145321 caaatggtaa agtgaaatta attggcacta tttctaagaa tgctataaac
ccaattgtag
145381 aggtaaatta tcgcaataat gttcagctgg attttaaaga gcatggtcga
tatgctagaa
145441 atgttgtgca gactttacaa aatgagtacg gaataaaaaa ggtaaatatg
gttggacatt
145501 ccctgggaaa tatttcgatt atgtattatt tgttgcaaac tgcgcatgat
cctgaaatgc
145561 ccaaactaaa taagcaagtt tcaatcggag gacattttga tggacttgat
tttaaacaat
145621 taccaattgc aattcgccag ccatcgaatt tacatgttga tgcagaagga
aagccaaata
145681 agaccaactc tacttatcgt gagatgatga agttaagaac tctttatcca
aacaagcaga
145741 ctagtgtttt aaatattatt ggtaatattg gaggtaattc agatggaatt
gtgaagaatg
145801 cttcgtcact ttcgcttgaa tatttggttg aaccaatggc taaaagttac
cgagtagtta
145861 aaattgtcgg taagaacgca gagcacggac agctaactta caataaacaa
gttgaaaaag
145921 aaattattaa gtttttatgg ctccagtaaa tttgaataat caaaaaaaag
atgctttgat
145981 gcatcttctt ttttaaattt tattttcatg ttgattactt ttatcaaaat
tatggagcca
146041 catgccccaa gcggcttgaa attcaataat taatacactg gcagaaataa
gtaccatgat
146101 aattaaggca taactagccg tttcacctaa ttcagatgta tcgaaattgt
aagtaaagag
146161 cttaattcct ccatagataa gtagaataac ccaatatata tttgctcccc
accataactt
146221 tttcattaca gataccgctt tcatttacta tataaagcta ttataccatt
cttatggtta
146281 attaaaagtt tggtgcatag ctaaagcgcc cttaagaagt tgtataataa
agagaaaata
146341 gagaggtgaa gtgaattaga tgggacaagc ttgggaaaat tttaaaaaga
atacaaacgt
146401 gcatcgcctt gccacgttat gcttaattat tttggtatta tatgcggcac
gttcaatgat
146461 gaatacaatt ttattaacgt ttattttcac atatttaatt gtgcatttaa
ttcgttttac
146521 gcaaaagaaa ttaccaaact tgccctcgca agtaattgtt gtgttagctt
atctattagt
146581 aattgcgatc atttactttg caattaccat atatgtgcca atattaatta
aacaaattgt
146641 taaaatgagt cattctttaa tgaagtttta tcaatctgat aatatggact
ggttaacgca
146701 atatttgaat cattacatta gcgatagtga aattacaacg caggcaaaac
atggtgtaac
146761 tatcttagtt catgccttaa ctaatgtggg cacattaacg attgcgttct
ttatgtcact
146821 aattatgagc tttttctata ctattgagct tgattctatg aacgaatttt
ctcatacttt
146881 tttgaatagt cgccatttat cctggttatt taaagatatt gcctactttg
gcaacaagtt

146941 tgttaatact tttggtgtgg ttttagaagc tcaattcttc attgccttat gtaatacagc
147001 aatgacaatg atttgcctaa ttattatgaa aatgccgcag attattgctc ttggattaat
147061 ggtctttatt ctaagtttga tcccagttgc tggtgtaatt atttccttga ttccgttaag
147121 ttttgtggct tattcggtag gcggaattag gtatgtaatc tatatcttta ttgttattat
147181 gataattcat gcaattgaag cctacatttt gaaccctaag ttcatgtcaa gtaaaacaga
147241 attaccaatt ttctatacct ttgtagtttt gcttgcaggt gagcattttc ttggaacctg
147301 gggactaatt gttggggtac caatcttcac cttcttgttg gatatcttag gagtaaaatc
147361 agttaaaacg aaaaagccaa aaatcttgaa aattgaaaac aaaaaataaa agcagctgtc
147421 aaagctgctt tttgcgtatt gtaaatagga ttgtaggtcc caaatgagaa tatatgaaaa
147481 atacgagata tgaatggagt ttttaatttt gctattcaca atacaatatt aattacgcaa
147541 aaaaatcaaa ttttttgtta tttcttataa aaatttttaa gcgctaataa tagcggtttc
147601 atatcttcct ttgttaaact aaaaatgaaa gaaagatgtg aggcaatgtt atgaaaaaat
147661 atgattatat aattttagga acaggtcctg cagcatatca atttattaag ctcttagcta
147721 cgcaacataa atcaatttta gttattgaaa gtggcttatt tggtggaacc tgtcccaatg
147781 tcggttgtga accaaaaatc tatttagatg gtgctgttca agctgcactt cttagcaaac
147841 aactagaagg acagggtatt gaacaggctg cagcgatgaa ttggtcacaa ttaatgaaag
147901 agaaaaagga acgatttact agttggccgg atgaaacgaa ggaaatcatt ggtaagactt
147961 gtgatgtaat tagtggaagt gcgcatttta ttaatcgaca aacaattgca gtaaatcaaa
148021 aatatttcca gggaaataag attattatcg caactgggag aagaccgcat gaattatcaa
148081 ttccaggagc aaagtttttg catgatagtt cagatgtatt atctttaaat aaagtcccag
148141 aacacgctac ttttattggc gcgggttatg tagcgatgga gttggctacg ttcttagctg
148201 cggcaggcag tcaggtaacc attttggtta gaggagagca agtattaaga catttttatc
148261 aaaagtatag ttctgaccta gttaatcgaa tgatgaagcg cgggattcaa tttaggtttg
148321 ttactgaggc gactcgcatt actcaattaa gtgataaata cgttgttgag ttaaatcaag
148381 gtagttccct tgtaactgat tacgttgtga atgctagtgg tcgaacgcca aatattgaaa
148441 aacttgactt atctgctgca cagattgact attcacctaa gggaattgag gtggatcgac
148501 atttacagac aaacgttaat aatatttatg cgatcggtga tgttacaagt caagatgtac

148561 ctaatttaac tacggtggca gaatttcagg cacgctatct tttcaattct
cttgaaaaag
148621 gtttaagtca gccaatcgac tatcctgcaa tcggaaccgg tgtattcgcc
tttccgcaat
148681 tagcccaagc tggggttaat cctgatagcg tttctgaaga tagagataat
tttgaaattg
148741 tagaatatga actaagtcag agcagtttat atgctggtca aagagaaaag
ggtctgctaa
148801 cggtagttta tgataaagct aattatattg ttggagttag cgaaatcagc
acaagtgcag
148861 taaatgatgt gaattatttt gttcctatta ttggattaag aatcaggaaa
aacgaatggc
148921 atcgaaatgt cttgccaatt tatccagcct tagctgataa gattgaagga
attttgagat
148981 agaaaaaatg gcaccattaa ggtgccattt aattatgaat ctaattttta
caagtcgctt
149041 ccaccgtagt taggagcttc tttagccatt tgaacatcat gtgggtgaga
ttcgcgaagg
149101 ccagcgttag taatttgaac aaattgactg ttttcaatca attcttcgat
tgtaccagca
149161 ccgacgtagc ccataccagc acgtaaacca ccgtcaattt ggtagataat
atttgaaacg
149221 tcacctttat aaggaactaa agcttcaaca ccttcaggaa ctaacttgtt
tgcttcgtta
149281 actccgcctt ggaagtagcg gtcagaagag ccatgttgtt gtgacattgc
tcctactgaa
149341 cccataccgc ggtaagactt aaatttttta ccttcatttg tgaaaattgt
accaggtgct
149401 tcagtagtac ctgagaacat tgaacctagc attactgcat ttccaccagc
agctaaagct
149461 ttaaccacgt caccagagta cttaattccg ccgtcggcaa tgatcttctt
gccgtatttt
149521 tgagcaacat tagcagcatc ataaatagca gtaatttgag gaacaccgac
accagcaaca
149581 atacgagtag tacaaattga accaggtcca attccgacct taacaacatc
aacgccagcg
149641 tcaaataagg cagcagttcc ttcgccagta gcaacatttc ctgcaatcag
agtagcttta
149701 ggaaagtgat cgcgaatttc cttgatttta cgtagaacac cagcagagtg
accgtgtgca
149761 gtatcaataa caattgcgtc agcacctgct tcaagtaagg cttcagcacg
ttcaaaagta
149821 tcacttgtga cgccaacagc tgcggcagct aaaaggcgac cttgatcatc
aactgcaggg
149881 tgagataagt taggatcaac agccttagtt ttagcctttt taacctcttc
aacctgggct
149941 tcgattgaaa ggttcttgtg aataacccct aaaccaccat tttcagccat
ggcaattgcc
150001 atatcacctt cagtaacagt gtccattcca gcagaaataa gtggaatgtg
aagttgaaga
150061 ttaggcgcta attttgtatc aagtttaact tcattaggca gcacatgact
ttcagctgga
150121 attaaaagta catcatcaaa agttaatcct tttttggcaa attttgtttc
ccacaaactc

150181 attgtaaagt cctttctata gaagcaacgt tttaatatta aattagctaa actttactcg
150241 aatctgagcc agaagtcaat tggaaatttg atttattttt ttaacaatta gattggctag
150301 taacttataa ccattgacgc cgaagtgaag accatcattc ttttttccgt tgaaaatttc
150361 gggaaagttt atattttctt gcattttact ggcaagatca ataacagaaa atttatattt
150421 gccagctact tttttaacta tattgctgta ttccataact aaacggttgc ttcttactct
150481 ttgtttattt tcatcaactg ctggtggtgt gataaagata acttttttctg gataataaag
150541 ccaaataatg cttgaagcaa ttaaatctaa attttttttca aattgacgaa gagggacttg
150601 cttatgcgtt gctaagtcat tagtgccaac taaaataatt agataattac acttaggctg
150661 attaaagacc aggctaggta gggaaagggc aagaccacca gaattaattc ctgatattgc
150721 agtgttgtaa atatcacaag aaagaatttt ttgtagctga aaattgagtt ctggaattga
150781 gtggttttca gagcgggcaa taatgctgtc cccagttaga agtaatttca tatcaaaatc
150841 ctttgggtag aaaaaagacc ttgagaaaaa tctcaagatc ttttctaatt ataaataatt
150901 tgatctttag aacttgttct tgtctaaagc aactggtgaa tcaggctttt caccgttgat
150961 taacttcaag ttgttgtcaa atgccttaac aaccatgtta cgtactgcgt gagtagtgta
151021 gaaagcagta tgtggagtta ctaaaacatt tggacgatca attaagtcag ctaaacgagc
151081 atctgggaat tctttgcctt cccaatcctt gttaaatacg ccaacttcgc cttcgtaagt
151141 gtccattacg aaaccaaaga tcttacctga gtctaaacca cggataacag catcagtgtc
151201 aacaagtgga ccacgtgagc agtttacgat tacaacgcca tctttcattt cagcgattga
151261 cttgtcgttg atcatgtgaa cgttagctgg aacatctggt acgtgaagtg aaattacatc
151321 agcttgcttg tataagtcat caagtgagtc aacgtagtaa cccttctttt caagttctgg
151381 gttcttgaag atatcgtaag caataacctt tgcgccgaaa ccttccataa ttctcataaa
151441 tacttgacca atgtgaccag taccaacaac accaacaact tggtcacgaa cttcacgacc
151501 aatagtaggt gcccagcgta agtcgcgctt agccatcttt tcatccatac gcttgtcttg
151561 acgtagtaca cgagcagctt ggattgcagc atgttcagca attgcgtcag gagagtatac
151621 aggaacgtta gtgatttcaa agcctaattc cttagctttg tccatgtcaa tgttgtcaac
151681 accaacgtta cgtaatgaca tcttagttac gccagcatca gctaaagctt gaagagtttc
151741 aggagtgtag tctaattgtt ggtaaacaac aacaccgtca gcacccttag ctaacttagc

151801 agtttcagga gtcaaaagct tgtcagtgta ttcaacttca acatccttgt gagcatcttt
151861 ccattcgttt aagaaaggtt cttcatcttt acgaatagcg taagcaaaaa tctttgtcat
151921 aaattcaacc tccgtcaaaa ttaatagtat gaccgtcttt tattatacac ttgactttgt
151981 taataaaaac acaaaatcga atttttttga aacttatttt aaagatgttt catgtgaaac
152041 gataattgca gttaaaaata gattttcttt tgcgtatcat tatagggtgc cttatgataa
152101 tgattagaaa aaaagaattg gaagtaaagc tgctaaaatg ttattaaaaa tcagtcaaaa
152161 cagatgaggg aataagtaat gtcagttgtt gtgaaatata tagatttaga taataattca
152221 gctgaattgg caagttcaga agaattgaat ggaaaagcag gagaaagaat taactatcat
152281 acagcagcag aagtccaaaa gctaacagct gctggttatg tattgattaa taattcgttt
152341 gatccaaata atgaagtaca ttttttgat gaaacttctc aggaatacaa gctcactttt
152401 aaacatagtc aagaagaagt aactgctaag aatctaaaat atgattgtca attaaatgat
152461 gtacaactta aaggaaagca agtagttcat tatacaggcc ttgaaaaaga tattcctgac
152521 aatgtagttg aagtcacttt taataggaaa attatttacg acaaagttac taagcagaaa
152581 gtttcactta gtgcctggca accagaaaag tattatttgc ctttagttgc tactcctgcc
152641 gttttagatt acacgccaga taaagcggta attggcggag aagctgttac agttcaaaaa
152701 cctaagcatg agtatgtcgt tacttatttg cctaataaga aaaatgctag aaggcagaag
152761 gctgaaatca agtttattga tattgatgat agtaatcaag aattagctag ctctggcgaa
152821 ttaaaaggta aacccggcaa agaaatttct tacagtacga cagaaatttt gaaagaactg
152881 acagataaag gatatgaagt cgttactaat gactttgatt ctaatgacca gaagcctgtc
152941 tttggaaata gtagagatta cattcaaacc tttattattg ctttaagaca taaaaagcaa
153001 gccgttaatt ctgatcatct ttcttccgaa attgaccatc gtctttatga aaaagaagta
153061 catcgccgaa ttaccttttc aggtttaaac ggtcaacagt tagatgatat agttcaaact
153121 gctgtcttaa aacgtagtct aacaatggac atggttacta aaaaaattat cccaggcgaa
153181 tacactagtc agtggcaaag cacagaaaca tatccttcag tggccgttcc tgttgttgca
153241 ggatatcata ctaaaactaa agaagtagta gcttgtcccg taatagaaaa agacataagt
153301 gaagaagtta agtattatgc aaatggttat ttgattccag ttgatgcaaa tcataataag
153361 ctttctgaaa tagataagaa gcaattaatt actaattcta ttgatccaac gaaagcaatt

153421 tttcctaaat tagagcttga aaatgtcgtc ttaaagccaa ttaaagaggt aaaaattgaa
153481 gatccggggc gagattatga ggttccatac ttattagttc ataaatatat tgccgttgat
153541 gagaagcatc ctaaaaagga agtaagtcca gcatattata gaagaattgt gacagctcgc
153601 gttcattatc aaggtgctgg cgatttgact cctactgacg cagaacagac agttacctgg
153661 acaagaacag taacctatga cgaagtaagt agagaaatca ttgataatgg aatgtacacg
153721 acaaaatggt tagctgataa agatatcttt gaagcaaccc ctacgccagt tattaagggc
153781 ttttgcgctg atattggctt aattggtgaa catccagtta cagaaacaga cttgatggcg
153841 actgttactt atgcgccagt tggaaaaatg atcccaattg atgaacatgg aaatgaaata
153901 aaaaatgcca tgcgatcacc atatattaat gatccatatg atccagtacg ggtacttttt
153961 accgaggaag ttccagaagt acaaggatat gctccggtta acaatacaat ttcggttaat
154021 gatccatttt ccgataccaa ggtaacttat accttgaagc cccgttatat tccagtgaat
154081 agtgaacacc catatcgccc gatcaagcca acgatgtata gtgtgccagt taaggaaaca
154141 gtcaaatatc agggagcagg agagcaaaca ccaacggctc gtttgcaagc tgcacgatgg
154201 actagaacat taacagccga tgaaaatacc agtgaattaa tagacaatgg taaatataca
154261 acagcttgga aagttgataa gaatcactat gctgcagtaa aaacgccggt aattgatggc
154321 tatcatgctg ataagaatat tattgaagca aaagaagtta aaaaagctga tcttaatttt
154381 tctgtagttt atcaagctaa tggacgtata gtaccagtgg atgcaaaaga caattcgtta
154441 gttggtgttg agcaaccatc atatgtaact gatccagaag atgcaacaaa ggtattacca
154501 aaacaaggtg tacctagaat tttgaattat gtaccagaac aagaagtgat tacggtagga
154561 gatgcaggag aagatacacg agttaaatat tacacattta gcgaatggag tgaattaaaa
154621 gctaagaaaa aggctgaaca aaaacataaa aatttaaaaa caaagaaacc gattgaaaat
154681 aaagatgagc aagaaaagaa ggaaaaagta gaagaaaagc cattaaaatc ggctgaagaa
154741 gagaagaaat ctgagaaaaa agataccggg ttgaaagcta tgttttcttg gcttaagtag
154801 ttatataaca aatttagtat agattttttg ttatataaaa atataaacaa aagcgctatt
154861 tctgtataca tatagaaatg gcgtttttta ttgtgaaaat ataactacga gcaaaaatta
154921 ctttataaaa gataaaataa caaatgaaca ttttctatta ctttattatg taaaaacaac
154981 atataaaatt cgaatattat tcataaaata aacacacttt tttgaataaa aaccagctaa

155041 atcctataaa aacactcgtt ataatagata atatattgaa ttaaataact
acttgaccta
155101 tttttggaat aaaaaatatt tgttataata acactaacat ttaaatagta
tgaccaaaat
155161 gtgtgattat atgatattaa acggaggtaa agagggatgt tatctaagaa
taactaccaa
155221 gaacgtttaa gaaaaatgga tgataaacaa gaacgttttt caatccgtaa
gttttctgtt
155281 ggtgcagctt ctgtattggt tggtacagct atattgagca tgcaaaatgt
tcaaactgta
155341 cgtgccgatg caacaactga tactgaaaaa ggcacaactg atgtaacatc
aaaaaatgat
155401 gaacaaaata agcaaaaagc ttataaccaa gttgtttctg aagatcaaaa
taagtcaagc
155461 aaaactactg gtactacgat ggagggtcaa gatagcaagg tagcttcttt
tagtgcgtct
155521 aaaaatgaag gtacatttgc agaaacctct agtgaagaca aggctgcatc
aactactgat
155581 gcaactgaga gcaagacgtc agatactact aaagcaactg aagataataa
agttgatgca
155641 gtagcagata agagtactga agacaaggct aacactgatg caactcagga
aagtagtgat
155701 agtaagtcaa ctgaaaataa aactactgat gcgcaaaagg tagaatctaa
agttgcaact
155761 actaaagcaa ctactgatac tgctaactcg gttaagtcag catcaactac
tagtactgat
155821 aaaactactt cagtaaacac tacgactttc aatactaatc aatcttcaac
tgcagcatta
155881 ttttcagcct cagcattgag tgaaagtaaa gcattggctg caactccacg
tgcaagttca
155941 gctactacta atactcaggc taagaacaat aattacaaac ttgtaactag
tgcaagtgca
156001 cttcaacaag caattaatag tggagtagct ggtgttaaca tcgatagaag
tattgatgct
156061 tctaatgttg atttagcaat taccaatact tttgcgattg ttggtattaa
tgacgctgct
156121 gtccttaact taggtcaaaa atctcttaat aattctggta acttaactct
tcaagatatt
156181 actatcaatg gtgctgtttc tggtaatggt actgttaaca ttaagggtaa
tgttacttct
156241 aatgtcaatg aaaataactc acttatcaaa ggtgcaactg ttgatgaagc
taatgcggct
156301 ttaaatgatc agacaggtac tggtaagatt ggaacacaag gtacaagttg
ggcaagtggt
156361 agcagtaatc aaaatggttg ggcagttaaa ggctggaatt atgcaaactt
tagtggttct
156421 aaggttaatg tagcagttga tgctaacttg actattaatc gttcagcaat
cggtgatggt
156481 attcatttag caaataatgg tacagttaac gttgctgacg gcggtcagtt
gactattaat
156541 atgaatacta acaacgattt aaatactact gctcgttatc acaatgcagg
tattttcgct
156601 gtgggtaatg gtaacctcac tactggttat aagtcagttg ttacattaaa
tacgagtatc

156661 ggtcaaggta ttgccatgac tggtatgaga ccttatgtta ctgatactga tgtttttggc
156721 ggctatagcg ctcgtgatcg tggcgatgga tcaggtcaaa ttaatttagg tcagtattca
156781 actttgaact tcactggacg tgatggtgtt attttaggta ataactctaa ttttaacgtt
156841 ggtgatagtg caaacgttca ctttgaaaac aaaggccgtg gtgttgccct tgatttagct
156901 gctaatagta atattaatat tgatgatcac gctgtaactt atttccattc tgttggaaag
156961 actactacta acgcacttgg taatactgtt ggcgcttcag gtagctttag tggctataac
157021 tatattggtg taaacgaagg cggtaacatc actgttggtc aatttgcaac atttagagtt
157081 attcttgaag gtcgtggcaa caataattat gatgatgttg tttcgttaga ctcacaaaat
157141 tctaatacca atgctgcttt tacctccaaa actggtgcaa ttgtagatat ccgtgatgac
157201 aacactaact tctatgctga attaatttca ttcccattag gtacatctaa cactagaatt
157261 gatatccatg atccattaat gcttaactta cagcgttact caagtggtgg tccaactact
157321 ggctggatgc caattggtgg agatatgatc aatacaacat cgaaccaata tactgctaac
157381 ttaatttata tgagtggtag taaaggtgta tttagtgtag atggtactga ttacgttgtt
157441 tatcaaaaaa ttaagtctga tggttctaag caaatttggt taaacgttaa tggcgttaat
157501 attccaatga gcggattcca aactaaagat atttggaata atcaggcaaa ccctgatgta
157561 tcaattaaag gtaccgactt aacaagtgga attagagcta accaagttca caattatgat
157621 ggtaccccat taactggtaa cgatgcacca tactacggta tttctactca acgtgctagt
157681 caacaaatct ggttcccaca caagacccaa atggaagtgg ttggcagcca taccaatacc
157741 attaaatatg tttacgaaga cggtactcca gtacttgatg aaaatggtaa tcaaattgtt
157801 aaaacacaaa accttaattt aactcgtaaa cttactcttg atatcaccga tgacaagatt
157861 gaagaaattc aaaaatatgc tttaacacat aatgctgatc aaactttaga gtatattaag
157921 aatgcacaaa gtgtaagtga agactctggt tgggtttata ctgatgctca aggtaatacc
157981 gttactgatc catatgctac agttgtttct ccagttgagg atggctacac tgcttctatt
158041 aaatcttcaa atgttcctgg aatcactgag ggagcagatg gaacttctgt aactgctaaa
158101 ttgcagtaca aagaagatct tgttcaaaac ggtgagttat caaataacta taaacaaaac
158161 ggacttagtg caattttacc tgacaactac gaaactgtag tagtttacgg tatgcctcaa
158221 gaagtaacaa atactcttaa gttctacgac gatacaacta agtcatacat ttctactgtt

158281 actgatcaaa ctgtaactgg taaagaaaat gatgatgtta actttaaaga tggagcaagt
158341 attgtaaagt cattagaaga tcaaggatac aagtttgtta atgtaaccga tggtactcct
158401 gatgatacta atgcaactgt tttatctggt gacacattta gtgatgttga tttcggtaaa
158461 tttggtaaag acggcaagac atttgtagtc cacttaactc ataaagtagt tccagtaact
158521 cctgatactc caaatgttcc atcaaacagt aaagtttcta aagatgatct cactaagaca
158581 gcaactcgta ctattcacta tgttgaaaat gatcaaaacg gtgcggaatt aaaagaatcg
158641 actgttcaaa ctgtaaacta cactggtact gcatatgttg atgtggtaac tggtcaaatg
158701 gtaaatgcta aggctgatgg tgaagatgct caaggcaata ctacttatgt ggttgatact
158761 gacaacaaga aacaaccatt aattgcttgg actacagata acgatggtaa gtttgctcaa
158821 gttactccag atgcaagcat caagaagggc gatgatactt ggacaactgg tgtaaaatct
158881 gttgatgaga agaatgctcc ggatgtttca actatcactg gcaagacaac taatgaagat
158941 gtctatgtac catatacttt aagccaaaaa acatatactg gtaccaagga aactacgagc
159001 gttacacgtg taatcagcta tcttgataat gaaactaaac aaccagtttc agatgccgta
159061 aaacaaacag ctactttatc tcgtacacga attaaggacg aaaaagataa cgtaattggt
159121 tacggtagtg tttctgaaga tggtcatagt tacactttaa ataatgactg gactattgat
159181 agcaatggtt gggtagctca agtttcacca gatgaaactg ctaagggtta caagaagact
159241 cctcactttg aagatggtaa ggacgcttca actgttgctg ctgatgctcc aagtataact
159301 gatccacaag atgttacagt taatgtcttc tacgatcacg acactactcc agtaactcct
159361 gacaagccag gtcatggttt aacccacgat gacttaaaca aagatgttac tagaactatc
159421 aactatgtag atacgactgg tgctgcagtt aacggtgctc cagatggtaa gagtacatac
159481 actcagactg ctcacttcac tcgtactgct atcgttgata aggtaaacga taaattactt
159541 ggttacgaaa ttaacggtga tggttctgtt gatattactc cagatgctgg cgactttgcc
159601 tggaagtcaa ctgacgctaa tttacctgca gtaacttcta agactcctag tgaagttgga
159661 tacgatagcg ttgatacccc ggtagttcaa gctacaactg ttgcttacaa ttctgaacca
159721 atcaatgtaa ctgtaactta cagcaagaat gctcaacaag gttcattcca aattcattac
159781 attgatgaag ataacaataa tgcaattctt catcaggata ctgtttctgg taaaattggt
159841 gattctgtaa cgtactcaac tgctgatcaa attaagcttt gggaaagcaa aggatacgtc

159901 cttgaccacg atggttacac tactcaaact acaattaatg aagataacaa tggcaagact
159961 tacattgttt catttaagca tggacgtaag aatggtacta ctgaaactat tgtaccaact
160021 gaaactattc acttccaata tgcagatggt actaaagcag ctgatgatgt atatggtaat
160081 gcaggtgact ttaagttcac gcgtactcca attattgata ctgtaactgg tcaagttgtt
160141 gatccaggta cttggaacaa ggaaagttac acttttgatg atggtcaaaa gaacgttaag
160201 gtaattaatg gctacgttgc agataaggta acttatggca acaggactgc aactccaaat
160261 gatttgaatg ttgaagatac tataacttac agaaagatca gcaacattat cccagttgat
160321 gaaaatggta accaaattcc aggaactact ccggttgact ataagaatga tccgagtgat
160381 ccaactaagg tgaccccaga cgaagagagt ccaaaagtac caagtggctg gacaatttca
160441 cctgaccaac cagaaggggt aactccaaac actactacta atactgctaa ggtaacgcca
160501 gtagatccta ctgagccaac taaagttgtt tatactaggg atgaagaacc agaagatcta
160561 caatatgcaa ctgtaaaggt tatttatcat gatgatacaa ctggtcaaga tttagagagt
160621 tggagttcag atactaacgg caataagaag gaagttggta cagatactgg atacactcaa
160681 gctgatatta atagagtagt tcaaggatat gaagctaagg gctactacta cgtaactact
160741 gatggcactc ttccaactac tattccagct gatggtgcta ctattgtggt tcacttagct
160801 cacaatcaaa ttccagtagg tcctgatact cctgataagc acggtgttga tccagatcaa
160861 gtcaagaagg tatacacttc aacactttac taccaagata gcgaaggcaa gaccttaagt
160921 cctgaccaac aacaaacttc aacttggact cgtacagtta ctgttgatgc tgtaactaac
160981 caaattgtaa atggtggtaa atacgacact aactggactt tacaagatgc aaatgatcaa
161041 tacagcaact ttactgttcc agtagttgaa ggttatgttg ctagaaagat tacaaataac
161101 ggtgcaactg taactactgt tgttgcaggt caaactaagg tacaacaaaa cttagaagat
161161 actgttgttt acgacaaagt tggtaagctt gtaccagtag gtccagatgg caagactcca
161221 attccagatg caccaactcc aagctatcca aatgatccaa ccgatccaac taaggtaatt
161281 ccaaacgaac cagttccaga cgttccaggt tatactcctg ttgatccaac tcctataaca
161341 cctgaagatc caactaagga tacgccaatt ccttacacta aggatccagt aaaggctggt
161401 ttaacagttc aatatattga tcaagataac aacaatagcg taattaagtc agatgcagta
161461 aacggtaata tcggcggcaa ggtcgactac agtactgcaa gttcaattac tgactttgaa

161521 aataaaggct atgttcttgt aactgatggc tttactggtc aagcagggga tgaatttact
161581 actgaaaaca acggtcaagt ttacaaagtt gtcttcaaac acggaactcg tccagtaact
161641 ccagaaaacc cagcagatcc aaacaagccg gtagatccag accacccaga tacaccaact
161701 ccaagtaatc caaacttgag taagacagac ttgcaaaaga cgattactcg tacagttgaa
161761 tataagtatg cagatggtac acaagcacat gaaccagtta agcaagaatt aacatttact
161821 ggtaaaggta ctattgactt agtaactggt aacttagtaa ctgttgataa agatggtaac
161881 attactagtc aaaacggtaa gattacttgg aaccatgaca gtcaagaatt tgaagcagtt
161941 ccagcaattg atcacgatga ttactacatt tcaagcatta accaaagtaa ttcaacagct
162001 agtgttgatg gtcaaacagg tgctgtaggt actgaaacag taactccaaa tagtcaaaac
162061 ggtaatattg taattacttt gactaggaat ccagatgttc cagtagctgc tcaaggctca
162121 attaactaca tcgacgatac tactggtcaa actattgaaa gtgcaaactt ctcaggtaat
162181 gttggtcaaa agattaatta cactactgct ggtagcatca agaactggga agctaaggga
162241 tacaatttag tatctaataa ctttaaagat ggcgaagaag tatttactga tggtaagaat
162301 gcatttgaag tacacttagt acacgctact actccggtaa cacctgaaaa tccaggtaaa
162361 ccaggtgaac cagtaaatcc aactaaccca gatgacccac acaagtaccc agataactat
162421 gtaccacaag agttagctaa gactgtaacg cgtgatgtaa cttatgttta cgcagatggt
162481 agccaagcag aagctccggt acatcaagaa gttaagttta caggtagtgg ttatcttgac
162541 ttagtaactg gtgaatacgt aactgttgat aataacggca ggatcactgg aaagggtcaa
162601 attaactgga ctccagaaag tgcaaacttt gacgcaacta agtcaattga cacttctaag
162661 taccaaattg ttggtattaa agaaaacaat actactgcaa atgtagatca gactactggt
162721 gttgttgcag gtgaaactgt aactcaaaac ggtaacaaca gttcaattgt aattacttta
162781 gctaataaac cagctccagt agttgaaaag ggttcaatta ctgtaagagt tcatgacttg
162841 actgataatg ttgacttacc acaatacggt aaggaaagtg gtgaacaaga agttggtact
162901 agctttactt atgataagag tgctgtaatt actgaactta ttaacaaggg ctacaagtta
162961 gttaatggtg gtgaagatgt tccatctgaa gtagctaaag gtgccaagac aattaccatt
163021 cttgtagaac acgatactgt tccagtaaca cctgaaaacc caagtaaacc aggtgaacca
163081 atcaacccta acgatccaga cggtcctaag tggccagaag gcactgatga aaatagtgta

163141 aagagaacag gaactcagac tattcattac gagggtgcag gagataagac acctagtgac
163201 gatgtacaaa cattcgactt cactaagaag atgttggttg acaaggtaac tggtaagatc
163261 attgattcag gtgaatggaa tgtaactagc cacacatttg gttacaagga tactccagta
163321 attgatggtt accacgcaga taagcgtaac gctggtggtt ctgtggtaac gccagatgac
163381 ttgaacaaga aggtagtagt aacttacaag ccaaacggca agatcattcc aactgatcca
163441 agtggcaacc caattccgaa tgtgccaact ccaacttacc caacggatcc aacagaccca
163501 actaaggtag tgccagacga accagtacct gatatcccag gcatgacacc aagcacacca
163561 acagtaactc cagaagatcc aggtaaggat accccagtac cgtatacccc agtagttcca
163621 gctaaggatc aagttgcgca agttatctat cgtgatgtac aagatggaac taacaagcaa
163681 ttagcaactt ctggtgactt gactggtaag agcggttcag aaatcaacta ctcaactgct
163741 gaccaaatta aggaattaat tagccaaggc tacgttctta agaatgatgg cttcccggca
163801 ggtgttgtat ttgataatga tgacagtaag aatcaagtat tttatgtaga cttcattcac
163861 ggtcaagttc cagttaaccc agataatcca catgaaggta ttgatccaag tcaatacgaa
163921 aagactgtta aagaaaaagt tcattatgta ggtgcgggcg ataagactcc agctgataat
163981 gttcaaaact ctaagtggac cagaacttta actgttgaca cagtaactgg taaggttgta
164041 gaaaatggtc aatacactac tgattggtca atcgctaagg gtgaaaagac tgtttacgat
164101 caagtaaata ctccagtaat tgatggttac cacgcagata agagagaagt tcctgctact
164161 gctgtaactc aagatgatat tgaagtaaca gtaacttata aaccaaacgg caagatcatc
164221 ccaactgatc caagtggcaa cccaattccg aacgtaccaa ccctaactta cccaacagat
164281 ccaacagacc caaccaaggt agtaccaaat gaaccagtac ctgatatccc aggcatgaca
164341 ccaagtacgc caacagtaac tccagaagat ccaggtaagg atacctcagt accatataac
164401 ccaatagttc cagctaagga tcaagcagct attgttagct acgttgatgc tgataatggt
164461 aacgctgaaa ttactaactc aggcaatttg actggtaaag caggcgacaa gatcgactac
164521 tcaactaagg caactattgc aagtcttgaa aataagggtt acgtacttgt aaatgatggt
164581 ttcccagcag atgctacatt tgacaatgat gacaacacta ctcaaagatt tgttgttgta
164641 ttgaaacacg gtacagtacc agtaactcct gaaaacccag gtaagtcagg tgaaccaatc
164701 aaccctaacg atccagacgg tcctaagtgg ccagaaggca ctgatgaaaa tagtgtaaag

164761 agaacaggaa ctcaaactat tcactatgtt ggtgcaggcg acaagacacc tagcgacgat
164821 gtacaaacat ttgacttcac tagaaatatg gtggttgata aggtaactgg aaaagtcatt
164881 gatggtggtt catggaacgt aactagtcac acatttggtt acaaggatac tccagtaatt
164941 gatggttacc acgcagacaa gcgtaacgct ggtggaacag ttgttactcc agatgatttg
165001 aacaagattg tgactgtaaa ctatagtcaa aacggtaaga tcattccaac tgatccaagt
165061 ggcaccccaa tttcaaacgt accaactcca acttatccaa cggatccaac agacccaact
165121 aaggtagtgc cagatcaacc agttccaaaa gtaccagata tgacaccaag tacaccaaca
165181 gtaactccag aagatccagg taaggacact ccagtgccat ataacccagt taagactctt
165241 gataaagtaa caactgttga aggcaaacaa attgttcact ttgtagatgg tgacaatggt
165301 aacactccac ttagagatcc aaatactcaa actcatgagt ttaagatcac taatggtgta
165361 ccggatgaat caagccacac atttactttg gttgatgttc cagtaattcc aggttatgtt
165421 gccgaagtta agtctgctgg cggaaagacg gtaacaccag atactcctct tgcagaagta
165481 actgttgtct accataaggt tggtaagatc gtgccagttg atccaaatgg caccccaatt
165541 ccaaatgtac caactccaag ctacactaac gatccaactg atccaactaa ggttgttcca
165601 gatgaaccag taccagctat tacaggtaaa actccagata agacttcagt gactccggtt
165661 gatccaacta aggacactcc agttgtttac aaggataatg aagttccagc aactccaaat
165721 tcacaaaaag ctgtagttaa ctttatcgat gttaatactg gtaaactcat caagacttct
165781 ggtattctaa gtggtcgtcc aggtgaagac atcaataagt tgtactcatc tgctgaagta
165841 attaagcaat tagaagaggc tggttacgaa gttgtttaca atgcatttga tggcgatggc
165901 gtaactaagt acttcgacga tgatgacaat acaacacaac aatttactgt tgctttgaaa
165961 ctaaaagaaa aagctaagac gccagatcca gttgttcctg ctccagaaac cccagctaaa
166021 gagccagaag ctccagcaga aaaggtatct agacctgaac aatcagttaa gcaaaatgtt
166081 agtgttccaa ctccacaaaa gcctgtagag aagaagacta ataataagaa agaagttcta
166141 cctcaaactg gtgcagatca taatgaagca gcttcaattt tgggagctgt tgcagcagca
166201 atcggtatga ctagcttaat tggagcaaag agacgcaaga aagatgataa ataattttta
166261 aaattaaata tctttttcaa attagctaat aaaaagggcg ttgagacttg attctcagcg
166321 ctcttttta atacattata tttgtaaata atacgtatat aaggtaggaa aagacataca

166381 atgatagaga gtaattcgct ttaataaggg gcagctagtg tatgaatagt ttaagtgaaa
166441 aagccgcatc cgtggttatc aagtataccg atttagataa taacttagcc gagctttcaa
166501 actctggaag tcttacaggg aatatcggcg aaataattaa ttatagtacg acagatgaaa
166561 taaaagagtt aacaaagcaa ggatatgtat tagttaataa tccttttgat aataaagaaa
166621 aagcgccagt atttagtgga gatcaagaca gctacatgat tacttttaaa catggcagag
166681 aacgtgtgac tgctgatgat ctaaaatacg gctgtaaact tgaagattta caagtaaagg
166741 gaacacaaac tgttcattat gttggtgcag gaagtcgaac tccgcgagat gaagtatcaa
166801 caattacttt taatcaaatc ctggtttacg atcaagtaac tggtaaaaaa atcggtagca
166861 aaggctggga aaaagttgaa cagtcttttc ccgtagttgc cgcccccgagc attttaggtt
166921 acattcctga tcaagtatta gtcggaggta aggcagtcac tgctgataat cctaaccgtg
166981 aatatactat tacttatcag gttaatgagc atatttctaa taaagagcaa aaggctgaag
167041 ttaagtatct tgacattgat agtaataatg aagagattgt agaatctgaa cttttaactg
167101 gaaaacctaa cactaaaatt aactatagta ctatcgatca acttaaaaag ctaggtgaaa
167161 agggatatga agttgtaagt aatggctttg acgctaacgg cgatgtccaa tttttgata
167221 ctagtgatga atatgtccaa acctttatcg ttaccttaaa gcataagcaa gtcttggtaa
167281 atgcagaaaa tccactcgat gggattgatg aaacagaata tcataagaca agcaaacgcg
167341 ttatttctta tgctggtgct gaggataaaa caccagaaga agtagttcag ttagtaaatt
167401 ggaatcgaaa tcttactgtc gatgcagcta caaagcgtgt tatcgcagac ggaaaatata
167461 ctactgattg gaagccagaa caggaatcgt actcagcaat ttctgtacca gtagttagtg
167521 gatatcatac tcgtattaaa gaaatacccg aagaaaaggt aagactagct aatataactg
167581 aaaagattag atatgtaaaa aatggctatg tgattccaat tgacaaaaat ggacaaaaaa
167641 tcgatagttt accaaaatta cattttgttt cagataaaga tgatccaact ttagtatcct
167701 tgccagagaa cagcttagaa gatgaaaagt atgagccaga agagattgat cttactggaa
167761 ttgatccagc aaaagatttt gaagctaaat atcttttgaa gcataagtat gtgactatta
167821 acaaagataa ctctcatttt gatgttaacc ctggttcata tcgtcgtact gctacggcaa
167881 tagttcgcta tgaaggagca ggagataaaa atcctaagga ttctattcaa actgttcagt
167941 ggaatagaaa tatcacttac gatgaagtaa caaaagaaat tttagaggat ggaaaatata

168001 ctactgattg gaagccagat aaagaatact ttgaagctgt agatacgcca
gtaatttctg
168061 gatttactgc tgatatcggt gttgttgcta agcatgatgt aacgcaaagt
gatcttttcg
168121 cgacggttaa gtaccaaaag aatggtgcta ttattccggt tgatgaagat
ggaaaagaaa
168181 ttgctaaagc gaaatcaatc ccatttctta atgatttaac agatccaact
agagttttag
168241 caactgaaga agttcctgaa atcaagggct accgtcgtac tgaagaatca
gttttaatta
168301 aggatccagc agaggatatt aaagttatct atatcttaaa accacactat
gtcttagttg
168361 atagtgaaca cccatatcga gctgttaaac cgtacaacta tagtattcct
gttaaagaaa
168421 ctattcatta tgtaggtgct aatgaagaaa caccagctga tcgagttcag
ggtgcacgct
168481 ggcgcaggtc tttgacagta aatgacaata atggaaaagt gattgaagac
ggaaaatata
168541 ctactgactg gagcgttgat aagaaagaat atagtgcagc tgtaacgcca
gtagttgatg
168601 gctatcatgc ggatcagtat caagttaaag cccgtgagat tgataaggaa
gatattgatg
168661 tagaggtaaa ataccagaga aacggtcaaa ttgtaccagt aaactctaaa
ggcgaaaaaa
168721 tcgaatatgc agattgtcca acttatatca ctgatccaac agatgcgaca
aaggttctga
168781 tggagcaacc tgtaccacga ttattgaact atatggcgca agattcttcg
atcgttgtca
168841 aagatccgag tcgtgatact aaagttactt attatacttt tactgaaatt
aaagaactta
168901 gctcagctca gaacttgaaa actgagattc agtcaattga tggaaagaac
gaatcttcaa
168961 acgtcgtctc tcttccagtt agtggcaaga gaagaaaagc tattgttaca
tttgttgatt
169021 tgaataacaa tgcaattcaa attgcatctt ctggtgtttt aagtggaaat
gttggtgata
169081 aaattactga cttatataac acgagtaagc aagttgaaga gctcaaaaag
aaaggctatg
169141 aagttgttta caatggtttt gatcccaaag gtgcaagcaa gtattttgaa
gaagatcaaa
169201 gaagggttgc taccttcaca gtcgctgtca aaaaagtaaa gcaggtaaaa
cctaagaaag
169261 agatgcaagc cgaaaaaact tctaaaatag aagaaaagcc aaaagcagct
aattctgata
169321 aaaaaaagaa gcaagatcat aaggttttga agcatatatt tccgtggatg
aaatagaaga
169381 tagaaggcta gatttgttta agcaaatcta gtttttttat tgcaaaattt
ttagaccaat
169441 ttttatgaag aagtgtataa ttaattttcg aaaaaacttt ttcggaggaa
atattgtgtc
169501 tgataaaagc gaatcactga ctaaattaga agaggatgaa aagtataacc
gactaacagc
169561 attgaagtta tttgcgatga cttcatcgat ggttatctca gttaatgaac
tagctccttt

169621 tggaaaaact ggaccaaccg cagtctttta tttactatta gctgggttaa tatggtttgt
169681 accaattacg cagatggcag gagaaatggc ttctgttgat ggctgggata agggtggaat
169741 tttcacctgg gtaaagggat cgctgggaga tcgtgctggc tggaccgcaa tgttttatca
169801 gtggattcat attacagttg ggatgaatac gatgatgtac gttattattg gtgcattgtc
169861 gattaccttt aatactcctt ggtttaatac aacaccaagt attagatttt tactgatgat
169921 ggtaattatc tggtcaatta ctattgttga aatggttgga gttaaaaaga ttggtcatat
169981 tgcagaatgg ttatttgcat taggaattgc gttaccagtc attttactaa tcgttacgtt
170041 ctttatatat ttaattcaag gccgtccctt atatatgcat ttaaactggg ataatattat
170101 tccacatcat ttaactggga ctaccttagt agcttttgtg ccatttgtac ttgccttttg
170161 tggtggtgaa gcatctgcac cacatgctaa atacttagaa aatccaaaga cgtattctac
170221 agttatgctt gctctagcga tcactgctat ttcttttgat ttattaggta gtacggcaat
170281 tggaatgtca gttcctaaag atcaaattca aaattcaact ggctttgttt atactttcgg
170341 aaaaatttta gattcaattg gtttacctgg cgacttaatt aaaaaattta tcggtgtgtt
170401 attggcagcg ggtattattg gggaattagg caattggctt gctggtccta gtcagggaat
170461 gtttgaagca gctaaagaag gttatatgcc taaatttttt gcaaaatcta ctaatcgtga
170521 tgttccgatg agattaattg ttttacagac tttgattgta acggcatcgg ctgttttaat
170581 tacttttacc agtggaaaga actcagattt tgcctttaat gtttctttag cagcaactac
170641 tgcgcaatat ttaatggttt atattttgat gttaatttct tatattgttt tgaaaataaa
170701 gcatggaaac ttaaagcgta gctattatat gactaaaaat aatgcgttag gcattacgat
170761 tgcgattatt gccctggtaa taactgtagt tgctttcttt atttcattta ttcctgctca
170821 aggtacgcca acatacttaa gaggggttta tgtttggacg atgattgggc tttgcatgat
170881 tgttacgatt ctgccactgg tgatttatca ttatcatcgc cacttttaag tagaataaat
170941 gaaagtttaa aaaatattaa taaaataaac tttttacttg taaaaagtta ttaataagat
171001 tacatttaag atgtattcaa gtaagaagtg taaagcaaaa gagctatggt aaatttgagg
171061 tatccatagc tcttttgttt tattaaattt aatgacttaa ggaataaaaa agaatgaata
171121 atcctgaaga aatacagcac ttcattgacg ttttaattaa agaagatagc tttgtcaaag
171181 ctgctaaaaa attatatatt tcgcaaccat atttaactca actgatcaaa agaattgaaa

171241 gtcatttagg tactccaatt attaatcgag ataaaaagac atattcacta acgcaagcag
171301 ggctacttta ttaccaatat ctagaaaatg tttcttacga taaacagcaa ttaaataaaa
171361 agattgctaa atatacacac cctaataagg aaattatcaa aattggtatt ctagaaagtt
171421 taggaacgta tttactacca gaaatattgc cagatttttt aaagcaaaat ccgcaagtag
171481 agattcagct tttcgaaaat ttccctaggg aaaatgaaaa gaacttgcta agtggaaata
171541 ttgattgcta cattggacaa actcctgaag caattgattc aagcctagat attgttagta
171601 atggtgggga aaagtattat atcgtaattt caccagcttc tccttattat caagctggaa
171661 agtttatact tgatccacat gaattagatc taaaagagtt actacaagag ccgttcgttt
171721 taagcgttcc gggttctgca attcgccatc aagtgaatgg cgtgtttcaa agatttcact
171781 tagagcccaa agtgatttta gagtctaaaa gtattattac ggctactagt ttggcaattc
171841 atgggatggg gctcacaatt tccactgcta gtattattaa acgaattggt gagacaccca
171901 ttaatttgtt tcctattagc catgacctaa ttaatgtcgt tttctttatt gcatctaggc
171961 gggaaaaaac taaatcaaag gctctgaaaa atctaattag agaatttaag aataaaaacc
172021 tgcaagcaat tatcaggtaa tattctctac tgcaaattag ctataacaaa tttgttattg
172081 gtaagttaca aaaaagatat ttaagcagtg cgtttcaatc acctataatt taggtatgaa
172141 aaaggtttaa gaaagggcat tagaattatg aaatcaggaa agtatgaagt aagagctaag
172201 ggccatggcg caagtttcat gcctatggaa gtaaccctct ctgaagataa aattgaagat
172261 attaaggtag acgctaaggg cgaaactaaa ggtgtggcag acgaagtctt tagacgtttg
172321 ccagaggaaa ttgttaaaaa tcagacttta aatgttgata ccgtcagtgg tgccactatt
172381 tctagtcatg gtgtgattga cggagtagcc gctgcaatta gtgaagctgg tggtgatcct
172441 gatgaatgga agaaacgtgc taagcctgca gaacaaagag aaaaagatga aacgtatact
172501 actgatgttg caattgtagg tgctggtggt gccggtttag ctgcagcagc aagaagcatc
172561 caacataata aaaaagttat tgtgcttgaa aaattcccac aaattggtgg taataccagt
172621 cgtgcaggtg gtccaatgaa tgcggctgaa cctgattggc aaaagcaatt taaagcttta
172681 gctggtgaaa aggaaactct tgaagaattg gcagcaactc cagttgaaaa aattgatcca
172741 gaatatcaaa cggattttaa agagttgcaa aagcagataa aaaaatatgt tgcttctgga
172801 gctaattact tatttgattc aaaattactt catgaaattc aaacttattt aggtggtaaa

172861 cgtacagatt tgaagggtaa tgaaattcat ggaaattatg ccttagttaa agaattggtt
172921 gataatgctt tagattcagt tcactggctg gcagatttag gagtagattt tgatcgtagc
172981 caagtgacaa tgccggttgg tgctttatgg agaagaggac acaagccagt tgagccaatg
173041 ggttatgctt ttattcatgt tttaggtgac tgggtaaaag aacatggtgg cacaatttta
173101 accgatactc gtgctaagca tttaattatt gaagatggca aagtttgcgg tgtaattgca
173161 aaaagacccg atggcagtaa aattactatt catgccaagg cagtaatttt aactgctgga
173221 ggatttggtg caaatacgcc aatggttcaa aaatacaata cttactggaa acatattgat
173281 gataatattg cgactactaa ttctccagct attactggtg acgggattgg ccttggtaag
173341 gaagcaggcg cagatttggt tggaatgggc tttattcaaa tgatgcctgt ttctgatcct
173401 aagactggtg aattatttac tggtcttcaa actcctccag aaaactatat tatggtcaat
173461 caaaagggaa agcgtttcgt aaatgagttt gcagaacgcg atgttttaac taaggcagcg
173521 attgacaatg gcggtttatt ctacttaatt gccgatgaca agatcaaaga tactgcttat
173581 aacacaacgc aagaatcaat tgatgcgcaa gttaaagcag gtaccttatt tagagcagat
173641 agtttagaag agttagctaa acaaatcggt atggatgcag atactttagt cgatacaatt
173701 aagaaatata attcatatgt cgatgcagga aaagatcctg attttgaaaa gtccgccttt
173761 aacttaaaat gcgaggtagc accattttat gctacgccaa gaaagccagc cattcaccat
173821 acaatgggtg gtttaaagat tgatactggc gcgcatgttt tagataaaac tggcaagcaa
173881 atttctggtt tgtatgcggc tggtgaagta gctggtggta ttcatgccgg caaccgccta
173941 ggtggaaatt ctcttgctga tatttttact tttggtagaa ttgccgcaaa cagtgctatt
174001 gatgaattag aaaaatagta tagatagatt aaaacgttgt aaacactgga gtttgcaacg
174061 tttttttgct aaaatactaa aaatacttga ttttttttgga ggacttgaat tgagagaagc
174121 gcaggaaaaa aagctaacta caaaacagta cttaattgta gcatcaatga tctttgcact
174181 gttctttggc gcaggaaact taattttccc acttcattta ggtcagcttg ctggtaaaaa
174241 ttggggacca gcagcaattg gcttctcaat tacaggggta gtcttacctt tactttcact
174301 tcttgcagtt gcaattactc gcagtaatgg tgtttatcaa attggattac cagtaggaaa
174361 gatctttgct ctttctttca tgaccttaat gcagctagca atcggtccat tatttgcagc
174421 accaagaaat gccactgtat cttatactgt ggggattgca ccgcttttgc ctaagcaatt

174481 tcatggaatt ggtttgattg tctttacgat tatctttttt gccatagttt atgtgattgc
174541 ctataacgag agtgatattc tttcttcttt aggtaaggtt ttgaatccga tctttttaat
174601 tttgcttttt attgtctttg taattgcgtt tgctcgcccg cttggtaatc ctgatatggc
174661 tacaactact aaagaatata tgaacggtgc gatagttaaa ggctttttag agggctataa
174721 tactatggat gctttagctg gtttagcctt tggggttact gtagtaacag cgattaaaga
174781 gctagttaat aatgatgaaa agaaaactgc taaaatgact gctaagtctg ggttaattgc
174841 agttattgca attggtgtaa tctatacttt attaatcgtt attggtgcga tgtcattggg
174901 gcattttaaa atagcgagtg atggcggtat tttattttca gagatcgtta aatattatgc
174961 tggtattttt gggcaggcgt tgttagctgt tttaatcttt ttagcctgtt taactaccgc
175021 agttggggtc ttggcggctt ttgccttaga tttcagtgcc cattattcaa agattagcta
175081 taaggggtgg ctaacaatcg gatgtctagg ttcgttagct acagcaaatt tgggcttgga
175141 aaaaatcatt cattggtcgc ttcctgtttt aatgttttta tatcccttgg ctattgtatt
175201 aattattcta tctgtatgtt caccgctatt taaagacgac atggtagtat acaaaattac
175261 gatgctttta actctagttc cagcgatttt tgatttaatt actaacttgc ctgcaccaat
175321 ttctggaaca cagttttata aagtagtaag tcagattcgc ttgcaatatt tacctttagc
175381 caatattggc ttatcttggg ttgtaccaac aattttagga ttagttatca gtgtcttgat
175441 tcatttttgg cgccgtaaag ctaataaaat atagtaattc agctatggtt catttgaatc
175501 atagcttttt tgttgggata atgaagctag gtgataaaaa tgagtttaac caataaagtg
175561 cccgaaaaag atgtgcatga tttatttact cgggtagctc tgcattatga tcagatgaat
175621 aatttaatta gcttaggtac gcaaaaaggt tggcgcaaga aatttctaaa agaattaaaa
175681 gtagcaccgg gtgaatttgc cctagattta tgttgtggta ctggcgatat cacgattgct
175741 ttggcaaaac aagttggtcc gtcaggaaat gtaattggac ttgattttaa ccaagaaatg
175801 cttgatttag ctgagcagaa aatacgccag caagatttac aaaaagaaat tcaattaaag
175861 caaggggatg caatggaatt gccataccca gatcagagct ttgatatagt aacgattggt
175921 tttggcttac ggaatgttcc tgatgcaaat caagtcctga aagaaatcta tcgagtatta
175981 aagccaactg gtaaagtggg agtgttagaa acatcgcagc caaccaaccc gcttgttaag
176041 ttaggatgga agagctattt taagttattt cctagctttg caaaattatt aggtgctaat

176101 gtatctgatt atcaatattt gtctcatact accgccaaat ttgtttctgc ggagcattta
176161 aaagcaatgc ttataaaaaa tggatttaaa aatgtgcatg tagataaatt aaatttaggt
176221 gcgggtgcaa ttcatatcgg aattaagaag taaatgagct aaaatataaa ataaaaacta
176281 aaaaaggaga attttatgaa aactggtact aaaattatta ctttagacaa cggttatcac
176341 ttgtggacta atactcaagg tgaaggcgat attcacttat tagcgcttca tggtggacct
176401 ggtggcaacc atgagtattg ggaagatgct gcagaccaat taaagaaaca aggattgaat
176461 gtacaagtta ctatgtatga tcaattaggt tcattatatt cagatcaacc agattactct
176521 gatccagaaa ttgcccaaaa atatttaact tatgaatatt tcttagatga agttgatgaa
176581 gttcgtgaaa aacttggctt agataatttt tacttaattg gtcaaagttg gggcggtctt
176641 ttagttcaag aatatgctgt taaatatggc aagcacttaa agggtgcaat tatttcatca
176701 atggttgatg agattgatga atatgttgat tcagttaaca gaagaagaca agaagttctt
176761 ccacaaacag aaattgactt catgcatgaa tgcgaaaaga ataatgatta tgataatcaa
176821 cgctaccaag aggatgtgca aattttaaat attaattttg tggatcgaaa gcaaccatca
176881 aagctttatc acttaaaaga cttaggtggc agcgcagttt ataatgtctt ccaaggtgat
176941 aatgaatttg ttattactgg taagcttaaa gactggcatt ttagagatca acttaagaat
177001 attaaggtac caacattaat tacttttggt gaaaatgaga caatgccaat ttctactgct
177061 aaaattatgc aaaaagaaat accaaattca cgtcttgtta ctacgcctaa tggtggacac
177121 catcatatgg ttgataaccc tgacgtttat tataagcact tagctgactt cattcgtgaa
177181 gttgaaaatg gcaattttaa gggtgaataa cgataaaaag tccatgcgat agcttctata
177241 ttgaagctat cgcatggact ttttaagcat ataaatcgct ttcaaggtat gtatcctata
177301 taattaatag taaatatagc tttattaatt taaggagaaa agccatgaag agaaaaataa
177361 gtaaactgct agcagcaact gctttaatag ggacattagg aatctcagta gtggcttgca
177421 acaataatga ttcaactagt aaaactaagc aatcaagtgt aaagaagacc gttaaagata
177481 aggcaagtaa tacaaaaaat acagcaagaa gaagtaagat taaacttagt caaactgaag
177541 caataaataa atttgataaa aagtattctg ataaaaaatt aaaagaaata gatctaaaat
177601 tagatggtaa taagtacttt tatgaaatta caggatttga taaagataaa gaatatgaaa
177661 tgaccattaa tgctataagt ggaaaagaag tcaaatctag ttctgaaaaa ttagacttag

177721 atgaacgttt gcaaaaagga cttgatttag ataaggtgat ttctcgtgat caagcaagtg
177781 aaatagcaga aaaagaagtt aaaaatagca ctgctaaaga atggactcta aaaatggatc
177841 aggataaagt catttgggat gtaactgttg aatcaggttc aagtaaacat gaagtagaaa
177901 ttgatgctat ttctaagaaa gttattaaat cagaaaaaga tgattaaaca aaaaattcag
177961 agtttttaag ctctgaattt tttattagta tttttgtaga aattaattta ataagtaatt
178021 agcttatcgg cgttcattgt tttatcgtct aacttgtaag tctttaattc aaaatcattt
178081 tcaaagttgc agtaataagt cttagtagca gcagaatagc aagcagtata gacagtgtat
178141 tcattgctac cttgtttgtt aactacgctg cctttaatca tcgcaacaga ctttaagatg
178201 ttaaagaact tggcaacgtt agctttttca cctttaacag ttggataatt aacatttaag
178261 taagctgctt taacaaagcg atctgctgga atgctatcac ctggtaaacc taagctgcca
178321 gtgccaacgc cccatggagc aactttttga ccgttccagc tttgagctgt agcgtcatgt
178381 ggatctaatc cagtatagtt gccgagatta gtaaggtgcc aattaaagtc tgggctgtta
178441 gttagaatgc caagcttatc atcaaagact ttcattccgt attgctttga aacctcgaca
178501 ataatagctt catctttgtc actaataatc cagtgaagag gagcaactgc aaacgatgaa
178561 ttaatggctt cattaactaa gttaacgttc tttaaagctt cttttacgtc gctgacttta
178621 gtaaagtttt gggtgaccca gagcataatt tcgtaagaag ctaaattaat ttttccatca
178681 attggaccgt cactgaattt ggcaaaatgc gggaagttta gaccagcaat tcccaaacca
178741 tcttcattga aacagtcaaa gtaagaagga tagccatcga ctacaattcc catgccgata
178801 acagcctttt tagtagttgt attatctaaa aatttatatg gaagaggata gttgcgaggt
178861 gtaataatta caccttcacc ataatcttgt ccaacgtcta agttacgtcc aaagtataga
178921 tttccttgat catcagtaaa tcttaaacca gtacacattt taaaatcctc cttatttatt
178981 cagtagtagt tttagtttct tgctgaatga tgtatttctt ttgacttctc ttaatttgaa
179041 taacagcaaa tactaagatg ataaaaccaa ttgctacaaa ggcaatcatg aagtaccaag
179101 cgactgtaaa gctggctttt cctttaataa ttccgaataa tggagcacca gtagcatatc
179161 caatcgcata agccaaacca acgtatccta acattacacc ctgatctctt gaaccaaaga
179221 ggtccttagc cataaatgca ggaccagaca tgtagctaaa tacggctaaa ccacagaaga
179281 tagcgtaagc aatacctgca attttactta cgtgagcgcc ataaggttga aaactgatca

179341 aaaccatcat taagatggat aaaacataca tgcagccagc ataggtcatt gatttagcag
179401 taccaaattt atcaaataag aatccaccgg aaacatttcc aattaaacaa ccaacaccgt
179461 acattgatcc aatgagacca acatcggtta gtgaaagttt agtatcaaga aaggctgcat
179521 aatcttcatt taaggaagct aagcctaagc caataattag aaaaccaaga ctgaaaatcc
179581 agaaccattt catttgtaaa acttgtttac tagtccagcc tttaaattcc ttagctttag
179641 ccaattctgc ttctttcttg ctttcagcta actcagcatc agaaataacg atttcatctt
179701 tcttaggagt tctaataaag caggcaataa ctataccaat tactaataga gcaactgcga
179761 agatgaaaaa tggtgccatt gaagtcaagt gaccggtctt agtattacca gtcataaagt
179821 gctttaaaat agcttgcgtt gcaggttgta agaaaatatt accaattgag ccaccgcaga
179881 aggcaattcc tagagcagct ccacgtcctt ttgcagggaa ccagtggtta ataacccaag
179941 gcacaccttg accggaataa aaagttgagc caatcataca aatgatagcg gcaatataga
180001 atcctggtag ttttgtacta attccaaaga ttacataggc aatagcggaa agaccaatac
180061 cgattaaata cataagtctg aaattaactt tctctaatgc tttaccaata aatggcgaag
180121 caactgaagc aaaaactgcc ccaaacgtaa agattaatgt atatgatgct aaggtaaagt
180181 gaaaagtatt aactaaaggg tggataaaca aaggttgaat attttgagca atcccgtaag
180241 gaatggcttg ggttaacata caaaggaaaa ccataaaata cttatagcct ttgctgacga
180301 ccttatcttt agtagcgaca tcagtagaca tgtaattacc ttctttctaa actaattact
180361 tatctaaaat aatatttggt ttgctgacga caatattttt ttgtatttgc ttaattttaa
180421 tagctgcaaa aattaataat ataaaaccaa ttgctacaaa gaaagtagtg cagcaccaag
180481 cagttgtaaa actggtagcg ccttttatga cgccaaataa tggggcgcca attgcaaaac
180541 caacagcata tgccaggcta atgtaaccta agttaacacc ttgggctttt gcaccaaata
180601 agctttttga cataaatgcg ggaccagaca tatagctaaa gacagataga ccacttgtaa
180661 aggcccaacc cataccagcg tagaaattaa cgcgatcgcc ataaggatgg aggctaataa
180721 agatcatcat taggatagct ataattaaca ttattcctgc atatgccatt gattttgctg
180781 tgccgaactt atcaaataaa taacctccag aaatatttcc gatgatacca gcgagtccaa
180841 ataccgagcc aatcattccg acttcagtta aggataattt agtatcaagg aaggccgcat
180901 agtcttcgtt taacgaggcc aagcctaagc cgataattaa aaatccaata ctaaaaatcc

180961 agaaccattt catatgtaga acttgtttac cgctccagcc ttgaaattca tgtgcttgct
181021 tttgagcagc ttcgtgccgg ttttgctcaa cttcttgagc agaagctaag atttcatctt
181081 tctttggtac tctaataaaa gccgtaataa tcaatccaac tattagcaaa gcaatagcaa
181141 agataaagaa aggtgccatg gaagttaagt gaccagtttt agtattaccg gtcatgaaat
181201 gctttaaaat ttcctgggta ataggttgga ggaaaatatc accaatcgag ccaccgcaga
181261 atgctaaacc taaagcaacg ccgcgtcctt taaacggaaa ccagtggttg ataatccatg
181321 gaacaccttg accagaataa aaggttgaac caaccataca aataattcca gctaaataaa
181381 agccaggtaa ctttgtgcta atttcaaaaa ttacatatgc tccagctgaa agacaaatac
181441 caatcagata taaaatttta aagtttactt tttgtaaagc cttaccaact aatggtgaaa
181501 ctaaagaccc tacgacagcc ccaaaagtaa aaattaatgt gtaagaagct aatgtaaaat
181561 gaaaagtatt aactagaggg tgaacaaata aaggttgaat taattgagcg attccatatg
181621 gaacagcttg agttaacatg cagagaaata ccataaagta tttgtagcct ttactgacca
181681 ccttttgtct atcgtttgac atcgtatctc tcttctttct gacaaaaact attagcttga
181741 taagttatat attattaagt gcgtgagctt tgactatagc cacctatatt gtggctcact
181801 gaatcgagta aatcaaaaat aacggcttat aatgttattt aataggcata ataaaaagcc
181861 tatctttttt aagatagact taagaaaatt atttttttac atatcctgcg atgtcatcat
181921 cgtcaaaact atagcttaat ttcttgccat atgccttttc ataggctttt ttcttttgta
181981 gataatcaat atcacgcatt cttttggcgt tttgatcaac tgttagacgg tcaattggat
182041 agattgttgg caaaacgtga agaatgtagc gagttttatg gaactcgcta ctatttttct
182101 ccatttcagg aagatcctga atctctacaa aacaggaaat aattggaacg ttggccttag
182161 ctgcgtagta gtaggcacct ttttgaagag gacgcggctt tcgatagtta taccacattt
182221 cttgttctgg atagatcaag acccagccct ttttatcaaa aacttgctgc aaatgtttag
182281 gaaattcacg ccccaaatat tggtagcttt gaacaattgg aattgcatta atgttgttca
182341 ttaagaaacc aaagaaaccg ggtaatttta aattagaatc ttcaatcaca atataaagct
182401 ttttgtgaca tttgttggct aattttttga ttggaagact atcagtttgt ttgaagtgat
182461 tactagtaac aattgcgcca tatttaggcc cttgagcacg atgtttattc ttgatttgcg
182521 tgtgggccat caaaattaaa gtatcaatat taataatgcc ttgggcaata atattcttta

182581 gtttatgact aaactttttt tgttgttccc aataatcatt aaccagcttg attctttcgc
182641 ttttagttag gataggatcg cctaattcga cttttgcatg catttttcct tttttaacag
182701 ctgctttgat gttttcgata acttttgcgc gattttctcc aataatcata atttaaccct
182761 ttcgcctttt cttttaactt gagcccaatt atttggatca agtgctgtgc tagcttcttt
182821 ttcaagcata tgatctaatt ttgctcggtc ttcggctcgc tcttggtcag tatagttgtc
182881 taattctccc tttaactcct cataaaaagg tgtgctagag gcatatttcc agaaataatc
182941 attatattgg acatctttaa agtgccaagg tttgaaaaac aaattatagt gaattagccc
183001 aggtttagaa atgggttcgg tattttcatt aggcattgca tcccagcgtg gatcaagatg
183061 caaaattctt ccatcgccaa tctcatttag gtagtcttga tcaggagcaa tacaatcaaa
183121 gtgatatcgt ttcaagtaaat gcataaagtg atcgataaag tgttcatccc taaaagcctt
183181 agaatttaag actaacatcc ccgaattgat atatttcttt ggatcgagag atagaacctc
183241 tttaatatat ttcaccattt tatctacata ttgaatcgat gagtctgtac aagcggcgaa
183301 tagcttatta tctaagtcgt tattatacag cttagcaatg tcatcaacga cgacggtgtc
183361 actatcgatg taaattgctt tatcatattg tggaaatagg tccggaataa agagacgata
183421 aaaaatagac attgtgaaaa agtctgctcg aaggtagttt tctttacgat tctggatcgg
183481 ttttactaat ttttcatcga tatggaaaaa tttaacatgt acatagtcat tagcaaaagc
183541 tgcaagttgc ttttggtgtt tctcagacag gtcttggttt aaaaaagtaa ctgtgtaatc
183601 attttgcgga ctagcattct taactaagga gttaagcgaa acagccgcat acttggtaaa
183661 atcatcacta atactataaa aaacagggat agtcattttt agtcctcttt atatctttac
183721 agatgttgtt ttcttaactt ttgcccaagt tacatcttca tctttgattt cgtcaacttt
183781 tttaatcatc caatcaaggt cagtacgtgc ttttttacgg tcctcatcgg taaagctagc
183841 taattgtgct tttagttctt cataatatgg agacttctta gcaacatccc agaagtattt
183901 gccatattga acatctgcaa aatgccaggg cttgaagaaa agattataat gaacaatttt
183961 aggattttta atttcatcca tgtgttcatt aggcattgca tcccattcaa gtggtaaatg
184021 ataaatttta tcttcgcaaa tctcattcat ataagcttga tcaggatcga tattatcgaa
184081 atgatacttt tcaattaatg aatagaattt atcgacaaat ttcttatctc taaaggcttt
184141 catattgaag agaataacgc cgttattaat atatttttct ggtggaaaaa taccttgaca

184201 ttccttgata taaacttgaa gtggtttaat aaatctgatt gacatatctg gtacgctggc
184261 aaacatgtta tcaccaattt cggtgttgta aagctcagcg atgtcagtac aaataatagt
184321 atcagcatct aaataaacag ccttgtcata ttgaggaaaa aggttaggaa taaatagacg
184381 gtagaaaata gacatcgtga aaaattgtgc ccgtaagtaa ttctcttcac tgttgtgaat
184441 cggagctacc atttcgtcat caatatgaaa aatattaaca tggacatttt tggttgaaag
184501 atcttccaaa tctttcttat gcatatcact aatgttttga actaagagag taatggtata
184561 gtccttgttt ggatcaacat ggtcaattag tgactgaatt gaaacagctg cgtaaggtgt
184621 gtagttgtcg ctaattgtat aaaaaacagg tatagtcatc aaaaatagac tccttatata
184681 ttaaaagttc ctaggcccgc tttaattgtg acctaagctc tgtatactgc ttaagtaaat
184741 catcatattc atgtaatttc aagacgctgt gcacacgctc tatatcccat ggtttaacag
184801 ttaaagtatg gaagtatggc ttaaagcgaa agctagttgt aaaatgttga atttagtat
184861 cgggtcgtaa acgatattgt tcattgtagc agcgcggagc aattttcttc tcagtagcaa
184921 gtttatttag agcagactga tctgggagaa gcattttttt atcactcatt ttttctctaa
184981 ctttggcaaa taattgtgtt tttttaattt cagacatatt aagaagcaag acgcctgagt
185041 ttaagtaatc aaatggctta tgagtatgga tattatgaaa gaagaaacga ccccagaagt
185101 ctaaaacacc aactagctcg atattagtta gatcttgatt ataaaagtcg ctaatatctt
185161 tacgcacaat aatatcatca tctaaatata agacacgatc aggaatttgt ggtagttgat
185221 ccgcaaaaag gcgtagcatt gcatatggag taaagcgtgt ttccatattt gctcgtggag
185281 gttctttttt aaataattca gtacaatcga ttaattcagc agtattgtta ggatcttttt
185341 ccttcagcag ttttttaatt agtctaaatg cttgctgatt aaaagctgtg tatccctctg
185401 cgtgcatcgt taaaatgtaa aagtgtaggg gagttgatgt gttttttagt aaagatagtg
185461 ttgtaattaa aataccatct tcggcatgag aatcgccgca aaacataatg ttcattttta
185521 tcctccgatt tttcgctgct ggtagcgata atactcatag ttgcttaatt tttttgaacg
185581 gtcctttaat tgagtaaata cttgatcatg tagctttttt tgctgtaatt ttttaggcag
185641 agaagtatct gggaaaaaag gcccatcaat atagacagta atcctaggtc gcttgctgaa
185701 ttttcttttc tggtaggttg tcgtcatgac aaagcttggc ttattcgttt gaactggaaa
185761 attaaaacta gttgctggaa atgggcgaat atcagtatag taaggccaga catgtgcttc

185821 tgggtaaata ataatatggg catcttcctt agttagagta ttaactgcct taagcaaatg
185881 aattgcttgt ttaatatttt tgccaactgg tagtcctcca taaggtaaga gaatttttcc
185941 aattattgga attccccagt ttgcttgatt agcaattgca taataatctt tccagccaaa
186001 gagggttaaa ggcataaagg catcattaac catttgagtg tgatttccat agacaaaata
186061 gctttgatgt ttatatttaa ataacttatc tctccctatt actttgacat gcattacgcc
186121 ataagtaaaa agataagcaa aaccagtagc taaaaaacgc attaaatagt taagaggagt
186181 acgtttaatg atttgataat tgctaggtag ggtgaaatct tgttgcttac ttttgacaat
186241 gtcatcagtt aaagaattat agtaaataac attacgttga gatgttttag tattcatgat
186301 attaattata aaagaagttt aaaaacaaaa aaagacatcg ccacagcgat gccttataaa
186361 tatgggtggt caggggatcg aaccctggac ccacggatta agagtccgtt gctctgccag
186421 ctgagctaac cacccaattg gataaccaac agatactatt atgccaaaaa aaaataaaaa
186481 tgcaagaact tttttgcctt ttttaaatac aaggagccaa aaacaaaaaa gacatcgcca
186541 cagcgatgcc ttataaatat gggtggtcag gggatcgaac cctggaccca cggattaaga
186601 gtccgttgct ctgccagctg agctaaccac ccaattggat aaccaacaaa tagtattatg
186661 ccaggaaaag aactaaagtg caagcttttt ttgcataagt ttaaaaaaag tgtggaaaac
186721 agtataatat agttattaat attgaggagg catcatttat gccaaaaaag attttagtcg
186781 ttgacgatga aaaaccaatt tctgacatta ttaaatttaa cttgactaag gaaggctttg
186841 atgtcgacac tgcttatgac ggcgaagaag cggtaaaaaa agttgatgaa tatgatccag
186901 acttgatgat tctagattta atgttgccta aaaaagatgg cttagaagtt gcacgggaag
186961 tacgtcaaac tcacgatatg ccaattatta tggtaactgc taaagatact gaaattgata
187021 aggtattagg gcttgagatg ggggcagatg actatgttac taagcctttc tccaatcgag
187081 agctagttgc tagagtaaag gcaaacctac gtagacgtga tttaacccaa aaagctactg
187141 aagatgatga agataagaat attactatta gtaatttagt aattatgcct gaggcctata
187201 tggtcgaaaa aaatggcgaa aaaattgaat taacgcatcg tgaatttgaa ttacttcatt
187261 atttagctca gcatatgggt caagtgatga ctagagaaca tttgctgcaa accgtttggg
187321 gctatgatta cttcggggat gttagaactg ttgacgtaac tgtgagacgt ttacgtgaaa
187381 aaatcgaaga caatccgagt tcgccaacaa ttttagttac acggcgtgga gtaggatatt

187441 acgttaaaaa cccatcagat gaataaggat caaagtcact agtaatagtg gcttttttg
187501 caattaggac aaatgaagaa aataaaaagt gtattaaatt ctatcaattt taaaatcgca
187561 gtaattttta tgctgctctt attagcaacg attgaagtgg ttggtgcgtc ttttacaagg
187621 caactcgaac aaaattcaat tcaaaatttt gaatcatcaa ttcaagttcc caatattatt
187681 actaaccaaa tttctagtca attaagtagg gctaattcta aaaaggctaa tcaacagtta
187741 agccagatta tttcaaacta taatttaggc gatattagcc agttaatggt agttgacaat
187801 aagggagtaa ttagagctgt ttcaaatgtg aatgatcaaa accggattgg acagcgaact
187861 agtaatgtag atatcaagag cgtactctca aatggcaagc aagtttcaaa agttattaat
187921 gacaacggaa attatatggt gcaaatttca cctttgactt ctgctaatgg aactaatact
187981 cctgtaggag caatttatgt ccgcgctagt ttacaaggtg tctttaataa cttaagacag
188041 gtatcaattt actttctgat tgcttcactg attgcagctg ttttgggagc aattgtagcc
188101 ttagtcattt cccgggcaat aactagacct attgaggaga tgcgtaaaca agcattaaga
188161 gttgcaaatg gagattattc tggacatgtt cgagtttatg cgcaagatga attaggtcaa
188221 ctagctgagg ccttcaatac tttatcagta agaattgaaa gaactcaaga aatttcagat
188281 agtgagcgaa gacggctaga taatgtctta acacacatga cagatggcgt tattgcaaca
188341 gacagacatg gaaatatcac gattattaat gaaacggcac ttgatttttt aggaaaaact
188401 gaaaaagacg ttattggaaa gccaattact aatttacttg gactaaaaga tgtcactatc
188461 caagatttat taagtactca gcaagaatta gttgttcgag ttaatgataa tacgcgcgat
188521 gaaatgattt tgcatgctaa cttttctctg atccagcgtg taacaggatt tgtttctggc
188581 ttagtttgcg tgcttcatga tattacccaa caacaaaaaa atgaacgtga gcagcaacaa
188641 ttcgtttcaa atgtttcaca tgagcttaga acaccgttaa caagcttaag ggcatatgtt
188701 gaagcattga atgatggagc atggaaagat ccaaatattg caccgcaatt tctccatgtt
188761 attcaagatg aaactgagcg aatgattcga atgatcaatg atttgcttag cttatcaaga
188821 atggatcgag gagtagcaag gatggattta gaatgggtta acttaaatga ctttgttaac
188881 cacgttttaa atcggtttga tatgatgctc aagtctgatt ctgataagat gcacaaaaag
188941 aaatatacga ttaagcgtga atttcctcat caagccttat gggttgagat tgatacggac
189001 aagatgatgc aagtaattga caacattatg aacaatgcta tcaagtattc acctgacggt

189061 ggcgttatca cagttcgctt attgcaggct caaaagcacg ttatcttaag
tatttctgat
189121 caaggattag gaattccaag aaaagattta aacaaaatct ttgatagatt
ttatcgtgtt
189181 gataaggcac gttctcgtaa acaaggtgga actggtctag gactggctat
ttctaaagaa
189241 atagtagaag cacaccatgg tcgaatttgg gcagatagtg ctgaaggagc
tggctcaact
189301 ttctacattt ctttgccata tgaagcaatt agcgaggagg gagaaaactg
ggatgaggtt
189361 taaagataag ttttctagaa ttacattacg aattagttta attgtaatgg
ttgtcttatc
189421 aattatctta tcagctatta tttggggttc tgatgcacga ttttcaagaa
ttgaagagac
189481 gtctaatcaa actcaaacta aagatttggg tcaacgttct ttaagagata
tttatttgcc
189541 tactcaaact ttttatttta aagataagca aatgtatcag gtttacgata
ctaaaaataa
189601 tcttccgcta gagttttcaa aattaactca atcagttaag ccattattgc
caattagagt
189661 ttggtctagt caaagtaaat atgaaaagtt attaaaaaat cctaactatg
ttcagttgac
189721 atatccagat cagataacta tctccttatt cttaactaat gtaagaaagg
ctgatagtcg
189781 ggaatttaat cgctttttttg tacccgctcg atccagtaag tatatctatt
taggtaatga
189841 cgaaaactat actatttatc gagttcgtct aagtgatgta tcatttgaca
atttggtaga
189901 acatattcaa agtgctaaaa cacagatgcc agttacactt caaaaagttc
atgatgatta
189961 cttgcccttc tatgacaaga atttaagttt accaacgtat agctatttaa
ctaaggaaga
190021 gtcagattca tactttgtgt atcgcttgct aggctctaat aatcctaccc
agcatagttc
190081 tggagaaagc attacatatt ctaatggagt atatgagcgg ttgattgccg
caaagcatac
190141 tcataattat gaatatattg attatcaaca agatcaggtt cccaagacta
ttagtcgaaa
190201 attaaatgat agtttatatt ttgttcgtaa aattggatta tccgagccag
atttaaggtt
190261 ctttgatgct gataataata cagttattta tcaaaattat gttgaagaat
atccgatctt
190321 tttgccagga aaatataaga tgcgggcgca agttaagttt gcttctaatg
gaatgacaat
190381 taactttaat agcttagact tgcagattcc aattccaact agtggagaaa
agaagacgct
190441 tattccgact aatgaagcga tggatgaatt atatcaaaag ggataccacc
aaaaagatat
190501 tgagcgcatt gttattggtt atacggctaa atctgataat agtaaaaata
aaaaattagt
190561 agatctagaa ccaacttatt atgtaaaaat taataagcag tggaagactc
tagatgaatg
190621 gctgaatatt aataatcaag ttgtgaattc tggaaaggag gggctagtag
atggacttta

190681 agagaattga atggattttt ttagtcgtat ttattggaat caatatcttt ttgggtattg
190741 aactttggca aacgccgacg cttttgtctg ctggctctac tccggctcaa actgatatta
190801 aaagtgaaac aagcgcagat cagattacta tccctaaagt taatgataag caagaggatg
190861 ggtattattt agctgccaaa gtggacaatt cttggacaaa aaaagcagca gcacaggtaa
190921 atcaacaagt agaaactaat gcaagtgaaa atagtatttc agttaatttg agcaaaccaa
190981 ttgctctatc gaaagattcc aaaaaagccc tgaaagaaat tattcacttt aaaaacaata
191041 gtcaaaacgt gtatgagggt aaagattatg tctatctttc agaactatct gaaggggatg
191101 attatgtctt taatcagaaa acaaagtatg gagaagtttt tgctgcaact gcacgattgc
191161 acattattgt taaaaataat caaattgtat cttattcgca acgctatgtt agcaatttga
191221 acccggtacg cgaaagacaa aatacaatta gttcaaaagc tgctgtaaat tccttatata
191281 cttatagtga attaccaaat aattccaaga ttatctggtt aaagcaagtt tataccaagt
191341 tgatcactgt tcgagaaagc gaaatttata ttcctacttg ggtggcagcg attgaaaata
191401 ataattcgca tacgatgacc cttaaacgcg taaatgcatt tacagggaca attatccaaa
191461 ataatattgc tgctgatgat tcaaaggagt agagaaattt gaaagtatcg gttttatcaa
191521 gtggttcagc aggtaattct acgttgattg agactccgca gcataaaatc ttaatggatg
191581 caggtttatc tggaaaaaag actaaagatt tactggcaca agtaggcgtt gatattaaag
191641 atattgatat ggtatttatc agccacgatc atactgacca ttccggtgga ttaggcgtat
191701 taatgagaag atatcctaaa atttcggctt atgcgaattc aggtacttgg aattatttaa
191761 ttgaaagtaa taagatagga aaattacctg ttgaacaaat taatacgttt gaatctggta
191821 taactaagac ttttggtgat ttagacgtga cgaattttgc gacgagccat gatgcagcgc
191881 agccacaata ttatgtcttt acaagtggtg gtaaaagatt tgcgtgttta acagatactg
191941 gttatgtttc aagtactgtt aaaggtgaaa taaaagatgc agatggttat ttgatggaat
192001 ttaactatga cgatatgatg cttagaaatg gtccgtattc ttggtcatta aagcaacgaa
192061 ttatgtctga tgttggacac ctttcaaatg atcaagcagc agacacctta cttgatgtgg
192121 tttctccaaa aactaaacat atatttttag cgcatcgcag ccaacataat aatacccagt
192181 atttggcgca tgagacggct gaagacttat tagtagctgg ggatgcaaat ttaccagctg
192241 atgttaagat tattgatact aatccaatgc aagctggctc tttaacgaaa atataaattt

192301 ttagcaaata atttgacttg aattcataaa aaattcaaaa tttgtgatta ggctagaaat
192361 gaagagaaaa ggagaaattt ttatggcaga aaataatact aagcacccta ctaaacataa
192421 cgctttaatc aaaactggta ttgttggagt ggttgccgga cttcttgggg gcggagttgc
192481 ttatgcaggt ttatctcaag ttaatggaca aaatgcacca caaacaagta tcgttccaac
192541 gacaaaagtt gaaaagtcta gcagtaaaaa tagtagtcag atgactaatg cctttaatac
192601 tgtaaaaaaa tcagttgttt ctgtggttaa cttaaagaga caaagttcat cttcaagtag
192661 tgatccgttt ggtatttttg gctctgatag ttctagttct tcaaagaaaa actctaaatc
192721 tgacttggaa acttatagtg aaggctcagg tgttatttac atgaagtcta atggtaaagg
192781 ctacattgta actaataacc acgttgtatc tggaagtgat gagattcaag tcatcttaag
192841 caatggtaaa aaagtaactg ctaaaaaagt aggaactgat tcagaaacag acttagctgt
192901 tttaaccatt gacggaaaat atgttactca gactgcgcaa tttggctcat ctaagaattt
192961 agagccaggt cagcaagtta ttgctgtagg ttcacccttta ggtagtgaat atgctacaag
193021 tgtaactcaa ggtattattt cagctaagaa tagaactgtt gatgtaacta attctgcagg
193081 ccaggttact aaccaggcaa cagttatcca gactgatgct gctattaatc cgggtaactc
193141 aggtggtcca cttgtgaata tgtctgggca agtaattggg attaactcaa tgaaattgtc
193201 ctcttctagt gacggtacag ctgttgaggg tatgggattt gcaattccaa gtgatgaagt
193261 tgtttctatt attaatcaat tagttaagaa tggaaaaatt actcgtccaa aattaggtat
193321 aagaattgta tcagttgatg aattaacaga gtatggtcgt aagaagctcg gtttacctga
193381 tagcgttaaa tctggtgtct atgtagctag cgttactaag aatggtagtg cagataaagc
193441 aggaattaag tcacatgatg taatcaccaa gattgatggc aaagctgttg atagtgttgt
193501 ttcactacat actgctcttt atacacataa agttggcgat acagtaacac ttcaggttgt
193561 tagagacggt aagtcacaag atatcaaggt tactttaagt taagtgaata ttattaaata
193621 gagataaaaa agctatgatt atgcgttttg taagcggtaa tcatagcttt ttcttaatat
193681 ttaaagtgtg aaacgtaata aagttcgact tttaatgctg tcaagaactg aaagttatcc
193741 ataagattgt gggaaaatgt ggaaaactct gtggattgtg cataactcgg ttaaaataaa
193801 aataaattgt gtggaaaacc tgtgaattgt gtgtaaataa aattttaaat ttatctgttc
193861 tgaacggctg ttcaagttga ctatgtaggg aatagactaa aaagtttgga aaaatagtag

193921 tgtttataaa aatgtggaat aaattcagta aaaaagttat ccactttttt ataaaccaca
193981 atttattgat aatcaattag tctgaccact aaatatagtt tttaataatc cacagttagg
194041 ggaaaactga tgtggacgct atgtcaatta ggggataaaa ttaaaaacta gatgcgtgaa
194101 aggttagata gttataatga atatcaaaat tgtttgtgta ggtaaactaa aagaaaagta
194161 ttttaaagat ggaattgctg aatacgtcaa aagaatggga cgttttgcga agatcaaaat
194221 agttcaagta cccgacgaaa aagcccccga aaaactaagt cctgctgaaa tggaacaggt
194281 caaagaaatc gaggggaaaa gaattctaga taaaataaaa gataaagaat atgtgtatgt
194341 gacggcaatt aagggcaaag aaagaactag cgaagacttt gccaaggagc tagctgactt
194401 gactacctat ggacattcag atattacctt tgtgattggg ggaagtttag gaacaagcga
194461 tgcagtaaat aaacgagctg atgatttaat tagttttggt aaatttacaa tgccgcacca
194521 attaatgcgc ttagttttag ttgaacaaat ttaccgtgcg tttatgatta atagcggaag
194581 cccttatcat aaataattat tttataaaga ataagattgg ttgaagtagg attaaaatac
194641 tactaaaaat aagccagtct ttttttgtta gagtaattgg cacgatgatg cttcgtgctt
194701 tatcttcacg atatccatgt gaaatcatcc cttcagctag gctatttgac caagagatag
194761 ctgctaaaat tgctttaaaa tataatattg gcgaataaaa ggagagatga taatggcgca
194821 tgtccccaac taagcgaata cgattaactt cagcatggat tttaggaatt acgttgaatg
194881 cggctaaagt cccataggca aatttacttg gtagtttgaa attttgttct aatgagcgag
194941 cgagcgcaag gacactggtg gtttcagtaa atgtagctcc taaaaatata taagcgtaaa
195001 ttcgcgtaaa taagacgctg gcttggtaga ggctatggcc gggagtaaag taatagattg
195061 taataaagac cactaaagct gcaaataatg gaattaaaaa aatcaagagt agttttttaa
195121 gacttagatg cttaaatgtg agatagctaa gactagctac aataattatt atattggcga
195181 tcaaattagg aataagcgat atttcaagcg aaataattaa aatcaaaaat agtttcaatc
195241 cgggattcat gttatttacc tttttattt agtgtagaaa agtttctgat ctttgatagt
195301 taagtgatag tcacagagat gctgaacttg ctcaagttgg tggctaatga caagtaaggt
195361 gtggttagaa tctagaaagt actttttaag caaataaatg accgctgaga cgctttcttg
195421 atctaaacca ctaaatggtt catctagaag taaaacctca ggataagcca ttaacatcac
195481 gagaatttgc agctttcttt tttgtcctcc agatagagaa taaacaactt gttcgtctaa

195541 attctgcaag ttgagtttag ttagtaggtg ttctagctta attttattta
aggaaagatt
195601 tcgattgtac tttaggctaa agtctaattc ttcctttact gtaacattta
agaattggtc
195661 atctggattt tgaaaaattt gtcctacttt tgctagatag ggcctccgat
gccatttctt
195721 gatgtcttta ctttcaaata ttaattgtcc ttgatacgga ataattttgg
ttagagcctt
195781 aaataaggat gtcttaccgc ttccgttttc tccagttaat aaagtggctc
ttccgggata
195841 gatttttaga ttagttggag aaattagatt ttttttggga aaagatagtg
agaatgattt
195901 aaggaaatag actggatttt gttttaagtc aggaagagag aaagataatg
cttgattttt
195961 tagcgtgttg tcaggcaaag taatagtctt aatatttttg tctttaatag
ccaagaccat
196021 agaagctagc ttttcatagc cagaaaaatt atggtcacta attaaaatcg
tttttccttc
196081 tttgtgaagt gtagctaagc aagttagtag aaactgtcga ttgtgagagt
cgcaattagc
196141 aaaaggctca tctaataaga ataagtcgct tttcatggca atcaggacgg
ctaaggcaac
196201 gcgttgcttt tcaccacccg aaagctgaag aaaaggttgg tctaataaag
atgaaatatt
196261 agttttagta cttgcgtaat taatacgctt taaagcagaa gtacgatcaa
ctagtttgtt
196321 ttctaaggca aaaattagct cttctcgagg agtcgccatc gtgaattgtc
gactaggatc
196381 ttgaaacacc atgccccaat tagtgaatgg ctgttttaaa gaaccggcag
ttattttgcc
196441 attgaaagtc gggtcaagac cagagataag ctttaataga gttgatttgc
cactaccagt
196501 tggaccagtt aatagaacaa aatcgccttg atttataatc aaatttaaat
ttttaaaaat
196561 tggctgattt tgatattgaa aagatagctg attaagttta atttgtttgg
acgacatgag
196621 cttttaccaa gagattaatg attaatttaa ctaaaacgca ggtaaataaa
agcattgaaa
196681 gccaacgaac tacgaagtag aaaatcatgt taaaagttga gaagtagctg
tagccgttct
196741 taatgaagtc gtacaggtaa gtaacaattg ttgtcgttgt agcagataag
aaaagcgtga
196801 accaattgta gcggtggtag ctagtaaagg taaagcctaa ttcacaccct
aggccttgaa
196861 cagctccaga gattagattt actgcgcccc attgttctcc agcaagtagt
tcaacgcaag
196921 aacctaaaaa ttctcctaga aaagcagccc caggaagtcg aactaagaat
cccgctaaag
196981 gacctgccat acaccagatt cccatcataa tatcattggc taacattcca
tagccaactg
197041 gtgtaaggag ggcagtcaag acgttataaa gaccgtcact agctaaataa
attaagccaa
197101 aaataatagc aattaaagca attaggataa tatctcgaac atgtaatttc
ttcatttttt

197161 cctcccaaaa taaaaaaagg ccacctcaaa gaggtgacta aataaaagtc taaaaataaa
197221 tcattttccc tacgctgatg ttaatcaaac aggttcaatg ggtatattct cagccacctg
197281 gcaccccagt tatatttcta ggtttagctt acgcttaata agccgaaatg acaattaaaa
197341 atagtaaaaa ggttataatt aaaatagagt aaaagcaaag gggaccagtt gagatggcaa
197401 cagcgacatt aaaaagttat ttatcaagtt taatgcaaaa tactcaaggt actgaaatag
197461 aaatccgtac taaaaacctt cttttgccgt atagtcttta tttagaaaaa gtgcattatg
197521 attttactga atatcaatct aatactagtc aattaattta ctgcttagcg ggtaagtgtg
197581 aggttgaaat taataataaa actttttatt tgaaggcagg aaatatcttg ctaattgaaa
197641 aatacactga ctatacaatc aaagttagcg ataaaaatgc aattattgtt aaatttaaac
197701 tagataacga tttttcatgg cgaaaacaat tagagcaggt agatgccaat acacctaatg
197761 aacaaagatt gagtacatta tttttagaaa agttaaatca agatcgtgcc tttttattta
197821 caactacttc tgtgatgtgg gggagtcaaa acttgaaagg tataattcaa gactatctta
197881 ataatgtcgc ttttaacgga acaattggct tagagctgct taaaattata atcttgcgca
197941 atttgcgtga acaaaacttt aaatccaacg aagtaaaaga aagcacattt aaggatgaaa
198001 cgttagatca gtacatcgac caacactata atgatattag tttagcgcaa gctgccaaat
198061 actttggttt taatcgaaat tatttttcta caatggtaaa agaaaaaacg ggaaaaagct
198121 ttgttgagca tgttgatgaa agacggatga aagaagctag aagattattg gccaagccaa
198181 atatttcatt aaaagaaata atcgaaacta ttggatattc aagcaaatcg tttttctata
198241 agaaatttaa gcattattat ggcatgactc cagcagaaat gagaaagaga ctatttagag
198301 aggctcatat caatttgaaa taatagatac gtaaaaaggg atgcatgtgc atccctttta
198361 ttacatgttt tctaatctct taattctgtc agctgtaggc ggatgagtat caaatagtga
198421 actcaacccc cgcttgtgca gcggattttc gatgtataag ccagcacttg acggatctgg
198481 atctttcatc ggttcactat ttgaaatctt ttctaaagct gaaatcaaac cttggggatt
198541 tctagtgagc tcaaccgaac ttgcatcagc taaatattca cgattacgag agagagccat
198601 ctgagctagt gcggcagcaa gaggtcctaa aattagtgtg aaaacaatgg ccactatttt
198661 aaaaattatt tctagaggac tagagtcatc gtcatcacgg tcagaattgc cgccccacca
198721 ccaaatacgg gaggcaaaac tagagataaa tgaaattaca gcagctaaag caactccaat

198781 tgtagatact aaaatgtcat aatttcgaat atgagaaatc tcatggccaa gaacgccctc
198841 taattcactt cgattaagtc gctttcttaa tccagtagta acagctacga agctgtgttt
198901 aggatcacgt ccagtcgcaa aggcattagg actttcatcg ttgataataa agactcttgg
198961 cattggaacc tgaccagcga gtgccatatc ttctacaata tgccacagtt ctgggtcatc
199021 ttcttcatga atctctcggc catgattcat actcatgact aaatttcccg gattttgaag
199081 aacaataaaa agataaatta gactaccaat tagggcaatt actattcctg atagcggttc
199141 accgttaatc aaatatccta gcccaccacc aacaagagct agaataataa cgaatataac
199201 aagcaaaaat gctgttttac gcttattgcg tgctatttgt tgatagagca ttgataaacc
199261 tagaatttaa ctttaggaac agccttttct tcagttggtg tttgaaggta atccatattc
199321 ttaaatccgt gaatcttagc gactaagtta gatgggaaag ttaatagttt ctcattaaac
199381 atagcaacag ttgagttgta taactgacgt gaataagcga tcttattttc ggtgtttgta
199441 agttcttctt gaagtgacat gaagttttga tttgctttta ggtctgggta attttcagct
199501 agggcaaaaa tactcttcaa tgaatcagta atttggttag aaagcttaat agcttcttcg
199561 tgatcagaag taggaatatt tactaattga ttccttaggg caactacttt ttctaaagtg
199621 ccactttcat gcttggcata acccttaact gtttcaacta agttaggaat taggtcgtta
199681 cgacgtttta attgaacatc aatttgactc catgcttcat cggcatgaac tttagctttt
199741 tgtaagccat tgtagattcc gatataaacg gccaccagta aaattaaaat gataataata
199801 atccatgtta aagtcataga agtaaatcct ttctattatc ataaaaatat ggttaaaatg
199861 ttaggaatat tgtaccaaat aaagaaagtt tagaatataa aaaaagcgca attttgcgct
199921 tttttatgat atttaaataa caaagtccaa aactatccaa cctagaattg tcagaataat
199981 taaacctgca aattctaata ctgaaaatac ccagaaaaat tttctctttg aaacagtccc
200041 gttttcaaca aaactcttga aaacaagcat attggccatt gaaccaacga ttgaaccaaa
200101 tcctccaata tttgagccaa ggaataaggc ctccgcaaag ttggtaaatt taccaattaa
200161 aattgttgaa ggaacgttag aaatgaattg acttgaaata atagaagtca agaatacaga
200221 atgttcagaa aacattgtct tagaaattaa agtaacaata aatggaattt gctggatatc
200281 gctaataaaa atgaaaaagc atgtgaaagt tagaagtaga gcataatcca catgtaacat
200341 aatagaggga ttaataataa gagcgaggat cactgcaaca attgcaggga cgtatgcggg

200401 tacaatattg aaaacaccaa agaagaagaa aattgaaaca gcaatcgtta aaagcattgg
200461 tcgaatgctg attctaatgt cttctaaagg cacagtaggg attggcttat ctttaacaaa
200521 aaagataaac actaaaacaa taattaaact aactagtaat aatggaattg accaactaaa
200581 aaacttaatc ggtgaaacat tataacggtt aacaacaaat atattatgag gatttcccca
200641 tggtgtaaaa gcagcaccaa tgtttgcgcc catcccaatt aaggtgacag gcaagatctc
200701 aggtaaatga tggcgtctag caatcgttaa atataacggt ataagagtta aaacagttat
200761 atcattagtt aaaaacatcg cagagataat tgacaaaatt gtgaagattg cagttaatct
200821 tcgggtactc ctagcgcttg cagttagttt gtaggctaag acatccaaaa cgtgtaagta
200881 ggaaaaaatt tggatgattg taagcattgc tagaattgag taaagtgtat gaaagttaat
200941 atcttcaata cgcggtcttg cgaagaataa gcttatgatc gttatgacaa ccgtaatttg
201001 taaaatacgg tctttagcaa tatttttgat gacggtcatt aaggtcccct cttaaaatta
201061 attcaacact actattttga actctttacg gcaagttcgc aacgcttttt tcgcattagt
201121 ccttcctatt agtaagattt tgagcatttt attagggaaa aaggtaatta acctgctata
201181 tttaagttag taaaagagga cggaggaaaa taaatatggt tgattctaag aatctcgaat
201241 taaaaatcaa agatagtgct aaagacgttt tagaaaagaa aattaagcca ggacaagttg
201301 ttttgctggc tttaaatgat ggttcaaatg gttattctaa attaggggga acttgtacta
201361 tcggtgctaa ttttcaactg gtgttattag atcatccgga tccagagttt tcaattgagg
201421 ttaaaaacaa catgggttta gatatgtata catctgataa agaattagca tttctagacg
201481 atggcttagt tcttaatgcg cgcaatgcta ctctatcctt atcttctaat gaaggaatca
201541 tcgatggtgg agttactatt tctgaattca aaggcgaaaa actttctgct gatgaaatga
201601 aaaaattagg tggaaagatc tgctaggtat tgaagtgacg tgcttcttcg tggtatgata
201661 ataaaagatt ttcgaaaatt ttatttgaga ggaagtatag atctatgcgt aaaggtgaaa
201721 actacaacac tggtgttaca ccaaacttaa gacctaagaa caagaaaaac tctaaggctc
201781 gtgttaagag agctgctgaa gtagttgctt tcttaaacaa agctgctaaa gatgaaaaca
201841 agtaattaaa tttactgcat aagataaaga gagacgctag ttagcgtctc tcttttttgc
201901 atgtaaaagt tatataaatc ctagtttatt taataacatt ttgttgtaaa tgaaattata
201961 ttactatata aaaagactta taaagtgtga caaattaagc ataggataga attttaaatg

202021 aataacattt caagaaaagg ggtagaagta aaagcaattt ttgcccttgt tttaccgtct
202081 atttttttag gactggcaag taacttttct cgtaatattt ttttaggggt tatgctgctc
202141 ttaattctag gagcagttat tggattgagc gcaccaacta ttgttagtac ctggcttatt
202201 ttaattttga ctgtgctagg aattagtgct ctaactcttg gatatgtagc gatagatttt
202261 tatgggaagg ccttgctact tgtttccttt cccttagaaa catatttaac tagtcaaatt
202321 aaagagtgca tctttagatg gcaaatattt aagaaaaatc agtctagcgc ctatcgctat
202381 ctcaagcact atgatcaaaa cgtaaaatta caaacgacat ataatgcaga aaagttatat
202441 aaaaagatta agaaaatttt aacagagaaa aattatttac cactgtggtg tgattttaca
202501 attattgatt gggaacatga tcagcaattt gcgcaattta atttagaaga tcatgacgag
202561 attttgcggc agatagcaac agtacttaaa aagagcaggt tagttgatga gaatttatat
202621 tacctgggaa atggtcaatt tctgattatc tctaatacga tcgcaccggg tacattaatg
202681 gttttaaatg atgaactaaa agctgagtta aaagggctac agtacaagga atatcagcct
202741 gcttttaaga tggcaactca gcatattaca gagaaagatt tacttgagta tcctgacttt
202801 aaagccattc ttaaacgtct aaaaagaaaa cttgaaacag acttagttgt tgaatattta
202861 aagggagtag aagtatgagt aattggttct ttttcttagt aattttgatt actttaacat
202921 ttttagtggc attttttgct atgtttcgta acctctggac tatttatcat caaaccgagc
202981 atacagtacg tcatttagga agaaagcata atgttaaatg attttttagc tgtttcaact
203041 ttagtttcaa tttggttttc gcttttagcc tcattagtta cgctttatgg tgcaacttgt
203101 ttttggctta agcatagtaa aaaagttatt tcaattacgc cattaaagcg ttatcccaca
203161 attacgattg ttgttccagc acataatgaa gaagtggtga ttgcaaatac caccaaggga
203221 attcttaact tgaattatcc tgcatcaaag atcgagctcc tcttatatgc cgacaattgt
203281 caagataaaa ctgcagctga aatgcgtaag gcgacagatt tacctcaata tagatacaga
203341 aatgtacaaa taattgaacg tcatggttca ggcggtaaag caggtgtctt aaatgatgct
203401 ttaaagatag cgcagggaga atatatttgt gtttatgatg ccgatgcatt acctgaacaa
203461 aatgctctct atttttagt gaaaaaagtt ttagagaatc cccagcgata tatggcaact
203521 tttggcagaa ataaaacacg aaatgcaaaa caaaattttt taactaaatg cattaaccaa
203581 gaagttattg tatctcaaag acttcaacat gtaggcgttt ggaatttatt taaaattggg

203641 cgaattccag gaacgaattt catcattaat acagaatatg taaaaaagat
tggtggctgg
203701 caaagcggcg ccttaactga agataccgaa atttcttttc ggattatgga
agatgggtac
203761 ttgattgctc tggcatataa tttagaagcc tttgagcagg agcctgaaca
tttacgtgat
203821 tattattatc aacgattacg ttgggctaag ggtaattatc aagtggtgat
gaataacttt
203881 aagcacttat ttgataagag taactggcgt gtgaaattag aaacttttta
tttaacatgt
203941 actttctttt ggtttaattt agctgttatt ctttcagata ttattttttt
tgtggatttg
204001 ggatgtataa ttgcacgatt ttttaatcca gaaattccaa ttatctttgc
gatgaattct
204061 aatgttctct taatgcagct actattaatt aactggttat taatgatctt
gctctatgta
204121 agtcaaataa atctagcttt agctacccaa tatggtcaag ccactagtga
ccagatctgg
204181 ctagctttag tgtcatattt tacttattca cagctcttca ttgctatttc
tcttcaagca
204241 gtctgctcag taattggaga taagttgttt agacgtgatg gtactaagtg
ggtgaaaacc
204301 aagaggtttg cagattagga ataagaaatg aaagtaaaga atttaatact
aattttagga
204361 actattattg cttatttagg aatcattggc tatgtgagga tgagcaatga
tcgaactcta
204421 gaagagcgat attatcatga gtggcgtgaa gattatataa aaaataaaaa
tcaaaatgaa
204481 caatacgtaa atgcagcagg ggaaaagggc ccttcgtttg ctttatctga
agcacaaggc
204541 tatggaatgt tactggctgc taaagcagga gagaaacatc taggaagccg
gattgatttt
204601 caaaagctag acaattatta cttggctcat cgcttaacaa attcaaattt
gatgagctgg
204661 agacagaaag ataggaaaaa tatttggcgt gataatcctg tgagtgcaag
tgatggtgac
204721 ataatgattg ctcaagcttt gctacatgct aatagagttt ggccaggaca
tggctacaag
204781 tcacaagctg ttaacttaat taatgatata aaaaggtttg agattaatca
gaaagctaaa
204841 atggtaactg tcggaaattg ggctaataaa gactcacgct tttataatgt
cctgaggaca
204901 tcagatgtaa tgccaaaagc gtttgaagaa ttttaccaag cgaccggtga
tcgggaatgg
204961 ctaacaatta aaagcaaaat gcttaactat ttgcaaaaat taagcaagga
acacgatact
205021 ggtttagttc ctgactttgc atgggtttct aataattatg tgaaacccgc
aaaagctaat
205081 gaagtggcga ctaatgatga tggtcactat agtgcaaatg cttgccgagt
cccaatgcta
205141 ttagcggaaa gtaatgataa gaatgcggct caagtagtaa agaaaatgct
aaaatttttt
205201 aagaaaaaag gaacttcggc tggttttacc ttaaaaggta aaaaattaca
ttattatcaa

205261 tcagcgagct ttagtgcacc aatctttgta gcagctcata agtatcgtaa tcaaggctat
205321 gatactttgg ttgaacaaga aaagtatatc tttttaagat ccctttcgaa agataactat
205381 tacgatgcta ctttaacgac tttagctgct ttaaatacaa atgagttgac tggattagaa
205441 aaatagagaa agattaagtt gttgaatctt tctctatttt tgatttgcaa gagtagaacg
205501 gttttgctat gatagtagaa attttagaca tttttatatt tctggaaggg gtaagttttt
205561 atgggattaa atgcatatat tcaaggtttt aatagtttag agtcaatcga tcgtgcacca
205621 ggttatttta aatatcagca tcattctgtc gctgatcata gttttagaac ggctgaactt
205681 gctcaaatga tgggagatat tgaagaagtt gttggtaagc aaaagataaa ttggaaagct
205741 ctatatgaaa agagtcttaa ccatgattat actgagcggt ttattggtga cattaagact
205801 ccagttaaat atgcaactcc acaattgcga aagatgattg gagacgttga agaaacaatg
205861 actgccaaat ttattaagga cgaaattcca aaagaatttc aaaaaattta cactaagcgt
205921 ctatctgaag gtaaagatga tactttagaa ggtaaaattt tatctatttg tgataagtta
205981 gatttacttt atgaagctta tggtgaaatt gaattaggaa atcccaatcc agttttcatg
206041 caaatgttta aagaaagtct tgaaacgatt aaaaagtttg atgacttaac ttgtgttcaa
206101 tattttatta agaaaatttt gccagactta tttaaaggcg actttgctgg taaagataag
206161 atgcaaagaa ttgcttttag tattttacta atgggggaag aatagtttga aattacaatg
206221 cgcatcctgc gggttgcgac ttgatagcct tcattttaag caagaaggat taatgaatcc
206281 caagttaaca agcatttgtg atatttgctt aacacgcaat ctggatgcag aagattatga
206341 aaacttagta attgaaaata tttcccaggt aatgctctat ggctttgtag gaaagaaaaa
206401 tacgggcaac gctaatccgg aggctttaaa tcagtatctt gtaggaagcg atcatagacg
206461 ccaatatgaa tcttttattc aagactatga tggtaatgaa gtagctttgc agcaattgcc
206521 acgattcaaa tttaatcagg caattattaa gagcgaagat aaagaaatag attatattcc
206581 taatagcaat gttgattttg ctgttaatat gaaaaacatg ggaattgaag tcgacttctc
206641 cttaaatgaa gtagattttg ttgaccgtga aagaattcgt cacaaatata attatcgttg
206701 ccaatattgt ggtagacgcg gaacaagcgt agatcacaaa gatccagttt ctttatcgca
206761 tgataattct tttgataatc tgattttgtc atgtagcgag tgtaatcgaa ttaaatctaa
206821 tatgccctat aaattattta ttaagttgaa taatcaactt agtgttgtaa ataaaaaatt

206881 ggttaagtac gaagacactc ttgctaattt aaaagaagaa tttcagcagg
caaaacgtga
206941 tttagctgga caagttcatt tgaagggaat agttaatgac cctgaattaa
atgcgatgag
207001 aaagcaaaat aaaaagcttc aagatgcgat tgatagctta caaagtgatt
atgatgcctt
207061 aagaaaattg cgtaagacat attttgaaac cggctggaag ctagcacaag
aaaaagaaaa
207121 tagtgagatt atatagaaaa aatgcattgg acaaaaatcc aatgcatttt
tttactatct
207181 taaagttcat tgcttctaaa gttacgtaag aacttcttag tcttctcttc
ttgaggatta
207241 ttaaagatct cttgtggtgt accttgttcc gtaataactc catcgctcat
aaagataact
207301 tgatcagaaa cgtcacgagc aaatcccatt tcgtgagtaa caatgaccat
tgttaatcca
207361 gttttagcta gtaactggat ggtattaagt acttcaccaa ccatttcagg
atctagcgca
207421 ctagttggtt catcgaaaag taagatttct ggatccatcg aaattgcccg
cgcaatagca
207481 acacgttgtt gctgtccacc agatagttgt tgaggctttg cagttaagaa
aggctccata
207541 ccaactttct ttaagttttc aattgcaatt ttcttggctt cctctttaga
gcgtccaaga
207601 accatttctt gaccgatcat acagttttga agcacatttt tattattaaa
caaattaaat
207661 tgctggaaaa ccataccaac ctttgatcga aatttattac gatcaaaatg
agaatctaga
207721 atattgttgc catggaataa aatcttacca ttacttggtt cttcaagaag
gttaatacat
207781 ctaagcatgg ttgatttacc accaccagat ggaccaataa tggtcattac
ttcaccttta
207841 ttgatatcaa aagaaatgtc ttttaatact gtgtgatcgc cgtatttctt
ttgtaaatgt
207901 tgtacttgta aaatgttttc ttcgttatta ttgttcatgt tcaaatccag
tctcctttgg
207961 atcttctact tgtaattgat tagccattaa gttgtagttc tttggacctt
caagcttctt
208021 ttcaatgaaa ttaaaaattc tagtgattga gaatgtcaaa attaagtaga
tcattgaaat
208081 aatgaagtaa gtttggaaga attggaaggt ttgactggca acagttgtac
caacaaagaa
208141 caattctgat acagaaataa tacttaatac tgaagtatcc ttaatgttaa
caataaattc
208201 attagtaatt gatggtaaac agtttctgat tgcttgcggt aagataatat
gccacatttg
208261 ttgtgagtga gtcataccga tagctgaagc tgcttcaaac tgacctttag
gagtagcatt
208321 aataccacca cgaataactt cggctaagta agcaccagta ttaattgaaa
caattactag
208381 agcagctact gtacggttta agttaaggtg ccataattgg gcaattccat
agtaaataac
208441 agctgcttga accatcatag gagttccacg gaacacttca atataaactg
ctaaaatcca

208501 gtcaacgatc tttaagcccc actttttacc agtagactta ggggttggga
tagttctaac
208561 aatcccaaca agtaaaccga tgaagaaacc aacaatagta ccaactaaag
ctaaaagaag
208621 agtcattcca acaccgccaa ggatcattga accatagcga tgcataattg
acatgaacca
208681 gttttctttc ttgctgctac cggcttgagg ctgctctttt acagcatcag
ccattaattg
208741 gtcgcgcttt ttcttagaaa taccgtttaa aatgctgtta acttggttaa
gtaattcagt
208801 gttgcctttc ttaacaccaa ttgaagtaat tgaatcatcg tgggcaacct
tgaatccctg
208861 catcttgttt aggttaacgg caacgatgtt agggttaact gccttataac
tttggtattc
208921 aatatcttct gcaacataac catcaatagt acctgattgc aagctttgac
gcattgcaga
208981 aaagcttctc attgctggtt cacgcttagc accatgtaat tgcttaatta
agtcatagtg
209041 caacgtacct tgttgagcag ttaattttgc tcccttaaaa tcgtttaagc
cttttgcttg
209101 agcaaatttt ccagtcttgc ttgtaataac aacgaaagta ctctttctgt
atggttcaga
209161 gaaattgatt gcctttctac gttcagcagt aggagacata ccagcaataa
ttaggtcgat
209221 tttgccagaa gttaaagctg gcaataaacc atcccattcg gtttttcaa
cgacaacttt
209281 acgatgaagc tttttaccga taatttttgc gatttgtaca tcatatccat
ttgcatattg
209341 cttagatcca tcaattggaa ctgcgccatt tgcatctgta gtttgagtcc
aattgtatgg
209401 tggataatta gcttccatac ctatttttag gggtgcttcc tttttagccg
cttcggtttg
209461 ggtagaatta gaactaaaac cgataaccaa acttaaaatt agggctaata
aagcactaaa
209521 ccatcttatt tttgacttca ctttgaaata cctccaaaaa ttatttcaaa
taaagccaat
209581 acaataaaag cgtaaataaa aagcctcgct gtttaccaaa aacaacgagg
tttgaaaatc
209641 atcagtaatt aaaccaaatc atatagcgct ccctggggac taccaggaca
gtatgtaaga
209701 tatgctctca catcccaaca agcttaattc gcgaaagtaa gcttgtttcg
gcgataatcc
209761 taaaaaacta ctgtcaatgt attcgcgtac tcaagtagat tttgtggtta
tcgcaacctc
209821 tattgcattg gggatttatt taactgatga aaagattaaa cgatttttaa
tcttttgtca
209881 aataaatccg tgattttttt taacatcttt tttcttaata tctgaaacat
agggattacc
209941 agcaactata aatcttagtt ttttcttggc ccatttagga tctgactgat
tgatcccgac
210001 tcttggaaga gcagtaattt cttcaatttt acgtttatga ttaatgtcaa
tatcgaaggg
210061 agaatcttct aaccgagcta aatcccattt tctactagta attcctaaag
cctgcatcat

210121 ctttccagga ccattagtta aaagtggacc agttttgcca ttacggtttt taatcatggt
210181 atcaattcca gttagtgggt caatggccct aattaaaact ccttgtggtt cgcctgctgc
210241 ttggcaggca acatcaaaga aaaaatattg ccgctgagaa tagatatata aactgccacc
210301 aggacgatat aagccttcat ttgcttgact gcgtctacca ccataagaat gagcagcacg
210361 atcttttaca ccaacatagg cttctgcttc aactattgtg cctgataaaa tttcgtttcc
210421 attattgaaa gatagagttc tacccaacag atctttgcta atttcactag tagaccgatt
210481 agtaaaaaaa tcttcatagt tcatattgct acttctttct aaaaatgttg tacactcgta
210541 tttgaaagta aatataaatt gtaaggagga actaaaaagt gaatgagata aaagttgtta
210601 gaatttatga tcatgagcaa cccgcaggtt atagaatctt agtagatcgt ttgtggccaa
210661 ggggaatgag caaggtaaag gctcatttag atgaatggga taaggaaatt ggaccaacta
210721 atgaattaag aaaatggttt aatcatgaag acgataagtt tcctgaattc aagactaagt
210781 atatagcgca actgaaagct aatccagtaa ccccgaattt tattaagaat gtgaaagaaa
210841 agttagctca agaagatgtt attttcttat atggtgctaa aaataaaaag cacaaccagg
210901 cagttgtact taaagatttt attgatagtc aattaggtta aggcattttt ttgccatgta
210961 agttttgtcg aacgtaatga aaaataattg ttaaaataac tgtaccaaaa acaatgcagg
211021 caaagattgc atcgggcaat ttaaagttta gcggtggcaa tgaaacaagt agtttaactg
211081 caataattgt aattaaaaca taggccattg tttgaagttc aggaataatt tccattaatt
211141 tgataataac ttctgcaact cctcgcatgc ataaaatacc aatcattccg ccaactaaga
211201 cgacaactgg attatttgac attgctaaag ctgctaaaac agaatcaatt gagaagacga
211261 tatccattga ctcaattgaa ataacagttc tccaaaataa tgaaagatgg tgctttcttg
211321 ttttggagtt ctttttcttg tgggcttctt tttttgctgc ttctttctta gcaacttgtg
211381 caggatggcg aacatcataa aaatacttat agactaaata aagtaaatat aaaccaccta
211441 agagtttgat ttcccagaaa ttaattaggt aagtaccgat cccaattaca ataaaacgga
211501 atagataagc accccaaagt ccatagacca atgatttcct ttgctcatct ttagttggta
211561 aaacttgcgt ttgtgcggct aaaactaccg cattatcaac tgatagcaaa cactccatta
211621 gaattagagt cagaatcatc atccaatctt gtccgctagt taggacatga gcccagttat
211681 tagcgtcaaa aaaaggctga taaagtttaa tcaattgcat tcaaatagcc ttcttcaagt

211741 tttattaagc gttgcttttc cttctctccc attctataac ttcccacctt gtctgatgct
211801 aaaattacgc gatgacttgg aataaaaatt agggatggat ttagggctac cgcatgtgca
211861 actgttcgaa ctgaagttga gccattaata gcagaggcta aatcattgta tgaaatggtt
211921 gttccgtaag gaatattttc aatcatttta acaacttgat tttgaaatgg agtaccaaag
211981 cttgaatagt cgataggaac ggtaaaagag cgacgcgtgc ctgcaaaata ttcttttaat
212041 tgttgaacat aaggagctaa tttctttgaa tcttgaacaa gaatgtggtt aggataaaat
212101 cctaaaatag aagattcaat attactattc ttggaaccaa cgaatgctaa tcccaagtta
212161 ctgatgacta attcataggt ccatggctta atttcaacaa aatcgtaata aaaaactttt
212221 gcttgggcaa gtggcattta atcctaacct ttcttttaat taatatatta actccatctt
212281 atattgtaca acaaaaaata acagaaagag ctatggataa attaaataaa atgttagtta
212341 agataatatc ggttatatat ataatttgtc aaatactttt tcagcatttt caataattgt
212401 taaaacttct ttaggagctg gtacaggagt attattgata acccatactt tagcagcagc
212461 ttgtcgatag gcaagaagtt gagcgaaagg atagacaaca aaactagttc cagcaataat
212521 aactaagtca ctattttgaa ttgctttgat tgattcagtt aaaaccattt cattaattgg
212581 ttcaccgtat aaaacaatgc caggacgaat tattccgtgg tcttttgcgt gtagatatga
212641 ttctttatat tcttcgtatg aaatttttc atgacactta gtacaaaaaa tattataaag
212701 attgccatga aattcaatga catgtttgtt gccggctttt ttgtccaagc catcaacatt
212761 ttgggtaatt aaagtgccat ttttattaca gctagctgca attttttggt gaataatatt
212821 tggttgagca ttaggaaaat acatattctc cattacaaag tgatggaact tagctggttc
212881 gtgaaagagg gtgtcctcgc ttaaaatttg ttccgggctt tcagatatgc cattataaat
212941 gccatttttt gatctgtagt caggaatatg agagggcgta gaaactccgg caccagttaa
213001 gtacgtgaca tggtgagcat tgttgagatc tgcttgcaga gcagcaattt gattgtctaa
213061 atccatatga aacctcctat acttttaatt ttcaactaag tcaattatag aatgctgaga
213121 gaaaaagtgg tatttgttat aattgaagta agaattttaa cgttaataaa taattttaga
213181 aggaaagata tgtcactttt gtatattgct tttgatattt tgctaattgg ctatattttt
213241 tatagttggt attggcaagc aaacattgat tataaggctc gctttagaag ttcatcggtt
213301 atttgggcct tgatttttct attaattgga ttttatttag attactttac tgatccaact

213361 ttactcatga atgtgtttat tgcgacattc ttgttaatga gcattattga
tggcgttagt
213421 ggattagcta aaaagagatt agttgtaagt ggatatttta aacgaacagt
taagtatagc
213481 gacattgcac atgttacttt aattgcagtt cctaatccta aaaagcctac
tgtaatggcg
213541 atatttcaga ctaataatcg gcaagcgtat tatctacgct tttcacagca
aattagtgat
213601 gtaattgcta acatcagaaa atatttaggt tcaaacgttg gtattgaagt
gcagtcaatg
213661 atgtaaatgt aaaaatacga atataaaaaa aggataagta ctctgatcac
tacacgaaga
213721 agcgaaagag tgcttatcct tttttgtttt ttataaaatt ggagaaagta
atctagaaaa
213781 ggcctgttta aagcgaagat aacgagacca attattggtg atgtctaaag
ttaattcagt
213841 acagttagta agatcctttt taaaaatctg atttaattcc cgggtaatat
ttttatcgta
213901 gaaaacggca tcggtttcaa aattcagact atatgagcga aagtcttgat
tcatagaacc
213961 aactgctgaa aatcgttcat caaccacgac agtcttagca tgaataaagc
cattgttata
214021 gacataaatt tttattccct catgtaataa gtagtttgca taccactgag
tagctcggta
214081 aatgaagggg tgatcgggca ttgaaggaat catgattctg acgtcaactc
ctgaacgggc
214141 agcaataacc caggtcgcga tcattggatc gtctggaact aggtaaggcg
tttgaatcca
214201 gagcgttttt cttgccatca tcatcattct aatcatccca ttacgtaagt
aaggggtaga
214261 ggtatcaggt ccatctgaca cgatttgcgt tgcaacatca tcttcagtta
gcttattttc
214321 ttcaatcttg ggaaaaagct tttcattgaa tgaaattgtc tcattcttat
tagtaactga
214381 agcattccag tccattacaa aacgttcttg gagcagtagt gaggctgatc
ctacaattct
214441 aagatgtgtg tcgcgccagt aaccgaactt tttgctttta cttacatatt
ggtcgccaac
214501 gttgaaacca cccgtccaag atattcggcc atcaacaaca acaatcttac
ggtgaaggtg
214561 ataattcatc cgatattttt tgatcatatt ctgagacgtg ataaaagctg
ccacttctcc
214621 ccctagatca gttaaaggct taaaccaagc ttttgaagca cctggcgatc
cccacggatc
214681 ataaatcaat cttattttca cgccttcacg agcttttttg attaataaat
taagaaactt
214741 gtttcctaaa tcatcattca tgaaggaata atattcaaca ttgattgttt
ctttagcctg
214801 attgatatca ttaaaaagag ctttgaattt ttcttctccg tcagtataaa
aactaatttt
214861 attgttttta gaaagcgggg aatcatattt aaagttgaaa tagtcaatta
aaatttttgc
214921 ttctttagaa gtatctgttg gtccagtatc cttagggata cgcggaatca
tcttatttac

214981 tttatttaag ccgatgtgtt tttgattatt aattgcaaaa aggttttctt gcgataatcc
215041 acgcccaata aaaccgtaaa gaataaagcc gagtacagga aaaataagta aaataattag
215101 ccaggcccaa gaggtagcga ccgatctttt tctatggaac actacgtaaa aggctaaaag
215161 tgtattaact gcccaaagaa gtgtccaaaa ttcatttgaa aaaataatta tccctccctg
215221 ttaactcagt tgttatcgtt gttatgtcct ccaaaaataa gaagaagtct aaataattgt
215281 agcatgcttg ttaaaactgc tgcgacataa gttaaagctg ccgcaaataa tactttacgt
215341 gccatcggca tctcttgtgg agataaaata gcgccattat ttagcattct tagtgcacgg
215401 tgtgaagcat taaattcgac tggtaaagtt actacctgaa aaagaagaac tagtgcaaaa
215461 caccaaatgc caattctaat caaagtttga ttcattccta agactagtcc cagaagaatt
215521 aatggcattg ataagttaga accaagagaa acaattggca ctaaggcaga ccgcatacgc
215581 attggtgcat agtggctagc atcttgtaag gcatgtccga cttcatgtgc tgctactcca
215641 accgcagcaa tagaagaaga gttataaaca gactgcgaaa ggctgagaac cttattagta
215701 ggattgtaat tatcggttaa gttcccagca acctcacgaa tttctacttc atataaacca
215761 gcttggtcta gaatataacg agctgctacc ttaccagtgt aattaccttg atttcgaact
215821 tgatcgtaac ggttaaaagt cgaattaaca ttcattgagg cccagccgga aattgctacg
215881 cctaaaataa tcatccaaaa atatgggtca tagaaaaatg gcatatagta cataacgatt
215941 ttctttcttt ataaaattaa tcagttaact tcaaaaactt attgataata ttaattacaa
216001 aaggattatc atgtagaaca ctatgctcag ctgcgtcccc gcgaacttcg acttcttgga
216061 aggtgtgggc accaggagcg aggatatagc gaattgattt agcagaaaca acagaaataa
216121 acctatccgt attagtttca tctaagatat ttccataaat atttaaaaca gaaatattag
216181 gattaaagcg acgctggttg aatagcattc ctaagtagga aatactcatc aaaataggac
216241 gtccttttc attaagacga ttaacattgg gtaaatcacc taaatacatt actccatcaa
216301 aaggaccagc aattaaagca caatgatcta ggtggggaaa gttctttctt aagcttgttt
216361 tcatttcaac ctttactact gaagggggcgc ctagagaatg ggcgactgca ctatattttt
216421 tgaaatggta tctcttactt aagtaagaca aggcagtgcg caagtaatag gaacttgcat
216481 aaacgccagc aattcggtct ttaaaaacta attgaacgat gggttgtgga tcagctgtcc
216541 aagttccttc caaaataaaa ttaccaaagg gatcaattgt aactttaaga aatttatctg

216601 tgccttttgc cttgcaagca gcatgaacca ttttgttagt tgtataatcg cctccgcgaa
216661 aaccatgtac gtaaaaaatt ggcgtttgcg taattgtaga agttgctgct tctgttgttg
216721 aactatcttc aactttgcgt ttgtgcattc tgagcagcca gcgagtagga gtaaaagtag
216781 ccattaaacc taaaaaagtc attcttgcaa tactaggttc tttattttta tgtttattat
216841 tcttttcaac ttcttcgcta tatctttttt gaataaaaaa catgctacat cctcccttcg
216901 tattagtttt agcgttaaaa ttattctaac attttatcag ctagagcaga taggaaagga
216961 atatttatga aatactatgc agtaaaaaaa ggtcgtacac cgggtgttta ccgaacatgg
217021 gaagatgcta aaaaacaagt tgatggcttt tccggtgcgg agtataagtc ttttgaaaag
217081 ataactgatg cgactgagta tttagactgg aataaagaaa cacaaccaga tattgttaaa
217141 gaagattctt taagcaatgc gattaagaaa atccaagctg ctaacatcac acaccagtct
217201 aagaagcaaa aagtaaaaaa gactagttct aataattcag cagactattt tgcaacaatt
217261 tatacagatg gtggatcacg aaatactggg gtacataaag gtgggcacgt taataagact
217321 gatagagcag cttgggctta tttaattgaa tggcaagatg gccgtactta tggatctggc
217381 ggagaattcg gcgctacgaa taataagatg gaattattgg gtctaattaa tgctttagaa
217441 aaattgcttg agttgaaatt taatgacaaa catcttttat ttgttttaga ttcacaatat
217501 gttttaaatg gaattaataa acactggatt gagggatgga agaaacgtgg ctggaagcga
217561 tcaaatggcc ctttaattaa tgctaccgaa tggaaaaggc tagatagttt actaggtcat
217621 tttccggata gtacttttag ctggaccaag ggacacgctg ataatgaagg caacgttttt
217681 gtggaccatg aattaaatcg ttatatggat aaaatgaagt aataatctgc ttagtaaaca
217741 aaaaaagtct gcaatttgca gacttttta atgcactttt tttagtagtt gctataataa
217801 taactgaaag actagaacgc acgcaggtac tagtttttga cgacggagac ggctatttta
217861 agccgtcttt ttttaacgtt ttagtttaag ataatgacac cgccaaagat acttctaaac
217921 cagctaacga ttgaatcaat taatttttga aaccagttac gagtttcagg agtattaaat
217981 tttgaaaaga tattcttagc accagattga atttggtctg ctagttgacc agcttgttgt
218041 tggaagtttt tgtcttttaa tgcaccagaa tttttaatct taattaagac attaataatt
218101 tgttcttttt gattattggt aatagtgtca ccgaggtggt taatgttaat ttggttatta
218161 acaatatccc tgatttggct atcagaaata ttttgcccct gtttagccat ttcttttttt

218221 gcaccagcta tggcattgtt taattgggcg tcagaatagc catcttttcc cttgttttgg
218281 ctagtaatct tacttaaggt attcatttca gtttgtgctg cattaatttg attttgattt
218341 aaagaattgc catttttagc atatgcggca taaacgccgg ctagggcacc tgatccgtca
218401 attggtgtag cagaagtgac gtaaatgtcc gcgtcagaaa taccagcagt taaagcagca
218461 tttttgtatt ggttagcagt aatagttgta atgttattat ggccattata aggtaaaata
218521 tgaacattaa tcccgttacc agagctagtt ttttggatca tggcactaga ccaaacgccc
218581 gaattattag tgaagtcata gcctgaagga ttaagatatt ttacaagatc tgcgccagta
218641 atagtgatgg tggtataatt actaccattt aaggactgcg tcagagtgtt aattgttccc
218701 tgcttttgag aagaggtaag ggatgtacct aaggttacaa caggagtgtc attttcattt
218761 gccttaactg ttttagtatt actaatagca gttaacccta aaattccgct tgttaagata
218821 gctacaagcg cggttagtat tttttcata ataaaatttt ctccttatga ataaaatata
218881 ctcaatatga tggctatatt ataacctaaa tagctaactg aattatgtaa ataagtatta
218941 agatgctata atcataagca aaaggaggca gacgctgtgt tacgccagcc aatgttaacg
219001 gtaattatgc ctgtctacaa tatggaaaaa tatttgggcc gggcattaga agctttagca
219061 aaacaaaaag ataaaaattt taaattatta attgttaatg acggatcaaa agataaaact
219121 cgcgaagtag ctgagaagta tcgtgatcgg tttctttatt ttgacattat taataaaaag
219181 aatggtggcc tttctgatgc tagaaacgtt ggaatggctc atgtaactac cccatatttt
219241 acttttcatg atggggatga ctgggttgat cctggctata ctgcttattt tgtaagagca
219301 tttgatgagc atccagatgt agatattgtt tcttgtggtt tttggattga ttcagaaaaa
219361 agacatgaaa gtcatccagt aggaagtaag caaagcgggg gcttgatcga caagagagaa
219421 gcttatttga agatgactaa tgtctttggg tcacctgtaa aaggatatac ctggaataag
219481 ggatataaaa ttagtgtcat taagaagtat catttgcgtt ttgttgaaga tttagccttt
219541 atggaagatc aaatcttcaa cgttaagtac gtatcagtga ctaatggttt ttactatagt
219601 tcaacacctt actatcatta ttggcaacgt gcggatagta tggtgcatga tcttaatccc
219661 aagaaaatac cagataattt ccgagctaat tatcgtgtat ggcaccaaat tattaaaagc
219721 ttgatggcag agcgtgaagc tcaaaaagag aataaaaaga ttgcaaaaaa agcatcttta
219781 agtgatttgg atcatcaaga tttataagaa tttatagcta aaggaattgg tatgaaagta

219841 aaagttaatg gagttgaaat ttactatcag aaattaggta aagggcatcc tttaattttg
219901 ttacatgggc accaccaaga tggtggaatc ttcgataaat tagttgcgcc gctttcttta
219961 tattacacag ttgtggttcc tgatatgcga ggccacggtt taagtgaagg agaagcaagc
220021 gagcattatc aaactgaagt tgaagattta cgggctttta ttagcgcgct taaacttgaa
220081 aagccttata tcttaggctt tgggtctgga ggtttagttg ctctttcttt ggccgcccaa
220141 gcaccagaac tggtttctaa aataattgtt gctggaactt acgttaatgg aaatggcgtg
220201 aatgcaaagc acattgcggc taatactatt cgaggctttt ttaagggaga tcgtgacagc
220261 agagtggctt taagagagag tcatattccg gttgaaacgt taaaacggat taagacgcct
220321 actttatgcg tagtaggtga aaaggattgg gttaaggttg aacatgttcg ctggtatagt
220381 caattgcttc ctaattgtcg tttagtttta atgcctcgtc agacgcatta tagttatgtt
220441 gttcatagtt taaagttact agatttaatt aaagattttt gtaaagagag ctaaaaaagt
220501 agcgctcttt ttctttttga tctaaaaatt cgatagaatt aatatataaa tggaatttaa
220561 taaatagcag aaggttagaa taatgacaaa aatagtggtt caagggatga atggcgagat
220621 gtctataaat gatgaaaaaa ttacaattaa gcatactgcg ccgttatttc caggtaaacg
220681 agaagtaata ctagatattc acagtttaca agctgttgta tataaaaaag cgcacatctt
220741 aattaatggt tttctaaaat tggttcctaa aggtgataat cccatgattt atcaaacggc
220801 tagtttacac caaatgggaa aagatgagct ggcaattgtt ttgagagctt ttgaaaatac
220861 aaatcccaaa gatgcagagg atttttataa ttatgtcgtt ggtcgcttag atcaaatcaa
220921 ggcagcagaa aataatcagt tataatctat agctagaaaa gtagagcaaa acaggaattt
220981 ttagttataa taaaactagc tgttaatttt acaaggaagg gcaaattgat gaataaagaa
221041 gaattgcacg aaatgttgca cccaatgaaa ttaatcggtc ttggtggaaa ccaagcttta
221101 gcagagagaa ttgcggcagc tttggataaa ccattgttag agactgctgt tcaacacttt
221161 agtgatggag aaattcaagt taacattacg gaaagtgtta gaggctgtga tgtctacgta
221221 attcaatcta ttcaagatcc agtaaatgaa aactttatgg aattgatgat tgtattggat
221281 gctttacacc gtgcatctgc tcactctgtt aacgtagtaa ttccttatat ggcatattca
221341 cgtcaagata ctaagaatcg ttcacgtgaa ccaatcactg ctaaattatt agctaacttg
221401 cttcaattga ctgatattga tgacttgatt gctgttgact tacatgcatc acaaattcaa

221461 ggcttctaca atattcctgt tgatcacttg cacgctatgc caatcttagc tcaatacttc
221521 ttagataacg gtattgcaag taaggatgat gatgatgtag tagttgtttc tccagatcac
221581 tctggtgcaa aacttgcacg tacttttggc tcatactttg acgcaccgat tgcaattgtt
221641 gatcaacgtg gtgcacgcta cgatgcagaa gctcatgata tgattgggga cgttaaggat
221701 aagactgtta ttattgttga cgacttaatt gatactggtt ctagaattgc ttcttcaact
221761 aagtcagttt tagctgctgg agctaagaaa gtttatgttg cagcaactca tgctttgctt
221821 tctcaaaatg caattgatgt attaaacgaa ttgccagtag aacaaattgt tgtaactgat
221881 actattaagc ataagaagta tccagataga atggttcgtt tatctgttgc acgtttgctt
221941 gctaagggta ttgattacat ttacaacgat aagtcaattc accgaatctt tgatgaacaa
222001 aatagaattc aaggatgatt cacattaaat tttaatattt attaaaaggc tgaaaattag
222061 taactatgct aatcttcggc ctttttattt acgataaatt tatcctattg ttgaatcctc
222121 atcaataatt aagatataat taaacgaaaa ttagcataaa tgaggtgatg aggatgaaga
222181 aagtgtttca ggcgcaacta tatctaaata ttttgatagc tgtaattatt ttaattaatt
222241 atcatacagt taaggattgg atttatcttg gaattttagc tctagcagtt gtagtaagta
222301 aaaataaaag gatttcgcag ctaataaata ttgtacttat acctatgatt tttatcgatc
222361 aagttagaaa cttgagtgat attctcattc agcacttttc tcagttaaca ctactcatct
222421 tttggattta tgctgttggg accataatag tattgattcc ggttacaata gtcgaatatg
222481 ggaaaattaa aaagccaatt tggcgattga tagcatcggt ctggatgatt aattttgtta
222541 ttatgttttg ctatctttta accttaaaaa atgtaaatcc agacggcttt ttagtgagtt
222601 taaataaatc tggcttggtt tatgctttag caattttagt ttatgtatat tttgctgtta
222661 aaagttgggg ttatgaattt tgttttaatt taccaacttt taaaggcaaa aaattgcaac
222721 tgctttcatt tattttaatt tttgggatag cgatttggct ttcatttttc caaacattca
222781 gtagatttgc tcagagatgg caagaattat tctggaattg ggattttttct ttacttgatc
222841 ctactgagtc ggtccgtcta aaaaacgctt ggtctgtctt tctctactca atagaagcgg
222901 gaattggtga ggaagcagcc cgatatataa acttagtttt attattagtg atttttaaaa
222961 gcaaaaagcg gcaaattaat ggtgcagttt taggcagtgc aattctattt gcattacctc
223021 atattggaaa tgcctttgcc tcagagctta agcaaacgcc actagcaact gcttttcaag

223081 tgattgatac atttggcttt gggtggcggc agtgctaatt ttatatagcg
gtaaattatg
223141 gccgacaatg gtaatccatg cactatatga tattcttaca ttttctgaaa
ccccattgac
223201 tcaagatggc gtgggaattt ttggtggaaa tataggttcg cttattcatg
taattattag
223261 tttagtactt tgggtaagtt ttgccgcttt tcttttaatc aagaacagaa
aattaataaa
223321 acagaatgtc caaatcttga cccaagtcca aaaaacagat ttgacaattt
catgaagcaa
223381 gttataatac aagcaaataa aatagctaat tgatttacta tacaagaatt
ttaatttcat
223441 aaaatagaaa tgcctctagt ataagttaag tactagaggc atttttgtct
ttaaggggga
223501 gtataaaatg gatgaaaagg agaagctaaa actaaagaga tcgcttaaga
gccgtcatgt
223561 aacaatgatt gcaattggtg gagctatcgg tacggggctg tttttaggat
ctggtacagc
223621 tattcaaagt gctggtccat caattatctt gtcttatttg attgttggta
ttattagctt
223681 tttcatgatg agagcgttgg gagagctaat cttaactgag cctgatcaac
attcatttat
223741 tgaatcagtg aaaaagtatc taggaaatag aatggaattt gttgcggggt
ggacttattg
223801 gttatgctgg cttagtttag caatggctga tttaactgca actggaattt
atttgaaata
223861 ttggtttaaa gatttacctc aatgggtagg gccgttagtc attgttgtgc
tgctaatgct
223921 tgttaacatg gttaatgttg gactatttgg cgagcttgaa agttggtttt
caatgattaa
223981 ggttctagct atcttagcct tgatcattac tggagcgatt ctactcgtta
tgcataccaa
224041 aattgactct agaacagttt ctcttactaa tctggtaaat tatgggggct
tttttccaac
224101 aggctcatgg ggctttttaa agtcatttca gatggtagtt tttgcctttg
tcggaataga
224161 aattgtcggt ttaacagcag gtgaaacggc caatccagac aaggatattc
ccaaggcaat
224221 taatactttg ccagtcagaa ttggattatt ctatgttggt tccatgattg
ctattatggc
224281 tgtttaccct tggaacaaga ttgttactac aactagtcca tttgtacagg
tttttggcgc
224341 aattgggatt cctgcagccg ctggaatttt aaactttgtt gttttaacgg
cagcaatgtc
224401 cgctactaat agtgctatct tttcaactag tagatcacta tatgcacttt
cacgtggaaa
224461 taatgcccct aaaagatttg gaaagttaag tagaaaagcg gttcctaata
atgccttgac
224521 cttttcatcg ttaattttat ttatcgttgt aattctgaat tatattatgc
cagcacaagt
224581 tttcgatatt atttctagtg tatcaacaat caattttatt atggtatgga
taattattat
224641 gtggacgcat ttgaaatatc gcgcagaaaa taaaggaaaa ctaggtagct
ttagaatgcc

224701 gggatttcca ataactagct gggttacaat cattttctac ttagtaattt taataatttt
224761 ggcgctaatc ccagcaacga gactaccatt acttatctca attattttta ttttagcttt
224821 atcattgagc tacacttttt tagaaagaaa aaattaaata tcacgtaaag actgacaaat
224881 aaaattgtcg gtcttttata tagcattaaa tttgaaaaaa agcaaaatcc tatctcgtta
224941 ttagtaatag ggcttggtat aatgattgta attaatgctg tgaaaacgct atactgtata
225001 ggaatagaat aataataacg aggcaaagga tatggcaaga caaaaaaatt tagcaagacg
225061 acatgaaata ctgcgcaata cttttatgct cctgaaaaaa agaggcatgg aaaatgtatc
225121 actacaaatg attgcagata aatctggcat ttctaaatca ctactccagt catattaccc
225181 acataagaat ctattaatta ccgaaattgt tactaatttt atgacagcag tcttaaagag
225241 tttaaatgcg actgatttca aaaatttgaa tacatattct aagatgaaaa tctttattta
225301 tttgatttta gaacaaggta tccaagatga aggcgttgaa cgcgttgtca aaagtatttt
225361 aagtgatggt gattcattgg atcgctggag ccagattctt gatgattggc ttaagcatga
225421 aggcgttaag catgatttgg gaaatgatcg tcaagttcag attggtttaa actttattgt
225481 tacagctgga ggaggtctat acgtaaaacg gactgaattt gatttagatg ctgatcaaat
225541 ttctgatata atggtaaaaa cttttatgtc aacattttta tcaatagatg agtcaaagat
225601 aagtcaaaca cttgcagagg ctaaatctgc cttacaaaaa tacgaattaa ctactttaac
225661 tcaggctatt aataaaatgt tcgacaatta attttaagga aggtcgagga aatggcagta
225721 cgcacacgca gtgagctgcg tagaattgat caggctcaaa ctgtgcatga taaaagatct
225781 tttggaattg caatctttac aattgtggtt ttgtttttca gcttagtaac ttttatgaac
225841 ccgattttta tgaggagtca gattgccaaa gaaagcaatg gagttgtagc tgaacgctat
225901 atcaatcaga agtttgataa tttcgctgca gcaattggtg ctgatcgaag tagtaacaac
225961 agtactaata atctattaac tgttgagcaa acaagaccga ttgcgaatgc aatgattgac
226021 tatactttgg gaattcattt atttagagca gaaaattcta gcttagctag tcaaattcgt
226081 aaggttatct tgactaaaat tgatgacaat tcttccatgg aagctaagtc tgttcaggaa
226141 aaattgaaga agaataagga aagtgggctt tacgcaatta ttacaagctt tggcttggct
226201 aatgccactt tagcagcaaa tgttgagctt gtatttttga ttttaaatat cttggttatc
226261 atttttgtgg gcatcttagc ttatcaacaa attaaaagac tcaaagaaat tgtttcaact

226321 gggcgcttaa ttaatatgtt tgccgctgga gggatgagag catcactagg attgatcgtg
226381 atttttggac tattggcgtt cattccgaca attttcaatg ttgaaggttt gatcttgaat
226441 attggctatt ttttggagat agcgagtggc attttcttag agttagtaat tgtcggagta
226501 gttttgtttg taattagcac tgttacttgg cagcttacga gtgcaaaata ataagaagtt
226561 aacgaatata caaaaaaggt ctcaggtttt tacctgaggc ctttttctgg gaaatggcgc
226621 cggattggat gatcttaatt gtattcaagt aagtgtaaag tagttagggt ataaaataat
226681 tgtaatatgc aacagccata acggctcttg taagggtcct caaccatttc tcactactta
226741 tttttacagt aattagggtt taaatcaagt tgaaaggcct aattattctt aaataatata
226801 atatgtttag cttgtgctga aagaatattc taaaattata aattcgttac tttaaagcct
226861 ttttcaagat gttagtttat taattattct gcctgatcca ctggtttttg ccgagataat
226921 accaagttat atgatcggta gtaattgatt gaccataagc gtaacttttt ccagaagtaa
226981 catgattggc agtgcgtttt cgtagatttg gataaggact agtataaata tttgtatcaa
227041 atgactggtt attgatattg actacaccag cagtaaaatc tggattgttg taaacataaa
227101 aattatgaaa ccactgatca ttgcctaagt tgtagtaaga ataaccatct tgataagaac
227161 ggccaaaaat cactgtattc atctggcgct ttttattcaa agaatcacgt agtttttctg
227221 cttgaagtaa aagagctgat ttaattgttg tagatgaatc aggatcagct acttggttta
227281 aaattttgcg agttgtcttg tcgctactat ctttaatatt ttgcgaggag gatatttttt
227341 cagagctctt ttctccgttc ttagtcttag tgttatcgtt tgaagttttt agtttaggct
227401 gattagcagc aacttgatta aataatttaa aactatattg gtagacatta tcaatattta
227461 cgttatattc atgccctgaa actaacttaa tatctcctgg gaagtaagtg ataattgttt
227521 tgaagtagcc atatgcctta ttactgaaat tattaacatt tgtggcttgg ctaacttcgc
227581 ctgtatctaa atcggtgacg gtgattttag gaataccgga ggtagtttta tcagataaat
227641 aaagagacca agcaatgtta tttccttgaa gtaattctat tgggaaaacg ccactattag
227701 gataggcaac ggcactttta gcagcagcct tatagccatc gcttgaatat gcaacttgct
227761 ggactgaata gcggtagcta ttttcaccgt atgctgcacc aataccagtg gtagtaagtc
227821 tacttgataa aagccacgca cgatgtccag tatccgatcc atcaagatta taggtatcag
227881 ttaataaatc tgtgattaca tcaccagcag attgattgct tacattaaaa ttcaaattag

227941 agctagcaga aacatttttg gcaattgtcc agtaagtatc gctaatatag ttcggtcgtg
228001 tttcactagg taacccgtgt tgattagtaa atgggttagc cttaattgca gccataacac
228061 ttgcggtaat ttgcgcgttg tcattatctg tcttatttgt agaaatactt ggcagatcaa
228121 acaaaccacg ataaaaatta atatagtcta attgagaatt aatataagaa ttagtaacag
228181 aaccaggtga aaatggtgct gttaagtgag ggctacttgc ataaagatta ccagaattaa
228241 aagtttgttt gggtaatctt gaatattgat tttgaattcg atgaacttct tggatttcat
228301 ctttggtaaa tgttgcggcc agcgcttcgc taatcgtaac ggtgctagtt gtagcactga
228361 gtgtaatagc aactaaaaaa gcggtggctt tttttgtaaa attcatctta tctcctttgc
228421 gggatttatt attagagtaa tagtaatata tataacttaa ttataaaaaa ttttcattct
228481 tttgagactt tttttcaaaa ataatgcatt atagcgtcac gattgtttat aataaggttt
228541 cgaggacaaa aaggagataa aaatatgact gacaaaatta aggtaggttt gatcttcggt
228601 ggaaattctt cagaatatga agtttctatc atgtctgccc acaacattta tgaggaaatt
228661 gatactaata aatttgatgt ttatccaatg tggattacga atgatgggta tttggctgac
228721 gatgccgatt ctcgcagagt cttagacaat cctaaaatgg aagtagctaa ccctcataaa
228781 gtagcaaata tttctaatat tgttgaatta aaagatcgtc ctgaaattga cgtatttttc
228841 ccaattgtac acggaaactt gggtgaggat ggttgtttac aaggtttatt tagagttcta
228901 gataagccat ttgttgggga tgatgtctta gctgcagctg taacgatgga caaggaaatg
228961 accaaaattt tagctcaacg tgctggtgtt cctgtcgcaa agtggattgc agttaagcgt
229021 tttgaatata acgatccaga taatgaaaag ttagactatg aatacgtagc tagtcaatta
229081 ggctcagatt tattcgttaa gccatcaaat caaggatctt cagttggtgt aagtcacgtt
229141 actaacgaaa aagaatacaa ggtcgcttta gctgaagctt ttaaatatga tgataaggtt
229201 ttagttgaag aaactgttca cggtactgaa gtagaaactg ctgttttagg taatgataag
229261 ccaatcgttg ctggtgttgg tcaaattatt aacgccaagg attcattcta tacttatgaa
229321 aataagtatg atgatgattc aacttcaact ttagagattc cggctaaatt gcctgaaggg
229381 attgttgaaa aggttagaaa aaatgcttta aaggtatttc aagcaactga atgtagtggc
229441 ttagcacgta ttgactcaat gcttcgtacg gaagacaagg aagttgtttt aacagaagta
229501 aatgctcttc caggattcac taatattagt atgtatccta aactatttga agaggttgga

229561 attccatata ctgatttgat tactaaatta attgactacg caatggaacg
ctacgatcac
229621 aagaaaactt tgttacataa acatgattaa acttagttta agtaaaggga
gaatttagga
229681 tggcaagcga ccataatttt gaagaacttg aggccccagc aaaaacggcg
gaagaacttc
229741 atattagcgt agcaacatta agaaagtatt ccttaattgt tgaaaaagtt
actggaaata
229801 aagactacta tgagaggact aagcagaagt ctcgcttata ttcagctaaa
gatattgaag
229861 acctaaaagc ttttaaagct ctgtctaaaa aagctgatct gactttacag
gaagcagcaa
229921 gacaagtttt tgcagtgagt gataaaaaag ctgaagaagc caaaaaggct
gcaaaaaatgc
229981 aaaaagaagt agcaactaag actgctactg tagacgctga tcaagtagtt
aagcttttga
230041 ctatgctcca aaatacgatt gcaagtcaaa atcaagcaat tcaggatttg
caaaagcaag
230101 tagcgcgcgt tgaagctcaa aataagaagt taattgatag ctcacaccaa
ttagctgagc
230161 cagaatcaac agattcacaa aaagaagttc aagctaataa agatcaggaa
gctgccgata
230221 agattactga agaacaaaag agtgagcaag aggctcatga agaaatctta
agaaaagcta
230281 gagagaacga agaaaagcgt cgtaagcaaa aagtagaaga gaatattcat
cgtactttag
230341 ctcagatgca aattcatccc aagaaaaaac accattggtg ggatcgcttt
tttaattaaa
230401 aaataggatg cctgtttgta ggcatcctat ttttattcgt cttcaggttc
aataattaat
230461 gactctgcta ataaaacgtc gttatataga tttaaaatat aggttcgata
tttgccttcc
230521 ttttgtccaa tcatttctag atattcgcgt tcaatcgcaa aagccctaaa
aaaagcaaga
230581 cgctgtagat actgttctga tttgcttagg ttaggctggt caattgtctt
tagcagaact
230641 cgcatatact cacgcgtttt aacaactcgt ttttgattaa gtagggtata
gataacatgg
230701 cgttttactc tttgcggtaa atacatttta tgaacggcaa ctaaagcgcg
tttaaccatt
230761 tcacttcgta ttttatccat tacgatgtga gcctcttcgt cactgacatt
atgcggaaga
230821 ataaattgaa agacaattgt tggaacaagc atacttaaaa taattaaaac
agcctcagac
230881 atgatgacta ggttataacg ttgacttcct aggaagtcaa cgcttatcgt
taatgctaat
230941 gctaaagtaa ctgcaccatg aattcctcca agtgaaaaga ctatgctttc
tcttcgatca
231001 taacgaataa ttaagcgacc atataggtaa cgaactagca agttggcaag
gtagactata
231061 atgccaacaa taatccattt agctagatct ccattaaaga gcttattacg
tgaaattctt
231121 accatcatca atcctaaaat aatgaaaacc atactattaa agatatcaga
aataagattt

231181 tgtaagtctt ggctcatatg aacctgtctt gaattaagca ataagctttg
ttgagattct
231241 gcattatgta agagaccagc aaccaccacc gcgataattc cagagacatg
gagatgttct
231301 gctagagcat acaaggctaa tggggtaaga atataaataa gattttgagc
atttaaggaa
231361 ttaaatctgg atctgacaag agtctgacga aaaataatta ttacccatgc
tacagcgaaa
231421 cctagaattg caccgccaat cgaagaaata caaaagtcct gaattgtctg
tccataatta
231481 atatatccat ttgcaaacca aagaaggctc atgttgagca agataattcc
agatgcatca
231541 ttaaaaagtg attcagcctt taatgctgac gttacacggc gaggaattct
taggccatta
231601 gttactgctt cacttgctgt ggcatctgtt ggcgtactaa tactgccaat
aataaatgca
231661 agcggtaagc taacgtcaac aagcagatga atgctaaagc cgcttacaat
taaagctagt
231721 aaaaccatga taatagttag cccaattatc tctttaatac gtctgccaac
gttgtgaatc
231781 ctagtctttt gcccttcaaa aaataaaagt ggcgcaacga tcaactccat
aaaaatttca
231841 gaatcaaaac tagctatttg ttggtttaaa aagggaataa gaccaataat
aattccgatg
231901 atcatactta tatagttaac tgatacttta tcaattgctt gagcaataat
aatactgata
231961 gcagctgcaa ttgctagagt aaaagttgaa atcataagtt ccatagttta
gatttacctc
232021 agataaatta ctattttta ataattagta aaaactttta atgctactat
attagcatac
232081 tcaccattta ggttattagg gaaggtgaag atgtgaaaaa gaatcaaaaa
gtttggctta
232141 gtttaacgac tttaattgtt ggcatagttt ttacgttgat tacgtatttt
tctacaactt
232201 ttgctagcaa accaattgct gtaattaccg ataatcacat tcaagacacg
ctaattaata
232261 aaaccaaaga caattatgaa aataaagatg aagttaggca acaaatattg
catttaaaaa
232321 tcatctcagg aaaataccga ggtaagagat tcgttgttac caatacctac
tctccttcac
232381 aggctgtttc acaaaaatat cgtcctcatc aacgtgtgat tgtatctttt
attaaagaaa
232441 agccaaaatt agttgaacct aaacgcgatt gggtagttgt cttgagcatg
tttttaacta
232501 ttagtctgat cattttaatc accggaaaac aagctagttt attgttaatt
tcaatgattt
232561 tgaatagtat tattttttat tttgtgatta agaatgatat taaagaaaat
ggaaccaaaa
232621 ttttcttagt ttatggtatt gcagctattt tgtttacttt tatttccctg
ataattgtac
232681 aaggatttaa tcaaaagatg cttgttacac tagttgctac gcttctaggt
gtttttgtgt
232741 catttggaat tttttattta gttatgagat tgacacatga acgcgggatt
gactatgaag

232801 cagttgatta tgcaacgcaa gatccacgag cgctattttt atctcagacc attttaggag
232861 ttcttggggc agtaatggat gaagcaactg atattgtttc gagtctttat gcgttagcta
232921 aacataaggt tgacttaacc tttaaggagt tatttcttag tggccgtaca ttaggacaag
232981 aaattatggg accattaata aatgtattag tactgatttt tatggccgaa gcactgccaa
233041 tgacaatttt gtatctaaga gataataata ctttagttta tacttttaaa tatactcttt
233101 ctttaggtgt aattcagagc ttaagttcag caataggcat agttttaact gtgatttttg
233161 ccactcttgc tagttctgtc tttttaaaaa ataaaaaaat agaggaggca gcgaaatgac
233221 aactttaacg gctctattga taattctctt aattttaatg attattgttg gtggaaaaac
233281 gggccttaaa agttatctta gtgtggttat taatgcctgt ttgataattc ttgttgcttt
233341 gttaatttct tggggagtaa atataatctt ggtaggaata atttttattc ctttaaaatt
233401 attaacaatt atttatttag ggacacacga ttatactgta gctaagaacg cgtttttaac
233461 cgctttatgc gtgagcttga tcgtaatgct gataattatc ctgcttgaaa gcttagctca
233521 aactcagggc tttggagacc aagcaggaga agaattaatt ggtttatcgt tgaatgtagg
233581 aattagtttc gctcaaatag ctattttagt tgcaattttt tcaatgctag gggcaattgc
233641 agaagcaagt gtagcgatga gtgcaggctt acttgaatta aaaaggcacg atccgaatat
233701 tacgcagaag caattgatca agagcggaaa tgaagttggt tctgatgtgt taggaacagc
233761 catgaacaca attttatttg gtttatttgg tagctttttg ccaatattta tttggtatat
233821 gaggttaaat tattcattat ttgagatttt aaatgataaa ctttttgtag atgaattctt
233881 aattattgtg tattcattta tcggcgtact tttaacagtc ccattgacaa caatattttt
233941 agcacatacg ttgacgcata atgaacttaa gaaataaata gcatagaggc ttagcaagat
234001 gaaagtagag tatttaacac aaaaaaattt taataatcgc gaatttaaaa tcacgtatta
234061 tggattacaa gataatccgc agcttttgca aaaaaagaga ccattgatac tcgttttccc
234121 tggaggaagc tttgaccatt tatctttaag agaaggagaa ccagtagcat tggcatatgc
234181 agctcatggt tttaatgctg ccgttgtttc atataatcta actaccgatc caggtaaaat
234241 atatcctgat gcagccttat caggattaaa agctgtttct tattttagag aaaatagtga
234301 aaagttagga attgatcaag agcgaattac tacaattgga ttttctgctg gcggtcatgt
234361 tgttagttca atgaacgtga tggcagaaag tgaaaaatgt caggcaaaat atgattttaa

234421 ccatgatcaa gtagcgccta atgccacaat cttgggctat cctttaatta atattaaaga
234481 tattggtttt ccgttgccag ctgatgctaa tcaaaaaatg cctactgcta aagagttatt
234541 agatacagct ttaggagtga cgcctgatac gcccccgact tttattttcc aagctgttga
234601 tgatccagtt gtcttgattg ataatacaat cgaatatatt acagctttaa gaaagaaaaa
234661 agttcatttt gaagcacatt tgtttaataa aggcggccat ggcttttcac ttgctactcc
234721 tgaagtggct gtgggtggac gtgagcccga ttcgcatctt gcacattggt ttaaactaag
234781 ccttgaatgg ttaaaagata gaaaaattta tatttagttt cacgtgaaac aagacctgat
234841 tttatataaa aaagtccgaa aagaaaatga agttaatcat tttgcttttc ggactttttt
234901 gttagtttag atgtttttat tctttagcca taaagaataa aacttcttta ccagaagcta
234961 gattatcaga ggtagctaaa attctaagtt taattgcttt tgctactacg tctcttaaac
235021 cgattacttt gtttttagca tcatttggcc aattaatttc tttactgaaa tcgctgaagt
235081 tttcaccgtc ttttgaaatg gcaacttgaa ctcgtaagac atgacctatc cggtcgcgtg
235141 agcgaggtac gtaaaccatc cggctcagct tataaatgtg attgaaatta aaagtcaagt
235201 ctagtggact atctttacta atttggtctt ttgagagcca ttcactagct agcttaagat
235261 cagttaagtt tttgacggga gaatcgtttt cactttctgc tgtgctagta acttttacat
235321 tagaaattgc ataatccatt tgatcgcgct tggtatgagt tccaaagtat tcagaccaat
235381 ctgatacctt gttaccgaat ttatttctaa ttctaaaccg atagcgtgtg ttaggctcaa
235441 gttcgtggaa agtgaaagta tctccatcaa tattagtatg aacaattcca ttaacttcga
235501 tatcagccat tatattttta ccgacgaagt tagttggatt agtccaagta atggtaatgc
235561 tctttgaagt taaacggtcc gtcaaaattg cggcacttct tggtgaagcc atagctgaat
235621 cgataatagc atgttgttcg acttcactac cataactata gttttcaatc gtaagttcaa
235681 atttattgtc atggatgtca acagctggca tttttacctt aagagcaggc tgcaattgac
235741 ctgtaaattg accaaattct ttgcatggca cgtaatcttg ctggtagaaa ataccttctt
235801 ctgatttgtt aaaactatct aaatcattat attcaggtag tggaataact tgatcattta
235861 atttaagtga aactttacca ggatagcgat cggccatgat tgttaagata gtagcttgtt
235921 tttcttccag tccctcatag cttccctcgg ttggataaac cgtaatatga agtttagatc
235981 cattaaccga actggtaatt tgcgttgtag ctgcatttcc cttttcataa tcattagttt

236041 gattatcatc ttcatataag atggcagaac tcttattttg tgggtaaaca agggcacttc
236101 taagtgattc ctgtaagatt gcccctccgc gaacgtaaac aggaagatgc cacaatggat
236161 agtgtaactt atcgtaaatt ctgccaccga ttaatttttt accagtaaag agatcgatcc
236221 acatagttcg atgatcaggc aggtaaagat tgtcttttaa tgaagctcca cttggatctt
236281 ctcgaccatt agtaattggg gctattaata agttgtttcc tagcataaac tcatgtttta
236341 cctgatttgt gtagttaact ttttcgtgcg ggaaggacaa aaacagaggt ctaacaatcg
236401 gcatgccatc ttgagcagct cgggtaagag tatagagata aggcgtaatt tcttccctaa
236461 gttttaggta agcgcgagta attcgcgtca tttttgcatt aaatgcaaac ggtgtttttt
236521 ggatattgcc gtaattatca attgagtaaa gcagaggcgt gaagactttc cactgtaggt
236581 cacggatgtt tacttgagca ttaccgccgc cttcttttcc atcaatagca cttccgatat
236641 ttggttgacc agaaagagac atacttaaga aactagcaat ctctgttgca atttgttccc
236701 actcgcctcc tacaccttca tttgcggtgg tagctaaggt ttgaacagcg ctccagccat
236761 tacttgccat tgcccaagga cgattagttt ggtttttatc aactaaaggc tcactagcaa
236821 ttgcaaaatc ttgcataatt ttgtcattag ctaaattagg agcttctgtc ttaaatttta
236881 agaaattagc tggtgcgttt gcaggtaaag ctccgtagaa accaagtttt atattttgct
236941 ccttggcaaa atcaactaag ttagctaatt cggtttcatt taaagtttgc ttactgttgt
237001 agttaggaac aaaccagcta agtggaaaat gatgtgagcg gtaacgttca atcattgcac
237061 gagcagaaaa ttgatactgc tcttcaccat taagggagga cttagcaaca atttcattat
237121 cagcatgggc aattttataa gtattaccat cttcaaattt aatggcatta ttttcttgag
237181 gcttcgcaac actccagcaa gtatcaagat agttacctaa gtatcctagg ccaagagcat
237241 atttaggcaa gaagagggga gagccggtaa ttttgtagta ctgacgtaag atttcagctg
237301 gagaatctcc taaaagataa aaattatcaa aaataggagt ttgatgggta agaatagcag
237361 tattgccatt ttcaccttta aagtcataaa ttccgttttt ccaagtattg cgcaattctg
237421 caaagccagc attggaccag aaaaagcctt ctggacaagc aactgcacca ggaccggtta
237481 aattcgtatt tttgatttca attatttttc ctttatgact aaagcttcca ttttgcattc
237541 cgccgccata atagaattca tttttatttt gacgtaagaa ttcactagaa taatctgctc
237601 ctagttcaat cggactactt tgcatcatac gatagcgatg aagttgatca tcaaaaatgc

237661 taaagattgc aggatttttt tgtaaacgaa gtgaaaattg accactttta atcgtgaaag
237721 tttcatcagt gactaaaagt tgtgattctt caaaaggacg caaactaaag ttacctgcgg
237781 taatcgttaa agacggcgac aaaggtttaa attctgctgc cggatcgata attaagcgaa
237841 agattccttt agcaataatg tatagacgtg ctctttttcc atctgcaaat tgcagatcgt
237901 agtagcgagt acctttcgct acttgcacta aacgacttaa ttgttgtggt tgttggttgt
237961 tagtaatttc agacatatta catttcttct ttaaattaaa tttttcattt acgtatagtt
238021 catattataa cgaaaaaatg catttcgtgt ttttttagat ttgacataaa gcgctttcta
238081 gaatattctg tagaaggtgg tactaaccag ttgaagattg gatggggtta gaagatgaaa
238141 aagccacttt atcagcaagt aattttagat cttgaaaaga aaattaagac tgaaatgaag
238201 ccaaataatc gactgcctag tgaaaggcaa cttttaaaag aatatggtgt gagtcgaaat
238261 acgattcgat tagcattaaa taatttagaa gagcgaggca ttatttatcg attgcatgga
238321 aaaggtacat ttgtttccac aatttatcta gatcagacca acttaggaag tatgtattca
238381 tttagtgaac agatgtcaga agcaggacac aagccaacaa cacaaaaccg cacattagag
238441 cttgttgttc ccgacgcagc tgtcgcaaat cagcttaatt tgaaagctca agaaaaagcg
238501 tacaagttag ttaggttacg tctagctgat ggagtaccac taatgtatgg agaaacatat
238561 ttaccagaaa gtatttttcc ggatctaaaa atgtcggatt taaatgaaaa tcatttgtac
238621 actgttatga agcaaaaata caatgaacaa agtgttcttg cctttgaaga tgtacaagcg
238681 gttaatttaa atgaaagaga cagtaagatt ttaggcgtaa agcccgggga tgctagttta
238741 aaaattttta gacgggcaat caatgataaa aacgtgccga ttgaatttac catttctcta
238801 gctagagggg atagattcat ttatcgatca cggcaataca atcatttagt ttagttatcg
238861 aaaggaatag gccaatgttt acaaaaagtg atgcagaact aaaaaagatg ggcgcagata
238921 tcactactag agaaattcag caacaacctt ctttatggaa agagactttt aagatttatc
238981 aagaaaagaa acaagaaatt gataaatttt tagctaaaat caatcaaaaa tttaataaag
239041 taaaagtcat ttttacaggt gcaggaacaa gtgcttatgt tggcaacact gtgatgccat
239101 atttgagaaa gcatgccgat cgtactaagt atgactttga agcaattgat actactaaga
239161 ttgtttcaac accagaagat tatcttgaag cagaaacacc aacaattcta gtttcttttg
239221 ctcggagtgg taattctcct gaatcagttg caactgttga acttgctaag caattagtta

239281 cgaatttata ccaaattgct attacatgtg ctccagaagg acacttggct caagacttaa
239341 aagatgatcc aaatggactt gttcttttaa tgcctgctaa gtcattagat caaggttttg
239401 cgatgacagg ttcattttca tgtatgagct tagctacttt attggtattt gataccagaa
239461 gtgatgcaga aaaagaaaca attgttaatg aaattgctga gatgggcgaa agcgtagttg
239521 atcgtgaagc tgaaattcaa aaattagttg atactgactt tgacagaatt acttatatcg
239581 gtagtggtgc tttaggcggc ttagctgaag aagctcgact taaaattttg gaattaacag
239641 ctggaaaagt tgcggcttta tttgatactt caatgggatt aagacatggt cctaagtcat
239701 ttttagataa gaagacgatt gtttttgact ttgtttcaaa taattcttac accagaaagt
239761 acgatttaga tattttagat gagattaaag ctgatgaaat agttccattg gtaatggggg
239821 taggacaact taagaaggga caagactttg atggtaaatt cttcgccttt tctggaaaag
239881 agttaccaga tgcatattta gcattgccgg atattatgtt tggtcaaaca attgccttgc
239941 ttacttcggt taaggttgga aatactccag atactccttc accaaccggt acagttaacc
240001 gtgttgttaa gggagtaaca attcacaaat tcatcaaata aaattcaaat ttagaaaaca
240061 ctttcaattt agaatagaag ttaatttaag gagttaaata ctatgtcata ttatattcat
240121 gccggaaagt tctttttaga aaataaaact gaaaatggtg gttatcttga agttcaagat
240181 gatgggaact ttggtttata ttatcgagaa gatgaaaagc ccagtcatag tctaattaag
240241 gaatatggcg atcaatggat tgcacctggt tatgttgaca cgcatattca tggcttgctt
240301 gatgaagata cgatgaagag cgactgggaa ggcgttaata agatttctga aggtttgctt
240361 caagctggtg taactagttg gctaccaact acaattactg caagcgataa agaattaact
240421 gaaacttgtg aaaagtttgc ggcacataag ggtgaagaaa ctggtgctaa aattcaagga
240481 ttacactttg aaggtccatt ctttactcct aagcatggtg gggcagaaaa tcctaagtat
240541 atgactgatc cttcgattat gcttttaaag aagtggcgta aagcttcaga taacatgttg
240601 attaagatct caatggcacc tgaaagaaag aatgcccgtc aatttgtacg tgaagctgta
240661 aagatgggct tagtagttgc tttgggacat tcagattctg attttgaaga tgctattgct
240721 tgtgttgaag ctggagctag cgtctttaca catactttca atgggatgaa tggattgtca
240781 caacactcac ctaacattat tggtgctgca ttttcttctc gtttaacaac ggatgaatta
240841 atttgtgacg gtcaccacgt tgaagagcca gctgttcgcg ccttagtaaa tgcacgtggg

240901 ccagagcata ttgccttgat tactgattgt atgcaggctg gattaatgcc
tgatggtgat
240961 tatgttttag gtgaattgcc agtttatgtt aaggacggta tggctcgttt
gaaagatact
241021 aataacttag ccggcagtat tttgctctta aaggatgcag ttaagaatgt
agtggactgg
241081 aatgttgcaa cacctgaaga tgcagttatg atggctagtt atgtaccagc
taagagttgt
241141 aacttactcg ataagtgtgg tgtgattaaa cctgatcatc cagctgactt
tgttgttttg
241201 aatcatgata tgactgtcag tgaaacttat ctaaatggtg agtcacgtta
caaggcttaa
241261 aagagtatac ttttagtagg aactttagaa attcttaaaa aagtggtcac
aaccagttga
241321 ctagtaaaag ttaatatact aagattgaaa ctgtagaaag cgtttacaga
aatactttac
241381 ggaatatgga ggaatctata tgaatattgt gggagctaga attgatggac
ggctggtcca
241441 tggtcaagta gctaatcttt ggaccccgaa gcttcaggcg gatcgcatta
tggttgttga
241501 tgaagatgca gcaaaaagcg atatccaaaa aagtggtttg agaatggcta
cgcctcttac
241561 gactcgtctt agcgttttgc cagcacaggt tgcagcagat catttgattc
atgagcgtta
241621 tggtaatcag cgaatattta ttgtcgctaa aaagccagca gccttcttag
atttacttaa
241681 cttaggtctt aagcttgata ctttaaatgt aggtacaatg tctcaaacag
atactactaa
241741 gcaagtaact aagcagatta atgttgaaga aaaagatgtc gaagatttta
aaaagatagc
241801 agataagggc gtgaaaatca cagctcagtt aactccaagt gatgattctc
atgactttat
241861 gaaattaatg aatgaaaaga ttaaatagga ggtctaacta tggcttggtg
gcaaatatta
241921 ctacttaccc tctatgctgg ttatgaaatt cttgatgaat tacaaatcta
ttcatcgttg
241981 aatacaccag ttggtgccgg tttaattgct ggtttaatta tgggtgactt
aaagactggt
242041 ttaattattg gtggtgccat gcagcttaca gttttaggtg ttggtacttt
cggtggtgct
242101 tctaagattg atgcaactac cggtacagtt ttagcaacag cattctctgt
aagtattaaa
242161 ggaatgagcc cacaagtagc tatttcttca attgcggttc cagtagctgc
aattatggtt
242221 caattggatg ttttagcaag atttgctaac acttactttg cacacagaat
tgaccactta
242281 attgaagaga acaactacaa gggtattgaa agaaacttcc tctatggtgc
aattccttgg
242341 gctttatctc gtgcaatccc tgtatttatt gctttagcat ttggacgtgg
tttagttcaa
242401 actattgcta actcattaaa cggaaacttg aagtggttag gtactggttt
aacagtagct
242461 ggtgcggtac ttcctgcagt tggttttgct attttgcttc gctacttacc
agttaagaaa

242521 cacattgctt acctaattat gggattcaca tttactactt tattctcagt attattcaca
242581 agtattcaaa ctttaggtac aggtcttcaa gcagcaaaca agtcatttac tgcaactttc
242641 aacggcttgc caatgcttgc aatggcttta attggattag catttgcaat cttgcactac
242701 aaggatgaaa ttagtggtaa aggtggcagc ggtaaaggtg gaaccaaaga agccgcagct
242761 actacagaat caactaatgt agcttcagag ggggaaatca ccgatgatga actctaaacc
242821 aaaatataaa ttaactgaca aagactttaa tcaaattaac agacgttctt tatttggttt
242881 ccaactaggt tggaactatg aaagaatgca aaacactgga tatttattct tgatcttgcc
242941 acaacttcgt aagatttatg gtgataatac tccagaatta caagaaatga tgaagacaca
243001 cgtacagttc tttaacactt ccaacttctt taacacaatt atcactggta ttgatttagc
243061 cattgaggaa aatgaaggtg ttgaaggtaa ggatactgtt actggtttga aggttggttt
243121 aatgggacca tttgccgcaa ttggtgactc aatctttgct gctttgattc caaccatctt
243181 cggtgccttg gctgcttcta tggcttcaca aggaaaccca gtaggtgtat ttatttggat
243241 tgccgctcaa attgcgatct gtttcttcag atggaagcaa ctacgttttg cttatgataa
243301 gggtgtaact cttgttactg aaatgagaga ccgcttgaat gctttaactg atgctgctac
243361 agtacttggt gtgttcatgg taggtgcttt agttgctacg atggttaatg ttaagtttgc
243421 ttgggtacca caaatcggta aagttacgat ggatatccaa aacaacttag atatgatcat
243481 tcctaagtta ttacctgcct tgatcgtcgg ctttgtttac tggttacttg gaaagaagaa
243541 gatgacttca actaaggcta ttttaattgt tttagttcta tcagttgttc ttggcggtat
243601 tggtgttctt gctaagattt agcagtcaat taaaggaaaa atattatggc aaaagaatta
243661 gtattgatca gtcacggtaa gatggctgaa gaagttaaaa agagtgcaga attaattatg
243721 ggtccccaag agcatattca tgctgtatgc ttgcttcctg aagaaggacc tgaagatttt
243781 gaaaagaagt ttcaagatac tattaatggc attcctgaag aagatttaac agtatttgct
243841 gatttaatgg gaggtactcc tgctaataca gttagtcgtt tgattatggg tggtcaaaat
243901 attcatttag ttgcagggat gaacctagca atggtgattg attggctcaa tagtcaaatg
243961 attggcaatg attcagatgc tgttaatgct ggtaaagctg gaattgtgga tatcaatcaa
244021 atgcttgcta gcatgaagaa atagattatt tagatagatg ttgaaaacga ctcacggtgg
244081 taaaaccaca cgtgggtcgt tttttataat aaaaaaattt atagatttat gcgtaataaa

244141 catatgggat aacccctcaa atatttttag agaggattct atgagtcgta ccatacttat
244201 tctgatcaag atagatcaaa acaattgcag gatggcgtga caaagattat tattattaat
244261 tcacaagttt cagctgaggg gcagagtgaa gacttaaagg ctctagctaa actgatgaat
244321 aatgaacctg tgaatttaaa taaatatttt gattacgcgc aaagaagaat taaggaaatc
244381 aacgaagatc cagaaatgag ggaaaagacg gtgttgaaca aggaaaagta gattccgcca
244441 aagttattct tgagaatcaa atggataatg gtagcacctt agaacaagct acagaatttg
244501 ttagaaattt gaaattgatt tctgatgaag aattgaacaa gctaattgcc ttgtataaat
244561 agtttgtaat tacacaaagg ctataaaaag ctgatagatt tcatttctat cagctttttt
244621 tgtcgagtaa tttgttacaa tataaataat ttgatgatag cgctataatt taccctttta
244681 gaaaggtcaa gatatgaata aattttgtcc ccattgtggg aaaccaatta agcctacgga
244741 tcgattctgt tcacattgtg gaaagacagt tatatcttcc aaatctggaa aacaatcgat
244801 gcctaaaaga agtgaaattc acagcaataa attaaaatca caaaaacgaa atatcattat
244861 tggagcaatt atcgctgtat ttgtccttat tcttggtggt tggggattat atcaacatgg
244921 tcgaaattca gcggaaagca cagtaattgg tggcaacgga aggctaacta atgctgagat
244981 taaaaagatc ccgcgtaagc aattagcagc ttgggcaatt ctttatgctg ataaaaaata
245041 tggcaaaaaa tggggcgaag cagccgatga tctaagaagt ggtcacttga caatcaaaag
245101 ttattcaaaa tatcgttttg gagattatga aatcagtgct actgacggaa gtactttata
245161 tgttataaat gatcaggttg gctatttggt ttctaaagat aacaaagaga taacttatgt
245221 tggaaataag tctagctata gtaacagaag tgttttaagt cagatttatc cagaaattaa
245281 atctgaaaat acggagaaaa atgtaaatat ttggaatgat aatctatcga ttgttgctgg
245341 taagtcggat gaaaatgtta acgatcagag ttctaaaact agcacaaaat ctaagaagac
245401 tacgtctgaa aataaattat ggtcaagcaa tcaaaatgat gaactaatta gttacatgga
245461 tgaatttggg gagaaaatgc atcaatcgta tgagcattat attgatggca aagatgatct
245521 agtaacgtta gctggagaac attttccaag tgatattaat gaaaatactt atccatttaa
245581 attgagttct ggagatcatg atagtaggga tcctaaagat atggacagca ttaaattgaa
245641 atggaatccg caagtaacta gtaaagataa tgacagtgat acatataatg tggttgctat
245701 ttttaatcat aatggtaaaa gtgcagaaga tcatataacg tatctgttct gtttttacca

245761 tggggaacct gttgccttgg ttgatcagac gacaaatggt ggttatgtag tagttcaccc
245821 aacggctaac caagacttaa cttctcactt tgaagaaatt gcgaataatg attaaaaaac
245881 tacttggaga taaaaatgaa aaatagagta tttatttttt tactagtagg atctgctgtt
245941 ttagctatgg ctggatgtca aagccgttat aagcaagaaa aaggcagtaa tgaacaagct
246001 aaaattatta aacaaagtat aacaaataaa aatgtaagta tcgtaggaag ttatcgtgat
246061 gatactgatg gagcagcaat tgcttttaat tctgatcata ctggaagata tgtatatgct
246121 gatcctaaga attcagatac tgatgatcgg ttaacctgga agaaaactgg taaaaatact
246181 tatataatta gacttaatga caatgatgtg aatagtgcct taactgctaa gcttactggt
246241 agcactttgg ttatatcagg ggatgacgac tggaatacag aaacgtttaa taaaacaaag
246301 aataaactta atttggatga ctttctttca agcaagaaag tagaaagttc aacatcaagt
246361 aaggctaaaa ataatgtaaa aaattctaag tctacaaata gtgttccagg tgatgaagga
246421 ctatttgata tgccaaatga attacgtggt acatggtatt tagcaaatga aagcgacgga
246481 agcgtaagta aattgataat tgataaaaat accattactg gtgagggatt tatagaaaaa
246541 ttgcataaaa tgaataagaa ctttgatttt accagttatg ctcaaaatca tccgtcttat
246601 caagatatga cgaaggattg ggcaagagta gttgttttat ctgaaaatgg cgaaagtaaa
246661 gttaacgttc gtggttggtt gcaaagtgct ggagctggag aatattataa tttagctaca
246721 gaacatggac aaaaagtttt agtaataggt ggcggtgctg aggcttgggt agatggcatt
246781 ggctggagaa ataaaaccga tgccgaaaaa tatggaaaag aaaaatttag tgatcttcat
246841 tatcgagaag acattagttt agaaaataat gatgattctg atagcgctgc cacaaatgac
246901 gaagattaac caatgaataa ttggagaagt tctttggtta ataatttatg tacaaaaata
246961 agctgataga gtaaaagaac tctaccagct tattttatct aattaatata cagcaccatc
247021 gtagaagttg tatcttggac ggttgaaatc aaatgtagct aattcgctca tcttgattac
247081 gttgtctgca acaccccagc tcatcaagtt gattgattcg ccgtataatt catcaggaat
247141 tacaagtaaa acgtatttac cttgagacat tgcataacct aattccatac ctaaaccaac
247201 atcttcttct ttaggaacgt aaacgccaag catgatgtcg ctagttttaa ttccgactaa
247261 atctccattg taggtagctt gagcccattc tttgtcgtgt aaatattcag ggtgttcatc
247321 aacacgaatg tctttgtatt gattttgaag tggaacatag ctgttttcta gatcgatagt

247381 tgggttagca tttaaagcac tcattgcatc tttgtatgcc ttgttttgag
tttcagtgaa
247441 ccaaccagca ccaaagtaaa ctgtcttttg ttttgtcatg gaaaaacctt
tctttcttat
247501 cggtggaaca tttttacatt aactaacttt aacaaaattt gaacaaaagg
aacacccact
247561 tttgtgtttt tgaaatcggt tttatataat gaaactagaa atttttgctt
aggaaaggcg
247621 gaacctacta cgaatactaa aaaaagtctt ttcgatccag aagctgtctt
tgtaacagat
247681 ttaactgatc gaaatgaaat ctttaagacc gtttctcaaa aattatatga
cttaggctat
247741 gttaaagaag atttttttagg cgatattatt gaacgcgaag ataaatatcc
aactggaatt
247801 tctatgagac ccttatctcg agaattgcct gatgttgccg tacctcatac
tgagggtaat
247861 tatgttagca cgcaattaat tgtacctatt gctcttagaa actctgcaac
ctttaataac
247921 atggttaatc caactgaaaa gttaaaggta aaattcctgt ttttaatttt
gaacaatgac
247981 ccagatatgc attcaactat tttggctaag attatggact ttttagctgg
aacttcagtt
248041 gatgatctaa atgaattatt taattcggat tcaaatgaag aaatttatga
tttcttagaa
248101 actaattttg aaactgagaa ataaaaaaag aagctgctta gcagcttctt
tttgtctcca
248161 gattaaatta ttaaactccc ggatagtcag tacttgagaa tgaaagtgaa
aagtaatcgc
248221 aacgcttata gccaacgact aattcaacta cttctcctgt agaagataaa
atagttttct
248281 tgacttggcg tacaacggga taatctttag caattttgag ttgctggcta
atttcactat
248341 tagcttttac aattgtatcc gtttctgtaa agggctcatc gaataagtat
aattgcttat
248401 ctttttgaag cgtttcataa atactatgat agttattagg atctttagct
aaatttttat
248461 taatgaattt agctggaata aaagaacggt gcaccataaa aggtgtgtcg
tcaattaaac
248521 gcaagcgttc aatatagtaa taactctcat attttggtaa accaagtttc
tttaacattt
248581 tagcttggtt atcttcttgt agctttacaa ctttaactct gtcttcttct
tgtttatcag
248641 agaaaacttc attatcactt aaacggacta atttatcctt tcttgagcgt
gaaatgaatg
248701 tacctttacc ttgatgacga actaagtatc ctgccttaac tagatcttta
acagatctaa
248761 taacagtaat agatgaaaca ttaaactttt cttttaattg agcttcacta
tagaatttat
248821 ctccgtattt aaatttgcca gagataattt ctttctttag ctcgttttca
atttgtaaat
248881 attttggaat atccataata tatcaatcct taagatagat tctcctaaga
tccatgatat
248941 cacggcttga gagcttcatg gtagaacgaa ggtattcatt gattccacca
tattgctggt

249001 ttagaacgtg tatggcatga tctaagtatt ctggatagac tgactgaata tccttaatag
249061 attgcaagac gcgcttgcta gagccgtcag cggcagcttg cttaagcatt ccatcaacga
249121 aatctttcgt ggtaacattt gttaaagtgt agtcatattt gatagtagta aaaggtacac
249181 ctaatgctga taaaactagc aatgcaccga atccagttct gtcttttcct gcggtacagt
249241 ggaagagcag ggcatttttc tcttgatcgt tagcaagtaa aagatcgaaa aacttacgat
249301 aggctttttg agctgtttgt ccattaatca tatcttcata tgcaaagaac atatgcttaa
249361 agccgaattc gggatccttt tccgcatttt cttctaaagc aaaaatccct ttagaagcgt
249421 tagttaagtc atcgctgaaa actggatcaa aaatatattt tgcaccttca ggtacacgat
249481 caggatgctc agtttttttct ttttcggttc taaagtcaac gtcatatttt acgccgtgtc
249541 tttgcaataa atctaagtct gatgaagaaa gatcagctaa atttccggtt ctaagcaatt
249601 tattgtattt gatagtttga ccagatacgg ttttatagcc cccaagttcg cggaaattgc
249661 gaccattttg gatgccaatt aattttgtct cataattttc gctcattaat atttttcctc
249721 actaaagtta tttaaataca gtattattac tttaacatat tttttcataa atattttaga
249781 tttattagat ctataaaaag aaaacaaatt tattaattgt tataattaat ggtaaaatta
249841 caatgtaaag atgcaaattc catttggaga tgattactta tggtaaaaga ttataacaat
249901 attttagttc ctgttgatgg atctgaaaca gcagagcgtg catttgataa ggcagtaaag
249961 attgctcttg ataataacgc tcacttggat gtattaaacg ttatcgatac tcgtcaattc
250021 atgggggaaa tgcaagatac tttgatttct ggagacacca tttatcaaat gacccaagat
250081 tctgaggagt atcttaagag cttaaaagaa tgggctaagg aacattttaa ctttactgat
250141 gtttcttatc acattcgtta cggttcgcct aagagaatca ttgcggttga ttttattaag
250201 gaccaccaca atgacttaat tattatgggt gcaactggta tgaatgcagt ggaaagaatg
250261 ctaatgggaa gcgttactgc ttacgttaac caacatgctt tatctgatgt tttaattatt
250321 aagactgata ttgataacaa aaaagtcgca aaacctaaga aaaaatattt ctaaaaagcg
250381 aacattaaaa aatcactcta ttatttacta atggagtgat tttttataa agtggatcga
250441 tccaattcaa tcaattaaaa attacgagcg ctttcattaa aataatagta tccaagtacg
250501 aatatttttg gtatgataca taagaacgaa cgttagtact agggaaggaa gagcgatgat
250561 ggtgaatttc gattacaaca ttataaaacg taaccaagat tcagtggctc gttcttgggc

250621 tggttctttc tgggaatcag tcttcattgg tgttggcgct ttcgttggtt ttttagcgac
250681 ttttaagact caatgtgatt agctattttt gtcagcttat catattgagt cttttttttg
250741 tatttgtcat ttaatgtgtt tcattaggag gaaacgatga aaaagttcaa aaaagtcttg
250801 gttggccttt ttgcaatttt acttgctgtt gttgcaactg cttgttctaa caagtcaaat
250861 agttcaaaag atggttatac acctaagtca ttaactatcc aatttgtacc tagtcaagct
250921 gctaataaac ttgaagctcg tgctaagcct ttagaaaaga tgctttcaaa gaaacttggt
250981 atcccagttc atgtttcaat gtcaactgac tacaacactg ttgttgaagc aatgaaatct
251041 aaaaaggttg atgttggttt cttgccacca gatggttacg tattagctca caagcaagga
251101 gcagctaatg ttttacttca agcacaacgc tacggtgtaa aacaaccagg tggtcaagct
251161 actaagaact tggttaactc ttatagagcc gaaattcttg ttaagaaggg ttctaagatt
251221 aagtcttgga aagatttaaa gggtaagagt atttcagttc aaaacccaac ttcttcagca
251281 ggatatgttt tcccagttgc tgaacttgaa gaaaaaggat taaacgttcc aaaagattgt
251341 aagttagtta ctgttactgg acacgaccaa gcagtgctta acgtattaaa tggcgatact
251401 gacgcagcat ttgtttttga agatgctcgt aacatcgtta aaaaagataa ccctaagatt
251461 atggaccaag ttgttccaat ttacttcact aagccaattc ctaacgatac tatttctatt
251521 agaagcgata tgtctaagtc attccaaaag aaacttgcaa aggcatttat tgaagttggt
251581 caatctaaag aaggtaagaa gattatcgaa tctgtttaca gtcacgaagg ctacactaag
251641 tctaaagaca gcaacttcga tattgttcgt aaatatgaca agattattgc caaggacaag
251701 aaatagtcaa ttaaatatag cttagttagt aaaaagataa ttgtctctaa aagatgatta
251761 tctttttatg cactgtaaac aatataaata tgttaaggag aaatttagat taaatggcag
251821 ctactaatca gccaatgatt caacttaaag acgttagcaa aatttatgat aacggaacag
251881 ttggtttaaa ggatattaac ttaaatattg ataagggaga atttgtagta gtggtaggtt
251941 tatctggtgc aggtaaatca acattacttc gctctattaa tcgtctacaa gatgtttcta
252001 agggagatat cttaattgat agtaaatcaa ttacgtctgc taaaggaaaa gacttgcgtg
252061 aaatccgtcg tgatatcggt atgattttcc aaagttttaa cttggttaaa cgttcaagcg
252121 ttttgcgaaa tgttttaact ggtcgagttg catattatcc aacttggaag acaactttta
252181 acttatttac taaagaagat aagcaaaaag cgtatgaagc attgcaacgt gttgatttgg

252241 cagataaggt ttatactcgc gcagaccaac tttctggtgg acaacaacaa cgtgttgcta
252301 ttgctcgtgt acttactcaa aatcctaaaa ttattttagc cgatgaacca acagcttctc
252361 tagatcctca aacttcacgt cgagttatgc atgacttaaa gatgcttaat gaagaatacg
252421 gtatgacagt tgttgctaac cttcacagcg ttgaacttgc taaagagttt ggagatcgag
252481 taatcggtgt ccgtgccggt caaattgttt atgatggcaa gatgagtgaa acttctcaag
252541 ctgtctttga cgatatttac aatggcggaa acggcaagaa aggggaagaa gatgcctaaa
252601 tctaaggaac agcatttagc tgaattgcct aaaaagaaat ttaaaattat gaaccttgtt
252661 tgggtcgtaa ttatggtttt aggtctcttt ctttcggtaa atgtaaccaa tacgcacatt
252721 agcgttttgt ttaataactt tagccaattt accgatattt ttgtgcaaat gtgtcaccca
252781 gactggggat atgctgctac agcagttccg cttttaattg aaacaattaa aatggctatc
252841 ttaggaactg tgattggttc agctattgca tttgtatatt cgcttttaat tgcaagaaat
252901 attgttaaaa ataaggcagt tactggcatt ttgagagtga tcatgaatat tgttagaact
252961 attccagatt tacttttggg tgcaattttt gttgcaattg ttggtattgg cccagttgcc
253021 ggtgttttag ctttatcagt atttactttt ggtgtagttg ttaaattgtt ttatgaagca
253081 attgaaacca ttgatccagg tccaattgaa gctttaactg ctgttggggc aaacaagctt
253141 caaattatcc attttgctgt gatgccacaa attattactt actttatttc ttatgtttta
253201 tatgcatttg aaattaacgt cagagcttca acagttcttg gatatatcgg tgctggtggt
253261 atcggtcttt acttgcaaca aactctacaa gtctttgact atgctaaaac tggtctaatt
253321 attttgatta ttatcgtcgt cgtagttctt atcgactatg tttctactaa atctcgggag
253381 gcgttgatga aataatggat actacaatga aaaaactacc tcctaaacca attgacacta
253441 agaaaaaaat taagcattgg gtaattgcaa ttgttactgt agctttaatt atttggtcat
253501 ttgtaggaat gaactttggt ggaataaaag cgactgctgg tcaaattgcc ggtgctattt
253561 tggctggtat tttccatcca gattggtctt atgtatataa cggaagcggc gaagatctaa
253621 tttctcaatt atgggaaaca atctgtattg ctttcttagg tacttttatt tctgcagtta
253681 tctcattacc ttttgctttt tgggcagcta acacgcgcca caaaaaatgg tatacttccc
253741 gctcgggaaa aattgtttta gctgttattc gttctttccc agaaattgtt ttggctttga
253801 tgtttattaa agctgttgga cctggttcag cagccggtgt tttagcttta ggttttcact

253861 ccgtggggat gcttgctaaa ttgttctcgg aagcaattga gaaccttgaa gaaggagcta
253921 atgaagctgt tacagcagct ggtggatcta agtttaacat tattatgttt gcaacaatgc
253981 caaacttaat gccagcttta atttctaata ctttgtaccg ttttgacgtt tcaattcgtt
254041 cagcatccat tctaggttta gttggtgctg gtggtattgg ttatccatta attattgcat
254101 tgcaatatcg tcaatggaac cgtgtaggtg ttatcttagt tggtattatt gtcatggttg
254161 tcattattga ctggatttct ggctggattc gaaagaaatt agtctaattt attatttaaa
254221 cgagtagaat ttttagctac tcgtttttat ttttgtcaaa ttctaaattt ttttgtaata
254281 tctacctata ataatgataa gtaaaggaaa aaaagaactt ttttgttata atggaagttg
254341 tatttcaagg aggacaatga tggataattt aactaccatg acattggctg atgatattgc
254401 tttatcacaa gaaaaaatta tcaacggtca acaagacctg aataccaaaa gaatttataa
254461 tgaaattaat agtctaggca ttcttgataa gccaatcgaa gaatactttg atatgactca
254521 agatgaatac tacaacagtg aatctgatgg taaattaact ctacttcatt tagatcaacc
254581 actaagtgaa ttaagcgatc gcattttgac taatcatgta gatggctatg ttgataagga
254641 tgaaattaat ttaacctata atcatgaaga tccattgcaa gatggtagct atgatcgtgc
254701 tactgactta catgtacttt tatatagttt gaaagttatt ggtgctgtca gagcaattgc
254761 tccagatgac ttaaaaaagg ttttgtctaa agatgcggtt ttatcattgg gtcttgccgc
254821 agctgctttg gcaaaaaatt aaccccaagg ccaattaaag gccttttttt gtgtgatgag
254881 tggggaagag tatcatgaaa attatcaaca aaaccaaagg tatgattgat aagttttccg
254941 ggatgactcg gaagatgatg cgaaagaaaa ataatgttcc atttgacgga atgcaaaaat
255001 acatgactgt cggtgagtat aacgttggag caccagaagg cacgccacat ggcgaagaat
255061 ataagcttat tgtttacaaa gatactgata atgttttgca ccaagctttg cgttcagtgg
255121 acgcttctga tttagcacca gcttatgtca gaaagtttga taaaaggtta aatcggtata
255181 ctgcctttgt taataagcgg accggacgtc gttatatcgt tgaagatcag ttagatcaat
255241 tagtcgacta tgtaaaaaag cgtagcagaa aaggctacaa ttcgattaat attggcgttt
255301 taactgatac tcattataaa gatactgata gtattgattt ttatggccat aacggtttaa
255361 gacatgttaa ggaatttaat tatcttgaag acaaagacat tttagactta aaagctcatt
255421 taggcgattg gatggatggg tctgatccag gcttagtagg tgaagccgaa ttaatttcct

255481 tatcacgaac atttcgctct aaaaagacta attttgctgt gattaagggg aatcatgatg
255541 aaaatgacaa atttgatgaa catcatgatt taagggctag ttttcctgaa aatgaattcg
255601 aagatattat gtggccaagc atgtatgcac aagatggcat tcattacatc acgcgtaaac
255661 atggtgtcgc atacttcgat aaagatgatg tcagaattat taccgttaat acttctgact
255721 taccctatga attagacgat caaggaaaga aaaagtatga cacgaagctt tctttagcta
255781 ttcgtgaaga ccagatgcaa gaaattatcg aaattcttga aaattcaaat ggtaaaacga
255841 ttattttcat gagtcatgct aaccctatta ctcgcaaggg aacaaatgcc ctaaaattca
255901 atgggcgttc gctacatgaa ttgatggttg cttttaatca acatgaaaaa ggatacctta
255961 attctagaga cgttcctcct gaatttacct tagctaatag ctttgatttt acgaaaatta
256021 agaatgcaaa aatagttgca tatttctgtg gacaccgtca tactgaggac caatatcgga
256081 ttaatggtat ccaatatata ttttttaatt gttcggccct tatgggacca aaccatgcgc
256141 ttactacgca atataataag cgctggaata gacaaatgga tcatgctaat gaagcagcag
256201 gctacattgt aaatgttgat attaaaagac atttactaca ggtatttggt tatggtgcgg
256261 cttctaagag aagattctat cgaatttagt ttttgaaatg taaatagctt ataataagac
256321 tactgtttat tttgaggtga aagttactta tgtgtggaat ttgtgcgttt ttcgatcctg
256381 aattgaagga taaagactgt gctattcaag gaatgatgga cacgatcaag caccgtggac
256441 catcttcaga tggaaagtat accaatgatc aggtggcttt aggttttaga cgtttatcaa
256501 ttattgattt gcgtggcgga agtcaaccta tttttaatga agataaaagt agagcaatta
256561 tttttaatgg tgaaatttat aactttaaac cattaagaaa agaattaatt gatgctggtc
256621 acaccttcac tactaaagct gatactgaag ttttacttca cggctatgaa gaatggggaa
256681 tggacggctt attgaagcgc gttcgtggta tgtttgcctt tgtaatttgg gatgataata
256741 ctaagacttt atatggtgca cgtgatttct ttggaattaa gccaatgtat tattcagacc
256801 aaaatggtaa gttgatcgtt ggtagtgaat tgaagagttt cttagcttat cctggcttta
256861 agaaggaatt aaatactgaa gcagttaagc catatttaat gaatcaatac aatgacttaa
256921 aggaaacttt ctttaagggc gtttaccgtt ttccagcagg tcactggttt gaatataaag
256981 atggtaaaat gaagactcat caatactggg atgctaagta tgttgagaat agcttaagct
257041 ttgaagaaac tttagataag atcaacgaag atttaaagga aactgttgac ttatatagaa

257101 acgctgacgt accagttggt gcgttcttat ctgaaggtat tgattcttct tacttaacta
257161 gcttacttga tcctgaagat gtcttttcag tttcttttga tgattcaact tataacgaag
257221 catcgaaggc taaggcactt tctgatttac atggctggaa gttcttcgct gataaggttg
257281 atgcagatga agccatgcgc gactttccag aaatgcaata tcacatggat gaaccagatg
257341 ctaacccatc aattattcca ttatggtact tgtgtaaatt ggctagaaag catgttactg
257401 ttgctctttc tggtgaaggt gctgatgaat tatttgctgg ttatgtaaac tatggaatgc
257461 atacgcataa cgacgtaatc aaggtcttta ctgccggttt gaagaaatta cctaagaaga
257521 gccgtgttcg cttagctcat aagattaaga agatgcctaa cttcccaggt aaggtacata
257581 tgtacactaa cttagcagaa ccaagcgagt tttacgttgg tcaatccgta atttatgata
257641 tggattatcc gactatcttt acttctaaag atgctaataa tatcttacgt gataagtaca
257701 agaatgattt gacagttaat ggcatttatc aagaagactt caagaaagtt aagaatattg
257761 atgaagttaa gcaaatgcaa tatattgatc ttcaccactt tatgcttaat gatattgagc
257821 aaaaagcaga taagatctca atggctcact cattagagtt acgtgtgcca tatcttgata
257881 agaagattgc agagcttgct aactcaattc caactaagta tttagttaac cgtcatgata
257941 ctaaatatgc tcttcgtaag gcctctgaaa aagtcttgcc agaagaatgg gcacaaagac
258001 caaagcttgg cttcccaaca ccaatcaagc aatggcttaa ggagcctaga ttctataagc
258061 aagttagaga attgtttact gaagactttg taaatgatat ttttgaccaa aagaagattg
258121 ttaaattact tgatgataac tacaaaggcg atggctcagc tcgtcgtcaa atctgggcaa
258181 tttacacttt cttagtatgg tacaagttgt tctttgttga ctatgatgaa actgttaaaa
258241 agtatcagca tgttcaacca gaggttgcag agttaattga aagtggcaga cttgtttaat
258301 taaagttaga gagaagtacg gatacgaaat ggtacaagca aaatttactc ctattttatt
258361 aggtagcgac atcaatgttt atggaatggc acgttcattt aatgaagcct atggaattaa
258421 ggttcaagca tgggcagcta gccaattagc agcaacgcgt tactctaaaa ttgttgatgt
258481 ggaagttcat gaaggctttg aagaagatcc aggctttatg aacgtaatga agcagaaaat
258541 tgaagaatac aagaatcacc cagaaccagt tattttaatc gcttgtgggg atggatatgc
258601 agaattactt gctaagcaca aagatgaatt aaaggatact ttcgttgttc cttatattga
258661 ttatgacttg ctagagaagt taatttctaa agagggattc tacgaaattg ctgaaaaata

258721 tggtcttcca tatccacata ctaagattgt gaccatggat gactacaagg ctgaaaatta
258781 cttaaattta ccatttgatt atccaattga attaaagcca gaagatcctg tttcttggtt
258841 aaattgtcaa tttgaaggcc gtaaaaaggc atttactatt cacgatgaaa ctgagttagt
258901 cgatattgtg ggcaagattt atactaacgg ttatactgaa gacttgattt tgcaagactt
258961 tatcccaggt gatgattcta atatgcgtgt tcttaatgcc tacgttgata aagaccacaa
259021 ggttaagatg atgtgtctag gccatccact tcttgaagac cctactccac agtcaatcgg
259081 taactacatg gcaattttgc cagcatttag tcaaaaactt tatgatacag ttcaatcatt
259141 ccttgaaaag ttgaattaca caggaatggc caattttgac attaaatatg atccaagaga
259201 tggagaatac aagttctttg aaattaactt gcgtcaaggt cgttcaagtt tctatgtaac
259261 tttaaatggc tataacttgg ctaagtggta tgtagatgat tatgtagaag ataatttaaa
259321 agataagcca actgtatacg gcaacaaaga tggtgctaac tacatgcttt ggcttggtgt
259381 tcctaaaaaa atctttaagg aatatgctta tgataacggc tcaaagcgtt tagctgaaaa
259441 attaattgat gaaggtcatt atggtactac tgtcttttac gataaagatc ggagtttcaa
259501 acgttggcta ttaatgcgtt acatgttcca caactactat gctagataca agaagtacta
259561 tgaggtaaat aagggacaat attttgaaga agaagctaag aagttaaaaa aacaagcact
259621 tcgtgatggt caacaagaaa accatgaaaa tgaaatttag ttaaggaata ccctctaagt
259681 agaatgagat tgcttagagg gtatttttgt atctaaaagt attaaaataa agatgatagg
259741 aaagctactt gagaaagtag gaaaatagaa tgattgactg gggattaatg gctctatgca
259801 tagtaacgat gttactagga ttttttgagc tgtaccgtac ttttagattt tataaatggg
259861 ataaaaaaac taaagaaatg ccaactgcac cttatgtgat ttattttgga acgtttttta
259921 gtggggtatt aattgtcgtg tcagctatgt ttatgatggg gaatacttca ttgacacttc
259981 ctaaaatttt ttacataatt ttgggaatta ttctagtggt cgtggcagtt ttaatgtatc
260041 gtcgtgggca ccaaatggcg aagaaattag gtaaggatga tagtaacatt gctgtttggc
260101 aaacctattt gattagcaca gtaattttaa ttactggctt aattaatttt cttagatgat
260161 aaaagagagc tgaaattcag ctcttttttt gtccaataaa ttgtggctta ttatttgcta
260221 ataattttct actttactga ttatagaaaa agtttaaaat tctcttcttt cttgtctgac
260281 gtgaattttg ctaaatacaa tatatatgtt ctgaaaagtc aatacctaca atatcatgta

260341 gtttttggta gaatataacc atacatattg tgaaagaaga gaatgaccat gttacaacaa
260401 gatgctcaaa ttaaatcaat tactcaaatg acccttccag aaacagccat taaaagagat
260461 ggtagcatct atccttttgc tctttacaaa attgaaatgg tcttagacaa cttgcatctt
260521 acagaacatg aagcagatat tttaccttct attttgaaaa aattaaatgg tgtaaaacaa
260581 acttctacga aagatattgc agatgccttt gtccaaactt taacagaact aggatttgat
260641 gatgaagcca atgcttacgt agactaccgc agggaagacg aagctaattt tgaaaagcaa
260701 actgaaacta ccaaccgtct ggatcgttta gtccaccatg atccaacgat tgttaatgaa
260761 aacgccaata aagactcacg tgtttttca actcagcgtg atttaactgc tggtgttgtt
260821 ggtaagacga ttggattgac aatgatgcct gaacatattg ctaaggcgca cctacgtggc
260881 gatattcact ggcatgattt agactatact ccacttagcc cgttaactaa ttgctgtttg
260941 attgatttta aagaaatgct tagtcatggc tttacgattg gaaatgccaa tattgagtct
261001 cctcattcaa ttgaaactgc tacggcgcaa atgtcgcaaa ttattgctaa tgtagcttct
261061 agtcaatatg gtggctgctc agctgataga gttgatgaag ttttagctcc ttatgctgaa
261121 aagaattacc ataagaatat taatgaagcg cgcgaatttt ttgatgatga agaaaaaatc
261181 aaggcttttg caatcaagag aactaagaaa gacatctacg atgctatgca agctcttgaa
261241 tacgaaatta ataccttatt ttcaagtcag ggacaaactc catttactac tttaggattt
261301 gggttaggga cttcatggat tgagcgcgaa attcaaaagg ctatcttgaa gattagaatt
261361 gaaggtttag gtaaagataa aagaactgct atctttccta aattagtttt taccttaaag
261421 aaggggctta acttacatcc tggcgatcca aactacgata ttaaggaatt agccttagaa
261481 tgttcaacta agcggatgta cccagatatt gttagctatg acatgattaa gaaaatcaca
261541 ggttcattta aggctccaat gggttgtcgt tcatttttac agggctggaa agatccagaa
261601 actggtaagg aagtaaattc tggtcgaatg aacttaggtg ttgttacttt gaatttacct
261661 agaattgcaa tggaatcaaa gggtgacaaa gatttattct ggcaaatctt tagagaaaaa
261721 gttcaaacag cacatgaagc tttacaaatt aaggctaagc gttgtactga agccgttcct
261781 gataatgccc caattcttta cgaatatggt gcttttggaa agcgtctgaa tgcagaagat
261841 aatgtaaatg acttgtttaa aaatggtcgt tgtactgttt ctttaggcta tatcggctta
261901 tatgaagtcg gtaccgtttt ttacgggcca aactgggaac acaatgaaga agctcatcaa

261961 tttacaatta atattgttaa agagcttcat gactactgcg ctaagtggga
aaaagaagat
262021 ccaaatcact accattacag tgtttactca actccaagtg aaagtttaac
tgatagattt
262081 tgccgtttag atactaagaa attcggtaaa gtgaaagata ttactgacaa
ggagtactac
262141 actaattctt tccactatga tgttagaaaa cacccaactc catttgaaaa
attagccttt
262201 gaagcaccat atccatttta tgcggctggt ggctttattc actattgtga
gtacccaaac
262261 cttaaacaaa atcctaaggc tttagaagct gtttgggatt gggcttatga
caaggtagga
262321 tacttaggaa ctaacactcc aattgatcaa tgctataagt gtggttttaa
gggtgaattt
262381 aaggcaactg ctaaaggatt tgaatgtcct caatgcggca accacgaccc
agaaacttgt
262441 gattgtgtaa aacgtacttg tggctatctt ggtaatcctt tgaagcgtcc
gatggttcat
262501 ggtcgtcacg aagaaattgt tcaccgtgta aagcacttga acttaggaat
ggaagaaaaa
262561 atggctaagg atgaagttag aaagaatgac gaatattaat gccagaaaaa
gataaaaaca
262621 gtcaagaagg tcctgatgtt cgaggtaact taattaaagt taatgttggt
ggcgatacaa
262681 tttttgtgga ttctaaccaa tataagccgc aagttgaaca tgctaaaaaa
ttaaagcagc
262741 aacataggaa acctaaaaat cctaaaccac aagaatggat atcggaagaa
tattctaagc
262801 ataaaattgc ggactataaa ccatttaact ttgttgatgg ggaaggcgtt
agatgcagtt
262861 tatatgtaag cggatgttta tttgattgcc ctggctgcta taatttggca
gcgcagaact
262921 ttaattatgg acgtccttat acacaagagt tagaagataa aattattgaa
gatatgtctc
262981 aatcttatgt gcaaggattg actttattag gcggagaacc atttttgaat
acttgggttt
263041 gcttaaaatt gataaaccgg gtgcgcaaag aattcggaca tactaaagat
atttggtctt
263101 ggtcaggtta tacttgggat gaactccaaa aagaaacgcc agataagaaa
gaaatgcttt
263161 caaaaattga tattctggtt gatggccgct ttatggatga cttaaaggat
cttactttac
263221 agtttcgtgg ttcaagtaac caaagaatta ttgatgttca aaagtcactt
aaagctaaga
263281 aagtcattat ttgggacaag ctagttagat aagtttgata gaataaggga
aacgataaag
263341 tttctcttat ttttttaggt gaaaaagatg caagaaaaga atttgaaatt
agtcgataaa
263401 attatgacag ctcttcaaaa agtagaagat cctgaactac tagttgatgt
ggttaatcta
263461 ggcttgattt atggaattga tattgaagga gatcatgcca cgattaagat
gactttaact
263521 attgttggct gtcccttgtc gacatatcta caaaatgcaa ttgaaaaggc
agttttgtcg

263581 gttcctgaga ttaagacctg tgatgtgaaa ttagtttggt acccagtttg
gagtccagaa
263641 agaatgacga cagctgctaa gcagcaatta gggatgcttg ataatgaaca
agctcttgat
263701 caagaagaag aaattgaaga caccgaggaa aagcaaaaaa tcgttgattt
ctcagtgccg
263761 attaagaaac tagctgaaga atatccggat ttcgttcaaa ttatgtatga
ttgtggtttt
263821 actagaatta aaattcccgg cttattatca acagtcggac gtgtgatgac
cattccgctt
263881 ggagcacagg caatgaaaat tgatcttaat aagataaagc aggcttttga
aaataaaggc
263941 tacaaggtga ttgataaatg aaagaattag ctaaacaacg ccaagaggca
attttgaaaa
264001 ttttaaattt tattcaaaat ggtggagaat tagaagaagc aaaaaaaatg
tttcaagctg
264061 cttttgatca ggttgatgtg gctgagatta cagcagctga aagagaatta
attgcgcaag
264121 gacttgatcc aagaaagatt caatacctgt gtaatgttca tgcggatgtt
tttaaaggca
264181 atattaaaga aaataaagaa agtccagaat tcgaaactcc tggtcatcct
gttcatactt
264241 ttaagttaga aaatattgta attaaatctt taattaatga tgccttgctt
cctgatttgg
264301 ctaagtggga agctggatct tctgatgctc tacctaagtt gaagcaagag
ttaaaagatc
264361 tggcaaaaat tcactatcac tatgcccgta aagaaacttc tatgtttcca
cttatgacta
264421 agtatggtat tactgctcca cctaaagtta tgtggggtgt tgatgataaa
attagaaaat
264481 taattggtca agctaatatg cttgttaatc aaaaaaatgt ggatccaagt
gaagttgcta
264541 gtgtaataaa aagtgctgct catgaagttc ttgagatgat tttcaaagaa
gaaaaaatta
264601 tgcttccaat gcttgatgaa gttgctaatg aagaagactg ggcaatgtt
aagaatgaag
264661 aagagcagat tggctatact ttaattcaaa agccgatgaa ctggaagcca
aagataaagc
264721 caaaagctgc agggccgatc tctttagata agttaagcag tttagccttg
aactttgcag
264781 aaggtagctt aaatttagaa cagctgtcag caatcttaga cttattgcca
tttgcgatag
264841 cttttgtcga tgaaaatgac aaggttgcat attttggtgg gggagtaagt
atttttccgc
264901 attctaaaaa tgcgattggc aatagtgtat attcttgtca tcttcctgag
agtgtgccac
264961 gagttaagaa aatttttgat gatttccatc aaggaaagaa agataagtat
gaattttggt
265021 ttgagccgcg gcatatgggg cgatatttat atttgcagta tttagccgtt
cgaaaagatg
265081 gaaagtattt aggatgctta gaggttgcac aggatgtaac agaaattaga
agctggacag
265141 aagaaaaaag ataaaaaata gctcgtattt tgcgagcttt ttttgtttgg
gatctattgt

265201 gtgttatgtt aaaaatgtaa tctataaaac ttaattttgt tttccattag tttacgtgaa
265261 tgtcaatcat aacttttatt taacctgtaa agtatatgta ttggaaattc gtaataatgt
265321 aataattgat aaaattctag tataataaga atagcaatct ctagagttaa tactgataaa
265381 gtagtaaatt gagttgttat aataaattaa tcaaaaatcc tataagaatt atttttaacg
265441 cgttaaaata actatagaaa tcttataact attaccaatt taaaattgaa gggacaggct
265501 aaaatgttat tcaataaaaa agactttgat tctaaacaac gttttagtat tagaaaattg
265561 agcgttggtg tttgttctgt tttactttca actttatttc taacttttaa tgaaggtcag
265621 acagttcacg caagtacgga tgttaatgcg gcggacgcta caacgactag tgtatcatct
265681 aatactacta ataatagttc tgataataat actgataaaa aagatgactc aaaagtagag
265741 gcatcttctt tggcaaccgc tgaaaagcat gccccaattc caattgctgc tgaaattaag
265801 agtaacaagt cagaagatca gacaaatagt gcatctgctg aaactgatac tcaagtaagt
265861 aaatctaagc aagcacaaag tcaaacaact attaatattg ttaaagctac tgctactcaa
265921 gcatctactt cttcaaaaga tagctctgac acaacaagct ctaaggataa ttctgcccaa
265981 aatactgttg aaaaaaatag atcaacctca acttcaaaat atacacctgt cttaactagc
266041 gaaacttccg tttctactaa aactaatcaa agtaatagta attctgctac tcaagaaaaa
266101 tcgaatatta aacctgtaac ccctcatgct gattcaactg aacatattgc accagagact
266161 tatactatgg atagcgactc acctgcttat gtagttgctg gaagtgattt aacgaatttg
266221 gcctcaaaat ttatttccaa tgcggatgct ttaactcaaa gcggggctac tttttcttgg
266281 gatggcaatg ttccaactgc aacgcaacaa gatgccattg atggtaatac gatatctgga
266341 actatcagag taacgtataa agatggtaca agcacaacta ttgatattga atcttacgtt
266401 gaagcacgat cacagcttca accaagtacc ttctactatg tgaatagggt aaacgatacc
266461 ccagacttta ataccgccga tgctaatggc aatgattttt ctacaattct taacaataca
266521 tacgtatatg atccgactgc cttttcttac aagatattag ggacactgga tacaagtagt
266581 ttaggtatcc actgggccaa tgttcaagtt acggataaca atacttttgc tggtgatccc
266641 gatgctccta aagttatcgg tggtctttat gttgttaaag ttccttacgt agttcaaggg
266701 cttaaattac gggatgatat tccaactgat gataatggta atcctgtaat taatgctcag
266761 ctagcaacaa tgccgaagtc aacatctacg acaccgacaa caccagaggt tgcttttgat

266821 cctgcccaag gaaattctac taattttcca gcaggcttat ggggacaata tttctaccaa
266881 gattatgctt tagcttacgc attaggtatc aaagtgcaag tagctgaaga tgacctagtt
266941 aataatggaa agtggaaaaa caaaccaact gatttagtca atactaagac caaccacttc
267001 gcaataactt ttaacaagtt gtctaatgct acccagcagg aagtagacgt aaattacgta
267061 tctgctccac atgctgagga tatgtatatc tataatgcca caggcaaaac ttatatgact
267121 ggtcgaccag aaaatgcaaa taaaattaag gaattgcttg ctgacggcta catgaatgat
267181 aaagtttggg taacgttagc tgatggcact aaggtctatg cttctgaact tgaatatatt
267241 gaaaatcgta ataactttgt tgttggtggt gcaagaagcg gcaaatacaa cattaacttc
267301 aatgctagtt ttaaccagaa aaatagcgct ggccaaccgt tgattttttc aaataacatt
267361 tttagtgcag caagtaaaga atttaatgat gagctaaata agtggtatca aaagactaac
267421 aatccgacgg ttgctaatta tgctgcacct actgccacaa cctgggataa ggtttcaact
267481 aatgggattt ccttagcacc aacgatcaaa gccagtgatg atccccacgt tcaaacttta
267541 accaacggtc cagttacttt tgatagtgag gcactcgaaa aattggtaaa aaacccaagt
267601 acgggtgcag aggtaaagaa agtccagggg acttggatag tcaatatgac gcctaaccca
267661 attttggaca ataacacgaa atggccagaa ggtactaaac tcacttgggc cggtaatgat
267721 ggcagtacca aattaacctt cgataaagcc ggtgaaagca agaccgggaa cgtaacaatt
267781 actcttccca gtggttcgtc atacacggtt aaagacatta cagttatctc taaagctaat
267841 gtgatcgcaa agagtgaaac tgttgactat gggactactt tgacagcagc tgacttagta
267901 actaataaga atgtcttccc agaaggaact acttatcaat tcgttgataa ttcagaacca
267961 acctggggga cagctggttc atataataat gttaaaatta cagctactta caaggatgct
268021 agcggagcaa gtgttactac accagttgca aactgtgcgg ttgctattaa cgatagccgc
268081 agcatcaagg tattagttgg cagtgatgtt ccttcagttg atagtgtctt gaacttgcca
268141 agcacttggg aagaacatac tgcatcttgg actacttcac ctaacaccaa ctcgactaat
268201 gagggtgtaa ttacgatcca ttatccagct agcaatttgg atcaacaaat taaggtttat
268261 gtaacggtta ttccgaagac caccgcagtt gatggacaaa acttcttcac caatggcgag
268321 aagtatgatg gaactactgg tagtattgct aacggcaata accaaggtgc aattttaacc
268381 caagctaatg gtcaagcaat tgactacacc gcttatagtg gtaccaatac agcgggaacg

268441 caaacatctt ataatacgaa gacgactagt tacacaccaa cttacagttt gtcaggtctt
268501 caaaccaata gcgatggcag tctagttagc ggtgctcaaa ccgcaacagt tcgcgtgtct
268561 gtacctaagg gaacgcttgg cgccaaagta gatactgatg gtaattacta ctatgaagtg
268621 aatgctaagg tcaatattac tcaaccagtt acctttgagt ttgtggatga atataagaat
268681 gatcaagtag ttggtcagac ttactcacaa gagtttattc caggtgttgc cactaatctt
268741 aactttaata tggcaatgcc agacggctat gaactaggaa acggtgcgtc aattccaagt
268801 cagtatactt tgactgcttt tagtcaaacg acgctggttg ttcaagtgcc aattcaccaa
268861 aagatgcact tctacattac tttccacgat gaagattcaa atactaatct tggtacggta
268921 gatgtgtcag gcagtgctgc agcaggaaat ggtggatatt atccaaacat taatacgaaa
268981 ttaacattcc cagcaggtgc taatgcagga gactactatt ctgtctcaac ttctggagtg
269041 ccagatgggg tacagctact tggaagttat ggagtaccac ttactgatcc aaaagctgat
269101 tggactactc caaactatca gtgggagaat actttctccg ttcttaaggc tttaaccggt
269161 gcaacgatga ccattaacct taagcataag atccaggaaa cacaagaaca acaaactcgt
269221 aagccgactg tgaactatat taaggctaaa gttaatgccg atggtactta tacgccagac
269281 gggtctgcat ttacttctgc tgtccttgat gtgtactact caagaactaa gtcaaaggat
269341 ctggttacta agaaagttac ctacactcca tggctttggg ataccaagca agggcagaat
269401 ggttaccatg ttgaatctgg taagtggact agtctgccac agcagtgggc agctgttgtt
269461 gcagatgtgc cgacattaga tggttacact gctgctacaa ttaccgataa atctggtcaa
269521 ttagcaaatc aatttgtctt cccaacttgg aataattcag atcaagatac atctgatatt
269581 aataaggaat ccatagcata tactgcgaac gctccaattt atgaagcacg tccagttcat
269641 actgttttgt atattcctaa tgaaactcaa actcggactg tcactgaaaa ctatgtttat
269701 gctgacgctg gcgatttaac tggggaagaa gtattttcac catataagct tgagatcttt
269761 tataaaaaga ccggaagcct aaatgtgtca actaacacaa tcacatatag tggttggagt
269821 tgggatactg atgctggcga tagcgataca ccagggttcc acgttatctc tggtcagggt
269881 aattggactg gcttaggaag taacggtcaa ccttcaggat cttcgcttgg aattaacgtt
269941 cctactaaag atggctatac tgcgatcatc agtgcttatc cagcagcaat ttatagttca
270001 actgtttttg gcaatccagg agtcaattct ggaacgggtt atacaaatga tactgattca

270061 acttggtact ggcgtaattc gttaactact tactatgttc ctaattctaa
acttagtaaa
270121 acagttacaa gaacaattaa tattactcaa ccaggtaaag aaacagaaac
gacagttcaa
270181 aagtctacaa ttacacgtac cgcacacttg aatgttaatg atagtggaat
tgcttataac
270241 gattggggta ctgattcaat gtattggtat gcattaaatg taccaaaagt
aggcggctac
270301 acaatgactg ttactaaaac agttaatgga gttactactc caattcaact
cgttaatggg
270361 caaattccag ctgaaacagt caatggtact tcgcaagata caactattaa
tatcacttgg
270421 ggtgctgtag ctacagctac attaactggt actggtacta gtacttataa
tggtcaagct
270481 attactaatg ctgaacttaa taatgctttg aaagtgaatg ttacaggacc
aactgcaggc
270541 gcaggtacat atactcttca agccggcgat gttgaatttt caactgatgg
tacaaactgg
270601 actactgcct taccaatcaa tgctggcaac tatcaagtgc gcttaactgc
tcaaggtaaa
270661 aatggcatta ttggtaaata cggcaacaac agtattattt ggactcaaaa
tgggcaatca
270721 actattacca gcaatgcaac ttacatgatt aataagcttg ctgataatgc
tgtaatggct
270781 aatgctcaga gcggcaatta tgagatgact tataatggtg ctgctccaag
tacaattgat
270841 ccaagtaagt ttgtatttac aacgactgtt aatggtcaaa ccgttacgct
tgatgccact
270901 ggcttaaata gcagcgactt tacttgggct gatggtaatg cgccagttaa
tgtgggtact
270961 tacaaggttg tcttgactga tgctggtttc gctaagttaa atgctaataa
tccaaacttt
271021 gacttaacta attctggcaa cgggactttc acgattaacc aagcaaatgc
ttcagctact
271081 ctttcaggta gcgcaagccg tgaatacaat ggccaagcag tcagtgttga
tgatctcaac
271141 tcaactagta ctggtaacaa cattactttg actttgcatt atccaaagga
tggtaatgct
271201 aactattcaa ctacagttaa attaaatact ggctacttta cttggaatac
accaaatggg
271261 caggcgccag ttaatgcaaa tgatcagcct tacacgatta gtttgaacag
tgatgcaatt
271321 gaagggatca ttaagcaagc tgtcggcagc ggtcaaaatg gagtaagcaa
tgtgaagttt
271381 gctaataatg caattagtgg tagtgcaagc tacaagatta cgccattaga
aactgcagct
271441 gaactttcta acattactga tggtaactat acgaaagttt acgatgcaca
agctactaat
271501 cagattgatg cttccaagct gcacatcact gcaaatcttg gtggtcaaaa
tgtggactta
271561 gatactacag gactcactgg tagcgactat caatgggttg atgcaagtgg
tacagcgtta
271621 acttataatc caaagaacgt tggtacttac tacatcaagt taagcgatac
tgcttttgct

271681 aagttgcaag cacagaaccc taacttccaa ttgacaagta acgacggatt aggtgtttac
271741 acgattacac caactactgc taatattact tttggcggta ctaactctcg tgtttataac
271801 ggccaaggta ctactactgc tgaaattaac agtaatggtc aaattaaaat tcactttact
271861 ttccctggct cagtttcaga tgcctcttat acgcttaaag atcgtgatta ttacttggtt
271921 gatcaggctg gggctggtta tgatggtgct caaataatta atgttggcac ttatacgatt
271981 aagctgaccc aacaggggat gcaaaatgtc attgatacga ttcatcaact atcaggtagt
272041 ggcgatagcg gctataacat taatgttgac tccgcaactc aattgggtaa cttaacttca
272101 acatttaata ttgttccttc gcaaaatatg attagcgttt ctggtacaca aactgaaacc
272161 tacaatggtt caccaattaa tgttgtctac aatgctgatg gcactaacag tgtgaaggtt
272221 tcaatcgctc aaactagtgg taataccact ggtgctgttg caagtttaac cgatgtaact
272281 cttgattcag gtgactttgc aatcgttaac ggtccagctg aaaatgcagg tagctatcaa
272341 gttaagttga ctgatgctgg cttagctaag attcaaaatg cagttggcga taattatcat
272401 attagtattg atccaaatgc ttttggtact ttagtaatta aaaaatacaa ggctagtgca
272461 gtcttcagtg gtgatccttc atacacttat accggaacgc ctgttagtgc tgatgattac
272521 ttgggtaaat actcaattac gttaaatgag cctaacaatc caacttataa cttggttgct
272581 ggcgatattg aatttaatgt tgacggcaat tggacaagcc aagcccctgt taaagtcgga
272641 acgtatcaag ttcgcttatc acagtcaggt tggaaccaca ttaaggcaat taatagcgat
272701 aatgttgagt gggctgctac tgcttctgca ggtaaaggat cttacacgat tgaccaggct
272761 aaagttaccg ctgatcttag tggtaataac tcaatgattt acaatggctc acatgtaact
272821 actgctgact tgtatactga gggttcaaca attaaggtag ctattaacgg tactggaatt
272881 gccaacttgc caaagagctt cactttagaa gatggtgact atacttggtc aacgagtgat
272941 aaccaatcgc cagtaaacgt tggtaattac acgattaagt tgacacaggc tggtatcaat
273001 aagatccaaa gtcagattga tcaggttgtg ggtgctggta acgtggtcct tacgacaaca
273061 gctgacgacg gaggttctgc aagctttgag attaagcagg cagttgccgc taatgttcag
273121 ctatatggta gagaaaatac tatctataat ggcaatccag tcagctttga tccaactaag
273181 actgaaacag ctaacaactt tggcttccat aacgttgaaa acttaacagt tcctactttc
273241 acagctagtg actttacttg gtatgatggt caaggcaata aactttcatc tgcaccaact

273301 aatgctggta cttattactt gaagctgaat actcaaggtg aaaaggcctt
tgcggatgct
273361 aaccctaatt acacttttga agaaaatggt aagagcacaa tttctggaga
aatcccatat
273421 gtagtaactc cagctgaatt aacaattggt gtttcaggta gtgcttctaa
agtctttaat
273481 gacaagaatg ctgaaattac gcaagagcaa atcaacaatg gcgatattaa
attagtttgg
273541 ggcaattcaa ctactgagcc aactgattta ggtaaattta ctttaactcc
atctgatctt
273601 gaagtagttg atgccaacgg caaacatgct atccatgcta atgccgggat
tggtgccgat
273661 ggtcaacctg taaaaggtaa tccttacaag gttcaattga ctgctgacgc
tattaaaaga
273721 attaagcaat tatcaggtgc tgctaattat aagattagtc agtcagctaa
gagcggtgag
273781 tacttcattt atgaacataa agctgaatta accttaactg gtaatcaaac
gactgtttat
273841 ggaacagcaa tgccatttag tccaagtaag tatcaattag cctttactaa
ttggctttct
273901 actgatttac cagttccaaa cttaacttgg aaagacaata agcttttaaa
taatgataat
273961 gatactggac ttacctggac agttggtgac ttatatgttg aaggctatcc
ggatggtggt
274021 gttccaacta atgtcggcac ttaacgcgta aaaatttcta agcagctaac
tgataagtta
274081 cgccaattat tcccagacta tgacttctcg ggtaacattg gggaaaacgg
tgttgatgaa
274141 caggataatg aagagtttgt tgaaccaagt catacaccag cttcatatgt
tattacgcca
274201 gctaatacaa cggtaaaaat tactggggct caacatgtga aatatggtga
atcgactaac
274261 attaatgttg gtgattacag tatttctatc accgcacctg ttcacaatga
aactgttgat
274321 aataatgcag aacaaattta tacaaacgta actttagctg ctggtgatct
tgagtttgtt
274381 actgtaccta ctaatgtggg tacttatcaa gttaagttat cagcacaagg
tttgcaaaag
274441 cttaaagatt taacgggttc aagcaactat gattggacgc aagctgcgga
tgctaaggct
274501 aacttctttg ttgaacaaat gccagtaact attgctgttt caggtcaatc
aagtgtaact
274561 tatggtacgc aagattggct caatgcaatt aaagtaaacc catctggcta
tgtattaaca
274621 attaccactg aaaatggggc tgctctaagt tatcacacag ttgatggtga
tctagtattt
274681 aaccaaaccc caggtaatgt tggtgaatac caagtagaat tgtctgcaca
aggtttggct
274741 aacattgaag aaaagctcgg cactaattac tcatacccac aaaccgctgc
tgatgtaact
274801 gcgaagggaa cattcacggt taaacaaggt gaagttactg ttactcttaa
tggtagtgac
274861 ggcaaaactt ataatgccgt accaacctta tcaagtggtc ttaacttaga
taagtacaac

274921 gtaacttact ctgctacggt ttactccgct gatggtaaag cacagactct aactttgaca
274981 gctaatgatt tacaaattat tggtgatgca actaatgtcg gtacttatca agtaaaacta
275041 tcagaaattg gccaagaaaa gctaaaagca ttaactggta atcagggtgc taactacaag
275101 tgggcattta atactaatgc tgattatgta gtaaaagcag ccacagctag tgcagaactt
275161 agtggctcta accaaaagac ttttgatgga actgctgtaa caactgctga agttaatagc
275221 aatgggcaga ttttagttca ccttacttac ccaggctcta atgttcaaag tacttacact
275281 ttgcaagacg gtgactacat ttgggagact gaggatggtc aaataatttc tgcaccgaca
275341 aatactggta cttacactat taagttgaac aagcaggcta tcttagttca cttacaagct
275401 gctctgaacc agcaagctgg tttaggtgac aatgatcagt ctaatgtgac tgtttcagca
275461 gataaactaa gtggtcaagc aagctttaaa attaatccac aaacactcac tgacgtaaca
275521 atttctgcac ctgatcaaag taagacttat gatgctcaag cagcggatct tgatgttaat
275581 ggaataacta ttactgcaaa tggaatcgtt gctaataatc cattagtaaa tccgggtatt
275641 agcgctagcg acttcacttg gtatgatgaa actggtaata agcttgaaag tgcaccggtt
275701 gatgttggaa cttaccaggc acgcttaaat gcaagtacgc ttgctgaatt gcaaaataca
275761 aatccaaact atcaatttag ctctgtagct ggtttgatta actacacaat taatccagct
275821 cctgccactg caaaaattag tggttcagct gatcgtgact acaatgctca aactacttct
275881 gtttcggatg taatgaacaa tatcaagtgg aaggcaacag gtttagttac tgaccaagag
275941 cttaatctta atggtttaac tgctaatagt tatgcttggt acagtaaaga tgctgacggt
276001 aactacgttg caatgactgg taatccagta aacgctggca cttactactt acgcttgact
276061 aaggatgcaa ttgcacaagt aaaagctgat aattcaaatt acaactttac ttcagttgat
276121 ggcgaattta cttacacgat caacgttgta aacggtactg caactcttag gggctcatct
276181 tctaagactt atgatggtca agctgcaact actgctgaag tgaatagtac aaacggtgat
276241 attattgtta actttgcttt cccaggatca agtgctcaaa gtacttatgc tttgcaagct
276301 ggagattaca tttgggagaa caaggatggt caagaaatat cagcaccaac taatgctggt
276361 acttacacta ttaagttaag tgcagatggt attacaaact tgcaaaacgc aatcaaccaa
276421 tatgctggcc aaggaaatgt aactttagac gcacaagatc ttttaggtgc tgctgtttac
276481 acaattaagc aaaaagctct taatgtaatc cttggtaata atagtactgg tacagatggc

276541 aagacttacg atggtcagcc agcagtaatc aatactcaag ctgaaaactt tggcgtgttc
276601 accactagtg gcctagtaaa tggcgaagac ttgaatgccg ctaacttaac taatgacgac
276661 tacgaatggg tagacgcaaa tggtaaggca atatcagcac caactaatgc tggtacttac
276721 tacatttctt tgaccgcaaa tggattgaag aaattacaag ctgacaatcc taactacgca
276781 atttctgaaa gtggacaatt tacttacgta attagtccag ctgaagaaaa tgtaacaatt
276841 tctggtagtc aggaaagcac atcaacctca attgataatg caaacttcac ggttcacgct
276901 ccagcaggta ttacagttcc aacagggatg acttatgagt ttgcaactgg agttccatct
276961 gaatctggcg tttatgtaat taaattaact ccagaaagta tcgctgctct tgaaaaaaca
277021 aatccaaact acaagctgaa tatttcttca gacgctagat ttaccttgga tgcaacttta
277081 agtattgagt ttgaagatac acaagatggt aataagcaag tcggtaagac aattactaag
277141 acaggtgttg ctaattcaac tatcaatgat cttaaattag ttgttcctga caattacgaa
277201 ttagctccag atcaagaatt accaactagt tatacttttg gtaagacatt gaatcaaaac
277261 ttgtatatca agttagttca taagttgaat gagcttaatc caactgatcc aagtactaat
277321 ccagatccaa ctaataagaa ttggttcaga gaaaatggct tagttaagga cgttactcgg
277381 acaatcaact acgaaggatt aagtacagcc caattagctc aaattccaga aactcaaaaa
277441 gctcaaactg ttgagtttac tagaactgct aagtatgact tagttactgg taagattgtt
277501 gtaaatagtg aaggttcatg gactgcagta gatggtaaag atatcttcgc tggctttact
277561 ccatttgcct ttgctggata cacagcagct cctgctaagg ttgagcaagt gaaagtaact
277621 ggtaatgata agaatagtca aattactgtt acttacactg caaacaatca aacaggtaag
277681 atatcttacg ttgatagtga tggtaaagaa gttggccaaa ccacaatcag tggtaagact
277741 ggtgaaactg tcaaggtaac gccagaagct ccaacaggct ggcaaattgt ttcaggtcaa
277801 gatattcctg aaacagtagt cgctactcca actggagtgc caacagtagt tgtgaaagta
277861 acgcatagca caattactgt tactccagaa acaccagaaa aagatattcc aacaggtcca
277921 gtaccgggcg atccaagcaa gaactacgag aagatggaaa gtttaactgc ttcaccaact
277981 cgtacgattg ttgtaactga tccaagcggt aggactacta aagtcactca aattgtaaac
278041 tttactagaa cggcaacgtt tgatgaagta acaggcgaga taacttacag tgactggaca
278101 agttctgaac cagcagaatg gtcagaatac acagcaccag aagtggcagg ctacaccgca

278161 actcaaatgg taaaatcaca agctgttagc gctgaaacta agaatgaaac agtaagtatt
278221 tcttacactg caaataacca aacaggtaag atttcttacg ttgatagtca aggcaatgaa
278281 gttggtcaaa ctgcattaac aggcaagact gatcaaaacg ttgcagtaac tccagaagca
278341 ccaacaggct ggataattgt agcaggccaa gatattccta agagtgttac agcaacagct
278401 actggtattc caacagtaac tgttagagta gagcacagta cgacaattgt tactccggaa
278461 acccctgaaa aggatattcc aacaggtcca gttccaggag atccaagcaa ggattacgaa
278521 aagatggaaa gtttaacagc ttcaccaact cgtacgattg tggtaagtga tccaagtggt
278581 aagactactg aggtaactca aactgtaaac ttcactagaa tggcaacgtt cgatgaagta
278641 acaggcgaga taacttacag taactgggca agttctgaac cagcagaatg gtcagaatac
278701 acggcaccag aagtagcagg ttacactgca acttcaagtg ttagtgcaaa accagtgact
278761 gccaaaactg cagatgaaac agtaagcatt tcttacactg caaacaacca aacaggtaag
278821 atctcttatg ttgatggcga tggtaaggaa gtcggtcaaa ccacaatatc tggccagact
278881 ggcgaaacag tcaacgtaac tccacaagtt ccatcgggtt ggaagattgt accaggtcaa
278941 gatattccta agacaataac aattagcgaa aatggtattc caacagtaac tgtaaaagta
279001 gagcatggaa cagttatcgt aactccagac acaccagaaa aggatatccc aacaggccca
279061 gttccaggag atctaagtaa gaactacgag aagatggaaa gtttaacagc tacaccaact
279121 cgtacgattg tggtaactga tccaagcggt aagactacta aggtcactca aactgtaaac
279181 ttcactagaa cggcaacgtt tgatgaagta acaggtagag ttacttacag cgattggaaa
279241 cttcaaaatt caaatgcttc taatcatgca gcccaatggg atagttacac tcctcatgtc
279301 attactcact atgttccaag cgtagctgaa gtacctgcta aagtagttaa ttcacatact
279361 gctaatagcc aagttgaaat tacttacgct cctgcttcag aaagccaagt aattcgctat
279421 gttgatcaga atggtaaaga aattagtacc caaattgttt caggtaagta cggcttagat
279481 acaacattta cagcacaatt acctaacaac tggcaagctg ctaatactat tccaacttca
279541 atcaagattg gtgaaaatgg tggtttaact acaattatcg ttgaagctaa gactgaaaaa
279601 gtacaacaat ctaagactgt tactgaaact atccattatc acactgctaa tggcaaacaa
279661 ttatttgctg ataaagaaat ggaagttaac ttctttagaa ctggtgtaaa gaatcttgta
279721 actggtgaga ttacttggaa taactggaat aaagataaag aaagccttaa tgaagttctt

279781 tctccaaaag tttctggata cacggcttct ccagctaaag ttgcagctca
aactgtaact
279841 cctaactcag aagacctagt attcaacgta atttacacta agaattcaca
aacttatcca
279901 acaattccag aaaataaacc aaataagcca caagaagaaa atgtttctaa
gcaagaaact
279961 aagactcaag ataaactcat tcacgaatat ggatataaga aacgtgcaga
tggtcgttta
280021 gtagaccata ctggtcatgt atatccagca agtagcaaag ttaaagaaaa
tggagctatc
280081 tacagtgaaa agggtgaact attatcagtt ggttcaagaa gaaaacatga
attgccgcaa
280141 actggtcttc atgacaatag tttaattgct gcaattggat cacttcttgc
tggtattagt
280201 atctttggtt tacttggtgg acggaagaaa aaagatgatg ataaataaaa
ctgaataggt
280261 tttgaaagag gcaatttgcc tcttttttt aactctcgtg caaaatagtt
cattaattgc
280321 tagactgaat gtaagagttt tagagaaaag ggtgaaaaaa tgcacggcaa
acagaatatt
280381 caaccagatg atgagattaa gcttcattgg cttctattag gtgagttatt
tacctggatt
280441 ggagctagtt ttatctggcc attgacttcg gtttatttaa ataagagatt
acacgtatct
280501 ttggcgatga ttggaattgt actgctcttt aactgtttag ctaatatgct
tggctccttt
280561 gttgcaggtt gggcgtatga tcatttcaat ccctattatt taattattgc
aggagcaggt
280621 ttagatgcct tagttttgtt tggaatggct gctaatcata cttggccgat
ttattgggtt
280681 tggatgactt tgactgggct acttggtggc tggaatgggg ctttaattaa
ttcaattgca
280741 actagcatta agtctaagcc cggcaggtat gttttcaata cgatctattt
tgcacaaaat
280801 ctaggtgtgg tacttggtac tttaatcgta ggttatattt atgattactc
tgttacgatt
280861 ttattcatta ttgcggctag tcttttcgta attgtatgta taaactcaat
tattaattat
280921 cgtccaatta ttaagtttca tttggaaaga aaagctcagc aaaatactaa
tagtaaaaat
280981 aagataaccc caatgcctag tgctaatcta aaattaacta ttggcttttt
tactactttg
281041 gctgtgatct ggctaatgta catgaactgg gagtctaatc tttcagttta
tatggtttct
281101 ttgggcattc catttcactt atatagttta ctttggacaa ttaatgccag
cattattgtg
281161 attgtacagg ctttttaag tcgttttcct aatactttta agaatttatt
ccatcaagtt
281221 gtgttcggag ttacaatgtt tgctatctct tttgtaactt taattttcgc
aaaagattat
281281 gcacattttg tattctcaat ggttactttg actttaggtg aatccactgc
gatgcccgca
281341 atgccagcct atgtaaatga tttatctcca gttgaaagta aaggaaaata
ccaaggatta

281401 acaatttcaa cgtctgcgat tggtagagcc tttggtcctt tatttggagg actagtgatt
281461 gataagttcg gctatatcaa tttctttatt gtagctgcag ctggaatctt tatgatgttg
281521 gcaattattg tgccgctcca cgcacaattg aaagataagt tgaaaatatt taggtaaact
281581 tttacatgat ttttctcttt agatgaaaac ctttacagaa tgtaagcgat ctcttctatt
281641 tgtgaaagcg ccatcttata atatagatgt gcggttaagt aaaaggagat acacaatgag
281701 aaaaatttca cctgaagttg caaaacatat ggacaatttg tctaatgaag ctggagtaat
281761 cagcgcatta gcaattgacc aaagaggatc attaaagaga atgttagctg aagcagcaaa
281821 taaacctgcc gatgaaacaa caatcgtaga ttttaaagaa gctgtttcta aagaattaac
281881 accatatgct tccgcaattt taacggatcc agaatatggt cttcctgcaa ctaaggtaag
281941 ggataagaat tgtggtttat tactatctta tgaaaagaca ggttatgaca ccactgaacc
282001 aggtagaatg tgtgatttaa ttgccgacca atcagcttta agaattaaaa atgatggtgc
282061 cgatgcagtt aaattcttgc tttactacga tccagacgaa agtgatgaaa ttaatgataa
282121 gaagaaggct ttcgttgaaa gagtaggagc tgaaactaaa gcaaacggtc ttccattttt
282181 ccttgaatta ttaacttatg atggcaagat ggatgacgtt aagagcgagg aatatgctaa
282241 agtaaagcct gaaaaagtat ttaagacaat tgaagaattt tcaaaacctc aatatgacgt
282301 tactgttctt aaggttgaaa ttccattcaa tattaagtat gtagaaggtt tcaatggcga
282361 caacaatgtt gtttatactc aagaagaagc aaagaagttg ttgaagaaac aatcagactt
282421 aactgattta ccatacatct tcttatcagc tggtgtaaca agtgaagaat tcattgccga
282481 aattaagatg gctgaagaag ctgatgcaga cttcaacggt gttctttgtg gacgtgcaac
282541 ttggaagcca gcaattaagc catttgctgc cgaaggtgaa aagcaaggta gagaatggtt
282601 atcaactgaa ggtaagaaga atatcgaaaa cttgaacgat gcacttaagg gtgctaagtc
282661 atggaaagat aagctcgaag ttgaagatta atagattagt ttaataacta atgaaaaaca
282721 aggctagaaa tttagccttg tttttttgcc ttttttttaaa ttaattgatt tttatagaaa
282781 gaatgcgata gttatgcact tgaaaagcca ttcataactc attagaataa aagagtaatg
282841 ttaattggag gagaaaaaat gaaaggatta aaatacacaa gtttagctgt ggcagcatta
282901 actgctacta ttttggcaag caccaataat ggtaaagttc aagctgcatc aactaatgaa
282961 aacgatactg ttactgtagc aaaatcagat caagaaaatg ctcaagcagc tgttaatgaa

283021 gcacagaatt cagttaataa tgctcaagca gctgttgata gcaagaagag tgcattaact
283081 gatgctcaag caaaagctaa agcaccggat aatgcttact ctgctcaaca agctaaagta
283141 aatgacagtc aaaagaatct ttctgctaag caagctatcg caaaacaagc acaagataaa
283201 ttaaatagtg tcactgaaat ttcaaagaaa gctactccag aaaatattca aaaagcacaa
283261 aagagtattg ccgctcaaca agatgcaatt aaaaatgcta agcaaaatgt tagtgatgca
283321 aatgtagatt tgaataaagc acaagctcaa ttagcaactg ctaagcaaaa acaagctgat
283381 gctcaaaatg tagttaatgc taaagaatct gctaaacaaa cagccgataa gaatgttgct
283441 aaggcagaag ctgatgttaa gaacagcggt ttagatgaag caaagcaaga cgttgaaaag
283501 gttcaaacga cattaaacga cattaaaaag accaatgcta acaatgaatc tattttaact
283561 acaaataagt ctgctttaac tgaaaacaat caaaagattg caactgttaa taataagatt
283621 gcaactgcaa atcaagctgt agtagatgcc caaaacaatg ttaatactaa acaacaagca
283681 ttggctgatg ctaaaaaggc tttaaaagat ttacaagatc aagttgctaa agatcaagca
283741 agtggtgcgg ctggtttctt taagtcaatt gccgataata aagataattc tgaaagttta
283801 agagaagatg cacgtactgc atatgatatt gttactggta agccaaatgc ttaccaagat
283861 attactgtaa gatgggttaa ccaagctgtt gagttgggtc aagaaaaaga ctctacttca
283921 ttcagtaata tggaaaaagc tctaggatat tatgaacatt ggatgacata ccgtgataag
283981 tatggcttga gtcatccatc aatttcttta actgctgttg ccattgcaat gcttagttct
284041 gattttcaac attattctga tgaatttaat caccctattt tattaactgc taaatatggt
284101 ccattttatg aagatgaaga agatatttca gctggagacg ttaatccaat tgataactgg
284161 atgagtgaaa aagaagatat cgataactat attaaggatc atccagatgc agctgcttat
284221 agttttgaaa gtagtcatcc attaactgaa gatgattggg caagagatgt tgatttctgg
284281 agtaataaac cagtagatat tggtcactat acttctatga taaaaccaga tgccaactat
284341 gttggattag ctggaaatga atatgagatt gaaccattca tggataatgc agatagcatg
284401 gatgaaattg aatttgatcc aattaacggt atggacttca atagttacca aaatctagtt
284461 cactcatatt tgcaaaacat taaacaagaa gatcaaatta acactcttaa agctaacgta
284521 gaaaccgcaa gccaaaactt aactgctgca caaaatgccg ttcaagcagc ccaaaacaat
284581 gttaagagtt tacaagctaa cttagcagaa ttaaacaatg gttctactaa gattaacaag

284641 gcaattgctg atactcaaga tgctcttgca aagggcagag aaaatctcga
atttgaacaa
284701 aagcaactag atcaagcaaa caagaattta gcagctgtac aagctaaagt
tggtgcaaag
284761 gcaaaagcat tagatgacgc aaaagctgct caagctaagg ctgccgatgc
tttagctcaa
284821 gctcaaaatg ttttagctgg agctactgct gcagtgaagt cagctcaaac
aactgttgat
284881 aatgcacaag gtaatgttaa tgccaagaac aaggatctta aggctgcaca
agcaagtcta
284941 gaatcattga agcaaacatt aagtagctta gaaaatgcta aggctaactt
agcagcagct
285001 caagcagctt taactaccgc aaacaatgat gtattaacag ctgataaaga
tgtaaaagca
285061 caagaattaa ttttatcttc tttaggagaa gccaagggta agtcagatgc
tcaagttgca
285121 agtgcagaaa aagatttgga gaaggctgag tcagatttag ctaatgaaca
aagtaaattg
285181 gctgccgcaa aagctaaatt agctgaattg aataaaaaag ccaacaacaa
tactggtaag
285241 tcagattcta aacctgaaat taataatcaa aaacctgcta aaccagcaac
taagccaggt
285301 tcagaaagtg taaatactga aaagccagaa cctgattttt caggtaataa
caaatcagat
285361 tcaaagaaag aacaaaattc tacagataat gtaaaatcag atgttaaatc
tgaaaacaag
285421 tcaaataaga ctaaggaaga atcaaaatca tcatcttctg taaagattgt
tgataatggt
285481 gacgactcag ctgcatctgt tgttttaact ggctctgata ccgcagttaa
agtttttggt
285541 gaaaagactg taaacggcac gacttactac aagatcggag caaatcactg
ggtaaaagct
285601 gataaagcac atgttgtttc tattggtggt caagcaacta ctaaacactt
gccacaaact
285661 ggtgctaaga atgaaattat tgcagcaatc tcaggcttaa cagttgcttc
agttggtatt
285721 gctagtgcaa tgggcatgag ccgtaagaaa aagaataact aatttataaa
atttaaacat
285781 ttatacagaa agagttgata aacaaatggt ttatcaactc ttttttctat
ctttaaaatc
285841 aagtattaac aataattatt gcctagcaaa acctgttcta actgaataag
catctgaaaa
285901 gatccgagtc ataatctgta aaggtagtct agaacgtact tcatagtcgg
ggaagtagtt
285961 ggttttagac atgtatccca taaaagttaa tgtcgaaagc gaagcgagtt
ggccgttggg
286021 attagtagta aaggtaataa taggaacatc gtggtgagat aagcttttag
ctgcatcaac
286081 taattcgggc gtattgccgt ttaggctaat aaaaataacc agagcgtctt
gtttaattcg
286141 cttagaggcg gttttaataa tgttaggatc agcatggaac tcgacatatt
taccagttag
286201 ttgaagttta accatgattt cttgaccaat cgattcggat aagccacgcg
caaagatata

286261 gactactttt gcttgtttgg tcatagaaat actatcttca atattgctat
agctaagatt
286321 atggagagta ttattcatct caatctcatt tttggtgatt acgctcttaa
ttttatcgtc
286381 agcgtcattt aaaacgtcaa agttgctatt agaacgatct tgcaacttta
aagattctcg
286441 ataagaagta tagccactat agcccatctt ttgcattgtt ctaacaatag
tagctgttga
286501 aacatgagcg aactggctca attttacaat tgacattttt gcaatttttt
gcggattgtt
286561 ttgaactatt tcccataaat gtagttccgc attgctaaga tcattatttt
gcatattagt
286621 acttcccctt ctgtaaaact ttaccactta ttttataaaa cgctttcaat
tcagaaaatc
286681 cttacgttca tatctaagta tactaataat gtaagcggta agaaaaggga
gaaaactcat
286741 gatttataca ataacactta atccagcgat cgatttggta attattacta
aaaaattaga
286801 atcaaatact gttaatcgaa ctgaaaattt tgagcttcaa cctaatggta
agggtgtaaa
286861 tgtatcgttt attttgaaaa aaataggtat aaaaaacatg gcgaccggaa
ttggaggagg
286921 ctttactctt gattacatta cagcaggttt agaagaaaaa ggcatcaaga
ctaagtttct
286981 taaggtgaaa gaaccaacta ggattaatgt ctttacacga gttttagatc
aaaatgtgga
287041 atacaaagaa gtaaatcctg gtccagaagt tggacaagag gttcaagata
agttcttgaa
287101 atacttaaaa gacaccttaa aagcagacga tactttaatt atttcaggta
gtttttcaag
287161 aggaattaaa gcagaatatt tggttgaaat tgcgaagatt acagcagaaa
agaaggtcaa
287221 actagtaatt gattctagtt ctaaagttgt gttagatacg cttgagtatc
agccgtattt
287281 attaaaacca aacgatcaag agctagctag tttctttgat ctgaatgaaa
aattagatca
287341 gaaaaagatt attgaattag ctcgtaagtt aattagtgct ggctgtcaaa
atgtcttggt
287401 ttcacttgga gagaatggag cagccttaat taataaagat catgcctact
ttggcaatgc
287461 accgaaaatt caagcactta atacggctgg tgctggtgat acaatgcttg
gaacctttat
287521 tggagaaaaa gaaaaagata aaagtgatac tgcagcttta aaatctgcaa
tcgcagcggg
287581 tagcgataca gctagccgat ctgggttaac agattttaaa ttagaaaaat
atttaaacga
287641 aattcaagta agtgaaatta agtagtagga aggatgaaat aagatggctc
aatatgattt
287701 agttggggca acgggatgtg ctactggagt tgcccatacc tttatggctg
aagaagcttt
287761 agaaaaagct gcccaaaaat taggctataa gatcaaaatt gaaacacacg
ggcaaactgg
287821 tgttgaacac gaacttacac cagaagaaat aaaagaggca aaaggtgttg
ttattgcttc

287881 tgatgttgac gttgacccag atcggttcgc tggaaaaaga gtggtaattg ttccagttgc
287941 caaaggtatt aaagaacctg aaactttaat tagagaggca ttaagtttgg acacgccaat
288001 ttataagcca gggcaaacgg ctaaaagttc tactggaaaa agtagtgctg gtgctgagaa
288061 attaggtact aaaatttata cttcattaat gaatggtgtt tcccacatgt tgccatttgt
288121 tgtggctggt ggtgttttaa ttgctgtttc attcttctgg ggtatttatt cagctgaccc
288181 taagagcagt cagtataatc aatttgccta catgctaaat acaattggta gcacaacaat
288241 gggattgatg gttccaattt taggtgcttt tattgcagaa gctttagcta aaagatcagg
288301 gctagttgtt ggttttgcgg ccgggatgat tactagcgtt ggtggtggtg gcttcttagg
288361 tgcgatcatt ggtggttatt tagctgctgt tgtagtactt ttattgcaaa aattaatgaa
288421 accattgcca gacaaagaat ttagaggatt aaaatcaatt ttcttattgc ctgtattagg
288481 ggtattcatc actggtgcga ttatgttctg gttgaatgga ccaattaagg caattaatac
288541 aggaatgatg tcttggctaa aaggttttga aaattcaagt ccaattcttt taggtattat
288601 cattggtgta atgtgtgcct ctgactttgg tggaccaatt aacaaggctg cttatgtaac
288661 tggtactgca cttcttgctc aaggtaatta ctacttcatg gcaggtgtat ctgctgcatg
288721 tattgcaccg cctctagcaa ctggttttgc agttttgtta aacaagaaag catattctaa
288781 gaacgagcgt actgctggat atgttaactt cttattagga tcaacgcaca ttacagaagg
288841 tgcaattcca tttgctgcta aacacccatt atggaatatt ccagcattta tggtcggttc
288901 cgcaattgca gcagctttga cttatgtttc tagaattcaa gttccagctc ctcatggtgg
288961 ttttattatt ttgccactcg ttaacaagcc atttctatgg gtattatgga tcgtagtcgg
289021 tgcactagtt tccggaattt tactagcttt aattgccggt agatttgcta agacagaacc
289081 acaatttgaa gatggtccag atattgatgt ctttggtgaa gaagaaaatg ttaacaaacc
289141 agtagctgaa aaagaaacta ctgactataa ccctaaagat attttggata ctcaaaatat
289201 taaaattaat gttgatgttc cagataaaga ttcagcacta aagtatttag ctaacttagc
289261 tgtaaagaat ggcttagctt ctgatagcga ggaagtttat gataaatatt tagctagaga
289321 aaaggaaagc cctacaggga tgactgatgg ttttgcaatt ccacatgcac aatctactgc
289381 tattaagcaa tctgccatgc ttgttttgaa gttgaaaaat ccgattgatt ggaattcctt
289441 agatgggcaa aagattgata cggtgatttc tttcttaatt ccagcaaagg atagtaagac

289501 tcacttacag tacttatcta atacagctaa gttgcttact cataaagact ttattgaaaa
289561 attgaagcaa gctaacacgc cagaagggat taagaagtta tttgataagg aatagaaagt
289621 tggattttat aattatttaa gttttagacg tttaattatc aaaaaaatta gaaagcctat
289681 taaacctaaa ctcattccta gtaaagttgg ttttaaactt gaattgagtt taggtttttt
289741 cttatctttt tttgtcgaat gttttatatt gcgtgttgct tttttagtag aagaattatg
289801 tgttgttttt ttagatgaat tagagcgtga agcattagaa taagaagatg tacttttgtt
289861 taaagatgat tggtagttag ttgaatgttt ataattttct tctttagttt tattaggatc
289921 ataacctaga tatttttgga ataaataaag agcatgcgag ctgggattat aaccagctct
289981 atcagaagga aggggatgtc ctagaaaaga catataagaa tgataaagat catgaagatt
290041 tacattacct tcatcgtcca cttgtaagcc atcttcatta agtttaggat tagctgtttt
290101 attgctattt tcattatttt ttatgttatc tatagcttgt tgagtttttt tgtaggtttc
290161 agaattggtt ttaaccattg tccatttaat ttctgttact ccacttttat ttttccaatt
290221 tactggaatc atagctgaag gattgcctac aaatgcgtaa ccatcaatat gaacaggatt
290281 aataccatcg gcatcaaaca ttcgctgagg actatttgta tatccaactt gagtagctaa
290341 taaaaatctt cctagatatt tatctgtatc ttgatcataa actttcgcat atagtttttg
290401 tacactacgt tcatgcgttg aggaagattg aatttttgaa ttaatatttg tatcttcagc
290461 atagactata ttattggaaa atatacatag aaaaactgta aataaaattg aggttaaact
290521 aaacaatttt ttgctgtatg tttgaagtaa taaattattt cttttcataa tatttttctc
290581 cattattttt acctatctct attatctagg agtagaaata aaaagactag aaaagaataa
290641 acaaatgtta gattttaata atagactaaa aattttaata atgatatcat ctatataacc
290701 acttatgaat tacaaggagg aattaattaa tggcattatt caatgtacgt ggcggtgctg
290761 gtgatatcac taagccagat cttaacgcaa gaccacaaga taatctttac ttagcagtga
290821 actctgaatg gattgaaaaa gcaaagattc cttcagaccg ttcaagaatt agctcatttg
290881 atggcattga cttgaatatt gagaagagct taatgcaaga ctttgctgat tttgcagatg
290941 gtaaaaagaa attgccagaa gttcctaatt ttgaaaaggc agttgaactt tataagatcg
291001 ctaataattt cgataaaaga aacgaagatg gtgcgaagcc aattaaggct gaccttgaag
291061 aaattactag tttgcgcaac ttagctgatt tgaacttaag agcagctgat ttctataaga

291121 atgcctttga cttgccaatt agcgtttctg ttgatgccga catgaaggat acaaaagttc
291181 acgttgttta ctttggcgga cctggtttgt tcttgcctga tacaactact tatgacaatc
291241 ctgcagctgc tgatttatta aaggtattgc aagaccaatc tgaaaacttg ttgaagatgg
291301 ctggtgtaag tgaagcggat gctaagaagt atgtcgaaga tgcattgaag tttgataaga
291361 aactttctaa agttcttaag tcaactgaag aatgggcaga ttatccagca atgtacaacc
291421 caatgtcttt ggctgaattt gaagcaaaat ttgatagctt taagattgat aacttcttaa
291481 ctagtttatt tgctcaaaaa cctgaaaagg ttatcgtaac tgaaccaaga ttcttagatc
291541 atgttgaaga attgatcaac gaagataatt ttgacgaaat taagggatgg atggttgtta
291601 agttcattaa cggcgtagct gcatatcttt ctcaagactt ccgtgaagct gcctttccat
291661 ttagtcaagc tttatcaggt caacctgaat tatcttctgg aactaagcaa gcttaccgtt
291721 tagctaatgg tatgttcagt gaagtagttg gtgtttacta tggtcgaact tactttggtg
291781 cagaagctaa ggctgatgtt gaagatatga ttcataagat gatcgatgtt tatgaaaagc
291841 gtatctctga aaatgactgg ctatctgaag acactaagaa gaaggcaatc gttaagttac
291901 gtgctttaat tttgaagatt ggttatccag aaaaaattga aaaaatttat gatcgtcttc
291961 aagtaactcc tgaatctgaa ggtggcagtc tttactcaaa tgaaagtgcc gctgctgttg
292021 aatcagttaa gtacaacctt gaaaaattaa acgaaccagt tgatagaact gtttggttaa
292081 tgccaggtaa cttagtcaat gcttgttatg atccacaaag aaacgacatt actttcccag
292141 ctgcaatttt gcaaaagcca ttctatgact taaagcaatc acgtagcttg aattacggtg
292201 gtattggtgt ggttatcgcc catgaaattt ctcacgcttt tgacaacaat ggtgctaagt
292261 ttgatgaatt cggtaactta aagaactggt ggactgatga agactttgct gaatttaaga
292321 agcgtacaca agatgaaatt gacttgttta acggcattga atatggtcca gttaccttaa
292381 acggtaagca aattgtttca gaaaatattg ctgaccaagg tggtttaact gctgctgttg
292441 aagctaataa gggcgaaaat ggcaacatga aggaagtctt tgaaaacttt gctcgtgttt
292501 gggcaaacaa gcaacttcct gaatcaatta agacgcaagt ctcagttgac gttcacgctc
292561 ctggtcctga aagagctaat gttcaatcac aatgccaaga agaattctac aaggcatttg
292621 atgtgaagcc agaagatggt atgtggcttg atccagaaaa acgtgttgta atttggtagg
292681 aattaaataa gaattattaa gaatcaaatg gtaaaaagct gtttgattct tttttcatag

292741 tttgcaaaaa aatttcgtta ataatattat gaattggtat tcttattacc
taagacaata
292801 ttcttaatta gaaaaggtta aaaatgttag aaaaaataaa aactaataag
ttctgccaat
292861 ttatttggcc atatctatta attgctttgg ccactacaat cgttatctcg
ccacaattaa
292921 tttcgcatac tattctagct agcgatgata cagcagatac tttgtttcac
tatagtcgtt
292981 tttatgatgc aggaatgcag ttaaaaactg gaatttttag tatctttcaa
actaatttta
293041 ctttccagca aagcggcaga attattaatg cgatttacgg gccattattt
gcctacttta
293101 atggaatttt agttcttatt gctggtaatt ggtttaacta ccaagtcatt
ttagcctatg
293161 tcatttcgct attaggcgct ggtagtatgt attacttact tcaaaaagta
ggtattaata
293221 agctactagc tacagtaatg ggaattattt acattaatat cgggatgatt
ccagccttta
293281 ttaaccgtag ttcatttaac ggttggggac aagcattaat gccgttagtt
gtgttatgcg
293341 gcgtacgcat gattcaagat agagaaaagc caattaactg gattcagtta
atgttagtaa
293401 tgagtttgtt agtgcaggtt catgttttaa gtaccttaat ggcagctctt
ttattacttc
293461 ctttctttat ctattcctta attattaatc aaaattctaa aaagatatgg
ataccttttta
293521 ttaaggctgt tgtcggaaca attgtactat cagctaatgt cattggggcc
tttttagttt
293581 taatgccgaa taatcaccta gctgcaaata ctgaatttga tctatctcgt
ggtggcttac
293641 gtccacatgt gctatggcat aatgaatatg gcacagcctt tgcctatatt
ttgccaattt
293701 ttgttttatt aattattgcg caatttgtct atgttgtaat gcactacaaa
aaagataaat
293761 taaatacttt cgtgactatt tggggtacgg tcttactagg attatcttca
tttgtttttc
293821 cttggggatt ggttcaaagt atttttccaa gtttaaagtc atacttccaa
tttccgttta
293881 gattaactgt tgttgcctat ccactaattc tgctagggat gacgctaact
gttaatcatt
293941 tactaaagca aggagttaag ccaagggcag ttggaattag tttagtagta
gttcttcttt
294001 tagaagctgc tttgccgaat atcattacta atcacatctt tactgttgct
tgtggaaaag
294061 atgcgcaagt tcaaacagta gctaaagcat caaacagtga tcagatgctt
aagatattga
294121 aatggcacta cttgcctgat tatttaccaa ctaaagacaa gtctattgac
caaaacgcag
294181 cttatcttta caaaataaat gtagttgatc aatatcaaaa atttaatcat
aaagttgcta
294241 aaaatggtga tttgattctt tcttggaaag gaaaaaagga taaagatatt
gttttgcccg
294301 ttgttttata caagcaaagt gaattaactg tgaacagtaa aaaattcact
ggtgcaaaaa

294361 acgtaattgg aaatccaatt atttctcaaa aatcaggtct aaatcgtgct gttttacact
294421 ttattgctcc ggcttggttc tatgtagtca gcgcaatttc gttaatagca tgccttagcg
294481 tactcgtatt ttacttgtgg aagaaattta ttaataataa aaacaataaa ataaaagttg
294541 cttaatcata tacaaaagta gtcttttaat aagatagtct tattaagagg ctgctttttt
294601 ttgtatggtt ttacttacta ttaatatcaa tttatttttt gattattaca tggctatagc
294661 tgtcttgtta tgttatgatt taattgtaat cattgaaacc gctttctatg gtttcagagg
294721 aggaaaaata agttgagtac acaagaacgt tattctcatg atgagttaag aaaagctaac
294781 aaaaaattta gtcggattcg ttctactatt gaatctgcct tttatggcaa caatgtacac
294841 gaagtaacta gtgttgctga agcttataat ttagctaaga aacaatctgg tgttatcgaa
294901 actgacctac caattttaca tactaaggaa ctaggtttgc cacaaggagc aaagcagtta
294961 gtttataacc acggtaaaat tttgggtaga acagcaagtg cacgtcactt tgttgatgat
295021 ccaaaagaag atccagaggc gttagcaggt aacttacgtg aagatattta taagggccac
295081 gatcaaaaat tcttaaaggc aacagttcta gtgggattag acccaagctt tactgttaag
295141 gcacatatta tgatgccaga ggatcaagcc tttaacttac tttcatatat tttgaatttc
295201 cacttctttg atgaagaagc agagaagatt tacaagaatt ctaaagtata tgacgaagga
295261 gatatttact tctacttcga tccaaatact caagatgaag attatcctaa aggctttggc
295321 gtctttgacg caccacataa ctgtgctgca gtctttggct tacgttactt tggtgaattg
295381 aagaagggta ctttaactct tgcttgggct atggctcaca gaaatggtta tactgcttgt
295441 cacggtggtg aaaagtcatt ccactttaaa gataaggacg ataaagtatt tgccttctat
295501 ggcttatctg gttcaggtaa atctactttg actcatgaaa tgtatgatgg taaatatgat
295561 atcaccgttt tacacgatga cgcatttatt atttctcgtg aagatggttc ttcagtagca
295621 ttggagcctt catactttga taagactcac gactatccag caggtcacca cgagaccaag
295681 tactacacta caattatgaa ctgtgcagtt actcaagatg aagatggtaa gaaagttatt
295741 gtaactgagg acttacgtaa caacaatggt cgtgtcatta agactcgtta cacttcacca
295801 catagagttg actatgaaga tgcaccaatt actgctttgt tctggattat gaaggatgga
295861 tcactaccac caatcttaaa agttgatgat ccagttttag caacaacaat gggacttact
295921 ttagctacta agagaactag tgctgaaaac ttaccaaagg gctttgacat gaatactttg

295981 gtaattgaac cattcgcaga cccattccgt gcttatccag tatctggtga
ctactcagac
296041 ttcaaggaat tattcactaa gcgtggagct tcttgttaca tcttgaacac
cgatgccttt
296101 atgggtaagg acattccaaa ggaagtaact aagaagctag tagaagatct
tgctaatgac
296161 agtatcaaag atagtgattg gaagccattt ggtaacttca agggtgtatc
ttacttgcca
296221 attgaaggtt atgaagttca cctagaagat cctgaatacc aaaagacctt
agctaagaga
296281 atgcaagacc gtttagactg gttgaacaaa tatgatgaag aacatcctac
tcaaccaatc
296341 caagaagaag ctaagaagac tttggaaggt atcattaaag aattaaatta
gttaattgaa
296401 aaaatcccct atcaatttct atattagaac attgaaaaac gcatcccaat
tgggatgcgt
296461 tttttatatg tctatatcta tttattactc tttgagttcg ccttcagctc
gaagtctctt
296521 tttgatatct tcaaatttct ttggtgcgat gcgagtaata tgttgtcttc
gttgatctcc
296581 taagatcaat ggtacccgtt caataactgt atcagcataa tctaagccaa
accatgaagc
296641 tgggttagaa gatagatttt gaagccatac acgtgcttca actaagaatt
tttcataact
296701 tctaagttgt gtttgtggac taattacatc gttaacgatg acgaatgaaa
aatcaccaac
296761 atctctacca ggtgtagtag tgtattcctg cggctgtgat tcaatcgtgc
catcagcaat
296821 tagatcatgt acgatttgac gtaagtatac attcacacta gtttgctgct
tgaatcctag
296881 gaagagctga acgttaatta cgttcttagt tccgtaagta ttaacggcgt
attcagcagt
296941 aaatggctca ttagtaacgt taactgttac gaaccagtat gctcgtgcac
gttttggacg
297001 tttatctaag atagagtaga gaatttctcg cttaatgaat ttattgtatt
taactttggc
297061 catgtaaacg aggttagtag caaaagtagg gtaatcttca tcatgactca
agtttgttaa
297121 catatctaca tattcatcaa gccttacata agcattgcgc gattcacgtt
tgtctctgat
297181 cttattacca tagaaccaaa catacataat gattccgatg aaaccggcaa
taattaatga
297241 tacgtaacca ccatgtgtaa atttactcaa gctggccaac ataaacatga
tgtctaagaa
297301 accgaagaaa attaagaaga tccagttcca gattgtacga atgcccttca
ttgctaacca
297361 ttcgtaaagc aagatcgttg tcattaacat tgaaatagta attgacaaac
cataggctgc
297421 ttccatgtga gcggaagttt ggaagaaaag aacaatcgca attgttgcaa
tacagatcat
297481 cttattaaca gaaggaatat aaatctgtcc ctttttagtt gaaggataca
taatattcat
297541 tcttggtaag aacttcaatc cactagcttc agctactaaa gtaaatgaac
cagtaattaa

297601 agcttgtgag gcaataacgg cggcaatagt agctaagaca atggcagcta gacgaatgtt
297661 ttgtgggatg atttcaaaga atgggttgaa atcaccgttt ccagcgttat agttaggatt
297721 atttagaatc caaacgcctt gaccaaagta attgagtgat aaacaaacaa agacatatgg
297781 ccaagaaccg ataatattcc ctttaccaac gtgaccaacg tcagaatata atgcttctgc
297841 accagtagta gcaaggaaga tggatcctaa aataaagatt ccaaccttgt tatatggact
297901 aaatagaagc ttaattgcgt aataagggtt aagagcttgt aagatagata agtcacccat
297961 catattcatc aagcccataa cacctaaaaa ggtaaaccag ataaacataa ttggtccaaa
298021 agctttacca ataatacttg ttcccattcg ctgaatcgaa aacagaatca gcaaaattac
298081 taccgtaatc gcaataacag ctccctgcgt agaaactgga acgttgccgt gaccaaattc
298141 aattcctttt aacccttcaa tagctgttgt aacagtaaca gcaggagtta atgtaccatc
298201 agctaaaatt gcagctccac caattaaagc gggaagcact aaccattttg cacgtttacg
298261 tactaaagta tagagagcaa aaataccacc ttcaccgtgg ttagtagcct ttaatgcaat
298321 aaagacagtc tgcaaagttg ttaataaagt aactgtccaa agtactagag aaatagatcc
298381 gacaatgaag tcacgattta cgtgagcaat cccaccattt ccggcgacaa tcgacttcat
298441 aacatataac ggactggttc caatatcacc atagacaata ccaatcgcaa ttaataatcc
298501 cgcggccgat atatttttcc gtgtttttc attcatttt attctcccaa taaatagcca
298561 aaatgactaa atatctaaat aacgtaaaaa aagaatgcca aaaaagggac attcttcttt
298621 aaacatatct attttcatct atttatgcaa gagttgcaag tatctatcgg agaataattt
298681 aaaataatta tcttagtttg gttctgtgaa tagatttaac atcttgcgaa cctagaatta
298741 atggtacgcg ttcaattact gtatcggcat attctaatcc aaaccaaaca gcaggattag
298801 aggataagtt ttgaagccaa acccggcttt caacgagcca cttttcataa gagcttagtt
298861 gagtttgagg actaattacg tcatttacaa ttacaaactt aaagtcacca acatctctac
298921 ctggagtagt agtgtattct tgtggttgcg attcaattgt gccatcttta attaagtcat
298981 ggacaatctg acgtaagtaa acattaactt tttgctgctg cttaaatcct aagtatagtt
299041 gaacgttaat tacgtttttg gtgccatagg tattaactgc atattcagca gtaaaaggtt
299101 cgttagttac gtttacagtt acaaaccaat aagcatgagc tcgcttggga cgcttatcta
299161 agattgaata cagcatgtca cgcttaataa acttgttgta tttaactttg gccatgtaga

299221 ccaagtttgt tgcatatagc ggaatacttt cgtcatggct tagattgctg agcatagtag
299281 tatattcgtc taagcgaaca taggcatttt cggcttcacg tttatcacga actttattac
299341 caaaatacca aacatacata attgccccaa taaaggctgc aataattacc gtgacataac
299401 caccatgtaa gaatttagct aaactagaaa tcaagaacat ggtttcaatt gccccgaata
299461 aaagcaaaaa gattagtcgc aaactaaagt taacttttct aagtgatagg tattcaaaga
299521 gcagaactgt cgtcatgagc atggttacag tgattgctaa accataagcc gcttccatgt
299581 gttcagaggt tctaaataag aagacaattc cgattgtagc agcgcaaagc atcttattaa
299641 ttgacggaat gaaaagctgt ccctgttcag tagaaggata aataattttc attctcggta
299701 aaaacttcaa accactagct tcagaaacta gggtaaagga gccggtaatt aaagcttgcg
299761 aggcaatgat tgcggctaga gtagctagaa ttattgcaaa gaacttccac tggcttggca
299821 atgcttcaaa gaatgggtta aagtctgtgg caccagcatg ataattaggg ttttctaaga
299881 tccaaacgcc ttgaccaaga tagtttaggg ctaagcaaat aaaaacgtaa ggccaagaac
299941 ccataatatt acctttacca acgtgtccaa catctgaata aagggcttct gcaccagtag
300001 tagctaagaa tactgctcct agaattaaaa caccgacttt attagcggga ctaaataaaa
300061 ttttgatagc taaaagtgga ttgagtgctt caagtaagga ccagtcatga cttaagttca
300121 taattccagt taatcctaag aaggtaaacc agaccagcat gattggccca aatgtcttac
300181 cgataacgct cgttcccatt ctttggatag agaatagaaa aagcaagatg acaatcgtga
300241 tcataatgac actattttga tttggcactg gaatatcgtt accaaatttc atatttttaa
300301 gaccttcaat agcggttgta acagtaactg ctggtgttaa agtgccatca gccagtaaag
300361 cagctcctcc aactaaggca gggataacta gccatttagc ctttttttcta actaaagcat
300421 aaagagagaa aattccacct tcaccatggt tagttgcttt aagggcgatt aaaacatact
300481 taacagtggt taaaagtgta atcgtccaaa gaataagtga gatggaacct acaatatatt
300541 cgcggctaac atgggcaatt ccaccatttt cgtttacgat tgctttcata acataaagtg
300601 gacttgtacc aatgtcgccg taaacaattc caattgaaac taaaagtccc gctaaggtga
300661 ctttttttacg taaattacta ttcataatga gctttcctct taaaaagtat taaataacat
300721 tttgaataaa caaaaaagaa tgtattgtaa taatacattc tttttatttt cgttagcact
300781 attttggctc tttggaacct aatatgcaaa taataaattg tgattttttt atgaaagagt

300841 ttgtttgatt tcgttacgac gatcagtgta ccatttgttc caatcttcgt aagtcttcat
300901 attttgtgga tctacgccag tttgcttttg aatattgtta agcatatcca taaagcgttc
300961 tgcatgaata tttaatacgt gcttgactaa gttttgacgg ctgaaggcca tgttgttagt
301021 taagtaagtg ttaatttcaa taacgtcatg attaccatcg taaacatcca taatttcaag
301081 atcgctttcg tcgaattttg aaatatagaa gttagcaaat gtctttaagt aatcaggttg
301141 ttctttaaat tcagtagctg gcatttctac acgatttgcc tctggcataa ctacttcaaa
301201 ttcttctttt tcttgagttt cttcggtgtt cttcttgtct tcggtcaaag ttaagagctc
301261 ctttactgtt tagctgtaca aaaattcaat ataatcttac attgaattac aagcaaaagc
301321 aaattttgta gggtttattg gaagttttct acatcttgtt tagtgtatga atcaattgta
301381 gtgtgaccat tattagcagc tgatctcatg aagttaacta ctttttggtc agaccaagag
301441 ctagtatcaa caccaagtga acttaattgg ttacgagttt gtgcaattac tcgaccagta
301501 actgtttggc catcgatcat catgccacta tttgagtttg atgaagaatt agtatttgag
301561 ctattgttag cattactatt tgaagtggta gcatgggatg attgtttgct tgctgcagag
301621 ttgttttgag tgttagcagt atttgaatta ctttggttag agctagaact agtattggta
301681 ttactactat tagatgaatt agattttgaa ctagtagatt tgtgtgaagt agttgcttca
301741 tcgtcatcat tagcatctaa attatcagta tcaatatcgt cgttaacgtc ttcgccattt
301801 aatttagcat tagcaacttt acgtaaatgc tttacatttt tctttacttt tttgtagtac
301861 ttaccattaa tgtaagataa atctaaaatt tgatcgcaag attcaactgt tgcttgatag
301921 ttaccatcac gatagcttaa tttagcttgc ttgtataaag gattgatatc gtgagtgata
301981 tggtcttgta ccttgttaat cgtcttataa agcttagcag cttgcttagt cattacagaa
302041 tagccattag ttttgttagc tgccttgtct aattcaccta aagcatcttt aaattcgtac
302101 tctctgattt cttcttgagc atccttcata gcgcctgctt gatcggcata ggcttttgat
302161 tcagaagttg cttttacatc gtgagcatct tcaaattttt cttcagctgc ttcgtacttt
302221 tgactagagg cagcatcttt tccttgactc atataagtag tgtacttgtc tgagccatta
302281 ttagatgagc tatttgatga attagaacat ccaactgcga ataaggtaat aaagaatgcc
302341 ataatggcaa ctccgatttt tgatcgtttc attgtttctt cctcctataa aaatgaattt
302401 atcttactta aattatacca aatttaactc ataaaaaatt cattgaaaaa attggcacaa

302461 atctttgtgg taaatcgtta gtaatagtgt aaggcaattc accggtgatg acttacaaaa
302521 tatatttagt tactaattag attatcagag gtagaaaaat ggaatttgtt ttaaaagatc
302581 aatcagttca atttactttt gcaggtgaaa aatacagcaa tggtaagaaa aacagaattt
302641 tgaagaacgt taaaaagcaa gctagtgctg aagacgtgat gaaagttggc aaggctttgt
302701 ctaaattgca aaaagatgat ggtattaaaa gtgctagatt aatttcttat tcagatattc
302761 aagcttaaga aatgaaagaa taatatatag aaaattttaa aggagaattc aaaatgacta
302821 ctactaaaac tttaagactt acatttaaaa atgctaaaaa taaaaaagta aacttatcat
302881 tacctgatgc agcagaaaat ttaaccgaag aagaggttaa aactgccatg aacgaaatct
302941 gtgaagctaa cttgtttgtc aaggaagaag ttgatcaata tcaggctcct ttatctgctc
303001 aatatgttga acgtacagtt acttctgtgt ttgatgatag tgaaaagaat tagagcaata
303061 ttttatgaga taagggtctt aattaagtta ttaaataaac tcttgaaagg gtaatgtttc
303121 ggcattacct tttttatttt gagacgaaag tatgtaattt atgttacttt tttatttcag
303181 aatgtctgtt cggttgtatc tgacaacttg ccacactaca ataaagagtg tatgtaaatt
303241 ggagggagct taagcaatga aaaaaagaac attcactggt attgctaccg cggcacttat
303301 cacaactgcc ggtatatcag ttaccaacaa ccttaaacca gaaaatccat taaaaactgg
303361 tgaaggtact gttcaagctg caacatatca acaagaattt ttaaacaaag ctattccagc
303421 tgctacaact gcatcatcta agtatggtac ttatacttca gttatgcttg ctcaagcagc
303481 ggtcgaatca gcttggggtc aatcaggttt ggcacaatcg cctaataata acttattcgg
303541 tattaagggc tcttacaatg ggcaatcagt aaatatgaat actggtgagt acggcagcaa
303601 tggttattac actactaacg ctggatttag gaagtatcca tcatatactg aatcatttga
303661 agataatggc tctttattac gtaaccaaat gggtaactat tactctggta cgtgggtaga
303721 aaattcaaag aactacgctc aagcaactca aaatggttta caaggtaagt atgcaactgc
303781 gccaaactat gctcagacac ttaatagtgt tattgcagct aatggatttg ataagtacga
303841 tcccgttacc caagttgtta acgaaaatcg tacagttgca caaaccacac caattatgag
303901 tgccccagtt gatgctagcg ttggtactca agttggtact gcaagaactg gtcaaaacgt
303961 aaatgttact aagtacatca cttataacaa tggtgttaag cgtgcatata ttggtactgg
304021 ctggattaac gcacttgcat ttagtccaat tactactaat acaactacta agcaaaatac

304081 tgccgctaat actaataatc aagcaagtca agcagttaag acgccagtag
cacaaactca
304141 acaagttaag agtcaagcac ctgcagctcc agtaaaaact gcaactgtaa
aagtaaagag
304201 tgccgctgaa gttaaaactc cagttcaagc taccacttta aacgttaaaa
ctgaaaataa
304261 agcagcacaa cctgtaaagc aattagctgt ttcattagct gttgcaccta
agaaggttga
304321 agttaagaag aacgttgtga aagctgaacc agctaagaca actccagtaa
aaactgaaac
304381 tactaaaact gaaaacaaag aagttaagac tgcaactcca gtagttaaaa
ctactgatac
304441 agttaaaaaa gttgaaactc cagcagttaa accagttgaa tctgtaaaga
aggaatcaac
304501 tccagttgtt aagactgctc cagttactgt aactaaagaa gctgcaaaga
ccaccgaagc
304561 tccagtagtt aaacctaaca aggttgaagt taaggaaatt aagacaactc
cagtaactac
304621 tagtgctaaa actgttgtaa agcctgttca aagttaccaa agtgcagtaa
ctactgctgc
304681 aaacaataac tatggtactc aatggattag tactaatact gctcctaagg
ttgcatctac
304741 tgttttaatt aaggttacta agactgttga tgttttatca gcaccagatg
gtcaaaaatt
304801 agaccaacaa gttgaagctg gctcagaatt tgttgttatt gcttctaagt
actacaatgg
304861 caacttatat tacgaaatta gtaatggtaa atggattatg gcaaaatata
ctactcaaga
304921 agcacaaatt actgcaaaat ctggtgtatt aactattaat tctaagccag
actatggtgt
304981 accagtatgg cgtgttccag gtcaagatca aattgctggt aagttcttga
aagacggctc
305041 aagctggaga tacttccgtg tagcaaacgt tcaaggacaa acttggtatg
accttggcgg
305101 aaatcaatgg gtatctgcca agtctgtact agttcgttaa tgtataaatt
gggatagaat
305161 ttaattcatt aaaatttttt ggaggagaag tttttaatgc gttcaacaaa
taaaaaattt
305221 attagtgcat tggcttgtgc tagtgctatg acagctttgg caattgctga
tccaatgggt
305281 attactaaga cccatcaagt agtacaggcc gcaactgata atgtacctgc
aaaatctgca
305341 ggtgtggatg tttcaagctg gcaaggtact aatttgactc aacaagctaa
gtcaggtgct
305401 aaatttgctg tagttaaagt atctgaaagt acaaactacc aaaacccaca
tgctcaaggt
305461 caaattaata atgctgaaca aaacaatatg atgccaatgg gttaccacta
tgctcacttt
305521 ggtgctgata gcaacagagc tgttcaagaa ggtaattatg ctgttaattc
agctcaacaa
305581 gctggtttgc cacaaggttc atatttagca gctgactggg aacaagatgc
acataacaac
305641 actaacggtg gtcgtgaagc aagtgctaac gcaattacta gctttatgga
tactgttcac

305701 aatggtggct ataacccaat gctttattca agtgaatggt tattaaaggc taaggttgat
305761 actaacaagg ttactcaaaa gtatccaaat tcattatggg tagcttctta taaaactatg
305821 ggtcgtcaag atcaacctga ctataactac ttcccatcaa tgaataatgt tgctatttgg
305881 cagtatactc aaaattggcg tggtcaaaac gttgacggaa atgttaacgt actccctctt
305941 tcaaataaga gtaacaacgc tagccaagct aacaccaaca ctttgaacgt tcatactaat
306001 ggccaaagca gtagtcaagc cccttcgaat ccatttgtta atacttctaa caaggcagtt
306061 aatactaact gggttaaaca agaaggtact ttcactactg gtggtgcaat taacttgaga
306121 acaggtgcaa gtactaatag tagtatcatt gctcaacttc cagcaaacag tgaagttaaa
306181 tacgatgctt atagcactag gggtaactac acatggttaa gacagccacg tgctaacaac
306241 caatatggct acttagtggg acgcgcaaat ggtcaagatt ggggtacttt taaagttgct
306301 ccagttacta accatgtaac taaaccaact actaacactg ttaagccagc taacaagcca
306361 agtcaaccag tacacgtaaa tacaaacaat aatgtaaaca ataactggac taagcaaaac
306421 ggtgtattta ttaccggtgg cgcaattaac ttaagaacag gtgcaagcac taatagtaag
306481 gtaattgcat tattaccaac caatactgaa attaaatacg atgcttaccg tactgaaggc
306541 caatatacat ggttgagaca accacgtgca aatggtcaat acggttattt agtaggacgt
306601 gataatggtc gtgcatgggg cactttcaaa gcaggctctg ctgttgcaac taaaccagca
306661 actaataata agccagctca aaaaccagca actaacaata gtgactggac taagcaaaac
306721 ggtgtattta tcacaggcgg cgcaattaac ttaagaatag gtgcaagcac taacagcaag
306781 gtaattgcat tacttccaac aaacactgaa attaagtacg atgcttaccg tactactggt
306841 caatacacat ggttgagaca accacgtgct aacggtcaat acggttactt agtaggacgt
306901 aacaacggca atgcatgggg tactttcaaa gcaggttctg ctgcagcaag taagccagca
306961 acaaataata agcctgcaca aaacactaac aagccaagtc aaccagtaca cactaataca
307021 agcaataatg taaatagtaa ctggactaag caaaatggcg tatttatcac aggcggcgca
307081 atcaacttaa gaacaggtgc aagcactaac agtaaggtaa ttgcattatt gccaacaaat
307141 actgaaatta aatacgatgc ttaccgtact gaaggccaat atacatggtt gagacaacca
307201 cgtgccaacg gtcaatacgg ctatttagta ggtcgtttaa acaaccaagc atggggaact
307261 tacagatagt tttcaaaaat aaatgaataa cttgaaaaga catgatccga ataggaatca

307321 tgtctttttt gtcttataat aaattatgaa aaaagtatgg aggacatagt
tttgatttat
307381 acagtaactt tagatcctag cgaaagtgta gtaggtaagg gaattaatat
ttcacttgtt
307441 ttaaagaaat taggtattga atcgattgca actggtattg tgaatcataa
aaacgtagaa
307501 caagttgcag aagaacttga taaagcagag attgaaaatc aatttattga
acaaaagcgt
307561 ggaattaaga tcaccgctaa aaatcaacaa aagttattag attatttgaa
agtcttaaaa
307621 gccggcgaca ttttagttat tgccggtggt tttgccaaag gaattgatcc
agtttactta
307681 accgatcttg ctaaagtagc tgataaaaag gcagcaggct tagtagtgga
tgttccttat
307741 gaaaacgtac ttgatatctt accaatgaat cctttattga ttaagccaaa
tgaaacggaa
307801 ttaaagcatt ggtttgaaaa agataatcaa aaggtaacta cgaaagaatt
aattaatatg
307861 gcgcatgact tagtagttaa gggtgcacaa catgttcttt tgtctttggg
tgcaaacggc
307921 gctgcaattg ttaatatgat ggatgcactg atggcacatg cacctgaaat
tgaagaagta
307981 gttgatagta cgggtagtgg ggatgcactc cttggtactt tccttgctgg
gatgctaaaa
308041 ggatacatgc cagttaaaaa tttgtcagat gcaattgcgg caggaagcga
cacagctcga
308101 agcgactggc ttactgattt tagaactaca ccagcacttc aaaagcaggt
aattgcaaga
308161 cgtattgcct ttgaagaagc agaataaaga aaataagttg gctttttagg
gccgacttat
308221 tttttgatgc tttttttagt ataatgacag taattttata ataagggaga
agaaaagtgg
308281 acttatctct atttattgtt agtctagctg ctctagttat tccaattatt
atggcacggt
308341 tcaagatcaa tgctattcca actgccgttg ctgaaattgt aactggaata
attttaggta
308401 agagcttatt aaatacaatt aaaattacca gtagcgtatc acttctatcg
aatttaggcg
308461 tgatattact gatgtttcta tctggaatgg aaatcaattt tgacttattc
aagaaaaaag
308521 atttacctga tactaagcaa agtagcatta atccagctcg gatagcagtt
ttttctttcg
308581 caatgattac agctatggca cttattttag ctgaggcatt aaaattaatc
ggactcttta
308641 gcgatgtaat gctagcaatg atcatcttta tgactgtagc tttaggagtg
gttattgcaa
308701 ccttaaaaga aaaagaaatc ttatctcgtc caattggaca aacaatttta
ctaacagcag
308761 ttttaggaga agtaatacca ttactgcttt taactatcta tgcttcgatt
aatggtggta
308821 atgcaggaaa attatggtta atcattttat tattcctagt ggcaattctt
ctactaaaaa
308881 gatttaaaca gccatatcag tggtttaacc gaatatctaa atctactacg
caattggata

308941 ttagactagc ctttttctta attttcactt tagtgacagt tgccgaacga gtaggtgctg
309001 aaaatattct tggagccttc ttagccggaa tggttatgaa attacttgaa ccaagcgaag
309061 caacgatgga taaattaact tctattggtt atggcttttt tattccaatt ttctttatta
309121 caactggtgt taagctagat cttaagactt taatcactaa tccgaatgcc ttaatgttaa
309181 ttccagtttt ggttttattc ctgcttttat caaagctgcc aatcttttta gtttatgttc
309241 gtagctttaa taagcgtaat agtattgcag gaacattttt aattatgact acgattacga
309301 tcgtgcttcc aactttagaa gttgctcgta agttaaatgc aattacagaa actcaatcag
309361 atgcctttat tttagcagcc gttgtggtat gtattttagg accaattttg tttaactcaa
309421 tctttaagct gactaaagaa gataagatta agcaaagagt tgttattatg ggaacaaatg
309481 tgatgactgt accggttgcg caggaattac atgataactg gtacgacgtt cttttagtaa
309541 ctcataagca agaaaattat gatacttata agagtaaggt cgctaattta aagttgattt
309601 caagtcttga agaaacttgt ttagaaaaag ccggcgtctt taactgtgat attttggtcg
309661 ctggatttat tgaagatgaa ttgaaccgta aaattgctcg aatggctaaa gaacatggcg
309721 ttcaaagagt aattgcaagt caagagcgac caaaaccaga aacaattaag aacttaacca
309781 atgaaaagat tgaaatctat aatctcttta atgttcaaac ttcagtttta cgtgctttga
309841 ttgaatcgcc atctattcta agaattctaa ctgatactaa gaatggtctt tttgaagtta
309901 cggttagaaa tcataaatat gctggtcaaa agttgatgaa tcttgacttt attgaacaaa
309961 tgacggtaag tcgaatttgg agaaatggta cttggttagt accacatggt aatacgatta
310021 ttgaaactgg tgatcatttg atctttacag ctaaaggtga agatgcagaa agagtacgag
310081 aagaactagg aagaaaaaat taataaaata aaagggcagg attttaattc ctgtcctttt
310141 ggttaacgtt gacttttagc ttcttgtaaa acagataagg tatttaaact atggacaaag
310201 gcgtgatcag cttctttgaa cttatgctga ccaataattt tagtaaattc aataaattca
310261 gccaccattc ggtgagaata cttattgttg ttgataactt taacttcttg tccttttaat
310321 tgcattccga ctttaggtat ttcattagga tgaccataaa taataagtgc accttttca
310381 ccttcaatag tgctgacatt aggtgaaaaa ctgtctttag cggcgattga actagcttgt
310441 ttgtcagaat aactgaggtg caggatgcct gaggtgtcga tatttttctg cattgtaggg
310501 aaataattga tagattctgg tttaccaaat aacttgataa taatatgcaa attgtaaatt

310561 cctagatcca tcaaagcgcc accgtctttt ttaggatcaa aggctggttg aatatctcca
310621 gctagaaagg catcataacg actagaatat tgagtgtaat taaggttaac tacgtgaatt
310681 ggagcaattt tatctagatt ttcttcgata aatttaaagt tttccaaata aatgttagtt
310741 atggcttcaa tgatgatcag cttcttttga tcagcaatta cttttaattc tcttgcttca
310801 tcaacagttg caacaaaagg cttttcgcaa ataacatttt tacctgcttc aagggcttct
310861 tttgaaatag aaaagtgcaa actgtttggt accgctacat aaacagtatc aacatcagga
310921 tcattaaata agtcagcatt attatcgtac aatttagtaa tctgatattt tttttgtaaa
310981 tctagcccaa tctgatgact tctcttagta gttgcaagag ctgtaagttc gagatcggaa
311041 atttgatcag caattgttag aaaatcatgg acaatttttc cagaaccaat aatacctaat
311101 ttcataagtt tctcctttaa tttgatttaa tatttagttt ttttgcattt ttagtaaccg
311161 aggttggaat ctttagtttc ttaacttggt taacatcgta taaatgaagt gataaggtgc
311221 cttctttttt actatagtaa gtaactttgt agtagagatc ggttagttct ttttggcggt
311281 taaccttaac tcgaagttca attcttttta cattacttga ctgcgggtca cttgtggaag
311341 ctactgaagt ggctttttgc atgcttttaa tgacatcttg atcatcgcca tggtaagcaa
311401 ttacatagcc attgaagcca ttagatttga gttttaactg cttaaaagca tcatcagaga
311461 attgcttgaa gttatgagct gtatagagtt ttttgtagcc agagtaaact tcattaaaga
311521 ttgcattttg cttaatataa ttccacgctt cattattatc acgatggtag acatagtgat
311581 cgttagacca gagctcggaa tttgtatctg attttggaat acttttcatt gtgatatgaa
311641 ttgtattatt aggaccaacg acgtaatttg agtacatttt gccttttgaa ttggtagttt
311701 taacatcaac acgagctgtt ttgatagcag tatctgctgc tttagttaag gcagctttag
311761 gactaggctt gccaaaggcg aagatggcac cccaaataat tactactaaa ataataatgg
311821 ctaaaattaa agcggcaatt ttgccgcctt gattgttagg aaaaataatc tttttatttt
311881 ttgctttatt tttctgcatt agtttttctt tctaaaatga ttattgattg ttttgctcag
311941 cttgagcttt cttaatctct tcagcgtcaa tcgcattctt ctgaatatca gcaggaactg
312001 ctaagtcggc atgttggcca agttgatcat aagttaagtt ccaagtgaag ttatattttc
312061 caccagcagt gtattgagcc ttgaaagtca tcgactcaat ctttttttgtt gcaggatcaa
312121 ctaaataagt aatttttaag ttctgtactt gagctgattt aactagacga gctacttgca

312181 tgttttgtga acctggagtg ttcatagcgt caatgacaag tggattgact gcattccata
312241 ggttagaatc agtaccgtta aagctgattt catagttgcc atttttcttt tgaacagtag
312301 ctttttagc aagtgcttta ttgattttct taaaagttgc aggatcaaat ctttctttta
312361 cttgatctgg atcaaaagta tcggcattaa ccgaattttt aatccagtgc cctttgtttt
312421 gctctagaag aaggtacatg tctttttag ttaaccacat ttgttccgtt tgtgattttt
312481 tcttttgagt caaggtgtaa gtaagattcg ttacatcgcc tttatcttta aaaagacctt
312541 ccgattttga tttttgactc atttcatcag aattaattgt ttgagtgaaa tgaccgctgg
312601 ctaagctaga attaaatttg gcattaatta gtgaacttgc agaaggctca ggattagttt
312661 ttgatttact ggtattcttt gaacaagctg tagttataga aactaaaatt gctcccaata
312721 aaagtagcaa gccgaatttt ttcttcataa aatattcccc ccaataaatg tatctatcgt
312781 atactatttt actctatttt ctgttgaaaa aagtattata agagcttaac tttagtgctt
312841 tttaaggaca tttaaaatat ttcgtaactt actacccttg acttgcaaat ttttaactgc
312901 ttgtgttact tcgatgcaac caatatattt tccatcttcg ttgtggagac tatagaaaga
312961 aatatttatc ggctgttcat ctttagtgat cataattgaa atatttttac gattacctgc
313021 gtgcatttgg tttaaaactt ctttaacgtg tctttggcta tggccgggat gcacctcaaa
313081 aacagttttg ccaatatctt tttctgttcg cttgaagagg cgatttttat tcattgatga
313141 ccagacaatg cggtcatttt catctaaaac gtccatttct tgtggaatag ttctaaaaat
313201 tgcatttagc tgttcaagtg agagatgacc tccgtctaaa ttgatattat ccataggtaa
313261 ttcttccaat ctatccaaat taatatatct attgttattt taacattaaa aaattgtgga
313321 ttaataaagc tttttttcgt aattcctatg aaaatttctc ataagcgtgt aaaatagtta
313381 gtgaaacatt gaattttaaa ggagatacag ttcatgtcga aattagtttt aattcgtcac
313441 ggtcaaagtg aatggaacct ttctaaccaa tttactggtt gggttgatgt aaacctttca
313501 gaaaaaggtg ttgaagaagc taagaaggct ggtcgtttaa ttaaggaaca cggtcttgaa
313561 tttgatcaag cttacacttc attattaact cgtgctatca agactttgca ctacgcactt
313621 gaagaaagtg accaactttg gattccagaa actaagactt ggagattaaa tgaacgtcat
313681 tacggtgctc ttcaaggttt aaacaagaaa gctactgctg aaaaatatgg tgacgaacaa
313741 gttcacattt ggcgtcgttc atacgatgtt ttgccaccag ctattgatga tgacaacgaa

313801 tttagtcaag cacatgaccg tcgttacgca aacttggatc cacacatcgt tcctaaggca
313861 gaaaacttac acgtatgtct tgaccgtgta atgccattct gggaagatca cattgctcca
313921 gatttacttg acggcaagaa cgttattatt gctgcacacg gtaactcact tcgtgcttta
313981 actaagtaca ttgaaaacat ctcagatgat gacatcatga acttagaaat gaagactggt
314041 gaaccagttg tttacacatt tgacgataag ttagatgttg ttaacaagga aaagcttgac
314101 gactaatttt tagttattga gtattaaaaa actccgacga aagtcggagt ttttttttata
314161 tagtctacaa acatgtgttt gataaaagta taaaagttat atataattag ctttaataaa
314221 agaatttatt tgattttagc gatagattcc tttagactac taatagaaag atcaatccct
314281 tcgctttaat ggatacgtcc tatcgcgaag gtttttttt acataaattt atgaaaaaac
314341 atgatatttc cttaagatat attgaccaat tagcactatt taaaatgaga ggcgtagatg
314401 gtatctcatt atctgttcat agagcagaag aggaaaaacg taaattatta aataagcaat
314461 taaaaacatt aagtacgatt gggtactata atttgaaaaa ttatgctaat ccatattttg
314521 attcaagtga aggtaagtat aaaaatttat cttttgataa gctggtagct cgatactatc
314581 gggataagca tttaaagcag gcagtattac atgcgattga agatatagaa acaacattaa
314641 atactaagat tgcaagcgta ttaggtgaaa gatatggagc gtatggttat acggatttta
314701 gactatggtg tcaaagaaat tcagtgaata agtttttacg tggaaaatat atggataaat
314761 ttgcaattca aaaggaacaa ataaattttt taaaagagtt gcaaaatatt gtaaggaaat
314821 cctactctga ggatttgcaa gactatatta aaaataccga aaaaatatat cccccaattt
314881 ggttggtaat gaatgagttg acgcttggaa ctagtattca tctatataag ctaatgagca
314941 aaagtaatca acgtagaatc tcatcatatt ttggatgtaa gacggatgaa ttagttagtt
315001 ggttagaaag tattaatctt attcgaaata tttgttgtca taatggcata ctagctgatt
315061 ttaaacttaa aactagagca aaggttccaa aagaatataa atctagttcg gaattaaaaa
315121 cagtgttact taaaattaaa ccagaagttt atactaatcg tttagccttt cagttatgca
315181 taatagtaaa gttgatgtca aaaattaata ataattatac atatagagat ttaagaaatt
315241 ctgtaaaaaa gctcttagat gaatctacac cagctacgta ttacgggttt caaaatcaag
315301 aagctattca aaagttattt aaagtaaaaa tactaaaaga taatagtagt ttgattttat
315361 attaaatata atctttgatt tactaacaaa gattaatttc ttaaggctaa acaaaaaaat

315421 aagaaaaaag cctcccattt gggaggctat ttaagtatga ctattatatt ttgtatttaa
315481 gttttgcttt aactaagagc tagtcttata atttttacca actagcctta cgtacaccag
315541 gaagttgtcc tttatgtgct agttctttga agcgtaaacg tgacatacca aacttgcgca
315601 tataaccatg tggtctacca tcgtgtaaat cacggttgtg gtagtgagtt ggatgagcat
315661 caagtggcaa tttcgctaat gcttctacgt caccagcttc ttttaattcg tagtattttt
315721 taattaattc gcgctgctta gcagctttaa caatttttga tttttttgcc atatatcttt
315781 cctttcgaat atagaatttg aatgtgataa agattaacta taataatttg agctaactta
315841 aagtaagctt attgtattaa attattaata atttgtcaaa aagaaagtat tttaaaaaga
315901 attagtttaa aataagtaat gattgattaa agaagggaaa agaagccgtg ggtattttta
315961 aaagaatact tcataatgaa gacttgcgac agctgattat ttatgtatta attggtgtgt
316021 taggattagg tgttgatttt ggaattttcg cactccttac ccattttaag atgcaagtcg
316081 aagtagctaa ttttatttca tcatcttgtg ggttaatcaa taactttttc tggaatagtt
316141 ttcttaactt taaggttcac gataagttgt taataagatt cgtttcttac tatttagttg
316201 gacaaattac gactttattt actacattat gtttatttat cttcgtaact cagttaggtt
316261 ataatcaact aattgttaag gccgtttcta catttatcgc taccttaatt caattcgtaa
316321 ttaataagtt acttacattt agaaaaatta aaactactaa gtcaaaagta gacgttagaa
316381 agtaatcaga aagcctgtta aagtatgaaa aaattgtcaa ttattgttcc gtgttataac
316441 gaagaggaat ctgtaccact tttttatcca gctgtgaata aagtaatgga tacaattcca
316501 gatttagagc cagaatattg gttcattaat gatggttcaa aagataatac tcttaaggaa
316561 attaaagaat tacgtaaaaa agatccagaa catgttcact ttgtctcatt ttcaagaaac
316621 tttggtaagg agtctgcgct ttatgctgga cttcaagctg caactggaga ttatgtagtc
316681 gtaatggacg ttgacttaca agatccacct aagtttttac cacaaatgta tgatttgatt
316741 aagactggag aatatgactg tattggaact cgtcgtgttg accgtacagg ggaagctaag
316801 tttaagtctt tcttaagcga tatgttctat aaggttgtta ataagatttc tgatactgaa
316861 attgtaccag gtgctcgtga ctaccgaatg atgactcgtc aaatggtaaa tgcggtttta
316921 gatatgcctg aatacaatcg tttttcaaag ggaatcttct cttgggttgg atttaagact
316981 aagtatttgg actatcacaa tgttgaacgt gtagctggcg aaagtgattg gaacacttgg

317041 aaattgttca aatatgcaat ggacggaatt gcggactttt cacaagcacc acttaactta
317101 gctgtttgga ttggtactgg atcatttgtt ttatcaatta ttggcttgat cattgtgatt
317161 attcgcagag cactctaccc tggttcaagc atctttggtt gggcttcgat ggtatgcatt
317221 attttgctgc tgggtggttt gcaactactc tgcattggta ttttaggtaa atatatcggc
317281 agggtatata tccaagttaa gaatcgacca atttatatta ttaaagagaa aaaataaaca
317341 tggcaaaaag tttcggaatt atcacctaaa ttccgaaact ttttttgtga gattataaat
317401 ttgaaaagtg agataatctt aacgagggag aattttagaa tgaaaaactt tagtcaaaat
317461 acgtggataa aaattgcttt atctattctt ggagttttgg ctgcaatttt ggtttatgtg
317521 cctaactacg cagcagttga tcagggactt aagggtagat gtgcaatttt ctttattatt
317581 gcagtggtct tactctggct accaatggaa ttaaaaagca gtatttttgc tttgaatttc
317641 tattatgaat taggattgat tttaattatt gtctttttaa tgactaatca aatggcaatg
317701 aatttcgtta ttggtctagt tattgctttc cttttattaa cctggattgg gatggtaatt
317761 tttaaaaata atcttgtaat gaaacaaaag aatagtgaat tgttaattat taggttagtg
317821 atgatgctgg ctttagccta tttggcctat atcagcggtt ttgcggcact aatgggtgct
317881 gaaattttta ctgtacaagg acaaacttgg ctacttattt taggatttct actttgtgta
317941 tactatctag ttttagcagg aagtatttgg cagccttggt ttagaagatg gacttcttgg
318001 atcatgattt tagcggcagt tgtcctgcac atgatctttg caagtgcaac ctcgttaaat
318061 attatctttt tgccaataat cggtgaaata attatgctaa tttcagtctg gcgtagcaat
318121 cggatcttaa tgaaaaagaa aaggatttca taattgactc ttgccagaca gtattaatcc
318181 ctgatagaat aaattgttta aaagtttagg gagagataca aaattatgaa atatgtttat
318241 ttgtttttac cagcaattgg ctggggactg atgccccttg ttattgctag tgtaaagaat
318301 agtacagttt ataatcaaat cgttggtaca gttgcagctt catttatttt tggtgcaatt
318361 gtaatggcaa ttatgcatcc agcaatgagc tggtcactat ttttgctttc tgcattaggt
318421 ggtgcttgct gggtaattgg tcaagttggt caatacattt cttatgaaaa aattggtgtt
318481 tctgaaacaa tgccgatttc aactggtttg cagttgattg gtgtacctct tgtaggtgtg
318541 cttgcttttg gtgaatgggc tagtccgcaa gctaagtttt atggctttat tggtattcta
318601 gtattaatta ttggagttgt tttaacttca ttaactgatc gcggaacaag cgaaggtaat

318661 aagtctaatc agataagtac aattattttg cttgttttaa cttcacttgg ctacattact
318721 tcaagctcaa ttccaaaggt tttacatgga agcagtattt caatcttctt tggtcaaacc
318781 tttggtatgc ttgtagctgt gtttatctat acgcttgtta ctaaaaattt acatgtttgg
318841 aaagaaaaat ctactgttca aagtggtgga gcaggtattc tatatgcaat tgctgcttta
318901 gcttacattc tttccgttca agataacgga gttaacatgg cttttgttat ttcacaactt
318961 tgcgtagtta tttctacttt aggtggcttg attttcttac atgaaaagaa aactcgcaac
319021 ggtttgattt ttaccattgc aggcctagtt ttaattattg gcggtgcaat gttaactaca
319081 ttattctaga aatgaaaaaa ggcatcccag agggatgcct tctttgtatc aacttgtcct
319141 aattcttaaa tatttttagt attagaacac ttttaattgt attagaacca aattaaaagt
319201 caagttaaat acaatagagg tttaaaaggt atattgtacg taaaaattaa ataacaaatt
319261 aagaaataaa gaatccaata ataaactata tgttatcgct tacagttgac atattgagtg
319321 tggcgtgata ttttcagaat aggatcggtc gaaattgttt aggaggacaa tggatgctaa
319381 caattgaaaa caaccagtta aaggttgaaa ttaatgaggt cggtgctcag cttacgcacg
319441 tagtcgataa gactactaat gccgactaca tttggaatgg gagcgagtgg gaaagacatg
319501 ctccaattct ttttccggca atcggaagat caaatgacaa taaatatatt ttaaatggga
319561 aaacatatga aatgaagcaa catggcttcg ctcgtgacta tccttggaca gttgttgata
319621 agggagacga tcgagtaagt ctgactttaa ctgaaaatga agaaactcta gctgtttatc
319681 catttcattt tagtctgatg gttacctata cgctagaagc taatcaatta aaggtagaat
319741 ttttggttaa aaataattca gaagaaacga tgccatttgg acttggtttt cacccaggct
319801 ttaatgtgcc aatagctgga gatgaattga aattttcaga ttatgagtta actttaagcc
319861 cagaagtgac aaaattaact caatttcaaa ttgatccaat tccatttaga aatggtaagg
319921 tcattccagt acctaatgca caaaagggaa ctttgccgct atcgtattca gaatttgatg
319981 atggtttgat tattattaat aatccaggtt taaccgggat tgagttaagt agtgccaaat
320041 cagaacataa aatttcttta acgttggaag atttcccata tgtagcgcta tggaccatga
320101 ctgatccaga agctaagttt ttatgtatgg aacctttttgc tggcttgcca gatattaaga
320161 gcagtgaatt gaccgactgg atggaaaaag aaggcaataa cttcctagca ccagatgaaa
320221 gttcacattt ttcaactaca attactttag aataaatatg aaaagaggga agctgtgtga

320281 agttttcctc ttttttttact aattgtttaa gatttggcat aataaagttt tcagagtgta
320341 attttgctat aatatccttg ttttaagaaa gtttgagaaa gaatattatg gataataagc
320401 ccaaagaacc tgttctctca agagttgagc tgaacaaaga aaaacatagt aagaaaaagc
320461 caatccgtat tattattgcg gttattttag tgctggtttt aggacttgga gcctatgcag
320521 gatctgttta tttaaaagct aaaaatgcct ttgataaaac atatgaccca aagactgctg
320581 taaaacaaga tagcttttct ggtaaagaac cattcaatat tctcttgtta ggtacagata
320641 cgggagcttt tggtagaaaa gaagttcgcg ggaactcaga tacaatgatt ttggtaaccg
320701 ttaatccggc taaaaagaaa ctttctctaa tgtcgattcc gcgtgacacc atggcaagaa
320761 tgattggcac cgaaaacttt agcgttcata agattaatgc cgcttataat attgacggtg
320821 ccaaaatggc aatgaagact acaagtaagg tccttaatgt gccaataaaa tactatattt
320881 caatgaatat gggcggaatg agaaagattg tagacggcgt tggtggcgtt acagtgacgc
320941 cgcctcttac ctttacttac gatggctaca cctttaccaa aggtaaaaag gtacacttaa
321001 atggtagtca agccttagct tactctcgaa tgcgctatga cgatcctcgc ggagactatg
321061 gtagacaatt acggcagcgt gaagtaatta tgtccgtttt agaacatgca atgtcattta
321121 gtactctaaa gaatttagat tcaatcttag gctctgtttc aactagtttg agaactaact
321181 taacttttga ttcaatggtc aaaattagta aaaactaccg taaatgtact caaaacatgt
321241 ccagcgacta tttgcatgga gtaggagcaa tgattggaga tgcttcatat caagtaatgt
321301 ctgatagaga attgcaacga acatctaata ttgtgagaaa tgatcttggt ttagattcaa
321361 tgaatattga taataatgaa acttatcaaa attcaatgaa ttctcaattt gattggacca
321421 gtggtgattc taatcaggta tactacatat atgatcctta tacggatgaa ttatggaacg
321481 gagatcgtgc ttattaatga atgaattaat ttcactagta ataggatatt tcctcggaaa
321541 tatccttttt gctatgattg tcgcaaaaat atttttacat aaagatccaa ctaagtatgg
321601 ttcaggtaat cctggtacag ctaatattgg tgcagttttt ggtaaaaaat ggggtattct
321661 cacttgtatt ggagacttag ctaaaacttt ggcagcatta ttaattgtct actttcttta
321721 tcatggtaat cgcttagatt tatcttttgc tggtattgga gtcgtcttag ggcattcatt
321781 tccgttctgg aatcacttca agggtggtaa aggagtggca gttactgcat tgtggattgt
321841 ctttttgac tggcgagctg gtttgattgc tttattaatt ggcttgtttt tagtgattat

321901 tatgaaaaat cttactattc cgccacttgt atatatgttg gggttcagta ttttacttg
322961 gcttaacttt ggctgggaac aaggattaat ctttttaatt gctactttaa tcatgatttt
322021 tcaatttaga aaagatattg tcgacttctt tactggacac ggtaagcgag tagatgtttt
322081 ggtaacaatt aagaaaaagt tagggatata caaatgaaat cagctttaag aaaaacaatc
322141 cagtggatct tacttttgtg tttgttgctg ggaatattaa ttcaaacctt aggcttttgg
322201 aattataatc caacgtcagt ttctactaaa acgcgaattg gcatggtaat tagcttaatt
322261 caattgatag tggttgtctg gtatgggatg agttacggca ataaggagta tagctttaaa
322321 gaggcagtta agaactggct tgaaggcgtc gttactctta ttattttcta tttagtattt
322381 gtcatttcgt tgccacagtt tttctcagct tggaatttat ggggaatctt tttcccagtt
322441 ttaactagta catcagcttt atttagcgga attataattt ctttattctt tcaaccattt
322501 atttttagat tacaagagaa attaaatact aagcaaaatg tgttgctgct gacagccatt
322561 acagttttga tttttgcctt aagtgcagga aattcgcttt taactagtta tagtattttt
322621 ggcctttact tagctgtacc ttttgcctgg ggaatgctta tttcaaagat taaggcatct
322681 aagaaagtag ttcttggctt agtagtagca acgattattt tgctaccagc agtttattat
322741 cttacgatta aattaatgcc gattcagacg ccgcaaggat tcatcttttc tcagatgaat
322801 atgtcttgga atactagtct tttaatggcg ccatcatcac cattgatgat tttatttgtc
322861 gttgctggag cattattatt tagaagttca atgcttggag tatctcacag attattttca
322921 attctaattc cagcaattat ctttggcact acttcttatg ggatgagttt gtggaaggaa
322981 aaattgcaac ttttgcttgc acctgtaagt aagaaagtta ctgttttatt gattctttct
323041 ttattagttg caagtttcat tattaatttt gtttttgtta agtttctact ttcaaataag
323101 catgttcaga gatttttgaa taagtttgat gaaaataatc tggatggtct tattaaactt
323161 ttagaagcgg gagtagactt tttaaagcga cactctaaat caattatctt atttgcattc
323221 ctgatgttct taagtgtcat tggcttttat actgttcgag acattcaatc tgcaagtgac
323281 ttttgggctg ctctagtctt tatttttact agcaaatttg gcaccttagt tttatcgtcg
323341 attttcttat ttgcaattta tgagatcttc tatgtaatca ctactaggtt ttgggtatcc
323401 gcaagtattc caactgtttt ggctttagga attgcaattg cagatggaat taagatggat
323461 ttaagagaag agcctgtcta tccaaatgaa attagtgaga tagtcaattg gaaaacgtta

323521 attccaatga ttggtgtgca gactctaatt tacattcttg ttggtattgc acttttaatt
323581 gcaattattg tttatctaga gctaaagcac ccacataact taagaagaaa gaaaaagagt
323641 tggcttgttt taattggtag tctcttaatt ttgattacac cggtgtggtt taatgatgaa
323701 aattcagcga tttactatat ttcaaagggt tttgataata atcctgactt tagaaatcca
323761 ccggatagta cagctaataa cggtgctgtc ttaactttct tagatttcat caaagtgcca
323821 attatggaaa aagttgatgg ttattctgaa catgccatca agcaaattac taaaaagtat
323881 gaaaaagaag ctattgctat taataagact agaaagaaca agttatccga tcaaacaatt
323941 gtctttaatc ttagtgaaag ctttgtagat ccaaaagaat tcccaggagt caaaatttct
324001 gataatgtta gagatccgat gaagtatatc cgtagcttaa tgtcgcaaac gacttctggc
324061 aagatgctta gtgctggata tggtggtgga actggaaata tggaatatga gtccttgact
324121 ggatttaata tgggaaactt ctcgagtgct cttactccat atactcaggt aacttcacgt
324181 tataattttt acccaacaat tgggatgaat ttcccttata gtagcgcaat tcatccgttt
324241 aatggtactt attatggccg aattgataat tatcgccggt ttaaatttaa taagtttgct
324301 tatttaggct ctaaatataa gatctatgat aaaaagtcgc ttggaacgag tccatatctt
324361 tctgatgaga cggcttacca gaatggcttg agacaaatta agagtagaaa aaatggtcaa
324421 tttattaact tgatctcaat gcaaaaccat atgccatatg gtgattatta ttcaccaaat
324481 gaatataagg ataatgtcag tggctcttct cttgcagatg ataatgttaa gactagcttt
324541 gcggcatata caaagggtgt tgaatatact gataaggcag tgaagaagtt tattaaagaa
324601 atcgatgaaa ttaataagcc aatcacgctt gtcttttatg gcgatcatta tccatcaatt
324661 attgatcaat cgttattatc taagtatcca attaaaatgc attctattac ttactttatt
324721 tattcaaata agtacgctcg tgagcatggt gctaagaata aaattgttcc agataagtat
324781 gttgcaacga gttcctttat tccgatggct ttagagcaaa cgaattctaa agttacagct
324841 taccaagcat tacttactcg gatctacaag gatcttcctg caatgacaat taactatagt
324901 agcagtgatg gttttgaact agttgatcaa aatggtaaga aagtttctga aaagaaatta
324961 actaagaagc aaaaagaact acttaaagac tatcaattga ttcaatatga tatgtcagca
325021 gggaagggat acacattaga cgtcaagggc ttttataaat aagcggttga ctaaaaggct
325081 attctccctt attatcatag gtaaataaaa gtagctactt aattcagtgt tcagagaact

325141 agcggttagt gtgagctaga caggttaagc aagcgaagta cgtaatgggg tagtttcctt
325201 atcaactgac ttaaggcgca ttgagcgcaa acgtgggtgg taccacggct aatgaagtaa
325261 tttagtcgtc cctagatttc gagagaagtc tagggacgtt ttttgttagg agaaattatt
325321 atggcaaaat ttaacatttt agaagattta aaatggcgcg gtgctattaa ccaagaaacc
325381 gatgaggaag gtttacgcaa gtacttagca gagcatgatg atttagctct ttactgcgga
325441 actgatccaa ctggtgattc ccttcatatt ggacacttaa ttccatttat gattttaaaa
325501 agattccaaa tggctggcta ccatccagta attttgattg gtggaggaac tggttcaatt
325561 ggtgacccat caggtagaaa gaccgaacgt gttcttcaaa ctgctgaaca agttaagcat
325621 aatgaggaaa agttgactgc tcaaatgaaa aagttattcg gtacggaaaa ctttgaaatt
325681 aaaaacaatg ctgattggct tggaaagctt aatttgattg atttcctacg tgactatggt
325741 aaattttcc aagttaataa tatgattaac aaggatgttg ttgctagccg cttagagaat
325801 ggtatttcat ttactgaatt tagttaccaa atcttgcaag caattgattt ttaccacttg
325861 aacaaagata atggcgttca actccaaatt ggtggttctg atcaatgggg caatattact
325921 gctggtattg acttgattca caagcttgaa ggtcaagatc atccagcatt tggtttaact
325981 attccattga tgcttaaggc tgatggtact aagtttggta aatctgctgg tggtgctgtg
326041 tggcttgatc cagaaaagac tagtccttat gaattctacc aattctggat taaccaagat
326101 gatcgggacg ttgttaagta ccttaaatac tttactttct tatctcgtga agaaattgaa
326161 gacttagccg aaaagactga aaaagaacct tggaagcgtg cagctcaaaa gagattagca
326221 gaagaagtaa ctaagtttgt tcatggtgaa gaaggcttaa aagaagctca aatgattact
326281 gaagcactat tctcaggtaa cgttaagagc ttgtctgttc ctcaaattga acaagctttg
326341 aagaacgcac caagtgcaga agcaactcat gaagctaaga atattgttga attcttagtt
326401 gaaactaaga ttgaaccatc taagcgtcaa gcacgtgaag atgttaagaa cggtgctatt
326461 tacgtaaatg gtgaacgtca aaatgatatt gacttcatta ttgaaccaga tagtgatttt
326521 gatggtaagt atgtaattat ccgtaagggt aagagaaagt acaccttagt taaaattaaa
326581 tagttgataa aaaagaagac gagggatgct agaggcactc ctcgtctttt ttgttataat
326641 taaactgtgt atgccgtttt agctcaggag gtagagcacc gccgtggtaa ggaggaggtc
326701 cccagttcaa atctgggaaa cggcttagtt attaaataag cttaggctag cagaagcttt

326761 tttgatggat gatgcattaa gggatggtca aaacgactgt ctctttttat aatggagata
326821 aaatattgta atttatgaaa gaagaataga catggcacat aaacttacac tagaagaaat
326881 tgaaaaattt caacaagatt atcaacaaaa caaacaaaat aagatcgctg aattagctgt
326941 tgtgaataac ggcgtgcaaa aagccagctt caacagtgaa ggaattcgtg atttaaatcg
327001 caccttctca attgaaattc caactgataa tgtgactgat caaaaacaat caggtcgttg
327061 ctggttgttt gcggcattga acgttttacg tcacaagttc gctaagcagt atcatgcaaa
327121 gaactttact ttttcacaaa gctatctctt tttctgggat cgaatcgaaa gagcaaatat
327181 tttcttcaat catatcttag aaactgcgga taaacctgtg ggtgatcgaa ctgtgcattt
327241 ttatttgcaa gctccagata ctgatggtgg acaatggcat atggctatct ccttgattag
327301 aaaatatggc ttagtgccaa cttatgcaca agatgaaagt tttactgcta acaatacagc
327361 agcgtttaat caagccttga acatgaagtt acgtgaagat ggactagttt tacgtaaact
327421 agcacaagaa ggcaaaaatg atgaaattga gcaaaaacgc caagaatttt taagtgaagt
327481 ttaccgtatg gctgtgattg cctttggaca acctgtgcaa aagtttgatt tagaatttaa
327541 agatgatgat ggaaaatata agttagatca aaatcttagt ccactagact ttttccacaa
327601 ttattttgaa gatgatttgg atgactatgt tgttctcttt aatgcgccag atcatgaata
327661 tgacaagctt tatgcttttc catttgaaga taatgttgaa ggcggtagtc caattagatt
327721 tttgaatacg aagattgaaa atcttaagga agcagctatt aagcaattaa aagatggaga
327781 aacgatctgg tttggttgtg atgtaggtaa gcaaagcgac cgtcaaaagg gaattttagc
327841 tgctgattta tatgaaacag atactgtttt taatattgaa actaaattaa gtaagaagga
327901 gcgtcttgaa actggtgcta gtggctctac tcatgcgatg acttttgttg gagtagatgt
327961 agttgatggc aaacctcgtc aatggaaagt tgaaaattct tggggaacca aagttggtga
328021 aaaaggctac tttgtaatgg acgataagtg gtttgacgaa tacctcttta aggttgtagt
328081 gaagaagcaa tacttacctg aaaaattagt aaagattgct gaaggagaag ctactccagt
328141 tccttgctgg gattcaatgg cttaattttg gtgataaaca gatggatatt gaattagtta
328201 agcagtttac ccaagatcat ttaaaaggtg aaaaaacagg acatgactac tatcatggac
328261 aacgagtagc aaacttagct acaaaaatgt atttaagtga ttatcccgat gcgcatgcag
328321 atagccgagt tgttgctatt attcagaccg gtagttactt gcacgataca attgatgaga

328381 aaatttgcga tgacccaagt aaggtaattg cagaaattaa agagttacta
cctaaagttg
328441 gatttactga gttagaagca caggatattt tatttacgat tcagcacatg
tctttttccg
328501 ccaatattga acatcactac aagttgccct tgagtggaca atatgtgcaa
gatgcagatc
328561 gaatcgaaag cttaggtgca atcggaatcg cacgagcatt tacttatggc
ggaaagcatg
328621 gaaataagat ctatgatcca gagattaagc ctgaaaagtt agtaagtcat
gaccaatatc
328681 gtaaccatgt tgagacaacg attaatcact tctatgaaaa attatttgat
ttagagggat
328741 tgatgaatac tcctgctgct aagaaagaag cacatcgaag aactgaatat
atgcgtgaat
328801 tcgttcaaga atttatggat gaatggaatg tgtaagataa taaagctagc
taatcttttg
328861 gctagcttta ttattcactt aattaagggg atgaagtcat gtcttatatt
gcagattata
328921 ctaatagtta tgacattcat ggacaacgaa ttacaattac tgcgcctgct
cgttttgacg
328981 agaatactgg taaggtaatt gctgacttag aacttgatga tcatgcagca
aaattagctt
329041 tggaaaagta tcggagaaag tttggtatta tttcaccaac tgatcttaaa
aatttacgac
329101 atcattggaa ttttagtaaa gaaaaatttg ctgaagtttt gggatggaat
gtggcaacaa
329161 tagctcttta tgaagccgga gctgtgccat cgattagtga taatcgttta
ctaaagattt
329221 taattaaaaa tccaaaactt atggaaaagt ttattgaaga tagtgaaaaa
gaagaattat
329281 aaaagggatc tgtgtctata gcgacgcaaa tccctttta tcaatatatt
ttattttcac
329341 taagcaagtg aatcccaagc tggaagtgga gtaggcttgc tttctgcaag
tttcttttga
329401 tcgtcagtta agaattcttt tctaacaact acttcataaa caaactcatc
aaaccactta
329461 tcgctcattg tgaagtaacc tttacggcct gacttttcac tccatgagtt
ttcaactttc
329521 cacttacgga tttctccctt gtctaagtca acacctacta aggtcattgc
gtgactaact
329581 tcaccttcgc cagtagctaa tctttcagcc ttagtcatgt aagtatcaac
gtcaaataat
329641 tcgtcggtct tgtacaattc agtatctaag taaccagtct tacggtccat
ttcctttaag
329701 acgtcgttac cgaaccaaac actttcgccg tctttcaatt gcttgactgc
agcttctttt
329761 aagtattcga ttggaacatt taagaaagta attggaagtc cgccattaac
gttatcttcg
329821 aatggaagtg agtaaagctt gccatattcg tggtcaggtg cattagtcaa
gcaaacgtag
329881 tcatcgaagt taacatccca atacttgttg aagaattcaa ctggagttaa
gttcttatct
329941 aagtgcaatt ttttatcgtc gtcgcgatac tctaagtcaa aggtcttagg
aggctcgcca

330001 acagcaatcg cagtcatacg gtagatttca cttaagaatt ctttgcgctt
cttttcaact
330061 tcttcagttt tgcctgcttg aactaattta cgtaaaacta aggcatcttt
tctttcctta
330121 cgagctaaag catcagctaa gccagcagtg tgattggtat taaaactttc
aggcattgca
330181 ttagttggga caacaccgta cttcttaact agcgaagcag ccattgccca
ttgaccacca
330241 tctgaaccgg caaaattcat atatgcttta acagtacgat catctaatgg
cttatcagca
330301 gaatcaatga ttgcgtcgta gaagatattt gcacgttcaa ttttatccca
gaagaagtta
330361 tatgcttgag ataaagtgaa gttcttagcc ttatactttt taccaaagtc
atgacgtaag
330421 gtgtttaaag tagcaaataa ccagcaacgc cctgattgca tttggttagt
aacattatca
330481 gtttccactt caacagagaa aacatcattt aagcggttag taactgatgg
gttaaatgaa
330541 gcttctaaaa caccagaacg cattgctgcg cgagaaacaa cttcattgcg
tgaattatca
330601 aagtcattac ggaattgatc aatttcttgt agggttaatt cgtgagacat
aaattacact
330661 cctttaattt ttttataata tttttcatta tatttactat acagcaaata
atgatttaat
330721 acactaattt tatgaatgaa agtaactttc ataagtattt caatagacgt
tatgaagtat
330781 aatgtttata acaatatcgg aggtaagaac tatgaaaaag aatatttttg
tatcaggaat
330841 taccctaatg acaatgcttc ttgctgtagg ttgcagcaat agtgccaatc
aatctagtac
330901 taataacgaa gcaaaaagca gtaagacaag tcacaagaag agtcataagg
ctaagaaaca
330961 cactactaag aaagataaga ctgatgtaga tactgatgag tcagaaacta
ctaattctga
331021 taagaagagc gataagagta gtgaaaattc aagtaatact accagcaaaa
agactaataa
331081 gaacaaatca actgactcta acaattcttc tgattcaact ggatcaacta
attcgaacaa
331141 tgcatcaaat tcagctgcat ctaacaccca aactaatggt aatagtcaaa
atgcatcatc
331201 atctcataat gcttcagatg ctatgtttaa caagatggtt tctgacacaa
ttaaagagca
331261 tggttattca agtaattatg gtccagacaa ctttacctta attgatggtg
gcaatggtca
331321 atactcttta gctgaaaagt caactggaac aattgttggt cattattcag
tcaagaatgg
331381 tgaactttac aagcaagaca ttcttactgg aaacgatgtt aaggttaaat
aatggtttac
331441 tatcaaaaaa tgacgccaga aggctatcaa gaaattaaag acgagatagc
ccgtttaaaa
331501 aaagatcgac cacgtagaat taaaattttg caagaagctc gtagtatggg
agacttgtca
331561 gaaaattcag aatatacgac tgctaagatg gaattagggc atttgcagag
tcgactgcgt

331621 tatctagata aacaattaag atattcagaa attattcaaa aagataaaag
tggcacaatt
331681 gatttaggtt caaatgtaaa actgcttttt gaaggtgatg atgatgcgga
agaataccgg
331741 attgttggta gaatggaagc agacctggct aaaggaaaaa tatcttttga
ttctccttta
331801 ggacaggcct taatgaagca aaaagctggt gcaacagtaa ctgttgaagc
accagctgga
331861 agttatgatg taaaaattat tgcagtaaat taaaaagcac ctcgctttgc
gggatgcctt
331921 tttttgtaag caatcaaatt tattcaattg tgattgtgtt ttctttgtct
tcttcagtta
331981 attttggcaa ggtaatggtt aaaacgccgt cgatgtcctt agcagaaatt
tctttagaat
332041 caacatcagg aatacgataa cttctttgaa tgtgaccaac gcttctttca
cggtgaataa
332101 ggttgccatt cttgtcatct aagttgtcaa atgagtcgcg gctacctgaa
acattcaaga
332161 tgccatcttt ataagaaact ttgatgtctt tcttatccat tcctggcatg
tcgactttaa
332221 cgatatagtc cttgtctgtt tcagctacat cagattgcat aatgttctta
attgaactat
332281 cgtcaaaaaa gtcttttgga aaactaaacc aatcgcttaa ttgatccatc
atattatcat
332341 tgttacgatt cttcatcatt tcgtttgcca taataaaact tccttccatt
acgctctcta
332401 cactttctat tgtagtaggt cagaatagaa tgtaaaatat ttagcaccct
aattgacaaa
332461 gtgctaattt tagcacaaaa aaataggatt tagagtaatt ctagatccta
tttttcatga
332521 ttagtctaag tctttgccgt tagattcaat aactttcttg taccagtcaa
aggaatcttt
332581 ctttgaacgc tcaagtgttc cttcaccctt gtcattttta tcaacgtaaa
taaagccata
332641 acgcttgtcc atttgaccag ttccagctga tactaagtca atgcatcccc
aaggaagata
332701 acccatcaag tcaacaccat ctaaaacgac agccttttcc atttgttcaa
tgtgctttct
332761 taagtaatcg attctgtatg ggtcatgaac tgagccatca gcttcacgtt
tgtcataagc
332821 accaagacca ttttcaacaa taaagagtgg cttgtggtag cggtcagtta
agtggttaag
332881 agaaattctt aaaccttctg gatcgatttc ccaaccccag tcactacgtt
gcaatgtagg
332941 attttcaaca attgcttctt gtaagtcagt taactcatca ggtttaacct
tcttacttga
333001 gacagtagtt gattggtagt atgaaaagcc aacgtaatca acagtacctt
cctttaaggc
333061 aatcttatct tcttcagtaa tatctggacg gtagccattt ctttcaatgt
agtcttcaac
333121 tgcgtttgga tattcaccat taacgtgaac gtcagagaag aagtcacgtc
tttgttcaaa
333181 cttatcagca agtaagacat cgtctgaaga tggagttaaa ggccgaacag
gtgagtaatt

333241 aatcatacag ccaatttgga agtctggatt aatcttgtgt ccttctttaa ctgctaaagc
333301 agaagcaact aattcatagt gagctgcttg atacattgct gcttcttttt ctttatcgcc
333361 cattccattc ttgaataaaa ttccggagtt agttgccatc aagaagtcat tgttaaaact
333421 agattggttg tcaatttcgt taaaggtcat ccagtactta accttgttct tgtaacgctt
333481 gaagcatacg cgagcaaagc gaacaaagta gtcaattaat ttacggttag tgaaaccgcc
333541 atattcttta actaagtgat aaggaatttc aaagtgagaa agagtgatta caggttcaat
333601 accgtatttg tggcattcgt caaataagtc atcatagaac ttaagacctg cttcattagg
333661 ttctttttcg ttgcccttag gaaaaattct agtccaagca attgaagtac gaaaagcttt
333721 aaaccccatt tcagccatta atttgatatc gcccttgtaa tggtggtaaa aatcaatagc
333781 atcatggtta gggtagttct taccagggat gacgccatct gtgatttcac gtggtttagt
333841 tgcggatcct acagtcatta catcggcaac tgacattcct tttccgtctt cattccatgc
333901 accttcaagt tggtgggcag caacagcgcc accccataaa aagcctttag gcatagtgta
333961 tccagtcata ttcaatacgt cctttctaaa gtaagcgttt tcgaagttca ttctacaaat
334021 taaaatctga cttgtaaagc ttaaaagtat agaattttat ttttcataag aaaacagact
334081 tcattgttgc aaccgttttc aattagtagt attatacagg tgttgtctga attgttcggg
334141 aacaatattg aaagggcaga tacaataatg actaaaaaag atcagagttt agaacaaaag
334201 aaacatgaca tgagtgtaag atctataaat tttagccgct atttaatgat tcgttatttt
334261 tctgcggcgt tcttattcac taatctattt tggttagttt ttgcaatttg ctacaaagat
334321 attgttgctt cgataatttc gggattattg tttgttttat tattagtagc ttcagttgag
334381 caagtaagta aatggaatgt gaaaaatact gacttgaaat ttacgaaact ttattatcaa
334441 ctgcagctag gtgcaaatgt aatctttgct ataggttgct atttaccatt tggaaagctt
334501 ctatttcctt ttatgactac aaatgatgtc gcaaatgtaa ttgttacgat tttagtaatt
334561 ggaatgctag gttccatttt gatcttaaga agaattagta acattcaaaa tggtcgcgac
334621 aaatatgcac gagcaattaa gacatttgaa agtaatagac agtaggacaa attgaggggg
334681 atgtactatg tctgacactg cgcaaccatc atttaaagat aaaatgatga agatactagg
334741 caagttctca ggttcacgtt ttgtacgtgc aattatgggt gctggttatt cgattattgc
334801 cttttctatt attggatcaa tgtttttggt cttaacagtt ttaacgcaag ttattactgc

334861 taaaggattt gtagattttt acaataatac atttggacgt tttaacaata
tttatactgt
334921 tatttacaac gccacgatgg gtattatcgc aatttacttt gctggatcat
ttgcttataa
334981 ctatgcagat atttatcgta aagaagaaaa cttattgctt gatccattga
acgcagtttt
335041 cttaacttta atgggtttat tcattaccgt tccacaatta gtatggaagg
gcggtaacac
335101 tgtctttgtt aacattttga agaaagataa tgttattgct ggtggttatg
gcgtttctgg
335161 ttctggttta actagaattg gtgctacagg tatctttact ggtttaatcg
ttgcctggtt
335221 aactgttcaa atttatcgtt tctgtattaa acacaattgg cgaattaaaa
tgcccgcatc
335281 tgttccatca ggtgttgcta actcatttac tgctttgatt cctggttttg
tgattgcgat
335341 tgtcattgct ttaattgacg ttatcttaat tgttcttggt acagatattt
tccaattatt
335401 gtttattcca ttctcatttg tttcaaacat tgctaatacc tggtggggag
ttttgattat
335461 cttcttccta attcacttct tatggtggtt tggtattcac ggtgcgacta
ttatttcaag
335521 tttctaccaa gcaattgttt tatcaaacat ggctgctaat gcttctggtg
ctcactatgt
335581 ctttgctggt gaattctcaa acgcattcgt aatcatgggt ggttctggtg
ctactttagg
335641 tatggcactc tggatggctt ttgcttctag atctaaacaa ttacgagaac
ttggtaaact
335701 tgaagctgtt cctgctgtat ttaacattaa cgaaccttta ctatttggtt
tgccaattgt
335761 ttataacgta cgtttatttg ttccattctt actagcacct atgacttgtt
caatggttgc
335821 ttatgcagct attgcaactc acttagtacc taagattatt gttcaacaac
catggccaat
335881 tcctgtaggt cttggtggta tgatggccac tgctagttgg caaggtgctg
ttttagcttt
335941 agtcaatgct gtagttgcat tcttaatttg gtacccattc attaagcatt
acgacaatga
336001 attgcttaag aaagagcaag ctggtgctga gaaagcttaa ttataaaata
tagactttga
336061 aaattctcat agttagaata taactatgag aatttttcat ttagggatgt
aaataatgaa
336121 gaaatatgaa ttagttgcgg ataaaataaa aaattatatt aataaagagc
aacttcatca
336181 tggtgataaa ttgccaacta taactgactt aatgaagaaa tatcaagtcg
gaaagtcaac
336241 tattttgcag gcaattactt tacttacaca gcgagggatt gtctataagg
tgcaagggtc
336301 gggcgtcttt gttaggccta ccgggcaaga tggttatatg tctctaacag
acaatgctgg
336361 ttttgcccat gtattaaaaa agccgactac ggaagtaatc gagttttcgg
agaagaaggc
336421 acctaaacca atcagtgaac atcttcattc tgatgaagaa tgctacttta
ttaaaagatt

336481 gcgcaagcaa gatggtaagc cctttgtctt agaagagtca tattatcgta aaagtatagt
336541 tcccttcatg agtaaagaaa tcgcagaagg ctcgattttt gattacatcc gagaaggact
336601 aaagaaagaa attcggtttt ctgataagta tatgcgtgtt agaaaattaa ctgctgatga
336661 ggcaaaatac cttgaattaa aagaaggaga tccttgttta gaagtctatg ataccttcta
336721 tctagccaat ggaactgctt ttgatagttc taaattagtt tataattaca aaaactctaa
336781 attttacgat caaagttctg atgatattat ttaacaaaaa tagccctaat ctcgaaatat
336841 tgagactagg gctattgtta tttggcgtaa gctaacacat tattcttcat ttgttttagt
336901 tgttttttag caccttgttt taactgccag ttgtaaaacc aggtaccaag attagaatgt
336961 ttcttttcat caaaactaag aacgtcttct ttaaaagtga tctgacagcc acgttcattg
337021 tcttctaagt aataagcaat tttaacatct agcttagcag atttaaaaac cgaaacatat
337081 tttttgccaa attcgtactc agtaatttca gcttttacat tttttctaa taggtagttc
337141 ctgcgacaat ttttactggt aagtcattgc ctcgtgcttt tttaattgct tgtgtgagct
337201 gcatattaag gtaattaaag aaatcttttg ctttaaactt tgcttggtct gtaattgtaa
337261 tcattttgcg tctttctaat attaatttgt caaaaaaata gagcctagaa aatgctaggt
337321 tctattttgc ctttagtcta aatcttcacc tgtgatgcaa taaccttttt gtaccagtta
337381 aaggaatctt taggctacct ctttaatgag ccgttaccca tgtcatcgcg gtctacataa
337441 acaaaaccat agcgcttctt catttcgcca gttccgtttg aaactaggtc aattgctgac
337501 cacattgtgt agcccattaa tggaataccg tcttcattaa ctgcttcttc catagattta
337561 atttgggcac gaagatagtt aatgcggtaa tcatcatgaa ccttatgatc ttcagttaat
337621 tcatctcccc agccaagacc attttcaaca ataaacagtg gcttgtggta tctatcccag
337681 agatcattta aggcaattct taaaacatca gggtctgttg cccatcccca agcattatag
337741 tcaagatatg gattcttgac gccacctgct gacatattac cgtttgcttt agcatcagct
337801 tcatgagtag tgaggacatt agatgtgtag caagagaaac ttaaaaagtc agcgggatat
337861 tctttgatta attcataatc ttctgggcgg tcatctagct taattccatc tcgttcatac
337921 tccttaaggc ggtattcagg gtagcggcca ccagtttgaa catccgaata gaaaagcatc
337981 tcttgcttag ctttcatgac taaaagctga tctgctggat ctgatgtata tggatagtaa
338041 gcagagtagg caagcatttg accaacttgg ttatttttat caatttcatg agctaactta

338101 acgactttgc tgctggctac aaattgatta tgagctcctt gagcacgatt ttgcttgctt
338161 ccatcaatta gacctccacc catataagga gccatatcca tcgcattaat ttcgttaaaa
338221 gtaagccagt acttaacttt acccttgtag tggttcataa cgatgtctgc atagtgaacg
338281 aagatatcga tcatttttct gtctttccag ccgccaaagc gattgatcag agatagggga
338341 gtttcatagt gtgaaagggt aacaagtggt tcaattccgt acttagcaca ttcatcaaag
338401 accttgtcat agaaagctaa accttcttca tttggttctt gatcgtctcc atttggtaaa
338461 atacgtgacc aattcaggga taaacggaac atattaaagc ccatttcagc catatattta
338521 atatcttctt tatagtggtg gtagaaatcg gtgccttcat ggctaggata gtaatagtta
338581 tctaaaagtg caggctttgc accatcaggt aatttaaatt cttttcccca cgtcattgga
338641 gtaacaccag ttttgccatc gtttgttttc caagttactt ttcttggctc agtagcagaa
338701 ccattagtca aaacatcaat agcgtttaat cctttgccac cttcgttata accaccttca
338761 tattggttag cagcactagc accgccccaa taaaaatctt tcggcattga gtaggtttta
338821 gtcattattt ttcctttctt tatttgttta aatttataaa aaaagttcta gacttgctct
338881 agaactttta cttaaaagct gtttgtctct aaaacgacat aatttcgttc atcgtaacga
338941 ttacgactgg ttgaatattc aactggttga ccattattta accatacgat ttgctcaagc
339001 tctaaaattg ggtcatcttt tttagcttta agatattttt gatcccaatc gtctgcttta
339061 gcagccttga ttttacgata tgcgacgcca aacttaagtt ttaagtcatg atgaagataa
339121 ttataaatag aattatgcaa gatatcctca gttaagttgg gaactaagtg aactggcata
339181 aaagtatgtt caagaatcaa tggctttcct tcaagtcttc ttaagcgacg gatattataa
339241 actgggtcgc tagactttaa ttttaggtaa tgttgaatat cttcactggg aaactcaacg
339301 ttaaactcaa taatatcgct agtaacctca tctgatccaa gtagattggc taagccacta
339361 aaggcatttg ctggtgaatc aaatttatcc tggattggaa ccggacctaa gacaaaagtt
339421 cctaaaccag attctttata tacaagacct tttctttgta atccatctaa ggcttttttg
339481 acagttaacc ggcttacttg taattcgtct gcaagtgttt tttggtcagg aaggggagct
339541 tgagttttgt aaactccatc ttcaattctt ttttgtagaa ttcgagcaac ttttaagtat
339601 tttgcttccg cagtcattat ttttcagcca tttttttgta aacagctact acttctttag
339661 ctaaatcaat aaaggtaata gctgtcatta agtgatcttg agcgtgaacc atgtaaaggt

339721 caacttttac gtggttacct tgtgcttctt gagttaacat atcagtttga acattatgag
339781 catttactaa gccttcattt gcttgcttaa taaattcatc agccttgtca aagtcgccct
339841 tcttagcagc ttggattgct tgcatagcag cagcttttgc attacctgct tgcataataa
339901 ttcccattac aacttgcatg ttatcgctat tttcttgtgc catgatttaa ttctcctaga
339961 tagtattttc aaaataaaag atgctcctga caatatttta ttttactatg tcagcgagca
340021 tctcccataa attatttttg ttttaattct tgaattcctt gatcaattgc tgcatcacag
340081 tcaattggta gttgcagcat caattttcca tctaattcaa gtcccttcgg aatctgaata
340141 atacctggaa aatgatagcc gtggtgagct ttagtttcat cgtctatata gatagaaact
340201 tgtccatctg cattaacgcc aattttgaag tgatgaccat cacgatcaaa ttcattaaca
340261 atttttactt cgtcattcat tttgactatt tgtcgcccat taattcttga gcagtttcaa
340321 gaacatgttc accattcatc atgccatagt cttgcatgtt gatgactgtt aatgggatac
340381 ctgctgcatc agtaatcttc ttaacactgt cttccatgta ttgaacttgg ggtccaagca
340441 ttaatacatc tgggtgttcc ttgtcaattt catcttgaat gcctgatgct gcagtagcaa
340501 agatcttata gtccttgcct ttttctttag ctgcgttttg catctttgat actaataatg
340561 aagttgacat tcctgctgaa caagcaagca taatagtttt gtctgccata ataattatct
340621 cctttgtgaa tataattaag tgtttttcag tgtctgagtg ttgatctata aattaaatgt
340681 atgcactttc atcattgaaa gcatttacat tatagcactt ttttaatgat aagtgtcaat
340741 taataactga aaaaattatt taccttcagc ctcgtctttc ttgagagcgg cagcttcacg
340801 tttaacaaga atgttgtcgt atttcttagc aaatggacag tagattaaag ttgaaattac
340861 taaggtacat gcttgccaaa ttgccatctt ccagccgcca attaggaagc cagaaataac
340921 tgctggcatc gtccatggac aggcaaaacc atttagagga ggaacaatac cagttttaat
340981 tacgaagtag gttgaaatag caactacaac tggagttaag aagaatggta ttgcaaggaa
341041 tgggttcata acaattggta aaccaaatag gaatggctca ttaatgttaa agataccagg
341101 aactaattca actttaccaa ttgaacgtaa ttgtgctgac ttagctgcaa ttaagataaa
341161 gataattaaa ccaatagtaa tacctgaacc agttaagtta atgaagttgt tgtagaattt
341221 gttagtaaca acgtgagcac cattagcaat ggttaatttc ccagccttgt ataattgagc
341281 gttgtcaaat gagtttgctt gaagcatagg accagcaaga gaaccggacg attaaaccac

341341 cgtggacacc gaagaaccag aagaatggta ctaagaatcc aatggcaatt gcgccaccaa
341401 gtgagtcaga gataccttga agtggaattt gaagagtttg gtaggtccat tgtaagaagt
341461 ctgaaccagt agttaacttg aagcctgcgt aaataagcat agtaccaatt aagataattc
341521 ctgatggaat catagcagta aattggttaa aaacagctgc tggaacttgt tcaggcaact
341581 taatagtcca gccttttttc ataatagctg agtaaatcca gccgactaat aaaccaacaa
341641 taattgcggc gatcataccg tctccgccaa gccaggtaat attaaataca cctgtgaccg
341701 gggattctaa gaagctttgc aaggcatgtg gtaacttatc gatattttcg gttacggctt
341761 tagcagtcat ttgaccgtca atacctttag acatcgcatt ctttaatgga ctatcaatgg
341821 ttaaagtttg aagcattaag aatgctgcta aagaagtaag accgggagca agtggctcat
341881 agccttcatt tttaacataa atataggcga taccaacaga tgcccacata gccataatac
341941 taaaagtagt agtgtatgct tggttaaaga atgctcccca accagaagca ttaatggcat
342001 cagctactga tttgatcgga atatttccta gaataaggaa gatagaaccg attaagataa
342061 atggtaaagt gtaaatcata ccattttgca aggcctgaat agccttggtg ttaacaaatt
342121 tcattactgg ggggcaagat gtgtttatta acgaaaacac taacgcccga tttttctgt
342181 tcactcatta tatttcgtct ccattatctt ttaatacttt ttggaaccaa taaaatgatt
342241 tcttaggaac acgttttaaa tctttcaaat catggtttga ccggttaaca tagacagcgc
342301 cataacgctt ttccatatcg gcatgtgaac ttggaatatc aattaatccc cagcctaggt
342361 agccaagaac tttgacacca tctaagaaca ttgcatcttt cattgctttg atatgttctt
342421 catggtaagc aattctttca tcatcttcga tcatatgctc accgtcccaa gtttctttta
342481 agccaattcc gttttcaatt gggaagactg ggatcttgta atggttgtac attgtcgtaa
342541 tggcgtttct aaaaccaagt ggatcaatcg tccagcccca atcattagtc ttgagatatt
342601 tattttcaac accgccataa ttcatgtaac ggttaggtgc tgcatctttt ggaattttat
342661 cggctgagag agtccaagaa gagtagtagc tgaaagctaa gaagtcagct tgagcttttt
342721 ccatttcttc tttatcttcg tcagtgatat cccaattaat atcgtgatcc tttgcatatt
342781 gcataacttc tggcgagtaa ccgcgtccag tatctgcatc ataaatgttg aagttcaaaa
342841 attcttgagc ttgtttagca gcccaaacat cagtagcttt tgatgtttct ggataaattt
342901 gctgataggc aagcataccg ccaattttta catcatcata tttctcatga atgtaattag

342961 ccaggcgaat atgactaatc attgtgtgat gaaagatagt atacatgtcg tctaaagact
343021 tgttgccttt ttcatagcca gaaatattga atacttcgtc ttgaaagtaa agattatgtt
343081 cattaaaaac aatccaatat ttaactcggt ctgaaaaatg atcaatcatt tttttaccaa
343141 atctaacaaa ggcatctaca acgtgtcttg acataaagcc gttgtattcc ttagccagat
343201 gaagtggcat atcgaagtgg taaagacaaa tcattggttc aatgccacgt ttgagcattg
343261 cattaatcaa tctgtcgtaa aaatcgattc ctttaggatt aaattctcca tctccttcag
343321 gacaaactct tgaccaggaa atttgaatac gatacatatt catattcatt tccttcatca
343381 gatctaaatc ttcatcataa cgatggtact catcaattgc ggtgtgccaa tctgtggtgt
343441 tttctgtagc aggtcgtaca tcgtaaactg ataagccttt accatcctca ttccaggcac
343501 cttcagtttg catacttgaa actgaattac cccagaagaa gtctttaggc attttctgct
343561 tattcataat atttcctctt tgcaatcgct tacatatttt acgtgtataa atataaactt
343621 atttctagta aaagtaaagt actttaatat aaaaatatat acttttataa aattggataa
343681 caaaaaagga ccgagatagt attctcagtc ctttttgtga tgctttcaaa cacatttatt
343741 taataataaa aaagtgtcta tataaaattt aatgctcttt ttcaaaatct gcaacagctc
343801 cacgcaaatt tgcttcattt tttaaggtac agatgtctaa aattggctta acagtcgcaa
343861 tatttaattt atttcttatt tttgaaattt catcattaag aagaggtatt aattcttggt
343921 tttgagaaat tccgccaccg atgatgattt tttctggatc aaagctatgt tgaatattat
343981 aaatagcagt agctaaagca tgaatcagct tgccacgttc ttcctgtgca accaaatcat
344041 cagtgtcagc aagttcaaat actgtcttac catcaaaatc tttaccagtt ttttcattgt
344101 agcgtttagc cattgaaacc ggagaagcca aggcactaag ttgctgacca tcaataatca
344161 taaagccaaa ctcaccgcca tatagatgag caccatgcca aatttgatta ttaatgataa
344221 tactaccgcc aactccggtt ccaataacta aaaaagccat gctgctgcat cctttagctg
344281 atccgtcaac aagttcagct agagcggcac aatttgcatc attttcaata cttactggca
344341 agccaaagcg tttttctaac tcaggtacaa ttttgaaatt gtgaatataa ggtagagcac
344401 tagcaccttc gattacccca gtctttttat taacagcacc aggtgaagaa atagctacac
344461 catcaatttt attactagct ttgatttcat taactgcatt acttaattca ttgtaaaaag
344521 tttctaaatc attaggcgta tcaatagtga agatcatctt taattttta tttatccaag

344581 tagcgatctt gattgttgtc ccaccaatat caattgcaat taagttcata
ataaatgttt
344641 cctctcttgt ttttcttcac tttcatgcta attataaatg aaagcgttgt
caataagtat
344701 atatttttaa tgaactataa ataacgttaa gtgctttgtt ataataaatg
cagttgatat
344761 aaaggggaag aagccatgaa tacgccatca tcaaggcagg gaaatttaac
tagatatgct
344821 gtttatttgg ttctttattt aatagtaatc ggagtaaaga aattaacatt
attgagtgac
344881 cgagtaaata agattggata ctttttcttt ctgatctttg cgctttttac
cttatatttc
344941 tatatcagac aatttaatcg cgaacagcgt ttttttgaaa aaaagccaag
tatgcctctt
345001 ttagcagatt acggatttat tgtgggagtt tcgatcttag tgattctagg
aagaattttt
345061 atttcatatc tacaggctaa aggcgcagtt cctttgatga gttttcaagt
aatttatcaa
345121 aagggtgagt gcaatctctt attttggttt ttcatttttt caatcggaat
attgatacca
345181 gcgatgcaag tgtatttaac taccggcttt ttctttaatt atggttttag
agcacaaaat
345241 atgactagcg gaatagttgg gatcatttgt tcaggttttt tcttcggaat
tctcaatttt
345301 caaacttcat ttgccttgtt tattattaac agtctttatg ggatgcttct
tgcttggtcg
345361 tacctatatt cacagcgaat aatgatgcca atttatctag caattttaaa
tggaattttt
345421 atggttatct tggcataaca aaaaggagga acaaaaagtt cctccttttt
taatataagc
345481 ggatagcgtg aatcgaaccc gcatcttcag cttgggaagc tgacatttta
ccattaaact
345541 atacccgcat aagcaaaatg attatatcat ttttgttaac gatgtggcaa
ctgatgtcga
345601 accaaattat attctgaacg gttaagtaga gaagtgaagt tttgaaagag
ccagcgacgt
345661 aattcggatg gctttaagac taatggcatg cgttcatgaa caggaagaac
ggtactgtta
345721 gcagcagttg ttaccattga aaaatcgtcg ccttgataaa tgccagcgat
taaagtcatt
345781 ggttcttctt tagcagtaaa agaagagtgt tcatggtagg tctgattatt
tgaagcaagg
345841 taagtttgtt tactgctttc ccaaaattca ttggctaaga taatgcagcg
ctgccgagca
345901 aatgagttgt cccacattga tggtttttct tcataaaaac gttctatgcg
agcgttgtaa
345961 agaacacgat tcttttgaaa agggctcgga tagccccatt tcttttcaac
cagttttaat
346021 tcgttgttgc tgtaaagcat tactaaggat gaagatttag gatagatttg
cgtgccagca
346081 gaaaattttt cttcaatgtt tttaggtaca atcagtggta attttaaatc
agttgtcaag
346141 taagtcttaa tttctttaag agagggcatt gcatagtggc tacacatgat
ttttcattct

346201 ttctagtttg attagttctt tcaatttaat tctagcaatt tttgagtaag
tttaaacgaa
346261 taaggttttt tgctagaatt ggaggtaaag ctaaagatta acaaatattg
gagaaaatta
346321 tgacgaaaga aattttatta actggagata gaccaactgg taagcttcac
attggtcact
346381 atattgggtc cttaaaaaat cgtgttaagt tacaaaatga aggtaaatat
gagccttata
346441 ttatgattgc ggatacgcaa gccttaactg ataacgcaag agatcctgaa
aaaattaaaa
346501 atagtttgat tcaagtagct ttagactact tagctgttgg tttagatcct
gaaaaatcta
346561 ctatttatgt tcaatcgcaa attcccgctt tgtttgagtt aactgcatat
tacatggatt
346621 tagtaacagt tgcacgtcta gaaagaaatc caactgttaa aactgaaatt
aagcaaaaga
346681 acttcaagga ttcaattccc gtaggatttt taaactatcc ggtttctcaa
gcagcagata
346741 ttactgcttt taaggcaaca gtggtcccag ttggggatga tcaagaacca
atgcttgaac
346801 aaactcgtga aatagtacgt acttttaatc gggtttataa cactgatatc
ttagttgagc
346861 caaagggcta cttcccacca aagggtcaag gaagacttcc aggattagat
ggaaatgcca
346921 aaatgtctaa atcattagga aatgcaatct atttatctga tgatgcaaag
actgttcaaa
346981 agaaagtaat gtccatgtac actgatccaa accacatcca cgtggaagat
ccaggacaag
347041 ttgaaggaaa tactgtcttt acttatcttg atgtgtttgc accagacaag
gataaagtgg
347101 ctgaacttaa agaacaatac caaaaaggtg gcctgggcga cgttaaaatt
aagcgctact
347161 taaataaggt tctcgaagaa gagcttggac caatcagaga acgtcgtgaa
aagtttgctc
347221 aaaatccaga cgctgtttat gaaatgctac tagaaggatc aaagaaggca
aataaagttg
347281 ctaaccaaac tttaaaagaa gttagagaag caattggttt aaattatttt
gacagattaa
347341 ataaataatt taaagagggt aagaacaatg acaagagaca ggattccgtg
gaagcaatat
347401 tttatgatgc aggctttagt tattgcacaa cgttctacct gtgatcgtgc
cttagttgga
347461 agtgtattag ttaaagatga ccggatgatt gcgaccggat ataatggtag
tgtttcggga
347521 cagcctcatt gtgatgatgt tggtcatttg atggtggatg ggcactgcgt
aagaacgatt
347581 cactctgaaa tgaatgcctt aattcaatgt gcaaaaaatg gtatttccac
tgaaggtact
347641 gaaatttacg ttacttattt tccttgcttt aattgtagca agtgcttagt
gcaagctggc
347701 attaagaaaa ttaactatta ttatgattat catgataatc ctttagcctt
aaaactattg
347761 cgtgaaaaag gtgttgcaat tgaacaagta acacttgatc ataaatacgt
tcaaagctta

347821 gcagacaggc tagagaaggc tgacaatgaa aaataaaatt attggtttgg
tgtcactgct
347881 tatttgttta ttaacgttga ctgctagcac caagtctttt gttagtgatc
cgcaaggatt
347941 atttgataat cagacaaaag aacttgttaa tcagaaaaat gagagttatc
aaaagactaa
348001 aactaaacct gaaattcgcc ttttaagctt aaaagacagc aatgatttaa
atcgaattca
348061 gccattaaag aatcagataa ttattgctgt tgctcttcaa aacaaaaaga
aaaatgttca
348121 aattattgtc ggtaagaatt atcaagatcc tttaactcaa agtaaatgtg
gcaatattgt
348181 tcgttatgcg gggaataaat taagaagtga aaaggcagca acttttaatt
tcggaattcg
348241 attagtgttt aatgcttgcg ctactttggt tgcccaaaaa gataagctgt
cttatgataa
348301 aaattcgcta acacaagatg aactgcaaaa aatagatcat ccacagagtg
taaacttggt
348361 ttggggatta gtaattgcag ctttaatcag tttcggacta gttctattta
agagttttcg
348421 aaaaaagaag tagggacttg actaattctt agttagccgt taaagtatca
atagaaatta
348481 aatattagag caaataaact gttaggtgag actcctacgt gaacatatgc
tgtcgcccag
348541 caacatccag agatgccaac gggtcaaaca gggatcgtcg atataaggcc
ctccccagcc
348601 agctagttat gttaaataac cttacgtcac gtagtgttaa aactgaacga
cgagtataaa
348661 aatcgcttgg atttgagccg atgcagtttt tgcatcggtc tttttgtttg
tttgctaggt
348721 tcttaagaaa ggatgttgaa gatgttcaaa atcaacactt ccggcacaag
aaatactaca
348781 gacaccaagc tggtgcgccg gattgcatgc gaagataagt ttgcgacgct
gcaacgatta
348841 aaaacctcaa ttagcggcct tcaaagtaaa gatgcgaggc aacgcttagc
aaaatacgga
348901 tctaatgaaa taataattaa acatgataag acaaaacttc actatttgtt
aaaggctttt
348961 ataactcctt tttcccttgt tctgcttttt ttagcaattg tagcaatttt
tttaaattcc
349021 tctcaacata ctcttaacct aagtccagca attttgtttt agcaatttta
tttatttcag
349081 ccattttaac tgctatgcaa aatattcgta ttaatactaa tattcaaaat
ttgataaatc
349141 gaatttctag aacaactcaa gtaatgcgtg attgtgaacc acaccaactt
ttaacccgtg
349201 atctaacaat tggagatgtg gtactactta aagctggaga tattgttcca
gctgatgtta
349261 aattactgaa aagtaatgat ttagagtgct cattacttgg tgaagaagac
actgtgaaaa
349321 agagtgctac taattacact ggtaaaaata ataacttaga ttacgctaat
attttatatg
349381 caggcacaat tattctttca ggttcaggca ttggcgttat ctttgcgact
ggtaaagaaa

349441 ctgtttttgg aaaggtagtt caaaatcttt ctaagattaa tctagagaac catctcttta
349501 attttagtat gaaaaaatta accaaaatct ttttaacatt aattctagtt attgttccag
349561 ttgttttgct aattaatgaa ataatgcgtg gtaattggga gaatgctttg atttttgcta
349621 tagcaattac tattggcata acggctgagg taatgccaac agctattgta aataacttgg
349681 taaaagccaa tatcgatatg cctaggattc gagcgacata tgacaattaa ttatctaaat
349741 aatttatagg aattccgtat tttatagaat tcctttttt aatgtacaaa ttaagtccta
349801 catgctatgt tatactagca attaaaagac ttatgagagg aagaataaag atggctgaaa
349861 agaaaacttt ctatattact acacctattt attatccatc tggaaaatta actatcggta
349921 atgcttatac tacgattgct gctgatacaa tgactcgcta caaaaaatct cgcggttttg
349981 ataccttctt tttaacaggt actgatgaac acggtttgaa gattgaacaa aaagctgaag
350041 cacagggtat taaacctcaa gaatttgtag acaagatggc agcttcttat aaagagttat
350101 ggaagatgtt agatgtttct tacgatcgct ttattaggac aactgataaa gaccacgtta
350161 aggctgttca aaagattttt gaaaagttgt taaagcaagg cgatatttat ttaggcgaat
350221 atactggctg gtattcagtt gaagatgaag aatactttac tgaatcacaa cttgctgaag
350281 tttataaaga tgataatggt aatgttattg gcggtaaagc tccgtcaggc cacgaagttc
350341 gtttggttaa agaaccttca tacttcttta gaatgagtaa atatgctgat cgtcttttac
350401 agtattataa ggatcatcca gactttattt tgcctcactc tcgtgaaaag gaaatgataa
350461 ataactttat caaaccaggt cttgaagatc tttcagtaac tcgtacaaca gttgactggg
350521 gaattcctgt accaagcgat cctaagcacg tagtttatgt ttggattgat gcactttcta
350581 actatattac tgccctaggt tatggctcag atgatgattc attatttaag aaatattggc
350641 cagctgatgt tcatttagta ggtaaagaaa ttgttcgttt ccacactatt tactggccaa
350701 ttatgttgat ggctcttaac ttgccacttc ctaagcaaat cttcggtcat ggctgggtat
350761 tgatgaaaga cggtaaaatg tctaaatcta aaggtaatgc cgtttatcca gaaatgattg
350821 ttaaacgcta tggattagac gctcttcgtt actacttgat gcgtgcaatt ccatttggtt
350881 cagatggtat ttttaccccct gaagatttcg ttgagagagt taactttgat ttagctaatg
350941 acttgggtaa cttgctaaat agaactgttt caatgattaa ccaataccaa gatggtcaag
351001 ttgcaccagt tgcagcagaa caagatgaat ttgctcaaga cttaattaag acagctaacg

351061 aaacaatcgc agaatatcaa aagcacatgg atgctctcca cttctctcaa
gctttagaca
351121 gtgtttggaa gcttatttct cgtgcaaata aatatattga tgagacaact
ccttggactt
351181 tgaataaaga aggcaagagc gaagaattat ctaaagtaat gtctaattta
gctgaaagtc
351241 ttcgtttagt tgctatttta attgcaccag taatgactca aagtccagtt
aagatgtttg
351301 aacaacttgg tcttgactgg aataatgagg aacaaaagaa acttgaattt
ggcggctttg
351361 actggaatgt taaagtaact gaaaaaccaa ctccaatctt cccaagactt
aagaatgatg
351421 ttgaagttaa atacattaag gaagaaatga agaaggcaaa accaaagaag
aagactagaa
351481 gccagcaaaa agaagaaaag catatcacca ttgatgactt tgacaaggtt
aagattcaag
351541 ttggtcaaat tttatcggta gaaccagtaa agggttcaag taagctgttg
atgtttaagc
351601 tagactttgg cgatggaaat gagcgtcaaa ttttgagtgg tattcgcaag
ttctatccag
351661 atgcaagcga acttttagat aaaaaggtgc tagcagtaac taacttgaaa
ccacgtaaaa
351721 tgttaggtca tgaaagccaa ggaatgcttt tatcaagtga aaaagatggt
caagttaaat
351781 tagctatcgt aggcgatgaa catgaagttg gggctgaatt aggctaatga
aaatttttga
351841 ttcgcatacg cacttaaatg atgtgccttt tcgaggaaaa gaagagatct
accttgatcg
351901 agcaagagaa ttaggtgtgg taaaaagtgc ggttgctgga caagatccag
aatttaatga
351961 acgagcagtt gatctaagtc aaagatttga taatttatac gctattgttg
gctactgccc
352021 agacgtagct aaggattatg atcaaaaatc agaagataag ctagttgagc
agctaaaaaa
352081 gcctaaaact gttgctttag gagaaattgg actagattat tactgggatg
aatcgccaag
352141 agatgttcaa cgaaaagttt ttgcgcagca gcttgactta gctcatagct
taaagatgcc
352201 agtaaatatt catacacgtg atgcttttga agatacatat caagttttaa
aagatagtca
352261 tgtcggtgaa tatggaggaa taattcataa ctttaatggt gatcctgatt
ggcttaaaaa
352321 gtttcttgac ttagggatga tggtgtcatt ttctggcgtt gtttctttta
ctaaagcaac
352381 tgacgtacat gcttctgcca aagttgtacc atgggataga tttctgattg
aaactgatgc
352441 tccatattta acgccaaagc cataccgcgg aaagcagaat gaaacaggat
atgtgcgtta
352501 tgttgcagaa gcgatcgcta agttacgtga cacatctgtt gaagaagtgg
caaagcacac
352561 gtttgaaaat acgatgaggg catatggaat caaataatca aaaacagttt
aatgcggtag
352621 ttgttgttga aggacgagac gataccaaga gattaaaaca gtttttccca
ggaattgaaa

352681 ctattgaaac taatggctcg gaggtatctg atcaaacttt agcagaaatt aagaaactgg
352741 caaaaacacg agaaataatt atctttacag atccagacta taatggtgaa agaattagaa
352801 gactggtgac taatgctgca cccaatgcta aacaagcttt tatcactcgt aaagagggag
352861 agccaactaa gagaggaaat agcttaggag ttgaacatgc gtcaaaagaa gcactagtac
352921 gtgctttggg tgatttgcac gaggttcaag cagtagagag tgatattact aaagaaaagt
352981 atgatgattt gggtctagca gctagtccag aagcgcgtct tttacgtgaa aaagttggga
353041 ttaagttagg aattggatat ggaaatagta aacaattctt aaagcgctta aaaatgtttg
353101 gaattactta taaagaatta aagagggcag ttgaagatgt caaatagtat gccgattgct
353161 agtccagttc ggactcaggc gattgttaat cgctacttta tgcatgcaaa aaagaatttg
353221 gggcaaaatt tcttagtaga tttagccgca attaagggaa ttgttgaagc ggctgatatt
353281 caaccaggtg atcaagttat tgaaattgga ccaggaattg ggtctttaac tgagcagctt
353341 cttttagctg gtgctaaggt gttagcttat gaagttgatc aagatttacc tgaaatttta
353401 aaaaatgaat tgccacaaaa agttgctggt gaagaattaa gcaatcgctt taaattagtc
353461 atgaaagacg tattaaaggc aaactttact aaagatagcg atggttttct tgatttaaat
353521 aagtcggtta aaattgttgc taatttgcct tactacatca caacgccgat aatttttaat
353581 ctaattaaga gtaatttagc tttcagtagt ttaacgctaa tgatgcaaaa agaagtggct
353641 gagcgtttag ttgctaaacc caagactaag gaatatggcc ctttaacaat tgctgttcaa
353701 agcagaatga atgtgaagct agcagaagaa gtgaaaagta cgtcattcat gccacgccca
353761 aaggttgact ctgcagttgt tgtcttgacg ccacttactg aaaagccaga tattgacgat
353821 tattttttct ttgatcatgt tgtaaaaatg tgctttgctc agcgccgaaa aacactagct
353881 aacaacttaa aatccttagt caaggataaa gatatgcgtg aaaaaatgat taatgattta
353941 ggactagatc ttcgagttcg tccagaagaa ttaacgctaa atcaatttgt agagttagca
354001 catcttttaa aagatcagca agcatagaac gcttacaatt tacagacttg aaaaaatatt
354061 aattttttga tagaatagta accaagcaaa ggagtgacgt ccagtgccaa caacactaat
354121 ggcaattaaa cataatttgg attctcattt gggcgaaagc ttggtagtag tagctcaagc
354181 aggaagaaag aaagtaacac gccgtaaagg tagattaact aagacttatc gtgcagtgtt
354241 tgtagttgac cttgatcaag atcaaaacaa ctttgaacga gtatcttaca gttacacgga

354301 tttattaacc aagaacattg agcttgagtt tgatgaaatg taagcgacaa tagaatatat
354361 tacgaacaat cactagtatt tttcttaaaa tgctagtgat ttttctttag atagagtata
354421 tttaagttta gaaatttaat ttaatgttta ggaaggtaaa agcatgaaac gttcagaaag
354481 attagttgat atggttaagt atcttttagc tcgccctcat actttgattg cattgccatt
354541 ttttgcagat cgctatggcg cagctaaatc ttcaatttca gaagacttgg caattttacg
354601 tcaaactttg gctagcgacc aaaatggtat tttagaaact gtagcaggag ctgctggtgg
354661 agtgagatac attccttttg ttgggaaaaa agaggctact aactatcttc atgatttagc
354721 tgaccgtatc gaagatcctg atcgtatttt gcctggtaat ttcgtttact tatctgatat
354781 tttaggctca ccacaagatt tgcagcaaat tggacaattg attgcaacca aatatgccta
354841 cagtaatgtt gattatgtaa tgacaattga aactaagggg attgctatcg cgcaagcagt
354901 tagtcgcttt ttaaacgtac catttgtgat ggttagaaga agacctaaaa ttactgaagg
354961 atcaacaatc tcggttaact atgttgcttc gtcaagtgaa cgagttgaaa aaatggaatt
355021 agcaaaaaga ctacttccag aaggaagcaa tgtattaatt gttgatgact tcatgaaagg
355081 cggcggaact ttaactggga tggaagaact agttaaggaa tttaagggaa cagttgctgg
355141 aatgtgcgtt ctctgtgaga caaaatacgc ttctcaaaag gtcgtagatg attatcaatc
355201 actgattaag attactgaag ttgacagaac taaaaagcta attaaagtac ggccaggtaa
355261 tttcttagag cagacagact tcaatcgttt tccaaaatga tatactgata gttgtttaaa
355321 tataaaaatt gaagggatat aaaactaatg aataagtatg ttgttatctt agctgctgga
355381 aaaggtaccc gcatgaagtc ccaactttac aaggtattac acaaggtttg cggtaaaact
355441 atggtagagc atgtggtgga tgctgctaag ggcactaatc cagataagat tattactgta
355501 gttggaaatg gtgcagatag cgttaaagat gttttagcag gtcaatcaga ctttgctttt
355561 caaaagcaac aattaggtac tggcgatgct gtactggcag ctagtgattt attagcaaat
355621 cttgagggtt caactttagt tgctactggt gatactccgt tatttactgc tgaaactttt
355681 aacaacttat ttaagaagca tgaagaaagt ggaaatagcg caactgtttt aactgcaaaa
355741 gcgccaaatc catttggtta cggccgtatt attcgtgacg aagatggaaa tgttttaaga
355801 attgttgaac aaaaagatgg tacgccagaa gaattagcag tagatgaaat taatactggt
355861 gtcttttgct ttgataataa agaactattt aaggctttaa aacaagttgg caatgacaac

355921 gctcaaggcg aatattactt aactgatgtt ttagaaatta tgcgtaaagc
tggtcataaa
355981 gttggtgcgt atgaaatgcc agattttagt gagagcttag gagttaatga
ccgaattgct
356041 cttgctcagg caactaagat tatgcaaaga aggattaatg aagagcacat
gaggaatggt
356101 gtttcattta ttgatccaga tacagcatat attgatagtg acgttaagat
cggtaatgac
356161 actgttattg aaggaaacgt tgttattaaa ggaaacactg aaattggcag
tgactgttat
356221 attactaata gttcaagaat agttgattct aagattggta atcatgttac
tattacttct
356281 tcaactcttc aagaagctca aatggatgat aatactgata ttggacctaa
ttctcacctt
356341 cgtccaaagg caattattag aaaaggtgct catatcggaa actttgttga
gatcaagaaa
356401 gctgaaattg gtgaaaatag taaagtagga cacttaacct atgttgggga
tgctacttta
356461 ggtaaagata ttaatattgg ctgtggtact attttctcta actatgatgg
tgtgaagaag
356521 ttccatacta atgtggggga tcattcattc attggtgctg gtgctacgat
tattgcccca
356581 gttaatattg cagatcatgc atttgttgca gctgactcaa ctattactaa
agatgttgaa
356641 cgctatgata tggcaattgc taggggaaga caaacgaaca agcctgatta
ctggcataag
356701 ttaccgctct caaaagataa agaatgggaa taaattaaaa gaagttgtta
aacacaactt
356761 ctttttttgc tatttacaag cgtaaaggat atttttttgc tattattaag
tatggaattg
356821 taatttttca gtggaggaaa ttatgtctca attagacaaa gaaatcaaaa
tttttgcgct
356881 taactctaat aagcctttag cagaaaaaat tgctgataag gttggagtta
agcttggtaa
356941 atctgatgtt aaacgtttca gcgatggtga aatccaaatt aacatcgatg
agtcagttcg
357001 tggtaaagat gtttacttgg tacaatcaac ttcagctcca gtaaatgata
atttaatgga
357061 attattgatc atgattgatg ctgttaaacg tgcatcagct agaagcgtaa
acttagttat
357121 gccttactac ggatatgcac gtcaagatcg aaagacccgt gcacgtgagc
caattacagc
357181 taagttagta gctgatatgt tgcaaaaagc tggtgctgat cgagttcttt
cgcttgattt
357241 acatgcccca caaatccaag gattcttcga tattccagtt gataatttaa
tgggtgcccc
357301 actccttgct gactatttct tgagtcatga attagaaaaa gatgctgttg
tagtttcacc
357361 tgaccatggt ggcgtaacta gagcaagaaa attagcagaa tttcttaaga
ctccaattgc
357421 tattgtggac aaacgccgtc caaaggcaaa tgttgctgaa gttatgaata
ttattggtga
357481 tgtagaaggc aaacgtgcaa tcattattga tgatatgatt gataccgcag
gtaccattac

357541 cttagcttct caagctttaa tggatgctgg tgcaactgaa gtttatgcat ctgcaactca
357601 tgctgttttg tctggtccag caattgagcg tttaaataat tctcctatta agaaattgat
357661 tttaactgac tcaatcaatc aacctgaaga aaaagacttg tctaaggctg aaattgtttc
357721 tgttgggcca ttgatgggtg aagcaattaa attgatccaa gaacataaac cagttagtcc
357781 actgtttaat actcgttttc aatcacataa ctaaatgaac tataaggtga tacctataag
357841 taggtatcgc cttttttat tgctttaaaa ataatagttt taatttttag actcgctaag
357901 cgtttacatt attagaataa ttacggtaat attaagttat acagttttaa aaaaggactt
357961 agaagtatta tgactacttt atctgatgta gcaaaaaaag ctagcgtgtc taagatgacg
358021 gtatcacgcg tcattaatca tcctcaacag gttacgcctg aattaagaaa aattgtcgaa
358081 caggcaatgg aacaactaaa ttatcatcca aattcagttg ctagtgcact agcccaccac
358141 cgtacaaatg tagttaaatt agtaattctg gaggatattg atacaactga accttactac
358201 atgaatttac ttttcgggat tgctagtgga ttaggtaaaa aacactatgc actacagtta
358261 gcaactgata gtgaggttgg tggcggcgat ggttatatta tcacaggtgg cagagcaaat
358321 gatgccaaat ggctggacca attaaataaa ccatttgtgt tattcggtga aaatcgctat
358381 ggctatgatt ttattgatac cgataataaa ttaggtgaac aaatagccac acaatatgct
358441 ctggataaaa attatcaatc aattgttttc attggaattg atgaaaaaga gccttttgaa
358501 tattcgagag aagctggata tatcaacacc ttgcagaagc ataatatgat tcctaagatt
358561 tttagaatcc aaaatcatag tagtttagct gaaaaactaa ttattagtaa ttggaaaaag
358621 tttgcccccca acacttgttt tatttgtgca agtgaccgaa tagcggttgg ggttgtacgt
358681 gctattcaaa gaaaaaatgg aaatattcca cgggattttg gaattattgg cttcgatgga
358741 gttttcttag atcaggtatc aaatcctaag ttaacaacta ttaaacaaaa tttatttaag
358801 ttaggtgaac tcttagcaga aatgattttg caaaagattc aacaaggcgg agctcagcag
358861 ggcgaggttc ttattgaacc ggaattaata aaaagtgaat ctactagaat ttaagaagct
358921 atggatgagc ttcttttttt gataccggta acatttaaat agcattgtaa gcgcttaaaa
358981 ttattgctta aaataggatt tatggaggaa gtttattatg agacattggt atgaccaagc
359041 aattatttat caaatttatc ctaagtcttt ccaggattct aatggtgatg gtattggtga
359101 cctaaatggt attaggaaaa gaataccgta tttgaaggaa ttaggggtta atgctgtctg

359161 gctgaatccg gtctttgttt ccccgcaagt tgataatggc tatgatgtat caaactattt
359221 tgccattgat cccaaaatgg gaactatgga agatatggat aatttaatca aggaaatgca
359281 tgaagctgga attcatgtaa ttatggattt tgttttaaat catacgtctg atcaacatcc
359341 ctggtttaaa gatgccatta aaaatcctga tagtatttat cgggactatt atatttttgc
359401 cggccaaaat aatcagcgac caaataattg gggaagcttt tttggaggta gtgtttggga
359461 gaaagatcct gctggaacag gacagtttta ttttcatctt tttgataaaa gaatgcctga
359521 tttgaactgg aaaaatcctg aagttagaca tgcaatgagt gaaatttcaa agtattggct
359581 agaaaaagga attgatggtt tgagattaga tgcctttatt catattgcta aagcagactt
359641 aagacagaat ttcccagtta atagtaaagc tgaagaaccc gtagtagccg aaccattttt
359701 tgctaactta ccgcaagtac aaaaatggat gcgacctttc tgtgaggaaa taaagcaaga
359761 ttatcctgat gcactcttgt taggagaagc agcgagtgcg aatgttaatt tagcagtcga
359821 ttatacaaat aaagataacc acttgatgga tagcgtaatt acttttaggt actttacaga
359881 agatcaaagt caggtggatc ctagtttttc agctcaatat cagccgaaat cattagattt
359941 agtaaaattt aagcaaaatc aggctgtctg gcaacaaact ttaagtggcg tgtctaagcc
360001 cactttgtac tggaataatc atgatatggc tagaatagct actagaattg ctaaaaatga
360061 tattcaagct cagagcttag ccatgctaat gtatttacaa aatggcctgc cgattattta
360121 ttatggcgaa gaactcggaa tgaagaactt ggagtttgaa gacgtaacac aatttgagga
360181 tgaaacagtt aaagaattta ttgaaagtgc cagaaaggct ggaaaaacta gtcaagaagc
360241 attgttaatg gtaagtaaaa ctcataaatt gccagctaga gggccaatgc cttggaataa
360301 tgcaaaaaat aatggattta gtgatgggaa accttggctt aaaggaaaga aagttgatca
360361 tgctaatgcg gctgatgaaa tagcagatag ccatagcatg tttaatttct acagaaaatt
360421 aattagccta aagaaagaag cattgtttga agatgggggc tattatttat tgccaacgtc
360481 aaacgatagt tatgtttatg agcgtaattt aggagatcaa aaagcattag tcgctgtttc
360541 tttaagtaaa gatgctatag aacttgacgt agatcctgaa tttaagcaag aaaaattagt
360601 agcaggcaac taccaattga aagatggaaa attaaaattg gctccgtatg caggagtagt
360661 attaacaaaa gactgaggag taaaatatgc aactagcagc tttaagacat agaactgaaa
360721 gcgaagactg tactgtaatt gatcattcgc acgtaaaaat tagacttcat actgctaaaa

360781 atgatgtaaa aaaagttatt gttcactata ttgataatta tttgccacct gcagatgcag
360841 caacgctaga aatgaaaaaa gcaggtagcg gccaagtaaa cgattattgg acagcgactt
360901 taacagcacc atatcatcga attaagtata cttttgaagt tatgggtaat gatggcagcc
360961 acgttatttt tggtgatcgt agtattgaaa aatttagcga tgagaaatta agagaagatg
361021 gcttatattt taaagtgcca tattttcatg atattgatcg tattaaaacg cccgcttggc
361081 tcaaagacat tgtctggtat caaatttttc cagaacgctt tgctaatgga gataaaagca
361141 atgatccagt taacgttaag ccttggaatc cagaggatca tcccggcaga gaagactttt
361201 acggtggaga tttgcaagga gtgttagatc atttagatga tttacaaaag ttaggaatta
361261 ctggcttata tttttgtcct atttttaagg caagctctaa tcacaaatat gacaccattg
361321 attacttaca agttgatcca gcttttggcg ataaagattt atttgcaaaa gtagtcaatg
361381 aagcccacaa gcgcgggatg aaagtgatgt tagatgcagt tttcaaccat cttggtgacc
361441 aatcaatgca gtggcaagac gtggttaaaa atggtgccaa ctctcgcttt aaagactggt
361501 tccatattaa ttcttatcct gttgaacctt accgggatcc aagtaaggga gaaaagaatc
361561 cgccatatga aacttttgct tttgaaaagc acatgcccaa attaaatact gctaatccag
361621 aagtacaaga tttcttatta gaaattgcaa cttattgggt aaaatatttt gatattgatg
361681 cttggcgctt agatgtagca aatgaagttg accatcactt ttggaagaga ttccatgacg
361741 aattaattaa aattaagcca gacttctata ttgttggcga aatatggcat tcagctcgcc
361801 cgtggcttca aggagatcaa tttacgggtg taatgaatta tccatatact ttgcaaattg
361861 aggatcattt ctttaagcac aacttagatg ctaaggaact tagtgaacac ttaactgatc
361921 aattaatgat gtatccagat attgtggacc aagcgatgat gaatatgctt gattcgcatg
361981 atacagctcg aattttgact ttagcaaaag gtaatcaaga cttagctttg caagcgatag
362041 cttatgaatt tgctcaaccc gggataccat gtatttacta tgggacagaa atggggatga
362101 gtggcgataa cgatcctgat tgtcgtaagc cgatggattg gagtaagttg aatggaccag
362161 tctggaagag agttcatgaa ttagtcaaat tccgtttaaa acatcgagat actttaaatc
362221 aaggtgacat taagttaacg gtaacagaaa atggtctact aaaagttgaa cgaataggaa
362281 aagaaacaat aaatgcatac tataatacca gtgctcatga tgtgaaaatt gataagaaac
362341 cagcgcttag tcaaaactat gaagacggta ttttagcgcc agatggcttt ttggtagaag

362401 taaaagctta aatttatgat aagaaaaagc agcgataaga ggctgctttt tttcttataa
362461 ttttttgata ccggtaacat gaaaatgatc attgttaagc gctttcagta aatagataat
362521 agacttgtaa acaaaaaacc tcacagaagt gatggagaat gatgatgaag agaatttttg
362581 aagttgatcc ttggaaagta acaacgcata aattttctaa ggaagataaa agattacaag
362641 aaagtattac cgcaattggt aatgattata tgggaatgag aggaaacttt gaagagggtt
362701 attctggtga tactcttcaa ggaacctatt tagcaggtgt ttggttccca gataaaactg
362761 ttgttggttg gtggaaaaat ggttatccaa aatactttgg taagactcca aatgcaccaa
362821 gctttattgg aattggaatt actgttaatg gtgaaaagct tgatttagca aaagttaaat
362881 ttagtgattt tgaattatca cttgatatgc atcaaggtct tctctcaaga agctttgtct
362941 atgaaggaaa agatgtcaaa gttaagtttg aatttgaacg ttttcttcat attattcaaa
363001 aagaagcagc cttaattaaa gttaaagcaa ctgtacttga aggacatgca aaaattgatt
363061 ttgattcaac tttagatgga acagttgtta acgaagacag taattatgat gaacgtttct
363121 gggttccact tggtgaagat aaagatagaa gaactatcca agtaaaaact aagccaaatc
363181 catatgaagt tcctcagttt acggttttgc ttaagcaagc attacgccat aacggggaag
363241 cacttcaagg tgaagtttca agtcaagaag ccaagttaag tgaaaaaatt tcagttgaac
363301 tagatcaaga tcagagctat gagcttgaaa aagacgtcat cgttgtaaca agtcgtgacg
363361 ttgaagagaa agatcaagct gatgttgcag aaaacttaat gagcaagctt caagcaaaaa
363421 gctttgcaga aaacttggct gaacatactg ctacctggga aaagcgttgg gaaacaagtg
363481 atgttgaaat tgctggggat gcagccgctc aacaaggcat tcgtttcaat attcttcaat
363541 tatttatgac ttactatggt gaagatgaaa gattgaatgt tggtccaaaa ggatttactg
363601 gtgagaaata tggtggtgct acttattggg acaccgaagc atttattgtt ccaatgtact
363661 tagccattac taaaccaagt gttactaaag cattgctaga atatcgtcat aatcaattac
363721 caggcgctta tcacaatgct aaggaacaag gtcttccagg tgcgttattc ccaatggtta
363781 cctttaacgg tattgaatgc cacaacgaat gggaaattac ttttgaagaa atccaccgaa
363841 atgctgatat tccgcacgca attgcaatgt acaccgaata tactggtgac gatagctatg
363901 ttaagaacga aggcatggat gtcttagttg ggactgcaag gttctgggca gctagagttc
363961 attggtctaa atggcgtaat aaatatgtaa tgcatggtgt tactggacca aatgaatatg

364021 agaataatgt caataataac tggttcacta gtactatggc aagatggctc
ttgaaatata
364081 cgctagagcg tttgccgctt gcaactaaag aagctcaaga aagagttcat
gtcactgagg
364141 aagaaaagga aaagtggcaa gacattgttg ataaaattta tcttccagaa
gataaagatt
364201 tgggcatttt cttacaacag gatgacttcc ttgataaaga aattcgtcca
gtaagcgaaa
364261 ttgaggatca aagaccaatt aatcaacact ggtcatggga taagatttta
agatcgccat
364321 ttatcaagca agcagatgta ttacaaggaa tctacttctt cccagaaaat
tacactaaag
364381 aacaaaaaga gaagaacttt gatttttatg aaccattaac cgttcatgaa
agctcattat
364441 ctccttcaat tcactcagtt ttagcagctg aactaggaaa gaaagataag
gctgttgagc
364501 tttatgaaag aactgcaaga cttgacttag ataattacaa caacgataca
gttgacggtt
364561 tgcacattac ttcaatgagt ggctcatggc tagcaatcgt gcaaggcttt
gcaggtatgc
364621 gttatgatca cgatcaatta aaattcaagc catttattcc agataactgg
aatcattaca
364681 gcttcaagat taattatcgc ggtcgcttga ttgaagttta tgttgatcac
caagaaacta
364741 agatttcact gcttaaaggt gaaaatctcg aaattttagt taatgatcaa
aaagttatgc
364801 tcaaagaagg tgaaactgaa tgcttaaagg cttaattttt gacttagatg
gggttttaac
364861 taactcggct gtttaccact tgactgcttg gaataatttg gctaaagaat
taggaattaa
364921 cctaacagat gaacagctta atagcttaag aggtatttca agaatggatt
cattaaattt
364981 gatcttgaaa tacggtggtc aagaaaataa atattctgaa gcagaaaaag
aaaaatttgc
365041 tgcagaaaag aatactaagt ttgttgaaca agttgaaaag atgacgccag
cagatatttt
365101 accgggcatt cctgaattac ttagtgatgc aaagaagcaa aatttaaaga
tggttattgc
365161 ttctgcctca aaaaacgcac ctaagatctt aactaaatta ggaattatgg
atgagtttga
365221 tggcattgtt gatccagcta ctcttcatcg aggaaaacca gatccagaaa
tttacgaaaa
365281 agcacaaaaa ttactgggtt tgaatgcaga tgaagttatt agctttgaag
atgctcaagc
365341 tggtgttcaa tcaattaaga gtgctggaca atttgcagta gggattggag
ataaagaagt
365401 cttaaaagag gctgattatg ttgttccaac tactaaagaa ttaaaacttt
cagaaattga
365461 atcagttttt aataaaaaat aagagggaag tagggaaaga gaatggttga
agtagattta
365521 aaccatatct ataaaaagta cgaaggaaat gataaatatt cagttaatga
ctttgattta
365581 cacatcaaag acaaagaatt tatcgttttt gttgggccat ctggttgtgg
taagtcaact

365641 actttaagaa tggttgctgg tctggaagat atttctaaag gtactttaga
aattgaccat
365701 aaagtaatga acgatgttgc tcctaaagat agaaacattg cgatggtatt
ccagaactat
365761 gctttatacc ctcacatgtc aatttacgac aacatggctt ttggattgaa
gcttcgtcac
365821 tacaagaaag atgaaattga taaacgggtt aaacatgctg ctgaaatttt
gggcttgtct
365881 gaataccttg ataaaaagcc atcggaattg tctggtggtc aaagacagcg
tgttgcctta
365941 ggacgtgcaa ttgtgcgaga tgcaccaatc ttcttgatgg acgaaccttt
atcaaaccta
366001 gatgcaaaat tacgtgtcac aatgcgtgct gaaattgcta aattacacca
aaacttagga
366061 actactacta tctatgttac tcacgatcaa actgaagcta tgactttagc
tgaccgagta
366121 gttgttatgt cagttggtca agttcagcag atcggaacgc cacttgaagt
ttacaacaag
366181 cctgtaaata tgtttgttgc tggttttatc ggttctccgc aaatgaactt
cttcaatgtt
366241 cactttaagg gtaatcgaat tagtgacggc aaaggtttaa atatcgaaat
tcctgaaggt
366301 aaggctaaga tgcttaaaga taagggatat gaagataaag acttagtctt
tggtattcgt
366361 ccagaagaca ttcactcaga agaagccttc cttgagactt ggccaaacca
aactgttaaa
366421 tctacagttg tcgtttctga attgctggga tcaactattc aactttatca
aaaagtagat
366481 ggcacagaat ttgtagccat cgtaaatgcc cgtgattacc atactccagg
cgataaggta
366541 gaaatgggat ttgatgtcaa caaagctcac ttctttgata aagatacaac
gaatgccatt
366601 cgttaacttg cagaagagga cgcttctgca aatattaatt aagcttacaa
cgagtgtgaa
366661 acttggttca cggttgtaat aatgtgttta gccttttatg gctaggagga
aatatcatga
366721 agttatggaa aaaattggct ttaggtagtg tcgtagcttt atcagctatt
tcattaactg
366781 cctgtggtaa gagttctaat tcaaacgaat caaagagctc agataaacaa
ctaacccttt
366841 gggtagatac tgctcgtgtt agttggtaca agggtgttgt aaaagacttt
gaaaaagaac
366901 accctaacat taaagttaag gttacccaaa accctaatgg ttctgctaat
gccaagactg
366961 acgttggtaa ggacccttca aaggccgcag atgtttttgg tgttccaaac
gaccaattag
367021 gtcaaatggc agattcaggt tatatcaacc cactttcacc atctgatact
aaagaaatta
367081 agaaaaactc agttccagaa gcatataagg gtgctgaatg gaaaggtaag
ctttacgcat
367141 atccatacgc agaacaagca caaaccgttt actacgacaa gtctaagctt
tctccagaag
367201 atgttaaatc ttggaagacc atgacttcta aaggtgtagt tgcaaccgac
tttactaacg

367261 cttacgtaat gtggccagta atgttctcag caggcactaa gctatttggc
gacagcggag
367321 aagaagtaaa gggttcaaca tttgactcac aaaatggtgt taacgcaatg
aagtggtatg
367381 cagaacaaaa gaataacaag ggtgttatgc aaacctctaa cgcacttaac
caattaaaga
367441 agggtcacgc tcaagctatc ttagatggtc catggaatgc agcaaacatt
aagaagatct
367501 taggtaagaa cttgggcgtt gcaccatatc caactattga acttggtggc
aagactgctc
367561 aaatggaagc tttcttaggt attgaatgtt tcgcagttaa ctctcatact
tctaacgcta
367621 agaatgctgc aatattggct aaatacttat caaacaaaga aaaccaatta
atcgttcaca
367681 agaatgctgg tgaaattcct gtaaacaagg cagctcaaaa tactacagaa
gttaagaatg
367741 atccagttgc taaggctgta attcaaatgg ctcaaccagg ctactcagtt
cttcaaccaa
367801 aattgccaca aatgtcaatc ttctggaacg actcagctcc attaattagt
ggtgcatacg
367861 acggcaagat taagccatca caatacaaga ctaagcttgc taaattacaa
aagagtattt
367921 ctaagaaaga ataatcttaa aaaatcacat aagcaaaggt ttgattaatt
atgtttaaga
367981 aaaaacatgc tacgcctgcg gttaccttaa aggaaacgtg gaaagatgga
gataccgcaa
368041 ccaaattaac attcttcatc atgggtttaa atgctataaa gcatcggcaa
tggctaaaag
368101 gattttcgct tttattttcc gaaattgtat ttttaatttg gttcttttta
agtggaattc
368161 attcaattgc tggcttgagc aatttaggtg caataaaaac gaaacgagtt
atatttgata
368221 aagcacaagg agtatatgtt actcagcaac cagataattc agttttgatc
ttattatttg
368281 gtattttggc tttattcatc gttgctggct ttatttacct atatatcatt
aatctgaaat
368341 ctaataagca tactttctta ttagaaaaaa gacatgaaca tattccaact
aaccgcgaag
368401 agattgcatc tttgtttgac caaagattac atgcaacctt gatgacaatt
ccaattgttt
368461 taattatctt atttactgtt ttgccaactg tcttcatgat ttccatggcc
ttcactaatt
368521 atgatcgtca acactcaatc ggattttcat ggactggatt ccaagcattt
ggaaacgtgt
368581 taggaggaga cttagctggt actttcttcc cagttctagg ttggactttg
atttgggcag
368641 tggcagctac tgctactact ttcttctttg gagttttgct tgcacttttg
attgaatcaa
368701 aggggatcaa gcacaagaac ttatggagaa caatctttgt aattgtctat
gctgtaccac
368761 aattcgtttc tctattaatg atggctcaat tccttgatta tcaaggacca
ttgaatacat
368821 tacttatgaa ctggggctgg attagccacc caattcactt cattgataat
caggctagtc

368881 cacttgtcgc aagaattaca gttatcgtgg ttaacatgtg gattggtatc ccagtttcaa
368941 tgctaacttc aactgctatt attcaaaact tacctcagga ccaaattgaa gctgcacgta
369001 ttgatggtgc aagtccagta caaattttta atcacattac attcccacaa attctctttg
369061 taatgtctcc agctttgatt cagcaattta ttggtaacat caacaacttc aatgttatct
369121 acttgctgac tggtggtgcg ccaatgaata ataactacaa cggcgctggt tctactgact
369181 tgctagtaac ttggctatat aacttaacat ttggtcaaga gcaacgctac aatgcctcag
369241 ctgtcttagg tatcttgatc tttatcatta gtgcaacatt ctcactaatt gcatatcgtc
369301 atactaatgc gtacaaggag ggctaaaaat gaagtcttat cataatcaaa gaaagattgc
369361 attaatcttt agatatgttc tattagccat cttggccgtt ttatggatat tcccaattat
369421 ttggattatt ttagctagtt tttcatacaa caacacagga ttcgtatcta ccatttggcc
369481 agataagttt acttggcaaa attatattgg tatctttact aactcacagt atccatttgt
369541 caactgggta ttaaatacat tgtttgtcgc tgttatctca gcaacattat caacttttgt
369601 tacaattgcg gttgcttacg tattatcaag attacgtttc cgcttccgta agccattctt
369661 acaaattgct ttagttttag ggatgttccc aggcttcatg tcaatgattg ctttatacta
369721 catcttaaaa gctttgaaca tgttaaacct tggcggcttg atcttagttt atgttggtgg
369781 tgcaggatta ggtttctaca ttgctaaagg attctttgat acaatgccac gtgcaattga
369841 tgaagccgct gaaattgatg gtgcaactaa gtggcaagta tttattcata ttggattgcc
369901 actttcaaaa ccaatgatcg tttataccgc acttacttca ttcatggctc catgggtaga
369961 cttcattttc tcaggtatta tcctctctac ctctggaaat ccgaagactt acacggttgc
370021 gtatggttta tacaacatgg tccactcatc aaaaggtatg atggctcaat tctttactca
370081 attcattgct ggttgtgtgg ttatcgctat tccaattaca attttgttca ttgtaatgca
370141 gaagttctac gttaacggaa ttacagcagg tgctgataag ggttaatcaa attaaagttt
370201 atgtgaaaaa agtgttctgc gtttgcagga cactttttat ttaaaggagg aaatttatga
370261 caccatggtg gaaaaaagca gttgtttacc aaatttatcc caagtcattt caagatagca
370321 atggagacgg aattggagat attcctggga ttgtctcaag acttgaatat ttagaaaaat
370381 taggaattga tgcaatttgg ctgtcgcccg tttatttatc gcctgggatt gataatgggt
370441 atgacatttc tgattatcaa aaaattgacc cacaatacgg cacgatgaaa gatatggata

370501 atctaattaa agaagctaaa aaacatcata tacggattat tatggacctt gtcgtaaatc
370561 acacttctga tcaacatcct tggtttgttg aagcaaagaa gagtaaagat aatccatacc
370621 gtgattttta tatttggcgt gattcagtag atggacatga acccaacgat ttaaaatctg
370681 ctttttcagg atcagcttgg aaatttgatg aaaaaacagg acaatattat ttgcattttt
370741 ttgcagatca acagccagat ttgaattgga agaatccgaa actaagaaat aaaatttatg
370801 acatgatgaa ttattggatt gataaaggaa tcggcggctt tagaatggat gtcattgaac
370861 taattggtaa agagccagat caacgaatcc gtgaaaatgg tccaatgctt cacccatatt
370921 taaaagaaat gaatgagcat acctttgccc gtcaagatat gatgaccgtg ggtgagactt
370981 ggaatgcaac gcctaaaatt gcagaagaat attctgaccc agcacgtcat gaattgtcga
371041 tggtctttca atttgaaaac caaaatttag atcaagagcc aggaaaagaa aagtgggatt
371101 taagaccgct tgatttggga gaattaaaaa aagtattagt taaatggcag acagaaattg
371161 attttaacca tgcttggaat agccttttct gggaaaacca tgatattcca cgtgttattt
371221 cacgttgggg aaacgatcaa gagtaccgcg ttcaatgtgc taaaatgttt gcgataattt
371281 tacatttaat gcatggaact ccttatattt ataatggtga agaaattggt atgactaatt
371341 gccctgttaa gtctattgct gaggtcgaag atattgaaag tatcaatatg tatcatgaaa
371401 gactagagca aggatataaa aaagctgatc taattaagtc aattaacgcc aaaggccgcg
371461 ataatgcaag acggccaatg caatggtcta atgaagagaa tgcaggattt actagtggaa
371521 aaccgtggtt aaaagtaaat ccaaactatc aaaaaattaa tgttaaagct gctctagcag
371581 atccaggttc aatttttat acctatcaaa aattgattaa gcttagacat gaaaatccag
371641 tcgtagtcga tggtgatttt gaactcgttg aaaatactgg cgatagtgtg ctagcctttt
371701 ggcgtaaatt aggaaatgag aagtggctag ttgttgctaa tttgtctggt gaaaaacaag
371761 gatttaattt agatagtgat ttcaaagaag ttttaattag taattatgaa aaacgagcta
371821 gtttaaagaa tattgttcta aagccatatg aggcttttgc agttaaggcg taaagaaaaa
371881 gtacattgat ttctgcaatc aatgtacttt atttcataac tattttttct tatttggacg
371941 tcttaagtaa cgcaatgtat tgtttctgat atacttttga aattcttgat aaattggatc
372001 aaaaatttgc ggctcatcag tttctttaac cattacttta ggaaagaaga aacgttcgtc
372061 tccttgaagc gtaccattta aggatttaac taggggactt aactcagata gttctactaa

372121 gctaccatca gcttgcatga tttcaattgg gctatgagca tttttaccgg tgggcttgta
372181 agcgtcatat ggttcatcaa atgcagaatt agtggcagtg tagtagttag gatcaaaacc
372241 tgcttgttta attaaattct ttaatttggg tagtaaactc ttagtatctt cattaatctt
372301 gacactttca aggggatgac gatataaata acggcttgaa agatctgata gaatttggtc
372361 acctgaatta gtccataata gaaaattggt ttccattacg ccatcatcta aattcagata
372421 atcttcaagt gtccaatttc cttttagaaa ggcctctagt tgtggagtaa cttggagctt
372481 acctgcttca tatatgattt gcgctctttc aagtaagtga tgcaagatga cttccattga
372541 acgatttacg cggtgaaagt aaacttgttg atacatctgg tagcgactaa taatatagtc
372601 ttctacagcg tggataccct tatcagtgaa gcaaattcca tcacggtaag gacgaatcac
372661 ttctaaaatt ctagttaaat caaaactgcc gtaggttact ccggtaaagt aagcatcgcg
372721 aagtaagtaa tccattctat cagcgtctgc ttgactagaa attaatttca caacttgtgg
372781 gttagagtag gtcttggcaa ttacgctagc tactaattct ggaaaattag gactaacttg
372841 tcttaaagct tgattgactt cagtattttt atcagtaata atttgttggc ccattttttc
372901 atggttagta ccaaagagat gctcaaaggt atgagaatat ggaccatggc caatatcatg
372961 aagtaaggca gcacattctg ctaaaagacg ttcgttagga tcccataacc catctccggg
373021 ttctttgcta gtatattttt cttcaaaaat atcgcagatt cggcgtgtta gttcataaac
373081 acctaagtta tgttcaaagc gggtatgtgt tgctccctga aaaacatagg aagcgggtcc
373141 tagctgttta atacgacgca ggcgttgaaa ttcttttgaa ttaataatgt ctagaatgac
373201 tttatcttta acatgaatat aattgtggac aggatcacgc aatacttttt catgatccaa
373261 ctttttactt tgaaattttt ccatttttct tatactattt tctcggataa taaaataatc
373321 ggttaactct taattaactt gattataaca ataatgctac tgtaaggcta tcttagcatg
373381 gatgaaggtg aaaataatga ataaagaagt tgagataaag attaccagtg aaattcatca
373441 agaagatgat tcggctgttt ttgaaagaga aggtttggga agccttgaaa gaattggtaa
373501 taattggcga ttaaagtata acgaaaaaaa caaggaaggc gacgttgagg ttaaactatt
373561 aattaagcca aatgaattag taatgcagcg aggagatatt aaaggcgatc atacattaat
373621 gaaatttgaa ccaggagaga aacaaaaatg taaagttatg gctgctggta agcaaatgga
373681 tttagtatct ttgaccaata aattagattt ttccaaaatt tctccaggaa agatgcaact

373741 taaagttgaa tatgatctct tttctggtct atacttagta ggtaactatg caataaagat
373801 tgaagtaaca gatctttagc aaatttagat aaattgatta cgtgaaagga cgtgccactg
373861 tggggttaga tgattttaaa ggccaaaata aagacgaatt gtcaatgatc gaagttgctc
373921 gtgcaatttt acaagatagc ggcaagcgaa tggcctttgc tgatatcgtt aacgccgtcc
373981 agaactttct gggtaagagt gacgaagaaa ttcgtgagcg tttgccacaa ttttacactg
374041 atatgaacac tgatggtgaa ttcatttcaa tgggtgataa tgtttgggca ttgcgttctt
374101 ggttcccata tgaatctgtt gatgaagaag taaaccatcc agaagatgaa gatgatgtgc
374161 cacgtaagaa gcaccacaag aaggttaatg cctttattgg tgattctgac gatgatgaca
374221 ttattgacta tgattcagat gatccagaag atgaagattt ggacgttgat gacgacgaca
374281 ataatgaaga tgattactca gatgatgatc ttgacgatgc tgacgacaat gaattagatg
374341 acggtatcga aggacaatta tcagaacttc atcaagatga tcttgatgac gatgacgatt
374401 aaagtacttg acgggaagag aatttccctt tagtattttg tatgggcact ctgtgtgcat
374461 ttaagctccc atgaacttgg gagctttttt ttgattttaa cgaataataa aagaaaagag
374521 aggaagcacg gcatgacaaa gtatattttt gtaactggtg gggttgtttc atcattaggt
374581 aaaggtattt cagcctcaag tttaggacgc ttacttaaaa atcgtggttt aaaggttacc
374641 atgcaaaagt tcgatcctta cattaacatt gacccaggta caatgaatcc atatcaacac
374701 ggtgaagttt acgttactga tgatggtact gaagccgacc ttgatttggg tcactacgaa
374761 agaattgttg atgttagaac aagtaaatat tctaacgtga ctacaggtaa gatttaccaa
374821 gaagtacttg ataaagaacg tcgcggtgac tatcatggtg caactgttca agtgattcca
374881 cacatcactg acatgattaa gaaaaagatc atgcgtgctg ccttaacgac tgattcagat
374941 gtaattattt ctgaaattgg tggtacagta ggcgatattg aatcaactcc atttatggaa
375001 gcaattcgtc aaatgcgtcg tgaagttggt gaagaaaatg taatgtacat ccactgtact
375061 ttagtacctt atcttcatgc agctcacgaa atgaagacta agccaactca acactcagtt
375121 gctgaactta gaagtattgg tattcaacct aatatgcttg ttttaagagc agaaaaacca
375181 gtacctcaag atttaaagaa taagatttca acttttacag atgttccagt agatcgaatt
375241 attgagtcta ttgatgctcc ttcacttttt gatttaccac ttgcctttca agcacaaggg
375301 atggatcaaa aggtttgcga tttcttacat ttagaaagtc caaagccaga agctgatatg

375361 gaggcttgga agaagcttga tgagcgtgca aagaacttga aacacgaaac
tactattact
375421 ttagtaggta agtatgtaga gcttgaagat gcatatattt cagttactga
tgcattgcaa
375481 cacgcaggct acttgtacga taccaagatt aaagttaata aagttcaagc
agaagatatc
375541 actgaagata atattgcaga aatcatggaa ggttctgacg gtttaattgt
acctggtggt
375601 tttggtacac gtggtcttga aggaatgatt acttcaatta agtatgctcg
tgaacatgac
375661 attcctttct tgggtatttg tttaggtatg caaatggcaa gtgtcgaatt
tgctagagat
375721 gtacttggat taaaagatgc aaatagtgca gaggctgatc cagaaactaa
gaataatatc
375781 attgatatca tggctgacaa gcgtgacgaa gaaaatatcg gtggaactct
tcgcttaggg
375841 ttatacccag caactttaaa gaagggtacc aagactcgcg cagcttatga
tgatcaagat
375901 gttattcaag aaagacaccg tcaccgttac gaatttaaca acgattatcg
tgaagctttt
375961 gaaaaggctg gcatggtctt ctccggcgtt tctccagata accacttagt
agaaattatt
376021 gaaattccag ataagaaatt cttcattgca gctcaatacc acccagaatt
tttaagtcgt
376081 ccacaaagac cagagggttt attcaaagcc tttattggtg cagctagtgg
tcttccagca
376141 caacatttta attaggcttt tcaaaaatta aatttcggta acaagagggc
cataatacac
376201 gggttctctt gttttttttg cgtttttcat ttaatatatt ttgtgataca
tttgggtatt
376261 acaaatagag aaaaggattg tttccccaat gaaacagatg attattcatg
gtggaaagcc
376321 cctgaagggc gacgtatgga tcggcggtgc taagaactcc actgtagcac
tgattccagc
376381 gtcaatactt tcgcggactc ctgttaccct agaaggggtt ccgcgaattg
ctgatgtaga
376441 taatttgatg gatctgctta gtgaaatgga tgtgaagtgt gattttcacg
aaaccacttt
376501 acggataaat cctgatgaca ttaagcgtag tccccttcca gcaggtaaga
ttaagagttt
376561 gagagcttct tactacttta tgggtgcatt gttaggtcgt tttggtaaag
ctgtagtagg
376621 ctttccaggc ggcgatgata ttggccctcg tcctattgat caacacatta
aaggttttga
376681 agcactaggc gcaactgttg aaaatgagaa cgatcaaatt atcatcacag
ctcctaagac
376741 tgggcttcgt ggtgcaaaaa ttcatttaaa gatgccgtct gtcggcgcaa
caatgaacat
376801 aattatggct agcgtaatgg ctaaggacaa aactattatt gaaaatgcag
ctaaagagcc
376861 agaaattatt gatttagcaa catttttaaa taatatggga gccgttattc
gtggtgctgg
376921 tactgatgta attcgaattg agggagtaga agagttaaaa gctcaaactc
cgcatactat

376981 tatccctgat cgaattgaag ctggaaccta cgtggcttta gctgcatgca ttggtgatgg
377041 aattagaatt cataatatca ttgaagaaca ccttgattca tacttagcaa aagtagaaga
377101 gatgggtgta gtaattgatg cggacgaaga ttcactttat gtgtatccag caggtgactt
377161 aaagatggtt caggttagaa ccgacgtcta tccaggattt gcaactgact tgcagcaacc
377221 aattactcca cttcttttaa ccgctaagac gggtgagggt gtagtaattg atcagattta
377281 tcctaagcga gtaggacata ttcctgaact tcaaaagatg ggtgcaaaca tccatgttga
377341 agataatatt attttagttc acccaactca tcatttacat ggagcacacg tcagcgcagg
377401 tgaaattaga gcaggagcat gtttaatgct agctggttta atggctgatg gcgagactat
377461 catttcaaac gcaggtaaca ttttgcgtgg ttatgataga attgagcaaa agttgcgtca
377521 attaggtgca gaagtatctg taattgatgt ttaaagtttt tgaaaataga aaaataattt
377581 aaagataaaa aggttgatag tattttgttt aagaacaacg atattatcaa ccttttttgct
377641 ttgtaaccga aataatgcaa aaaacatagc tgattattaa tttaaaatga aaatatttgt
377701 tgaatttaat aaaacttaat gtaaaatata atctatcaat tgggaaactg aggtgaatta
377761 gctaatgata aaatcatttt ttaagtctaa ttataaatat acttttattt ttattgtttc
377821 gactgttatt ggcggggcct tagttgctta tggaagctat ggcttaactc ctgcgactga
377881 ttccttaaaa agtcttgata ctaaaaaatt tattttatgg attgttgtaa taacaatcgc
377941 atcagctggt ggtagagtat tacaattctc agctggttat ctctttgcaa aacaggagca
378001 ggaatatttt catagtttaa ggaaaaaact tataaatcat tattatgaaa gtacatctat
378061 agataagcta tctaatatgc aaaatagatt aacaaatgat ctaaaaatta ttagcgatca
378121 agctttagat ccagtatcta atgtagtcct ttgtgtagtt gatttagtat tttctgcata
378181 tgtagtttta acttttaatt tttggctgct ttgtttaatt ttagttttag cagtaattat
378241 gctttatttg ccagatttgt tgtctaagca attagaaaaa gcaacaaatg aagtttcctc
378301 taactatagc aagtatattg ataccattga aaaatggtta gcagggctag cagttttaca
378361 aagatatcgg tcaaggacaa aattatctgc tgccttagct agaagttctt caggattaga
378421 aaaagccaca gtaattcgag ataaaaaatc tgttgaatta cgtaatctca cttatactgc
378481 taatatggtt gctcaaggag ctgttcttct ggcaactgga ctattagtat tgaatcatca
378541 gttaactatt ggtactctta tgagtattgc gaatttttct agcttgattt tttcccagct

378601 tgttaatgtt acttcacaga ttggttcaat caagtcaact agatcgttag
cagatagtgt
378661 aatgaaagaa ataaaagagt taccaaattc agagaaaaaa gaagactctc
taacaaactt
378721 tgacactctt tctactcatg gtttagttct ccagtatcct aatggcgaaa
aaatcaagta
378781 tcctgatatt gagattaaag ctggagaaaa agtactttta agtggtgaca
gtggaacagg
378841 taagtcaacg ctgtttaaaa tgattttaaa tgaaataaaa cctactcaag
gtaaaattgt
378901 ttttaaagat aagctaggca agattctcaa tcctgattat tcgcaaattg
gatatattcc
378961 gcaagatcca gttgtttttc caggaacaat tgaagataat attacgatgt
ttaatagtag
379021 actgaataaa aaggcagcga attgggcaca aaaggttcag ttagcaacag
actttgaaaa
379081 gtttcctgat ggtattaaaa ctgttgtaga tctagataaa aataatcttt
ctggagggca
379141 aagacaaaaa ataattttgg ctcgaactga agtatataac agtaagatta
tcttaattga
379201 tgaaggtact tcagcaatag attcggttgc gacagagcaa attctaaaaa
atattcttaa
379261 agaacctgca acggtagtct ttatcgcgca taatttaact gatgaaatgg
ttcaattatt
379321 tgatcgtagg attaacttaa gtaagtaaaa aggacttgga aaattccaag
tcctttttgt
379381 gtggttttaa gaaaaatttt taattagtgt tgtacctcaa tttttctgg
tgcagcaggg
379441 atatgctgct cttctaagcc agattttcct ttaaagagga ggttgaggat
agtagcactt
379501 aagctagcta caacaatacc gtttccaagg aataactgaa cagtttcagg
taaagattgg
379561 aaaacttgcg gataaacagt aacacctaat cctaaaccaa ttgaaatggc
tacgattaag
379621 atgttgcgag tatcatcgaa atcaactcgc ttgagcatcc gcattccttg
gacggcgatc
379681 atagtgaaca ttactagcat tgcaccgccc aagacagaat caggaataat
agttactaaa
379741 gcaccaaatt ttggtaatag tcccattaac attaaaaatc cagatgccca
gtaaattggt
379801 cgtttagtgg tgatgcctga taattcaagc aaaccaacat tttgagaaaa
agtagtatat
379861 gggaaagtgt tgaagatacc accgagaatt tgagctaatc cttcagcacg
gtagcccttc
379921 ttgaggtcat cgcttgagat atctttgtgg agcaagtccc caatagcaaa
gaaaacaccc
379981 gttgattcaa ccattgaaac tagtgcaatg ataatcatgg ttaagctaga
tgaccactca
380041 aattgtggca taccgaaata aaatggttgt ggtaagtgga accaagaagc
ttgtgcaact
380101 ggtgttagtg ataccattcc aagcatactt gcaattagag ttcccgcaat
taatccaact
380161 aaaacagcaa ttgatttgat aaatcctttg ccccaaacct gtaatgccaa
aataattgct

380221 acagttataa aaccagttaa aagattttg ggatcaccaa agctcttagc
agcagaattt
380281 cctccgccaa gattttggat agatactgga attaaagaaa gcccgataac
tgtgattaaa
380341 cttccagtta caactggtgg aaacaatttt ttgatttttg aaaaatatcc
tgctactaag
380401 aagacgaaaa tacctgcgac aataatagcg ccatacatcg ttccgatagt
aaatttctta
380461 ccaatcattt gaagaggagc aactgcttga attgcacatc ctaagacaac
tggcaagcca
380521 attccgaagt agcgattacg caagagctgc agtgcagtgg ctaaaccaca
cataaaaata
380581 tcaattgaaa ctagataagt catttgtgtg gagttaaatt ttaaggctgt
accaattaaa
380641 agaggaacag cgatagctcc agaatacatc gccaataaat gctgcaatcc
taataaagct
380701 gctttttgt ggttaacttg catatgcttc atttattttt ctcctacaaa
atgtacttga
380761 ttattttcaa agttagcaat tctagctagg gcttcaagac gatagccatg
ttctttaacc
380821 cagtcgctac caccttggaa ctctttggct actaccacgc cgacgcctgc
tacagatgca
380881 tctgcttgcc tggcaatatt aagcattccc ttaacagctt caccgtgtgc
taagaaatcg
380941 tcgataatta ataaatggtc atcactactt aaaaatttct tttcaacaca
aattttattg
381001 ttaacctttt ttgtataaga aaatacatcg gcccagtaaa cggcatcatt
gagagtagat
381061 ggctttttct ttcttgcaaa aaccattgga acacccaatt gataagctgc
cataatacca
381121 ggtgcaattc ctgaagcttc gcaggtaaga actttagtga ttttttcgtt
ttcaaataaa
381181 cgtttgaatt ctgctcctac ttgcatcata agttccggat cgacttgatg
attaagaaaa
381241 gaattaatct ttaagacgtc accatctagt acttcaccat ctttgcgaat
ccgttcctca
381301 agtaatttca atttgttcct cctgcataaa aaagacttct tctacccgtg
aatttagggt
381361 aaaagaagtc tattgtagtg ctacttatga catcgattac ctatagtcca
gaattaattg
381421 gttccgggta gaaactgtcg accttattac gacgatatat agatattttg
atatttagag
381481 tgtagcagaa tcattttccg aacacaagtg ccttttagc gatgaatcat
tctgaaactg
381541 ctttttcttt ttttagcgac atgactgctt caaagattcc atatgcaatt
ccagctaatg
381601 gccaaatgat ccagctctgg ttccagttat tacttaaaaa actataagca
agatagataa
381661 aagaaattat catccagtaa atagttgcgt atttttcaat caacttttta
ccagcttttt
381721 tctcacttgt gtagtcatca agttgtaaga taatattgaa actatcatga
atgatattgg
381781 ttttaactaa gaaaaagacg ccaatgccaa cgagtaaaag cgtcgctgaa
gaaaagccag

381841 tcattaagtc atcaagtcta ctgctagcaa gtgcatctac aaataaggcg ccactcatta
381901 acggaatagc agacaaaatg caaaaagtaa tgccaataat tttaaaaatc atatgttggt
381961 tttcatagac tttgttttct ttgataattt tttctcttac attgtttgaa agattacagt
382021 cttcatattc gtaattttca tgaactttaa gccatcgatt cccagctatg aataacgcaa
382081 ctgctgtggc gataaaagtt agtaaaacaa ttactccaat tccagttgca aaattgattg
382141 accaggttaa gatgtctaat ctagtaagac taattaaaat taataagaca attggtgaga
382201 aaatacatag cattacgcca ctagcaatta atgtagaact gcgtcttctg gcagataaga
382261 aatcttgggt agcagaataa gatatgtaat tttcgttaga catgataatc acctctttaa
382321 tatataaata gaagtttgta tagactattt atattttatc gatcaagaaa tgaatacgct
382381 attttaatgc ttagggatag aaagataaaa gaaatgattg accaattgaa tttttcttag
382441 taaagtttct agtaaatagg gatgaattat tttagaagga gaaaattgtg gctaagacta
382501 atctaaatga ctttgacaag attatcgtgc ttgattttgg tagtcaatat aaccaactga
382561 ttacccgtcg aattcgtgat tttggtattt attcagaact tttgcctcat gacttgagca
382621 ttgaaaagat taagaaaatg gcacctaaag gaattatctt ttcaggtggt cctaacagtg
382681 tttatgatga aggcgcatta aaggttgatc ctgaaatctt taagctaggt attccaattt
382741 taggtatttg ttacgggatg cagctgatga gttacgactt aggtggcaac gtagaaaagg
382801 ctgataattc agaatatggc cgtgcagata tcgaagtaac tgatccaaat gcggtcttgt
382861 ttgaaggttt acctaaagag caatatgtat ggatgagtca tggagattta gtgactaagg
382921 caccagaagg ctttaaagtt actgctaaga gtaagaactg cccaatttct gcaattgcta
382981 atgatgaaaa gaaattctat ggaattcaat tccatgcgga agttcgtaat tcagaatatg
383041 ggttagatat tttaaagaac tttgcattca atgtttgcga tgcaaaagct aattggacaa
383101 tggacgattt cattgacatg caagttgatg aaattcgtga aaaagtcggt gataagaaag
383161 tcatcttggg cctttcaggt ggtgttgatt caagcgttac agcaacactt ttgcacaagg
383221 caattggtga tcaattgact gcgatttttg ttgatcatgg gatgcttcgt aaggacgaag
383281 gcgaccaagt aatgcaagcc ttgaacaagg atcttggtgt aaacattatt cgtgttaatg
383341 cacaaaagcg ctttttaggt aaacttaagg gcgtaactga ccctgagcaa aaacgtaaga
383401 tcattggtaa ggaatttatt gaagtcttta atgaagaagc taagaagctt aaggatgttg

383461 atttttagc tcaaggtacg ttatatactg atgtgattga atcaggtact aatactgctc
383521 aaacaatcaa gtctcaccac aatgttgggg gcttgcctaa ggacatgcat tttgaattga
383581 ttgaaccact gcgtaagctt ttcaaggatg aagtgcgtga acttggtgaa aaattaggta
383641 ttcctcatga cttagtatgg cgtcaaccat tcccaggtcc aggtcttggt attcgtgtac
383701 ttggcgaagt taccgaagat aagctcaaga ttgtgcgtga aagtgacgca attttacgtg
383761 atgaaattaa aaaagcagga ctacaagaag atgtttggca atacttcaca gtacttcctg
383821 gtatacgttc agttggtgtt atgggggatg gacgtactta cgactacact attggtatcc
383881 gtgccgtaac ttcaattgat ggaatgactg ctgactttgc aaaaatacca tgggatgtat
383941 taagtaagat ttctacaaga attgtagatg aatgcgatca tattaaccgt gtagtttacg
384001 atattaccag taagccacct tccacaattg agtgggaata atagaatcta caataaagct
384061 cgcaattaac tttgcgggct tttagttttt aaaatatgct ataatttaag taaagaaaaa
384121 ggagatccga agatctcctt tgcagcccgc tttaagagcg gtggtcgaca ttataaaaaa
384181 acgttgaaaa cgacgctcta taactcgcca aagttataga gcgtcgtttt tgtttttgt
384241 ggtggtcgat ataagttagc agagctaaca aaaacgtacc aaacaaaagc attaaagaaa
384301 tactttcgta aaccgacatg gttgaaccct ttctgcagtg tattgaccat aggcctcacc
384361 tccgggagga aaaacaaccg ccgctcataa aactttctgc ggaaataatt atacagaaac
384421 tgtgtttgta aaagaagacg gacaagataa aaagctcgca attaactttg cggacttttt
384481 taatgaacat catcgcgtgg tgttgatact attcttattt atggtataac tagtttataa
384541 aattttggag gatttgatta aatgaatttc aaaaaacaat tattttttgg gggggcaat
384601 tgcactcctt actgtcggtt taacaggttg tgcaaataat acttcttcta atagttctaa
384661 caatcaagct acgtctagta aatcaagtaa aactaatcaa agttcaagcc atgagttaac
384721 tatcacggat atagttggaa catatgtaaa taaagataag acagccgcag ttgtgttaca
384781 aaatggacat acattatggt attaccaaac taagaaccat aatgatggtt caaaaaatga
384841 ttctggtaat tggcattacg aaggtcacaa taaattttca attaccatgt cacacagaga
384901 tgataataat caagaagtag atactaaagg ttctttggtg ttagatggta aaactttgac
384961 aatgactgaa aataataatt cagctaaaca agtctttact aagacaagta atgaggaaaa
385021 agaatatgat gagatcttta atcccggtaa aaaagtagac ttggatgatc catcaactag

385081 cgcaaataaa aatgatcaaa gttcaaataa ttaactttat ggttaataaa ttaaaaagct
385141 cgcaattaaa tttgcgggct ttttgagtaa attattcagc tttttcttta ataaagaagt
385201 atgtaaatag acagtaaaca ataatactga agtctatgaa aagtgtagga ttaaaaaagg
385261 gtatatacaa gccataagga agtctagtag taatataaaa aatacccagg acacagaaag
385321 cataaatcca actgccatag acaaacaatc tgtggagctt aattggcaaa tgctttttat
385381 gcttaatcaa aatagaaaga taatttcttt ggaataaata aaaagtaaat aaatatgaag
385441 caccactaaa aattataaaa gttattcccc aggtaataat aaatcctatg ctggagcttg
385501 aaaaattact gaaccaatta atcattggtc cgataatact taaaacggct aaaatactag
385561 caaagactac gtatttatca ttattttggt gaaataattt aattgtgaat tctaatttct
385621 ttctctcttc agtcaaatca aagtgttgct ttttgatttg tttgaactca cgcatattta
385681 atagactata aataaaaagt cccaatgcaa tggcaatagt aataatagcc gctacgtagc
385741 cactgagaga aattgtgaaa gttagaggag tttcatagtg actgtcgtgg tcgaagaaga
385801 tagataagat acatgcaaaa catatccaca caggactaaa aataattaag gcaatagcta
385861 aagcacgtaa tttggaactt ttctgactag cagttaaata ttttttaact aattcatcag
385921 ataaaacagg taatttatct ttatcattaa tatcttcatt tttaagctca aatgagggct
385981 cgccgctctt taatagatag tcagttgtta caccaaataa ttctgataaa ttaactattt
386041 tttcaatatc tggaattgat tgttcacttt cccattttga aactgcttgg cgactaacgt
386101 tcattttttc agctaagctt tcttgagaaa gattattctt tttccgcaag tctgcgattt
386161 tttgacctaa tctcataata gatcgtcctt tctgttttta ttgatcttaa tttataaaaa
386221 atcttggttg cagacaacca ctttttgatt gcttagcacg caactattag ttgcagccat
386281 ctatttatag gctaaatgac gccacatgta tctacaaaat gataaaatgt aatcacataa
386341 aatgattggg aggtagtctt atggactttt tgaaaggtaa agaaaataat aaaacagcca
386401 aagtttttaa agcaggaaat tcagaagcaa ttaggttaac taagcaagat actgctatct
386461 ttaaagttaa acctgaatat caggtaatca aagaggtcgg atgtcgatta cattaaaaaa
386521 gcctgaagta tcagacgatg taaagaaaaa agttagcgag atcttaaatg aagatgcaga
386581 tttaattcaa gcattaaagg atttgtaaag atggaaacgc atgatccatt aataaaaata
386641 tgctataatt tgtgtaaaga aaaaggagat ccgaagatct ccttgcagcc cgctttaaga

386701 gcggtggtaa aaattataat actttgtacg aaaaatcgtt caatataacc ctaccaaagt
386761 tatagtattg agcggttttt tattttttgt ggtggtcgat gtaggttagc aaagctagca
386821 aaaacgtacc aaacaaaagc attaaagaaa tactttcgta aaccgacatg gttgaaccct
386881 ttctgcagtg tgttgaccat aggcctcacc tccgggagga aaaacaaccg ccgctcataa
386941 aactttctgc ggaaataatt atacagaaac tgtgtttgta aaagaagacg aacaagataa
387001 aaagctttca attaatttcg cgagctttta atttttcttt acttcttgag ataacaatgt
387061 tatcattttg actaaagata acatcgttat ctaggaggtg catatgaata cacaagataa
387121 gttggttggt aagactatta ctgtcataga caaatatggg tatcaaaatt taagtttaag
387181 aaagttaaca gctacactgg gattaactac aggtgctttt tataagcatt ttaaaagtaa
387241 agacgcctta tatgaaaaag tagcaattga attgtcacgt aattttattg ataagatatc
387301 tttacctgat aatgcgacag ctcaagaaag attattaata attgctaaaa acttttgtca
387361 gtatgtagat caacatagta atttagccga atttttgttt tttaatccaa ctactttaaa
387421 agcattttct actaaaagat caaaatatga ttttttaaat aaaatgaaag ttttagttaa
387481 tgaactaaat ccaaaaaaga atgtatcaaa tcaagagcta tttatccaaa tatggtcatt
387541 tattcaagga tatgctgctt tagttaataa taaaatagtc gaatacgacg aggatttagt
387601 gaaagggaca ctagatgagt ttttacagta gaaaagagaa tattttatgg attgcttgat
387661 tatttattgt catccctacg ataaaagttt taatcatgct gtactggaaa atgtagaaaa
387721 taatttaaaa caaaataata aggattacaa attaatagat ttatacaaag atggttttgt
387781 tccaatttat gatctcgaag aaatgcgatt gtttcatacc ggacagacgc atgatccttt
387841 agtaaaaaag tatcttagtt ggttgaaaga aacaccagaa gtgattttta ttactccatt
387901 ttggtggaat ggaattcctg gaatgttaaa aggatttatt gataaagtta tgaaagaagg
387961 accaggactg tcacatacag ttacgaaaac tggcatccat ggtgaactaa ctaatgttaa
388021 acatacctac atttttacta cttcaacttc tccaacattt tatttgaaac tgtttttagg
388081 aaatggtatt aaaagaattt ttgtcaataa gactttaaaa caattaggaa tgcaagatcg
388141 ccattggtat aacttaggtg gcattagcaa ttcctcaatt gaaaaacgta agaagtattt
388201 gaagaagata gctaaaatta acttataaaa agattcaaca tagtaaatca actatcaata
388261 aaatatttct tgtaaaaata tgctataatc aaaataaaga aaaaggagat ccgaagatcc

388321 tcttgcagcc cgctttaaga gcggtggttt taaacgttaa tttttacgat
gaacgctact
388381 caataactga ccaaagttat acattgagcg gcgtttttat tttttgtggt
ggtcgatata
388441 agttagcaag gctaacacaa acgtaccaaa caaaagcatt aaagaaatgc
tttcgtaaac
388501 cgacatgggt gaaccctttc tgcagtgtat tgaccacctc cgggaggaaa
aacaaccgcc
388561 gctcataaaa ctttctgcgg aaataattat acagaaactg tgtttgtaaa
agaagacgaa
388621 caagataaaa agctcgcaat aaaaatttaa ataaaatatt aaaactccca
acaaaaattg
388681 ggagtttttt gcgtaataag gattagaaat aaatttttta gtagaagagg
aaaaatcatg
388741 gaatatatta aaaaattatt tgctgatcca aaaatgtaca atgaagttga
gtttggtaag
388801 aagattcatg aagagaatgt attactaaaa ttgatgcaag aaattcttcc
tgaagaacat
388861 gttgttcagc cggattttgt tttcccgaag ataaggataa tcgaaatatt
tttgctaaaa
388921 ctgctgatgg agaaactata atgattggac tgcttgtagc tgataaagaa
acttggccga
388981 gtggtttaaa ctatcttcaa aatatgttga aagatgaagt ctatcgtttt
atgagaaatg
389041 aattattgct ttgcagaacc tattttattt tgttaactcc agtagatcca
tggaaaaatg
389101 gaagagaaaa atacactatt tcatttcgaa atgagcaatc agaagaattg
actgagatgc
389161 ttaaatctaa gttagttatt gtttgtccag agaattaata ggtaatagct
ttgtgtaatt
389221 tagatataga taggtaaaat agaaaggaga agataatgat tttactgatg
agaaaaggtg
389281 agagaatgtt atatccatac attgagttgc cagatgaaac gattattaca
cattcggaaa
389341 ttttagaaaa taactccgtt ttgattaatt ttgaacagcc ttatgatgac
ggatttaggg
389401 aagcaagaat tgagttgcca agttataagt ggttatataa tgacggcttt
agtaatgaac
389461 aaataaagtt ctttgttcaa tttgcacata aaaatgctgc tgtaatttat
aaatatgcta
389521 gattaggtgg aatagataat gcctaatatt tttagtactt taggatatca
tatatatttt
389581 tagtcaaatg agaatgatga acctattcat gttcacgtat ctaaaggaag
accaaacgct
389641 catgcaacaa agtttggctt actaaatctg gaggatgtgt gctggctagt
aacggaagta
389701 atcttagcag tcatgatatt aataaattga ttgatgttat tacggcgcaa
tacgatttga
389761 tttgtgaaag ctggttgaaa tactttaatg caaaacaaat tcagttttat
atataaccaa
389821 atggttctaa atcaaaacta tattttataa ttaatgccaa aaagctcgca
attaattgcg
389881 agctttttac tatgcttaga ttaattatta ccatactaat cctataagcg
agaagatgat

389941 ttcactgact agtactaaca aaacattcgt taaaattcct ttaaacgtag tttctttatg
390001 aggatcctta acaaaggttc tagttgaacg atagactagt ggaatggtga gtaaaccgcc
390061 taaagcaagg gtaggtagat agtgcaagac aacgcttaga attaaaaaaa tatatgctaa
390121 aacgtaatat atgcatagaa caatcaaagc attcttacga ccaatataaa atgggagagt
390181 tttacgacca attttaacgt cttcttctac gtcacaaata ttgttggcta gcattacgtt
390241 ggcaataaca aagatcgcag ggcaggcaac aatgattgct ttagcccaga aaaaactatc
390301 aaagggatgg atgttgtaaa tattgacgta gatacctaaa agagtgatgt tatagcccat
390361 tgtaattcca gaggcgggtt cgccaaaagg agttttcaga attgggtaag gaccgcctga
390421 gtagcaataa ccaattaagt agctaaagaa accgattaag agaattatcc aaccgacttt
390481 aaaactaagt atgatgccaa tagtcgcgga aacaagggtt aatagaataa ttacatttct
390541 agcccatttt cgggagatgt tgaatttttc taaaatatta ttgtaactag taacatgatt
390601 tttcttatag cgaagataat cagtatattg attatgacca ttaacggcta aatgaaaaga
390661 aattacggct agaaaaagcc aaagacaata agataaattg aaatggtgat aatagtaact
390721 agccattaat gagccaataa tatatggcaa ggctgaaatg ataattgtct gtatttgaac
390781 aagtgcgtta aatttttgaa ttatagagta ttttttcata agtaattacc ttaaatagtg
390841 attacttaaa attatagtct aaactttgct aagttgaaaa gaatgataaa aatggaaact
390901 cagtaaaatt atatacaagt ttatttttat taaggctttt taaactggct tatgtctact
390961 ccttcaagtt gaagtagttt aatcttttta tcaataccac caccataacc ggttaaattg
391021 tggttaacgc cgacaacacg gtggcaagga acaataatgc agatatgatt tcgaccaaca
391081 gccccaccag ttgctctaat aggtaaatgt tttttatctt gtgcagcttt catagcggta
391141 tctttataag tagtagttgt cccataaggg atttttaaca atgcttgcca aactcttttc
391201 tggaaatcag taccagtaag gtgcaattga ggaataaaat taggctcttt tccttgaaaa
391261 aaatatttaa ccatttttta gcagaagata gatatttgtt ttctccttcg gaatagtttt
391321 ttgcaagaat atctccatag taacgatcag tatctagcca tagaccaact agaccttgct
391381 cgtcactagc gagcaatatt tttccaatag gtgaattgta gtaagaaata gtttgcatga
391441 aatacatcct cttttgttta atatatatca attctaagcc tagcaaaaag accagtgact
391501 tgaagtgttc actggtcttt tcattattta gcgatcttat gtaccttatg tgcaatgacc

391561 atttctttat tacggtcagt ataaatcgca tctttgagat aatttaaagg ttgtcttaac
391621 aaacctttat tagctaagaa taaaaaatct gcatgatgat gattcaaatg acgcaaatat
391681 cttttagcgt tcttaatctt agcatgatgc tctaaagcaa gtttgggctc aaagtcagta
391741 atcttgtgtt cagcattata tactggcgta actttgtgat gccacttgtc gatatggtgg
391801 gtattatgtg caccaatttc tttacttgcc ttaacaatgt ctttttagt ggctaaatta
391861 tcttgatgct ttaactgtga gtttttgtca tagtagtaat ttccaaggcc atcttgacga
391921 taaagctgca aattctttaa tgacttatct ttattatcaa cagttacctt cagaacatcg
391981 ccatttttat gattcataag atacatatca tggtcaattg tgtaattatc cttcatgact
392041 aaaatatcca cctcttttat ttatatctga atgatattat agccacattt taagagaaag
392101 tatagtgatt aagttatagt tttaatcaat tattaaatta gttaaacttt attaataatt
392161 aaaaaaatta ttacgctaaa aggcagttag aaattaatcc tagccgcctt ttagtgtata
392221 tacttaaatt tattaatctt aattagtgtt cttttccatc aatgataacg tgaacacgag
392281 gattctttgg acctttggct ttagaaataa cgcccttgtt aactaaatct tcgatttgat
392341 ctgcatcata accaagttca gttaaaattt ccttgttgtt ttcaccaagg tcaggtgctc
392401 tgtcataagc tggtttgtag tttgaaagac tgaatggtaa accaatagtc ttgaacttac
392461 cacgattacc accttggtcg atagtaacga ttgtaccatc gctatttaga tctggatcgt
392521 tttcaagttc gtgccagtct agaactggtc caactggaac gccagcagca ccgagctctt
392581 tagttagagt atatttatcg tattgcttag tgtaggcttc aattaatggg tataattctt
392641 ctttgtgtaa ttgacggtgt tgccagtcga caaatcttgg atctttttcc cattctgggt
392701 gacccatggc ttcacacatc ttatcccatt tcttattttc gttttgaata acgatgtata
392761 catatgcatt aggatcagtt tcccaaccct tagccttgta agtccaaccg ataacttgtc
392821 caccttcagt gttctcagca cgcggaattg ccttgcccca cttccagtca gggtaaactg
392881 catagtgagg taaagcaccc aagttatcaa gcataagttg gtcacgtaac ttaatacggc
392941 aaaggttaag aacagcatct tgcattgatt gataaacaaa agtaccttca cctgtgtgct
393001 cacgttgtaa taaagcagct aaaataccga tagctaagtg cataccagta tttgaatcac
393061 ctagagcagc tgcagattga gtaggaacat tgtttgcacc tttgttccaa cctgttgcag
393121 atgcagaacc accagcagct tgagcaactg gttcaaaggc tttaacattt tcaaaacgtg

393181 atccttcgtt aaatcccttt aatgaagcat aaattaatct aggattgatg gagtgaagct
393241 tttcccaacc aaagccgtta cgatcagctg aacctggtgc aatattttca acaaagacat
393301 cagctttttt ggctaaatcg tacataatct ttttaccttc aggagtctta atatccaaag
393361 ttaaagatct cttgttggca tttaattgta agtagtaaag actccatgaa tctttaatat
393421 caagcaattc atttctagtt gggtcaccgg tattagtacg ttcaatttta ataacatcag
393481 caccgagcca agctaaaagt tgagtacatg atggacctga ttggacttga gtccagtcaa
393541 caattttaat accttttaat ggagcatatt cattttgttc gttttttttcg ttttcactca
393601 tcttaattac tcctcatcaa ttttcttttg ttcagcttct tcaagaggct ttaagtttaa
393661 ttctgggttt aaattaccaa tgtggcctga ttctttgccc attgaaggat cgatttgaac
393721 gttgataata cttggacggc ctgagtcaac agctgtagca aaagtatctt tcatttcttg
393781 atagttagtt acgtagtaat tatcaccgcc gaaggcttta gcaattaggt catagcgacc
393841 agttggatca agtgtagttg gacccaattg atctggaaca acattatcaa caccattata
393901 aataccacca ttgttaataa cgacaacagt aattggtaaa ttgtaacggc acattgtttc
393961 aacatccata ccgtcaaaac caaatgcact gtccccatct aaggcaacaa catgcttacc
394021 agtttcgaca gcagtagcga tggcataccc catgccaaca cccatgacgc cccaagtacc
394081 agtatcaagt ctgtggcgag gaagtttcat accaatcatg tcacgaccaa tatcaagggt
394141 attagcacct tcacttacaa ggtaggtgtc tggatgttct tggaagtaat cattaatagg
394201 ttcgatagcg ccataatatc caaattctgg gttagtttcc ccagctttaa ttcttttagc
394261 gaacttagcg tcattcttag cggtatcttg agcaattgca tcaagccagt cagaaggtac
394321 cttgtagcca gttgcagtaa gagctggaac taatttattt aaaatagagg ttaagtcacc
394381 ttgaagtggt gcagaaatct tttgtgcaga atcaaattgg gttgcatcaa tatctaattg
394441 gataaactta gcatctgggt taaattgtgg tgcatcacca tatgaaagca tccagtttaa
394501 tcttgcgcca attagaatta ctacgtctgc attctttaat gataatgaac gagcagcagc
394561 agcagagtgt ttgtcgtcat ctggaacaag acctttagcc attgacattg gtaagaatgg
394621 aatatcagtc ttgttaatca attcttgaac ttgcttttca gttctgtcat aagcagcacc
394681 tttaccaaga atgatcaaag gctttttagc ttgtttaatg atgttaactg cgcggttaat
394741 tgcatcgtca cttggttctt gaagtggagc tggatcaact aacttgtaaa cacccatgtt

394801 cttttcaccc aggtcatcat taagttgggc aatagtgtca gctggtaaat caaggtaaac
394861 accacctggt ctaccagata cagcagtacg aatagcacgt gcaactgcta gaccaacatc
394921 ttgtgctcga tcaacacggt aagctttctt acagaaaggc ttagcaacat tatattgatc
394981 taaaccttcg tagtcacctt gagctaagtc gataatatga cgttctgaag aaccagaaat
395041 cataattaat ggaaaacagt ttttagtagc ttgtgctaaa gcagttaatc cattcaaaaa
395101 acctggtgcg gaaacagtca ttgctacacc aggtttttta gttaaaaatc cggcagctgc
395161 ggcagcgtct acggctgagt cttcacgacg aaagccataa tacttcattc ctcttaattc
395221 agctaatcta gctaaatcag taatcggaat acctacaact ccgtataagt tgttaatacc
395281 atttttttgt aaagcgtcaa ttaataaact ggcaccagtt ctatttaaag agtcatctac
395341 cataacgtaa cctccatggt aatgataatt actattttat ataaaagcgc tattccaaaa
395401 tgggattgcg tacaactaat ttataacttt ttttgaattt gttagcaaga aaaaaactat
395461 aaaaaaactt ttatttcaat ataaaaagcg ctttctaagt atgaatatat aatagttatc
395521 ttctattaat tgtttcttat gtattgcttc tgatatacag tgaatttaaa ataacgattt
395581 tgtcagaatt atgaaatcgt tccacttgct tatatcttga taaagtctat atttagtttg
395641 tgatgaagga agttttcatc gggagtaatt aaactgatca gaatatgaac gaggtataaa
395701 gatgattagt atcttagtaa atgacataat ccctatttta gtgattatgt tactcggtta
395761 tctctgcgga aagttttact ttttcgataa tgatcagaga caaggactaa ataaattagt
395821 gcttgatatc gcattaccag cagctttatt tatttcaatt gttaaagcta ccagaaaaat
395881 gtttgcgcaa gatatcgtat taacgttgat ttctattgtt ggtgttgttg ggctatttat
395941 gctgagttat tatttggata aattgctctt ccatagaact acgcaacaag cagctgtttg
396001 tgctttgatt gcaggttccc caacaattgg tttccttggt tttgctgtat tagatcctat
396061 ttatggaaat aatgcaacaa ctaacttagt tattggaatt gtatcgattg tggttaatgc
396121 tattaccatt ccattgggat tagctttaat taacaaaggt caaaccttaa ctaagaagaa
396181 agctaaagca aatccaaata ataagggaac taaagttgaa gtagaagaca taaagggaaa
396241 gggcttaaat aagactaaga agaaggtcgt agtaactatt cccgatggtg ttccagttac
396301 tgatgcggaa gctaaggctt tgaaagaaaa ggggattgag cgtgaaattg atttagctag
396361 agcagaaatt gctgatcatg ctgatgaacc tcataagccg atgaacccaa ctttaaaatc

396421 agttattaat gcaattaaac aaccagttgc agcggctcca ttaattgcgg ttattttagt
396481 tttaattgga attagaattc ctacttcttg ggccccaact tttgatttaa ttgctaaggc
396541 taacgcgggt gttgctgttt tagcagctgg tcttgcctta tctacagtta aattttcaat
396601 tgacaaagaa gttatttgga atactttctt tagactattc ttaacaccgg cagtgattgt
396661 tgcagcagca tatctttgcg gtatgggatc agatcctacc aagatttcaa tgctttgttt
396721 ggcaaccgga ttgccaccag cattttctgg aattattatt tccagccgct acaatattta
396781 tgtcaaggaa ggtgcaagtt cagttgcagt ttcaactgtc ttctttgcag tcagctgtat
396841 tttctggatt tggctattac caattttggc gcatatgttt tagagagaat ttatatataa
396901 ttaagagaca ggaaggtaac ctgtctcttt ttgttaggaa taaaaaatga ttgataataa
396961 aaatttattg aaacttttac gaattgaatt gcgaaagatg agtaatggta acaaacttaa
397021 atttttaact tataaaaagg atcgcagcgt tcttgtggaa aagaatgggg ataattttag
397081 aattgttgaa gatggctatc gtaaaatcgt ttgggatgat ttgactgaag cagaagtttt
397141 gaaaagagta aaaagattgc agaaaataga gtttcccagg agcaataaat tatatttagc
397201 tagagaaaga caggctttat aaaacctgtc ttttttactc actatttaat tttaatatct
397261 ccgtcttggc tagaagctgt taatacatta gagctatttt gattttcaac aaagaagtca
397321 ttagaagagc ggcccttaac gttaatatca ccatcactaa catttagttt ataaccggta
397381 aaattagatt gagttatctt aatatctccg tcgtctgtat ttaccttaaa gttattttgg
397441 ttacttaatt tgttaatggt gatatcacca tcgtttgtat caatttttcc acttggagct
397501 tttaggttgt taatgttaat atcaccatca tttgtctcaa gtgaaattgg acttgttgta
397561 gcaatatttt caacttcgat gtctccgtcg ttagttgata aggtcagatt ctttaatagt
397621 tgtttgggaa ctgtgattgt aatagctggg tcgccgttaa catgatggat accagtagct
397681 gattgtttaa tagttaaaat accgtttttt ctgctaacag ttggcttcaa attcttaggc
397741 ccttcatatt caacctgata tttatcgcca tatttgacag aaatatcagc gagagcaaca
397801 gaaatttcta gtttattgaa tccttgatta ctgattgtag ttttgatttc tggcttagta
397861 tcatcatgat tcgaaatgat aatactacaa gcggaaagga tcattgctaa agaaaatagt
397921 agagaaatta agccaaactt tttactaata tttttcataa aaaactccta agataatagt
397981 taggttaatt atcttatatt cattaaatga agtaaattca aaccaaaagc aggaggataa

398041 gttaatgaaa aaattaccac caattcaaaa aattttagaa gcttatactg caattgtgga
398101 taatcatgtg aatttaacag agaatgaggc aaaagtaaca tcatctaatg gagctaaaac
398161 ttacacagta agttgggaga acgatgtata tcattcaaat gataacgcga cttactggca
398221 aggatatgct ggctatcccg tgattgcggt tttaatgctg cagggaaaat taccgtttaa
398281 tcaaaaacta gctgataatt ttaaagcagt taattggaat gaaataaatt taagttttaa
398341 gcgcaactac gccaaggctg ctactgaagt tattgaagaa aaggggctaa atgaaaatga
398401 agtaatggca gaacttgaag ttgtttatga aaacttaaaa aagttaccaa ttacaattaa
398461 gcgtgggtct cttagaccgc ctaaggctag aaaataatta agaagtacat tatttttcta
398521 tcctactcct tatttgctag ccagttctta tatatttgaa ttagatctgt attaatagga
398581 tctttttcag cttcagggtt accttgattt ttaaccatga tcattcctgt caaaacgtta
398641 ggatcatcgg gaaaatctgg tcgatcatta gcaatgacaa tattagtagg ataaccatag
398701 ccagcattac gttcttcttt taaataacga cgacagttta ttttcagttt tattccagga
398761 tttaatattc ctaggctttt aaatccttta gttaaattga ataataaata gtctggtatt
398821 tttaaatcta tattattcag atctttacaa tctttcatgt atactacagg acgataatag
398881 aatgttccgt tgctatcaac actttgttga acaccgtagc gatgaaatgt tccgataact
398941 ttaacacatt cgtaagtacc ttgcatatta tgtttcctct ttatcttttt agatttggtt
399001 ttcacattat tatatacgca aagaatacta ataatttttt attaattttt taatttttaa
399061 tttctataaa aatatttaat ttatattttc tgataataaa atctgggata agataatatt
399121 ctagaccgta aaaagaacgt tgagttttat gctcaacgtt ctttttacat caatgaattt
399181 ttacaaatta atcaagtaaa tcatacttca aagtcattaa accatgacct tcgttaacca
399241 tgtttcctaa taattcaaca gtattattgt agaccgtatc agccatcttt tggtttgctt
399301 cattaagctt gtcttgaagt tcgtcgccag aatatttttc ggcttctttg tcagtttcat
399361 gttgaatttt atggcattga gcaagactct tttgttcaaa gtcagcttct aattcgccat
399421 aaacgcggaa gttagtatca cctagctgag cagttaactt gtttaaccag aagatcttat
399481 ttagatcaaa cttagggcca gtttgaaagc ttgctggagt agtagtgata ttagtgtaga
399541 aaggaaccat gctgttaaaa gtatttggac caaatgctaa ccattgaaca ccagcaattt
399601 tttctggaac atcatttcta atttgtaaga tatgagtttc aaagtttctg ttaataccaa

399661 ttggacggaa tgtcttcttt tgttcagcac ttccttgatc accatatgcg tcaaaaggtg
399721 tgtcttggta gtgagaactt tctgcccatt taatatcttc aatgctaatc ttacggttag
399781 cgtgacagat gaagggttga tcttgatcac ttggctcgcc gccaaaatca gggtcaaagt
399841 aattgtggat ataccaagca cgaggattat tgtagtgagc atctttgatt gttgatgaac
399901 caaaaatatg acgtaaattg tagccttcga gatctggatt taagtgatat tcttcaatta
399961 aatctttaag atcttttgat gaagcaaaat catcgtcatc aaaataaaat tcatcaatgt
400021 ttaaacggtt aggtgcaata acataagcgt tatctggaat acgtctagca gcccagtggt
400081 gtccgccaat agtttcaaga taccaaattt cattcttatc agaaaaggcc ataccgttca
400141 tttcgtaagt accatacttt tcaagtagat aacctacacg ttcaacacct tcatatgcag
400201 aatgaatgta aggaagggtc aaggtaacaa aatcttcttc acctaagccg ccttcttcaa
400261 gtaatggatc aataccttga atacgtgagt tagtagtaat tgtttcagtt gcagtcatag
400321 caatattatc tgagttgata ccagcagctg cccagattcc attttcacca gatgcatcag
400381 gtgcactagt gtagcggagc ggattaggaa gaagatcttt gtcatcaatc ttttgatggc
400441 taattacgct ttcgtagtgc ttgggttgat cttttggcat tactacgtcg aatctctcag
400501 gaataatggt acgaccacca tcttcactac gagcgatcat tgttgaacca tcaatagttg
400561 catcttttcc gactaagata gtagtacatt cggtttgttt cattaaaaaa agctccttct
400621 ctattaacgt atttatttat attttaaaag atctttctaa tataaagcaa accttgaagc
400681 taaatttaca acaaatataa ggtataattt ggtaaagcat aaagtttctg aaagcatggg
400741 aggaattaat cgatgaatta tggagagaaa gcaccagcac tttgggatgc aattaagagt
400801 gaagaaaaaa ggcaagaaga tacaatagag ttaattgcat cagaaaatat tgtttccgat
400861 gctgtcagag aagcacaagg atctgtgtta acgaataaat atgcagaagg atatccgggt
400921 aaaagatatt acggtggttg tcaatatatt gatcaagtag aacaattggc gattgactat
400981 gctaaaaaat tatttaatgc ggaatatgca aatgttcaac cacactctgg ttcacaagct
401041 aatatgacag tttataatgc tttattaaag ccaggagata caattttggg gatgggaatg
401101 gatgctggtg ggcaccttac tcatggctca aaggttaatt tctcaggaaa gatttttaat
401161 tctgttagtt atgacctaaa tccagaaact gaagaattag attttgaaaa aattagacaa
401221 attgccttag aaaataaacc caaattaatt attgctgggg catcagcata tagtagaatt

401281 attgactggc aaaaattccg tgaaattgcg gatgaagttg gtgcttattt aatggttgat
401341 atggctcata ttgctgggtt agttgctaca ggggctcacc caagtccaat tccggttgca
401401 gatgtagtta ctactactac tcataaaact ttacgtggac cacggggcgg gatgattttg
401461 tctaataata agaaattagg taaaaagatt gattctgctc ttttcccagg tactcagggt
401521 ggaccgttag agcatgtaat tgctgcaaag gcacaagctt tttatgaaga tttgcagcca
401581 caattttcta catatattga gcaagtggtt aaaaatgccc aagcaatggc agacgaattt
401641 aaaaagagtg aaaatattcg cgtagtatca ggcggaactg ataatcactt gatgattatt
401701 gatattacta aaaccggtgt aacaggaaaa gatgcgcaga acttgcttga ctcagtaaat
401761 attacaacta acaaagaatc aattcccggt gatactagaa gtccgtttgt cactagtgga
401821 ttgagaattg gaacgccagc aataactagt agaggcttta aagaagatga tgcaagagaa
401881 gttgcaagaa tcattattaa agttttagat agccctgaag ataaagatgt attaacaaat
401941 gccaaaagta gcgttaaaag cttggtggag aagcatcaaa tcaaataaaa aagagagcta
402001 gtttactcag aaaagacgca gtatttgaat aagttagctc ttagtaaggt tgaaacaaat
402061 gattgatgta attgataata aatatttcta tctacataat aaagaagttg cttatttatt
402121 tgatttgtta cctaatcaac aattagagca cttatattat ggtgaagatt taggtaaatt
402181 atctaagaac gatttggaat atatgtcgct tcatccaaat aaagcttctg gaacagtaaa
402241 atttctaaa aaaataaaaa atttgacact cgctgatcga atgcaggaat ttccaacgta
402301 tggtacaagc gattttcgtg agggcgctgt aagtattgaa aatgaagacg cttatctata
402361 tcctgagttt aaatatgatc attttgagat tacaacaggt aagaggagga gcttagattt
402421 tcctacttca tatggaaatg atagtgaaaa tttaattgtc tacttaatcg atactgagca
402481 tcaattagaa ttgcaacttt catatagtat ttttgaaaac ttgtcagcaa tagtgcgtaa
402541 ggcagcaatt attaatcatg gtcagcaaga gattaaactt accaatttgc aaagtacagt
402601 tcttgatttg ccgaatgata gttatgattt tcttcaatta tctggagctt ggaccaagga
402661 gcggcatatt atcaggcgcc ctcttacaca aggaattacc aaaattgaat cattaagagg
402721 agcatcaagc catcaagaaa atccttttat tgcacttgtg gataaggatg tgacaaatga
402781 tggtggaaat atttatgcaa gcaatttgat ctattccggt aattttatca gtcaaataga
402841 agttgatgag tggggacaaa gtcggttgat gactggaatc aatccagcta cctttacttg

402901 gagattaagt cggaatcaag tattttctac accagaagct gttttgtttt atagcaagca
402961 tggatttaac ggtttaatga aacagacgca taattttgtt acaaagcaca tcattgatca
403021 aaaatggcaa cggcgagata gaccaattgt aataaataat tgggaagcta cttattttga
403081 atttaatgaa gctaaattgt taaagcttgc aaaagaagct aataagctag gaattgagtg
403141 ttttgttctt gatgatggat ggtttggtaa acgagatagt gataacagtt cattaggaaa
403201 ttggacagta gataaaagaa agtttccaca aggattggat cattttagtg cacaggtgca
403261 taaaatgggg atgaaatttg gattatggtt tgaaccagaa atggtttcac cagacaccga
403321 actctataat atgcatcccg actggatcgt tagacacccc tataatcgtt tttctgttgg
403381 acggagccaa tatgtcctag attttgctaa tccagaagta gtcgataata tttatagaca
403441 aatggaaaaa gtaattgaaa cagcccaggt tgactatatt aagtgggata tgaatcgaaa
403501 tattactgaa gcatattcac catatttgaa caagtcgggt tattcacagg gagagttttt
403561 ccacagatat attcaaggcg tttacaagtt atacgcaaaa ttattaacaa gttttcccga
403621 tctgttaata gaaggatgtg ctagtggcgg tggtcgcttt gacttaggta ttctatatta
403681 tagtccacaa atttggccaa gtgatgacag tgatgcggca gaaaggttag atattatgtc
403741 aggcacctta cttgcttacc ccttatctac gtttagtaat catgtttctg ctgttcctaa
403801 tgatcaagta aaaagaataa caagtttaaa atttagacaa aatgttgctt gctttggtcc
403861 tttaggatat gagctagacc taggtaagtt atcaacagaa gaaaagaacc aaattaaaaa
403921 ttggattaga tggtataaag aaaatcgtaa attgttagtc aatggaactt ttgatcaact
403981 actaaaattt gatccctcac aaaatactta tgcttggagt gtaagtaact ctaataaaca
404041 gattgtcgga ttttatcgca aactcgctcg acctaatgaa actttagatc aatatctccc
404101 tcttaaacat ttaaatgatc agcaaaaata tcagattaat tctgaccaaa tggtatcggg
404161 tagggtttta caaaaatttg gattacgtga accatatcaa tttaatgctt caaatcctga
404221 cactgcgcaa attagcggtg actttcaatc gtatctatat caaataaaaa ctattgacag
404281 aaaaatctaa gccaattaca atatagataa attttttgag gagtacttaa ttatgaatac
404341 acaagctaag tttattaaga ataatcgttg gcaaggccag taaatcaggt tgttagcaag
404401 atacaacctg aaagattcag attgtatctg tgctggctgt gtatttattg tggtctgcat
404461 ggaagaaaca tgcagacctt ttcttttaca ataaaaaatg atagggatct gcatggagtc

404521 agattcctat tttttgtgga gaaaaattat gaaaagaatg tatggattaa
ttgcaatttt
404581 aatagctttt ttagcagtgg ctttttttcac cgaaaatcgt aaacaagaaa
cagagagaaa
404641 agtaccgcag gtcggtgttt taactttaat gcatcaccca gctcttgatg
aaatatatcg
404701 tggttttaaa gctgaattaa aagcagcagg atatgtagat gggaaaaata
tcaagattga
404761 ttatcaaaac gcaaataatg atcaaagcaa tttacgtact atggctacta
agttaactga
404821 tgagcagtca aaagtccttg ttgggatcac tacgccggct gcgcaagcat
tagcaaattc
404881 aactagtaaa acaccaattg ttttaggagc agtaacaaat ccgaaagcaa
gtggtttggt
404941 aaaaaatgaa cgtcatccag aatcaaatgt aactggtgta tccgatgccg
ctccgattaa
405001 gcaacaatta gatttaatta aaaaatttat gcctaatctg aaaacattag
gggtaattta
405061 tacttcaagt gattcttccg cagttagtgg ctttcgtgaa atcgagcagg
aatgtaaaag
405121 aagaaaaata aatttaagag tattttcaat agcaaatagc aatgatctta
atcaagtatc
405181 agagcaaatg tttaggcagg tggatgcggc tatagtacct acggataaca
caattgctgg
405241 agcaatggaa accttggcta aaaatggcaa tgcagctaag aaacctattt
tcccagcggc
405301 cgcaacgatg gtaaagcaag gggcttagc aacttacagt gttaatcagt
acaagctagg
405361 acagatgact ggaaaaatga cgattgctat tttaaaaggt aaaagggtca
gctcattacc
405421 agtagaaaga gttcaaaaag gagaacctgt ggttaactta aaagaagcaa
aagaattgaa
405481 tctccaagtg ccacaaagat ttctaaaaga gtgtcaaaga aatggggttg
tttacagatg
405541 aatttgatcg tatctgcaat tggtcaagga ttattatggg cattattagg
cttagggtta
405601 tatttaactt ttagaatctt aaatttcgct gatatgacag tcgaaggaac
ttttccctta
405661 ggagcagctg tggctgttgc taatcttgct catgggatgt caccatattt
ggctactttt
405721 ttagctttgc ttgcgggaat gatcgcagga ttagtgaccg gtttattata
tacaaaggga
405781 aaaattccga ttttgcttgc tggtattttg acgatgactg caatttattc
gattaactta
405841 aggattatgg gcggttctaa tatttcatta attggtaaga aaaccctgct
taataatgag
405901 tttttgaaat ctctacctca gtattttaat agtgtggtct taggagtaat
agttgtagta
405961 attattacag gaattttggt tttcttttta aatacggatt ttggccaggc
ttttattgct
406021 acgggagata atcctaaaat ggctaagtct ttaggaatct atactgattc
aatgataatt
406081 attggtttaa tgctttcaaa tggaattgtt gctttatgtg gagcattaat
tgcacaaaat

406141 aatggctatg ctgatattaa tatggggatt ggaacgattg tcattgcctt agcttcaatt
406201 attattggtg aagtagcttt tggcgaatta accttaaatc aaagattagt agcagttact
406261 ctaggaagta ttatttatcg cttaatttta ttagcagttt tacaactagg cttttcagct
406321 aatgatttaa acttaatttc ttcaattgtt ttggcacttt gcatgatgct gccacaatta
406381 gaaaaaattg tacatttgaa aaaaccaata gagagaggag tgaagaagca tgactaagcc
406441 aattttagaa ctaaaagatg taaaaaccac tgtaaagaca gctgatggtg aaatagttcc
406501 tattttaaaa ggaattgatt taaaaattaa agctggggat tttattacta ttattggaac
406561 gaatggggca ggtaaatcta cactgttgaa cactattgcg ggtagtttga gaccggatag
406621 tggttttctt ctccataatg gacacgacat tactaaaatg agtgaggaaa aacggacgca
406681 atttattgga cgcgtttttc aagatcctaa gatggggact gctcctagaa tgacagtggc
406741 ggaaaatctt ttgttagcaa ctaagagagg taagagaaga tttttgaaaa taagaaagtt
406801 aaagcaaaac ttaccacgtt ttaaaaaatt ggctgcagtt atgaacaatg gactagaaaa
406861 tcgcttaaat actttcgtag aaggtttatc aggtggtcaa agacaggctc taagtttctt
406921 aatggctacg attgaaaggc cggatatttt gctgttagac gagcatacgg cagcactaga
406981 cccacataca agtgaaaatt tattggctgt aactgatcag cagattaagg aaaataaatt
407041 aactgcctta atgattacgc atcacatgga agatgcttta aagtatggga accgtctttt
407101 agttttaaaa gatgggaaag ttaaagccga tattaatgaa caagaaaagc aaaatttaaa
407161 agtaagtgat ctatataaat attttgaaga ttgaaatgaa agctagagcc agttgtcggc
407221 tctgcttttt ttagttaaaa taatttttaa ttgttttaag tgtgtattta ataataaaaa
407281 tgggggaagc tgtgaataat aaaggacata aaataagttt attttcagca ataatgctag
407341 cccttaattc attgattgga tcgggatggc tttttggatc gggatcagca gccaaaattg
407401 caggtccagc agctatcttg tcgtggataa ttggtgcagt tattattatt gcaattgctc
407461 taacttatgt tgaactcgga gcgatgtttc ctgagagtgg cggaatgagc aaatacgctc
407521 aatatagtca tggtcccatg cttggcttta ttgctgcttg ggcaaattgg atttctttag
407581 taactttagt accaatggaa gcagttgctg cagttcaata tatgagttca tggccttgga
407641 aatgggcaaa ttggactcat cattttatgc aaggtggtaa tataacgaca cctggattat
407701 tagttgtgtt tgcgttcatg attatttta ctttaattaa cttttggtca attaagatta

407761 tgacgcactt tactaacttg atttctgttt tcaaggtttt actacctaca attacaatta
407821 tcatgttatt tgcatctagt tttcaccctg ctaattttgg tcatgatgtg aaaactttta
407881 tgccttatgg aagtcgagca atttttgaag cagcttccgg agctgggatt attatgtctt
407941 atgatgcttt tcagacgatt attaatattg gtggagaatt aaagaatcca cgtaaaaata
408001 ttattcgcgg agttttgata tcgatgctgg taactgcagc tatctacatc ttgcttcaaa
408061 tagcctttat tggcgcagta gatcctgcga taattgctaa aaactcatgg caaggaatta
408121 actttgcttc tccatttgcg gatattgcaa ttttgttagg aattaattgg ttggttatct
408181 tactgtatat ggatgccttt gtttcgccat tcggtactgg ggtagctttt gtagcaacgg
408241 cttctagagc attggctgca atgactcata ctaaacattt accagcttgg ctaggtagac
408301 tcaatcgacg ctacttagta cctcgctttg cgatggttgc tgatttaatc ttagcgatga
408361 ttttagttag tgtttttaga aattggaatt tattagcaac ggtaatcact tgtgcaactt
408421 taattgctta tttaactggt ccagtaacta cgattacttt aagaaaaata gcacctgact
408481 tagaccgccc gtataagcca gtatatatgc gatggtttgc tccattaaca tttgtactaa
408541 ctagtttggc tatttattgg acaatgtggc caacaacaat tcaagttatt ttggtaatcg
408601 tgttaggcat accgatttat ttctattatg aggttaaata tcaaaaaaga aattttaagt
408661 cacagtttaa gcatgctgct tggcttttag gttatttagt atttatctcg attatgtctt
408721 attgcggtag tgatggtttt ggcggtcaaa attggatttc atatccttgg gatttcttgg
408781 ttattagtat tgcatcatta ttgttctata aatttgcaat caacagtggt ctagaaaaaa
408841 ttgatgctac tgcgataaaa gtaaacgaaa aaattaagct tgaagaagac taggatttga
408901 agtcgtaact ataaaatagc ttgaattatg gttagaaagg tgctattctt aactagtcgt
408961 gaaataatct atgatctgaa atattggtca tggcaaagga gtattaaaca atgagaaaag
409021 ggattcatcc agattaccaa gaagttgttt tcatggactc agcaactggt gctaagttcg
409081 tagctggttc tactttaaag ccagaagaaa caattgaatt tgaaggtaag acttacccat
409141 tagttcgtgt tgaaatttct tcagattctc acccattcta cactggcaag caaaagtttg
409201 cacaagcaga tggtcgtatc gaaaaattca acaagaagta cggtatttct tcaaagaact
409261 aataattctt ggtatttttt accaaaagag cagcccactt tggactgctc ttttttcatg
409321 attaacgtgg tataattctt attgatatgc aggtgacctt tttctgataa gtgtaaatca

409381 gtataatatg gttacatgca attttaggta ggagtaattt atttattatg aaaatgcaac
409441 ttgctgagat cgccaaagca ttaaatagtt cagttaatat gggagaagat aaggtcatca
409501 cttctgttgt tttgactcaa agaaaagcaa cacccaattc tttatttatc ccattagccg
409561 gtgtaaggga tggacatgat tttgtatcga atgcaatggc aaatggagca gaagcaactt
409621 tgtggaaaaa aggtcatgaa ggtaagccaa ctgatgtgcc agttattgaa gttgaggatc
409681 cattaacagc acttcaatct ttagctaaat attacttagg taaagttaac ccaaccgttg
409741 ttggaattac tggttctaac ggtaagacta caaccaagga catggttgca gctgttttag
409801 ctaaaagatt taacgttcat aagacacaag ccaactttaa taatgaaatt ggtgttcctg
409861 taaccatttt agagatgaag ccatctactg agattctagt acttgagatg ggaatggatc
409921 gtccaggaca attacatcat ttaagtgaat tagtaagacc agatgtttgc gtgattacca
409981 tgattggcga agctcacatt gaattctttg gtactaggga taaaattgct gatgctaaga
410041 tggaaattac cgatttctta aaagaagacg gtaagtttat ttataatggc gatgaaccat
410101 tgttagaaga acgtgcagct aagattaagc aagacaagag tacttttgga tttagagaag
410161 aagatactgt ttttgcaact acttttaggt cttacatgca ccatgctact tttgaagtaa
410221 atgattcaga gcagcaattc aaaattccaa tgattggtaa gcataatgta tcaaatgctc
410281 ttgcggcaat tagtgtagga cgtcactttg gcgaaaatga tgaacaaatt gctcaagcct
410341 tagccaactt taccccaact gcaaatagaa tgcagtggaa gaagggagac gtaggcgaag
410401 caattatgga cgatgtttac aactcaaatc caactgcggt taaggctgtt actacttcat
410461 ttggtcaggt gatggttaaa gatggcggac gtagaattgc agtcttaggc gacatgttag
410521 agcttgggga tgaatctgct gagcttcatg cttcacttgc taatgcgtta gatccagcaa
410581 ttattaacga attatatttg tatggcccag aaatgaagaa tttatatgat gcacttgttg
410641 ataagtatcc agcagccaac ttgcattatt atccacaaga tgaaatgaat catatgattg
410701 atgacttaaa gaatgatatt aagccagatg atattgttat gctaaaagga tcacatggaa
410761 tgcatttaga aaaggtttta gaccgcctaa tgtaggaagg aattagatat ttgaaatttt
410821 ctgaaatgaa cttgaaacct gaaattttaa aagctattaa acgttcaggt tttgaagaag
410881 ctactccaat tcaagaaaag actattccct tagttttaga gggaaaagat gtaattggtc
410941 aggcacaaac tggtactggt aaaactgctg ctttcggttt gccaatttta cagaatttag

411001 ataaacaaca cgactcgatt caagccattg ttattgagcc aacgcgtgag
ctcgctattc
411061 aaacccaaga agaattattc cgtttgggta gggatgaaaa ggctcgtgtc
caagttgttt
411121 atggcggggc agatattcgt cgtcaaattc gtgctttaaa gcaaacccca
gcaattttag
411181 taggaacacc aggacgatta ttagatcact tgaaacgtgg aacaatcgat
atttctaagg
411241 tgaaaacaat tgttttagat gaagcagatg aaatgcttga tatgggattt
attcaagata
411301 ttgaaagcat cttgaattat gcttcaagta aacatcaaac tttgcttttc
tcagcaacca
411361 tgcctaagcc aattctacga attggtgaga aatttatgca tgatcccgaa
attgtaaaaa
411421 ttaagggaaa agaattaact gccaatttaa ttgatcagta ttttgtaaga
gcaaaagaaa
411481 acgaaaaatt tgatatttta tgtcgtttga ttgatgtcca aaatcctgac
ttggctgtta
411541 ttttcggtag aaccaaaaga cgtgtcgatg aattaacacg tggtttacaa
gctcgcggct
411601 ataacgctgc tggaattcat ggtgatcttt ctcaagctaa gcgcatgagc
gtgttaaaga
411661 gattccgtaa aggtaagctt gatattttag tagcaactga cgtggctgcc
cgaggattag
411721 atatttctgg tgtaagtcac gtttataact atgatattcc acaagatcca
gattcttacg
411781 ttcaccgtat tggtagaact ggacgtgctg gtcaaaacgg aatgtcagta
acttttgtta
411841 caccaaatga aattggctac atgcgtacta ttgaacaatt aacgcataaa
aagatgatgc
411901 cgctaaaacc gccaacagat gaagaagcat ttaagggaca attgagtgca
gcaaataaga
411961 aggtaacaga attattagat ggtgatcttt ctaaatatac tgcagaagca
tcacaactac
412021 ttgatgacta ttctgctgta gatttagtag ctgccttgtt aaagaattta
tctaaagatg
412081 ctgaaagcgt aaaggtaaaa attacacctg aaaagccatt gccatttaag
tcaaagcatg
412141 gtaacaataa tcgccatttt aaacgtaact tcaagcgtgg tggcgatcgt
aatgaaagat
412201 atcatcgtaa gccaaatgcc cgtcgtggtg gtagaaaaca tgactttgta
attaagaaaa
412261 aagactagtt tttaattggg agctgtagtt gctacagctc ttttttgtaa
tataatgtat
412321 caatgtgtga ggtaaaaata atgattagag gctttggaat tgactctgtt
gaagttgaac
412381 gcatgaaaaa aattgttgaa aaaggcgata aattcgctaa aagagtgtta
actccaaagg
412441 aatttacaca atatcaacaa ttaaaaggaa agcgaaaggt tgaatatctt
ggtggacgtt
412501 tttcattaaa ggagtctttt tctaaggcat tagggacagg tctaggtaag
tatgttggct
412561 ttcaagatat tgaaacctta tgggatgatt taggacatcc agtaatgacg
tcgactaagt

412621 tttctggaaa tatttttgcc agtatcacgc atgatgatca tgagattata acaggtgtag
412681 ttttggagga attaaattaa tggttccagg tattcatcgc ccagctgtag taaaggttaa
412741 tttgggcgca attaagagaa atattgaaaa tgaaatgcag catcttgagc caggccaaaa
412801 gatgcttgct gttgtaaaag ctaacggtta tggccatggc gcagtagaag tagctaaatt
412861 agcagatcag gttggagctg ctggattctg cgtggcaatt ttagatgagg cgttagaatt
412921 aaggcgtgca gatattacta aaacgatctt agtcttaggt gttgtttctc ctgaatatgc
412981 tcctattgct gcagccaatg atatttcact gactgttcca aacctagaat ggcttaaaga
413041 agcagaaaaa tatttagaaa attcagactt acaattgaaa attcatttag gtattgattc
413101 aggaatgggt cgaatcggat ttaatgaaga taaggacttt attgctgcta acaaattttt
413161 gaaaaataat gacaattttt acattgaagg aatgtttgct cattttgctt ctgctgatag
413221 cagtgatgaa acttacttta aacatcaatg tgaaaaattt aaccatatga agagtttgct
413281 tacagtaaag ccaaagtgga ttcacgttga taatactgca gcaagtattt ttcatgatgg
413341 aattaagagt gatttagtta gatttggaat tggtatttat ggcttaaatc cttcttctaa
413401 tcctaattca actgatttga agtcaaaaat tgccttagat ccagctttat catttgagag
413461 tgaattaaca catgtgaaga caattcatca aggcgatggg gttggatatg gttcgacatt
413521 tgttgccaat aaggatacga ttattggaac agtgccagtt ggttacgcag atggttttat
413581 cagaaaattc caaggtttta aagttaaggt aggtaatgca tattgcccaa ttgttggccg
413641 tatttgtatg gaccagttca tggttgaaat gccaaaagaa atgccgattg gaactaaagt
413701 agtaattatt tctaataatc ctgatgatcc aaataatatt aaagcagcag ctgattatgt
413761 tgatacaatc cattatgagg ttgcttgctt attgaacgat cgtttaccgc gtgtttatta
413821 cgaagattaa attaaaaaga gctcgtttag ccaaattaaa atggctaagc gagctttttt
413881 attgattaaa atttaatctt ttaattcccc aggttgttct gcgctacgaa ttgttagtcc
413941 agattgaaga tcttcaattt cgggaactac aaagcctttc ttttctaggc gatttacaat
414001 tgcagtcata gtttcgcgag agacaccgat tggcaaagta aataaattac gacgaactag
414061 ttcgccagtt ttaacatcta aagttaggca gcctgcgata ttactgtact tagaaatgat
414121 ttttgccatt ttaactaagt taccacgatc attgtcagaa agaatagtta aaacataaga
414181 accattctta atgttccaag catcggaaag catgtctagt aaacgagcgt gagttaaaat

414241 tccatagaag tggttttctt catccaatac agcgatgtat gggaggtctt taatattaaa
414301 gaatacacgg aagaaaggtg aatttacctt aatagtttta gtagcattct taagcaagta
414361 agttacaggt agattcatgt cgccaccttg ggacttatga cggtagatat gcatcttata
414421 aatatttccg cggaaaatag ttcctgtatc atctaaaata ggaacacaac ggaaaccgga
414481 gtcttctaag atttttaatg cttcggcaag agtagctttt tcgttgactg tagttaagta
414541 atcttttttta taaacaagag atttaattag catgattagc ctccatacat ctgaaattta
414601 caagccatta taccatagtt ttatataaaa attaaagtct gattaaacaa ttttaatttg
414661 aagaagaaaa aagcattcac gataagtgaa tgctcttccg tagaaaaagt cttaattatt
414721 gacgaacttt tacgccagtt tctttgaagg ctttgtccat aacttctttc aattgttttg
414781 cagaagcttc catcttagct tgttcgtcag cgtttaatgg catttcaata acttgctcta
414841 aaccgtgacg accaacaact gctggagtac caatgtgaag atcgtgtaaa ccgtattcac
414901 cgtccatagg tactgaaagt ggaagaactc tgtgttcgtt gttcaagata gctttagtaa
414961 taaatgctaa tgcagtaccg ataccgtaga aagtagcacc ctttttgtta atgatatcgt
415021 aagccatgtc agcaacttct ttgtggattg cttcaagctt gttttcacca acttctggat
415081 gagcctttac ccagtcgcta acttttacgc caccaacatt gttgtagctc catactggga
415141 attcggtatc accgtgttca ccaagcatgt atgcattaac tgaacgagga tcaacgtgtt
415201 ccatttcacc gataactttt tgaagacgac cagtatcaag tgaagtacct gaaccgataa
415261 cacgatcctt agggaaacct gacattctcc aagttgcgtg agttaaaata tcaactgggt
415321 tagcagcaac taagaaaata ccattgaatc cagattcaac gattggttca acaattgaag
415381 ataaaatctt taagttcttg ttaacaaggt caagacgagt ttcacctggc ttttgtggag
415441 caccagcaga aataactact aagtcagcgt cttttgcgtc tgaatattct gcagagtaaa
415501 tagtctttgg tgcaatccaa ggagttgcgt ctgctaagtc aatagcatca ccttgagttc
415561 tttctttaac gatatcaacg atacctaatt cttgggcgat accttgttga actaatgaaa
415621 atgcgtaagt agaacctaca gcaccgtcac caacaagaat aatcttatga atttttttctt
415681 ctgacatttg ctaatacttc cttccatatt agtgatttat ttaacaataa gattatatca
415741 tattgaaatt taaaaattaa aaatataact ttaaagatgt agtttattat ttgtaagtaa
415801 gcctgtgcta taatctcaag agcgaaagaa tatagaatta gatatttgtc aaagaggatg

415861 aaaaataatg aaattaattg caggtttagg aaatccgggt aaaaaatatg atcagaccaa
415921 gcataatact ggctttatgg cattagacca ttatttaaac aaaaacaact tagatttaga
415981 taaggataaa tttgaaggat tatggactaa gcagaagatt aacggcgaag atgtgatttt
416041 tcttgaaccg caaactttta tgaatgattc cggaaagtca attgcgcaag tggccaattt
416101 ctttaagatt gctccagaag atatcttagt tattcacgat gacatggata tgccaatcgg
416161 gaagatcaga attcgtgcaa atggaaaatc aggaggtcat aatggaatta agagtattat
416221 ggcctgttta ggaacaaata atttcaaccg attaaagatt ggaattcgtc atccgcaaaa
416281 agaaagcgtt gtttcttggg tattaagtcc ttttaatgat gaacagcaaa agttaatgga
416341 tgcagctttt gatgttagcg aaaatatcat taacgatttc attaagggga agaatgccca
416401 gtatttaatg aatcagtaca attagaatga acattacaga attacttaat aaagacaaag
416461 atttaaataa ttttattaat aaagttccaa aagaaaaacg ttcactttta actggagtaa
416521 attctgtagc ttttagtgct gtcttgatgc aaatgctaag cacttggcag cgcccattaa
416581 ttttggttga agatagtgaa gacaaggcac agatgctttt agatgaacta ggaaacttat
416641 tacctgataa tatggtattt agttttccag ttgatgccac aattgcaaca caaactgcta
416701 tcgcttctcc agatgagcta agtcaaagac tgcaaacttt gaagtttttg actgaaaaaa
416761 gagctggaat tgtagtagtt actccgcaag cattgcaata taagttaagt gatccgcgag
416821 attttactaa agcaaagcaa gtatttaaac ctgaggcaga atttgactta gatgaattaa
416881 ctgcatggtt cacacaagct ggatatcgta gagaaaatat tgtcgcacgt ccaggagaat
416941 ttgctcgtcg tggcgatatt cttgatgttt atcctttaga ccaagaaaat ccttttagaa
417001 ttgaattttt tggtgatgag gttgataccg taaaagaatt tgatgcagca acacaacgta
417061 gcttagaaga aaaagatatt gtttcaatcg gcccggcttt agatagagtg ttttctagtc
417121 gtaatcttca tgaggcagtt aagaaaataa aacaagacat gaaggaatca attgccaaag
417181 aagaaaatgt taaaaatcat tttgttaaag caattgattt gcttgaagcg gatggacttc
417241 ctgaaaatta tgcttttttta gttgattact tgcttccaaa gtcattcaat ttgattgatt
417301 atcttgataa aaatggacta cttttatttg atgattggca agcaatcaaa aagaatgttg
417361 ctgatgtaga tgctcaaaat gaagctttta tttccgaaga aataaaagca ggtgcaatgt
417421 taaatagcca aaaattacga cataattttg agcaagtagt tcaaaaggtg aaacaagcac

417481 aggtaatatt ttcactgttt caaaaaggaa tgggtcgctt aaaatttgac caaatactta
417541 attttactac gcgagaagtc cagcaattct ttagtcaaat gccgttaatt aagagtgaat
417601 tgaagcgctt tgcgaatgaa gaaaatacgg ttattttaca ggctgattct gcaactcgtg
417661 ctaagcaaat cactcaaaat ttggtagatt atggtgttga tcttccgatt gttaaatctg
417721 atcaaatttt gataaaaaaa gctcaaataa ctgttggtga ttttgcacac ggttttgcac
417781 ttcctagttt gaatttagtg tatttaactg aacgtgaact atttaatcaa agaccacacc
417841 atcgtaaacg tattaaaaca ctcgaaaatg ctcagcgttt gcgaaactat aacgaattaa
417901 agccaggtga ttatgtcgtt catgttaatc atggtattgg tcgttttgag ggaattaaga
417961 cgttagatgt agatggtaaa aaacgcgatt acataacaat tacctatcag catcatgatc
418021 aattatttgt tccagctgat caattaggat tagttcaaaa atacgtggca tcagagggta
418081 aagttcctca tattaataaa ttaggaagtg cagagtgggc caagaccaaa aaacgagttc
418141 aatcaaaggt tgaagatatt gcagatgatt taatcgaact atacgccaaa cgtgaatctg
418201 aaaaaggttt tgcctttagt ccagatgatg atttacaaaa agaattcgaa gatgcttttc
418261 cttatccaga aacgcctgat caattgcgtt caattagaga aatcaaggcg gatatggaaa
418321 gccctaagcc aatggatcgc ctcttagttg gagatgtagg ttttggtaag actgaagttg
418381 ctttaagagc tgcttttaag gctattcgtg atggaaagca agttgcattt ttagcaccga
418441 ctactatttt ggcacagcaa cattttgaaa caatgcaaga tcgctttaaa aatttcccag
418501 tcaattgtgc gcttttatcg cgctttcaaa ctcctgctga agtaaaggag ataattgaag
418561 gtgtaaaaaa tggtcaaatt gatatggttg ttggtacgca tcgcttatta tcaaaagatg
418621 taaagtttaa agatcttggt ttattaatag ttgatgaaga gcagcgattc ggcgttaagc
418681 acaaagaaag attaaaggaa ttaaaagcta atattgatgt tttgactttg acagctacgc
418741 caattccgcg tactttacac atgtcaatgg ttggcgtgag agacctttct gtaatggaaa
418801 cgccgccaac taatcgatat ccaattcaaa cctatgtcat ggaagagatg cctagtgttg
418861 tacgcgaagc agttcttaga gaaatgaagc gaaatgggca ggtatttttc ttgcataatc
418921 ggattgatga cattgataaa gtagttagcc gattagaaga actgatacca gaagctaaaa
418981 ttgaatatat tcatggtcgc atgagcgaaa accaaatgga agatattatg tatcgttttt
419041 caagaaatga gtttgatatc ttagtaacaa caacaattat cgaaactgga gttgatatgc

419101 ctaatgtcaa cacaatgatt gttgaaaatg cagatcacta tggattaagt cagctttatc
419161 aattacgtgg tcgaattgga cgaagtgctc gtttagctta tgcctatttc ttgtatcagc
419221 ctaataaggt tttaacggaa attggtgaaa aaagactaag tgctattcgc gattttactg
419281 aattgggatc tggctttaag attgcaatgc gggacttgtc aattagagga gccggcaaca
419341 tgctaggaaa acagcaacac ggctttattg atagcgttgg ttatgatcta tatgctcaaa
419401 tgctagatga tgcaattaag aaacgtaaag gcaagaaagt tgctgttaaa tctaatgcag
419461 aagtgcaatt taatttggaa gcatatattc ccgatgatta tattgctgat caggaacaga
419521 agattgaatt ttataaaaaa attaaaggga ttaatgatca aaaagaagca gaaagtattg
419581 ctgatgaatt aattgatcgt tttggagatt atccattagc ggtggaaaat ctgttaaatg
419641 tgtcaaaagt taaagcagat gctgatttag ctcaagttgt agcaattaat cagaagtcag
419701 atgataaatt acaggttatt ttcaataatc gtgctagtga agaattagaa gggccaaata
419761 tttttaaggc attagaacat gtgtcgttaa gagctaggat taatatggat ccacaaaaga
419821 gaatgaatgt catgcttgaa cttaaaaaaa agatgagcac tagacaatta tttaatgaat
419881 tacatgtatt tttggcagcg gcgagcgata ttgttcaaag atagagagga aaataaaatg
419941 agaatcgata agtttttaaa agtatctaga ttagttaaga gacgtacagt tgctaaagaa
420001 atggcagatc aagggagagt aaaagttaat gatcgagtag ttaaatccag ttatgacgta
420061 aaagtcggtg atgttattga aatcggattc ggtacgaaac aattgaaagc tcgtgtactt
420121 gacttgagag aaacgactaa gaaggctgaa gcaagcgatt tgtatgagct aattgattaa
420181 tgacaaggga agagtaaggt gttatattaa taacaagtgg tgatataatt aagatttaga
420241 atgtaaacga aaaggcaggg acacgcgtat gcagagaggg ccacgacttt ataatgcctt
420301 aagcgatgaa gaacgcttgg cgcgcttacg gcgcaaagca gctaggaaag aaaaagaagt
420361 tcatcgcgtt cggcgaaacc gaattgttgc ttgttttgca attgcaattt taatttttgg
420421 actacagtta attatggtcc atgtacaaac gggtagaatt aatagtcaaa ttcaaacgaa
420481 taagacagag ttaaaccaag ttaataaaac taataaaaaa ttaaaggcgc aagtggcaga
420541 tctgaaagat cctgattatg tggctaaagt aatacgttat aagttctact attctaaatc
420601 tggcgaatca atatatactc ttcctgataa agaggaggat tattagtgaa atttccaatt
420661 ggattaagac ttaatgctaa agttaacaat attactaaac ttgggatttt tgtgacttta

420721 ccgcaccatc atcatggctt gatttttcat aaagattttg gtgataagtg ggaaagtgct
420781 aggaacaagt acagtattgg agatgagatc agggtagtta ttatcaataa tcgtgaggga
420841 aagctttcgc tctctctgag tcaagtaaat aatcctgatc tgattgatcc gactaatgaa
420901 tttaaagata gtaaagactt tactccttta gcaaagctag tagaaagttc tacaaagaaa
420961 atagcagatt taaagaattc aattgagtta agtgaccagt aaaggtcact tttttattta
421021 acaatgacag aatttacaaa ttttttact aaaaataata ttgaaattaa aaataaaaag
421081 ttcttgttag ctgcaagtgg tggacctgat tcagtggcat tattccatat gttagttaac
421141 ttcttacctg atccttcaga acagttaatt gtggcacatt tggatcattg tctaagaaaa
421201 gatagttatt tagaaagtga gttactacaa agattaacca ctgcttttaa agtaaaacta
421261 atagaaaaaa gctggcccct tgcgcttcat cctcaaactg gtattgaagc aagggcgcgt
421321 gagtttcgct atgcattttt agccaatacc gggaaaaaat atcaggttga ttacttgtta
421381 acagcacatc atggcgatga tttaattgaa aatattttac tcaaatttat tcgttcaggg
421441 gatgtagctg aaatgaatag tcttcaggtc gtaggaaatt ttaactctat gaagctatta
421501 cggccgttaa ttaagtattc aaaagatcag ttgttagaat atgataagca gaatgactta
421561 gactatattg aagacgagac taattttgaa gatgatactt taagaaatcg tttaaggcat
421621 catgttgttc ctttattgaa aaaagaaacg aatcatttag ttgaaaatgc ctatcgtttt
421681 agcgaaagtg cagcgctact ttcagactgt caaagtagct tttttgaaag tttgatttta
421741 cctgttagct ttggtaaagc tttacaaggt aggaaatcag atctaataga cttaaataaa
421801 aatcagttag cagctttttt tgattatcta atttataaaa agtggcatca acgagttcac
421861 tttgatgaaa ttaggtggag caaaaatgca acttttaaca aagaaaattt tcaattaatt
421921 ttttatcaag attattatta cttgataaat agaaaccagt taacgccaat tgacgttaat
421981 aaaaagaaaa ttaagctcga tgaaaagttt tctttaaacg gaaaagaata catgatttca
422041 aatgataaaa aactgggcaa cttagttgga tatttctatg gagcaaaaag tgatttctta
422101 gaggtgggaa gtttacctca gggaagcaaa ttaagactag ctgatggcag gcaaacaaaa
422161 gctaagaaaa aatttgcgga gaatggaatt cctttgatat taaggccgta ttgtctaact
422221 atttggcaag gaaaaaatcc agtttatgtt gaaaatgtct atcaaaatca agaatataat
422281 ccagatttta ttcgatataa tgtatatatt tatctttaag tttgtgcaaa gttctatggt

422341 aaaagcgtaa tttgtggtaa aatcgttgga gtaattccaa gaagtgtcgc ggaggtttat
422401 agatgaaaaa tagaagaaat agactgcttt ctaacggtct tttctatatc atagtattcg
422461 tgatcctctt ggctggaatc aattgggctg tgggtggctc aggtagtaat ggttccacag
422521 aaaatatccg ttattctcaa ttagtaaaag acttaaaagc gggtaaagtc aagagtatca
422581 atgtacaacc atcgaatggt gtatatacgg ttactggatc ttataagaaa gctcaaaagt
422641 caaagaatca aaataatact ggatttggac ttattccaag ttcacaaagt agtagtgaag
422701 taactcgttt cagtactacg atgcttcaaa atgactcaat ggttaagaca gtttcagatc
422761 ttgctcaaaa gagcggagca tcaatttctg gacaaggtga atctcaatct ggaacctgga
422821 tttctactat tgtcatgctt gttccaactt tgatttttat tgtcatgctt tggatgatga
422881 tgaaccaagg tggacagggt cgtggtggcg gcatgatgaa ctttggaaaa tctcatgtta
422941 agccacaaga tccaagtaaa aataaggtta gattctcaga tgtagctggt gaagaagaag
423001 aaaagcaaga gttagttgaa attgttgaat tcttaaagaa tccagcaaaa tatactaaat
423061 taggtgctag aattccggct ggtgttcttc tcgagggtcc tcctggtact ggtaaaactt
423121 tacttgcccg tgcggttgct ggtgaagctg gcgtaccatt ttattcaatt tctggttcag
423181 actttgttga aatgtttgtc ggtgttggtg catctcgtgt acgtgactta tttgaaacag
423241 ctaagaagaa tgccccaagt atcattttca ttgatgaaat tgatgccgtt ggacgtaaac
423301 gtggcaatgg tgttagcggt ggtcatgatg aaagagaaca gactttgaac caactattgg
423361 ttgagatgga tggtttcgaa ggtgatgaag gtgtcattgt tatggcagct actaaccgtt
423421 cagatgttct tgatccggca cttcttcgtc ctggccgttt tgaccgtaag gttttagttg
423481 gtcgtccaga tgttaaagga cgtgaagcta tcttaaaagt tcatgctaag aataaacctt
423541 tagcaccaga tgtcgactta aaagaagtag cacgccaaac tcctggtttt gtgggtgctg
423601 atcttgaaaa tgttttaaac gaagctgctt tagttgctgc tcgtcgtaat aagactgaaa
423661 ttacagcatc tgatattgat gaagcagaag accgcgttat tgcaggacca gctaagaaag
423721 acactgttat ttctcctgaa gaaagaaagc gtgttgcata ccatgaagca ggacacgcaa
423781 tcgttggttt agtccttagc gattctagaa ccgttcgtaa agttacgatt gtacctagag
423841 gtcgtgctgg tggatataac attatgcttc caaaagagga tgaaaatatc atcactaaga
423901 agcaattaat ggaacaagtt gctggtttaa tgggtggtcg tgctggtgaa gaagtagttg

423961 ttggtgataa atctacgggt gcttcaaacg actttgaaca agccactaac attgctcgtg
424021 gtatggttac tcaatatggt atgactgatg ttggtatgac tgagcttgaa tctccaagta
424081 tgcaatcacc ttatggaact aagccataca gtgaagctac agctgctaag atcgatgaag
424141 cagttaaaga aattcttgat gaaggtcaca aacaagccgt tgatatcatt aagagccacc
424201 gtgaaactca caagattatt gcagaagcat tgctcaagta tgaaacttta aacgaaaagc
424261 aaattctttc attattcaag actggtaaaa tgccagaaaa tgatgagaat gaatttccaa
424321 gtgaatctaa ggcttcaact tatgaagaag caaaagctgc aatggaaaag aaggatcaac
424381 aaagagaaga aactgaaaat aaggatgaca aagaagacgt tcatccactt cctgatgatc
424441 ataagaatat tgacgatatt ccacttaatc caccatctga aaagaaagat tctgacgaac
424501 acaattcaga tgaaaacgag gataggtaag agtttattgt aagagtaatg tttatactcc
424561 tttctaatac tgaaattaca gattttagaa aggagttttt tggtattaag aatgaaatat
424621 ttaagtaaaa aagacaaaaa gattttaaaa ttaagcttaa taagttgttt gacaactata
424681 gtgctctttt tccttttatc tactttcact gattgtaatc caatctttgg aggagtattg
424741 tacggaggag atttacctaa tcagtactta tctttctttc aatactatcg tcatttagtt
424801 ttaggaaatt gggctagtgc tgggtatact ttttctaatg gactaggtgg agatatggct
424861 ggtaacattg cttactatgt cttaagtccg ttaaatttta tagtttttct ttttcctgca
424921 aataaaatca atattgccgt ttatgtaata atcctgctta agctaggttt gatgagtggt
424981 acatttactt ggcttatttt aaaatggttt aactttaaat atcaggccta tgcaattttt
425041 ttaggaattg cgtatagttt aagtggctat tcagttgctt atgctggaaa tgtcatgtgg
425101 tttgatggat taattttgtt gcctttaatc acttatgctt tggttagagg aatcaagact
425161 aataaatggc tcgcgtatag tatcttactg gcttgtgcca ttatatttaa ttactatatt
425221 ggttacatga tttgtatttt tatggtaatt attttcctag catatacaat caataatttt
425281 aaagacataa gactattttt ccaccaattc ttgaattttg cgatcagttc aattattagt
425341 ggtttaatta gtgcaatagt cctattacca actctattta atctctcaag taataaatta
425401 gcacagtcgg attttaattc agattttaat attaagctac ttattactgg gggaaaaaca
425461 gtatcaagac tgtttattgg ggatacttac aatgattggc cgccaatttt tgtaggtaca
425521 ttagctgcta taatttttat tatctacttt attgattctc gtaattcagt taaagctaga

425581 attactaatc tgataattgg aattatgttt gtcttaagca ttatcgtgcg
tccgttatac
425641 ttattctggc atggtggaca acaaactatt gcttatcctt atcggtttag
tttttaatc
425701 gtattttgga ttttgctttt agcagcaaaa gagctgtcat atcaagactt
taagagaaaa
425761 gatagaatta tagctaccac tatttattta ttgctgagtt tgatgactgt
atatattcgt
425821 cgtcgaattg gaccaaataa tttttatgaa tggatagccg tagctttagt
acttctcttt
425881 ggactattaa tctacttttc aaataaaaac ttcgttcgaa ttattctaat
attagttgga
425941 ctagtcgaat tatcgggaaa cgcatatact ggccttagcc atcttggaat
gaagagtgat
426001 tactatccta gatacgttgc agaaaataat gaaataattt cgaagatccc
agcagctgat
426061 aagacaggta gaatcgctaa aaattatgaa ctaaataatg atcgcggtga
aggatatact
426121 tttaattatc gtggggtaga agaattttca tccaataatg attcacgaat
tagtagtttg
426181 atgactgatt taggattttc aacatttaga tacttctatt attatcaaac
tggtactgta
426241 gttactgatg ctatttttaa tgttaagagc tttattaaca gttctttgac
taatcaaagc
426301 atttcaccag aatatgttaa ctatggttta agagatgatt taaaaactag
accagttatt
426361 ttaaaacagg gcgataaaac tgtgtatcga aatgaaacac ttccatttgc
ctttgctggt
426421 aatttatcca ataaattaaa gtttaaagat gaaaatcctg tctataatca
aaacttagtt
426481 ttaaattctt tgacccaatc taaatcaaat gtgttggatt atagtactaa
aaaggctcga
426541 attacaacaa ataacttgtc agtgaaatat ggtacagtaa agtatcatgt
cataaagaaa
426601 cataagaaga ttactaaacg tactgaacaa aaatatttca caattaaaag
atatgataaa
426661 gagcgtcctg gtacgatgag tttcacctat gataatctga aacctaacca
agtgggatat
426721 attcgattta gcaaaaattt gatgcaatta gtacttcctt taaataatta
tcaatggaat
426781 aaaaatcctg attatcgtcc tccatttagt ttgactataa acggtaaaca
agttcaactt
426841 caagaatata cagatcaact aattggcgtt caagccgata agaatggaaa
agttgatatt
426901 aaaatgacca tagatggaaa aggcggcaag ttcattctca aataccctaa
atttgtgaat
426961 atcgactttt cagctttgca tgataaagtc aaaaaagctc gtaacagaga
aatgaaattt
427021 acaagcttta aagatggata tgtagctggt aaagttacaa ttgataaaag
tcaaaactta
427081 gtgactacta tcccttatag taagggctgg caagctaaag tagacggaaa
accagttaaa
427141 attaatcgta cattgggtgt atttattgga ttaaagatga aaccaggaac
tcatcaaatt

427201 acattgaaat atagaacccc aggtgtatta gtaggtgcta tcttgagcat tattgggatt
427261 atttccttaa ttgtatttac tttattttta aagaaaaata agaaagatta attgattaga
427321 tacacttctc gatgcttgtt ttggagaagt gtatttttta agtaggtaaa aattcagtaa
427381 tatcgacagt attaaattgc ttattggcgc tgtaacaagt atcataggta taatcctact
427441 agtggtattt aatttttttg aaaaagaaaa agtattagtc aaaaagtaaa gtctatgttt
427501 tataggcttt atttttagg ggaaagaaaa aatgaaagat tatttagtaa aagcaattga
427561 taaaactaaa aacttacgat tgattacagt taatgccaaa ggtcttgttg gtgaagcgca
427621 aaaaagacat gatacatgga gcgcatcgtc agctgtactt ggtagaactt taattggcgg
427681 tctgctttta tcagcagcct tattgaaaga taaagatgaa ttgacagtta gattactagg
427741 caacggtcca gtaggagcaa ctgttgtaac agcaaaagct gacttgacta ttaaggggta
427801 tatacaaaat ccgcatattg ctcttcctcc taagaaagat ggacatattg atgtagcaag
427861 agctgttggt gaaggttggc ttgaagtaac caaagatcaa ggcctaaaag aaccttatac
427921 cggtcaggtt ccaattgtta gtggagaaat tgcagaagat ttcacatatt atttagcaaa
427981 atctgaacaa attccttcag ctgttggctt gtctgttttt gttgaaccaa acaatagtat
428041 cggagctgcg ggcggttttg tattacaagc attgccagga gcaactgacg aacaattagc
428101 tcaagtagaa aagagaatta aagctcttcc gaatctttca actttattcc tagatggaat
428161 gacacctgaa aatctagccg aaaggatcct aggaactgat tgcaagattt tggctaaaga
428221 agatgttgcg ttttcctgtg attgctcaaa agaaaaatac agcaaaattt tagctacact
428281 taagccagga caactaaaag aaatgattga aaaggatcac ggtgcagaat tagtttgtcg
428341 tttctgcaaa gaaaaatatc actttactga agatgaatta aaagatgtcc ttaagaaggt
428401 aaattaaagt ggtgtgtagt agatagtttt gctatgatat tagggtattt gaaaggatga
428461 gcaagtgtga aaaacgatag ttggaaaatt agggatgttg aaattcctaa tcgtgtagta
428521 gttgcaccga tggctggaat ttcaaatgct gctttccgag tagtatgtaa agaatttggc
428581 gcaggcctag tggtttgtga aatgatttca gatcatggaa ttatttaccg caataaaaag
428641 actttagaga tgttgacagt tgatcctaga gagcatccga tgagtattca aattttcggt
428701 ggaagtgaag aaactttagt tgaagctgct cattatgtgg atactcatac tgcagctgac
428761 attatcaaca tcaatatggg ttgtcctgta ccaaaagtaa ccaagactga tgctggagct

428821 cgttggttat tggatccaaa taagatttat caaatggttc atgctgtagt gagaaatgtg
428881 aacaaaccag taactgttaa aatgagaaca ggctgggatc aaaagcatat atttgctgtt
428941 gaaaatgcct tggctgccca agaagctggt gctagtgctg ttgctatgca cggacgtact
429001 agaaaacaaa tgtatatggg tgaggcagat tgggaaacct taaaagatgt tgcagatgca
429061 ttaaccatac cttttgttgg taatggagat gttacaacac ctgaaaaagc aaaatcaatg
429121 cttgaagatg ttggagctga tgcagtaatg gtcggtagag cagccttggg aaatccatgg
429181 attattaagg acatggttca ttatttagac actggtgaaa aactacgtcc acaaactgtt
429241 gaagaaaaag tggcaactgc taaagaacag ttaaatggct taattgatct taaaggtgaa
429301 aaaattgctg taccggaatt tagacgtcaa gctgcttatt atttgaaagg aattccccgt
429361 tcagctcgaa cccgtgctaa aataaatgat gtttggacga aacaagaagt ttttgacttg
429421 ttagatgact ttgtggaaaa atatgaagca agacaaaagc aaattaatcg ataaaaggag
429481 tttttagagt ggcaaagaat gaaatgaacg accaactaat tgttcgtcgt gaaaaaatgg
429541 acgaaatgcg tgaaaatggc attgaacctt ttggtgtaag aaagtttgat cgtcaagact
429601 tagctcgtac tttgaatgaa aagtatagta aagaagataa agatgaatta aatgccgata
429661 tgcctatgac caaaattgca ggtcgtatgc ttgccaagcg tggtaagggt aaagtaggtt
429721 ttgctgatct ttatgaccgt actggtaaga ttcaaattta tgtacgtaaa gatattgttg
429781 gcgaagacaa ctacaagatt tttaagaaat cagatattgg tgacttctta ggcattgatg
429841 gggaagtaat gaaaactgat actggtgagt taactattcg tgctactcac gttactttct
429901 tagctaaagc tcttcgccca ttacctaata aatgggacgg tttgaaagat gttgaacaaa
429961 tttatcgtca acgttatctt gatttgatta ctaatcacga aagttacatg cgttttgttc
430021 atcgtactca aattattcaa gctattcgta actacttaaa taaccaagac tttttagaag
430081 ttgaaactcc agttcttcac aatattccag gtggtgctga agcacgtcca tttattactc
430141 accacaacgc tttagacatt gatctttaca tgagaattgc gttggaactt ccactgaagc
430201 gtttgattgt tggaggtatg gaaagagttt atgaaattgg ccgagtattt agaaacgaag
430261 gtgttgatac tcgccataat cctgaattta ctgagcttga aacttatgct gcttactggg
430321 atttccacga tgtcatggat gaagcggaag gaattatcag agcagctgct aaagtagttt
430381 ctccagatgg taagattaac taccaaggta ctgatattga cttaggtaag ccattccgac

430441 gcgttcatat ggttgactta attaaggaaa agactggtgt tgacttctgg aaagaaatga
430501 ctgacgaaga agctaagaag atcgccgaag aacatggaat tcatactgaa ccattctgga
430561 aagtcggtca tgtaattaat gctttctttg atgaattttg tgaaaagact attgttgatc
430621 caacctttgt ttatgggcac ccggtagaaa tttcaccact tgctaaaaag aatgcagatg
430681 atccaagatt tactgatcgt tttgaaatct ttattttggg aatggaatac ggaaatgcct
430741 tctcagaact aaatgatcca gttgatcaac gtcaccgttt tgaagaacaa atggaagaac
430801 gcgaagctgg taatgatgaa gctgatatga ttgatgaaga ttatatccgt gcaatggaat
430861 ttggtatgcc tcctacaggt ggtctaggta ttggtattga ccgtttggta atgcttttaa
430921 ctgatgctcc agctatccgt gatgtattgc tcttcccaac aatgcgtcct gaaaaggttg
430981 aagacgttga tgccgaagtg caagaagact tgaagaaaaa gaataaataa tttaaaaaat
431041 aggctgtctt tgaagatggt ctattttttt gatttttca gaaaaagtac ttgcaatcga
431101 atgatgtttt gctatactag taaatgtact gcggaagtag ttcagtggta gaacatcacc
431161 ttgccatggt gggggtcgcg ggttcgaatc ccgtcttccg ctccagtata aaagactcac
431221 cgaagcgtga gtcttttttg atattcggga tgtggctcag cttggtagag cgctacgttc
431281 gggacgtaga agtcgcaggt tcaaatcctg tcatcccgat tgaatattat ttttattaaa
431341 aggttcgtct taagctagaa agacgaactt ttttgtatat aatgttcatg aaatatcaaa
431401 tagtttacat ttactaattg taagtgtaag atatttgtct aaagattgta gtatgatctt
431461 aaaagaaata aggcggtgca gaatggaaaa atcatatagt gatagtgcaa agaatgttct
431521 tgaaattgct aaagagcaag cacaaaactt tcaccatcga attattggaa cggagcatgt
431581 tttgcttgct ttagtaattg aagctaacgg agatgccggt aagcttttgc gtgaaagaaa
431641 cgtaacacca accttagtgc gagaagaaat tgaaagatat actggctatg gttcaagtcc
431701 caaagctaca tatatggaaa tgtcaccacg cttaagctta gttttgaatt ttgctaagca
431761 aaaatctgac gaattaggca ctcctcaaat tgaaacaaag catattttgc tagggttatt
431821 agctagtgac caaattttgg caagtttaat tttgaaaaat attggtgtag aaccacgtga
431881 cttaagtcaa gatattaatg atagtttcat ggatgattct ggagaagcaa acaatatttt
431941 tgatgtttct tctgcaacta gaactaaaaa aggcaagtca ctgacaccta atttagataa
432001 agtaagtgta aatttaaata aacgtgcacg cgagggtgca attgatccag taattggtag

432061 agataaagaa ataaagcgag taattcaaat tttatctcga agaactaaga
ataatcctgt
432121 tttagttgga gaacccggag ttggtaagac agcagttgca caaggaatag
cttctgcaat
432181 tgtaaatcgt gaggttccgg ataacttagc taaaaaacgt gtcatggcac
tcgacatggg
432241 aagcttaatt gctggaacta agtatcgcgg ggaatttgaa gatagaatga
aaaaaattct
432301 aaaagaaatt caacgtgatg gttctgtaat tctatttgtt gatgaaatgc
ataccttgat
432361 tggtgcaggt ggagcagaag gtgcaattga cgcttccaat atcctgaagc
catctttagc
432421 gcgtggtgat attcagatga ttggtgccac tacttttgat gaataccaaa
aatacattga
432481 aaaagatcaa gccttggcaa gacgttttca acaagtgaaa atcggtgagc
cttcaaagga
432541 agaaacagtg gacatcttaa aggggttacg tcctaagtat gaaaaattcc
ataatgtaaa
432601 aattgaagat gaagcaataa atgatgcagt agaattttca acaaggtata
ttgctaaccg
432661 ctttttacct gataaggcaa ttgacttaat cgatgaagca agtgcagcgg
tcaagattca
432721 agcagttggt aaggctgatc cacgtttgac taaattagat acacaaatta
atgatgttat
432781 tcaccgaaaa gaagaggctg ctgaaaatca aaactttgtt caagcggcta
aattacgtga
432841 tgaagaaagc aagcttacac aagatcgaga taaattaatt gcaaaagtag
aaaataagaa
432901 ttctaagaag tctattgttg attcaaataa aattgcccaa attgtatcag
aatggactgg
432961 cgtaccagtt actcgaatga agaagagtga aacgaaacgt ttagctcacc
tcgaaagcat
433021 tttgcatgaa cgcgtaattg gacaagataa ggcaattagt gcagtaagtc
gtgcgattag
433081 acgaagcaga agtggaatta aggatgaaaa tcgtccaatc ggttccttct
tgttcttagg
433141 tcctactggt gttggtaaga ctgaattagc aaaagcattg gcagcagcag
tctttggctc
433201 tgaaaggaat attattcgag ttgatatgtc tgaatatatg gatcaagtag
ctacaagcaa
433261 gttgatcggt tctgctcctg gttatgtagg ctatgaagaa ggtggacaac
tttcagaacg
433321 ggtaagacgt aatccatatt cagtaattct gctagatgaa gtagaaaaag
ctcatccgga
433381 tgttttcaat ttattgttgc aagttttaga tgaaggtttc ttaactgact
ctaagggaag
433441 aaaagttgat ttcagaaaca caatcattat tatgacttct aaccttggtt
ctcgtagctt
433501 gcaagaagat aaaactgttg gttttgcagc tgataatgaa gataaaaata
aacttcaaca
433561 agaaaaggtt acagcagcag ttaagcaatt ctttagacca gaatttttaa
atagaattga
433621 cgaaacagtt gtctttgata gtttaactaa gaaacaattg cgtgaaattg
ttagtttaat

433681 gactagtcat ttgatcactc gattagctcg gaaagaagtt actctaaaaa tttctccagc
433741 agcgttagat gtgttagcta aagacggttt tgatccagaa atgggtgctc gtcctttacg
433801 tcgtgcaatt caacatgaac ttgaggacgt gattgcagaa gatttaatta gtgaaaaggt
433861 taaagccggg caaacagtta aggttggcgc tcatcaggga aaattaaagt tcacaattgt
433921 tgatgagagt aagcctttag ttaaaaatta gttccttttg acaaattagt aatataagtg
433981 taaaatacaa aacgtttaaa aggctgaaat tcaggatttc gctgatctat tgtccagcga
434041 gatgataaaa caaaaacgac tattacgttg tttttgtttt tttgtaccca aattttgact
434101 ttttgcaatt tgtaacacaa atgtaatatt tagattctta tataagaaag gatgttttct
434161 ttgttaggac acgctgtgaa ctacggtagt caccggacaa gacgtagctt ctctcgaatt
434221 aaagaagtat tgaagttacc taacttgact gatgtgcaaa cgcaatctta caagtggttt
434281 cttaatgaag gtattcgtga aatgtttgac gacattatgc cgatttcaga cttttcagga
434341 aaactttctt tagagtttgt tgactacaaa cttctgaagc caaaatatac tcttgaagaa
434401 gcacgtgatc acgatgctaa ttattctgct cctcttcatg taactttaaa gttaaccaac
434461 catgaaactg gagaaattaa gactcaagat gtcttcttcg gagaattccc attaatgact
434521 gattcaggta ctttcgttat taatggtgct gagagagtta ttgtttctca gcttgttaga
434581 tctcctggtg tttactacca ttcagatttt gataaaaatg gtcgccaaat tttcggtgct
434641 acagttattc ctaaccgtgg tgcatggctt gaatatgaaa ctgatgctaa ggatttagct
434701 tatgttcgta ttgaccgtac tcgtaagctt cctttaacag ttctaattcg tgctttagga
434761 tttggctctg acagtgaagt tgctgatatg tttggtgaaa gtgattcact tcgtttcact
434821 ttagaaaaag atatccataa aaatcccgct gactctcgtg ttgccgaagc tttaaaggat
434881 atctatgaaa gattacgtcc aggtgaacct aaaactacgg attcatctcg ttcactttta
434941 tatgctagat tctttgatcc aagaagatac gatttagctc ctgttggtcg ctacaagatc
435001 aataagaaac tttctttaaa gaatcgttta ttaagacaaa ctttggctga aactttagct
435061 gatcctgata ctggtgaaat tatcgctaag aagggtgacg ttgttactca tgaaattctt
435121 gataaattgt caccttactt agatcgtgat gactttaaga tggtaactta tgaaccttca
435181 aaggaaggtg tattacctga tccagtaaca gttcaagaaa ttaaagtata ttccaaagtt
435241 gatcctgaac gtgtaattaa gttaatgtct aacggtcata ttgctgatga cgttaaacac

435301 ttaactccag ctgatgtttt ggcatcaatt aattacttct ttaaccttca agataacatc
435361 ggaactactg atgatatcga ccacttaggt aaccgtcgta ttcgtcgtgt aggggaatta
435421 cttcaaaacc aatttagaat tggtttagca agaatggaac gtgttgtgcg cgaaagaatg
435481 tcaattcaag atatttctac agttacacca caacaattaa ttaacattcg tcctgttgtt
435541 gcttcagtta aggaattctt tggttcatca caattgtcac agttcatgga ccaaaacaac
435601 ccacttggtg agttaactca caagcgtcgt atgtctgcct tagggcctgg tggtttatct
435661 cgtgatcgtg ctgggtacga agttcgtgac gtgcactata ctcactatgg tcgtttatgt
435721 cctattgaaa ctcctgaagg acctaacatt ggtttaatta actctttggc aacttacgct
435781 attgttaata agtatggttt tattgaaaca ccataccgtc gtgtttcttg ggatactcac
435841 aaggttactg acaagattga ttacttaact gctgatgttg aagataatta cattatcgct
435901 ggtgccaacg ctccattaaa cgaagacggt tctttcaagg ataagattgt tttagctcgt
435961 cataaggaag ataaccttga agttactcct gataagattg actacatgga tgttattcct
436021 aagcaagtag tttccgtaac ttctgcatgt attcctttcc ttgaaaacga tgactctaac
436081 cgtgccttga tgggtgctaa ccaccaacgt caggctgttc cattgattaa tccacacgct
436141 ccaattgttg gtactggtat ggaatatcgt gcagctcatg actctgggga tgcattagtt
436201 gctaaagcac ctggtgtagt tgaatacgtt gatgcaaacg aaattagaat tagaagagac
436261 gatgatactt tagataagta tgttcttgaa aaattccgtc gttcaaatgc aactaagaac
436321 tacaaccaaa caccagctgt taagcaaggt gaaagagtag tagctgacga agttattgcc
436381 gatggtccag caatggaaaa cggcgaatta gctctaggtc aaaacccaat tattgctttc
436441 ttgacttgga acatgtacaa ctatgaagat gccgttatga tttctgaacg tatggttaaa
436501 gatgatgtct acacatctat ccatattgaa gattatgaat cagaagctcg cgatactaag
436561 cttggacctg aagaaatcac ccgtgaaatt ccaaacgttg gtgaagatgc acttaaagac
436621 ctcgatgaag aaggtattgt acgtattggt gccgaagttc aagacggtga tatcttagtt
436681 ggtaaagtta ctccaaaggg tgttactgaa ttatcagctg aagaacgttt gcttcacgct
436741 atctttggtg aaaaagctcg tgaagttcgc gatacttcct tacgtgtacc acatggtggc
436801 ggcggtattg ttcaaaacgt acaagtcttc actcgtgaag caggagacga attaccacct
436861 ggtgtaaaca agatggttcg tgtctacatc gttcaaaagc gtaaaattca ggttggtgac

436921 aagatgtctg gtcgtcacgg aaacaagggt actattgcct tagtttgtcc tgaagaagat
437041 atgccatact taccagatgg tcgtccagta gacatttgtt tgaacccaat gggtgtgcct
437041 tcacgtatga acattggaca ggttcttgaa ttacatttag gtattgcagc taagcaattg
437101 ggtattcatg ttgctactcc agtatttgat ggtgcttctg aagatgatat gtggaacatg
437161 gttcgtgaag ctggtattgg taaagacggt aagactgttc tttatgatgg ccgtaccggt
437221 gaaccattcc acaaccgtgt ttcagtaggt attatgtact acttgaagtt gactcacatg
437281 gttgatgata agatccacgc acgttcaatt gggccttact cacttgttac tcaacaacca
437341 cttggtggta aagcacaatt tggtggtcag cgttttggtg aaatggaagt ttgggctctt
437401 gaagcttacg gtgctgcata cacattgcaa gaaatcttga cttacaagtc agatgatgtt
437461 gttggtcgtg tgaaggcata tgaagctatt gtcaagggtg aaagaattcc aaaaccaggt
437521 gttccagaat catttagagt tcttgttaag gaacttcaat ccttaggttt ggatattaaa
437581 gtcttagata tggaccacaa ggaaattgaa ttacgtgaca tggatgatga ttctaatgat
437641 cacttcaaca ttgacacttt atctaagctt gctgaacaac aagaaaagaa gaagttagcc
437701 gaagaagctg caaagaaaga tgataagtca gccaaacctg tagatcagag tgattcttca
437761 acttcatctg atgataaggt ttctaagtaa taggaggtta aacttttgat cgacgtaaat
437821 aagtttgaaa gtatgcaaat tggtttggct tctccaaaca agatcagaag ttggtcttat
437881 ggcgaagtta aaaagccaga aactatcaac tatcgtactt tgaaaccaga aaaagatggt
437941 ttgttcgatg aaagaatttt cggaccaact aaggactggt cttgtgcttg tggtaagtac
438001 aagggtattc gttatcgcgg tattgtttgt gaccgatgtg gtgtagaagt tacttctgct
438061 aaagttagaa gagaacgtat gggtcacatt gaattagctg caccagtttc acatatctgg
438121 tatttcaagg gtattccatc tcgtatggga ttagtgttag atatttcacc tcgtgcactt
438181 gaagaagtaa tatattttgc tgcttacatt gtaattgacg caggtgatac tgatcttgaa
438241 gataagcaac ttcttacaga agctgaatat cgtgaaaaga aagcaaaatt cggtaaccgt
438301 tttgaagcta agatgggtgc tgaagctgta aaggaacttc ttgagcaagt agatattgat
438361 aaagaagttc atgacttaaa agaagaatta aagactgcta ctggtcaaaa gagaactcgt
438421 gctatcagaa gattagatat cttagatgca tttaagaatt caggcaacaa gccttcatgg
438481 atggttatgg attgtattcc agttattcca cctgatttaa gaccaatggt tcaacttgat

438541 ggtggtcgtt ttgcaacttc tgacttgaat gatttatata gacgtgtaat taaccgtaac
438601 aatcgtttga agagattatt agacttaaat gcacctagaa ttattgttca aaatgaaaaa
438661 cgtatgcttc aagaagctgt tgatgcatta attgataatg gtagacgtgg tcgtccggtt
438721 gtaggaccag gtaaccgtcc acttaagtct ctttctcaca tgttaaaggg taagcaagga
438781 cgtttccgtc aaaacttgct tggtaagcgt gttgactact caggtcgttc agttatcgat
438841 gtttcaccaa agttgaagtt ttatcaatgt ggtgttccac gaccaatggc tttagagtta
438901 tttaagccat ttgtgatgca tgaattagta aaacgtggtt tagcatctaa cattaagaac
438961 gctaagcgta agattgatcg tgaagatgac gatatctggg atatcttaga agatgtaatt
439021 aaggaaagac cagttctttt gaaccgtgca cctactcttc accgtctagg tattcaagca
439081 tttgaaccag ttttggtgcc aggtaagtca attcgtcttc acccacttgc ttgtgaagct
439141 tacaatgccg actttgatgg tgaccagatg gccattcacg ttccattatc tgatgaagct
439201 gtagcagaat cacgtttgct tatgcttgct gctcaccata tcttggctcc taaggatggt
439261 aagccaatcg ttactccttc tcaggatatt gttttaggta actactggct aactcaagca
439321 gaacgtggtc gtgaaggcga aggtatgatt tttgattcac cagctgaagc agctattgca
439381 tatgcaaatg gtgatattca ctaccacact cgtattggtt tagcggctga ttcaatgcct
439441 gaaaagccat ggccgaaggg atatgaacac ggaattttg taactactta tggtaagtta
439501 gtcttcaacc aaatttttcc taaggatttc ttctacatta atgatcctac tcaagaaaat
439561 cttactcatc ctgtggatga aagatacttc ttacaaccag gtgaagatat ccatgaaaag
439621 cttgacaata tgaagcttgg aaaggcattt aagaaaggat tcttatcaga ttccattgct
439681 caaatttaca aggattataa agttcaaaga acttctgatt tcttggatga cttgaaggaa
439741 ttaggttaca ccgtatgtac tacttctggt ttgactattg gtgtcgaaga tattcctaca
439801 attactgata aggatgatat cgtagcagaa gctcgtaaga aagttgatgt agtttctaag
439861 cagtatcgtc gtggtttgat tactgatgaa gaaagacatg atcgagtaat tagtatttgg
439921 aataattgta aagatattgt tcaaaacgaa attgctcaaa ttcatgcccc aagaaaccca
439981 attacaatca tggcggattc tggtgctcgt ggtaacattt ctaactttac tcaacttgct
440041 ggtatgcgtg gattgatggc cgctcctaac ggaggaatga tggaaattcc tgttacctca
440101 aacttccgtg aaggattatc tgttttggaa atgttcatgt ctactcacgg tgctcgtaaa

440161 ggtatgacgg atactgcttt gaagactgcc aactcaggtt acttaacccg tcgtttggta
440221 gacgtagctc aagacgttat cattcgtgaa gaagattgtg gcactgatcg tggcttgact
440281 gttcatgcaa ttactgaagg cgatgaaatg attgaaccat tatttgatcg tctagtaggt
440341 cgctacactt ctaagagcgt ttatgatcca gaaactcatg aagttatctg tccagcagat
440401 gtcttaatgg atgaagatat ggctcataag attgttgatg ctggcgttac tgaagtaact
440461 attcgttccg tatttacttg caacactcaa catggtgtat gtaagaaatg ttatggtatg
440521 aaccttgcta ctggtgacga tgttgaagtt ggtgaagcag taggtactgt tgctgctcaa
440581 tctatcggtg aacctggtac tcagttgact atgcgtaact tccacaacgg tggtgttgcc
440641 ggtgctgcag atattactca aggtttacct cgtgttcaag aattgtttga agctcgtaat
440701 cctaaaggac gtgctacaat ttctgaagta actggtgaaa taacttccat tgaagaagat
440761 ccagcagaac acactcgtca aattacagta aagggtcaaa cagatactcg tacttatgat
440821 gtaccatata ctgcatctgt agcagttgct gaaggtgacc atgttgtacg tggagataaa
440881 ttgactcttg gttctattga tcctaaggaa ttgattcgtg tacgtgatgc attgactact
440941 gaaaaataca ttctaagcga aattcaaaaa gcttatagaa tgcagggtgt agaaattgcc
441001 gataagcacg ttgaagttat ggcacgtcaa atgcttcaaa aggttcgtat tcttgatcca
441061 ggtgaaactg atatcttacc aggtgaattg atggatattg gtgaatttaa ggcaagaaac
441121 cgtgaggtaa ttatttctgg tggtattcct gcaactgcac aaagtgtaat tcttggtatt
441181 accaaggccg ctcttgaaac taacagtttc ttgtctgcag cttcattcca ggaaactact
441241 cgtgttctta ctgatgcttc tattcgtgga aagaacgatc cattacttgg tcttaaggaa
441301 aatgttatca ttggtaagat tattcctgct ggtactggta tgcctgttta ccgcgaaatg
441361 gaacctaagg ttgacattcc tgaagatgaa aagaaaaagt cagtttattc tatagctgat
441421 attgaaaaaa aattagctgc agctgatgca gaaaagaacg atggctcagc tgactaacta
441481 tccttttaaa acttaaacat ttgtgttaaa aaagtctatt cgcttttgtg cgaatagact
441541 ttttttatat gaaattataa aaaataatgc tcagaaatat aaaaggtatg aaaggatatt
441601 gtttttttgtt ccgagcaatt aaatgagtaa ttatcagaaa aatggatgac aataggaaaa
441661 gatactcagc aatgtagaag ttatagtaaa aaataagaat aataaaaatt attgtatcgc
441721 cataaccaaa tttctttttc aaattcatta ttaaaaaact taatataacg aaagaaatta

441781 acatccattc aattggagtg taatcagaaa taaagtagga aattttaaat cctactactg
441841 ctataagtgg gataaggatt tgaactggaa aagattgatc aaaataatca aaaatactaa
441901 ttaaaaaaat aaaaataatg aaaaagtatg aaatttgatt agtaagcgga tttaacggca
441961 taaaagctaa tccacctaaa atttcgcaat aaaatgtttc aataggaata taagcgtgac
442021 agaagaaaca ttttccttga tatcttagaa aagaaaaaat aggtatttgg ttaagaagcg
442081 taagtgggat ttggcagtta tcacaatgag atttatcggt tataaagtct tctgtctcaa
442141 agcgctgaac aattactagt aaatgagaac caataatact accgattata aaattaagta
442201 tataaataaa tgaatttgac atcttttaac ctccaatatt tatatacgca aaatcaaaaa
442261 atttttttta aaatgtttac ggaattaaaa aatcatgtta cactagccat tgtgctatta
442321 aaaacatgat atccgtgagg tcaaaaaaga aactcccgga tgtgtgaaca attttaggag
442381 gaaattttaa tgccaacaat taaccaattg gttagaaaag gccgtcactc aaagacgact
442441 aaatctgatt caccagcttt aaactatgct tacaacagca tgaagaaaaa gatgaactat
442501 aatccagcac ctcaaatgcg tggtgttgca actcgtgtag gtactatgac acctaagaag
442561 cctaactcag ctttacgtaa gtatgctcgt gttcgtcttt caaacttaat cgaagttact
442621 gcttacattc ctggtattgg tcataacttg caagaacact ccgttgtttt aattcgtggt
442681 ggtcgtgtaa aggaccttcc tggtgttcgt taccacatca ttcgtggtgc tttggatact
442741 gcaggtgttg atggtagaaa acaaggccgt tctaagtacg gtgctaagaa aggttaaaac
442801 agttaaatag gagggactac taatgcctag aaaaggtaat gttgctaaga gagatgtttt
442861 agcagatcca gtttacaact ctaaattggt tactaaatta attaaccact taatgattga
442921 tggtaagaaa gcaaaggcat cttcaattct ttatgatgct tttggtatta ttcaagataa
442981 gactggtaaa gaaccagttg aagtttttga agaagcaatg aacaatgtta tgccagtttt
443041 ggaagttaaa gctcgccgta tcggtggttc taactaccaa atcccagttg aagttcgccc
443101 agaaagaaga actactctcg gcttaagatg gcttgtttct tacgctcgtt tacgtaatga
443161 acatactatg gatgaacgtc ttgcaaacga aattatggat gctgctaaca atactggttc
443221 agcagttaag aaacgtgaag acgtccacag aatggcagaa gcaaaccgtg catttgctca
443281 ctaccgcttc taatcgttag tttgtaaaag gagattaatc ttatggctaa caaacgtgaa
443341 ttccctttgg ataagacacg taacattggt attatggctc acatcgatgc cggtaaaact

443401 accactactg aacgtatttt gtactacact ggtaagatcc acaaaattgg
tgaaactcac
443461 gaaggtgact cacaaatgga ctggatggat gaagaaaagg aacgtggtat
cactattact
443521 tccgcagcta ctactgctca atggaaggat taccgaatta acatcattga
taccccagga
443581 cacgttgact ttactatcga agtagaacgt tcccttcgtg ttcttgatgg
tgcggtaact
443641 gttcttgatg ctcaagctgg tgttgaacca caaactgaaa atgtttggcg
tcaagctgaa
443701 acttacggcg ttcctcgtat tgttttcgtt aacaagatgg ataagattgg
tgctgacttt
443761 gataaatctg ttaagtcatt gcacgaacgc ttaaacgcaa acgcacatgc
tgttcaaatg
443821 cctattggtt cagcagatac ctttgaaggt gttattgact taatcaatat
ggtagctgat
443881 gtttacgatg aagataagct tggttcaaaa tgggatacta ttcctgttcc
tgatgaatac
443941 aaggaagagg ctttaaagcg tcgtaacgaa ttaattgaag ctgttgctga
tgttgatgac
444001 ggcattatgg acaagtacct tggcggtgaa gaaatttcta atgatgaatt
aaaagcagct
444061 atccgtaaag ctactttgaa cttggaattc ttcccagttt atgctggttc
agcatttaag
444121 aacaagggtg ttcaaatgat gcttgatggt gttattgatt acctaccatc
accacttgac
444181 gtaaaacctt acgttgctca tgatcctaag actggtgacg aagttgaact
tatggctgac
444241 gataagaaac catttgcagc tttagcattt aagattgcaa cagatccatt
tgttggtcgt
444301 ttaactttca tccgtgttta cactggttca cttcaatctg gttcttacgt
attgaatgca
444361 tcaaagaatg ctcgtgaacg tgttggacgt ttacttcaaa tgcacgctaa
ctcaagaact
444421 gaaattcctg aagtattctc aggtgatatt gctggtgcta ttggtttgaa
gaacactact
444481 actggtgatt ctttaactga tccagatcac ccacttattt tggaaagttt
acaagttcca
444541 gatccagtta ttcaagtatc tgttgaacct aagtcaaaag ctgaccgtga
taagatggat
444601 gttgctttac aaaagcttac tgaagaagac ccaactttcc gtgctgaaac
caacccagaa
444661 actggtcaaa ctttgatttc aggtatgggt gaattacact tagacatcat
ggttgaacgt
444721 atgaagcgtg aatttaacgt tgaagcaact attggtgaac ctcaagttgc
ttaccgtgaa
444781 acctttacta aggaagctaa agcacaaggt aagttcgttc gtcaatctgg
tggtaaaggt
444841 caatatggtg atgtttggat cgactttact ccaaacgaag aaggaaaggg
ttacgaattc
444901 gaagacgcta tcgtcggtgg tgttgttcct cgtgaattta ttccttcagt
tgaccaaggt
444961 ttacaagaag ctatgaagaa tggtgttctt gcaggttacc cattaattga
tgttaaggct

445021 aagttgtacg atggtagtta ccacgaagtc gactcatcag aagctgcttt caaggttgct
445081 gcttctcttg ctttgagaaa tgctgctgct aaggctggcg ccgttattct tgaacctatc
445141 atgaaagttc aagtaaccac tccagaagaa tacttaggtg atgtaatggg atcaattaca
445201 gcacgtcgtg gttctatgga aggtatggaa gacagagcag gtgctaaagt aatcaactca
445261 tttgttccac tttcagaaat gtttggttac gcaactactt tgcgttcatc aactcaaggt
445321 cgtggtacgt ttacaatggt atttgaccac tactcaccaa ctcctaaatc tattcaagct
445381 gaaatcatca aaaaacgtgg tggagaagac gctgaataat ttataaatag agagtttgaa
445441 aaagagatat catttgatat ctcttttttt gtataaaaaa ataaaataaa tgctgagaaa
445501 gcttgatatt taaggaaaga caaagtacaa tagtaaacgt gcgaaaatat tggaggggta
445561 taccccgcgc acaaaaagca atgctttgac gcaaaaggtt gcggcacacc aggctgcatt
445621 gccacagagg tgagtcggta atttttgccg agctagtcat cttttaaaga agacgaagga
445681 ggttattaga tggcaagtca acaaattcgt attagattaa agtcatacga acatggtatt
445741 ctcgatgaat cagctgctaa aattgtagct accgctgaaa gaactggagc acaaatttct
445801 ggtcctgttc cactacctac ggaaagaact ttattcactg ttttacgttc accacacaag
445861 aacaaagatt cacgtgaaca atttgaaatc cgtacacata agcgtttaat tgatattttg
445921 aatccaacac ctaagactgt tgattcatta atgaagcttg atttaccaag cggcgttgat
445981 atcgagatta aattataatt taaaacatat atggaggtgt aatcatgacc aaaggaatct
446041 taggaagaaa ggtcggtatg actcaaatct tcactaaaaa cggtattttg gttcctgtaa
446101 ctgttattga agcaacccct aacgttgttt tacaagttaa aactaacgaa tcagacggtt
446161 acgaagcagt tcaagtaggt taccaagaca tgcgtgaagt attgagcaac aaacctgcca
446221 aaggtcatgc tgcaaaagca aagacttcac ctaagcgctt cattcgggaa atccgcgatg
446281 tcgagcttaa ggattacgaa gtcggctcag aaatcacggt ggactcattt agtgaaggtg
446341 acgtagttga cgttactgga actacaagag gtcatggtac tcaaggtaac atcaagcgtt
446401 ggggccaatc aagaggtcca gaaacccacg gttctagata ccacagaatc ccaggttcaa
446461 tgggttcaat cattaaccgt gtacctaagg gtaagaagtt acctggtcac atgggtggca
446521 agaaagttac cgtacaaaac ttagtaattg aaaaagttgt gcctgaaaag aacgtacttt
446581 taatcaaggg taatgttcca ggtgcaaaga actcattaat ttttgtaaaa tctgctgcta

446641 aagctgctaa ataggaagga ggactataat ggctaattta gaaattatcg
atcaaaaagg
446701 taagtctgca ggtaatgtag atttaaatga agaaatcttc ggtattgaac
ctaatgaaag
446761 tgttgttttc gatgcaatca ttagacaaag agctggtaag cgtcaaggca
cttctgctgt
446821 aaagaacaga tcagctgttc gtggcggtgg taagaaacct tggagacaaa
aaggtactgg
446881 tcgtgctcgt caaggttcta ttagagctcc acaatggcgt ggtggtggta
ctgtatttgg
446941 tccaactcct cgttcataca agatggacat gcctcgtaaa gcacgtcgtt
tagctatgaa
447001 gtcagttctt tcccaaaaag ttgctgacaa agatttaatc attcttgatc
aattaacttt
447061 agaagcacct aagactaaag aattaaaggc tatcttagat aatgctaatg
tttctggtaa
447121 ggttttagtt gtgtctgatg ataagaatgt tcaattatca ggtaaaaacc
ttccaaaagt
447181 gaaagttgta cctgttaatg gcttaaatgt tgttgatgcg gttgactacc
aaaaacttgt
447241 attaactcaa gacgctatta agagaattga ggaggttttg gcataatgga
tgcacgtgat
447301 atcatcttac ggcctgtagt taccgaaaaa tccatgaatt taatggatga
taagaagtat
447361 acttttgatg tgcttgtatc agctaccaag actcaagttc gtaatgctgt
tgaagaaatt
447421 tttgatgtaa aagttaagaa tgttaacatt atgaacgttc gcggtaaaga
aaagagagtt
447481 ggtcgttaca ctggtaagac tgctcgtcgt agaaaagcaa tcgttacttt
aactgaagat
447541 tctaatgata ttaagatctt caatgataat aaagaaaatt agtaaatagg
aggtcagtca
447601 attggcaatt atcaaatata agccaactac aaacggcaga cgtaatatga
cttcttccga
447661 ctttgctgaa attaccaaga aaaagcctga aaagacttta cttgaatctc
aaagtcatac
447721 tgctggtcgt aactcatacg gtcatattac tgttagacac cgtggtggtg
gtcacaaaca
447781 aaaataccgt atcattgact tcaagcgtaa taaggatgat gtaaaagcag
ttgttaaggc
447841 tattgaatat gatccaaaca gaactgctaa catcgctctt cttcactaca
ctgacggtat
447901 taaagcttac atcttagcac ctaaaggtct aaaagttggt actgttgttg
aatctggtcc
447961 aaatgctgat attaagcctg gtaatgcatt gccattatct gctattccag
ctggtactga
448021 aattcacaat attgaattga agcctggtaa aggtggtcaa ttagtaagaa
gtgcaggtac
448081 tgttgcacaa gttcttggta atgatggtaa gtacacttta gttagacttc
aaagtggcga
448141 agttcgtaaa atcttatcaa cttgccgtgc tactatcggt tcagttggta
atgaacaaca
448201 ctcattaatt caattaggta aagctggtcg taagcgttgg ttgggcaagc
gtccacaatc

448261 tcgtggttcc gtaatgaacc ctaacgatca cccacatggt ggtggtgaag gtaaggctcc
448321 agttggtcgc ccacaaccta tgactccatg gggtaagaag tctcgtggta ttaagactag
448381 aaactctaag gctagaagtg aaaaacttat tattcgtcac cgtaaaggta acaagtaatt
448441 aaacgaagga ggttaattaa tgagccgtag tattaaaaaa ggtccttttg ctgatgcaag
448501 ccttttaaag aagatcgaag cccaagaagg ttccgaaaag aagcaagtaa ttaaaacatg
448561 gtctcgtcgt tcaactattt tcccttcctt tgttggtttc acaatagctg tttacgatgg
448621 aagaaaacat gtgccagtat acattactga agatatggtt ggtcataagt taggtgaatt
448681 cgtacctact agaactttcc gcggtcacaa ggtcgctgat aaggccacta ctacagtagg
448741 taaataatag gaaggagaga catctaaatg gcagaacaaa ttagttcagc taaagctgaa
448801 gcaagaacag ttcgcatcgc tcctagaaaa gcccgtttgg tagtagacct tattcgtgga
448861 aagagtgttg ctgaagctct cgcaatttta caatttacgc caagagctgc ttccccaatc
448921 gttgaaaaag tgttgaagtc agctattgca aatgctgaac acaactacga tcttgaaagt
448981 gcaaatcttt acgtatcaga agcatacgtt aacgaaggtg caactttaaa gagatttcgt
449041 ccacgtgcta agggtatggc ttctccaatt aacaagagaa caagccacgt agtagttgta
449101 gtatctgaga agaacgatta agggaggtaa atttaatggg tcaaaagatt aacccaaatg
449161 gtttccgtct cggtgttaac cgtgattggg aagcaaagtg gtatgcagac aaaaactacg
449221 ctgacacttt aaatgaagat ttacgtatta gaaagttcat ctctgaaaag ttagctgatg
449281 catctgtttc cactgtggaa attgaacgtg ctgcaaacag aattaacatt tctatccaca
449341 ctgcaaaacc tggtatggtt atcggtaaag gtggtaaaga agttgaagct ttaaggaaac
449401 aacttagtgc tttaacaaaa aagaacgttc acattaatat tgttgaaatt aagaagcctg
449461 atttagatgc taaattagta ggcgaaggta ttgctcgtca acttgaagca agaattgtat
449521 ttagacgtgc aactcgtcaa gcaactcaaa gatctatgcg ttctggtgct aagggtatca
449581 aagttcaaac tgctggtcgt ttgaatggtg ctgatatggc aagaagagaa tggcatactg
449641 aaggtagtgt tccacttcat actttaagag cagatattga ttatgcttgg gtagaagctg
449701 caactactta tggtcaaatt ggtgttaagg tttggatcaa tcgtggtgaa attttaccac
449761 aacgtaaaaa caaaccttct aagaaagcga agggaggaaa ctaatagtgt tagtacctaa
449821 gcgtgtaaag caccgtcgtg aattccgcgg taaaatgcgt ggtgaagcta aaggcggaaa

449881 gaccattgca tttggtgaat atggtttaga agctgttgaa tctcactgga ttactaatag
449941 acaaatcgaa gccgctcgta ttgctatgac tcgtttcatg aagcgtggcg gacgtgtttg
450001 gattagaatc ttcccacaaa aatcctacac tgcaaaaggt gttggtgttc gtatgggttc
450061 cggtaaaggt gcaccagctg gttgggtagc tgttgttaaa agaggcaaga ttatgtttga
450121 aatcggtggc gtttcagaag acgttgctcg tgaagcttta agacttgctt caaacaagct
450181 tccaattaag actaagtttg ttaagaagag ttcggaagta ggtggcgaat ctaatgaagg
450241 ctaaagatat cagagcatta accactgatc aaatgttaga aaaggaaaag caatataaag
450301 aagaactttt taatttgcgt ttccaacaag caacgggtca attagagaac accgctcgct
450361 tgagacaagt ccgcaagaac attgctagaa ttaagacaat tcttagcgaa aaagaattga
450421 gcaagaatta gttaacggaa gggagttatt aacttgagcg aaactaacga aagaaataat
450481 cgtcacgtat accaaggtcg agttgtttct gacaagatgg ataagactat tacagttgtt
450541 gtagatacct acaagaacca ccctgtttat aagaagcgta ttaaatactc aaagaagtat
450601 tacgctcacg atgaaaacaa cgaagctaag attggcgata ctgttcgtat catggaaacc
450661 cgtccattat cacatgcgaa gcgttaccgt ctaactaaga ttgttaagaa gtcaatttaa
450721 ttacgcggat tttattgagg ggaggataaa ctagtgatcc aacacgaaag ccgtttaaag
450781 gttgctgaca actccggtgc aagagaactc ctagttatca aggttttagg tggttctaag
450841 cgtaagaccg gtaatattgg tgatatcgta gttgctgctg ttaaacaagc aacaccaggt
450901 ggcgttgtca aaaaaggtga tgttgttaaa gcagttattg ttagaacaaa atcaggtgca
450961 cgccgtgaag atggttcata catcaagttc gacgaaaatg cagcagttgt aattaatgct
451021 gataagagtc ctcgtggaac tcgtattttt gggcctgtag cccgtgaact gcgtgagcac
451081 gatttcatga agatcgtatc tcttgctcct gaagtcttat aattttttag ggaggtgcac
451141 tgatgtttgt taaaacaggt gacaaagtaa aagttattgc cggtaaagat aaaggcaaag
451201 aaggcactgt actttcagtc aacgccaaga ctaaccgtat tgttgtcaaa ggtgttaata
451261 agatcaaaaa gcatgagaaa ccttcacaag ccaacgctaa tggtggtgtg gtagaaaaag
451321 aaggttctat ccatgcatca aatgtaaaag ttattgctaa aaaagaagat aacaacaaat
451381 aggagggagg acgcacatgg caaacagttt agtagaaaaa tattcaaatg aaatcgcacc
451441 agctatgaac aaaaagttta attacgattc agttatggaa attcctaaaa ttgataaaat

451501 cgttttaaac atgggtgtcg gtgatgcagt ttctaatgca aagaatcttg atgaagctgt
451561 agaagaattg actttaatct caggtcaaaa gcctttgatt actaaagcta agaaatcaat
451621 cgcaaacttc cgtttacgtg aaggtatgtc tattggtgct aaggttaccc ttagaggcga
451681 tagaatgtac gatttcttgt acaaattaat caacgtttct cttcctcgag ttcgtgactt
451741 ccgcggaatc agttctagat catttgatgg tcgtggtaac tacactttag gtatcaaaga
451801 acaattgatt ttcccagaaa tcgactacga caaggtaaac cgtgttagag gtttggacgt
451861 tgttattgta actactgcca atacagatga agaagctcgt gaacttctta ctgagtttgg
451921 tatgcctttt gctaaataat ttggaggaca ttaatggcta aaacatcaca aatcgtcaga
451981 aatcatcgtc ctgctaagtt ttcatctcgt gaatacactc gttgcgagag atgtggccgt
452041 ccacactctg tttaccgcaa gttcaaatta tgccgtattt gcttaaaaga cttagcacac
452101 aagggtcaaa ttcctggtgt taaaaaggca agttggtaat taacggtaaa ggaggcaaat
452161 taaatggtca tgacagatcc aattgcagat tacttgacta gaattagaaa tgctaacatg
452221 gcaaaacaca gttctgttga gattcctgca tcatcaatga agaaatcact tagtgaaatc
452281 ttaaagaacg aaggctttat tcgcgattac caagttgaag atgacaacaa acaaggtatg
452341 atcaagatct tcttgaaata cggtcctaat aatgaacgtg ttatttcagg tttaaagcgt
452401 atttctaagc ctggtttaag aaactacgta agtgcagaaa acttaccaaa agttcttaat
452461 ggtcttggta ttgctatcat ttctacttct gcaggtgtga ttactgataa agaagctaga
452521 gaaaagaacg tcggcggcga agttattgct tacgtttggt aattttgaca gaaaggagaa
452581 ctattaatga gccgaattgg tttaaaggtc atcgaggttc ctgaaaaggt cacagttacc
452641 aaaaatggtg acgacatcac tgttaaggga ccaaaaggtg aacttactag atactttgat
452701 ccacgcatta cctttgaaca aaaagatgga gaaattcatt ttagtcgttc aaatgaagct
452761 gataaagctc ttcatggtac tgaaagagca aatcttgctt ccatgattga aggtgtaact
452821 gatggatatg ttaagaagtt aactttagtt ggtgttggat accgtgcagt tgcacaaggt
452881 aagaaattaa ctttaaatgt tggttactct cacccagttg tatttgaagc accagaaggt
452941 gttacagtta aaactccatc agcaacttca attgaaattg aaggtatttc aaaacaagtt
453001 gtaggtcaat ttgcggcaga aattcgtgat gttcgtccac cagaacctta caagggtaaa
453061 ggtattcgtt acgaagacga atatgtacgt cgcaaggaag gtaagactgg taaataataa

453121 atagaaaatt tttgaggtga aattgtgatt tctaaaccag ataaaaacaa attacgctta
453181 aagcgtcata aacgtattcg tggaaagatt tctggtactg ctgagcgccc acgcttaagt
453241 gttttccgtt caaacaaaaa catctacgct caattaattg atgatgtaga gggtgtaacg
453301 cttgcaagtg cctcaacaaa tgataaaaat atttcagctg aaggttctaa gatggaacaa
453361 gctgctgaag taggtaaagc tttagctgaa actgctgcta agaagaacat taagagtgtt
453421 gtatttgata gaagtggtta cttataccac ggccgtgtac aagctcttgc tgatgcagca
453481 cgtgaaaacg gattagaatt ctaggaaagg agaattaact aatggcaaac cgcaacgatt
453541 ctcgtagaga ttctcgtaaa gatcgtaaaa aagacgatat tgaagatcaa ttagtagcaa
453601 ttaaccggat caccaaggtt gttaagggtg gtcgtcgcat gagatttgct gctgttgtaa
453661 tcgtcggcga tagaaaaggt catgtaggtt ttggtactgg taaggctcaa gaagtcccag
453721 aagctatccg caaggctgtt gaagctggta aaaagagaat gattaaggta cctactgttg
453781 gtactactat tccacatgaa gttatgggcc attacggttc tggtaacatt atgttgaaac
453841 ctgctgaagc tggttctggt gttgctgctg gtggtgctgt tcgtattatc atggatttag
453901 ctggtatttc agatgttact tctaaatcac ttggttcaaa tacaccaatc aatgttatcc
453961 gtgctactat ggacggtttg agtaagttaa agactcgtga agacgttttg aaacttcgtg
454021 agtcagcaaa aagcttagaa gattaaggag attaaataat ggatttaaaa gttaccttaa
454081 ttaaaagcgt cgcacaccgt cttccaaaac aaagaaagat tgttaaagct cttggtcttg
454141 gtaaggtaaa tagcactgtt gttcttccag acaacgctgc aactcgtggc gctttattga
454201 agattgctca cctgatctca gttgaagagg ttaataagta attagaatcc aaggaggtgc
454261 caattaatga agcttaatga attaaaacca aatgaaggtt cacgtcgcaa cagaaagcgt
454321 gttggtcgtg gtacttctag tggttacggt aaaactgctg gtcgtggtca aaagggtcaa
454381 ttagctcgta ctggcggtaa aacacgttta ggtttcgaag gtggtcaaat gccattattc
454441 agaagaatgc ctaagcgtgg tttcaagaac gttaaccgca aagaatacgc tattattaat
454501 ttaaatgatt taaatagatt tgatgatggt agcgaagtaa ctatcgatac attaaaatca
454561 tcaggtttag ttaaaaaaga acttgcagga gttaagttgt tagctaacgg tgaattaaag
454621 gttaagttga ctgtaaaagt taataaagcc tcagaagctg ctaaaaaagc tgttgaagct
454681 gctggcggaa ctgttgaggt gatctaatgt tctcgacctt gaagaacgcc tttaaggata

454741 aagatattag atctaaaatc ttttttacac tctttatcct gttgctttat
cgaatcggag
454801 ctaatataac agttccggga gttaatgcaa aagctattac acgggttgca
caaactggtt
454861 tagtcccaat gttggataca gtcagtggtg gtggattaga tacatattca
atcttttctc
454921 taggtgtttc accttatatt actgctcaaa ttgttattca attactccaa
atggatattg
454981 ttcctaagtt agttgagtgg ggaaagcaag gagaagttgg aagacgaaag
acaaaccaag
455041 taacacgcta tcttacttta gtagttgctt ttgttcaaag tcttggaatt
actcttggct
455101 ttaacgtttt aaccgaaatg ggtttagtta aaacgcaaac tcctcaaacc
tatattgaga
455161 ttgccattat tatgacggct gggactatgc ttttgacctg gctgggtgat
gaaattactg
455221 ataaaggttt aggaaatggt gtatctgtaa ttatttttgc aggtattatt
gcccgtcttc
455281 ctaatggaat ttatcaacta tttaaagatt tcattattaa taatagtgct
agcgatcgtt
455341 ggcaggggat tttgttcttc atcgcgatca ttattgccat tttactagta
actcaatttg
455401 ttacatggtt tcaacaggct gatttgcgag tacctatcca gtatactcgt
agggcagcaa
455461 caagtggctc cgaaagtttc ctacctttaa aggttaacgt ctctggagtt
attccagtta
455521 tttttgccag ttcctttatt attacaccag ctacaatttt gatggctttt
caaagatctc
455581 atggaaatga acaatggttt aagatttgta atcaaatatt tagtttacaa
actacaccag
455641 gtgcattaat ttatacactt ttgattattt tgttcacttt cttctatgcc
tttgttcaag
455701 taaatccaga aaagttggct gagaacttgc aaaagcaagg agcctacatt
cctagcgttt
455761 ggccaggaaa agatacacaa aagtatgtat ctaaaatttt aattagatta
tcaacagtag
455821 gtgcggtatt tttaggactt gttgccttgc tgccgcagct tgctacaaat
atttttggtt
455881 taccaagttc aattggactt ggcggaacaa gtcttttaat cgttattggt
gttgttttag
455941 agttagcccg tcaagttgat ggactactaa tgaagcgcga atacgttgga
tttattagat
456001 agtaaggata aaaatgatta atttaattct tttaggattg cctggtgcag
gcaaaggaac
456061 agcttctgaa agcattgtag ataaatatca cttagcacat atttctacag
gtgatatgtt
456121 cagagaagct atggctaata aaactccagt tggtttagaa gctaaaagtt
atattgataa
456181 aggcgattta gtgccagacg aagttactgc taagttagtt gaagaacgtc
ttaagcaacc
456241 tgacactaag aatggattca tcttagatgg atttccaaga accactgttc
aagctgaact
456301 tcttgaaggt ataactaaac ggttagaaaa gccgttaact aatgtaattg
caattgatgt

456361 tgatgaagat gttttaatca agcgcttatc agcacgctac atttgtaaaa attgtggtgc
456421 aacttacaac aagatttcaa atccaactaa agtagaaggt acttgtgatc gttgcggagg
456481 acatgaattt ttccaacgcg aggatgacaa gccagaagtt gttaaaaatc gcttagaagt
456541 taacaagaag atgaatactc ctcttcgtga tttctatgaa gaaaaaggaa ttctttcaac
456601 tgttaacggt gaacaaactc cggaaaaagt atttgaagac attgacaaaa tcttaagtaa
456661 agattagtct cttattgttt tttttacatt ccgtgttata atgcctaagt gtgactattt
456721 gttcatgtgg aggaacaatt ttggcaaaag aagatgtcat cgaagttgaa ggtaaggtag
456781 ttgatacctt acctaatgct atgtttaaag ttgaattaga aaatggtgca acaattttag
456841 ctcacgtttc tggtaaaatt agaatgcact acattagaat tttacctggt gatagagtaa
456901 cagttgaatt atcaccatat gatttaacca agggtaggat tacgtatcgt tttattaagt
456961 aattacggag gcaaacatga aagttagacc atctgttaaa ccaatgtgtg aacattgcaa
457021 gattatcaaa agacatggtc gtgttatggt gatttgctct gcaaacccta agcacaaaca
457081 acgtcaaggt taatagttat atatttttat taggaggtgc aatttaatgg cacgtattgc
457141 tggtgttgac ttacccagaa ataaaagagt tgtagtcgca ttaacttata tctatggtat
457201 tggtgaatca actgcaaaga agatttgtaa agatgctggt atttctgaag atattcgtac
457261 taatgacttg accccagaag atcaagaaaa attacgtagc gaagtagata agtaccgtgt
457321 tgaaggtgac cttcgtcgtg aagttagtct taacattaag cgtttagttg aaattggttc
457381 ataccgtggt attcgtcacc gtcgtggatt gccagttcgt ggacaaaata ccaagaataa
457441 tgctcgtact cgtaagggtt caaagagaaa ataatttatt aaataggagg ttaagttaat
457501 ggctgcaaag aaaacagcac gtaaacgccg tgtaaagaag catgttgaaa gcggcgttgc
457561 tcatattcat tctacgttta acaatacctt ggtcatgatt actgatgtac aaggtaacgc
457621 agtcgcatgg tcttccgctg gtgctttagg tttcaagggt agtcgtaagt ctacaccatt
457681 tgctgctcaa atggccgcag aagcagctgc aaagtctgca atggatcaag gcatgaagca
457741 tgttgaagtt tcagttaaag gaccaggtgc aggtcgtgaa gctgctatta gagcacttca
457801 agctgcaggt cttgaaatca ctgctattcg cgacgttact ccagtgccgc acaacggttc
457861 cagaccacca aaacgtcgtc gtgtctagtt tattcctgcg tgcaggacat tacgttttga
457921 aaggggccca gtaatgattg aatttgaaaa accaaatatt accgtagtag accaagagga

457981 ggcatacggt aagtttgttg tcgaaccact ggaacgcggt ttcgggacta ctttaggtaa
458041 ctcactccgt cgagttttac ttacttctat tccaggtacg gcactttctt atattcagat
458101 tgatggtgtg ttacatgaat tttcaacaat tcctggcgtt agagaagacg tcacaaaaat
458161 tattcttaat ttgaagaaac tagagttaaa gtcactctca gatgaagaaa aaattgccga
458221 aatcgatgta actggtccag caatcgtaac tgctgctgac ttaaaggtcg actctgatat
458281 tgaggtctta aatccagatc aatatatttg tagtattgct gatggtggcc atttgcatat
458341 gaacgttgcg atcaagaatg gtcgtggtta tgttcccgca agtgaaaaca agactgatga
458401 catgcctatt ggcgtcatcc ccgttgattc actcttttca ccaatcaaaa aagttaacta
458461 ccaagttgaa agtgctcgtg ttggtaaaag agacgattat gacaaattaa ctttagaaat
458521 ttggactgat ggttcaatca cacctaatga tgcccttagt ttcgctgcga agattcttgt
458581 agaacacttc aaagtattta tgtctactga tatggatgcg cagtttgatg atgtaatggt
458641 agaaaaagag gacgataaga atgagaaaaa gcttgagatg acgatcgaag agcttgactt
458701 atctgttcgt tcatataact gcttgaaacg tgcaggaatt aatactgttc aagaattaac
458761 tgataaatct gaagcagata tgatgcgcgt acgtaactta ggacgtaagt cattagaaga
458821 agtaaagaat aaacttgccg aacttggtct atcacttcgt caagatgact aagttggaaa
458881 aataaggagg gaaaactcat ggcataccgt aaattaggtc gcgatagtgc acatagaaaa
458941 gcaatgctta gagaaatgac tacccaattg atcatgaacg aacgcattgt tactactgaa
459001 acccgtgcta aagaaatccg taaaactacc gaaaagatga ttactttggg taaacgcggt
459061 gatttaagtg ctagaagaaa ggctgctgct tttgttcgta atgaaattgc tgatattcat
459121 gaagaaaaag atgcagttgt tgtaaaatca gcacttcaaa aacttttcag tgacattgca
459181 cctcgttaca aagaccgtaa tggtggttac actagaatgt acaagttagc taacccacgt
459241 aagggtgatg ctgctccaat ggtaatcatt gaattagttt aattgcgcaa aatataatat
459301 tattttataa atctttcaca aaagcgagaa taagcgtgat gatggtggct tttgcctagt
459361 ctagcttaga tgttaaatca tccctctttg tggatgattt ttttttgctc tttttttata
459421 attgatacaa tgacatattt gaaaaagagc aagtgatatt tatgaaagac aatatagtaa
459481 ccgtcgagca tctttctttt acttataaag acagtgagga acctgcagta aaagatgtta
459541 gtttttcaat tcctaaaggt acttggacca ctttggttgg tcataacgga agtggaaaat

459601 caactattgc tcgtttattg aatggtattc ttttaccaga tgataatcct aatactttga
459661 tcaatatcga tggtataact ctaactgaaa aaactatgtg ggatattaga gatcgtgtgg
459721 gaattgtatt tcaaaatcct gataatcagt ttgtgggagc tacggttgaa gatgatgtag
459781 cttttggttt agagaaccgt caagtccctc gtaccaaaat gaaaagcatt gttcaggatg
459841 tacttaatca agtagaaatg atagcttttc aagaaagtga accccaatat ctttctggtg
459901 gtcaaaagca aagagttgct attgcaggaa ttttagcaat tggtccgaaa ttgataattc
459961 tagatgaatc aacgagtatg cttgacccag caggaaagtc aaaaattctt aggcttataa
460021 aaaaattaca aaaagaaaat gagctaacaa tattttcaat tacgcatgat attaatgaag
460081 cggtgcacgc agaccagatt ttagttttaa ataaaggcag cttgctggct agcggttcgc
460141 ctggagatat atttaaagat gattctctta ttagaagcgc ggggttagat cttcctttat
460201 tttataaaat aaaaaataag ctaataaatg aaggaatagg tatcccgcaa gaagtcaata
460261 ctgaggaaaa gttggtgaac tatttgtgtc aattgaattc aaaaatgtag attatattta
460321 tgcgccagaa actccatttc agactcaagg attagctgat atatctttta aaataaaaag
460381 tggctcattt gttgcaattg ctggtcatac gggaagtggt aaatctacct taatgcagca
460441 ttttgatggc ttattattgc caagtagtgg agcagttaat attgatgata agactataac
460501 ttctaacact tctagcaagt ctttaaaaga aattagaaaa aaagttggtt tagtattcca
460561 gtttcctgaa agccaattgt ttgaagaaac agttcttaaa gatgtcatgt ttggaccgct
460621 taattttggc ttttcggagc aaaaggctaa agaacaagct attgagtgga tagaaaaagt
460681 gggtttatct gagcaagtta tgaataaatc tcccttcgaa ctttctggtg gacagatgcg
460741 ccgagtagca attgctggcg taatggctta tgaaccagac attttatgtt tggatgaacc
460801 agctgctggc ttagatcctg aaggacagaa gcaaatgttt gatatattta aaaattatca
460861 acaggaagga catactgtca ttttgatctc gcataatatg gatgatatca gtcaatatgc
460921 agatgatatg ctggttcttg aacatggtcg tttaataaag catgcaagtc caaaggaagt
460981 tttttctgat cctaactggg taaaggagca ttttttagat gagcctgcta ctagtaaatt
461041 tgctagagaa ttagaaaagg gtggctttca attttcagaa atgcctttaa ctattgattc
461101 tttagtaaat aaaattgcga atgaattaaa ggcgaaagga ggaaatgaat gagtaaaatt
461161 attatcggtc gatatattcc tgggaattct gtcgtctata agatggatcc aagaggggaa

461221 ataatcgcta ctttttgtt tattgtcatt atcttcctag ctaataactg
gttatcctat
461281 ctctttctat cttttttac tttacttgct gttggagcaa ctaaattaaa
accaaaagta
461341 ttttgggatg gagtgaagcc tttaatttgg ctgatccttt ttacatcagt
tttacagctg
461401 tttttacta ctggtggaaa aatctattgg cattggggaa tttttaatgt
tagtgaatac
461461 ggcattcaaa actctatttt tatttttatt cgttttgtaa tgattatttt
aatttctacc
461521 gttctcactc tgacaacaac ttcattagaa attgcagatg gaatggaatg
gattctaaga
461581 cctcttggct acttaaaagt tcctgtagcc cagattgcac ttgtaatgtc
gatagcactc
461641 cgttttgtac cgactttgtt agatcaggct gttagaatta tgaacgctca
gagggcacgt
461701 ggagcagatt ttaatagtgg tggattaatt aaaagaattc atgcaattat
tccaatttta
461761 attcctttat tcattagttc cttaacaatt gcaattgatt tagctactgc
aatggaagct
461821 agaggctatc gagaagggac aaaaaggacg cgctatcgtg ttttaaaatg
gtcaaagtat
461881 gattggatta atctgggata ttttgccctc ttgacgctga ttttattact
tacaaggaca
461941 tattaaataa tgactacgag atataagttg acaatggcat atgatggaca
ccttttcat
462001 ggctttcaaa ttcagcctaa ggagcggact gtgcaaggcg taattgaaga
agcactaaaa
462061 aagatgacca aaggaaaacg ggtagtggtg catggatctg gaagaactga
tgctggtgtt
462121 catgctaaag gtcaagttat ccactttgac tatccaggaa aagaaattcc
agctaaaaat
462181 atgatgcttg ctctaaatgc attaatgcca attgatacag ttttttttga
tagtgaaatt
462241 gttaattcag attttcatgt gcaatattct actaaaggaa aatggtatcg
ctatattgta
462301 gatcaacatc gttttactga tccttttaac cgtttttata cgggacatta
tccatatccg
462361 ttagatattg atttgatgca gaaggctgct aaagacttga tcggaaaaca
tgattttact
462421 agttttgcag caagtggtgg acaaattgtt gataaggtac gaacaattta
ttacgtaaat
462481 atcacttctg atccagataa taaaaaaatt atttttgatt ttattggaaa
tggattttta
462541 tataacatgg ttagaatttt agttggtact ttactggaga ttggtaataa
aaaacgtcca
462601 gttgatgata taccgagagt tttagcagca aaagatcgac aagaagttcg
cacaacagca
462661 ccagcaagtg gattatattt gcagcatgtt ttttatgaag aagttcctaa
aaaatatcga
462721 ttagacctta aaaaagattg actttttgag caaaacgacg taaattagta
tacgtatgtt
462781 tgtccacgat aggccccgga aacttattgt ttgtcaaaca gaagaaaaac
ggaggaattt

462841 aaagtgcgta ctaccccatt agctaaaact agtgatatta aacgtaagtg
gtatgttatc
462901 gatgcgactg atgtttcact tggtcgttta tcaactgctg tagcatcaat
tttaagaggt
462961 aagaataagc ctcaatatac accaaatgta gatactggtg attatgttat
tgttgttaac
463021 gcaggtaaat taaagttgac tggtaaaaag gcaactgata agatctacta
ccaccactca
463081 gattaccgtg gtggtttaag agctactcct gccggtgaat tattagctaa
gaacccagta
463141 agattagttg aattatctgt aaagggcatg ttgccaaaga atactcttgg
tcaccaagaa
463201 ttcatgaaga tgcatgttta cgctggtgaa gaccacgaac acgcagcaca
aaagcctgaa
463261 aaattagaca tcaacgaatt aatctaggag gttttaaata atggcacaac
aagcagctta
463321 cgctggaact ggtcgtcgta aagattctgt tgcccgcgta cgtttagtac
caggtaacgg
463381 tcaaattacc attaacaaag aagacgtaac taagtacatt ccatacccta
acttggttca
463441 agatatgaag caaccattag cattaactga aactgaaggt caatacaacg
ttgaagttaa
463501 tgttaacggt ggtggtttct caggacaagc tggtgcaatt cgtttaggta
ttgcacgtgc
463561 acttcttgaa gttgacccag atttccgtgg tccacttaag aaggctggca
tgttaactcg
463621 tgacccaaga atgaaggaaa gaaagaagcc aggtttgaag aaagcccgca
aagcttcaca
463681 attctcaaag cgttaatatt tatattacgt ttttatcgaa aagacatctc
gaaagaggtg
463741 tctttttgt tttgctgtga aaaacattgt ggaaaaatcg gggggaata
tacttaaggt
463801 gagagagtaa gcaagactag gaagtgctaa ccatgacaga tttattgact
actaataact
463861 tacgaaaagt ttttaaagat caagttgtac ttgagggtat tgatttacat
gtgcctagaa
463921 atcaagtcta ttgtctttta ggacctaatg gagctggcaa aactactttg
atgaagatct
463981 taaccggaat gatttctaaa accagtggtc aagttatttt tgataatcat
gagtggagtc
464041 gaaaggattt gaaaaatatc ggttccttaa ttgaaaatgc accattatat
gggaatttaa
464101 cggctcaaga aaatctaaaa gtaattacaa aattacgtgg ggtagatgat
tctgttatta
464161 gtaatattct taatactatt ggtttaacta atactggcca taaactgtct
aaagatttct
464221 cattggggat gaaagaaaga ttaggaattg gtttagcatt agtaggtgag
ccaaaattat
464281 taattttaga tgagccaact aatgggctag atccagtagg aattcaacaa
cttcgaaact
464341 taattgcaga atttaaaaat aaagatatta caattattat ttctagtcat
atgctttctg
464401 aagtagaaca tttggctgat actatcggta ttattggtgg tggaaagctt
ttactagaaa

464461 agccatatga ccatcaagaa aatttagaaa aactgtttaa tgacactctt gagaaagcag
464521 gtgtcttaaa tgtttaacta ctgttcaatt gaaatattaa aaactaaacg cactattatt
464581 agaaaattag tctggatttt tccgctttta gttagtatca ttactttttt atttttttgca
464641 tctactggat acgtagttca aagtattatt aatcaatgga gctttatttg ggttaattta
464701 tttttagctc tagaaatagg attaattgat cgtcatgaaa gaaatagtac agattataaa
464761 atgattttaa gctcccccga tgatttgttt cagtatgaac ttggtagaat cttacatgat
464821 gttctattgt ctttaattac aacttgtacc ttaggcattc tcgttatatt gttaagtttt
464881 ttgatgccaa tgaaagtatc tcttggagct tgtttacttg cactctttgg aatatttgca
464941 gcaactttgt gggagattcc attgtattct tggttaagta gagtaacaaa tttatatgtt
465001 tctgttgggc ttgcttttgc tggcagttta attggaattt gggtagataa tttatcattg
465061 ggtaagctat ttccatttac ttggtcagcg ataatgcctg tttcgttgat tcaaatgcat
465121 gttaacggac tgttagtaga agatacagat aaaattccga ataatagttg gacattatta
465181 gcttccctga tactttttat tgtattgagt ggtttaagtg cagccgcgtt taaaaagcag
465241 gtggttaaaa atgcttaaac aagtagttga agctgagaat attaaaatac ggcgtagtct
465301 attgctatat ttgcatctta ttgttttatt tttatttcca gttttatttg gattatatta
465361 tggaagtcga aaacaagtga tcgtacctgc aaatatgatt attggttttt atgaaatatt
465421 agctattgcc tccccaatta taattagcgt agtcgtttgc ttagtttttg atcgagaaga
465481 gaaagcgggc aattttaaga attggttaac tgagccgata agtaaaggat ggatgatcca
465541 agctcagctt agctattatt ggatttggta tgtaattgag attatgggaa ctagtctaat
465601 tttcagtggt gtcttatttg gaatatatca aataaggttt tctctgatta agatagcctt
465661 aacaagtatt gcattttctt tatgcggaat agtgcaatat gaattaacgc aaataattgc
465721 attaaggtgg ggaattggtg atgcgcttat attagggttc tttggtaccg ttatttcctt
465781 attaagtatc acaagcttat tggattttgt ttggccaata ataccatggg cttggcaaat
465841 aagacttact actttttggc agactaatat tgatctaggc ttggtaaaat taagtctaat
465901 tgaatatctt attccactta tgttaacttg tttaattatt ttttgggggga aacgatattt
465961 tgataattgg cagggaaaaa actagtacaa aaactatact tttaaacttc gcttaagaaa
466021 ttgtgaaagt tttgaactat aataatatag acaggttatc ctaagaaagg ttctatattg

466081 tttacatgaa atatttaatt accggagcaa caggtaactt aggtgaaaag
gttactcgtt
466141 ggcttagaac tatgactccc gaaaataata ttcgagttgg aattcacaat
ttaaaaaaag
466201 cgaataagtt tgacgactta gatgttgaaa aggttaaact agattacttt
gatttagata
466261 cacttgaaaa agcagtttct ggcgttgatt tggttattta tattcctagt
attacttatg
466321 atttgcagag aagaatcaca gaatttgaaa atgtactgca agctgtgaaa
aaggcaaaag
466381 caaagttgat ttttgtcagc ttttttgcgg accaagaaaa taatcccttt
gtgatgtcac
466441 cattttatgc ttatgttcca aggcgactag ctagttccaa ggttgaatat
agtattgtta
466501 aaaatagtct ttatgctgat cctttagttc catatcttga agaattaatt
aagcgtaaga
466561 acattattta tcctgttggt gcacaaccac tttcttttat tactcgaaat
gatagtgcac
466621 atgcgattgc ctgcttagct atgcaggcta taatgcgtga ccaaggtcag
tcttatttac
466681 taagtatgga taaaaactat aatatggttg aattgtccta catcatgtcg
cgaattactg
466741 atcataaaat tggttatgca ccagtcacaa ttactgaatt tgctaaaatt
tatgcccaag
466801 aaggcgatgg aaaagaatta gcatcaatgt atgctggtgg tgcaaagggc
ctattaagcg
466861 caacatcaga cgattttcat cgcttaactg gacgtgaacc agaagagatg
gagcattttc
466921 tgcgaaacgg ctaccagata gctcgtttat aaaagaattc tatattaata
cataatattt
466981 caagttaaaa taaaagatgt tatactatag atagtaagtg ctgagagaaa
attaattgta
467041 attaaagtgt agatgcagag ctgagtaaga ccgtaggcat gctagtaata
cacagacttt
467101 tagcttaggg atgagggtaa aagtcaactt cttattttaa ctgttatata
cgaaagaaga
467161 gatcagacat taaaatctga tctctttttc gtgaagttta ttaattaaag
actagcatta
467221 atattcttag ccaagtagtc gtaaagcata cctttctcta agtcactggt
atcatgtccg
467281 agtgtccgag cgatagtgta agcatatgcc caagcagtag ctggaccacg
actagtgata
467341 attttatgtt cttggtcaac aacagtaatg tcagtgctaa agtgaccatt
tggacattct
467401 tcttcgattt gtttttcaaa gcctggataa caagtatagt tagcaccttc
aagtaaaccg
467461 tagtgcccta aagcgattgg agctgcacac attgcggcat cccacttacc
tgctttattt
467521 ctttgaatca tcaagtctgc aagcttttta ttatttctta aattcaaagc
accagttctg
467581 ccaccaggga aggcaacaag atcataatca agaagtgagt catccacaac
cttatcacag
467641 gtaagtaaga tatgatgatc accatcaatt tctttttat ctagtccgac
catgtcgcag

467701 tcaatattta aacgacgtaa gacatcaacg acacttaagc cttcaacttc
ttcacaaccg
467761 tctgcaaaga caacagctac ttttgtcata aaaaagtcct ccttttagt
aattactata
467821 cattcttatt ttaacgcaat gactttatct ttttttattt gaagattgaa
aaaaataact
467881 aaaaattgcg tatataaata tagagaggaa aatgaaagaa tgggtgagga
tcttgtagta
467941 gaaaatatgt gaaacactaa atatagtatt gatttttaaa atacttgaaa
taacgcagta
468001 ttctgttaac attgagataa ttgtaataaa atttgtgaaa caatttaagg
aaagagggta
468061 aaaaatggct actgaagttt atttagtaag acatggagaa acgatgttta
atcagttgaa
468121 taaagttcaa gggtgggctg attctccatt gactgtaaaa ggaattaatg
atttaaaagt
468181 aactgctgct aatttatctc aggttcactt tgataaaatg tatagttctg
acttgaagcg
468241 agccattgat acggttcact taattgcaga tacaaatgaa gtttcagaaa
ttggcaaaat
468301 aaaaaagctt ccagcctttc gcgaggtatt ttttggaact tttgaaggcg
acgatattga
468361 tgaaacttgg agaaaagttg cgaaagctgg tggcatgaaa ccaacgagtg
atgtggtaaa
468421 aattattcag acacttggta ttcatgattt tcgcgaagct actaaaaagg
ctgatccccg
468481 tcacttagct gaagacgccg aggcactaga tgatcgcatg gatcaagcaa
ttagtcaatt
468541 agcacaagaa actaaaggat taggaagagt attagtagta tctcatggtg
actttatcaa
468601 gactttaggt attaaatatt gggatcaagc aagtggtgac catgatattc
catttcctga
468661 taacggaagc gttactcgag gaattattga cgataatgga aaatttagaa
ttattaatta
468721 tggtgtaaaa aatactgata ttccaaattt ataagatgta actagtttgt
tgcatctttt
468781 ttagtatagg ttagagggat gttctttagc ggtatgttca acatgctcaa
tcattgcgtc
468841 gatcattgat aagacatcag gatcatttaa aaagtaaaaa acgtgtgtcc
catgcttatg
468901 atgtttaact agattgtact tttctaaaat tcctaattgt tttgacacga
cgggttgagc
468961 caattcaagt gtagttgcaa ttgttgaaac atcgacttct ttagaagaat
tgcgtaagaa
469021 aaataaaatt gaaattcgtc tgctattact caaaaccttg tatatcttag
ctgcttcgtt
469081 aactagggat gtgtttaaac tcattatgat caccaaacct tattgacaat
atatcgaaaa
469141 tggaatatta tactttctgc atataagtat accagattcg atatatttat
ggagaaaaaa
469201 catgaaagat aaatcatcat tacgctattt ttgggtaaca ctattaaata
ttattattac
469261 aattgctgaa tttattggtg gggcaatttc tggctcgtta gctttgctat
ctgatgctgt

469321 gcataactta agtgatgttg gttcgatagt tttagctttt gtagctaatt taatttctaa
469381 aagaaaaaga aatactagca aaacctttgg ttatgatcgg gccgaaattt tagcagcttt
469441 tactaatgga attattttaa ttgtaattag tatctattta tttattgaag ggattcaacg
469501 cttcagccat ccagaaccga ttagagggac aattatgttg attgtttctt taatcggtct
469561 agccgcaaat gtcatttcga tgtttgtagt tatgaaagag gctaagacta gtcttaatgc
469621 taaagctacc ttcttaaata tgctctctga tgcaattacg agtgtagcgg tagttttagg
469681 tgcaattgtt atttcgatct ggaaaattta ttggattgat ccagtattaa caatcgctgc
469741 ttcggtattt ttactcaaag aagcatatga agtaacaatt aaggcagcta atattttgat
469801 ggagacaaac ccaagtatcg atttgaacaa ggtaaatgag ttagtcttgt cttgtccaca
469861 tgtgaataat attcaccatg tccatgtctg gcaatattca gataatgtga caatgctaga
469921 tgctcacatt aatgtggata agaacttaga tgctgttcag ctagaaaaaa tctatacaga
469981 aattgcaaaa aagttaaaat ctcttggaat taatcatgta acattgcaag cagaatgcac
470041 gcgcggaatt gatgataaga tgatttttgc agataaagaa gattagttat tctggttttg
470101 ctgctatttg gtaaaatagg ggcaaagcct tttattttta gggagaaaaa aatgaagttc
470161 gatacagatg acttaaaagc aagcctaaaa aattatcaac aaaaagctag tgatcaattt
470221 aaagatttcg ctgaaaacga ttggcctgac attaaagatc atgtgacaga aaaaggacaa
470281 caagcgggcg actatttgaa gaaaaatggt ggaaaagttt atcaaaattc tgttaaagct
470341 tttaaaaaag gcgttagaga tcttgatcag tgggtaaacg gaactagcgt acataaggac
470401 gataaataat atgaaagtaa atatttgggt agcattaatt gtcttggtag ttggtttatg
470461 gaacttatat tatgcttata atcgttatca agaaaagaag ataactaaaa gaggtagtaa
470521 agcagacgct atttcttttg caattttagg gacaattttt acaattggcg gaattattat
470581 gctgtttatg cattaggatt aggtggtaag aattttggtt aagttggttt tagttcgaca
470641 cggcgagagt atagcaaatc gggacaatgt ttatactggc tggaatgatg tgccattgag
470701 taaaaaagga attgaacaag caaaagatgc ggggctcaaa gtagcaaaaa ttcctgactt
470761 tgtgccaact catattcata cttccgtttt gtcgcgtgca attatgactg caaatataat
470821 tgcggatgtt tgtagttttc tctatttacc aattactaaa acttggcgct taaatgaaag
470881 acactatgga gctttgagag gtattaataa agatgtttca aagaagatct ttggtaaaga

470941 acaagtatta aagtggagac gtgggtttga tagtgtacca ccgcttttaa ctcagcccgt
471001 tcaagaccgc agatatcaaa agtatgatat gcgcttaatg ccgcagggag aaagtttgca
471061 tcaaactcaa gaacgattga tgccatattt ttgggatcat attgcacctg aattaatgtc
471121 cggccatgat caattgattg tagctcatgg ctcaagcttg cgcgctctta ttaaaaaaat
471181 tgagggtatc agcaatgagg atattgtgaa ggttgaagtt ccaaatgcag aaccaattgt
471241 ttatactttt gattctgact tgcatattat taaaaaagaa attttacact aagcgagcag
471301 acttttggtc tgctcctttt ttaatagttt gtataggtgt ttttatggta aaatttttta
471361 cgagtatatt tgaacaaatt cttgtagagt aggagaaaat aatgcgtaaa atatttttat
471421 taagaggagc accaggatca ggtaaatctt ccttcattgc tcgtcaccac ttgcagcctt
471481 acgctatttc tcgtgatgaa attaggctgc tcttggcgga tttgacggtc tattatgaag
471541 aaagtactga tcatttgcac caagttattc ctcgtcatgt aactgtgcgt acagagcaaa
471601 tggttgataa tttggtccag cataaaatgg cctacggcga aactgtcatt gtcgatggca
471661 cacatatcac acctgataaa attgaacatt ttcgtccttg ggttgaaaaa tatcgctacg
471721 aactatttgt agttgattta atgcaaaata atagcttaga aagcttacta cgcagaaatc
471781 aaactagaat gcactatgac tgggttaaac ctgatgtaat taagatgatg tatgaacaat
471841 atgaagcaaa tccagaagta ccatcttggg cctattcgat tttacccaat gggatggaaa
471901 gagctttgag ccaaagggaa aagaacctag atcattattc acatgtggtt tgtataccag
471961 ataaggttaa gccagaagat tttccacatg ttcatatttc taatttttat ttttctttta
472021 atgatgaatt tactagaaaa tatggaactt atcgaaatgt tataacctta gctaagactc
472081 aggatgaggt aattgaacaa tttagacttc catattttgt atttaagttc caccacaagc
472141 atttcttaat ttctgcttat ccaattcgaa atgaaatgtt agatcctatt cgaaaagtaa
472201 aaggagtttg gtcttactcg actgggctat ataatgtagc tgattttgta aaagaatttc
472261 cagaaaatga acatcaacat gttcatcagt tcaatttaag taagattgac cctactcgct
472321 tgctacatat ttggtaaaaa gaaaacactt caaactgtat ttttaacagt tgaagtgttt
472381 tttgtttgtt ttaagagtta aaattaatag tttttagcta aagcgcccat tggatcccaa
472441 ggaagtaatt gaattggggc tttgcttcct tcatcaaatg ccttctttaa gtcgtcgcta
472501 aggtatttct tgttgataac tgcttggtaa acaaatttat caaaccattt atcactcatg

472561 acaaagtaac ctttaaatcc tggtttttcg ccccaagaat tttcaatctt ccacttagta
472621 ggtttgccat ctactaagtc aacaccagta ataaccatgg cgtgatccat catgctttcc
472681 cctgaatcaa gcatatcagc ttttgacata tcaaagttaa catcaaataa atcatcacgc
472741 ttgtataatt cggtgtctag taaaccggct tggcgttcag agtccttaac aacatttgaa
472801 ccaaaccaaa caacttcacc atttttaagt tgtttgataa ttaagtcctt catttcatca
472861 actggtaaat tcaagtgacg aacttgacga ccgccttcaa catttcctaa gtattcaaca
472921 gaaaatacct tatggaaagg cttatcttta gttggtgcat taattgtgga aatgtattcg
472981 ctcaagtccc agttaacata tttcttgaag aagtcttgtg gactcaagtc agcatctctg
473041 tggtaattac catcatcatc tttatattca aaatcaaatt gcttaggtgg aacaccaaga
473101 gaaattgcta aaatacggta aacttcgcga agcatttcat tcttgcgttc ttgaacttcc
473161 gcttcactct taccagcttg aaccatctta cgtaattcaa gaccatcttt tctaagtaaa
473221 gtattcaagg tatcgtttaa tgcactagag ttagttgcat tagctgtttc tgggtaaact
473281 ttcttaggaa caataccgta tttttcaata attccgcaaa gcatatccca ttgaccacca
473341 tcttgttgcg gagtagcaaa taggaaagca actttacgat cgcctaagtc cttgtcagca
473401 gtagcgatta cattttcaaa gaaccagttt gatttttcaa atttatccca gaagttagtg
473461 tagttttgag ataattcaaa atctttaatt ttgaagttct tttgaattga atgacgcata
473521 gtgtttaaag cactgaacat ccagcaacgg ccagattgtt tttggttagc aactttacca
473581 gtatcaattt cgattgaaaa agttggatct aaatcaatct tagtttgtaa attttgacta
473641 gccttgaaaa taccgttttc ttgagcagca cgagctgcaa ttttgttagc tttacttgag
473701 ttaaagtcat tttcaaagtc attaataaga tcgtttgtga tttccttact catagtcttc
473761 ctccttaatt aaaaaaagct taagtatatt ttaacttaaa ttaaagataa atgtctagtg
473821 attgagaaaa agaatattgg ttcataaaat ttattaaaaa aataaatgat tttttcgta
473881 tatataatca gggacatctt acgagaatta acgtaaaata taggtctagg aaaaagaggg
473941 gaaaataatg aaaaaacgtg gttttgaaat agtatctaaa tatcataatg caggcttgca
474001 cttgccgcaa agacaaacaa ttgccagtgc aggttatgat tttgaatgtg cgcaagatat
474061 agttttacca tcaatttggt gtatgaattt tgttagaatt ttcagattaa ttagaaatag
474121 ccatgagctg aatgaacgag accttgaatt agcagaaaaa gttttaaagc ctttttagt

474181 accaacggga attaaagcct atatgccaga agatgaagtt ttgattcttg ccaatcgctc
474241 gtcgaacact tataagcgta atgttacctt gccgaatggg attggagtaa ttgatagtga
474301 ctattataat aatgaaaaaa atgaaggtga gatttacttt cagatgatta attacggagt
474361 tcgtccacta aaaattaaga aaggcgaacg aatcggtcaa ggaattttta tgccttatct
474421 aaaagctgat aatgatgaac cagttcaaag acaacgtctt ggtggatttg gctcttctgg
474481 aacaaggaat gaagaataat ggcaaaagtt aaaacacgtt ataagtgccg taattgtggt
474541 tatatttcag cttcttattt aggtagatgc cctaattgcg gtgcctggaa tcaatttgaa
474601 gaagaaacgc aagaagtaaa aaaagtatca actaaggcaa ctgctagtcg cttaatgact
474661 aaaattggaa ataatgatcc agtaaaatta aatgaagtaa aagctgaaaa agaaaaaaga
474721 attgtaactc cttttgaaga actaaatcga gttctaggag gcggaatagt tccgggttct
474781 ttagttttaa ttggtggaga tcctggaatt ggtaagtcaa ctttgatgct tcaaattacc
474841 ggtgctttag ctaaagagca tagtgtttta tatgtttcag gagaagagtc ggctagtcag
474901 ataaagatgc gagctgatag tttaggtgta agcaacagcg gaattttgct ttatcctgaa
474961 accaatatgc agaatattcg cgaacagatt gatgaaatta agccggattt tttggtaatc
475021 gactctatcc aaacaatgaa tgagccatct cttgattcga tggttggatc agcttctcaa
475081 gttcgagaag ttacgagtga gttaatgaag attgccaaga atgatcagat tactactttt
475141 gttattggac atgtgactaa agaaggtgca attgctggcc ccaagattat ggaacatatg
475201 gttgatactg tcctttactt tgaaggggat ggccaccatt catatcgaat tttaaggtct
475261 gtcaaaaatc gttttggtgc agctaatgag attggtatgt ttgaaatgaa aaatgaagga
475321 ctaacagaag taaataatcc ttcagcgatt ttcttagacg aacgtttacc aaattctaca
475381 ggttcagcag ttgtcgtttc gcttgaagga acaaggccac ttctagccga tattcaagct
475441 ttggtaactc caactgcctt tggctatgct aaaagaacta cgtcggggtt agatttcaat
475501 cgtgtagcct tattattagc ggttttagaa aaacgtggta acttaatgtt acaaaatcaa
475561 gatgcatttt taacggcaac aggtggaatt aaattaaatg agccagcgat tgacttagca
475621 atttgtatgg ctgtagcttc tagttataaa aataaagaaa tttcttctac tgattgtttt
475681 gtaggcgaag ttggcttaac cggtgaaatt agaagagtta atcaaattga agctagagtt
475741 aaagaagctg ctaaagtagg ctttaagcga atttttattc caaaacataa tttaactact

475801 gagcttaaga ataattctga gattgaagta attggtgtag caagtttgcc gcaagcttta
475861 aaactggttt ttaattagaa gatagattga acttttgctc gttaaaagtt acacttaaat
475921 ggatgaattt taataaattt tgaaagaggc gctttaattt tggcaaaaca aaaaatccgt
475981 gttcgttacg caccaagtcc aacaggtcac ttgcatattg gtaacgcacg tactgcatta
476041 tttaactatt tatttgcacg tcataataaa gggactatgg tcttaagaat tgaagatact
476101 gaccaaaaac gtaatgttga aggcggttct aagtcccaaa tggagaactt gcactggtta
476161 ggtattgact gggatgaagg tcctgataag ggcggcgact ttggccctta ccgccaatca
476221 gagcgtaaag atatttacaa taaatacatt aagcaattaa ttgatgaagg taaggcttac
476281 tattcataca agactgagga agaactagaa gcacaacgtg aagaacaacg tgcaatggga
476341 attgctcctc actacactta tgaatacgaa ggtatgaccg ctgatgaaat taagcaagct
476401 caagcagatg cagaagcaaa aggcttaaag ccagttgttc gtattcacat tcccgaaaat
476461 agaacttatg cttgggatga tatggttaag ggtaaagtat catttgaatc tgatactatt
476521 ggtggcgact ttgttatcca aaaacgtgat ggtatgccaa cttataactt cgcagttgta
476581 attgatgacc acttaatgga aattactcac gttcttagag gagacgacca cgtagctaat
476641 actcctaagc aattagtagt ttatgaagca cttggctggg aaccacctaa gtttggtcat
476701 atgactttga tcattaactc tgaaactggt aaaaagcttt ctaagcgtga cgaatcagtt
476761 cttcaattta ttgaacaata tcgtgacctt ggttacttgc cagaagcaat gtttaacttt
476821 attactttgc ttggttggtc acctgtaggt gaaagcgaaa ttttctcaca aagagaacta
476881 attaagtcat ttgatcctaa gcgtttatct aagtcaccag ctgcttttga tcaaaagaag
476941 cttgaatgga ttaataacca atacgttaaa aaagccgatc gtgatgtttt attagatctt
477001 gctttgaaca acttacaaga agcaggattg gttggcaaag aaatttcacc tgaaaagatg
477061 gaatgggttc gtcaattggt aaatatttac gctgttcaaa tgtcttacac taagcagatc
477121 gttgatatta ctaagatctt ctttgaagaa gctccagaat tatctgatgc agaaatcgaa
477181 gagattaaaa aagacgacgc tcgtcctgta attgaagaat tcaagaaaca attaaatgca
477241 attcctcgct ttactgctgt tcaagtaatg aacgcaattc aagctactcg tcatgaaact
477301 ggcgttaagg gtagaaaatt atttatgcca attcgaattg ctgctactcg ttcaatggtt
477361 ggacctggta tcggtgaagc tattgaatta atgggtaaag aaaaagttaa caagcacatt

477421 gatttaactt tgaagcaatt aagcgattta ggtctttaat tatttctgat gaagttaaag
477481 ctacgtgaaa gcgtagcttt ttttgttagg aagataattt ataattttat ttaatttaga
477541 gatagttaaa acgttgatat tataggattt ttgtttttat tgattttctg aaattactct
477601 aaatttgagt aaatcggagc aatattagga acaatatttt tatttttcgt aaatgtgcta
477661 atcaaaagct tttaaatgat ataatttaag tatcttgtta ttaatttata aaattcatgt
477721 ttaatcaggg tgtagtaaat tgctacgccc tttttatgtt ggtgcatccc tttaagaata
477781 ggcaatttat agtaatctat gagagattaa ataaagggga gttgttaggg atgctagaac
477841 tgaatgagcc agctacaata aaaaatacta gaaaattctt tgaaaaggat ttagaacgat
477901 atgaaaccgt gtgcgctggt cggtatagcg tccaaggttt gaactttgaa aatatcaaag
477961 tgaacagttc aaataataca agtgcagtaa tgaatagata taaccaaata attacatata
478021 gccagcgcct agcgtgttta aaaccagcta tagacaactg cactaatgaa ccaaataaac
478081 catataaacg tgttttagaa ctcagatatt taaaacatat atcagcttgg aatgtagcaa
478141 ataatattgg atatagtcac acccaatacg gtgtaatcct taatagagca tttttgcagt
478201 ttgcagatgt ttatctgttg gaacaattaa agcaaggcgt gaaagatatt ctagacttgc
478261 atgtatatca tggagtcacg aattgttaca ccatgcttga aacataaaaa ataaacagtt
478321 attaatcttc tgaaagttga taatttcact atgtttctga aacagagcgg taattttaag
478381 gtcgtgaaaa acgattttaa gaattaaaat atttatcttt tttgaatgta aaaaaagcct
478441 tccatgtagt ttcttgaatg ctacacgaaa ggctttttat aatgatttag tatgattttg
478501 gttattgagc taccccaata aaatttaagg cacggttttc gaccgcaaga atattttatc
478561 ataatcagca ttaatttata ttcttaccta cctgtttttg attctaagct gtttttatta
478621 tttgaacgca taatcatact aaataaaaat aaatccattc taagggctta aaaatagatt
478681 tatttaacat cttggatttt gccgtaataa ataatgctta atttcgccta aaattaggtt
478741 gtaccaagca tttactaaat agccccctct aaaaactttt taaaaatata tatttcacat
478801 aaaagaatga cattggtgcg gctctttcta ccgtgagtag tgggcggggt gcaaaataaa
478861 acaggtcgtt cagaacgact tgtttaaatt catttatcgt ttattttta atccgttgtc
478921 gttttgatcg atcacgaaaa tttaaacatg acattcaaaa ttattctaaa aaatttaaat
478981 acgtctgaag cgtttagaaa aaatatctaa atcaatttaa ttgagttcaa atagtctaca

479041 ttcatccctc aaaggtgctg acagagctat tacatggacg ttttaatatt ctttattgat
479101 taattattga tttaaattaa ttgtttgctg ttctacttga tgaattatta aacatgaaca
479161 gttaagaaag caggaagcaa aaagaaatag agaaaaataa cgtcgcaaaa tgcgacgtta
479221 aagaactctt tcacaaagat cctacattag gatggaagtt atagggatga agacataata
479281 aaagagcagt agcaagacaa taaaactact gctccctttt gtgatcctga tttaaattaa
479341 ttatagctta tttttatttg ctgttcagta aatctcactg cgcaaaattt cgcagtgaaa
479401 aatatgtgta gggtagtttt aataaataat tttcataatt aatttttata attaatttta
479461 atatgcttag cgaatcgcta agacccactt aacaaatcgc taagacccac ttagcgaatc
479521 gctaaggctg tggataactt gcaaagtaaa taaaaatacc gtcgacagtt gcgacggtat
479581 aaccttgtaa tctatattac atatttaatt gttctttagt taattgtcct gttaaccata
479641 aagaaacgcc gtgcgtactt aaaaagttat cacggtgtat ttttaagtat tgtattagtt
479701 ctttttgtga acggtattct tttatttttt gctggatagc ttttttttatt tctgttgttg
479761 gcttgattgg ttcaaaatca tgttcttctt taaacatgcc gtttatataa ttgataactc
479821 caattacttg gctttgctcg gtatagtgcg acattttgat agtttttatt attcgcttag
479881 ttttattagt attttgtctt tttaagacaa cagtaataaa tcctttatct tttaagctat
479941 taatattttt tgaaacagta acagggtgta aattaaaata ttccgcaaaa tatgcgttag
480001 tagcaaaaca tccttgtcct ctgtctaatg tttcgatagc tttgagtata attgcttctg
480061 tgggcgtcaa tccttttttta aacgtatttt tatctactgt aaattgaacc catttttcttt
480121 cgtccgtcac ataaaacacc acctcacaac cttaaaacta atatcgctta tttgatccat
480181 tcaataggct gtggcgtgta tttattaata tgctgaacgt gtgggacttc ttgaaggaat
480241 atagttgtat tctgtccgtt tttcatggtg gtaaaagtca taatttctaa gctaagccat
480301 tcgataattt taaaaaatgt ttttgatgaa atgcagtcat tgccattaat aaagttagtt
480361 aaagttccac gtgaaacatt agccattgct gacagttcag cataatttag tttaaagttt
480421 tctttttttgg ctattaattt ttctcttact tcttgaatag acatattaat taaattgttt
480481 tcttccatgc tttacctcgc taaaattatt aaaagtcttt gattaacaca tttacgcttg
480541 ccgttggtct gctataatta aaaagagaat taaaaatctt tgattaaccc atttattttt
480601 atttcttttt gccgttgatt actgctatag tcaacggctt tttattttgc tcttttttag

480661 gttgttcttt acttttaagc cactctgtaa tgctttgttt tccgtataac ttacgtccat
480721 caataaccgc aataggcatc cctaaagtag cccagtagct tatagtgctg tctgcaactc
480781 caaaatactt tgctacttct ttgcggttta aatagggctt gaagttggtt gcttgcttaa
480841 ttccatcatg gaccgcttca ataaccattg attggatttt ttgccgttct tctgggtcta
480901 gtattacctt catttttata cctcacaaaa aagagaattt aaaagactgc ctaaagtatt
480961 aactttgtca ttggttaaat ccatttcatt aagtccatac tctgaacgcc aaacaataat
481021 tttatttttt tcagaattta taaagattgc taattcgatt tctttttgc tatagctgtg
481081 cgctttgccg ttgcaaataa cggttaaagg gaagcttata cttgcttggt cggtttcttc
481141 tacataagca tgcaaaccgc caatattttc agctaaattc ttgccgtgct taataaataa
481201 aatcttgttc attgttctaa tcctcgtcaa attcaaataa atcttgttga cctgataagg
481261 ttgtaccgct gttaaaagta ttcatcattg cgctaattgt tttacttaat gtgttacgtt
481321 cgctgtttag catccctaca acttcaaaaa attcgttcat tgtctgaatg cgataataac
481381 cgctcttgtc ttttaaacgg cttgacccaa tcaaaacgcc cttaattctt agcttttgga
481441 ttgtataagt aatatttctt ttagttgtat tgttgattat tgctagcttg ttggaagtaa
481501 tagggtgctt tcttcctgaa gggatgcact ttaaaacttg cttttcaaat ttagttaatt
481561 tcttgttggt tggtttcatt gtttctgttt cttctttcat aaaattctct agtttccttt
481621 gcatcgtttt cataatatcg atcgaaccaa tctgctaaat ctgctaactc tagggcggtc
481681 ttagcaacat ctcgccagat ggtagatgta gggtaaagtg tcccgtctat gcggtaatta
481741 ctttcatttt cagcaaactt gatcgattct tttaaataat taagtttgtt tgaataatta
481801 tcatcgtgtt tattgaattc gtggtctgta tatttcattt tctaaactcc aattctttat
481861 ttaattaatg ccaggaacat tcccagcgta cgggtaaagg ttacgactgc aacgagtgca
481921 gccaaaccca aaacgacaaa aagaaatata ttaaataatt cggcttcatt gccatctggt
481981 aaataaagcc agtctttaaa agccttataa attctcttca tgtttatttt tccctgtggt
482041 tgttactact gtatgaactt tcataaactt atcaatatct gttttcttaa tctttttaga
482101 cttacccaca attgaaattg gtaagcctgc attaattagg tcttttaatc ctgtgtatga
482161 ttgaatacct aaataacgca ttgcttgctt aaaattaaag tattctgtgt ttggcatatt
482221 atccactttc ttcgttacat atatttgtaa tttgttttac attgatatgt tacattacaa

482281 ataaatgtaa ttcaataaaa atttcaaata tttgtaactt ttttgtataa
ttaagcagta
482341 aggaagtgat ttaatgaaca gaattaagca gttaagagag caaaaaggct
tatctcaacg
482401 tgactttata aaatcgttta atttatttct gaaagaaaat gctaataaat
acgatggtaa
482461 gccaggtatt aaagcggtaa gttttgccac tgggtcaagg tgggagaatg
gattaaataa
482521 gcctactagt tcaatgtggc aagcattagc agatttcttt ggtgtgtatg
tcccttactt
482581 acaaggtgca tatagtaagg tcgaaatatt aaaggtctta caagaatatt
atttaaggta
482641 ttatattggg gattattcta ctgatgatat agaagactta atttacaccg
atatcgggga
482701 tgtagtagat gactttgtta taagcaaaaa aataaaaccg tggaatatta
aaaaagaaaa
482761 tgtattacta agtaaagaag aagtaagtag cactaaattt tggtgggaac
attttcaagt
482821 ggtgtttgac cacatagcta ttatttggtt gcttactaaa ccaagcctta
acgctacaaa
482881 aagagatgtg gcagatgcgt taatagacgc tttaagtgga gaacaaaata
atatgttact
482941 tactaggcga atgaaattta ttgataagta tctgtatttt atgaaaggga
aaacaatcaa
483001 gtctatttac gactttgagc atccacactc tttagatggt aagaaccatt
atatagatga
483061 aattcattag catccctgtt cggtaaaaaa actcggtaaa tttgattgat
ccactttcta
483121 aacattcttt tttatggttg tcccaatgaa gttattaaaa gtgcaatata
aaaccagtcc
483181 cctgaattag ggtagtggtt ttaattactc caaaaattgc ctgttatttg
attaactccg
483241 tagtctgttt aacggtcctc gaatagaacc taatatctgc gctaccaatt
tagtacctaa
483301 ggtaacgttc cgttacatta aggggtctgt attttaggga cacctttatt
tttaatctgt
483361 cttgttcttg ttaaatcaat gtttcatata tcggcaactg ctattgctga
tactcaataa
483421 ttaaaagcct ttgattagca catttacgct tgtagcaaaa taacaaggtg
attaaacatg
483481 aaacaaaaca aagatataac aaggtataag acccaaaacg gcaaaaaacg
ctataggttt
483541 aaagtttatg taggtaagga cgaggagaca ggcaacagca ttcaggcaag
gaagcaaggc
483601 tttaagtctt acgatgatgc tttaaagtcc tactttgaaa ttaaagacaa
ggtagcaaag
483661 ggaacgtata cagggaatga gaacaaaaag cataaattct ctgaactcta
tgagatgtgg
483721 ttagaaaatt ataagaatac tgtaaaagtt agtacgttaa gcaatactaa
attatttttc
483781 aggcttcata ttttgcctga attaggtaat ttgtatattg agaaaataac
cacttttaaa
483841 tgtcaaaaaa ttgttaataa atggtttaag gaactaccaa aaacatataa
ggtatacact

483901 aatttagcta gtaatatatt caagtttgct gttaataatg atttaattgc
taaaaatcct
483961 atgaagtcgg taattaagcc aaaattacag gtagaaaaga agccatttac
taacttttat
484021 tcaaaaaacg aattaaatat atttctaaaa gaagctaagg aatgcaaagg
gctaaaggta
484081 tatatgcttt ttagattgct cgcctataca gggatgcgac ggggtgaagc
attagcttta
484141 aaatggtctg atattgattt cagtaaccaa acaataaaaa taaacaagac
tgtaacaact
484201 gctgaacatg ctgctttgat aattggaaca cctaaaacaa aagctagcta
tcgaactata
484261 gccatagata ggcaaactat taaatattta agtggttgga gaaaagagca
aagaaaacaa
484321 ctatttcagt taggatttaa tgctctatct gatgaccagc ttatttttag
caatggtgat
484381 aatactttga ttagacaagc tactgtcagg aagtgggatc aaagcatatc
aaaacatgca
484441 ggtctgaaat atattacagt tcacggcttc aggcacacac acgcctcttt
attatttgaa
484501 gctggtacac caatgcagga cgtaaaggaa cggctagggc atagctctat
tactacaaca
484561 atgaatattt acactcacgt cactaagtca gcaaaaaaga agacagccga
caattttgcg
484621 caatttatgg aagggtagga gcaatattag gagcaatatt taaatataat
ctttgaaaat
484681 aggcgcaaag cttgatataa cagtattttg aaaagatata gcctatattg
ttagaatata
484741 ctaggaaata aatgtcttaa gtgaaagaag gatgatcatg aaggtcttta
atactttaac
484801 tcggcaaaaa gaagagttta agccattagt agctggtaaa gtaagcatgt
atgtctgtgg
484861 accaactgtt tataactata ttcatattgg aaatgcccgt agcgtgattg
cattcgatac
484921 aattcgtaga tattttgaat atcgcggcta tgatgtgaaa tatgtttcta
actttacaga
484981 tgttgacgat aagatgatta atgaagcccg caaagaacat acaactgtgg
gtgaactggc
485041 taaccgctat attaaggctt ttatggagga tacagaggca ttaaatattg
agccagctac
485101 tcttcatcct agagccactc atgaaattaa agaaattatt gattttgttc
aagatctaat
485161 tgataagggc tttgcttatg aagttgacgg ggatgtttat tatcgtgcta
aaaagttccc
485221 tcactacggt caattaagcg atcaaaatat tgctgaactt gaagaaggtg
cttctgaaca
485281 tattaatgaa gaagaacaaa ataagaagga ggatccaatt gattttgcct
tatggaaggc
485341 tcaaaaagaa gacgatgaaa ttgcttggga ttctccatgg ggaaaggggc
gtccaggttg
485401 gcatattgag tgttcagtaa tgtcaactaa gtatctcggt gagacgattg
atattcacgg
485461 cggtggtcaa gacttagaat ttccacatca tgaaaatgaa atcgctcaaa
gtgaagctaa

485521 aactggtaag aaatttgtta attattggat gcacaacggt tttgtaactg tgggtaaaaa
485581 acaagaaaaa atgtcaaagt cgctacataa ttttgtaact gtgcatgata ttttaaagga
485641 aattgatcca caagttttac gtttctatat gtctagtgtt caatatcgtc gtccaattaa
485701 ttattctgaa gacggtttaa aacaagcaga gactgttctt aaacgttatc aaaatacctt
485761 gcgtaatctt gatgctcgct tagcagatga gcaagaacct cttgaagata atactttaag
485821 ggataattta acgcaggcaa aggctgagtt tattgaagca atggatgacg attttaatgt
485881 tcaaaatgct ttgagcatta tgtatgacct agcttcaaac ctaaatacgc atcttcaaaa
485941 agatcaagtg gataaacctg ctttaaaaaa ggccaagaaa ttgttgcttg attggcttga
486001 aatttttggt gttagcttta atgaaaagca ggctgatgac gaagacgaga tcgaagctat
486061 ggttgctaaa cgtgatgaag ctcgtaaaaa taaaaactgg gctgaaagtg atcgtttaag
486121 agatgaacta caagcaaaag gcattgtaat tgaagatacc ccacaaggaa cgaggtggca
486181 ccgtgcttaa gccaaaggtt ttgacagaaa aaaagactaa tccagatgcc ttaggaccgc
486241 ttaccctagc ttatttaggt gatggagtgt atgaagttta tattagacgc caccttgtga
486301 aaggcggtat tgtgaaacca caaatgcttc aaagatatgc aacgcactat gtttcagcta
486361 aagctcaagc tgctttgatt acaaagatgc aagacttaaa tatgcttgat gaaaatgaat
486421 tagcagcctt taagcgtgga agaaatgcta aaagtcatac ccacgctaaa aatacttcga
486481 tcaatactta taagttatca actggttttg aagcaatgtt tggctattta gatttattag
486541 gcaaaaaaga gcgtgttgat gagctagcaa attggtgcat tgaccaagta gaacagggag
486601 gattagacaa ctatgagttc gaataatgat gagtttgtgt atggacgtca tgcaggaatc
486661 gacttcttaa agacgcaaga tgctaactta attaataaag tttttttaca aaatggcgtt
486721 caagaagaat ttgctaatca agtttatcaa ttagcacgta aaaagaattt agttgtgcag
486781 aatgctccga aaaataaatt ggatcaaatg gttaatcatg aaaaccatca aggattagtt
486841 ttaacagtcg ctccatttga atatgcggat ttagataagc ttcttgataa atttgaccaa
486901 gatgggaaag atccctttat tttaatgctt gattcaattg aagatcctca taatttgggt
486961 tcaatcttaa gaacttgtga tgcaacaggc gttgatgcag taattattcc aaaaagaaga
487021 gcaactggct taacttctgt tgttgctaag acttcaactg gtgctattga ctatgttcca
487081 gttgctcgag ttaataattt agtacaaact actaaaatgc ttaaaaagcg cggctactgg

487141 atttttggta ctgccatgaa aggtgaagat taccgtaagt ggaatgctaa
tggtaagact
487201 gtacttgtaa tcggcaatga aggtaaagga atttcacctc ttcttttaaa
gcaaatggat
487261 caaactttaa cgatcccgat ggttggacat gttcaatcgc taaatgctag
tgtagcaact
487321 ggtgttttac tttatgggat gtttaatagc cgtcatccag aaaaataaaa
atgtaggaaa
487381 acgcttgcat atattaattg aacctgatat gataacaatc agtaagagat
tgaaaaagta
487441 gattgttact attggattag gcgagactac tgaataactc taactagcaa
tacgttagtg
487501 gagtttattc agtagtcttt ttttatcctt ttttaaggag gtggggagaa
ttgttgttga
487561 tcaagcgcgt gtttaataat aattcagctt tggttgagct aggcaataat
cgtgaagcag
487621 ttgtagtcgg aaacggaatt gcttttaaaa aaactgaagg aaaggagata
gatacgagta
487681 aagttgaaaa tatttttttat cttgaaaata gtgcaacaaa acggactatt
ttctcattat
487741 taaagaatac accagtagat attgctgtaa ctactatgca tgtcgttgat
cacgcacata
487801 gaaagtatca ctatgaatta tttgattatt tttacttgtc gttgagcgag
catattttag
487861 gagtatatca tcgaatcttg gataatactt atcaagcaag tcaaattcct
gatattaaag
487921 atgaatatcc tctggaaaat aaaattgcac aagaaagcgt aaaaataatt
gaacgggatc
487981 taaaaattaa tttgccggta tcagaaagaa aaaatattat gttgcattta
attaatgcaa
488041 agattcctga aaaaaagcag ttcaatcgag aaaaaaatat gatcaacatt
gaagaaatgc
488101 tgcatatagt tcaggtgatc cttgatcaac ataatattta tcgattagct
agtaataatg
488161 atgaatatga gcgttttatg attcatttgc aatatctttt caggagatta
gagcagggag
488221 aaaaatttcg ctcatcggat attactaaaa aagtaaagga tgaattaata
tccgaatatc
488281 cggaatcatt tgcagtagta aaagaaatag atgaacagtt gaaacaagat
tttcaatgga
488341 aaatatcgga tgaggaaaag ttgtatttga ttgttcatat tcaaagaatt
tatgagaaaa
488401 gttcaaaata ctaggagaaa aagatggtta gtaaagaaga aatttcaatg
gttggattta
488461 caattgttgc ttatgctgga gacgcaaaaa cagacttgtt aaatgctctt
aaattagctc
488521 gtgaaggaaa atttgaaaaa gctgaaaagt tgattgaaga agcaaataat
tcattagtag
488581 atgcgcataa tgcacagact aagttaatga gtcaagaagc taatggagaa
gaattagaga
488641 caacttttat tatgtctcat ggacaagata cactaatgac aacaatgctt
ttacgtgatg
488701 aagttaaata tttcattgat ttgtataagc aaaatttagc tttgcgagct
gaaattgagc

488761 agcttaaaga agaaatgaac taaataaatg gaggagtaaa aatggatggg ttggtaaaaa
488821 gaattgaaaa agcgcagcca ttttttagaa aagttgcgac taatcaatat ttaagagcaa
488881 ttcgtgatgg ctttatcgcg ttaatgccaa ttattatctt ttcaagtttt ttcttgttaa
488941 ttgctaatgt gcctcaaatt tggggatttg tttggcctaa aaaagtagct acagctttaa
489001 atttatttta taacttatca atgggctttt tgtccttaat ggccgcttct actgtagcta
489061 aggccttaac tggaagttta aatctaaagt tgccaaaaac taatcaaatt ccagttgctg
489121 gagttcaata tacagctcaa atttcctttg catttgtggc aatagatact ttgttaaata
489181 aaggaaattt atatttagct actgatatga ttggaactaa aggattaatt tgtgccttct
489241 tagtagcatt tattgtacct aatatttatt atttttgttt taaacataat gtcacgatca
489301 cgctacctga tgtagtacct caaaatattg ctcaagcttt taagaatatt attccgtttg
489361 ctttagcaac tactttcttt tgggcattta cgctaatctt tagagcttta actaatatga
489421 atttggcagc ttggataatt aaaagcctaa ctccactttt taccgcagcg gatgggtatg
489481 ttgggttggc aattgtttat ggtgcaatgg cgtttttctg gtttatcgga attcaagggc
489541 cctcaatcgt tgaaccagca gttacggcaa tatacctaac taatgttgaa gctaatttaa
489601 gagcatttaa ttcaggaaat attcaaggcg ctaatcatat tttgtcacaa ggaattcaaa
489661 tgtttgttgc tactttagga ggaactggag caactttagt agtaacgttt atgtttgctt
489721 ttttatctaa gtctaaacaa ttaaaagcag ttggacgtgc atcaacaatt ccagttattt
489781 ttggtgttaa tgagcctata ttgtttggtg ctccattaat cttgaatccg attttcttta
489841 taccttttat ttttgcgcca attcttaatg tttggatctt taaaattttt gttgatgtgc
489901 ttcatatgcc gtcatttatt tacaacttac catggaccac gccgccaaca ttagggttgt
489961 ggatgggaac tggttttaat attttggctt tattactagg aattttatta ctagtagttg
490021 attcattatt gtattatcca ttttttaagg cttatgacgc ttcaatgcta gcccaagaag
490081 aaaaaaagga agaacttgaa gaaaaagatg ataaaaaatc ggttgtaacg tctgctgaac
490141 ctaaatttaa tgaatctaaa attccacttg gcaaaactaa agatcaacaa gcactcaatg
490201 ttttagtttt atgtgccggc ggtgggacat ctggaatttt agctaagtca cttaataagt
490261 tagctaaaga agataaatta cctttaaatg cagcagctgc tgcatttgga tcacatcatg
490321 atttaatatc tggaatggat gtagtaattt tagcgccaca aatggatact atgaaagatg

490381 aacttgcaaa ggaatgtaaa caaaataatg ctcgtatgat taccactact gggaagcaat
490441 atatttatat gacgcaacat gctaatgaat gtttgaaact attaatcaat gatattaata
490501 aatgacggag gcagtatgtc aactttattg aaaagggtaa aaaagttacc caagaatttt
490561 gtatggggtg cagccactgc ggcttaccag gttgaaggaa atacaaattt agatggtaaa
490621 ggaaagatta tgtgggatga ctatttgaaa agtcagggaa atttctcacc caatcctgct
490681 tgtgattttt atcatcgtta tccagaagac ttaaaattgg cccataatct ggggctaaat
490741 gcagttcgta tatcaattgc ttggactcgt attttcccta atggttatgt aacgaacaag
490801 ccaaatgaag caggggttaa atactatcat cgactttttc atgaatgctt aaaaaataat
490861 ttagtaccat atgtaacatt acatcatttt gacagcccta agactttgtt tgatcaaggt
490921 gattggctta atagagaaat gatcaatgaa tttgtaaaat atgcagactt ttgttttcaa
490981 gaattcgccg aggttgataa ttggtttacg attaacgaat tgatttcatt agctatgtgt
491041 cagtatattc aaggcacttt tccccctaat aagttatttg atgtttctga tgcaattcag
491101 gctcagcata atgaattagt agcacatgca aaagtagtta atctctttaa agctaaagga
491161 tataagggac gaattgggtt aattcatgtg attcaacctg tttatccagc aacaaattca
491221 gaagcagatc gacatgcagc tgatttaaga gatgcattta tgaatcgctt tttattagat
491281 ggaaccttat taggtttttta ttctgatcga acgcagcatt taattaagga aattttagtt
491341 gcaaataatg ctgaattaaa gattcttcct gaagatcttg agattctcaa acaagcaagt
491401 aagcagaatg atatgctggg gattaattat tatcaaaatc aaaccgttgc tagttatgaa
491461 ggaccaagtg aaacttatca taacggtaat ggaaaaaaag gtagtagtat ttttgccttt
491521 catggggttg gtaaagatgt gcgaaatccc gctattccga caacagattg ggactggaat
491581 atatatcctg aagggctata tgacgtacta aagagaatag aacacgatta tcctgactat
491641 cctgtgatct atataactga aaatggaatg ggtgcaaaag aagcgtggga tagttcgaaa
491701 cagtatcttg atgatgatta ccgaattgat tttattgacc aacatttggc agctatctta
491761 aaggctcgaa atgaaggagt taatgtgcaa ggatatttcc tttggtcttt acaagatcaa
491821 ttttcatggt caaatggcta taataaacgt tacggcttaa tttatgtgga tttcgctagt
491881 caagataggc atttaaaaag aagcgccttg tggtttaagg ccctaagtaa gactatgcaa
491941 taataatttc ttaacagata agaagttatc gttccgaaac taagcacaac tttactatac

492001 ttgtatccta taaacattcg ttcccttaga aagaattcat tcttttttta
ttctgattat
492061 caaatagaaa aaggaggaat ttcctgagat ggaaaaagtg aatgaagttt
acttgattag
492121 aagggtgaaa gaaaagcaag atatgaaggc tttacatgag cttttggatc
gctaccggcc
492181 tttaatcaat ggcgctaaaa tgaatttttt cattcgtaat tttgataacg
atgactggga
492241 acaagaagcg ttaattatgt gttatcagag ttgctgcaca tatgatgaag
atcgagcaga
492301 taattttggt gcctactatc gaacaaaatt ctataatcat ttaagatcgc
ttttaaggta
492361 tgaactagcc caaaaaagga ccgcttttac taaatcaatt tcttacgaca
atgtagtgca
492421 gaaaaattta attaaagaag aagtcgaaga aatgcatatc acgcctcgaa
aagaaatgta
492481 tcaaaagttt attaaaaagc tttctgataa agaagtaatt gctctaaaag
tttttttagg
492541 agaattaacg atcgaagagg caagtcagaa aacaaaatat agccgttatc
aattattgca
492601 agcaaaggct cgctgcaaag caaaaatgca aaaagaattg ttttaaattc
gcatagaggt
492661 gataaaaaga ctataataat atttgtagaa tttatttagg aggtaatttt
aagatggcag
492721 atgaagttat taagacagaa ttgttagacc gtcacatgaa ggaagttttt
gattggagcg
492781 attctgacat gcctgttaga gacgcactat gggactactt tatggaaaag
aatggtagag
492841 ataccatgaa gacggaaagt gacatgcttc cgttcttgaa agactctgac
gataagatcg
492901 aagccttcgt caacgaaaat cttaagaagt aacatcacct aaaaacaata
attctacgca
492961 ccgtggtcac gcattgtttg accaccaaag aacaatctct agggattgtt
cttttttat
493021 gagaaaatat attttaaaag aaaggggcgg tagaatggac tatattattg
gaatggatat
493081 tggcacaaca gcaagtaagg gagttttata tcaagaaaat ggtaaaaaga
ttgtagaatc
493141 aacaattccg tatcccttaa ttcaggaaaa agtggatcaa gctgaagaag
atccgcaagt
493201 aatttttgat gctgtgcaaa agattatttt tgacttaagt aaaaaggctt
caggaaaagt
493261 aaaggcaatt tcgtggtcaa gtcaaatgca tagcttaatt ggacttggta
aaaataagca
493321 attattaact aatagtatta cttgggctga taaccgcagt agagaagttg
taactgctgc
493381 agagaagagt ggattagcta atagtattta tcagcgaacc gggatgccgc
cccatccaat
493441 ggctcctgtg tataaattac tgtggattca agaagaaatg ccagaacttt
ttgcacaggt
493501 gcaaaagtgg attggaatca aagaatacat tatctggcgt ttaaccggaa
aaatgatgac
493561 agatacaaca atggcagctg ggacgggatt gctgaactta aataatttat
cttgggatga

493621 agatttactt agccagatca ctttagaaaa agaaaaactg ccaatcctag ataaaccaga
493681 gactgttgtg ggaggaataa tccccaatta tattcaaaaa ttaggattaa ctgatcagac
493741 caaaattatt ttaggtgcaa gtgatggata tttatcaaca attggggttg gagttctaaa
493801 tgataaatct tttgctttaa atattggtac atcaggtgct gtgagagtaa ttgcgtcaaa
493861 ggcgatagtt gattcagaaa atcgtttctt ttgttatcca gtagataaga agcactacct
493921 attgggtgga ccagtaaaca atggcggtat agtatttgaa tgggctagaa agacaatttt
493981 tggtcctgat cagacagctg aagattttat caatgtggca gaaagtgtcc ctgctggaag
494041 caatggattg atctttcatc cttacttggg cggggaacgg gctccaattt ggaatgccca
494101 ggcgcgtgga tcttttattg gactcactag aaatcacact aagccgcaaa tggcacgcac
494161 agtgttttag aaggaattgt ttttaatctt ttgggtgcag ctagaggctt acgtgaaaaa
494221 attggtgaac cagaggcact aagagtgact ggtggttttg taaagagtga ttttgtgaga
494281 cagctaattg ccgatatttt taatttgcca gtaattataa ttaaaaatga ccaaagcgga
494341 actttagcag caatgttttt agcacaagtg gggatgaaaa gagaagcaaa tttagaaaaa
494401 atcgttaaga aaattgatga gagcaatgtt tattttccta atcctaaaaa tgttgcagtt
494461 tatcaagata ttattcccat ctatcgtgaa gttgagcacg atttagatca aagttatgaa
494521 aaaatatcaa gttttcaaga aaaatatcct agattatttg aataaattaa aaatgatcgt
494581 ttagcataaa aactaaatga tcattttttg tttatagata aatattttta atcaaccggc
494641 caatattttc actaatctga gcaagatcat gttgactatc cttaattgct tgtttcaaat
494701 ccttagcgcc actttcagtc gcaaaaattg catcaattgg accataaagc tcgttaactt
494761 ttttaccaat gttaccagtt aggcaaatta cagtggaagt tggtgcgact tctttggctg
494821 ctatagcaac accatatgga gttttaccaa acttggtttg aaaatcagtt gctccttcac
494881 cagtaaaaat aaagtctgca ttctttgttt tttctttgaa attggtcatt tcaagtacgg
494941 cttcaacgcc agactggata gtagcatttg taaaggtgag taaaccaaag ccaagaccac
495001 cagcagctcc ggcaccagga gtattttcat aattttgttt aacatctctt cgtacaacag
495061 cagcgaaatg atgtaaattt tgatctaatt cttttaccat ttcaggcgta gctccttttt
495121 gcggaccgaa gacagaagag gcaccatttt tgccagtaag tggattagtt acgtcactag
495181 ctaaaatgat ttcagtatct tttagacggg gatcaatttt agagttatca attcttacta

495241 gatgatttaa gtttccaccg cctagaggaa actcttggcc ctgtaaatta agcagtttaa
495301 caccaagtgc ttgagccatt ccagctccac cgtcattagt acaacttcca cctagtccga
495361 caattatttt tctaattcct gtgtctagaa ctgttttaat taattctcca acaccgtaag
495421 tagtagttat tagtgggttt ctagtttgat cattaacata ttgcagacct gctgcttcgg
495481 ccatttcgat gacagcagtt tctttgtcgc ctaaaatacc ataatgagca gtgacagaag
495541 tatttaaagg accggtaact gatttagtaa aaatttttcc atttgtggca tccactaagg
495601 cttgcattgt accttcaccg ccgtcagcca ttggaatttc aatgtattcg gcagaaggga
495661 gggctcgttt taggccagta taaattgaat tagcgacttc ttttgcagta agtgagcctt
495721 taaatgaatc aggtgcgatg ataattttca taaatctacc ttttctgttt agtaaaatat
495781 ttttcaataa aaaagggtgt aaactgtaca accttaagta caatttacac cttttgacaa
495841 aaatatctaa attatttaat tatctccaaa acttcactgg caatttgctc tggcatcaaa
495901 tgatcacgct tataaataga tccttttggc tcgttatcag tgtattcttt cttagcaccg
495961 taattcttaa ccatcatctt agttggtccg taatagcttg cgactttttg accaaaacca
496021 ccatcaatac tattatcttc aagagtaaca actaattcgt gatctttttg taggtcatct
496081 aaagtatcag tatcaaggtg agcagcagat tttggattaa ttaaagtggc gttgatattg
496141 tctttcttta gtaaatccac tactttttca cctaattgat aaaagtcgcc taaaccaata
496201 attgctactt tagaaccaga tttaacatca tagtgaatag ttgagtaatc ttcactaact
496261 acgttgcctt caggaagtgg gttaactggt gacttaattg ctacgggatg ttttctttgc
496321 ttgactgccc attcgagcat tgattttaat tcattcaagg tagtaggagc caagtattcc
496381 cagttaggcc aatttgcaac cattgtattg tcgaaaatac ctaaatgagt tttagaagca
496441 gcagtaattc cgccaccagc aacaatcatt acgactggta agtcattagc agcaacatcg
496501 tgagaaagtt gatcatatgc acgttgcaag aaagtagaat tttcaaataa aactggcgta
496561 attccttcct taacagcacc ggcagcaaag gcaactgatt cttgttcagc aattccaaca
496621 tccttgtagt tctttggata attcttttta atcgcaccta aaccaaagac gcctggaata
496681 gcagcattga ttgcaagaat attttcttgg ttttcaatat gtttctttaa taaatcaaga
496741 attacagtac ttgcatttgg agtttttggt gcaggaacag tggttttatc agtcttaagg
496801 tcaaatggaa ggacccagtg gtgactagct tcattttcaa ctgctggttc atatccctta

496861 cccttgagag tattaatgtg taaaacgatt ggatgatcaa tatccttaac
tgctttgaag
496921 gcatcgatca tagatttaat atcgtttcct tgaccaacat atttatagtc
aagtcccata
496981 gctgtgaatg gattgtcttc agcttgaccg tttgattcac gtaatttctt
taaggcagtt
497041 actacgccac caacgttgtt atcaatagac atttgattat cattaacaac
aatgattaag
497101 ttgtgctttt catcagctgc attattgaag ccctcaaaag ctaaaccacc
agttaatgaa
497161 ccatccccaa ttaaagcagt aatattttca tgtttgccca tcatgtcacg
tgctctagcc
497221 atccctgtag ctaaggcaac agaagtagat gtatgaccta ctgcgtaata
gtcatatgga
497281 ctttcatcgg gatttgaata aggagttaca tcttcatact tatctggatc
aagccaggca
497341 taagcacgac ctgttaacat cttatgtgga taagtttggt gagaaacgtc
ccaaataatc
497401 ttgtcctttg gtgcatcaaa aacataatga taagcaattg tagcttcgac
aattccaagg
497461 tctggtccaa ggtgaccacc ttctgcagca tctttttcta ggattaacgt
tctaatctca
497521 ctagctaatt tttctaactc ttttaaatct aatttttaa gatcttttgg
actattaatt
497581 ttgttcagta aaaattctgg atgtttgttc atgtttttc accccaacat
taataaaaaa
497641 gcttactcat ttattgtagg tcaatgagta agcttttact aatataaagt
tagcatagtg
497701 atctatatat ttaatctata ctttcttcta attcttgaga agttgcttgt
tttttaaggg
497761 tcaagaataa tgcaataaaa ctaagggcaa aagctgcgga gttaattgcc
cagccaaagt
497821 tgtaagaacc agttgtatca aataacagac cattcaagta aacagaagca
gacattgcaa
497881 atgccccagt catgttaatg acagaaagaa ttgtactgta atctttagca
ccaaactctt
497941 cacgaatgag gtaaggaaga gcaactaagc aaacaccttg tcctaatcct
agaatgaagg
498001 cagatgcgat aagaggcaaa ctagttttaa agaaaatctc accgctccag
cctagtaaac
498061 caaataaaga gtagattaat aaggttagac gagtattaaa acggtcaagc
ataaaaccaa
498121 tagagatttt accagctgct gcccctagca ttactcctga tactactgat
gctccaattg
498181 ttaaaggcag accttgacta gtgatatgtc ctgaaatgtg ctgtacagat
ccagaaacaa
498241 attgtaaagc tagcattgcg aaggctagag catagaagac tggggtcttt
aaggcagctt
498301 ttaggctaat accagtagtt tcaagaactt tttctggaat atttttaact
ttgccataag
498361 catggtgttt ttcatttggc ttttcacgta aaaagaatgc cgcaggaact
aaaattccaa
498421 gaattaatag accctcacaa ataaaaccgc ctcgccagcc aaagttagta
ataatatcac

498481 caattattgg attaaagatt gtaccaccga aggcagaaac gccaagtgca atccccataa
498541 ctgtacctaa tttttcatta aaccaattcc ctaataagac aggaattgat aaggtaatgg
498601 caattggctg acaaattcca ataatgatcc aagcaatgta aaaatgagtc aagctttgtg
498661 ctacagacaa acttagcatt gaaagaccaa ttgtggcaaa acagaatgtt aaaagccagc
498721 gtaaattaag cttggtcata attttaccag caaataaaag cgtaatagct gccgctgcat
498781 tttgcagagt ggtcattaaa gcaacacttg ctcggccaac atggaaactc ttactaattg
498841 gttcaaagaa gaggccgatt gtattaacta ttagaccgaa gccaactaat gaaatcaagc
498901 atgaggcaat aaagacccac catgcaaaaa agtgtgaagt attttgtttt ttgttcactt
498961 cttatcctac tttctagagt atttatccaa ccaaatatta tatcgaaagt gaatgttaga
499021 ggaaaagaaa agagcgttct tcttaacgct cttttaatat tgtcttttta ataatctacc
499081 tgttaattgt tcgatcaaag cacgattctc attatctggc caatgttttt ttagactagc
499141 tagggctttt tcggtatatt gatcagctaa cttttgtgct cgttccacac cgccgagatg
499201 attaaccaaa ttttcaattt catgtaattg atcttgggtc agatcttttc ccatttttac
499261 caattcgtgt aaatgattat tattatcgtt ttgcaaggcg aaaattaaag gacccgaata
499321 gattccatct ttaacatcaa gtaagaccgg ctttttaaaa gtagagcttg tagttgtgta
499381 atctaaaata tcatctctta gttgaaaagc ttgccctagg tattctccaa actgtttagc
499441 ttttaaggct ttagttactt taattccact ttcataagca ccaataaaac aagttaagcc
499501 aaataaaacg ccagttttgc ccttaatttg gtttaaatat tctttttcag aaatattgat
499561 attataggca ttgttatatt gttcagtttc cccaactagg atattttgca ttgtttttcc
499621 gtcagcaatt acactgtgta aactttgagc attttcactt aaaagcgtta acgaaagggc
499681 gaataaatag tcacctgcat aaacggcgat atgtttgccg tacttagttt gaatagatgg
499741 acgcccatgt cgcatactag attcatcaat aatgtcatca tgaattaagg ttgcgttatg
499801 aagcgtttca atagaagttg ctattttctg gagatcgtca tgtttttgag tagcgtcagg
499861 accaaatgca ccaaagagca ttacacaagc agggcggagc atttttccag gaacagcaaa
499921 agtatcttct agtgcttgtc ctaataaacc gggaacgcta ttaatgtgtt ttaagataat
499981 ctgattaatc tcaatcatat ctttttgcac ttgagggaaa ctatcccaaa aagtgaattt
500041 atttttgata ttatttcttt tttctagcat agtataggta attaaaagac taagttatta

500101 acttgtcttt tgtctttgat ccttccagtc attgaatatt ggttataaga
gtagtcaaag
500161 ttaataaaaa taaagtaagt tgaagcaatg gatgttattt ttccgcaata
tttttaatt
500221 tgaattatta agaactagaa gatgtagatc taaatagata attataaaaa
atgcgatata
500281 tcaagactta ctattcttta tgattatata aaatatcttt atttttagtt
aaataagatc
500341 ttataatgta taattttaca ctatctggga tcattattta aaagtagata
ttgaatttat
500401 atttatttat taattatgct aaaattaata aataaatatt aaaaaatatt
gaaaaaatta
500461 aataatacga ctataatgca attaataaat ctattaaatg taaaggagaa
tcaagatgaa
500521 attaaaaaag agtttattaa ctattggtgt tgtagttact ttggggttaa
gtgcatctgt
500581 tgtttcttct aacaaagttt ctgcagcaga agtaggtaat accgagacaa
caacatttga
500641 aatcaaaaat aatgtacaat cttttaatca agaacatctt ttcttggaaa
gcaaagcagg
500701 aggatggctt aatcatattg cacatacagg atactgggga ggcggtcctt
taggcggtgg
500761 agagagtcca actaatccaa ttaaagagta ttatccttgg agatagcgaa
aattacaaaa
500821 aaatatttaa tgtactaata ttatttttaa taatatatta atttatttac
ttataaaatc
500881 tccaagtata ctaaaaggag aaccgaaatt tcggttctcc tttttatata
aacatagaaa
500941 aagggattat gaattaatga aaaaaatcag ctattccaaa attttaacaa
tccagatagt
501001 actaaatata gtatttacgc tacttatatt taatcaaata ggtatatttt
tctttatttt
501061 tcaagagatt attttaatac tcgtcttatt cttaaaaaag aaaaatattt
atttaagtgt
501121 agtatttttg ccatttattt ttatgtatac gtatgggtta atactttata
cgatagctca
501181 aacaaatctt aaactaactt tgcccatata tatagtctat tttactggta
tgcttctaat
501241 cttaattcca gttgttaaag aaaaatttgg aaagataaag caagaattaa
tcagattaat
501301 atctctaatt tggcttgttt tgataagtat tcattcagta ggtattttcc
caatagttac
501361 aagggaacaa aatgaattat tatatgcaat aaatgcgagt ggaattttat
atgcttttgt
501421 tacatttatt tgtgtatata tattgttaaa gctttgggga tatgattttc
actttaatct
501481 tcatgtaaaa aatacaggaa tgagatatta tggtgtatta ttattgtcgc
ttctatttat
501541 tatttggtat actttttct ctatttttt aggtttagca caatcaagta
atgagatttt
501601 taataattgg aacgtttctt tgataaatcc agattattca gcaaaatata
taaatgttgt
501661 agaaattgta ttgaattctt tagatcccgc tatctatgaa gaagtagaaa
gatatggata

501721 tattataatt ttattagtta tttttaaaag caaaaaatat caaatgcaag gggcaatatt
501781 tataagcgct ttattatttt ccttaagtca tattggtaat tttttcacta ccgacatgtc
501841 tttcaatgac gttatatccc aaactatagt agcatttgga ttaggctgtt ttttagctgt
501901 aatgctacta tatactggta aggtatggat aaatattttg attcatttta gttttgattt
501961 tttagttttt tcgataacgg atattggata tttaactgta agtatattta ataaaaataa
502021 tggttggcta ttaaagtcaa gtatagagct atgtattctg ctttttgcta catttatatt
502081 tgcgaaattt aataaaaaaa tattgaaaat gaacattgaa gaattgatct agccataaaa
502141 tataatacta attagaaaat ctatgaatat aaaagaggat caagacgaga ttagtctatt
502201 gaaattcttg ctatgaagta caattatctt acactttact ttttagatag taattgctaa
502261 aataacgttt ttttgctaag ataaatgcag tttaggagtg gttttatggc agtaaagaaa
502321 gcagctcttg catgtactga atgtggctct cgcaattatt caattgcagc cagcaagaat
502381 agaacgcaaa gacttgagct taagaagttt tgtaagcact gcggcaagca gactttgcat
502441 aaggaaacga ggtaggtaag cgatgtttaa attttttaag agtgttaatc aaactatggc
502501 aaaggtatca tggccaactt ggaaacaaaa tagacgtgat accggggtcg tagtaattag
502561 ctcaatttta tttggtgctt accttggatt gctagattta ttatttagct acttaacgca
502621 attgttctta tagtgacagt atatgtgtta taattgaatc agctaaaaag cctcccttgg
502681 aggttttttt atttgcacta aggtattatt ttaaggagtt taaagacaat ggttgaaacc
502741 agcgaaaaga aatggtacgt tttacacact tattctggct acgaagaaaa agttaagaaa
502801 gatttacttt ctagagccca atcaatggga atgcaaaact atatctttcg agtaattgtt
502861 cctgaagaac aaaagactga aactgttcgt ggtaagaaac aagaagtcga tgaaaaggta
502921 tttcctggat acgttttggt agaaatggta atgactgacg aaagttggta cgttgttaga
502981 aatacgccaa acgttactgg atttgttggc agtcacggtg gtggatcaaa gccatctcct
503041 ttatacgatg aagaaatcga aagaattctt aagtctcaag gacaaccagc taaggagcaa
503101 aaagctgact atgaagttgg tgaaacagta actattatcg atggtgcatt tagcggcatg
503161 agcggtaaaa ttactgaaat taatccagaa aaagataaac tttatgtttc aattgatatg
503221 ttcggcagag aaacaaatgc tgaacttgac tataaccaag taaaagaatt tgatgaataa
503281 taaagtctaa ttaactattg tcaatttgag taattgctgg tagtatatta gagtatcttt

503341 attattgtgg gaggagaaga aggaaaattc tccattataa ccgcatcacg gaatcaagga
503401 ggtattgacc gtggcaaaga aagttattaa cgtagttaaa ctgcaaattc ctgctggtgc
503461 agcaactcct gcacctccag ttggtcctgc attgggtcaa gctggaatca acattgttgg
503521 tttcactaag gacttcaatg cacgtactgc tgatcaaaag ggtatgatta tccctgtagt
503581 tattacagta tatgaagatc gttcattcga attcattact aagactccac cagcagctgt
503641 tcttttgaag aaggctgcta aggttgacaa gggttctggt gaacctaaca ctaagaaggt
503701 tgctaaggta actaaggacc aagttaagga aattgccgaa accaagatga aagatctaaa
503761 cgctgcagat gttgaagctg ctatgcgcat gattgaaggt actgctagaa gcatgggctt
503821 cactgtagaa gactagtgtt tttaggaacg tcggatcgtt aatcaaaatt aacgaatcaa
503881 ggtgggagag tttgagaaga ctcgtttgac cacattttca ggaggatttt tacatggcaa
503941 agcatggaaa gagatattta gaagcagcta aaaaagttga ttcaactaaa ttttattcag
504001 tagatgaagc tatgaagtta gcaaaagaaa cttcatacgc aaactttgac gcaactatcg
504061 aagttgcata taacttaagc gttgacccaa agcaagcaga tcaacaaatt agaggtgcat
504121 tggtacttcc aaatggtact ggtaaatcta agaaggttgt tgtttttgct gaaggtccac
504181 aagctgacca agctaaagaa gcaggtgctg acgaagttgg ttctgacgat ttagtagaaa
504241 aagttcaaaa cggttactta gactttgatg tagttattgc tactccaatg atgatggcta
504301 aggttggtcg tttaggtcgt atcttaggac ctaagggctt aatgccaaac cctaagactg
504361 gtactgttac tatggacgtt gctaaagctg ttgaaaacca aaaagcaggt caagttgaat
504421 accgtgttga taagcaaggt ttaatccacg caccaatcgg taaggcttca tttgacgctg
504481 ataagttggc acaaaacttt gatgcattac gtgacgttat tcttcgtgca cgtcctgcat
504541 cagctaaggg tcaatacatt aagagtgttg ccgtttctgc aacctttggc cctggtatcc
504601 acttggatcc attgaactta gactaattaa tagttactta attaagcaaa aagacttcac
504661 atttgtgaag tctttttttg tactaaggtt gaaagtacaa ttagatttta gttaaaatta
504721 aacaatacgc tatttttaga acaggaggtg aaggaagtgc taaattatat ttttagtatt
504781 ttaccttcat tattttccgg tgcttggtta actatcgaaa tctttttttg gacgactgta
504841 gtttctattc cattaggttt gttagtcgct tttggcttac attcaaattt aaaactggtt
504901 aaggctattt tgaaattcta tgtatggatt attcgtggta cgccgctttt actccaatta

504961 atttttattt tttatggctt accaactgtc ggaatagttt ttccaagata tgaggctgct
505021 ttagttgctt ttattattaa ttacgccgca tattttgctg agatttttcg tgggggttta
505081 caagccgttc ctgttggaca atttgaagct gcacaagtgc tggggttaag tcgctggcaa
505141 actattaatc gaattgttcg accacaagtt gttaaaatcg ttcttccttc aatcggaaat
505201 gaattaatta atttggtcaa ggattctagt ttagtttatg taattggctt aggcgattta
505261 cttcgtgctg gaaatgtggc aacggcgcgt gatgtatctt tagttccact tgtcttagta
505321 ggatgtgtat atttggcaat gactgcggtt ttaacagctt tattacgagt agtggagaag
505381 aagagcaatg tctggaaata aagttttaga gcttaaaaag ataagcaaga gctttggtac
505441 aagaaaaatt attgataaat tagatctctc acttgatcaa ggcgatatcc taagtatcat
505501 tgggccatca ggcgcaggta agacaacatt attacgttta attgcgggcc ttgaaagtgc
505561 agatagtgga aaatttattc ataatggaaa agaatttgat ccaactgatc gtaataatca
505621 tttggtaggg atggtttttc aagattataa tctttttcct aatctttcgg taatggataa
505681 tattacctta gctccaatta tggtgaataa gatgagtaaa tctgatgctg aaaaagctgc
505741 agagcctgtt ttaaagcaac tagacttaga aaagtttaag aatttgtatc cttaccaatt
505801 gtcaggtgga caaaagcaac gtgtagcaat tgctagagca ttgcaaatga agccagaaat
505861 tttgtgttat gatgaaccaa cttcagcttt agatcctgaa ttggtaggaa aagtaaaaga
505921 aattttctta aagcttaaac aaactggaat gacgcaggtt gttattacgc atgatcatca
505981 atttgggaaa gctgtcgctg ataaaatctt aaaagttgag ccaatagaaa aatgatgaaa
506041 aaaataataa aagttttagg cttactgatg attgttatta cagccttggg attatatggt
506101 tgccaaagac aggaaagctc ttggcaaaaa attaaagagc gtggaacctt agtaattggt
506161 gtggatgata gttttgtgcc gatggatttt agacaaaaaa atggtaagtt agttggcttt
506221 gatgtcgact tagcaacagc tgtttgcaag aaaattggtt taaaacctga ttttcaaact
506281 atcgattggt caatgaaaga aaccgaattg cgtaatggaa caattgatgt tatttggaat
506341 ggttatacca aaacgaaggc tcgagcaaaa aaagtagcat tttcacgacc ttatcttgaa
506401 aatgaccaaa ttttagttac gcgtaagaaa gataagatag ataattatcg tcaaatgaag
506461 ggaaaagttc ttggcgttca aactgggtct tctggagcag acgatttaga taattatcct
506521 aatgttctaa aaaagcgggt taaaagcata gttttatatg atacttataa taatgccttc

506581 attgatttga aagccggtag aatcaccggt ttattgattg atggagtgta
tgccaattac
506641 tacctagctc atagtaagta tcaaaaagaa tttaagaaag aaaaactacc
atatccgagc
506701 gaaaagtttt cagtaggaat aaataagaaa gacaagattt taaaagataa
gattaatcaa
506761 gctcttgagc atctagaaaa gacaggggag ttagagaaaa tacgacaaaa
atggtttaat
506821 taaaaaaagc tattgacgac ttatctaatt taaggtaata tattgtacag
taaattctac
506881 ctaagactcg ggtggctaac gtgccttaat atcccgccga ggccagaaaa
actaagagtt
506941 ttacaactcc atgtcttttt ggcatggagt ttttatttcg gctgatagaa
tttatcttaa
507001 agtaaatttg gaggtgaatt cgttgagtaa agcaatcatt gctaaaaaag
aaaagcttgt
507061 tgatgacttt gcagcagaac ttaaggaagc taaagctatc ttagtaattg
actacttagg
507121 tttaactgtt gaagaagtaa ctaacttacg taaggattta cgtgattcta
acgttaagat
507181 gaaagttatc aagaatactt acttaaagcg tgctgctgaa aaggctggta
tcgaaggttt
507241 agacgatact tttgttggtc ctactgctgt tgtttacact gctgatgcag
atgacattac
507301 tgaaccagct cgtattgttt ctaaatacga agatgatatc gacgcattat
caattaaagg
507361 tggtatgctc gaaggtaagg ttgccagcca agaagaaatc aagaaacttg
cagctattcc
507421 aggtcgcgaa ggtttacttt caatgcttgt atctgtatta caagctccag
ttcgcaactt
507481 tgcatatgct gttaaggctg tcgctgattc aaaagacgaa taattatagt
aaatttacac
507541 atatctaatt tcggaggaaa cttataatgg ctttagatac tgaaaagatt
attgaagatt
507601 taaaaggtgc ttcaatcctt gaattaaacg acttagtaaa ggctattgaa
gacgaatttg
507661 gtgtttcagc tgctgctcca gttgctgctg caggtgctgc aggcgcaggt
gcagaaaaga
507721 ctgaatttga cgttgaattg actgatgttg gtcaagaaaa agttaaggtt
atcaaggttg
507781 ttcgtgacat cactggtctt ggccttaagg attctaagga ccttgttgat
ggtgctccta
507841 agaacgttaa ggaaggcgtt tctgaagacg aagctaacga catcaaagct
aagcttgaag
507901 aagttggcgc aactgttact gttaagtaat aaggtcttaa ctgaacacac
tattaaaaat
507961 gaggactcta ttttacggag tcctcatttt tgtttacaat aaaattatgc
ttaagaaaaa
508021 cttttataaa tggtttaagg ctactttagc tatcgtttca attgtattga
tcgttctaga
508081 ctttgcagcc ataattgata ttaatggtac taatagcaag tggttttggc
ttaacaacat
508141 tattttaatt attttagcaa ttgattattt ttataggcta tatcttgcta
aagataggcg

508201 ggcttttttt agaaataata tttttgaatt agtggcaatt attccggtcg gaattgcttt
508261 taactggatg aaatttgcgc aattgggaga tattggtcta tattttagac ttttgcgttt
508321 aattcgactg gccggactag tggggatgct gaaagatatt ttgcacactc atggcatttt
508381 atacgtaata tactttagta ttgcattttt aatgttagga tcagttgcaa tatcaattac
508441 tgaacacgtt tcacttgatc aagccttttg gtgggcgatt accactgcaa gtacggttgg
508501 ttatggtgac atttcacggc atacaatttc tccgcaatct ttgttgggaa aactagtgat
508561 tctagcaatg atcttgattg gagtaggagt aatgggaatt gttactagtt ctcttactgc
508621 ctacttgatg aggagaaatg aaggaaaagt cagtattaaa gataaagatg cagaattaac
508681 cttaatttta gataaattag accggctaga gcagcaaaat aagcttctcg ctcagcaaac
508741 ccaaaattta caagaacaaa ttcaagaatt aaaagatagt catacaccta gcgaatggaa
508801 caagtttaag gattggatcg aaaaaagaaa agataaaaat taaagcgtaa attcaaaaaa
508861 agatgaattt acgctttttt acatggttaa ataaaaatgt aaaatgatca tgaataaaag
508921 atagcaaccc gcgatgataa taataaccca cccagcagtc agccaaaatc tagcccaagt
508981 ttgactgtta gttgaaagaa cacggccaaa gacagcttga ttgcctttta ttctgaatcc
509041 catccataga cagaaaattc cgacaataat tgctaaaata aatataggaa tagaaactaa
509101 cattctatct cttcctttct gtaatcattc taattataaa gctttgtaga aaaaagcaaa
509161 gtagaattga taaactataa gatagaaatt gaagaagcga gaataattat gacaaaaaag
509221 aatcaaatgt attttgcggc taatccggat gctaaacatg atgaacattt agttgattat
509281 catattgacg atattgattt gaaatttaca actgatgcag gcgtattttc aaagctaaga
509341 attgactatg gttctggtgt attaatcaaa accatgaaag acttaacctt tccaaaagct
509401 gggattttag acgttggaac agggtatggt ccaatgggct tatttgcagc taaattttgg
509461 ccagatcaag aagttgatat ggtcgacgta aatgaacgag ctcttgattt agctagaaga
509521 aatgcaaagt ttaatcaaat taacaatgtt aatatctacc aatcaaatat ttatgaacaa
509581 gtggataaaa agtatgggct agtgattact aatccaccaa ttcgggctgg aaagaaagta
509641 gttgatcaaa ttttatctga agctaaagag cacttagtgg aaaatggaat tttgctagtt
509701 gtcattcaga aaaaacaagg agccccaagt gcaaagaaac taatgacaaa ggtatacgga
509761 aactgtgaaa ttttagctcg ggataaggga tattacattt taatgagcaa aaataattaa

509821 aaagataacg ataatggttt ataatgaaag cgaatacaaa gtagaaaaga taatactttg
509881 aaagaagtga atcattatga agctattgat taatattatt ttagtcttaa ttgttttggc
509941 tgggtttggt ggaatgattt atttatcagt taatggtaat agattccttg gtgcagctgg
510001 cttggttatg ctttattttg tcgggtcaat tggttatttc gttaactgct atacgattaa
510061 aaaacgtggc cgaatttaat agaagagatc caaaagatct tttttatttt cacttcttat
510121 actatacatg aacatgttta atgatgaact tctttacgtt agccagttaa tttaactggc
510181 tttttcgatt acaataataa tgattaataa gaattatagg aaagatattg aataaaatat
510241 atgtttgaaa aaagcgataa ggaagaatac atgcagctag cttttgcaca ggcaaaaaaa
510301 gctgaagacc agggagaagt accgattggt gcaattgttg tggataaaga tggaaatgta
510361 attggcgaag gctacaacag acgcgagctt gatgaggatg caacccagca tgcagaaatg
510421 attgcgatta gagaggcctg caaaaatctg ggttcttggc gtttagttga ttgcagcttg
510481 tttattaccc ttgaaccatg tccgatgtgc agcggtgcaa ttattaattc acggctagct
510541 gaagtttatt atggcgcttt tgatcctaag gccggagctg ctggaagtgt aattgattta
510601 ttcaaagtag aaaaatttaa tcaccaccct caagtttttg gaggcttatt taaggatcaa
510661 gcagcgcaaa tgttaaaaga ttttttccgc gaaattaggc gtaaacaaaa gcaagataaa
510721 tagtggaaaa aatagttaaa atactgtatt atagtttgtg ggcaatgtcc gacctccaaa
510781 ttgtgtcagg accggaaggt agcagcacta aggttgcttg ggcatgtgcc tttttttgag
510841 aaaataaaca gctcttagct cgtttgagct aggagttttt ttgataaagg aagtgcaaaa
510901 tggcttatca agcgctatat cgtaaatggc gtcctagaac ttttgatgga gttgtagggc
510961 agacagcgat tactgataca ttaaaaaatg caatcaagcg tggcaagata tcacatgctt
511021 ttttgtttgc gggaccacgt ggtactggta aaacatcatg tgcaaagatt tttgcaaagg
511081 ccttaaattg tactaatttg caagatggtg agccatgtaa tgagtgtgaa aactgtcttg
511141 cggcagatca agggacgatg aatgatatta tcgaaatcga tgcagcatca aacaatggtg
511201 ttgatgaaat tcgtgatatc cgcgataagg taaaatatgc tcctacacag ggaaaatata
511261 aagtttatat tatcgacgag gttcatatgc tttcgatggg cgcctttaat gccttactta
511321 agacactaga agagccacct gagcatgtag tatttatctt agctacaact gaacttcaaa
511381 aagtcccagc aactattatt tcaagaactc agcgctataa ttttaaacgg attgatcaac

511441 atgatttaat tgctcgcatg acctatattc ttggtcaaga aaaaattgcc tttgaagaaa
511501 aggcaattga ggtaattgct caagtagctg atggtggaat gcgggacagt ttaagtatct
511561 tggatcaaat cttatcctat gatcaggata aggttaagta tgacgatgcc ttaaagatta
511621 ctggttatgc tgctcaagaa aagattgagc aagtcttatt agacttactt aagggcgaaa
511681 cgtcagaagc attcgaaata gttcataatt tacttcaaga tggggcatca actaagaata
511741 tcttagatga actaattagc ttaactgtta aagggatgct ggttattaag agcaataaag
511801 aggcaacttt tttaactaaa gattatgcag cagaattagc taagatccca gaagaaaagt
511861 tttataattt agttcaagca gccaatactg ctttaaatga tttgcgctac actaatcagc
511921 aacaaattcc acttgatgtg tttgtggttc aagtcacgca aggggtaggc ggaaatgtac
511981 cagtagaaca aggtgattca gtttcttcaa ctgtcgttca aaagttaaaa aatgaaattg
512041 ataatttaag taaacaagtt gctgaactaa aaaatagacc agttcaagta actaatagaa
512101 gtagctctga cttttctggg actattaaga cagaaactaa ttctcgtcct aaacaagttg
512161 ctgctcaaac agttaatact gatcaaaaac ctaagagaag aaaagctagt gcaaataatc
512221 aagcagcaca ggaaaataat cgccgtcaag tctaccatgt acttgaaaat gcgacgaaag
512281 aggacttaaa tacagtcaaa gatatttggc cagatttatt gtctactttg aaggtatctc
512341 aaagagccgt catggaagtt ttgaatcctg tagctgcaag tcatgaacaa attgttttaa
512401 aatgtaaata tgaattatgg tttgaacggg caaatcaaga tggagattta atttctcagc
512461 ttgaagctga aattgccaag ttaacgaagc ataattatag tattgtttta gttgcagatt
512521 cttcatggct aaaagtacgg catgattttg tagcaagtca taaggaagaa ttacttgcta
512581 agaaatttca aaaaatagaa aaatctgaac aagaaacaaa tgaaaatcag gtagatcctc
512641 aagctaaaaa ggaagttatc gataaagcaa aagaactttt tggagatcta gcgcagatta
512701 aagattaagg agagaaaaaa tgagtagaag acctaatttt ggtggtatgg gtatgggcaa
512761 catgcaaggt ttaattaagc aagctaaaaa gatgcaacaa caaatggaag ctgaacaagc
512821 aaatttagct actcaagaat tagttggcaa gtctgctgat gatatggtag tagcaacttt
512881 tagtggtgac cgtcaattga aagacttaaa gattgataag gaagcaattg atccagatga
512941 tccagatatg cttcaagatt tagttattga tgctgttaat aaagggatta aggcagtcga
513001 tgatgctact caagcatcaa tgggaaaata cactaaaggt ttgatgtaat tatgcaatat

513061 ccattaccaa ttgcacgttt aattgataat tatatgaagt tgcctggtat tggtgaaaaa
513121 acagcaacgc ggttagcttt ttacacaatg gacatgcctg aagatgatgt ggaagacttt
513181 tccaagtcat taatgcaggt aaaagaaaat cttcactctt gttctatttg tggaaatatt
513241 accgagagcg atccatgtga aatttgccga gatgcaaatc gtgatcgttc aacaattatg
513301 gtggtagagc agccaaaaga tgtaatggct tttgaagaaa tgggtgaata taacggttta
513361 taccatgttt tacatggtgt tctgtcccca atggatggtg ttggcccaga agaaattaat
513421 attaagagcc tgattactag actgcaaaag caagatgaag ttaaagaagt cattttggct
513481 ttaaattcta gtccagaagg tgaagcaact gcaatgtact tagctaagct aatcaaacca
513541 gccggcttga aagtgacgcg cctagctgca gggttagctg taggtagcga tattgaatat
513601 gcaaactcaa ttactttgaa gcgcgctgtt caagggagaa cagatctatg attggaaaaa
513661 ggcataaagt taaaaaagca ggcgatgaac gactattaaa agtagttgct caattacaaa
513721 gacaacttgc agaacaaaaa gtttttgatc agacaacaat tgattattca tttgataatc
513781 gcgttttaaa taaaattcta catgcgaaat ttattttttt gtatgaagaa gcaagaagaa
513841 gaaacactaa atctagtagc tcatcaagcg tgattactag ataatgtagt ccaccagtgg
513901 tggacttttt ttgtggaaaa ataaataatc tgaaaaaaac aagtctgtct ttcttttttc
513961 ttttacagta caatgccaat tgggggtaga catgaaagga tatttaatta catttgaagg
514021 cccagatggg gcaggaaaaa ccacagttat caacgagctt atcaagcagt tgccaaaaga
514081 aatacagaaa aagacgatta ttacacgtga acctggtgga tcaaagattt ctgaaaatat
514141 tagaacaatt attttagatc cagcaaataa ggaaatggat gatcgaactg aagctcttct
514201 ttatgcagct caaagaagtc agcatgttag tgaagttatt cgcccagctt tggcagctgg
514261 aaaaattgtc ttgtcagata gatttattga tagttcttta gcttatcaag gtgttggacg
514321 taatttgggt attgaagctg taaagcaaat taatgatttt ggaactggcg gacttgaacc
514381 agatttaacg atctttttag acttagatcc tgcaactggc ttggctagaa ttgaaaagga
514441 acgagcaggg caagaagatc gacttgaaca agaaaaatta gctttccaca aaaaagttta
514501 tgccggatat atagacttgt taaaacgata tcctgataga attaaaaagg tggatgctaa
514561 tttgccaatc gaggaagttg cggctaatag tgttaaaata ataagaaagc aattacctga
514621 tatttttatg taaggatttg gtaagaatga aactcgtttt agcaattgta caagataaag

514681 acgcagatgc attggcaaga gaatttattc aaaataatgt gcgtgcaaca aaattagcta
514741 caagcggcgg ctttttaaag gcaggaaata caacttttat tgttgggatt gaagatgacc
514801 gcgtaaatga agtattagaa attatcaaaa agagttccca cactagagaa cagtatgttt
514861 ctaatatgaa tatggatgca gcaggtactt caatgctagg taagcctgtc caagttactg
514921 taggcggagc aacggtcttt gtattaccag ttgaggaatt taagcatttt taatgattga
514981 tttaaaaaat ataggtcaaa agcagactga tcttttaagg gatgcatatt taaataaaaa
515041 agttgcccac agttatttgt ttgtcgatcc aatgcaaaaa aaaggactta atacagctta
515101 ttggctggcc tgtcttttta attgtacagg agaaaataag cctgatggga cgtgcaataa
515161 ttgccaaaga attttagatg gcaatcatcc tgatgtcttt ttagtaaaac tagaaggaaa
515221 acagacgctt tcaattgatc aaattcgtcc tttgaaagaa gaattggcaa aaagtcctgt
515281 tgaaggcaag cgacgttttt ttatcattga aaatgctgaa aagctaactt tagctgcaag
515341 taatgccttg cttaacttat tagaagagcc ggtagcacct gtagttacga ttttgataac
515401 caataatgaa aatcaaatct tgccaacagt taagtctaga acgcagattc ttaatttctc
515461 tgatgaaaaa attgatagta agagagcgca gttgcttgaa tatggcttaa ctgatgaaga
515521 aattgatgat ctaggcgata cggctaagct tgaagaagaa agtaagtatt tgttccaaga
515581 gttattagag cagaatgatt tggcactggt acgcgtgagc cagattagcg gtcttgctac
515641 aaagccagca agtcaaaaat ttgtttttta tcaacttaag actttagcga tgaagagtct
515701 agcagctggt gaaaagttaa ggaaaagtgc attcttattg gagttgttga tgactgctga
515761 taagatgcga gctagtaatg ttagtttcca caatacgtta gattatttag tattgagttt
515821 tgaacggtag gtgtttttga gtggatccat tttcacaatt gtcacaatta cagcaaaatc
515881 ttcaggcgat gacaaaaact gttgccggtc tggaaaatga catgcttgaa gtattgaaag
515941 aaaatacaga attaaaggtt gaaaatcaac tattaagaga aaaaattagt aaattagacg
516001 ccaacaaaga accagcagaa agtaagtccc aagctggtct aaaatcacta cgtaatattt
516061 acgattctgg ctatcatatt tgcaacatgt attatggctc tcaccgtgaa tctggtgaag
516121 attgcatgtt ttgcttagat attttagata atttcgtaaa tcacggacaa aaaaaatagg
516181 ggataaaaga tgcaagcgca aagtagtttt aatgaaaaag ataaggggcg tctttacctt
516241 gtccctacgc caattggcaa tttagaagat attactttaa gggcgaggag gattctaact

516301 gaagctgatt atattgctgc tgaagatact agaaccagcg gaatcttact tgaaaaaatt
516361 ggcgttcata ataggatgat ttcttttcat aagtataatt ctaaagaacg tgcgcgagaa
516421 ttaattaaat tgcttaaaga aggaaatacg attgccgaaa tttctgatgc tgggatgccg
516481 gttatttccg atcctggtta tattttggtc caagaatgta tcaaaaatga tatcccggtt
516541 gtttcgcttc caggaccttc agcttttgcg acagctttaa ttgcatctgg ctttgatgct
516601 caaccattta cctattatgg ctttttaccg cgtaagagta gtgagcaaaa agtatatttt
516661 gagatgatga atcaggcacg tgcgacttca attttctatg aagcccctca tcgtttaaag
516721 aagactttaa agaccttggc tgaagtaatt aaaccagata gaaaaattgt gttagctcgt
516781 gaactaacga agattcatga ggaatattta agaggtacaa ttagtgaaat taatgaatac
516841 ttcactaaga atgatcctcg aggcgagttc gtagttttgg tttcaccaaa tgatgagaaa
516901 gaaaagcaac tttcttggga tgagctaatc aagcaagtgg ctgatcgagt tgaagctggc
516961 gaaagtaaaa aggatgctat taagttagtc gctaaggcta ataaagtatc aaagaatgag
517021 ctctatgata aatatcatca aaattaattg cgtggtgatc gctaatggaa aagattttta
517081 aagaagaaca tcaggttact tatggcgact gtgatgaaac ggggaaaatt cagcttccta
517141 aattaattga acattttatg aaagtttcaa atgatcaatt aactcaaggc aatgctggga
517201 ttcatgattt attaaaacaa aacttaggct gggtggtagt tgaataccat cttgatcttg
517261 agcgtcttcc tgaagctggt gaaaaaatta ccgttacaac aaacggtagc ggttataatc
517321 gctttttga atatagagat tttggtataa tagactctac taataaaaag atagtagatg
517381 tcaaaagcca atgggtgatt ttagatctaa aaaatcgaaa aattactgaa gcagatagtc
517441 aaatgatgga aaagtttgga aatccatact tgaaacatat gccacgtttt aagagattac
517501 gtcctttgaa ggattatgaa tcgagtaaga catatgcggt tcgttactat gatttagata
517561 caaatcatca tttaactaat agtgtttatt ttgattggat gattgatact ttaccaagag
517621 aatttttgaa ttctcatact gctaaaagtg tcgatatttc atttaaaaaa gaagtacaat
517681 atggcgatca ggccgtatct gagcttacgc ttgatcaaga tatgcttacg tcgtatcatt
517741 tgatttctaa tcaaggtgaa atttctgctt tggctgaaat tagttggaga gaaaattaag
517801 agcatgactt gttagtacag tcgtgctttt ttggaaggta gggatgccaa tgaagacggg
517861 aataaagaaa atagatttgc aaggtttagt tactttggca ctatttgtgg cgatggatat

517921 tgtattagaa aaaatttctt ttggtccttc aactgtaaaa gtaggattgg gatttattgg
517981 aagtgcgctt ttaggatatt attttggccc actttggggt ggaatcggcg cattaattag
518041 tgatttattg cggtcagcta tttttggagt tgagggaggt ttcttccctg gctttactct
518101 gtcagctatt gttggcgtgg tgatctatgc cttattttta taccaaaagc caattaaatg
518161 gtggcgcgtt attgcagcga ctttattagt cactttagta gttaatgtat taatgaatac
518221 ttattggctt cacctactat atggcctaga cttacgagca gccttcttac aaagaattct
518281 caaggaatta attacgccat ggattcagat tgttgtactt tacgtgattt taaatgcttt
518341 tcaacgtgtt aaaataagaa ggtaatttcg atagtattaa ggatagagtt taaatgaaga
518401 tattaagtat cacgactgca accaatcatt tgagtgtagc tttgaatgat ggggataaaa
518461 ttgttgaaaa aaatgaagag gatcatcgca accatagcga gcatttagat cctttgatta
518521 ctgaattact aaaagaaaat aatcttacct taaaagatat tgatcgtttt gcagtggcag
518581 aagggccagg ttcttatacg ggattgagaa ttggaattac aactgctaag atgtttgctt
518641 ctattttaaa taaagattta gtcggagtat caactttggc tgctttagct aatggagtaa
518701 atgacggagt gattgtaagt gagttagatg ctcgtaacaa gaatttcttt gctggggttt
518761 atcgaaaagt aaatggtaag ctagagaact tagttctaga tggtcattat catttagacg
518821 atttaattaa taaggttaaa gaattgggtc tgcctgaaga agttgtcttt gtcggcagtc
518881 caattgataa atatcaagat gagattgtgg aattattagg gtcagacaac tttaagcaag
518941 ctgcaggcga taatcaaatt catgctgggg aaattggtaa gttagcactt aataaggagg
519001 caattgatcc tgataagatg gtgccaaaat acttaagacg tacacaagct gaaattgatt
519061 ggcataataa gactggaaag gcatttggtc cagatagcga ctatgttgaa gaagtttagt
519121 ttattttttc atccagcaga aattgacttg agtttttcgc cttttgcttt gagacttgat
519181 aatcaaactt atcaaattat gaaggcaagc gatgaaaata ttgctgactt gttgggctta
519241 gagcaagaag tttattttgg cagaacccca tggagtcgat tttcttttaa gagcgaattg
519301 aaaaagcatg ccaattcttt gtatcttgta gtttatcagg gttcaacttt ggtaggcttc
519361 gtcgggatga gaatgcaggc acaagaagga catattacta atattgctgt taagcctgct
519421 tttcaaaaaa gaggaattgg cggatttttg ctcaggacga tgattgaaat tgctcaaaga
519481 aatcatgctg ttcagatgac cttagaggtt cgaagcgata attacaatgc gcagagcttg

519541 taccggaaga ttggatttag ggataatttt attcggaaaa attattattc
ggctgaacat
519601 gcggacgccg taagcatgat taaaaagata gcagatagag aacaggaaga
agtaaattga
519661 caaaaaaaga tattcgaatt ttagcatttg aaagctcatg tgatgaaact
tctaccgccg
519721 taattaaaaa tgggcgcgaa attgaaagct taattgtagc tactcaaata
aaaagccacc
519781 aacgttttgg cggggtagtg ccagaagttg caagtcgtca ccatattgaa
gtaattactc
519841 aaattactaa ggaagcttta gctgaagcaa acgctacttg ggatgatatt
gatgcaattg
519901 ccgttaccta tggaccaggt ctagttggag ctttgttaat tggggttagt
gccgcaaagg
519961 cagcttcaat ggcaactggg attccattga ttggcgttga ccatatcatg
ggtcatatta
520021 tggcagcgca attaaaggat gaaattgaat atccagcact tgccttgcaa
gtatctggtg
520081 gtcatactga aattgtcttg atgaaagatc cgatccactt tgaaattgtg
ggtgatacaa
520141 gagatgacgc agctggtgaa gcttatgaca agattggtcg tgttttagga
gttaattatc
520201 cagcaggtaa gacaattgat gagtgggcac ataaaggaaa ggatactttc
cacttcccac
520261 gtgcaatgat ggaagatgat gactatgatt tttcattctc aggattaaag
agtgccttta
520321 tcaacacttg ccaccatgca gatcaaattc atgaaaagct tgataagtat
gatttagctg
520381 ctagttttca ggcttcagtt gttgatgtgt tgagccacaa gacaattaga
gcgatcaaag
520441 aatataagcc gaagacattt atcttaggcg gcggtgtcgc cgcgaaccac
ggcttacgcg
520501 atagattagc agaagagatt gaaaagttac ctgctgatat taagccaaaa
gtgattttac
520561 cagacttgaa actttgcgga gataatgcgg caatgattgg agcagctgca
tataacttgt
520621 ataaggctgg taagtttagc gatgagaatt tgaatgcaga tccatcactt
gagttgccat
520681 atgcagagag catgttgaag taaaaaaata gttatgaaga gagtcgaaca
gggatgtttg
520741 gctctttttt ggtagaatgt ttttattttc tagttttaaa taactgacac
attaacattt
520801 cattaaaaac aagcaatttc tccgttgctt gtttttttctt atcactaaca
tgtttccgca
520861 agcccttatt ctgccgtttt ataacaattg ttgttataat aaatacgaaa
gataaatagt
520921 cttgaggagg aattattatg aaagatgaag ttcaatctcc acaagaagaa
actcctaaca
520981 cttattggcg ttggttagta gaatcatggc gttatccttt aggtcacaat
cctgcagaga
521041 agtggtatgg agttgttacc ttattaggtg aagatattgt attattcatt
ggtttagcag
521101 tggcaggtaa ttatattgct aacctgtttg acatgccatt agtacatcat
ggatctggtg

521161 caactttctg ggcaatagta attttatttg ttgttcagat tattacaatt ctttgttcca
521221 tggttggtca cttattcgtg tttggtaaca caggtggctt ttggcagtat gtcaataagg
521281 ttgcacaagt aactaactgg aacttctgga ttgcaatttg tgcattcttt ttcttaattt
521341 tgagcttaag ctcaggcttg ctcggatttg gagaaggttt agtttggatt ttctacgcac
521401 tatttgcatt gggatcattt cttttagttg tctgcccaga tccaggaacc gaaaagatga
521461 ttcacgaccc attctgggga gcagttattt acttagtaat ggtagctgta gtttatgcca
521521 ttatttgggg catagcatta agtattttaa ttaacttgta gaaagttatt atttctatat
521581 aaaaaggtgt tgatcatctt cgcttattgc gttgaatgat cgacaccttt tgttttgaaa
521641 atatttatga attattcttc tgcatgttta actttaggca agttagatag cttgtgataa
521701 ttcatctgaa ttcttagagc tgcgtaaatt ggttttccac ctaagagatc taacacaaag
521761 taagcgataa aagtagtaat cagcattggg aagatttgtt caactgaacc taccatttct
521821 gttaaaagcg tcagcgcagt gaaaggagcc ttttcaatag taccaaagta agcacacatc
521881 gaagtgacaa taataatgcc atagcagcgc tttggcgcaa tattgaagtg aatcataagc
521941 acagcgaata ctacgccgag aagggctcct aagactagaa ttggcatgaa gataccgcca
522001 ggaacagatg ccccataaga aatcattgaa aaaataaacc gaacggcgaa ccaaacaagt
522061 ggtaatagta tcagacttaa ggctccagtt tgaatacttt tcatgaaagt tgggtcaaat
522121 agacttgaaa taaagtcgtg tgagccgcca agtaggttag cattccataa accaattgga
522181 attacgagca gaagcggaat aacactatga taaattgctg gaattttggt gatctttgca
522241 tagactggtt taaggcttaa gaggcagtat tgataaaggt atgcgagtag gcctaagact
522301 acaccaataa ttgctacaaa ccaataagaa gtaagtggca atgacttgtc taaaggaaga
522361 tataggcaag gctgcgtgcc aaagaagcaa agcgtcatta agtcagatga catggcagca
522421 gctaaggcag tcaggacttc tttgggagtg aagttaaagg ttatttcttc tactaggaag
522481 aagacaccag ctagcggtgc ggaaaatgcg gcgcttaatc cagcagctat tccgcatccc
522541 tggagtcttt taaggtcatc tggatcagca tggaagagat tttcaccaaa ggcctgacca
522601 accattgcgc ccatttgaat acaaggacct tctcgtccta agaatagacc gggacaaata
522661 gctagcaagc cacctacaaa cttgcgccag agaatgctcc accagttcat tttattttcg
522721 ttaagcatca cagcttcaac ttgcggcaca ccggaaccag taatattact tagaacgggt

522781 ttaataatct ttcccagaat tagaacgatt aacaaggttg caatgatgta
aggaactaag
522841 tagatcggtt tttgccgcat taggggatag ataaagaata agaacttcat
tgtgtggtcg
522901 ataatccacc taaaggtacc gacaattaat ccagttatga tcccgatgat
aattccctgt
522961 aagaagataa agaagaagtt tgatgtaaac ggcttagcta agacttgctt
ggcagtttta
523021 atcaattttt tcgccagctt tctaaaaagt acttgcttaa tttagtttac
atcaattaga
523081 agttaaacta ataaaaataa cgatagctgt gcgccatcgt tgtttttatc
agtattaatc
523141 tgttggtaac ttatatagtt gaggattttg cattaaataa agttgattgt
tgtcgaccat
523201 tagtccttta tgctctaagt tttcatacat ttcatagaaa tattgtggat
cttcttcata
523261 aaaataatcc ggtggaaaac atagaaaatt atagactttt gattctttaa
ataaattttc
523321 agctttttcg atcgacatat gatcatttgc agcaattaaa atactgactt
ccttagtaat
523381 tgcattaata aatgattcgg ttacgtattt atttttcttc ataaaggaca
cctccatatt
523441 ttaaacagtt aattgaattt tgagttttaa aggcccattg gtcagttaaa
ttttctggtt
523501 taagacgaga tatagctttc tcaaaagata ttgtaccatt agaatatagt
gccaatgctt
523561 cccaggaatt tgctccatca actacaggac ctataactag atcataatct
gtatttaaag
523621 caaaatcgat attctttcta ttttttatga tatagtttgg ccaatcttcg
cttacatgat
523681 caaatatttt gactcttaat gaatagaatt ggtttgtatc aaatacatat
ttagaaatta
523741 aaatgttgta cggattttgt tttaattttg aagaattaat ttttatttta
tgtttgctcc
523801 attttgctgc ttgattaata tctgtagtta agtaaaagcc tttaccgaag
tcatgtcttg
523861 gttttcccgc atgaatatct ggtttttcta caataacgtc actgccatgg
tacaaaagat
523921 ttggaatgga aattttcata ctaaatcaac tttctaaaaa tatatagaga
aaaagttagt
523981 ctctatataa ataagtacgc aaaaagaaga tgatttttttg tatgaaaatt
gaatgaatat
524041 aaaaaccgaa gtatctactt aatatgaata cttcggttct catgatttaa
attttattta
524101 tcgccatcaa aaatggaaga ttttacaata acataatcta ctactcgaat
tgcactcaag
524161 tctttaccgc cagcgtatga aatagaagac tgtaaatctt cctgcatttc
acgcaaggta
524221 tctttaatag agccacgata aggaactaac atttgctttc cttcaacatt
gcggtaggct
524281 ccttttttgaa cttcagaagc tgaaccccaa tattgcttat atctcttgcc
atcaattgta
524341 attaagttgc caggagattc ttcatgtcct gcaaatagag atccaatcat
taccattgaa

524401 gcgccaaagc gaacagactt ggcaatgtca ccattatgac gaataccacc gtcagcaatc
524461 aatggctttc tagctgcttt tgaacacatt ctaagtgctg ctaattgcca acctccggta
524521 ccaaaaccag tttttaactt agtaatacat gctctaccag gtcctacgcc gactttagtt
524581 gcatcagctc cggcattttc taattcacga actgcttcag gagttgcaat attgccagct
524641 gttaggaaag tatctggcaa cttatcttta atgtatttaa tcatcttaat tacataatct
524701 gaatgcccgt gagctacgtc gattgtaata tattcaggga tgatcttttc tttagccaag
524761 tagtcgataa aatcgtattc gctatcttta atcccaacag aaattgaagc aaataagcct
524821 ttatcatgca tcattttaat aaagtcagcg cgcttttctg gataaaaacg gtgcattaca
524881 tagtagtagc cgttttccgc taaccaaata gctaaatcat catcaattac gctctccatg
524941 tttgctggaa caaccgggat tttaaatgtt cggcttccaa attttacgct tgtatcggcc
525001 tctttacggc tcttgataat gcatttatta ggtacaagtt ggatgtcatc ataatcgaag
525061 gcttccatac taaagtaatt gctcatctaa tttacctcgt gctttttaaa aactacatga
525121 gttaatatac gggggttatg aggtaaagtc aagagtaaaa atgaatgttt attttattga
525181 ttattaaaaa tgttcggaaa ttaattgact attaagatat agattgttaa actagtcttc
525241 ggttttaaga aaatgatttt tatgaggtga aatcaaatga cagcaatagc agttgtaggt
525301 agtcaatggg gcgacgaagg aaagggtaaa attaccgatt tcttaagtaa agatgcagca
525361 atggctgtac gttcaaatgg tggtaacaat gcaggccaca ctattgaaat tggtgacaag
525421 actttcaaga tgcgcctaat tccatctgga atttttgctg ctaagaaggg tgctgtaatt
525481 ggtaatggtg tcgttatcaa tccagaagta atgttcggag aattagataa ccttgaaaaa
525541 gaaggtatcg atattagtgg cttgaagatt tctaatcgag cacatgtaat tatgccttac
525601 cacattttgc aagatactta tcaagaagaa gctaagggtg acaagaagat cggaactacc
525661 aagaacggta tcggtccttg ttacatggat aaggcttcaa gaattggtat tcgtgtttgc
525721 gatttacttg aaagagatac ttttgaagaa aaattacgta ctaatttagc tgaaaagaat
525781 gctttattta ctaaggttta taacaaacct gctcttaagt ttgaagatat ttttgaaaaa
525841 taccttgaat acggtcaaaa gatgaagaaa tacgttactg atacttcagt agtagttaat
525901 gatgcgttag ataagaatga aaaagtctta ttcgaaggtg cgcaaggtgt aatgcttgat
525961 attgatgaag gtacttaccc atacgtaact tcatcaaaca ctatttctgg cggaattgct

526021 tctggtatcg gaatgggagc taaccgtttg aatactgtaa ttggtgtctg caaggcttac
526081 actactcgtg ttggtgaggg tccattccca actgaattat tagatgaagt tggtgatcgc
526141 attcgtgaaa ctgctcatga atatggtact gttactggcc gtccacgtcg tgttggttgg
526201 tttgactcag tagctcttcg tcacgcaaag cgtgttgctg gtatcaatgc tttaagcttg
526261 aacttattag atgtattttc tggctttgac aagattaaga ttgctactgc ttatgaactt
526321 gatggtaaga agattgacta ctacccagca agcttgaaag aattatacag atgtaagcct
526381 gtttacgaag aattaccagc ttgggatgaa gatattacta atgtaaagac ttgggaagac
526441 ttaccagaaa acgctaagaa gttcttgaac cgcgtttctg aattagttgg tgttcctcta
526501 gttactgttt ctgttggtcc agatcgtgaa caaactattg ttttgaagaa tccttgggaa
526561 atgtaattta tgctagaacg ttatactcgt cccgaaatgg gcaaaatttg gtcattaaaa
526621 aataaatatg ctgcttggtt aaaggttgag attgcagcaa ctaatgcttg ggcagagctt
526681 ggtgaagttc ctgctgaaga tgctaagaaa attgctgata atgcttcttt tacagtagaa
526741 cgcgttagtg aattagaagc aattacgcac cacgatgttg ttgcttttac gagaacagtt
526801 tctgagagtt taggcgaaga aaagaagtgg gttcactttg gcttaacttc aaccgacgtt
526861 gtggatactg cacaaggcta tatcttaaaa caagctgacg aaattattct taaagattta
526921 gagaccttaa aagaaacaat tgcagatagt gctcgtaagt ataagtacac cgttgaaatg
526981 ggaagaactc atggtgttca agcagaacca actacttttg gcttgaagtt agctcgttgg
527041 tatgcagaaa ttaatcgtga cattgaacgc ttcaaggcag ctgctaaaga tgttgaatcc
527101 ggtaaaattt ctggagcggt tggtactttt gccaacgttc caccagcagt tgaaactagc
527161 gttttgaagc aactgggatt aactcagcaa ccagttacta gtcaagtttt gccaagagac
527221 ttacatgctt actacatcgc tacgattgct ttaattgcta caagtattga aaattgggca
527281 acagaaattc gcggtttgca acgtagtgaa attcatgaag ttgaagagca ctttagagct
527341 ggtcaaaaag gttcttctgc aatgcctcac aagcataatc caattggttc tgaaaactta
527401 tgcggtatgg ctcgagtaat gcgtggtttt atggtgccag cttatgaaga tgtagcttta
527461 tggcatgaga gagatatttc tcattcaagt gctgaacgtg tgattttacc tgatgcaaca
527521 attggaattg attacatgtt aaaccggttt aaccggattt tgactaacct tgatgttttc
527581 ccagaaacaa tgctcaagaa tatggaccgt acttatggtt tgatttattc acaacgggtg

527641 cttttgaagt taattgatga agcaggtttg tcacgtgaaa aagcatatga tatggtgcaa
527701 aaattggcta ataagtcatg gcaagaacag acttcattta gaaagttaat tgaagatagc
527761 gaagttatga actacttgtc tgaagctgat ttagatgatg cctttgatta tcattatcac
527821 ttgaagcatg tagatgagat tttttgaaaaa tgcggcttag attaggatag aaatagaaac
527881 gttttgttgg atacaattat gtctgacaga acgtttttta ttttggggag agatatttga
527941 tcatgaaaca tgaacattca gcaggtgcaa ttatctggcg caagaaaaat aacgaaatcc
528001 aatatttatt aattcaaagt cagccttata agcaattcaa gagtgcatgg gctttttcaa
528061 aaggacatct tgaagctgga gagacagcgc aagaagctgc aaaacgagaa atttttgaag
528121 aagtaggttt gaaggctgaa tttaattttg attttagtga aagctattca tatcaagtta
528181 cttcagaaat tgaaaagact gtaactttat ttttagcaaa atacaatcct gaccagaaaa
528241 taaaacggca agaaaagtgaa attaggcaaa ctgcttggct aaactatgaa gatgcgcaaa
528301 aaaggattag agagcaaaac tttaaggaat tcagttttga agatctgtct tctattttgg
528361 aaaaggctaa tgattatttg aatggacatt gattttgaga atagagcctg atgagattct
528421 ttataattca agtaagatta tatctagtta aaattaaata caatgtatat aaattgtagt
528481 ataattttag aaaagggagt gattaatatg gcacatacat atagttatcg tgcagattct
528541 aaagatcatg atgaagttaa aagaatttta gataatctag gtttagatat gtctactagt
528601 attaaaatgt attttaaaca aattattaga cataatggaa ttccctttttc agtaactaat
528661 tctgatacgc ttacagaaga tactaagaaa gctcttttac ttgcagaagc taaggatatg
528721 ggcttaattg aagacgatac tccagccttt aaagatacta ataaattgat tagttatatg
528781 aaaaaacgtg caaaggaatt agagtaatgt atgaaataaa agccgaagat acttttattc
528841 aggatgtaaa tcgttggagt aagaaagttc ctgagttatg ggatgaaatt caagcaataa
528901 cttcatatat gcaagaaact ggagaaatac cggaagaata tgatccacat cttttaacta
528961 atgaagagct gaattatgta ggatactttg agtttcattt atttgagggg aaacttgact
529021 tgcttgttat tcatacaaaa aacagaaata aaaaagtctt tcgattggtt agactagggt
529081 ctcataatga attatttcat tctgatttac gataattaaa attaaagtcg ttgctatcaa
529141 acggcttttt attttgctca tttttatcta aaataagact tgaatacagt taaaacaaga
529201 aagtgtatat aaaaatgtca gaaccaaatt atgcaaagct agaagcaaat aatcttcatt

529261 tagacaaaga gaagccatat ccactagctg ggatcttagt tgtggatttc acccatgtct
529321 tgtcaggtcc aacttgtaca agaatgcttg ccgacgcagg tgcacgagta atccacattg
529381 agagaaaaac aggggatgat acgcgtcaca tgggaccata cattgcggat ggttcaagcg
529441 aatatttcag aatctgcaat gcaggaaaag aatcaattgc cttagatctg aaagatccaa
529501 aagaccatgc cttagctgaa aaaatgattg ccaaggcaga tgtagttgta gaaaacttcc
529561 gtccaggtgt tatggcgcgt ttaggttttg atccagaaga aatggtaaaa aagtatccta
529621 aattgatttt tgcatcagta tctggctttg gacaatacgg accttggtca aagcaagctg
529681 cgtacgacac aattattcaa gctgtttcgg gattaatgga tgctacaggt gaaccgaacg
529741 gtaaaccaac tcgtgttgga acttcagttt ctgatgttgt tgcaggaatc atgggttata
529801 gcgctattac caccgcctta gttgcaagag agcgcactgg aaaaggaacc acaatagatg
529861 tttcgatgct agattccaca ttttcattaa tggtgcagga cttgatgctg gcacttgggc
529921 cacatgaagt accacacaga attggtaatc gtcaccctga tatgtatcca tttgatacat
529981 ttgattgtca agaccagcca attgctattt gttgtggtaa tgatcattta tggggattat
530041 tatctcatgc gttaggccat gaagaatgga tgaatcaagc agattttaag acaaatgatt
530101 tgcgtgagaa aaactggcaa aaagttaaaa atgccatgca agatgtactt aaaaccaagc
530161 cggctgctga gtgggacaag atcttacacg aagcaggtgt gccagctggt ttagtgctca
530221 atgtggataa gaccagacgc cttgatcaaa ttattgagcg cgggatggtt aagactttgc
530281 ccgatggcaa tgaagtttta ggttcaccac tcaagtatgg aacttggaat tcttatggat
530341 tgcaaaaaga tgcaccaaaa ctagatgaac aaggtgagca gattcgtaag gaatttgaat
530401 aaaattagac atgaaaaaaa gatcaagatt ttctattttt tgagaatctt gatctttttt
530461 atgattaaaa atttattctt ttagttggtt aaacgcctct tctggcgtgc aattgtaaac
530521 ctttttgaat gcattataaa gatttcgata gcatttaaat cctgattttt ctgcaatttt
530581 ttctatattt aaattgcttt gcaaactgag tcgaagtgat tgctcaagtc taagtgatgt
530641 taaataatca gaaaaagagt ggtccaaatt ttttctgatt aatcgagaac aataagctac
530701 tgaatagcca aaatgatcag caaccatgtg taaactgata ttttctgtgt agtgttttga
530761 aacgaagtca ataaattcat tgagaaaagg aatattttta ttagtggagt aatgtgtcac
530821 aactaaatat ttctttatga gttcttgtag aatctgtagg cctaagatat aaatagataa

530881 attaatagaa atgtctttat cttgtgtttc aagagctttt actaaattta tgatatttgc
530941 tgacaaggtt aaagatgaaa tggaattaag attggaatta aattgaaaaa attgctgaaa
531001 ttttccatta gtaactaaat ttagaaatgt gaatggatat tgtatacaaa gaatgtggct
531061 atttttaagc gcattagttg aatgaattac acatgggttg ataataataa attgattttg
531121 attaatttct tggagctttc cattgatagt ggtgttcatt ttaccactta gaacaaaaag
531181 aatttcagtg ctttcatgcc aatgtgatgg aatgattctt gatgaatctc tggagcgaaa
531241 agagaaagct ttaaatggtg caattgactt atttaaaatt tgttcgtgag ttattttcat
531301 aatataaaaa ttgctccttt cttttatcat gtgattgttt ttagtgtaga tgtaaatatt
531361 ttacatataa gtagtaacat tctggtattt agtgtaagcg attaattatc taaaatctat
531421 agtgaaaagg ataaataaat atatcgagga taaaaatgaa acaagagaaa atatttacat
531481 tattaaatga aatgtcacta gatgaaaaaa ttggtcaatt aattcagcta tctggagaat
531541 tcttttcagc ttctgatatt tctattggtc cgattgaaaa attgggtatt tctaaaaaga
531601 tggttaattt atgtggatca gcattaaatg ttgtaggtgc ggaaaatgtt cataaagtac
531661 aagatcaaca aatgcagaag cagccacatc acataccgat gctttttatg tcggatgtta
531721 tttatggttt taaaactatc tttccaattc ctttgggatt aggaagttca tggaatccaa
531781 aattagtaaa aagagcatat tctgttgccg ccgaagaagc atcagcatcg ggtaatcaag
531841 tagcatttgc acccatggta gatgttgtac atgatgcgag atggggtaga gttttagagt
531901 caccaggtga agatccatat ttaaattctg tttactcaaa aaatatggtt gaaggtttac
531961 aagaaagcct tgcaaataaa acagggcaag tatcttgtat aaagcattat gctgcatatg
532021 gagccgtaga ggccggacga gaatacaatc aagttgatat gtctttaagt aatttatatc
532081 aaaacttttt accaccttat aaagctggcg tgaaagctgg ggcaggaatg gtcatgactg
532141 cattgacaac tttaaatgga gtaccttcta cagcagatag gtggttgttg tcagatgtac
532201 tacgtgaaaa atggaagttt aacggtatta taatttcgga ttatgcatct atttatgaat
532261 taattaagca tggttttgct agagattcaa ctgatgctgc tttaaaagct attgatgcag
532321 gcgtagatat tgatatgaaa tctccgtgtt acgcaaatgg actgcatgag ttggtaacga
532381 atggagcatt agatgaaaga aaaataaata acgcagttct caaggtatta aatctcaaaa
532441 atcaattagg actatttgaa aacccatact ttggtgcttc tgttgaaaaa gaaaagaata

532501 gtatacttac tcctcaaaag cgcaaattag cgcgaaaatt ggctcaagaa
tcattagttt
532561 taattcaaaa taagaataat atattacctt tgaagaagga tacaaaagtt
gctttaattg
532621 gtccttatgc caatagtcaa aaattaattg gaatgtgggc aattcatgga
aattccaaag
532681 atactgtaac gatagctgat ggattaaggc aatatactaa taagttaact
gtaaatcatg
532741 gaacagactt gtgtcgtgat aaagcactat taaagaagct aggatttttg
acagaaaaag
532801 atattaataa aattgtatct tcaccagaaa aagaaaggct gaatattcaa
aatgcaatta
532861 atgctgcaaa agcagctgat actatagttt taacattagg cgaagaaaca
tatgaagcag
532921 gagaagctgg tgctaagaca aacttaaata ttgcgcccaa tcaattgaaa
ttattagata
532981 aattatccca attaaataaa ccaatcattt tagtacttat tagtggaaga
ccattagtac
533041 taacaaaagt taaagataag gccgatgcaa tactggaaag ttggtttcct
ggtactgaag
533101 gtggagatgc aattgcagat atcctatttg gtaaggtaaa tccttcagga
agattaacta
533161 tgagctttcc gtatgcttca gctcaagagc ccttatatta caatcatctg
tgtacaggaa
533221 gaccagagaa taactctcaa catgttggta gatttgtatc taagtatatt
gatgcaccag
533281 ctaaagcttt atatccattt ggttacggac tttcgtatgg aaaaataaag
tattcaaatt
533341 taactatttc ttctaagcga ttatttctgg ataagcctat aaaaatttca
atggatattg
533401 aaaatttaag tgattatgat cgaaaagaaa ctatccagtt atatatccat
caagaaacag
533461 ctagtatcgt tcagccagtt aagcgattga tagattttac caagattgcc
atatcaggtc
533521 attcaagtca acatgtagaa ttttatataa atgcaagtca attgtcattt
tatgacaaca
533581 ttggtaggtc acataaagaa agcagcaaaa tacaggttta cgttggtacc
aatggagtac
533641 agctattaaa aggtaaattt gattttataa ataagaggtc ataattttac
atattagatg
533701 taacagaaat gtatttaatc gtatctgata agcgcttaca ataattggcg
taagggatat
533761 aaaagaaatg gagaaagaaa atgtctagta aagaaaacaa aaagatagct
gaggaggtta
533821 tagccgccat tggcggaaaa gaaaatatcg tttctttagc ccactgtgct
acaagattaa
533881 gaatagttgt aaaagatcgt aaaaaggtaa atgaaaaagc actagataaa
attgataagg
533941 caaaaggata cttctttacg gctggtcaat atcagattat ttttggtact
ggcttagtaa
534001 atgatgttta tgaagcggta aaagaagaag gagtttctac aacatctaaa
gatgatctga
534061 atgatgtagc tgcaaaacaa tcaggatggt ttcaaagatc aataagaatg
tttgcagatg

534121 tctttgtacc aattatccct gctttggttg caacaggtct ttttatgggg ttgcaaggtt
534181 tattaacaca accagctctt ttaaagatga tgggaatgtc agctaaatca attccggttc
534241 aatttactac ttactttaat attttaactg gaacggcttt tagcttttta ccagttttaa
534301 tttgttggtc agcatttaga gtttttggtg gtagtccaat tttgggtatt ttacttggtt
534361 taatgctagt taatcctgct ttaccatcag catgggatgt tgcccaaggg aaagttgctc
534421 cactaatgtt ttttggtttt attccagttg ctggctatca agcttcagtt ttaccagctt
534481 ttattttagg atttattgga gcgaaaactg aaaagtggat tcataaacat gttatcagtt
534541 ctcttgattt aattgttact ccatttgcta cattattaat tggaagtatt ttaggactac
534601 tttgcgttgg cccaatcttc agaattgttg aacaaggaat tttacatgca gcagaatgga
534661 tagttcaatt accatttgga ttaggcggac taatctgggg tgctgtttgt cagatgattg
534721 tggttactgg attacaccat gcactaaact tagtagaaat ccagctatta gcaaatactc
534781 actggaatcc tgtaaatcca attggatctg ctgctattgc tgcacaagcc ggagctgcct
534841 tggctgtagc tttacgaatt cattcaaaca aaatgaaaca aattgcatat ccatcaacaa
534901 tttcagcttt actcggtatc actgagccag caatttatgg tgtaaatatt aaatatatca
534961 aaccattctt atttggttgt attggtggtg gtatcggtgg atttctttca agtattttaa
535021 atttgaaagc tactggtatg tcaataactg ttatcccagg tatattatta tttttgaatc
535081 aacaacttcc actatatatt cttgtttgtg tagttggttt tggagtggca tttggtttaa
535141 cttacgttta ttacggtaaa catgcttttg atagtaataa aaatagcaag aaagaagata
535201 tagagaaaaa acagaatact tttacgcaag agcctagtca tgtatcagac gaagtaatag
535261 atgctcctgt tagtggtaaa attgaagctt tgaatttagt ttctgataaa gcctttgctt
535321 caggaatgat gggaaaggga atagctattg aaccatcatc aaataatata gtttctcctg
535381 ttaatggaac gattactgtg gcttgtccaa caggtcatgc atatggaatt aaatcagatg
535441 atggggcaga aattctcatt catctaggta tagatacagt aaatctaaaa ggaaaaggct
535501 ttaacactgt agtaaagcag ggccaaaaga taaataaagg tgaattgctt ggaacgtatg
535561 attataaaga aataggcaag gagggttatg acaatatagt tatagttttg attaccaatt
535621 cgaaggatta tgcagaagtt gatccaattg cttctggaaa tgttaaagca caacaaaaat
535681 taattgcctt aactgaacct acaatgactg agcaagtaaa aggtactgta aatgaattga

535741 attaataagc tttaagtgta agaaagctaa gtgtatcaat acatccccct ctttttaggt
535801 acaattagct ttcttttttt atcaaaatga gtgtgttaaa aatgactaat agaagagaac
535861 agtttgaaca cttagtttca aatctaaagg atattcctaa ggaaaactta atcaaagaaa
535921 aaaacagtgt caaaaaatca ccgtatcgtc aaagaattca tattgagagt ttatttggca
535981 tgttgggtga tccaaatggt tttcatatt ttaatggata ctatcattta tttcaccaat
536041 ggtttccgat gaaatatagt acgaatccga attattttca acagggatgg tttcattgga
536101 aatcaagaaa tttggttgat tggataccag ttggcgaagc aatgaacaat gatactgtat
536161 ttgataagta tggagtatat tcgggtagtg ctattcctat caataataaa ttgtttctta
536221 tgtacaatgg taattcttgg accaatactg atacagatga ttggcataga gttccgtgtc
536281 aattaggagc ctatatgaat gaaaatgata atgtagctaa attggcgaat ccattaatta
536341 aaggaccaat ttctggttat accagtcatt ttcgagatcc taaaattttt aagaaaaatg
536401 gtaaatatta ttcaataatc ggtgcacaaa caaaagcaaa aactggtact gtcttaattt
536461 atgaaagtca gaatttaagg atttggaaca aagttggtga agttaaaact aattttgaaa
536521 aaactggcta tatgtgggaa tgtccagatt attttgaatt agataataag ggaatattgc
536581 ttttctgtcc tcaaggattg aagagtaaag gaagtcaatt tttaaataca tttcaagcat
536641 gctacgccat tggaaataag ttagatttta gaaacttata ttttgaaggg aaaaagttcg
536701 aagaaataga ttctggtttt gatttttatg ctcctcaaac aatgatcgag cctgatggaa
536761 gacgtatttt aagcgcatgg atgagtatta tgaattcaaa ttctccacta attaaatatc
536821 attatgatgg atgtttaatt ttcccgagag aattatcagt acaaaatgat aaactcattc
536881 aaaaaccagt aagcgagata aaaaaactgt atacaactga ttacaagggt aagagagcaa
536941 tcagtaaaaa tcaggagatt gtcgctggtt tagttaattg tagagatatt aaatttgaca
537001 tttctactaa agataatagt gctataagta taatagactt gtttgctaat agagaaaata
537061 cgagtcattt gcgtttagtc ttaaatagga tgaaaaatag atttatagtg caaagaggta
537121 aagctgggat agaatttgaa gacgagtttg gaaatgaaag aagttgtgag ctgaaagttg
537181 caaattctgt cagcatacga ataattcaag atatatcatc ggcagagata tttattaaca
537241 atggagaaaa tgtttttaca atgcgagtat ttgttcctaa agatcaatat catatttttg
537301 tgaaaaatgt taatggggaa gtaaatgtta agtatcaaat tcatcaatta agaaaaatga

537361 gcagtgaata attttataaa agtagacatg aaaaaagatc aagatttcca gaaaatagaa
537421 aatcttgatc ttttacttta ttcaaattca tctagtgcat ccaaggcatt ttcccagtca
537481 ttatttgcct ggtctaattg cttttgaaca gcagataatt tttcttgcaa tggtccaagc
537541 ttttcaaatg aagcggcaat ctctggattt gccatctcag tttgaatttc tttttcttct
537601 gcttctaatt tttcaatctt agcttcagca tcgctaactg cacgttctaa ttttctcttt
537661 tgagaatcgc gtgctttttg ttcttgataa gatagtttac ctttattttg actggcagcc
537721 ggagtggttt cagtttctgc tttttgtgct tctgcttctt gagcagctgc agcttgctta
537781 gccttttcat ctaggtagta agaatagtta ccattataga tcttagcgtg gccatcgcga
537841 acagaaataa tcttgttagc taactcatta atgaagtaac ggtcgtgaga aacgaagagc
537901 aatgttccat cgtaattatc tagtgcttct tcaagaactt ctttagcttc aatatctaag
537961 tggttggtag gttcgtccat tagtaagaag ttatcttttt caagagacaa aacagttaga
538021 gtcagccgcg ctttttgacc accagataat tgaccaactg tcttatcaat atcttcggca
538081 gtgaacaaga aggaagccaa aatagatcgg acgtcttttt caggcattgt cttgtgacga
538141 tcccaaattg tatcaagaac agtttttgaa ggatctaaac tttggagttc ttgatcatag
538201 taaccaatat ctaaacttgc accatattta attgaaccac cttttggttc aagctttttc
538261 atgatggtct tgagcaaggt agacttacca ataccgttag gtccgataat agcaactcgg
538321 tcattcttat taacttggaa gtcgatatcg gaaaccatta ccttatctgg atagccaatg
538381 gttagatctt tagcgatcaa cacttcctta ccagaaggac gctcactagt gaagttaata
538441 cgaacttttt gtttgtgctt aggtggcttg atacgctcca tcttgtccaa aactttacgc
538501 cggctttggg cgcgtttagt agtagaagca cgaactaggt tcttttgaat gaactcttct
538561 tcctttttaa tcttttcttg ctgcttttca taagcggctt cttgctggct atccatcaat
538621 tcacgttctt taacgtattg actgtaatta cccttgaaag tagtcagctt tccaaagttc
538681 agttcaaaaa tttgattagc taagtgatct aagaagtact ggtcgtgaga aacagtaata
538741 attgcgcctt gataattctt taggaacgct tctaaccaat ctaaagtatc taagtcaaga
538801 tagttagtag gttcgtcaag cagcagtact ggtggctttt gtaaaagcaa tttaacaaag
538861 gcaagtcgtg tcttttcacc gccagaaagg gtgccaatta ccttattcca agtattttct
538921 ttaaaattaa aaccgttaag aatactctta atttcagcct ggtaggtaaa gccgccttct

538981 tgttcgaagt cgtaagctaa ttgatcatac cgcttgagca agtcttcatc ttcgggatgg
539041 tcggcaattt gctcttgcat tttagtgatt tttttatttt tttcgattaa gtcattaaag
539101 acagtaagca tttcatccca gattgttttg tcttcatcga ggccattttc ctgagcgatg
539161 tagccgactt ttaatccctt attaatagta aattgaccac tagttggttc ttgttgacca
539221 gtcatgatct ttaataaggt agtttttccg gctccattag gtccaacaag accgattctg
539281 gcattgtcag gaaccgagaa agaaatgtta ctgaagataa tattgcctcc aaaacgttgt
539341 tcgaggtctt gagcttgtgc aataatcata attattttct cctagtgctt attttaacat
539401 agtgaatctt ttaaatttta tgaaaatatg acctttgtga aaattgtttc ttgatttatc
539461 cttaaagaat gcctattatt agtttgatta gtaaatattt cacattcgta tttataaaag
539521 aaagaggcct cttaatggat gaaattaaga ttcctaaggc tactgccaga cgcttgccgc
539581 tatattatcg ctatttaatc tttttaaatg atgaaggaaa agaaaaagtg tcctcaactg
539641 aacttgcgga agcagttcaa gttgacagtg cttcaattcg tcgtgatttt tcttactttg
539701 gtgccttagg taagcgtggc tatggttatg atgttaagaa cttgcttaat ttctttaaaa
539761 agattttgaa ccaagatact ttaactaacg ttgccttagt tggtgttggt aatatgggac
539821 atgcgctttt gaattataac ttcaagcgta ctaataatat cagaattagc gctgcttttg
539881 acattaatcc tgaaattact ggaacaatta tgtcaggtgt gcctgtttat gatatgagtg
539941 aaatgaagaa gcagcttcgt gagcaacaaa ttacgattgc aatcctttgt gtaccgcaaa
540001 cagcagcgca aaagactgcc aatgaaatgt ttgatgctgg gattaaaggg atcatgaact
540061 ttacgccact tcgtttatct gcaccttcta gtgttcgtgt acaaaatgtt gacttggcaa
540121 ctgaacttca aactttaatc tacttcttgg attcagataa agataagaac aaaaaataat
540181 ttactattaa tatagctttt gcacaagctt aactaggctt gtgcattttt tatacatttt
540241 tatttgtaat cagagagaaa ataattttaa gataaatagg catagaattt ttaaatttag
540301 aaataaggta gataaaatga ttaaaaagat ggcagtttat tgtggtgcac gctcaggtaa
540361 ccgccttgaa tatgctaaag ctgcttatga atttggtaaa aaaatggcag accaccagat
540421 tgagctagtc tatggcggcg gtaagtatgg tttaatgcga gcagttgctg acggcgtttt
540481 agaaaatggc ggtattgttc atggcattat tactgaagaa ttaaaagatc gtggcgcagc
540541 ttacgataag gtgcaagatt ttcgcgtggt cccaagtatg gataagcgaa aagatacgat

540601 gatgaacttg gctgatggga tggtagctct tcctggcagt attggtactt tagaagaaat
540661 aagtcaagct atttcttgga ctgcaattgg cgataatgct aagcccgttg ctttttataa
540721 ctatgctggc ttttatgatt atttagataa gctattaaag cgcatgaatc aggatgattt
540781 tttagaaaat atttacttag atacggtcta ttttgaaact gattttgata agattttgca
540841 gtttatggag gattatcagg ctccagctta tcgtgaatat tgatttatta aactaaagaa
540901 gtatagccta aattttcccc tcgtatttag gttcatgctt cttttttga ccacttagaa
540961 ctttaaaatg accgcttagt caatttcact agattttaat cttatattca tgtttaatat
541021 aaatatcaag aacgaaacaa ggaagtgaga attgtgaaag ttaagcttga acttgatcca
541081 gatcagcaag agacagaaat aacgattcat gctaaaagct taacgcctga agtagaaaga
541141 atttatcatc aacttcaaac tgattcagaa catcctgatc aaattgaggg gatgatggat
541201 aatacttctt attatttaag tattaacaat attctctttt ttgaaacaga cgctaagcag
541261 gtaatggcgc atacgacgag gaatgcatat tttgttaagt ataaattata cgaactagaa
541321 aacttattaa gtggacagtt catgcgagta tcgaaatcga ctattttaaa tttagatcag
541381 atctacgctg ttactaagtc gatttctaat tgtcagatta agtttcatga ttcctataag
541441 acagtctatg tttcaagaag atattaccga gatttaaatg aaagattaaa agagagaagg
541501 gcattatcat gagaaaggcc agttttggaa gaattctttg ggggttaggt ttgcttgcgg
541561 cagctgcgtt tttagtttta gatcagctcc acttgatgcc attagaatta ggcttttggg
541621 caattttttg gacagtagtc tttggagcaa ccttaattac ttctttaatt aataaaagtt
541681 taggcggcag catcttttca attgctttcc tattaattat ttatgctaaa ccacttcata
541741 ttgaacgtat tgttccttgg acagtacttt tagcagcgtt cttaatttgg ggaggtttga
541801 gattaatctt caaaaaaagc tggaagccga ctgtgattat taatggtgaa aaagttaacg
541861 ctaattggtc agacttgaag gcaccacata aatttaaggc tgagcatgta ttttctgata
541921 cgtctagttc agataatgat gacaatattg tgattagcga aaagttgtct tctacttcac
541981 gttatgttca ttcgcaacac ttagaaacgg taactgtgaa tgtttcaatg ggagatgtta
542041 atgtatatct tgattcagct aagcctgctg ggatgaagt acttgctaac atcaatatgt
542101 cgatgggaga tataaatatt tacattccaa cttcttggcg agttgatgat caacttgaac
542161 ataaatttgg tgatttcact attgatggtg atcaaccagc agaaggtcca acattagtac

542221 ttcaaggtcg tgctaatatg ggggatgtta ctattaagag agtttagaac tagaaaagtc
542281 gatcagctct aatttgaagt aaaaattaat ttgctgtaaa atacttgtag taaagaaata
542341 cttgtagtat aaaaaagaga gctactgaga atagctctct gaagcacaag ccgctaaaag
542401 tgcggtggca aaattattat tagcaaacta aaaagtcgct atcaaactcg gccaaaagtt
542461 atggagcggc tttttttgtt tgcttatttt ttcttccaca aatcgtggat agcctttatc
542521 agtcgttttg cagcttttgc taactcggtt aaatcttttg caagttttag ttttattaaa
542581 acaagaacaa gacaatccat attaaaattc aaacaaagat tcaagtaaat tagcactttt
542641 tagcactcag acaataaaag tgctaatttt tgtgtctaaa tacttgcttt tcttttataa
542701 acaaggtact atcgtaaatg taagttagca cttatgagat atgagtgcta agtgatgtaa
542761 taatagatta aatatgtaca ggagggacta acgtgttaca accaattggt gatcgcgtga
542821 tcgttaaagt aaaagacgaa gaagaagaaa aagttggagg cattgtcctg gcttctaacg
542881 ctaaagaaaa gcctcaaatg ggcgaaatta ttgccgtagg aaatggtaag cgtaacgcaa
542941 atggcgactt aattcctatg tcagttgcaa aaggtgaaac agtattcttt gataaatact
543001 ctggcactaa cttgaaatac gaaggcgaaa agtacttagt acttcgtgaa agcgacttat
543061 tagctgtcgt taagtaatta aattagaaat aaaaggtggc atataaatat ggctaaagac
543121 attaaatttt ctgaaaatgc aagacactca ttattaaagg gtgttgataa attagcagac
543181 actgttaaga caactttagg acctaagggt agaaatgttg ttttggaaaa gagctatggt
543241 gctcctgaca ttactaatga tggtgttact atcgctaaga gtattgactt agaagatcac
543301 tttgaaaaca tgggtgcaaa gcttgtttct gaagctgcac aaaagactaa cgatattgct
543361 ggtgacggta ctactactgc tactgtttta actcaagcaa ttgttcgtga aggtatgaag
543421 aacgttactg ctggtgctaa cccagttggt attcgtcgcg gtattgaaac tgctactaag
543481 gcagctgttg acgaattgca caagatcagc cacaaggtaa gcaccaagga tgaaattgct
543541 caagttgctt ctgtttcatc agcttcaact gaagttggta acttaattgc tgacgcaatg
543601 gaaaaagttg gtcatgatgg tgttatcaca attgaagaat caaagggtat tgatactgaa
543661 ctttcagtag ttgaaggtat gcaattcgat cgtggttact tatcacaata catggtaact
543721 gataacgata agatggaagc agacttagac aacccttaca ttttaattac tgacaagaag
543781 atttctaata ttcaagacat cttgccatta cttcaagaaa ttgttcaaca aggtaagagc

543841 ttattaatca ttgctgatga tgttgatggt gaagctcttc caactcttgt tttgaacaag
543901 atccgtggta ctttcaatgt tgttgctgtt aaggctcctg gctttggcga tcgtcgtaag
543961 gcaatgcttg aagatatcgc tattttaact ggtggtactg taatttcttc agacttaggt
544021 cttgaattaa aggacactaa gattgatcaa ttaggtaagg ctggcaaggt cactgtaact
544081 aaggattcaa ctactattgt tgaaggtgct ggctcaaagg atgctattag tgaacgtgtt
544141 gaccaaatta agaagcaaat tgctgacact acttcagact ttgaccgtga gaagttacaa
544201 gaacgtcttg ctaagcttgc tggtggtgtt gctgttatca aggttggtgc tgctactgaa
544261 actgaattaa aggaaagaaa gtacagaatc gaagatgcct tgaacgcaac ccgtgccgct
544321 gttgaagaag gttacgttgc tggtggtggt actgcattag ttgatgttat gaagtccatc
544381 caaggtaatg ttaagggcga tagccaagac gcacaaactg gtgtaaacat cgttatgaaa
544441 gctttaggtg ctcctgtacg tcaaattgct gaaaatgctg gtaaagacgg tgctgttatc
544501 ttagatcact tagaacatga agacccagaa attggttaca atgctgcaac tgacaagtgg
544561 gaaaacatgg ttaaggctgg tatcatcgac ccaactaagg taactcgttc agcccttcaa
544621 aatgctgctt caattgctgc cttgttgtta actactgaag ctgttgttgc tgatgctcca
544681 gaagatgaca agaaccaagc tcctgcagct ccaaacccag gtatgggaat gggaatgtaa
544741 tttaattcct aacaaaaaat aagaaagaat tatcattatg acactattag tttactgata
544801 ggcataatgt aaaagaatcg agcaatcgct gatggttgct cgattctttt tttatgggct
544861 ttgtaagatt aagggatatt tctttaggtt gtacggcaat aaatataaaa agtgaaatcg
544921 aattcaatat tagtatagaa tttataaaaa taagtgttat tatatagttg tcgattgaaa
544981 acgttattta attaaggaaa taaccattta ggtctattgg aggtaatttg tatgagtagt
545041 aagatgtttg gagataaaca caaccattat agtattagaa aactttccgt tggggcagca
545101 tctgttttaa ttggtgccac aatttacttg ggaggggtaa acgcccaagc tgtacgcgct
545161 gatagtgtta atgaggatgc tactgaacaa gtagagaaga aggatgaagc taatgttaag
545221 gcagcagaag taaagactac tgaacaaaag caagaaaata ataagacatc ggtttctgct
545281 actaatgaaa atgctaagca aaatgttgct gaaactacat cagatagcaa aaaggttgca
545341 tcaaatgggg atgttaatgt tattaaaaac gatgtaacta ctgatgaaga agcagctact
545401 aagtctagtg ttcaagcaga taaagatgtt aatgcaaata agttggacac taatactgtt

545461 tcagtgaata agcttcaaag aagtagcaat gttgctggac ttactgagag taaggctgct
545521 gatgagaata ttgaacaaaa agctgaggag agtccacttg gtacacgttt tatgaaccat
545581 acaaatgact ttcctggtga tgctacagca atacgtacag taactttaaa taaaccacaa
545641 tctgctggtg gtagtgaaac tattacgcaa acgttgggcg catttggctt ttctgaagta
545701 ataaatgaaa gtaatacatc aaacacaact aaagtaaagc ttgattttcc tttgacacca
545761 acccttaatg gtaaacctgt gttgagtagt actcgccatt tttcaacgaa tttagttttt
545821 tctaataaca aatggacatt tgcatctgca gaagatgaag aaaattataa taaatttcta
545881 gcttttcaac catctgatta taaagcaact ttaccagcta ttgaagatag tcaaattgct
545941 gtagatggat atacttggaa ggttacaagt gctactccag atggcgcagg acttggtgct
546001 gaaacattat caccatatgc tggtatttca caaaatatcg ttattgatta tacaaaagaa
546061 gaacacactg taacctacaa atttgaagat ccatctggta atcaagtagg tagttcagta
546121 ccagtaaccg gtgcagctgg tagcaatcaa ccagttagct taactcttcc agctggttac
546181 caacttgtat caggtagcct tccaactagt gttactatcc ctactaatag tgataaaact
546241 atttcgcttc ctgtaaaata tcaacttaca atcactcttt taggtgacaa tactatcaat
546301 tatgcaggcc ctaactggca aagtatagct aagaataagg ataatgtttc cgctagtggt
546361 tatagagtca agtttaatga tgatgaagct ggtgtcaact gactaatgga gatttaacat
546421 atgatggaaa tggaaatatc gggacttatc atgttagtct aactgaaaag ggtcttaatg
546481 acattaagga gcaattaggg gataatttca tttatccaga ttttaaagat gtaacaaata
546541 aggctacttt aactatagat aaaggaaata aagaaattga gcttaatggt ttagatacaa
546601 aggtatttga taatacttct actcttccag atcaggatac tttctattca ggattaagtc
546661 ttggtaatga tgatgtgggt aggatatcaa tttataagca tgacggaact attaccacaa
546721 ttcaattagc ccctaatgat gtagaatttt ggctcaatgg acagaaagtt gctaaagacc
546781 aggctaagaa tgttggagaa tatgatcttc gtttaactga tgactttatt aataaagtta
546841 agtctgccga tggtaataat ggaaacaact acgagtggac ttatggaact catactccta
546901 gtggagatac tacttacact gctatatata agatctatca agcaaccggt aaggcaaagt
546961 taagtggtaa caattctaaa ctttatgatg gcaatgctgt aactactgct gacgtcaata
547021 aggatggaaa gattactatt gatttaactt taccagtcta taacccagat gatgagtctc

547081 aattattgag aaccgttcct ttaggtaagt acacactcca agatggtgat
tatacatgga
547141 atactgcaga tggttctgca ccaactaaag gtggttccta cacaattagt
ttgaataagg
547201 ataagatttt atctcatttg caagatcgtt taaagacttt agcaggtgtt
ggtactgatc
547261 ctgattcaga agatccagat aatcctactc ctatggataa tgttactgtt
tcagctgaag
547321 atcttgctgg tacagcaaac tatgatatta atactagcgt ttcttatcaa
ttcattgata
547381 atgataacaa tgatagtaaa gttggtgctc ctgtagttgt aacgggactt
aatggtgaat
547441 caaaggatgt tagcttagtt gtaccagaag gttatgaact tgtagatgta
ttaccaacta
547501 aagcaacttt tggtgatatt aatcgaacta ttagaattaa tttgaagcat
aagaaaacat
547561 cagttgatgg cagagatcat aaagatgata atgacattta tcgtacagta
actcgtacca
547621 ttacagttaa tattgaagga caacctacac aagttatatc tcaaaaagtt
tcactatata
547681 gaatcaagac agttgatgaa gctttaaagg cagcaggtaa gccagaatct
gagtacacaa
547741 cttacagtgc ttggacttca aatatgtctg atggatcaac ttcatttgca
acagtatcag
547801 taccagcaaa tggaaacggt tatgttcgtt caattactgg tggcacaatt
gaaaaggata
547861 aagttacaga acaatcagct ttaaacaacg gtgaaccagc caaggatata
actgtaactg
547921 ttaactatgc aaaggctgag caatctacaa ctgtaactta cgttgataaa
gatggtaatg
547981 aaattaagat gccagatggt cagccagtag cagggagtca ctatgaaatt
tcaggtaaga
548041 ctggagacac agttaatact gatgtaaaag acaaagtacc agcaggctgg
gagattattc
548101 caggaactaa gtatccaact acagtaacgt ttggctcaac tccaactagt
ccaattaaag
548161 ttaaagtaga gcacaagatt actaaaactg atggcagaaa tcacaaagat
aagcaagatt
548221 tgtaccgtga agtcactaga acaattttaa tgaatgttcc aaatgcaacg
agtcaaggta
548281 aagaaactga aactttgagt ttctacagaa ttatgaccca tgatgaagta
actggtgata
548341 atacttacag tgaatggaaa tcagatgtag caggtgataa gactgccttt
gatgaatttg
548401 atgtttcaaa gactaatgat ggtaaagaaa tcgctgctgg ttatactcca
acttcaagag
548461 atgtaacgtt agaagctaag aacggcgaca aatttgtacc aagtcaatca
gccttgaaga
548521 atggggttcc agcagatagt ttcattgttg aagttgctta cacaccaaat
gcacaaaaga
548581 caacagtaac ttatgttgat gaaagtggta atgaaattac caatccagat
ggtagcgtaa
548641 ttcctggtag tcactatgat ttaactggtg taacaaataa gaaagttgat
actaatattc

548701 agaagaatgt accaactaat tggcatatta ctgatccaga agttccagca actattacct
548761 ttggcgcaga tggtcatact ccaatcacag ttcacgtagc tcacaacact aagcctgtgg
548821 ataagaatga tgttccagat ggttacacta aagataattt tgttaaagag attaaacgct
548881 caattactgc taaggagcca actggtgatg ttgatctaag tcaaacaact gaattgacga
548941 gaactggaac ttatgacgaa gtaactaaga aggttatttc ttatggcaac tggacaactg
549001 gtagctttga tgaagttaca gctccagaag ttgcaggtta cacaccaagt caagctaaag
549061 ttgatggagc tgatgtaaca gtagatagta agtttgatcc aactgttatt acatatgcac
549121 caaatgcaca gaagacaaca gtaacttacg tcgataaaaa cggtaaggaa attaccaacc
549181 cagatggtgg tgcaatcact ggcagccatt acgatgtaga tggtgtaact ggtcaaaaag
549241 atattccaac taacatcgaa aagaatgttc caacaaattg gcacattact gataagacta
549301 ttccaggaac tattagcttt ggtccaaatg gacatgatcc aattaaagtt catgtagctc
549361 acaacactaa gccagctgat aaagataatg taccagatgg ctacaagagg gaagactttg
549421 ataagactgt aactcgtaca attactgcca aagaaccaag tggcgatgtt gatctaagtc
549481 aacatgttga attaacaaga actggtactt acgatgaagt aactaaagaa gttacataca
549541 acccttggac tactagcagc tttgttgaag taactgctcc agaagtagct ggttatactc
549601 caagtcaagc taaggttgat ggtgttaaag taactgcaga tagtaacttt gatccagttg
549661 tgattactta tgttgaagat ccaattggtc aagacatcac agtaaagaag ggtgacaccc
549721 catcaccaga agatggcgtt aagaatcatg gtgacttaga caaggtcact gatccaaagc
549781 atccagatgc taagacaacc tacacttgga agaagacgcc agacacaagc gttccaggta
549841 atgtcccagc aacagtgatt gttcattacc cagatggttc taataagcca gttgatatta
549901 cagttcatgt tatagatgaa actccaatac caaccaagga cccagatcca attggccaag
549961 acatcacagt aaagaagggc gacaccccat caccagaaga tggcgttaag aatcatggtg
550021 acttagacaa ggtcgctgat ccaaagcatc cagatgctag gacaacctac acttggaaga
550081 agacgccaga cacaagcgtc gcaggagatg tcccagcaac agtgatcgtt cactacccag
550141 atggttctaa taagccagtt gatatcacta ttcacgttgt agatgacact ccagtagtac
550201 caactaagaa ttcagatcca attggtcaag atatcacagt gaagaagggc gacactccag
550261 atccagaaga tggggttaag aaccacggtg atttagataa gatcacagat ccaaagcacc

550321 caggcactaa gacaacctac acttggaaga agacgccaga cacaagcgtc gcaggagatg
550381 tcccagcaac agtgatcgtt cactacccag acggttctaa taagccagtt gatattactg
550441 tccatgttgt agatgacact ccagtagtac caaccaagaa tccagatcca attggtcaag
550501 atatcacagt aaagaagggt gatactccag atccagaaga tggggttaag aaccacggtg
550561 atttagataa gatcacagat ccaaagcacc caggcactaa gacaacctac acctggaaga
550621 agactccaga tacaagcgtc gcaggcgatg tcccagcaac agtagttgtt cactacccag
550681 atggttctga aaagacagtt gatatcacag ttcatgttgt tgacgatact ccagtagtac
550741 caacaaagaa cccagatcca actggtcaag atattcatac tccacaaggt aaggttccaa
550801 ctcctgaatc agctattact aacaaggaca agatgccaga tggaactaag tacacttgga
550861 aggaaatccc agatgttaat actttaggta aacatcctag tgtagttgtt gtaacttacc
550921 cagatggaac tgctgttgaa gtgaaagtta atgtctttgt cgacggcact ccagaagcaa
550981 agaaagaaac taaagctcca gtagttaaga aacatgttgt tgaaccaaca aaagttgaaa
551041 caagacaaaa gttggttaat aattacgtgg ctcctcgtgc agtagaagtt caaagagctc
551101 aagctaaagg taagcgtcaa ttaccacaaa ctggtgctaa agaaaatatt gcaagtgaag
551161 tactaggcat gctttcagta ggcttaggtg cattgactgc aggatttgct tcaaagcgtc
551221 gtaagaagaa tagatagttt ttaggataac taattaaaaa cactccatga tttttcagaa
551281 atcacggggt gtttttgcgt agaaaaaaca taaaatttta atttaaaaat aattttgcat
551341 gatataattt aataaaaata ttatcttagg aggatgaaaa aatgatcatg gtagtaacaa
551401 acgctcatca aaatgtacaa atcactagat aatagatgag cggaacttaa ttaaatttca
551461 ttttgttaaa tccgttcatc gaggaggcaa aatgaaacat ttacataaat taaaccaata
551521 cttcaaagaa ttaactcgaa cttatcacct gttctttaaa tcagctcctt tagttgccgc
551581 ttgtgtctta ttacttgcac cacttcaagc agtaattcct ttacttgctg tggcagcagg
551641 acaaaaggta attgatcaag ttactaatca ttcacctttt atggaaatga tcattgtttg
551701 gattgtggca acggcatttc aacaattttt accatcgctt tcgacaatgg ttcaaggagt
551761 tctaactgat aaattaacag gctttatcaa tattagcttg atgaaaaagt cggcggactt
551821 acaatcgatt agtatttttg atgacagtaa gtattttgac gatttacaaa tgctcagaga
551881 tgatgcaagc tggcgtccag ttaatttaat tgtttttggt gtatcggttt tgcaatcatt

551941 tctaacgcta gctttcatgc taatatatct ggcgcgatac aattggtggt tagccttgct
552001 tttattagta gtgatggtgc cgcagagtat ttcttactat cgtattcagc agcagtcatt
552061 tgagacaatg gttgaaagaa gcaagaacgc aagatacttg cactactata gtggattgtt
552121 gcttgaccgc agggatgcta aagaagttag acttttcaac atgtttccaa agatcattga
552181 aaagtacata aacttgtttg agcaaacgaa aaaagatgtt aatcaaattc gtaaaaggca
552241 acttgcgact agttcgctat ttgttgtatt gactgttgga gtgtttggct atggctttta
552301 ttggtttact aatccagtaa gaacaggtgc attagaagtt ggtgtcttat tgatgtttgt
552361 ttcagtaatt ggctatattt ctaccagtat ggcgcgagta gtagaagaca gcagcttgtt
552421 atacgattca ttattatgga ttgaaaaata ctttaagttt cttgagtatc aagataattt
552481 tgaaaacggc agccagaact ttccggatga ttttgatgat attaatgtta agaatctgtc
552541 ttttacatat ccgttttctg atactgaaat tttacacaat gttagtttct cagttaaaag
552601 tggagaaaaa attgcagttg ttggagaaaa tgggtcggga aagtctactt tggtaaaatt
552661 attaatgcgt ttttatgacc cgactaatgg aaaaatttct gttgataatt atgatttaaa
552721 agactttaat atttttgact tacataaaaa tttatcagct acttttcagg atttttctcg
552781 ctttaaatta acgcttcaag aaaacgtgat tactggatat tcattcaata aagatagggt
552841 aaataatgtt cttaaagcat ctggattggg tgatttgtta gctaatgacc atcttaatct
552901 gaatacgatg ctggctaaag atttcaaaaa tggaactgat ttgtcaggtg gtcaatggca
552961 aaaagtagct ttggcacgag acttatatgc tgatggtaag attgaatttt tagatgaacc
553021 aacggcagct ttagacgcta agagcgaagc agaaatttat caacgtttct tgaaggaaaa
553081 tgataagaag acaattttct ttgttactca ccgcttgtca gcagttagat ttgctgataa
553141 agtgctattt ttagatggtg gaaaaattag cgggtttgac acgcatacta atttgttgca
553201 aactaatcca aaatataaag aaatgtatga cttacagaaa gatgcatatc tgtaaagtta
553261 aattttaaat aagtaataaa attcttcatg caagcaggca tggagaattt tttttgcctt
553321 ttaacagtta agatttttta tcagttttta cgttataata gaacataggt ttttggaaga
553381 aaggatattt ggtaacgaat ggcaaaaaaa gatacgacac caatgatgaa gcaatactac
553441 gagattaagg aacaatatcc cgatgcgttt ttgttttatc gtgtgggcga tttctacgaa
553501 ttatttgaag atgatgcagt taagggtgca caaattctag aattaacgtt aactcatcgt

553561 tcaaataaaa ctaaaaatcc gattccaatg gcaggagttc ctcacctagc tgtggatact
553621 tacgtcaata ctttggtgga aaaaggatat aaggttgcac tttgtgaaca acttgaagat
553681 cccaagaaag ctaaaggaat ggtcaaacgc gggattattc agttaattac gccaggaacc
553741 atgatgcagg agcgtcctga ccaagcaaaa gatagtaact acttaacttc agttatttca
553801 acaaattctg gctttggcct agcttacagt gacttatcca ctggggaaac tttttccaca
553861 cacttaacta attttgaagg tgtcgcaaat gaattattat cccttcaaac aagagaagtt
553921 gtgtataacg ggcatttaac agaagcaaac aaagactttt tgaagaaagc taatattact
553981 atttctgaac cagttaaagt agaaagtgaa catgcagaaa tttcttatgt agcccaaaac
554041 ttaactgacg atgcggaaat taaggccact aagcaactag tagcttactt actgtcaacg
554101 caaaagcgta gtttagccca cttacaagtt gctcaaagct atgagccgac gcaatatttg
554161 cagatgtctc atactgtgca aacaaactta gaattaatca agtcagctaa gacttctaag
554221 aagatgggct ctttattctg gctcttagat aagacaagta cggcaatggg cggtagattg
554281 ttaaaatcat ggattgagcg tccgctttta tcagttactg aaattaaccg acgtcaagaa
554341 atggttcaag cacttcttga tgattacttc acccgtgaaa aagttattga cagtttaaag
554401 ggtgtgtatg acctagaaag actgactggt cgaattgctt ttggctcagt caatgcacgt
554461 gaaatgctac aattagcgca ttctttaggt gcaattcctg atattttgaa ttccttgctt
554521 gaaacgaata atccacatct gcaaaacttt gctaagcaaa ttgatccttt aaagggaatc
554581 catgatttga ttgttaatac tattgtggat aatcccccac ttttaacgac agagggtggt
554641 ttgattagag aaggagtttc agaccaatta gaccgctacc gggacgcgat gaataatggt
554701 aaaaaatggc tatctgaaat ggaaagccac gaacgtgaag tgactggaat taacaactta
554761 aaggttggct ataacaaggt ctttggttac tatattgaag ttacgaattc aaataaagat
554821 aaggtgccca ctgatcgcta cactagaaag caaacattaa ctaatgcaga gcgttatatc
554881 acgcctgaac taaaggaaca tgaatcatta attctagaag cagaagctaa gtcaactggc
554941 ttagagtatg acttgtttgt aaagctaaga gaagatgtta aaaagtatat tccagcttta
555001 caaaaattag ctaaacaagt tgcaagttta gatgttttga cagattttgc gacagttagt
555061 gaacaaaata attatgttcg cccagactat acagttgata agcaagaaat taacgtagtt
555121 aatggtcgcc acccagttgt tgaacaagtt atgactgctg gtagttatat tccaaatgac

555181 gtgaagatgg atcaagatac tgatattttc ttaattactg gaccaaatat gtctggtaag
555241 tctacttata tgcgtcaaat ggccttgatt gcaatcatgg cgcagattgg ttgttttgtt
555301 ccagcagata gcgcaactct accaattttt gatcaaatct ttactcgaat cggagcagca
555361 gatgatttga tttctggtca gagtactttt atggtagaaa tgagcgaagc taatgatgct
555421 ttgcaacatg cgacaaaacg ttctttaatt ttatttgatg aaattggacg aggcactgct
555481 acttatgatg ggatggcttt ggccggagca attgtgaagt atcttcatga taaggtcgga
555541 gcaaagacgc tctttgcgac tcactaccac gaattaactg atttagacca aacactaaaa
555601 catttaaaga acattcatgt tggtgcgacc gaagaaaatg gcaagttgat tttcttacat
555661 aaaattttgc caggaccagc tgatcaatct tatggtattc atgttgctca attggcaggc
555721 ttaccacgta aagttttaag agaagcaact acgatgctga agagattaga gcggcaggga
555781 gcaggagagt tacagccggc tagtgagcaa cttgatctct ttaccgctga agaagcaagt
555841 gaaccggcaa tttcagatga tgaaaaagat gtcttggatg atattcaaaa tgtgtatctt
555901 gctgacaaaa ctcctttgca agtcatggaa cttgtatctc agtggcagca agagttgaaa
555961 gataaggact aataatcatg agtaagattc atgaactttc acctgaatta actaatcaaa
556021 ttgcggctgg tgaagttatt gaacgaccag ctagtgttgt taaagaatta tgtgaaaact
556081 cccttgatgc tggtagtagc cggattagaa ttgattttat tgatgcgggc ttaaagcaaa
556141 ttacggttca agacaatgga agcggaattg caaaagatca gcttgactta gcttttactc
556201 gtcatgcaac aagtaaaatt gcaactgagc gagatttatt taatatttct actttaggat
556261 ttcgcggaga ggccttggct tcaattgccg cagtttctca tgttgaagta ataactagta
556321 gcgataattt aggcggtgtt cgtgcaattt ttgtaggcag cgaaaagaaa ctacaagaag
556381 atgcagcttc accaaaggga actaagattt cagttagtga tttattttt aacacgccag
556441 ctcggcttaa gtatttacgc tctgaaagaa cggaaattct aaagattgtc gatattgtta
556501 accgacttgc tttaggacat cctgatgttg cttttactct cactaataac ggtaagatct
556561 tactaaaaac caatggacgt aatgatttaa gacaagatat tgcgaatatc tatggccgtc
556621 agcttgcgga aaagatggag gttcttaagg gcactagtcc tgattttaag attactggct
556681 tactttctga tcctaataca actcgttcaa atcgaaactt tatttcttta ttattaaatg
556741 gccgctatat aaaaaattat cgtctgactc aggcaattat agctggttat ggcagtaagt

556801 taaggccgcg ccgttatcca attgctgttg tcaacattga acttgatcca
cttttagttg
556861 atgttaatgt ccatccaact aaacaagaag tgcgcttgtc aaaggagcag
gaattagagc
556921 gtttattaac tacaagtatt tcagaagcct tagatcagac cagtcagatc
aattcaggtt
556981 tgaataatct attaactcct aaaaagtcga caaatattga ccaattaaaa
tttaacttaa
557041 ataaagatgt ggttgatact gttcgtccag tggagtttat tccgcaagtg
gaagctgata
557101 aaagtgctga agttcatgaa acagcagcaa attttgttag tttagataag
gttcgcaatg
557161 acgataaata tgtgattact gcaagctggg atgaaaatgt ggataaacag
gtgcggctaa
557221 gtccatttga tgaagaaaaa gatattcaag gaaaagatga tagtcttatt
tcttcagggg
557281 atgaaatttt agctaacaat ttacccgaat taatttattt aggtcagact
aagtcatatt
557341 tagttgcgcg ccataatgaa gacttatact taattgatca aatagcagct
tatcggcgtt
557401 tagcctatga tcaaattttt catgatttaa atagtgaaaa tatttctcag
cagggtttac
557461 ttagtccttt gattttagat tttagtaatg ttgactattt gaaactaaaa
gaaaatctta
557521 ataatctaca agaattaggg atatttttag aggattttgg tcaaaatagc
ctgatcttaa
557581 gaacttatcc gatctggctc caacctgatg tagaaaaaaa tgtgcgcatg
attttagatt
557641 tatatttaaa tcaaaatgaa caagatttta ccaagcttaa agcacaaatt
gctggcgaaa
557701 taacaatgcg ccaaagtact agaagaagaa atttaaatcc agttgaagca
caagaactat
557761 tgaaagactt gagaaatagt agtgatcctt atcaagattt tgaaggaaaa
atcattatta
557821 ttcaattaag tgagaatgat ctcaataaga tgtttaaaaa agatgagtag
gtaaaatgtt
557881 tgaatatctc aaaggaatag tagcaaaaat tgatccagct tatgtagtat
tagatgtaaa
557941 tggcattggg tataagattc tttgtcccac tccttatagc tatcaagaga
atcagccagc
558001 aactatttat gttgaacaag ttgttagaga tacaggaatt accttgtatg
gctttttatc
558061 tttagaagac aaagaattat ttttaaaatt attaagcgtt tcaggaattg
gaccaaaatc
558121 ggccgtagcg attatggcag ctgaggatac ggattcgtta gccagtgcaa
ttcaaaatgg
558181 agaggtaaaa tatttaactc gttttccagg agtcggaaag aagactgctt
ctcagattgt
558241 cttagactta aaaggaaagt taggcgacta tgtgaagaaa tcaacagttg
ctgccgatct
558301 ttcgccgagc ctgcaagacg ctgtgcttgc cttggtagca cttggttata
ctcaaaagga
558361 agttgatcga attactccaa aattagccaa gttacctgaa aatactgccg
atggctatat

558421 taaagaagcg ttggctttac tacttaagaa gtaaaagtaa aaattagtta aggataagga
558481 agtgaaagat gtgaatgatg aagaacgaat tataggcgct gaaagtagcg aagaagatga
558541 aacgatcgaa ttgtctttgc gtccgcaact acttgctcaa tatattggac aagataaagt
558601 aaagagcgag atgaaaatct atattaaagc tgctaaaaag cgagacgaag ccttagacca
558661 tgttttgtta tacggacctc ctggtttagg taaaactact ttggcatttg taattgctaa
558721 tgaaatgggt gttcacttaa agagtacctc gggcccagca attgaaaaag cgggagattt
558781 ggtggctctg ctttcagaat taaatccagg ggatgtctta tttattgatg agattcaccg
558841 cttagcaaag ccagtcgaag aagtcttata ttcagcaatg gaagattttt atattgatat
558901 tgtggtcgga gaagggcaaa caactcatgc agtgcacgtg ccacttcctc cttttacttt
558961 gattggagca actaccaggg ctggtcaatt atctgcgccg ctaagagatc ggtttggaat
559021 tattgaacac atgcagtact attctgttga tgatctagaa aaaattattc aaagatcaag
559081 cgtagttttt aacactaaga ttgatccaga agcagcaatt gaattagcgc gacgttctcg
559141 tggaacgcca cgtgttgcta accgtttact taagcgggtt cgcgattttg cggaagtaaa
559201 gggagaagaa gctatttctt tagcaactac taagcactca cttcatttgc tagaagttga
559261 tgatgaagga ttagatcaaa ctgatagaaa acttttaaga atgatgattg aaaattatgg
559321 tggtggtcct gttggaatta aaaccattgc agctaatgtt ggtgaagaca cggatacaat
559381 tgaggaagtt tatgaaccat atttacttca aaaaggattc attactcgaa ctcctagggg
559441 aagaagcgtt acgcaaaaag cttatttaca attaggatat ccaccaaaaa aagcagagta
559501 agtttttagt tagactaaaa atacggtata attattattt gaaagttaat taataaaaat
559561 aatgaggtgt aatactttga atacattttt cttagcacaa agtgctgctg gtatgaataa
559621 catgttcatg attattgcaa tgattgcaat ttttgtcttc ttctactttt caatgattaa
559681 gccacaaaag aagcaacaac aagaacggat gaagatgatg tctgaactta agaagggcga
559741 tcaagtaatt atggttgatg gtcttcacgg taaagttgat tcaatcaatg acgcagataa
559801 gactgttgtg attgatgccg atggaatctt cttaacattt gaaagaatgg ctattcgtcg
559861 cgttcttcca actgctgccg ctccagcaaa agatgttgaa gctaatgaag caaaagaaga
559921 aaaggttgaa acagaagaaa agcctactga aagtagtaaa acagaagaaa caacttctga
559981 caaagctgat aatactaatt ctgaagataa ataaaaaaac gtccgcaagg acgtttttat

560041 tttttcattg tcatagatta atattgtaaa atatgaattg gtaaagtaaa ttttaataag
560101 tttaataggt aggtagttag aatggaaaat attccagttg tcatgattat ttttggtggc
560161 tcgggtgatt tagctcatag aaagctttat ccagccttat ttaacttata tcagaaggga
560221 ttaattcatg atcattttgc cgtaattggt acagctagaa gaccatggag tcatgaatac
560281 ttacaagaac aagttgttgc agcaatcaaa gaaagcaaca atagctttga tgagaaaaat
560341 gcaaaagaat ttgcttcgca tttttattac cagtcacatg atgtaactga tgttgatcac
560401 tacattgctc ttaaagaatt agctacaaag cttgataaga aatatcatgc agaaggcaac
560461 cgtattttct acatggcaat ggcaccaaga ttttttggca caattgcaac tcatattaat
560521 gatcaaaagc tagttggtag tggttttaac cgcttggtaa ttgaaaagcc gtttggtcat
560581 gacctagctt ctgctgaaaa attaaatcaa gaaattggag aaagttttgc tgaagattcg
560641 gtatatcgaa ttgaccacta tctcggtaaa gagatggttc aaaatattat gccgcttaga
560701 atgactaatc caattgttaa taatatttgg tgcaagaagt acattgctaa tatgcaagtc
560761 actcttgctg aaagcttagg cgttggtact cgtggtggat attatgaaac ttctggtgct
560821 ttacgcgata tggttcaaaa ccatattttt caaattatta ctctcttagc aatgccagaa
560881 cctaaagcct tagattcaga tcatattcat gaagcaaagc aagaactact tgatagcttg
560941 gttattccaa ctccagaaat ggtaaagaag catttctcac gtggtcaata tttagcaagc
561001 gacgatgaag tagagtactt aaaggctgac caagttgctc ctgattctaa agtagagacg
561061 tttgttgctg gtgaagttaa cttcaaaaag ggtcctgtag ctggtgttcc aatctacttt
561121 agaactggta agaagatgaa ggataaagtt tctagaattg atattgtgct ccaccacatg
561181 aataatttgt acggcaatgc acattcaaat aatatttcaa ttattattga tccaagaagc
561241 gagattttct ttacaattaa tggaaagaag attactactg agggcttaag aagagaaaat
561301 cttagctata agttttctaa agaagaaatg gatcaagttc cagatggcta tgaaagatta
561361 cttcatgatg tctttgtagc tgatcgtact aactttacgc actggtctga attaaagcaa
561421 tactggaagt ttgttgatgc agttgaagct gcatggcaag atgaaaacaa agatattaag
561481 caacttgagc aatatccaag tggtgaattt ggtactgaat caagtaacca tatttttgaa
561541 aaagatactg agcattggat ttaccgttaa tggacttact gcctaaaaat aatattaagc
561601 gtaaaattat tcatctggat atggatgctt tttatgcctc agtagagatt cgagataatc

561661 ctgctttaaa aaataaagct gttttaattg gcggcgatcc taaaaaaaat aggggacacg
561721 gggttgtggc aactgctaat tatttggccc gtcaatatgg tgcgcattca gccatgccaa
561781 ctgctaaggc agttaggtta attccagcag aaaaacttgt aattattcaa ccacattttg
561841 aaaagtatcg agctgtttca gctgaaattc accgtttaat gcatgagatg actgatcgag
561901 tagaatcagt ttctttagat gaagcatact tagatgtaac ggagaataag cttcacttag
561961 atgatcctgt tcgaattgca actatccttc aagagcaaat ttatcaaaag gttggattga
562021 caagttcatt tggcgtttct tacaacaagt ttttagctaa aatggggtcg gaatatgcta
562081 aaccctttgg tagaactgta attaagcctg aaactgcact tgattttta gcaaagcaaa
562141 aaattgaaaa gtttcccggc attggaccta aaacgcaaga aagattagca gagatgggag
562201 tctacaccgg agctgaccta ataaaaattc caacagatgt gttgatcaaa aaatttaatc
562261 gtatgggcta tttgattgcc caacatgcac atggtattga cttgagagag gtagttactg
562321 attcagaaag aaatcgaaag tcgattggga ttgagcgaac ttttaatcag agtttatttg
562381 atgaaaatga agctttaact aaactgcgag cttatagcgg tgaacttgaa aataagttaa
562441 aaaagcgaca ttttcttgca aattgtgttg ttttaaagat tcgcgatgtt aattttaaga
562501 caattactaa aagaaagaag ctaaaacaag gcactaatga taaaattgtt atttacgaca
562561 ctggtcgtgt cttgtttgaa acagaaaaag ggatgctcgc aactggagta cgtctgttag
562621 gacttactgt aaccgatttt gaagaacatc cagttgagaa cttatcgtta gatattttg
562681 aaaacaagta atgataaaat aaatttagta aacaacaagg agaacagatt aatggcaact
562741 tttgatgaaa tttatgaaaa gataaaagaa tacccaacaa ttattttgca ccgtcacact
562801 agtcctgacc cagatgcaat tggctctcaa gctggtttag caagatcatt gagattagct
562861 tttccagata aaaagattct ttgtgctggt gaaaatgatg aaggtgactt gatctggatt
562921 aataaaatgg atgaagtaac acctgaagat tatcaaggag ctttagtaat tactactgat
562981 actgcaaata ctccacgtat ctctaataag aattacgata agggagatct tttaattaag
563041 attgaccacc acccagatgt agatccatat gcagatatga gctatgtaga tccagacgca
563101 ccagctgctt cagaaattgt ctttgacttt ttgaaagcag agaacttacc agttactaag
563161 gaagttgcgg cagctttata tgcaggtatt gtcggcgata caggtagatt tatgtatcct
563221 gaaacttcag cgcacacttt tgacgttgca gccgaattaa ctaagacagg aattaacatt

563281 actgatattg ctagagaaat tgcagatgtt acttttgatg aagctaagtt gcaagcttta
563341 gcaatggata agatggaaat taacccagtt ggtgcagctt acacaatttt gatgcaagac
563401 gatttaaaga agatgggatt aactgacgat caagcaaatg ttgctgtttc aactccaggt
563461 cgaattaagg acgttttagc ttggaatgta tttgttgaaa aaccagacgg tactttccgc
563521 gttcattatc gttcaaaggg tcctgtaatt aatcatttag ctgaaaagca tgatggtggt
563581 ggccatgcat tagcatctgg tgccaatgcc aaggatatgg atgaagtaaa acaagtcttt
563641 gacgaagtag tagaagtaac aaagaagtac aatgaagaac atggcacaaa caaatagtat
563701 atttcaaagt gaaaagatta gaccagaatt acgaactggt ctagaaaaaa ttaactttag
563761 taaacccact aaggttcaag ctgcagttat tccggctctt ttaaataata aaaacgtcgt
563821 agttcaagca gctactggtt caggtaagac tcatgcctac ctgcttccac ttttgaacat
563881 gattgatgaa aatgccccag taacgcaagc tgtagtaacc gcgcctagtc gtgaattagc
563941 aaatcaattg tataaagtag cgcgtcaatt aagggatgcg agtggtctaa atatttcaat
564001 tgaatattta ggtggtggca atgaccggaa tcgccaaatt gaaaaagctg aaaatagggc
564061 tccacaatta attattgcta ctcctggtcg tttacatgac tttgcatcaa agaaagttat
564121 taatttagat aacgtcaaag cttttatcat tgatgaagca gacatgacac ttgatatggg
564181 ctttttaggg caaatggatg agattatctc taagttagat aaaaaagtcg ttttaggtgc
564241 tttttcagca actatcccag ttaagctgga aaatttctta aggaagtata tgtcaaagcc
564301 ggactttatc gttattgata atccagcaat tattgctcca acaattcaaa acgacttaat
564361 tgatgttggt tcaagagata aaaaagaaat tttatttaag ctcttaacaa tgggacaacc
564421 atatttggcc ttagtttttg ctaatactaa aaagactgtt gatgaattaa ctgattatct
564481 tgaagagcaa ggccttaagg tggctaagat ccatggcgga attactgaac gtgagcgaaa
564541 gcgtataatt cgccaagtta gagaaggtca gtaccagtat gttgtagcta gcgatttagc
564601 tgctagaggt attgacgtac ctggcgttag tttagtggtt aactatgaga ttcctaagga
564661 tcttgaattt gttattcacc gaattggtag aactggtcga aatggcctta aaggtcacgc
564721 aattaccttg atctatgatg aggaaatgcc acaaattgaa gacttagaaa aattaggtat
564781 tcactttgac tttaaagaaa ttaaaaatgg tgaattagtt gaaagaacac actaccatcg
564841 tcgtgacaat cgtcaagctc gcagtcacaa gcttgataat cgcatgattg gaatggttaa

564901 gaagactaag aaaaaagtaa agcccggtta taagaagaaa attaagcaag ccattcaaaa
564961 agaccgccag caaaagcgta agctcgaaga gcgtcatcaa attagaaaag ctaaacgtag
565021 acgtaagcgt gagcgcgaac aagcacgcgg aaattttgac aactagaaaa tttgtagtaa
565081 aataaatcac gtttaggaca tattactgaa gtattttctt caaagagagg accaggcggg
565141 tgagaaggtc tagaagtact tagtaatgct acctaattta actaagaaat taaatattta
565201 aagaggtaac aaacactgtt gcaatcaagg tggtaccgcg ctgggagcgt ccttgtttta
565261 tgcaatagtg tttttatat ttgtaggaga ctttttatga aacaattgac tagttcacaa
565321 gtacgtcaaa tgttcttgga cttttttaaa gagcatggcc atatggttat gcccagtgca
565381 tcattaattc cacaagacga tccaacctta ttgtggatta actctggtgt tgctacgatg
565441 aagaaatatt tcgatggttc tgttgtacct aagaatcacc gaattacttc ttctcaaaaa
565501 tcaattagaa ctaatgatat tgagaatgtt ggtaaaactg cacgtcacca aactttcttt
565561 gaaatgcttg gtaacttctc agttggggac tactttaaga aagaagttat tccttgggca
565621 tgggaatttt taactagtcc aaaatggtta ggcttagatc cagataagct ttatgtaact
565681 gtttatccta aggatacaga agcataccat atgtggcatg atgttgttgg cttacctgaa
565741 gatcacattg tgaagttaga agataacttc tgggatattg gtgaaggccc atgtggtcct
565801 gactcagaaa ttttctatga tcgtggtcaa gaaaataacg atgttgctga agatgatcct
565861 gaaaacttcc caggtgggga aaatgctcgc taccttgaga tttggaacat cgtattttca
565921 caattcaatc acttaccaaa tggtaaatat gttgatcaac cacacaaaaa cattgatacc
565981 ggtatgggat tagagcgtgt tgtttcaatt attcaagatg caccaactaa ctttgaaact
566041 gacttgttca tgccaattat taaagaaact gaaaagttaa gtgatggcaa gaagtatgca
566101 gcaaacaagg aagatgatgt ggcatttaag attattgctg accacgttcg tgctgtaagt
566161 tttgcgattg ctgatggagc tcttccttca aactcaggtc gtggatatgt tttacgtcgt
566221 ttaattagac gtgccgactt gaatggtcag cgtttgggta ttaagggtgc atttttgtac
566281 aaattagtac ctgtagttgg cgaaatcatg aagagtcact acccagaagt tgttgatcaa
566341 caagcattca ttcaaaaagt aattaagaac gaggaagaaa gattccaagt aacgctttca
566401 tctggtttga acttacttga taacattatt gctgaagcta agaagagcga tgacaagact
566461 gtttctggta aagatgcgtt taagttattt gatacttacg gcttcccata tgaattaact

566521 tttgaagctg ctcaagatgc cggtcttaag gttgataaga agggctttga tgaagaaatg
566581 aaagcccaaa aagaacgtgc tcgtaaggct cgtggtaact tgcaatcaat gggatcacaa
566641 gatgttactt tgatgaacat caaggacaag agtgaatttg agtacggcac tctagaagaa
566701 aagcatgcta agttaatcga tattgttgtt gatgataagc tagttgataa agccgatggt
566761 gaacacgcaa ctttaatttt tgataagact ccattttatg cagaacgcgg tggacaagtt
566821 gctgaccatg gtgaaatttt gaaccaaaat ggtgaattag ttgctcgcgt aacagatgta
566881 caacatgcac caaacgatca aaatttacac ttcgttgata ttattttgcc acttgaaaag
566941 ggacaagaat atgtcttgaa agttgaccaa aaacgtcgtc gcggcttaaa gcacaaccat
567001 acagctactc acttattgca tgctgcttta cgtgaagttt taggtactca cactcaccaa
567061 gctggatctt tagttgaacc agactaccta cgttttgact ttactagttt agagccaatg
567121 actaagaagg aaattgctaa cgttgaaaag atcgttaacg aaaagatctg ggaagaaatt
567181 ccagttaaga caacagttac tgatcctgat actggtttaa agatgggtgc tttagcccta
567241 tttggtgaaa aatatggtga cacagttcgc gttgttcaaa ttgatgactt ctcaactgaa
567301 ttctgtggtg gtacccactg tgaaaacact gaccaaatcg gtatgcttaa gattgtttct
567361 gaatctgctg ttggtgctgg tactcgtaga attattgctg ttactggccc agaagcttac
567421 aagtatgtaa cagaccgtga cgagatcttg aaagaagttc aagatgaagt taaggcaact
567481 aaggctgaag atgtaactaa caagatttct tctcttgaag aagacttacg tgcaagccaa
567541 aaagaagctg aacaattaaa ggcacaaatt aacaaggcaa aagcaggcga cttgtttaat
567601 gatgttaagc aagttaaaga tttaactgta attgctgctc aagctgatgt agaaggcatg
567661 aatgatttac gtgaacttgc cgacaattgg aagagcagtg ctaaatctga tgttttagtt
567721 ttagctgctg aagttaacgg taaagccaac atggttatta gcttgaacga taaggcaatt
567781 aaagctggtc ttaaagcagg cgacttaatt aaaacagctg ctccaatctt tggtggtggc
567841 ggtggcggtc gtccaaatat ggcacaagcc ggtggtaaaa atccagcagg tttaaaggac
567901 gctatcgcta aagtgttaca agaagttgaa gaaaaacaaa attaattgag ttcgttcgat
567961 atttcaagta aaattaagaa gaataggagg aattttatta tgagttcgct agataaaaca
568021 atgcattttg actttaacca aaataagggt aagaatgtat acgacactct acaagacgta
568081 tacaatgcac ttgaggaaaa gggctacagc ccaattaatc aaattgttgg ttacttactc

568141 tcaggcgacc ctgcatatat tcctcggcat aatgatgccc gtaatttaat cttgaaacat
568201 gaacgtgatg aaattattga ggaattggtt aagagttatt taggtaaaaa taaataatgc
568261 gattacttgg actggacgtt ggctctaaga ctgtgggggt tgcaattagc gatcctttgg
568321 gaatcactgc tcaagagctt gaaacaatca aaattgacga gagcaagttt agttttggca
568381 tgcgtcaaat cagaaagatc gtccgtaaat atgacgtcga aggttttgtt ttaggactac
568441 ccaaaaatat ggatggcagt agtgggcatt ctgtagaaag aagtaagcag tacggtgaac
568501 gcttgaaaga gaaatttgac ttgcctgttc attatatgga tgagcggctt acaactgtac
568561 aagctgatcg tattctagtt caagaagctg gcgtacatga tcgtgttgaa agaaaaaaag
568621 ttattgacca aatggctgct gttttgattt tacaaagtta tctagaagca accagaaagg
568681 ataagtaaaa tgagtgaaaa aattaacgct aaccaagata atgatcgtca aatcacttta
568741 gttgatgatc aaggcaatga agaacttttt gaaatattat tcacttttac gtctgaagat
568801 tatggtaaat cctatgtttt actatatcca gcagccgtta gcgacgatga tgatgtggaa
568861 gtacaggctt ttagctacga tgctgacgaa gacggtgatg taaccagttc tgacttacac
568921 gaaatcactg atgatgacga gtggaatatg gtacaaggcg ttttaaatac atttttgtca
568981 gacgatcgct taagtggcga ataatcttca aaaaagactg gcggaggggc tagtcttttt
569041 tgcttaataa ggatgaacaa tgatttctat tctcatttta ttaattttta tttattgttg
569101 ttatgtaggt tatcgacgtg gcatagtgta tgaaggcttg gctgcaggtg gatatgtagt
569161 agggttgatt tttgcaacat tattgtacaa accctttagt aacttcttaa atctttgggt
569221 gccatatcct tcagcgagtg atcgaagtag ttttgccttc tttgataaga ctacaggatt
569281 aactttagat aagtcatttt atgcagcaat agctttttcg attgttttgt tgcttgtttg
569341 ctgtatatgg cgtttggtga tgttaggttt taatcagcta aggtatatta ctgtagatgc
569401 tagattaaat acatggggca gcattattat tgcttttatt gtgacccaga ttagtgttta
569461 tttgctttta tttattttag ctacgattcc taataatagt ttgcaaaata tgctggggca
569521 ttctatttta gcaagtggta tcttgcattt ttctccagga atttcacaaa ttttcataaa
569581 attattcatt acaacaatat aaaaagtggg cccagttatg ggcccttttt tgctaggtgg
569641 aagatatgaa ctctaaaatt attgaaaaat tagaatataa tcgcataatt aaacaattaa
569701 gtgacttggc aattactgca cctgcaaaag ctcaggcttt gaaattaatg ccaagcagtg

569761 actttgatga agtaaaaaaa tcgattgatc aaactagggt actttcgaat atcttgcggg
569821 ttaagggacc aatgccgata acggacttta aagatgttcg acctagtttg aagcgactta
569881 aagttaaggc taacttaaat ggcgaagaat taggtaatat atttctagtc cttagtctgg
569941 ctaaagacgt gggacaattt acatcggacc ttgaagagcg tgaaattgat acacgtccta
570001 ttgaaaaata tctcaaaaat ttagcagttt cagaagattt gtttaaaaaa ttaaatcagt
570061 caattgaata tgatggaaca gttaaggata cagcttcttc caaattgatg cagcttagac
570121 atgatattca gagtaatgaa actgatatta agaaccacat gaatgactat attagtggca
570181 aacatacgca atatttgtct gaaaatattg taactatcag agatggacgt tgtgttttac
570241 cggtaaaaca agaatataag aataaatttg gtggagttgt tcatgatcaa agcgctagtg
570301 gtcaaacttt atttgttgag ccacaagcag tcttggtact taataatcgt cagcaaaatc
570361 tgttggctca agagcgacaa gagattcacc gtattttaat cgagttatca gaacttgctg
570421 gtacttatca aaaagaaatt aagaataacg cagtagcttt agctcaatta gactttttaa
570481 gtgctaaaag taaattagca aagaaaatga aagctacaga gcctgtccta aatcaagatc
570541 atgttatcaa attaagaaag gcacgacacc ctttaattga tcctaaaaaa gtagtaccca
570601 ataatattga actaggaact acttttgaca ctatgttaat tacaggacca aacaccggtg
570661 gtaaaacgat cacgttgaag actttgggat tacttcaatt aatggcacaa gctggcttat
570721 ttattactgc agaagagggg agtcagttaa cggtctttaa tgaaatctat gcagatattg
570781 gagatgaaca atcgattgag caatctttaa gtacattttc atcgcatatg gatcaaatta
570841 ttaagatcat gaataatgtt actgaagatg atttggtttt aattgatgaa cttggtgctg
570901 gaactgatcc agaggaaggt gcaagtttag caatcgcaat cttagatgat ttacgccaaa
570961 cacaagcaaa gattgcgatt actacgcact atccagaatt aaaactgtat ggttataatc
571021 gcgagcgaac taccaacgct tcaatggaat ttgacttaaa aaaattggca ccaacttatc
571081 gcttgagaat tggaattcct ggtcaaagta atgccttggc gattgcacat caattaggga
571141 tgaatgaagc tgttgtagat aaggctagaa gtttaatgaa cgatgaagac agcgatatta
571201 ataaaatgat tgagcgttta actgaacaaa ctaaggctgc tgaacaactt catgaaactt
571261 tgaagcaaaa tgttgatcaa agtatcacgc ttaaacgtca acttcaaaat ggacttgatt
571321 ggtataacca acaagtacag aaacaacttg aaaaatctca ggagaaggct gatgaaatgc

571381 ttgctaagaa acgtaagcaa gctgaaaaaa ttatcaatga tcttgaagag caaagaagag
571441 ctggcggaca agttagaaca aataaagtaa ttgaagctaa aggcgctttg aataaacttg
571501 agcgtgaaaa tcaaaactta gctaataata aggtgctaca gcgtgaaaaa aagcgtcatg
571561 atgtaagtgt aggcgataat gttaaagtcc tttcatatgg ccaacaaggt gtaattacga
571621 agaagttagg cgatcatgaa tttgaagtcc aaattggtat tttgaaggtg aaagtaactg
571681 atcgtgatgt tgaaaagatc gctgcacaag ccaatcaaaa aaagccagaa aaatctgttc
571741 gctctagccg cggtcttcgc tctagccgtg caagcagtga acttgattta agagggcagc
571801 gctatgaaga agctttaact aacttagatc gttatcttga tgcctcgctt ttagctggtt
571861 tgaatacggt gaccatcatt catggtattg gtacaggtgc aattcgaaat ggtgttcaac
571921 agtatttgaa acgtaatcga catgtaaaga gttataatta tgctccagcc aatcaaggtg
571981 gaacaggggc tacaatagtt aatttgcaat aagcaatata cttgcaaaaa gtaagtagtt
572041 ctactaaact atatttataa tttaaagaat ttattttgtg aggtaataac tatggttgat
572101 gaaattacag atgcaacttt tgaagatgaa actagcgaag gtgttgtttt aactgatttc
572161 tgggcaactt ggtgtggtcc atgtaaaatg caatcaccag taattgacca actttcagaa
572221 gaaatggacg atgtaaagtt cactaagatg gatgttgacc aaaatcaaga aacagctcga
572281 aacttaggaa tcatggcaat tccaacttta ctaattaaaa aagatggtaa aattgttgac
572341 cgcctaactg gctatactcc taaggaaaag cttgaacaaa ttttagatca atacactgat
572401 taatttacag tcaaaaaggg tacaaactat gaattttaaa tagtttgtac ccttttttagt
572461 attaatattt tttcttcatt aagagtaatt aatcagactg agctgtgatt tgaacgacat
572521 gtttttttagt aatcgtagct gaagcctcag taggagtacc attttggcgt tctaaacatt
572581 ctgcaataat tccactttct aatgaaaact catcactttg actatctagt cgatcagtaa
572641 caacttgtcc tgaaagttcg aaaactgcag tgtgtttacg atctttgagt acttttaaag
572701 ttccaaattg agccatgtca aaaaattcga ttaagtcatc gatatctgat aagtcatatt
572761 tacgactgac atgtttacca gcccagtaaa gaatttcttt atcttcgcta cctaagatag
572821 taggcaagat aaagtcacgg tataatgaat ttaaaaaata aagatgttcg tttgtatgtt
572881 ccatgctttc atttcctttt gtcttctata acattttaac ttaaaatagg tggaggggaa
572941 aagagatttt aatcatcatt tgtttaaagg agttttttta atggataatc gaccaattgg

573001 agttttagat tcaggcttag gtggcttaac tgtattaaaa aaagtaattg aaaaaatgcc
573061 taatgaatca actattttca ttggtgatca ggctaatatg ccatatggag atcgctcaaa
573121 agaagaaatt atttctttaa ctcgcgatag tgtaaacttc ttattaagta aagatgtaaa
573181 gattattatt tttggttgca acacagcgac tgcagttgca atgtctacta ttcaaaaaga
573241 agttcctttg caaataattg gagtggttca atctggtgcc ttagctgcag ctaggaccac
573301 agaaacaaag aatgtcgcgg taattggtac taaggcaact gttaatagtc attcttattt
573361 aaaagaaatt caatatcgtg atccagaaat tcaagtaagc gaatttgcac aacctaaatt
573421 agcacctctt gctgaggaag atcctgctga agagattaaa caggctgttg tcagcgaaag
573481 cttagcgcca ttgaaaaata ttgattacga tactcttgtt ttaggctgta ctcattatcc
573541 attattaaga gatgaaattg ttgcagtggt tggtcaagat aaaaaaattg ttgatccagc
573601 tgatcaggtg gcacaatata cctataacgt tttacggcgt gacggcttat ttgctgctga
573661 tgattctaat acaacacatg agtactatac gacaggggaa gctaagaagt tcactgaaat
573721 aacacgtcaa tggatgaatg atgaaacaat tgttggtcat catgtagatg ctgaggatta
573781 aaatggatac tttattattt gcaactaaca acaaaaataa ggctaaggaa gttgaagagg
573841 ccttacaaaa gaataacttt ccaattcatg tgattactaa tcaagattta actgatccgc
573901 cacatgtttt agaaactggt accacatttt tagctaatgc caagctaaag gctcaccaga
573961 tggcagaatt tagcaatcta ccgactttag ctgatgattc aggattatcg gtcgataaat
574021 taaatggcgc tcctggtgtc tattctgcgc gctatggcgg ggaagctcat aatgacgctt
574081 tgaacaatgc taaattatta gctgaattag gcggcgtgcc aaaagaagaa agaaaggcta
574141 cttttcatac aacaatggtt gtttcctggc caggtaaatt tgaggatgat ttagtaaccg
574201 aaggcgaaat tcgcggtgaa attttaactt atccacaagg taaaggcaat ttcggttatg
574261 atccgctttt ctttgttcct gataagggca aaacgtttgc tgaaatgaca gtggatgaaa
574321 agaatgcaat ttctcatcga ggtcaagctt tgagaaaatt acttactgaa ttgccagcct
574381 ggtggaaaaa gatggaaaat aaataaattt cgaagaactt attctgatta atggataagt
574441 tcttttattt tctactttta attgttaaga tgaaagcgct atactaaaat tgtgagttta
574501 cttgataaat gtaagtttag ttgaaattga ggttatagcg tatgaaaaaa gttttagcaa
574561 ttaattcagg aagctcttcc tttaaatata aattattttc tttccctgat gaaaaagtca

574621 ttgcttcagg aatggctgat cgagtgggga tggaaaatgc tgtctttaaa atcaagctaa
574681 gtaatggccg agaatatatc aaaaatatgc ctattcatga tcaagaagag gcagtgaagc
574741 ttttaattga ggatttgaaa aaatttcatg tagttgaaga cctgagagaa attactggaa
574801 ttggtcaccg aattgtaaat ggcggtgaaa tatttaagga atctgttcgt gttggtgaca
574861 aagagttaca agaaatattt gatttaggtg aacttgcacc tttgcataat attcctgagg
574921 caaatggaat caaggccttt ttgaaaattg ttcctaatgt tccacaggta gcggtttttg
574981 atacttctta tcatcaaact ttagatccaa tccactatct atattcgatt ccttatgaat
575041 attatgaaga ttatggcatc agaaaatatg gtgcgcatgg aatttctatt tcttatgttg
575101 caccgcgtgc agccaagatg cttaagaaaa atcctaactt agttaacttg attgtttgcc
575161 acttaggatc aggtgcttca gttactgcgg ttaaacgtgg aaagtcttat gatacttcaa
575221 tgggaattag cccgttaact ggtgtaacaa tgggaacgag aagtggagat tttgatcctt
575281 cagcgctgca acgtttaatg cataagaccg gaatgaatat tgatgaagca attgatgttt
575341 tgaattataa gtcaggatta ttaggtattt caggtgtatc ttctgatatg cgtgatttaa
575401 ttgaaagtaa agataagcgt gctaagttag cacgtaagat attcattaac cgagttgttc
575461 gctatgttgg cgcctatgca gcagagttag gcagaattga tgcagttgtc tttactgcag
575521 gagtaggtga acatgatcct ggcattagag ctggtattat gtcttcatta agatatttgg
575581 gtttagaacc agattttaag gctaatcgaa cagatggcga aaagtttatt tctaaaccta
575641 agtctagagt aaaggcactt attgtcccaa ctaatgaaga attaatgatt gctcgcgaag
575701 tagttcgtgt tgtgcaataa aaaaggatta agcaatagat aaaagtctag ttgcttaatc
575761 ctttaagatc cagaactatt ttcttgtttt gttggcatgg gactgaattt aagcccgcgt
575821 tgattgaatt ctttgatata agcactgaga taggcatttt taatcgtact ttggctattt
575881 gcaattactt gaaaatgaat ttgataggta atacttgagc cggtttgatt aatgattcca
575941 ataatttgag ggcccttttt gatttgtttt ttaaacttat cttttaaagc ggcgtttacc
576001 gtatcaacgg catgattaat ttcagtaaga ttattttcta ctgaaagatt taaattaata
576061 tccatgctcc agccagagtg tgaaccatga ctcaaatttt caactacggt aatgttacga
576121 ttaggaatat agattacagc gccattagta gttttaaggc gggtattgcg caaggtaaag
576181 gagagaacag ttccggtgta gttgccaatt ttgacagtat cgccgatatt gtattcacct

576241 tcgcttaaga tgttcatccc agttatgaca tcgctaacta agccctgtgc gcccattcct
576301 aaggctagag atactaaccc tacactagct aacaacgttc cgactgggat tcccattaat
576361 gttaaaacgc tatataaata aaataagaga acagtatatt gaaaaagagc aattcctaat
576421 tgagctagag ttcttttcct tccagtcata cgttcttgaa atttaggatt ctttaataaa
576481 tatttcgtta ataagcgttt ccctatatgc caaattaaaa agaaaataat tgtggtaata
576541 ataatttgta taccacgact taataaagta tgaagtacat tgtcccaatt aattaaggaa
576601 tcaatgttat tagttttttt agttgtagaa gttcctttta gaagagtagg ccagttcaac
576661 tcatgccctc cttatctaat ttctttaaaa atagtatatc aaaaacttgg gtactatttc
576721 attgcttgag gggaggaata atgctagact tttttatgag aggaataatt aatggaggat
576781 agattatgtc aatttcatat ggtgcattag ctggcttaat tgcagcagtt gccttttttaa
576841 tcttggtctt atttacttta cctttaattg ttagagttac taagacaatg aaaaaagttg
576901 acgcaactat ggacagcgtt aatacagctg ttgatgattt aacgaaacaa acttctgtat
576961 taatgaagca aagtgaagat ttattagaaa aaagtaatgc tcttctagct gacgtaaatg
577021 gaaaagtaac agaattagaa ccagttgtaa aggcggcagc agatttaggt gaaagcttgt
577081 cagacatcaa ttcttcatca agaagaatgg ttgaacgctt ctctggtatg ggtattagag
577141 gagccggaat tggcatcttt tcatctctag ttagtcgcat gtttgcaaga cgtaaacgtc
577201 gtcgcggtga agactaatat ttattaaagg agaacaatta tgcgtaaact tggtggattt
577261 ttattaggta gcgttgttgg tttaggtgtt ggcttaattg ctggctcatt attgttgcca
577321 gaagacgcaa ctgacgatgt aaagaaaaaa cttgctgaaa atgaaaaatt gcaagatttg
577381 aaagaaaagt atgataaagg aactgaagca attaagaatc aattagcttc tttccctaag
577441 tcagttgaag atgattcaga attaagagat tttgatgata tcgtaattga tgacactaac
577501 aaagatcttg gcgaagatga aaaatctgac aaggatgctg ttagtgactt aaacaatgct
577561 gaagatactg aaaataacta aaattaaata tttataaaga gaatcaagtt ttgcttgatt
577621 cttttttttac aataaaaaac ctgctgataa agtttatcaa caggtttttt gattagtctt
577681 taactggaat gtattttaat tccttacttg tcttagtgaa ggtttcaaaa ccgtttttag
577741 tgactacacc acaatcttca attctaacgc cggctacgtt aggaatgtaa ataccaggtt
577801 caattgagaa gcacatacct tcttcaagaa ctacgtcgtt tccttccatg atttgtggaa

577861 attcgtgaac gttcatccca ataccatgac caagacggtg aataaagtat
tctccataac
577921 cagccttagt aataatatct ctagcaacag catcaagttc gctggcagta
atacctggtt
577981 tcgctgcttc aatagctgct tgttgtgctt cacggtcgac ttcataaatt
tctttttctt
578041 tagcagttgg ttcgccgtaa gcgacagttc tacttgagtc agaagcatag
ccgttgtgca
578101 tggttccaag atcgaataaa actaattcgt ttggttcaat tttgttcatt
gttggaccaa
578161 ggtgagggtt agctgcattt gtacctgctt ggacgatcgt ttcaaaacta
gtatgcatta
578221 caccttttttg aagttttagt tggtaatcaa tttgtccagc aacataacgt
tcagttacgc
578281 cgtttctaag agcattgaaa ccaatttgaa aagcaaagtc ggcttctgca
ccggcagctt
578341 ttaattgttt tacttcttct ggggttttac gtaaacgtgc ttcagctacg
aatggagaaa
578401 cgttattggt aaatgaagaa tcaggaaagg caatgcgtaa ttgttctaaa
cgatcaactg
578461 agagatgtga tttttcaatt gcgatattat taggtttacc gacacgttga
tttactagat
578521 cagcaatttt tgcccatgga ttttcatggt caagatagcc ataaacatca
ccatcccagg
578581 cagaattttt agcttcttca acatttaatt caggcgcaaa gataaatgca
ggcttgtctt
578641 taaaagcaag taaagcaaaa attctttcat gtggatccat gctatagcca
gtgaaataat
578701 taatactgat tggatcagaa atataagcaa cgtcattatt ggttgaaatt
aaccaatttt
578761 gtaacttgtc taagttcata attttggctc ctttttttaat atatactggg
agtataccat
578821 ataatatggt aaaataatga gtttttagtc gtaaacggtt gcaatgtgtg
aatattcttg
578881 ataagttata aaagagtgtt tttacaggag tgtgagaaat ttatgcaaaa
gcaagaagta
578941 actatttatg atgttgctcg tgaagcaaaa gtttctatgg ctactgtttc
aagagtagta
579001 aatggaaata ataatgtacg taaagaaaca cgtgaaaaag tattagctgt
cattgatcgc
579061 ttgcattatc aacctaatgc agttgctcaa ggattggctt caaagagaac
tacaacagtt
579121 ggcttaatta tgccagattt gacaaatatg cactttgctg aattatctaa
aggaattgac
579181 gatattgcta cgatgtataa gtacaatatc ttactttcca gtgttggtga
tactttgttg
579241 aatgaagatc aagtaatcca aaacttactt aataagcaag ttgatggtgt
aatttacatg
579301 tcaaacttaa tgagtgaaaa ggctcaagaa atcttcaagc gtacaaatac
accagttgtt
579361 ttagctggta ctgctgatgc taaccaagac tttccaagtg taacaattga
ttttaaggct
579421 gctgaaaagg aagctttagg tttattgttg aagaatggta agaagaacct
tgctttagtt

579481 gttggtgacg gtaaagctta tgttaacaag gacaaccgtt tcgttgctta taaggatttc
579541 atggaagaac ataatttgaa gaatgttcat atctatgaag atgctaaaac ttatgaagat
579601 ggctataagt tatttaagca aattaaagct gataaagtag atgggattat tgctactcgt
579661 gatgtaacag ctgccggtat cgttaatgcg gctgttcatg caggcgtaaa gattccagaa
579721 ggcttagaaa ttatctctgc tgcttcaact aatgttgcta aaattgttcg tccgcaatta
579781 actacagttc aacaaccact ttatgatatt ggtgctgttg caatgagaat gttaactaag
579841 ttgatgaacg atgaagaatt agatgatgct catgttatct tgccatatac tttgaagaag
579901 tctcgttcta ctaagtagat aagttaatat taaaaaagta ctgttactct gcggttgaaa
579961 aaacgtcgat gttaacagta ctttttgta ttgtgaattt aaatggtttc actagctgta
580021 tttttattt catttaaatt aactactttg ttagctaatt gttcgatttc gttccttaag
580081 tgatgagtta ccataatcac tgttttatcc tgtttagtca gaatcaaacg ttcgagctgt
580141 gtacttgagg ctttatctaa tgcagacgtt gcttcatcta gtaagtagat tggagtatca
580201 cgtaatattt ctctagcaaa tgcaatacgt tgcttttggc cgccagataa ttgctttcct
580261 tgatcatcta aaactgtatc aattccatta ggtagtttag taattaaatc ttctaggcca
580321 cattctttga ttaccttatt aattttctga gttgatatct tttttcctaa agttaaattc
580381 cattttaggg atgctgaaaa aatatacgga tcttgctgta aataagaaat tttctgatgt
580441 aggtcatgat ctgaaatttg atcgtaactc atattgccaa gattgatttc tcctttatag
580501 tctcttagta aaccaccaat tagatttaaa atcgttgatt taccagctgc agaatctcct
580561 gttagaatat atttattttg caatataaaa tctaaattca aattatggaa aattggttca
580621 tcactttcag tataatggta ggaaacattc tttaggttaa gttttcctag ttgccaatta
580681 gaaattgaat gagtctcact ttcattgtta gatacagatt taaattttac cattattggt
580741 tttatagact taaattcctg ccaattaaaa ctaataccgc ttaattcagc aaaaattgta
580801 ctagcaaaga attgtgcacc actaatcgtt cctactgggg tgagatgatg taagattagc
580861 caaccagttt gtcctaaaat aatgacttgg ctaataaatg ctaagccatt agttattgct
580921 tgagttattc cagcggtttt actgtagttt agtgtatgct tttttacatc gtctgaagaa
580981 ccatcgattt tttttacgat agtctgaggg agattagcta aaaagagtgt attgaagcca
581041 tttaatatat cattaattgt attaactaat cgttcattag catgagtata ttctaatgat

581101 cgtttagcta agattttaga aaacatattt ggcacgattc ccataataac
tgttaagaca
581161 acaacagtta ctaccaaact tatattgaaa tatgctagtg taagcatccc
aaaaactatt
581221 tcactaactt gtctgacaat cattaaaaag tcagtaattc cgaaattatt
aatagtagta
581281 atatcattag ttagccaaga actgtatgtt gctacagttt gtttatgaaa
agtagcataa
581341 tttgccttgg ataagtctgt tgcaatacta tttctgatta atttgtctac
attttgagat
581401 aaacgtgtct gttctacgtt aacaagacaa ttaacaatag cataaatgat
ataggctccg
581461 cccattattg caacccacat aaagaattca gcggcacgta atttagcaac
cgaactcaat
581521 gcattagcgc tagaaatacc tgctagtgtt aaaaatccag cattaacaac
tatgagaaag
581581 actactaata gaaattccca ttttaacttt ttgaaatatt ttagtaattc
cattaattct
581641 cctttaaata ttaaaatttg atttaaataa taatactttg attaatttta
ttcaatatat
581701 tttaataatt agtttgaaaa ttatagtttt agtcaaacaa aaaactgata
atcgttaatt
581761 ttgattatca gtttttattg attttaaaat tatatttttc tatggagcag
ttggaccatt
581821 attagtggct gtactgcctt cagtggtctc gctgctagta tttgtgttag
cagctgaacc
581881 ttcattagaa ctaatatttg aatttacatc atttgcagca ttgttgctct
cggaattact
581941 actttcagcg gaagaagatg attcacctgt ggaacttgaa cgatttcgcc
tagtagaagt
582001 tgatgatgaa gaaccattat tatttcctcc ttgaccagta cttcgagctt
gccgatcatt
582061 agctccgaca tttcggttag tcgaagacga ggaattatta tttgattcac
ggtaaacttc
582121 gtggtttttg ccgtaataat ttttagcatc ttctgaagtt gaaccatcag
cgacagcaaa
582181 taggctgttg atgtttttct tcttagaaat tgtcttgcca gtatagatta
atactttacc
582241 atagtcatta ttcttgcctt ctaagtagtc atagaagagg ttgtagtctt
tagttgatcc
582301 accgatacca aatctagcag tagcagctgg agcaggggac aaacttaatg
cagtagtctt
582361 tttgccagaa agttcaatat tatcaccatc atagcttaca gttcctggta
atgttccagt
582421 tcttgctaag actctatttt tataaaccga gcttggacga cttggtgcat
catttaattt
582481 aaaaattgat ggatcttctt cgtaaagtgc attagcgata tttgcccaaa
ggtttaagtt
582541 ggtctcacta gaattagaat ctaggttgta tgagtgaccg tagaagttgt
cataacccat
582601 ccaactagaa atcgtgattc caggagtaga cccattaaac caaatatcac
gataatcgtt
582661 actggttcca gtcttaccaa ttaagttctt gtaattaaac ttcaaagtac
cggttaaact

582721 tgatgcagta cccttagtta ctacttggtg aagcatcttc tgcataatgt aagaagtacc
582781 agtagagaag accttttgtg ggttttgctt atgcttataa attactctac ctgatggatc
582841 tcgaatttct tcaatgtagt aaggatcagc acgcttacca ttgttataga agttagagaa
582901 ggcactagca ttatcggcaa ctgaaaaacc ataatctgta ccaccgaggg ccaaacctag
582961 gttttcaaat tcgcttttac tcaagtttaa gccgagtttt ttcatatcct gacgcaagtt
583021 aatgttctta tcgttgacta acttactgta taagtttacg gcaggtaagt tgtaagattt
583081 acttagagct tcttgagctg agataaaccg gttttctaca gttgaattat agtcagttgg
583141 aatatagttg ccaaatctag tagggaaatc tgccagtgct gtttggctag aaatcagctt
583201 atgttcaatt gctggaccat agactaggta tggcttaata gatgaaccag gtgaacgata
583261 agtatcaaaa gcgtggttaa tttgagagtt cttaaagtca actccgccac tgaatgctaa
583321 taccttgcca gttgcgttat caattacgac actaccattt tctacatgct cagtagtatt
583381 aacccacttg ttagtagatg agtcaaaatc acgagatgtc ttatcttgac catatttatt
583441 ttgcttaact atacgttgca tggtttgata aagtggctgt ctaatcgtgg tcttaatatg
583501 atagcccttt tgatgaagta attcagttgc actagttagg tattgattat aacggttagt
583561 atcttgttta acatctgata cctttaaacc atcttgctca attaattttt ctgctaagag
583621 actggtactc ttattcatta ctagattata taagtagcca ctttgcttct tttgtttgga
583681 tgctttttca ggagcaagaa aatcagcgcg taaatcatat tttttagctg caagatagtc
583741 ttttttacta atatcgccat ttcgatacat ccgaaataga acaatatctt ttctgcgcat
583801 tgctaaatca aggtcttttt taatttttcc gtttaaacga tatggggtat aaatagatgg
583861 actttgaggt aaaccagcaa tgaaagcagc ttgtggcaag ttaagttcag aaatagactt
583921 accaaatata ccttgtgccg cagttttaat acccacaatg ttttctccac ggttgttttt
583981 accataagga gcagcattta aatatgaacg gagaatatct tcttttgaga aatgcttttc
584041 gatcttatga gcataaaaca tttcggtaac ttttcttctc caagtggtct gactagttaa
584101 aaactgcatc ttaaccaatt gctgagttaa agttgatcca ccagtttgta caccaacacc
584161 agttagttca gaaaggacag ctctaactag ggatttaggt aaaactccct tatgaatata
584221 gaaattttca tcttcagtag ctgttacagc attcttaact aaaggggtta gttcgctttc
584281 acttgccttt ttggtgactg tatctgagcg aactgtagca atttttttat tgccagcata

584341 gtaaagtgtg gctgaatttt gtgcatggtt tatttgctga tttaattcgc taattgttgg
584401 aatgggctgc tggcgaacaa tgccaagggc gtagccacca ccaaaaccaa ttaggagaaa
584461 taaactaaaa actgcaatta agatcaaata atggaaaaca cgacgtaaag ttaaataaac
584521 gatgccgctg taaaatttcc attggctttc gtctgattcg ctttgaagtg ttttaacttc
584581 tggccccgca gttaggaact ctatgatttt ttcttttaaa tttttcacag atcaggatcc
584641 tttgttaatt actttccccc tattatagca aaaaaaggac tagcattgac gctgtccttt
584701 tttacgaatt tattaatatt ttgatttatt tagttaattc atagattgct tcagcataaa
584761 tagcaattga attaatcaaa tcttcaacct tcatgtattc atttggttgg tgcataacaa
584821 gtggggcacc ttctggttga gcaccaaagg ctacaccatg tttaaataaa cgaccatatg
584881 taccgccacc aatgataact tcatgacctt tcttgccagt ttgcttttca taaactgaca
584941 gaagagtttt aacgattgga tcgtctccag gaacataatg aggagcttct gctgagccat
585001 caactctggc atctaagata ttgccaaatt tttcgttgat gtttttaacc atttcttctg
585061 gttcaatacc ttgtgggtag cgaacgttgt ttagtaggta tgcattttga ccttcaaagt
585121 taaacatact tggtgaactg gttaagtcgc ccattaagtc gtcatgatgg aaaataccta
585181 acttcttacc gttaaagtct ttatgttcaa cagttgcaag gaagtgtaag aaattcttgg
585241 cttgaccatc aaaattaaga ctatcaagga aaagagcaag gtaagtagca gcatttctac
585301 cagtttcagg agctgaagca tgagcaccgt gaccggttag agttaatgaa agacgaccgc
585361 taagcatttc tgcttttcct tcaagtttat gttctttaag gaataagttg aatttttctt
585421 tgatgccatc aatatcgcct tcaagttgag catgagcggt ttgagggatg acattagttg
585481 caattcctga ttggaatgtc caaagtttaa cgtcgccttg gtttggatca tctttaaagt
585541 caagttttaa ggtaacaatg ccttgttcac cattgataat tggaaattct gcgtcaggtg
585601 agaaagcaac atctggggca ggttgatgct ttaagtagta gtcaattcca acccagttag
585661 tttcttcatt agttccaact acgaagtcaa ttttcttttt aggtttaaag ccgtgttctt
585721 ttaaaattag catcccatag taagctgcta atgatgggcc tttatcatcg gcactaccac
585781 gtccataaat tttgccatct ttgatagtca tcttaaatgg atcggtcttc cagccttcac
585841 cagcaggaac gacatccatg tgaccgataa taccgacacg cttgtcgcca gaacccatgt
585901 tgattcgacc agcatagtta tcaaagtttt cagtatcaaa gccgtcacgt ttagcaaatg

585961 ataaaaactt tttcatagcc ttaactggtc cgggacctac aggatattct ttgctagtat
586021 tatttaaatc ttcagaagaa tcaattgcaa tcaattcttt taaatcattt aaaatagcat
586081 ccttcttttc ttgtgctaat tttttataat ctagttcttc cataacatac tcctttattt
586141 ttatctctga ttaaatatta cagcatttac atctgaataa gtcgaccaaa agcttttatt
586201 tgggactttt tcaactggta aatagggaaa tagggcaacg tttgtttgac gataagcagg
586261 atcaagtttt tgataattct tataggcaaa tgtattttta ttaaataagc cataatataa
586321 gactttagaa gacttaaccc agccatcatt agtaagaagc cataaatctt taactccgcc
586381 acttacacct agtattttat agtgttttct tggcataagt cgattctttt taccagattg
586441 cttatcaggt ttattataag tgaaaattgg attttgagta gggttaataa tgcttaaatt
586501 ccaatatggc agaatccatt ctggctgtaa gtctaaacta actaggtcgg caggaacaaa
586561 ttccttatcg ccaatttgat aaaaagtttg tcctgaggcg ataacagctg aagaaacatg
586621 aactgggtct aaaaagttaa gttcttttga tgttgtcact tttagatatg gattttcact
586681 aattgcagta ggcatccgac caaagtaaat tccatcttta ttgacgtcat actttttagt
586741 accagtttta gctaatttgt atttatagcc ttttgcgggg aagttagtga caactccatc
586801 aacatcattg ttgattaacc agttccatag cttcggcgtt tcattcattt cagcccaaac
586861 aaatactttt tgatgtagtg cgtgaagttg actaattaac ttgggatttt gagtaattag
586921 atcagatgag atattgatcc cgtttacgta acttaatact tcaaaattaa ttctttgcaa
586981 agaaccaaca ataaagattc ttggaacatc tggtagcagt tgactcatag tttttaagct
587041 cggtgcagaa aagctgtgaa tcattacccg atcttgcata tggtactttt tgatactggc
587101 agcaagtaat tcttccatat ttttaggact attatgttta gttttttggg tttcaagtag
587161 aaatttagtg ttaggtttat ctttgtagta atcaaataat tgatctaaag agataattga
587221 ttcaccgtta gcttgtttta aggtgtttaa ataggagaaa ttattttgtg atacaataac
587281 cgatgatcca acaactcggc ttaaatcacg gtcatgagaa acaactaaga cgttatcttt
587341 ggaaacatgc aggtcaagtt caacatagtc cgcgccatcc gcaaaagctg agttatcact
587401 ttgaattgtt tcttctggat atttactggg attaccgcgg tgaccaatga ctaggaatcc
587461 cgattcaaaa ataaaaatca aactaaaaaa aagggctagg aaataatgcc tgcttgtttt
587521 catgtgtaca cctctattct ttttaacaat agcatggatg tatacttttt tctataatta

587581 atatagtagt taataatcat tttaagaaag tagatgctga aatatggctt
atgagcaact
587641 agatttaatc gaagaagtta ctcgcaatga cggatctaaa tattatgaaa
tttcaaatat
587701 tgatcaaaat ggtatcgctg aattagccgt agaccgtgga ttaataaaga
aagtacggat
587761 acttcagcta aatttggcta gaacaaaggc acttcaatta tacgaagaat
atattaataa
587821 aacatatcaa ctagaaactt taactaatga ggatgattgg aaagatccgc
agtgggttga
587881 gtgggaaaaa cctaaaggaa aagttttaga cgcctttaat acagtattaa
aagccaatca
587941 tattggttag aagagagtaa agtatggaaa aattacgtat tgttcatacc
aatgacttac
588001 attctcactt tgaacaattt cctaaaataa aaagatattt acatcaagcg
caaaatgata
588061 aaagcgttga tcaaactttt acctttgatg ctggcgactt tatggatcgt
tctcatcctt
588121 tatcagatgc aactgagggt caagctaata ttaaattaat gaatgaattt
aattatgatg
588181 cgatcacaat tggtaataat gaaggaattt ctaattcaca tgcagtttta
gaaaaattat
588241 ttgatcatgc gaattttcca gttattttgg ctaacttacg tgaagaagat
gaaagtatgc
588301 cgaaatggtg tacttcatat aaaattttg aaactaaaaa gaaaacgagg
attgcagttg
588361 ttggtttaac tgctgcttat ccaatgacgt acggccctaa ccattggcat
gtaaagttga
588421 tttctgatac tttagataga ttacttccta caattaaagg acaatatgat
atgttaatca
588481 ttgttagtca tattggctta aggatggatc gatatattgc atatcattat
ccagaagtga
588541 acttgattgt tggtggacat agccatgatt atttgcccaa gggcgagaaa
gtaaacaaaa
588601 catggattac tcagactgga aaatggggtc agcatgtcgg cgatattgcg
attcaattaa
588661 atgataacca ccagatagtg tcaattgtgc caaatactgt caaaacagct
gatttaccaa
588721 gtttagaatc ggaccaagct aaaattgctg cttggcgtaa gcaaggtgag
cagatgcttc
588781 aatcacgtca aattgcaaag ttacctgcta aatttaacaa tgataaaatg
gcggcaattc
588841 aagtttctct cgatgcaatt agtaattttg ctggaactga tattgcggtt
ttaaacagtg
588901 gcttgttttt aacgccattt catcagggaa ttttaacaga agctgattta
caagctgctt
588961 tgcctcaccc aatgcatgta gtgcaaacta aattgcttgg aagtgatgtt
tggcgcatgg
589021 taatggagat ggaaaagaat cgtcattttc taagtaagtt tgctcttaaa
ggaatgagtt
589081 ttagaggaaa aatctttggc gatcttgttt ataaaggtat tcaagttgat
catgcaagta
589141 gaacagtcta catcaacgga aaagagattg atcctgaaaa agagtatacg
cttgccttgc

589201 ttgatcatta tgttttaatt ccatttttcc ctaccatttc aattatgggc aaaaacaagt
589261 ttttgttccc tgattattta cggactgtaa ttggaaacta tttaaaagaa aaatacccga
589321 tagataaata gaaagaaaaa taatggagta taaagaagca gataaagaac aaccgcttta
589381 tcatcctttg gctcatgtaa ttgtcaatga agacacgata atgattaatg ggcgaaagta
589441 tgaaatttta gctaatgtaa aagatgctct agatattgaa ttgctaaagg aaaaatatga
589501 tccttacctt gatcagtacg attatttagt aggggatatt tcaagtgagc atttaagatt
589561 aaagggattt tatgatgaaa aagatcgcgt tgcaattgat aaaaaagcta atgcgattgt
589621 ggattattta gaggaatatt gtaatcccgg cagtgcgtat tttgttttac gactagctca
589681 agaaaatcag atcaagaagg ttgcgtttgg cagacaaaat aagagaaaaa acatgtctaa
589741 taatcgctat cattctaaac gtccctactt taaagaaaga cgtgtccaca aaacaagaat
589801 tggtggtcat aaaacagctg taaaatttca acgtggaaag ggaagtcata aaaatagggg
589861 cttcgtaatt aagaaaagaa agggctaatt gtggaacata aagattataa atgttactta
589921 attgatttgg atggaactat ctatcgcggt agcgatacga tcgaaagtgg cgtgagattc
589981 attcatcgtt tgcaagaaaa aaatattccc cacttatttt taactaacaa ttctactcgt
590041 acgccgcaaa tggtagttga taagttgcgc ggacacggag ttaatacaga tatttatcat
590101 atttatacgc ctgttttagc tacagagtct tacttgctag ctcaaaatcc agatactgct
590161 aagataccag tttatatcat tggacaaaca ggattagtgc aagggctatt aaaaaatgaa
590221 cgtttctact acgatgatcg aaatcctaaa tatgtggtag ttggaatgga tacggattta
590281 acttatcata aaattcgcgt ggctactcgc tcgattagaa atggtgcaac cttcattggg
590341 acgaatgctg ataaaaattt accttctggg gatgagctat taccaggtaa cggtgcattt
590401 tgcacaatgc tagaagtagc aacaggtgtt aagcctattt atattggtaa accgtcatca
590461 attattgtgg ctagtgcttt gaagatgcta aatgctcaag gcaaggatgc aattttggtt
590521 ggggataact atgatactga tattatggca ggcgtcaact gcaatattga ctctttgtta
590581 actttgactg gtgtaactac taagaaacaa ttggcagaaa gggataagca gccaacttac
590641 gttgtagaaa atctggataa atggaaacta tgaagaaaaa gccaatcgtt gcagttgttt
590701 acaattgttt gatggctctt gctactgcta ctttgggagc agtagtttta agctggccgt
590761 tattagcagt ttttgtaaaa atccaaaaaa caaatttaat agtaaaaaca accttagcaa

590821 cagtgatgaa gaactataca cagctcttag attatttatt gttgccattt aatgataaac
590881 ttcagatgtc taattttccg acttcttcaa gcgcagcgcg tcatttttat gagtgtaagt
590941 tattatttga attagcaata attgtttttg tagttggatt acttattctt atttcttaa
591001 aaatgagaaa gagaatgagt tatatttata tttcgagaac aacagcgcta atttcatga
591061 ttttacctgt gataatctta ccgtttgctt taatgaattt tgatgaattt tttatagcat
591121 ttcaccatct cttgttcaac aatggtgatt ggctgttcaa tcctgcaact gatcccatta
591181 ttaacgtatt aactgaagaa ttttttgctg gttgttttgc aactggcgga ataatttatg
591241 agttatactt ctcttgcttt attttgtcaa aaaaataaag gagctttaac gaagctcctt
591301 ttttaatact aattttttct ttaaccagat aattaagact ggcaaaaccg aaattaaaat
591361 gattgcaatc acgatcaatg agaaatgttc ctggacaact ggaatgttcc caaagaagaa
591421 tcctaatgca gagaatagag ttacccagag aaaaccacca ataaagttat aaatgatgaa
591481 agttcgataa tgcatcttac ttgcacctga tacaaatgga acaaatgtac gaataaatgg
591541 aataaatctt ccaattacaa tagtaatgcc gccgtgtcga tcgaaaaagt tttcagcggc
591601 ttttcgttta tcttgattaa ttaatttgtt gaaccaacta tgtttagttc cttcagcttg
591661 cgaccagtgt ccgatttcat agttaatcgt atcacctaaa acagcagctc ctatgaagat
591721 tagatagcaa acccagaaat gaagattata tttaggtgtg gctgccatag cacaacatgc
591781 aaatataagt gaatctccag gtagaaatgg aaagataacc agacccgttt cgataaagat
591841 aattgcaaat aagattaagt aagtccagtt tccaaacata ttcacaatgg tgactaaatg
591901 gtcatcaata tgaagaataa aatcgataag tcccataaat aaaaatatat tcctttcaca
591961 aattaaatac tggtagaatg aagagtcatt gtttttctg gaaataggtc ttgcataata
592021 ttgctaattg caatttgtgc ttcaccaaat cctaaagcaa tcattggtac tcttccagga
592081 taagtaatgg catcgccgat tgcatataca tggggaagat tagtatgcat ttgttgagaa
592141 acagcgatta gattatggtc taatttgatt ccccatttac gtaattgtcg attatcactc
592201 ttgaagccat atgcaactag tatttcatca acatttttag taacaaaatc ttgacttgca
592261 ccaactttat gcaaaactaa ttccattcta ttattgttta actgcacatc ttttggaaga
592321 taaggagtta aaagttcaac attttttaaa cttttaagct tgttgacgga gctttcaaga
592381 ccacgaaact catttctacg gtgaattaaa aatatatctg atgtggttgc taactctagc

592441 gcccagtcaa gtgcagaatc tccaccacca agaatagcta cttttttgtt
agcaaagatt
592501 tcaggatgct gcattgagta atggatatgt ttttgtatgt ctaaagtagt
tgataaagga
592561 agtgttttag gcttaaatgc tccaagccct gtagcaatta ataaactttt
aacttcatat
592621 gttttatcga ttaggatatt ttcgttttct aaaaaagaaa tctcttttac
tttatgagag
592681 agtatgaatt tagtattagt gttttgctca agtaattgag aaactagttt
ttcaccttta
592741 atagaaggaa aagcgggaat atccttaata ttttttgtg gataaagcat
attaatttgc
592801 ccgccaactt cgtctaatgc atcaaaagaa attgtcttta atccgtgtaa
attagcgaaa
592861 gaggctgcaa aaagtccaat aggacctgta ccaataatag ctaaatcata
tttttccatc
592921 tttataagaa atcctccata aaaactaagc atagaagtca tgtctttagc
gttaattaaa
592981 aagacagtta taattaatat tagtaaggga tgagatagta tgcaagttaa
aattggcgat
593041 atagttcatg gaagaattag tgggatacaa caatatggaa tttttgtaag
gctagatagc
593101 aaggtagaag gcttgatcca tatctctgaa atacatggtg gatatgttaa
agatatcgga
593161 agagagtatc aagttggcga aacaataaag gtacaagtta ttgatataga
tccatactct
593221 aatcagatta gtttatcaag aagagcagtc cttccagaag taaaagaagc
aagaaaaaag
593281 cgagtccatt tttggacttc aaaacgagtt aaaaaaggtt ttactccttt
aaaagaggta
593341 ctgagtaccc aaattaaaga agcaaaaaat agatattcga aataaagagc
aaataaacat
593401 gttaagtatt actgcttaac atgtttattt ttatatccga atttttaaaa
taaagattgc
593461 ttttaagttg gaaaaataaa agacgaaaat cagacaattg aaatctatta
atagttaata
593521 ttcggaatat atctttttgt taaaaattat attgacgaaa aggagaaaag
tctgtaaagt
593581 aatacatgct gtttgaggca agcaagaagc agagcgagaa gcttgtcaag
agagcagaaa
593641 gatgataata ttataaagct gtcatttgag agatatgaga aagaggaaaa
aagacttgtc
593701 aaaactcaag aggcatgata taatataaaa gttgtcacac ggcaacggta
gtaccttgaa
593761 aactgaacaa agtttcgctg aaagtgtgcg ggatataaaa a
//
LOCUS   Contig_2   151424 bp   DNA   linear   18-
OCT-2022
DEFINITION Genus species strain strain.
ACCESSION
VERSION
KEYWORDS   .
SOURCE   Genus species
  ORGANISM Genus species

```
            Unclassified.
COMMENT     Annotated using prokka 1.12 from
            https://github.com/tseemann/prokka.
FEATURES             Location/Qualifiers
     source          1..151424
                     /organism="Genus species"
                     /mol_type="genomic DNA"
                     /strain="strain"
     tRNA            53..127
                     /locus_tag="OCPHGMPK_00563"
                     /product="tRNA-Asn"
                     /inference="COORDINATES:profile:Aragorn:1.2"
                     /note="tRNA-Asn(gtt)"
     tRNA            148..239
                     /locus_tag="OCPHGMPK_00564"
                     /product="tRNA-Ser"
                     /inference="COORDINATES:profile:Aragorn:1.2"
                     /note="tRNA-Ser(gga)"
     tRNA            354..427
                     /locus_tag="OCPHGMPK_00565"
                     /product="tRNA-Glu"
                     /inference="COORDINATES:profile:Aragorn:1.2"
                     /note="tRNA-Glu(ttc)"
     tRNA            449..524
                     /locus_tag="OCPHGMPK_00566"
                     /product="tRNA-Val"
                     /inference="COORDINATES:profile:Aragorn:1.2"
                     /note="tRNA-Val(tac)"
     CDS             669..1877
                     /gene="metK"
                     /locus_tag="OCPHGMPK_00567"
                     /EC_number="2.5.1.6"
                     /inference="ab initio prediction:Prodigal:2.6"
                     /inference="similar to AA sequence:UniProtKB:P66767"
                     /codon_start=1
                     /transl_table=11
                     /product="S-adenosylmethionine synthase"

/translation="MRKEKRLFTSESVSEGHPDKVADQISDAILDAILAKDPNGRVAC

ETTVTTGLVLVVGEISTSAYVDIQSVVRKTILEIGYNRPELGFDGNNCAILVDIDEQS

SDIAGGVNESLETRENHEDKDDLDKIGAGDQGLMFGFAIKETPELMPLPISLAHSLMR

RVASLRKEGRLDWLRPDAKAQVTVEYDDDNKPKRVDTVVISTQTDATVTNDEIREAMI

DMVIKKVIPSQYLDENTKFLINPSGRFVIGGPKGDSGLTGRKIIVDTYGGYARHGGGA

FSGKDLTKVDRSASYAARYVAKNIVAADLAYQCEIQLAYAIGVAHPVSIMVDTHGTSK

VSEDLLVEAVRNVFDLRPAGIIEMLNLRRPIYRQTAAYGHFGRTDIDLPWEHTDKVEA
                     LKNYVSEHAK"
     CDS             1867..3324
                     /gene="bmr3_1"
```

/locus_tag="OCPHGMPK_00568"
/inference="ab initio prediction:Prodigal:2.6"
/inference="similar to AA sequence:UniProtKB:P96712"
/codon_start=1
/transl_table=11
/product="Multidrug resistance protein 3"

/translation="MQNRSNTKIVTIAIFLTTFMTAIEGTIVSTAMPTIVSDLNGLEI

MNWVVSTFLLMTAVSTPIYGKLADSIGRKPVFLFGIAVFVVGSALCGIAQNMVELILF

RVIQGLGSGAVQPVAVTIIADLYTLEKRAKMLGLNSGFWGVASVIAPLLGGFIVQHLS

WHWIFYINVPLGIVAFLLVVFFLKERKTSSNSTLDLKGTTCLVIFLLALMVFLQELEN

GLNIVLLGLVVIIIVSAIIFFKAEKRAKDPIMPLDMLSSKEFTMINLITLLISGVVIG

FEFYIPTWMQGINATSASVAGFAVTPSSLMWIVGSFLIGGMLGRWGIKKTYDYMLLIL

IIADLALILVPIYTSFWVFCLIAAFNGTAFGAITTASQVRSQVLVGRDKIGVATSFNT

LMKYLGQTMMVSIYGITFNVVVAKQLSHHPNLTQKIMNDIVSADKAKHLAANLIPGLR
QVLLSGLKSVYVVSLIVIVLSLILNQLYKQKKIAK"
CDS complement(3374..4024)
/locus_tag="OCPHGMPK_00569"
/inference="ab initio prediction:Prodigal:2.6"
/codon_start=1
/transl_table=11
/product="hypothetical protein"

/translation="MKKKDNNIVVHDTLVPGFIFLILYVVWALLVIYKNPFIHSFDQT

IIHLISNNNPVNIAIATKLTVIGNTSTLTIATIVLFIGLLIAKKFSYAFFSAGLMICA

NGYNWITKHAVERHRPLVHHLVFADGYSFPSGHSVGSATFFGILIIILTILLVKNKFWR

TTLIIIWIIFPLFIGYTRIFCHVHYPSDVVGGWLEGTTFVLLGFSVLYHSYLKKHN"
CDS 4325..6739
/gene="leuS"
/locus_tag="OCPHGMPK_00570"
/EC_number="6.1.1.4"
/inference="ab initio prediction:Prodigal:2.6"
/inference="similar to AA sequence:UniProtKB:B8ZKS5"
/codon_start=1
/transl_table=11
/product="Leucine--tRNA ligase"

/translation="MYNHKTVEKKWQKYWAEHDTFKTGNDPKKKNYYALDMFPFPSGK

GLHVGHPEGYTATDIISRMKRAQGYNVLHPMGWDAFGLPTEQYALKTGEDPEKVTKEN

IANFKKQLNKLGFSYDWDREVTTSDPNYYKWTQWVFEQMYKKGLAYEAEVPVNWSPDL

GTVVANEEIVDGKTERGGYPVYRRNMRQWMLKMTAYADRLLEDLDDLDWPEPVKEMQR

NWIGRSEGAQVTFKVKDSDKTFDVFTTRPDTLFGVSYTVLAPESKLVQEITTPEQKAA

VDSYIKKIESKSDIERTDLNKDKTGVFTGAYAINPVNGKEVPVWISDYVLASYGTGAV

MAVPAHDDRDYAFATKFGLPINRVIEGGDLEKEAFTGNGKHINSDFLDGLDNEEAKKR

MIEWLEDHNAGQKKVNYKLRDWDFSRQRYWGEPIPVIHWEDGTTSLVPEDELPLRLPH

ATDIKPSGTPESPLANLTDWVNVVDENGRKGKRETNTMPNWAGSSWYYLRYVDPHNDK

ELADYDLLKQWLPVDLYIGGAEHAVRHLLYARFWHKVLYDLGVVPTKEPFQRLYNQGL

ILKNHEKMSKSKGNVVNPDDVIDEYGADSLRMYEMFMGPLDASIDWDDNGPASTKKFL

DRVWRLFVNDLDLKAIPQERIVDKNDGELDKVYAETVKKVTEDFDALHFNTAISQMMV

FINAAQKAKTIPREYAEGFVKLLAPVAPHMMEEIWQVLGHDESITYAKWPTYDPAKLV

ESTVEIMVQVNGKLRGKFKAAKDSDKDALEKEALSLDHVQKFLDGKDVKKVIVIPNKI
VNIVAK"

```
     CDS             6841..8499
                     /gene="murJ"
                     /locus_tag="OCPHGMPK_00571"
                     /inference="ab initio prediction:Prodigal:2.6"
                     /inference="similar to AA sequence:UniProtKB:O34674"
                     /codon_start=1
                     /transl_table=11
                     /product="Lipid II flippase MurJ"
```

/translation="MENNTKNTKETMVKGSAWMTFGSILSRILGALYIIPWYAWMGSH

GNIANALTAKSYNIYSLFIIISTAGIPGAVAKQVAKYNALNEYDIGRKLFRRGLILMA

IFGVICAAIMYFGAPILATDNIIGALLHGAESDPRQVAVMRSLSYAVLIIPILSIMRG

YFQGYADMMPPAMSQFVEQLARVLWMLLTAYIIMQVQHGSYVHAVVQSNLAAAIGAVF

GILLLVWFLYRRRNELNALMEHSNHAIKISTAGIFWEIINQSIPFIIIDSGITLFQLI

DQYTFHPMIAMFVHASSDQIESWYALFGLNANKLIMIIVSLATAMSVTAIPLLSGAHA

RRDYASISSQIENTLELFLFVMIPASLGMAAIATPIYTIFYGYDPLGSNVLYLSSFTA

ISLGLFTVLMAILQGLSENGLAIKYLVLGIIIKFAVQLPMIQLFKVYGPLLATNIGLI

ITIWLSLKHLKVRYKYNSGRTSRRFIGIAIFSMAMFVVVTGVVDFGGKLISPERRPTS
FILVILAVAVGGILYAFLSVKFGLAQKIIGPKVNRVLARLHIRI"

```
     CDS             8607..9740
                     /locus_tag="OCPHGMPK_00572"
                     /inference="ab initio prediction:Prodigal:2.6"
                     /codon_start=1
                     /transl_table=11
                     /product="hypothetical protein"
```

/translation="MNNMLVYAAKERKSFSEASLNEIDSMIFSQLAYCNFDLLPQITK

LTDLVSDDEIKTLTQGNLGGKNTEKLIKIMIKNPRFKNVSWHQAISKVDAKTQMQFNA

VTFYVATGEYYVAYRGTTATTIGWKENFNMSFRDWVPAHYFARSYYRKVRNKFPGKFY

IGGHSKGGNLAFAVALGLKEVDLANIVRIDCFDGPGFHNQDRLKNKFLKLKGKIHKYI

PQGSLIGILQDDLIGEKDYCTIVAAAGVNILQHDMFNWRVKNGKFVTLKQLDSTSEIS

WKAIAEWLKNTTDAERRDFIEMLYLTVSDTNQIYFRNLLTPKTTYKLATRLIKNSSVK
KEVWEPIIRKLFQAYVNSGTTTLSEQRRNNLMSFKNILDEQRR"
CDS 9812..10558
/locus_tag="OCPHGMPK_00573"
/inference="ab initio prediction:Prodigal:2.6"
/codon_start=1
/transl_table=11
/product="hypothetical protein"

/translation="MKKDTYMTLLGLRDTCYNNFNFDIDEKDFYLGTDTYKKIDKILK

NAQDEQLLIQFEEGTHLYQDYDPEYLSVAQKREVQHDINAARDFLDNYDGSNDDKHDN

YAAYLDDNSLSYSFGADSSLDEVQNNLNDIETDAVIVWQRRKDEYILLPHTIHSLERI

KKQISSNKHVDDKKLRYAMERLFDNITDFLKPFSDSELGKYENSISEELKDNFEHYAL
GVQTDARETAADKLTKYAVELAEKENNDKL"
CDS 10604..11449
/gene="nnrD"
/locus_tag="OCPHGMPK_00574"
/EC_number="4.2.1.136"
/inference="ab initio prediction:Prodigal:2.6"
/inference="similar to AA sequence:UniProtKB:Q833Y3"
/codon_start=1
/transl_table=11
/product="ADP-dependent (S)-NAD(P)H-hydrate
dehydratase"

/translation="MKSISKELMSEVIKKRESATHKGNYGRVLLIGGNKKYGGALIMS

AEGALNSGAGLTTVATDSVNISALHTRDPEIMALDWDKRDELKNLIVGSNVVVCGMGL

GLDDQARDILALIRDSISLKQVLILDASALDLISQQKDLLPVNSKLVIFTPHQMEWQR

LSKIKIADQNDQSNQAFLNELVPRKNAILVLKSNHTKVYDQAGNIYQNPFGNPGMAIG

GMGDTLAGIIGGFCGQYTPNLKTVAGAVGIHSITADEIAKKHYIVRPTQLSALIPAMM
RKYEK"
CDS 11455..12132
/gene="yciB"
/locus_tag="OCPHGMPK_00575"
/EC_number="2.-.-.-"
/inference="ab initio prediction:Prodigal:2.6"

/inference="similar to AA sequence:UniProtKB:C0SP99"
/codon_start=1
/transl_table=11
/product="Putative L,D-transpeptidase YciB"

/translation="MKKISKYQNSVLIIGTILTILVFAIILFPHRNVSLASPNKIVNS

KVTKKVAQKVKKVTHKDSNLPYPDPKDLQPAGSWKVKSESKAHPDLVRLNNLWIRVSI

KGNRVYIMDGNKPVYTMLASCGVYHNGKSATPTGTYAVEAERGATFFNQSLQVGARTY

ISWHGHGTYLFHSVPTDGNNKIMKNEAKKLGKTQASHGCIRLSIPDSKWLYEKLPVGT
KVVVKDE"
CDS 12142..13095
/gene="dacA"
/locus_tag="OCPHGMPK_00576"
/EC_number="3.4.16.4"
/inference="ab initio prediction:Prodigal:2.6"
/inference="similar to AA sequence:UniProtKB:P08750"
/codon_start=1
/transl_table=11
/product="D-alanyl-D-alanine carboxypeptidase DacA"

/translation="MQRKKLGDEMRHAKKKSRKKFLWIIGIAIIIICFFLWKNDHGPN

GMPSNYHADQVNLNVKAAVAIDAKNENVIYAKNANQSLPIASMTKLLTVYLTLKAIKE

KKISWDTTVSPTQEIINLSSNPDYAGVPLSLGQKYTVRELYDAALIKSANNAARMLAI

AVSGSETNFLNQMRQQANKWKLYNVKLVTVDGLPEKNKNFLGMTTTIENKMSANDMAI

IARKLVTDYPEVLSTTKVAKSDFRNTLMTNSNKMLSGLSDYDPNYPVDGLKTGTTDGA
GACFTCTMNKNNKRVITVILGAQNDNERFSETKKLLNYSFN"
CDS 13376..15052
/gene="argS"
/locus_tag="OCPHGMPK_00577"
/EC_number="6.1.1.19"
/inference="ab initio prediction:Prodigal:2.6"
/inference="similar to AA sequence:UniProtKB:Q54869"
/codon_start=1
/transl_table=11
/product="Arginine--tRNA ligase"

/translation="MDFKQKVVDLVSEQVDLPKEKIAMLIERPKNAKMGDYAFPAFAL

AKIEHKNPALIAKDIAEKISDDNFTSIQAVGPYVNFAIDHAKLVNATLNDVLTEKDHF

GDQQLGEGNVPIDMSSPNIAKPMSMGHLRSTVIGNSIAKTLEKVGYTPIKINYLGDYG

TQFGKLITAYRLWGNEEDVKKDPITNLFHYYVKFHEEAEKDPKLDDEGRAAFKKLENG

DEEEIKLWKWFREVSLQEFNRIYKELGVTFDSYNGEAFFNDKMQPVVDELREKGLLEE

SRGAQVVNLGEDENPALILKSDGSSLYMTRDLAAALYRKKEYDFVMSLYVAGGEQSGH

FKQLKQVLKKMGYDWADNIHHIPFGLITQGGKKLSTRKGNVVFLDKVLKDAVSLAEQQ

IEEKNPNLANKDQVAHDVGVGAVVFHDLKNDRMDNFDFDLEEVVRFEGDTGPYVQYTN

ARAQSILRKANKEISMDNLSLNDDWSFAVAKALADFPAIVAKASEKFEPSIIAKYALD
LSKKFNKYYANVRILDEDDQLNARLALVQATSIVLTEALRLLGVNAPKEM"
CDS 15164..16081
/gene="fba"
/locus_tag="OCPHGMPK_00578"
/EC_number="4.1.2.13"
/inference="ab initio prediction:Prodigal:2.6"
/inference="similar to AA sequence:UniProtKB:Q5XA12"
/codon_start=1
/transl_table=11
/product="Fructose-bisphosphate aldolase"

/translation="MAYLVNGNDIFKAARENHYAVGAYNTNNLEWTRALLRGAKETRT

PLLIQVSTGAAKYMGGYKTVKDLVLNEMDNMDIDVPVILNLDHGDYESAKECIALGYS

SVMFDGHNLPTDENLAKTKEIVKLAHERGISVEAEIGKIGENQGADGGELASVEDAKT

FVAAGVDKLACGIGNIHGVYPEGWKGLNFDRLKEIAEAVPGEPLVLHGGSGIPQDQIE

KAIKLGIAKININTEFQLAFQAATRKYIEDKMDLDKGNKGYDPRKLLRAGTDAITDSM
KEMISWMGTAPIDSKESSVKFDEASLNEE"
CDS 16151..16468
/locus_tag="OCPHGMPK_00579"
/inference="ab initio prediction:Prodigal:2.6"
/codon_start=1
/transl_table=11
/product="hypothetical protein"

/translation="MRVEFYEKGCKSFFKKYNKQKDTIVEFVEDTIDKEVASGMTKVK

LATRKRIKGRNIYEFRLNVGTIGSIRIAFSIFDKKAIVYFISKNIQKSAFSKDFEKII
SKL"
CDS 16518..16787
/locus_tag="OCPHGMPK_00580"
/inference="ab initio prediction:Prodigal:2.6"
/codon_start=1
/transl_table=11
/product="hypothetical protein"

/translation="MKILKIKDLDQEIENKLLVIWESSVRATHNFLSDPEINNIKKYV
PQALKGVAHLVIAYNDANESVAFMGINDQKLERLFVAANLTASII"
CDS 16942..17274
/locus_tag="OCPHGMPK_00581"
/inference="ab initio prediction:Prodigal:2.6"
/codon_start=1
/transl_table=11
/product="hypothetical protein"

```
/translation="MSLTVNLYYTGKNGSARKFAEEMESSGVADRIRQEPGNEKYDYF

IPMNDPEMILLIDSWKNQKSLDAHHASPMMKELADLREKYDLHMRVERYISDENGMPD
                     TDQKFIRK"
     CDS             complement(17362..17469)
                     /locus_tag="OCPHGMPK_00582"
                     /inference="ab initio prediction:Prodigal:2.6"
                     /codon_start=1
                     /transl_table=11
                     /product="hypothetical protein"
                     /translation="MKLLLILGVSAMAVLAANAAIVNKPVPVPVKVRRK"
     CDS             17712..18248
                     /gene="hpt_1"
                     /locus_tag="OCPHGMPK_00583"
                     /EC_number="2.4.2.8"
                     /inference="ab initio prediction:Prodigal:2.6"
                     /inference="similar to AA sequence:UniProtKB:P99085"
                     /codon_start=1
                     /transl_table=11
                     /product="Hypoxanthine-guanine
phosphoribosyltransferase"

/translation="MNNDIERILCTQSDLDSRMDEMAAELNVKYKDEEPIVVPVLNGA

MIFASDMIKRLNFKLTIDPIKASSYAGTQSTGEVKITQDIKSDVKDRPVIFMEDIIDT

GRTLQALSEVMKGRGAKSVEVVAMLDKPETRVVDFHADYYGFKAPDEFLVGYGLDYNG
                     LYRNLPYVGILKHEVYAK"
     CDS             18263..18931
                     /locus_tag="OCPHGMPK_00584"
                     /inference="ab initio prediction:Prodigal:2.6"
                     /codon_start=1
                     /transl_table=11
                     /product="hypothetical protein"

/translation="MRVNVLQHTPNEGPGSIKTWADQHHYDFYVYHPETFGKLPTVEE

TDLLIIILGGPMSPNDDLIWIKQERKLIKAMLDAHKPMFGACLGGQQIAKTVGAKILDA

PHKEVGWAPVYLKDQTIPNLPEKLTALHWHQQMFEIPEGAKLLFSSDLVKNQGFLLGD

NVIGLQFHFEPEEDNVREIAINDVDYPLENNDLHQTGEEIIAHGVPKENQKVMFKLLD
                     FITK"
     CDS             19007..19639
                     /gene="yhfK"
                     /locus_tag="OCPHGMPK_00585"
                     /EC_number="4.-.-.-"
                     /inference="ab initio prediction:Prodigal:2.6"
                     /inference="similar to AA sequence:UniProtKB:O07609"
                     /codon_start=1
                     /transl_table=11
                     /product="putative sugar epimerase YhfK"
```

/translation="MAKIFIFGGSGRVATDLIKNLVADGNTITAAARHPENIIKLDGV

TAEKLDLHADVDDIAKQVKGFDAIYFTAGSRGKDLIQTDAMGAIKTMMAAEKAGVKRY

IMLSSMLSLDINSWKKIPSLEDYLAAKFFADTYLMDSTNLEYTILQPGSLIEETGTGK
IQLNVEATDSNPIPDVAKTLAAILKYPNTIGKVIPMSSGKTPIDDALKEI"
CDS complement(19698..20156)
/gene="msrC"
/locus_tag="OCPHGMPK_00586"
/EC_number="1.8.4.14"
/inference="ab initio prediction:Prodigal:2.6"
/inference="similar to AA sequence:UniProtKB:P76270"
/codon_start=1
/transl_table=11
/product="Free methionine-R-sulfoxide reductase"

/translation="MSATTESNYQLLVKQAEALVDGESDWIANTANISALLFNSLDNV

NFAGVYRYENNELILGPFQGKPACVHIAVGKGVCGTTAKERQTQIVKNVHEFAGHIAC
DSDSNSEIVIPIFKKNGELWGVFDFDSTKIANFDELDQKYLEAISNVFKF"
CDS 20365..21360
/locus_tag="OCPHGMPK_00587"
/inference="ab initio prediction:Prodigal:2.6"
/codon_start=1
/transl_table=11
/product="hypothetical protein"

/translation="MIGKETQIRLVNKLENIHHVVVQASALDGSNVFVLQLLHRQSDV

IVYQTPNDSETVTFNEDHPILYLKGPNSAGTAGGHTQTWVQSGEDNKWFVGTKPKKHG

NTYWTTQIARVTVPGYQTQIFTSNTELPRLSYLNRAGSGYGDGSVAYPGKDLVRVEAA

VSPNKQYFLIASIDINHTGHFAIYNLDEVNKKLDQAEEKAEDVNIQSLNCLGAFNVPH

FNDQKIISIQGYGIDDNKNIYISSQPSPHTTFLGFPKQGKPREIVKIPWGISDPSKWS

VVNLDNSLKLDALNFCTEFEGIQVTSDCLYLTVAYHQRNSDLTTLMNRIYQVEKF"
CDS 21423..21989
/gene="tag"
/locus_tag="OCPHGMPK_00588"
/EC_number="3.2.2.20"
/inference="ab initio prediction:Prodigal:2.6"
/inference="similar to AA sequence:UniProtKB:P05100"
/codon_start=1
/transl_table=11
/product="DNA-3-methyladenine glycosylase 1"

/translation="MKLSRCSWGNSKNSLYQKYHDQEWGKLNLDSTYLYEMLVLESFQ

SGLSWETILNKRRNFRKAFANFDYHKVAEFNQDDFERLMQDKGIVRNRLKINAAINNA

KILVKLEKENRTFENFLTEFIPKPIMHHPQKMEDIPASDGLSTQISKEMKKLGFKFVG

```
                     PVTVYSFLQAVGLINDHLENCSFKNGGR"
     CDS             21989..22927
                     /locus_tag="OCPHGMPK_00589"
                     /inference="ab initio prediction:Prodigal:2.6"
                     /codon_start=1
                     /transl_table=11
                     /product="hypothetical protein"

/translation="MSLLLPTKQVVIMFILMIVGWICYQVKFLHEQTVKDLSKVLLYV

VSPCLIINSFRQSFSAARLLQFGLVFLLVIALFIFKIITSEFIFGKNLVKDRQKRTVL

RYAGTYTNAGFMGVPLVQAILGTKGVFFAVPYLIAYNIFMWTHGIRMFTQKKQSFRES

FRQAVINPNIIAAVIGMILFITQVKLPDVVSDPMNYIANLNTPLSMIVIGTNLGSINL

KADWQDKLAWSGVFVRNLFFPIVILGILYALPLPAIAKMTTLIMATCPVAGVVVLFSL
                     LSNFDVKFPTKLMCLSTLGAIITIPLVIFLATLIGL"
     CDS             22949..23731
                     /locus_tag="OCPHGMPK_00590"
                     /inference="ab initio prediction:Prodigal:2.6"
                     /codon_start=1
                     /transl_table=11
                     /product="hypothetical protein"

/translation="MKKDSYLAISKTNASIKWMLIASIIYLVVYSLAGRLEFQSQSIW

MQILYLIIVCVLAITFKKFYKTDKVFIKLPKEGKVKIITIIFCTLVILLYALLGSVVS

WAEVLNSMPEMMWGSICVALAAGIGEEVLCRVLLFNLFAKIFENKKYVLVWASLASSI

LFGLFHLINLTHGAAINATMQQVFYATAIGLIFSYIHIFTNRIWLCIVMHFLLDLQPN
                     IGTMEAQASPWGLILLIFGTAMIVSLFSIYVFNRRANKVFEH"
     CDS             complement(23771..24526)
                     /gene="gla"
                     /locus_tag="OCPHGMPK_00591"
                     /inference="ab initio prediction:Prodigal:2.6"
                     /inference="similar to AA sequence:UniProtKB:P22094"
                     /codon_start=1
                     /transl_table=11
                     /product="Glycerol facilitator-aquaporin gla"

/translation="MEHSWMLKYFAEFFGTLILVLFGNGSVANSFLKGTTGNAPDGKA

NGGWILVAFSFGFGVMLPAMLFGSISGNHINPAVTVAQAAAGNFPWSQVAPYIICQLL

GAICGQLLVLAMYWPHFKESTDPDIVFSCFATSDCTNSKWNGFISEVVGTAVLMFVAI

GLYKGMFFKQAVDIANIGVGFLITALVMALGGPTGPALNPARDFGPRLVYSLLPIPNK
                     KGGAHWSYGWIPVIAPTLGAIIGIFLYKIPFGN"
     CDS             24727..26205
                     /gene="bmr3_2"
                     /locus_tag="OCPHGMPK_00592"
                     /inference="ab initio prediction:Prodigal:2.6"
```

/inference="similar to AA sequence:UniProtKB:P96712"
/codon_start=1
/transl_table=11
/product="Multidrug resistance protein 3"

/translation="MNKKQIRMVTVALMLGNVMAGLDGTITNTAIPAIVSALHGIQFM

GWIVAIYLLGMSVSIPIWTKIGEKITNKLAFEIALGLFVLGSTLEGLAPNIYFFLVAR

MIMGIGGGGMGSLPYIIAGYVFPNIKKRTQILGYLTASFNGAAILGPLVGGWLIDALS

WHWVFYINIPIGLVALLIALVYYKPVTPKSAPVFDLRGAFLLVSGLIMFLMGIQLLGL

TATWIVVGLILLSLVLLIFFFLHEAKAENPIIPLSIFRNRDLNGDLILFATTWGAFIA

VNTYLPMWAQALLGMSALMGGMTLIPNSVVEIIASQTVATIQEKIRTFTLVMIGIVTM

MISSGGLFLANNHTPLWVLIVVGAFSGIGVGFIFVALQVKVQIDAGMKYMATATSTSY

LIRILAQTVMAAVYGVIMNLALASGINSHSNITMKMMNELSDAKSAKLLPQHLLPEMR
EIFHSGIHEIMAVSFILLLIATVFNFYFNFKQPNKKIKNYSK"
CDS 26311..27762
/gene="bmr3_3"
/locus_tag="OCPHGMPK_00593"
/inference="ab initio prediction:Prodigal:2.6"
/inference="similar to AA sequence:UniProtKB:P96712"
/codon_start=1
/transl_table=11
/product="Multidrug resistance protein 3"

/translation="MIKKQVTMVTFAMVLANVMAGLDSTIINTAIPAIIADLHGIQFM

GWIIAIMLLGMSVSTPIWTKVGEKIGNKATFELSLLFFVLGSLFQGLADNMYFFLLAR

ALMGIGAGGMGSLPYIMAGFIFDNIKARTKILGYLGAAFSVAAIVGPLVGGYLVDSLS

WHWVFYINIPIGLLAILLSLMYYHEGKIRKTPKFDILGSFLIIVGLTLLLLGIQLLGL

TKPWSVASLIIAGLVLLVLFFMHEGGHPNPVVPISMFKNRALVGDFLLFIFSWGAFLA

INTYLPMWAQGLLGTTALIGGMTLIPNSLVDVVGTLVVNPLKARFNNRTLLSMGLICI

LISSLGLALAPQSTNIWWLAIIGTFSGFGVGFIFVLLQIKVQVDASEKNMAPATSLSY

LIRILAQTVMAAVYGVIMNVQLAKGVAENSGITMGMLNKLSDASSAKSLPQNLLPTMR
NIFHLGLQEIMWCATALLVIAIGLNFIFNKNSK"
CDS 28018..29604
/gene="yheI_2"
/locus_tag="OCPHGMPK_00594"
/EC_number="3.6.3.-"
/inference="ab initio prediction:Prodigal:2.6"
/inference="similar to AA sequence:UniProtKB:O07550"
/codon_start=1
/transl_table=11

/product="putative multidrug resistance ABC transporter
ATP-binding/permease protein YheI"

/translation="MSFQKLIKTNLLLFIVIIVLEILFAAGSATSSYIIQFAYNQLVK

NILLVFLLIIASSVFLSFLSYILSSLATYLFSKQTQKYIHSIRHKLISKYYHDKAPKV

AEMENELNSNLQVLTKNYADQSLSIIQSGFLLVTSISSLLLMNWMLTLLAIILSVITL

YIPRFTRQKASNATQKVVSRNSKYLLAIEEWFNGLEELRKYTAFNKLDLVMQKVSQNL

ETSFVKRKKVISVADFLNGCANSFSQIAITLLAAILFFNHQVTFGVIIAAGNFSSMIL

NCLLTITTSLTRIQSVQGLNKQIIESQKLASSHKETNTDEIYSISTHNLSISFKNGET

IYFPDIHIERGEKVLLSGDSGTGKSTLFKLILNKLQPTNGQVIFENKYGKEVSPDYAE

IGYIPQDGKLFPTSIINNIIMFDNKLKKLVKKAVENNDLSKDIASMPNGLETEIDLDT

NNFSGGQKQKIILARNEIHNFSIILADEATSAIDSKSSYRILKNLVSSNKTVIVVAHN
LTPAIEKLFSRKISLVNNKR"

CDS 29611..31182
/gene="yheI_3"
/locus_tag="OCPHGMPK_00595"
/EC_number="3.6.3.-"
/inference="ab initio prediction:Prodigal:2.6"
/inference="similar to AA sequence:UniProtKB:O07550"
/codon_start=1
/transl_table=11
/product="putative multidrug resistance ABC transporter
ATP-binding/permease protein YheI"

/translation="MSFKDLYAVNRIRMISLLILEFLTSGLIIGVSYINTYQITAIKN

RKWQQFIFLITLSLILFIISYAGLNVCQYWIEKQIQQYNHQIRFKIVNHYFYDNKTHS

TAQVQNRLTNDLNLIKDSKLAVYTDIPYYLAQIIFASIGLLLFHWSLLIVVLILGTLS

FYLPKLLRPAMQAAALKLSQANKQYLDTAEKWLDGLSELKKFSVGSQLSRIMDNASDT

LENANIARTGTMQELAVLNKATSALLQFALLAVTAILVTNHIVIFGVIVTVESFSSYI

NVSVKMLATELGQIHSVDRLSSEVNADTAVVEHTGDLQSPASLITKNVSVEFSNGKII

KFPDLNIKTGEKILLTGDSGSGKTTLFKVLLGKIKLHTGMINFKNKYGDDINVDKAKI

GYIPQDPILFPDSIENNITMFNSKLKNKVLDYVKDVSFESDIRKMPLGLKTELNLQKL

NISGGQRQKIVLARACVHDDDFILIDEGTSAIDKKATLDILKKLLDGPYTVIFIAHNF
NEEMNSLFDREIHLT"

CDS 32009..33316
/locus_tag="OCPHGMPK_00596"

/EC_number="4.2.1.53"
/inference="ab initio prediction:Prodigal:2.6"
/inference="similar to AA sequence:UniProtKB:B9E972"
/codon_start=1
/transl_table=11
/product="Oleate hydratase"

/translation="MTPEKDLQDKKINEVFDDEFFKSNFWLYWQTMFAFMPWASAMEM

RRYLMRFVQHVATLKNLSSLRFTKYNQYEDLIMPLISYLKKHDVKFHYDTIVDNIIVN

RTEDEKVATEIKMTEKGEQKVIKLTPNDLVFVTNGSITESTTYGDNTHPAEQKHELGP

SWQLWKNLAAQDEDFGHPEKFCENIPAANWVISATVTFTNDDIVPYIEKVNKKDPHSG

SIVTSGPTTLKDSNWLLGYSISRQPQFHKQKPNELIVWLYGLYSNTKGNYVKKTMPEC

DGIELCEEWLYHMGVPESEIKKMAIDATTIPNHMPYITSYFMPRALGDRPKVVPKNSK

NLALIGNFAETERDTVFTTEYSVRTAMEAVYTLLNVDRGVPEVFASAFDVRMLMNAMY
YLNDRKKLTELNLPLPEKLMVKEGLKKVKGTYVEELLKKYKLI"
CDS 33564..33992
/locus_tag="OCPHGMPK_00597"
/inference="ab initio prediction:Prodigal:2.6"
/codon_start=1
/transl_table=11
/product="hypothetical protein"

/translation="MVLMSLRKKLQLNAVYDGEVFCGMVCYYISDNTVYLAYLAVESE

LRGNGYGSKILHMLEEKYPDQQIVLDIEPLDPDAENYHQRVSRLRFYQKNGWRRTHQM
LVDADGEFEALVDQNHFDKKDFAKTLKQMSLGFYRFRIEK"
CDS complement(34132..34920)
/gene="ybjI_1"
/locus_tag="OCPHGMPK_00598"
/EC_number="3.1.3.-"
/inference="ab initio prediction:Prodigal:2.6"
/inference="similar to AA sequence:UniProtKB:P75809"
/codon_start=1
/transl_table=11
/product="5-amino-6-(5-phospho-D-ribitylamino)uracil
phosphatase YbjI"

/translation="MTKLIAVDMDGTFLRDDKSYDEEKFAKIYQELEKRNIIFTVASG

NQYYQITTFFKKFPAVVYVAENGALVRTKEKILALHAFSEENVKKIEDFLLKQAELQF

LVSGVECAYFPVQFTDAYYEISTKYYYRLKKIHDFSEIGDKVLKFSISCPDEKTDYYI

RFLKKNLGNYCNVTSSGHGDIDLILPGIHKAHGLAELGKVLGISLTEMTAFGDGGNDL
EMVKEVGDGVAMSNAAPILLKVANHTTTSNNEQGVLTYIKNNIL"
CDS complement(34946..35752)
/gene="yidA_1"
/locus_tag="OCPHGMPK_00599"

```
                     /EC_number="3.1.3.23"
                     /inference="ab initio prediction:Prodigal:2.6"
                     /inference="similar to AA sequence:UniProtKB:P0A8Y5"
                     /codon_start=1
                     /transl_table=11
                     /product="Sugar phosphatase YidA"

/translation="MIKLIALDTDGTLLNSKNKILPSTKAAIKKALDQGIKVVLCSGR

PIAGLAHFMKELGIEGSDQYAVTLNGAITRTADGKIMTQDLVSNKLYRELTKFAKEQK

VPFNIVDPDSRIITANHDVDYFELLQAWENTAPLFIRTPDEMPNDFQISKGCFVGDKV

LLDQFEPILRTKFDQDLYIVRADDHFLECLHPNVNKGNGLKELGEKIGISTNEMIAFG
                     DERNDISMFDIVGTAVAMGNGSQEAKDHANFITASNDDDGIAKALDKFIF"
     CDS             35858..36694
                     /gene="rhaS"
                     /locus_tag="OCPHGMPK_00600"
                     /inference="ab initio prediction:Prodigal:2.6"
                     /inference="similar to AA sequence:UniProtKB:P09377"
                     /codon_start=1
                     /transl_table=11
                     /product="HTH-type transcriptional activator RhaS"

/translation="MVEHEEVQNNQRLPFYFYRDNQVNEPEHWHQGIELNYLIKGKDL

RFALEGKTYHFNSGDIWLVNRRQIHSASGENEDWKYEGFIIDDDFLLSQYPTSINWNL

DLLGKNSTNNQTAYKELKKEVVELGELCQKSLTDARRFMSLSHLMRIIVLLDQNFNKK

EPVTRSPNLPLGDEIIKYINDHFQEDIQVSDITNKFNISHVTLNQQLKQSNNMSMGKY

LKLVRLMHARELLLSTNKSIELVAFESGFSNARVLNRNFKEWKHKTPTEYRNEFSKYF
                     IK"
     CDS             36666..36947
                     /locus_tag="OCPHGMPK_00601"
                     /inference="ab initio prediction:Prodigal:2.6"
                     /codon_start=1
                     /transl_table=11
                     /product="hypothetical protein"

/translation="MSLVSILLNKLLIFFSNKNTIRYTKIFSNLSKQDKKFVLAMAQS
                     SNERVTIKEIREKLDRPSNFIANYRRRLLDDEIIKATNYGEVAFTLPFF"
     CDS             complement(37047..38378)
                     /gene="pyrP"
                     /locus_tag="OCPHGMPK_00602"
                     /inference="ab initio prediction:Prodigal:2.6"
                     /inference="similar to AA sequence:UniProtKB:P39766"
                     /codon_start=1
                     /transl_table=11
                     /product="Uracil permease"

/translation="MKKMPKDTFHNDDAILDVYEKPELGQGILLSMQHLFAMFGSTVL
```

VPILVGINPSIALLSSGVGTLVHMLLTHFKIPAYLGSSFAFVATMQALMRQDGYPAIA

QGAIAAGLVYVIVALVIAKVGSDWVNKVLPPIVVGPIIIVIGLSLATTAANDAMMKGT

KYNLTYFGVAIFTLVMTLIFQMCFKGFTSLVSIMLGIVCGYALSCVLGIVDFSGVAKA

AWFSMPALDLPGISYHFKWYPAAILTMAPIAFVTMTEHMGHIMVLNSLTKRNFFKNPG

LHRTMMGDGLSTIIAGFIGGPPTTSYGENIGVLAMTKVHSVWVLAGAAVFAIIFSFVG

KVAAVIESIPMPVIGGISFLLFGTIASNGLKILVDDKIDFGEKRNMLIASVILVIGIG
GAYLQLGNFQLTSVALCTIFGMLMNWILPKKAASEKALEAEKKEKGQKYFN"
CDS 38796..39560
/gene="lacR_1"
/locus_tag="OCPHGMPK_00603"
/inference="ab initio prediction:Prodigal:2.6"
/inference="similar to AA sequence:UniProtKB:P67744"
/codon_start=1
/transl_table=11
/product="Lactose phosphotransferase system
repressor"

/translation="MLKRERLRNILEIVTDRRFVTVDELAKALQVSDMTIRRDLNELN

TTGKVMRIHGGAQVISQQDSVEKNYMQKREINISEKKAAAEVASKIVQDGETIYVGPG

TTLEFMVAKLNQKKLRVVTNSLPVFEAAKKNPNNYELILIGGSYRRVSGAFIGALANN

QLRGISFDRGFVGVNGIKDTSMMTANLEEGQTQGLGLNRSRKKYVVADYHKINRNDFY
EFYHLYDVDGLITSPGLDPDIVKHYSQITTLYQGSE"
CDS 39576..40004
/gene="lacA"
/locus_tag="OCPHGMPK_00604"
/EC_number="5.3.1.26"
/inference="ab initio prediction:Prodigal:2.6"
/inference="similar to AA sequence:UniProtKB:P23494"
/codon_start=1
/transl_table=11
/product="Galactose-6-phosphate isomerase subunit
LacA"

/translation="MKVVIGSDKKGFELKEKVKKYLEDGGYEVLDVTPEPAEDFVESS

LKVTHEVLDHGIKKAIMFDEYGVGSAMASNKVKGMVTADVNEERTGHMTAMHNGAKAL
AIGSGIVGEKLAESIIDYYLHTEYAGGRHQVRLDMLEKMI"
CDS 40018..40593
/gene="lacB"
/locus_tag="OCPHGMPK_00605"
/EC_number="5.3.1.26"
/inference="ab initio prediction:Prodigal:2.6"
/inference="similar to AA sequence:UniProtKB:P23495"
/codon_start=1
/transl_table=11

/product="Galactose-6-phosphate isomerase subunit LacB"

/translation="MFDNDRPQPEFVDPKIDKHTVVALGNDHIVTPIKMALSDHLKEE

GYQVLDFGTYDNTRTHYPMYGKRVAEAVADGRADVGIVMCGTGIGISTAADKNEGIRA

AMVGDVAQAKYAKRELNANVLGMGGIVLGRDFIFYIADAFLNEKYQPTEENKKLIEKI
DNIAKPNPDQKDNEHFFDEENKKWAEGVYHD"

CDS 40612..41292
/locus_tag="OCPHGMPK_00606"
/inference="ab initio prediction:Prodigal:2.6"
/codon_start=1
/transl_table=11
/product="hypothetical protein"

/translation="MSKLEEFRKANPQYKILTLDDPSFKEYGVLYTNDFNLDDVYKVM

EKVPIPETGMKYIANIPELADTYTMLAVKRDIFGEIPIDAGVTLGHSDTFTAFEYHQC

SEVNIMLDDVLMVLGKRETLEEKHFVDPNTEARMFYMPKGTIVELYNDTLHYAPIQIT

KKGYKVIVAVIHGTNAVLPEGVKSLNPRVVKCGKFQVVHPSRKDKIEQGYQVGLSGDV
IKTTPLDE"

CDS 41533..42024
/locus_tag="OCPHGMPK_00607"
/inference="ab initio prediction:Prodigal:2.6"
/codon_start=1
/transl_table=11
/product="hypothetical protein"

/translation="MTVATSLFAPDAVFTSDKTSKEEVLKEVYGKLLKAGYVKGNFLS

HIIEREEHYPTGIDTSPISKELPNVAIPHTEGEFVNARLIVPIALKHPIKFQNMIAPD

ESLEVKFLFMILNNDPEGQANVLAQIMGFLSTTPVEDLIRLFNLTSTKEIFDFMSENF
KQE"

CDS 42052..42366
/locus_tag="OCPHGMPK_00608"
/inference="ab initio prediction:Prodigal:2.6"
/codon_start=1
/transl_table=11
/product="hypothetical protein"

/translation="MAKEVKLLAACGAGVNSSHQIKDAVEEEMKKRGHNVHVDAVMIK

DVNEDMLNHYDAYLTIAKTDLAFEPKIPLIEAGPILYRIPAMAQPVYDKVEEVVKKVE
NE"

CDS 42391..43809
/gene="gatC_3"
/locus_tag="OCPHGMPK_00609"
/inference="ab initio prediction:Prodigal:2.6"
/inference="similar to AA sequence:UniProtKB:P69831"
/codon_start=1

/transl_table=11
/product="PTS system galactitol-specific EIIC component"

/translation="MNAIIDFANAIFKPLIDMGAPTIMLVVLTIIALCFRVKFSKALE

GGMKLAIALTAIGDVMNLLTTSFAPAMNQFVKNTGMHKDIMDMGWAPLATITWGSPYT

LYFLLVLIIILNVLMLIFKRTNTLDVDIFDVWHSAFVGLFAVFCGAPLWLATLLLILLIG

YLKIVNSDLMKPTFDDLLDAPSSNPMTTTHMNYMMNPVIMVFNKFFDKCLPWLDKYDF

DSAKLNEKIGFWGSRFAIGVYLGIFVSLLAGYNFTTEQGWKTMFTLSFTAGACIELFS

VIGSWFIASVEPLSQGISNFATSKFKGRTFNVGLDWPFIAGRAEIWAAANVLAPIMMV

EAMVIPGNRLMPLGGIIAMGVTPALLVVTRGKIIRMIIIGTIELPIFLWAGTLVAPFV

TQVAHQVGANIPAHTLVSDTTMEGPIEKFLGYLVGNAWKHQGMFIVYAILAIAAYILI
FWWYSTEMKKRNAKYAAAAKEA"

CDS 43847..44095
/locus_tag="OCPHGMPK_00610"
/inference="ab initio prediction:Prodigal:2.6"
/codon_start=1
/transl_table=11
/product="hypothetical protein"

/translation="MKTKKVSREEEARRKRVLYWTIFVIAINIPQLFFKNIFIEGFAM
IGTIYALYRLVVFDNKKNRLSRKYYDWKGRPLDAKNKN"

CDS 44588..45238
/gene="glcT"
/locus_tag="OCPHGMPK_00611"
/inference="ab initio prediction:Prodigal:2.6"
/inference="similar to AA sequence:UniProtKB:O31691"
/codon_start=1
/transl_table=11
/product="PtsGHI operon antiterminator"

/translation="MLSNVPLDFVTTSYDLIDQVQAKYHFAAEPYIYVTLTTHLFGAY

QRLINGDDDVNYLPDLSDNYPVAYQIADDVIKGFRNSLDISFPESEHKSIALHFINAH

TSGSVEEEKSQTENEEIIKVVQNELERNGIYRTQKNANDYDRLLVHLKYFINRLNNHE

PDSLPVSKGMIGEIEASYSKAWRMVQRIKELMQEKLEIKLSLSEQTYLTIHIQRLI"

CDS 45250..45606
/gene="lacF_2"
/locus_tag="OCPHGMPK_00612"
/EC_number="2.7.1.207"
/inference="ab initio prediction:Prodigal:2.6"
/inference="similar to AA sequence:UniProtKB:P11502"
/codon_start=1
/transl_table=11

/product="PTS system lactose-specific EIIA component"

/translation="MDAENQITKEDISMTGFSIVAYSGDARTYLMQAMDKASEGKITE

AKELVKKAEESINLAHNEQTKLLSKEAGGDDMDVTFIMVHGQDTLMTTMLLKDQMKTI
INLYERVNKLEGNSIE"
CDS 45657..47354
/gene="lacE_3"
/locus_tag="OCPHGMPK_00613"
/inference="ab initio prediction:Prodigal:2.6"
/inference="similar to AA sequence:UniProtKB:P24400"
/codon_start=1
/transl_table=11
/product="PTS system lactose-specific EIICB component"

/translation="MNGFTKTMDKMKPKFEKIAANPYVSAVRDGFIAAMPIILFSSLF

TLIAYVPNAWGFYWPKAVENALVLPYSYSMGLLALYVTATCAKNLTDYKNLKLPKTNQ

INPMNVILAAEISFIIIAIKVGKNGLDLTYLGTQGLIASYIVGLIVPNIYYACVKNNV

TIKMPDVVPQNIAQTFKDIFPMTFSVALFWIVQIILNQLFGANLSECVVKVLSPLFHA

SDTYGGLALVAGAMAFFWFVGVQGPSIVAPAVAAIETTNVGLNQQLVHAGMQASHALT

INCQDYVMNMGGTGSTFVVPFIFLLLAKSAQNKAAGKAAVIPGCFSVNEPILFGAPII

MNPVFFIPFLVTPMFNVCAYKFFVQVLHMNALYNTLPWTVPAPIGIIVSSGFAGLSFV

YVILTLVVDTLIWLPFFKFYDNDLYKQEQAKLAAEQASGVATATDDSTASLAATDKEA

KEGITKDTNVMVICAGGGTSGILAKALNKMAKERNLPLHAAARAYGQHMDIIHDMDLI

ILAPQMDSMKDNLKEIADHDGSKLVTTTGRQYIELTQKPDLAFKFVVDSLEGKNEDK"
CDS 47370..48797
/gene="lacG_2"
/locus_tag="OCPHGMPK_00614"
/EC_number="3.2.1.85"
/inference="ab initio prediction:Prodigal:2.6"
/inference="similar to AA sequence:UniProtKB:P67768"
/codon_start=1
/transl_table=11
/product="6-phospho-beta-galactosidase"

/translation="MKEFKFPKGFYFGGATAAYQCEGATKEDGKGEVIWDKYLREQGT

INPDPACDFYHRYPEDLELCKKFHINAIRVSIDWARIFPEGEGRIEPRGVEYYHKLFK

KCHEEGVEPFVTLHHFDTPQALQDKGGWLSQEMLDAFVEYAKFCFKEYAGEVRYWITI

NEPTSMAGQQYVGGTFPPAYKDQFDKCFKAEYNQNLVHARIVNAYKEAGYTGQIGIVH

ALQTVYPATDSEGDKHAAELKDVLENKFYLDGTLAGKYSKHTLALVKEIIDANGQQMI

DIKPEDEKVLLKAAKQLDFVGVNYYFSKFMKEYHGDTEIIHNGTGEKGTDVNRMKGVG

EEIHPKDLPATDWDWIIYPQGMYDQLKRVYDDYPLVHSIYITENGMGYKDKFEGEDKI

IDDTPRIDYLKQHMEEVAKAIEAGIPVNGYFVWSLQDMFSWTNGYSKRYGLFYVDFKT
QKRYAKKSAYWYKKLAETREIPPVD"
CDS complement(48945..49829)
/gene="cysL"
/locus_tag="OCPHGMPK_00615"
/inference="ab initio prediction:Prodigal:2.6"
/inference="similar to AA sequence:UniProtKB:P39647"
/codon_start=1
/transl_table=11
/product="HTH-type transcriptional regulator CysL"

/translation="MNDKDLLYFCKLVETQNYTVTANSFNVTQPTISMAIKRLQNRFD

DPLIFQKNRKSKITLSPAGELLYQKAKYLLKEMASIDYDVKHANIKKIRVAFSGIAGS

IYIPEIIEKFYKEDIVNLLDARLERSATAFKDLNNGDVDVAIYSWMVPINDPAYFIRN

LDRTELVIITGKDDPWKNKKVIDAQELRNRKFVARQKGYLTRECLEEEAKLGDFTPNI

LYTARTMKLMIDLVKRNVGIALAMENSVKDEKDLHIIRLRSGQKLWAYMQIAMRKSFI
PNKYQKQGIDILRKFHLN"
CDS 50040..51833
/gene="urdA_2"
/locus_tag="OCPHGMPK_00616"
/EC_number="1.3.99.33"
/inference="ab initio prediction:Prodigal:2.6"
/inference="similar to AA sequence:UniProtKB:Q8CVD0"
/codon_start=1
/transl_table=11
/product="Urocanate reductase"

/translation="MVYKTKVSWDATYDVIVIGFGGAGAGAARFAADNNAKVLLIDAA

PEGSEGGNTRYAGGAFAWGSNFDQLKEYYKQTYYPFEYDEEALDTFIKNVMQMKEYSK

KYFGIEAQPTGRRPNGEYPEYKDAASMQSQSMTKGMYNGGFWKLLRKQVFDRLDKIDI

WFESPAEHLIQDPDTNAVIGAVIKRNNREVNVAAKRGVVLSLGGFENNQSMVQDFLGQ

GSLAPIGTLYNRGKGIDLAVEAGARLWHMSNYDSHGLSLRQNEPREKFAYMINWKSLF

NGSIFVAGDDGTRYFREDEEDRHGYKYNHGNWVMIPNQNHPHIIMDQAQYEQLSNDQS

AKADQIKELISYAIKADSIEKLSKKIDAPYLKQAVEDFNFLTDVKKRDMFLNRKIETM

RSLKEGPYYAIPVRHNILHTHGGAQRNSKCEVIGLDGKAIPNLYEAGELGDIFATKYL

GANSIADLLISGKIAGTNAAKIKNNVDLPDAVTSASQVHEDLKSDAHQVKMHFEAGKN

QGIGSSDQGINDLPIVVRVTLDENQNLKNIETLQEKESGDLGGQAIPKLTQEMLDHNS
                     ADVDSVSGASTTSRAFKDAVKQAIENARKSK"
     CDS   51860..53275
                     /gene="yflS"
                     /locus_tag="OCPHGMPK_00617"
                     /inference="ab initio prediction:Prodigal:2.6"
                     /inference="similar to AA sequence:UniProtKB:O34726"
                     /codon_start=1
                     /transl_table=11
                     /product="Putative malate transporter YflS"

/translation="MLAKVNWKGFILPVIIGCVLWIITPFRPAAITPIAWHLFALFIA

TIVACITKPLPMVGSTLVAVTIGTLLHIFNIKEVSASFGNTTVWLIAMCLFMSAGFIR

SGLGKRVAYLFIRSFGKRTIGLAYSLSTVETLMAIAIPSNIARVNGIMYPIIDALSRQ

MGSDPKEGTQRKLGSYLIFNEYEVNIVTSTMFLTGLAGNMVALGIAKTQGITVSWMQW

FLAAIVPGLISLILVPFILYKIYPPEVKETPNANAWASKKLEEMGKMTPAEKIMACVF

VLALLLWLLGSSIGIDATLVGFIAVSILLISGVITVKDMMDQKGAWSLIVWLSIVMLM

SQKLMKLGFFPWFSKELGLMLHGVNWVWVLVILFLVYFYIHYMFPSITTQSSAVLAGF

LSIALGAGVPKLMAGLMLGFAGSIFCSTTPYSAGPAALLSTTGYVKSSDWWKLSAIFG
                     VFLNIVWLGGGLLWTKIIGFW"
     CDS   53410..54330
                     /gene="glsA2"
                     /locus_tag="OCPHGMPK_00618"
                     /EC_number="3.5.1.2"
                     /inference="ab initio prediction:Prodigal:2.6"
                     /inference="similar to AA sequence:UniProtKB:O07637"
                     /codon_start=1
                     /transl_table=11
                     /product="Glutaminase 2"

/translation="MIQLEELVKKNLPKEKEGKVASYIPALAEVNPKQLGVALYDLDD

GEIGEAGQSEVRFAIESISKVITLMLAIKKMGIQEVFKHVGSRQSGFAFDSILNMEIE

KAKYPLNPFINAGAIATTSMVVSKTGEDAFEEILSFARDICNDPNLYLDQIIYHSERR

TGNLDRSLAYYMESKNMMLGDVITSLDTYFKQCSMMVTAHSLANLGAVLANGGVKPWD

NKRIISDEEARCVKSLMMTTGLYNQSGTYSRLIGVPTKSGVGGGLMSAAPHKYGIGIF
                     SPALDKDGNSVAGLDLLRDIVEGLELDIFD"
     CDS   complement(54403..55374)
                     /gene="purR_2"
                     /locus_tag="OCPHGMPK_00619"
                     /inference="ab initio prediction:Prodigal:2.6"

/inference="similar to AA sequence:UniProtKB:P0ACP7"
/codon_start=1
/transl_table=11
/product="HTH-type transcriptional repressor PurR"

/translation="MIKLTDVAKKAGVSPTTVSRVLNNRGYISNATKIKVNNAIKELN

YQPNNIARSLGGKATHIIGLIFYDTSHPFFGELTSKLESKLFEKGYRVFLCNSANSPD

KEKRYLDMLAANKVDGIISGTHNLDILEYKNIQAPIVSFDRNLAPYIPVVSSDNFKGG

IKATKYLINQGAKHIHIISSHVRSHNPTDARVEGFFTTLKKYNLIPHEHAFSFTMSPT

IKKELIKKILQNEPIDGLFCTDDLTAILAYEAAQEVGIKIPQDLDLIGYDGTNLIRNY
FPQLSTIQQPIEEISSLLIELLLKQINGYTPKRNETFKLPVKLIVGD"
CDS 55609..57549
/gene="treP_1"
/locus_tag="OCPHGMPK_00620"
/inference="ab initio prediction:Prodigal:2.6"
/inference="similar to AA sequence:UniProtKB:P39794"
/codon_start=1
/transl_table=11
/product="PTS system trehalose-specific EIIBC
component"

/translation="MDHKQVAKRVLKYVGQDNIQAAAHCATRLRLVLKDDKKIDQSGL

DNDPDVKGTFETDGQYQIIIGPGDVNKVYDEMVKIANLKTATTDDLKKVAANKNHNVI

LDLFKLLSDIFIPIIPALVAGGLLMALNNVLTAQHLFAAKSLVQMNPQLNDFANFINM

LASAPFTFLPVLICMSATKRFGGSRILGATMGFAMVMPQLISGYAISTTPHIPTWNFF

GLHVMQAGYQGQVLPVLGVAFILASVEKFCHKHLKSAIDFTFTPMIAIIVTGFLTFAI

VGPVLRTCSDWITNGIIWFYNTTGWFGMGLFGLFYSAIVITGLHQTFPAIETQLLANI

SKTGGSFFLPVAAMANIGQGAATAAVWLTSKNKKQKSLAGSSAFSAMLGITEPAIFGV

NLKMKYPFICGAIASGISSAILGLFHVLSISMGPCSLISFICIKPGFIPQFMLGMIID

LIVGFSITYIYAKRYNAKHPVEDDTVEEIDVNDEVITAPISGQVESLRDTKDKVFSSE

MMGKGAAIIPESENVVAPASGEITVAYPTGHAYGIKTDEGANILIHLGIDTVNLKGKG

FSSKVKQGDRVNKGDLLGTYDFKLVEKEGYDPTVMVVVTNTADYAEVKRVTGKEVKEN
DNLIALTEPSKGKTSVATADPV"
CDS 57559..59229
/gene="malL_2"
/locus_tag="OCPHGMPK_00621"
/EC_number="3.2.1.10"
/inference="ab initio prediction:Prodigal:2.6"
/inference="similar to AA sequence:UniProtKB:O06994"
/codon_start=1

/transl_table=11
/product="Oligo-1,6-glucosidase 1"

/translation="MKWWQNSVIYEIYPKSFYDTNGDGIGDLKGVEKKLPYLKELGID

AIWLTPIYLSPQVDNGYDVADYRKVDPMFGSNDDLKELIRKSHDLNIKIILDMVANHT

SDQCAWFKESKKSRNNYFSDFYIWKDPKPDGSEPNNWAASFGGSAWTYVPERKQYYFH

YYASQQPDLNWENPIVRQYIYDAMRYWKAQGVDGWRLDVITQISKDLTFKDVPRVVDR

KYVPLKNTSGPHMHQYIHELNQEVLKPFEMMSVGEAPNSSIKEILDLTSPKRQELDMA

FSFEHMRIDKAAKENGALALKQPDFLQLKKVLSKMQYELNNKAWNALYFENHDRSRIP

SRWGNDTKYRYESATAFATILHGLKGTPFIFQGEEIGMTNPKYSVEEYEDIDLKNKYQ

EQVKTKHLLNANEFLAIEHKIARDNSRTPMQWNGEKNAGFTTGIPWYKINPNYFRINV

ESDKASEKSIFKYYQRLIKLRHNEEILTKGNYEELLRDDKNIFAYKRNLNRKSWLIIV
NMTDNILSLKSLEADLKRYSKVILHNYKGKITNNEFKPYEACILEKRL"
CDS 59258..61444
/gene="agaSK_2"
/locus_tag="OCPHGMPK_00622"
/inference="ab initio prediction:Prodigal:2.6"
/inference="similar to AA sequence:UniProtKB:G4T4R7"
/codon_start=1
/transl_table=11
/product="Bifunctional alpha-galactosidase/sucrose
kinase
AgaSK"

/translation="MILLFWRKMLKIDNNKNQILFHLYNTQISYVIEIIDGKFVTNRY

FGPKLPYFSGSTSLDNGKHAFSIYQQEDAFSVSSLPLEYSVSQSGDYRETSAELNDKF

NLPIIDMKYVRYDQEKVELSSLPHIRQAKGSSLNIHLIGNNGRLAITIHYFLLNDFPS

IIRWISYKNIGKSLIHIKKADSLQLDVPNREWKTLSFYGTHASEFIPSIKPIFDGKVE

INSTRGSSSPQHQPFIALIDKNFTLDRGKFISCNLIWSGNFRISIEKEQENYVRLNAG

VNNKLFNYTLRPSEQFITPQAIITTGQNGLGEMSRLSQEIISNYIVNSRIQQPLIALN

TWEMSYFDVTEKKCIKALNQAEKIGANLLVIDDGWFNNRNSEKGQLGDWIPDYNKFPN

GLKHIAELTHKKEMKFGIWIEPEMVTTSSDLFKKHPDWVLGLDSVERALYSRNQLVLD

LSKKEVQNYLINIISNLIIENRIDYLKWDFNRQLAPVFSQGRSALDQGKVGFDYILGL

YRILSSLREKFKNLIIENCAAGGGRMDLGMVYFTDQTWISDLTDALGRFRIITNMATI

YPIRIFSSHFSKSPNEQDGRILPIETRLVLSSLGSLGFELNLELMGEDQIDKIKSYVK

KYKEEYSLMHDSVCLPLTPLREKATMPMAIMLKDAKKIGVIYSYGATDAVHIPRWLPL
IFLDNKCDYKVNDDYSCSGVELNNAGLTIFPVTGDFCVDVEYLKKE"
CDS 61799..62419
/gene="ybjG"
/locus_tag="OCPHGMPK_00623"
/EC_number="3.6.1.27"
/inference="ab initio prediction:Prodigal:2.6"
/inference="similar to AA sequence:UniProtKB:P75806"
/codon_start=1
/transl_table=11
/product="Putative undecaprenyl-diphosphatase YbjG"

/translation="MKKYSKAIYWGLPISLILAISIILHLGWINAFDNFFEGLVHIVP

NLKGLMLKITFLADTKVDLVWMVLIAVILWFRKQRPLATNLVLTMITGDAVGWVIKHL

IQRARPVQHLAVDDGFSFPSGHTLGMGMIVIWIIMVLLPVVLKDRTKRFWIDFLLIVW
LVIVMISRVYVYAHYPSDVCGSVAIALTWVAVMQFVFEKIKQTVNK"
CDS complement(62509..63525)
/locus_tag="OCPHGMPK_00624"
/inference="ab initio prediction:Prodigal:2.6"
/codon_start=1
/transl_table=11
/product="hypothetical protein"

/translation="MELTGYWWPQVAANYSNNPPQGPRGSHCSGFVATGSATRDGKPV

IAHESFDDFWSGQYFNVCEEVHPTNGHAFKMQTVPGYIDSMTDFYVTDAGLGITETTI

AGFVGYDVNGMPEFIRARKATQYANNIDQWVKLVNEGNNGGYANIWLLCNINTCEIAR

YEQGLKNQELLESTDGFYYGCSACHNPRIRNLECVDNGYNDTRQQTGARRARFEQLLP

EVSGTIDDSVAKKILADKYDPYLGYQCASSRNICAHYDVDPQYYADDPHGVWNVPFFP

GGSCDGKCADATDIENLNMWGIFGRADGEPFDAKDFMKQHPEWNWQKGYLYDRPNQAW
TLFD"
CDS complement(63947..65647)
/gene="adeC"
/locus_tag="OCPHGMPK_00625"
/EC_number="3.5.4.2"
/inference="ab initio prediction:Prodigal:2.6"
/inference="similar to AA sequence:UniProtKB:P39761"
/codon_start=1
/transl_table=11
/product="Adenine deaminase"

/translation="MTQVDLLIKNAQVFNVFLKKFIHSDVTIKDDKFYWISDNLANVT

AKKVIDLKGKYLIPGFVDAHMHIDSSMTTPEIMGQTIGKYGTTTIIADDHEITNVAGI

KGLKNFINQPSPIDIYFGIPSSVPSTNSHMETTGGQIGPDEVKELLKDPRFICLGEVM

NFKGMTSPDNTLIKKIIKTCRNIHPTMPLEGHVPAYTGEDLTKVIYAGITTDHTQQTA

ELVEEKVRNGMFVEVQLKSMHQDVIDTIIKNDFFEHVALVTDDSMPDTLLHSHLNALV

KKAIDMGMTPEQAIYISTYTPARHMGLWDRGSIAPGRIADFIILDNPRTLSINTVYKN

GLPIENKFKKSDYQFLPSLRSSVKAPKLEINDFKLKTDLINNGQVVANVIQINPVGTF

TNHIKVKLEVKNHEVQWKKANLNLIMVQDRYGKNNGNYTLGLVDRGISKDGAVGATWA

HDHHNIMIMGTNIKAMLKIQHQLIDEQGGYVVAKGDEIIEDAPLPIGGVVSDEPMTKL

GEKISNIRHTLQDLGYKNTNEIMSFSTLSLLVSPQIKISDKGLFDVKSQEKIPLFEKL
"
CDS complement(65712..66761)
/gene="spuD"
/locus_tag="OCPHGMPK_00626"
/inference="ab initio prediction:Prodigal:2.6"
/inference="similar to AA sequence:UniProtKB:Q02UB7"
/codon_start=1
/transl_table=11
/product="Putrescine-binding periplasmic protein
SpuD"

/translation="MKFKKFYLWSLILITAGVAIGTSACSSKSSEKLVVSTFGLSTKQ

MRSDVLNPFSKKYNVKVSTQFGDSSTRLTQIEHNPNSNVDVIELAQNNAVTGAKKKLF

KKLDFSKIKNFKYLSKDQQQLAKKTNTVPYTVNSVGIIYNPKKVNIKDWNDLWSDKFK

QKISIPDMTTTFGPAMLYIAGDHAGTQVTKDDGTAAFKAMKELKPNIVKTYTQSSDLS

NMFKTGEISAAVVGDYAVGMLQATNPDLKYFVPASGTYANYDNVSILKNSKNTNAAYQ

YINYRLSESVQKKVANTKSLNNAPVNQQVNLSKKEAANKTYGDVAKRAKTIDFFYVNS
HISKWINQWNKIMNN"
CDS complement(66790..67833)
/gene="potA_1"
/locus_tag="OCPHGMPK_00627"
/EC_number="7.6.2.11"
/inference="ab initio prediction:Prodigal:2.6"
/inference="similar to AA sequence:UniProtKB:P69874"
/codon_start=1
/transl_table=11
/product="Spermidine/putrescine import ATP-binding
protein
PotA"

/translation="MANLEIKNLVKSYDGKNNILKNISFNIKDGDLVSILGPSGCGKT

TTLRIIAGLIPESKGQILLNGKQITNIPVYKRNFGMVFQSYALFPHLNVFDNVAFGLK

MHKVSKDEIKDKVTSILNTTGLYQLKEKYPAQLSGGQQQRVSLARALVTNPQLLLMDE

PLSNLDAKLRLKMREEIRNLQQKLKMTVLFVTHDQQECFAISDKVLIMKDGHIEQYDT

PQNIFHHPRTEYVARFIGYDNFISINSNERNDDHHILVKNTLLTTKESNSNAKILTIR

PENILLDQDNHSENSFLGTITSREYLGQSFKYTITSDLGIFQVEEARAKMRSSGDTVT
LTLPAKHLHPLSE"
CDS complement(67844..68632)
/gene="ydcV_1"
/locus_tag="OCPHGMPK_00628"
/inference="ab initio prediction:Prodigal:2.6"
/inference="similar to AA sequence:UniProtKB:P0AFR9"
/codon_start=1
/transl_table=11
/product="Inner membrane ABC transporter permease
protein
YdcV"

/translation="MLDRKNKVLTAISLIILALLLIPLILVIITSFNAESSISLPIKG

FTFNWYANIFQQPDFISGFESSLIVALLASSLALLIGIPAVYALTRFHIQHAAWFQSF

FLSPTLIPEIVIGFALYQAVVVTLRIPVFIGLVIGHFLLCLPYVIRLVTASMLLMDKH

IEEAGWIYGCSPRKGFFLIVLPNIKSSIIAAFMLCFINSFNNIPISLFMNGPSLTMLP
PAILNYLQNNYDPTVSAISVLLMIFTALMMVLVEKAIGINSLTK"
CDS complement(68625..69455)
/gene="potH_1"
/locus_tag="OCPHGMPK_00629"
/inference="ab initio prediction:Prodigal:2.6"
/inference="similar to AA sequence:UniProtKB:P31135"
/codon_start=1
/transl_table=11
/product="Putrescine transport system permease
protein
PotH"

/translation="MNRKSVIWLLLPAFMGVLLFALFPIIQIIIPTFQTRYGFWGLYS

TFLRNTYNWQIIGRTFFIAIVTVILVLILGLPISLWIARQKKSIKQILMILILFPMLT

NAVVRNFAWIIILGRDGIVNKFLLNLHLINHPLSLLYTNLAIIIGSIYLFLPIMIITL

TSNISELNLEVEEAAAVLGSTPLQTLFKIIIPQLTTGIFTGCILVFAGTMTAYTTPQI

LGGNRHLVMSTLIYQQAMTLGNWQKASVIALILIIISALSMWIMKKINYFLDRRPSRA
"
CDS complement(69778..70662)
/locus_tag="OCPHGMPK_00630"
/inference="ab initio prediction:Prodigal:2.6"
/codon_start=1
/transl_table=11
/product="hypothetical protein"

/translation="MVKKIKPWFGFTEDKVFGMVMENKDFCKYLLEIIIPDLKIKKID

WLDKQVEINNSERKNEAKEVRLDVLVTDHEGRVFNIEMQTTDQDDIGRRLRYYLSRLD

LRYTLNKGKTYRNLKDAFIIFLCNFKPKKDDKFYESHHTYSDQDRSKQLQDGVTKIII

NSQVSAEGQSEELKALAKLMNNEPVKLNKHFDYAQRRIKEINEDPEMREKIMLYETRM

LEREQAAGKAGYEQGMQHGIKQGLEQGKIDSAKIILENQLNNGSTLTQATEFVRNLKL
IPNEKLEKIIAMYDSHKN"
CDS complement(70847..72496)
/gene="ycaM_2"
/locus_tag="OCPHGMPK_00631"
/inference="ab initio prediction:Prodigal:2.6"
/inference="similar to AA sequence:UniProtKB:P75835"
/codon_start=1
/transl_table=11
/product="Inner membrane transporter YcaM"

/translation="MDELNALNPDKKFISWQTLFLMALCTVIGLDDIMFNFQNQGMAV

IFSWIVMVLFYVIPYSLMVGQLGSTFSTEGGGLSSWVRGTDGEFLGYFTAWTYWAASI

PYAVDSANEVIVDLGWALTGSEKFQDQIPNSLFALLTFVTFIVFIIIEHHFSNSMEFL

STIGGGLMVIMTILFVVLAFVGLAKSGGHMATTNFTWKDIFPKFDWHYFSTIAMLIYA

MNGAELVAPYVTKMKKPQYDFPKAMIALAAMTAFLTILGSFALGIYFNANHMPNDLKM

NGAYYAFKMIGHQYGWGNFLLYLWAWTSFFFNCALLAVLLDAMTRMLISDTGSRYMPK

FLRKKDKNGLPINGYILTVALSSFIMILGIFLPDMNDIFNWLLNLNAIVSPGVTCWIF

YSFMRVRKDSKKFPSKYVFIKNDKIAYAVGLILLLVTAIATIMGITPQDVPEGSSIWW

YELIINIVAVIVLIGLGAILPGIRHREVEYGIAFDKVQWISMIAITMCSIIFGIYLGG
LKHLSGRGIYIAVEGIFSLLIVWLIGRRKPNLANGFKAEED"
CDS complement(72687..73898)
/gene="yjhB_1"
/locus_tag="OCPHGMPK_00632"
/inference="ab initio prediction:Prodigal:2.6"
/inference="similar to AA sequence:UniProtKB:P39352"
/codon_start=1
/transl_table=11
/product="Putative metabolite transport protein
YjhB"

/translation="MTKKSDKPIWKKNLYVLSIAVFIAGIAFSEIMPFLPLYIDTLGH

FTHQQLNFWSGFIFSGVYFVSAIISPWWGKLADKKGRKLMILRASLGMAIVLGSMGLV

TNVWQLFFLRMLQGVFAGFVSNSNALIATETPKEKSGQALGTMASSFTAGNLLGPFLG

```
GALASAFSYRVTFFITGGLLLISFLLSLFFVHEEGFKPITEKKLDKASGVIATLRSPA

LIFGLLLTTLIIQAANNSINPIVSLYVKQLMNGNGNVVFVSGIIAALPGIATFLAASR

FGALGDRIGTHKIIIGGFIGATILFFLTAFVHNTTQLGILRFLVGFTDACLFPQVQTL

LTKNSPAAVTGRIFSWNQSAMYIGNIVGPLLGSFIAGISSYSMVFIVTAAIVVLNLIL
                     FNLNVVRNLAK"
     CDS             complement(73900..74109)
                     /locus_tag="OCPHGMPK_00633"
                     /inference="ab initio prediction:Prodigal:2.6"
                     /codon_start=1
                     /transl_table=11
                     /product="hypothetical protein"

/translation="MSKFNPLWEYIKNNKNSDFQLSFDQIQEIAGLPIDHSFLTYKKE
                     LKDYGFEVGKISLKNKTVNFKKLEE"
     CDS             complement(74102..74533)
                     /gene="slyA_1"
                     /locus_tag="OCPHGMPK_00634"
                     /inference="ab initio prediction:Prodigal:2.6"
                     /inference="protein motif:HAMAP:MF_01819"
                     /codon_start=1
                     /transl_table=11
                     /product="Transcriptional regulator SlyA"

/translation="MRKRPLANLLYHIGKLENLLINQRLSSINLRMTHAHALRYIQKH

PGCIQKEVAEYLFYQPASFTNVVKLLEKRQMIERRPDPKNGLKKQLFLLPAGKDVLDQ
                     VNQAFEEVNQLVGTVKSDTLTKLEEISNNLEKQVQRRHNDE"
     CDS             complement(74619..75206)
                     /locus_tag="OCPHGMPK_00635"
                     /inference="ab initio prediction:Prodigal:2.6"
                     /codon_start=1
                     /transl_table=11
                     /product="hypothetical protein"

/translation="MKKLVLVLFSTILLTACSNTSSNNTENKSSSSKSSITTSKKSKT

ATPKPNLNKKYPGFKLATIPDNFQGTWYQTDIYSTQARKFIITKHTIMDSVVYQKTDP

NLNLSHRSEKDNKTYAGNATMVSFEDKNGSQWLRARGFLDTVDIIYITGTFKGHRCLY
                     LSYSSGDIHSAIFKDRKAALKYRKYDFSQVTNPSN"
     CDS             75511..76596
                     /gene="ysdC"
                     /locus_tag="OCPHGMPK_00636"
                     /EC_number="3.4.11.-"
                     /inference="ab initio prediction:Prodigal:2.6"
                     /inference="similar to AA sequence:UniProtKB:P94521"
                     /codon_start=1
                     /transl_table=11
                     /product="Putative aminopeptidase YsdC"
```

/translation="MKKEQEIEMLKEFSDANSTSGFEEEFVKLFSTYAKNTADITVDG

MLNVYAAKKENKGDRPVIQMDAHSDAVGFITQAVRPNGLIKFVPLGGWVKYNIPALKV

KIRNREGEYIPGVVATKPPHFMTAAERNSIPEVADMSIDVGSLSREETIKDYKIDTGC

PIFVDVKCEYHEKSGLFFGKDFDDRFGAGAMVGVLDNLKGEETDFDVVAALSSQEEVG

LRGAYVTGRKIKPDLCIVLESCPADDTFEPDWLSQTGLKRGPMLRDMDTTFLPNPKFQ

QYACDLADKNRIPYTRSVRTGGGQDGAAIYYEYGAPTIVIGIPVRYEHSPYCFSAYKD
FKASVDLATAIIRDMTQEKLDSFKKVI"

```
     CDS             complement(76854..78140)
                     /gene="psd_1"
                     /locus_tag="OCPHGMPK_00637"
                     /EC_number="4.1.1.65"
                     /inference="ab initio prediction:Prodigal:2.6"
                     /inference="protein motif:HAMAP:MF_00663"
                     /codon_start=1
                     /transl_table=11
                     /product="Phosphatidylserine decarboxylase
```

proenzyme"

/translation="MKKDAKFNVGQWLPSDQEFYNNWLKQVLKEAEAEQDQTLLPPVQ

ALKDLIESDRYLWNLFHMMFEEVPAKYVETPMDTPAVHNYHQMLRVINRVMHRAPEFN

ATGLVGFPINAILDYPMATKAGYVAFMDPRVNQKLRDILDYWGRFLQSPDSTYVLNRS

KEGWLGDEALKSMCNAAYGENFHDIFACPNNDIDQQYGFKSWDHFFTRTFNPGVRPVA

NADDPDSIANACESAPYRIAHNLPLKAKFWIKGQPYSLIDLLHNDSLTSEFVGGTLYQ

AFLSALSYHRWNSPVSGTVVKAYNLYGSYYSETLPQGFENPNGPDPAAPNDSQAYITE

TASRAVIFIKADNPKIGLMCFVAVGMAEVSGNEITVKVGQHINKGDQLGIFHFGGSTH
VLLFRPGVNVKFNLHGQEPSLGSHNIKVHDEIARVE"

```
     CDS             complement(78166..79599)
                     /gene="aaxC"
                     /locus_tag="OCPHGMPK_00638"
                     /inference="ab initio prediction:Prodigal:2.6"
                     /inference="similar to AA sequence:UniProtKB:Q9Z6M8"
                     /codon_start=1
                     /transl_table=11
                     /product="Arginine/agmatine antiporter"
```

/translation="MTKTKSHKIGLFGLIAMVVGAMIGGGIFDIPQNMAASSSLGAVL

IAWILTGIGMFGLAFTFKILAEERPDLNIGIYSYARAGFGKYVGFNSAWGYWIEAITG

NVAYAVMLNDSFGRYFPVLLKHQWPTVIFGIILIWIYNFLVLNGVKEASFLNNITVII

KFISLGIILICMLIFFRFPTFFWGFWGQGLHLGNFVSQIKAPMLVTLWCFIGIEGAVV

ISNHAKNPKDVGKATVMGYLISLIAYVLISIVAYGIYHQPTLSKLTDPSTAYLMKNLL

GPWFIDFVTISVIISVGGSWVAWTVMMAQLPYAAAKGHVLPKFFAHENKKKVPSVSLY

ISSILMSLFMILVVTANNVYLACIDITGVIILPCYLLSSIYLWKIAQKREIFANDSRK

RNKSLFIGILSTIYCLWLLYAAGLNYLLISTIIYAVGIIFYYFARKEYDKKGTPLFNK
WELALAIIICILAVVSVYLLVTKKISL"
CDS complement(79634..80920)
/gene="psd_2"
/locus_tag="OCPHGMPK_00639"
/EC_number="4.1.1.65"
/inference="ab initio prediction:Prodigal:2.6"
/inference="protein motif:HAMAP:MF_00663"
/codon_start=1
/transl_table=11
/product="Phosphatidylserine decarboxylase
proenzyme"

/translation="MKKNIQYNVGQWLPSDQEFYNKWLKKVLKEAESEENQKLLPPVQ

ALKNLIESDRYLYNITQMMFDEIPEKYVDTPMGTPQVRNYRQMLLMLNRIIQRAPEFN

TTGLVGTPINAILDYPMATKAGYVFFMNPKINEKLRDILNYWGKYLQTPASTYVLNTS

KNGWLSDYALNEMSKAADGDNFTTIFKCESDDREKHLGFTSWDNFFTRLFNPGIRPVQ

DPEDPNSIANACESAPFRIARDLPMKAKFWIKGQPYSLMDLLHNDPWTSKFEGGTLYQ

AFLSALSYHRWNSPVSGTVVKAYNLPGSYYSESLHQGFANERGADPVAANDSQAFLTA

TATRAVIFIKADNPKIGLMCFVAVGMSEVSSDEITVRVGQHVNKGDQLGMFHFGGSTH
VLLFRPETQVEFNLHDQEPGLGSSNIKVREEIARVK"
CDS 81347..83389
/gene="ganA"
/locus_tag="OCPHGMPK_00640"
/EC_number="3.2.1.23"
/inference="ab initio prediction:Prodigal:2.6"
/inference="similar to AA sequence:UniProtKB:O07012"
/codon_start=1
/transl_table=11
/product="Beta-galactosidase GanA"

/translation="MKRILNTNEFLHGGDYNPEQWWDEPDVINQDFALFKQAKINTVT

VGIFSWAKLEPEEGNYDFSWLDSIFDRVEEMNGHVVLATPSGARPAWLAQKYPEVLRT

DNLGNKRGFGGRHNHCLTSPIYREKVREINTKLAEHFGQRKSLVLWHISNEYSGECYC

ELCKNAFREWLKNKYGNLDNLNHAWWNTFWSHTYNNWLQVNPPSPLSEMGNKGMNLDW

KRFITDQTISFIDNETAPLKKITPNIPVTTNMMAGNPLMDPFAGFDYQKVSKHLDFIS

WDSYPAWGNDNQTTAELGRNVGLIHDFFRSLKHQNFLVMENTPSRVNWHSVDRAKRPG

MHELASLQDVARGSQGVLYFQLRASRGSSEMFHGAVIEHLHPEQTRAFKDVTTVGKDL

ENIRPIINTNYAKARVAIVFSYDSYWALQDAESYSKDKKIWQTIQKHYRYFYKHDIPV

DFVSVEDDFSNYDLLIDPMHFLMSKAYLKKLASYVKNGGRVVGTYISGVVDENDLAYM

NEWPKELQDIYGVEPLETDVLYPGQSNTLNFDGHEYKAHDYCETLINCRGKVLAKYAS

DFYQDTPAVVEHEYGAGKGYYLACRTDYDFLEKFYEKITANLIPEFPVKKFSSNISIQ

VRENKDQKYYFVQNFSDKSEQIKVDGELEDLLEKKIDRGEVVLNPFGSKIYYKKGN"
     CDS   83709..84203
                     /gene="sorB_2"
                     /locus_tag="OCPHGMPK_00641"
                     /EC_number="2.7.1.206"
                     /inference="ab initio prediction:Prodigal:2.6"
                     /inference="similar to AA sequence:UniProtKB:P37081"
                     /codon_start=1
                     /transl_table=11
                     /product="PTS system sorbose-specific EIIB
component"

/translation="MPITLARIDQRLIHGITVNQWNKVLHPKRFMVIDDEVSQNEMVK

ASMRMSKPTGTGMSIINEEKAITNFKNGKYDSHSVFLIVKEPHYILDLINAGVEVPKV

NVGALFEEGNRKPYTKRVALDDQELSDLKEIAAKGIPVFFEYTPDDENEVSLAEVLKN
                     KGEN"
     CDS   84206..85003
                     /gene="sorA_2"
                     /locus_tag="OCPHGMPK_00642"
                     /inference="ab initio prediction:Prodigal:2.6"
                     /inference="similar to AA sequence:UniProtKB:P37082"
                     /codon_start=1
                     /transl_table=11
                     /product="PTS system sorbose-specific EIIC
component"

/translation="MPIILQNILIIILLAAYMAWDNGTGNQITGNWPVIIGMLVGLIMG

NLSVGLVIGGTLQLMSLGVAAIGGSSIPEYGVATIVGVFIAIRTGASTGTAIAVGLPV

GMLTLQLDVVIKIINNFFAHWSQRLLHEKKFNKMDGVFYLTIFVWMLKYIIPIALVVM

FGTPIVKLILHVIPAWVTNGLTIAGGMLPVVGIGMLLRYMPVKKYLAYLLAGFVLAGY
                     LKVPILGIAILGLAATYEIYRINAAKKDTEEVQTAGTVENEGDDFDE"
     CDS   84996..85829
                     /gene="manZ_3"
                     /locus_tag="OCPHGMPK_00643"
                     /inference="ab initio prediction:Prodigal:2.6"
                     /inference="similar to AA sequence:UniProtKB:P69805"

```
                     /codon_start=1
                     /transl_table=11
                     /product="PTS system mannose-specific EIID
component"

/translation="MSKELNQPENGKKLTTHERKDMFRRWIFNAGLGYNFETQQAPAV

AFSMRKALRKIYPNDDEYIEAMDNHYKYFNANPLMANIILGATLAMEEKDGIKAKDAV

QSLKTSLMGPFSGVGDSVFWILIPTIMGSIAGYMALQGNAVGAILWMLLQLSFYWLKM

RFFKTGYDSGTKLITSLGEKINIFTDAISVMGLMVVGSLIPTVVKVYTPLKFRTGKVM

LSIQSGILDKIMPALLPVAVTGLVYWLLGRKKWTPTKIILLIIVICLAGAATGILGVA
                     K"
     CDS             85842..86273
                     /gene="manX_2"
                     /locus_tag="OCPHGMPK_00644"
                     /EC_number="2.7.1.191"
                     /inference="ab initio prediction:Prodigal:2.6"
                     /inference="similar to AA sequence:UniProtKB:Q5XAF5"
                     /codon_start=1
                     /transl_table=11
                     /product="PTS system mannose-specific EIIAB
component"

/translation="MSNIKRHIVIASHSTLAEGMANALKFFEGNSLKLTVLSAYVDNK

PIEGQIEKIFNQFDKNDEVIVLTDLLGGSVNQKFFPYISRPHTHIITGMNLSLAMAIT
                     MEPVEGYLTGEKIQSIVSEAQNQVKYVNELVTDSSEDDEDE"
     CDS             86266..87933
                     /gene="malL_3"
                     /locus_tag="OCPHGMPK_00645"
                     /EC_number="3.2.1.10"
                     /inference="ab initio prediction:Prodigal:2.6"
                     /inference="similar to AA sequence:UniProtKB:P29094"
                     /codon_start=1
                     /transl_table=11
                     /product="Oligo-1,6-glucosidase"

/translation="MNDWKKKAIVYEAYVQSFNDSDGDGIGDLPGLIEKLDYIKNLGA

NVIWLTPIFKSPLVDNGYDIADYEAINPIYGTMNDFKKLLRKAHERDLRIVMDLVVNH

TSDQHKWFKESKKNKNNKYSDYYIWRDPKEDGSAPTNLGSAFGGSAWTYVPERNQYYL

HLFAKQQPDLNWNNPQVRNDIYHMMKFWLDMGVDGFRMDSISFISKPSKFEDAPLEDN

KEYGAYYYGSANGPHIHEYLREMNQKVLSKYDVISIGETPHTTAKEARLFVEPDRHEL

DMVFQFEHMHVDYGEFGRYSDVTYKMSDLRNSMTSWQNDLSWNSNYLGNHDQPRIVSR

FGNDSKYRKESAKMLAMVSLLQKGTPFIFQGEEIGMTNLHIKNIDELKDLEAHNIYSF
```

LKSKGISDKDALKMVNRKTRDNARTPMQWNKQQNAGFSSGNPWIEVNPNYLEINVKKD

LQDPKSIYHFYQKLLKLRNNNQVLLDGNYDLITPEDSAVFAYTRSSEKDKIAVICSFV
PYKVKYELPKDFVNKNKVILSNYDDKEDMASNTIYLRPYEGVVIKRE"
CDS 87926..88909
/gene="ccpA_2"
/locus_tag="OCPHGMPK_00646"
/inference="ab initio prediction:Prodigal:2.6"
/inference="similar to AA sequence:UniProtKB:P25144"
/codon_start=1
/transl_table=11
/product="Catabolite control protein A"

/translation="MSNMKDVAREANVSVATVSNFLNGKKVRPKAAKDIDLAIKKLNY

VRNNTARFLKTQRSAFVVFVVPSVWSPFFSELTFWVQKYLDNLGYKMVLCVSENDYQK

EKSYVTMAEEQRVAGILSISYSDLTSHVHSDIPLVSIEKESTGQFSLVSSDNYEGGKI

AAKELNERNIDKYIFIGSANHSSVEMNARKAGFIDYCNNHGLKFEIAEVDSQTDDKEK

IDSLIDEISNNGKKYGIFAHTDEIALILTQKFRDAGIKIPEQVQIIGFDGWKLTPDTH
LTISTIRQNIEEIAKTAVEQLHKEMEDPDHREISRLMIPVTFRAGDTTKNK"
CDS 88921..90927
/locus_tag="OCPHGMPK_00647"
/EC_number="3.2.1.177"
/inference="ab initio prediction:Prodigal:2.6"
/inference="similar to AA sequence:UniProtKB:A7LXT0"
/codon_start=1
/transl_table=11
/product="Alpha-xylosidase BoGH31A"

/translation="MFNTKNTQFLDYFYEGEHLCIYPWGHGLRVHDSFFDGMHNWALT

EKSDFDSNFAVTYNDDEATIVNGKTKAIVNKNGKITFYDKNKIILEEFWRERNFRKIK

INNSQFLNQKENEYSSALKIKARDFCPNNKGSYEVAVRFEANDNEKIYGMGQYQQSQL

NLKGCKLELAQRNSQTTVPFLISNYGYGLLWNNPGIGEVTFANNITEWKNIDTDCIDY

WICAEDTPKEIISAYTDVTGHSPKMPKYALGLWQSKLRYRNQNEVISIAKHYHDLHIP

LSVIVIDYFHWPKQGDFKFDKRYFPNPEKMVKDLKKLNVELMVSVWPNIDCTSENYNE

MNKKGYLVRADKGKSITMTFHGNTTFFDTTNPEARLFVWNRIKRNYYQKGVKHFWLDE

AEPEYSTYDFDNYRLHLGRNSQVGNIYSALYARGFYEGEKREGQNNPISLIRSAWAGS

QKYGALVWSGDIDSSFRSFKYQYQAGLNIGLAGIPWWTSDIGGFHGGSSTDSEFKELL

IRWFEFAVYCPVLRMHGDREPHEFPIDNQVEGTGAPNEIWSFGKETFDILRKQVNIRE

SLKPYINSCMNEAHKKGYPVMRTLFFEFPEDKASWNIEDEYMFGRKYLVAPIFEFGKR

RRQVYLPLGSRWKDINKGSIRSGGIWINCEAPLDRIPIFERVDY"
CDS 91035..92528
/gene="dtpT"
/locus_tag="OCPHGMPK_00648"
/inference="ab initio prediction:Prodigal:2.6"
/inference="similar to AA sequence:UniProtKB:P0C2U3"
/codon_start=1
/transl_table=11
/product="Di-/tripeptide transporter"

/translation="MNNSTTEKTFFGQPRGLSTLFFTEMWERFSYYGMRAILLFYMYY

AVERGGLGMDETTAASIMSIYGSLVFLSTVAGGWLADRIWGARSTVFFGGVLIMIGHI

VLALPMAELGLYISIAFIVIGTGLLKPNVSNMVGGLYSLDDRRRDAGFSIFVFGINLG

SALAPWLVPWASEGFGFNLFGNHLNFHAGFSLAAVGMFFALVQYVWGGRKYLSDDGMQ

PTDPIDPATRNKLLKRVLLGALVLAAVLGVMAALDQLNVDNIITLITIVAIALPIYYF

VLMLRSPKVTKEERSKVWAYIPLFIAAAIFWGIEESGSVVLALFAAQRTVLHIGNWHF

TAANFQTLNPLFIMILTPFFVWLWDNWKKQPSAAGKFAAGLVFAGLSYMWMALPGMLY

GTNGRVSPFWLVGSWFIVEIAEMLISPIGLSVTTRLAPKAFRSQMMSLWFLADATGQA
VNSQIVKYYSSKTEVAYFLAVGLVSVLFGVVMFFFTKKIHNLMADAE"
CDS 92681..93913
/gene="naiP"
/locus_tag="OCPHGMPK_00649"
/inference="ab initio prediction:Prodigal:2.6"
/inference="similar to AA sequence:UniProtKB:O34691"
/codon_start=1
/transl_table=11
/product="Putative niacin/nicotinamide transporter
NaiP"

/translation="MDTMVKHRHSLSKNQKWVIASTSSGFALENMDVLFLSFAMSSMI

ADLHLSGGAAGFISTITNLGMLVGGIAFGILGDKIGRVKTFSHTVIIFAIATALMAFA

NNIYMIYGLRFLAGIGAGGEYGVGIALIAENFHKEQIGKMTSVAAIGGQIGAILAALI

AAWIIPMAGWHMLFLVGIIPVVLTIFIRKHVHESEQFLEAKASEKNSLLSDVAKKMFS

TLRLAWQTLGLMIMMTVQIAGYFGLMNWLPTIVQKQLNLNVSNSSLWMIATIIGMSIG

MMTFGSIFDYFGPRRAFAIFLIGSALMVYTLSLAQNMATLLVIGAVVGFFSNGMFGGY

GAVISRLYPTEIRSSANNIIVNVGRAIGGFSSVVIGILMDRYNLGVVMGFLSALYIIS
FIVMITLPGLKDLSKVTK"
CDS 94011..94862
/locus_tag="OCPHGMPK_00650"
/inference="ab initio prediction:Prodigal:2.6"
/codon_start=1

/transl_table=11
/product="hypothetical protein"

/translation="MKKRKIYLASFFVALFLLVLTGCSQKSQEIPTKKVTVSLSNTKT

IPTFFFHGWGSSVNAEKHMANAAKKAGATNTIVQADVSKNGTVKLSGTIPKSAKNPII

EVNLEDNQSGKTSYVKDVITAVSNKYHYAKINLVGHSMGNLQIANYINENYNNKKLPQ

INKVVSIAGHYDGYLGEEAGQKAKIKNKETGQPDIYSDGFKKLLPLRKHYPKQIEVLN

IYGNKKDGSNSDGSVSVASAQSYKYLINGRAKSYREVEMKGKNAQHSKLHENKEVDRL
LIDFLWK"
CDS 94951..95391
/locus_tag="OCPHGMPK_00651"
/inference="ab initio prediction:Prodigal:2.6"
/codon_start=1
/transl_table=11
/product="hypothetical protein"

/translation="MELSETEKLNFAIIRGSKAYEEWDRQQGMPAYLTIILYELLLRK

RLTQKDLVYLSDLPKQSINKGIHRLLEKDYLKLTIDPDDNRVKYCQLTESGKKYAKEK
MTPLFEIENKVAQKMGAQKMKQLVALNEEWSETFWKFLRKKGEK"
CDS 95392..97125
/locus_tag="OCPHGMPK_00652"
/inference="ab initio prediction:Prodigal:2.6"
/inference="similar to AA sequence:UniProtKB:P9WQJ1"
/codon_start=1
/transl_table=11
/product="putative ABC transporter ATP-binding
protein"

/translation="MQILKPHFKKYRKEIILAVITILVSAFATLWQPRLLENIQKAIL

ADNHDTVLRDGIGLVVLGLLAIIAGIFNVYYAAKIAQGITSDLREETYAKIQSFSFGN

IEKFSSGSLVVRLINDMNQVMNMMMILFMQLLRMPIIILIGSFVLSIVTIPHYWWAPVL

MLALMMGVGFIVIQNMNQLFAKFQKYMDKISTRVKENLQGVRVVKSFNQVENEIKRFN

KTSDSLNELNIKIGYWISTIMPAFMLIAYLVIALVVFLVGRSANLNPTDVAVVSPYVS

YILTLLFAILIGGFVIMNFTRGMVSLRRIKEVLDTEPDVQFNPNASAAPEKGSIEFDN

VSFTYPDGDKPTLKNISFKVKPGEMVGIVGATGSGKSTLAQLIPRLYDPTEGMIKIGG

KDLKTIGEKALRNTVSMVLQKAILFSGTIASNLRQGKEDATDYELKRASEIAQAQEFV

GQYPDEFDHEVEERSANFSGGQKQRLSIARGVIGQPPILILDDSTSALDAKSEKLVQE

ALEHDLKDTTTVIIAEKIVSVMNADKILVLDDGKLVAEGTHEELLKTSPIYQDIYRTQ
KAKEKRGEIDE"
CDS 97118..98887

/locus_tag="OCPHGMPK_00653"
/inference="ab initio prediction:Prodigal:2.6"
/inference="similar to AA sequence:UniProtKB:Q9WYC4"
/codon_start=1
/transl_table=11
/product="putative ABC transporter ATP-binding
protein"

/translation="MSNTKKALKYFAKYLKNYWKGVTVVVVLSLVSTLSQVLGPLFLG

NAVQELTKAVSKVASRSHDLSSFYQSLGSMVSVYAAGIIASFIAWMVMSKFTANATND

MRENLFAKFQRMLIRYFDTHQDGKLLSLFNSDLDNIFNALNNAIFEIISQGALLIGTI

IMMFIVNPVMAWFTIATTPFILIISLVIMKKARIYLDKQQDKISDLNGYVNEQINGEK

VIITNGLQKESVQNFKKYNNDVRSAMFKGQFYSGMLFPLLQGISLLNLAIVIGGGAWL

IVSGQVSGAVGLGLIVAFVEYSQTYFQPLTQLTSIYSMTQLALTGARRLATVEEQDEE

DTVPNGKVLQGIEKGVKLENVHFGYNADKEILHGVSIDVDKGKSVAVVGPTGSGKTTI

MNLINRFYDVNSGKVTFDGVDVRDITLESLRNNVGIVLQDSVLFTGTVEENIKYGKPT

ASREEVIAAAKEANIHDFIMTLPEGYDTQVSEENSVFSTGQKQLISIARTILTDPEFL

ILDEATSNVDTVTEAKIQKAMDAVIAGRTSFVIAHRLKTILNSDKIVVLKDGNVIEEG
NHDELIKKRGFYYGLYTNQMAFE"
CDS 98937..99758
/gene="glnH_1"
/locus_tag="OCPHGMPK_00654"
/inference="ab initio prediction:Prodigal:2.6"
/inference="similar to AA sequence:UniProtKB:O34563"
/codon_start=1
/transl_table=11
/product="ABC transporter glutamine-binding protein
GlnH"

/translation="MKRKLRIFSLFTVLLSVFLLSACQKNTRNVYQEVKKSNEITWGV

KADTRLFGLMSIKTGKIEGFEVDLANALTKEMLGKNAKASFVQTTAKTKIPLLKNGNI

DAVLAAMTITPERKKQIDFSNPYFYAGQSLLVKEDSSIRNVQDLNGKTALAVKGTTAV

ANVKKFAPKAKVLEFDDYGQAFTALKAGQGQAMTTDNGLLAGIATENKGYKLVGGTYT

NEPYGIAVNKGQKQMKNAINRALNKLKKDGTYNALVKKWFSGIPGFNIKEAEGEK"
CDS 100210..100959
/gene="glnQ_3"
/locus_tag="OCPHGMPK_00655"
/inference="ab initio prediction:Prodigal:2.6"
/inference="similar to AA sequence:UniProtKB:P27675"
/codon_start=1
/transl_table=11

```
                     /product="Glutamine transport ATP-binding protein
GlnQ"

/translation="MAAIIDFKHVNKYYGKFHALKDINLSIEEGQVVSIIGPSGSGKS

TLIRTMNGLERINSGTLMVTGYDLADKHTDLNKIRKNVGMVFQHFNLYDNHTVLENIT

LAPKIVLHRPEKENHDIAMDLLKKVGLEEKANMYPRQLSGGQKQRVAIARSLAMRPKA

ILFDEPTSALDPEMIQDVLDVMKYVADQGITMVVVTHEMGFAREVGDRLIFFDQGRIL
                     EDAKPEEFFEHPKTERARQFLSKVITEKLGG"
     CDS             100961..101785
                     /gene="glnH_2"
                     /locus_tag="OCPHGMPK_00656"
                     /inference="ab initio prediction:Prodigal:2.6"
                     /inference="similar to AA sequence:UniProtKB:O34563"
                     /codon_start=1
                     /transl_table=11
                     /product="ABC transporter glutamine-binding protein
GlnH"

/translation="MKKSIKIFILPLVLGLLITLTGCAKKQKSSNVYEQVKASKTITW

GVKADTRLFGLMSIKTGKIEGFEVDLAKALTKQMLGKNAKAEFVQTTPKTRIPLLKNG

NIDAILATMTITPDRKKQVTFSEPYFTAGQSLLVKDNSTIKNVKDLNGKTALAVKGTT

AVDNVKKFAPKAKVLEYDDYGQAFTALKAGQGQAMTTDNGLLAGIASENKGYKLVGGT

YTSEPYGIAVEKGQTGFADRINKALNELKKNGTYHRLLVKWFSGIPGFNIKEVENS"
     CDS             101782..102420
                     /gene="glnM"
                     /locus_tag="OCPHGMPK_00657"
                     /inference="ab initio prediction:Prodigal:2.6"
                     /inference="similar to AA sequence:UniProtKB:O34671"
                     /codon_start=1
                     /transl_table=11
                     /product="putative glutamine ABC transporter
permease
                     protein GlnM"

/translation="MIGILTNHWSEFLSGFWNTILCSLIALFFSLILGVGFALLEVAP

NKFGRAVAHVYIEIFRNIPLLVITMIFYLVVPQIWFKVSGFAAGTIGLTLYTSAFIAE

TVRAGINSVRDGQMEGARSNGMTYTQAMRYVILPQALKIVIPPLGNQFINLIKNSSVL

AFVAGFDLMYQADVIAFSSFETINTYVVVGLFYLILTLPLSYYMRHLEKKLA"
     CDS             102434..103084
                     /gene="glnP"
                     /locus_tag="OCPHGMPK_00658"
                     /inference="ab initio prediction:Prodigal:2.6"
                     /inference="similar to AA sequence:UniProtKB:O34606"
                     /codon_start=1
```

/transl_table=11
/product="putative glutamine ABC transporter
permease
protein GlnP"

/translation="MENFIHAYSWINIRFLLQGLWVTIYVSLISIALSFILGLVFGFI

RYVKIKYLSTIVGFVIDIIRNLPLLLIIFFTYFGLPELGIVTNPTVASIIALVVFEGA

MLAEIVRSGIAAVDPGQMEGARSNGMTYMQAMYHVIIPQALNKMIPSLLSQFVSLVKD

TSLATIIVLPELLFHAQIIYSQNTTYLIPMYVIIAIMYFIVCFSLSMIAKHLQSKY"
CDS complement(103131..103433)
/locus_tag="OCPHGMPK_00659"
/inference="ab initio prediction:Prodigal:2.6"
/codon_start=1
/transl_table=11
/product="hypothetical protein"

/translation="MRGKINTNDSFIQKLQSDVEKYKTNPERRKELMDYQMKLDDMRY

VGEKTGKEEERIDAIKKMIKLSRKLNASNDFILKQLTEDYGSYFSQKELKQFIKNN"
CDS complement(103647..105305)
/gene="treA"
/locus_tag="OCPHGMPK_00660"
/EC_number="3.2.1.93"
/inference="ab initio prediction:Prodigal:2.6"
/inference="similar to AA sequence:UniProtKB:P39795"
/codon_start=1
/transl_table=11
/product="Trehalose-6-phosphate hydrolase"

/translation="MNNLGNKVIYQIYPKSFYDSNNDGIGDLRGIIEKIDYIKKLNVD

FIWFNPFFVSPQNDNGYDIADYKKIDPRFGTMSDFEELVAKLKAINVNVMLDMVLNHC

STEHEWFKKALAGDKKYQKFFYLRKGKDRQLPNNWQSKFGGPAWSKFGDTDYYYLHLY

DPTQADLDWHNPEVRAALIDVVNFWRNKGVHGFRFDVINVTGKDTILVNSTDPVKEKS

LYTDTPVVQQYLKELNAKSFGQDPESVTVGEMSSTTIENSIAYTRPEDHELSMVFTFH

HLKVDYKNGEKWSKMPFDFKKLKDIMLTWNSKIDQGGGWQALFWNNHDQPWALNRFGD

TGKYHNKSAEMLALALHLLRGTPYIYMGEEIGMMDPHYTSMRDYVDVEALNAYDALIK

KGIDEKEAFEIVQSKARDNSRVPMHWNNSQYAGFSGHKPWLMPTDQNRINVEKELQDG

EIFNFYQKLIKLRKSEKLISEGHIKPFLMEHPQVMAYERYLDDSNEKLLVFANFYGKE
TEVKIPAEYLNKEGHILIQNYDKNLASLPSVLQLKPYEALAIKL"
CDS complement(105319..106044)
/gene="treR"
/locus_tag="OCPHGMPK_00661"
/inference="ab initio prediction:Prodigal:2.6"

                     /inference="similar to AA sequence:UniProtKB:P39796"
                     /codon_start=1
                     /transl_table=11
                     /product="HTH-type transcriptional regulator TreR"

/translation="MAQSKVSAIAQDLVDKIKHQQYKPGEYLPSEHQLMDLYGASRET

IRKALTSLTDLGLIQKIRGKGSIVLNLDRYTFPISGISSFAELNNQLGLKAETKVLLL

QKEDHLPTGFIKYFPEEKNSAGFHLERLRLIEGQADVLDCDYLLSPPINSLPKDAAET

SIYNYLENKEKLEISYATKEISVCKVDKRIQDLLQLDNKLAVLVASRNYLADTTKFEL
                     TLSYHRPDKFKFVDFARRKKIKF"
     CDS   106239..108167
                     /gene="bglF_2"
                     /locus_tag="OCPHGMPK_00662"
                     /inference="ab initio prediction:Prodigal:2.6"
                     /inference="similar to AA sequence:UniProtKB:P08722"
                     /codon_start=1
                     /transl_table=11
                     /product="PTS system beta-glucoside-specific EIIBCA
                     component"

/translation="MVQDTTNLLAPATGAVIALAKTSDPIFSQGTMGDGFGLTPTIGK

VVAPVSGKISMVADTKHAVGIVTNEGLEVLVHMGVDTVGLKGAPFTIKIENGQEVKAG

DEIAEMDLDEIKAKGLDTTIMVLITNSADKISGLDVTEGEAKAGEAVATAYLKTAEAE

DTSSKKLSYPELASFIIKNVGGKENVNNVIHCITRLRFYLKDESKANDDVLKNQRGIL

DVMHASGQYQVVIGNEVTNVFDEVVKQLGPLDSEAAPAPEDDGKNPVSKAFGNLIGFI

TGSMSPIIGVIAAAGIIKGILALLTLPQLGSLLSVKSTAYITTSAIADSAFFFLPVLV

GYCAAKRLNSDPIIAAVIGGVLVYPQLVEWGKAFKTMFEVGGIKFEFLNYTYSIFPMI

LAAWLAKKCEDWLKKWLPSYLQMIFIPLITVLVVSALTLMVTGPIIQGVANGIAVFIN

WLVHASGWIGGFIIGGFYQVLVIFGLHWGVVPLVAQQIAGTGESALNAIICSSMIAQG

AAVLAVAIKSKKEDLKELGFASTISAMCGVTEPAVYGISLRYKKVFISGCIGSAFGGL

VTGLMHGTMYGFTGGLIGFSSFFNPKHPTDLSSFYTFLIASAVTIVVSFIVTWVWGYN
                     DSMVQGAKVAKKKRPGSK"
     CDS   108309..109103
                     /locus_tag="OCPHGMPK_00663"
                     /inference="ab initio prediction:Prodigal:2.6"
                     /codon_start=1
                     /transl_table=11
                     /product="hypothetical protein"

/translation="MIFEKLFSKENKKDEIVDVKLSDKKFNELRIKLSIGDVTVEQGE

IFSVNFTGKRHYLPLIRLEDGKLRLKQKMNLIEKGEAHLKVVIPAKATLDSLDIQTSS

GDIEIVDIKTEKAYLKSTEGRVRVRRVAIAESLLESTDGNVTVRNSDLADGKLATYDG

EIKVSGSILSRISLNVTDGDMNLTDVTIDEGSANLKAGNFTLDHAVFKSDYEIKNIEG
NNTVWSANLKYAHVRTANGLNNILFDPQPSGVVLTMTTVDGDNVVE"
CDS complement(109144..109779)
/gene="udk"
/locus_tag="OCPHGMPK_00664"
/EC_number="2.7.1.48"
/inference="ab initio prediction:Prodigal:2.6"
/inference="similar to AA sequence:UniProtKB:Q5SKR5"
/codon_start=1
/transl_table=11
/product="Uridine kinase"

/translation="MQSKRPIIIGIAGGSGSGKTTIAHEIYDQLQQDDHILIMTQDSY

YKNNDDLSMSERKKINYDHPDAFDMPLLVDQLRQLMDYKAVEMPVYDFTAHTRSEKTI

HTEPADIIILEGILVLGEENLRNLMSIKVFVDTDDDIRFIRRLERDTQERGRSVHSVI
DQYLKTVKPMYNQFIEPTKRYADIIVPEGGENDVAIDMLTTKIRSVLSNVK"
CDS 109941..111308
/locus_tag="OCPHGMPK_00665"
/inference="ab initio prediction:Prodigal:2.6"
/codon_start=1
/transl_table=11
/product="hypothetical protein"

/translation="MTAQEKAAIKNWNPRYRRNVYLYLWIYGILGAVTGITNDAALSY

FDIVAPGLISGLNIFNAITALLMSLMIVWVHELGYRKILLILPPLTSIFLALTTITTN

QIIIMFAYIISWTAIGVYDLMYPLMWTSYVPKEIRTKMFTVVMVVNLVAQTILTFIGG

KAVVYFFSKMQGISYDIASNLSAHTAQLSGSYLANYTNAFRIVLILTALVNVVAFVLA

IFIKDEPKDYRTVGMKKPQTPEEKKKAFEALINKKTIMWILYIAGIQLGARLVVPYIP

IYLNDYLHIPRGITSTINTFQTAAMFIGYFFAPFLAKKLGAIVSIAAGTLTCAPLMFL

MANGRHFGTGITLFVIVGVLLFLRSGLANATMPIQQEVQMIIVDKDMRPAFTAVVQIA

YAVIGIIDGLFTAFYLLKTPAGYANAYYIATALYVVVSILLIVMFAKQYNWILDEKKS
TSNEK"
CDS 111451..111603
/locus_tag="OCPHGMPK_00666"
/inference="ab initio prediction:Prodigal:2.6"
/codon_start=1
/transl_table=11
/product="hypothetical protein"

/translation="MKEIRKENIVKTTTLSEEEMIEVVGGTRVNGFVNGNFSKGIFYW

TRNIWK"
CDS 111606..112913
/locus_tag="OCPHGMPK_00667"
/inference="ab initio prediction:Prodigal:2.6"
/codon_start=1
/transl_table=11
/product="hypothetical protein"

/translation="MLVVYSFLMLISGITDLWIFFRVSQIAASKKNILILGGVLFLLS

ILCGGVVELATFLEILFFIFYFRKSKEKNLAIGAILIVGVVDSIIDILSSILMELLHI

EDIIYLNLLVQISIILLAVFIIEIFYKYLHSYLMNENHNVFIGLLIYLYVSTTLTMIF

YGQTQKNASLSMFFTIFVLIQIVFAMATYVELINIQKHLLKKSQQDKLIKEQKQLQDY

AQYLEESEDELRAFRHDYRNMFNSLKISAQEGNTKEVIQKLDEYTKANLNAKAFEKYR

DVNHIKIKSLKSIIIAKLTEMYREHIPYNFECSGEITELPKNINEIDLVRIVGISCDN

AIEESKVLIQQNQEAHIEIMAYSNERGEFEYEIQNKRSDSEISIKQIQQSGYSTKKSH
SGLGLANINSIKNKYENMTVSYEVPEGYFDFYLVIEPEEESEE"
CDS 112910..113707
/gene="agrA"
/locus_tag="OCPHGMPK_00668"
/inference="ab initio prediction:Prodigal:2.6"
/inference="similar to AA sequence:UniProtKB:Q5HEG2"
/codon_start=1
/transl_table=11
/product="Accessory gene regulator A"

/translation="MKYSVIVCDDDEVLAKNLAKNIKYAVSNFTDDNTVYENIEINLE

LVATTFEQVVSYIVANDIQNAIYFLDIELSQNSKAKNGVDLAEFIKKQDPNAQIIFVT

AYDKYAPLTYRRRIGAIDYINKTLDSREMMQRLEETLTGAVQSINNLMKSGRKELIYK

NGRRVNKVEDTTIYYLENSTSQHKVNLITETGVAEFRSNISKINEENDFLVKVSQSCV
VNPNNINSIDFSKKTIKFPNGDEVVFARSCRKTVEDLLNKYPEINVK"
CDS 113720..115879
/gene="lagD"
/locus_tag="OCPHGMPK_00669"
/EC_number="3.4.22.-"
/inference="ab initio prediction:Prodigal:2.6"
/inference="similar to AA sequence:UniProtKB:P59852"
/codon_start=1
/transl_table=11
/product="Lactococcin-G-processing and transport
ATP-binding protein LagD"

/translation="MLFKYKSVYVPQVDEMDCGVACLAMILKQYHSRVSLAHLRHEAR

TNLEGTTALGLVKTAQKFNFKTEAVKADMSLFDENSIQYPFIAHVLKQGELLHYYVVL

KNAKNYLVIADPDPSVGVIKMPKDRFAQEWTGIALFMVPNEDFEPIKEKKNNLWSLFP

YMFKQKRLMINIILAALLMTIISICSSYFVQGIIDTYIPDGTYQTLSILAVGLLIAYV

FNSIFSYGQNFLLNILGQRLSIDLNLRYIRHIFELPMEFFVTRRTGEITSRFSDASRI

IDALASTVISLFLDLSIVIVMGLVLAAQNMTLFGITLLALPIYAVVILGFTKKFEKLN

NEQMESNAVLSSSVIEDIQGIETIKALNSENTRYRRIDSQFVDYLKKSFKYSKTESLQ

TALKTFIQLSLNVIVLWVGAKIVMQGQLSIGQLMTFNALLAYFIDPLQSIINLQPRLQ

SASVAQNRLNEVYQVKSEFNQKATIEDRKLLEGNIEYKNVDYSYGYGTDVLKDINLKI

SQGEKLTIVGMSGSGKSTMVKLLVDFFSPSKGQVTLNGHATSEIDKHTLRSYVNYVPQ

TPYIFSGTVKENLLLGCRPDITEEDVIKACQIAEIDQEIANLPLQFETKLDENAKILS

GGQKQRLTIARALLSPAKVFIFDEVTSGLDTITEKKVVDNLMKLKDKTIIFIAHRLAI
AERADKVVVIDHGQIVEEGSHSELMSKHGFYYDLVKG"
CDS 115890..116495
/gene="lcnD"
/locus_tag="OCPHGMPK_00670"
/inference="ab initio prediction:Prodigal:2.6"
/inference="similar to AA sequence:UniProtKB:P0A3G5"
/codon_start=1
/transl_table=11
/product="Lactococcin A secretion protein LcnD"

/translation="MDAKEYESTEFYSYKFKNFSTMIIIPAAIFVLLLFIGSFFAVRQ

STVSSVGVVEPTVVINQKNVSYDEGQVVTKHGQKWVAHVDQDSGISLMPVMKAKGKVR

IVTYVPTNKISSIKKGQKLNFSVPTGEGLTSRLTGKVEKIGVYPINVNKQSMYEVISF
AKVSDINVKYGMQGNATIVTGRSTYRSTYFKYFLDKVLNKR"
CDS 116545..116829
/locus_tag="OCPHGMPK_00671"
/inference="ab initio prediction:Prodigal:2.6"
/codon_start=1
/transl_table=11
/product="hypothetical protein"

/translation="MNETKLKEMLTKIKANSDIAIDPEAQKLIEAAIERIDKKVDLQR
VISKLKQDLDIYSVSHQFKFPKSLVELQLMVNKYPDKWEGAGAGGVWPVI"
CDS 116976..117260
/locus_tag="OCPHGMPK_00672"
/inference="ab initio prediction:Prodigal:2.6"
/codon_start=1
/transl_table=11
/product="hypothetical protein"

/translation="MSENNFKELLTKIKANSDITTDLETQKIIEAAIERIDKKVDLQK
VIFKLKQDISMYFVAHGFKLPETLTKLQLLLDKDPNKWAGAGETMSLTNF"

```
     CDS             117706..117939
                     /locus_tag="OCPHGMPK_00673"
                     /inference="ab initio prediction:Prodigal:2.6"
                     /codon_start=1
                     /transl_table=11
                     /product="hypothetical protein"

/translation="MKVIEENEKEASRRERWIILGVSLVFGDLVNKLFLRLTSIDSFV
                     LSMIIGLGSMYVFESGYYYFRNDIQKMIKKVRG"
     CDS             118326..118607
                     /locus_tag="OCPHGMPK_00674"
                     /inference="ab initio prediction:Prodigal:2.6"
                     /codon_start=1
                     /transl_table=11
                     /product="hypothetical protein"

/translation="MPVGYIPIILLLLIVLVDIVASLKLHKLVKTSDVSNILILGITA
                     MVIFALKDLSSFSWSSAIATVFMSIVVLKDFYDEYQSQVKDGKDEKTTK"
     CDS             119031..119432
                     /locus_tag="OCPHGMPK_00675"
                     /inference="ab initio prediction:Prodigal:2.6"
                     /codon_start=1
                     /transl_table=11
                     /product="hypothetical protein"

/translation="MSKKEKVIGFRILSWTITSVCLIVFLGDIYSYGFSWQSFNQVVA

LILVVLLLISSIIFTSTKNLLEWNQQYKLAQWIGELVILPAFFLGNLYNSTHVWYSIL
                     FLSFFGGGSILFMLVQYFRRRSRNKCKTKLR"
     CDS             119557..119871
                     /locus_tag="OCPHGMPK_00676"
                     /inference="ab initio prediction:Prodigal:2.6"
                     /codon_start=1
                     /transl_table=11
                     /product="hypothetical protein"

/translation="MKNELKQVLNDFISLAKTHYDRKGNEYLLEQLEVALDKLEHNVQ

DEVDEARATYQNINTICLTNHLRLETDEEALLEKIKKISMSKGWLGGLNSWNTTNTWP
                     GR"
     CDS             119987..122788
                     /gene="pacL"
                     /locus_tag="OCPHGMPK_00677"
                     /EC_number="7.2.2.10"
                     /inference="ab initio prediction:Prodigal:2.6"
                     /inference="similar to AA sequence:UniProtKB:P37278"
                     /codon_start=1
                     /transl_table=11
                     /product="Calcium-transporting ATPase"

/translation="MITLTKNDENDEIIKVAQLSVEDALKELNTSQSGLTIDEVHTLQ

QKYGKNIFAKAKQEPLWKKFLANFTNLMAILLWVAGIIAFCADLVQLGIAIWAVNIIN
```

GIFSFWQEFQADKATEALANMLPSYTRVIRNGKEEKILAKDLVPGDIVKLEEGDDIPA

DIRVIAATTAQADQSSLTGEVNPVHKGAHAVEDVKKKNHADLNNMIFSGTNMMKGNIT

GVVVKTGMNTDFGKIAELTQNVAQQKSPLEKELDTLTKQISILAISIGIIFFLIATFF

VHYPLVKGFVFALGMIVAFIPEGLEPTVTLSLAGAVQRMAKKHALIKRLSSVETLGST

SVICSDKTGTLTKNEMTVKELWTLEKSYHVSGEGYAVRGHIKEGPKHVFAKDNDTLKE

VLLGGVFADNARIQAPDKKHVRYQILGDPTEACLEVVARKGKVDVEAELKNTPRVKEL

PFDSSRKMMTVIQSSDGTHRFNTYTKGAPNCVVDKCTSYLEHGEIKPITQEIKDKIMR

ANDGYAKDGLRVLAVAGRNLDQKMMNNLDSATIETVEENLTFLGLTVMMDPPRAEVYK

AARECRKAGIKVTMVTGDYGLTAKSIARQIGLTDPDKPLTVITGDALKTMPDDELRHY

LEGEVVFARMAPEQKYRVVSMYEKMGKIVAATGDGVNDAPALKKANIGIAMGGTGTDV

AKEAADMILTDDNFASIVGAIKEGRGVYSNIRKFLIYILNSNMPEAVPSVLFLLSGGA

IPLALTVMEILFIDLGTDMIPALGLGREDPEKGIMDRPPRSPKDHLINKHVLAKAFLW

YGLIASIIATAAFFGANFYRGHIFPNLPVVGWDYRQATTVTLAAIIFCQIAAVLNIRY

SRQSMFNRHFFKNSMIFIGIIMEIVLLLCISYVPVFQSFFGTEPLNAHDWIMLVCIPI
PLILIDELRKWILRKNYKK"
CDS 122861..123709
/locus_tag="OCPHGMPK_00678"
/inference="ab initio prediction:Prodigal:2.6"
/codon_start=1
/transl_table=11
/product="hypothetical protein"

/translation="MKKGLVMEGGAMRCMFTAGVIDTLMKEKIKFDGAIGVSAGAAFG

CNYKSEQIGRVLRYNIKYAHDPRLSSWQSWLKTGDLYGADFCYRELPVKLDIFDTNTF

QKNPMDFWCVATDIDNGEAVYHKLLSGKEADLKWIRASSSIPVFARPVEIGTRKYWDG

GISDSIPIKFFEKIGYQKNVVILTQPLSYRKEASKLYPALELVLHKYPAVLKKLKARA

TDYNDVLSYIRKKEVQGKMLVIRPPYPLDIGTMEKDPKELKRVYNIGVKEAQKCLKAV
QSYLKE"
CDS 123811..125307
/gene="artQ"
/locus_tag="OCPHGMPK_00679"
/inference="ab initio prediction:Prodigal:2.6"
/inference="similar to AA sequence:UniProtKB:P54536"
/codon_start=1
/transl_table=11

/product="Arginine transport system permease protein
ArtQ"

/translation="MKKTKLFSFFFAFLLFFSLGATFIQPAQAASEKKVAEGSVLKKI

KKSGELVVGTSADYPPLEFTASENGKTKYVGVDIELAKDIAKDLNVKLVIKNMSFDSL

LVALETGKVDMVISAMTPTPERKQSVAFSKIYYKPSGEYFLVNKKDKDKYTSIQSLKG

QTIGAQTGSNQYNLVKSQMKDSKLKGLGKINNLVLALQSGKVSAVVVEELIAKAYAEN

NNSLAAVRSNLKETQSGNAVAIAKGQDELVAQVNKTIDHVQKKDLINKQYLPAAAKYM

KTEKKSNTFAKYIPYFFKGIWYTIVITVFSVIIGIVLGIILALMRLSKNPILHWLAVC

YIEFIRGTPQMVQILFVYFGIGYLISNLSALVAGIIAIGLNSGAYVAEDIRSGIDSLP

KGQMEAARSLGLSRQKAFRYVIIPQAIKNIWPALGNEFITLLKDSSLVSVIGVAELMY
QTQLVQTSTYKGVLPLFIAMIIYFIMTFSLTRLLNHFEKRMKRNDSND"
CDS 125291..126025
/gene="artM_2"
/locus_tag="OCPHGMPK_00680"
/inference="ab initio prediction:Prodigal:2.6"
/inference="similar to AA sequence:UniProtKB:P54537"
/codon_start=1
/transl_table=11
/product="Arginine transport ATP-binding protein
ArtM"

/translation="MTAMIKVEHLKKSFGKKEVLKDISANVDKGKVISIIGPSGSGKS

TFLRCLNVLEKPSSGKIVFDGQDLTHINEKELDVLREKMGMVFQSFNLFPNMNVVENI

KLAPMKVKGVSEDEAEKQALELLAKVGLKDRAEQYPSSLSGGQQQRVAIARALAMDPE

VMLFDEPTSALDPEMVGEVLKTMQDLADSGMTMVIVTHEMGFAREVSDEVWFMADGYL
QEQGSPEQIFENPQSPRAQDFLSKVL"
CDS 126116..126646
/locus_tag="OCPHGMPK_00681"
/inference="ab initio prediction:Prodigal:2.6"
/codon_start=1
/transl_table=11
/product="hypothetical protein"

/translation="MSLMKIRQATINDYDQIMTILKDGANQLAERGVDQWQGDYPSPD

QIKDDIEKGFAYLAVSADGETVGAISIVEAPDHSYDNLKGKWLLNTDKYVVIHRVAIH

SKHAGNGYATKLLTEVIDYIRDNRKDIDSIRIDTHENNTAMQHLIDKMSFTKVGELHG
VYRPDEISYVYENVRE"
CDS 126764..127633
/gene="pstS1"
/locus_tag="OCPHGMPK_00682"
/inference="ab initio prediction:Prodigal:2.6"

/inference="similar to AA sequence:UniProtKB:Q97Q31"
/codon_start=1
/transl_table=11
/product="Phosphate-binding protein PstS 1"

/translation="MRTNFKFKIAAVLGLLAILIGLTGCANNNSNKITVVGSSAMQLL

AEQAGNDYRLSHPDSNIVVQGGGSGTGLSQVQAGAVEIGTSDVFAETQKGIDAQKLQN

HLVAVVGIVPIVNKSAGVKNLTRQQLSDIFTGKITNWKQVGGKNQNITVINRSKGSGT

RGTFEGLILNGKKPIQAQEQDSNGTVRKIVSSTPGTISYISFPYANDENIQKLSIDKI

KPTNKNVETNRWHLWSYEHMYTKGKPNKNVQKFIDYMLGRKVQNDLVPKLGYISINKM
QVERDSNNHVVQK"
CDS 127650..128642
/gene="pstC1"
/locus_tag="OCPHGMPK_00683"
/inference="ab initio prediction:Prodigal:2.6"
/inference="similar to AA sequence:UniProtKB:P9WG07"
/codon_start=1
/transl_table=11
/product="Phosphate transport system permease
protein PstC
1"

/translation="MNNKDLKKVVESALDKQKVPHVKLKKIGASKVDVASLTEPSKET

RQEYWGKGLTYCAIILIIIILVASIIGFIGFHGLETFTKDHVNVFQFLASSDWDPGEGK

SHVGAAAMIVTSFSVTLLAALVATPFAIAVALFMTEYSSKKGARFLQSVIELLVGIPS

VVYGFLGLTIIVPFIRNIFGGTGFGILSATLVLFVMVLPTITSLTVDSLKAVPSDYRK

ASLALGATKWQTIYKVILRVASPRIMTAVIFGMARAFGEALAVQMVIGNAVLMPANLV

SPSATLTSQLTSQMGNTVMGTLPNNALWSLALLLLIMSLVFNFLVRLIGKRGQK"
CDS 128642..129532
/gene="pstA"
/locus_tag="OCPHGMPK_00684"
/inference="ab initio prediction:Prodigal:2.6"
/inference="similar to AA sequence:UniProtKB:P07654"
/codon_start=1
/transl_table=11
/product="Phosphate transport system permease
protein
PstA"

/translation="MNAKTRNKIATAGIYTLVSIVVIILFGILGDILVSGVPHLSWHF

LSSEASSYQAGGGVRDQLFNSLYLLVLTLIISLPIALGAGIYLAEYAKDNWFTNLIRT

TIEILSSLPSIVVGLFGYLLFVVQFGFGFSIISGALALTFFNLPTLTSNIEQAIEGVP

QAQRDAGLALGLSNWKTIRGIVLPAALPGILTGIILSAGRIFGEAAALIYTAGQSGST

IDYSNWNPLSPTSFLNVMRPAETLAVHIWKVNTEGIIPDATIVSAATSALLIIVVILF
NLGARALGNHLYRKLTAAKS"
CDS 129543..130343
/gene="pstB3_1"
/locus_tag="OCPHGMPK_00685"
/EC_number="7.3.2.1"
/inference="ab initio prediction:Prodigal:2.6"
/inference="similar to AA sequence:UniProtKB:P0A2V9"
/codon_start=1
/transl_table=11
/product="Phosphate import ATP-binding protein PstB
3"

/translation="MQNVNEAPTFIHQFDQDEQIISTKDLSVFYGGSIQKLFGASLQF

KKKTITALIGGSGSGKSTFLRCLNRMNDKVARVDGEIWYHGLDINKNNINVYQLRKNI

GMVFQKPNPFPKSIRENITYALKANGEKDKQKLDQIVEESLRAAALWDEVKDKLDKSA

LAMSGGQQQRLCIARALALKPEILLLDEPASALDPVSTSKLEDTLKQLRTDYTMIMVT
HNMQQASRISDYTAFFHLGHVLEYDKTENIFTNPKGEITEDYIRGSFG"
CDS 130354..131109
/gene="pstB3_2"
/locus_tag="OCPHGMPK_00686"
/EC_number="7.3.2.1"
/inference="ab initio prediction:Prodigal:2.6"
/inference="similar to AA sequence:UniProtKB:P0A2V9"
/codon_start=1
/transl_table=11
/product="Phosphate import ATP-binding protein PstB
3"

/translation="MENIITSRDVHLSYGNVEALHGISLDFEEKELTALIGPSGCGKS

TFLRCLNRMNDDIPNIHISGDIQFEGQNIYGSKMDLVELRKEVGMVFQQPSPFPFSVY

DNIAYGLKIAGIKDKELIDQRVEESLKQAAIWKETKDNLDRNAQAFSGGQQQRICIAR

ALAVRPKVVLLDEPTSALDPISSSEIEETLLELKHEYTFIMVTHNLQQASRISDYTAF
LMSGDLIEYGKTADMFMNPKKQITSDYLNGRFG"
CDS 131127..131810
/gene="phoU"
/locus_tag="OCPHGMPK_00687"
/inference="ab initio prediction:Prodigal:2.6"
/inference="similar to AA sequence:UniProtKB:P0A3Y7"
/codon_start=1
/transl_table=11
/product="Phosphate-specific transport system
accessory
protein PhoU"

/translation="MHEVFLDELRKLNTRFMGMGIDVSESIEEATQAFVDHDKKLAQS

LVKDDQKVSRAATKVEKRTLKLMALQQPVASDFRNVISILKATGDLERIGENALSIAW

ETIRVKGNPRIPEVETIIKSMSKKVNFMLDQVLKAYVQGDEKLAREVAKKDDEVDEDY

VKARKLIIAGIKQDPEAAVASSSYFMVIRLLERIGDHVVNLAQWVVYKMSGELVDLNT
KDTDEMTEL"
CDS 131965..133503
/locus_tag="OCPHGMPK_00688"
/inference="ab initio prediction:Prodigal:2.6"
/codon_start=1
/transl_table=11
/product="hypothetical protein"

/translation="MSNTVQKKKKHQFPTAYTVIIIVLLLVQILTFFIPSGNYATLEY

DQPNKEFLITKPNGSKHKEAATQKTLDKYKVKIDVKKFTNGTIYKPVAIPDSYEKIDQ

KKPGLGGAIYQFLSSQVNGIAQSIDIIAFVLILGGCIGVVHANGAIDAGMQALSKKIK

GKQVLLIVLVMGLIAIGGTTFGLAEETMAFYPILIPVFLLAGFDRMTVVATIFLGTSI

GTMASTINPFSTVIASNTAGVNFTEALPLRICMWATCVIVGMIYVIAYAKKVQKNPKA

SYVYNDFVKEDQEYLNQDQTIQEHEFTWRQKLTLLVFAIAFIVMIWGVQQKGWYFTEI

AVVFLATGYIFAFISGLSEHKFVESFVNGAGDLLGVALTIGLARAVSIVMEESHTSDT

IMNFFSQQVSGMPPLLFIWFMFLVYIVLGFFIQSSSGLAVLSMPIMAPLANVVGIDRA

SIIDAYNWGQGFISLVAPTGLILMSLMMVNIGFNKWFKFCWKLLVIEFGICLAFLAIG
LVVY"
CDS 133584..134051
/gene="dps"
/locus_tag="OCPHGMPK_00689"
/EC_number="1.16.-.-"
/inference="ab initio prediction:Prodigal:2.6"
/inference="similar to AA sequence:UniProtKB:Q9KWH3"
/codon_start=1
/transl_table=11
/product="DNA protection during starvation protein"

/translation="MAYPKTKKIMNEIVADLTQAHMVVHQHHWYMLGRGFLKLHPYLD

DVMDELAEQQDGVAERLIEINGSPISTYEEVLEETNVPDQVGSWDLSMEERFQLIVNA

YKQLRDDYERGIKISEDEGDNSTNDLLIAYHTAVEKRIWMMSAELGKRPGEGE"
CDS 134174..134515
/locus_tag="OCPHGMPK_00690"
/inference="ab initio prediction:Prodigal:2.6"
/codon_start=1
/transl_table=11

/product="hypothetical protein"

/translation="MDLFNNQQARDAAIKAMHEYHLAYKAGQSIDTTEVKQSLKKLSQ

SDRTFMFIIGNTSNPLVAIPSINYDIKKNTLNKLIMAYFYFKKETENKMDRQILVVQA
DNFYKSVALNK"
CDS 134574..135497
/gene="rbsK/rbiA"
/locus_tag="OCPHGMPK_00691"
/inference="ab initio prediction:Prodigal:2.6"
/inference="similar to AA sequence:UniProtKB:P83534"
/codon_start=1
/transl_table=11
/product="Bifunctional ribokinase/ribose-5-phosphate isomerase A"

/translation="MNKVTIVGSINVDNIMHIKKLPQPGETIAMSEFSKAAGGKGANQ

AVASSRAKNETIFVGRVGDDDNGRFMLEQFKDNGVNVDHVTVTPNEQTGQAYILLQES

GQNSIIIQHGANFDVTAEDVRNAKDQIEDSDFVIAQFETPLEATIEAFKIARAAGKTT

ILNPAPARTDIPKELLELTDLITPNETEAESITGIKVDSEESMKKSSEVFHQMGIKGV

IITIGERGSYVSYEDIEEIIPAFKVKAVDTTAAGDTFLGALSSELKPDLSNLKESVVY
ASKSSSFTVQKLGAFPSIPTRDVVEQALKEN"
CDS 135501..136199
/gene="rpiA_1"
/locus_tag="OCPHGMPK_00692"
/EC_number="5.3.1.6"
/inference="ab initio prediction:Prodigal:2.6"
/inference="similar to AA sequence:UniProtKB:Q8DTT9"
/codon_start=1
/transl_table=11
/product="Ribose-5-phosphate isomerase A"

/translation="MDKSEQDQLKKEAATKAAMMVEPDSVLGVGTGSTVAFFIDALGE

RKEKEGFSLKHIVTTSNRSKKQLEGLGFKVDELADIDQADLTVDGADRVDDNLDGIKG

GGGALTLEKNVAINSKKIIWIVDESKLVHHLSGFPLPVEVLPVSAEQNFKRFEVEGLK

PQWRLDDNGKRYVTHYGNYIIDLAADPTPVPYGLADYLDHTVGVVEHGLFLDMCDEVI
IAHSDGTIEDKKRK"
CDS 136315..137238
/gene="rihC"
/locus_tag="OCPHGMPK_00693"
/EC_number="3.2.-.-"
/inference="ab initio prediction:Prodigal:2.6"
/inference="similar to AA sequence:UniProtKB:P22564"
/codon_start=1
/transl_table=11
/product="Non-specific ribonucleoside hydrolase
RihC"

/translation="MSKKPLIISTDPGIDDVAAMTISLFATELDVKMIVPTWGNVSLE

HTLQNTLDLEKFLHTKVPVVKGANQPLVRPAISAASVHGKTGIAGFEFETANDDLLEE

GLAATKMYEAIKNSPEKVTLLGIGPLTDFALLFKQYPDVVDNIAEVYIMGGNIGHGNH

SPFAEYNIAGDPEAAQIVFHSGLPVYVAPLEVGDKAHLTKDQMDKIKECGEVGKMLYS

MFSHIHEPDGDLRIKIYDPTAVGIMLHPEFFTLKPANVEIELAGRYTYGASVMDFLSK
EHNAQIATDVDTERFATWFVQSIQAANNGRK"
CDS 137238..137966
/gene="gmuR_2"
/locus_tag="OCPHGMPK_00694"
/inference="ab initio prediction:Prodigal:2.6"
/inference="similar to AA sequence:UniProtKB:O05509"
/codon_start=1
/transl_table=11
/product="HTH-type transcriptional regulator GmuR"

/translation="MTDLVYRSVMRDIKQKILNNEYEDMRLPDERSLSDYYHVSRSSM

KRALGLLAQQGIVFKKRGSGTFINPLYLKNQALFKYEGSNLGITDSFSVPGKKQSIEL

LDYQVIKADEDLRQDLFLKESDFVYQIKRLRLLDDQPFLIETGFIPIKITPELNPDIL

KGSLFNYLEDTQNKTVTRSFLTITVEPSTLEDQSKLMLAPNEPVGVMEGIFFLDDGTP
FEVSNMRIHYKYMKYNTFVNLSQE"
CDS complement(138033..138359)
/locus_tag="OCPHGMPK_00695"
/inference="ab initio prediction:Prodigal:2.6"
/codon_start=1
/transl_table=11
/product="hypothetical protein"

/translation="MLETTITKQNEIVKTLKDLYASFSKVQIKAYLPLEQTILKVIAK

AENHDDATAWSNKLVMFLQSQIALKQIPITKEQDALINSLSEQCKNTNLNYVYLAPIN
DSLQFD"
CDS 138624..139400
/gene="glcR_1"
/locus_tag="OCPHGMPK_00696"
/inference="ab initio prediction:Prodigal:2.6"
/inference="similar to AA sequence:UniProtKB:P94591"
/codon_start=1
/transl_table=11
/product="HTH-type transcriptional repressor GlcR"

/translation="MQSQVERLDLIRRTLDERKTISTREIMQLCQVSFDTARRDVIKL

TNTGQAIRIHGGLMKIKQGTVPDYNSRAHVLSPIKNKMAKMTAKYLTADKLIYLNAST

TISQLCHYLTGLNATIMTNSIDNAGALMMDNLPNVILLGGYLNKENHYTSSLNSLKEI

DQYEFDVAVIGTSSVNKNGVFVVNHPDAQIEREVVNRTKTVILLAEEYKFKEDRSSPY
KVMDCDQADILITDNKLDTKYRKYFKKNIKVREVLEEEKA"
CDS 139397..140191
/locus_tag="OCPHGMPK_00697"
/EC_number="3.1.3.-"
/inference="ab initio prediction:Prodigal:2.6"
/inference="similar to AA sequence:UniProtKB:P44447"
/codon_start=1
/transl_table=11
/product="Putative phosphatase"

/translation="MIKQIFSDMDGTILNGSGEINTKTVDFLKDYPIPLTLVSARAPM

EMEFAIDELGLTGPQIAFNSGLIFEKNAHGITKLLSHPIPHDLAINIIDFIKKEFPKA

TISWYTANSWNVFEFNEDVDYQVRLTKRKYHLQTEIGKEDIYKIMILDVKEKITCPLT

AALRKKFPNLVNTKTTGPKYCEITSCKAIKSSGIRYIQKMRNLQKEELIAFGDGENDL
EMLKEVGVSVAMGNAVNEVKKIATMVTDSNEEDGLVTALSKIGPSQ"
CDS 140276..142681
/gene="xpkA"
/locus_tag="OCPHGMPK_00698"
/EC_number="4.1.2.9"
/inference="ab initio prediction:Prodigal:2.6"
/inference="similar to AA sequence:UniProtKB:Q937F6"
/codon_start=1
/transl_table=11
/product="Xylulose-5-phosphate phosphoketolase"

/translation="MVVNYDSEEYLKSVDAYWRAANYLSVGQLFLMNNPLLKGELKAE

DVKPKPIGHWGTIVPQNFIYAHLNRAIKKYNLNMFYIEGSGHGGQVMVSNSYLDGSYT

ERYPEITQDEKGMAKLFKQFSFPGGVASHAAPETPGSIHEGGELGYSLSHGVGAILDN

PDVIAAVEIGDGESETGPLATSWFSSKFINPIKDGAVIPILQINGFKISNPTIVSRMS

DEDLTKYFEGMGWKPYFVSAYKDGEFNGYKDHMEVHHEMAKTMDEVVEEIKAIQKHAR

ENNDDSLVKWPMIVFRVPKGWTGPKFDLDGNPIENSFRAHQIPIPVSQGDMSHKEMLT

DWMESYKPEELFNEDGSPKEIVKENTLSGDQRMAMNPVTNGGINPKVLNMPDYRDFAV

KFDKPGSVEKQDMAEWAKYLDKMAALNPTNFRGFGPDETKSNRLFQLLDNQKRQWMEN

IHTPNDENLAREGRVIDSQLSEHQDEGWLEGYVLTGRHGFFATYEAFGRVVDSMLTQH

MKWLRKAKEQTWRHDYPALNLVDTSTVFQQDHNGYTHQDPGMLTHLYEKNRPDLIHEY

LPADTNSLLAVSDKAFRDRECINVLVTSKQPRPQWFSIEEAKKLVDKGLGYIDWASTD

KGAKPDVVFASTGTEPTIESLAAIDLLHKKFPDLKIRYINVIDVMKLMSPEKNPNAIG

NEEFDRLFPKDTPVIFAWHGFKPMMESIWFDRGRGKNDVHIHGYEENGDITTPFDMRV

LNHMDRYDLAKDVVESVPELSEKNADFIDEMDSLLAKHHQYIRDNGKDMPEVTEWQWN
GLK"
CDS 142847..143503
/gene="nfnB_1"
/locus_tag="OCPHGMPK_00699"
/EC_number="1.-.-.-"
/inference="ab initio prediction:Prodigal:2.6"
/inference="similar to AA sequence:UniProtKB:A0R6D0"
/codon_start=1
/transl_table=11
/product="Nitroreductase NfnB"

/translation="MEFKDVLAREHATRKFTDQRVSEKTVRKVIEEAQRTPSLLNSQP

WRIYVAEGEVAKAIRKEHEEKTLANEEPHEEFDSLLKVEWDTFPSKNMATMSETLDYF

LRGETDDFDQAQLKLFNAPVIVFLTIPKQSPAWSIFDLGGFSQTLMLAANNRGLSTMP

AHAFVKYPEVIRKYLNIPEDETIGIGIGLGYPNKKATINDYKSKRVPLDEILKIKKNL
"
CDS 143626..144321
/locus_tag="OCPHGMPK_00700"
/inference="ab initio prediction:Prodigal:2.6"
/codon_start=1
/transl_table=11
/product="hypothetical protein"

/translation="MTLLVSPQQWANWDQWYNYTLPGYVLILGTIFLFVGLIPFLCVK

NKLTAILFGLTTTTLCVFLGLSYFKNKESTEYRKENHYLTAMVRVYDAQIFAKKYYDP

NEIETFKYVADINTPSHLPSIYQKIPVKQEVTYLGKNDYYAFIKLDNTVMKFSLADCK

KIPGNKAYFTGYHFKIRNKKFLKLGFIDLKHDLRDKIELPADSYNKQVSENIEENYNH
PGLVANWIPDSEK"
CDS 144372..145751
/gene="corC_1"
/locus_tag="OCPHGMPK_00701"
/inference="ab initio prediction:Prodigal:2.6"
/inference="similar to AA sequence:UniProtKB:P0AE78"
/codon_start=1
/transl_table=11
/product="Magnesium and cobalt efflux protein CorC"

/translation="MSTSTITTNLIVIFITFLIAIFFVVAEFALVQTRSSQLEDMLAN

GQGSPKKLKRALHMVHNLNESLSTTQVGTTLVGVILGWIGQGTIEELLTDVFKMTPWF

GVKTSGVLGATLGVVLLTYLEVVVTEIVPKNIAIDMPVKCLMAVVTPLTVFHTVVYPF

VWLLNHSANGLLHLLGMQSADEESEVYSQSEILRLSRNAVTGGSLDKDDLLYMERAFE

LNDKVAKDIMTDRTQLQVLNATDNVKTALKKYIEDGFSRFPVVRDNDKDDIVGYVYAY

DIVSQDQENPDVPITRIIRAIITVPESMLIQDILKLMIKKHTPIVLVVDEYGGTSGIV

TDKDIYEELFGSIKDEIDDVSDEYIVRDDHGDIHVSGKTTLYDFERYFHTKLKSFEDS

DIITIGGYMMEHYPNLKKGESVDLEGFKFKLVNIEQGFMRWFLVSKIAKDTGKKAADS
DQNSEEKAK"
CDS 145849..146418
/gene="hpt_2"
/locus_tag="OCPHGMPK_00702"
/EC_number="2.4.2.8"
/inference="ab initio prediction:Prodigal:2.6"
/inference="similar to AA sequence:UniProtKB:P99085"
/codon_start=1
/transl_table=11
/product="Hypoxanthine-guanine
phosphoribosyltransferase"

/translation="MQNSDNIDNIIDRVLFSQDDIEEICQRLGKQLTEDYAGKFPLVI

GVLKGAVYFMTDLTRHMNVKMQNDFMDVSSYGDGFESSGKVRLDIDVQADVKNRDVLL

VEDIVDTGHTLKFMKDLLTKRGAKSVKCCALLNKTERREDDVVVDYFGSKVENEFVVG
YGLDYLNAYRNLPYVGVLKPEVIQANLNR"
CDS complement(146502..147812)
/gene="steT"
/locus_tag="OCPHGMPK_00703"
/inference="ab initio prediction:Prodigal:2.6"
/inference="similar to AA sequence:UniProtKB:O34739"
/codon_start=1
/transl_table=11
/product="Serine/threonine exchanger SteT"

/translation="MKRQISFGQALATVVGTVIGGGVFFKIGSISHETSTPSLTLFVW

ILAGIVSIASGLTVSEIAAALPVTGGSIKYIEYTYGKVWGFLFGWAQMLVYFPANIAA

LSVVFGQQFAVLFGFPTKYATLIALLLALFLMSLNFISTKFSTRMQSAMTILKAIPIA

LIVIFGLFNPAKISVNLLPLSSGHNVSFWAGLSGGLLAALFAYDGWINVTNLAGEVKE

PEKNLSRAIIIGLSAITLIYVLVNYAFLTAIPFKEIIGNQNTAYLASLKLFGSLGGKL

ITIGILISVYGAINGFMLTGMRIPYTLAKDKMLPFSNKIGRTNINTGVPTISALIILT

VSLIMIFLGTFDLLTNMLVFVMWTFTTLISIAVVVLRYREPKLDRPYVVPWYPIIPII
SIGGGLFIVISTVINEFWLSITGIGLTALGLPVYYYMKKHNQQN"
CDS 147932..148642
/locus_tag="OCPHGMPK_00704"
/inference="ab initio prediction:Prodigal:2.6"
/codon_start=1
/transl_table=11

/product="hypothetical protein"

/translation="MHRYRHKYTFPSIIILLILAFSGLVWGYFNMKQNTTVPRGSNMS

VLGIELDQTKDYIDLHKLEKNGISFVYLRSTQGKSYFDDNYLLYRDQLQGTNLNFGTI

IAYSDETSNQDQYQYFVNKVGTNTGSLPVMIVPATNHLTKSYWKRMGEFAQMINNLGK

RTMIAGDYHYHNYFPEGTRFLYTGASLKDRRHYAFWCYTQNGRVKNIDNLDRDVTMFA
YIGTNTQYAQLYSQEKTQ"
CDS 148653..149888
/gene="yfmL"
/locus_tag="OCPHGMPK_00705"
/EC_number="3.6.4.13"
/inference="ab initio prediction:Prodigal:2.6"
/inference="similar to AA sequence:UniProtKB:O34750"
/codon_start=1
/transl_table=11
/product="putative ATP-dependent RNA helicase YfmL"

/translation="MYSQAVSQVLEREHKTKPTLIQERTYDAIRNGASLVGLAKTGTG

KTLAYGLPVMERVADIGGRGVILEPTTELAIQTRNNLLHYAKALGLKTIALVGAGNRK

RQLERLKKEKPEIIIATPGRFFDFLSENRIKYQDINALVIDEADDILEFAKLDLLSSL

GQNLSSTAQILLFGASESEVTKNAENLFNHTFLLVDVRPEQKSEVKHYFLQVSNEYKM

QFLQRLAKLDKFKGILFFDSSETEMRFARIFNHSKTKFAVLSNETSKQNREKLLSDFR

AGRIKFLFATDLAARGLDLPDVSYVINFEIPSEVNTYLHRSGRTGRMNKSGTVVTLGD

DHDFRNLKKLLAGTYQIERAYFAGYSLTTEKPKLKKEENAEKKEHKKVEKNKPKHKKK
RWRNKKNKGYHPRKSRGEK"
CDS 149888..151219
/gene="gmuC_3"
/locus_tag="OCPHGMPK_00706"
/inference="ab initio prediction:Prodigal:2.6"
/inference="similar to AA sequence:UniProtKB:O05507"
/codon_start=1
/transl_table=11
/product="PTS system oligo-beta-mannoside-specific
EIIC
component"

/translation="MSSLVKWLETYVLPPASRLAQVRWLVAMRDTFISLLPITMAGSI

AMLFNSIIRAAKVQMHWDTFYSLMQPIVAIDNIIWEGTFALFAVYFAISWGYHLAKTY

EVNRFAGSVASLVAFAMSISDSVKLRIDGDVVDIKNAFDIKQFSTMGLFTAIIFGCIG

TALFIVFYKARIRLKVDTSMPHAEWVAFSTLIPILLSVFIVGFVNYAFQRLTGTYFGN

WLLATIQRPLVNLGQGFGVVLLVTFLVQIFWFFGINGISVLTPVMDSLWLTPENINVT

AVRNSEHVPYIWVRDSFNVFAWFGGAGGTLVLIIAILMFSKRKDYRTIAKMAVAPGVF

NINEPIIFGLPVVFNPVYLIPFVVAPLVNVSLAYWATTAGLVNPVQIFVPTVMPPLIG
PFLACNYDWRAIVLALINMVIALLIWMPFVFAADKIAKQDNNQRNFYLPQY"
tRNA 151269..151343
/locus_tag="OCPHGMPK_00707"
/product="tRNA-Arg"
/inference="COORDINATES:profile:Aragorn:1.2"
/note="tRNA-Arg(cct)"
ORIGIN
1 ttggtgggaa actgcctgcg agggtaggca gtcgccgtgc tcttttttaa
tattccggct
61 tagctcagtt ggtagagcac ctgactgtta atcaggttgt cgtcagttcg
agtctgacag
121 ccggagttta tggcaataat aaaatgatgg agtgttgtcc gagtggctga
aggagcatga
181 ttggaaatca tgtatacggg ctttacctgt atcgagagtt cgaatctctc
acactccgtt
241 ggagaatatt tttcgccata atgtgtattt catttattga gtatttttca
aaaaaagttc
301 ttgatttttg aaaatcatct ggtatagtaa tatatgtgct caattatgag
atctgacccg
361 ttggtcaagt ggttaagaca ccgccctttc acggcggtaa catgagttca
aatctcgtac
421 gggtcattac catattaaat atggttgttg gaggattagc tcagctggga
gagcatctgc
481 cttacaagca ggaggtcaca ggttcgagcc ctgtatcctc catcgttcat
ggactaccga
541 atatggtagt cttttttgt gtttaaaaat taatgaacga aattttgtat
atattgttat
601 tcggttaagg tcccgaaagg attcgttttt tagcgaagat gccttgtaac
cgaaagatgg
661 gggactctat gagaaaagaa aaacgtttat ttacttcaga atcagtttcg
gaaggacacc
721 ctgataaggt tgccgatcaa atctctgatg caattttgga tgctatttta
gctaaggatc
781 caaatggtcg tgtcgcatgt gaaactacgg ttactactgg tcttgtacta
gtagttggtg
841 aaatttctac ttctgcttat gtagatatac aatcagtagt tcgaaagaca
attttagaaa
901 ttggttataa tcgtcctgaa ctgggttttg atggtaataa ttgtgccatt
ttggttgata
961 ttgatgaaca atcgagtgat attgctggcg gtgttaatga atctctagaa
acacgtgaga
1021 atcatgaaga taaagatgat ttggataaga ttggtgctgg agatcaaggc
ttaatgtttg
1081 ggtttgcgat taaagaaaca cctgaattaa tgccacttcc tatttcttta
gcacattcat
1141 tgatgagacg ggttgctagt cttcgcaaag aaggacgtct tgattggcta
agaccagacg
1201 caaaggcaca agtaactgtg gaatatgatg atgacaataa gccaaaacgt
gtagacacag

1261 tcgttatttc tactcaaact gatgcaacag taactaatga tgaaattcgt gaagcaatga
1321 ttgatatggt aattaagaaa gttattccat ctcaatatct tgatgaaaat actaagttct
1381 taattaatcc ttctggccgc tttgtcattg gcggacctaa gggagattct ggtttaactg
1441 gtcgtaagat tattgttgat acctatggtg gatatgcccg tcacggtggt ggtgcatttt
1501 caggcaaaga cttaacaaaa gttgaccgaa gcgctagtta tgcagctaga tatgtagcta
1561 agaatattgt agctgctgat ctagcttacc aatgtgaaat tcaacttgct tatgcaattg
1621 gagttgctca tccagtttca atcatggtgg atactcatgg aacaagtaag gtaagtgaag
1681 atttgctagt tgaagccgtt agaaatgtct ttgatttaag accagctggt attattgaga
1741 tgcttaactt aagacgtcct atttaccgtc aaacagccgc ttacggacat tttggaagaa
1801 cagatattga cttaccttgg gaacatactg ataaagtaga agctttgaaa aattatgtga
1861 gtgagcatgc aaaatagaag taatactaaa attgttacaa ttgctatctt tttgacgact
1921 tttatgactg caattgaagg taccattgtt tcaacggcga tgccaactat cgtttctgat
1981 ctaaatggat tagaaatcat gaattgggta gtttcaactt tcttattgat gactgctgtt
2041 tcaactccaa tttatggaaa attagctgat agtattggca gaaaacctgt ttttctgttt
2101 ggaattgcgg tttttgtagt tggttctgcg ctctgtggaa ttgctcaaaa tatggtagaa
2161 ctgattctct tccgtgtgat tcaaggatta ggttcaggtg ctgttcagcc ggtggcagtt
2221 acaatcatcg ctgatttata tacactagaa aaaagagcaa aaatgttagg attgaactcc
2281 ggcttttggg gagtagcttc tgttattgct cctttattag gtggatttat tgtacagcat
2341 ctttcatggc actggatttt ttatattaat gtgccgttag gaatcgtagc ttttcttttg
2401 gtcgttttct tcttaaagga aaggaaaact agcagtaatt ctactttgga tttaaaggga
2461 acaacttgct tggttatctt tttattggct ttaatggtat ttttacaaga gttagaaaat
2521 ggcttaaata ttgtcttatt aggtttagta gttattatta ttgtttcggc gattatcttt
2581 ttcaaagctg aaaaaagagc aaaagatcca attatgccac tggatatgtt aagcagtaaa
2641 gaatttacaa tgattaactt gattacgtta cttatttctg gtgtggttat tggttttgaa
2701 ttttatattc cgacatggat gcaaggaata aatgccacaa gtgcgtctgt tgctggcttt
2761 gcagtaacac caagttcttt aatgtggatc gtgggttcat ttttaattgg tggtatgtta
2821 ggtcgatggg ggataaagaa aacatatgat tatatgttgc ttatcttgat aatcgctgat

2881 ttagcattaa ttttagttcc gatttacaca tcgttttggg tattttgctt aattgcggcc
2941 tttaacggaa cagcttttgg ggccattact acagcttcac aagttcgctc tcaagtactt
3001 gttggacgcg ataagattgg tgttgctact agttttaata cactaatgaa atatttagga
3061 cagactatga tggtatcgat ctatggtatt acttttaatg tggtcgtagc taaacaatta
3121 tcgcatcatc caaatttaac gcaaaagata atgaatgata tagtttcagc tgataaggct
3181 aagcatttgg ctgctaattt aataccaggt ttaagacaag tattattaag cgggttaaag
3241 tctgtatatg tagtttcttt aattgttatt gttttatctt taattcttaa tcaactttat
3301 aagcaaaaga aaattgcaaa ataaaaagcg ctaactgaga ttgatctaga ttagatctca
3361 gttagcgctt tttttaatta tgcttttta agtaagaatg gtacagaact gaaaaaccta
3421 gtaaaacaaa agtagtacct tctaaccatc caccgactac atcggaagga taatgaacat
3481 gacagaagat tcgagtataa ccgataaaca gtggaaaaat aatccaaata ataattagcg
3541 tggtgcgcca aaatttattt ttaactaata agatagttaa aataattaga attccgaaga
3601 aagttgcgct gccgacagag tggcctgatg gaaagctata accatctgca aaaactaaat
3661 gatgaactaa aggacgatgg cgttcaaccg catgtttagt aatccaattg tagccattgg
3721 cgcatatcat taatcccgct gaaaagaaag cataactaaa ttttttagcg ataagtaaac
3781 cgatgaataa aacgatagtt gcaatcgtta aagtactagt attcccgatc acagttaact
3841 tagttgcaat tgcaatgttg actggattgt tattagaaat aagatgaata attgtttggt
3901 caaaagaatg aataaatgga tttttatata tgaccaaaag ggcccagact acatagagaa
3961 ttaaaaaaat aaatcctgga accagtgtgt catgaacgac tatattgtta tctttctttt
4021 tcaaaaaaat ttctcccctt gtaatttcta gtagatgtgg taaattataa tcattatcca
4081 taaaaaaata aagactaggg agtagtagca agtcataatt gtttttagag atgagctggt
4141 taggtgcaaa gcttaacagt tatgctgtga acttaccctt atgagaagtc tatcgtttgc
4201 tttccgcgtt aaggggcatg agtgtcgcac ataatctgtg tggaacttag gtggtaacgc
4261 gaaaatgaag ttcgtcctat gatttttatc atagggcgtt ttttaatttt aaggagagat
4321 ttaggtgtac aatcacaaaa ctgtagaaaa aaagtggcaa aaatattggg cagagcatga
4381 tacttttaag acaggaaatg atcctaagaa gaaaaactat tatgctttag atatgtttcc
4441 atttccatct ggtaaggggc ttcatgtagg acacccagag ggctataccg caactgatat

4501 tatctctaga atgaagcgag cacaaggcta taatgtgctt catccgatgg gatgggatgc
4561 atttggtctg ccaacagagc aatatgcttt aaagactggt gaagatccag aaaaagttac
4621 taaagaaaat attgctaact tcaaaaagca gcttaataag cttggctttt catatgattg
4681 ggatcgcgaa gtaacaacct ctgatcctaa ctactataag tggactcaat gggtttttga
4741 acagatgtac aagaaaggtt tagcttatga agctgaagtt cctgttaact ggtcaccaga
4801 tttagggaca gttgttgcca atgaagaaat cgttgatggt aagactgaac gtggtggcta
4861 cccagtttac cgccgtaata tgcgtcaatg gatgcttaag atgactgctt atgcagatcg
4921 tttacttgaa gatttagacg atttagattg gccagaacca gttaaagaaa tgcaacgcaa
4981 ctggattggt cgttcagaag gtgcacaagt tacttttaag gttaaagata gtgataagac
5041 ttttgatgtc tttactactc gtccagatac tttgtttggt gttagctaca cagtacttgc
5101 tccagaaagt aaattagtcc aagaaatcac taccccctgaa caaaaagcag cagttgattc
5161 ctacattaag aaaattgaat ctaagtctga tattgaaaga actgatctaa ataaggataa
5221 gactggtgta tttacaggtg cttatgcaat taatcctgtc aatggtaagg aagttccagt
5281 ttggatttct gattatgtct tagcaagtta cggtactggg gcagtaatgg cagtgccggc
5341 tcatgatgat cgtgattatg cttttgctac taaatttggt ttaccaatta accgagttat
5401 tgaaggcgga gatttagaaa aagaggcctt tactggcaat ggtaagcata ttaattcaga
5461 cttcttagac ggcctagata atgaagaagc taagaagaga atgattgaat ggcttgaaga
5521 tcacaatgct ggtcaaaaga aggtcaacta taaacttcgt gactgggact tttctcgtca
5581 acgttactgg ggtgaaccaa ttcctgttat tcactgggaa gatggtacta cttcattagt
5641 tcctgaagat gaattaccac ttcgtttacc acatgcaact gacattaagc catctggtac
5701 tccagaaagt ccacttgcta acttaactga ttgggtcaat gttgttgatg aaaatggtcg
5761 aaaaggtaag cgtgaaacta atacgatgcc taactgggca ggttcttctt ggtactactt
5821 gcgttatgtt gatccacaca atgacaaaga attagctgat tacgatctct taaaacaatg
5881 gcttccagtt gacctttata ttggtggtgc agaacacgca gttcgccacc tgctttatgc
5941 tagattctgg cacaaggtac tttacgactt aggtgttgta ccaaccaagg aaccattcca
6001 acgtttgtat aaccaaggct tgatcttgaa gaatcacgaa aagatgtcta agtctaaggg
6061 taacgtagtt aacccggacg atgtgatcga tgaatatggt gcagactcac ttagaatgta

6121 cgaaatgttc atgggtccac ttgatgcttc catcgattgg gatgacaatg gtccagcttc
6181 aactaagaag ttcctagatc gtgtatggcg tttatttgtt aacgacctcg acttaaaggc
6241 aattccgcaa gaaagaattg ttgacaaaaa tgacggtgaa cttgataagg tttatgctga
6301 aaccgttaag aaagttacgg aagattttga tgccttacac ttcaacacag caattagtca
6361 aatgatggta tttataaatg ctgctcaaaa ggctaaaact attccacgtg aatatgctga
6421 aggttttgtt aagttacttg ctccagtggc gccacacatg atggaagaaa tctggcaagt
6481 cttgggtcat gatgaatcaa ttacttatgc taaatggcca acctatgatc cagctaaatt
6541 agttgaatca actgttgaaa ttatggttca agttaacggt aaacttcgtg gtaaatttaa
6601 ggctgctaaa gattctgata aagatgcctt agaaaaagaa gctctctctc ttgaccatgt
6661 tcaaaaattc ttagatggaa aagatgttaa gaaagtgatt gttattccaa acaagatcgt
6721 taacattgtt gctaagtaat ttaagttttt cctaaataat taatatttgt aaatactgtt
6781 tacattaatg agtaatatat aattcagacg cttaactgga tatagggaaa atttattttt
6841 atggaaaata atactaaaaa tactaaagag acaatggtca aaggctccgc atggatgacc
6901 tttggctcta ttttgtcacg aattttaggt gcattgtata ttattccttg gtatgcctgg
6961 atgggaagcc atggaaatat tgctaatgcc ctaacagcga aaagttataa tatttatagc
7021 ttatttatca ttatttctac ggctggtatt ccgggtgcag ttgctaagca agtagcgaaa
7081 tataatgctc taaatgaata tgacattgga cgtaagctgt ttagacgtgg cttaattttg
7141 atggcaattt ttggcgtaat ttgtgctgct atcatgtact tcggtgctcc aattttagct
7201 actgataata ttattggcgc actcttacat ggggcagaaa gtgatccacg acaagttgct
7261 gtaatgagaa gtttatctta tgcagtttta attattccaa ttttgagtat aatgcgtgga
7321 tatttccaag gttatgcaga tatgatgcct ccagctatgt ctcaatttgt tgaacaatta
7381 gctcgtgtat tgtggatgct tttaacggca tatatcatca tgcaagttca acatggttcc
7441 tatgtccatg ccgttgttca atcaaattta gctgcagcca ttggtgcggt atttggaatt
7501 ttgcttttag tttggttctt atatcgtaga agaaatgaat taaacgcatt gatggagcac
7561 tcaaatcatg caattaaaat ttctactgct gggatttttt gggaaattat taatcagtca
7621 attccattta ttatcattga ttcaggaatt acattatttc aattaattga ccaatacact
7681 ttccacccaa tgattgcgat gtttgttcat gcaagttctg atcaaatcga gtcatggtat

7741 gcgttatttg gattaaatgc aaataaatta attatgatta tcgtgtcttt agctactgct
7801 atgtcagtaa ctgccattcc acttttgtca ggtgctcatg caagacgtga ttatgctagc
7861 atttctagcc aaattgaaaa cacattggaa ctattcctat ttgttatgat tcctgcttct
7921 ttagggatgg ctgcaattgc aacaccaatt tatacgattt tttacggcta tgatccatta
7981 ggctcaaatg tcttatacct gtcgtctttt acagcaatca gtttaggact ttttacggtt
8041 ttaatggcaa ttttacaggg actatcagaa aatggactag caataaaata cttagttttg
8101 ggaatcatta ttaaatttgc tgttcagtta ccaatgattc aattatttaa ggtatatgga
8161 ccacttttag ctacaaatat tggattaatt attactattt ggctttcttt gaaacattta
8221 aaggttagat acaaatataa tagtgggcgt acatccagaa gattcattgg aatcgcaatt
8281 ttctcaatgg ctatgtttgt tgtagttact ggagtagttg attttggtgg aaaactcatt
8341 tcgcctgagc gtcgtccaac ttcctttatt ttagttatac ttgctgttgc tgttggtggc
8401 attttgtatg cattcttatc agtgaaattt ggcttagcgc aaaagattat tggaccaaag
8461 gttaaccgag tattagcaag attgcatatt agaatttaag aatatagaga aaaagggaaa
8521 cttagtgatt atacctaagt ttcccttttt tgcccctgcc aaggaacttc attatataat
8581 attctagaga aagtgaggaa gaaaagatga ataatatgct tgtatatgct gcgaaagaaa
8641 ggaaaagctt tagtgaagct tctttaaacg agatagacag catgattttt tcacagttag
8701 catattgcaa ttttgatctt cttcctcaaa tcactaagtt gaccgatttg gtttcagacg
8761 acgaaattaa gactttaaca caaggcaatc ttggcggaaa aaataccgaa aaattaatca
8821 agattatgat taaaaatcca cgttttaaaa atgtatcttg gcatcaagcc attagtaagg
8881 ttgatgcaaa aacacagatg cagtttaatg ctgttacttt ttatgtcgct acgggcgagt
8941 attatgttgc ttatcgtgga acaacagcga cgactattgg atggaaagaa aactttaata
9001 tgtcttttag agattgggtg ccagctcact attttgcccg ttcatattat cgaaaagtaa
9061 gaaataaatt tcccgggaaa ttttatattg gaggtcattc gaaaggtggt aatttagctt
9121 ttgccgttgc attaggccta aaagaggtcg atttagctaa tattgttaga atagactgtt
9181 ttgatggtcc cggctttcat aatcaagatc gattaaaaaa caaattttta aaattaaaag
9241 gaaaaatcca taaatacatc ccacaaggat ccttaattgg aatacttcaa gatgatctta
9301 ttggagaaaa agattactgc acaattgtag cagctgcggg tgtgaatata cttcagcatg

9361 acatgtttaa ttggcgtgta aaaaatggca agtttgtgac tttaaaacaa ttagatagta
9421 cctctgaaat ttcctggaag gcaattgctg aatggctaaa aaatactact gatgctgaac
9481 gaagagattt cattgaaatg ttgtatttaa ctgttagcga tactaatcaa atttatttta
9541 gaaatctatt aacgcctaaa acaacgtata aactagccac tcgtttaatt aaaaatagtt
9601 ctgtcaaaaa agaggtatgg gaaccaataa ttagaaaatt atttcaagct tatgttaact
9661 caggtacaac aacgctcagt gagcagagaa gaaataattt aatgagtttt aaaaatattt
9721 tagatgagca acgccgatag taataaaaaa tttaaaattt cccgcttata aggaaaaaga
9781 gtaaaataac gtttgaaaga ggtggacaaa attgaaaaaa gatacatata tgactttgct
9841 aggattgcgt gatacttgtt ataacaattt caattttgat attgatgaaa aagatttcta
9901 cttaggaacc gatacatata aaaagataga taagatctta aaaaacgcac aagacgagca
9961 gcttctaatt cagtttgaag aaggtactca cctttatcaa gattatgatc ccgaatattt
10021 gagtgtagct caaaagagag aagtgcaaca tgatattaac gcagctcgtg actttttaga
10081 taactatgat ggtagcaatg atgataagca tgataattat gctgcttact tagatgacaa
10141 ttcattaagt tattcttttg gcgcagatag tagtttagat gaagtgcaaa ataatttaaa
10201 cgatattgaa actgacgcag ttattgtttg gcaaagaaga aaagatgaat atatcttact
10261 cccacatact attcacagcc ttgaaagaat taaaaaacaa atttcatcga ataagcatgt
10321 tgatgataaa aaacttcgtt atgcaatgga gcgtttgttt gataatataa ctgatttcct
10381 taagccattt agtgatagcg aactaggaaa atatgaaaat tcgatttctg aagagttaaa
10441 ggacaatttt gagcattatg ctttaggcgt tcaaacagat gcacgagaaa cagcagctga
10501 taaattgaca aaatatgctg ttgaacttgc agaaaaggaa aataacgata aattataaag
10561 ctgaagctca tcgatttgat gggctttttt ggataagata aaaatgaaaa gtatttcaaa
10621 agaactaatg tctgaggtaa ttaaaaagcg tgaaagtgcc acacataagg gtaattatgg
10681 gcgagtacta ttaattggtg gtaataaaaa atatggtggt gctctcatta tgtcagctga
10741 aggagcacta aacagtggtg ctggtttgac tacggtggcg acagattcag taaatattag
10801 tgccctacat accagagatc ctgaaattat ggctcttgat tgggataaaa gagatgaatt
10861 aaaaaatcta attgtgggct ctaatgtagt agtttgtggg atgggacttg gtttagatga
10921 ccaggcacga gatattttgg ctcttattag agatagcatt agcttaaagc aagtattgat

10981 tttagatgca agtgcacttg atttaattag ccagcaaaaa gatttattgc
cagtaaactc
11041 aaaattagta atttttacac ctcaccagat ggagtggcaa agattgagca
aaataaaaat
11101 tgctgaccaa aatgatcaat cgaatcaagc ttttttaaat gaattagtcc
ccaggaaaaa
11161 tgctatttta gtattaaaat ctaatcatac taaagtttat gatcaagcag
gaaatattta
11221 tcagaatcct tttggcaatc caggaatggc gattggcgga atgggagaca
cgttagctgg
11281 aatcattggt ggtttctgcg gtcaatatac acctaattta aagactgtcg
caggtgctgt
11341 gggcattcat tcaataactg ctgatgaaat tgcgaagaaa cactatattg
ttcgaccgac
11401 acaactgtca gcccttattc ctgcaatgat gagaaaatat gagaagtgaa
taaaatgaaa
11461 aagataagta aatatcaaaa ctctgtttta attattggaa caatactaac
tattttagta
11521 tttgcaatta ttttatttcc tcatcgtaat gtatcgctgg caagtccaaa
taaaatagta
11581 aacagtaaag taacaaaaaa agtagcccaa aaagtaaaaa aagttacaca
taaagattct
11641 aatttaccat atccggatcc taaagattta cagccagctg gtagctggaa
agttaaaagt
11701 gaaagtaagg cacatcctga tctggtaaga ctaaataatt tgtggataag
agtttcaatt
11761 aagggaaatc gtgtttatat tatggatggt aacaagccag tttacacaat
gcttgcatct
11821 tgcggggtgt atcataatgg taaatctgca acgccaactg gaacttatgc
tgtcgaagct
11881 gaaaggggag caacattttt taaccagagt ctccaggttg gcgcacgtac
ttacattagc
11941 tggcacggcc atggaactta tctctttcat tcggtcccaa cagatggtaa
taataaaatc
12001 atgaaaaatg aagcaaagaa acttggtaaa acccaagcct cgcatggttg
cattcgcttg
12061 agtattcctg attctaaatg gctatatgaa aaattacctg ttggcactaa
ggttgtagta
12121 aaggatgagt agactaaata tgtgcagcga aagaaattag gtgacgaaat
gaggcatgca
12181 aaaaagaaat ctagaaaaaa atttctgtgg ataataggta tagcaataat
tattatttgc
12241 tttttctgt ggaaaaatga tcatggtcca aatgggatgc cgtctaatta
tcatgccgat
12301 caagtgaact taaatgttaa agctgccgta gcaattgatg ccaaaaatga
aaacgtcatt
12361 tatgccaaaa atgccaatca aagtttgcca attgcttcaa tgactaagtt
gttaactgtt
12421 tacttaacct taaaagcaat taaagagaag aaaatttctt gggatacaac
tgttagtcct
12481 actcaagaaa tcattaattt aagttctaat cctgattatg caggtgtgcc
acttagttta
12541 ggtcaaaaat acactgttag agaactttat gatgcggcat tgataaagtc
agctaataat

12601 gcagctcgta tgctggcgat tgcagttagc ggaagtgaga ctaatttttt aaatcaaatg
12661 cgacagcaag ccaataaatg gaaactttat aatgttaaat tggtgacagt tgatgggtta
12721 ccggagaaaa ataaaaactt cttagggatg acaacgacaa ttgaaaataa aatgagtgct
12781 aatgatatgg caattattgc gagaaaatta gttacagatt atcctgaagt tttaagtacg
12841 actaaagtag caaagtccga ttttagaaat acattaatga ctaatagtaa taaaatgcta
12901 agtggacttt cagactacga tcctaattat cctgtcgatg ggttaaaaac aggaacaact
12961 gatggagcag gcgcttgctt tacttgtaca atgaataaaa ataataagag agtaattaca
13021 gtgattttgg gtgctcaaaa tgataacgaa cgcttttcag aaaccaaaaa gttacttaat
13081 tattctttta attaaaattt agctatgaat tcgttttaat attgcacttt ggctaagatt
13141 gctgatataa tcttcatgat aaagagttaa aagtagtaat attaatctat tttcagcgag
13201 tctatggttg gtggaagtta ggcaatagag tgatatgaag gcgtgtttaa ctacaatttt
13261 aaaatcaatt aataagcgga tactttgtat caagtagagt ggtaccgcgg gaaactttct
13321 cgtctctttc tagattagct aggaagagac tttttttatg caagaggtgt tatcagtgga
13381 ttttaaacaa aaagtcgtag atttagttag tgaacaggta gatttgccta aagaaaaaat
13441 tgcaatgtta attgaaagac cgaagaatgc taagatggga gattatgctt ttccagcatt
13501 tgctttggct aagattgaac acaagaatcc agctttgatt gcaaaagaca ttgcagagaa
13561 aattagtgac gataatttca ctagtattca ggcagttgga ccatatgtta actttgcgat
13621 tgatcatgct aaacttgtta atgcaacttt aaatgatgtt ttgacagaaa aagaccattt
13681 tggtgatcaa caattaggcg aaggtaatgt accaattgat atgtcttctc ctaacattgc
13741 taagccaatg tcaatggggc atttacgttc tactgttatt ggtaattcaa ttgctaaaac
13801 tttggaaaaa gttggttaca ctccaattaa gattaattac cttggcgact atggtacaca
13861 atttggtaag ttgattacag cgtatcgttt atggggcaat gaagaagatg ttaagaaaga
13921 tccaattact aatcttttcc attattacgt taaattccat gaagaagctg aaaaagatcc
13981 taaattagat gatgaaggac gggctgcatt taagaaactt gaaaatggtg acgaagaaga
14041 gattaaattg tggaaatggt tccgcgaagt ttccttgcaa gaatttaacc gtatttataa
14101 agaattaggt gtaacttttg attcatacaa cggtgaagct ttctttaacg ataagatgca
14161 accagtagtt gacgaattaa gagaaaaagg attactagaa gaatctcgcg gagctcaagt

14221 cgttaactta ggtgaagatg agaatccagc attaatctta aaatctgatg gctctagtct
14281 ttatatgact cgtgatttag ctgctgcatt atatcgtaaa aaagagtatg actttgttat
14341 gtctctttat gttgctggtg gtgaacaaag cggtcacttt aagcaattaa agcaagtttt
14401 gaagaaaatg ggatatgact gggctgataa tattcaccac attccatttg gtctaattac
14461 gcagggcggt aagaagttat caacaagaaa aggtaatgtt gtcttcttag ataaagtatt
14521 gaaagatgcg gtttctttag cagaacaaca aatcgaagaa aagaatccta acttagctaa
14581 taaagaccag gtagctcatg atgttggtgt gggtgcagta gtattccacg acttaaagaa
14641 tgatagaatg gataattttg actttgactt agaagaagtt gttcgtttcg aaggggatac
14701 tggtccatac gttcaatata ctaatgctag agcacaaagt atcttgcgta aggcaaataa
14761 agaaatctca atggataatt tgagcttaaa tgatgattgg tcatttgcag ttgcgaaagc
14821 tttagctgat ttcccagcta ttgtagcaaa agcttcagaa aaatttgaac catcaatcat
14881 tgccaaatat gcgttagatt taagtaagaa atttaataaa tactatgcaa atgtgagaat
14941 tttagatgaa gacgatcagt taaatgctcg tcttgcacta gttcaagcaa cttcaattgt
15001 tttaactgaa gctttgagac ttttaggcgt aaatgcacct aaagaaatgt aatatttatc
15061 aaaataaaaa tattcactga attttttga gctaaagtat gttaattttt gcactaatgg
15121 gttaaactaa tgatgtgtta aaaatttagg aggtattttt ttaatggctt atttagtaaa
15181 tggtaatgac attttcaaag ctgctcgtga aaaccactat gctgtaggtg catacaacac
15241 taacaacctt gaatggactc gcgcactttt aagaggtgct aaggaaacta gaactccttt
15301 attgattcaa gtttctactg gtgctgctaa gtacatgggt ggttacaaga ctgttaagga
15361 tttagttctt aacgaaatgg acaacatgga tatcgatgtt ccagttattt tgaacttgga
15421 ccacggtgac tatgaatctg ctaaggaatg tatcgcactt ggttactcat cagttatgtt
15481 tgatggtcac aacttaccaa ctgacgaaaa cttagctaag actaaggaaa tcgttaagtt
15541 agctcacgaa agaggtatct ctgttgaagc tgaaatcggt aagattggtg aaaaccaagg
15601 tgccgatggc ggtgaattag catctgttga agacgctaag actttcgttg ctgctggtgt
15661 tgacaagctt gcttgtggta ttggtaacat ccacggtgtt tacccagaag gctggaaagg
15721 cttgaacttc gatcgtttga aggaaatcgc agaagctgta ccaggtgaac cacttgttct
15781 tcacggtggt tctggtattc ctcaagacca aattgaaaag gcaattaaat taggtattgc

15841 taagatcaac attaacactg aattccaatt agcattccaa gctgcaactc gtaagtacat
15901 cgaagacaag atggacttag acaagggcaa caagggttac gacccacgta agcttttgag
15961 agctggtact gacgcaatta ctgattctat gaaggaaatg atttcatgga tgggtactgc
16021 accaatcgac tcaaaggaat catcagttaa gtttgacgaa gcttcattaa acgaagaata
16081 atcaactaat tctattaaaa aagacctcag tttttctggg gtcttttttg tttaggtgag
16141 ataaaagata atgcgtgtcg aattttacga aaagggttgt aaaagtttct ttaaaaaata
16201 taataagcaa aaagatacca ttgtagaatt tgttgaagac acaattgaca aagaagtggc
16261 tagcgggatg acaaaggtta aattggcgac cagaaaaaga ataaagggta gaaatattta
16321 tgaatttcgt cttaatgttg gtacaatagg ttcgattcga attgcatttt caatttttga
16381 taaaaaagca atagtttact ttattagtaa gaacattcaa aaatcagcgt ttagtaaaga
16441 ttttgagaaa ataatttcta agttatagta aaatactttt aacgcaatac atatttaaaa
16501 agtaagaatt tgttactatg aaaattttga aaattaaaga tctagatcaa gaaatagaaa
16561 ataaattatt agtaatttgg gaaagtagcg tcagagctac gcacaatttt ttatcagatc
16621 cagaaatcaa caatattaag aaatatgttc ctcaggctct aaaaggagta gcgcacttag
16681 taattgcata caatgatgca aatgaatctg ttgcctttat gggaatcaat gatcaaaagt
16741 tagaaaggct tttgttgct gctaatctta cggcatcaat aatttagata ttaatgagct
16801 tgccgttaat gagcaaaatc caggagctcg tggtttttat gaacatatgg gatttaaagc
16861 agtagaaaga tctgagtttg atgatcaggg taatccttac ccaattttaa ttatgaaaag
16921 aatttagaaa gaaggattta gatgtcttta acagttaacc tttattacac tggaaaaaat
16981 ggaagtgccc gcaagtttgc tgaagaaatg gaaagtagcg gtgtggctga tcgtattaga
17041 caagagccag gcaatgaaaa atacgattat ttcattccta tgaatgaccc agaaatgata
17101 cttttgattg atagctggaa gaaccaaaaa tctttagatg ctcatcatgc ttcgccaatg
17161 atgaaagaat tagcagattt acgtgaaaag tatgatctac atatgcgtgt tgaacgttat
17221 atttctgatg agaatgggat gccagatacg gatcaaaagt ttattagaaa atagtaacag
17281 aaaaactcgt tgatctatca atcaacgagt ttttgcgttc aatactaaaa atagaggaaa
17341 atgaagaata gaattataga tttatttccg acgaaccttt acaggaactg gaactggttt
17401 atttacaata gcggcatttg cagcaagtac tgccatagcg ctaactccta gaattaataa

17461 taatttcatt aaaccattcc tttctttatt tgattgatat ccatcttaaa gtgaagttgt
17521 gaatttttg tgacttatat ttaagagaaa atctaaaaaa ctataaataa ttttattccc
17581 tttataaata cgaacaattt tgcaatatta agtaaaaata attaataaat aagtgtaaaa
17641 agactaaaaa aatctcttta tttatgttaa aatttcatca ttgacgattt taagtaaaga
17701 ggaattacag aatgaataac gatatcgaac gtatattgtg tactcaaagc gacttagata
17761 gtcgtatgga tgaaatggct gcggaattaa atgtgaagta taaggacgaa gagccgattg
17821 tcgtgcccgt cttaaatgga gccatgattt ttgctagtga catgattaag cgattgaatt
17881 ttaagttgac tattgatccg attaaggcct ctagttatgc tggtacgcaa tcaactggag
17941 aagtaaagat tacgcaagat attaagtcag atgtaaaaga tcgtccagta atttttatgg
18001 aagatattat tgatactggt cggactttac aagcactcag tgaagtcatg aaaggtcgtg
18061 gagctaagag cgttgaagtt gttgcaatgc ttgataagcc agaaacacgt gtagtagatt
18121 ttcatgctga ttattacggt tttaaggcac cagacgagtt tttagttggc tatggcttag
18181 attataatgg gttgtacaga aaccttcctt atgtgggaat tttaaagcat gaggtttatg
18241 ctaaataagg gggggataag taatgcgtgt aaatgtttta caacatactc caaatgaagg
18301 acctggttca attaaaactt gggctgatca acatcactat gatttctatg tttatcatcc
18361 tgaaactttt ggtaaactgc caactgtgga agaaacagat ttattaataa ttcttggcgg
18421 accaatgagc cctaatgatg atttaatttg gattaagcaa gagagaaaat taattaaagc
18481 aatgcttgat gcgcataaac caatgtttgg tgcatgtctt ggtggacaac aaattgcaaa
18541 aactgtgggc gctaagattt tagatgctcc ccataaagaa gttggatggg caccggttta
18601 cttgaaggat cagacaattc ctaacttacc ggaaaaatta acagctcttc attggcacca
18661 gcaaatgttt gaaattccag aaggagctaa gttattgttc tcaagtgatt tagtaaagaa
18721 tcaaggcttt ttacttggtg ataatgtgat tggtttacaa tttcattttg aaccagaaga
18781 agataatgtg agagaaattg caattaatga tgttgattat ccgttagaga ataatgattt
18841 acatcaaact ggtgaagaaa ttattgctca tggggtaccg aaagaaaacc agaaagtaat
18901 gtttaaatta cttgatttca ttactaaata aaaagtgtaa attctatgct actaataaaa
18961 agtaagctaa tataaagctg taagtttttc gaggaggaaa atgattatgg cgaagatttt
19021 tatttttggt ggttctggaa gagttgctac agacttaatt aaaaacttag tagcagatgg

19081 caatactata acagcagctg cacgccatcc agaaaatatt attaaacttg atggtgttac
19141 ggcagaaaaa ttagatttac atgctgatgt tgatgatatt gccaagcaag taaaaggttt
19201 tgacgcaatt tactttactg ctggttcacg tggtaaagat cttattcaga cagatgccat
19261 gggtgcaatt aagacaatga tggctgctga aaaggcaggc gttaagcgct acattatgct
19321 tagttcgatg cttagtttag atattaattc atggaagaag attccaagct tagaggatta
19381 tttagcagct aagttctttg cagatactta tttgatggat tctacaaatt tggaatatac
19441 tattttacaa ccaggcagtc ttattgaaga aactggaaca ggtaaaattc aacttaatgt
19501 tgaagcgaca gattcgaatc caatccctga tgttgccaaa acattagcag caattctgaa
19561 atatccgaat acaatcggca aggttattcc aatgagtagt ggtaagacac caattgatga
19621 tgctttgaaa gaaatctaat aaagagaaaa aagactccat tatgggagtc tttttgtgtg
19681 ttaaggaaat atgctattta aaatttaaag acattagaaa ttgcttccaa atatttttga
19741 tctaactcat caaaattagc aattttagtt gaatcaaagt caaacacacc ccataattca
19801 ccatttttct taaaaatagg aataacaatt tcagaatttg aatcactatc acaagcaata
19861 tgtcctgcaa actcatgtac attttttaca atttgagttt gcctttcttt tgcagttgtt
19921 ccgcaaactc ctttaccaac agcaatatga acgcatgctg gttttccctg aaatggacca
19981 aggattaatt cattattttc atagcgataa acaccagcga agttaacatt atcaagtgaa
20041 ttaaataaga gtgcagaaat atttgcagtg ttagcaatcc aatcactttc tccgtctact
20101 agagcttcag cttgttttac taatagttgg taattgcttt ctgttgttgc tgacataagt
20161 ggtacctcaa tttttagtaa atttatttaa ttttaacact tataaaacat ttaatagaga
20221 aaattatatt tttaatttaa acatttgtct ttcttcacat cctaatcaga tttatgttag
20281 cagtttactt tatttgttgt ctggattcaa atcgctcata ataaaattga gagaagggta
20341 aatgaagaaa ggatttgtcc aattatgatt ggaaaagaaa ctcaaatacg tttagtaaat
20401 aaattagaaa atatacacca cgttgttgtt caagcttcag cactcgatgg aagtaatgta
20461 tttgtcctac aattacttca tagacagagc gatgttattg tttatcaaac tccaaatgat
20521 agcgaaactg tgacctttaa tgaggatcat ccaattttat acttgaaagg acccaattca
20581 gctggtacag cgggtggaca tactcaaact tgggtacaaa gtggagaaga caataaatgg
20641 ttcgttggaa ctaaacctaa aaagcatggt aatacttatt ggacaacgca aattgcgcga

20701 gtaacagttc ctggctatca aactcaaatt tttaccagta atacggagtt
gccaagactt
20761 tcttatctta atcgcgcagg ttcaggctat ggcgatggaa gtgtagctta
tcctggcaaa
20821 gatttagtta gagtagaagc agctgtttca ccgaataaac aatatttctt
aattgcaagt
20881 attgacatca atcacacagg tcattttgct atctataatc ttgatgaagt
taataaaaaa
20941 ctagatcaag cagaagaaaa agctgaagat gttaatattc agagcttgaa
ttgtttaggt
21001 gcatttaatg tcccgcattt taatgatcaa aagattattt caattcaagg
ctacggaatt
21061 gatgataata agaatattta catttctagt cagcctagcc cgcatacaac
ttttttagga
21121 tttccaaaac aaggcaaacc acgtgaaatt gttaaaattc cctggggaat
atccgatcca
21181 agtaaatggt cagtggtaaa tttagacaac agtttaaaat tagatgcact
aaacttttgt
21241 actgaatttg aaggaataca agtaacaagt gattgtcttt acttaaccgt
tgcctatcac
21301 caacgaaaca gtgatttgac tactttgatg aatcgaatat atcaggttga
aaaattttag
21361 aaaatggaca gactattgag tttgtctatt ttgttataat tgataaaaat
gaggtgtaaa
21421 aattgaaact aagtcgttgt agttggggaa acagtaaaaa ttctctgtat
caaaagtatc
21481 atgatcaaga atggggaaag cttaatttag attcaactta tttatatgaa
atgctagttt
21541 tagaaagttt tcagtcagga ttatcatggg agacaatttt aaataaacga
agaaatttta
21601 gaaaagcctt tgcaaatttc gattatcata aagtggctga attcaatcag
gatgattttg
21661 aacgattgat gcaagataaa ggaattgtac gcaatcgttt aaagataaat
gcagcaatta
21721 ataatgcaaa aatattggtt aaactagaaa aagaaaatcg gacttttgaa
aattttctaa
21781 ctgaatttat tcctaagcca ataatgcatc atccacaaaa gatggaagat
attcctgctt
21841 cggatggttt atctacgcaa atttcaaaag aaatgaaaaa acttggcttt
aaatttgtag
21901 gaccagtaac tgtgtactct ttcttacaag cggttggatt aattaatgat
cacttagaaa
21961 attgtagttt taagaatgga ggacgttgat gtctttatta ctgccgacaa
aacaagtagt
22021 aatcatgttc attttgatga ttgtcggctg gatttgttat caagttaagt
ttctacatga
22081 acaaacggtt aaggatctaa gtaaggtcct tttatatgta gtatctcctt
gtttgattat
22141 taactcattt cgacagtcct tttcggctgc tagattatta caatttgggc
tagtgttttt
22201 actagtaatt gctttattta tatttaaaat tattactagt gagtttattt
ttggtaaaaa
22261 tttagtcaaa gatcgtcaga aaagaacagt attgcgatat gcaggcacct
ataccaatgc

22321 aggcttcatg ggagttccat tagtacaggc aattctagga actaaaggag tctttttgc
22381 agttccttat ttaattgctt ataacatttt catgtggaca catggaatta gaatgttcac
22441 acagaaaaaa cagtcattta gagaaagttt tcggcaagcc gtgattaacc ctaatattat
22501 tgcggcggtt attgggatga ttttatttat aacgcaagtt aaattacctg atgtagtttc
22561 agatccaatg aattatattg ctaatttaaa tactccctta agtatgatcg taatcggtac
22621 aaatcttggt tccattaatt taaaagcaga ttggcaagat aagctagctt ggagcggagt
22681 gtttgttaga aacctatttt ttccaatagt cattttaggg atcttatatg ccttaccatt
22741 accggcaatt gccaaaatga ctacgttaat tatggctact tgtccagttg caggtgttgt
22801 agttctcttt agtttactta gtaattttga tgtgaaattt ccaacgaaat taatgtgttt
22861 atcgacttta ggagcgatta ttactattcc tttagtgatt tttttagcta cgttgatagg
22921 cttataagct aaagggtgag gaaagataat gaaaaaagat agttatttag caatcagtaa
22981 aacaaacgca tcaattaaat ggatgctaat tgcgtctata atttatctag tggtttattc
23041 cctagctggg agattagaat ttcagtctca atcgatttgg atgcaaatct tgtatttaat
23101 cattgtctgc gttttggcaa taacgtttaa aaagttctat aagacagaca aagtttttat
23161 taaattgcct aaagaaggaa aagtaaagat catcacaatt attttttgta ctctggtgat
23221 cttactctat gctctattgg gtagtgtggt aagttgggcg gaagttttaa attcaatgcc
23281 tgaaatgatg tggggatcca tttgcgttgc tttggctgca ggaattggcg aagaagtttt
23341 atgccgagtt ttactattta atttgtttgc taaaattttt gaaaataaaa aatatgtttt
23401 agtttgggct tctttagctt cgtcaatctt attcggatta tttcatttga ttaatttaac
23461 gcatggagca gctattaatg ctactatgca acaagtattt tacgcaactg ctattggatt
23521 aatattttca tatattcata tttttactaa tcgaatttgg ttgtgtattg tgatgcactt
23581 tttattagat ttacagccca atattggaac gatggaagca caggcatctc catgggggtt
23641 aattttatta atctttggta cagcaatgat tgtttcttta ttttctattt atgtatttaa
23701 tagaagagct aataaggtgt ttgaacatta aaaaaagcag atcaactgat ctgcttttt
23761 acatacttaa ttaatttcca aaaggaattt tgtaaagaaa aattccgata attgcaccaa
23821 gagtaggagc gataacagga atccagccat aactccagtg tgcgccaccc tttttattag
23881 ggataggtaa taatgagtaa acaagtcttg gaccaaaatc acgtgctggg ttaagagctg

23941 gaccagttgg accaccaaga gccataacta atgcagtgat taagaatcct acaccgatat
24001 tagcaatatc aacagcctgt ttgaaaaaca taccccttgta aagtccgatt gcaacaaaca
24061 ttaaaactgc agtaccaaca acttctgaaa taaaaccgtt ccatttgctg ttagtacaat
24121 cgctagtagc aaaacaagaa aatacaatgt caggatcagt agattcttta aagtgtggcc
24181 aatacattgc tagaacaaga agctgaccac aaattgcacc tagtaattgg caaattatat
24241 aaggagcgac ttgtgaccat ggaaagttac cagcagctgc ttgagcaaca gtaacggccg
24301 gattaatatg gttaccagaa attgaaccaa atagcatggc aggaagcata acaccaaagc
24361 caaaactgaa ggcaaccaaa atccaaccac cattagcctt accgtcaggt gcattaccag
24421 ttgttccttt taagaaagag ttggcaactg aaccattacc aaataatact aaaattaaag
24481 taccgaaaaa ttcggcaaaa tacttgagca tccaactatg ttccatagat acttttttcct
24541 ctcttgaaaa aataagtgaa aaataaacac agctactgat tatacctaaa gttctagaaa
24601 aatcctagat attttttaag attcttgcat ttttgagcaa aaagtttgca taatataaag
24661 ataaatgttt tagctctaat tacttgatgt aataagggct ctttttattg ttgaggaagt
24721 gctttaatga acaagaaaca gatcaggatg gtaacagtgg ccttgatgct gggaaacgta
24781 atggctggac tagatggaac gatcactaat actgcaattc cagcaattgt atcggcttta
24841 cacggaattc aatttatggg ttggattgta gcaatctatc ttttaggaat gtctgtttca
24901 ataccaattt ggaccaaaat tggtgaaaaa atcactaata aattagcttt tgaaattgca
24961 ttaggtctgt ttgttttagg gtcaacttta gaagggttag caccaaacat ctactttttc
25021 ttagtggcaa gaatgatcat gggcattggt ggcggtggta tgggatcact accttatatt
25081 atcgctggct atgtttttcc aaatattaaa aagagaacac aaattttagg ttacttgact
25141 gcaagtttta atggtgcagc aattctggga cctttagtgg gtggctggct aattgatgcc
25201 ctatcttggc actgggtttt ctatattaat attccaatcg gattagttgc tttactaatc
25261 gccctagttt actacaaacc ggtgacacca aaatctgcac cagtctttga cttaagagga
25321 gcatttttat tagttagtgg tttgattatg tttctgatgg ggattcaact cttaggttta
25381 actgcaactt ggattgtagt cggattaata ttattgagct tagttctttt aattttcttc
25441 ttcttacacg aagcaaaggc tgaaaatcca attattccat tatcaatctt tagaaacaga
25501 gatttaaatg gtgatctaat tctatttgca actacctggg gtgccttcat tgccgttaac

25561 acttatttac caatgtgggc acaggcgctt ttagggatgt cagcgttaat gggtggaatg
25621 actttaattc ctaactcagt tgttgaaatt attgcttcac aaactgttgc tacaattcaa
25681 gaaaaaatta gaacttttac tttggtaatg attggaattg tcactatgat gatctcttct
25741 ggtggattat ttttagcaaa taatcacact ccgttatggg tattaattgt agtcggtgct
25801 ttttctggaa ttggtgttgg ttttatcttc gtagcactcc aagtaaaagt tcaaattgac
25861 gctggaatga aatatatggc gactgctact tcgacatctt acttaattag aatcttagcg
25921 caaacagtaa tggcagcagt atatggtgtg attatgaatt tggctttagc tagcggaatc
25981 aattcgcata gcaatattac gatgaagatg atgaatgaat tgagtgatgc aaaatctgct
26041 aaattattac cacaacacct tttgccagag atgcgtgaaa tttttcatag tggtatccac
26101 gaaataatgg cagtttcttt tattctattg ttgattgcta ctgtttttaa tttctatttc
26161 aacttcaaac agccaaataa aaaaattaaa aattattcaa aatagttctt gtttttaagt
26221 aagtgagcaa gtatatttta gatagattat tagccttgat tattgcgaaa taattggggc
26281 tttttatttt ttagaaaagt aggtgtagac atgataaaaa agcaagtaac gatggtgacg
26341 tttgccatgg ttttagcaaa tgtaatggca ggattagata gtacgattat taataccgcg
26401 attcccgcca ttattgcgga cttacatgga atccaattta tgggctggat tattgcaatt
26461 atgctcttag ggatgtcagt ttcaacccca atttggacca aagtcggtga aaaaaattggt
26521 aataaagcca cctttgaatt atcgttactc ttttttgtct taggctctct ttttcaagga
26581 ttggctgata atatgtattt cttccttttg gcacgtgctt taatggggat cggtgctgga
26641 ggaatgggat cgctgcctta tattatggct gggtttattt ttgataatat taaggcaaga
26701 accaagattt taggttattt aggtgctgct tttagcgttg cagcgattgt gggaccacta
26761 gtcggtggct atttagtaga ttctctttcc tggcactggg tcttttatat taatattcca
26821 attggtcttt tagcaatttt attatcgtta atgtattatc acgaaggcaa gatcagaaaa
26881 acgcctaagt ttgatatact gggttctttt ttaatcattg ttggattaac tttgctttta
26941 ttagggattc agttgcttgg tctaactaag ccatggagtg tagcaagttt aattattgct
27001 ggtttagtgt tattagtttt attctttatg catgaaggag ggcaccccaa tcctgtagta
27061 ccgattagta tgtttaaaaa tcgagcttta gtcggtgatt ttttactctt catttttagc
27121 tggggtgcat ttttagcaat taatacatat ttaccgatgt gggcgcaagg tttgcttggc

27181 acaactgcgt taattggtgg aatgacctta attccgaatt ccttagttga
tgtggtagga
27241 actttagtgg ttaatccctt aaaggcgcgt tttaataatc gaacactact
ttcgatggga
27301 ttaatttgta ttttgatttc gtccttagga ctagcactgg caccgcaaag
tactaatatt
27361 tggtggttgg caattattgg aactttttct ggctttgggg tggggtttat
ttttgtattg
27421 cttcaaatta aagttcaagt tgatgctagt gagaaaaaca tggcgcctgc
aacatcactt
27481 tcatatttaa ttagaattct ggctcaaact gtcatggctg ctgtttatgg
tgtgattatg
27541 aatgtacaat tggccaaggg agttgctgaa aattcaggta taacgatggg
gatgcttaat
27601 aagttaagcg atgcttcatc tgctaaaagc ttgccacaga atttattacc
aacaatgaga
27661 aatatttttc atttaggatt gcaagaaatt atgtggtgtg ccactgcttt
gcttgtaatt
27721 gcgattggat tgaattttat ttttaataaa aatagtaaat aagaaaaaag
ctggtagacg
27781 aatcctaaag ttggaattgt ctatcagctt tttttgtagt attaaatcgc
tgtagtctaa
27841 acactactta gtgctggcac taataaatta aagaaatatt gacattttaa
taaaaaaata
27901 atattattgc ggtaatttct aaatggttat ctccaactaa cagatatgta
tatttatcag
27961 ttcccttaga aaaagcggac gctattaaaa ataattatag gatctagatg
attaaaaatg
28021 agttttcaaa aattaattaa aactaatttg ctcttattca ttgtaataat
tgttttagag
28081 attttgtttg cagcaggatc tgcaacaagt tcgtatatta tacaattcgc
ttataatcaa
28141 ctagttaaaa atattcttct agttttttg ctaattattg ccagtagtgt
ctttctttca
28201 ttcctttctt atattttgag ctctttagct acatatcttt ttagtaagca
aacgcaaaaa
28261 tatattcaca gtattaggca taaattaata agcaaatact atcatgacaa
ggcgcctaaa
28321 gtagctgaaa tggaaaatga attaaatagc aatttacaag tgctgactaa
aaattatgcc
28381 gatcagtctt taagtattat ccaatcaggt tttttattgg ttacgtcaat
tagcagcctt
28441 cttctaatga attggatgct gacattgtta gcaataattc tatcagttat
tacgctttat
28501 attccccgct ttacaagaca gaaagcttct aatgcaacgc aaaaagtagt
tagtagaaat
28561 agtaaatact tgttagctat tgaagaatgg tttaatggcc tagaagaatt
aagaaaatat
28621 actgctttta ataaattaga cttagtcatg caaaaagtta gtcagaatct
agaaacaagt
28681 ttcgtaaaaa gaaaaaaggt aatttctgtt gctgatttcc ttaatggatg
tgctaattct
28741 ttttctcaaa ttgcgattac tctgttagcc gcaattttgt tttttaatca
tcaagtaact

28801 tttggagtaa taatagcagc aggtaacttc tcctctatga ttttaaattg tttattgaca
28861 attactacat cgctaacaag aattcagtca gttcaaggtc ttaacaaaca aattattgaa
28921 tcccagaagt tagcctcttc tcataaagaa actaatactg atgaaatata ctcaattagt
28981 acgcataatt taagtatttc atttaaaaat ggagaaacga tctatttccc agatattcat
29041 attgaaagag gagaaaaagt gttgcttagt ggcgacagtg gtactggtaa gtctacgcta
29101 tttaagttaa ttcttaataa attacaacca actaatggac aagttatttt tgaaaataaa
29161 tatggtaaag aagttagtcc tgattatgct gagataggat atattcctca agatgggaag
29221 ctttttccta cttctattat caacaatatt attatgttcg ataataaatt gaaaaaactg
29281 gttaaaaaag cagttgaaaa taatgattta agtaaagata tagctagtat gccaaatgga
29341 cttgaaacgg aaattgattt agatacgaat aatttttctg gaggtcaaaa acagaaaatt
29401 attttagctc gtaatgagat acataatttt tctattattt tagctgatga ggcaaccagt
29461 gcgatagatt caaaaagtag ctatcgaatt ctaaaaaact tagtatcatc gaataaaaca
29521 gttattgtag tagctcataa tttaactcca gccattgaaa aactatttag tagaaagatt
29581 agccttgtga ataataagag gtgactataa gtgtctttta aagatctata tgctgttaat
29641 agaataagaa tgatatcgct attaattcta gaattcctaa cttctggttt aataattggg
29701 gtcagctata ttaatacata tcaaataaca gccattaaga accgaaaatg gcaacaattt
29761 atttcttga ttactctatc tttaattttg tttattataa gctacgctgg tcttaatgtt
29821 tgtcagtatt ggattgaaaa acaaattcag caatacaatc atcaaataag atttaaaata
29881 gtaaatcatt atttttatga taataaaacg catagtactg cccaagttca gaatcgatta
29941 actaacgacc tgaacttaat aaaagattca aagcttgcag tatatacgga tattccatac
30001 tatttagcgc aaattatctt tgcatcaatt ggcttgctgc tgtttcattg gagcctatta
30061 attgttgttt tgatattagg aacgcttagt ttttacttgc ctaaacttct tcgtcccgcc
30121 atgcaagcag ctgctctaaa gttatctcaa gcaaataagc aatatttgga tacagctgaa
30181 aaatggttag atggtctatc agagctcaaa aagttttcag taggttcaca gctgtctagg
30241 attatggata atgcttccga tacattagaa aacgcaaaca tagcgagaac aggtactatg
30301 caagagttag ccgtattaaa taaggctact tccgccttgt tacaatttgc tttattagcc
30361 gttacggcta tattagttac aaaccatatc gttatatttg gtgtaatagt aactgttgaa

30421 agctttagct cttatatcaa tgtatcagtt aagatgcttg caactgaatt aggacagatt
30481 cactcagtag accgtttaag cagtgaagtt aatgcggaca cagctgtagt tgagcatacg
30541 ggcgacttac aatctccagc tagtttaatt acaaaaaatg tgtcagtaga attttctaac
30601 ggtaaaataa taaaatttcc tgatttaaat ataaaaactg gtgagaaaat tctgttgact
30661 ggtgactcag gatccggaaa aactacttta tttaaagtac ttcttggtaa gattaaactt
30721 cataccggta tgatcaattt taaaaataaa tatggtgatg atattaacgt cgataaggct
30781 aagataggat atatcccaca agatccgatt ttatttcctg atagtatcga aaataatatt
30841 acgatgttta attcaaaatt gaaaaataaa gtgctagatt atgttaaaga cgtttctttt
30901 gaatctgata ttagaaaaat gccgctaggt ctaaagacag aattaaattt gcaaaaatta
30961 aatatttctg gaggtcagcg acaaaaaatt gtgttagctc gggcttgcgt tcacgatgac
31021 gattttattt tgattgatga ggggactagc gctatagata aaaaagcaac gcttgatatc
31081 ttaaagaagt tattagatgg cccatacact gtgatattta tagctcataa ttttaatgaa
31141 gaaatgaaca gtctctttga tagagaaata cacttaactt agatgcgttt tttgtattat
31201 taattatttg ctttttctat ttttccaact gcaattggaa gagtaacatg tggaccatat
31261 tttactacat ttatattgaa aggaaccatt gttgtgcctg acactagctc agtttctggt
31321 aaatcaacaa atccatctcc atttaagtca ttgtccattg ttgctttacc atcagggaaa
31381 ccatggaagt gttgctagtg ttcgatattt gctggagtgt cgaacatttc aatcataata
31441 tgtaacgtat cttgttcctc atcttcagta aaagtagcag ttccgtgagg agagcaggct
31501 atttttcgg cattaagtgg tccaatttta gcaatatatt tctttgtttc aatttccttt
31561 ctaaaaactt aaactttcaa ctgtattata gaaaacaaaa ttgagaggaa gaaaatatta
31621 tgcttataaa ttagtaatag ataatgataa cggagaattt agctattgat gacgatatga
31681 ttctcaatat atgataagag ctcattaatt ggcttttgat aatcatgacc aacccaccaa
31741 accaacagca tgtaaatacc acctgactga tagctaattg catagggagg aagttttaaa
31801 ttagggtgag agtaattaat taattctttc aaaaatttgg tttgctggtt aagtaattct
31861 ccttctaaac cagcattaag gaataatttt attagatccg gatgatcctt aaaaaattta
31921 aaatagactg ctaattgatc cttgaagtcg gttagatggc gatttacttc ttttaccaaa
31981 ggctgttaaa gaaatcttga atttagtaat gacaccagaa aaggatttgc aagataagaa

32041 aattaatgaa gtctttgacg atgaattttt taagtcaaac ttctggttat
attggcaaac
32101 aatgtttgca tttatgcctt gggcaagtgc aatggaaatg cgtcgttact
taatgcggtt
32161 tgtacagcac gtagcaactt tgaaaaatct atcgtcactt cgttttacta
agtacaacca
32221 atacgaagac ttaattatgc ctttaattag ttacttaaag aagcatgacg
taaaattcca
32281 ttacgataca attgtagata acattattgt taacagaact gaagatgaaa
aggtagctac
32341 ggaaattaag atgactgaga agggtgagca aaaagttatc aaattaacgc
ctaacgactt
32401 agtcttcgta actaatggct caattactga aagcacaact tatggggaca
acacgcaccc
32461 agctgaacaa aaacatgagc ttggaccaag ttggcaattg tggaagaact
tagcagcgca
32521 agatgaagac ttcggtcatc cagaaaagtt ctgcgaaaat attccagctg
ctaactgggt
32581 aatttcagct acagttactt tcactaatga tgatattgtg ccttacattg
aaaaggttaa
32641 taaaaaggat ccacattctg gttccattgt aactagtggt ccaactacgc
ttaaggattc
32701 taactggtta cttggatatt caattagtcg tcaaccacaa ttccataagc
aaaagccaaa
32761 tgaactgatt gtttggttat atggcttgta ttcaaacact aagggtaact
atgtcaaaaa
32821 gactatgcct gaatgtgatg gtattgaact ttgcgaagaa tggctttatc
acatgggtgt
32881 tcctgaatct gagattaaga agatggcaat tgatgcaact acgattccaa
atcatatgcc
32941 atatattacg tcttacttca tgccacgtgc attaggcgat cgtccaaagg
tagtgccaaa
33001 gaattccaag aatttagccc tcattggtaa ttttgctgaa actgagagag
acactgtctt
33061 tactactgaa tattcagtac gtactgctat ggaagctgtc tacactttgc
ttaacgtaga
33121 ccgcggtgtt cctgaagtat ttgcatctgc ctttgatgtg agaatgttaa
tgaacgcaat
33181 gtattactta aatgacagaa agaagttaac tgaacttaat ttgcctttac
ctgaaaaatt
33241 gatggttaaa gaaggtttga agaaagttaa gggaacttac gttgaagaat
tacttaagaa
33301 gtataagttg atatagataa aaatttaatt atttgactaa tagggatgag
tggaatagct
33361 tgtccctatt ttgtatagta ttagaaattt gctttttatt gaaaagtatt
ttttaattag
33421 agaagtaaat tatattatta gaaaagagaa ggatttttat tttagacgaa
caacccgccc
33481 gatggggctc taagaattat cgtaaagtta aagatttata ttatagtgct
tttccagaat
33541 gggaaagatt ttcatggatg tcaatggtat taatgagttt gcgcaagaag
ctacaactta
33601 atgccgtcta tgatggagaa gtattttgcg ggatggtttg ttactacatt
agtgacaata

33661 ctgtttatct ggcttactta gctgttgaat ccgaattacg tggtaatgga
tatggtagca
33721 agattttaca tatgcttgaa gaaaaatatc cagatcagca aattgtgtta
gatattgaac
33781 cgcttgatcc tgatgccgag aattatcatc aacgtgttag ccgtctgcgt
ttttatcaaa
33841 agaatggatg gcgtagaact caccagatgt tagttgacgc agatggtgaa
tttgaagctt
33901 tagttgatca aaatcatttt gataaaaagg attttgctaa gaccttgaaa
caaatgagtt
33961 taggctttta tcgctttaga attgaaaaat aatattagca gtcacttaac
tattgagtga
34021 ctgtttttta tgaaagtata tttaaaatat aaagttgaag aatttggcga
tatgtattag
34081 aaaaagtaaa aaagcttgaa acagagaggg ggattgtttc aagccttttt
actagagaat
34141 attattttta atgtaggtga gaacaccttg ttcattgttt gatgttgttg
tatgattcgc
34201 gacctttaaa agtattggag ctgcatttga catagctacg ccatcgccaa
cttctttaac
34261 catttctaag tcatttccac cgtcaccgaa agcagtcatt tcagtaagag
aaataccaag
34321 tactttacct aattctgcta agccatgtgc cttatgaatt cctggtaaga
taagatcgat
34381 atcgccgtgc ccactacttg ttacattaca gtaattgcca aggttctttt
ttagaaaacg
34441 aatataataa tctgtctttt catcaggaca ggaaatagaa aatttcagaa
ctttgtcgcc
34501 aatttcacta aagtcgtgta ttttctttaa acgatagtaa tacttagtag
agatttcgta
34561 ataagcgtcc gtaaattgaa ctggaaaata ggcacactct actcctgata
ctaaaaattg
34621 taattcagct tgctttaaaa gaaaatcttc aatcttttta acgttttctt
cagagaaagc
34681 atgtagggct agaatctttt ctttggttct aactaaggca ccattttctg
caacgtaaac
34741 tacagcagga aatttcttaa agaaagtagt aatttgataa tattgattac
cactagcaac
34801 tgtgaatata atgtttcttt tttctaattc ctggtagatt ttagcaaatt
tttcttcatc
34861 atagctttta tcgtctctta agaaagtacc atccatatca acggcaatta
atttgtcat
34921 tttgcagtct ccttttaata atttattaaa aaataaattt atctaaagcc
tttgcgatgc
34981 cgtcatcatc gtttgatgca gtgataaagt ttgcgtgatc tttagcttct
tgacttccat
35041 tacccatagc cacagcagtt cctacgatat caaacataga aatgtcattt
ctttcatccc
35101 caaaagcaat catttcgtta gtgctaatgc caatcttttc acctaattct
ttcaaaccgt
35161 ttcctttgtt tacatttgga tgtagacatt ctaagaaatg gtcatctgca
cgaacaatat
35221 ataaatcttg atcaaacttt gttcttaata ttggttcaaa ttgatcaaga
agaactttat

35281 cgccaacaaa gcagccttta gaaatctgaa agtcgttagg catttcatca ggagttctaa
35341 tgaacagtgg agccgtattc tcccaggctt gcagtaattc aaagtaatct acgtcatgat
35401 ttgcagtaat aattctcgaa tctggatcaa caatattgaa aggaactttt tgctctttag
35461 caaatttagt cagctcgcgg taaagcttat tgctgacaag atcttgcgtc attatttttc
35521 catctgcggt tctcgtaatt gcaccattta aagttacggc atattgatca ctgccttcaa
35581 ttccaagttc tttcatgaaa tgcgcaagac ccgcgattgg tcgaccagag caaagtacga
35641 ccttgattcc ctgatctaaa gctttttaa ttgctgcttt agtagaggga agaattttat
35701 ttttagagtt taaaagtgta ccgtctgtat ctaaggcgat taatttaatc atttttattt
35761 cctcctcgta tttttacac tattaagata ccatttataa atagaaacta agtgtcattt
35821 ctgtaactaa gaagtaatta ttttgaaaga gattacaatg gttgagcatg aggaagttca
35881 aaataatcaa agactaccat tttattttta tcgtgataat caggtaaatg agccagagca
35941 ttggcatcag ggaattgagc taaactattt aattaaagga aaagatttgc gttttgcact
36001 tgagggtaaa acttaccatt tcaatagtgg cgatatctgg ctagttaacc gccggcaaat
36061 tcatagtgct tcaggtgaaa atgaagactg gaagtatgaa ggttttatta ttgatgatga
36121 ctttttattg agccagtatc caacgagtat caattggaat cttgatttat taggtaaaaa
36181 tagtactaat aatcaaactg cttataagga gttaaaaaaa gaagttgttg aacttggtga
36241 attgtgtcaa aaaagcttga ctgacgctcg acgttttatg agtttaagtc atttaatgag
36301 aattattgtc ttacttgatc agaattttaa taagaaagag ccagttacta gaagtcctaa
36361 tcttcccttg ggggatgaaa taattaaata tattaatgat cattttcaag aagatattca
36421 ggttagtgat attacaaata aatttaatat ttcgcatgta actctgaatc agcaacttaa
36481 gcagagcaat aatatgagca tgggtaaata cttaaagctg gttcgtttga tgcacgctag
36541 agaattgttg ttgagcacaa ataagtcaat tgaattggtg gcgtttgaga gtggattttc
36601 gaatgcacgg gtgttgaata gaaattttaa agaatggaaa cataagacgc caacggaata
36661 tcgaaatgag tttagtaagt attttattaa ataaattatt aatattttt agcaataaaa
36721 atacaataag atacacaaaa atatttagta atttgtcaaa acaagataaa aaatttgttt
36781 tggcaatggc acaaagctca aacgaacggg taactattaa agagatacga gaaaaactag
36841 atcgaccgag taactttatt gctaattatc gaagacgttt attagacgat gaaattatta

36901 aagctacaaa ttatggagag gttgcattta ctttgccatt tttttaagga gtatatattg
36961 cagcaatata gatttgaaca aaaaatggac tactaaaaaa gctagaatcc gtattacacg
37021 ggttctagct cttttcatat cttcaactaa ttaaagtatt tttgtccttt ttctttcttt
37081 tcagcttcta atgctttttc actggctgcc ttcttaggta agatccagtt cattaacata
37141 ccgaagattg tacataaagc gactgaagtt aattggaaat taccaagttg gaggtaggca
37201 ccaccaatac cgataactaa aataactgag gcaattaaca tattacgttt ttcaccaaaa
37261 tcaatcttgt cgtcaactaa gatcttaaga ccgttagatg caatagttcc gaataatagg
37321 aaagaaatac caccaataac tggcattgga attgattcga taaccgcagc gaccttgcca
37381 acaaaactga aaataattgc aaatacagca gcgccggcta atacccaaac tgagtgaact
37441 ttggtcattg ctaatacacc aatattttca ccgtaggaag tggtaggtgg accaccaata
37501 aaaccagcaa taattgttga aaggccatca cccatcatag ttctgtgaag accaggattc
37561 ttaaagaagt ttctcttggt taaagagtta agaaccataa tatgtcccat gtgttcagtc
37621 atagtaacaa aggcaattgg agccatagtt aaaatagctg caggatacca cttaaagtgg
37681 tagctgattc caggtaggtc taaagcaggc atggagaacc aggcagcttt tgctacacca
37741 gagaagtcaa caattcctaa gacgcaagat aaggcatacc cacagacaat tccaagcata
37801 atggaaacta gactagtaaa gcctttaaag cacatttgga aaattagtgt cataactaat
37861 gtgaagattg caactccaaa gtaggttaag ttgtatttag tgcctttcat catggcgtcg
37921 ttagcggcag tagttgctag agataaacca attacgataa taattggacc aactacaata
37981 ggtggcaaaa ctttattaac ccaatcagaa ccaacttttg caatgactaa agctacaatt
38041 acatatacaa gaccagcagc aatcgcacct tgagcaattg ctggatagcc atcttgtctc
38101 attaaggctt gcatagtagc aacaaaggca aaacttgaac ctaagtatgc tggaatcttg
38161 aagtgggtaa gaagcatgtg gactaatgta cctacaccag atgaaagcaa ggcaatactt
38221 ggattgattc cgactaaaat tggaactaaa acggttgaac cgaacatggc aaacaagtgc
38281 tgcattgaaa gcaaaattcc ttgtccaagt tcaggctttt cataaacgtc taagatcgca
38341 tcatcattat gaaatgtgtc tttaggcatc tttttcattt tttcctcctt gaaaacgaca
38401 tttggatgac ctaaagaaaa aacgactatc taaagaatta cctttaagat agtcgtttct
38461 gtttaaaact atgcaaaaaa tagtttacgc gcagacttcc ttgatagcct cacaggacta

38521 aattaaaggt catcacttat atttcttttt tatttaacta agttatagga aaaaagtcaa
38581 gtgtgaaatt tgagctgttc taacttgtga gtttgaaagt tcacaaaaaa tttaaatatt
38641 ttatttgtaa gcgatatcgt tgctataata cagcttagaa gggctatttg gcaattttta
38701 atgaaaatga acacaaatta actttgacaa acataaatga acatatataa taaagatgta
38761 atgagttggt tagccaaata gaattaaggt gaacaatgct aaaaagagaa cgattaagaa
38821 atattttaga aattgtgact gatagaaggt ttgtgaccgt tgatgaattg gctaaggcgt
38881 tgcaagtttc agatatgaca attagacgag acctcaatga attgaatacg actggtaaag
38941 tgatgcgaat tcatggggga gcgcaagtga tttctcagca agattcagtt gaaaagaatt
39001 atatgcaaaa aagagaaatc aacatttctg aaaaaaaagc agctgctgaa gttgcaagta
39061 agattgttca agatggcgaa actatttatg ttggaccagg aacaacctta gagtttatgg
39121 ttgctaaatt gaatcaaaaa aaattacgag ttgttactaa ttctttacct gtttttgaag
39181 cagctaaaaa gaatccaaac aattatgaac taattttgat tggtggctca tatcgcagag
39241 ttagcggagc atttataggt gcattggcaa ataatcagtt aagaggaata tcttttgatc
39301 gtggctttgt tggcgtaaac ggaatcaagg acacctcaat gatgactgct aacttagaag
39361 aaggacagac gcaaggcttg ggattaaatc gctcacgtaa aaagtatgtt gtggctgatt
39421 atcacaagat taaccgcaat gacttttatg agttttatca cttatacgat gttgatggtt
39481 tgattacaag tcctgggctt gatccggata tcgtaaagca ttacagccaa atcacgactt
39541 tatatcaggg tagtgaatag cgaaaggaga acaacatgaa agttgttatt ggtagtgaca
39601 aaaaaggttt tgaattaaaa gaaaaagtta aaaagtatct agaagatggc ggctatgaag
39661 tattagatgt aacgccagaa ccagcagaag actttgtaga atccagctta aaagttacgc
39721 atgaagtctt agaccatggt attaagaagg ctattatgtt tgatgaatat ggcgttggtt
39781 cagcaatggc atcaaacaag gttaagggca tggtaactgc tgatgttaac gaagaaagaa
39841 ctggacacat gactgctatg cacaacgggg ctaaagcctt ggcaattggt agtggcattg
39901 ttggtgaaaa gcttgccgaa tcaatcattg attactactt gcatactgaa tatgcaggcg
39961 gacgtcacca agttcgttta gacatgcttg aaaaaatgat ttagaggagg cttgaaaatg
40021 tttgataatg atagaccaca accagaattt gtagacccaa agattgataa acatactgtt
40081 gttgcattag gtaatgacca cattgttacg ccaattaaga tggcattatc tgaccatttg

40141 aaagaagaag gataccaagt cttagacttt ggtacttatg acaacactag
aactcactac
40201 ccaatgtatg gtaagcgtgt tgctgaagct gttgctgacg gcagagctga
tgttggtatt
40261 gtaatgtgtg gtactggtat tggtatttca actgcagctg ataagaatga
aggtattcgt
40321 gccgcaatgg ttggcgatgt tgctcaagct aagtatgcta agcgtgaatt
aaatgctaac
40381 gtattaggta tgggcggtat tgttttagga agagacttta ttttctacat
tgctgatgca
40441 tttttgaatg aaaagtatca accaactgaa gaaaacaaga agttaattga
aaagatcgat
40501 aacattgcta agccaaatcc tgatcaaaaa gataacgaac acttctttga
tgaagaaaac
40561 aagaagtggg ctgaaggtgt ataccacgat tagttagaat ggggtaataa
catgagtaaa
40621 ttagaagaat ttagaaaagc aaatccccaa tataagattt taactttgga
tgatccatct
40681 tttaaagaat acggtgtact ctacactaat gactttaatt tagatgatgt
ttataaggta
40741 atggagaagg ttccaattcc cgaaaccggc atgaagtata ttgctaacat
tcctgaacta
40801 gctgacactt atactatgct tgctgtaaag cgtgatattt ttggtgaaat
tccaattgat
40861 gctggtgtta cattagggca ttccgatacc tttacagcat ttgaatatca
ccaatgttca
40921 gaagtgaaca ttatgcttga tgatgtgtta atggtgcttg gtaagcgtga
aactttggaa
40981 gagaagcatt ttgttgatcc aaatactgaa gctagaatgt tctacatgcc
aaaaggtact
41041 attgtagaac tctataatga tactcttcac tatgctccaa ttcagataac
taagaagggc
41101 tacaaggtaa ttgtggctgt aattcatggt actaacgctg ttttacctga
aggcgttaag
41161 agtctgaatc cacgcgtagt taaatgcgga aagttccaag tcgttcaccc
ttctcgtaaa
41221 gataagattg aacaaggtta tcaagtcggt ttaagtgggg atgtaattaa
aacaactcca
41281 cttgatgaat aaataataag cgcaataaac tacaatattt ataaatttcc
ctcaactttt
41341 attaatagaa acttatggag catatgatta tatgctccat ttttttatgc
aaaaataagt
41401 tcaaaattat ttttgaaagc gcttgtaatt taacgaacag tattatatga
taatggtgta
41461 gtaaatgaac aatatctaac atataaaaac aaaagtaaaa gttcatttac
agatactgga
41521 aaggtggttc atatgacagt ggcaactagt ttatttgctc ctgacgcagt
ttttaccagc
41581 gacaagacaa gtaaggaaga agtcctaaaa gaggtctatg gcaaattatt
aaaggctggc
41641 tatgtaaaag gaaatttcct ttcacatatt attgaaagag aagagcatta
tccaactgga
41701 attgacactt cgccaatttc gaaagagttg ccaaatgtcg caattcctca
tacagaaggc

41761 gaatttgtaa atgctcgtct gattgttcca atcgctttga aacatccaat taagtttcaa
41821 aatatgattg ctccggatga aagtttagaa gttaaattct tatttatgat tttaaacaat
41881 gatccggaag gacaggcaaa tgtcctagca cagattatgg ggttcttatc cacaacgcca
41941 gttgaagatt taattaggct gtttaatctc acttcaacta aagaaatatt tgattttatg
42001 agtgagaatt ttaagcaaga ataggaagat attttttggg aggtatttat tatggcaaag
42061 gaagttaagt tattagctgc atgtggtgca ggggttaact cttctcacca aattaaggat
42121 gctgttgaag aagaaatgaa gaaacgtgga cacaatgttc atgttgatgc tgtgatgatt
42181 aaggatgtaa acgaagatat gcttaatcac tacgatgcat atttaactat tgctaaaact
42241 gatttagcat ttgaacctaa gattccttta attgaagcag gtccaattct ttacagaatt
42301 ccagcaatgg ctcaacctgt ttatgacaag gttgaagaag tagttaaaaa ggtagaaaac
42361 gaatagcagt tattcctggg ggaatagaaa atgaacgcga taattgattt tgcgaatgct
42421 atctttaagc ctttaattga tatgggagca ccaacaatca tgttggttgt tcttactatt
42481 attgctctat gtttcagagt taaatttagt aaggctcttg aaggtggtat gaaattagct
42541 attgccttaa cggcgatcgg cgatgttatg aacttattga ctaccagttt tgcaccagcg
42601 atgaatcaat tcgttaagaa tactggtatg cataaagata tcatggatat gggatgggca
42661 ccacttgcca ctattacttg ggattctcca tataccttgt acttcttgtt agttttaatt
42721 atcttgaatg ttttaatgtt aatcttcaag agaactaaca cactagatgt tgatatcttt
42781 gatgtttggc actcagcctt tgttggtttg tttgcagtat tctgcggcgc accactttgg
42841 ttagcaactt tattaatttt acttattggt tacttgaaga tcgtcaactc agacttaatg
42901 aagccaacat ttgacgactt gctagatgct ccatcaagca accctatgac tactactcac
42961 atgaactaca tgatgaaccc agtaatcatg gtcttcaata agttctttga taagtgctta
43021 ccatggcttg ataaatacga ctttgactca gctaaattaa atgaaaaaat tggtttctgg
43081 ggcagtcgtt tcgcaattgg tgtttactta ggtatctttg tttcattact tgctggctac
43141 aactttacaa ctgagcaagg ttggaagaca atgtttactc tttccttcac agccggtgca
43201 tgtattgaat tgttctcagt tattggttct tggttcattg cttctgttga accattatca
43261 caaggtattt caaacttcgc tacttcaaaa tttaagggac gtacattcaa tgttggtctt
43321 gactggccat tcatcgctgg acgtgctgaa atttgggcag ctgccaatgt tttagctcca

43381 attatgatgg ttgaagctat ggttattcca ggcaacagat taatgccttt
aggtggtatt
43441 attgccatgg gtgttactcc agccttgtta gttgtaactc gtggtaagat
tattagaatg
43501 attatcattg gtaccattga attaccaatt ttcctttggg caggtacttt
agttgctcca
43561 ttcgtaactc aagtagctca ccaagttggt gctaacattc ctgctcacac
tttagtatca
43621 gatactacta tggaaggtcc tattgaaaaa ttcttaggtt acttagtagg
taacgcttgg
43681 aagcatcaag gaatgttcat tgtttatgca atcttagcca ttgctgctta
cattttaatc
43741 ttctggtggt actcaacaga aatgaagaag cgtaatgcta aatatgcagc
agctgctaaa
43801 gaagcatagt taataatatt atttagaaac ggtagagatg atttttatga
agacaaagaa
43861 agttagccgc gaagaagaag cacgacgtaa gcgagtatta tattggacaa
tatttgtaat
43921 tgcgattaat attccacaac ttttctttaa aaatattttt attgaaggct
ttgcaatgat
43981 tggtactatt tatgcccttt atcgcttggt ggtattcgat aataaaaaga
atcgtctcag
44041 ccgtaaatat tatgactgga agggcagacc tttagacgcc aagaataaga
attaagacag
44101 tttagaataa gttattaaaa aaagttaaga tttatatttt tagaaaaacc
gcacgcaaaa
44161 agagattgga attgaaacca atctcttttt atttagggac aataaataat
aaaataaaga
44221 aattctaata aaagcgttta cattagacgg attacatgct aatatacatg
tgtaagcaaa
44281 aataaatgtg gattgttact aatacgatta ggcaaaaccg cgaattttta
atacagatga
44341 ctaactgtaa tctcgtatta aaattcgtgg ttttttattt tttcctggag
gggtgagaga
44401 tggaataccg aatagttcaa atattaaaca ataatgttgc tattgttcat
accaggaata
44461 ataaacaaaa cattgttatg ggaaggggtg ttgcttttca taagcaaaag
gagatttaat
44521 ccaagaagag aatgtcgaca agatatttga gataagagat aaaaataccg
ttaatgatct
44581 aacaactttg ttatcaaacg taccgctaga ttttgtaact acaagttatg
acttaattga
44641 tcaagtgcag gcaaaatatc attttgctgc tgaaccttac atctacgtaa
ctttaacaac
44701 acacttgttt ggtgcatatc aacgcttaat caatggcgat gatgatgtta
attatttacc
44761 agatttaagt gataactatc cagttgctta tcaaatcgcg gatgatgtaa
ttaaaggttt
44821 tcgaaatagt ttagatattt cttttccaga gtcagaacat aagagtatcg
cccttcactt
44881 tatcaatgct catactagtg gaagtgttga agaagagaag agtcaaacag
aaaatgaaga
44941 aatcataaaa gttgttcaaa atgagcttga aagaaatgga atatatcgaa
cgcaaaaaaa

45001 tgccaacgat tatgacagat tgttagttca tttgaagtat tttattaatc ggcttaataa
45061 tcacgagcca gatagtttac cagtttctaa aggaatgatt ggagaaatag aagctagtta
45121 ttcgaaggct tggagaatgg ttcagagaat aaaagaatta atgcaagaaa aattagaaat
45181 aaagttatcg ttaagcgaac aaacatattt aacaattcat attcagagat tgatctaagg
45241 agaataaaaa tggacgcaga aaatcaaatt actaaagaag atatttcaat gactggtttt
45301 tcaattgttg catattcagg ggatgcaaga acttacttaa tgcaggcaat ggataaagcc
45361 agtgaaggaa agattactga agcaaaagaa cttgttaaga aggcggagga atcaatcaac
45421 ttagctcaca atgaacagac taagctcttg agtaaagaag ctggaggaga tgatatggat
45481 gtaaccttca ttatggtcca tggacaagat acattgatga ccacaatgct tttaaaggat
45541 cagatgaaga caattattaa cctttatgaa cgagtaaata agctcgaagg aaatagtatt
45601 gaataagtag tttaaaaata aatgattttt aaacttagga tgaggaggaa gaaaagatga
45661 atggttttac caaaacaatg gataaaatga agccaaaatt cgaaaaaata gcggctaatc
45721 catatgtttc agcagttcgt gacggcttta ttgctgccat gccaattatt ttgttttcaa
45781 gtttgtttac gctgattgca tatgtaccaa acgcatgggg attttattgg ccaaaggcag
45841 tagaaaacgc attggttttg ccttatagct attcaatggg cttgcttgct ttatatgtaa
45901 ctgcaacttg tgcgaagaat ttaactgatt ataagaactt aaagcttcct aagacaaacc
45961 agattaatcc aatgaacgtt atcttggctg ctgaaatttc attcattatt attgctatta
46021 aagttggtaa aaatggatta gatttaactt acttaggtac gcaaggatta atcgcttcat
46081 atattgttgg tttgatcgta cctaatattt actatgcatg tgttaagaac aatgtaacta
46141 ttaaaatgcc tgatgttgta ccacaaaaca ttgctcaaac ttttaaagac attttcccaa
46201 tgactttctc agttgcattg ttctggatag ttcaaattat tttaaaccaa ctctttggtg
46261 caaatttatc agaatgtgta gtaaaggttc tttcaccatt attccacgca agtgatactt
46321 atggtggttt agcattagtt gctggtgcga tggcattctt ctggttcgtt ggtgtacaag
46381 gtccttcaat tgttgcacca gccgtagctg caattgagac tactaacgtt ggcttaaacc
46441 aacaattagt tcatgcagga atgcaagcaa gccatgcttt aactattaac tgtcaagact
46501 acgttatgaa catgggtggt actggatcaa ctttcgtagt tccattcatc ttcttacttt
46561 tagctaaatc agctcaaaac aaggctgctg gtaaagccgc cgttattcct ggatgtttct

46621 cagttaacga acctattttg tttggtgctc caattatcat gaacccagta ttcttcattc
46681 catttttggt aacaccaatg ttcaatgtct gtgcttataa attctttgtt caagttcttc
46741 acatgaacgc tttatacaac actttacctt ggactgttcc agctccaatt ggtattatcg
46801 tatcttctgg ttttgctggt ttatcatttg tttatgtaat tttaacttta gttgttgata
46861 ctttaatttg gcttccattc tttaaattct atgacaatga cttatacaaa caagaacaag
46921 ctaagcttgc tgcagaacaa gcttctggcg tagctactgc tactgatgat tcaactgcta
46981 gtcttgcagc tactgacaaa gaagctaaag aaggtattac caaagatact aacgtaatgg
47041 ttatctgtgc cggtggtggt acttcaggta tcttagctaa ggccttaaac aagatggcta
47101 aagaacgtaa tttgccactt cacgcagcag ctcgtgctta tggtcaacac atggatatta
47161 ttcatgatat ggacttaatt attttggctc cacagatgga tagtatgaag gataacttga
47221 aagaaattgc cgatcatgac ggttctaaat tagtcactac tactggacgt cagtacattg
47281 aattaactca gaaacctgat ttagcattta agtttgttgt tgatagcctt gaaggcaaga
47341 atgaagataa ataattgagg taaaaattaa tgaaagaatt taaatttcca aaaggatttt
47401 actttggcgg tgcaactgct gcttaccaat gtgaaggtgc aacaaaagaa gatggtaaag
47461 gtgaagtaat ctgggataag tacttgcgtg aacaaggaac aattaatcca gatccagctt
47521 gtgattttta ccaccgttat ccagaagact tagaattatg taaaaagttt cacattaacg
47581 caattagagt ttcaattgac tgggcaagaa ttttccctga aggtgaagga agaattgagc
47641 caagaggtgt tgaatactac cacaagttat ttaagaaatg tcatgaagaa ggcgtagaac
47701 ctttcgtaac gcttcatcac tttgatacac cacaagcctt acaagacaag ggcggctggc
47761 tatcacaaga aatgttagat gcttttgttg aatatgctaa gttctgcttt aaggagtatg
47821 ctggcgaagt tagatactgg attaccatta acgaacctac atcaatggct ggtcaacaat
47881 atgttggtgg aacattccct cctgcataca aggatcaatt tgataagtgt tttaaagctg
47941 aatataacca gaatctagtt catgcccgaa ttgttaatgc ttataaagaa gcaggctata
48001 ctggccaaat tggtattgtt catgctcttc aaactgttta tccagcaaca gacagcgaag
48061 gtgacaagca tgctgctgaa ttaaaagacg ttttagaaaa taagttttat cttgatggaa
48121 cgttagctgg taagtattct aagcatacgc ttgctttagt aaaagagatt attgatgcta
48181 atggtcaaca aatgattgac atcaagccag aagatgaaaa agtacttctt aaagctgcaa

48241 aacaattaga ctttgtggga gttaactatt acttctcgaa gtttatgaaa gaatatcatg
48301 gtgacaccga aatcattcat aacggtactg gtgaaaaggg aactgatgtc aaccgtatga
48361 aaggcgttgg tgaagaaatt cacccgaagg acttaccagc aactgactgg gattggatca
48421 tttatccaca agggatgtat gatcaattga agcgtgttta cgatgattat cctttagttc
48481 attcaatcta tattaccgaa aacggaatgg gttataaaga taagtttgaa ggcgaagata
48541 agatcatcga tgatacacca agaattgatt atctaaagca acatatggaa gaagtagcta
48601 aggcaattga agctggtatt ccagtcaatg gttactttgt atggtcactg caagacatgt
48661 tctcttggac aaacggttat tctaagcgtt atggcttatt ctatgtcgat tttaagacgc
48721 agaagcgtta cgctaaaaag agtgcatatt ggtataagaa actagctgaa actcgagaaa
48781 ttcctcctgt tgattaaaaa gatcatttgt aaaaagctaa gtactaaatg aaagtactta
48841 gctttttga tggaaaagtt taaaagagtt taaaagtaga tatgaagttt aagtgaacgt
48901 gcaggtaaaa accagctaag cattcagtgt ttagctggtt ttgtttaatt tagatggaac
48961 ttccgaagaa tgtcaattcc ttgtttttga tatttattgg gaataaaact ttttctcatt
49021 gcaatttgca tgtatgccca gagtttttgt cctgaacgaa gacgaattat gtggagatcc
49081 ttttcatctt taacggaatt ctccattgct agagcaatac caacgtttct tttaactaaa
49141 tcgatcatta gtttcattgt tcgagccgtg tataaaatgt ttggagtaaa atcaccgagc
49201 tttgcctctt cttccaaaca ttctctggtt aagtaacctt tttgtctggc cacaaacttg
49261 cggtttctta attcctgggc atcaatgact tttttatttt tccaaggatc atctttacca
49321 gtgatgataa ctaactctgt tctatctaaa ttacggataa aataggcagg atcgttgatt
49381 ggcaccatcc aagaatagat agcaacgtct acatcgccat tatttaaatc tttaaatgca
49441 gtcgcacttc tttctaagcg agcatctaac aggttaacaa tatcttcttt ataaaatttt
49501 tctataattt caggaatata aatactacca gcaatcccag aaaaagctac tctaattttt
49561 ttaatattgg cgtgtttaac gtcgtaatcg atacttgcca tttcttttaa aaggtatttg
49621 gctttctgat aaagtaattc acctgcgggt gagagtgtaa ttttactttt gcgattcttt
49681 tgaaaaatca agggatcatc aaatctattt tgaagtcgct tgatcgccat cgaaattgtc
49741 ggttgtgtaa cgttgaatga attagcagtc acggtatagt tctgcgtttc aactaatttg
49801 caaaaatata atagatcctt atcgttcata ttattatctc ttttataaat atttttata

49861 aactaatctt ttttaattat aagttacatt tttcacaatt gcaaggaagc catgatatgt
49921 gcggctttat agagttgagt agttcaattt tattcacaat tacccactag aaaaagttta
49981 taaagagata taaattgcta tgtgtaaagg atgaaacaag tttgtgagga gaagagcata
50041 tggtgtataa gacgaaagta tcttgggatg ccacatatga tgtaattgta attggttttg
50101 gtggcgccgg agcaggtgca gcgcgttttg ctgctgataa taatgcaaaa gttcttttga
50161 ttgatgctgc tcctgaagga agcgaaggtg gtaatactag atatgctggc ggtgcttttg
50221 cttggggatc aaatttcgat caactgaaag aatactataa gcagacctat tatccatttg
50281 agtatgatga agaagcttta gataccttta tcaaaaatgt aatgcaaatg aaggagtatt
50341 caaaaaagta ttttggaatt gaagctcaac caacgggtag aagaccaaat ggagaatatc
50401 cagaatataa agatgcagct tcgatgcagt ctcaaagtat gaccaaggga atgtacaatg
50461 gcggattttg gaaattgctt cgtaagcaag tttttgatcg attagacaaa attgatattt
50521 ggtttgaatc gccagcagag cacttaattc aagatccaga cactaatgca gtaattggtg
50581 ctgttattaa gcgaaataat agagaagtaa atgttgctgc taaaagaggg gttgtcttaa
50641 gtctgggcgg ttttgaaaat aatcaaagta tggttcaaga ctttttagga caaggttctc
50701 ttgcgccaat tggtactttg tataaccgtg gtaagggaat tgatcttgct gttgaagccg
50761 gtgcgcgatt atggcatatg tctaactacg attcacatgg cttatcgctt agacaaaatg
50821 aaccacgaga aaaatttgct tacatgatta attggaagag cttatttaat gggtcaattt
50881 ttgtagcagg cgatgatggt actcgttact ttagagaaga tgaagaagat cgtcatggct
50941 ataagtacaa tcatggaaat tgggttatga ttcctaatca aaatcatcca catattatta
51001 tggatcaagc tcaatatgag cagctttcta acgatcagtc agctaaggca gatcaaatta
51061 aagaattgat ttcatatgca attaaagctg attcaattga aaaattatct aaaaaaattg
51121 atgcgccata tttaaagcag gcagttgaag actttaattt cttaactgat gtgaaaaaac
51181 gagatatgtt tttgaacaga aaaattgaaa ctatgagatc tttaaaagaa ggtccatact
51241 atgcaattcc tgttcgtcat aatattttgc atacacatgg tggtgcacag agaaattcta
51301 aatgtgaagt aattggactt gatggaaagg caattcctaa tttatatgaa gccggagaat
51361 taggagacat ttttgccact aagtatttag ggccaattc aattgctgat ttattaattt
51421 caggaaagat tgctggaact aatgcagcaa aaataaagaa taacgtagat cttccagatg

51481 ctgttactag cgcaagtcaa gttcatgaag atttaaagag tgatgcacat caagttaaga
51541 tgcattttga agctggtaaa aatcaaggaa ttgggtcaag cgatcaaggg attaatgatc
51601 ttccaattgt tgttcgtgta actttagatg aaaatcaaaa cttaaagaat atcgaaacgc
51661 ttcaagaaaa agaatctggc gatttgggtg gtcaggcaat accaaaattg acccaagaaa
51721 tgctcgacca taattctgct gacgtggact cagtttctgg tgcaagtaca actagtagag
51781 catttaaaga tgctgttaaa caagcaatag agaacgctcg taaaagcaaa tagctttggt
51841 taggggagaa gatatacaaa tgttagcaaa agtaaattgg aagggattta tacttccagt
51901 aattattgga tgtgtattgt ggatcattac accttttaga ccggctgcaa taacaccaat
51961 tgcatggcat ttatttgcct tatttattgc tacaatcgtg gcatgtatta ctaaaccatt
52021 accgatggtt ggttctactt tggttgctgt tacaattggt acgctacttc atatttttaa
52081 tattaaagaa gtatctgcat cttttgggaa tacgactgtt tggttaattg caatgtgctt
52141 gttcatgtct gctggattta ttagatcagg attgggtaaa cgtgttgctt atctatttat
52201 taggtctttt ggtaaaagaa caattggctt agcttattca ctttcaacgg ttgaaacctt
52261 aatggcgatt gccattccaa gcaatattgc acgtgttaat ggtattatgt atccaattat
52321 tgacgcctta tctagacaaa tggggtcaga tccaaaagaa ggaacacaaa gaaagttagg
52381 atcgtattta atttttaacg aatacgaggt aaatattgta acttcaacca tgtttttaac
52441 tggattagct ggaaatatgg ttgccttggg tattgcaaaa acgcaaggaa ttactgtctc
52501 atggatgcaa tggtttttag cagcaattgt tccaggtctt atttctctga ttttagtacc
52561 atttatttta tataaaattt atccaccaga agttaaagaa acacctaatg ctaatgcatg
52621 ggcaagtaag aagcttgaag aaatggggaa aatgacgcct gctgaaaaga tcatggcttg
52681 cgtatttgtt ttggcactac ttttatggtt attaggatca agtatcggaa ttgatgcaac
52741 tttagttggt tttatcgcag tttcaatttt actgatttct ggtgtcatta ctgttaaaga
52801 tatgatggat caaaagggtg cttggagctt gattgtatgg ctatcaattg tgatgcttat
52861 gtcacagaaa ttaatgaaat taggcttttt cccttggttc tcaaaggaat taggtctaat
52921 gcttcatggc gttaactggg tttgggtctt agtaattcta ttcttggttt acttctatat
52981 tcactatatg ttcccatcta ttactacgca aagttcagcc gttttagcag gttttctatc
53041 aattgcactt ggagctggtg ttccaaagct aatggcagga ttaatgctag gctttgcagg

53101 gtcgatcttt tgttcaacga ctccttattc agctggacca gcagcgcttc tttcaaccac
53161 aggatatgta aaatcatcag attggtggaa attgtccgca attttcggtg tctttttgaa
53221 tattgtatgg cttggcggcg gtctgctttg gactaaaata attggttttt ggtaaaaaat
53281 aaaatatcag tggtacacgt tatcgctgat atttttttgt tttattttgt aatcccaaat
53341 atttcaccta atcaattgaa tcatctatgc ttaaagtgag agtgtgtttt tcatagaaaa
53401 gagatagcta tgattcaatt agaagaattg gttaagaaaa atttacccaa agaaaaagaa
53461 ggaaaagttg ctagttatat tcctgcttta gctgaagtta atcctaaaca attaggcgta
53521 gctttgtatg acttagatga tggtgaaatt ggtgaagcgg ggcaaagtga agtaagattt
53581 gcgattgaga gtatttctaa ggtaattact ttgatgcttg ccattaaaaa gatgggaata
53641 caagaagtgt ttaaacatgt tggatctcgt caaagtggat ttgcttttga ttcgatttta
53701 aatatggaaa ttgaaaaagc aaagtatccg ttaaatcctt ttatcaatgc tggtgcgatt
53761 gctacaactt cgatggttgt aagcaaaact ggtgaagacg cgtttgaaga aattctttct
53821 tttgctcgcg atatttgcaa tgatcctaat ctttacttgg atcaaataat ttatcattca
53881 gaaagaagaa ctggcaatct tgatcgatcg cttgcgtact atatggaatc taaaaatatg
53941 atgcttgggg atgtgataac aagccttgat acgtatttca agcagtgctc aatgatggtt
54001 acagctcatt ctttggctaa tttaggtgcc gttttggcaa atggtggtgt gaagccatgg
54061 gataataagc ggattatttc tgatgaagag gctagatgtg ttaaatcgct gatgatgaca
54121 acgggtcttt ataatcaatc aggaacctat tcacgtttga ttggcgtgcc aactaagagt
54181 ggggttggtg gtggtttaat gtcagctgct cctcataagt atggcattgg tatctttagt
54241 ccggcccttg ataaggatgg taatagtgtt gccggccttg atttattaag agatattgtc
54301 gaaggattag aattggatat ttttgattag ttaaaaattg gatagaaaaa agctaagcta
54361 ctcgctattt tttgcgggat gcttagcttt tttatatata aattaatctc caacaattaa
54421 tttaactgga agcttgaagg tttcgtttct tttcggcgta tatccattaa tttgtttaag
54481 gagcaattca attagtaaac tactaatttc ttctattggt tgttgaatgg tcgataactg
54541 aggaaaataa tttcgtatca aattcgtacc atcataacct attaaatcta agtcttgtgg
54601 aattttaatt cctacttctt gagctgcttc ataagcaagg atagctgtta aatcatcagt
54661 acaaaaaagg ccatcaatag gttcattttg aagtattttc tttattagtt ctttttttat

54721 agttggtgac attgtaaaag aaaatgcatg ttcatgtgga attaaattat atttttttaa
54781 tgttgtaaaa aagccctcaa cacgggcatc ggtcggatta tgagatctga catggctgct
54841 tataatatgg atatgttttg cgccttgatt tattaaatat ttagttgctt taattccacc
54901 tttaaaattg tcagaactca ctactggaat atatggtgcc agatttctat caaaggagac
54961 aattggcgcc tgtatatttt tatattctag gatatctagg ttatgggttc ccgaaataat
55021 tccatctact ttattggcag ctagcatatc tagatatctt ttttctttat caggactatt
55081 cgcggagtta catagaaaaa ctctgtatcc tttttcaaat agctttgatt ctaattttga
55141 agttaattcg ccaaagaatg ggtgagaagt atcataaaat attaagccaa taatatgagt
55201 tgcttttcct ccaagtgatc gagcaatgtt gttaggctga tagtttaatt ctttgatggc
55261 attatttact tttattttgg ttgcatttga tatatatcct ctattattaa ggactctaga
55321 aactgttgtt ggagatacac cagctttttt tgcaacatca gttaatttaa tcataatggg
55381 ttcctctcat aattaaatat tgaaccaatt tttactcaaa agcaagcgct tactataatg
55441 aatttatgct atccgcatgc cagtatactg ttttcttagg ttgatagacg taactaattt
55501 aggaatttat aagcggcttt catgtgaaag cggttgacat attatgaaac aagactatga
55561 ttgtagttgt agaaagcgtt tacagtatta atgcttagga ggaaaattat ggatcataaa
55621 caagtagcca aacgtgtttt aaaatacgta ggccaagata atattcaagc tgctgcgcat
55681 tgtgctacgc gtttaagatt agttttaaag gatgataaaa agatagatca gagtggatta
55741 gataatgatc cagatgtaaa gggaaccttt gaaactgatg gtcaatatca aatcattatt
55801 ggtcctggag atgttaataa ggtctatgat gaaatggtta agatagccaa tctgaaaaca
55861 gcgactactg atgacttaaa gaaggttgct gcaaataaga atcataatgt gatattagac
55921 ttatttaaat tattatctga catttttatt ccaattattc cagctttagt tgccggtggt
55981 ttattaatgg ctttgaacaa tgttttgaca gcacaacatt tatttgctgc aaaatcatta
56041 gttcaaatga atcctcaatt gaatgacttt gctaacttta ttaatatgct agcatcagca
56101 ccgttcactt ttttaccagt gttaatttgt atgtcagcaa ctaaacgctt tggtggctca
56161 agaattttag gtgctactat gggctttgct atggtaatgc cacaattgat tagtggttat
56221 gctatttcta ctactccgca tattcctacc tggaacttct ttggtttgca tgtaatgcag
56281 gcaggttatc aaggtcaagt tttacctgtc ttaggtgtgg catttatttt ggcgagtgtt

56341 gaaaagtttt gtcataagca tttgaagtca gcaattgatt ttacttttac gccaatgatt
56401 gcaattattg taactggatt tttaactttt gcaatagttg gtcccgtttt aagaacttgt
56461 agcgactgga tcactaatgg aattatttgg ttctacaaca cgactggttg gtttggaatg
56521 ggactatttg gtttattcta ttcagccatt gttattactg gtttacacca aactttccca
56581 gcaattgaaa ctcaactttt agctaatatt tcaaaaactg gtggttcatt cttcctacct
56641 gtagcagcta tggctaatat tggtcaaggt gccgcaactg ctgctgtatg gttgacttca
56701 aaaaataaaa aacaaaaatc attagcaggc tcatcagcat tttctgcaat gcttggtatt
56761 acagaaccag ctatctttgg tgttaactta aaaatgaagt atccatttat ttgtggagcc
56821 attgcttctg gtattagtag tgcaatatta ggtttattcc atgttctttc catctcaatg
56881 ggaccttgtt cattaattag ttttatttgt attaaaccag gctttattcc tcagtttatg
56941 ttagggatga ttattgactt aatcgtggga ttctcaataa catacattta tgccaagaga
57001 tataatgcaa aacatcctgt tgaagacgat acagttgaag aaattgatgt taatgatgaa
57061 gtaattactg cacctatcag tggtcaagtt gaatctttaa gagatactaa agacaaagtc
57121 ttttcatcag aaatgatggg aaaaggagct gctattattc ctgaatctga aaacgtggtt
57181 gccccagcaa gtggtgaaat aactgtagct tatcctactg gacatgcata tggaataaaa
57241 actgatgagg gagctaatat tttaattcat ttaggaattg atacagtaaa tttgaaaggt
57301 aaaggatttt ctagcaaggt taaacaagga gatagagtaa ataaaggtga tcttttagga
57361 acgtatgatt ttaagttagt tgaaaaagaa ggttatgatc caacagtaat ggtagttgtt
57421 acaaatactg cagattatgc tgaagttaag agagttactg gtaaagaagt aaaggaaaat
57481 gataatttaa ttgctttaac tgaaccttct aaaggaaaaa catcagttgc aacagctgat
57541 ccagtttagg agagaacaat gaaatggtgg caaaattcag taatttatga aatttatcct
57601 aaaagttttt atgatacaaa cggtgatggt atcggagact taaaaggtgt ggagaaaaaa
57661 ttaccatatc ttaaagaact gggaatagac gcaatttggc taacacctat ttatttatct
57721 ccacaagttg ataatgggta tgatgtagct gactatcgca aagttgatcc aatgtttgga
57781 agtaatgatg atttgaagga acttattcga aaatcacatg atttaaatat taagatcatt
57841 cttgatatgg ttgctaatca tacttcagat caatgtgctt ggtttaaaga aagtaagaag
57901 tcaagaaata attattttag tgatttctat atttggaaag atccaaagcc agatggaagt

57961 gaacccaata attgggcagc tagttttgga ggttctgctt ggacttatgt accagaaaga
58021 aaacaatatt actttcacta ttacgcctct caacaaccag atttaaattg ggaaaatcca
58081 attgtacgtc aatatatata tgatgcaatg cgttattgga aggctcaagg agttgatggt
58141 tggcgcttgg atgtaattac acaaatttct aaagacttaa cctttaaaga tgtaccaaga
58201 gttgttgata gaaagtatgt tccattaaaa aataccagcg gaccgcatat gcatcaatat
58261 attcacgaat taaatcaaga agtattaaag ccatttgaaa tgatgagcgt aggcgaggca
58321 ccgaatagta gtatcaaaga aattctagat ttaacaagtc ctaaaaggca agagcttgat
58381 atggcattta gttttgaaca tatgcgaata gataaagctg ctaaagaaaa tggtgctctt
58441 gcccttaaac aaccagattt cttacaatta aaaaaagttt tatctaaaat gcaatatgag
58501 cttaataata aagcttggaa tgctttatat tttgaaaatc atgatcgttc aagaatacct
58561 tcgcgttggg gaaatgatac aaagtatcgt tatgagagcg caacggcatt tgcgactata
58621 ttacatggat taaaaggcac gccatttatt tttcagggtg aagaaattgg aatgaccaat
58681 cctaagtatt cagtggaaga atatgaagat attgatctta aaaataaata ccaggaacaa
58741 gttaaaacaa agcatttatt aaatgctaat gaatttttag caatagaaca taaaatagca
58801 cgtgataatt cccgtactcc tatgcaatgg aatggtgaga aaaatgcagg ttttacaaca
58861 ggaatacctt ggtataagat aaatccaaat tattttagga taaatgttga atcagataaa
58921 gctagtgaaa agtcgatttt taagtattat caacgtctca taaagttacg tcataatgaa
58981 gaaatattaa caaaaggtaa ttatgaagaa ttacttaggg atgataaaaa tatttttgcg
59041 tataaacgaa acttgaatag aaaaagttgg ctaattatag tcaatatgac ggataatatt
59101 ttgtcattaa aatcattaga agctgatttg aaaaggtatt caaaagtaat tttgcataat
59161 tataaaggca aaattacaaa caatgaattt aaaccatatg aggcatgtat cttagaaaaa
59221 aggttgtaaa ctaaattaag agtatgcagg atccgtaatg atcctgcttt tttggagaaa
59281 aatgttaaag atagataata ataaaaatca aatattattt catttgtata acactcaaat
59341 ttcttatgtg atagaaataa ttgatggaaa atttgtgaca aatagatatt ttggccctaa
59401 gctaccatat ttttctggca gtacttcgtt agataatgga aagcatgctt tttccattta
59461 tcagcaggaa gatgcttttt cagtttcgag tctgccatta gaatatagcg tttcacaatc
59521 aggtgactat cgtgaaacta gtgctgaact caatgataag ttcaatctgc caataattga

59581 tatgaaatat gtgcgatatg atcaagaaaa ggttgaactt tcaagtttac cccacataag
59641 gcaagccaaa gggagtagct taaatataca tttgataggt aataatggta ggttggctat
59701 tacaatacat tattttttac tgaatgattt tccaagtatt atacgttgga tatcgtataa
59761 gaatataggc aaatctctga tacatataaa aaaagctgat agtttacaac tagatgtgcc
59821 taatagagaa tggaaaacgt taagtttttta tggaacgcat gcaagtgaat tcataccatc
59881 aataaagcca atttttgatg gaaaggttga aattaatagt acaagaggta gtagtagtcc
59941 gcaacatcag ccttttattg cattgatcga caagaatttt acattagata gaggtaaatt
60001 tattagttgt aatcttattt ggagtggaaa ttttagaatt agtattgaga aagaacaaga
60061 aaactatgta agattaaacg ctggtgttaa caataaatta tttaattata ctttgagacc
60121 ttctgaacag tttattactc cgcaagcaat tataacaact gggcaaaatg gattaggaga
60181 aatgagtaga ttatctcaag aaataataag taattacatt gtgaattcga gaatccaaca
60241 accattaata gctcttaata cttgggaaat gtcatatttt gatgttacag aaaagaagtg
60301 cataaaagct ttaaatcaag ccgaaaagat tggagctaat ttgttagtaa ttgatgatgg
60361 ttggtttaat aatagaaact cagaaaaagg acaattgggg gactggatac cagattataa
60421 taaattccca aatggtttaa aacatattgc tgagctaacc cataaaaaag aaatgaaatt
60481 tggaatttgg attgagcccg aaatggttac tacatcaagt gatttattta agaagcatcc
60541 agattgggtt ttaggcttag attctgttga gagagcttta tatagtagaa atcagctcgt
60601 tttagattta agtaagaaag aagtgcagaa ttacttaatt aatataataa gcaacttaat
60661 tattgagaac agaattgatt acttaaaatg ggatttcaat cgtcaactag cacctgtttt
60721 tagccaagga agaagtgcat tagatcaagg aaaagttggc tttgattata ttttaggctt
60781 gtatcgtata ttaagttctc ttagagaaaa atttaagaac ttaattatag aaaattgtgc
60841 agctggtggc ggaagaatgg acctagggat ggtatacttc actgatcaaa cttggattag
60901 tgatttaact gacgctttag gtcgttttag aataattact aatatggcta caatttatcc
60961 gattagaatt ttttcatccc atttttcaaa atcccctaac gaacaagatg gaagaatatt
61021 accgatagaa actagattag ttctttcttc attaggatcc ttgggttttg aattaaattt
61081 agaattaatg ggtgaagatc aaattgataa aattaagtct tacgtaaaaa aatataaaga
61141 agaatatagc ttaatgcatg attctgtatg tttaccgtta acgccattaa gagaaaaagc

61201 tacgatgcca atggccatta tgttaaaaga tgccaaaaag attggggtta tatattcgta
61261 cggtgcaaca gatgctgttc atatccctcg atggctacca cttatttttt tagataataa
61321 gtgtgactat aaggtaaatg atgactattc ttgttcaggt gttgaattaa ataatgcagg
61381 attaactatt ttcccagtta caggtgattt ttgtgttgat gttgaatatt taaaaaaaga
61441 ataggaatct gactaataaa ttttctagaa atagaagatt attagtttaa aagtgattta
61501 tagaaagaat atattgatct aaaaaagcta agtattatct taggtgctct tggtacatgt
61561 gccatacgta tcgcaaaagc ttacgcaata ataaaaaaag ctaatcacaa ggattagcta
61621 aactcaaaag tcatcttaag ttacgattgg agttagtgac gggtactcac tagctccttt
61681 tcttttacaa ttatacacta aaaagttcat ttttggtatg aaagtgtatg taaagctcat
61741 ttttgatcct atttaagtgg taaaataata aaaaacatta atggaaatag gagtaaaaat
61801 gaaaaaatat tcaaaagcaa tatattgggg attaccaatc agtttaatcc ttgctatttc
61861 aattattttg catttaggtt ggattaatgc ctttgataat ttctttgaag gattagttca
61921 tattgtgcct aatttaaagg gattgatgct aaagattacg tttttagctg ataccaaggt
61981 tgatttagtt tggatggttt taattgccgt aattctatgg tttagaaaac aacgtcctct
62041 tgcgactaac ttggttttaa caatgattac tggggatgca gtaggttggg taattaaaca
62101 cttaattcaa agagcacgtc cagtacaaca cttagcagtt gatgacggtt ttagttttcc
62161 aagtggccat accttaggaa tgggaatgat tgtaatttgg attataatgg ttcttttacc
62221 agttgtttta aaggatagaa ctaaacgttt ttggattgat ttcttttaa ttgtttggtt
62281 agttatcgta atgatttcta gagtatatgt atatgctcac tatccatcag atgtttgtgg
62341 ctccgttgca attgccttaa cttgggttgc tgttatgcaa tttgtctttg agaaaattaa
62401 gcaaacggta aataaataat taacaaaata gggttaagcg taatgcttaa ccctatttgt
62461 gtgagtagtt ttttgatttt gtttctgatt aagttaatag aaatttattt agtcaaataa
62521 tgtccatgct tggtttggcc ggtcgtatag atagcccttt tgccaattcc attctggatg
62581 ttgtttcata aaatctttgg cgtcaaatgg ttcgccatcg gctctaccga aaatgcccca
62641 catattcaag ttttcaatat cggtagcatc tgcgcattta ccgtcgcaag agccaccagg
62701 gaagaaggga acattccaga cgccgtgcgg atcgtcagca taatattgtg gatcaacatc
62761 gtagtgagca catatattac gtgaagaagc acattgataa ccaagatatg gatcatattt

62821 atcagctaaa atctttttag ctactgaatc gtcaattgtg cctgatactt
ctggcaataa
62881 ttgctcaaag cgagcccggc gagcaccagt ttgctgacga gtatcattat
agccgttgtc
62941 gacacactca agattgcgaa ttcgaggatt atgacaagca ctgcagccat
agtagaaacc
63001 atcagttgat tctagtaatt cttgattctt taatccttgt tcataacggg
caatttcaca
63061 ggtattaata ttgcaaagaa gccaaatatt agcgtagcca ccgttattcc
cttcattgac
63121 tagttttacc cattggtcaa tattattagc atattgagta gctttacgag
cccggataaa
63181 ttctggcatc ccgtttacat cataaccaac aaatccagca atagtggttt
cagtaatgcc
63241 aagacctgca tctgttacgt aaaagtcggt cattgaatcg atgtagccag
gaacggtttg
63301 catcttaaag gcatgaccat tagttgggtg aacttcttcg caaacgttga
agtattgtcc
63361 actccaaaaa tcgtcaaaac tttcgtgagc aatgacaggt tttccatccc
gcgtagcaga
63421 tccagttgca acaaaaccag agcagtgtga tccgcgtgga ccttgaggtg
gattgtttga
63481 gtagttagct gcaacttgtg gccaccaata gccggttaac tccatccaat
cgttccagct
63541 ccagttttag cattattggt agcatgttac ttggtattta actttacttg
ggaaaccatc
63601 ttactttcaa tcattgtagc agctgtaact gccattcttt atctattctt
gggtaaaaag
63661 agattggcag agattggcgg tattcctact gcaattaggg agcagaagaa
tcatcaagac
63721 gctaagaaac aaaattaatt tttttgaagt atatttcaat ttttagcaat
ttatgtgctg
63781 aaatgaagta tacttcattt ttttatgaat ttaaggcaaa tttgaagtat
aattcaagaa
63841 aataatttag aaacaaaaat acgccatttc tatctaattt aaattaggta
agaaatggcg
63901 ttttttgtta gttattagaa tttaagaaaa agcgactata taagcattat
aatttctcaa
63961 ataaaggtat tttttcctga gattttacat cgaacaatcc tttatcagaa
atttttattt
64021 gaggagaaac caatagagac agtgttgaaa atgacataat ttcgtttgta
tttttatatc
64081 ctaagtcttg cagagtatgt ctaatgttac taattttttc tcctaatttt
gtcattggtt
64141 catcactaac aactccgccg ataggtaatg gagcatcttc tatgatttcg
tctcctttag
64201 caaccacata accaccttgt tcatctatta attgatgttg tatttttagc
atagccttaa
64261 tgtttgttcc cattatcatt atattgtggt gatcatgagc ccatgtggct
ccaacagcac
64321 cgtctttaga aattccgcga tctactagtc ctaaagtgta gttaccattg
tttttgccat
64381 aacggtcttg caccataata agatttaagt ttgctttttt ccattgcact
tcatgatttt

64441 taacttctag ttttacttta atatgattgg taaaagttcc aacaggatta
atttggatta
64501 cattggcaac tacttgacca ttgttaatca gatcagtttt taatttgaaa
tcattgattt
64561 ctagtttagg agctttaact gatgatctta gagaaggcaa gaattgataa
tcagatttct
64621 taaatttgtt ttctatagga aggccatttt tgtatacggt attaatagat
aatgttctag
64681 gattgtctag aattataaaa tcagctattc ttcctggtgc aatacttccg
cgatcccata
64741 agcccatatg tcttgcagga gtatatgttg aaatataaat tgcttgctct
ggggtcattc
64801 ccatatcaat tgctttcttt actaaagcat ttaagtggct atgaagaaga
gtatcaggca
64861 ttgagtcatc tgttacaaga gcaacatgtt cgaaaaagtc atttttaatg
atagtgtcaa
64921 ttacatcttg atgcatagac tttagttgga cttctacaaa cattccattg
cgaacttttt
64981 cttcaactag ttcggcagtt tgttgagtat gatcagttgt aatgccagca
taaatgacct
65041 ttgttaagtc ttctccagtg taagctggaa cgtgaccttc aagtggcatt
gtagggtgga
65101 tgtttctaca cgtttttatt attttcttga ttaaagtatt atcaggggaa
gtcatacctt
65161 taaagttcat tacttcaccc aaacatatga agcggggatc ctttaatagc
tctttaactt
65221 catcgggccc aatttgacca ccagtagttt ccatatgtga attagttgaa
ggaactgatg
65281 atggtattcc gaaatagatg tctattggag aaggctgatt aataaaattt
ttaagacctt
65341 ttataccagc gacgttagta atttcatgat catctgcaat aattgtcgtt
gtaccatatt
65401 ttccaattgt ttgtcccatg atttctggtg ttgtcattga actatcaata
tgcatatgag
65461 catcaacaaa gccaggaata agatatttac ctttcagatc tataactttt
ttagctgtaa
65521 cgttagctag attatcgcta atccaataaa atttatcatc ttttatagtt
acatcgctgt
65581 gaatgaactt tttcaagaag acattgaaaa cttgagcgtt tttaattaat
aaatctacct
65641 gtgtcatgtg ttgctcctta taaataaaag agttggtaat tccaactctt
ttataatgtt
65701 tgcgttaaaa actaattatt cattatcttg ttccattggt ttatccattt
ggaaatatgg
65761 ctgttaacat agaagaaatc aatggtttta gcacgtttag ctacatcacc
gtaagtttta
65821 ttagctgcct ctttttgct taaattaact tgttgattaa caggagcgtt
attaagtgat
65881 ttagtattag caactttctt ttgcactgat tcgcttaaac ggtaattaat
atattgataa
65941 gctgcattcg tatttttact atttttcaaa atagaaacat tatcatagtt
agcataagtg
66001 ccagaagctg gtacgaaata ttttaaatca ggattagttg cttgaagcat
tccgacagca

66061 tagtcaccta caactgcagc actaatttct ccagtcttaa acatattact
taaatcagaa
66121 ctttgagtat aagtttttac gatgttaggt tttaattcct tcattgcttt
aaatgcggca
66181 gtaccatcat ctttagtaac ttgggtacca gcatgatcac cggcaatata
tagcatagca
66241 gggccaaatg tagtagtcat atctggaatg gatatttttt gcttgaactt
atcagaccaa
66301 agatcgttcc aatctttaat gtttactttt ttaggattat aaataattcc
tacactattt
66361 actgtatagg gaacagtatt ggttttctta gctaattgct gttgatcttt
agatagatac
66421 ttgaaatttt tgatttttga aaagtctagt tttttaaata atttcttttt
tgctcctgta
66481 acagcattat tttgagcgag ttcaataaca tcgacattag agttgggatt
atgttctatt
66541 tgagttagtc gagttgaact gtctccaaat tgagttgaga ctttgacgtt
atattttttt
66601 gagaagggat ttaaaacatc tgagcgcatt tgcttagtag ataagccaaa
ggttgaaact
66661 accaatttct cacttgattt tgatgaacaa gcagaagtac cgattgcaac
accagcagta
66721 attaagataa gggaccaaag ataaaacttt ttgaatttca tgatattcct
ttctgaaaat
66781 aaaattcaat tattctgata gaggatgaag atgtttagct gggagagtga
gagttactgt
66841 atctcctgaa ctgcgcatct ttgctctagc ttcttcaact tgaaaaattc
ctaaatcact
66901 tgtaattgtg tatttaaaac tttgtcctag atattcacga gatgtaatag
ttcctaagaa
66961 agagttttct gaatgattat cttgatcaag aagaatattt tctggtcgaa
tggttaaaat
67021 tttggcattg ctgtttgatt cttttgtggt tagcaaagtg tttttaacta
gaatatgatg
67081 gtcgtcgttt ctttcattag aatttataga aatgaagtta tcgtaaccta
taaaacgagc
67141 tacatattct gtacgagggt gatgaaaaat attttgcgga gtatcgtatt
gctcgatgtg
67201 accatctttc ataattaaaa ctttgtcaga aattgcgaaa cattcttgct
gatcatgtgt
67261 aacgaaaaga acagtcattt ttaacttttg ctgtaaatta cgaatttctt
ctctcatttt
67321 taaacgtaat tttgcatcta agttgcttag aggttcatcc atcaataata
attgtggatt
67381 tgtaactaaa gcacgtgcta aagaaactct ttgttgctgc cctcctgaaa
gttgagcagg
67441 gtatttttcc tttaattgat agagtcctgt tgtgttaagg attgaagtaa
ctttatcttt
67501 tatttcatct ttagagactt tatgcatttt taaaccaaaa gcaacattat
caaagacatt
67561 tagatgagga aacagagcgt agctttgaaa aaccatccca aagtttcgtt
tatagactgg
67621 aatatttgta atttgtttac cattgagtaa aatttgtccc ttactttctg
gaattaagcc

67681 agcaataatt cgtaatgttg tagttttacc acatccagat ggtcctaata ttgaaactaa
67741 atcaccgtct tttatattga aagaaatatt ttttaaaatg ttgtttttttc catcgtatga
67801 cttaactaaa tttttaattt ctaaatttgc caaaataata cctctattta gtaagactat
67861 taatcccaat tgctttttcg actaagacca tcattaaagc tgtaaagatc attaaaagaa
67921 cggagatagc tgaaactgtt ggatcataat tgttttgcag ataatttaaa attgctggcg
67981 gaagcattgt tagagaagga ccattcataa acaatgaaat tggaatgttg ttgaatgagt
68041 taataaaaca cagcataaaa gccgcaataa tactggattt aatattaggt aaaactatta
68101 agaaaaatcc ttttcttggt gagcatccat atatccagcc cgcttcttct atgtgtttat
68161 ccattaatag catgctagct gtaactaggc gaattacgta aggtaaacat agtaagaaat
68221 gacctataac tagacctata aagactggga ttcttagagt aacaactacg gcttgatata
68281 gggcaaaacc aataacaatt tctgggatta atgtagggct gaggaaaaat gattgaaacc
68341 aagcggcatg ttgaatatga aatcgagtta gtgcataaac agctggaata ccaattaaca
68401 aagctaagct actagctaaa agagctacta ttaaacttga ttcaaaacca gaaataaagt
68461 caggttgctg aaaaatattc gcataccaat taaaagtgaa accttttata ggtaaagaaa
68521 ttgaactctc agcattaaat gacgtaataa taactagaat taaaggaatc agtaataacg
68581 ctaatatgat aagtgaaata gcagttaaaa ctttattttt tcgatcaagc acggctaggt
68641 ctcctatcta aaaagtaatt tattttttttc ataatccaca tagatagtgc agaaataata
68701 attaaaatta gagcaattac gctagctttt tgccaattac ctaaagtcat tgcttgttgg
68761 taaattaacg tagacataac caaatgacga tttcctccca aaatttgagg agtagtataa
68821 gctgtcattg ttcctgcaaa aaccaaaatg cagcctgtga agattcctgt cgttaactga
68881 gggattatta ttttaaacaa agtttgtaat ggagtagagc caagtacagc agcagcttct
68941 tccacttcta gattgagttc agaaatatta ctagttaatg tgatgatcat aattggtaaa
69001 aatagataaa tggatccgat tatgattgct agattggtat aaagcagtga caagggatga
69061 ttaattaaat gcaaatttaa taaaaatttg tttactatgc catctcgtcc taggatgata
69121 atccaagcaa agtttctgac aactgcgtta gttaacattg gaaataagat taggatcatt
69181 aaaatttgtt taatggattt tttctgacga gcaatccata gagaaatggg taaccctaaa
69241 attaagacaa ggataactgt tacgatagct ataaaaaaatg ttcttccaat tatttgccaa

69301 ttataagtat tccttaggaa tgtagaatat agtccccaga aaccatatct agtttgaaat
69361 gtaggaataa tgatttgaat aatgggaaaa agtgcgaata ataaaactcc cataaaagct
69421 ggaagaagta accaaatgac tgattttcga ttcattttta acctccgatt tgtctattag
69481 cttaaatgat tgtaagtcta aagtcaatga taaatacgaa tattattaaa catatgcttg
69541 tctaaagatt gtatttatgc ataaataaag ctgataaaat ttttgaataa tgaatcaaat
69601 aaataaaaac gcgaacatta aaaaggaata atgtttttgt aagatatagc attactataa
69661 ataattggat aaaaaaaaac tgatataccg gcgttgtgat tataacgtct gcatatcagt
69721 gctgattttt tattttagat tttaattttg aaaattatac ataaaaatat aaacgcatta
69781 gtttttatgc gaatcataca tagcaataat tttttctaat ttttcattgg gaatcagttt
69841 caaatttcta acaaattctg tagcttgtgt caaagtacta ccattattca gttgattttc
69901 gagaataatt ttggcagaat ctatttttcc ttgctctaat ccctgtttaa taccgtgctg
69961 cattccttgc tcatagcctg cttttcctgc agcttgttct cgttctagca tccttgtttc
70021 gtaaagcatg atttttttccc tcatttctgg atcttcattg atttccttga ttcttctttg
70081 cgcataatcg aaatgtttat ttaatttaac tggctcatta ttcatcagtt tagctagagc
70141 ttttaattct tcgctttgac cttcagctga aacttgtgaa ttaatgataa tctttgtcac
70201 gccatcttgc aattgctttg atctatcttg atcagaataa gtatggtgcg actcataaaa
70261 tttatcatct ttcttgggtt tgaaattaca gagaaaaata atgaaggcgt ctttcaaatt
70321 tcgataggtc tttcctttat ttaaagtata gcgtagatca agtcttgaaa gatagtagcg
70381 caggcgtcta ccaatatcat cctgatccgt tgtttgcatt tctatattaa acacgcgtcc
70441 ttcatggtct gtgactaaaa catcaagtct aacttcttta gcttcgttct tacgttcaga
70501 attattaatc tcaacttgct tatctaacca atcaatcttc ttgatcttta gatctggaat
70561 gatgatttca agcaaatatt tacaaaaatc cttgttttcc atcaccatcc caaacacttt
70621 atcttctgtg aacccaaacc atggtttaat ctttttaacc atatgagcag tcctctctaa
70681 agatatttga ggaagtaatc caatatgttt attacgcaaa gtggtaagat tttttataat
70741 taatttaaaa tttattctag ctaaaaaagc atatcccaat ataaaatcag aatatacttt
70801 ttcactctaa ttaataatgt actttaaaaa ataattatta gggcctttaa tcttcttcag
70861 ccttaaaacc attggctagg ttaggttttc ttctaccaat tagccaaaca atcagtagac

70921 taaatatacc ttcaacagca atgtagattc cacgtccaga taagtgcttt aatcctccta
70981 ggtaaattcc gaagataatt gaacacatag tgattgcaat cattgaaatc cattgaacct
71041 tatcaaaagc aataccgtat tcaacttctc ggtgtctgat tccaggcaga attgcaccta
71101 aaccaattaa gacgattaca gcaacaatat taataattaa ttcataccac caaatactgc
71161 ttccttctgg tacatcttgt ggcgtaattc ccataatcgt ggctatagca gtaacaagaa
71221 ggagaattaa tccaacagca taggcaattt tgtcgttttt aataaagaca tattttgaag
71281 ggaatttttt cgaatctttt ctaacccgca taaatgagta gaagatccag caggtaacac
71341 ctggtgaaac gattgcgttt aagttaagca accagttaaa aatatcgttc atgtcaggca
71401 aaaagatacc taagatcata atgaatgatg acaaagctac agttaatata tatccattaa
71461 ttggtaagcc attcttatcc tttttctaa ggaacttagg catatagcga ctaccagtat
71521 cggaaattaa cattctagtc attgcatcaa gtaaaactgc tagcaacgca cagttaaaga
71581 agaaagatgt ccaagcccag agataaagaa ggaaattgcc ccaaccatat tggtgaccaa
71641 tcatcttaaa ggcatagtaa gcaccgttca tttttaagtc gtttggcata tgattagcgt
71701 taaagtagat tcctagagca aatgagccta aaattgttaa aaatgcagtc attgctgcta
71761 aggcaatcat tgccttaggg aagtcatatt gtggcttttt cattttggtt acataaggag
71821 ccactagttc agcaccgttc attgcataga ttaacatggc aatcgttgag aaatagtgcc
71881 agtcaaattt aggaaaaata tctttccaag taaaattggt tgtcgccata tgaccgcctg
71941 attttgccaa gcctacaaag gcaagaacaa caaaaagaat ggtcataatt accattaacc
72001 caccaccaat tgttgacaag aattccattg aatttgaaaa atggtgttca ataataatga
72061 atacaataaa agttacaaaa gttaataggg caaataaaga attggggatt tgatcttgaa
72121 atttttctga tccagtaagg gcccaaccta agtcaacgat aacttcatta gctgaatcaa
72181 cagcataagg aattgaagca gcccaataag tccaagcagt gaaatatcct aagaattcac
72241 catcagttcc acgtacccat gaagaaagcc ctccaccctc tgtactaaag gtagagccta
72301 attggccaac cattaaagag taaggaatga catagaataa aaccattaca atccatgaga
72361 atatgactgc cattccttgg ttttgaaaat tgaacatgat gtcgtctaag ccgataacag
72421 tacagagcgc catcaaaaat aaagtctgcc aagaaataaa tttcttatct gggtttaatg
72481 catttaattc atccacgttt aaattttctc tcttttctag taataattat ttatgcgtta

72541 ttactaggat taactcggtg acttattttt aaatttaata ttcataaatc taaaatcgat
72601 ccttatctat tttttcttta tctagtatag attaaaattg tcgttaaaaa tagcacctct
72661 caagtttata agggatgcta tttttgttat ttggcaagat ttctaacgac attaagatta
72721 aacaaaatta aatttaagac tacgattgct gctgtgacaa taaataccat gctgtagctg
72781 gagattccgg cgataaagga accgagaaga ggtccgacaa tatttccgat atacattgca
72841 ctctgattcc atgagaaaat tctaccagtt accgctgcgg gagaattttt agtaagtaaa
72901 gtttgtactt gaggaaaaag acaagcatcg gtaaatccca ctaaaaatcg taaaatacca
72961 agttgagttg tattgtgaac aaaagcggtt aaaaagaaga gaattgtcgc tccgataaaa
73021 ccaccaataa taatcttgtg tgtaccaatt ctgtcgccta acgcgccaaa acgagaagca
73081 gctaagaaag tagcaattcc tggaagggcc gcgataatcc cagaaacaaa gacaacattg
73141 ccgtttccgt tcattaattg cttaacataa agtgaaacaa tcggattaat tgaattattg
73201 gcagcctgga taattaaggt tgttaagagc aaaccaaaga ttaaagcagg agagcgaaga
73261 gttgcaataa cgccgcttgc tttatcaagt ttcttttcag taatgggctt aaagccttct
73321 tcatgaacaa agaataatga aagaaggaaa gagattaaca ataaaccgcc cgtgataaag
73381 aaagtaacac ggtaactaaa ggcagaagct agtgcacctc ctaaaaatgg tcccagcaag
73441 ttgccggcag taaaagatga agccatggtt cctaaagctt gaccagattt ttcctttggc
73501 gtttctgtag caattagagc atttgaattt gatacaaaac ccgcaaaaac tccttgtagc
73561 attcttagga aaaatagttg ccagacgtta gtaactagtc ccatgcttcc aagaacaatt
73621 gccattccta gagaagctcg caagatcatt agttttctac ctttttgtc ggctaatttt
73681 ccccaccaag gcgagataat agcggaaaca aagtatactc ctgaaaaaat gaagccagac
73741 cagaagttta attgctggtg agtaaaatga ccaagagtat caatatacag aggcaaaaaa
73801 ggcatgattt ctgaaaaagc gattcccgca ataaaaacgg caattgaaag gacataaagg
73861 ttctttttcc aaataggttt atcagatttt tttgtcatcc tattcctcca actttttaaa
73921 gtttacggtc ttatttttca aggagatctt tcctacttca aatccataat ctttgagttc
73981 ttttttataa gtcagaaaag aatggtcgat tggcagacca gcaatttctt gaatttgatc
74041 aaaggataac tggaaatcac tatttttatt gtttttaata tattcccata agggattaaa
74101 cttactcatc attatgcctt ctttgaactt gtttttctaa attatttgat atttcttcta

74161 gtttagttaa agtgtcagat tttaccgtgc cgactaattg attaacttct
tcaaaagctt
74221 gattaacttg atcaagaaca tctttgccag ctggtaaaag aaataattgc
ttctttaaac
74281 cgttcttggg atcaggacgg cgttcaatca tctgcctttt ttctaataat
ttaactacat
74341 tggtaaagct agcaggttga taaaagagat attcagcaac ttctttttgg
atacaaccgg
74401 gatgcttttg aatataacgt aaggcatgag catgcgtcat tcttaagtta
attgaactca
74461 atctttgatt aattaaaaga ttctctagtt taccaatatg atatagaaga
ttggctagtg
74521 gtcgttttct catagaattc cttcttaaaa agttgtaatt acaactaatg
tcaaaaataa
74581 ttataacgta attaattgta attacaacta gtcattgatt aattacttgg
attagtgact
74641 tgtgagaaat catatttacg atattttaaa gcagcttttc gatccttaaa
aatagcactg
74701 tgaatatcac ctgatgaata tgataagtag aggcagcgat gccctttaaa
agtacctgta
74761 atataaataa tatcaacagt atctaagaaa ccacgtgccc gtaaccactg
agagccgttt
74821 ttgtcttcaa aggataccat tgttgcgttt ccagcatagg ttttattgtc
tttttcagaa
74881 cgatggctca aatttaagtt gggatctgtt ttttgataga ccacagaatc
catgattgta
74941 tgtttggtaa tgataaattt tcgagcctgg gtgctataga tatcagtttg
gtaccaagta
75001 ccttgaaagt tgtctggaat tgtagctagt ttaaagcctg gatatttttt
atttaagttt
75061 ggcttaggcg tggcggtttt actcttcttt gaagtagtga ttgaagattt
tgaactggaa
75121 gatttatttt cggtattatt agacgaagta ttgctgcagg cagtgagcaa
tatggttgaa
75181 aaaagtacta ggactagctt tttcatgttt tttacttcct taagatatga
atttttgcta
75241 taagtataac aaaagatagg aaaatctatg atattttttta accgcaaaac
aaaaagcaga
75301 catctggggg aatagaagtc tgcttttttta atgtcgtatg ttattctggg
agaataagta
75361 aggtgtagaa gttcccttac ttgtctaata gaatacctct aattttttaa
ataaagctag
75421 gcataagatc taaaatttgc aaagaatttc tttaaaaagt ttaaagaagt
aaatataatt
75481 aaaataaaga cgtttgcgag aggaaaaatc atgaaaaaag aacaagaaat
tgagatgcta
75541 aaagaattct ctgatgcaaa ttcaacctct ggatttgaag aagaatttgt
aaaattattt
75601 tcgacttatg ctaaaaatac agcagatatt acagttgatg gcatgcttaa
tgtgtatgct
75661 gcaaagaagg aaaataaggg agatcgtcct gtaattcaga tggatgcaca
ctcagatgca
75721 gtgggcttca tcactcaagc ggttagacca aatggattga tcaaattcgt
tccacttggt

75781 ggctgggtaa aatataatat tcctgcttta aaagtaaaga ttagaaatcg tgagggtgag
75841 tatattcccg gcgtagttgc tactaaaccg ccacatttta tgacggcagc tgagagaaat
75901 tcaattccag aagtagcaga catgtcaatt gatgttggct cattaagtag agaagaaaca
75961 attaaagatt acaaaattga tacgggttgc ccaatttttg tagatgtaaa atgcgaatat
76021 catgaaaaat caggtctttt ctttggaaaa gatttcgatg atcgatttgg tgctggtgca
76081 atggtcggtg ttttagataa tttaaaaggt gaagaaactg attttgatgt tgtggctgct
76141 ttatcaagtc aagaagaagt tggtcttcgc ggagcttatg taactggtag aaagattaag
76201 ccagacttat gtattgtgct tgagagttgt ccagcagacg atacttttga gccagactgg
76261 ctatctcaaa ctggattaaa acgtggtcca atgttgagag atatggatac gacattttta
76321 ccaaatccta aattccaaca atatgcttgt gatttagctg ataaaaatag aataccgtat
76381 actcgctcag taagaactgg tggaggacaa gatggagctg ccatttatta tgaatatggt
76441 gcaccaacaa ttgtaattgg aattccagta cgttatgaac attctccata ttgtttctct
76501 gcgtataagg actttaaggc atcagttgat ctagcaactg caattattcg cgatatgacg
76561 caagaaaaat tagatagttt taaaaaggtg atttaatgga ctggaaaagt ttctttagaa
76621 aaatgacaat tatcatttgg atgctatgtt tgattaattt gttggtgtta atagttcgct
76681 acactgcaag cgatattgga acgttttttt cgtgggatta tccgctaata tgggctttta
76741 tactgggatg tatttttgt attttggcga ttttctggcc caagaaaaat aaataaaagt
76801 atgaaaaaag tgaaggtaca ttgaagtgcc ttcacttttt gtgtgatctg attttattct
76861 acgcgtgcaa tttcatcatg aaccttaata ttgtgtgaac ctagacttgg ctcttgacca
76921 tgaagattaa atttgacgtt aacaccaggt ctaaatagaa gtacatgagt tgaaccacca
76981 aagtggaaaa tacctaattg atcaccttta ttaatatgtt gaccaacttt aactgttatt
77041 tcattaccag atacttctgc catacctaca gcaacaaagc acattaatcc aatctttgga
77101 ttatcagctt tgatgaaaat aacagcacgt gatgcagttt cagtaatata agcttgagaa
77161 tcattaggtg cagctgggtc aggaccgtta ggattttcaa aaccttgtgg taaagtttca
77221 gaatagtatg aaccatatag gttataagct ttaacaaccg taccactaac aggactattc
77281 catctatggt aacttagtgc actaagaaag gcttgataga gagtaccacc aacaaattct
77341 gatgttaatg agtcattgtg aagcaaatcg attaatgaat atggttgtcc cttaatccag

77401 aatttagctt taagtggaag gttatgtgca atacgatatg gtgctgattc acaagcatta
77461 gcaattgaat cagggtcatc agcattagca acaggacgaa cgccaggatt aaaagttctc
77521 gtgaagaaat gatcccaaga tttaaatccg tattgctgat caatatcatt attaggacag
77581 gcaaaaatgt catggaagtt ttcgccataa gcagcattac acatactctt tagtgcttcg
77641 tctccaagcc atccttcttt agaacgatta agaacatatg tagaatcagg agattgtaaa
77701 aatcttcccc aataatctaa aatatcacgt aatttttgat taacacgtgg atccataaaa
77761 gcgacatagc cagctttggt tgccattggg taatctaaga tagcattgat tgggaagccg
77821 actaatccag tagcattgaa ttctggagcc cggtgcatga cacggttaat aactctgagc
77881 atttgatgat aattatgaac agctggggtg tccattggtg tttcgacata tttagctgga
77941 acttcttcaa acatcatatg gaataaattc caaagatacc gatcggattc aattagatct
78001 ttcaaggctt gaacaggtgg taaaagagtt tgatcttgtt ctgcttcagc ttcttttaac
78061 acctgtttta gccaattatt gtaaaactct tgatcgctag gtaaccattg tccaacatta
78121 aattttgcat ctttttcat ttttaacacc ttttaatatt aataactata atgaaatctt
78181 ctttgtgact agaaggtaga cagaaacaac agccaaaata caaatgataa tagccaatgc
78241 taattcccat ttgttaaata gaggggtacc ttttttgtcg tattcttttc tggcaaagta
78301 gtagaagatt atacctacag cataaataat tgttgaaatc agtaggtaat taagtcctgc
78361 tgcatataag agccatagac aatagatggt tgataaaata cctatgaata aactcttatt
78421 acgtttacga gaatcattag caaatatttc acgtttttga gcaattttcc ataagtatat
78481 tgaacttaat aagtaacaag gtaaaattat taccccggta atgtcgatac atgcgagata
78541 aacattatta gctgttacta ctaagatcat gaacaaggac atcaaaatac ttgaaatgta
78601 taaggataca ctaggtacct ttttcttatt ttcatgagca aagaattttg gcaatacatg
78661 tcctttagct gcagcatatg gaagttgagc catcataacg gtccatgcaa cccatgaacc
78721 accaacagag ataattacgg aaattgtgac gaagtctatg aaccatgggc ctaataagtt
78781 tttcatcaag taagctgttg atggatcagt taatttagaa agagttggtt gatgataaat
78841 accataagca acaatactaa ttagaacata ggcgattaat gaaattaaat agcccattac
78901 agttgcttta ccaacatctt taggattttt agcgtggtta gaaataacaa ccgcaccttc
78961 aataccgata aagcaccata atgttactaa cataggagct ttaatttggc taacaaagtt

79021 tcctaagtga agaccttgtc cccagaatcc ccagaaaaat gttgggaagc ggaagaagat
79081 tagcatacaa attagaatga ttcctaaact aatgaacttg atgataactg taatgttgtt
79141 aagaaatgag gcttctttaa caccattaag aactaaaaaa ttataaatcc aaattaaaat
79201 aataccgaat ataacagttg gccattggtg tttaagcaag accggaaagt aacgaccgaa
79261 tgagtcatta agcataacgg cataagctac attacctgta attgcttcaa tccaataacc
79321 ccatgctgaa ttgaacccaa catatttacc gaaaccagcg cgtgcatacg aataaatacc
79381 aatatttaaa tctggtcttt cttcagctaa gattttgaaa gtaaaagcaa gtccaaacat
79441 gccaataccg gtcaagatcc aagcaataag gacagcacca agtgatgaag aagcggccat
79501 attttgagga atatcaaaaa ttccgccacc aatcatggcg ccaactacca tagctattaa
79561 gccaaagaga ccaattttat gacttttagt tttagtcatt ttgaagtctt ctttctttta
79621 attaaaatcc aacttattta acgcgagcta tttcttctct aactttaata tttgagctac
79681 ctaatccagg ttcttgatca tgtaaattaa attcaacttg agtttcaggc ctaaatagaa
79741 gtacatgagt tgaaccacca aagtggaaca ttcctagttg atcacccttg ttaacgtgtt
79801 gaccaacacg tacagtgatt tcatcacttg atacttcaga catacctaca gcgacaaaac
79861 acatgagacc aatctttgga ttatcagctt tgatgaaaat aacagcacga gtagctgtag
79921 cagttaagaa ggcttgtgaa tcgtttgcgg caaccggatc agcaccacgt tcatttgcga
79981 agccttgatg taaactttca gagtagtatg aacctggtaa attatatgct tttacaacag
80041 ttccactaac aggactattc catctgtgat aacttaaagc acttaaaaat gcttgatata
80101 aggttccacc ttcaaatttt gaggtccatg ggtcgttatg aagaagatcc attaatgagt
80161 aaggttgtcc tttaatccag aatttagctt tcattggtaa atcacgagca attctaaatg
80221 gagcagattc acatgcattg gcgattgaat ttggatcttc aggatcttga actggacgaa
80281 tgccaggatt aaataaacga gtaaagaaat tgtcccagga agtaaaacct aaatgttttt
80341 cacggtcatc tgactcacat ttaaaaatag ttgtaaaatt atcgccatca gctgctttag
80401 acatttcatt taatgcgtag tcacttaacc agccattttt agaagtgtta agaacatagg
80461 tagatgcggg agtttgcaaa tatttgcccc aataattcaa tatatctctt aatttctcat
80521 tgatttttgg attcatgaaa aacacatatc cagccttagt agccattgga taatccaaaa
80581 ttgcattgat tggcgttccg accaatccag tagtattaaa ttctggagct ctttgaataa

80641 tacgattgag cattaaaagc atttgacgat aatttcttac ttgaggagtg cccatcggtg
80701 tgtcgacgta tttttcagga atttcatcaa acatcatttg agtaatattg tacaagtatc
80761 tatcagactc aattaaattt ttcaaggctt gaacaggagg tagtaacttt tgattttctt
80821 cactttctgc ttcttttaaa accttcttca gccatttgtt gtaaaattct tggtcacttg
80881 gtaaccattg tcctacatta tattggatat tctttttcat cttatttacc tcttgtattt
80941 ataaaaaata acaaaaaaac ataataaagt gcactttcat catcactata aggctaaatg
81001 taagtgagat caaaaaaaac gtatttttaa ataaaaaaac aggtagcgct aaactacctg
81061 tctttgcata catttaattt aaaaactatt atttaattct ttgcagcgtc actaataatt
81121 atggtgaaaa gcttggaaca attgttgttg aaaagaagga tggcaagatt aaagcatata
81181 gtgatgtagt gaaaaatttt gatttaatca ttcatgatgc aggaaaagaa gatcaaaagt
81241 taagtacaaa tgacggacaa attaagaatt aaaagcagta tgattatctt aaaggaatag
81301 ctacgttttc tgaaattgca gatagaaaga aacaaggaat aaagtcatga aacgtatctt
81361 aaatactaac gaatttctac atggcggtga ttataatcct gagcagtggt gggatgaacc
81421 ggatgtgatc aatcaagatt ttgcactgtt taagcaagca aagataaata cagtgacggt
81481 aggaattttt tcgtgggcaa aattagaacc agaagaaggt aattatgatt tttcttggct
81541 agatagtatt tttgaccgag ttgaggagat gaatggtcat gttgttcttg ctacgcctag
81601 tggcgcaaga cctgcatggc ttgcacaaaa atatcctgaa gttcttcgaa cagataatct
81661 aggaaataag cggggttttg gtggtcgtca taaccattgt ttgacctcac ctatttatcg
81721 tgaaaaagtg cgagagatta atacgaaatt agcggagcat tttggtcaaa gaaagagtct
81781 agtgctttgg catatttcta atgaatattc aggcgaatgc tactgcgagt tatgcaaaaa
81841 tgcttttaga gagtggttaa aaaataaata tggaaattta gataatctca atcatgcttg
81901 gtggaatact ttctggagtc atacttataa caattggttg caggttaatc caccaagtcc
81961 tttaagtgag atgggtaata aagggatgaa tctggattgg aagagattta ttacagatca
82021 gacaatttca tttattgata atgaaactgc acccttgaaa aagataacgc cgaacattcc
82081 agttactacg aatatgatgg ctggtaatcc attaatggat ccatttgctg gttttgatta
82141 tcaaaaagtt tcaaagcatt tagattttat ttcttgggat tcctatcctg cttggggaaa
82201 tgataatcaa actacagcag aacttggaag aaatgttggt ctaattcatg atttctttag

82261 aagtttgaaa catcaaaact ttttagttat ggaaaataca ccttcccgtg
ttaattggca
82321 tagtgttgac cgagcaaaga gaccgggaat gcatgaatta gctagtttac
aagatgttgc
82381 tcgtggtagt cagggagtat tatattttca actgcgagct tctcgaggat
catcggaaat
82441 gtttcatggt gctgtgattg agcatcttca tccagaacaa acacgtgctt
tcaaagacgt
82501 aactacagtt ggaaaagatc tagaaaatat tagaccaatt attaatacta
attacgctaa
82561 agcacgtgta gcgattgtat ttagttatga cagttattgg gctttgcaag
atgctgaaag
82621 ctatagtaaa gataaaaaga tctggcaaac aattcaaaaa cattatcgtt
atttctataa
82681 gcacgatatt ccagtagatt ttgttagtgt agaagatgat ttttctaatt
acgacttatt
82741 gattgatccg atgcactttt taatgagtaa agcctatttg aagaaacttg
ctagctatgt
82801 caagaacggc ggtagagtag ttgggacata tataagtggt gttgtagatg
aaaatgatct
82861 tgcttatatg aatgaatggc caaaagaatt acaagatatt tacggagtag
aaccacttga
82921 gactgatgtc ttatatccgg gacaaagcaa cacattgaat tttgatgggc
atgaatataa
82981 ggctcatgat tattgtgaga ctctaattaa ttgtagaggt aaagtgttag
cgaaatatgc
83041 tagtgatttc tatcaagaca ctcctgcggt agttgagcat gaatatggag
caggcaaagg
83101 ttattatctt gcctgccgaa ccgattatga ttttttagag aagttttatg
aaaaaataac
83161 tgctaatttg attccagaat ttccagttaa aaagtttagt agcaatattt
caattcaagt
83221 acgcgaaaat aaagatcaga aatattactt tgtacaaaac ttttctgata
agtctgagca
83281 gattaaagtg gatggagaat tagaggatct tcttgaaaaa aagatagata
gaggtgaagt
83341 tgttcttaat ccatttggaa gtaagattta ttataaaaaa ggtaattagt
taaaacgttt
83401 taaatatttt ctgaaaaata aatttcttat ataaagtgat aactttttaa
caaataaaaa
83461 atataaataa ataccgatgt atcaatgttt ttagtcgttg atataccggt
atttttagt
83521 ataaaaataa gattaaatag ttactttta aacactttt atagaaagcg
cttgcaattt
83581 ataaaaaaca taatatactt aaattgtaac ttgaaacgtt taaagttttg
aaaaacatgt
83641 tggttagtgt cttatcaaga caagattagt ttatttgaaa cgtttagatt
tttacagggg
83701 gattaaaaat gccaattaca ttagcaagaa ttgatcaaag acttattcat
ggaattactg
83761 ttaaccaatg gaataaagtt ttacatccaa agagattcat ggttattgat
gacgaagtta
83821 gtcaaaatga aatggtaaaa gcaagtatga gaatgagtaa gcctacaggc
acagggatgt

83881 caattattaa tgaagaaaag gcaataacta attttaaaaa tggtaaatat gattctcact
83941 ccgttttctt aattgtaaaa gaacctcatt atattctaga tttaatcaat gctggtgtag
84001 aagttccaaa ggttaatgtt ggtgcgttat ttgaagaagg taatagaaaa ccttatacaa
84061 agagagttgc tttagatgat caggagttat cagatttaaa ggaaattgca gcgaaaggaa
84121 ttccggtatt ttttgaatat actccagatg atgaaaatga agtctcgctt gcagaagtac
84181 taaaaaataa aggagagaat taattatgcc tatcatatta caaaacatcc tgataatttt
84241 acttgcagcc tatatggctt gggataacgg tacaggtaat cagattacgg gtaactggcc
84301 agttatcatt ggtatgttag tcggcttgat tatgggaaac ctaagtgtag gtttggtaat
84361 tggtggtacg cttcagctta tgtccttagg tgttgcagct attggtggat catcaatccc
84421 agaatatggt gttgcaacta tagtcggtgt atttatcgct attcgaactg gcgcttctac
84481 cggtactgca attgctgttg gtttacctgt cggaatgcta actttgcaat tagatgttgt
84541 tatcaaaatt attaataact tctttgctca ttggtcacaa cgccttcttc atgaaaagaa
84601 gtttaataag atggatggag tcttttattt aactattttt gtttggatgc ttaaatacat
84661 tatcccaatt gcattagtag tgatgtttgg tactccaatt gttaaattaa ttcttcatgt
84721 tattccagct tgggttacaa atggattaac tattgctggt gggatgctac cagttgttgg
84781 tattggaatg ttattaagat atatgccagt taaaaaatat ttagcttatt tattagcagg
84841 tttcgtttta gcaggatatt taaaagttcc aattttagga atcgctattt taggtttagc
84901 agcaacttat gaaatttatc gtattaatgc agctaagaaa gatacagaag aagtacaaac
84961 tgcaggaaca gtagaaaatg aaggagacga cttcgatgag taaagaattg aaccaaccag
85021 aaaatggtaa aaagttaact acccatgaac gtaaagatat gtttagacgc tggattttta
85081 atgctggctt aggttataac tttgagacgc aacaagctcc ggcggtagct ttttcaatga
85141 gaaaagcttt aagaaagatt tatccaaatg atgatgaata catcgaagca atggataatc
85201 attacaaata tttcaatgct aatccattaa tggccaatat cattttagga gctactttgg
85261 ccatggaaga aaaggatgga ataaaggcta aagatgctgt tcaatccttg aaaacttctt
85321 taatgggacc attttcaggt gttggtgatt ctgtcttttg gattttgatt ccaactatta
85381 tgggttcaat tgctggttac atggctttac agggtaatgc agttggtgca attttatgga
85441 tgttattaca actttcattc tattggttaa aaatgcggtt cttcaaaact ggatacgatt

85501 caggtactaa gttaatcaca tcattaggtg aaaaaattaa tatatttact gacgcaattt
85561 ctgtcatggg tttaatggtc gttggttcat taattccaac agtagttaaa gtatatacac
85621 cattaaaatt tagaactggg aaagttatgc tttctattca aagtggtatt cttgacaaaa
85681 ttatgcctgc tttgcttcca gttgctgtta ccggtcttgt ttactggctc ttaggcagaa
85741 agaaatggac accaactaag attattttgt tgatcattgt tatttgttta gctggtgctg
85801 caacaggcat cttaggtgtt gcaaaaataga ttggaagaaa aatgagtaat attaaacgtc
85861 atattgtgat tgcatcacat tcaactctag ctgagggaat ggccaatgct ttaaaatttt
85921 ttgaaggtaa cagtcttaaa ctgacagttt tatctgccta cgttgataac aagccaattg
85981 aaggacaaat tgagaaaata tttaatcaat ttgataaaaa tgatgaagtt attgttttaa
86041 ctgatttatt aggtgggtct gtaaatcaaa aatttttccc atatattagt cgtccgcata
86101 ctcatataat cactggcatg aatttatcat tagcaatggc gattactatg gaaccagttg
86161 aaggttattt aacaggtgaa aagattcaat caattgtttc agaagctcaa aatcaagtga
86221 aatatgtaaa tgaattagta actgattcga gtgaggacga tgaagatgaa tgattggaaa
86281 aagaaagcta tagtctatga ggcgtatgtg caaagcttta atgattcaga tggtgatgga
86341 ataggagatc tacctgggct aattgaaaaa ctagattata taaagaattt gggtgccaat
86401 gtaatttggt taacaccaat ttttaagtct cctttggtag ataatggata tgatatagct
86461 gattatgaag ctattaatcc gatttatggg acaatgaatg actttaagaa gttgctgcgg
86521 aaggctcatg aaagagatct gagaattgta atggatcttg ttgttaacca tacgtcagat
86581 caacataagt ggtttaaaga atctaaaaaa aataaaaata ataaatactc tgattattac
86641 atctggcgcg atcctaaaga agatggaagc gcccctacga atctaggttc agcatttggc
86701 ggatcggctt ggacttatgt acctgaacga aatcaatact atctgcatct ttttgcaaaa
86761 cagcagccag atttaaactg gaataatcca caagttagaa atgatatcta tcacatgatg
86821 aagttctggc tagatatggg agttgatggt tttagaatgg attcgattag tttcatttct
86881 aaaccaagta aatttgaaga tgctcctttg gaagataata aggaatacgg tgcatattat
86941 tatggatcag ctaatggacc gcatattcat gaatatttac gtgaaatgaa tcagaaggtt
87001 ctaagtaaat atgacgtgat atcgattgga gaaacgccac ataccacagc taaagaagct
87061 cgactttttg ttgaacccga tagacatgaa ttggatatgg tatttcaatt tgaacatatg

87121 catgtagatt acggtgaatt tggtaggtat tctgatgtta cttataagat gagtgaccta
87181 cgtaattcaa tgactagctg gcaaaatgat ttgtcatgga attcaaatta tttgggtaat
87241 catgatcaac cgcgaattgt ttctagattt ggaaatgatt cgaaatatag aaaagaatca
87301 gctaagatgc ttgcaatggt atctttattg caaaaaggga ctccttttat ttttcaaggc
87361 gaagaaattg gaatgaccaa tttacatatt aagaatatag atgaattaaa ggatttggaa
87421 gctcataata tatatagttt cttaaaatct aaaggtatat ctgataaaga cgccttaaag
87481 atggttaata gaaaaacacg agataatgca cggacccccta tgcaatggaa taagcagcaa
87541 aatgctggct tttctagtgg taatccgtgg atagaagtaa atccaaacta tttagaaatt
87601 aatgtaaaga aagatttaca agatccaaaa tcaatttatc atttttatca aaagcttcta
87661 aaattaagaa ataataatca ggtgttactt gatggcaact atgatttaat tacgcctgaa
87721 gattcggcag tttttgctta tactaggagt agtgaaaaag ataaaattgc ggttatttgt
87781 tcttttgtac catataaagt taaatatgaa ttacctaaag attttgtaaa taaaaataaa
87841 gtgattttgt ctaactatga cgataaagaa gatatggcct caaatacaat atatttaaga
87901 ccatatgaag gagttgtaat taaacgtgag taacatgaag gacgtggcgc gtgaagctaa
87961 tgtttctgta gcaacagtct cgaatttcct taatggaaag aaagtgcgcc cgaaagctgc
88021 aaaagatatt gatttagcaa taaaaaaatt aaattatgta cgaaataata cggcacggtt
88081 tttaaaaacg caaagatcag cttttgtggt ttttgttgtc ccaagtgttt ggtctccatt
88141 tttttctgag ttaacttttt gggttcagaa gtatttagac aatcttggat ataaaatggt
88201 gttatgtgta tcagaaaatg attatcaaaa agaaaaaagt tatgtcacaa tggcagaaga
88261 acaacgagta gccggtattc tttccatctc atattctgat ttaactagtc atgtgcattc
88321 tgatattccc ctcgtttcta ttgaaaaaga aagtacaggt caattttcac tagtttcatc
88381 agataattat gaaggcggaa agatagcagc taaagaatta aatgaaagaa atatagataa
88441 atatatattt attggttcag ctaatcattc ttctgtagaa atgaatgcac gtaaagcagg
88501 ttttatagat tattgtaata atcatggcct caaatttgaa atagcagaag tcgactctca
88561 aactgatgac aaagagaaaa ttgatagtct aatagatgag atttcaaata atggtaagaa
88621 gtatggaatt tttgctcata ctgatgaaat cgcacttatt ttgacgcaaa aatttagaga
88681 tgcaggtata aagattccag aacaggtgca gataattggt tttgatggct ggaaactaac

88741 gcctgataca catttaacta tttcaactat tagacaaaat attgaagaaa ttgctaaaac
88801 agctgttgaa cagttgcata aggaaatgga agatccagat catagagaaa tttcacgatt
88861 aatgattcct gtaacttttc gtgcaggaga tacgactaaa aataaataag agaggaaaaa
88921 atgtttaata ctaaaaatac tcagtttctt gattattttt atgaaggtga acatttatgt
88981 atttacccat ggggtcatgg actgagagtt catgactctt tttttgacgg gatgcataat
89041 tgggctctta cagagaaaag tgattttgat tctaattttg cggttactta caatgatgat
89101 gaggctacga tagttaatgg aaagactaaa gcaatcgtta ataaaaatgg aaagatcact
89161 ttttatgata aaaataaaat aattttagag gaattttgga gagagcgaaa ttttagaaag
89221 ataaaaatta ataatagtca atttttaaat caaaaagaaa atgaatattc aagcgctttg
89281 aaaattaaag caagagattt ttgcccaaat aataaaggct catatgaagt tgcggtaaga
89341 tttgaggcaa atgataatga aaaaatatat ggaatgggac agtatcagca gtctcaatta
89401 aatctgaaag gctgtaaatt agagctggct caaagaaact cgcaaacaac agtcccattt
89461 ttgatttcta attatggata tggtttgttg tggaataacc caggaatagg ggaagttaca
89521 tttgcaaata acattacaga gtggaaaaat atcgacactg attgtattga ttattggatt
89581 tgtgcagagg atacgccaaa agaaataatt tctgcttaca ctgatgtaac tggacattct
89641 cctaagatgc caaaatatgc tttaggtctt tggcaaagta aactgagata tagaaatcaa
89701 aatgaggtta tttcgatagc aaaacattat catgatttac atattccact atcagtaatt
89761 gttattgatt attttcattg gccaaaacaa ggtgacttta aattcgataa acgttatttc
89821 cctaatccag aaaagatggt gaaagattta aaaaagctaa atgttgaatt aatggtttca
89881 gtttggccaa atattgattg tactagtgaa aattataatg aaatgaacaa aaaggggtat
89941 ttagttcgag cagataaggg aaaaagtatt acgatgactt ttcatggcaa tactactttc
90001 tttgatacta caaaccctga ggcacgtcta tttgtttgga atagaataaa aagaaattat
90061 tatcaaaagg gagttaagca tttttggtta gatgaagcag aacctgaata ttcgacttat
90121 gattttgata actatcgatt acatcttggg agaaactccc aagttggaaa tatctactca
90181 gccttgtatg ctagaggatt ttatgaagga gaaaagagag aaggacagaa taatcctatc
90241 agtttaattc gttcagcttg ggcaggaagt caaaaatatg gcgcacttgt ttggagcggt
90301 gatattgatt caagctttcg ctcgtttaaa tatcaatatc aagccgggct taatattggc

90361 ttagcgggaa ttccatggtg gacaagtgat attggtggat ttcatggcgg
aagttctact
90421 gattctgagt ttaaagagct attgattagg tggtttgaat ttgcagtata
ttgtcctgtt
90481 ttgagaatgc acggtgatag agagccccat gaatttccaa ttgataatca
agtagaagga
90541 acaggtgcac ctaatgaaat atggtcattt ggaaaagaaa ctttcgacat
tttacgtaaa
90601 caagttaata tccgagaaag tcttaagcca tacattaatt cttgtatgaa
tgaagctcat
90661 aaaaaagggt atcctgtcat gagaacttta ttttttgagt ttcctgaaga
taaggctagt
90721 tggaacatag aagatgagta tatgttcgga agaaagtatt tagtggcacc
gatttttgaa
90781 tttggaaaac gaaggagaca agtgtattta ccattgggat cacgatggaa
agacattaac
90841 aagggctcta taagaagcgg tggaatatgg attaattgtg aagcacctct
agatcgaatt
90901 ccgatttttg agagagttga ttactgaaaa tggtctctat agatctacac
caatttgact
90961 acttattata aataggtata aaattaacct ttctgtgtga ataaaacaca
aaagacggaa
91021 aggaataatt tttgatgaat aattcgacaa cagagaagac tttctttgga
cagccacgtg
91081 gtttgtccac tttgttcttc actgaaatgt gggagagatt tagttactac
ggtatgcgtg
91141 ctatcctact cttctacatg tactatgctg tagaacgtgg cggactaggc
atggatgaaa
91201 caactgctgc ctcaatcatg tctatttatg gttcgctagt tttctttct
acagtagctg
91261 ggggctggct agcagataga atctggggag caaggagtac tgtcttcttt
ggtggtgtcc
91321 taattatgat tggacacatt gttttagctt tgccaatggc agagttaggc
ttatatattt
91381 caattgcatt tattgtaatt ggtactggat tgcttaaacc aaatgtttct
aacatggtag
91441 gtggtcttta ctctttagat gatcgaagaa gagatgcagg attcagtatt
tttgttttcg
91501 gcattaacct tggatctgct ttggctccct ggcttgttcc ttgggcaagt
gaaggatttg
91561 gttttaactt atttggcaat catcttaatt tccacgctgg tttttcttta
gcggcagtag
91621 gaatgttctt tgccttggtg caatatgtgt ggggaggtag aaaatacctt
tctgatgatg
91681 ggatgcagcc gactgatcca attgatcctg caactcgtaa taagctattg
aagagagttc
91741 ttttaggtgc tttagtttta gcagctgttt taggtgtgat ggctgcgtta
gatcaattaa
91801 acgtcgataa cattattacc ctaattacta ttgtggctat tgctttgcca
atttactact
91861 ttgttttaat gcttagaagt cctaaagtaa ctaaagaaga acgttcgaaa
gtatgggctt
91921 atattccatt atttattgct gctgcaattt tctggggaat tgaagaatct
ggatcagttg

91981 ttttagcctt gtttgctgcg caaagaacag ttcttcatat tggaaactgg cactttactg
92041 ctgctaactt ccaaacttta aatccattat ttattatgat tttaacccca ttttttgttt
92101 ggctatggga taactggaag aagcaaccga gtgctgctgg taagtttgcg gctggtttag
92161 tttttgctgg actttcatac atgtggatgg cactaccagg tatgctctat ggaacaaatg
92221 gacgggttag cccattctgg ttagttggtt cctggttcat tgttgaaatt gctgagatgc
92281 ttatttcgcc aattggatta tccgtaacaa ctcgtttagc tcctaaggca ttccgttcgc
92341 aaatgatgag tctttggttc ttagctgatg caactggaca agctgttaat tcgcagattg
92401 ttaagtatta ctcaagtaaa actgaagtag cttacttctt agctgttggt ttggtaagtg
92461 tattatttgg tgttgtaatg tttttcttca caaagaagat tcataatttg atggctgacg
92521 ctgagtaatt aagtaaaaaa ttgaatattt aaattaaaaa gattcaagct ttcgcttgag
92581 tctttttct gtaggcattg taaattatta aaaaacgagt agaatgaatt ctgaagaaat
92641 aattggatga cagagagcat tcgttttgga ggaattaaat atggatacaa tggttaagca
92701 cagacatagt ctaagcaaaa atcagaagtg ggttatagca tctacttctt caggttttgc
92761 tcttgaaaat atggatgtgc tttttttatc gtttgcaatg agttcaatga ttgctgatct
92821 gcacttatca ggtggagcag caggttttat ttcgacaatt actaaccttg gaatgttagt
92881 gggtggaatt gcttttggaa ttctaggaga taaaattggc cgtgttaaga cttttagtca
92941 tacggtaatt atttttgcga ttgcgactgc tttgatggct tttgctaata atatttatat
93001 gatctatgga ctacgtttct tagcaggaat cggagctggt ggtgaatatg gtgtcggaat
93061 tgctttaatt gctgaaaact ttcataaaga gcaaattggt aagatgacat cagttgcagc
93121 aattggtggt cagattggcg caattttagc agctttgatt gcggcttgga ttattccaat
93181 ggctggttgg catatgctat ttctagtagg aatcattcca gtagttttaa caattttat
93241 tagaaagcat gttcatgaaa gtgaacaatt tcttgaggct aaagcgagtg aaaagaattc
93301 attattgtca gatgtagcta aaaagatgtt ctctacgctt cgtcttgctt ggcaaacact
93361 gggcttgatg attatgatga ctgttcaaat tgcgggctac tttggcttaa tgaactggtt
93421 accaacaatt gttcaaaagc aattaaactt aaatgtttca aattctagtc tttggatgat
93481 tgcaactatc attgggatga gtatcggaat gatgaccttt ggttcaattt ttgactactt
93541 tggtcctaga agagcctttg caattttctt aattggttct gccttgatgg tatatacttt

93601 gagcttagct caaaatatgg ctactctttt agtaattggt gctgttgtcg gttttttctc
93661 aaacgggatg tttggcggtt acggtgctgt gatcagtaga ttgtacccaa ctgagattag
93721 atcaagtgcc aacaacatta ttgttaatgt gggtagagca attggtggtt tttcatcagt
93781 agtaatcggt attttaatgg atagatataa tcttggtgta gtaatgggct ttttatcagc
93841 cttatacatt attagcttca ttgtgatgat tactttacca ggtttaaagg atttatcaaa
93901 agtaacaaaa tagtatagct tttagcaata gtaaactagt gagaataaat atttttcaat
93961 taataattgt ccttgtatac tagagaaaag taagtgtgag gggaaagatt atgaagaaga
94021 gaaaaattta tcttgctagt ttttttgttg ctttattttt attggtttta acgggatgta
94081 gtcaaaagtc gcaggaaatt ccaacaaaaa aggttacagt ttcattgagt aatactaaaa
94141 caattcctac ctttttcttc cacggttggg gttccagtgt aaatgccgaa aagcatatgg
94201 caaatgcggc taaaaaggcc ggtgcgacta ataccattgt tcaagctgac gtaagtaaaa
94261 atggtactgt taagttgagc ggaactattc ccaaaagtgc caagaaccca attattgaag
94321 ttaatttaga agacaatcaa agtggtaaga cgtcctatgt gaaagacgtg attactgccg
94381 tttcaaataa atatcactac gctaaaatca atttagttgg acattcgatg ggaaatttgc
94441 agattgctaa ttatattaat gagaactaca acaataaaaa attaccacag attaataaag
94501 tagtttcaat tgctggacat tacgatggct atttaggcga agaagccgga caaaaagcca
94561 agatcaagaa taaagaaaca gggcaaccag acatttacag tgatggattt aagaaattgt
94621 tgcctttgcg taaacattat ccaaagcaga tcgaagtttt gaatatttat ggtaataaga
94681 aagatggctc gaattcggac gggtcagttt ctgtagcttc tgctcaaagt tataaatatt
94741 taattaatgg acgtgcaaaa tcatatcgag aagttgaaat gaaaggaaag aatgcgcaac
94801 acagtaaatt gcatgaaaat aaagaggttg atcgtcttct aattgacttt ttatggaaat
94861 aaaaataaaa aatattgaca aatagtcctt gtagatacta atataattaa acgattagta
94921 tctacaggga ctatttttgc gaggcaaagc atggaactaa gtgaaacaga aaaattaaat
94981 tttgcgatta ttcgtggtag taaggcttat gaagaatggg atagacagca aggcatgcct
95041 gcttatttaa cgattatctt gtatgaatta ctattacgca aaagattgac acaaaaggac
95101 ttagtttatc taagtgactt acctaaacaa tcaattaata aagggattca tcgtttatta
95161 gaaaaggatt atttgaaatt aacaattgat ccggatgaca atcgagtaaa gtattgtcag

95221 ttgactgaat ctggtaaaaa atatgccaag gaaaaaatga caccccttatt
tgaaatagaa
95281 aataaagtag ctcaaaagat gggtgcgcaa aagatgaaac aattggtagc
tctaaatgaa
95341 gaatggagtg aaacattttg gaaattttta agaaagaagg gagaaaaata
aatgcagatt
95401 ttgaagccgc atttcaaaaa atatcgtaaa gaaattattt tggcagttat
caccattttg
95461 gtttcagctt ttgcaacctt atggcaaccg cgattgttag aaaacattca
aaaagcaatt
95521 ttagctgata atcatgatac tgttttaaga gatgggattg ggttagtagt
acttggttta
95581 ttagcgatta ttgccggaat ttttaatgta tattatgctg caaaaattgc
ccagggaata
95641 acttcagatt tacgagaaga aacttatgca aaaattcaga gcttttcatt
tggaaatatt
95701 gagaagtttt ctagtggttc tttagtagta cgtttaatta acgatatgaa
ccaagttatg
95761 aatatgatga tgatcttgtt catgcagctg cttagaatgc cgataatttt
aattggttca
95821 tttgtattaa gtattgttac tattcctcat tattggtggg ctccagtatt
aatgcttgct
95881 ttaatgatgg gtgttggctt tattgttatt caaaatatga accagctgtt
tgccaagttt
95941 caaaaatata tggataaaat ttctactcgt gtaaaggaaa atttacaggg
tgttcgtgta
96001 gttaaatctt ttaatcaagt tgaaaatgaa atcaagagat ttaacaagac
ttctgatagt
96061 ttgaatgagt taaacattaa aattggttat tggatttcta ctattatgcc
agcgtttatg
96121 ctaattgctt atttagtaat tgcattagta gtctttttag taggccgcag
tgctaattta
96181 aatccaactg atgtagctgt tgtatcacca tatgtttctt atattttaac
tttgctcttt
96241 gcaattttaa ttgggggctt tgtgattatg aacttcaccc gtgggatggt
ttctttgaga
96301 agaattaagg aagttcttga tactgagccg gatgttcaat ttaatcccaa
tgcttcagct
96361 gcaccagaaa aaggtagtat tgaattcgat aatgtttctt ttacttatcc
agatggggat
96421 aagccaactc tgaaaaatat ttcctttaaa gtaaagccag gagagatggt
tggtattgtt
96481 ggagcaactg gatcgggtaa gagtacgctt gctcaactta ttccgcgtct
ttatgatcca
96541 actgaaggaa tgatcaaaat tggtggcaaa gatttaaaaa ctattggtga
aaaagctctt
96601 cgtaatacag tttcaatggt tttgcaaaaa gcaattttat tctctggtac
aattgcatct
96661 aacttacggc aaggtaaaga agatgcaacc gattatgaat tgaagcgtgc
ttcagaaatt
96721 gcacaagcgc aagaatttgt aggtcaatat ccagatgaat tcgatcatga
ggttgaagag
96781 cgatcagcca atttctccgg cggtcaaaag caacgtttat ctattgctcg
cggtgtaatc

96841 ggtcaaccac ctattttaat tttggatgat tcaacttcag ccttagatgc
taaatctgaa
96901 aaattggttc aagaggctct agaacatgat ctaaaggata ctacaacagt
aattattgct
96961 gaaaaaattg tgtcagtaat gaatgctgat aaaattttag ttttagatga
tggaaaactc
97021 gtcgctgaag ggactcatga agagctatta aagacttctc caatttatca
agatatttat
97081 cgcacacaaa aagctaaaga aaagagaggc gagattgatg agtaatacta
aaaaagcctt
97141 gaaatatttt gctaagtatc tgaagaatta ctggaaagga gttacggtag
ttgtagtcct
97201 ttccttggtt tcgactttaa gtcaagtttt aggaccgctt ttcctgggaa
atgctgttca
97261 agaattgact aaagctgtca gcaaagtagc tagtcgcagt catgatttaa
gtagttttta
97321 tcagtctctt ggctcaatgg tcagcgttta tgcagcagga attattgctt
cgttcattgc
97381 ttggatggta atgtctaaat ttactgcgaa cgctacaaat gacatgagag
agaacttatt
97441 tgccaagttt cagaggatgc taattcgtta tttcgatact catcaagatg
gaaagttatt
97501 atctttgttt aattcagatt tagataatat ttttaacgcc ttaaataatg
caatttttga
97561 aattatttct caaggtgcat tattaatcgg aacaattatt atgatgttta
ttgttaatcc
97621 agtgatggct tggtttacaa tagctacaac cccgtttatt ttgataatta
gtttagtcat
97681 tatgaaaaag gcacggattt atttagacaa gcagcaagat aaaatcagtg
accttaacgg
97741 atatgttaat gaacagatta atggtgaaaa agttattatt actaatggct
tacaaaaaga
97801 atctgttcaa aactttaaaa aatataataa cgatgttcgt agcgctatgt
ttaaggggca
97861 attttattct ggaatgcttt tcccattatt acaaggaatt tctttattga
acttagctat
97921 tgtaattggt ggaggtgcat ggctaattgt tagcggtcag gtaagtggag
cagtgggttt
97981 aggattaatt gttgcttttg ttgaatattc gcaaacatat ttccagcctt
taactcaatt
98041 aacttctatt tattctatga ctcaacttgc tttaaccggt gcaagaagat
tggctactgt
98101 tgaagagcaa gatgaagaag atactgtgcc aaatggcaaa gttttacaag
gaattgaaaa
98161 aggcgttaag cttgaaaatg tgcattttgg ttataacgct gataaagaaa
ttttgcatgg
98221 tgtttcaatt gacgttgata aggggaaatc tgttgccgta gttggtccaa
ctggatcagg
98281 gaaaacaact attatgaact tgattaatcg cttctacgat gttaatagtg
gaaaagtaac
98341 ctttgatggc gttgatgtgc gtgatattac gcttgaatct ttaagaaata
atgtaggtat
98401 tgttttacaa gattcagtac tgtttactgg aacagttgaa gagaatatta
agtatggtaa

98461 gccaactgca agtcgagaag aagtaattgc agcagcaaaa gaagcgaata ttcatgactt
98521 tatcatgacg cttcctgaag gttatgatac tcaagtttcg gaagaaaatt cggtcttttc
98581 tactggccag aaacaattaa tttcgattgc tagaactatt ttgactgatc cagaattttt
98641 gattttagat gaggccactt ctaatgttga tacagttact gaggctaaaa ttcaaaaggc
98701 aatggatgct gttattgcag gcagaacgag ctttgttatt gctcaccgtt tgaagacaat
98761 tctaaattca gataaaatag tagtattgaa agacggaaat gtcatcgaag aaggaaacca
98821 cgacgagctg attaagaagc gtggatttta ctacggacta tatactaacc aaatggcatt
98881 tgaataattt agagggatct tttccctcta attttagtta gggggaaac aaaaaagtga
98941 aaagaaagct acgaattttt tctttattta ctgttttatt gagcgttttt ctactatcag
99001 catgtcaaaa aaatacgcgg aatgtatatc aagaagtcaa aaaaagcaat gaaattacct
99061 ggggcgttaa agctgatacc cgtttattcg gtttaatgag cattaaaact ggaaagattg
99121 aaggctttga ggttgattta gccaatgcat tgaccaaaga aatgctggga aaaaatgcta
99181 aagccagctt tgtacaaact actgctaaaa ccaaaattcc gctgttaaag aatggaaata
99241 ttgatgcagt tttagctgca atgacaatta caccagagcg taaaaaacaa attgatttta
99301 gtaatccata cttttatgct ggtcaatctt tgctagttaa ggaagattca tcaattagaa
99361 atgttcaaga tttaaatggg aaaacagcat tggctgttaa aggtacaaca gctgtcgcta
99421 atgtaaaaaa atttgctccc aaagcaaagg tattagaatt tgacgattat ggtcaagcat
99481 ttactgcact aaaggcaggt caagggcaag cgatgacaac tgataatggt ttacttgccg
99541 gaattgctac tgaaaataaa ggctataagc ttgttggtgg tacatacaca aatgaaccat
99601 acggtattgc cgtgaataag ggacagaagc aaatgaagaa cgcaattaat cgtgctctaa
99661 ataaattaaa aaaagatggg acgtataatg ccttagtaaa gaagtggttt agcggtattc
99721 caggctttaa tattaaagag gctgaaggtg aaaaataaat ttttttactt gccttttttg
99781 aaaaaatatt attaaatgta atcaacattt aaatttaaaa agcgatgaaa agaaaagtaa
99841 ataatttatt tcttttagtg agttaacggt agtggaaaag ttagcagacc ctggattatt
99901 gaaagacact tttacaatgc tgctggctga aatctttaat gagagtaagt ttagccgtaa
99961 gcggtacgtt atctagccgc tggagcatgt ttgtgctcta gtcaagttgg tctttaagta
100021 gacaatttag gtggtaccgc ggaaaaaagc ctttcgtcct gagaaatcta ggagcgaggg

100081 gctttttct ttactccaaa ttaaaaaagg agttatgtct gagtaagcaa tagctaagct
100141 atattaaagt taattttaat aattttagag tagaatggaa aagtgagtaa aaaagagagg
100201 ggaagcacaa tggctgcaat tattgatttt aagcatgtaa ataagtacta tggtaaattc
100261 catgctttaa aggatataaa tttaagtatt gaagagggtc aagttgtttc aattattgga
100321 ccatctggtt ctggtaagag tactttgatt cgaacaatga atggactaga gcgaatcaat
100381 tctggtactt taatggttac tggctatgat ctagcagata agcatacgga tttaaacaaa
100441 attcgtaaaa atgtgggaat ggttttccag cattttaact tgtatgacaa ccatactgtg
100501 cttgaaaata taactttagc tcctaagatt gttttacacc gtccagagaa ggaaaaccac
100561 gatattgcaa tggatctttt gaaaaaagtg ggtcttgaag aaaaggctaa tatgtatcca
100621 agacagttat ctggtgggca aaagcaacgt gttgcaattg ctcgttcatt agcaatgaga
100681 ccaaaagcta ttttatttga tgaaccaact agtgcgcttg acccagaaat gattcaagat
100741 gttttagatg ttatgaagta tgtagcagat caaggaatta caatggttgt tgtaactcat
100801 gaaatgggct ttgctcgtga agttggagat agacttattt tctttgatca aggaagaatt
100861 ttagaagatg ctaagccaga agagttcttt gaacatccta agactgaacg tgcacgtcaa
100921 tttttaagta aggttattac tgaaaagcta ggtggctaaa atgaaaaaat caattaagat
100981 ttttatttta cctttggtat taggtttact gattacttta actggatgtg ccaagaagca
101041 aaaatcaagt aatgtttatg aacaggttaa agcaagtaaa accattactt ggggcgttaa
101101 ggctgatacg cgattatttg gtttaatgag tattaaaact ggaaaaatcg aaggttttga
101161 agtagattta gcaaaggctt taactaaaca aatgttaggt aaaaatgcaa aagctgaatt
101221 tgtgcaaact acgcctaaga cgagaattcc gttacttaaa aatggtaata ttgatgcaat
101281 tttagcaaca atgacaatta cccccgatcg taaaaaacaa gtaactttta gtgaaccata
101341 ttttactgca ggtcaatctt tactagttaa ggataattca actattaaaa atgttaagga
101401 tcttaatggt aaaacggctc tagctgttaa gggaacaaca gcagttgata atgttaaaaa
101461 atttgcacca aaagcaaagg ttttagagta tgacgattat ggtcaagctt ttacggcctt
101521 aaaagcagga caaggtcaag caatgactac tgataatggc ttgcttgccg gaattgctag
101581 tgaaaataag ggtataaat tagttggtgg tacctacacc agtgaacctt acggaattgc
101641 tgtagaaaag ggacaaactg gttttgctga tcgcattaac aaggcactaa atgaattaaa

101701 gaaaaacgga acatatcatc gtttgttagt taaatggttc agtggtattc caggatttaa
101761 cattaaggag gttgaaaatt cgtgataggg attttaacaa atcactggtc tgaattctta
101821 tcaggctttt ggaacacaat cttatgtagt ctaattgccc tgttctttag tttaattctt
101881 ggggtaggct ttgctttatt agaagttgca ccgaataagt ttggtagagc agtggcacat
101941 gtctatattg aaattttccg taacattcct ttgctagtta ttacaatgat tttttacctt
102001 gttgtaccgc aaatttggtt taaggtatca ggttttgcag ctggaacaat tggattaacc
102061 ttgtatactt cagcttttat tgcagaaact gttagggcag gaattaactc agttagagat
102121 ggacaaatgg aaggagctcg ttcaaatggg atgacttata cgcaagcaat gcgatacgtt
102181 attttgccac aggctctaaa gattgtaatt cctccattgg gcaatcaatt tattaactta
102241 attaaaaatt catcggtatt agcctttgta gccggttttg acttgatgta tcaagcagat
102301 gtcatagctt tttcatcttt tgaaactatt aatacctatg ttgttgtagg tttattttac
102361 ttaattttaa ccttgccatt aagttactac atgcgtcact tggaaaagaa attagcctag
102421 ggaggagaaa aagatggaaa actttattca tgcatattct tggattaaca ttcgctttct
102481 cttacaaggt ttgtgggtga ctatatatgt atccttaata tcaattgcct tgagttttat
102541 tctaggatta gtattcggct ttattcgata tgtgaagatc aaatatcttt caactattgt
102601 tggttttgta attgatatta taagaaattt accgctattg ctgataatct tctttacgta
102661 ctttggattg ccagagttgg gaattgttac taatccaaca gtagcatcga ttattgcttt
102721 ggtagtattt gaaggagcaa tgcttgctga aattgttaga agtggaattg ctgctgtaga
102781 tccgggacaa atggaaggag ctcgttcaaa tgggatgacc tatatgcagg cgatgtatca
102841 tgtgatcatt ccgcaagctc ttaataagat gatcccgtcc cttctgtcac aattcgtttc
102901 attggttaaa gacacgtcac ttgcaacaat tattgttttg ccagagttat tgtttcatgc
102961 acagattatt tacagtcaaa atacgacata tttgattcca atgtatgtaa ttattgcaat
103021 tatgtatttc attgtttgtt tctcgctttc aatgattgct aagcatttgc aaagtaagta
103081 ttaattatat gaaaaaccac gtgagttgaa ctcacgtggt tttttgttga ttaattattt
103141 tttataaatt gttttagttc tttttgagag aagtatgaac cataatcttc agttaattgt
103201 tttaaaatga agtcattgct agcattcaat tttctactga gcttaatcat tttcttaata
103261 gcgtcaattc gttcttcttc ttttcctgtt ttttctccaa catatcgcat atcatcaagt

103321 ttcatttggt aatccatgag ctctttcctt ctttctggat tagttttata tttctcaaca
103381 tcgctttgta acttttgaat aaagctatca tttgtattaa ttttaccacg cattaaatca
103441 aaaaatcctt gcagacgggc agttagttaa cccagctaaa gatgggatca gcggtgatat
103501 tagcttcatc aacccgtttt tctaattctt tcctatccat agaaaactcc tttaagtgta
103561 ttagagaaag atctctaatc ttaattacgc aaaagggagt tttatttttt tgaaaagaaa
103621 aagaagatca atctgatctt cttaaattaa agtttgattg ctaaagcttc ataaggcttt
103681 aattgcaaga ctgaaggcag agaagcaaga tttttatcgt aattttgaat taaaatatgc
103741 ccctccttat ttaaatattc agcaggtatt ttaacttcag tttctttgcc atagaagttc
103801 gcaaatacta aaagtttttc attagagtca tctaaataac gctcataggc cataacttgt
103861 gggtgttcca ttaaaaatgg tttgatgtga ccttcagaga tcaatttttc tgattttctg
103921 agtttaatta atttttggta gaaattaaaa atttcaccat cctgtagttc tttttcaacg
103981 ttaattctat tttggtctgt tggcatgagc caaggtttgt ggccactaaa accagcatat
104041 tgagaattat tccagtgcat tggaaccctt gagttatcgc gagctttaga ctgaacaatt
104101 tcaaaggctt ctttttcatc aataccttttt ttgattaatg cgtcataggc attcaaagct
104161 tcgacgtcca cataatccct catggaagta tagtgaggat ccatcatacc aatttcttca
104221 cccatataaa tgtaaggcgt tccgcgtagt agatgaagag caagagctaa catctcagca
104281 gatttattgt gatattttcc agtatcaccg aatcgattta atgcccaagg ctggtcgtga
104341 ttattccaga atagagcttg ccagccacca ccttgatcaa tcttactatt ccaggtaagc
104401 ataatatctt ttaatttctt aaagtcaaaa ggcatcttag accattttc accgttttta
104461 tagtcaactt tgaggtgatg aaaggtaaag accattgaaa gctcatgatc ttctggacgg
104521 gtataagcaa ttgaattttc aattgtagtt gatgacattt ctccaacagt aactgattct
104581 gggtcttgac cgaaggattt ggcatttaat tcttttaaat attgctgcac cactggtgta
104641 tcagtataaa gggatttttc tttcaccgga tctgttgagt taactaaaat agtatctttc
104701 cctgtaacgt tgattacgtc aaagcggaag ccgtgaactc ctttattacg ccaaaagttt
104761 acaacatcta tcaaggcagc acgaacttca ggattatgcc agtcaagatc tgcttgagtt
104821 ggatcgtaga ggtggagata gtagtaatca gtatcgccaa attttgacca agctggacca
104881 ccaaattttg attgccagtt attaggtagt tgtctgtcct taccttttct aagataaaag

104941 aatttttgat actttttatc tccagctaga gctttttttaa accattcatg ttccgttgaa
105001 cagtgattta aaaccatgtc gagcatcaca ttaacattaa ttgcttttaa tttagcgact
105061 agttcttcga agtcgctcat tgttccgaat cgaggatcaa tttttttata atcagcaatg
105121 tcatagccat tgtcattttg tggagaaaca aaaaatgggt taaaccaaat gaagtccacg
105181 tttagtttct tgatatagtc gatttttca atgatgccac gtaagtcgcc gataccatca
105241 ttatttgagt cataaaaaga tttggggtaa atttgataaa ttactttatt gcctaaatta
105301 ttcattgctg attcctttct aaaatttaat cttttttctg cgagcaaaat ccacaaattt
105361 gaatttgtca ggtcgatgat aggacagggt taattcgaat ttagttgtgt cagctaaata
105421 atttctactt gcaactagca ctgcaagttt attgtctaat tgaagtaaat cttgaattct
105481 cttgtcaact ttacaaacag aaatttcttt cgttgcatag gaaatttcta atttttcttt
105541 attttctaaa taattataaa tagaagtttc tgcggcatct tttggaagtg aattaatagg
105601 aggagaaagc aaataatcgc aatctaagac atcagcttgt ccttctatta atcgcaagcg
105661 ttcaagatga aaaccagcac tgttttttc ttcaggaaaa tatttgataa agccagttgg
105721 caaatgatct tctttttgta aaagtaaaac ttttgtttct gctttaagtc ctaattgatt
105781 attcaattct gcaaaactgc taattcctga aatgggaaaa gtgtagcgat cgagatttaa
105841 aactattgaa ccttttccac gaatcttctg aattaagcct aggtcggtta agctagtaag
105901 tgcttttcga attgtttctc ttgaagcacc atataagtcc attaattgat gttcactagg
105961 gagatattcg ccaggtttat attgttgatg cttaatttta tcaactaaat cttgagcaat
106021 agcactaact ttagattgag ccataaaaaa tcctcctttg tctttaatta tatacacttg
106081 tttagacaag taaagcaaaa ctttttcatc tataaaatag ttaatttaaa agaaaagtaa
106141 gctttttcat aaaatggtat tgacaacttg tacagacaag tataatattt aaatcaagat
106201 atatgaaagc ggatactttc aaagaggaga atgaaattat ggtacaagat actactaatt
106261 tattagctcc agctactgga gcggttattg ctttagctaa gacttcagat cctattttt
106321 ctcaaggaac aatgggagat ggatttggtt tgaccccaac tattggaaag gttgtagcac
106381 ctgtttcagg aaaaatcagt atggttgccg atactaaaca tgctgtcgga atcgttacta
106441 acgaagggct agaagtacta gtccacatgg gcgttgatac agtcggctta aagggtgcac
106501 cattcacgat taagattgaa aatggtcaag aagttaaggc cggcgatgaa attgctgaaa

106561 tggatttaga tgaaatcaaa gctaagggat tagatacaac aatcatggtt ttgattacta
106621 attcagctga caagatttca ggacttgatg taactgaagg tgaagcaaaa gctggtgaag
106681 ctgtagcaac ggcttactta aagacggcag aagcagaaga cacttcaagc aaaaaattat
106741 cttatccaga gttagcttct tttattatta aaaatgtcgg cggtaaggag aatgttaata
106801 atgtaattca ctgtattaca cgcttacgtt tttacttaaa agatgaaagt aaagcaaatg
106861 atgacgtttt aaaaaatcaa cgtggaattt tagacgtgat gcatgcgtca ggacaatatc
106921 aggttgtaat tggtaatgaa gtaactaatg tctttgatga agtagttaag caattaggac
106981 ctttagatag cgaagcagct cctgctccag aagatgacgg taaaaatcca gtttctaaag
107041 cttttggtaa tttaattgga tttatcactg gttcaatgag tccaattatc ggtgtaattg
107101 ctgcagctgg tattattaaa ggtattttgg cattgcttac tctgccacaa ttaggtagtt
107161 tacttagtgt caagagtaca gcctatatca ctactagcgc aattgctgat tcagctttct
107221 tcttcttgcc agttctagtt ggttactgcg cagctaaaag attgaattcc gatccaatca
107281 ttgcggcagt aattggtgga gtattagttt atccgcaatt agttgaatgg ggcaaagcct
107341 ttaagacaat gtttgaagtt ggcggaatta agtttgaatt tttgaactat acttactcaa
107401 ttttcccaat gattttagcc gcatggcttg ctaagaagtg tgaagattgg cttaagaaat
107461 ggctaccatc gtacttacaa atgattttta ttccattaat tactgtttta gtagtttcag
107521 cattgacttt aatggtaacc ggcccaatta ttcaaggtgt tgctaatggt attgcagtct
107581 tcatcaactg gttagttcat gcatctggct ggattggtgg atttattatt ggtggttttt
107641 accaagtatt ggttatcttt ggtttgcact ggggtgttgt gccgcttgtt gcccaacaaa
107701 ttgctggcac tggtgaaagt gcattgaatg caattatttg ttcatcaatg attgctcaag
107761 gtgctgccgt attagcggta gctattaagt ctaagaaaga agacttaaag gaattaggtt
107821 ttgcttcaac tatttctgca atgtgtggtg ttactgaacc agctgtttat ggtattagtt
107881 taagatataa gaaagtattt atttctggtt gtattggttc tgcatttggt ggtcttgtta
107941 ctggtttaat gcacggaaca atgtatggct ttactggtgg attaattgga ttttcttcat
108001 tctttaatcc taagcatcct actgatttat ctagcttcta tacatttttg attgctagtg
108061 ctgtaacaat tgtggtttca tttatcgtaa cttgggtttg gggctacaat gatagcatgg
108121 ttcaaggagc taaggttgca aagaaaaaga gacctggtag taagtaaaag aatttattaa

108181 attagataaa aaataaaaca acgttgaaaa tttcagcgtt gttttttgcg tttaggagtt
108241 gaaaatatta aatcatatta atataattaa taataattta attaagtcgt ggattagggg
108301 gaaaggctat gatttttgaa aagctttttt ctaaagagaa taaaaaagat gagatagttg
108361 atgtaaagtt aagtgataaa aagttcaatg aattaagaat aaagctaagt attggagacg
108421 taacagttga acaaggcgag attttttcgg ttaattttac tgggaaaaga cactacttac
108481 cgcttattcg cttagaagat ggtaaactac gacttaagca aaaaatgaat ttgattgaaa
108541 agggagaagc tcacttaaaa gttgtaattc ctgcaaaggc tactttggat agtcttgata
108601 ttcaaactag tagcggtgat attgagatag ttgatattaa gactgaaaaa gcttatctta
108661 aatcgactga aggtagagtt agagtaagac gagtggcgat agctgagtca cttcttgaat
108721 caacagatgg aaatgtaaca gtacgaaata gtgatctggc cgatggcaag ttagcgacat
108781 atgatggtga aatcaaagta tcgggatcta ttttatctag gatttcttta aatgtaacag
108841 atggagatat gaatctcact gatgttacga ttgacgaagg cagcgcaaat ttaaaagctg
108901 gaaactttac gctcgatcat gcagtattta agagtgatta tgaaataaag aatattgaag
108961 gaaataatac tgtttggagt gcaaatttaa aatatgccca tgtaagaacg gcaaatggtt
109021 taaataatat tttgtttgat ccacaacctt caggcgttgt tttaacaatg acaacggtag
109081 atggagataa tgtagttgag tagaaaaaaa taaacctcgt agagtaacct acgaggttta
109141 tttctattta acgttactta aaactgaacg aattttagta gtaagcatat caattgctac
109201 atcattttcg ccgccttctg gaacgatgat gtcagcgtag cgtttagttg gttcgataaa
109261 ttgattgtac attggtttaa cggtctttaa gtattgatca ataactgaat gaactgaacg
109321 accacgttct tgcgtgtcgc gctctaatcg tctaatgaaa cgaatgtcat catcagtatc
109381 gacgaagacc ttgatgctca ttaagttacg taagttttct tcaccaagga cgagaatacc
109441 ttctaggata ataatatcag ccggttcggt gtgaatagtc ttttcacttc tagtatgagc
109501 agtaaaatca taaactggca tttcaactgc cttatagtcc attaattgac gaagttggtc
109561 aacaagtagt ggcatgtcaa aggcatcggg atgatcgtag ttaatcttct tacgctctga
109621 cattgataag tcatcgttat ttttgtaata agaatcttgc gtcataatta aaatatggtc
109681 atcttgttgt aattggtcat aaatttcatg ggcaatggta gttttaccac tacctgaacc
109741 accggcaatt ccaatgataa tagggcgttt actttgcacg attaaaatct cctttttaa

109801 actattacaa taataccta aaattacgct ctgtgataga tttacaggca ataatattga
109861 caaatatgtg ttttttctga acatgtgaag taaaataaaa ttgaaagtgt tatatatggc
109921 tttaaaaaga aggttttact ttgactgctc aagaaaaagc cgcaatcaag aattggaatc
109981 cccgatatcg gagaaatgtt tatttatatc tatggattta cggaatttta ggtgcggtaa
110041 ctggaattac taatgatgca gctttgtcat actttgatat agtagcacca ggattaattt
110101 caggtctaaa tatctttaat gcgattactg cattattaat gtcgttgatg attgtctggg
110161 tgcatgaatt aggttatcgt aaaattttgt tgattttgcc gcctttaact tctattttcc
110221 tggcgttgac tactattaca actaatcaaa taattattat gtttgcctac attatttcat
110281 ggactgcaat tggagtttat gatttaatgt atcctttgat gtggacttct tatgttccta
110341 aagaaatcag aactaagatg tttacagtag taatggttgt aaacttagtt gctcaaacta
110401 tcttaacttt tattggtgga aaagcggtag tgtacttctt tagtaagatg cagggtattt
110461 cttatgatat tgcttctaat ctatctgctc ataccgccca attaagtggg agttatctag
110521 ctaattatac caatgccttt agaatagtat tgattttgac tgctttagtt aatgtagttg
110581 cttttgtatt agcaatcttt attaaagatg agccaaagga ttatcgaacc gtggggatga
110641 agaaaccgca aacgccagaa gagaagaaaa aagcttttga agctttgatt aataaaaaga
110701 caattatgtg gatcttgtat attgcaggta ttcaactagg tgctagattg gttgttcctt
110761 atattccaat ttatttgaat gattatctgc atattccccg gggaattact tcaacaatta
110821 atacatttca gaccgcagca atgtttattg gatatttctt tgctccattc ttagccaaga
110881 agttaggtgc cattgtttct attgctgctg gaaccttaac ttgtgcacca ttaatgtttt
110941 taatggctaa cggtcgacac ttcggcactg gaattactct ttttgtaatt gtaggggttt
111001 tgctattctt acgtagtggt ttagcaaatg caacaatgcc gattcagcaa gaagtacaga
111061 tgattattgt tgataaagat atgcgtccag cctttactgc tgtggttcag attgcatatg
111121 cggtaattgg aataattgat ggattattta cagcctttta cttacttaag acgccagctg
111181 ggtatgcgaa tgcatactac attgcgactg ctctatatgt agtagtaagt atcttattaa
111241 ttgtaatgtt tgctaagcaa tataactgga tcttagatga gaaaaagagc acatctaatg
111301 aaaaatagtt aattgataac ttaagaataa taataaaata ccgaattagt cgtaaaagtg
111361 actgattcgg tatttttggt attttcgagg gatgaaagat ggtaatattt aagacataaa

111421 aataatattt attatttaag gagaagaata atgaaagaaa tcagaaaaga aaatattgtt
111481 aaaactacta ctttgagtga agaagaaatg attgaagttg ttggtggtac tagagtaaat
111541 ggttttgtta atggaaattt tagtaaaggt atattttact ggacgcgcaa catttggaaa
111601 taaaaatgtt ggtagtttat agctttttaa tgcttatttc tggaataact gatttatgga
111661 tattttttag agtttctcaa attgcagcta gtaaaaaaaa tattttgatt ctcggtggag
111721 tgttattctt actttcaatt ttatgtggag gagttgttga actagccact tttttggaaa
111781 ttttattttt catattttat tttagaaaga gtaaagaaaa aaatttggct attggggcaa
111841 ttttaattgt aggagttgta gattccataa tagatatact aagtagtatc ttaatggagc
111901 ttttgcatat tgaggatata atttatctta atttattagt tcaaattagt attatacttc
111961 ttgcagtttt tataattgag atcttctata agtatcttca ctcatattta atgaatgaaa
112021 atcacaatgt gtttatagga ctgttgatct atctatatgt ttccactact ttaacaatga
112081 ttttttatgg acaaacccag aaaaatgcat cgttaagtat gtttttttaca attttttgtat
112141 taattcaaat agtttttgct atggcgactt acgtggaact aataaatatt caaaagcatc
112201 ttttaaaaaa atcacaacaa gataaattaa taaaagagca aaaacaatta caagactatg
112261 cgcaatatct tgaagagagc gaagatgagt tacgtgcttt tcggcatgat tatcgaaata
112321 tgtttaactc attaaaaatc agtgcacaag aaggaaatac taaagaagta atccaaaagc
112381 ttgatgaata tactaaggcc aatttaaatg caaaagcatt tgaaaaatat agagatgtta
112441 atcatataaa aataaagtct ctgaaaagca tcattattgc taagttaacg gaaatgtata
112501 gagaacatat tccatataat tttgaatgta gcggtgaaat tactgaatta cctaaaaata
112561 ttaatgaaat agatttagtc agaattgtcg gtataagttg tgacaatgcg attgaagaaa
112621 gcaaagtatt aatacagcag aatcaagaag ctcatattga aattatggct tattcaaacg
112681 aacgtggtga atttgaatat gaaatacaaa ataaacgaag tgattctgaa atttcaatta
112741 aacaaataca acaaagtggt tattcaacca agaaaagtca ctctggttta ggtttagcca
112801 atattaatag tattaaaaat aaatatgaaa atatgactgt ttcttacgaa gtgcctgaag
112861 gatactttga tttctatctc gtgattgaac cagaggagga aagtgaagaa tgaaatattc
112921 cgtaatcgtt tgtgatgatg acgaagtatt agcaaaaaat ttagcgaaaa atattaaata
112981 tgcagttagt aactttaccg atgataatac agtctacgaa aatattgaaa tcaatttaga

113041 gctagttgct actacatttg agcaagttgt aagttatata gtcgctaatg atattcaaaa
113101 tgcaatttat ttcttagata ttgaattaag tcaaaattca aaagctaaaa acggagtaga
113161 cttagcagaa tttattaaaa aacaagatcc taatgcacaa attattttg ttactgctta
113221 tgataaatat gctcctttaa cttatcgtag aagaattggt gctattgatt atattaataa
113281 gaccttagat tcaagagaga tgatgcagcg attagaagaa acattgactg gcgccgttca
113341 aagtatcaat aatcttatga aatcaggaag aaaagaactt atctataaaa atggacggag
113401 agttaataaa gttgaagaca cgactattta ttatcttgaa aatagtactt cacaacacaa
113461 agttaatttg ataactgaaa ctggtgtagc tgaattcaga agtaatatct ccaaaattaa
113521 cgaagaaaat gattttttag ttaaggtatc tcaatcttgt gtagtaaatc caaataatat
113581 caacagcatc gacttttcaa aaaagacaat aaagtttcct aatggagatg aagtagtgtt
113641 cgctcgtagt tgcagaaaaa ctgttgagga tctattaaat aaatatccag aaattaatgt
113701 taagtaaaag gtaagactta tgttattcaa atataaatcc gtatacgtgc cacaagtgga
113761 tgagatggat tgcggcgttg cttgtctagc aatgatctta aagcaatatc attctcgcgt
113821 atctttagca catttacgtc atgaagctcg tactaatctt gaaggcacaa ctgctttagg
113881 actagtgaag acagcgcaaa aatttaattt caaaacagaa gccgtaaaag cagatatgtc
113941 tttatttgat gagaatagta ttcaatatcc ttttattgcc cacgtcctaa aacaaggaga
114001 actacttcat tattacgtag ttcttaaaaa tgctaagaat tatttagtaa ttgcagatcc
114061 tgatccatca gttggtgtaa taaagatgcc taaagatagg ttcgctcaag aatggacagg
114121 aattgcactc tttatggttc ctaacgaaga ctttgaacca atcaaagaaa agaaaaataa
114181 tttatggtct ctctttccat atatgtttaa acaaaagcgg ctgatgatta acatcatttt
114241 agccgcttta ttaatgacca taattagtat ctgtagttca tattttgttc aaggaataat
114301 tgatacttac attccagatg gaacctatca gactctttcg atcttagctg ttggactatt
114361 aattgcttat gtctttaatt caatttttc ttatggacag aatttttat taaatatttt
114421 aggtcaaaga ttaagtattg atctaaatct gcgatacatc aggcatattt ttgaattacc
114481 aatggaattc tttgtaacaa gaagaacggg tgaaattact tcgcgttttt ctgatgcaag
114541 tagaattatt gacgctttgg caagtacagt tatttcgctc ttcttagacc tctcaattgt
114601 gattgtgatg ggattagtct tagcagcaca aaatatgaca ttatttggga ttacactgtt

114661 agctttgcct atttatgcag ttgtaattct aggttttact aaaaagtttg aaaaactaaa
114721 taacgaacaa atggaaagta atgctgtttt gagttcttca gttattgaag atattcaagg
114781 aattgaaact attaaagctt taaatagtga gaatacaaga tatagaagaa tcgatagtca
114841 gtttgtagat tacttaaaga agtcatttaa atatagtaag actgagagtt tacagacagc
114901 actcaagaca tttattcaat tatctcttaa tgtaattgtt ctttgggttg gtgcaaagat
114961 tgtaatgcaa ggacaattga gcattggtca gttaatgacg ttcaatgcat tgctggcata
115021 tttcattgat cccttgcaaa gtattattaa tttacaaccg cgacttcaat cagctagtgt
115081 tgctcaaaat cgattaaatg aggtctatca agtaaaaagt gaattcaatc agaaggctac
115141 tattgaagat cgtaagctcc tagaaggaaa tattgaatat aagaatgtgg attatagcta
115201 tggttatggc acagatgtct taaaagatat taatctcaaa attagccagg gcgaaaaatt
115261 aacgattgtt ggaatgagtg gttctggaaa atctacgatg gttaagttgt tagtagattt
115321 cttttcgcca agtaaaggtc aggttacatt aaatggacat gcaacaagtg agattgataa
115381 gcatacgttg cggtcatatg taaattatgt acctcaaact ccatatatat tttctggaac
115441 agttaaagag aatttgttgt taggttgtag gccagatatc actgaagaag atgtaataaa
115501 ggcttgtcaa attgctgaga ttgaccaaga aatagctaat ctacctttgc aatttgaaac
115561 aaaattagat gaaaatgcca aattttatc tggtggacaa aaacaaaggt taactattgc
115621 ccgggcatta ctatctcctg caaaggtatt tatttttgat gaagtaacaa gtggcttaga
115681 tacaattact gagaagaaag tggttgataa tttgatgaag ctaaaggata agacaattat
115741 ctttattgcg catcgactag caattgcaga acgggcagac aaggtagttg taattgatca
115801 tggtcaaata gttgaagaag gcagccacag cgaattgatg agtaagcatg gtttttacta
115861 tgacttagtg aagggatagg tgagtacaga tggatgcaaa agagtatgaa agtacagaat
115921 tctattccta taagttcaag aatttctcga caatgattat tattccagcc gctatttttg
115981 ttctactttt atttattgga tcttttttg ctgttagaca gagtacagtc tcttcggttg
116041 gcgttgttga accaacggtt gtaattaatc agaaaaatgt tagctatgat gagggacaag
116101 tcgttacaaa acatggtcaa aaatgggtag ctcatgttga tcaagatagt gggattagtt
116161 taatgcctgt tatgaaagct aaaggaaaag tgaggattgt tacctatgtg cccactaata
116221 aaatctcgtc tattaagaag ggtcagaagc taaacttttc tgtacctaca ggagagggct

116281 tgactagtag attaactggc aaggtagaga aaattggtgt ttatccaata aatgtaaaca
116341 agcagagtat gtatgaagtt atttcgtttg ccaaagttag tgatataaat gttaagtacg
116401 gaatgcaagg taatgccaca atagtgacag gtcgcagcac atatcgcagc acatatttta
116461 aatatttttt agataaggta ttgaataaaa gataaatttg ataaaataaa gcgatattaa
116521 attatattat gtgaggataa aaatatgaac gaaactaaat taaaagagat gttaactaaa
116581 ataaaagcaa attctgatat tgcaattgat ccagaagctc aaaaattgat tgaagcagct
116641 attgaaagaa tagataagaa agttgattta caacgagtaa taagtaagct aaaacaagat
116701 ttggatattt attctgtatc acaccaattt aaatttccaa aatctttagt tgaattacaa
116761 cttatggtaa ataaataccc agataaatgg gaaggtgccg gtgcaggcgg agtttggcca
116821 gtaatttaaa gtgacttttt aaatataagg attaattcgg ttagattatg caaaattaga
116881 tgcatacttt acaattttgc tccattttag catttataaa gtattttttg ataaaacatt
116941 aattaaatga tcaatctagg aagtaagtta caaaaatgag cgaaaataat tttaaggaac
117001 tattgactaa aataaaagca aactctgaca ttacaactga tctagaaaca caaaaaataa
117061 ttgaagcagc aattgaaaga atagataaga aagttgattt acagaaagta atatttaaac
117121 taaagcagga tattagcatg tatttcgtag cacatggttt taaattgcca gaaactttaa
117181 ctaaattgca gcttttatta gataaggatc caaataagtg ggctggcgct ggagaaacaa
117241 tgtcgcttac taatttttaa aaattaattt aatatagata tgaaagatta caagctatag
117301 tactattttg agaactttta gcgttgagat ttaattggta gaaagaaatg ttacccaata
117361 agaaaaagaa aatcaggtca atattaggat tatcattttt atttttactc gtagttttaa
117421 ttaatatatg gaaacaagaa ttaaatattc ttgagttagt tcttctaatt attggaatta
117481 ttatttgggt tagtgctatt acttggcgaa taatcgtcat tgtaaaaagt taacttttgc
117541 attctcaaaa aatcaaattt ggcttatgta gctagaatgt gtactacctt aaggaaccta
117601 attagtcgta aacaggacca gttcggttct ttttttattt ttactggctt taaaatgcta
117661 aaatttaaac aattagtaga aggattgagt taaagggtga aagaaatgaa agtaattgaa
117721 gaaaatgaaa aggaagcttc aagaagagaa agatggataa ttttaggcgt aagtctagtt
117781 tttggcgatt tagtgaacaa gttgttcttg cgtctaactt caattgatag ttttgtttta
117841 tcaatgataa ttggactagg aagtatgtac gtgtttgaat ccggatacta ttacttcaga

117901 aacgatattc aaaaaatgat taaaaaagtt agaggataat ttaggaggtt aagtgcagga
117961 ttaggtctgt ccgtaggtgc atttttagca acctatgttg gtatagccat ctatatgatt
118021 gatagtttat ggaatcagtg gtaaaaacta ttgtttttag tgttggatta atattttttg
118081 ctttaattaa atttactatt aagcaggatg aaaattcgaa aagattagtt ttacaaatga
118141 gcttagtagg atgcttttta attctttata atgcaatatg gagttatttt attagatagt
118201 ttgctagaaa gctactaaat aaataccaaa tttgttacga acaagaccga ataggtcttt
118261 tttatttttg agaactttct aaatgataga attggttcat agcaaagatt ttggaggcga
118321 atagaatgcc agtaggatac attccaatta ttttactttt attaatagtt ttagttgata
118381 tcgttgcttc gctaaaatta cataagctag taaaaacgtc tgatgtttca aatatcttaa
118441 tacttggcat aacagcaatg gttatttttg ctttgaaaga tttaagttca ttttcttgga
118501 gttcagcaat agcaactgtt ttcatgagca ttgttgtttt gaaagatttt tatgatgaat
118561 atcaatcaca agtaaaagat ggtaaagatg aaaagacaac gaaataatag acagttaatc
118621 attattagta ctctggttgg agcaagtatt tttactcttg aaactatcat agacctatta
118681 gtagggagta aattaactat ttgtataatt cttggaaaca ttctttgggg gatgattttg
118741 ggatatttaa tgttcaaact tgtgaagcta aggcggatga gaaagaagaa tatacatgaa
118801 attgagtaaa attagtcaaa gtaaagatac aaatgcagaa gaatggacga taattacgct
118861 aagtgtgatc ttcgcctttt gcatgaataa gctgttttta tatgtaacaa atagtgatag
118921 tatcatgcta agctttttttg tcggaattat aagtatgagt gtgttttcga tcacgtatta
118981 ttatggcaaa gataggatcc ggcaacgttt aaagagtaag aaaaatatcc atgagtaaaa
119041 aagaaaaagt tattgggttt agaatcttat cttggacaat tacatcagtt tgtttaattg
119101 tatttctagg cgatatttac agttatggat tttcttggca aagttttaat caggtagtag
119161 ctttaatctt agttgtttta ttgctaattt cttctattat atttacgtct acgaagaatt
119221 tattagagtg gaatcaacag tataaactgg ctcagtggat cggtgagcta gttattttac
119281 ccgctttttt tctaggtaat ttatataatt ctacacatgt ctggtattcg atattatttt
119341 tatctttctt tggaggagga agcattttat tcatgcttgt tcaatatttt cgaagaagat
119401 cgcgaaataa gtgcaaaaca aaattacgat aaaataccaa atcagtaaat gaatacgcaa
119461 aattggtatc tttttgaatt tcacaaaaaa tgatagtaaa attaatgaat aaatgataat

119521 atttggaaac aaaactacta tttggagggc agaaatatga aaaatgaatt aaaacaagtg
119581 ttgaatgact ttatctcact tgctaagact cattatgata ggaaaggcaa tgaatactta
119641 ctagaacaac ttgaagttgc tttagacaag ttagaacata atgttcaaga tgaagtagat
119701 gaagcccgtg caacttatca aaatataaat actatttgtc ttactaacca tcttcgcttg
119761 gaaacagatg aagaagcatt acttgaaaag attaaaaaaa tttcaatgag taaaggctgg
119821 cttggtggct taaacagttg gaatacaact aatacttggc ctggaagata agaaaaattt
119881 aacaaagaat gaagagtagt cgaaggctac tctttttta atatattaac tttatgtgca
119941 tattttttaa aagtgaacta aaatggatct gtaaaaagga atagaagtga taactttgac
120001 taaaaatgat gaaaacgatg agataattaa ggttgctcaa ttatctgttg aagatgcact
120061 gaaggaatta aatactagtc aaagcggctt aacaattgat gaagttcata cattgcagca
120121 aaaatatgga aagaatattt ttgccaaagc aaaacaggag ccactttgga agaaattttt
120181 agctaatttt actaatttaa tggcgattct gctttgggta gcaggtatta ttgctttttg
120241 tgctgattta gtgcaattag gaattgctat ttgggcagta aatatcatta atggtatttt
120301 tagtttttgg caagaatttc aagctgataa ggcaactgag gccttagcga atatgcttcc
120361 ctcttatact cgtgttataa gaaatggtaa ggaagagaag attttagcta aagacttagt
120421 tcctggcgat atcgttaagc ttgaagaggg agacgatatt ccagctgata ttcgtgtaat
120481 tgcagctaca acggctcaag cagatcagtc ctcactgact ggtgaagtga acccagttca
120541 taaaggggca catgctgttg aagacgtcaa gaagaaaaat catgctgatc ttaataacat
120601 gatttttct ggtactaata tgatgaaggg caacattacc ggtgttgtgg ttaaaacagg
120661 tatgaatact gatttcggta agattgctga attaactcaa aacgttgctc aacaaaagag
120721 tccgcttgaa aaggaactag atactttaac taagcagatt tcaattttgg caatttcaat
120781 tggaattatt ttcttcctaa ttgctacttt ctttgttcac tatcctttag taaagggctt
120841 tgtttttgcc ctgggaatga ttgtagcctt tattccagaa ggattagagc caacagttac
120901 tttatcttta gctggtgcgg ttcaacgaat ggcaaaaaag cacgctctaa ttaagcgttt
120961 atctagcgta gaaacgttag gttcaacatc agtgatttgt tctgataaaa caggaacttt
121021 aactaagaat gaaatgacag ttaaggaact ttggacacta gaaaagagtt atcatgtgtc
121081 aggtgaaggc tatgctgttc gaggccatat taaagaagga ccgaagcatg tttttgctaa

121141 agataacgat actttaaaag aagtattact tggtggcgtt tttgcagaca acgcaagaat
121201 tcaagcacca gataaaaagc atgtccgtta tcaaatcttg ggcgatccaa ctgaagcatg
121261 ccttgaagtg gttgctcgaa aaggtaaagt tgatgttgag gctgaattaa aaaatacgcc
121321 gcgagttaaa gaactaccat ttgattcaag cagaaaaatg atgacggtaa ttcaaagctc
121381 tgatggcaca caccgtttta atacctatac taagggtgct ccaaattgtg tggttgataa
121441 gtgtacttct tatttagaac acggtgagat aaaacctatt acccaggaaa taaaagataa
121501 gattatgcga gctaatgatg gctatgcgaa ggatggactt cgtgttttag cagttgcggg
121561 acgtaacctt gatcaaaaga tgatgaacaa tttagactcg gcaacgattg agacagttga
121621 agaaaatctt actttcttag gtttaacagt catgatggat ccaccaagag cagaagtata
121681 taaagctgct cgtgagtgtc gcaaagctgg aattaaggta acgatggtta caggtgatta
121741 tggcttaact gctaaaagta ttgcgcgcca gattggtttg accgatccag ataagccttt
121801 aactgtaatt acaggggatg ctttaaagac aatgccagat gatgaattaa gacattatct
121861 tgaaggtgag gttgtctttg ctagaatggc acctgagcaa aaatatcgtg tcgtttctat
121921 gtatgaaaaa atgggcaaga ttgttgctgc taccggtgac ggtgttaatg atgctccagc
121981 cttaaagaaa gccaatattg gaattgccat gggcggtact ggtactgatg ttgcaaaaga
122041 agcagctgat atgattttaa ctgatgataa ctttgcttct atcgttggtg caattaaaga
122101 aggacgcggt gtgtacagta atattcgtaa gttcttaatt tatattttga actcaaacat
122161 gcctgaagca gttccttcag tcttgttctt gctttcaggt ggtgcaattc cgcttgcttt
122221 aactgtaatg gaaattctat ttattgacct aggaacagat atgattccag ccctcggatt
122281 aggacgggaa gatcctgaaa aaggcatcat ggatcgacct ccaaggtcac ctaaagatca
122341 cttaattaat aagcatgttt tagctaaagc ttttttatgg tatggcttaa ttgcttcaat
122401 tattgcaacg gcagcattct ttggcgctaa tttctatcgt ggtcatattt ttccaaattt
122461 gccagtggta ggttgggatt atcgtcaagc aacgacagta actttggctg caattatctt
122521 ttgtcagatt gcggcagtat taaatattcg ttattcaaga caatcaatgt ttaataggca
122581 tttcttcaag aattccatga tctttatcgg aattattatg gagatagttt tgctgctatg
122641 catttcttat gtgccagttt tccagtcatt ctttggtaca gagccactta atgcacatga
122701 ttggattatg ctagtatgta ttccaattcc tctaatttta attgatgaat taagaaagtg

122761 gattctcagg aaaaattata aaaagtaaat aaactttaac tgctagtttt aactagcagt
122821 tttttcttgc taaaatatta aaaataaagg tggcgaaagt atgaaaaaag gattggtaat
122881 ggaaggcgga gcgatgcgct gcatgtttac cgctggtgtt attgacactt taatgaaaga
122941 aaaaattaag tttgatggag caattggtgt gtcagctggg gctgcatttg gctgtaatta
123001 taaatctgaa caaataggaa gagttttacg ttataacatt aaatatgctc atgatcctcg
123061 cttatcaagt tggcagtcgt ggctaaagac aggagattta tatggcgctg atttttgcta
123121 ccgagagcta ccagttaaat tagatatctt tgatacaaat actttcaaa aaaatccaat
123181 ggatttttgg tgtgtggcaa ctgacattga taatggagaa gctgtatatc acaagttgtt
123241 gtcaggtaaa gaggctgatt taaaatggat tcgtgcatct tcttctattc cagtttttgc
123301 aaggccagta gagataggta ctcgtaaata ttgggatggg ggaattagtg attcaatccc
123361 gattaaattt tttgaaaaaa ttggctatca gaagaatgtt gtaattttaa cgcagccact
123421 atcttaccga aaagaagcaa gtaaactata tccagcgtta gagttagtat tgcacaagta
123481 tcctgctgtc ttaaaaaaat tgaaggctag ggcaacagat tataatgatg tccttagtta
123541 cattagaaaa aaagaagtac aaggcaaaat gttagttatt aggcctcctt atcctttaga
123601 tattggtacg atggaaaaag atcctaagga attgaaacga gtatacaata ttggcgtaaa
123661 agaagctcaa aagtgtctaa aagcagttca atcttattta aaagaataat atttacttgt
123721 cttcattgcg tttcattcgc tataattaac aaaagcttaa aatttcagtt tttaattttt
123781 aatagtaata atatagaggg aatgagcgta atgaagaaaa caaaactatt tagttttttc
123841 tttgcatttt tactcttctt ctcactcggt gccactttta ttcaaccagc tcaagcagct
123901 agtgaaaaga aagtagcaga aggtagtgtt ttaaagaaga ttaaaaagag tggagagcta
123961 gtagtaggta catccgccga ctatccgcca ttagaattta cagcaagtga aaatggcaag
124021 actaagtatg ttggagtcga tattgaatta gctaaagata ttgccaaaga tttaaacgta
124081 aagcttgtta ttaaaaatat gtcttttgat tcgctgttag ttgcacttga aacaggaaaa
124141 gtagacatgg ttatttctgc catgacgcct actcctgaga gaaaacaaag tgtggctttt
124201 tcaaaaattt actacaagcc aagtggtgaa tattttttag taaacaaaaa agataaggat
124261 aagtatacaa gcatccaaag ccttaaaggc caaactattg gtgctcaaac aggaagtaac
124321 caatataatt tggttaagtc gcaaatgaaa gattctaagt taaaaggtct tgggaaaatt

124381 aataacttgg tcttagcttt acaatctggt aaggtttcgg cagttgtagt tgaagaatta
124441 atcgctaaag cttatgcgga aaataataat agcttagccg cagtacgttc aaacctgaaa
124501 gaaacacagt ctggaaatgc agtagcaatt gcaaagggac aagatgaatt agttgctcag
124561 gtgaataaaa ctattgatca tgttcaaaag aaagacttaa ttaataaaca ataccttcca
124621 gcagctgcca aatatatgaa aacggaaaag aaaagtaata cttttgctaa atatattcca
124681 tacttcttca agggcatctg gtacacgatt gtaattactg tcttttctgt cattattggt
124741 attgttttag gaatcatttt agccttgatg cgcctgtcta agaatccaat tttacattgg
124801 cttgctgttt gttatattga atttattcgc ggtacgcctc aaatggttca aatcttgttt
124861 gtatactttg gaattggata tttgatttct aatctttcag ccttggttgc tggtattatt
124921 gccattgggc ttaattctgg agcctatgtg gctgaagata ttaggtcagg aattgattct
124981 ttgccaaaag gacaaatgga agcagcacgt tccctaggtt taagccgtca gaaggcattt
125041 agatatgtaa ttattcctca ggcaataaag aatatttggc cagccttagg taatgagttt
125101 attactttat taaaggacag ttctttggtt tcagtaattg gcgttgctga attaatgtac
125161 caaacacaat tagttcagac ttcaacttat aagggtgtat tgccattatt cattgccatg
125221 attatttact ttattatgac tttctcattg acgagattgt tgaatcactt tgaaaagagg
125281 atgaagcgta atgacagcaa tgattaaggt tgaacattta aagaaaagtt ttggaaaaaa
125341 agaagtcctc aaagatattt cagcaaatgt tgataaagga aaagttatca gtatcattgg
125401 cccatcaggt tcaggaaaaa gtacattttt acgctgtctt aatgtacttg aaaagccaag
125461 tagtggtaag atcgtttttg atggacaaga tttaacgcat attaatgaaa aagagttaga
125521 tgttttacgt gaaaaaatgg gaatggtttt tcaaagtttc aatctttttc ctaatatgaa
125581 tgtagttgaa aatatcaaac ttgctccaat gaaggttaag ggcgtaagtg aagatgaggc
125641 tgagaaacaa gctttagaat tactggctaa agttggtttg aaagatcggg ctgagcaata
125701 tccttcgagt ctctctggtg gtcagcagca gcgtgttgcc attgctcggg cgttagcgat
125761 ggatccagag gtaatgctgt ttgatgagcc aacaagtgca ttagacccag agatggtcgg
125821 agaagtttta aagacgatgc aggatttagc tgattcggga atgacgatgg ttatcgttac
125881 tcacgaaatg ggtttgctcc gtgaagtttc tgatgaagtt tggtttatgg cggatggata
125941 tttacaggaa caaggcagtc ctgagcaaat tttgaaaat ccgcaaagcc cacgtgcaca

126001 agatttcctt tctaaagttt tataaatatt aatatttctt ttaaataagc
actttctttt
126061 tacaaggaga gtgcttttct ttataatggt ttcttgataa aaaagttgga
aagggatgag
126121 tttaatgaag attagacaag ctacaataaa tgactatgat caaatcatga
caattttgaa
126181 ggatggagca aaccaactag cagagcgcgg agttgatcaa tggcaaggag
actatccttc
126241 tccagatcaa attaaagatg atattgaaaa aggctttgct tatcttgctg
tttcagctga
126301 tggcgaaact gttggagcca tttcaattgt tgaagctcca gatcattcct
acgataattt
126361 aaagggtaaa tggcttctta atacagacaa gtatgtggta atacaccgtg
tcgcaattca
126421 ttcaaagcat gcgggcaatg gttatgctac taaattgttg actgaggtaa
ttgattatat
126481 tcgtgataat cgtaaagata ttgatagtat tcgtattgat actcatgaaa
ataatactgc
126541 aatgcagcat ttaattgata aaatgagctt tactaaagta ggagaactac
atggcgttta
126601 tcgtcccgat gaaatttcat atgtttatga aaatgtcaga gaatagataa
ctttttggct
126661 aggttgaaaa acctagcctt ttgtttaatt tagtcgttct gttgacaaag
aatattatga
126721 taaaatattc ttgttaaata ctgtaaatta catggagaaa ttgatgagaa
caaattttaa
126781 atttaaaata gctgctgttt taggactgct agctatttta atcggactta
caggctgtgc
126841 taacaataat agcaataaga ttactgttgt tggatctagt gccatgcagc
ttttggctga
126901 gcaagctgga aatgactatc gtctttctca tccagacagt aatattgttg
ttcaaggtgg
126961 aggttctggg actggtctta gccaagtaca ggctggtgct gttgaaatcg
gaacttctga
127021 tgtttttgct gaaacacaaa aaggaattga cgcccaaaaa ttgcaaaatc
atctagttgc
127081 agttgtaggt attgtaccaa ttgttaataa gagcgctggc gtgaaaaatt
taaccagaca
127141 gcaattgagt gatattttta ctggtaagat taccaactgg aaacaagttg
gcggaaaaaa
127201 tcaaaatatc actgtaatta atcgttctaa aggcagcggt actcgaggaa
cttttgaagg
127261 cttaattctg aatggaaaaa agccaattca agcgcaagaa caagattcta
acggtactgt
127321 gcgtaagatc gtcagctcta cgccagggac gatttcttat atttctttcc
catatgctaa
127381 tgatgaaaat attcaaaaac tgagtattga caaaataaag cctacgaata
aaaatgtgga
127441 aacaaatcgt tggcatcttt ggtcttacga gcatatgtat actaagggaa
agcctaataa
127501 aaatgttcaa aagtttattg attatatgct tggtaggaaa gttcaaaatg
acttagtgcc
127561 aaagctaggc tacattagta tcaataagat gcaagttgaa cgtgatagta
ataatcatgt

127621 tgttcaaaaa taaagaatag gacgttttga tgaataacaa agatctaaaa aaagtagttg
127681 aatctgcttt agataagcaa aaggttcctc atgttaaatt aaagaagatt ggagctagca
127741 aggttgatgt tgctagttta actgaaccct caaaagaaac gcgacaagaa tattggggta
127801 aggggttaac ttactgtgct attatcttaa ttattatttt agttgcttca attattggtt
127861 ttattggctt ccatggctta gaaacattta ctaaagacca cgtaaatgta tttcaatttt
127921 tggcttctag tgactgggat ccaggtgaag gaaagagcca tgttggtgca gctgcaatga
127981 ttgtaacttc tttttcagta actttattag cagctttagt agcaacacca tttgccattg
128041 cagtagcctt gtttatgact gaatattctt ctaaaaaagg cgcgcgcttt ttacaatccg
128101 taattgaatt attagttggg attccatctg ttgtttacgg atttttgggg ttaacaatta
128161 ttgttccatt tattagaaat atttttggtg gcacaggatt tggtattttg tctgctactt
128221 tggttttatt tgtcatggtc ttaccaacga ttacttcttt gactgttgac agtttaaagg
128281 ctgttccttc tgactaccga aaggcttctt tggctttggg agcaactaag tggcaaacaa
128341 tttataaggt tattttgcga gttgcttctc ctagaattat gactgcggta attttcggaa
128401 tggcacgtgc ttttggggaa gcattagccg tacaaatggt tattggtaat gcggtcttaa
128461 tgccagctaa cttagtaagt ccatcagcta ccttaactag tcaattaact agtcaaatgg
128521 gaaataccgt gatgggaact ttgcctaata atgccctttg gtccttagcc ttgttattat
128581 taattatgtc tttagtcttt aacttcttag tccgcttaat tggaaagaga ggacagaaat
128641 aatgaatgca aaaacgcgta ataaaattgc tacggctggt atttatacct tggtgagtat
128701 tgtcgtaatt attctttttg gaattttagg cgatatttta gtttctggtg taccacatct
128761 ttcatggcat tttttatctt ctgaagcctc ttcttatcaa gctggtggtg gagtaagaga
128821 tcagcttttt aattctttat atttattagt cttgacactg attatttctt tgccaattgc
128881 attaggggct gggatttatt tagcagaata tgctaaggat aattggttta caaacttaat
128941 tagaacaacc attgaaatcc tgagttcttt accatccatt gttgtaggtt tatttggtta
129001 cttattattt gtagtgcaat ttggttttgg cttttctatt atttctggtg cattagcact
129061 aacatttttt aatctaccaa ctttgactag taatattgaa caagctattg aaggtgttcc
129121 acaagcccaa agagatgctg gacttgcttt aggcttatct aactggaaga caattcgggg
129181 aattgtttta cctgcagctt tacctggaat tttaacaggt attattttaa gtgctggtag

129241 aatttttggt gaagcagcag ctttaattta tactgcaggt caaagtggat caacaattga
129301 ctatagtaat tggaatccgc ttagtccaac tagtttctta aatgttatgc gcccagcaga
129361 aactttagct gttcatattt ggaaagttaa taccgaagga atcattcctg atgcaacaat
129421 tgtttcagct gcaacttctg ctttattaat cattgtggtt attttgttta acctgggtgc
129481 acgtgcctta ggtaaccatt tataccgcaa gttgacagct gctaagtcat aaggagaaat
129541 ttatgcaaaa tgtaaatgaa gcacctactt ttattcatca attcgatcaa gatgaacaaa
129601 ttatttctac taaggatctt agtgtttttt acggtgggag tattcaaaag ctttttggag
129661 ctagtctgca atttaagaaa aaaacaatta ctgccttaat tggtggatct ggatcaggaa
129721 agtcgacttt tttacgttgc cttaatagaa tgaatgataa ggttgctcga gttgatggtg
129781 aaatttggta tcacggctta gatattaata aaaacaatat caatgtttac caattaagaa
129841 aaaatattgg catggtcttc caaaagccaa atccatttcc aaaatcaatt agggaaaata
129901 ttacgtatgc tctaaaagca aatggcgaaa aagataagca aaagctagat caaattgtcg
129961 aagaaagttt aagagcagct gctttgtggg atgaagtgaa ggataagcta gataagagtg
130021 ccttagcaat gtcaggtgga caacagcaac gtttatgtat tgctcgagcg cttgccttga
130081 aacctgaaat tctgcttctt gatgaaccag ctagtgcgct tgatcctgtt tctacctcta
130141 agcttgaaga tacgctcaag caattgagaa ccgattatac gatgattatg gttactcaca
130201 atatgcagca agctagtcga attagtgact acacagcttt ctttcattta ggacatgttc
130261 tagaatacga taaaacagaa aatattttca ctaatcctaa gggtgaaatt acggaagatt
130321 atattcgcgg aagttttgga taaggggttt actgtggaaa atattattac aagtagagat
130381 gtgcatctaa gttatggaaa tgttgaagcc ttgcatggga ttagcttgga ttttgaagaa
130441 aaagaattaa ctgctttaat tggaccttcg ggatgtggaa agtcaacttt cttgcgctgc
130501 ttaaatagga tgaacgatga tattcccaac attcatatta gcggtgatat tcagttcgaa
130561 ggacaaaaca tttatggttc taagatggat ctggtagaat tgcgtaagga agttgggatg
130621 gtttttcagc agccgagtcc ttttccattt tctgtttatg acaatattgc ttacggatta
130681 aagattgctg gaattaagga taaagagtta attgatcagc gcgttgaaga aagtcttaag
130741 caggcagcaa tttggaaaga aacaaaagat aatttagatc gcaatgccca agccttttct
130801 ggtggtcagc agcaaagaat ttgtattgcg cgtgctttag cagttcgtcc gaaagtagtt

130861 ttgcttgatg aacccacgag tgcgcttgat ccaatttcaa gtagtgaaat
tgaggaaacc
130921 ttacttgaat taaagcatga atatactttc atcatggtaa cgcataactt
gcagcaggcc
130981 agtcgaatca gcgattatac tgctttctta atgagtgggg acttaattga
atatggtaaa
131041 acagctgata tgttcatgaa tcctaagaaa cagattacaa gtgattatct
gaatggacgt
131101 tttggttaat aaggagaagt tttaagatgc atgaagtttt tttagatgaa
ttacgtaaat
131161 taaataccccg ctttatgggg atggggattg atgttagtga gtcaattgag
gaagctacgc
131221 aagcctttgt tgatcatgat aaaaaattag ctcaaagcct agtaaaagat
gatcaaaaag
131281 tttctcgggc tgctactaaa gttgagaaga gaaccttgaa gttaatggct
cttcagcaac
131341 cggttgctag tgactttaga aatgtaatta gtatcttaaa ggcaacggga
gatttagaac
131401 gaattggcga aaatgcactt tcaattgctt gggagacaat aagagtaaag
ggtaatccac
131461 gtattccaga agttgaaaca attattaagt caatgtctaa gaaagttaac
ttcatgcttg
131521 atcaagtttt aaaagcttat gttcaaggtg atgaaaaatt agctcgtgaa
gttgctaaaa
131581 aagatgacga agtagatgaa gattatgtga aggcgcgtaa gttgattatt
gctgggatta
131641 aacaagatcc agaagctgca gttgcttctt caagttactt tatggtaatt
cgtttactag
131701 aacgtattgg tgaccacgta gttaatttag cacagtgggt tgtttataag
atgtcaggtg
131761 aattagttga cttaaatact aaagatactg atgaaatgac tgaactatag
atgaaatggc
131821 tgaactataa ttgaaaatat ttaaattaga agactgcaat tttgcagtct
tttttgtgta
131881 gataaatgta agcgcgtgca aattttcata aagaaatcta taataaaact
ataaataaaa
131941 ctgtaaatgt gatcggggga agttatgagt aatacagttc aaaaaaagaa
aaagcatcaa
132001 tttccaacag cgtatacggt tattattatc gttctgctat tggtgcagat
tttaacattt
132061 tttattccat cgggtaatta tgcaacgctt gagtatgatc aacctaataa
ggaatttttg
132121 attactaagc caaatggtag taaacataaa gaggcagcaa ctcaaaaaac
tttagataag
132181 tataaagtta agattgacgt gaagaagttt actaatggta ccatttataa
gcctgtggca
132241 attcctgatt cttatgaaaa gatcgatcag aaaaagccag gactaggcgg
agcaatttat
132301 caatttttat cttcacaagt aaacgggatt gcgcaaagta ttgatattat
tgcctttgtt
132361 ttaattcttg gtggatgtat tggtgttgtt cacgcaaatg gtgcaattga
tgccggtatg
132421 caggcacttt ctaaaaagat taaaggtaag caagtattat tgattgtttt
ggtaatgggc

132481 ttgattgcca ttggtggtac aacttttggt ttagctgaag aaaccatggc
cttttatcca
132541 attttgattc cagtattttt actggcagga tttgaccgta tgacggttgt
cgcgacgatc
132601 ttcttaggaa ccagcattgg tacgatggcg tcaactatta acccattttc
aactgtaatt
132661 gcttctaata ctgctggtgt taactttaca gaggcattgc cgttaagaat
ttgcatgtgg
132721 gcaacttgtg ttattgtggg gatgatttat gttattgcat atgctaaaaa
ggtacaaaag
132781 aaccctaagg cttcatatgt ttataatgat ttcgttaagg aagatcaaga
atatctgaat
132841 caagaccaaa caatacaaga gcatgaattt acttggcgcc aaaaattaac
attacttgtt
132901 ttcgcaattg catttattgt aatgatttgg ggcgttcagc aaaagggctg
gtacttcact
132961 gaaattgcag ttgtcttctt agcaactgga tatattttg cctttatttc
aggcttaagt
133021 gagcacaagt ttgttgaaag cttcgtaaat ggtgcaggcg acttattagg
tgtagcttta
133081 acaattggat tagcgcgtgc cgtttcaatt gtgatggaag aaagtcatac
tagcgatacc
133141 attatgaact tctttagcca acaagtttct ggtatgccac cactactgtt
catttggttt
133201 atgttcttgg tctacattgt tttaggattc tttattcaat catcttcagg
tttagcagtt
133261 ctttcaatgc caattatggc accattagct aacgtagttg gtattgaccg
agcaagtatt
133321 attgatgctt ataactgggg acaaggtttt atcagcttgg tagctccaac
tggtttgatt
133381 ttaatgagct taatgatggt gaatatcggc tttaataagt ggtttaagtt
ttgctggaaa
133441 ttattagtaa ttgaatttgg tatttgttta gcatttttag caattggctt
agttgtttac
133501 tgataaggca gataaatagc ttttattaat ttttatagtt atgatttaaa
tataaatatt
133561 gttttattca aggaggcatt tttatggctt atccaaaaac taaaaaaatt
atgaatgaaa
133621 tcgttgctga tttaactcaa gcgcatatgg ttgttcatca acatcattgg
tatatgctcg
133681 gaagaggatt tttgaaatta cacccatatt tagatgatgt aatggatgaa
ttagctgaac
133741 aacaagatgg agttgcagaa agattaattg agattaacgg tagtccaatt
tcaacttatg
133801 aagaagtatt agaagaaact aacgttccag atcaagttgg cagctgggac
ttatccatgg
133861 aagaaagatt tcaattaatt gttaatgcat ataagcaatt acgagatgat
tatgaacgtg
133921 gtattaagat tagtgaagat gaaggcgaca attccactaa tgacttatta
attgcttatc
133981 ataccgccgt tgaaaagaga atctggatga tgtcagctga acttggtaag
agacctggcg
134041 aaggtgaata gattttaaag aataaaaaag gagcgaatga atttttgctc
ctttttatcg

134101 tttattgtat gatttagaaa aattataagt taaatatatt cttttaagtg aaatggaaaa
134161 tttaggtata acaatggatc tatttaacaa ccaacaagct cgagatgctg caattaaagc
134221 catgcatgaa tatcatttag cctataaagc agggcaaagt attgatacaa cagaagttaa
134281 acaatcgctg aaaaagttat ctcaatcaga tagaactttt atgtttatta taggaaatac
134341 aagcaatcca ttagttgcaa ttccgtcgat caattatgat ataaagaaaa acactttaaa
134401 taaattaatt atggcctact tttatttcaa aaaagaaacc gaaaacaaaa tggacaggca
134461 aatattagtg gtgcaggcag ataactttta taaatcagtt gccttaaata aataataaat
134521 gctccttttt gctataatta aaagaaaaag atcgtgcatg aaaggaaaaa ttgatgaata
134581 aagttacaat tgttggatca attaacgttg acaacattat gcatatcaag aaacttcctc
134641 aaccaggaga aacaatagca atgtccgaat tttcaaaggc agctggtgga aagggcgcaa
134701 atcaagcagt tgcaagtagt cgtgccaaga atgaaacaat atttgttggc cgtgttggag
134761 atgatgacaa tggccgcttt atgcttgaac aatttaagga taatggagtt aatgttgatc
134821 atgtaacagt aacgcctaat gaacaaactg gtcaagctta tatcttgctt caagaatctg
134881 gtcaaaattc gattattatt caacatggtg ccaactttga tgtaactgct gaagatgtta
134941 gaaatgccaa ggatcaaatt gaagatagcg attttgtgat tgcacaattt gaaactccac
135001 ttgaagcaac tattgaagcc ttcaagattg ctagagctgc aggtaaaact actattttaa
135061 atcctgctcc tgctcgtact gacattccaa aagaattatt agagttgacc gatttaatta
135121 ctcctaatga aactgaagct gaaagtatta ccgggattaa ggttgatagt gaagaatcaa
135181 tgaagaaaag cagtgaagtc tttcatcaga tggggattaa aggcgtaatt atcactattg
135241 gtgaacgtgg ttcttatgtt tcctatgaag atattgaaga gattattcca gcctttaaag
135301 tcaaggccgt agatacaacc gccgcaggtg acactttctt aggtgctcta tctagtgagt
135361 taaaacctga tttaagcaac ttaaaagaga gtgtagttta tgcgtctaaa tcttcatcat
135421 ttactgtaca aaaactaggt gcatttccat ccattccgac tagagatgta gttgagcaag
135481 ctttaaagga gaactaaaca atggacaaga gtgaacaaga tcaattaaaa aaggaagcag
135541 ccactaaagc tgctatgatg gtagagcctg attctgtttt aggtgttgga actggttcaa
135601 cagttgcctt ctttattgat gcattaggtg aacgcaaaga aaaagagggt tttagtctta
135661 agcatattgt tactacttct aatcgtagta aaaagcaact tgaaggttta ggctttaaag

135721 ttgatgagtt agcagatatt gaccaggcag atttgactgt agatggagca gatcgtgttg
135781 atgataatct agatggaatc aagggtggcg gtggcgcttt aactttagaa aagaatgtcg
135841 ctattaactc aaagaagatc atttggattg ttgatgaatc taaactggtt caccacctaa
135901 gtggttttcc gcttccagtt gaggttttac cagtttcagc tgaacaaaat tttaaacgtt
135961 ttgaagtaga aggcttaaag ccgcaatggc gtctagatga taacggtaag cgttatgtaa
136021 cgcattatgg aaattatatt attgacctag ctgctgatcc aactcctgta ccatatggct
136081 tagcagatta cttagatcac acagttggtg ttgttgaaca tggattgttc ttagatatgt
136141 gcgatgaagt tattattgct catagtgatg gtacgattga agataagaaa agaaaataga
136201 tagttttaaa gaaaataatc taacttttaa agacttgact agttcaagtc tttttttgta
136261 acaaatctta gttcataata aatatgatac gtaaatttag aaagaaggac ctttatgtca
136321 aaaaagccct taattattag tacagatcct ggtatcgatg atgtagctgc aatgacgatc
136381 agcctttttg cgactgaact tgatgttaag atgattgtac caacttgggg taacgtttct
136441 ttagagcata ccctacaaaa tactcttgat ttagaaaagt ttttacatac taaagtacct
136501 gtagttaaag gtgctaacca gcctttagtg cgtccagcta ttagtgctgc ttctgttcac
136561 ggtaaaacag gcattgctgg ttttgaattt gaaactgcaa atgatgattt acttgaagaa
136621 ggacttgctg ctactaagat gtatgaggca ataaaaaata gtcctgaaaa agtaactctt
136681 ttagggatcg gacctttaac tgactttgcg cttttattta aacaatatcc tgatgtagta
136741 gacaatattg ctgaagttta cattatgggt ggaaatattg ggcatggaaa tcatagtcca
136801 tttgcggaat ataatattgc tggagatcca gaagctgctc aaattgtttt tcattctggg
136861 ttgccggttt atgttgctcc actcgaagtt ggtgataaag cacatctaac caaagaccaa
136921 atggataaga ttaaggaatg cggcgaagtt ggaaagatgt tatattcgat gttttcacat
136981 attcatgagc cagatggtga tcttagaatt aagatttatg atccaactgc tgtaggtata
137041 atgcttcatc ctgaattctt caccttgaag cctgcgaatg ttgaaattga attagctgga
137101 cgttatactt atggcgctag cgtgatggac tttttaagca aagaacataa cgcacaaatc
137161 gcaaccgacg tcgatacaga gaggtttgca acttggttcg ttcaaagtat ccaagctgcc
137221 aataacggga gaaaataatg actgatttag tctatcgctc agtaatgcgc gatattaaac
137281 aaaaaatact taataatgaa tatgaagata tgcgcttacc agatgagcgg agtttaagcg

137341 actattatca tgtaagccgt tcatccatga agcgagcttt gggattactt gcacagcaag
137401 gaatagtctt caaaaaaaga ggtagtggaa cttttattaa tcctctttac ttaaagaatc
137461 aagctctctt taaatatgaa ggatcaaatt tgggtattac tgactctttt agtgttcctg
137521 gtaaaaagca aagtattgag ctattagact atcaagtaat taaagctgat gaagatttaa
137581 ggcaagatct ctttttaaaa gaatcagact ttgtttatca aattaagcga ttacgtttat
137641 tagatgatca accatttttg attgaaaccg gctttattcc aattaagatt acgccagaat
137701 taaatccaga tattttaaaa ggatctctgt ttaattattt agaagatacg caaaataaaa
137761 ctgtaacgcg gtcattctta acgattacag ttgaaccttc aacattagaa gatcaatcta
137821 agttaatgct ggctcctaat gaaccagttg gcgtgatgga aggtattttc tttttagatg
137881 acggaacgcc atttgaagtt tcaaatatgc gaattcacta taaatatatg aaatacaata
137941 cttttgttaa cttaagtcaa gaataaaaaa ataaggcatc gtttttctcg ggggatgaga
138001 gaaacgatgt ctttttttgat tgaggggata gtttaatcaa attgcaaact atcattaatt
138061 ggtgccagat atacatagtt taagttagta tttttgcatt gttcactgag agaattaatt
138121 aaagcatctt gttccttagt gattggaatt tgttttagcg ctatttgaga ttgcaaaaac
138181 ataactaact tattactcca agcagttgcg tcatcatgat tttcggcttt ggctataact
138241 tttaaaattg tctgttcaag aggaagatat gccttaattt gaactttgct gaagctagca
138301 tataaatcct tcagtgtttt aacaatttca ttttgtttag taatagtagt ttctaacatg
138361 ataaagacct cactatctta aataactaat ttatttctgg gcccacttaa atactagcat
138421 ctgcaatgta tgaaaaggtg tttttacgcc catattgcag cttttgttaa gtgtttgtct
138481 tgatttaaat aacatcatac tgaaatttat cagtgaatct tttcttaaat actgataaat
138541 acttagaata aagctgaaaa tacagagaaa cacgcaaata tgatagggct ttctgataag
138601 attagacttg tgaggtgaga caaatgcaat cgcaagttga aagattagat ttaattagac
138661 gtacattaga tgaaagaaaa acaattagta cgcgtgaaat tatgcaatta tgccaggttt
138721 cctttgatac ggctagaagg gatgtaatta aattaaccaa tactggtcaa gcaattcgaa
138781 ttcatggtgg attgatgaaa atcaagcaag gaacagtgcc tgactataat tctagagcac
138841 atgttttatc acctattaag aataagatgg ctaaaatgac agcaaaatat ttgacggctg
138901 ataagttaat atatttgaat gcgtcaacta cgatttcgca gctttgccat tatttaacag

138961 gtttgaatgc cacaattatg acaaattcaa ttgataatgc aggagcctta atgatggata
139021 atttgcctaa tgtgatttta ctaggtggct atttgaataa ggaaaaccac tatacatctt
139081 ctttaaatag tttgaaagaa attgatcagt atgagtttga cgttgctgtg attggaacgt
139141 catcggttaa taagaatggt gtatttgtag ttaatcatcc tgatgcccag attgaaagag
139201 aagtagttaa tcggacaaag acagttattc tactcgctga agaatataaa tttaaagaag
139261 atcgttcttc accatataag gtgatggatt gcgaccaggc agatatattg attacggata
139321 ataagttaga tacaaaatat agaaagtatt ttaaaaagaa tattaaagtc cgagaagtat
139381 tagaggaaga aaaggcatga taaaacaaat ttttagtgat atggatggca cgattttaaa
139441 cggctcgggt gaaattaata caaaaacagt tgatttctta aaagactatc ctatcccgct
139501 gactctggtt tccgcacgag caccaatgga aatggaattt gcaattgatg aactaggttt
139561 gactggaccg caaatcgctt ttaatagcgg tttgatattt gaaaaaaacg ctcatggaat
139621 aactaagtta ttatctcatc ccattccaca tgatttggct ataaatatta ttgattttat
139681 taaaaaggaa tttcctaaag ctactatcag ttggtatacg gcaaacagct ggaatgtatt
139741 tgaatttaat gaagatgttg actatcaagt caggctaacg aagagaaaat atcatctgca
139801 aactgaaatc ggcaaagagg atatttataa aattatgatt ctagatgtta aggaaaaaat
139861 tacgtgtcct ttaacagctg ccttgagaaa aaaatttcca aatttggtta atactaaaac
139921 aacaggtcct aaatactgcg aaattactag ttgtaaggca attaaaagtt caggaattag
139981 atacattcaa aaaatgagaa acttacaaaa agaagaatta atagcctttg gagatggcga
140041 aaatgattta gaaatgctga aagaggttgg tgttagtgta gccatgggaa atgcagtcaa
140101 tgaagtaaag aaaatagcaa ctatggtcac cgattctaat gaagaagatg gccttgtaac
140161 cgcactttct aaaattggac cgtcccaata attttaaaag ttgaatttca atgcttgatg
140221 aattatgatt aatactgtaa taagtaatga aaactatttc aaggagatac ttgctatggt
140281 agtaaattat gattctgaag aatacttaaa gagcgtcgat gcatattggc gtgcagccaa
140341 ctacttatca gttggtcaac tatttttaat gaataatcca ctattaaaag gcgaattaaa
140401 agctgaagat gtaaagccca agccaattgg tcactggggg acaatcgttc cacaaaactt
140461 tatttatgcg cacttaaatc gggcaataaa gaaatataac ttaaacatgt tctatattga
140521 aggatccggt catggtggcc aagtaatggt atctaattca tacttagatg gttcatatac

140581 tgagcgctat ccagaaatta ctcaagatga aaagggaatg gctaagttat ttaagcaatt
140641 tagtttccca ggtggagttg cttctcatgc tgcaccagaa acaccaggat cgatccatga
140701 gggtggcgaa ttgggctatt cactttctca tggtgtaggt gcaattttag ataatccgga
140761 tgtaattgca gcggttgaaa ttggtgacgg tgaatcagaa actggtccac ttgctacttc
140821 ttggttctca agtaaattta ttaatccaat taaggacggt gctgtcattc caattttaca
140881 aatcaatggc tttaagatct ctaatccaac tatcgtttcc agaatgagcg atgaagacct
140941 aactaagtac tttgaaggaa tgggctggaa gccatatttt gtttctgcat ataaagatgg
141001 cgaatttaat ggttacaagg accacatgga agttcaccac gagatggcta agacaatgga
141061 tgaagttgtt gaagaaatta aggctattca aaagcatgcc cgtgaaaata acgatgattc
141121 attagttaaa tggccaatga ttgtctttag agtgcctaag ggatggactg gtcctaaatt
141181 tgacttagat ggtaatccaa ttgaaaatag cttccgtgct caccaaattc caattccagt
141241 ttctcaaggt gatatgagtc ataaggaaat gcttaccgac tggatggaaa gctataaacc
141301 agaagagcta tttaatgaag atgggtcacc taaggaaatt gttaaagaaa acaccttatc
141361 tggcgaccaa agaatggcga tgaatccagt aactaatggg ggaattaatc ctaaagtttt
141421 aaatatgcca gattaccgtg attttgcagt gaaatttgat aaacctggtt ctgttgaaaa
141481 gcaagacatg gctgaatggg caaaatactt agacaagatg gctgccttaa atccaactaa
141541 tttccgtggt tttggtccag atgaaactaa atctaaccgt ttattccaac ttttagataa
141601 tcaaaaacgt caatggatgg aaaatattca tactccaaat gatgaaaact tggctcgcga
141661 aggtcgcgtg attgattcgc aattatctga acaccaagat gaaggatggc ttgaaggtta
141721 cgttttgaca ggcagacatg gtttctttgc tacatatgaa gcatttggcc gcgtagttga
141781 ttcaatgctt acccaacaca tgaagtggtt aagaaaggct aaagaacaaa cttggcgtca
141841 tgattatcca gctttgaact tagttgatac ttcaactgtt ttccaacaag atcataacgg
141901 ttatactcac caagatcctg gtatgttaac ccacctttat gaaaagaacc gtccagattt
141961 aattcatgaa tacttaccag cagatactaa ctcacttctt gctgtatctg ataaggcatt
142021 tagagatcgt gaatgcatta acgttttagt aacttctaag caacctcgtc cgcaatggtt
142081 ctcgattgaa gaagctaaaa agttagttga taaaggtctt ggctacattg actgggcatc
142141 aactgataag ggtgctaagc cagatgttgt ctttgcttcc actggtactg aaccaacgat

142201 tgagtcgtta gctgcgattg atttattgca caaaaaattc ccagacttaa
agattcgtta
142261 cattaatgtt attgatgtca tgaaattaat gtctccagaa aagaatccga
atgcaatcgg
142321 caatgaagaa ttcgatcgtc ttttccctaa agacacacca gtcatctttg
catggcacgg
142381 ctttaaacca atgatggagt caatttggtt tgatcgtgga cgcggaaaga
atgatgttca
142441 tattcatggc tatgaagaaa atggtgatat tactacgcca tttgatatgc
gagtattgaa
142501 ccatatggat cgctatgatt tagcaaaaga tgttgtcgaa agtgttcctg
aactaagtga
142561 aaagaatgct gactttattg atgaaatgga tagcttgctt gctaaacacc
atcaatatat
142621 tcgcgacaat ggtaaagata tgcctgaagt tacagaatgg caatggaatg
gcttaaaata
142681 gagtttaata aatgattttt atagaaagac ctaataaatg ttaggtcttt
ttttatggtt
142741 aattagttat taaaattagc tctatcctaa gtgcagcata gctgttataa
tgaacttata
142801 gaatgtaagt gtaattttat tagtaaaata ttgaagggat gattggatgg
aatttaaaga
142861 tgtcttagca cgtgaacatg caacaagaaa attcacggat caaagagtaa
gtgaaaaaac
142921 tgtacgtaag gttatcgaag aagcacaaag aacaccatct ttacttaatt
cgcagccttg
142981 gcgcatttat gttgctgaag gtgaagtagc aaaagcaatt agaaaagaac
acgaagaaaa
143041 gactttggct aatgaagaac ctcacgaaga atttgattca ttattaaaag
ttgaatggga
143101 tacgtttcca agtaagaaca tggcaacaat gagtgaaact ttagattatt
tcttacgagg
143161 agaaacagat gattttgatc aagctcaact aaaattattc aatgctcctg
taattgtttt
143221 tcttactatt cctaaacaat ctccagcttg gtcaatcttt gacttaggtg
gcttttcaca
143281 gactttaatg ctggctgcaa ataatcgtgg cttaagtaca atgccagcac
atgcttttgt
143341 taagtatcca gaagtaatta gaaaatattt gaatattcca gaagatgaaa
caattggaat
143401 tgggattggc ttaggatatc ctaataaaaa ggcaacgatt aatgattata
agtctaagcg
143461 tgtaccactg gatgaaattt taaaaattaa gaaaaatctt taaccatgat
ttcctgaaga
143521 attgaatata ggtattctag ttcctcagga ttttttatgt ccaattattt
aaaataaaat
143581 taaaatatgg cataattata aaaaatggta aaagggatga aaataatgac
tttattagtt
143641 tctccgcagc agtgggctaa ttgggatcaa tggtataact acactttgcc
tggctatgtt
143701 ttaattttag gaactatctt tttatttgtg ggcttaatcc catttttatg
tgtaaaaaat
143761 aaattaacgg cgattttatt tggactaacg actacaactc tatgcgtttt
tttaggccta

143821 tcgtatttta aaaataagga atcaacagaa tataggaagg aaaatcatta cttaactgca
143881 atggtaagag tatatgatgc tcaaattttt gccaaaaagt attatgatcc aaatgaaatt
143941 gaaacattta aatatgtggc agatattaat acaccttctc atttgccttc gatttatcaa
144001 aaaattcctg ttaaacaaga agttacttat cttggtaaaa atgattatta tgcttttatt
144061 aaactagata atacagtgat gaaatttagt ttagcagatt gtaagaagat tcctggtaat
144121 aaagcctatt ttactggtta tcattttaag atacgaaata agaaattctt gaaattaggt
144181 tttattgatt taaaacatga tttacgcgat aagattgagt taccagcaga ttcttataat
144241 aagcaagtat cagagaatat tgaagaaaat tacaatcatc caggattagt tgcaaactgg
144301 atccctgatt cggagaaata atgctaaaat agagatgtaa ttatttaata tataaaaaag
144361 gtgaagatca cttgtctaca agtactataa ctactaattt aattgtaatt ttcataacgt
144421 ttttaattgc gatattcttt gttgttgccg aatttgcttt agtgcaaact agatctagtc
144481 agctagaaga tatgctagca aacggtcaag gtagccctaa gaaattaaag cgagcattac
144541 acatggttca taacttaaat gaatcattat ctactactca agtaggtacg accttggtcg
144601 gtgttatttt aggttggatt ggtcaaggaa cgatcgaaga gttattgact gatgtattta
144661 agatgacgcc ctggtttgga gtaaagacta gcggagtatt aggagcaact ttaggtgttg
144721 tactattaac atacttagaa gttgttgtaa ctgaaattgt ccctaaaaat attgccattg
144781 atatgcctgt taagtgtttg atggctgttg ttactccttt gactgtattt catacggtag
144841 tatacccatt tgtttggcta cttaaccata gcgccaatgg cttacttcat ttgttgggaa
144901 tgcaatcagc tgatgaagaa agtgaagttt attcacaatc tgaaattctc cgtttatcgc
144961 gtaatgctgt gactggcggt tcacttgata aagacgattt gttatatatg gagcgtgcct
145021 ttgaattaaa tgataaggtt gctaaagata ttatgactga ccgtactcag cttcaagttt
145081 tgaatgccac tgataatgtt aagacggctt taaagaaata tattgaggat ggtttcagcc
145141 gttttccagt tgttcgtgat aacgataaag atgatattgt ggggtatgtt tatgcatatg
145201 atattgtaag tcaagatcag gaaaatcctg atgttcccat tactagaatt attagagcga
145261 ttattactgt gcctgaatca atgttgattc aagatatttt gaagttaatg atcaaaaaac
145321 acactccaat tgttctagta gtagatgaat atggtggtac cagtggtatc gtaacggata
145381 aagatattta tgaagaactc tttggttcga tcaaagatga aatcgatgat gtttccgatg

145441 aatatattgt tcgtgacgat cacggcgaca ttcatgtttc aggaaagact actttatacg
145501 attttgaacg ttacttccat actaagctta agagttttga agacagcgat ataattacta
145561 tcggtggcta tatgatggaa cattacccta acctaaagaa gggtgaatct gttgacttag
145621 aaggctttaa gtttaagtta gtaaatattg aacaaggttt catgcgttgg ttccttgttt
145681 ctaagatagc taaggatact ggcaaaaaag cagcagatag cgatcaaaat agtgaagaaa
145741 aagccaaata atcggcaaaa gcaccttaag atgggtgctt tttttgataa ttaaatgtta
145801 taattctact aataacaaag catttataaa aagatataga gggttaagat gcaaaatagt
145861 gataatattg acaacattat tgatcgtgtt ttattcagcc aggatgatat tgaagaaatt
145921 tgccaaagac tgggcaaaca attgactgaa gactacgctg gaaaatttcc tttagtcatt
145981 ggtgtattaa agggtgcagt ttatttcatg acggatttaa ctcgtcacat gaatgtaaag
146041 atgcaaaatg atttcatgga tgtttcaagt tatggcgacg gttttgagtc aagcggcaaa
146101 gttagactag atattgacgt tcaagctgat gtcaaaaata gagacgtttt actcgttgaa
146161 gatattgttg atactggtca tactttgaag tttatgaagg atttattaac taagcgcggt
146221 gccaagagcg taaaatgctg tgctttgctt aataaaactg aacgtcgtga agatgatgta
146281 gttgtagatt actttggatc aaaagttgaa aatgaatttg ttgttggcta tggtctagat
146341 tacttaaatg catatcgtaa tttaccttat gtcggtgttt taaaaccaga agttattcag
146401 gcaaacttaa atcgttaata attaaaatat tactttaaaa gacaatcata aaagggttga
146461 aagctactcg ttagagatag tttcaaccct ttatttgtaa attaattctg ctgattgtgc
146521 ttcttcatat agtagtaaac tggtaatccc aaagccgtta atccaattcc agttattgaa
146581 agccaaaatt cattaattac ggtactaatt acaataaaca aaccgccgcc gattgaaatg
146641 atggggataa ttgggtacca aggaacaaca tatggacgat ctagcttagg ttcacgatag
146701 cgtagaacta ccactgcgat tgaaattaat gttgtaaagg tccacatgac aaataccaac
146761 atattagtta agagatcaaa agttcctaaa aaaatcatta ttaaggaaac agtcaagata
146821 attaaagcac taattgtcgg cacacctgta ttaatgttag tacggccgat tttatttgaa
146881 aatggcaaca ttttatcttt tgctagagta tatggaattc tcattccggt aagcatgaag
146941 ccattaattg ctccataaac agagattaaa attccaattg taattaattt gccgcccaga
147001 ctgccaaaga gctttaatga ggcaagataa gctgtatttt ggttgccaat tatttcttta

147061 aagggaattg cagttaagaa agcataattt actaacacat aaatcaaagt aattgcggat
147121 agtccgataa taatcgctct tgaaaggttc ttttctggtt ccttgacttc accagctaaa
147181 ttggtaacat taatccagcc atcatatgca aataaagcag ctaataaacc accacttaaa
147241 ccagcccaaa aagaaacatt atggccagaa cttaagggaa gtaagttgac gctaatttta
147301 gctggattaa aaagaccaaa aattacaatt aaagcaattg gaattgcctt cagaattgtc
147361 atcgctgact gcattctggt tgagaacttg gtagaaataa aatttaaact cattaaaaaa
147421 agagcgagca ataaagcaat cagggttgca tatttagtag ggaaaccaaa taagacagca
147481 aactgttgcc caaataccac gcttaaagcg gcgatattgg caggaaagta gaccagcatt
147541 tgtgcccagc caaataaaaa gccccaaact ttcccataag tatattcaat atatttaatt
147601 gatccgccag taactggaag tgcagcagca atttcagaaa ctgttagacc agaagcaata
147661 gaaacgatac cggctaaaat ccaaacaaaa agagttaaag atggtgtact tgtttcatga
147721 ctaatgctgc caattttaaa gaagacaccc ccgccaatta cagttcccac cacggttgcc
147781 agagcttgtc caaagctaat ttgtcttttc aatatgtgct ttcctttcga ttcatttata
147841 acttttagta ttgatatcat tttatactaa attttagtga aagtggtata gaaaggagaa
147901 gttcgttaaa ataactaatg gagggatgat tgtgcatcgt tatcgacata aatatacttt
147961 tccaagtatt attattttgc tcattctagc cttttctgga ctagtgtggg gatatttcaa
148021 tatgaagcaa aatacaactg tgccacgtgg ttcaaatatg agtgtgcttg gaattgaatt
148081 agatcaaact aaagattata ttgaccttca taaattagaa aagaacggta tttcatttgt
148141 ttatttgcgt tcaacgcaag gaaagtcgta ttttgacgac aattatttgc tttaccgtga
148201 tcaacttcag gggactaatc ttaattttgg tacaattatt gcctatagtg atgaaaccag
148261 caatcaggat cagtatcaat attttgtaaa taaagtagga actaatactg gcagtttacc
148321 agttatgatt gtaccggcta caaatcactt aacgaaatca tattggaaaa gaatgggtga
148381 atttgctcag atgataaata atcttggcaa gagaacgatg atagcaggtg actatcatta
148441 tcataattac tttccagaag gaacaagatt tttatatacg ggagcaagtt taaaagatag
148501 gcgacattat gccttttggt gttacactca aaatggaaga gttaaaaata ttgataattt
148561 agatcgagat gtaacaatgt ttgcttacat tggtaccaat acgcaatatg cgcaattata
148621 tagtcaagaa aaaacacaat agaggaagta aaatgtattc acaagcagtt tcacaagttc

148681 tagaaagaga acacaagact aaaccaactt taattcaaga gcggacgtat gatgcaatac
148741 gcaatggtgc aagtctggtt ggactagcta agactggtac gggaaagact ttggcatatg
148801 gattgccagt aatggagcgc gttgcagaca ttggcggtcg aggggtaatt ttagagccaa
148861 caactgaact agcaattcaa acgagaaata atcttttgca ttatgccaaa gctttaggac
148921 ttaaaacaat cgcactagtt ggggctggta atagaaaaag acaattagag cgtttaaaaa
148981 aagaaaagcc agagataatt attgctactc caggaagatt ttttgacttt ctatcagaga
149041 atcgaattaa gtatcaagat attaatgcct tagtaattga tgaggcagat gatattctag
149101 aatttgcaaa attagattta ttgagttcct taggacaaaa tttatcttca acagctcaaa
149161 ttttactttt tggcgcctca gaatctgaag ttacaaaaaa tgcagaaaat ctttttaatc
149221 atactttttt attagttgat gtacgcccag agcaaaagtc tgaagttaag cattatttct
149281 tgcaagtttc aaatgaatat aaaatgcagt ttctacaacg cttagcaaag ttagataaat
149341 tcaaaggtat cctgttcttt gacagtagcg aaactgaaat gagatttgcg cgaattttta
149401 atcacagcaa gactaaattt gctgttttaa gtaatgaaac gagcaagcaa aaccgtgaaa
149461 agttgcttag tgatttcaga gcaggtcgaa ttaagttttt atttgcgact gatttagctg
149521 cgcgtggctt agatttacct gatgttagtt atgtcattaa ttttgaaata ccaagtgaag
149581 ttaatacata cttgcatcgt agtggtagaa ctggacggat gaataagtca ggaacagtcg
149641 ttactcttgg ggatgaccat gattttagaa atctaaagaa attgctggct ggtacgtatc
149701 aaattgaaag agcatatttt gctggctata gtttaaccac tgaaaaacct aagcttaaaa
149761 aagaagagaa tgcagaaaag aaagagcata aaaaggtgga aaaaaataag ccaaagcata
149821 aaaagaaacg ttggcgtaat aagaaaaata aaggatatca tcctcgaaag agcagaggag
149881 agaaataatg tcatctttag ttaaatggct tgaaacttac gttttacctc ctgcatctcg
149941 cttagctcaa gtccgctggc tagttgcaat gcgggatact tttatttctt tactaccaat
150001 tacaatggct ggttcaattg caatgctatt taatagcatc attagagccg ctaaagtgca
150061 aatgcattgg gatacctttt attctttgat gcagccgatt gtcgcaattg ataatattat
150121 ttgggaaggt acatttgcac tttttgcagt ttattttgcg atttcttggg gctatcactt
150181 agctaagacc tatgaagtaa atcgttttgc tggttcagtt gcttccttag ttgcttttgc
150241 aatgagtatt agcgattcag tgaaattacg tattgatgga gatgtggttg atattaaaaa

```
   150301 tgcttttgat attaagcagt tttcaacaat gggattattt acagctatta
tttttggctg
   150361 tattgggact gccttgttta ttgtatttta taaggcaaga attcggctta
aagttgatac
   150421 cagtatgccc catgcggaat gggtagcatt tagtacttta attccaattc
tactttcagt
   150481 atttattgtt ggctttgtta attacgcatt tcaacggctt actggaactt
attttggtaa
   150541 ttggcttctt gctactattc agcgtccctt ggttaatttg gggcagggtt
ttggagtcgt
   150601 cttattggtt acctttctag ttcaaatctt ttggttcttt gggattaacg
gcattagcgt
   150661 tttaactcca gtaatggact ctttgtggtt aactccagaa aatattaacg
ttacagctgt
   150721 tagaaatagt gagcatgtac catatatttg ggttcgtgat tcatttaatg
tgtttgcttg
   150781 gtttggtgga gcaggaggta cattagtatt aattattgcg atcttaatgt
tttctaaacg
   150841 taaagattat cgaacaatag cgaagatggc tgtagcacct ggggtattta
atattaatga
   150901 gcctattatt tttggcttac ctgtagtttt taatccagtt tatttaattc
catttgttgt
   150961 tgctccgctt gtaaatgtat cactagcata ttgggctaca acggccggtc
tagttaatcc
   151021 ggttcaaatt tttgttccaa cagtaatgcc gccacttatt ggaccatttc
tagcatgtaa
   151081 ttatgactgg cgtgctattg ttttagcgct tattaatatg gtaattgcac
ttcttatttg
   151141 gatgccgttt gtttttgcag ctgataagat tgctaaacaa gataataatc
aacgcaattt
   151201 ctatttacca cagtattaaa tttttaatag ttaaaagtca gtttttatgt
taaagtagaa
   151261 actgattctg accccgtagt tcatctggat agaacaatgg tctcctaaac
cgtagaggtg
   151321 agttcgaatc tcaccggggt catattgatg tagagctatt tgtgaggttt
tataaataag
   151381 ccaaaattga gtttgacccc caaatgaccc ctaagcaagt taat
//
LOCUS       Contig_3             218765 bp    DNA     linear       18-
OCT-2022
DEFINITION  Genus species strain strain.
ACCESSION
VERSION
KEYWORDS    .
SOURCE      Genus species
  ORGANISM  Genus species
            Unclassified.
COMMENT     Annotated using prokka 1.12 from
            https://github.com/tseemann/prokka.
FEATURES             Location/Qualifiers
     source          1..218765
                     /organism="Genus species"
                     /mol_type="genomic DNA"
                     /strain="strain"
```

```
     CDS             36..1085
                     /locus_tag="OCPHGMPK_00708"
                     /inference="ab initio prediction:Prodigal:2.6"
                     /codon_start=1
                     /transl_table=11
                     /product="hypothetical protein"

/translation="MIVISIVISHLDLEKIYSVSIIYWIYFISIIIAIILTEPCFKLR

LYVPDNDEEKQVLRPNFNKPIELYIFANRGKKYRYLGITPLEYIKERKVSLVKHIYDT

SYCKKQVIDFCKVPFVSGKNRFYLNLPHIKKLFNTEKIGIIIEERTNKEKKTLYYYRL

GKRNLDCINKHPIFFNMIAIPVLLANGLGAIVYGVCLYFHKYIKFLVNSSVLIKLTQK

FGDVKYKVELTNKISMGFLIVYLVMIFWIFTLPFWTEKFDEFVKKLGIVITLFLDFAA

CIIILIFLEGRGLNDHFSFFCLIVWIIFNIIEVIILIYKWMLEDKNTGGIPRTQLIMT
                     IIFGILSFIVGKLWR"
     CDS             complement(1264..1596)
                     /locus_tag="OCPHGMPK_00709"
                     /inference="ab initio prediction:Prodigal:2.6"
                     /codon_start=1
                     /transl_table=11
                     /product="hypothetical protein"

/translation="MINAFIGIDPGRYQSGEMDASLSITKRGNAIARKLLYRAIGQID

NAAKTNPCHIADYYESKKLSSQTKGFKKIAIASIHKLIRTIYALITNDQPYDYNVATH
                     NQKDFGRN"
     CDS             complement(1635..1904)
                     /locus_tag="OCPHGMPK_00710"
                     /inference="ab initio prediction:Prodigal:2.6"
                     /codon_start=1
                     /transl_table=11
                     /product="hypothetical protein"

/translation="MQLKGIPKKHAQKTSNKLKDLSNKAYPVVSENSVEVEQVQYYAQ
                     RLINLNEQRKNLISRMVKLAKSLPNHDLESLESILALSKLRLLEY"
     CDS             2654..3139
                     /locus_tag="OCPHGMPK_00711"
                     /inference="ab initio prediction:Prodigal:2.6"
                     /codon_start=1
                     /transl_table=11
                     /product="hypothetical protein"

/translation="MKYKKDKIFYKFKDKDKIYKLVTYDESKIDYYSLIKKYQREGYY

QLIINSRLMIDLIKDFKKIDLTVYDIELQEDDETFYGELRKTLKGLKNKHNSELCFKY

LLQSLNLLYCDNSIEINKISLGNSSDDVIVYSNGIVIMNEDIEEKIKAFLKKELQLII
                     Q"
     CDS             3136..3711
                     /locus_tag="OCPHGMPK_00712"
```

```
                     /inference="ab initio prediction:Prodigal:2.6"
                     /codon_start=1
                     /transl_table=11
                     /product="hypothetical protein"

/translation="MKKLYKNLLKPLMIIIFSLSCAHFFNLTRIIPIIPKKNLSLDIS

IYSVFFTYLINLIEDYYYKSKISVELYLNTGRQFLNKKDENLSMNEKKPATIYAKIHC

DDARKILNFNLEVILKVPDFVSASCGKSVVQENRDLHICKLDSEDMVIPISVMANEIS
                     GNKSDTIYLTIKENKNKRKLIIKTNKMKIGV"
     CDS             3713..4669
                     /locus_tag="OCPHGMPK_00713"
                     /inference="ab initio prediction:Prodigal:2.6"
                     /codon_start=1
                     /transl_table=11
                     /product="hypothetical protein"

/translation="MSNTTRWIFMEKQNTKTNEKDQNISIEKIGAKIEEVKNNTSVLA

DKQNTISKVEVNTAFPKFKKETFGSNQINFNFYNISYYRTSNIDNTSTEDNFWIILYT

LNQKKYLIINKNSGALTLLRKIFGFKGRNEIAQVPHGIDSRIIIWLISMVYNSSADYT

NSDKILSIDSINGFKGNTEDLLNTVSADGSGIMNILSTLSFLLESSSLRQIRLTLSFG

EHKNLELKIDVNNTIESNEDKYRGIFLIDEESTTDSVIYNQYLLIYNEIIPLIRQWFN
                     ESQDDEEENLTEEDWWKKFYKNFLIQVSDDLKNKVDEKRKRL"
     CDS             6106..7281
                     /gene="mta"
                     /locus_tag="OCPHGMPK_00714"
                     /inference="ab initio prediction:Prodigal:2.6"
                     /inference="similar to AA sequence:UniProtKB:P71039"
                     /codon_start=1
                     /transl_table=11
                     /product="HTH-type transcriptional activator mta"

/translation="MPKYYTSGEFAKKAHVSIRTIRYYDQKNLLKPSTHTTSGARRYT

DADFAKLQQILLLKYLGFSLSDIREMTLGSGDRQLLLDSLQIQKRLVEERLKEMQNVA

TAIDSTSSTLKAGQEVDWSKMLDLIHLTSMNQSLSTQYQNASNISARIRLHRDYSLNK

EGWFHWLFKQLDLKPGMKILELGAGNGALWSQNLDKLPKGLTIVLSDISEGILADAKN

EIGDNSEFQYAVFDAQKIPFADNTFDLVIANHMLFYCDDIPKTLKEVRGVMKKGASFA

CATYSKRHMHEITDLVQEFNPEIVLSSTNLYDRFGLDNGAEILSKDFADISCHKYQDA

IELSESTPVISYILSCHGNQNAILLDHYNEFKNYVDEKVSGGFHITKDVGYFICKKT"
     CDS             7363..8400
                     /gene="rffG"
                     /locus_tag="OCPHGMPK_00715"
                     /EC_number="4.2.1.46"
```

```
                     /inference="ab initio prediction:Prodigal:2.6"
                     /inference="similar to AA sequence:UniProtKB:P27830"
                     /codon_start=1
                     /transl_table=11
                     /product="dTDP-glucose 4,6-dehydratase 2"

/translation="MKVIVTGGAGFIGSNFIFYMLKKHPDYEIICLDSLTYAGNLSTL

KDVMDNPNFKFVKLDIRDREGVYKLFEEEKPDVVVNFAAESHVDRSIENPEIFLETNI

IGTSVLMDACRKYGIKRFHQVSTDEVYGDLPLDRPDLFFHEDTPLHTSSPYSSSKASA

DLLVGAYGRTFHLPVTISRCSNNYGPYQFPEKLIPLMIQRALNNEKLPVYGDGKNVRD

WLYVEDHCKAIDLILEKGTPGEVYNIGGHNEMHNIDIVKLICDYLDKPYSLIEHVTDR

KGHDRRYAIDPEKIHNELGWLPETMFKDGIKKTIQWYLDNKEWWENIISGDYQNYYDE
                     MYGKKQVLDKD"
     CDS             8407..9291
                     /gene="rmlA"
                     /locus_tag="OCPHGMPK_00716"
                     /EC_number="2.7.7.24"
                     /inference="ab initio prediction:Prodigal:2.6"
                     /inference="similar to AA sequence:UniProtKB:P26393"
                     /codon_start=1
                     /transl_table=11
                     /product="Glucose-1-phosphate thymidylyltransferase"

/translation="MKGIILAGGSGTRLYPLTLVTSKQLLPVYDKPMIYYPLSTLMLA

GIKDILVISTPADTPRFKELLGDGSQFGINLSYKVQPTPDGLAQAFTLGEDFINGEPC

AMVLGDNIFYGNGFTDLLKNAAEDAQKGKATVFGYYVNDPERFGVVDFDENGKAVSIE

EKPEQPKSNYAVTGLYFYPAGVSEKAAQVKPSARGEVEITSLNDMYLQDNNLGVQLLG

RGYAWLDTGTMQSLVDASNYVKMIEERQGVSVSAPEEIAYVHGWIDKDQLLEAAKHYG
                     KSPYGKHLKSVAEGKVRY"
     CDS             9324..9932
                     /gene="rfbC"
                     /locus_tag="OCPHGMPK_00717"
                     /EC_number="5.1.3.13"
                     /inference="ab initio prediction:Prodigal:2.6"
                     /inference="similar to AA sequence:UniProtKB:P37745"
                     /codon_start=1
                     /transl_table=11
                     /product="dTDP-4-dehydrorhamnose 3,5-epimerase"

/translation="MGQIKVEKNVGGIEGLAVITPTVHGDDRGYFMETFNQRDMMDAG

FSINFVQDNQSSSTKGVLRGLHFQKKYPQIKLVRAVRGSVFDVAVDLRSESKTYGKWY

GVELTAENKKQFLIPQGFAHGFLVLSDVAEFCYKVNDFWHPNDEGGMAWNDPEIGIEW
                     PHLKGDYPGSPDASGYTLDDGTKLNMAERDQEWKGLKDTFKF"
```

```
     CDS             9949..10935
                     /gene="strL"
                     /locus_tag="OCPHGMPK_00718"
                     /EC_number="1.1.1.133"
                     /inference="ab initio prediction:Prodigal:2.6"
                     /inference="similar to AA sequence:UniProtKB:P29781"
                     /codon_start=1
                     /transl_table=11
                     /product="dTDP-4-dehydrorhamnose reductase"

/translation="MKVFVTGVNGQLGHDVMNELAKRGYEGVGSDLAPEYAGVADGSS

VTKAPYVSLDITDAKAVDKVISEVNPDVIVHCAAWTAVDMAEDDDKVAAVRKVNVDGT

QNIANVAKKLDAPMVYLSTDYVFDGQGTTPWEPDFKGYKPLNVYGETKLGGEEAVANT

LDKYFIVRIAWVFGVNGSNFIKTMLKVGSNHDEVKVVSDQIGTPTYTFDLARLLVDMI

ETDKYGYYHATNSELPTTESGYDENGTKTGYISWYDFTKEIYRQAGYKTKVTPVTTEE

YGLSKAVRPFNSRLDKSKLEENGFKPLPTWTDAISRYLDTLKDDGFFDELKK"
     CDS             12037..13146
                     /locus_tag="OCPHGMPK_00719"
                     /inference="ab initio prediction:Prodigal:2.6"
                     /codon_start=1
                     /transl_table=11
                     /product="hypothetical protein"

/translation="MKKDDIKDILDSNKINSFSSYFNYSQLADLNKNLVESLSPAYSI

TSKVASLNKRYAAISSAYLERPTNTMSVNASKYIKNLHKDLALGVLPNIKNFNTDVWY

KGVIASFERIKKLTNVFERQFKVLNYFPHIMQFSPKDVSNLIIPKEEIALSLADNHWI

MLLRDSKIDKELLNECKESENNNFNEIVQNFYSRNNHKNVFRKIDYLIGADYSKKEVK

LVDGFHDQIKRVRKILFRDFSDSEVLISTVMTWIEYAVAEKFGVLEDKNGYKLGGRIE

EFEEKIEKLELKEITLLVVDYYNIFFYLSDLWNSRLQDFEDGKDQVGIGRHSIQHARV
                     NPKRYTDEVMSKLICLLYALVKLPDLDEFESFLQV"
     CDS             13380..13685
                     /gene="celA"
                     /locus_tag="OCPHGMPK_00720"
                     /EC_number="2.7.1.205"
                     /inference="ab initio prediction:Prodigal:2.6"
                     /inference="similar to AA sequence:UniProtKB:Q45399"
                     /codon_start=1
                     /transl_table=11
                     /product="PTS system cellobiose-specific EIIB
component"

/translation="MTKNVLLVCGSGASSGFMAANMRKAAKKAGLDYKIQARSEAELG

DYADQIDALMVGPHLKAEFDAIKAQVPDKVKVILMKPDYYSILDGKAAVEDLQSQLS"
```

```
     CDS             13706..14935
                     /gene="licC_3"
                     /locus_tag="OCPHGMPK_00721"
                     /inference="ab initio prediction:Prodigal:2.6"
                     /inference="similar to AA sequence:UniProtKB:P46317"
                     /codon_start=1
                     /transl_table=11
                     /product="Lichenan permease IIC component"

/translation="MNGFMNFLNTKFAPRAQKIANNGWIVTIKNSILEVLPFIFVGSF

ITLLNIPLNFWKWWPNLAPISSFTFGLVSIFIAFLFPFNFMEHMKLNKQRIIAGLSSV

ALFLMLANPTWDKSGSIISYQFSELGAGGMFVALVVGIYTALIMEAFGKFSFFSDDTS

MPDFVIAWFDSMLPITVVIATGWLLSDILHFNFYQLIVNIFSPLATSLDTWWGFTLFL

FFQCFIYSMGISGWVLAAIDQPVMLAGIAANAREVAAGHAATHIFTSELVYSFPWIGG

VGCTLPLVLLMLFATKSKRMKALGKACILPSIFNINEPVVFGTVVWNPYLMIPLWING

IVLPLITSLWFKAGLSPIPHSLMQMWYIPFPFVTWIVSPAISSLILLLILFVVGGLIW
                     YPFLKVYDNSLIKEEKA"
     CDS             14948..15286
                     /gene="lacF_3"
                     /locus_tag="OCPHGMPK_00722"
                     /EC_number="2.7.1.207"
                     /inference="ab initio prediction:Prodigal:2.6"
                     /inference="similar to AA sequence:UniProtKB:P0A0D6"
                     /codon_start=1
                     /transl_table=11
                     /product="PTS system lactose-specific EIIA
component"

/translation="MNENDKSVKDTMEIIINAGDARELISEALDNVADFDYKAAEGNM

EKAKEKLVIAHRLQTAKIQQEAEGKKVEYSVLFTHAQDTLMTINSEYKLTAHLIKVFK
                     KRDEKEKHND"
     CDS             15279..16745
                     /gene="bglH_3"
                     /locus_tag="OCPHGMPK_00723"
                     /EC_number="3.2.1.86"
                     /inference="ab initio prediction:Prodigal:2.6"
                     /inference="similar to AA sequence:UniProtKB:P40740"
                     /codon_start=1
                     /transl_table=11
                     /product="Aryl-phospho-beta-D-glucosidase BglH"

/translation="MIKMRKNKYPYFSKNFLWGGAQAASQADGAYQEDGKGLNSSDVQ

PYLKGLSNAEIQKIETEGMTVEQVKKNSEDTTDYYPKRFGIDFYHTYKEDLKLLADAG

FKTFRTSLDWSRIFPNGDDEKPNEAALKHYDEMIDYMIKLGIEPIITMNHYETPINIT
```

LKYGGWPNKAVIPMFEKFGKVLLDRFGDRVKYWIVVNQINMVQIEPWLSVGVASDQYD

NMQEALYQAMHNQFVAAAAIQKYAKSLHNPNLHIGTMVADGTVYPASSKPDDIVLAMQ

QNRMQYFVTDVQFRGEYPKFALNYFADNDIHLETTEEELKLIKDNPMDFLAISYYYSK

MVDSTKNEMQPADTSDNPNLKQSDWGWAVDPQGLYNSLSQYYDRYQKPIIIAENGFGA

YDKLEDNHEVHDQYRSDYLRAHIEQVGRAIHDGADVIAYCAWGPIDIVSCSSQQMSKR
YGFIYVDLDDEGKGSGKRYLKDSYNWYKNVISSNGAEI"

```
     CDS             16768..18654
                     /gene="licR"
                     /locus_tag="OCPHGMPK_00724"
                     /inference="ab initio prediction:Prodigal:2.6"
                     /inference="similar to AA sequence:UniProtKB:P46321"
                     /codon_start=1
                     /transl_table=11
                     /product="putative licABCH operon regulator"
```

/translation="MSKRDNLLKYLQKNPSWHTSQELAIEIGVSSRTIKNYINFWKAE

KYPIESGIKGYKLKYKKVSENEKTVVSTQSSSKERINWIINYFITKDSVINFYDLASK

LYVSETTILQDLTKVKNKVASFGMKLERHGDNFELIGDERRKRSLLSALIYEETSGTF

MNQEVIQKNFEDIDVSLLRKIILESFNQRNIYLNTFDLNNILLHFVIVIERLKHGHVI

SKTNFEKDSNINNKNVLAYKIVQSVENALSIKFELPDFKELELIVEASVIDKSSNLHN

KIKPETANMVTDLISYVWSNYHINLDNDNFKDRFALHLDRLIARARQEKIQHNPIATN

IKNSSPTIYECAVIIADRISQKLNITIDDNEIGYIAMHIGNAMAEQISDKQKLAAVIL

IPQYYDNAAVLTRKIQKHFAQYINIKDEIYDPIQLHARKLKIDLIIAVDTNYVDEKIS

TVNISQFFLKNDIQKLTIAIATKQHQLRKDIFQENLFKFFNSEDFEKSDEITNIDDCF

DLVGSRFSKERVVDKNFKQALYNRENMSSTAFGRVAIPHSLTMSANKSRGFIIINPRG

IKWSNNNEVYLVIALAIDPDNKKLFRDVFDELSNIVTDINNVSQLVHCNSYSEFIIKL
VDLL"

```
     CDS             18848..19273
                     /locus_tag="OCPHGMPK_00725"
                     /inference="ab initio prediction:Prodigal:2.6"
                     /codon_start=1
                     /transl_table=11
                     /product="hypothetical protein"
```

/translation="MSIYRNDDILITDLMDRIDKLENQVTDLKKVFYKQSGESHSTFL

YGCEVRGSDYLHLEDKVIPRLEENDPVLLIREADNKYDKNAISVATPAGLKLGYIPRE
HNLIFSRLIDSGNLLFGRVKNFHWDGKNLELVIKVYLAN"

```
     CDS             19363..20280
```

```
                     /gene="htpX_2"
                     /locus_tag="OCPHGMPK_00726"
                     /EC_number="3.4.24.-"
                     /inference="ab initio prediction:Prodigal:2.6"
                     /inference="similar to AA sequence:UniProtKB:O30795"
                     /codon_start=1
                     /transl_table=11
                     /product="Protease HtpX"

/translation="MLYKQIEQNKRNTWIILGLYTALLLVLSIFLGMIFTRYVGIGFF

IIGLIYIAHVYFDATQHLMKVTNAVEITKESDPEIYELVEELCLAGGLPVPKIYITPD

PDPNAFATGRDPEHASLALTQGLLSMMNKKEVQGVIGHELSHIRNYDTRITVLASALT

SLITITGIGIVLFGWGVLTSESKGWLGLIIKFFAMFLIIVGGIISIIGIPLAKLLFLL

VSRQREYLADIGSVDLTREPSGLISALSKLEKMEESESTPEINSQDLALEHLYFNLPH
                     PGNWIDRLFSDHPPLDKRIERLKNSDKIE"
     CDS             complement(20440..21279)
                     /locus_tag="OCPHGMPK_00727"
                     /inference="ab initio prediction:Prodigal:2.6"
                     /codon_start=1
                     /transl_table=11
                     /product="hypothetical protein"

/translation="MTLQINTRVDSITWPLNQNLTNQEIISQLELTVTENGQLISNDN

LSINKKAVNRSEAGVYPVIITAIGSNNEYATKKIQAWVTDKNNVNSTTRPPVQPTPAP

QYKTPSQTPSSKKKSKKKVVYIVLGVLVLLIAIIFGISACQAHNDTVQNEQAQSNALN

NTNSKLDNLSEQNKQLMQQNKELQNAINNYKQDQNKEELQNQLDQIKNENQQLKNNAA

NNQALQNQIDQLGSTIDQIKQNPNQADSLLNQMKNNQNEFMQNLNATFQKLVSDFQQL
                     IGK"
     CDS             21400..21762
                     /gene="mutX"
                     /locus_tag="OCPHGMPK_00728"
                     /EC_number="3.6.1.55"
                     /inference="ab initio prediction:Prodigal:2.6"
                     /inference="similar to AA sequence:UniProtKB:P41354"
                     /codon_start=1
                     /transl_table=11
                     /product="8-oxo-dGTP diphosphatase"

/translation="MMKRSEPVTLTNMCMIKKKDKILVLDRNDPVWPGLTFPGGHVES

HESFHDSVVREIKEETGLLIKNPHLVGVKQFFDKNDERYLVFFYVATEFTGTVKASDE
                     GKLTWMTKEELTSKKISL"
     CDS             complement(21874..22512)
                     /locus_tag="OCPHGMPK_00729"
                     /inference="ab initio prediction:Prodigal:2.6"
                     /codon_start=1
```

/transl_table=11
/product="hypothetical protein"

/translation="MKKLIVGALSCGLLLLAGCRNNIVEPKHKSSADDYAIIQSSSHK

KSHKKVEQPKKKSEDKKKKDKDKSKDENKKMDDQIDTLDKNENNSSSEAQSSSSALPS

LNNSKHENSTETNRSLKSNFGTSLYTLPRQNYSYYTPKSNYYYSNNWSTNNNTDSSHQ

NSAVSNSKSEPSTSTSSSSSNNASTSTEEKQSPTTQNNDSNSSESSEDVQND"
CDS 22717..23121
/locus_tag="OCPHGMPK_00730"
/inference="ab initio prediction:Prodigal:2.6"
/codon_start=1
/transl_table=11
/product="hypothetical protein"

/translation="MYDLEDVMMKSFRDDNGISSAFSIEPVDDNEYRVSTNVVDGLND

MIHFNLIVYPNGTYALNDRVFTARMIDDNIGSFSSIAADVARLASEFGVLLDDRGELT
RDYINSRDLRSEVNNFTQLLNRVVEFAQLNRY"
CDS 23199..23582
/locus_tag="OCPHGMPK_00731"
/inference="ab initio prediction:Prodigal:2.6"
/codon_start=1
/transl_table=11
/product="hypothetical protein"

/translation="MEESELKLIKHASFDWLKQNIQFIQAGEDIEVATPLIGAYGDLV

YCWIEKEKDGAYRITDDGGTLFKLDPAQENFDLLEEAADIVIGAGFEFDEDNSEIYQI
VDLENMAQTLSDLTQLQVALTYLAS"
CDS 23800..24603
/locus_tag="OCPHGMPK_00732"
/inference="ab initio prediction:Prodigal:2.6"
/codon_start=1
/transl_table=11
/product="hypothetical protein"

/translation="MMRQIKKRNIMTGLGAAALLASLSLNGTAFAASTDSANAGAEQA

KTYQTQLEQHKNLQAYTNPLSVKTLTTTKGVLPDATSGISNWNTVPAGTVANWDKTPE

INKVGTSYGTVYVTFPDGSRSRLAVYVTVKDTAVSQDSKASKKDLNSGSATSATDQQD

NASKQTTSVKKDVAKENTSKNSSEKTNKDVVVKDLGSTTVTEKNVSVVNNTKDNKKNS
KKSSNPVSVATQLPETEGKAVNPLVVIGGLLVAAIGAVAIFGKKIKNRL"
CDS complement(24672..26024)
/locus_tag="OCPHGMPK_00733"
/inference="ab initio prediction:Prodigal:2.6"
/inference="similar to AA sequence:UniProtKB:P9WQM5"
/codon_start=1
/transl_table=11
/product="putative transporter"

```
/translation="MTNNNSVPKKLSFISIYFLGINAVIGSGTFLLPSVIYRYMNLTA

ILVLLCTAVTVSMIALCYADLSSRFTQSGAAWLYSYNAFGRFAGYELGIFTWFLGCTT

LSAEVVALLTVLKSFLPIFKNNAVYIGSIIFLILLFSVINFFGRSWVKIVNNISAAAK

IITLIVFIIIGAFFIKKSNFTPVIPHAALTGVTPFFKHFGDAFTPIFYLFTGFSFIPI

AAKQMNNPEKNIPRVLVAVMTSVTILDCLMLLVAIGLSGQKLGSYSNPLASALKTGVG

QWGFAFMIIGMLISIFGVAFSASFNTPSLIASIANEHGMLPKWIGKKNKHDAPWVGII

FTAILSAALATQSYLFLVSCTVLASFVQYVPSILAVIKFKHSNEYPTHGFSLPGKYTI

PIIALIISCYMVTNFTPKTLLLGAVVAVFAAACYFFIDKDEKLEKEHENFLAKLRHIK
                     "
     CDS             26299..26940
                     /locus_tag="OCPHGMPK_00734"
                     /inference="ab initio prediction:Prodigal:2.6"
                     /codon_start=1
                     /transl_table=11
                     /product="hypothetical protein"

/translation="MTKIAILGAAGQIAQLVEPMLLKKNDINMVLYLRHPNKLKQIND

AREKVIKGDASNFNELKEALADVDLVYANLAGSNIEDQAKTVVKAMNANGIKRLIWVS

SLGIYDEVPGKFGEWNKNILGSYLTTYRAAADQITTSNLDYTIIRPAWLTNKNEISYE

KTVGATTPFRGTEVSRLSVADYIADLIENPTKDVKANVGLDKAGTDGDKPAWY"
     CDS             complement(27015..27626)
                     /locus_tag="OCPHGMPK_00735"
                     /inference="ab initio prediction:Prodigal:2.6"
                     /inference="similar to AA sequence:UniProtKB:P9WP07"
                     /codon_start=1
                     /transl_table=11
                     /product="putative membrane protein"

/translation="MTTWLTQFIESFGYIAIILLIAVENIFPPIPSEVILTLGGFLVS

GTKLTLIGVILASTLGSVIGAIILFSISRNLTLPRLERLLETKLFKLLGFKKDDAQKA

INWFDKHGIGAIFYGRCIPVVRSLISIPAGIAHVGWTKFLILTTLGSLVWNSVLVGLG
                     HYMGKNWQVVVRIFDDYTLVIIALLLILFIYFGIKWYKTRIKK"
     CDS             complement(27635..28561)
                     /gene="rihB"
                     /locus_tag="OCPHGMPK_00736"
                     /EC_number="3.2.2.8"
                     /inference="ab initio prediction:Prodigal:2.6"
                     /inference="similar to AA sequence:UniProtKB:P33022"
                     /codon_start=1
                     /transl_table=11
```

/product="Pyrimidine-specific ribonucleoside
hydrolase
RihB"

/translation="MKNIYFNHDGNIDDLVSLLLLLQAPDIKLIGISAIDGDGYIDPA

VEACRKMVDKFNLRGDKLEVARSNSRAIHQFPKEWRMATYSFNYFPILNESGEIKTPE

AALPAHLDMIDKIKKADGPVTLIMTGPITDLARALDEDDSIQSKIDKVYWMGGSLDGH

GNVVSVDADGTQEWNAFWDPEAVKRVLDSDLKIQMVGLESTEELPLTDELRQHWASLR

KYPAIDLVGLGYSLIISIPNAELYLWDVLTTMSALYPELVETRQVKANVITSGLASGR
MFIDPNGKEITEVTKADKDSFFEKIDKILERQ"
CDS 28705..30507
/gene="uvrC"
/locus_tag="OCPHGMPK_00737"
/inference="ab initio prediction:Prodigal:2.6"
/inference="similar to AA sequence:UniProtKB:Q5KWH6"
/codon_start=1
/transl_table=11
/product="UvrABC system protein C"

/translation="MATEHIENKLKLLPDKPGCYLMKDINGNVIYVGKSKNLKNRVRS

YFKSKQVGRRAELVREIRDFDIITVSSDKESFLLEITLIKKYQPYYNVQLKQGTGYPY

IEITNERDPQTRLTSIVHKDHGYYFGPYPNVYAAQATLKFIQKVWPLRRCSGHQGRPC

LYYHMGQCLGACFKEVPKSAYDKQIRKIKRFLNGDISQVKQDLTEKMTQASMDLEFER

AAEFRDQLKYIEQTVEKQKIISNDHTQRDIFNFYVDKSWISIQVFFLRQAKLLRRETR

LFPLTDTNEPQDAFVSFIAQFYGQRNRVLPKEVLVPAGIDNEALAEVLGVTVRTPQRG

QKRALLEMAHDNAKLKLDEKFRLLELGNRKTKGAQKEIFDALGLPYGHRIESFDHSHI

QGADPVSALVVFTDGEPDKHEYRKYKLKGEVEHQNAADEVGNTREVVRRRYSRLLKEH

KKMPDLILMDGGQIQVDACLDVLRNELNVNIPVAGMVKDDHHRTNHLIFGDPTQGEEL

KLIPLNPKSEGFYLMTRIQDEVHRFAITFHRRTHAKNALSSKLDEIKGIGPKSRNKLL
RKFGSLKKIKEASIEELRDAGLTLPQAQTVKLSL"
CDS 30615..31901
/gene="obg"
/locus_tag="OCPHGMPK_00738"
/EC_number="3.6.5.-"
/inference="ab initio prediction:Prodigal:2.6"
/inference="similar to AA sequence:UniProtKB:Q7A584"
/codon_start=1
/transl_table=11
/product="GTPase Obg"

/translation="MFVDQTKIDVQAGKGGDGAVAFRHEKYVPLGGPAGGDGGRGGSI

ILVADSGLRTLMDFRFRRKFKADNGENGRIKSQYGRGAKDVRLKVPMGTSVYDFNTGE

LLGDLVKNGQELVVAHGGKGGRGNIHFATPTRTAPEIAENGEPGEFRTLRLELKVLAD

VGLVGFPSVGKSTLLSVVTKAKPKIAAYEFTTLTPNLGMVVLPDGRDFSMADLPGLIE

GASKGVGLGIQFLRHVERTKVILHLVSMDPNNGRDAYEDYETIRKELAGYTKDLTSKK

ELIVATQMDIPGSEEKLAEFKKKLGDKTVYPISSVTHQGVSELMGKTADLVEEVAKEE

AEKPAEIKVAEKEYVYKKPEDDGFKVERTGEHSFIVTGNKLERLVQRTNLDHTDGIML
LARKLKRMGVDDALREKGAVNGDDVSIADFTFEFVD"

```
     CDS             31932..33857
                     /gene="oatA"
                     /locus_tag="OCPHGMPK_00739"
                     /EC_number="2.3.1.-"
                     /inference="ab initio prediction:Prodigal:2.6"
                     /inference="similar to AA sequence:UniProtKB:Q7A3D6"
                     /codon_start=1
                     /transl_table=11
                     /product="O-acetyltransferase OatA"
```

/translation="MKRKNRFITGYAGLRALAVIGVILYHLNPNRFMGGYLGVPIFLV

LSGYLVTDHMFHAYEERGKYDFKSFYVRRIKKLYPQMIALLWGCSAYILLFQQNLLAK

LNQIVVANLLNVYNFWQIKNGQSYFERFASNESPFVHLWTMSIEGQFYIIWPLVVFLL

VKLAKKKKIIFWIITALTVFSALEMAFLFKPGVDTSRIYYGTDTRFFSLGLGAMLAVF

WPTWKLNANLEKGDRRLLNVIGAIALVGILLMSTSPIFNPQKAFAYRGGMFLFSLVTM

IFVGVIAHPGSSWNKWLTNPVFSWIGSRSYGIYLYQFPVMIFFEDKVKDIADHVILYH

LIEIILILVIAELSYRFIEKPFGRIDWEKLRQFLSKALNLQANHYQSKALFATGIVVF

ILGSFGILKAPTVKAENPNHSELATQIKSNRNKQLKKNKKLIKEAQSKKKKSTTSKAE

ILAQAKKAATTKPVNKDFEKYGISQVDLQLAQKIQVTAIGDSVMAGSSQNLQKLMPHL

IVDAAVSRQLGDTIPLFEEYKKKGALNNNVLIGLGTNGAFAPKELDRLMQIIGPKRQV

FWINTHVPTRDWQDQVNGELDAAAKKYPNLTIIKWHEFAGSHQDWFYDDHTHPTTEGS
KFYSAYVTKTLVTKGKY"

```
     CDS             33871..34800
                     /gene="rnz"
                     /locus_tag="OCPHGMPK_00740"
                     /EC_number="3.1.26.11"
                     /inference="ab initio prediction:Prodigal:2.6"
                     /inference="similar to AA sequence:UniProtKB:P54548"
                     /codon_start=1
                     /transl_table=11
                     /product="Ribonuclease Z"
```

```
/translation="MEIQFLGTSAGQPSKSRNVSCTALKLLDELNEVWLFDVGEATQH

QILKTNIRPRKVTRIFISHTHGDHIFGLPGFLSSRSFQGDGGPLTIYGPAGIEQFVQT

SLRVSKTRVSYPIKYVVLKEDGLIFENNLFAVYTARLDHRVPSFGFRVVEKPRPGELL

MDKVAEYNVPNGPLLGQLKAGKTITLSDGQKLDGRDFLGKERPGRVVTIIYDTRPTEN

IGELADHADVLVHESTFNGDEEKMAHRYFHSTCLDAARIARDHHVGKLYLTHISARYT
                     GKAGKQLEHEARKIFKYTRLANDLDSFEITLRG"
     CDS             34805..35599
                     /locus_tag="OCPHGMPK_00741"
                     /EC_number="1.-.-.-"
                     /inference="ab initio prediction:Prodigal:2.6"
                     /inference="similar to AA sequence:UniProtKB:Q7A3L9"
                     /codon_start=1
                     /transl_table=11
                     /product="putative oxidoreductase"

/translation="MSNSLRDKVVVVTGASSGIGRSIVLESASRGATVILMARHQDKL

EEIANEARQLSGNDVFVFPTDISKADQIDRAFNEIISHVDHIDYLVNAAGFGVFKEFL

ETSPQVVTEMFQTNVLGLMYFTRLVARVMIDQKAGQIINFGSIAGIVPTTKTAAYSAT

KAAIIQFSNVLRLELKPFGVKVMTVNPGPVYTNFFNIADESGSYANNVQRFMLDPDDV
                     AWQVVHFFGSNRRELNLPLSLATLAKLYQIFPRIGDWASLKFASRK"
     CDS             35609..35935
                     /gene="lapA"
                     /locus_tag="OCPHGMPK_00742"
                     /inference="ab initio prediction:Prodigal:2.6"
                     /inference="protein motif:HAMAP:MF_01948"
                     /codon_start=1
                     /transl_table=11
                     /product="Lipopolysaccharide assembly protein A"

/translation="MNKQRNLVIGLIIVLVLVIFACLNTEPVAINFGFFQPKMPLIIV

LVIMLLLGALVSLLIGKGEKVSPDVKKALAKQKKELDNKYQSEIESKDKQIAELKKVN
                     NSEATK"
     CDS             36052..36243
                     /gene="rpmF"
                     /locus_tag="OCPHGMPK_00743"
                     /inference="ab initio prediction:Prodigal:2.6"
                     /inference="similar to AA sequence:UniProtKB:P07840"
                     /codon_start=1
                     /transl_table=11
                     /product="50S ribosomal protein L32"

/translation="MAVPARKTSKQKKRSRRGHIKLTTPAMHYDATTGEYRLSHRVSP
                     KGFYKGRQVANENKQQNND"
     CDS             36303..37868
                     /gene="cpdB"
```

```
                     /locus_tag="OCPHGMPK_00744"
                     /EC_number="3.1.3.6"
                     /inference="ab initio prediction:Prodigal:2.6"
                     /inference="similar to AA sequence:UniProtKB:P08331"
                     /codon_start=1
                     /transl_table=11
                     /product="2',3'-cyclic-nucleotide
                     2'-phosphodiesterase/3'-nucleotidase"

/translation="MKLVILHSSDTHGFLMPTDYQNKDNYDAPISLSRVSSVVKSEKE

KYGADNVLVTDSGDCLQGSPLASYTQKLPEDEGLRKFTEAYNEVGYDTRALGNHDFNY

GLDYLSYYVDNNRAPFINDNVLDADTNVPFFGKEYTIVEKAGLKIGIMGITTQYISHW

ESEDHIKGLKFVSGYEVLAKLAKKLRPQVDILCALYHGGFESDPLTRKATEPHTGENE

GYKILTEIPEIDVFLTGHQHRRLNLVTRNTAIVQPGYRGEAVGKVIIDFDKDTNGKVK

INDMTTELIDSKDYAPDPAIEKIVKSLDEETQKWLDQPIAHLSEPAPIDDAIKGRIEG

APFINLIQQMQLWFTGADVSATAIMSESAHGFSKNVTMREVLLNYPYANQLCIVKLTG

KELKEIIEYSAGFLEKDKDGKIKFIDRWITPKPMLYHFDLFYPVKYEADLSRPVGHRL

TKVTLNGKDLEDDKVYKLAVNNYRALGGGFYPAYSMDKIEKIFDQDYVQMFSEYLTHG
                     DIKVDTAKNYKFY"
     CDS             complement(37933..38133)
                     /locus_tag="OCPHGMPK_00745"
                     /inference="ab initio prediction:Prodigal:2.6"
                     /codon_start=1
                     /transl_table=11
                     /product="hypothetical protein"

/translation="MRYIIITMVWSCIFCEVIGFIAASLTQMQFNPMQSLIIGAVFGLL
                     FAIIIPAITAKSYKDKSKYNKL"
     CDS             38236..41352
                     /gene="dnaE"
                     /locus_tag="OCPHGMPK_00746"
                     /EC_number="2.7.7.7"
                     /inference="ab initio prediction:Prodigal:2.6"
                     /inference="similar to AA sequence:UniProtKB:Q9XDH5"
                     /codon_start=1
                     /transl_table=11
                     /product="DNA polymerase III subunit alpha"

/translation="MGAVSLQNLSSFTLLESPTKVKDLAENAKKKGYSALALTDVNIT

YGLVNFYKAAKETGIKPLLGMQLRINGLVDQANKYDLIVIAKDDQGYKNILRLSSAVN

LLTENGEKENVLELEELKKYLGHLVIITPSNIHSELKMLQANNPKMGANYIRTLKDSV

PTSSSIYLGVYADQGQQEYINYLRSLATQFELLLTAVEDGQYLNRNEQFLRRTLQAIK
```

SNTHLENVEQLAKQAGSHYLKNSEELQVNYRKFGIEDALENAEKIGQICSAQITFQDP

QLPKFKQNKFPTSKEYLHSLAQNGLAKRFKGRIPEKYQARLDYELKVINEMGFDDYFL

IVWDVMNFAHSVHITTGPGRGSAAGSLVSYALRITEVDPLEYNLLFERFLNPARQQMP

DIDLDIPDNRRDEVIKYMFEKYGMNHAAQILTFGTLAAKQVLKDVCRVFGLNKVETYR

WLDAIPHAKGKITLAEAYQKSKELQLLVNTNAFSKILFATAEHLENLPRHYSIHAAGL

VITDDSLAEIVGLQAGPLGIPVTQQTKLNVESLGLLKIDFLGLRNLTILGNIIAALKS

EGVEIDPNQIPLNDQETLALFQRGDTDAVFQFESDGIKRVLEQLHPDSFEDIVAVNAL

YRPGPMNNIGHFINRKHGKEKVQYPDPSLKKILGPTYGVLVYQEQVMQTAQVLAGFSL

GEADLLRRAMSKKNADVIQKEREKFIQGAVKLGRRKEIAEQVYDYIAQFANYGFNRSH

AVAYSKIAFWLAYFKVHYPGAFYLSLLNSNIGNRNKIAQYLMQAQEAGIKTLPPDIEN

SKADFSLENGKILVGLKAVRGLRSDLLKQILEIKRPIKSMTDFLWKIDNNLLSADAIA

NLIKAGAFDRLAPNRNELLKINKDLVESVKMAGSNLSLFETLEPKIEEEKMPTAAEKS

AMEVEAMGFSTGINPIIAVQKYARKYNAKRLQAFENNEQGIAVGKLMKIKQITTKKGD

NMAFAVFSDSSGDKDFTIFPQVWEKVGENLKIGEIYLLQVKTQSDRFSPTKTQFLLSN
ARKVNFKD"
CDS 41527..42486
/gene="pfkA"
/locus_tag="OCPHGMPK_00747"
/EC_number="2.7.1.11"
/inference="ab initio prediction:Prodigal:2.6"
/inference="similar to AA sequence:UniProtKB:P80019"
/codon_start=1
/transl_table=11
/product="ATP-dependent 6-phosphofructokinase"

/translation="MKRIGILTSGGDAPGMNAAIRAVTRTALANGIEVCGIRYGYAGL

VAGDIFQMTSETVADKINRGGTFLYSARFPEFKEEEVQLKGIEQLKKHGIDALVVIGG

DGSYHGALKLTRHGYNAIGLPGSIDNDIPYTDFTIGFDTACNTAMEAIDKIRDTATSH

QRVFVVNVMGRDCGDIAMHVGVATGADAIVIPEEPYDIKEIAENLKQGFANGKKHGIV

VLAEGVMDANKFKDELLKYGDFDARANILGHMQRGGSPTTRDRVLASEMGAYAVKLLL
EGKGGLAVGIENNKLSHHDILDLFDAKHHGNYALYSLNKDLAK"
CDS 42521..44290
/gene="pyk"
/locus_tag="OCPHGMPK_00748"
/EC_number="2.7.1.40"
/inference="ab initio prediction:Prodigal:2.6"

/inference="similar to AA sequence:UniProtKB:P34038"
/codon_start=1
/transl_table=11
/product="Pyruvate kinase"

/translation="MKKTKIVSTLGPASNDVETITKLAEAGANVFRFNFSHGDHEEHR

ARMNMVREIEKKTGKLLGIALDTKGAEIRTTEQEGGKFTINTGDTIRISMDDTKKGNK

DMIHVTYDGLYDDTHVGGHVLIDDGLVDLLIKEKDDTKRELVCEAQNTGLIGSKKGVN

APGVEIRLPGITEKDTNDIKFGLKEGINFIFASFVRKAQDVLDIRQLLEEAHCEYVKI

FPKIESQEGIDNIDEILKVSDGLMVARGDMGVEIPFINVPFVQKELIRRCNALGKPVI

TATQMLDSMQENPRPTRAEVTDVANAVLDGTDATMLSGESANGLYPVKAVKAMAEIDM

RTEKQLAKRNKLALQRFEEYKGSNVTEAIGESVVRTAEELGVKTIIAATESGYTARMI

SKYRPNADIIAMTFDEKIEHSLGVVWGVKPMLVEKPKTTEEMFDKAAELAKETGLAKD

GDLVIIVAGVPLGETGTTNLMKLQLIGTQLVKGLGVGEQSVIGKAVVATSAEEANSKV

QDGDILVAKTTDKDYLPAIKKASGMVVEASGLTSHAAVVGLSLGIPVVVGATDAVEKI
KDGSKITVDARRGAVYQGEATNL"
CDS 44404..45297
/gene="cvfB"
/locus_tag="OCPHGMPK_00749"
/inference="ab initio prediction:Prodigal:2.6"
/inference="similar to AA sequence:UniProtKB:Q7A5Q1"
/codon_start=1
/transl_table=11
/product="Conserved virulence factor B"

/translation="MLGEIRKGKVTDENQTAFYVQVDGVTFELKKIEVTQDEPIHLGD

EVQGFIYENKDKKKEMTQFYPFAQKDQYGWATVTEVRRDLGVFLDIGLNDKDVVVSLD

DLPLEKDQWPKKDDRLLVRLETDDKERIWAKMAEEDVFEQLAANFPAHLENKNMSGTV

YRNYEVGSFVITDQYYLAFVHKSEMFRPLRLGQKIKARVIGVSQYGRLNLSVLPRGFE

EIDDDAQMILVSLRREATKTLPFYDKSDAQEIKNHFGISKSAFKRALGHLLKNRLITE
DKDAGTISLVEKDESDTDDEG"
CDS 45269..46177
/gene="xerD_1"
/locus_tag="OCPHGMPK_00750"
/inference="ab initio prediction:Prodigal:2.6"
/inference="similar to AA sequence:UniProtKB:P0A0P0"
/codon_start=1
/transl_table=11
/product="Tyrosine recombinase XerD"

/translation="MNQTQTMKDNLEDYLRFSQVERGLSPNTIISYRTDLEEYLDYLQ

DQNETSWEVDYLVVDAFLATQKDKGKATTSISRMISSLRKFYQWLLRQDIIERDPLIK

IDSPKSERRLPTALSEEEVDKLLAAPDTNTPLGIRDRAMLELLYATGMRVSELINLRT

GDIHSDLKIIRVLGKGSKERLVPITEVALSWLEKYQKDVRDAQVLKSGQFTDVIFLNN

HGHQLTRQAVWQKIKKYCQLVGITKNVTPHTLRHTFATHLLENGADLRVVQEILGHSD
ITTTQIYTNLTQKHILEVYNQAHPRA"

```
     CDS             46183..46545
                     /locus_tag="OCPHGMPK_00751"
                     /inference="ab initio prediction:Prodigal:2.6"
                     /codon_start=1
                     /transl_table=11
                     /product="hypothetical protein"
```

/translation="MISKYKKDYEKTVMGYLSYLPDFKNIKNLQEEMKLYTSDSNQFE

VLIYKEKGNARGIIGIQEEKDFIIIRYLSLDPTMRDSETKRLVMKDLKHLYPDKMITA
LPDWAFLLSYLKEDKTDE"

```
     CDS             46538..47281
                     /gene="scpA"
                     /locus_tag="OCPHGMPK_00752"
                     /inference="ab initio prediction:Prodigal:2.6"
                     /inference="similar to AA sequence:UniProtKB:P35154"
                     /codon_start=1
                     /transl_table=11
                     /product="Segregation and condensation protein A"
```

/translation="MNNVDELTLDLPNFTGPLDLLLHLIRSQKIDIYDIPIAKITGQY

LANLARWQTLDLQIAGEYFVMASTLLRIKSQYLLPKNDFVEEDQYQEDPRAELVEQLV

QYSVFQRIAEYFKKRDEEMPITVAKDPSVSPKKEVEPLPLGEITSDELANTFKVVLER

FKLRKPQVGQIEVHETSIEEMTTFLKDKLHHRKKASFFDCIKNFQDLDQVIGLFLAVL
ELCRDHKILVKQNRDFGDLELEKVETNGK"

```
     CDS             47271..47861
                     /gene="scpB"
                     /locus_tag="OCPHGMPK_00753"
                     /inference="ab initio prediction:Prodigal:2.6"
                     /inference="similar to AA sequence:UniProtKB:Q97NX6"
                     /codon_start=1
                     /transl_table=11
                     /product="Segregation and condensation protein B"
```

/translation="MASKEAQLEALLYAAGDDGLETDNLLQLLEISPSALQELANHLK

DRLKNDENSGLQLICINHTYKLTTSAECGDVISKFFQKDLSKNLSQSALEILSIIAYR

QPITRVEIDDLRGVNSSGALQTLVWRGLIKVNGKKDVPGHPNLYVTTDYFLQYFNYES
LADLPVIEEFESDDNPVNLFDQDERNSKEINFDEGE"

```
     CDS             47863..48582
                     /gene="rluB"
```

/locus_tag="OCPHGMPK_00754"
/EC_number="5.4.99.22"
/inference="ab initio prediction:Prodigal:2.6"
/inference="similar to AA sequence:UniProtKB:P35159"
/codon_start=1
/transl_table=11
/product="Ribosomal large subunit pseudouridine
synthase
B"

/translation="MAERLQKVIAQAGVASRRKAEKMITAGQVTVNGKRITELGTKVE

PSDRIEVNGVPIEKETLHTYLFYKPRGVISSVKDDKGRKTVVDYFEDVPYRLYPVGRL

DYDTSGLLLMTNDGELANKLMHPRNEVPKVYVAKIKGLLSPEDIHSLTHGVRIDGRKS

APAKLKILKTDEKKNTQIVQLTIHEGHYHQVKRMFEAVHHFVEKLSREKYAFLTLKSL
TSGEYRELTHEEVSRLNHLSD"

CDS 48644..49384
/locus_tag="OCPHGMPK_00755"
/inference="ab initio prediction:Prodigal:2.6"
/codon_start=1
/transl_table=11
/product="hypothetical protein"

/translation="MKENKSQDNQEPKGPYKHFERPTTSRSAMHHRHRSEPAPEPTPS

RPDKNSKGTNHSQLWAGLIIVAIILIALIPIVSSKLHSNSNNLAEKSVKVSKSSSKSH

KSKSKKPKRSSSKKSKSKSSVKKEEKKSSSQSHDVTPAVTSQSQSSQTSQSSIQSYAS

SASQTTTQDQTQQANQNSYQNNQNTWQNNSGNSSSQNQAPSSYTVQEGDSLSKIAREN
NTSVHHLEQLNGLESADSISIGQSIKLK"

CDS 49646..50239
/gene="ribU"
/locus_tag="OCPHGMPK_00756"
/inference="ab initio prediction:Prodigal:2.6"
/inference="similar to AA sequence:UniProtKB:E5QVT2"
/codon_start=1
/transl_table=11
/product="Riboflavin transporter RibU"

/translation="MQRKTSLSHVRLAYWLAWAMIGALAFLIMKIEVPIIPGFDYLKM

DFSDSLVALSTLVFGPLGGTMIALFKSLLSLFISGFNPISMIGQLAAFLASLAYILPF

YFISKKHEDKVKYQIFGLIVGTLALTVVMSLANYFVLTPMYISLMGFKLNSSLLTYVV
SAIIPFNLIKGLINSIVVLILAKTLLPVLEKFVRRNF"

CDS 50345..51010
/gene="cmk"
/locus_tag="OCPHGMPK_00757"
/EC_number="2.7.4.25"
/inference="ab initio prediction:Prodigal:2.6"
/inference="similar to AA sequence:UniProtKB:Q97PK6"

/codon_start=1
/transl_table=11
/product="Cytidylate kinase"

/translation="MQVAIDGPASAGKSTVAKIIAHNLGYIYIDTGAMYRACTLIAHE

NHVDYGDEKAILDLVDKSTIEFKQEDGEQKVYVNGKDVSIAIRTPEITENVSQVSALK

SIREKMVELQREMAGKHNVIMDGRDIGTTVLPDAEVKIFLIASVASRAKRRFLDFQEK

GIHQDLKDIEHDIEVRDYKDSHREISPLKKAADAIELDTTNLTIDEVVAKISEIIQKK
QKN"

CDS 51073..52272
/gene="rps1"
/locus_tag="OCPHGMPK_00758"
/inference="ab initio prediction:Prodigal:2.6"
/inference="similar to AA sequence:UniProtKB:P50889"
/codon_start=1
/transl_table=11
/product="30S ribosomal protein S1"

/translation="MTDNSNQFLDALKEMQGVEVGNIVNVEVLSVEDGQIAVGVENAG

VEGVITKREFTNDRNADLNQLVKPGDKFEALVLRRAGGDKENGEFFFSVTRLKEREAY

KELEKVFEEGKTVEGTVTGAVRGGLLVDVGTRGFLPASLISNRYVSDLKPYIGKKMNL

KITEIDPNKNRLILSRKDLIEKEREEAFENVASQLVVGDTVEGKVSRLTGFGAFVDVG

GVDGLVHISEISYKHVDKPSDVLKAGQDVKVKVIGIDDDRHRISLSIKQTLPSPFEEA

TEGLHEGDIIEGEVKTLTSFGAFIEVADGIQGLVHVSEIANKHVDKPSDVLKVGQTVK

VKVLSVDPSDRRISLSIKQADPNAAKSENSRPRRRQDSVADKYMNDNDNGFALGDIIG
DQLKDKD"

CDS 52349..53656
/gene="der_1"
/locus_tag="OCPHGMPK_00759"
/inference="ab initio prediction:Prodigal:2.6"
/inference="similar to AA sequence:UniProtKB:P64060"
/codon_start=1
/transl_table=11
/product="GTPase Der"

/translation="MSLPIVALVGRPNVGKSTIFNRIINSRVAIVEDKAGVTRDRIYA

RAEWMGHEFILIDTGGITLDSGEIEEQIKAQAEIAIDEADVIVMLGDVTQHMTNMDET

IAKMLYRTKKPIILAVNKADNPEQRTDIYDFYSLGLGDPIPVSGSHGTGMGDLLDAIV

GEFGDKANQHEDDSIRFSVIGRPNVGKSSLVNAILGEQRVIVSNIEGTTRDAIDTTFT

NDGQKYTIVDTAGIRRRGKVYEKTEKYSVLRAISAIEESDITLLVLDASTGIREQDKH

VAGYAHDAGRGVIIVVNKWDLPKKDSTSMKDFENTIRQEFQYLDYAPIVFVSAKTGQR

VPDILKLVKEVHDNQTRRIKSSVLNDLLLEATRITPTPLVNGKRLRIYYMTQVAVTPP
TFVVFVNDPELLHFSYQRFLINQLRQNFDFVGTPIKILARKRK"
CDS 53820..54095
/gene="hup"
/locus_tag="OCPHGMPK_00760"
/inference="ab initio prediction:Prodigal:2.6"
/inference="similar to AA sequence:UniProtKB:Q99U17"
/codon_start=1
/transl_table=11
/product="DNA-binding protein HU"

/translation="MANKAELVSEVAAKTDLTKKEVAAAVDAIFSSIQEDLAKGEKVQ
LIGFGTFEVRHRAARKGRNPQTGAEIEIPASKVPAFKPGKALKDAVK"
CDS 54196..55455
/gene="lapB"
/locus_tag="OCPHGMPK_00761"
/inference="ab initio prediction:Prodigal:2.6"
/inference="protein motif:HAMAP:MF_00994"
/codon_start=1
/transl_table=11
/product="Lipopolysaccharide assembly protein B"

/translation="MTYSEQLLDAIQNHDFSDNHILLKKALENDDPEVLASLAENLTD

LGFTDLSKEVYRYLIAQFPREDLFKVYLAEILLNDGDEDDGLQLLYDVKPESDAYIES

LLDLADYYQSNGLIETARQKLLEAHKLAPEEDAINFGLAELDYLSGDYGEALGLYREL

AKENKTFGEVVLSQRIAACLAKLGEYEEAANEIKSHENDILSIDALYEAGLIMLSAGD

NKAAIKYLDQVIETQPDYVNAYPLLAQAYAAEDNNEEVLRTAQTGLSYNELDEVLYSL

GAKAAANLNQLDEAERLLKKGLEIAPDNSDLRLQLSNLYLHQHQDEANIALFKDLDDE

ELEPQAHWNMAISYQRLEDYDKAKSEFLLAYPAFQKNASFLRQMINLFYELREIPTTK
ELIKKYLQVQPDDIDMQDMLDELNSEE"
CDS complement(55531..56685)
/locus_tag="OCPHGMPK_00762"
/inference="ab initio prediction:Prodigal:2.6"
/codon_start=1
/transl_table=11
/product="hypothetical protein"

/translation="MILNKLFQPRRHIKEFHIFIDESISFKSQSDNTTEKFYSGFLVI

PTFQLKDLYNRYFQKIYHSRYKKEKKSVNVSDTMNRLALDIVKPFLTNAYIYQRPTYH

KHDTTLDNTIMQQVLMLQSYIYPIKRIIEDLRQQNPVVNLKLNIYLDQTNLNSQLESE

YFSCKLLSQIIKNLGEENNVFFTYQTCDSKQELGIQMADMFVGAYRKQEKYAANDDNT

TLIPFNVTSMIDTLAFQNQDTFLKLYGLVSLTMKGPTKKDLTTFKKFLKNKLDSDSYQ

YPKRNNFNTIRAKLTKAKNKTYQEEFNRKLVRILVSLNKSMIKIYGVKLPKAYIKNLS
QKYSRNNCNRTIANMQRNINKLQKIQLSIFKIFKLKLSLRRFNKQLDKLL"
CDS 56896..61302
/locus_tag="OCPHGMPK_00763"
/inference="ab initio prediction:Prodigal:2.6"
/codon_start=1
/transl_table=11
/product="hypothetical protein"

/translation="MSNFEFLNKDVLTEQYYQKAAEAEMSYAMGLYSNVLVSARTIAE

NLAREVADENYLVVDDRETFDNVLKRLKLGQYIDKYALQFFYDVKNVGNTAAHTLTTS

NQEEALTALKRLYSLCVWFVGNYYDEKVDITEFQEPKKDVLYQTTIQPISNAEKNLIY

IQTADNSLGKFSVYEGNQKVGKTGVGDLAEDNSDNSPYLRDWAKKRIKQYMNTSGVPV

NLEWAELAYRKSDGLWFSDHDVHRVLERSGVKHSHDLSGDEWFETDLETAKKAIKAVK

EGKDSLDKISNVEEKIVLRPEQQEAVNKTKLGFKSGQKMLWNAKMRFGKTLTALKLVR

EQKYKKVLIMTHRPVVDGGWFDDFIKIGMPDAGYVYGSKNKGHQNIKELEDLNKPYVY

FASIQDLGGSEIVGGKVSDKNRDLFAVDWDLVIVDEAHEGTQTELTQNILNLVVKKHT

KELDLSGTPFNILSDYDDEHVFTWDYTMEQEAKENWNKEHPHEDNPYSALPRVSMFTF

EMNKRFNDPRFTREGLGKYTFNFKEFFRTDKDGKFVYESDVKRFLDNITNPGTANYPF

STREFRNRLRHTLWVLPGIKEANALEELLKKHPVFGTEYNIINVVRNDRSDTELGTDN

DVDAERKEIAKADKAGKKTVTLTVRKLTTGATVPEWTGVLFLSNTNSAMQYLQAAFRA

QTPYSSPKFGKKENCYIFDFAPDRALTVMAESTSLATGVGKIQTKEQRAKMAKLMNFL

PILGESDQQMKPYKVDRLLSQLKRAYAEKAVRTGFDDDSIYSDELLLIKDADLKDFND

LKGIIGTTKKEKAPTKIDVNHQGLDEEEYDMAEKARKKKTRERSQEEQDAIDKVKKLK

KQRKTMISILRGISIRIPMMIYGMDIKFDENVSIKKFVDKIDDVSWEEFMPKGITKAL

FNKFVKYYDADVFIEAGKIIRRQVKELDKADPLERVEKIAEIFNTFKNPDKETVLTPW

RVVNMHLGKTIGGLSFFDKDYQYSYEDGNVVRRWIDTEYTDKIFNENSQILEINLKTG

LYPLYVATSIYWKEFNKLNDQTAGKFGFEDELMIWQRILRENIFLVAKTPMAKEIARR

TLAGYHDDWDINSEYVENIVEDSKADVNKEAKKIEGMFGNMKFDVVIGNPPYQQEAKG

ASTRDEPIYPQFMDLSYQLSNLVTLITPARFLFNAGQTSKAWNETMLHDPHLKVVFYE

QNSSKVFPNTDIKGGIAITLRDATKDFGEIGIFTSHPEMESIVNKVWKYKQHSFSKMV

SSSGLYRFSNLVFEENTKLKKSVSSGTGKKITSKIMSENNDIFRKDRVLDTDVSLIGL

VRNRRTHRFILRKYLQDNDFIDKYKVLIPKANGSGKLGEIISTPIIGKPSIGVTDTFI

SLGPVDSLEDAEAILKYIKSKFGRALLGVRKITQDNPKSTWQYVPMQNFTSDSDIDWT
KSISEIDQQLYKKYNLSDDEINFIETKVQEMD"
CDS 61302..62276
/locus_tag="OCPHGMPK_00764"
/inference="ab initio prediction:Prodigal:2.6"
/codon_start=1
/transl_table=11
/product="hypothetical protein"

/translation="MLNIVDQMFQYCEIPEDGQQYFFNDSIENKKIIVSAFKHNSFMS

KYIYMPILSLKFVFNTEKHKRIYHYLNHENLTSILKCQYFYVGNQQEMNDSLEGKYSW

DLAFDLLNEKDNSNQIQKMFEEIKRRRPFDSYIWSFTLDENNMALQNYGDLAVVMNPQ

DTFESLARKYTNSDFENMNTGNAYILPLHVVYDYQTQVKYLSNTLDIWLDSINSGNYG

DYQDASLAMYLYSLVFKSPKYHQEEEIRYVINKIPDINGNGYDIKINGKKKLEAPFLP
SLCSEIIVNHKADHVKNESIDTIQTKVKSMIENYGFNNTIVRKTDLEY"
CDS 62279..62959
/locus_tag="OCPHGMPK_00765"
/inference="ab initio prediction:Prodigal:2.6"
/codon_start=1
/transl_table=11
/product="hypothetical protein"

/translation="MSEKIIKSSDRVKDIGEVFTPKKTVNFMLDQLEIKEKINSLTAT

FLEPSAGEGAFLVEILRRKLKYAKTQSSSVEEMQNNYLQVLSTLYGIELMEDNIEMLV

MNMNNVFRDMYFNTFKAEDQNLKILKSAQTIISANMSQGDALTRKTATGKPIIFSEWK

PVGKNKVQRTEYTFDSIVEGGGPLGSVQKQYEQMDLFAEDNSESNDENVRHYLPVKWE
DIYKRLVE"
CDS 63065..64072
/locus_tag="OCPHGMPK_00766"
/inference="ab initio prediction:Prodigal:2.6"
/codon_start=1
/transl_table=11
/product="hypothetical protein"

/translation="MKEKAYQASNNIWLSSFTIQFLINFYFQKYDSDTYLRPNYHPLL

YIDIAELVISIVLSMGTVIFDYFKGKTINLTIKKFYMESDIENDIFRLVCIFELLGFI

YILLHDMCYLKLINRTTFYNLFGLFLIVIVLYFYYLSDKIKKMEWSSLSGNSKVSWKY

LIQNNHFDQYKGEIILSQLLKLLRVENVEIKVSANDYFNIYKKFVEKLQFLSNEKLIE

LRNYLQMQNRDDFKWSIQKILSANLGKVCISLLVSMLSISSIVQLGSNIKKEMILNGI

LSIMHYYILFYVSLLLIFYLIKLLIYLVFIPHQRKVRNENIESYLLSAIQTVLDLRSK
R"
CDS complement(64498..64761)
/locus_tag="OCPHGMPK_00767"
/inference="ab initio prediction:Prodigal:2.6"
/codon_start=1
/transl_table=11
/product="hypothetical protein"

/translation="MSIHTGFPFGNYHYDIAGPRIWDVPIVIMPAYFGMGYFTWILSL
VINNQYEKKLSGIKTFLIPFTATFIMVMWDVVLDPTASTLNGN"
CDS 64920..66731
/gene="ifcA"
/locus_tag="OCPHGMPK_00768"
/EC_number="1.3.5.4"
/inference="ab initio prediction:Prodigal:2.6"
/inference="similar to AA sequence:UniProtKB:Q9Z4P0"
/codon_start=1
/transl_table=11
/product="Fumarate reductase flavoprotein subunit"

/translation="MEQETKAPVINWKANYDVVVLGFGGAGATAARFAADNGAKVLIA

DVAPYGHEGGNTRYSAQLLGTGTDYDKLKEYYLGLTHPMDLPEDMVETYVRGMCNMRE

YVKKYLGVEPVSFIKDFSKIPKEYRIGVTQEYPELKGHETYDYTLVHNGWFDAALWKL

LRQKVLDRKDKIDVWYEARASHLIQDPISKVIKGVQIEKLGQKINVLARRGIILTTGG

FENNKEMIQNYLAAPKLAPLGTIYNKGDGISMAQEVGAKMWHMYNYESLGLLHGLSFD

TPEGERSKLVLSWPELNHGSVFMVADDGKRYFNEAGPNRHGHIFDHGIWRVPRSHEKP

YIIFDQKQYETIEAGQIPYDKFNEKLIKADTLEELAKKTGVDANGLKEQVEMFNRFVE

NGHDDQFGRDIKSMAKFAEDGPFYAIKMAYNVLNTQGGPERNTKAEILDVNNKPIPHL

YGAGELGGINANQYQGGGNLAECLIFGKIAGEQAANVTDEVTDEASDKYNGINELVDG

DKVKDIKLDKDQYLGESNAGIGGKLVVRVTYDNGIKNVEIVQNHESEDVGKRALKVIP
ERIVKENTPDVDAVSGASATSRAISEAVRKALKNVKE"
CDS 66886..67827
/gene="mrr"
/locus_tag="OCPHGMPK_00769"
/inference="ab initio prediction:Prodigal:2.6"
/inference="similar to AA sequence:UniProtKB:P24202"
/codon_start=1
/transl_table=11
/product="Mrr restriction system protein"

```
/translation="MIDYRTLKHSDNGMPTWDAFLGPVLQVASTKREWIGKELDQAVL

DEVDLPEELANLRYASKYHDLIAYNRVGWALSELKLSGLLNSPRRSIYRISETGKEAL

AKYGLNITRDFVHSQSAYIEHQNKLKNKKEIDNDITEDQTFELTEKQIKDWFDKQSSD

LSEQLIAKLRSTDPYQFEHMMVRLLNEMGYKGTNGQSVVTQKSNDGGIDGIINQDPLG

LRTVYVQVKRYSEKNIVGSPEINSFFGALRRKQADRGVFITTSSFTNDARDAAKQLNI
                     ALVDGEMLTNLMIQYKVGVQIRQNYELYNIDDDFFEE"
     CDS             67839..68501
                     /locus_tag="OCPHGMPK_00770"
                     /inference="ab initio prediction:Prodigal:2.6"
                     /codon_start=1
                     /transl_table=11
                     /product="hypothetical protein"

/translation="MKYESYLEMPREPKSKDKKIWQDYFKKFQQRFIHNERVFMLYRT

TSFEQKSIKKVNNEYKYLLENELIILSKAEEKYFRLFKEPYKISQQIKKEKLQYWIPY

LTRVDFVNMGAYMGNDVSLILMDNRYLVIEGRLDKDYKVIKAISKQKLIKSLINLEIG

YWSEVYHSSEAIVEDGSSWILKFKCIKNKKYKEFVFYGDNCYPYNFDDFAQIAYIKDF
                     VY"
     CDS             complement(68743..69957)
                     /locus_tag="OCPHGMPK_00771"
                     /inference="ab initio prediction:Prodigal:2.6"
                     /codon_start=1
                     /transl_table=11
                     /product="hypothetical protein"

/translation="MQKYVNRPEYLDFLKRWQNKQIIKVVSGVRRAGKSTLFYLFRNY

LKEHEGVKEEQFISINFENMKYDNLRNAQNLYDYLENKIIPDQMNYIFLDEIQHVKEF

ERVVDSLFLKDNVDIYLTGSNAYFLSSEIATLLTGRYVELKILPLSFSEFVAWHKQND

KFNNNDDMFNKYLESSFPYTLFIDTPKERNEYLQGVYSTIVLTDIVSRLNIKDTPVLE

RVIQNLYSSIGSQVSINKITITLKSAGYSSSNKTVDRYLGGILDSLLMYEAKRYDIHG

RQLLNSQSKYYGVDMGIRNILLPDHQEDFGHIIENIVYLELLRRNKAVYVGESNKYDV

DFVGINDMNELSYYQVALTTMDEKVLKRELRSLEQIRDSYPKYLLTLDTINRTANYNG
                     IQKLNLIDWLLN"
     CDS             complement(70160..71050)
                     /locus_tag="OCPHGMPK_00772"
                     /inference="ab initio prediction:Prodigal:2.6"
                     /codon_start=1
                     /transl_table=11
                     /product="hypothetical protein"
```

/translation="MTKIFNKQLFKQLFFITLGCSIYAFSLDAISIPNKLADGGISGI

ALLLRYWFHINPGLSTLLLNIPLIIIGYRFMGKRLLALTIWGTLCLSFFLSFWLHIPI

INQLNLEHDLFIAGVLAGLFSGFGIGIVFKYGGTTGGTDIIARILDLKLGIPVGKSLL

GLDAFVLTISLTYLDIKHMIYTLLASFVLARITDSIQAGAYAARGLFIISDKYEQIAK

MIDVQLDRGYTFLNAEGGYDHSAKKMIYCVVSPREISQVKELILREDPNAFVSVIDVH
EALGQGFTYERKKRHIFFRR"

```
     CDS             71106..72302
                     /gene="cca"
                     /locus_tag="OCPHGMPK_00773"
                     /EC_number="2.7.7.72"
                     /inference="ab initio prediction:Prodigal:2.6"
                     /inference="similar to AA sequence:UniProtKB:Q7SIB1"
                     /codon_start=1
                     /transl_table=11
                     /product="CCA-adding enzyme"
```

/translation="MKITNLPEVFTAALPVLKQINEAGYEAYFVGGSVRDLLLNRHIH

DVDIATSAYPMEIKQIFKKTIDTGIKHGTVTVLYEGESYEITTFRTESGYQDFRRPDH

VTFVQNLSEDLKRRDFTINALAMDVAGNVIDHFDGLGDLDKHLIRAVGKAENRFHEDA

LRMMRAVRFMSQLQFTLEPETEQAISDNHELLSKISVERIRDEFVKMGIAPGSQKAFQ

IFLDTGLSEEVPGFKGKKENLALYPQLNFSPTTEANLWALMIILLKLPNEKIPSFMRM

WKNSNAMERQVADIVAFFDLISSRAPNNYDLYQAGLETIASTIDLAHILGQPINGSAL

VDGYEALPIKNSHDLVIDGHFLLKNGIPAGPRVGLLLEEIKKAVLEGVISNNEAAITE
FLSLNN"

```
     CDS             complement(72350..73015)
                     /locus_tag="OCPHGMPK_00774"
                     /inference="ab initio prediction:Prodigal:2.6"
                     /codon_start=1
                     /transl_table=11
                     /product="hypothetical protein"
```

/translation="MSFKQLWQEPKNRSKTYNILNNIFSAITHGIGFCLAVAGLVILI

VKAAHTGSAVRVTAFTLYGSSLIILYLFSTLYHSLIFTRARRVFQIFDHSSIFILITG

TYTPYTLVAIGGAKGWVMFGIILALAIFGILFYIFNQGKHVILDTILYVLMGWIVIIA

SSTLYPVLGPTGFWLLVWGGIAYTVGAILFSMRGVPYIHVIWHMFVLLGSILMYFSIL
FYV"

```
     CDS             73157..74014
                     /locus_tag="OCPHGMPK_00775"
                     /inference="ab initio prediction:Prodigal:2.6"
                     /inference="similar to AA sequence:UniProtKB:Q97QT7"
```

/codon_start=1
/transl_table=11
/product="DegV domain-containing protein"

/translation="MAKIKVVTDSSVQLTPEEIDKYGITVVPLTITIDGQTYTDGVDI

SREEFVKKMDASKELPKTSQPSIGVFEKVFKDLTDDGSQVVGIFLARSLSGTIEAARQ

AADLIGKSKEVTLIDSDLTDRAEAYQVLAAAKDAKEGKSLEEIVAHVEQLKEHQKLYM

MVVNLNNLIKGGRLGPLAGKIATLLNIRIELHMPGGHLEVAKKGRGKKFSKNCDKRVL

KDIEEHKQEIKEVAISYVDTPEDMKVWTEKIKEINPDIKVLTRVTSPIISTHAGSGAY
AVFYTMEEY"

CDS 74014..74241
/locus_tag="OCPHGMPK_00776"
/inference="ab initio prediction:Prodigal:2.6"
/codon_start=1
/transl_table=11
/product="hypothetical protein"

/translation="MAYRESFYRFLMTQRNPGSVDDIAQFANNAQHDSSFPKQEEDYE
KLSDYLELNAGYLPSMSVFDKAYQLYLDNMN"

CDS 74249..75697
/gene="ctpA"
/locus_tag="OCPHGMPK_00777"
/EC_number="3.4.21.102"
/inference="ab initio prediction:Prodigal:2.6"
/inference="similar to AA sequence:UniProtKB:O34666"
/codon_start=1
/transl_table=11
/product="Carboxy-terminal processing protease CtpA"

/translation="MAEVEPNNSHQKKTTKNKKKHLPKYAQFLLTGIAGAAIGAGLTF

GIMEVKELKSPFYQVEKVYEQLQGSYYKKVSSQTLRQGAINGMLDSLNDPFSEGLSGA

NQEQVNNILEGSTFGGVGIQMAVRNNKVVVDSIVANSPASKSTIKPGDEILAVDNKKV

SAAQFTKVASLVRGKVGTKVTLKLKRANSTFNVTLKRAKISQSSLTKRTEGNATIITI

TQFDVNTAKDLKSALKSINTKKYPKLIIDLQDNPGGEMNAALKSASYFLPNKKIIMQY

KDRKEKEVIRSDKNLSGGFHTSLKPIILINGNTASASEIFTAALVQNHCGVSVGQTSY

GKGTVQQVGQTEDSEYKYTVAKWLTPNGTWINQKGLKPTYPVSESPLAKLPQFQSMSV

LKKSMTGVDVATLQQYLTALGYLPKHVTGVFDDETKNAVIKFQKEHNLTTDGIVNGQV
QAQLYLAVAQKLQDDNPALKKALSLNLKDMED"

CDS 75699..76538
/gene="rbgA"
/locus_tag="OCPHGMPK_00778"
/inference="ab initio prediction:Prodigal:2.6"
/inference="similar to AA sequence:UniProtKB:O31743"

/codon_start=1
/transl_table=11
/product="Ribosome biogenesis GTPase A"

/translation="MATIQWYPGHMNKAKNQLEDNLDLIDVLIEVLDARLPVSSRNPM

IGQLTKKKPHIIILNKSDLADPIQTKKWTRYFQDEGNFVISMDAQHNTNMTSLFKIIK

LAGKKKTEKLIAKGASNPMIRVAIAGIPNCGKSTIINRMVGRNAAIVGDKPGVTRGQS

WLKTKTNVQILDTPGILWPKFSDQEVGYKLAACGAIKADVFHPDDVALFVIEFLKQNY

QKDLAKFAQATSEELENMSNPDLLLAMTNKYGMRDDYDKFSLFMLQRLRKGKLGRITF
DLR"
CDS 76535..77287
/gene="rnhB"
/locus_tag="OCPHGMPK_00779"
/EC_number="3.1.26.4"
/inference="ab initio prediction:Prodigal:2.6"
/inference="similar to AA sequence:UniProtKB:O31744"
/codon_start=1
/transl_table=11
/product="Ribonuclease HII"

/translation="MTITEIKNLLQGEVSSEQLEELRADERKGVQKLLISYEKRQLKR

AQALAQFQNRFSYEKEFWQKNQLVAGVDEVGRGPLAGPVVTAAVILPHNFDLIDVNDS

KKLSPKKRKALFPQILAKAVSVSVGLANNDVIDRINIYEADRLAMAHAVQGLKVKPAA

LLVDAMNVPLNIPQIKLIHGDAKSNSIAAASIVAKVFRDNLMDAYGEVYPEYDFKHNA
GYGTREHMEALKKYGPTPIHRRSFAPVSEYEK"
CDS 77339..78184
/gene="dprA"
/locus_tag="OCPHGMPK_00780"
/inference="ab initio prediction:Prodigal:2.6"
/inference="similar to AA sequence:UniProtKB:P39813"
/codon_start=1
/transl_table=11
/product="DNA processing protein DprA"

/translation="MNLKEFCLRLKLQHGVGTTTLGKIAENFTAGEEVTVDKIESLSL

KSNIQNLVLAAMRNDKFNSWIERIKLQCDVVTIFDSIYPEGLREMYNAPTILFTRGDL

SLLKKEITTIVGARQPTNYSRFVLKQLIPQLIEQDFVIASGLARGVDGIAHQETLKNN

GKTIAVVGNGLNHFYPQENKELQEEIVAKGLLISEYLPDTPPRPYRFPERNRILAGLS

KNIIITEARKRSGSLITANIALEENRNIFAVPGPVSSPLSEGPNELIAAGAYPLVNAD
FRNLL"
CDS 78250..80331
/gene="topA"
/locus_tag="OCPHGMPK_00781"

/EC_number="5.6.2.1"
/inference="ab initio prediction:Prodigal:2.6"
/inference="similar to AA sequence:UniProtKB:Q7A5Y5"
/codon_start=1
/transl_table=11
/product="DNA topoisomerase 1"

/translation="MPTKRKYKKNLVIVESPHKAKTIEKYLGRNYHVIASKGHIRDLP

KSQMGVDVEHDYEPKYISIRGKGDTIKELKSEAKKAKYVYLASDPDREGEAIAWHVAH

ALNLDPKEHNRVAFNEITKDAVKNAFKNPRTIDMDIVDAQQARRVLDRLVGYSISPIL

WQKVKKGLSAGRVQSIALKLVIDRENEIKNFKPEEYWTIDADFEKGKEKFKGAFYGIK

GKKQDLPNNEAVQDILKQIDKRKNFEVTKVVKKERRRQPAAPFTTSTMQQEANKRLGY

RTRRTMRIAQSLYEGVNLGKGSVGLITYMRTDSKRIANVAKHEASKFIHEEYGANYAA

IKPQHFKNDADAQDAHEAIRPTSAFRTPASVKEYLTTEEYRLYTLIWSRFIASQMTPA

VYDTVRADIEQNGVTFRTTGSKLKFAGFTKVYDNQKEKNNELPELNEGDKVKLKKTDD

RQHFTQPPARYTEASLVRALEENGVGRPSTYAPTIDTIQKRYYVKLEGRSIVPTELGE

IVDKLIEEFFPDIVNVDFTAQLEDDLDGVEVGKKNWIKVVDEYYKPFSKELDKADQQI

EKVQIKDEPAGFNCDICGAPMVIKMGRYGKFYACSRFPDCRNTKPIVKKVGVTCPKCG

KGEVIEKKSKRNRKFYGCSRYPDCDFVSWDQPIGRNCPNDGHFLVQKKNKKGLIILCP
NGDYREEPEEN"
CDS 80429..81745
/gene="trmFO"
/locus_tag="OCPHGMPK_00782"
/EC_number="2.1.1.74"
/inference="ab initio prediction:Prodigal:2.6"
/inference="similar to AA sequence:UniProtKB:P39815"
/codon_start=1
/transl_table=11
/product="Methylenetetrahydrofolate--tRNA-(uracil-5-
)-
methyltransferase TrmFO"

/translation="MVKNVTVIGGGLAGSEAAWQLAKRGIEVDLYEMRPKKNTPAHET

ANFAELVCTNSMRSNQLSNAVGLLKEEMRQLDSLIMKAADETAVPAGGALAVDRDKFS

SVVTKTLKDLPNVHVHEEEITEIPKDGITIIATGPLTSDTLAEQIKEFCGTDSLHFFD

AAAPIVAASSIDRDIVYKKSRYDKGEAAYLNCPMTKEEFYNFYKNLVSAETATLHGFE

DKNVFEGCMPIEVMAKRGEKTMLFGPLKPVGLEDPKTGKTPYAVVQLRQDNAASTMYN

IVGFQTHLKYGEQKRVFSMIPGLENAKFVRYGKMHRNTYIASPEVLNANYEARKQAGL

FFAGQMTGVEGYVESAGSGLVAGINAAREAIGEETLVFPKSTALGSMAHYITTTSAKH
                     FQPMNASYALLPKLDYKVRNKQERHLEISKRALKDLEAFKEEEKLD"
     CDS   81748..82671
                     /gene="xerC_1"
                     /locus_tag="OCPHGMPK_00783"
                     /inference="ab initio prediction:Prodigal:2.6"
                     /inference="similar to AA sequence:UniProtKB:P39776"
                     /codon_start=1
                     /transl_table=11
                     /product="Tyrosine recombinase XerC"

/translation="MSKENDKLIKQFQDYLNYERNYSKNTVSSYLNDLDEAKQFFKEN

GGFSGWDKVKSRDVEIFLQNLATQNRSRTTQARKMSSLRSFYRFLVKREVLENDPMQT

ISLRLGEKKLPQFFYEKEMRQVFDSLVGHDRLVVRNRAMFELFYATGMRLSEMASLKL

DQIDFDLKIILVHGKGNKDRYVPFGKDALDALKEYRDDVRPALLGQNEDLGYVFLNNR

GQKLTGRGIEYIMQQVFIKAGVGGKVHPHMLRHSFATEMLNNGADLRSVQELLGHESL
                     STTQIYTHVTMKHLQADYQKFFPRKDKKDEA"
     CDS   82675..83199
                     /gene="hslV"
                     /locus_tag="OCPHGMPK_00784"
                     /EC_number="3.4.25.2"
                     /inference="ab initio prediction:Prodigal:2.6"
                     /inference="similar to AA sequence:UniProtKB:P65797"
                     /codon_start=1
                     /transl_table=11
                     /product="ATP-dependent protease subunit HslV"

/translation="MTTICSVRYNNQTAIAGDGQVTLGEKVIAKATAKKIRRIYHDQV

VIGFAGGVADAVSLQDMLEGKLETYSGDLRRAAVEMAQSWRKDPTLAKLQAMVIAFND

KDLLLISGNGEVLEPDEDVVAIGSGGNFAQAAAIAMTRHSSGMSASEIAKEAVKIASG
                     IDVFTDDHITTDEI"
     CDS   83210..84604
                     /gene="clpY"
                     /locus_tag="OCPHGMPK_00785"
                     /inference="ab initio prediction:Prodigal:2.6"
                     /inference="similar to AA sequence:UniProtKB:P39778"
                     /codon_start=1
                     /transl_table=11
                     /product="ATP-dependent protease ATPase subunit
ClpY"

/translation="MTEEKTPKQIVELLDKYIIGQNEAKKSVAVALYNRYRRLQLPKQ

MQQDITPKNMLMAGPTGVGKTEIARRLAKIVDAPFVKVEATKFTEVGYVGRDVESMVR

DLVEEAVRMEEKEQFDRVKVQATKKANNRLVKLIVPGIKRENRENSMQQMMQMLSGNF

NMNQAQDNEEVTDDIRNERLSVADQLNKGLLENREVTIEVEQAPKVNPMGDMMGQMGI

DMSSLMGNLMPKKTVKRTLKVSDAREVLIQEESKKLINYDSLYQRAIERTQQNGIIFI

DEIDKITAGNKKTSGEVSREGVQRDILPIVEGSTVSTKYGPVSTDHILFIAAGAFAES

KPSDLIPELQGRFPIRVELNALTQEDFVKILKDPQNSLLKQYIALLKADGIKLVFTQE

AIDRIAQIAFEVNQGTDNIGARRLATILEKLLEDVLYEGPDMNMGEITITQKYVDQKL
SDIIINKDLTKFIL"
CDS 84632..85519
/gene="galM_2"
/locus_tag="OCPHGMPK_00786"
/EC_number="5.1.3.3"
/inference="ab initio prediction:Prodigal:2.6"
/inference="similar to AA sequence:UniProtKB:P39840"
/codon_start=1
/transl_table=11
/product="Aldose 1-epimerase"

/translation="MDYQLKNNFLTVTLSDQGAEIQSVKDVNSGREYIWQADPKIWGR

HAPVLFPVVGRLKDDQYTYDGKTYHMGQHGFARDSKFTVEEQDDKHIVFLLTSNEDTK

KMYPFDFELRVTYSLVNNLLSEHFTVTNTDTKEMIFGIGGHPGFNLPTDNGLTKNDYY

FKFAPSMDHVKIPLKAPYLDWDNRSLLATDSLFEISDELFKDDAWVFELKQPNKISIR

TDKSDYHINVKMENAPFVGLWSQYPKSGNYICIEPWWGIADTLDSDGQLEHKRGMNHI
APNEVWENGFTMAFHDKAK"
CDS 85579..86181
/locus_tag="OCPHGMPK_00787"
/inference="ab initio prediction:Prodigal:2.6"
/codon_start=1
/transl_table=11
/product="hypothetical protein"

/translation="MRIGYLKNLGRHELEQASSKWQTLHENEKIDLEPVGYDEIEAKI

QNGEVDAVLVNSRNDIYQALASYSVSEIALLALVQAGNFNKYQQTVELDELKNVPCIM

VASVADEKSEHHYLKDILRIKNDLIAVETLGEAILMVESGSGYLIMNEMTASHVDDDQ
VQKLFLLRDGKQLREKYVLLAKPDDARVAEFSKIMKESIN"
CDS 86341..86904
/locus_tag="OCPHGMPK_00788"
/inference="ab initio prediction:Prodigal:2.6"
/codon_start=1
/transl_table=11
/product="hypothetical protein"

/translation="MLTKSHRICSIAIVELSLLLTNRLLIDPVNNIIILAATAIGASL

PDIDEYNSSASKKSVINFSLFLRHRGITHSFLGWAVFSGGLYYLMSKFIPIRINNLTS

QNYWSALWFGLVIGYFLHLIQDSFSKQGVEWLAPFYKKKKKSPFHYKVGGCFEKFLTP
MSYLAVITITFYWIWISLTPATQVFLF"
CDS 86966..87076
/locus_tag="OCPHGMPK_00789"
/inference="ab initio prediction:Prodigal:2.6"
/codon_start=1
/transl_table=11
/product="hypothetical protein"
/translation="MSQLLVVAGLISLLYVVIDMKSQAIPVENDTVHKGR"
CDS 87172..87633
/locus_tag="OCPHGMPK_00790"
/inference="ab initio prediction:Prodigal:2.6"
/codon_start=1
/transl_table=11
/product="hypothetical protein"

/translation="MDSNDLIFAKLEHKRLRYNLIADLFLIVGGILIFRQPAMSMSFQ

FLGSLMIIIAMIFTILSFRIRSSKQYRALSLLVKNEPSSLKEIMNSFGAGMTFLALFM
AIGFMVIIVIFFFLLSRSWIKALLFFITFETVIDLKDFMNNLTKLKDKSTI"
CDS 87831..88862
/locus_tag="OCPHGMPK_00791"
/inference="ab initio prediction:Prodigal:2.6"
/codon_start=1
/transl_table=11
/product="hypothetical protein"

/translation="MISIVKSRSFGLAAVMTLICSVAGIFIAKLPYLNLIGALVIALL

LGISLQVLPVGVREEAAPGIGFISNKFLRLGIILLGFRLNLTKLADAGIKTILVAMLG

VSGTIILTYWLSKKFGAEDELAALSACGTGICGAAAVMGVSPQIESRDEERKRENEVL

AVAVVCVMGTVFTLFEIVIKPMLGLTDSQFGIVAGGSLHEIAHAVAAGNAFGEASLDS

ALIMKLSRVLLLAPVALIVGYWYQRRLVKESAADHTQAPKKLPIPWFLGGFILTSVLG

TFLPFPPVVLDSLVQAAYVFLGMAMAALGISVNFNVIFKRGGTVFGAAAISSTCLMIF
MIIMSKLFF"
CDS 89025..89885
/gene="gltC_1"
/locus_tag="OCPHGMPK_00792"
/inference="ab initio prediction:Prodigal:2.6"
/inference="similar to AA sequence:UniProtKB:P20668"
/codon_start=1
/transl_table=11
/product="HTH-type transcriptional regulator GltC"

/translation="MEINKLKTFVELASTLSFSKTAANLFINQSSVSKQIKSLERELN

CELFIRSKRQVRMTEYAKILFPAAQKIIKLNDQALSDLRNCLQDRDDKIIIGVLPTFW

NSSFFDQISNFKRQNPNLRIIIKEGEINYLTNLFQQGKLDFAFVRTTYGYKSDFEEFT

VDHESFCACLNYEHPLATKKSLKLADLKNENFVFYSGEEMLYHTVFDLCRESGFEPQF

SLASHNIKSILNLVKNNEGISILMHAPEKAKDVTYVPIKPAFSTRLVCVRQPNQATKI
ASDFWNYLVK"
CDS 90005..91921
/locus_tag="OCPHGMPK_00793"
/inference="ab initio prediction:Prodigal:2.6"
/codon_start=1
/transl_table=11
/product="hypothetical protein"

/translation="MKGKRRVILLGGMVLTSLTLLTACQTSATNNNSSSANTSKVVAK

ATKYQVSNSDISNAKKLLINSSKWHYNAKNKVYYQVNVKYGTKTKSSYESMGIYIPAA

YVNAKKSGTNTYKISINTNKKVNGYTASTAPIVMPINTPGYSAQQAPTGYESGSAKFT

KAGFIYVLAGCRGLNQTDSSNGSSPWGVTDLKAAIRTLRLNENRIAGNTNRIFTFGHS

GGGAQSAIVGSSGNSKAYIKYLKAIGAPLATTSGKATSDATEGAMSWCPITSLDTANE

AYEWNMGQYSTSGTRASGTFTKALSNDMAKKYAEYINKLGLKDSKNQKLTLKKSSSGI

YTSGTYAEYLKAEVEQSLNNFLKDTKFPYTATSMNGPSGAAQQSGSKPSGQKPSGAPS

NNMKNGNNSKPSGKKPSGSMPNGGAGAPGGSQSSGKTYKTVQSYIKSLNKNGTWVKYN

AKTNTAKITSLKAFVTHCKEASKDVGAFDGLTRQQTENNLFATTTRKNGHFDSTMSNL

LSENASKYAKLKNYKSSYASAFASDLKQKDAQGNTMTTRLNMYNPMYYLSDYYKGYNT

TKVAKYWRIRTGINQGDTALTVETNLALALKQDSQVKSVNFATVWGQGHTEAERTGNN
ETNFIKWVNKCLKN"
CDS complement(91997..92860)
/gene="yahB"
/locus_tag="OCPHGMPK_00794"
/inference="ab initio prediction:Prodigal:2.6"
/inference="similar to AA sequence:UniProtKB:P77700"
/codon_start=1
/transl_table=11
/product="putative HTH-type transcriptional
regulator
YahB"

/translation="MIENYLLEELVTFAKYGTVAKTAEVLGLTQPAVTHSMKKLEEDL

GVTLFIRKPNKLYLSETGKYTAREAKKLIDDNLDFTKRVKQFEKDQTTLIVGVNAPGP

GIVLRSLHDQNIQIVSDLVESNFENLLADHQVTCLLLNFPIEDRDITSTYLGTESMSV

NLPAECNLNQYKELSFKMLKGKTILSPSPIGFWTKIYQDEIPDSKIIFQNESSEYSEI

LQYSVLPFFTTNLTSLDSQWGLNLPNNRSVRPLKDEVAHQKFYACYLKQNKDRVQPLI

EKLQDQWSKYD"
CDS 92975..93511
/gene="azoR_2"
/locus_tag="OCPHGMPK_00795"
/EC_number="1.7.-.-"
/inference="ab initio prediction:Prodigal:2.6"
/inference="protein motif:HAMAP:MF_01216"
/codon_start=1
/transl_table=11
/product="FMN-dependent NADH-azoreductase"

/translation="MARVVILTGSPHYEGASQKLADNFEKGVKEAGNHIYRYDAGLQG

DSQPHFLQLEHAPGMEVGIPDNDEVEKEVIPELLDADVVVLVSSLYYFEINAQLKTVI

DRFYDYNHELKDKKMVFMMAGYGTQEDMDAVKLHMQKLSDYMRWQMIDQIYADDSWNK
QKLAKFAQAAYNLGKSIK"
CDS 93545..94585
/gene="adh"
/locus_tag="OCPHGMPK_00796"
/EC_number="1.1.1.1"
/inference="ab initio prediction:Prodigal:2.6"
/inference="similar to AA sequence:UniProtKB:Q0KDL6"
/codon_start=1
/transl_table=11
/product="Alcohol dehydrogenase"

/translation="MKYAEFVKAGEMKIIDKPLPKIEKDDDVIIKVLRTCVCGSDLWA

YRGLEEQGHENSGHEIIGIIEEIGKNITTVKKGDFVIAPFTHGCGHCRACLAGFDGVC

MNHDLSENFSGGYQAEYVRFQHAQWSLVKVPGKPEDYTEGMLKSFLALADVMATGYHA

ARVANVRPGDSVIVLGDGAVGLSAIIASKLRGAKQIVSTSRHPDREKLAREFGATDNV

AERDEEGVQNLISITRGGADAVLECVGSKLSNEEAVQVGRPGAIVGRVGLPHGAELNP

GLIFYKNMSVAGGPASVTTYDKEVLLKAVLDGKINPGKVFTKTYDLDQINEAYQDMAD
RKVIKSYIKVSD"
CDS 94596..94970
/locus_tag="OCPHGMPK_00797"
/inference="ab initio prediction:Prodigal:2.6"
/codon_start=1
/transl_table=11
/product="hypothetical protein"

/translation="MNKWTNEEIAAFDKAQTLHNSPYNDDKTTSEEDNPVWEVTVDNQ

VFIRGAKGIKDTKWYQSGTKNGGQIEVAGQKFEVKYQAVNNAELIKDVTEAFNKKYHG
QYPIDLMVSDPVAKATIAIVKK"
CDS 95235..96743
/gene="yheS_1"
/locus_tag="OCPHGMPK_00798"
/inference="ab initio prediction:Prodigal:2.6"

/inference="similar to AA sequence:UniProtKB:P63389"
/codon_start=1
/transl_table=11
/product="putative ABC transporter ATP-binding
protein
YheS"

/translation="MSSIKISNLSFRYEDSSDNIFNNLNLDLDSSWRLGLVGRNGRGK

TTFLKLLNGKLLGRGKIQTRLNFSYYPISINDPKNITLYELQNQVQFEQWELERELNL

LGADPNLIWQPFNSLSGGEQTKILLALSFTDQNSFPPIDEPTNHLDEASRKEVSDYLR

VHHQGYIVVSHDRDFLNQVTDHILAIENTEIHLYQGNFATYEEVKAERDEFNREKNQK

LAGEIGNLHRQKEQFYHWAQKIEAKKKLGQKTQYIINRRSRINKAALGHASAKMMKKS

INARNRMDKRIEEKEGLMLNIEDVPSLTMNYCPNYHQVLLETEKLRLSIQDKKLFADL

NLVVRNHGVVSLEGKNGAGKSTLLKLLLGKARETKYQGKYSLASGLTISYLPQDFTIY

KGSLKKFAQEQGVLYEDLLNMLKKMGFPRSNFETPIEEMSMGQQKRVAVAKSLVEKAD

LYLWDEPANYLDVFNQDQLIKLLQEVKPAMLLIEHDKYFIEQVADQRIVISN"
CDS complement(96814..97125)
/locus_tag="OCPHGMPK_00799"
/inference="ab initio prediction:Prodigal:2.6"
/codon_start=1
/transl_table=11
/product="hypothetical protein"

/translation="MDTIIYSDHFTTSLTNEISYWKNTLLLSDEEITKYISLIYHNIQ

LLSDFPYLYQNVHQKYQLDKPTYKITIGKKYAIFYRVSEKNHTIIIGSFFSNKQMRLE
F"
CDS complement(97126..97383)
/locus_tag="OCPHGMPK_00800"
/inference="ab initio prediction:Prodigal:2.6"
/codon_start=1
/transl_table=11
/product="hypothetical protein"

/translation="MKKLKVRKIGNSLGSIFPKDWEVHDGELLSYTVDKKNHRVIIDL
SKNDLEHDRALIEEGFKDFETGNFATEKEMKAMFGKYSWGK"
CDS 97588..98433
/locus_tag="OCPHGMPK_00801"
/inference="ab initio prediction:Prodigal:2.6"
/codon_start=1
/transl_table=11
/product="hypothetical protein"

/translation="MKKQKKPDVAKLLGIIDDLMFQNVMKDPVNCRMFLHEVLPDLDI

QGLTVRTQERIAFNKEEKFSVLDVLIKDSQGRRYDIEMQVAPQKDLDKRARYYMYKMM

EDGFLHQGEEYGELTAVYVIFILPFDPKGKGLKRYTFTYSAREDTSVELNDESEIIYL

NSKGKKGSVSQGLEDFYSLMEGKNTTNSEFIKRIKKTMDNYRKTEEWSEHVMNTEQIK

EMALAQGVEQGLEQGRREARIFDIRKIVKILKRMNQSDEQILQELKQDYSDDFSDEEL
KKFLK"
CDS 98638..99276
/gene="fetA_1"
/locus_tag="OCPHGMPK_00802"
/EC_number="3.6.3.-"
/inference="ab initio prediction:Prodigal:2.6"
/inference="similar to AA sequence:UniProtKB:P77279"
/codon_start=1
/transl_table=11
/product="putative iron export ATP-binding protein
FetA"

/translation="MAILKLQNVAYQVGQKQILQNISFKIEDGAFLTLIGPSGAGKST

ILKLIANLISPTSGKIFFREKDIMTIDPVTYRRSVSYCFQQPSLFGEKVADNLSFPYE

IRKQAVDRERILRLLHDVSLEADYLDKDITSLSGGEKQRIALIRNLIFLPKVLLLDEV

TTGLDGDNKAIIHQLIERVHQQGVTVIQVTHDQGEIDKAQNIIKIKKGGILE"
CDS 99273..100031
/gene="fetB"
/locus_tag="OCPHGMPK_00803"
/inference="ab initio prediction:Prodigal:2.6"
/inference="similar to AA sequence:UniProtKB:P77307"
/codon_start=1
/transl_table=11
/product="putative iron export permease protein
FetB"

/translation="MKNVMVSNWSLVFAFALVLVSIAISAKEKLGLTKDILISVIRAI

VQLIVIGYVLKFIFHVNNYWLTFTSTLIIIFNASWNANQRDPNPDKKFYNSVIAEAIG

TYATLAILILSGVIKPIPMQVIPITGMIAGNEMVAVGLCYKSLNESFRDLRQTILEKL

ALGSDIKTASMPILRRSIKTAMQPTIDSAKTVGLVNLPGMMSGLIFAGVNPIYAIKYQ
IMITFMLLSATSLGAVIAGYLAYRNYFNERMQLK"
CDS 100164..101843
/gene="alsS"
/locus_tag="OCPHGMPK_00804"
/EC_number="2.2.1.6"
/inference="ab initio prediction:Prodigal:2.6"
/inference="similar to AA sequence:UniProtKB:Q04789"
/codon_start=1
/transl_table=11
/product="Acetolactate synthase"

/translation="MTEEKYYGANAIVDSLINHDVKFVFGIPGAKIDRVFELLEHSKN

SRTPKFILTRHEQNAAFMAAGVGRITGKPGVVLTTSGPGASNLATGLVTATAEGDPVL

AISGQVQRTDLLRLTHQSMKNAALFEPITKYSAEVQEPENISEVIANAYQEAEAAKQG

ASFVSVPQDVTDAEVKTSVIAPLEAPKLGPASPIESTLLSQRIKAAKLPVLLLGMRAS

DPETTNAIRRLIAETHLPVVETFQGAGIIPRELEDDFFGRVGLFRNQPGDQLLKKSDL

VIAIGYDPIEYEPRNWNSDRSANIVVIDSMRAEIDKNYQPERELVGDISQTLDFLLPY

MKGYSMPEESKDYLDSLRKTLQKRDTPPTVTKDQIKSHPLSIIQALQDRVTDDMTVTV

DVGSFYIWMARHFRSYEPRHLLFSNGMQTLGVALPWAISAALVRPNTKIVSMSGDGGF

MFSSQDLETAVRLGLNIVHIIWNDGNYDMVKFQEEMKYGDSAAVKFGPIDFVKYAESF
GAKGYRVEKAADLNKVLDQAFKEQGPTIVDVPVDYSFNQTLGKTLLDDQIH"
CDS 101863..102579
/gene="aldC"
/locus_tag="OCPHGMPK_00805"
/EC_number="4.1.1.5"
/inference="ab initio prediction:Prodigal:2.6"
/inference="similar to AA sequence:UniProtKB:Q8L208"
/codon_start=1
/transl_table=11
/product="Alpha-acetolactate decarboxylase"

/translation="MEKYESKVFQHGTLGMLVPGLFDGTMTIEDLLKHGDWGIGTASG

LDGEMIVLDHTPYLAQSNGEIRVLKAEEKVPFATVHFEQIKDKFTARNLTQKELEDHI

LKTYPYKNVFFAVKIVGKFSAVKTRVVEKQTRPYKTLLAVANEQAIFEKYDVSGTVIG

YFAPKMFQGMAAPGFHLHFLADDKSIGGHVLNFDVSEAKVYLQPFTMIDQHLPLDNQE
FLDKDLDIADMHDQIKKAEG"
CDS complement(102635..103048)
/locus_tag="OCPHGMPK_00806"
/inference="ab initio prediction:Prodigal:2.6"
/codon_start=1
/transl_table=11
/product="hypothetical protein"

/translation="MKDFIKNFVKSGLQAAGFGPLILVIFYSIYSFTINFQTISVQDV

NKNIISSLLLAFIAGGISAVFKVERIPLGLATTIDAIVIYLDYLLFYILNDWIKLQII
PFLVFTVLYIIGYLIIWLCIYHQVKNQVQKLNQKL"
CDS complement(103050..103487)
/locus_tag="OCPHGMPK_00807"
/inference="ab initio prediction:Prodigal:2.6"
/inference="similar to AA sequence:UniProtKB:Q5HDJ2"
/codon_start=1
/transl_table=11
/product="putative HTH-type transcriptional
regulator"

/translation="MKFKLFIEPSKAEIVEAYVHQRSDFSAQLKEFVLSNGNSNTIVA

YDDKDLTMVSFSDIVMITIVENKVLAICNNDKQYHIRKRLYQLTDLLPSNFWKINKSA
IANRNYIDHFEETKNSGVNIIMKNGLTDYVSRRCFAKIRKELN"
CDS complement(103753..104436)
/locus_tag="OCPHGMPK_00808"
/inference="ab initio prediction:Prodigal:2.6"
/codon_start=1
/transl_table=11
/product="hypothetical protein"

/translation="MRKDKHQHNFNDFSDQEKEKLKTVRQELAQAQKKEPERLWEHLQ

AFNDGVMAIIITIIVLEIQPAIHEVHYEQFIANIIVFLITFFVVADFWYDLHLAFSYY

IFKPTKTIAILDFFFLADLSLLPVMTKWIMAESSTFAVANFGIVFLVAKILEYLIQYF

GAKKTAKYSQIMNIIISRSFIRKVAVTLFLNVILIIILSLFIPKIAMILYLVIPVTSFL
FPVKHNKIV"
CDS 104637..106463
/locus_tag="OCPHGMPK_00809"
/inference="ab initio prediction:Prodigal:2.6"
/codon_start=1
/transl_table=11
/product="hypothetical protein"

/translation="MINLLNIFSKKRKIIINFVDSDNKNQIITNKSFFGKKGKQINID

LGSIIDELQNHGYQIPSNFDRKMALTFGKKDNIVNINVSHQKKIIDVNHITKNFPLNQ

VKRTIKQIVHYSGAGSRTPIDNVNSVEFYRTLEIDAVTKEIVSKTAWISNINTFLKIG

VPTLPGYVPDKTTVGGKEIVPDDGNQKYFVKYKLNEHPSTNKQAAIIQYVNLTANNAI

VKTTKLEGEPNFPINYDINNDLNDLIARGFKLVDNGFNGKGIQFFGNNDSYIPVFIVT

LRRDEIIVDSAHPYQKVDPEEYQKEITFTIKFSGAGKQTPENIIKTRHLQRKVLFSLV

LDKIVENNTYPTEWKTVKKDFPDKIQVPVVKGYHSNLSQIETENITNSKSEIKVSYIR

NNKIIPIDEDGKLIPTKEALYLATDPDDPTKLCTKQIIPQIPGYKSNYKIITLSNSNK

DIKVIYKKIAADFYTASSTNDQIAIVNFIDLDHEGKQITSSGPLIGKPNENITDLYST

SIPIAGLEKAGYHVIFNNFDGNNKIQKFDGNDLTTQVFTVGLSKNSEQNMQLQQLKSS
VNELIGQNSNNLSNPVVANLMNVLKSLIDLISILNKDNKNKN"
CDS 106483..106767
/locus_tag="OCPHGMPK_00810"
/inference="ab initio prediction:Prodigal:2.6"
/codon_start=1
/transl_table=11
/product="hypothetical protein"

/translation="MNKKCPNCGKEVNLGEAKCPFCGYDFVNNINAKIPGDLGMMGIK
LNNVPKKKKKTKPENKKIEKNINSKVEVAKTQELKQARKNKAAKKNKGWL"
CDS 106789..107328
/locus_tag="OCPHGMPK_00811"
/inference="ab initio prediction:Prodigal:2.6"
/codon_start=1
/transl_table=11
/product="hypothetical protein"

/translation="MTFGYIGFYVGSNFNSIQHSTSSESSSNNSHNHVKKSATSKTSS

RQQDKQSKSVQKQDTNTKLFTSMQVQPAISNQEEIKNFLTGVFTKPQANKFISGENNS

DFRQLRDVTLSWEPNDVNIKIKLLNVSQKGNDYTLNYLVYYNFITGRKQIMVYNNGLL
ARDDSAFHIVRLGSGCLIK"
CDS 107458..116442
/locus_tag="OCPHGMPK_00812"
/inference="ab initio prediction:Prodigal:2.6"
/codon_start=1
/transl_table=11
/product="hypothetical protein"

/translation="MNFRDMKRKERWSIRKLTVGAASVLLGTYFVASSQGVAKADTIK

DNNVNNNTVNEQTSEKKISQPITPNTQAVVIAKNNPQNSEDNTANLQNKADLIRQNNQ

KIINRLTVQKNTTQKVENSTFSEIKTREAANTQPFNIAENSSKSVATNSNNSLDESTF

GKVDVNSWDTETVNNGLNITGYHGSDQEHLVIPNAADFTKAGKLTNENAVITVNAAKF

KKILNTIKPTTVAISKTDNKKVQVSDKTAFGGNLTTENGVSTNDPNLKQMDLTNLDTS

NLTDMSGMFCGAYKVKSLGNLSGWDVSHVTNMQQMFDENTYDSPALESLDGINKWNVS

NVQNILCLFRNDINLKDIDISGWDLGKATGGWTTGQVFASFTNASKTVINLNNVKLPS

DFNFATSFANGQVVITNNGDLLQLDTTSKVPFKDNLTFRQYNSWVEYANPTVKTYSHS

IFYDSKGSSDAKTIVQDAIDTFLHNNYANYKKITIPTDAVYLSTKPSDSATAIANGTY

DIAKTETETSDTTYQVFRDDEIPMQLKYVETDTNKDLGTVTVVGNGITIVTSHSIGLN

PLPITNLADWLGNDDYTKSDMSITGQKHFGFDGIAEPVNVEIHLTKKVITEQRTVTIH

LYERDSNGNPVKDSNGNYVSVAPDLKATFNYQQDEFVTSKGKVVKKSNIWLWDEKAPK

TYDGANSQEVNVGYVEGVDGVTPIISNVKCWKVPINKEGTSYILVPNDGNWSTVEFST

PAVDGYYANPFIDNIVNGYPLLGRPANLFVNPRFNNSVRPGTSDASDSSIAYTNQDGI

YENKNEHIIWYFPKENTATIKRTINFVKNDASHTQLQETSTQDVNLNGSLVAANPNGT

GKDYFTIYPAEYNDTSIVTGSPIAVINNSNKPTYSWKAKDGQTSVNTDITGYTVSAVK

IGNADYSTNENDIKYAKEIAQNTATLDSPADLNISFVYAPVSLTSSYQFVDDDNKEQL

IAPATSFSGQTGETVELNIKLPDNYELAKGSTLPTSYTFKPSDNEQLKIHLIHQKKVL

SDDTRTIRRKVTITDPDGKVDPKSTTQSVSFSRSDTLDKVTNQHIYGKWNTKQQALPA

IILPVFAGYTPSGKVDAINVTADSNNPADINISYIADKQNSSYKFVDDNKNEATVGTS

HSISGTTDETIDLTKAIKDSVPTNYDLVPDQNMSYIFKASDNKPLIIHLTHQKKVLSD

DTRTISRKVTITDPDGKVDPKSTTQSVTFSRSDTLDKVTNKHIYGKWNKNQQTLPAIA

LSVFAGYTPSDKVDAINVTADSNNPADINISYTADKQNSFYQFVDDNEEGAKVGTSHP

ISGTTDETIDLTKAIKDSVPTNYDLVPDQKMSYTFKASDNKPLIIHLTHHISAVTNDS

NLTKTVSRTINVTKPDGTKLAPVVQRVSFLQTATKDDVTGKVTYNNDWKANGKDSFTE

YDAPTVDGYTANPVKIEKLTPNVNTKDSTKNIVYEAGSQSITVRFSDKDNKDQQVGEA

ISINGVTGENADFSTITIPTNYVLAEGEKLPTNHKFTANDQTVYTVELRHKTEDAKID

PNAKLSQKVSRTILITKLDGSVNKIIQEHTFTRTGTKDMVTGNISYGPWSENGKYNFS

DITAKAIAGYAIIGSAPEIVVTLETKDSIVNISYLANGQTNYYQFIDDDYQVGDPVIN

QKHNIAGKTGDSINLNITIPENYDLAKGNHVPATYTFKANNNQPIVIHLVHHLSAIIN

DPKLMKKVTRTINVTKPDGTKLAPVVQSVSFIQTATKDDVTEKVTYHNDWKVNGIDSF

AEYSAPVFAGYTASQTKVEKVIPNINTQNSTINITYQANPQKISVRFIDDDNQKQQVG

SAFIINGVTDGNADFSTVAIPENYVLAEGSKLPTNHKFTANDQTVYTVELRHKTEDAK

IDPNAKLSQKVSRTILITNIDGQVNRIVQEHTFTRIGKKDMVTGNINYGSWSENGSYT

FAAIPVKNIAGYATSGSAPEIVVTPETKNSTVNISYFANGQTSYYQFIDDDYQVGDPV

INQKHNIAGKTGDSINLNITIPENYSLAKESTLPTSYTFKVSDNKPLVIHLIHQKEAL

SDDTRIISRKVIITDPNGKIDPKSVTQSVTFNRSDTLDKVTNQHIYGRWNTKQQILPT

IALPVFAGYTPSGKVNEINVTADSHNPADINISYTADKQNSSYKFVDDNEKGADVGTS

HPVSGTTDEMVDLTQSIKDLVPSNYDLVPDQKMSYTFKASDNDPLIIHLTHHISKVVN

DPSLTKTVRRTILITNINGKVDKTVQEHTFTRTGKKDMVTEIIKYGPWSENGKYTFAA

IPVKAIEGYAINGSVPEVVVTPETMDSIINIGYVANGQTSYYQFIDDDSQAGDPVINQ

KHNIAGKTGETIKLNISIPENYDLVKGNSVPTTYTFTAEDNKPIVIHLVHQISAVTDD

TSLTKLVRRTILITNIDGKVDRAIQEHTFTRTGKKDMVTGNISYGLWSENGKYTFAGI

PIKEIAGYAINGSAPEVVVTPETKDSTINISYLANGQTSYYQFIDDDSQAGDPVINQK

HPIAGKTDETIKLNISIPENYSLIEGSVLPTSYTFESGNKPIIIHLIHQKESLPNDTK

TISRKVTIIDPDGKVDSKSTTQSVTFSRNDILDKVTNKHIYGKWNEAQQTLPSIDLPS

FNGYTPTGEVTAAEVTTNSGDIADINISYVPNEQTSYYEFVDDDNHESLIAPATVISG

RTGETIKLNIVTPNGYALAKDVTLPRSYRFKATNNEPIIIHLVHHMSKVTNDPKLTKV

ITRTINVINPDGTTDSIVQTVRFVQTATKDDVTNSVSYMNDWKNVGAESFAEYNVPEI

AGYTSSKTKVEKLTPKFGDQNVIVNIDYIKKSQINPVKPVKPIVTPIEKDNKEQSANK

QQTVTAEPNSKPKVTQEIKQPVSKPNKTNGKILERKDRQTYKFQGQTVTMKKVVNAKE
AGHINKIKAKKAALPQTGNDEAKTTAIIASGLAVDLSLIGLAGLKKKRSN"

CDS 116481..119408
/locus_tag="OCPHGMPK_00813"
/inference="ab initio prediction:Prodigal:2.6"
/codon_start=1
/transl_table=11
/product="hypothetical protein"

/translation="MLSRNNNRFNELKEISPRYSIRKFTVGAASVLIGMSIFGLNSQT

ARADSINENDTNKQNPAIEQKSDKALTATPASDIKNVVVTAKNVDAQNQVPAETSNGN

TSSKQNTINTNKENSQRAELQIENTRKVVAANKEESTQDTTAIQNQAKPVAKENYSVI

QHDVIANNGNTPHDSGYVQLNLGLQIKNTKNINPGDYIDIDLGLPLQSGQQKTYSDGL

AEKNTPVTIKDNAGKTDTIGSIATVGNIPNEFYQLSFNDHIQKYGTVLLNLDLKQYSL

IQRAISTVGYSHEKNGPTSYSAQDDLVIGDGAYKLTSGLSVPVKYIPQESNGSVIQRV

GENTWIQGRSTGKNSRVWTVYPDGSFTVNDKSALGLDGIVYFSKNFGNSATVTVYTLS

NNPYFDSNYASDDDIKEQIESAFAQLKSTNNLDQIAQDNSNVGFSINKIPENDISIAV

THNDNSEKAYSTVMRADGSKYSGDQLMLSRTYHITVNGAKLGQIYSLPISFVSEITKS

GVNVSKPANITKPEQDKIQTYQDSNKYEVLNNDLNNAYPILYKGIMIDNPGLMNFMKN

NLATWIDVKNDEDPSDELLGPAAYTLNVSVVNQPTNLKPQNIANGNSSSGQQLDQTIL

VVFQDLDENNKNILSKDLTGLSGADAGYSTANDIKALENQHYELVSDDTNGRNLKFGN

QEQVFYVKFRHVLKKEKQENKSVSRNITYVDDKGNPVKGSPDGKASYVQSASFVRFPV

KDLVTGVVGYSINNDGAIDTQDGTHAWKAIGSNYFEKVISKDPASLGFEHVNYAVIPE

KTVDANTKDQEIQVIYSGTTKNPDKPSEPSQPTKPNKPEVPNKSDKSNESSKPDKTSD

VKPTESVKLIHQTTADTSKNQSESIKEENLSKPKAVLDKTSFSPLARRNSPLVNEKTN

ILESSKDSTKTTKNRTVNSSKELPQTSSNTQQSINDEVIGSVALSIGLIGLAGVKKRK
NAR"
CDS 119515..121242
/locus_tag="OCPHGMPK_00814"
/inference="ab initio prediction:Prodigal:2.6"
/codon_start=1
/transl_table=11
/product="hypothetical protein"

/translation="MDDKFNQNENIDEHVVVDANHPGYGFTEEKLLKTITQTVHYVGA

ASRNPQDNVTRVSFSRVYIVNKKSGKVIEDRGFQPKEKSMQLIGTPTLPGYIPDRVVV

GGEKVTPNDLNKEYIVTFKLNMKPTNTMQKAKIRYVDLTTNNQLLTEDTVSGLANTPI

NYDPQFKIKHFEEQGYKLVANGFNHNGDVQFFGSSNDYCPIFIITFISAVEAVNINNP

VDWVDPSLYHRKSKLIVNFEGCPNPPKTVIQTINWFRTITVNNIVKKIIPNGVYDKTW

RADEERYQEVKIPIVQGYHADLDVINQKSVTLDDQTITVQYHPNGHLIPVDEAGAAIQ

GAPTPQFESDSNDASKVKNDQNVPKVSGYQATLATVTPANPGQDLTVVYEKINNNDDT

LYINLNHDHESDSQAAIDHENVDQQTDLTDNSKIVQTAIINFIDIDDHGHSITSSGKL

SGRVGDSINDLYSTEIPLKVLKEQGYRVVFDDFNENSERQYFKESVLPQIFTIGLSKK

SSDNQESSDLDEEYEKLRQTREQFKQIKPTIMDSSDELNSSQTVNKLIDIITGLLTLI
FMLSKGNKK"
CDS 121342..121941
/locus_tag="OCPHGMPK_00815"
/EC_number="3.1.3.-"
/inference="ab initio prediction:Prodigal:2.6"
/inference="similar to AA sequence:UniProtKB:Q8A947"
/codon_start=1
/transl_table=11
/product="D-ribitol-5-phosphate phosphatase"

/translation="MLKNIIFDFGNVIMNYNPDEILNHYELSPADHDLLRKTIFESKE

WSEIDAGKITEKEATEIFIDRVPDQLKSKVKQIMATWPENVDFYEPVFNYMEKLRQDG

YKIYGLSNTGMQFANFVKNSEMGNYFDGYVFSAQEKLMKPDRRIYEKLIARYILKPEE
SLFIDDLKANTDAAKKLGMQAFTFKIDKLGELQDYIASH"
CDS 121976..122599
/locus_tag="OCPHGMPK_00816"
/inference="ab initio prediction:Prodigal:2.6"
/codon_start=1
/transl_table=11
/product="hypothetical protein"

/translation="MKTNDQIANWAMDLQSLAQAGLRYGHDVFDRERYEEIRRIAGEM

MSARTGIPEEKLKTLFLGDEGYQTPKIDTRAAIFKDNRILLVREKKTQEWSLPGGWND

YDQTTAQNCVKEAREEAGRIVEPIKIIAVQDRNHHNKPVIATNITKIFYLCKEISGKF
TPNDETDACDYFNLDNLPKLSLGRNTKEQIAMCFDANNDPDWDTKFE"
CDS 122889..123635
/locus_tag="OCPHGMPK_00817"
/inference="ab initio prediction:Prodigal:2.6"
/codon_start=1
/transl_table=11
/product="hypothetical protein"

/translation="MFFNKNDETKDKVVDEVLSSDSFNNLNLDLIDLDLKVETGNKFE

VHYCGSEDEKPSVELKDDTLEIEEPRVERKHGKFWRKDFIEVNIVSQSDSGALVVTVP

DGHQLGAVKVSSVSGDTDFKSLNLDSLVISVVSGDVSLRTVQTDEVKLTTTSGDIDLK

EVTVKQGKAQLVSGDFTLKNSLVLDQFKASTTSGDNLVEDVKAAQYQLSTLSGDNSLF
GKNEAAAEGDSTNNAGTIILSTLSGDNTVK"
CDS 124095..125474
/gene="brnQ_2"
/locus_tag="OCPHGMPK_00818"
/inference="ab initio prediction:Prodigal:2.6"
/inference="similar to AA sequence:UniProtKB:P0AD99"
/codon_start=1
/transl_table=11
/product="Branched-chain amino acid transport system
2
carrier protein"

/translation="MMTDHTSRKLTWKDYLVVASLLFGLFFGAGNLIFPLHLGQLAGA

NWGTAAVGFLITGVLLPLLSVLAVAITHADGVYDIGKPLGAGFAVVFMVLIHATIGPL

FGTPRTATVSFTVGIAPFVPKNMQGTALLIFSALFFLAAFGFSYHQNNILSNVGKVLN

PLFLSLLFLVFLVAFARPLGNPQTAAVTAAYKHGALVNGFLEGYNTMDALAGLAFGVT

VVTAVRGMGQKNAKSVSKVVAKSGLLAVLAIGFIYLLLILMGAMSLGRFKVSDNGGVA

FNQIVNVYGGVFGQALLAFLLTVTCLTTAVGLVAAFAQDFHKHFPQVSYHAWLALSCL

ASFLAANFGLDTIIAWSTPMLMFLYPLSMVLILLSVCSPLFKTDGVVYFFVIVFTVVP

ALGDMVVAFPSVVSQSSFGLAVASVRNHLPLANMGLSWLVPALVGLAIGLVVHFVKNK
KLTSTLEED"
CDS 125669..125986
/locus_tag="OCPHGMPK_00819"
/inference="ab initio prediction:Prodigal:2.6"
/codon_start=1
/transl_table=11

/product="hypothetical protein"

/translation="MSEYKVHIINTGKVRIEPELAFGGDHCSTAKAAGFSIKRSPKIW

LPVNVFLVETPDKLVLLDTGWDHSMSPNGVFDKKAQIKSLGSSLLYHVNQGVVAKRMT
ASE"
CDS 126095..126514
/locus_tag="OCPHGMPK_00820"
/inference="ab initio prediction:Prodigal:2.6"
/codon_start=1
/transl_table=11
/product="hypothetical protein"

/translation="MVARSEYEYAKKHPYIRFKKKWWDDVDLNLYDWNDSQGPFGRSY

DLFGDKSIELINIPGHTAGLVATKITNSDGKYFLYYGDGGYGEKSWKEMVTSGISMDK
SAQKKSLEWIRKESLDPNCVRSLACHGIEEKPEVFEF"
CDS complement(126525..126905)
/gene="ytcD"
/locus_tag="OCPHGMPK_00821"
/inference="ab initio prediction:Prodigal:2.6"
/inference="similar to AA sequence:UniProtKB:O34533"
/codon_start=1
/transl_table=11
/product="putative HTH-type transcriptional
regulator
YtcD"

/translation="MYQRKLPKEYYCTVDYALDIFGGKWKPRLLCILGHKKSMRYGEI

KDQMENISDAALADSLKELQQQDIIERKQYNEMPIRVEYSLTNKGKTLLPVLNTISNW
AIKNSTPDIYKGMHFNDVHKDLFN"
CDS 127023..127316
/locus_tag="OCPHGMPK_00822"
/inference="ab initio prediction:Prodigal:2.6"
/codon_start=1
/transl_table=11
/product="hypothetical protein"

/translation="MAVKAYLEVTMQIPAENRKAAAKVYSDYRKPFLETIPGAESKAL

LVRDEDVQVLHGFDSVEHAQDYLKSEMFTKHVFPGLKPTWTAEPEVRIFSVVG"
CDS 127372..128544
/gene="dgoD"
/locus_tag="OCPHGMPK_00823"
/EC_number="4.2.1.6"
/inference="ab initio prediction:Prodigal:2.6"
/inference="similar to AA sequence:UniProtKB:Q6BF17"
/codon_start=1
/transl_table=11
/product="D-galactonate dehydratase"

/translation="MKITKIDLLYSDPVEDNWRPSFCRIYTDRGIYGDGEVALSYGGA

KEAAFIEMKELAPMLIGMNPLEHEVIWQKLYRNSFFGKNAGPVIFGAISAFDIALWDI

KGKYYQAPLWELLGGKQRDHLKAYASQLQMGWGESRKPAASAQDYANNTKIALEKGFK

TVKINFLTFDERGKQVPWQKQTAFLSNNYLNLAEERIKAVRDTLGKDGELILENHALT

DKLSAVQYGKMASKYNILYFEEPVKPDPDLLSYVHQETGLNIASGERMYSRWDFKRAF

DQNSIQVAQPDICTAGGVTEVKKICDMADTSELGVQIHVAGSNLATAVSLNLEAALPN

FVIHEYNVNTEMPKMLKLTKYDYEPNNGVFTIPNRPGIGNEISDYAFKHSRVVTVK"
     CDS   128774..130363
                     /locus_tag="OCPHGMPK_00824"
                     /EC_number="3.6.3.-"
                     /inference="ab initio prediction:Prodigal:2.6"
                     /inference="similar to AA sequence:UniProtKB:Q7A4T3"
                     /codon_start=1
                     /transl_table=11
                     /product="Putative multidrug export ATP-
binding/permease
                     protein"

/translation="MSIKQLFKSNKKQFILIFLMVIIGMASDSLSQYLMTPAYNHLRN

MNLLGFILFMCLALGCDGVRIIILISRSDYLYGKDVQNYLHQIRARISRYFFKNDINQT

AKVQNNMVANLDQLTTKYLTPIKNGFMYFLAVVFSIGILFSFNWILVVVTVILTIISL

LLPKAFEKLTSTATVTVTKKNEKFLNILDKWVKGLDELRRYASFDIFTSSINNGAKEY

KKAAIHQGATIAIADMATALVNIGGQMLLLGLCAYLYLQGRIAFGAVITTGQFSSTIM

NGVAAFVAQWNLIKSAKGLNEEILELEQQVEIPKDQHDDQKIAKIEIKDLTLQFKNGE

KISYPNLTFNKGEKILITGDSGSGKSTLFKLILNKLTPSKGTIRFIDQDGKEVYLNPD

KIGYLAQDSTLFPDTLENNITMFNAELNNQVKQATKKVDLDKDISNFPEGLATQVDLD

KNNLSGGQKQKLVLARALVHNSPWLFIDEGTSAIDSKSTKHILQHLLTTDGSVIMIAH
                     NFSNELVSMFDRVVKLDDGGH"
     CDS   130367..131950
                     /gene="yheI_4"
                     /locus_tag="OCPHGMPK_00825"
                     /EC_number="3.6.3.-"
                     /inference="ab initio prediction:Prodigal:2.6"
                     /inference="similar to AA sequence:UniProtKB:O07550"
                     /codon_start=1
                     /transl_table=11
                     /product="putative multidrug resistance ABC
transporter
                     ATP-binding/permease protein YheI"

/translation="MSFKDFIKTNYFRFIYMNILSVISGAAVIGAGYVQMYFLTDVKN

KDWRGILVTVLLIGLLYIGTQGMIYYIQFEIRIQEEEYNQQIRNKLASHYFLDQRNHK

VADVQNRMTNDLEMVRTNFFDWYIMIPFYGAIFVSALVALLSINWMIFLASIVIDIIS

YFIPKLIQKKMEQATTNVSKQNKYYLDVLAKWFSGLEELRRYFAGAKLFKIGEQASNK

VENAHIKQTAAQQELIVLSGFCNLVGQMLLLSLTAFFITKNAIIFGAIMSVQNFAANV

SIGLQQVIEGLSFMKSSKELMGEIDHDSSVINVDKVEGEEAPFAFTTKNLSLNFPNGE

KLTFPDIDVKPGEKILLSGDSGAGKSTLFKLILGDLKASTGKVEFENAAGDTIIPDMS

RIGYIPQDPNLFPGTIKENITMFNDKLDSRVEETINEVNFDKDIKKFKNGINEELDLD

KINISGGQRQKIVLARAKIHDSDIILIDEGTSAIDQKATMNILGKLLNSKATIVFIAH
                     NFNEHMRQMFDREIYLSKD"
     CDS   132114..132965
                     /gene="drrA"
                     /locus_tag="OCPHGMPK_00826"
                     /EC_number="3.6.3.-"
                     /inference="ab initio prediction:Prodigal:2.6"
                     /inference="similar to AA sequence:UniProtKB:P32010"
                     /codon_start=1
                     /transl_table=11
                     /product="Daunorubicin/doxorubicin resistance ATP-
binding
                     protein DrrA"

/translation="MLIETYNLTKKYGKKLALNKVNLKVDRGQLVAYLGTNGAGKSTT

INILTGLLKPTSGTITYAKDLKIGVVFQSSVLDDVLSVNENLNLRAQMYRDVSKAWLE

QLIDLIGIRSFLHQKYGTLSGGQKRRVDIARALIDHPDILFLDEPTTGLDLQTRIVIW

NLLQKLQKEQGLTIFLTTHYLEEAENADQMYILENGQVLANGSAYEIKDKYAPSKLVI

TLKEKQRLITTYPVIERNKETEIDGLDPHEVITLLHENQASIKSFEYRQGSIDDAFIK
                     IAGKELQ"
     CDS   132965..133819
                     /locus_tag="OCPHGMPK_00827"
                     /inference="ab initio prediction:Prodigal:2.6"
                     /codon_start=1
                     /transl_table=11
                     /product="hypothetical protein"

/translation="MFALIKRNTKIYFANKPGVIMSCFGALISFVIYIGFLQQNLVNS

WQHVSHAKQLLDLWMLGGIVSIAGITTSFQALGQMVNDKENRAIDDMKLTALTPISES

MAYVVSATIVSFLMQIITFAVMTVYFSVVDKADVVHNAYLPCLGFMLLGALGATLLNL

IIVMFINSSTTFSRLSAVIGAAAGFMVATYMPYGTLSKTAQNIVKLFPSSYEAASFRS

LLLNKLSQQDVPTRMRDQLIEYLGIHFKFGSHQLNNFDNALVMIGMIVLLAVIIVILA
LLLNRKKK"
CDS 133823..134275
/locus_tag="OCPHGMPK_00828"
/inference="ab initio prediction:Prodigal:2.6"
/inference="similar to AA sequence:UniProtKB:Q5HDJ2"
/codon_start=1
/transl_table=11
/product="putative HTH-type transcriptional
regulator"

/translation="MDVEFHSNPNLEEDQIEVKVISARQTTNVNNLLSYIKNFDKKVD

SLLPVKTEDRILTIKIEEIIKVEVEKTELSFYTTNAVIKTNGRLYQILEKLNNDFVQV
SKHGIINLNYLESLEAGFTGNMIAKLAFKQKTDVSRRYLPELEKKLGL"
CDS 134277..134672
/locus_tag="OCPHGMPK_00829"
/inference="ab initio prediction:Prodigal:2.6"
/codon_start=1
/transl_table=11
/product="hypothetical protein"

/translation="MKIWQKALDYFVTGMGFGAIIYLVILGVTYENIGDLRVPLNSVF

IVFVCSGLIGELSFLFKTSLPYFWALILHLIGTFALFSIMILLNHWLINWQTLLIFVI
TYAIIWLIIRLIQEKDIRRINRQIKKRNG"
CDS 134719..135438
/locus_tag="OCPHGMPK_00830"
/inference="ab initio prediction:Prodigal:2.6"
/codon_start=1
/transl_table=11
/product="hypothetical protein"

/translation="MYPDKFNLTQEQNRRFAKKNLVRLVFTTSRFEGLNTTLPQTQTI

IDGMSAAGVSIDDTNVIVQLKRGWQYVIREDKPLSLNIEQNINLLVARYDSLDPGSFR

TGNVTVELGNSNGEWQPPEINLDQERKFFDELMQKDTSITDKAMTLMYHNMRNQLFWD

GNKRTATLAANKLMIDNGAGLINVPLNKWYKWNELISEFYLTGKMDKLKDWTYENGIQ
GIEFSSDPGVPKPTIDPKDYE"
CDS 135544..135957
/locus_tag="OCPHGMPK_00831"
/inference="ab initio prediction:Prodigal:2.6"
/codon_start=1
/transl_table=11
/product="hypothetical protein"

/translation="MAKNEEEVKNSVFGFGNSNEAFAKFFKGTSYLKALAESKEAKTA

VHNVSFEPGCRNNWHMHTVPQILIAVAGEGWYQEEGKAPQKMLPGDVVIVNPNTKHWH
GATKDSWFAHVVVMVGDSKNTWLEPVSDEEYDKLD"
CDS 136587..137954

/gene="brnQ_3"
/locus_tag="OCPHGMPK_00832"
/inference="ab initio prediction:Prodigal:2.6"
/inference="similar to AA sequence:UniProtKB:P0AD99"
/codon_start=1
/transl_table=11
/product="Branched-chain amino acid transport system
2
carrier protein"

/translation="MKIKKASIRQSMIIASLIFGMLFGAGNLIFPVHLGQLAGSNWLI

ASSGFLVSGVLLPLMALLAISITRSNGIYDLALPNGRYYALIFLILVHATLGPLFATP

RTATVPYEIGIAPYLSKSANTWGLLIYSAIFFSLVYIFSNHEGKITTLIGKVLNPLFL

LLLFLIFLLAFIHPLGSGNTAAITASYARHAFSNGFLQGYNTMDGLAALAFGITIITA

IKELGIKKTRQVSIETAKSGFLGIAGVAIIYLALIWLGATSLHQFKLAANGGITLAQI

SHHYLGTAGDAILATLATITCMTSAMGLVIAFSQDFHRRFPKISYRTFLRINCGLSFL

FANLGLDQIIAWSTPILMFLYPLAITLIILGLLSPIFKHDRLVYQITTALTLIPAFFD

MLNALPANLHDSQLLQTLLGFAQQYFPFFSLGFGWLTFGIAGLILGLICHVLKVRKRL
GLLSE"
CDS 138139..139116
/gene="cbh_2"
/locus_tag="OCPHGMPK_00833"
/EC_number="3.5.1.24"
/inference="ab initio prediction:Prodigal:2.6"
/inference="similar to AA sequence:UniProtKB:P54965"
/codon_start=1
/transl_table=11
/product="Choloylglycine hydrolase"

/translation="MCTSILYSPKDHYFGRNLDYEIAYGQKVVITPKNYEFEFTDLPA

EKSHYAMIGVAAVADNTPLYCDAINEKGLGVAGLSFAGQGKYFPNAADKKNIASFEFI

SYLLATYETVDQVKESLTNANISNVSFAKNTPASELHWLVGDKTGKSIVVESDEKGLH

VYNNPVNALTNAPLFPEQLTNLANYASVVPGEPDNNFLPGVNLKLYSRSLGTHHLPGG

MDSESRFVKVCFALNHAPKDSDEVENVTNFFHILESVEQAKGMDQVGPNSFEYTMYTS
CMNLEKGILYFNCYDDSRISAVDMNKEDLDSSDLVVYDLFKKQDISFIN"
CDS complement(139253..141049)
/gene="uidA"
/locus_tag="OCPHGMPK_00834"
/EC_number="3.2.1.31"
/inference="ab initio prediction:Prodigal:2.6"
/inference="similar to AA sequence:UniProtKB:P05804"
/codon_start=1
/transl_table=11

/product="Beta-glucuronidase"

/translation="MESALYPIQNKYRFNTLMNGTWQFETDPNSVGLDEGWNKELPDP

EEMPVPGTFAELTTKRDRKYYTGDFWYQKDFFIPSFLKKKELYIRFGSVTHRAKVFIN

GHEVGQHEGGFLPFQVKISNYINYDQTNRVTALVNNELSEKAIPCGTEEILDNGQKLA

QPYFDFFNYSGIMRNVWLLALPQSQITNFKLNYQLANNKATITYNIEANNNAEFKVTL

FDNQKEVACATSKNTSSLTIKNPHLWSPNDPYSYKIKIEMLEDGKTVDEYTDKIGIRT

VKIVNDKILLNNHPIYLKGFGKHEDFNVLGKAVNESIIKRDYECMKWIGANCFRSSHY

PYAEEWYQYADKYGFLIIDEVPAVGLNRSITNFLNVTNSNQSHFFASKTVPELKKVHE

QEIKEMIDRDQRHPSVIAWSLFNEPESTTQESYDYFKDIFAFARKLDPQNRPYTGTLV

MGSGPKVDKLHPLCDFVCLNRYYGWYVAGGPEIVNAKKMLEDELDGWQNLKLNKPFVF

TEFGADTLSSSHRLPDEMWSQEYQNEYYQMYFDIFKKYPFICGELVWNFADFKTSEGI
MRVGGNDKGIFTRDREPKDIAFTLKKRWQQLN"

```
     CDS             141211..141828
                     /gene="hmrR_1"
                     /locus_tag="OCPHGMPK_00835"
                     /inference="ab initio prediction:Prodigal:2.6"
                     /inference="similar to AA sequence:UniProtKB:Q9X5X4"
                     /codon_start=1
                     /transl_table=11
                     /product="HTH-type transcriptional regulator HmrR"
```

/translation="MANDKLLSISEFAKLAGTTRRTLIFYDQKDVFKPMKIEETGYRY

YSYEPLYQIGFILGLRDLGFSVEEIKDYLGGSSRDALNKKLISLKNKIELRIQNLKQL

LAILRQKENDNFQLTDVDFYVVKECFLPDHDFWCLDPKADCSEAEIASSCNQFYQELG
TGVMANKNLSGFITDLPQAKANTYANSGFRIIKKIIQSKSQHPDY"

```
     CDS             141873..142031
                     /locus_tag="OCPHGMPK_00836"
                     /inference="ab initio prediction:Prodigal:2.6"
                     /codon_start=1
                     /transl_table=11
                     /product="hypothetical protein"
```

/translation="MQELKKGLATIRKYTKENNLKIGNDLWQFNIGLNIEKLGLIKNG
ILAYKILK"

```
     CDS             142065..144575
                     /gene="uvrA_1"
                     /locus_tag="OCPHGMPK_00837"
                     /inference="ab initio prediction:Prodigal:2.6"
                     /inference="similar to AA sequence:UniProtKB:Q9WYV0"
                     /codon_start=1
                     /transl_table=11
                     /product="UvrABC system protein A"
```

/translation="MELPTQIEIKGARVHNLKNIDVNIPLHKFVAISGLSGSGKSSLA

MGILYEEGSRRYLDALSTYMRRRIKQGAQADVTSVKHIPSALALRQRPSIPNERATVG

TTTETFNVLRLIFSRLGSPVCPNGHRVAPSLDIAEAMSKSGEEMGQITCPTCGVKFYV

PSAEDFAFNSDGACQECGGTGKVRQLDDSKLIADPNLSIEDGAVASWSLPGRNFMPSV

AQHAGVRIDIPYKDLTDKEKDFVLNGPEKKYKMDFLSGTGRVFHDFNALYENAHQAVL

RSAKSSKSERAQKRIAEFFTYSTCPVCHGTRLRPELLKQLVNGKNIYQVEQMQLGDLP

AWSKEVLAKLPEDMHKMASSLINEFLNNLQPLLDLGLDYLTMERAGNTLSTGELQRIQ

LARTLRTETTGVLYVLDEPSIGLHPANVDGLIKVLRKLVDQGNSLVVVDHNVDIIKAA

DEIIEIGPGSGEQGGQILDQDSPEEIKKDKQSLIAPYLNGKAHLMARKVSDQVNSDQI

SFEVEDYFNLKDVQAGIPLNQLTSVTGFSGAGKTSLILDSLVPAIKAQDKSEPLPKQI

KNFDCPINNVVSVDASPIGKSTRSTVATYTSIMDNLRKLFAKQSLAKEKHYTPSYFSY

NNKEGACPTCGGTGIVTLDIQFLPDMQQTCPTCHGDRYNLGIQKIKWHDMSIVDVLNL

DVNEAIPVFKRVPKIERDLQLLKEVGLDYLHLGESTPTLSGGEAQRLKLVTHLSHKQD

DTLFVFDEPTIGLHPLDVKVLVEVMQKLLDQGATIITITHDLNLVVNADYMLDLGPRG
GENGGRIVAQGQPLELIKDPQSLTVEYLTNYLKQFNLD"

CDS 144921..147599
/gene="mgtA"
/locus_tag="OCPHGMPK_00838"
/EC_number="7.2.2.14"
/inference="ab initio prediction:Prodigal:2.6"
/inference="similar to AA sequence:UniProtKB:P0ABB8"
/codon_start=1
/transl_table=11
/product="Magnesium-transporting ATPase, P-type 1"

/translation="MLKFNSKDRVVTNATLAKQIARQEKNEVLKQLHTTINGLNPVQA

KQRLEQDGLNEVSNKEHHPKLHFLIDAFMTPFTGVLLFLAGLSFLTNYVFVPADQKDL

STVIIMIMMVLISGITSFIQNVKTSDAVDSLLNMVSVTTNVKRAGEDKELPTKDVVVG

DIINLSAGDLVPADMYLLKSKDLFCSASSLNGESSPVEKMADQKPKESDNYLDYPNIL

YEGTNIVSGSGMGVVFATGDKTVFGNLARTLAQNKNKETTFDIGIKNVSKILLIMTAV

IAPLVFLINDLTKGDWLNALIFAIATAVGLTPEMLPVIVTSNLVKGSVEMAKHETIVK

RMNSIQNFGSADILCTDKTGTLTQDKVVLERHYNLNLEEKPKVLELSYLNSYYQTGMK

DLIDKAVIDAAQGELDTKKINQNYQKIDEIPFDFKRRRMSVVVMNQQHEHILVTKGAA

EEMLACSNRVEIDGAISPLDEDQRQKILTEIDEMNQDGLRVVLLGYKENPAPVGEFSI

DDENELILVGFLAFLDPPKDSAKAALTALKEDGITVKILTGDNEAVTRNVGKQVGLNI

DQVYQGKDLENKTDAELKQMVQACDVFVKLSPQQKAQIIDLLRQDGHTVAYMGDGIND

APAMKAADVAISVDTAVDIAKKSADIILLHKDLMILEKGVQIGRQVFGNTMKYIKITL

SSNFGNILSILVASSFLPFLPMLPIQLLILDLMYGTSCLSIPFDNMNKHYLSEPRKWS

TKKLPKFMFYFGPTSSIFDIITFALLFFVVCPQVTGTSYAAASATQKLIFSAIFCTGW

FIESLWTQEMVIHALRDPKLPFIKQHATAIVTWSTIGMAVVGTVLPFITPIAKAIKFG
PIPMYFLAIVFLLLILYIALTTLVKKWYLKSEKYLI"

```
     CDS             147645..148640
                     /gene="lmrA"
                     /locus_tag="OCPHGMPK_00839"
                     /EC_number="7.6.2.2"
                     /inference="ab initio prediction:Prodigal:2.6"
                     /inference="similar to AA sequence:UniProtKB:Q9CHL8"
                     /codon_start=1
                     /transl_table=11
                     /product="Multidrug resistance ABC transporter ATP-
binding
                     and permease protein"
```

/translation="MQPISNMLMMILVIGILTYGAIRVMQGSLTMGALISFLMYLFQL

MGPVIMVSQFFNELAKTSGSTERIREMLNEPIEESADDQKIDVAGKELQLQNVSFSYE

KDKQILHNINVVAKPNTVVAFTGPSGGGKSTIFSLIERFYKPTSGKITIGNENIDNID

LTNWRHQIGLVGQNSAVMPGTIHEKLVYGLDHEVNDDELWRVLKMAYADKFVKESEDG

LDTQIGERGVKLSGGQRQRIAIARAFLRDPKILMLDEATASLDSESEAMVQKALTSLM

KNRMTLVIAHRLSTIVDADRIYFIDHGTVSGSGTHEELVKSTPLYAEYVHNQFKK"

```
     CDS             148974..149795
                     /locus_tag="OCPHGMPK_00840"
                     /EC_number="1.-.-.-"
                     /inference="ab initio prediction:Prodigal:2.6"
                     /inference="similar to AA sequence:UniProtKB:A0QV09"
                     /codon_start=1
                     /transl_table=11
                     /product="putative oxidoreductase/MSMEI_2346"
```

/translation="MKQLTLNNGIKIPIFGFGTYEISPDKTKQAVLSAFEAGYRLIDT

AQYYQNEQQVGEAVKASGLNRDEVFITTKTMTDGYEDTKIGLDESLRRSGLDYFDLVL

IHWPMGYDIDTWHALEAAYKAGKTRAIGISNFNSRQTLDLIHQSSVRPMVDQIETHLL

LQQWKMHEFLEKENIVHESYSPLGNGQQHLMGNPVLTEIGEKYGKSSIQVILRYLVQN

```
NVVTIPRSTNPAHIKSNIDIFDFELDSDDLKRLRVLDERKPIDGWPAEMREDEDY"
     CDS             150063..150200
                     /locus_tag="OCPHGMPK_00841"
                     /inference="ab initio prediction:Prodigal:2.6"
                     /codon_start=1
                     /transl_table=11
                     /product="hypothetical protein"

/translation="MLASGLKLKAKNNVLSLVSDEGVIDDTISINDITKDKQSFVLNK
                     I"
     CDS             complement(150436..151008)
                     /locus_tag="OCPHGMPK_00842"
                     /inference="ab initio prediction:Prodigal:2.6"
                     /codon_start=1
                     /transl_table=11
                     /product="hypothetical protein"

/translation="MNKYHKKSKKTDQIIESALLKLMDSSDLNKITMNELADMANINR

VTIYRHFNDKWAIIEQIEARLFTALKKPHQKMLDQLALKSDNGIEYLTNFLQVFKDNL

VTIRILISSHGDLSFSTKLMDHLLEMEGLSHSLMHLQANNDLQELFSYYSISALIGII
                     RFWTIHPNYSAKEMATFFFKMRNGELTKLS"
     CDS             151098..152264
                     /locus_tag="OCPHGMPK_00843"
                     /EC_number="3.7.1.19"
                     /inference="ab initio prediction:Prodigal:2.6"
                     /inference="similar to AA sequence:UniProtKB:Q93NG6"
                     /codon_start=1
                     /transl_table=11
                     /product="2,6-dihydropseudooxynicotine hydrolase"

/translation="MTEIFKKHWSEYKYSKNMLQDALIKHVMDLMPYRMSDEGEVLEI

IMNIKESDEQSWINAWSNMAANLQNRAHKFEKAGKLISAGSSYLRASTYWRVSLMYFG

DWQDPRMQEFSQRSKDCYDRALELNGYPGEAIRIPYENTYLPGHFYRSPFAKNKKAPL

LIVIPGRDTWADDTIWVTDAAIKRGIHALTFDGPGQGMALRLQDLKFRPDFENVLGPV

IDFAQKQEEVDSEQIGAMGMSFGGFLVARGAAFDDRLKFIISDPGNMNWGGMIAKRLE

KALKMPAMIRPNMIDFVLKDYEWKHGATDKTIINELKKFDNTKIVQNIKTKTLVLDGS

AEVTHGAAKKFYDALTCPKDYLLFDENSTAQQHTQMGGYATGTEYIMNWIEDNF"
     CDS             152285..152932
                     /locus_tag="OCPHGMPK_00844"
                     /inference="ab initio prediction:Prodigal:2.6"
                     /codon_start=1
                     /transl_table=11
                     /product="hypothetical protein"

/translation="MKLFLIGATGRTGSEIVKQALTRNDELVAYVRNPSKLNINDPEL
```

TVIKGQLDDVAKMASEMKGCNAVLVTLGNPISNSSGKLFSFAIPDIIKAMDQAKIKRL

ISLSALGVGTTLANTSYPYRMGAKGFLKGNFSDHEAGESQLKNSDLNWTTVHPGPLFN

GKKTENPLVRDADSGYKMPGAPRTYRSDVAQVMLRIIKDRKTFGKQLIMCSKQHK"

```
     CDS             153166..154398
                     /locus_tag="OCPHGMPK_00845"
                     /inference="ab initio prediction:Prodigal:2.6"
                     /codon_start=1
                     /transl_table=11
                     /product="hypothetical protein"
```

/translation="MHKNIYYSWASNFYRLFIGQGLASIISETVNYSLLFYLTSISKP

ATILSISEIISLLPIALFAPFAGVMADRVSKKKILIFSDTISLIVTLLLFLQAINSHN

NLTIDLILISNLLRSTTISIQNPTIQSAVPELVPDDKLLKINGQYSSLQALNQFFPPI

LGGILYGLLSVDYILLLSGLANVIGICAVIITCFPRNEVNNANQRGFHLLFELKQGLH

SIIQVTGLNRVIIYKAFSVAIIVPSTALYPLMTSEHFHGSLKFDAPLVEVSIALGMAL

SGLCMGNLNNLSHRLTPAGIGVLGIGLSLFISGILPGNHYGFIIFIIINFIAGLCIPW

IDAPAQTLLQDKIEHKNLGKVISFYLMMIGLAGPAGLSLSSVISKFMSTAEIFIGSGI

```
                     LLILMLLVALFDRKLRSI"
     CDS             154723..155115
                     /locus_tag="OCPHGMPK_00846"
                     /inference="ab initio prediction:Prodigal:2.6"
                     /codon_start=1
                     /transl_table=11
                     /product="hypothetical protein"
```

/translation="MNNIKENIVLAFFVGLFLGAISIFLAIGGGPLNVSLFVIIFHFT

MKQSSVYSIATVFFSQITKIISIVASAQYQMFDMKMIPMLIIASIIGGYIGTVWNQKI

```
                     SSAKLENLYTVFMIAITAITGFNVIHFI"
     CDS             complement(155311..155577)
                     /gene="ribZ"
                     /locus_tag="OCPHGMPK_00847"
                     /inference="ab initio prediction:Prodigal:2.6"
                     /inference="similar to AA sequence:UniProtKB:Q180E3"
                     /codon_start=1
                     /transl_table=11
                     /product="Riboflavin transporter RibZ"
```

/translation="MKNKRIAALIAIAIFTFMTFFNLNSPLWLFVVTFLFYGVRMGLF

```
                     QSPKNSVIMSSVDQQFLGIAGSVNSLVRNFGMELGVSLSTIILL"
     CDS             155755..157119
                     /gene="npr"
                     /locus_tag="OCPHGMPK_00848"
                     /EC_number="1.11.1.1"
                     /inference="ab initio prediction:Prodigal:2.6"
```

/inference="similar to AA sequence:UniProtKB:P37062"
/codon_start=1
/transl_table=11
/product="NADH peroxidase"

/translation="MDKRKIVIVGASHGGHESAIELLDKYNDVDVTVYEAGDFISFMS

CGMQLYLENKVTAEDDVRNFAPEDVEKKGGHVYANHEVTAIHPENKTVTVKDLTNNSE

EEVQYDRLILSSGVTPKVLPVPGNDLKNIYLMRGRDWASKLMSAANDPAIKNVAIVGA

GYIGTEASEVFAKAGKHVTLMDMIDRPLGTYLNSELLDVLEPTFKKNMDLKMGVKIEG

FNGNGKVESVKTDQGDVPADLVVVSAGVMPNTDWLKGVVDLDQRGWIKTDPYLRTNVK

DVYAIGDAILPLSIPAGKPMPIALATTTRREAQYVVDHIFEDKPDRAFKGVIGASALS

VFDYHFATAGLNQFSAAKNKLDYQTSFYEDNMRPAYVPEADNPKVYVSLTFNPYTHQI

LGGAVLSKYDITAQGNVLALAISHKMRLEDLAEQDFFFQPGFDRQWSLLNLAAQHALG
MARF"
CDS complement(157224..157685)
/gene="ribH"
/locus_tag="OCPHGMPK_00849"
/EC_number="2.5.1.78"
/inference="ab initio prediction:Prodigal:2.6"
/inference="similar to AA sequence:UniProtKB:P11998"
/codon_start=1
/transl_table=11
/product="6,7-dimethyl-8-ribityllumazine synthase"

/translation="MTEYTGNFTNTHGKIAIVVAKFNKIVTKNLVRGAKETLHQFGIQ

DDEIDVIWVPGAFEIAFTAQKLIKSDKYAGIMTLGAVIKGETDHYAMIIQNVTNAIMQ
MNLQAKIPVTFGILTTENIEQALQRAGLKAGNEGSSTAQSLLEMISLNEQL"
CDS complement(157685..157975)
/gene="ribA"
/locus_tag="OCPHGMPK_00850"
/EC_number="3.5.4.25"
/inference="ab initio prediction:Prodigal:2.6"
/inference="similar to AA sequence:UniProtKB:P0A7I7"
/codon_start=1
/transl_table=11
/product="GTP cyclohydrolase-2"

/translation="MEKDQYYMNLALQEAKKGRFQTWKNPLVGAVIFKELKIKEINLL

TNNPDKIDQLNDYGIKINKRIPLELASNDVDRFYLQTKKKRFHHLLELKEAE"
CDS 158746..159678
/locus_tag="OCPHGMPK_00851"
/inference="ab initio prediction:Prodigal:2.6"
/codon_start=1
/transl_table=11
/product="hypothetical protein"

/translation="MKKSERLNQELLYLTDKKIFHLKELEEKFAISERTALRDITDLE

QLGLAFYSETGRSGGYYITNSKLLLPIRFNTQEINAIFFALQAIKNLSETPYSNEYQK

IQTKLLKNLPQKLQNQISLQKEFVHYISQPSLNEVPFFTLLLKACLNNSVILARNFQF

IKEEQKLQLLDIFYQAGNWFCHAYNLDLKRWFVLRLDKFETVKLLNTKQSVMNKEQLL

DSFRQYEREHYCIPYQCQLTKIGEQKVEYNSYPIMQIDHKHGKIYLNGKYNKEELHYL
VDYLISLGSEVKVLKPKELKNSYLEKLNEIIQQY"
CDS 159752..160138
/locus_tag="OCPHGMPK_00852"
/inference="ab initio prediction:Prodigal:2.6"
/inference="similar to AA sequence:UniProtKB:Q46604"
/codon_start=1
/transl_table=11
/product="FMN-binding protein"

/translation="MTKIMNEDFLDVLEDKGPATIISINGNPASVVNTWSQYINVVAD

DTILIPAAGMHSIEQDFKKDSKHELTITIGSYNYQGTAGMGRGYHIHGSGQFINEGAY
FDQMKAQFDWIRAVLVVKIHDVEQKI"
CDS 160284..160925
/gene="fetA_2"
/locus_tag="OCPHGMPK_00853"
/EC_number="3.6.3.-"
/inference="ab initio prediction:Prodigal:2.6"
/inference="similar to AA sequence:UniProtKB:P77279"
/codon_start=1
/transl_table=11
/product="putative iron export ATP-binding protein
FetA"

/translation="MGKRTILNNLSFGIKNGDFLTLIGPSGAGKSTILKLIANLISPS

SGTIFYHGSDIQKLDPIKYRRQVMYCFQQPVLFGKTVKDNLSFPYQIRNKEVDLARLD

SLLKIVDLPAEFLNKEINSLSGGEKQRVALIRSIVFLPKVLLLDEVTAGLDGKSKEIV

HRLIESVHRQGTTIVQVTHDQSEIQVAKNVLEIKSRGLVNEKSRGLVNEKCNG"
CDS 160906..161025
/locus_tag="OCPHGMPK_00854"
/inference="ab initio prediction:Prodigal:2.6"
/codon_start=1
/transl_table=11
/product="hypothetical protein"

/translation="MKNVTVSNLSLVLAFALVLVSIAISAKEKLGLTKDILLA"
CDS 161519..163420
/gene="zosA_2"
/locus_tag="OCPHGMPK_00855"
/EC_number="3.6.3.-"
/inference="ab initio prediction:Prodigal:2.6"

```
                     /inference="similar to AA sequence:UniProtKB:O31688"
                     /codon_start=1
                     /transl_table=11
                     /product="Zinc-transporting ATPase"

/translation="MVKIQQFFVKHQKIILALNTVLILFAETAKWLFHINAAYQVLML

IVGIIGLIPILLTAISSLKVKLVSIDVLVSLAVIGAIVIGEFNEAAIVTWLFALGDVL

EELTLKKTRKAVSDLTKMAPKTALVLQDDGATEEEDIDFIDLGEKILIKTGDQVPVDG

KIVAGSGYLNEANVNGESKPADKKQGDQVYAGTILEDGTLTVETTAAGDDTTFGKIIE

MVEEAQDSKSHTEKLINRFSKYYTPAVLVIAIIVGLITRDFKLTITVMVLGCPGALVI

GVPVSTVAGIGNGAKNGVMFKGSQVMDAARKVDEIAFDKTGTLTVGHPEVNKIEVLSG

NKDEIINLAANIESQSNHPLAKAMAKLSEQKIDQIETKTVKGNGMIADLDREKYYLGN

LALIEKNTFANSKLTQVVNKLSNLGNSVVVLANSNQSKLAVFGIKDRLRPEAETALRQ

LKQLGIKKLIMLSGDNQETAQQIAADLPIDEIHGQMLPADKAQFVKKEQENGHHVAFI

GDGINDSPALANADIAIAIGSGTDVAVDVSDIVLVKNDLRKIADALSISKRTVLNMNE

NIGIALLTVLLLFIGLFTGYVEMANGMFIHEFSILVVILNGMRLIKNHRKVDGHQFSD
                     KEKDLALNM"
     CDS             163443..163673
                     /gene="copZ"
                     /locus_tag="OCPHGMPK_00856"
                     /inference="ab initio prediction:Prodigal:2.6"
                     /inference="similar to AA sequence:UniProtKB:O32221"
                     /codon_start=1
                     /transl_table=11
                     /product="Copper chaperone CopZ"

/translation="MQKVMMKLGGMTCPSCLTKIEKAVEDVDGTDQIKVLFNAGKLKF
                     MMNTDKANVDDVKTAIEKMGYEVKGVKAKELD"
     CDS             163673..164224
                     /locus_tag="OCPHGMPK_00857"
                     /inference="ab initio prediction:Prodigal:2.6"
                     /codon_start=1
                     /transl_table=11
                     /product="hypothetical protein"

/translation="MKAKEAYKAELKQSDIDHHTPTAGAMVDHIIANLLIHTLKIDQA

KFFAKGSASLFLEQRADEWIRYEQNEFNQLNQILVNNGESIPTTTDQFKEYTMLEENG

ASKYENGQDQLFALVKDFDTQILFITKAIALTQKETWPELSANLIELLAWIKEQIRQS
                     QNFLGHELRERLYTEEDDDDDDF"
     CDS             164242..164871
                     /gene="aadR_2"
                     /locus_tag="OCPHGMPK_00858"
```

```
                     /inference="ab initio prediction:Prodigal:2.6"
                     /inference="similar to AA sequence:UniProtKB:Q01980"
                     /codon_start=1
                     /transl_table=11
                     /product="Transcriptional activatory protein AadR"

/translation="MAELCVHLVPLFNALPINDQMQIEELVHHQNYQNGELVMDPTSS

NNLVIVAHGGARLYTLDENGRENITQILKTGDYAGENWLFGEANTNTYVEATENSDIC

LLNRNDFLALMKKKPELSIQLLEQNIIKVRAMHKQIQLLSLPKVEDRLLSYLQAYAKQ
                     VNKNSFTLPLKMKDLALYLGTTPETLSRKFVLLEKQGSLKRKLRHIDLL"
     CDS             164998..165516
                     /locus_tag="OCPHGMPK_00859"
                     /inference="ab initio prediction:Prodigal:2.6"
                     /codon_start=1
                     /transl_table=11
                     /product="hypothetical protein"

/translation="MSLIYIRQAAKNDLEQIMPIIDEAKKFLKEEKNPQWQSGYPNVE

TITADIKDGAAWVLIVDQKIAVYTAITDGPDPNYKQIDGKWKNDLDPYVAIHRVAISD

EYRGMHLASYLMSSLTSMHYAESIRNYRVDTFRTNDVVWHIVEDAGFVKRGNIKNMDP
                     IDSYRVAFELNL"
     CDS             165590..166135
                     /gene="garR"
                     /locus_tag="OCPHGMPK_00860"
                     /EC_number="1.1.1.60"
                     /inference="ab initio prediction:Prodigal:2.6"
                     /inference="similar to AA sequence:UniProtKB:P0ABQ2"
                     /codon_start=1
                     /transl_table=11
                     /product="2-hydroxy-3-oxopropionate reductase"

/translation="MKIGFIGIGVMGTGIINNLRKNNYDVTVYNRTKAHAQTVLDNGA

KWADNPAELTKKVDILFTMVGFPKDVEDIYFGQDGILIVAKAGQYVVDMTTSKPSLAQ

KIDQIGEEKGLHILDAPVSDGDIGAKNGTLTVMIGGHKEDLNHLHDLFITFASKLNYF
                     GSYGSGQHAKIANQIMIAGEP"
     CDS             166132..166326
                     /locus_tag="OCPHGMPK_00861"
                     /inference="ab initio prediction:Prodigal:2.6"
                     /codon_start=1
                     /transl_table=11
                     /product="hypothetical protein"

/translation="MTGMTEMLVYAKAAHLNLEQICQTLQSGAAENFSLDSYGPKILQ
                     GDYTPGFFAKHFLKDLRIAL"
     CDS             166339..166455
                     /locus_tag="OCPHGMPK_00862"
                     /inference="ab initio prediction:Prodigal:2.6"
                     /codon_start=1
```

/transl_table=11
/product="hypothetical protein"

/translation="MQLDLPATEEARHLYEILVDQKKLGNDGTQALIKLWWK"
CDS 166573..166887
/locus_tag="OCPHGMPK_00863"
/inference="ab initio prediction:Prodigal:2.6"
/codon_start=1
/transl_table=11
/product="hypothetical protein"

/translation="MKIYNYINKVKKLDILLSKEIAQILLRQGVPFTKSQIEVVMYLY

HHQDQVVYQRDLPDILGITRPMANGLVKHLIQKNAVKLIEDSDDSRLKRIKLMPVVIA
EY"
CDS 167142..167663
/gene="ogt_2"
/locus_tag="OCPHGMPK_00864"
/EC_number="2.1.1.63"
/inference="ab initio prediction:Prodigal:2.6"
/inference="similar to AA sequence:UniProtKB:P9WJW5"
/codon_start=1
/transl_table=11
/product="Methylated-DNA--protein-cysteine
methyltransferase"

/translation="MQKIAYYNSPVGQILLASDEQGLTGLWLRSDRFYADNLAKDHQL

GKNKYLDDAIRWLDLYFEGKNPKFLPKLHLIGTDFQNQVWLALLEIPYGEVTTYKKIA

EKVAGRADHLPIRAVGSAVGRNHIALIVPCHRVIGSNGNLTGYSGGIDKKIQLLQLEG
IDISKFSLPANYK"
CDS complement(167654..168112)
/gene="ywrO"
/locus_tag="OCPHGMPK_00865"
/EC_number="1.6.99.-"
/inference="ab initio prediction:Prodigal:2.6"
/inference="similar to AA sequence:UniProtKB:P80871"
/codon_start=1
/transl_table=11
/product="General stress protein 14"

/translation="MKTTIFLFHPDLKESSVNQALIKDITTEVRNIYEPYPDENINIK

AEQDALLRSDRIVFQFPMYWYSVPPLMKKWFDLVLEHGWAYGSKGHALDNKEVLVAVS
TGARLADYQFGSKQNHTINEYLLPLFATFASTRMKILKLAQLPLKQSRHL"
CDS complement(168378..169151)
/locus_tag="OCPHGMPK_00866"
/inference="ab initio prediction:Prodigal:2.6"
/codon_start=1
/transl_table=11
/product="hypothetical protein"

/translation="MGIVAKRIAMAKSHLKIMENKYTNETNLSIKQSKIYSPKDFLQK

```
PHIKYTHYANLSFKEENTAHAANNLKNIYPNLKTAILSFADYLTPGGGYLNGSGAQEE

VLCHESNLYSVLTNFTDFYQWNREHINHHLYQNRAIYSPNIVFTNLDGTLVNKFDVIT

CAAPCYNRANFDNISYRIACEVLQNRMYFVKTIAEENHVNNLILGAWGAGAFGFRGKE
                     VAKMWHQIWAESSSVENVCFAVIEHISTNNVDIFEAEFS"
     CDS             complement(169307..169522)
                     /gene="sugE"
                     /locus_tag="OCPHGMPK_00867"
                     /inference="ab initio prediction:Prodigal:2.6"
                     /inference="similar to AA sequence:UniProtKB:P69937"
                     /codon_start=1
                     /transl_table=11
                     /product="Quaternary ammonium compound-resistance
protein
                     SugE"

/translation="MTWIYLVIAGIFEVVWSTTMKLSQGFTKIGWAIATIIGMIASFA
                     GLIIATKHLPLESRIQFGQKLELLDQY"
     CDS             169806..170888
                     /gene="luxA"
                     /locus_tag="OCPHGMPK_00868"
                     /EC_number="1.14.14.3"
                     /inference="ab initio prediction:Prodigal:2.6"
                     /inference="similar to AA sequence:UniProtKB:P29238"
                     /codon_start=1
                     /transl_table=11
                     /product="Alkanal monooxygenase alpha chain"

/translation="MNIELGISSFGETTQLEETGKAISHDQRIRDLLEEIILADKLNI

DAFAIGEHHRKDFAVSAPEIILAAAASKTKQIHLSSATTNLPTINPIRVYEQYATMDA

IAPGRVEIMAGRGSFTEAFDLFGYDLENYDELFIEKLEMLLKMNKEEVLDWPKGKFTP

RVDHKGIYPRTKKPIPISVATGGSIESTIRTAELGLPIVYAIIGGDPLHFKPLIKLYR

RVAEKVGNDLTKMPIAAHSWGWLNQDKEKAIKDYFYPTKLLVDQISMERPQWNGMTYE

QYLEAVGENGAIFVGDSETVANKIIKVMEELGLTRFYLHLPIASMPHEDVMNAIRIYG
                     EEVVPKVKAYFRKKTEQDCLKLKWKN"
     CDS             170980..171471
                     /gene="ypeA"
                     /locus_tag="OCPHGMPK_00869"
                     /EC_number="2.3.1.-"
                     /inference="ab initio prediction:Prodigal:2.6"
                     /inference="protein motif:HAMAP:MF_01127"
                     /codon_start=1
                     /transl_table=11
                     /product="Acetyltransferase YpeA"

/translation="MIIRTVTMDDLNDIVELESDAFKMTKKQTEQDMIGRIENYPDTF
```

LVAQEDGKVIGHIFGPAFNKRYIEDELYFKNHPNQKKDRYQMVLSLAVLPKYRKQGVA

TKLIEALAQEAIKQNRQAISLTCLPKLIKFYEKRGFHNEGETNDDIPDPTGVTSVNMV
RAL"
```
     CDS             171473..172129
                     /locus_tag="OCPHGMPK_00870"
                     /inference="ab initio prediction:Prodigal:2.6"
                     /codon_start=1
                     /transl_table=11
                     /product="hypothetical protein"
```

/translation="MTKLTEEMYAIFDRDEFSFKKLKEEYSEEELAQIKASFKDVWQT

WKKVNLSVYLKLPQDKFAKVHVESWTNGWNLRDHYWAAYRLNTLAEKSPCIGVMLDKK

QLQVYLMFQHYKSEKRGDSSEQYNKLLADIPTWAKNRKISNWYLWDKNEMEFVDHLSL

NDYLKDTQKQTTFNEEAIKTSFLLGKFAFRNQDQVENMEEFILDGIKQLLPLYEKIER
"
```
     CDS             172208..173488
                     /gene="rarA"
                     /locus_tag="OCPHGMPK_00871"
                     /inference="ab initio prediction:Prodigal:2.6"
                     /inference="similar to AA sequence:UniProtKB:P0AAZ4"
                     /codon_start=1
                     /transl_table=11
                     /product="Replication-associated recombination
```
protein A"

/translation="MPIRKPLADLIRPKDLSQFVGQKDLIEKERPLYQIIKKHVPISL

LLWGPPGTGKTSLAQIIAREFDYPLATFNASIDNKAKLTQIINTYPYQSFVLLIDEIH

RMTTTLQDFLLPYLENGRILLIGATTENPIMSIVPAVRSRCQIFEFKALSDEDIEQVL

IRALKEVFHFKDENIDRKAVKIITISADGDLRVALNLLETIHAVNEDLSVEEVRQFVK

GQHFAYDRKATKHYDYLAAYSDSMAGSDTDAALYYLAVLLKNGDLPSVVRRLREIPYT

YIGLANPEQATQIVIAANQAEKIGMPKAKYPLMFATMLMCLSPKSGVFDEIWQKLDED

TDHPSNHPMPRGLRDMHYKHSEEITGGGLIDNPFAQPFQIAKQNYMPKGLEGKRYYFA
KDNASEKKFEQQYLKLHQYIYGKDYDKEGNNYEN"
```
     CDS             173774..174382
                     /locus_tag="OCPHGMPK_00872"
                     /inference="ab initio prediction:Prodigal:2.6"
                     /codon_start=1
                     /transl_table=11
                     /product="hypothetical protein"
```

/translation="MLAMNNIPASLKILENIEKSPYLVGAQIFTRHLGKSIADLEFEP

ILLKAAELNLPLWLHPVFDNRKPDNNLVFSWEYELSQAMLQLVQSDIFKKAPDLKILA

HHAGAMVPFFSGRIDHILDQEHAEMFKNFYVDTVILGNTPALQLALDYFGINHVLFGT
DAPFAVMPAGADKIVTDAIENLDIFDLYDKKIFETNYQHMIK"
CDS 174522..175235
/gene="ybhL_1"
/locus_tag="OCPHGMPK_00873"
/inference="ab initio prediction:Prodigal:2.6"
/inference="similar to AA sequence:UniProtKB:P0AAC4"
/codon_start=1
/transl_table=11
/product="Inner membrane protein YbhL"

/translation="MYNFDQEPERRHLVDESGLNTFLSKLYGLMALAVLVSAVSSYLT

VTIFARQMLNLMNHHPWAMWFLLLLPIALTLVINFNATRSPVMSFVLLMLTAIVYGVT

FAFIAGAYTGEDIASAFVASASVFIVMAILGTVTKRDLSRIGSYASAALIGLIVAMLI

NLFLKNPTIDYIFSFIAVIIFTILTAWDAQRMKNIYLQFDGELSTNGLTVLGALQLYL
DFVNLFLQFLDIFGSNDNN"
CDS 175351..175587
/locus_tag="OCPHGMPK_00874"
/inference="ab initio prediction:Prodigal:2.6"
/codon_start=1
/transl_table=11
/product="hypothetical protein"

/translation="MTANEKIIALVKPEYLKKIPAIFRKHATEGTCKLIAREHPDLYS
TFEKEVEPTEEEKQEMANLVNGIFEERMKKHKML"
CDS 175621..176415
/locus_tag="OCPHGMPK_00875"
/inference="ab initio prediction:Prodigal:2.6"
/codon_start=1
/transl_table=11
/product="hypothetical protein"

/translation="MEIGTMLRNKRKILGLTQDQFIHGIISKSFYSKVERGLNEISAK

DLFAILEANNINLNDFINNAGSTSKSDRLKVEILVAFNNKNKHEVQELNKKIQVSNCD

QTIKLSSLLINAILNNQLDKINLNTRNEIKKKLFDVEKWWNNVYTLQLFCNSMLIFSF

DDLTFYMQQLTKQKNINSLSFDVQRLIGSICINYFHNCYKNHQTDCAKWGYQIIDSLA
KLPDLAIYRIIKNYYQNFFTGNQKEASKIIDFFKDYDLKEIYTVLP"
CDS 176510..177625
/locus_tag="OCPHGMPK_00876"
/inference="ab initio prediction:Prodigal:2.6"
/codon_start=1
/transl_table=11
/product="hypothetical protein"

/translation="MNPTYLKSNLLIGLKSKKNQLLIILLVLFIIFSLFIVEKQKIGD

GAKSWENYGQSLQANANYFDSEMLKRPTYKSTYDNLNKQSQELASLQNSQVFVDPVQY

LTSYRNLLNTMQAGYHNHYAGAKTLNLPSQFFIKQELNKTDYLIKHKIPIFMNNESSA

TYLIYILSVLGTIIFFYLSFISSDSWIINLRHQSILKNIPYRFKDEVFGKIIINSTLV

FLPLLLGMILAYIFAGLRHGFVTLNYPVIFYSTSILAIPLWLYCFLFLVYLSGLIIFT

TSLSLFLNQLTKNVYLTIFIEVILYALLFLPKATLKWLVFLPFPYLNLTDLLNGNLAN
KLNLNSINLLTGYCVLLIWSIFLLILFKYLAAGRKKQ"

```
     CDS             177622..178869
                     /locus_tag="OCPHGMPK_00877"
                     /inference="ab initio prediction:Prodigal:2.6"
                     /codon_start=1
                     /transl_table=11
                     /product="hypothetical protein"
```

/translation="MKKQYFLFQLKRFFTNPKNIGLFALTFILSLYFGLVSVPNRQLI

EKVDPYAINGEYRRYTLFLNKATTKIKEMQESKNTHEVPNEGYVNAIKTYPDILNFDK

HRINGIKANNWKEYVIYSSKWYKYVDHLIFVDENQQFLYPVQYYQNDNYREDGHFAYQ

RTAHFYDALAKSKQPLTKNIVEEKTTLQILQNSISGWTTLILILFVVLFAADIVTNDR

KYYSVLKGIPLSKNTILWIKTGVVEIGVLFNYLLAFIVVTLCTAPRYGFGSFNLRTTF

YTGRLYFIQPFKYPTLGNYFIQFGTFAILIAFLFIRLTILLSIIFKNEYVAELLASVF

AVSGKVLYFSLGMGFVYPILQSLPMTYFTVGESLSGNLSYLMDSPGWGFNSGIVPLML
SIVIIEIFMLIISRSKRIKLIRR"

```
     CDS             178876..179745
                     /gene="natA_1"
                     /locus_tag="OCPHGMPK_00878"
                     /inference="ab initio prediction:Prodigal:2.6"
                     /inference="similar to AA sequence:UniProtKB:P46903"
                     /codon_start=1
                     /transl_table=11
                     /product="ABC transporter ATP-binding protein NatA"
```

/translation="MLELKNIKLSFGNHIVLDNLNYSFQSGKIYGLVAPNGTGKSTLM

NVILNNLKPQNGYIAYDNLQYKNEKTIVRLHKKICAFPDQSDLFPFMTGKDHLKLYAN

LWKNNPESIEKIISTLKMESYVSRKVETYSLGMKQRLCFAMVVAANTPVMLLDEVMNG

LDQQNVQLISQVLLDLRSQKKLIIMASHLLQNLQQYADQVLFLKDGKIIEDFNNKAET

KQYLEFKSSALLTDILNNVEVQTLPNKTNLVAIDNINQETFNQITNYLIKNNISFSVT
KMSLENLFNKFYG"

```
     CDS             179772..180149
                     /locus_tag="OCPHGMPK_00879"
                     /inference="ab initio prediction:Prodigal:2.6"
                     /codon_start=1
                     /transl_table=11
```

/product="hypothetical protein"

/translation="MAHPFGTQTVRRKQDLFSEKDKYGKTVDIDRPIIDEVEKIAQNH

NTSMATIALAWLKTKVTAPIVGATKAHHLDALEEAIKINLSKEEVHALEAPYKAHDLE
GVMADNRDREHNWTQYLTNSAEK"
CDS 180219..181094
/locus_tag="OCPHGMPK_00880"
/EC_number="1.-.-.-"
/inference="ab initio prediction:Prodigal:2.6"
/inference="similar to AA sequence:UniProtKB:A0QV10"
/codon_start=1
/transl_table=11
/product="putative oxidoreductase/MSMEI_2347"

/translation="MTEVPTITLNDGNKMPQLGLGVFRIPDHEKARQTVEMALANGYR

MIDTAEAYDNQIAVGEAIAESAVNRDDIFLTTKIWVSNMSYEKATKAIDHDLKELGTD

YIDLVLLHQPYGDTFGAWDALAAAKRAGKIRSIGLSNFYPDQYMNLELAHEDKPAINQ

IEISPWFQQTNDVKFFQGRDVAVEAWAQFANGKHDIFNNKVLKEIAENHHKTVGQVIL

RWLTQKSIVVIPKAVHDEHQKENINIFDFKLTQDEMAKIKELDKGESQFFDHRDPAAI
DNIFGESLRQLRDSE"
CDS complement(181141..181791)
/gene="nth_1"
/locus_tag="OCPHGMPK_00881"
/EC_number="4.2.99.18"
/inference="ab initio prediction:Prodigal:2.6"
/inference="protein motif:HAMAP:MF_00942"
/codon_start=1
/transl_table=11
/product="Endonuclease III"

/translation="MKKIDLNELYDLMYDHLDPNGWWPGRSDWQVIWSTILIQNTNWK

NVDKALATLYQATNFWPENILKMPDDKLEKAIASAGFYTRKAATLKRLATYFQKYDFD

LDKCRQLSKDQLRSELLSIKGIGPETADVILMYGIQKGEFVVDKYARRLFNCLDYQLP

VSYQKAKDLVEANVDHFTLRNYQNFHAMIVIFNQQYKLPKDFESTFLGDYHLVIAK"
CDS 181914..182528
/gene="maa"
/locus_tag="OCPHGMPK_00882"
/EC_number="2.3.1.79"
/inference="ab initio prediction:Prodigal:2.6"
/inference="similar to AA sequence:UniProtKB:P77791"
/codon_start=1
/transl_table=11
/product="Maltose O-acetyltransferase"

/translation="MEENTKKMLAGLPYRPDTEELSRISTKAHRLSRDYNQTADEDKE

VRKAIIDELFPNHETGVYLQGPIEVDYGKFTKLGKNFYANFNLTILDTCPVTIGDNVM

CGPNTSLVTPLHPLLPEQRNARLQKDGKIADIEYGAPITIGDNCWLASNVTVCPGVTI
GKNCVIGAGSVVTKNIPDNSLVLGVPGRVVRKITEKDRLDNYPY"
CDS complement(182631..183290)
/gene="plsY_2"
/locus_tag="OCPHGMPK_00883"
/EC_number="2.3.1.-"
/inference="ab initio prediction:Prodigal:2.6"
/inference="similar to AA sequence:UniProtKB:Q45064"
/codon_start=1
/transl_table=11
/product="Glycerol-3-phosphate acyltransferase"

/translation="MSTLNYLLVFILAYLIGSFPTGVLVGKIFFHEDIRNFGSGNIGT

TNSFRVMGPIAGSAVLIIDVLKGTLATDLPLIFHLKGPKYLLLIAGACAILGHTFSIF

LKFKGGKAVATSAGVFLGYNLKFFGLCAVVFLPMLFITSYVSLSSLVSIVIIFICSFW

FHDVFLTIITGIMMVLLFVRHRSNIKRLINHEENIVPFGLWYWYKKSHGLLNSKSKIN
K"
CDS 183390..185348
/gene="parE"
/locus_tag="OCPHGMPK_00884"
/EC_number="5.6.2.2"
/inference="ab initio prediction:Prodigal:2.6"
/inference="similar to AA sequence:UniProtKB:H7C794"
/codon_start=1
/transl_table=11
/product="DNA topoisomerase 4 subunit B"

/translation="MAKTNKTTSSYDDSSIQILHGLEAVRKRPGMYIGSTDRHGLNQL

VYEIVDNSVDEAMAGYGKEINVTIHKDNSVTVQDFGRGMPTGMHASGIPTIEVILTVL

HAGGKFTEKNYQTSGGLHGVGSSVVNALSSYLKVRVVRDGQAYEEEFENGGHPIGTLK

HLGKTKDKNGTTITFKPDTTIFSTTTYNYETIQERIRESAFLLKGVKFTLTDEREPEH

HDEFLYEDGIKSFVSYLNEDKDTMGDVFYFSGQQNGIEVEFAGQYNDGYSENFVSFVN

NVRTADGGSHEAGARSGFTKAFNDYAKKQGLLKNKDKGLEGSDYREGLSAVLSVKIPE

ELLEFEGQTKGKLGTPQARSVVDSIVYEQLSYYLMENGEFAQSLVQKAQKARDAREAA

KKARDESRSGKKRRKKEILSGKLTPAQSRNPKKNELFLVEGDSAGGSAKQGRDRKFQA

ILPLRGKVLNTQKAKLQDIFKNEEINTMIHTIGAGVGSDFQIKDANYDKIIIMTDADT

DGAHIQILLLTFFYRYMRPMVEAGRVYIALPPLYKLQKGNGAKAKIKYAWTDEELSED

SKDMGKGFALQRFKGLGEMNADQLWETTMNPETRTLIRVKIDDAQLAEKRVTTLMGDK

VAPRRKWIDENVKFRMGEDGSILETSRD"
CDS 185365..187845
/gene="parC"
/locus_tag="OCPHGMPK_00885"
/EC_number="5.6.2.2"
/inference="ab initio prediction:Prodigal:2.6"
/inference="similar to AA sequence:UniProtKB:P72525"
/codon_start=1
/transl_table=11
/product="DNA topoisomerase 4 subunit A"

/translation="MVKTTERIRELPLEEVMGERFGRYSKYIIQERALPDIRDGLKPV

QRRILYAMYKDNNTYDKPYKKAAKAVGNVMGNFHPHGDSSIYGALVHLSQDWKMREPL

IEMHGNNGSMDGDGPAAMRYTESRLSKISNLLLQDIDKETVQMILNFDDTEYEPTVLP

AHFPNLLVNGSTGISAGYATEIPPHNLAEVIDAAVYLLKHPDATTDDLMEFVKGPDFP

TGGIVLGTKGIKEAYETGRGRIQVRSKTTIQDIKGHRQQIVVTEVPFGVNKAMMVKKI

DEIRLNKEIDGISEVRDETDRHGLSIVIELKKGADSQNILNYLFKNTDLQVSYNFNMV

AIDRMTPVQVGLKRILSSYLTHKKEVVTKRTKFDLKKAKQRLEIVEGLIHALDILDQV

IKTIRSSKDKKDAKNNLIAKYEFTPNQAEAIVSLQLYRLTNTDVNQLKKEQTELNKKI

DKFNEILSDNKVLEKVVVKELNAVKKEFGSPRRTEITAKAAKIEINEKALVADEDVRV

LVSKDGYLKRSSIRSFQSTDTSDNGLPDGDKVIFEKTMSTLDNLYIFTNKGNLIYRPV

HELIETKWKETGQHLSQEIGLDNDEEIIRVFEINDLKASLNFLIATNDGYIKQVTLAD

LQPTRTYKSRAITAIKLKSKDSKVVRIDKVKPDSKQEITLITNQAYAVRFDISEIPTS

GSKAVGVKSVNLKDNDYIVNYVLADPKYVDLINIGIITQRGAFKQLKLNLINKVTRAK

RGVLVLRELKTKPHRIMAISSYGENHILHLTTSSDRHIELNTSEFPLGDRYSNGSFVL
DPMTDGTPISLTLGQPLADGRDRTEDLF"
CDS 188082..189035
/gene="cmpR"
/locus_tag="OCPHGMPK_00886"
/inference="ab initio prediction:Prodigal:2.6"
/inference="similar to AA sequence:UniProtKB:Q55459"
/codon_start=1
/transl_table=11
/product="HTH-type transcriptional activator CmpR"

/translation="MKTNTDAVLSAKSLRYFLQLIDNMSYTQAAQILGITQPALTQQI

KKLERAVGSPLFGQMGKKLYLTDAGLKMEETAKALFSTVNNAVDSIQQYTKSDTGTIS

LGVLSTIEARVIDQFLIEFNQKYPEITLHVGFYNRTELWDKLDNNQLDLAVLYMPDHN

ATVKNMKQYMAKQIYPESVVFLTHNPETKFYKLAQHKWVTYPKDYYLPQIVRRFYATE

FPNNELLTPVTFTAPYQLIKFAEQTDYDTYVSKSFYKAHKAQITLSPLEPKHETNFES
CFIYRKNKSEIPRLVNFLKEWDKFINEKDYDSRLEEVKVNI"

CDS 189155..190090
/gene="ppaC"
/locus_tag="OCPHGMPK_00887"
/EC_number="3.6.1.1"
/inference="ab initio prediction:Prodigal:2.6"
/inference="similar to AA sequence:UniProtKB:P65756"
/codon_start=1
/transl_table=11
/product="putative manganese-dependent inorganic pyrophosphatase"

/translation="MAKELIFGHQNPDTDAIGTAIAYSYLQNKLGYDTEAVALGEAND

ETKYALNKFGFTAPRVIKTASNEVDAVMLVDHNEPQQSVSDIDKVKVTHVVDHHRIMN

FNTADPLYYRAAPVGCTSTIMWQMYNEKEIEIPQDIAGIMLSAIISDTLLLKSPTTTD

QDKEAVEALANIAGVNYKEYGLKMLKAGTNIADKSEEDLIDLDAKSFELNGSNVRVAQ

INVVDLPEALERKEAFLKAMDEASKSEGYDMFMLLITNILDSDSEALVVGSDESKAKF
EKAFNTKLSDSEVKLPGVVSRKKQVVPPLTNAFEA"

CDS 190164..191957
/gene="recQ"
/locus_tag="OCPHGMPK_00888"
/EC_number="3.6.4.12"
/inference="ab initio prediction:Prodigal:2.6"
/inference="similar to AA sequence:UniProtKB:P15043"
/codon_start=1
/transl_table=11
/product="ATP-dependent DNA helicase RecQ"

/translation="MKSLQVLKDVFGYTTFRPGQEKVIDLVLKGENVLAVMPTGAGKS

MCYQIPALVNSGLTLVISPLISLMKDQIDSLKQNGINAAALNSATPQEEVNPILRQAY

EGKIKLLYVTPERLAMDYFRYQLNFLDISLVAVDEAHCISQWGHDFRPAYRQIMDGVK

SIKSNPNILALTATATPSVQKDIAEQLQIPNKNYVITSFARPNLSFKVVDNPKNTNLY

LLDYIKKHPNESGIIYASTRKHVEELTDYLAQNGILTASYHAGLSNEERSDVQDAFQF

DKVQVIVATNAFGVGIDKRNVRFVIHANSTPNLESYYQEAGRAGRDGERCEGIMIYHP

KDIRLYRWFIEQSDLADEYQKIQYQKLAMITKYVNTTECLQQFIVSYFGQRCEPCGKC

SNCLGTFIEKDITSESKSIISTIYELDGRFGKTVVADVVSGANNQRMREIDAAHLRHY

GSLKIGKSRVINLINYLISHNYLQLTDSQYPVVHVTNLGWDVLDNKKRVTQKISKKIE

KLSQTYNQIPKEENDLFVRLKEVRLDLAKKQGIPAFYIFSDKSLREMALQKPKTQAEF
LNISGVGQAKLKSYGQIMLAAIKNYLKEDAD"
CDS 192020..192607
/locus_tag="OCPHGMPK_00889"
/inference="ab initio prediction:Prodigal:2.6"
/inference="similar to AA sequence:UniProtKB:Q5XAQ1"
/codon_start=1
/transl_table=11
/product="Putative bifunctional
phosphatase/peptidyl-prolyl cis-trans isomerase"

/translation="MQYPQLDLENAKGPKALIQTNHGDIKIQLFEKEAPMTVENFVRL

AKKGYYDGTTFHRVISDFMIQGGDPKGDGTGGESIWGHPFEDEFSNKLFNVRGALSMA

NSGPNTNGSQFFIVQNKNVPKRMIKEMDAAGYPKELVKAYKQGGTPWLDGRHTVFGQV
IKGMDVVDEIAKVPRDKTNDKPKEDVIIKSIHIED"
CDS 192675..193373
/locus_tag="OCPHGMPK_00890"
/inference="ab initio prediction:Prodigal:2.6"
/codon_start=1
/transl_table=11
/product="hypothetical protein"

/translation="MGIFSDEKYRPSESRILATALTFSAGFIDAYTYIQRGHTLSAGQ

TGNVIFFASAFADHNIAGMLNRATTFIAFTLGLLLVGLFHKYVKSNYWRVFCLFPILF

ICLGVGFVPKNVPNYYVVPVIAFGLAVQNASFSKIEGMGYNNAFTTGNLKKSVVAWSA

YFFGEDKSQHNAAVNYMFLVIAFALGAIVSALLQKIFVLKTIWFAVLLLGAINIVYTI
SLNKRKKLSFSKNE"
CDS complement(194368..195303)
/locus_tag="OCPHGMPK_00891"
/inference="ab initio prediction:Prodigal:2.6"
/codon_start=1
/transl_table=11
/product="hypothetical protein"

/translation="MEVAKRNYGVDVSSYQDTNVANYSGAKFAIVKVSEGLDYRNHKA

QSQISTAKANGMLPMAYHYGRFSSNSNVAVQEGNYAVNSAKAVGLNVGSYLALDYEQG

SGNETGGDAGANTTAILAFLDTIVSAGYQPLLYSGVALLKSKIDTGKVLAKYPNCLWV

ASYPVSGATSEANFGYFPSMNGILIWQFTDNWRGLNVDGNISLIDLKTDGKPVAQPSK

PAVKQSTPQSWVDDIGDTWYKEEGKFYPNGTINIRYGARTTSDIIGTVTKGDCVKYDA
YSRHGGYVWIRQPRANGQYGYLVCRQGNDPWGRFE"
CDS complement(195316..195714)
/locus_tag="OCPHGMPK_00892"
/inference="ab initio prediction:Prodigal:2.6"
/codon_start=1

/transl_table=11
/product="hypothetical protein"

/translation="MSFSHILDLVLVVTSVTAVIVVSVYAKHKIAIDKKVAQGDLLAK

AEKIVAQSVSPLVYQAEKRGGEGEDKLTFVVQGLFLLLDMAHLPHPTMSFVKGMVEKA
VTAMKQTQSIADTVDKPKTTIVGELKEVKK"
CDS complement(195752..195928)
/locus_tag="OCPHGMPK_00893"
/inference="ab initio prediction:Prodigal:2.6"
/codon_start=1
/transl_table=11
/product="hypothetical protein"

/translation="MQDLSNLILAIAYLIGTIATLYQVKAKHDAPPDETDMSVDNLKK
EIKSLQDKLKDKQK"
CDS complement(195943..196344)
/locus_tag="OCPHGMPK_00894"
/inference="ab initio prediction:Prodigal:2.6"
/codon_start=1
/transl_table=11
/product="hypothetical protein"

/translation="MRENYQKIRSDPFKLVLTVLQTLISLWIAGIGIFLYNDQHYFFW

PPDWSNIENDVRIDTFIVLVGLVLFLCTIFRVKNRKIIATLLVCCGAISFSIATLSLL
HVIMSNYWVMGLNVIGETILFILILLVAHYL"
CDS complement(196501..196932)
/locus_tag="OCPHGMPK_00895"
/inference="ab initio prediction:Prodigal:2.6"
/codon_start=1
/transl_table=11
/product="hypothetical protein"

/translation="MADIKFMNLSENGNPATTDSVLIGNSQDGLKRTTLGTVGNMFAV

HGALHFEEVSVLTSTKASEVTDPKVAFSIGYDITAPAVPGYTFKCWIGSQSNGFTCGN
YVINKTNSAAQVWVAAVIGSILTDSNNSVTATALYVKNELA"
CDS complement(196947..199463)
/locus_tag="OCPHGMPK_00896"
/inference="ab initio prediction:Prodigal:2.6"
/codon_start=1
/transl_table=11
/product="hypothetical protein"

/translation="MNNIPTINNNGQPYYFPADIAKEGEGYVRLSNFFKVRVNDNGKV

LPFKWYDQGRVMNVHGFIPFIQGAVGKHYEDPDTNEIIMAPDALYREWQGSMENAHDG

GVMDYILEDQMFPQEGIFKGHFGLKDGNGNVLTSVNIVFEVLGNDLRIGNTYKYYSSQ

LDSLEREYQVKTDQMVADGTQKVNQLIVTTKNNIDTSLQTSRETLDALNGEIKANRAE

QANISQHLAGTQQQIANYDIVTRPEFQTGMDTMNSAINERLSQMKTNPIAVANAGELT

TNYPNGADGIFITADTGHKWVYLYGAWKDCGAYQAIGIDNSEIAPLKEQILKQEGKIE

QNTNDIGLNSLGIKKNSVDIQNLEGAGHLMDILLVDDFGNHITDDYGNRIGGYKWLPL

TDVTLTQAGLPADGQAVGEAIKNATSFKSEKYGMPVLYLWGSNILSLKDKSKTLKNEV

TYSFPAYGVSGTVEKFKVQGASSVGQPKKNYTLNLDKSFEAFRGYGKNHKYVIKANYT

EPSQALNVVGARLWGSIRATHRTADTGILNTNGDQLVDDKGNRIIAETDPQLSIGGTY

GAVDGFPIGVYINGQYWGIYTFNIPKDDWMAKMPKKSENKYAIIDTIWTPQGAFLKET

NLKDDQMELQFCSTKDTTWVKDSVNELIRAVLANYDTADDFNKAVSPLLDIDSAIDYY

IFSVLVDNDDGIFRNYLLQTFDGKKWYFAAYDLDSIFGRTPDFLEHMPAKSDTNDWRD

HGVTFENVTNDNRLMYQLWKFYKDEILKRTKALVDGVMSDSAVDTAFVDFVRHIPLKA
FDAELDVWPYTPNTSVDNVNRIGRWYMQRVAWLKNRYFNN"
CDS complement(199463..199792)
/locus_tag="OCPHGMPK_00897"
/inference="ab initio prediction:Prodigal:2.6"
/codon_start=1
/transl_table=11
/product="hypothetical protein"

/translation="MADVFEKEGSPTQDAAYREHLDRNWEAGNKKFAELESKVKNTEE

KCQDIIGDQDSNDVNKKKKEERIKALETQVAKLSLAVFGDGATEIQTISPNQENHSQI
AEEVKLD"
CDS complement(199842..201176)
/locus_tag="OCPHGMPK_00898"
/inference="ab initio prediction:Prodigal:2.6"
/codon_start=1
/transl_table=11
/product="hypothetical protein"

/translation="MISIKTAAGKVAIVKGQDVQVTSTLGSLDTLSFNFYNLHTNKVD

GDVLAPFSIISVPEKGEDYVLQTYNTDDIGNYIQYSVSAIQIARMFHYHYVKDKIGVE

TTTTKSGDSTDTSTTSKPVKIKDALNFLFKDSGFEVVINSDVNQNSVKTFSDGLGGGY

ADELLQTAATSYGVEYYWKNKTCYVAKEIGSKDKFVFVDNVNCTKISVQEDDTAITTR

ATGIINITKQSGDNSSVNTLTSTYISPLVKEKGWPIIDATPYTEDFTDDGKSFVMSQQ

LLDDKVKKLVHDYPSVQYTVDGANFKKFAKYLHDVSIGDYGYLRTRQGIDVETRVQSI

TSYPQDPSKGNTITFGNLAFNLIDYMTYQHNQQDKYREWYTSMSLKMSNLAEIVHKAN

AGFDTFTTNWGESQQQNTQDIQKLQQELQQLLNSQKDKDTSDKGKDENDTNK"
CDS complement(201188..202009)
/locus_tag="OCPHGMPK_00899"

                     /inference="ab initio prediction:Prodigal:2.6"
                     /codon_start=1
                     /transl_table=11
                     /product="hypothetical protein"

/translation="MSSIFVKRLDGTEYDLDKLGFRVITFEPPGVNYAHTYVQQSKVG

QVLTDVVIDKMTIPLTLMIQAEDTIDLELKRLDLKRIFNSDEPFYIYTNRIPYLRWKC

VVDGAISYPQIENFWQATATINLSCPLGLAETVATTADSDFSYESGKWGLGLNIPHGV

ELKYVFNSSPCRVYNASNIDLKADELPVEITFNGNVKDALTITNSTTNQVFKLSGSYT

KQDTIVIDGIVPTINGIEQYSKTNHAYLDFSKGWNDINVDGATDYTIKFNTRFYY"
     CDS   complement(202002..207053)
                     /gene="iap"
                     /locus_tag="OCPHGMPK_00900"
                     /EC_number="3.4.-.-"
                     /inference="ab initio prediction:Prodigal:2.6"
                     /inference="similar to AA sequence:UniProtKB:P21171"
                     /codon_start=1
                     /transl_table=11
                     /product="putative endopeptidase p60"

/translation="MADGRITIDIDIPVDKVKTDAQLIDQILNSVGRDAGKELDSSFE

ESTDKVKQKADETSKDVDEKLSKPVDIKANLDNKDVQEKTNQTKRNLDSVPKETKTEQ

KADNKDAVEKARQAKEEIDKVPDRKNSKLDSTDNTKKATDSASRNADNAGKHFSKLHE

IIKGTFIGNFAANAAQTALGTIKNAIGGVITEGTHYNRLQQDMLAQWNTLTGSAGKGK

ELVKETNDLAIAAQNSVEMVNDLNQKFYAVTNSSSKTRDLSKAVLTLQDAFGQSDDAV

KNFAMQWSQMIGNGKANAQDMMSIQNVFPKFMEELVEYERKVTHNSKLTTAQVRDMMS

NGKISADAMNTVLIGMGKKYKNATDNFSQTMDGMERTIHARIPVLAGAIVKPFQDLKN

PLLGKMSNWITSSGAEKSFENFGKSIAGIMNGVMTVINTFAMSFRANIAGAFQGSHLS

DIGNSFRDIGKAVTPALQAIAGFVGVISANVFNVFANLLSGIVEGFRNIGKQRSSLDF

SGVAKTFQSLSQSINAVMVRLRPLIKEFGEFIGIFAKGTFEGIVTVFQDISNAIGKVT

SKVAEMLPYMDSTGKAVDGVTKHRASIERLGKVFGGLIAAIVAGKTTFKVLDTMRLGI

VGIGNTISTLRKAPTIIAGISKAFPVLGKAINGVKGAFDLLKLAFLANPFMATVAVIA

ALGLAFYEAYKHIKPFREWVNKTADTVHKSFDGMVRNVQAFTKAFVKDWDDTKKNTGK

FFSDLGKNISDSWNSISKTTSKFFTDLPKNISKGMKAAIDWIKKNWSGLTLLMVAPIA

GAIKLLYDNNPKFKKWVDSLGQNLKKGFDGMLKNSHNFFKGLWTGIGNWGKQVSKNWG

NFVKGLSENRYVKAFKKGNLFGTLFKDAQSRMKDFSKKWDKAWKNNKKALADSFRNMQ

RNIGKWGTNTHKWYDKFSEQFKKKWDNGWKNSKQALIDSFDRMKRNTSNWGNNIHKWY

DNFNKNFSKNWNRGWSDTRKNLGTAWSKMQDRTSRFGSDMKNWLDNFGPNFKAGWKSL

SKGVQNIFGDMWSAMKKLGKDAMGGLIDIVNAGISGINTVIYAFGGKGNTIKKIPKKF

ASGTGAFSGPRRAITEPTLAMVNDGFDSPETGNKEALFRPATGEFGVFQGRNTTTMLM

PGDEILNASETAMIMQGMGITHFAKGTGFMGWLGGIGSWIGNTAGHLKDLFNMATKII

AHPIDYVKDIFNFKGFNSGKRSMKELASGLFDQANKNVQAFWSTLWSMVSGKLDGGGA

EGGLLGAVEKYGKGKPYVWGASGPDAFDCSGLVMYALQKAFGKSFPHYSGAQYNATTP

VSNPQPGDLVFFGPGGSDHVGVYAGGGKYWSAMSPSSNPNIGMSAVKDGPSSNISYRR

VPGLKGEGSSSNAKANNGLEAFIKKMPGLKGFWSMIDKLNGLFGIGADSADPSGFGVS

RWGETIKQVAAEMHADVTGTDIAKILSMIKGESGGNPKAVQPGSDPDGDGSGPAIGLL

QYKRGTYKAYTMGGSIMGGTNQLRALFNDSNWKNDIHFGAGWSPTGHRRFSGSWHGNG

GEFDKPEVIGVGEDGPEFVINPQKSTADHLIDKAILQRAKVAPESPTASLARIMEQVK

YSSVAGYGTPDSNTVAGQNIVKVNNQGQAIDGNTVIKFIVSDKEMARATYPTIKMLQA
HDITIKQQGGAVPIV"
CDS complement(207057..207674)
/locus_tag="OCPHGMPK_00901"
/inference="ab initio prediction:Prodigal:2.6"
/codon_start=1
/transl_table=11
/product="hypothetical protein"

/translation="MLSLTDTPLSAIKFDGETYQINLAFDNVIKYLELVEDDSENKEL

KALKLFFGDQEIPLDPDFIESSFKLINETITKSAYQGNSSKDWSMNIAPQHIYSYEQD

ADAIYSSFMMQYHIDLLKERGKMHWCVFRALFDGLGEDTPIQRIIELRQKNLADVPDE
QRGRVMQLQQYYSLKLKKPKTEEDVFNSSSLSSAFASLMNTAKGG"
CDS complement(207679..208107)
/locus_tag="OCPHGMPK_00902"
/inference="ab initio prediction:Prodigal:2.6"
/codon_start=1
/transl_table=11
/product="hypothetical protein"

/translation="MSIIDLDKRIKVDNKVDVKLAGKTYKILFDDNFQKTVAKASVEV

MNGLKALDDPSWADKDMAVQKKDVENSFNSVKASAISALDKLLGNGEGKRLYKYYNYS
TDALGAVLNALNDEAVKSVEVKEKKLKKLKHLATPTSVRG"
CDS complement(208143..208721)

```
                     /locus_tag="OCPHGMPK_00903"
                     /inference="ab initio prediction:Prodigal:2.6"
                     /codon_start=1
                     /transl_table=11
                     /product="hypothetical protein"

/translation="MATNLVQIKGTEIPTDGAALNVANRLYIDITDNDNDLSDITTGK

WAWLARGISEITPSWQEKTQKTAYYDGDGHDDTEVTGKSMQLAVKGVRYLGDPAQDYI

DGKQYAIGSAAKTRVLWINNGMPVVSACTLTAVTPTGGAADAQQNFSLTIAFNGAPKT
                     TTGKLTMTESDQSRVFTASVDDKTPATTPNAH"
     CDS             complement(208734..209135)
                     /locus_tag="OCPHGMPK_00904"
                     /inference="ab initio prediction:Prodigal:2.6"
                     /codon_start=1
                     /transl_table=11
                     /product="hypothetical protein"

/translation="MSDIKFDLQEALAQSIIKGTELPIKIAYLAPDNSIGLVPEQGSH

KLSTDFSGREYWVYNYAITERGKSAREIKDDLFKISLFLDDLQPGAIESDSSNFVFDK
                     IDVSSAPSETEQDMQGTVTYLLDVAVFVYTK"
     CDS             complement(209128..209499)
                     /locus_tag="OCPHGMPK_00905"
                     /inference="ab initio prediction:Prodigal:2.6"
                     /codon_start=1
                     /transl_table=11
                     /product="hypothetical protein"

/translation="MGIKVRTNLNIMNHKFSEDQLRRGRLAIANDAQQAMEKYVPKKS

GDLRDHAKVATDGSSIYYVAPYARAQFYGFITNQYGGPFRIHNYTTPGTSRRWDLRLK
                     GNLSEMALVKEAFINGVKWHE"
     CDS             complement(209509..209859)
                     /locus_tag="OCPHGMPK_00906"
                     /inference="ab initio prediction:Prodigal:2.6"
                     /codon_start=1
                     /transl_table=11
                     /product="hypothetical protein"

/translation="MLKPPKSMCNQSIIIKRKFEDDLYNEATYDDGVEIQNCVVHLRT

IYSGTNNDRQIVANGTVVLYQGISEPFISLSKQDIENKAKIIYEGQEYTLTNINEDYE
                     PFSNKLYQYKLTMI"
     CDS             complement(209853..210242)
                     /locus_tag="OCPHGMPK_00907"
                     /inference="ab initio prediction:Prodigal:2.6"
                     /codon_start=1
                     /transl_table=11
                     /product="hypothetical protein"

/translation="MALLNKDEFQKLTGIVPDADFDKLEKAAESMINPLTGMYYELHS
```

IDEDTDINRVNWFKKALALQIQYMSDIGASSTYEMAQKDIKSVSIDGTSVSTGTSPTD
SATNGVYNLALEYLFYTGLLYRGISSC"
CDS complement(210265..211317)
/locus_tag="OCPHGMPK_00908"
/inference="ab initio prediction:Prodigal:2.6"
/codon_start=1
/transl_table=11
/product="hypothetical protein"

/translation="MAETHLSDMIIPTVFGNWVQNLSTKTNNLVNSGILTPDSDLGNK

LTQPGTKITIPYINDLDGTPDNWTDEQDINVSNLTSGSQIGMKFYQTKAYGETEISRL

LSGAPIQEQIASRFANFWNTSDEQMLLAVLEGAFQVDDVANAKILDLTSKSPTNAEFS

AKGFIAALGLMGDQPENMLTGISVNSATYAMMKAQNLIDTIQPSNGGTPINTYNGKQV

VIDDSIPVEKSGSNSTSVAYLFGTGAVRYSTLLNSTQVVDEPLKQGGRESVVQKRVGC

IHPAGISISPTFVPNKPNFPTPDDFKKKEAWSLPKDMDVRNVRLVEYKFQIDPLFVPA
DTSKQQNAASAPSRNS"
CDS complement(211324..211950)
/locus_tag="OCPHGMPK_00909"
/inference="ab initio prediction:Prodigal:2.6"
/codon_start=1
/transl_table=11
/product="hypothetical protein"

/translation="MERDFLEKQGLNAEQIKAVMAQAGKETNALRDDYDRKLASLNDQ

VDGYKSQISDRDKQIKALGSQAKDNEELKAKVAEFEKANKEKDKEWSSKLASQKKEFA

ISTALSKAGALENKAVLPFIDTDKVSLDEKGNLIGFQEQVDAAKQDYGFLFKQDKPKE
EPKPATHVVVSGNGTSEVPKDPANMSLDQQTEMFKKDPQGWANLFRKK"
CDS complement(212693..212869)
/locus_tag="OCPHGMPK_00910"
/inference="ab initio prediction:Prodigal:2.6"
/codon_start=1
/transl_table=11
/product="hypothetical protein"

/translation="MNKTIGIYIIINKATGKVYIGQSTDIHQRFIDHFKKSAINQRPY
NLHKDIAKYGIQNI"
CDS complement(213030..213887)
/locus_tag="OCPHGMPK_00911"
/inference="ab initio prediction:Prodigal:2.6"
/codon_start=1
/transl_table=11
/product="hypothetical protein"

/translation="MDPELNKMMKQASKIVDYYDYLQQYTFYLLIDAFKKHKGMLTRV

DDKSILEWRSKALAEMGGLTDKVVDFIAKNIGYSKQAIYDLIQGQGLTVAKRMNSELS

TALKQPMRGVSDDTVAIINAYADQTFRNVNNYVNQTLLTTNVQKNSALKTYQQIVDKT

VLDVSTGNKTADRALKDNILQWYDKGLPTALTDRGGHEWTLEGYTRTVITSATHRVFN

EARAQSMKEFGSVLATMSSHPAARPACAPIQGKVVCIVPKSDPKADLSYPNIYDYGYG
KPAGTQGRR"
CDS complement(213838..215469)
/locus_tag="OCPHGMPK_00912"
/inference="ab initio prediction:Prodigal:2.6"
/codon_start=1
/transl_table=11
/product="hypothetical protein"

/translation="MGLWASIKSLFRKGGAKLGMIKSLGAITDDPRIAVPAEEYTRIR

RAKDYYSDKPVDVKYWVLGNERKRKMNTVNMTQKASKRLASIIFNEQCSIKVNDNDLQ

KQLDEIFRESRFYTTFETNLQRAIALGSSAIRPYVEDDKIKLNWSDALSVYPLTANTT

EVREIALARKIVKTVNDEPHYYTLLEFHQWGNKQADENGHEYTPYTITNELYESTDES

ETGTQVPLTSIEEYADLPQQATFTHITKPLFAFYRNPGDNNKSFTSPLGLGLCDNCRN

ILDDINVTQDGFYWDVKTGRRRVTIPQSWVRRQTQINGNPIPENQQMYWDTDDDVFVP

INARIDDSSAFKDLTINIRTDQYQAAMSYFLHEFENEIGLSEGTFTATPTGIQTATGV

VSSNSMTYQTRSSYLTQIEDTIDQLVYAIAELLQTPELWSDQQPKWTGDLDSLVITPD

FNDGIFVDQDAQFKNDLSALNAGAMPVKEFVKRNYNLSDDEAENWAEQLQKEKATPAP
DFEQFNPLAPDTKNKGTDKHGSGTKQDDETGKQDS"
CDS complement(215482..216786)
/locus_tag="OCPHGMPK_00913"
/inference="ab initio prediction:Prodigal:2.6"
/codon_start=1
/transl_table=11
/product="hypothetical protein"

/translation="MAIVKLSQEINPHFYTAWNSNKPYQVYKGGRGSFKSSVISFKLV

TTMMKYIAQGKTVNIICVRENQRYLRDSVYNQILWAMNKLHVESEFRTRVSPLTITHI

RTGSTFYFYGANDPMKLKSNIVGNVIAVWFEEFANLKSVDVFDQSVPTFIRQKPDFAE

QVKVYISYNPPRNPYAWVNEWITQRQTDPDYFIDTSTYLDDKLGFTTKQQLDLIETYK

RNDPDYYRWLYLGEAVGLGTNVYNMNLFKVVDKIPDDEYITEIFYGMDTGFMVSATAC

VACALTNKYNVYVLDTFYYDPTKYARKLSASEQAERVHDFINEITNKYGVLPYNQTID

SADGGIYTQYWQMYNTQWSKVHKLSEAAMIDRVEDLLAQGRLYVLKTPGNEIFLQEHQ
KYQWDPATVNSDNPRVIKEDDHSCDAIKYAIVDNEQLLGLAA"
CDS complement(216755..217420)

/locus_tag="OCPHGMPK_00914"
/inference="ab initio prediction:Prodigal:2.6"
/codon_start=1
/transl_table=11
/product="hypothetical protein"

/translation="MAHAEYKKWLEPDNLTKLRSWARDGLTNEQIAKKIGVKRQTFQR

WLSTYSDMSDALKKGKEIVDAEIEDSFISVMKKHTLTTTQYKMVKKDEFNLKAERTKF

ANIYKLDHPQATREEIAIATAENVDVYEKIPISKTVTEVDPNASAIIFWLKNRRPDIY

RDQTFQKLNEANARKTLAEAQLSEAQLKALEENDDPSNKTIIVDDIREIDSNGNSKTK
PGD"
CDS complement(217911..218096)
/locus_tag="OCPHGMPK_00915"
/inference="ab initio prediction:Prodigal:2.6"
/codon_start=1
/transl_table=11
/product="hypothetical protein"

/translation="MKFLAIVLTIIFAVARIMNLITWSWWLVLSPFLIYYGGILLILL
VGIICGLIWNLVDKFRS"
tRNA complement(218323..218398)
/locus_tag="OCPHGMPK_00916"
/product="tRNA-Phe"
/inference="COORDINATES:profile:Aragorn:1.2"
/note="tRNA-Phe(gaa)"
ORIGIN
1 gtagggtatt aatatattta ttaaggatta ttttcttgat agtcatttct
atagtgatat
61 cacatttaga tttagaaaaa atttatagtg taagtataat ttattggatt
tattttattt
121 ctattattat cgccataata cttacagagc cctgttttaa attgagatta
tatgtacccg
181 ataatgacga agagaagcaa gttttgagac caaattttaa taaaccaatt
gaactttata
241 tttttgctaa ccgaggtaaa aagtacagat atttaggaat aaccccatta
gaatatatta
301 aagaaagaaa agtaagttta gtaaaacata tttatgatac ttcttactgt
aaaaagcagg
361 taattgattt ttgtaaggtg ccttttgtta gtggcaaaaa tagattttat
ttgaatcttc
421 cgcacataaa aaaactattc aatactgaaa aaattggaat aataatagaa
gaaaggacaa
481 ataaagagaa aaaaacactt tattattatc gcttaggaaa aagaaattta
gattgtataa
541 acaagcatcc tattttcttt aatatgatag ctattcctgt gttacttgct
aatggattag
601 gagcgattgt gtatggcgta tgtttatatt ttcataaata tattaaattt
ttggtaaata
661 gcagtgtatt gataaaatta actcaaaaat tcggtgatgt aaaatataaa
gttgagttaa

721 ctaataaaat aagtatgggt tttcttatag tgtatttagt tatgattttt tggatattta
781 ccttaccttt ttggactgag aaatttgatg aatttgtaaa aaaacttgga attgtgataa
841 cattatttct agattttgca gcatgtatta tcattctaat ttttcttgaa ggaagaggtc
901 taaacgatca ttttagtttc ttttgtttaa tagtttggat aatctttaat attattgaag
961 tgattatttt gatatataaa tggatgttag aagataaaaa tacaggagga ataccgcgta
1021 ctcagttgat tatgacgatc atatttggaa ttttatcctt tattgtagga aaattgtggc
1081 gataaatcag ttcaactatt actacatgta aattttccat gtagggtcaa tcacaacaag
1141 atgctttact ttatttttga gtgtagcaaa tataggatga attatttcag aaaaaaggta
1201 cgacaaataa acctgtaaaa catgtcgctt ttacaagctt aggtctataa taaagttgat
1261 caattaatta cgaccaaagt ctttttgatt atgtgtggct acgttgtaat catatggttg
1321 atcattggta atcagagcat aaatagttct gattaattta tgaattgaag caatagctat
1381 ctttttaaac cctttggttt gagaggatag ctttttgctt tcataataat ctgctatatg
1441 acaaggatta gttttagccg cattatctat ttgtccaata gcacgataaa gcagtttacg
1501 tgctattgca ttaccgcgtt tagtaatact taaactagca tccatttcac cagattgata
1561 gcgtcccgga tcaataccga taaaagcatt gatcatatta ggattagaaa atctacggat
1621 gcttcctaat tcagctaata ctctaatagc cgtagtttgg acaaagcgag aatactttct
1681 agactttcaa gatcatgatt gggtaaagat ttagctagtt taaccattct agaaattaaa
1741 tttttctttt gttcgtttaa gttaattagt ctttgagcat aatattgcac ctgctcgact
1801 tctacgctgt tttcagaaac aacaggataa gccttgttag acagatcttt caacttattt
1861 gaagtctttt gcgcatgctt tttcggaatg cctttaagct gcataagcca tttaataata
1921 tccttttctt gagtttatct aactaattca caatgcgggt aatgactgat tattttccaa
1981 taattaaagc ctttatctgc actaaataag ctttctattt cagaaaaagt agattgcaaa
2041 gattggtgga acctatttat gtctataata attagaatta tgataacatt cattaataag
2101 attagattaa gctacagtct cctaagagaa gtattacaca gaacttttta ttttcttaac
2161 taaagtttac tagcattggt agtaatgaaa cgaacgttag aaaaatagtg accatcatgt
2221 ttaagattag tagaatagca ataaaaaaag agttctgaat aagtcagcta tcaagacttt
2281 ctgagacttg tcatcaattt tttcagaata atcagaaaaa atgatactag tacctttaat

2341 atgatgttga attttaatac aatcattaag agaggacata agtagtcaac ttattgggat
2401 tagaaaattt aatgcagtga gacttatacc tctttttttg tataataaaa atgacaagaa
2461 tagtataagt atttgatgaa taaatcagag ttttgtgtat agggaataat atttgaattg
2521 gataaagtga tagttaaaag aaaaaatggc tattatgtta aaaatacaaa gtgactattt
2581 atatggtttg atttaaaatt gaattatatt tttttactgt taattcacaa gagtgaagta
2641 tttggtggct ataatgaaat ataaaaaaga taaaattttt tataaattta aagataaaga
2701 taaaatttat aaattggtaa catatgatga atctaaaata gactattact cattaataaa
2761 aaagtatcag agagaaggct attatcaatt aatcattaat tctagattaa tgattgattt
2821 aatcaaagat tttaagaaaa ttgatttaac tgtgtatgat attgaattac aagaagatga
2881 tgaaaccttc tatggggagt tacgtaaaac tttaaaggga cttaaaaaca aacataattc
2941 agaattatgt ttcaagtatt tgttacaaag cttaaattta ttatattgtg ataattcaat
3001 agaaattaac aaaatttctt taggaaatag tagtgatgat gtaattgtct attcaaatgg
3061 cattgtaata atgaatgaag atatagaaga aaaaataaaa gcttttttaa agaaagaatt
3121 acagttgatt attcaatgaa aaagttatat aagaatttat taaagccttt aatgattata
3181 atctttagtt tgagttgtgc acactttttc aacttgactc gtattattcc aattattcct
3241 aagaaaaatt tatcacttga tatttctata tatagtgttt tttttacgta tcttataaat
3301 ttaattgagg attattatta caaaagtaaa atatctgttg aattatattt aaatactggg
3361 aggcaatttt taaataaaaa agatgaaaat ttatcaatga atgaaaaaaa gccagcaaca
3421 atttatgcaa agattcattg tgatgatgct agaaagatcc ttaattttaa tttagaagta
3481 atattgaaag ttccagattt tgtatctgca agctgtggta aatccgttgt tcaagaaaat
3541 cgtgatttgc atatttgtaa attagactca gaagatatgg ttattcctat atctgttatg
3601 gcgaatgaaa taagtggaaa taaatctgat acaatctatt taacaattaa ggaaaataaa
3661 aataaaagaa aattaataat taaaacaaat aaaatgaaaa taggagttta atatgtcaaa
3721 tacaacaaga tggattttca tggaaaagca gaatactaaa actaatgaaa aagatcaaaa
3781 tattagtatt gaaaagatag gtgcaaaaat tgaagaagta aaaaataata cttcagtgtt
3841 agcagataaa cagaatacta tcagtaaagt tgaagtaaac actgcatttc caaaatttaa
3901 gaaagaaact tttggaagca atcaaattaa ttttaatttt tacaatatta gctactatag

3961 aacttctaat atagataata catcaacaga agataatttt tggataatat tgtatacttt
4021 aaatcaaaaa aaatatctta ttattaataa gaactcaggt gcattgacgc ttttgagaaa
4081 gatttttgga tttaagggca gaaatgaaat agcacaggta cctcatggaa ttgacagtag
4141 aataattatt tggctaatct ctatggtata taatagtagc gctgattaca ccaactcaga
4201 caaaatatta agtattgatt caattaatgg atttaaaggt aataccgaag atttattaaa
4261 tacagtttct gcagatgggt caggcataat gaatatttta agtacacttt catttttgct
4321 ggaatcatct tctttgagac aaattagatt gactctttca tttggtgagc acaaaaattt
4381 agagttaaaa atagatgtca ataatactat agaatcaaat gaagataagt atcgtggcat
4441 atttttaatt gatgaagaga gtactactga tagtgtgatt tataatcaat atttgttgat
4501 atataatgaa ataataccat taattagaca atggtttaat gaatctcaag atgatgagga
4561 agagaatcta actgaagaag attggtggaa gaagttttat aaaaactttt taattcaagt
4621 tagtgatgat ttaaaaaaca aagtagatga gaagagaaaa agactatgat tgcaattatt
4681 gttattgatc caagctctgt tttattttaa tagcttgaac gcgtttaatt acgattggat
4741 tctatttcaa atatttcatt tgatgatata tgacatacca atatgcttat tgaactatac
4801 aaagatgtaa cattgataac cttcttaact atttttatca taagaggtat aaggatagag
4861 ataatagttc aagatccttg atacttcttc aaaacttgat aaatcagcaa aattaaaact
4921 accagttgaa tatatgaatt tgaagagatg attttattat tcactaaatt ttttatctaa
4981 tttgtactct caaatagatg tttttgtgat tgttgatatt ctattcaaac atgtctagat
5041 gaacttgttc atttgtttaa ttttacagtc agccattttt aaccaagtaa aaacatataa
5101 catttcttct tttacgtaga gcgtgaagca cagttttgat gaaattattt ataagagttt
5161 tgtcattaag ctgatacata gggaaggtaa cctatatctt taatttaatt atgagaaaga
5221 tctttatgaa gaattagata aaaatacttc tactcaaaaa gctactgctt ttaataaatt
5281 ggagattaat atattttaaa cttaagaata aaaaggtatt atctatttgt agaaattagt
5341 actgtataat catagtcaca tgaagaatag attagaatgt ccccaatcgt gctaattact
5401 gtaaatagaa cgatagactg actatcttct tccggagtaa agataaaaaa cgtaattatt
5461 tttaataaaa gtttactttg gcacataatg acttaattga gtgaaagtac agataagata
5521 ctgaactatt ttaagtataa ttaataaaac ttaaaagata gagctaattt tgaccaaaaa

5581 gttgataagt agtagtttat ccagtagagc ttgctcgttt caataaaagg ttaagtattg
5641 agatttaaga agataattcg tggatcctaa taggtgggaa gaatatggaa atcaaaattt
5701 ggaatagaaa tgcagaataa attagggatg tttgaattag ccctttgctc ttgaaattat
5761 gatttcattt attgagaata ttcatgaatt aagaaatgtc tgtgctcata ataaccgttt
5821 gttagatttt aggtgtaagg ctgattcaaa atatcttaat gctttacatt cgaagtatag
5881 tattgaagat aatagtagta gaagatatgc ctattcagtc tttgtaagca tgcaatgttt
5941 cttaagtcca actgaattta aaattttgag taataccatt cgaaaaagat taaatttgtt
6001 gaaaaaacac ctgaattcca tagatataca agaaatacta aatttattga aatttccaaa
6061 tgaatggttg actcaaccta gattgaaaca gtagtgggat tgaaaatgcc aaaatactat
6121 acaagcggtg aatttgcgaa aaaggctcat gtgtcgattc gtacaattcg ctattatgat
6181 caaaaaaatt tattaaaacc atcaacgcat actaccagtg gtgctaggcg ttatactgat
6241 gctgattttg caaagttgca acaaattctt ctattgaaat acttaggatt ttctctaagc
6301 gacattcgag aaatgacctt aggttcaggg gacagacaat tgctgcttga ctcactgcaa
6361 attcaaaaaa gattagtaga agagcgactt aaagaaatgc aaaatgtggc aactgctatt
6421 gattctacga gtagcacatt gaaggctggt caagaagttg actggagcaa gatgctagac
6481 ctaatccact taacttcaat gaatcaatct ttaagtacgc aatatcagaa tgcttcaaat
6541 atttctgctc gaattaggct gcatcgagat tattctctca ataaagaggg atggttccat
6601 tggctgttca agcagcttga cttaaagccg ggatgaaga ttcttgaatt gggcgctggc
6661 aatggtgcct tatggtctca gaatttagat aaactgccaa aaggtttaac aatagtttta
6721 tccgatattt cagagggaat cttagcagat gctaagaatg aaattggcga taattctgaa
6781 tttcaatatg cagtttttga tgctcaaaag attccctttg ccgataatac ttttgatctg
6841 gtgattgcta accacatgct cttttattgt gacgatattc caaagacttt aaaagaagtt
6901 cgcggggtaa tgaaaaaggg tgcatctttt gcctgtgcaa cttattcaaa gcgccatatg
6961 catgaaatca ctgatttagt tcaagaattt aatcctgaaa tagttttatc ttctactaat
7021 ctatatgatc gttttggtct cgataatggc gcagaaattc taagtaaaga ctttgctgat
7081 atttcttgcc ataaatatca agatgcaatt gagctgtctg aatctacacc agtgatttct
7141 tacattttat cctgtcatgg taatcagaat gcgattttgt tagatcatta caatgaattt

7201 aaaaactatg ttgacgaaaa agtaagtgga ggatttcaca ttactaaaga cgtgggatac
7261 tttatttgta aaaaaacata gttttttat tattaagctt gaaggtgacg taacgtaatg
7321 ttatatactt catatgaact aaaaaatatt tggaggaata atatgaaagt tattgttact
7381 ggtggtgccg gttttatcgg ttcaaacttt attttttaca tgttaaagaa acatcctgac
7441 tatgaaatta tctgtttaga tagtttgact tatgcaggta acttatctac tttgaaggat
7501 gttatggata atcctaactt caagtttgtt aagttagata ttcgtgatcg tgagggtgtt
7561 tacaagttat ttgaagaaga aaagcctgac gtagtagtta acttcgctgc tgaaagtcac
7621 gttgaccgtt ctattgaaaa tccagaaatt ttcttagaaa ctaacattat tggtacttct
7681 gttttgatgg atgcttgccg taagtacggt atcaagcgtt tccaccaagt atcaactgat
7741 gaagtatatg gtgacttacc attagatcgt ccagatcttt tcttccacga agatactcca
7801 cttcacactt caagcccata ctcatcaagt aaggctagtg cagacttatt ggttggtgca
7861 tatggtagaa ctttccactt gccagtaact atttcacgtt gttctaacaa ctatggtcca
7921 tatcaattcc ctgaaaaatt gattcctttg atgattcaaa gagccttaaa taatgaaaaa
7981 ttaccagttt acggcgatgg taagaacgtt cgtgactggt tatacgttga agatcactgc
8041 aaggcaattg atcttatctt agaaaagggt actccaggcg aagtttacaa catcggtgga
8101 cacaacgaaa tgcacaacat tgatattgtt aagcttattt gtgactactt agataagcct
8161 tactcattaa tcgaacacgt tactgaccgt aagggacatg accgtcgcta cgcaattgat
8221 cctgaaaaga ttcacaatga attaggttgg cttccagaaa ctatgtttaa agatggtatc
8281 aagaagacta ttcaatggta ccttgacaac aaagaatggt gggaaaacat catttctggt
8341 gattaccaaa actactacga cgaaatgtac ggcaagaagc aagttttaga taaggactaa
8401 ttcagcatga agggaattat tttagcaggc ggttcaggta ctcgtttata tccattgact
8461 ttagtaacca gtaaacaatt attgccagtt tacgacaagc caatgattta ttacccatta
8521 tctaccttaa tgttagctgg tattaaggat attttagtaa tttctactcc agcagatact
8581 ccacgtttta aggagttatt gggtgatggt tcacaatttg gaattaacct ttcttacaag
8641 gtacaaccaa ctccagatgg tttggctcaa gcctttactt tgggggagga tttcattaat
8701 ggtgaacctt gtgccatggt cttaggtgac aacattttct acggtaacgg ttttaccgat
8761 ttacttaaga atgctgctga agatgcacaa aagggtaagg ctacagtatt tggttactac

8821 gtaaatgatc ctgaacgttt tggtgtagtt gactttgatg aaaacggtaa
agcagtttct
8881 attgaagaaa agccagaaca accaaagagc aattacgctg taactgggct
ttacttctac
8941 ccagctggag taagtgaaaa agctgctcaa gttaagccaa gtgcacgtgg
tgaagtagaa
9001 attactagct taaacgatat gtatttacaa gataataact taggagtaca
gcttttaggt
9061 cgtggttatg catggcttga cactggtaca atgcaaagtc tagttgacgc
ttcaaactac
9121 gtaaaaatga ttgaagaacg tcaaggcgtt tcagtatctg cacctgaaga
aattgcctat
9181 gtacatggct ggattgataa ggatcaactc cttgaagcag caaagcacta
tggcaagagt
9241 ccatatggca agcacttaaa gtcagttgct gaaggtaaag ttcgttatta
atatttttaa
9301 ttaaagaaag aagcttttta aaaatgggac aaattaaagt tgaaaaaaat
gttggtggca
9361 ttgaaggttt agcagttatt acaccaactg ttcatggaga cgatcgtggc
tacttcatgg
9421 aaacttttaa ccaaagagac atgatggatg ctggctttag cattaacttt
gttcaagata
9481 accaatcaag ttcaactaag ggtgtgcttc gcggtttaca cttccaaaag
aagtacccac
9541 aaattaaatt agttcgcgca gttcgtggta gtgtatttga tgttgctgtt
gaccttcgta
9601 gtgaatctaa gacttatggt aaatggtacg gtgttgaatt aactgcagaa
aacaagaaac
9661 aattcttaat tccacaaggc tttgctcatg gtttccttgt tttaagtgat
gtagcagaat
9721 tttgttacaa agttaatgac ttctggcatc caaacgatga aggtggtatg
gcttggaacg
9781 atcctgaaat tggcattgaa tggccacacc ttaaaggtga ttacccaggt
agtccagatg
9841 cttctggtta cactcttgat gatggtacca agctcaacat ggctgaacgt
gatcaagaat
9901 ggaagggctt aaaggatact tttaagtttt aattagtgag gtaaatgaat
gaaagttttt
9961 gtaactggcg ttaatggtca acttggccat gacgtaatga atgaacttgc
taagcgtggc
10021 tatgaaggtg ttggttctga cttggcacct gaatatgctg gtgttgctga
cggaagcagc
10081 gttaccaagg caccttatgt ttctttagat attactgatg cgaaagccgt
agacaaggtt
10141 atttctgaag taaatcctga cgttattgtt cattgtgctg cttggaccgc
agttgatatg
10201 gctgaagatg acgacaaggt tgcagctgtt cgcaaagtta acgttgacgg
aactcaaaac
10261 atcgctaacg ttgcaaagaa attagatgca ccaatggttt acttgtcaac
tgactatgtc
10321 ttcgatggcc agggaactac cccttgggaa ccagacttta agggctataa
gcctttgaat
10381 gtttatggtg aaactaagct tggcggggaa gaagctgttg ctaatacttt
agacaagtat

10441 ttcattgtta gaattgcttg ggtatttggt gtaaatggct caaactttat
caagactatg
10501 cttaaggttg gttcaaatca cgatgaagtt aaggttgtta gcgatcaaat
tggtacccca
10561 acttatactt ttgacttagc tcgtcttttg gtagatatga ttgaaactga
taaatatggt
10621 tactaccacg caactaattc agaactacca acaactgaaa gtggctatga
tgaaaacggt
10681 actaagactg ggtacatttc ttggtacgat ttcactaaag aaatttatcg
tcaagctggc
10741 tacaagacaa aggttactcc tgtaactact gaagaatatg gcttatctaa
ggcagttcgt
10801 ccatttaact ctcgtttaga caagagcaag ttagaggaaa atggtttcaa
gccacttcca
10861 acctggactg atgctattag ccgttactta gataccttaa aagatgacgg
tttcttcgac
10921 gaattgaaga agtagacttt tttaattgaa taataccgaa ttgaaatgga
actaagtgtt
10981 ttgaggctta gttctttttt tgcgttggtg cagtgtataa tgaatttata
cttatattag
11041 aaaaaattag tgaaaaagta tcagtaatag ttacaggtta taatgataaa
aaatacttaa
11101 atcagtgtgt tggtagcatt ttgaattaaa attattcaaa cattgaattg
attttgatag
11161 atgatggatc acaatcaatt cacctaaaat ttgtgaagag tacagacaaa
aatatgatca
11221 ggttagggtt cttcataaga aaaatggcgg cgttgaatct agtagaaatg
atgggttagg
11281 ctatggcaag tggtgattat gtctcgttta ttgataataa taattggctg
ccagaaactc
11341 atattgaaga tttgtgtaat ctcttggaaa aaatgctgat atttcggtta
gcaattttaa
11401 cggattcgat gaagaaaaat ctgcattttt atattggatg aacgagaatg
attattttga
11461 aaagtgctat tcatcaaaag attggtttaa gctggaatat agaaccgctt
attacaacat
11521 atcaatggtt ttcgtagttc catggaaaag ataaatcaag atcttgttta
tccaattaat
11581 gcaatagttg aagatgattt aaccacctgg aaaatttatt tactcgctga
caaaattgca
11641 tatatgaata aaagcatcta catgcatcgt atcttgaacg ccagtatgac
agcgcaagtc
11701 gataagtcct ccgtttttcc agcagaagca gttgaacagc gagtcaattt
tttgaagtca
11761 atcggggttt tgatgtaact gaagaaaaaa cctacgaaaa gcgcttaact
ctcttgcaat
11821 ttgatgatga ctattttaag tcccgagatg ccaggcaaaa attagctatt
ttgaagaagt
11881 atcgagggga gtagaggcgg tgaagcgttt atgctattta ttttgtattg
ttattaaatg
11941 aaaaaggatt tcatctaggg acacataggt cataaaagat gttatgatat
aatatttgtt
12001 agaacatatg ttttacacaa gtgagggagt atctctatga aaaaagatga
tataaaggat

12061 attttagact ctaacaaaat aaatagcttt tcttcatatt ttaattattc tcaattggct
12121 gatttaaata aaaatttagt tgagtcatta tcgccagctt attctattac ctcaaaagta
12181 gcttccttaa ataagagata tgcagctata agttctgcgt atttggaaag accaacaaac
12241 acaatgagtg ttaacgctag taaatatatt aaaaatttgc ataaagattt agctctaggg
12301 gtattaccca acattaaaaa ttttaataca gatgtttggt acaaaggcgt aattgctagt
12361 tttgaacgga taaaaaaact cacaaatgtc tttgaaagac aatttaaagt tttaaactat
12421 tttccacata taatgcaatt ttctcctaaa gatgtctcta atttaattat acctaaggaa
12481 gaaattgcac ttagtcttgc tgataaccat tggataatgc tgttacgtga tagtaagata
12541 gataaagaat tacttaacga atgtaaggag tccgaaaaca ataattttaa tgaaattgta
12601 caaaacttct attcaagaaa taatcataag aatgttttta gaaagataga ttatcttatt
12661 ggtgctgatt atagtaagaa ggaagtaaaa ttagtagacg gatttcatga tcaaataaag
12721 agagtgagaa aaatactatt tcgagatttt agtgattctg aagtattgat aagcacagtt
12781 atgacttgga ttgagtatgc agttgctgaa aaatttgggg tattagaaga taaaaatggt
12841 tataaattag gtggaagaat tgaagaattt gaagaaaaaa ttgaaaaact agaactaaaa
12901 gaaataacgc tactcgtggt tgattactat aatatttttt tctacttatc tgatttgtgg
12961 aattcaagat tacaagattt tgaagatggc aaagatcagg ttggaatagg aagacattct
13021 attcagcatg cacgcgttaa tccaaaacgc tatactgatg aagtaatgtc aaaattaata
13081 tgtttacttt atgcactggt aaagcttcca gatcttgatg agttcgaaag ttttttgcaa
13141 gtgtaattta attatttatt ttgtttctca cattaaaaat attagtatag gaaaaaacag
13201 aacgataact ttccataaat agtttgtcgt tctgttttta ttttgtacag tttggataat
13261 accctccaaa ctaattttca cccctacggt gcaaaagtgt attttaaatg taagcgcttg
13321 tttaatatta tataagtgtc aagagaaata aaaataaagt attaatggag gatttaaaaa
13381 tgactaagaa tgttttatta gtatgtggtt caggtgcatc atcaggtttt atggctgcca
13441 acatgagaaa agcagcaaag aaagctggtt tagattacaa gattcaggct cggagtgaag
13501 cagaattagg tgactatgca gatcagattg atgctttaat ggtaggacct cacttaaaag
13561 cagaatttga tgcaatcaag gcacaagtgc ctgataaggt aaaagtaatt ttaatgaagc
13621 cagattatta ttcaatatta gatggcaaag cagcagttga agacttacaa agtcagctaa

13681 gttaaattca taagaggtaa ataaaatgaa cggttttatg aattttctaa atactaaatt
13741 tgcacccaga gcgcagaaga tagctaacaa cggttggatt gtaaccataa aaaattctat
13801 tttggaagta cttccattta tttttgttgg ttcgttcatt actttattaa atattccact
13861 taatttttgg aagtggtggc caaatttagc accaattagt agctttacat ttggtttagt
13921 ttcaattttt attgcttttc tatttccatt taatttcatg gagcatatga aattaaacaa
13981 gcaaagaatc atagcaggac tgtcatcagt cgctttattc ttaatgctcg ctaatccaac
14041 ttgggataag agcggatcaa ttatttctta ccaattttcc gaactcggtg ctggtggtat
14101 gtttgttgcc ttagttgtag gtatttatac tgctctaatt atggaagcat tcggtaaatt
14161 ttcatttttt agcgatgaca cgtcgatgcc agactttgtt attgcatggt ttgattctat
14221 gctaccaatc acagtagtaa tcgcaacagg ttggttattg tcagatatct tacactttaa
14281 cttttatcaa ttaattgtaa atattttttc accattagct acatcgctag atacttggtg
14341 gggatttacc ttattcctgt tcttccaatg ttttatttat tcaatgggaa tttcaggttg
14401 ggttttagca gctattgatc aacctgtaat gcttgcagga attgcagcta atgcacgaga
14461 agttgctgct ggccatgccg caactcacat cttcactagc gagttagttt atagttttcc
14521 atggattggt ggggttggtt gtactttacc attggtttta ctaatgctat ttgcaactaa
14581 atctaagaga atgaaggccc tgggtaaagc atgtatttta ccttcaatct ttaacattaa
14641 tgaaccagtt gtatttggta ccgtagtttg gaacccatat ttaatgattc cgctttggat
14701 taacggaatc gttttaccat taattacttc attatggttt aaagcgggtt tgtctccaat
14761 tcctcactct ttaatgcaaa tgtggtacat tccatttcca tttgtgactt ggattgttag
14821 tccagctatt agttcactaa ttttattatt aatcttattt gttgtaggtg gcctaatttg
14881 gtatccattc ttgaaggttt atgacaatag tttaattaaa gaagagaaag catagggaga
14941 gtttaagatg aatgaaaatg acaaatctgt taaagacacc atggagataa ttataaatgc
15001 tggcgacgct agagaattaa tctctgaagc tttagacaat gttgcagatt ttgattataa
15061 ggctgcagaa ggaaatatgg aaaaagcaaa ggaaaaacta gtaatagctc accgtttaca
15121 aacagctaaa attcagcaag aagcagaagg caagaaagtt gagtattcag ttctgttcac
15181 tcatgcacaa gatactttaa tgactattaa tagtgaatac aagttaacag ctcatttaat
15241 caaggtcttt aaaaagcgtg atgaaaagga gaaacataat gattaagatg cggaaaaaca

15301 agtatccata tttttctaaa aattttcttt ggggtggagc acaagcagct agtcaagctg
15361 atggtgctta tcaagaagat ggtaaaggct taaattcatc tgatgtccaa ccttacttaa
15421 aaggtttatc aaatgctgaa attcaaaaaa ttgaaacaga aggaatgaca gttgaacaag
15481 taaagaagaa ttccgaagat acaactgatt attatccaaa gagatttgga attgattttt
15541 atcatactta caaagaagac ttaaaacttc ttgcggatgc aggttttaaa acttttagaa
15601 catctctaga ttggtcaaga atttttccaa atggggatga tgaaaaacca aatgaagctg
15661 ctttaaagca ttacgatgaa atgattgatt acatgataaa gttaggtatt gaaccaatta
15721 ttactatgaa tcactatgaa acgcctatta atattacttt gaaatatggt ggttggccaa
15781 ataaagcagt tattccgatg tttgaaaaat ttggtaaagt tttactcgat cgttttggcg
15841 atagggtaaa gtactggatt gtagttaatc aaatcaatat ggtccagatt gaaccatggc
15901 tttctgtcgg agtagctagc gatcagtacg ataatatgca agaagcattg tatcaagcta
15961 tgcataatca atttgtagca gctgcagcta ttcaaaaata tgcaaaatca ttacataatc
16021 ctaatcttca tattggaacg atggtagctg atggaactgt atatccagca agtagcaagc
16081 cagacgatat agttttggct atgcagcaaa atcgtatgca atattttgtt acagatgttc
16141 aatttagagg agagtatccc aagtttgcat tgaattattt tgctgataat gatattcatc
16201 ttgaaactac tgaagaagag ttaaagctaa tcaaagataa tccgatggat tttttagcta
16261 tttcttatta ctattctaag atggtcgatt ctacgaagaa tgaaatgcaa ccggcagaca
16321 ctagtgacaa tcctaaccta aagcaaagcg attggggatg ggcagtagat ccacaaggat
16381 tatacaattc actttctcaa tattatgatc gctatcaaaa accaattatt attgctgaaa
16441 atggatttgg ggcatatgac aaattagaag ataatcatga agttcatgat cagtatcgtt
16501 ccgattattt aagagcgcat attgaacaag tagggcgcgc tattcacgat ggagctgatg
16561 taattgctta ttgtgcttgg ggaccaattg atattgtctc ttgttcttca caacaaatgt
16621 ctaagaggta tggctttatt tatgttgact tagatgatga aggaaaaggt agcggtaaac
16681 gttatttgaa ggatagttat aattggtata aaaatgtaat ttcttctaat ggcgccgaga
16741 tctagttaga aagtgggatg gttgtcaatg agtaagagag ataacttatt aaaatactta
16801 cagaaaaatc caagttggca tacatctcaa gaattagcca tcgaaattgg tgtttctagt
16861 cgaacaatta aaaactacat taatttttgg aaagcagaaa aatatccaat agaatctggc

16921 attaaaggtt ataagttaaa atataaaaaa gtttccgaaa atgaaaagac
tgtagtttct
16981 actcagagta gttctaagga gagaattaac tggattatta attatttcat
tacaaaagat
17041 tcagtaatta atttttatga tcttgcaagc aaattgtatg ttagtgaaac
tacaatattg
17101 caagatttaa cgaaggtaaa aaataaggta gcctcttttg gaatgaagct
agagagacat
17161 ggtgataact ttgagttgat cggggacgag agaagaaaga gaagtttgtt
gagtgcatta
17221 atatatgaag aaacttctgg aacctttatg aatcaagaag ttattcaaaa
gaattttgag
17281 gatattgatg ttagcttgct tcgaaaaata atcttagagt catttaatca
acgaaatatt
17341 tatcttaata cttttgattt gaataatatt cttttacatt ttgtgattgt
aattgaaaga
17401 ttaaaacatg gacatgtaat atcaaagact aactttgaaa aagattctaa
cattaacaat
17461 aaaaatgtac tcgcatacaa aattgtgcaa tcagttgaaa atgcactttc
aataaagttt
17521 gaattaccag attttaaaga attagaatta attgttgagg catctgttat
tgacaaaagt
17581 agcaatctac ataataaaat taagccagaa acagcaaata tggtaacaga
tttgattagc
17641 tatgtttgga gcaattatca tattaattta gataatgata attttaagga
tcgatttgcc
17701 cttcatttag atcgtttaat tgcaagagcc cgtcaggaaa agattcagca
taatcccatt
17761 gcgactaata tcaaaaattc gtcgcctact atttatgaat gtgcagtaat
tatagcagac
17821 agaatatctc aaaagctgaa tataactatt gatgataacg aaatcggata
tattgcgatg
17881 catatcggaa atgcaatggc agaacaaatt tctgataaac aaaaattagc
tgcggttata
17941 ctaatccccc agtattatga caatgcagct gtgttaacaa gaaagattca
aaaacatttt
18001 gctcaatata tcaatattaa agatgaaatt tatgatccta ttcaattgca
tgcaaggaaa
18061 ctaaaaatag atctcattat cgcagttgat acaaattatg ttgacgaaaa
aataagtacg
18121 gttaatattt cacaattttt cttaaaaaat gatattcaga aattaacgat
tgcaattgct
18181 actaagcagc atcagcttag aaaggatatc tttcaagaaa atttgtttaa
gttttttaat
18241 agtgaagatt ttgaaaaaag tgatgaaatt actaatatag atgattgttt
cgatttagtt
18301 ggttctagat tttctaaaga acgagtcgtt gataagaatt ttaagcaggc
gttgtataat
18361 cgtgaaaaca tgtcgagtac agcatttggt agagttgcaa ttccgcattc
gttaacaatg
18421 tcggctaaca agagtcgtgg ctttattatt attaatccgc gtggtattaa
gtggagtaat
18481 aacaatgaag tatatttagt tattgccctc gcgattgatc ctgataataa
gaagttgttt

18541 agggatgtat ttgatgaatt atcaaacatt gtgacagata ttaataatgt gagccaatta
18601 gttcattgta atagctacag tgagtttatt attaaattag tcgatttatt gtaatattta
18661 ataatttgaa aaatctatca gtttagcaaa gttaaacgct atgctgatag atttttatat
18721 taatatgaat aatatattta tataaaatat tagttttcaa attgaataat tgatttaaaa
18781 aatttatagt aagcatagga ttagaagtta tttaatagat ataatttaaa aggaggttga
18841 ccggacaatg agtatttata gaaatgacga tattttaatt acagacttaa tggatagaat
18901 tgataagtta gaaaatcaag ttactgattt gaaaaaagtc ttttataaac aatctggaga
18961 gagtcacagc acatttctat atggttgtga agtacgtggt tctgactatt tacatcttga
19021 agataaggta attcctcgtc tagaagagaa tgatcccgtt ctattaatac gtgaggctga
19081 taataagtat gataaaaatg ctatttctgt agctacaccg gctgggctaa aattaggtta
19141 tattccacgt gaacataatt taatttttag tcgtttgatt gactctggta atcttttatt
19201 tggtcgagta aaaaacttcc attgggatgg taaaaatttg gagttggtaa tcaaagttta
19261 cttggcaaat tagaatattt taaataaggt aaataaatat ttttattaaa ggttttaggg
19321 aattaaactc atagattttt agcataatag ttggagtggg aaatgctata taaacaaata
19381 gaacaaaata agcggaatac atggatcatt ttgggattgt atactgcttt attacttgta
19441 ttaagtattt ttcttggaat gatttttaca agatatgttg ggattggatt ttttatcatt
19501 ggtctgatct atattgcaca cgtatatttt gatgccacgc agcatttaat gaaagttacg
19561 aatgcggttg agattactaa agaaagtgat cctgaaatat atgagcttgt tgaggaactc
19621 tgtctagcag gaggcttacc ggttcctaaa atttacatta ctcctgatcc agatccgaat
19681 gcttttgcaa cgggacgcga tcctgaacat gcaagtttgg ctcttacgca agggctgttg
19741 agtatgatga acaagaaaga agtgcaagga gttatcggac atgaactttc tcatattaga
19801 aattatgata cacgcattac tgttctagct agtgctttga ccagtttgat taccataact
19861 gggattggaa tcgtattatt tggttgggga gtattgacta gcgaaagcaa gggctggcta
19921 ggactgatta ttaaattttt tgctatgttt ttaattatag ttggtgggat tatttcgatt
19981 attggtattc cactagctaa gttattgttt ttattagttt cgcgtcaaag agaatatttg
20041 gccgatatag gttcagttga cttaacacgt gaaccgagcg gattgatttc agcgttgtct
20101 aaactggaga aaatggagga aagcgaatca acaccagaaa ttaattctca agatttagct

20161 ttggagcatt tatacttcaa cttaccacat ccaggtaatt ggattgatcg actattttct
20221 gatcatcctc cgctagataa gagaatagag cggttgaaga atagcgataa gattgagtag
20281 aagagaggac tcccatcatt ttttgatgtt gggtcttttt gttttattaa tcaaagtaat
20341 ggactgataa atcaaggaac ttttagtttt ctctacgcca ggggagccac ccaaaaaagg
20401 atgtttctat ataccagaaa catccttttt aaagtacact tatttaccaa tcaattgttg
20461 aaaatcgcta accaattttt ggaaagttgc attcaaattt tgcataaatt cattttgatt
20521 atttttcatt tgattcagca aagaatcggc ttgatttgga ttttgcttaa tttgatcaat
20581 cgtagagccc aattgatcaa tttggttttg caaggcttga ttattagctg cattattttt
20641 caattgttga ttttcgttct taatttgatc taattgattc tgcaattctt ccttattttg
20701 atcttgctta taattattga ttgcattctg caattctttg ttttgttgca ttagttgctt
20761 gttttgctct gacaaattat ctaacttgct gttagtatta ttgagcgcat tactttgagc
20821 ttgttcattt tgaacagtat cattatgtgc ttggcaggca gaaataccaa aaataattgc
20881 aattagtaat acaagaacgc caagtacaat atagacaact tttttcttgc ttttcttttt
20941 tgaactaggt gtttgagatg gggtcttata ttgtggagca ggagtaggtt gcactggagg
21001 gcgagtagta ctatttacat tattcttatc agtgacccag gcttgaattt tcttagttgc
21061 atattcgtta ttagaaccaa ttgcagtaat gattaccgga taaacgcctg cttctgaacg
21121 gttaacagct tttttattaa tacttaaatt gtcatttgaa ataagctgtc cattttcagt
21181 tacagtcaac tctaattgag aaattatctc ttggttagtt aagttttggt ttagtggcca
21241 tgtgatagaa tcaacacggg tattaatttg caaagtcatt tttaattcct tctaaaactc
21301 taatctatgt atattaatta tactaataaa taagtattta gaaaaatgaa tcgatagtag
21361 taaaataaaa cttaattatt atagaaagtg aaaccttaaa tgatgaaacg ttctgagcct
21421 gtaactttaa ctaatatgtg tatgattaag aaaaaagata aaattttagt tcttgatcga
21481 aatgatcctg tttggcctgg tttaacgttt cccggtggcc acgttgaatc gcatgagtct
21541 tttcatgatt ctgtagttag agaaataaag gaagaaacag gtctcttaat taagaatccg
21601 cacttagttg gagtaaaaca attctttgac aaaaatgatg aacgatattt agtatttttt
21661 tatgtagcta ctgagtttac tggaacagta aaagcttctg atgaaggaaa attgacctgg
21721 atgacaaagg aagaattgac aagtaaaaaa attagcctat aattttgatc acgatttgcc

21781 agttttcttt gataaaaatt taagcgaaca tttactagat ggaaaaatag atcaaaccta
21841 ctaaaaaagg cactcaacag agtgcctttc atattagtca ttttgaacat cttcacttga
21901 ttcgcttgaa ttagaatcat tattctgagt agttggtgat tgcttttctt cagttgaagt
21961 tgaagcatta ttggatgaag aacttgaagt tgatgtactt ggttctgatt tagaattact
22021 tacagctgaa ttttgatggc ttgaatcggt gttattatta gttgaccaat tattactgta
22081 atagtaattg ctttttggag tgtaataact ataattctga cgaggaagag tatataaaga
22141 agtaccaaag tttgatttta agcttctatt ggtttcagta ctgttttcgt gcttagaatt
22201 atttaaagat ggaagagctg aactggaact ttgagcttct gagcttgaat tattttcatt
22261 cttatcaagg gtgtcaattt gatcatccat ctttttattt tcatctttag acttatcttt
22321 atcttttttc tttttatctt cgctcttctt tttaggctgt tccactttct tatgtgactt
22381 cttatgagaa gaagattgaa tgattgcata atcatccgcg gaagacttat gtttaggttc
22441 cactatgtta ttacggcaac ctgcaagtaa caaaagacca caacttaaag caccaacaat
22501 taacttttttc aaattaaatt cctccactta aaactttctt tattataacc aaaattctcg
22561 atataagata gcaaaattag actattttaa ctttatatga aaataatata tatttttcaa
22621 atttagaagc ttaaagaaca aattcggcga gagaaaagat ataatagaaa gtgtagttaa
22681 ttaatttgtt aaataaagag gttggaggtc aagcttatgt atgatcttga agacgttatg
22741 atgaaatctt ttagagatga taatggaatc agttctgcat tttctattga gccagtcgat
22801 gataatgagt accgggtttc tactaatgtt gtcgatggtt taaatgatat gatccacttt
22861 aatttgattg tatatcctaa tggaacttat gctttaaatg atcgtgtttt caccgctaga
22921 atgattgatg ataatattgg aagtttttca agtattgcgg ctgatgttgc acgtttagct
22981 agtgaatttg gtgttttgct tgatgatcgt ggtgaattaa ctcgtgatta tatcaatagt
23041 cgcgatttga ggagtgaagt taataacttt actcaattat tgaatcgagt agttgagttt
23101 gctcaactta atagatatta aagaagtcac tataatcagg cttctttttt ttttgccatt
23161 ttaggttata atttgttgaa aataggggag tgataaaaat ggaagaatct gaattaaaat
23221 taattaaaca tgcttcattc gactggttaa aacaaaatat tcaatttatt caagctggag
23281 aagatattga agttgcaaca ccgctaattg gcgcctatgg tgatttagtg tattgctgga
23341 tcgagaagga aaaggatgga gcataccgaa ttacagatga tggtggaaca ttgttcaaat

23401 tagatccagc tcaagaaaat tttgatttac ttgaagaagc agctgatatt
gtaattggtg
23461 ctggttttga atttgatgaa gataattcag aaatttatca aattgttgat
ctagagaata
23521 tggcacagac tttgagcgat ctaactcagt tgcaagttgc tttaacttat
ctagctagtt
23581 agaaaaatat ctcatcgtga tctcaagtat aaacttttgg cttttaaaaa
tagatatgat
23641 ataaagataa tgaaagcgct aaaagtagcg atttcttaaa atattacaaa
gaatgtaaaa
23701 aatctattaa tttgtaatat ttttgtaaaa ctactgtata ataaaagtaa
atagaacagc
23761 aagttaaagt atagcttacc ttgctgagga gcgtgattta tgatgagaca
aattaagaaa
23821 agaaacataa tgactggttt aggagcagca gcgttgctgg cttctttaag
tttaaatggt
23881 actgcttttg cagcaagtac tgatagtgca aatgcaggtg ctgagcaagc
aaagacttat
23941 caaactcagc ttgagcaaca taaaaactta caagcttata ctaatccttt
atcagttaaa
24001 actttaacca ctaccaaagg cgttttacca gacgcaacaa gtggcatttc
taattggaat
24061 acagttccag ctggtacagt tgccaactgg gataaaactc ctgaaattaa
caaggtaggt
24121 actagttacg gcactgttta tgtgactttt cctgacggat caaggtcaag
gcttgcagtt
24181 tatgtaactg ttaaagatac tgctgtaagt caagatagca aggcaagcaa
gaaagactta
24241 aatagtggtt ctgccacttc agctactgat cagcaagata atgcttccaa
gcaaactact
24301 agtgtaaaga aagatgtagc taaagaaaat acaagtaaga atagtagcga
aaaaactaat
24361 aaagatgtag tagtgaaaga tcttggttct actacggtaa ctgaaaagaa
tgtcagcgta
24421 gtaaacaata ccaaagataa taagaaaaat agtaaaaaat cttccaatcc
tgtgtcagta
24481 gctacgcaat taccagaaac tgaaggaaag gctgttaacc cgttagtagt
aattggtggg
24541 ttactagtag cagcaattgg tgcagttgct atcttcggca aaaagataaa
aaatagactt
24601 taaaaatatt atagaaacta aaaaaagagc ttgcgattta aaatcgtaag
ctctttaatt
24661 tatccaaatt tttattttat atgtcttagt ttagctaaga agttttcatg
ctctttttct
24721 aatttttcat ctttatcaat aaagaaataa catgcagcag caaatacagc
aactactgcg
24781 cctaaaagta atgtctttgg cgtaaaattg gttaccatat agcaagaaat
aattaaagca
24841 ataattggaa tggtgtattt cccaggaaga gaaaaaccat gagttggata
ttcattactg
24901 tgcttaaact taataacggc taaaattgat ggcacatact gaacaaatga
tgctaaaaca
24961 gtacatgaaa caaggaatag gtagctttga gtagccaagg cagcagatag
aattgctgtg

25021 aaaataattc ctacccaagg tgcatcatgc ttatttttct ttccaatcca
ttttggtaac
25081 attccatgtt cattagcaat agaagcaatt aatgatggag tattaaatga
tgcgctgaaa
25141 gcaacaccaa aaattgaaat cagcattcca ataatcatga aagcaaaacc
ccattgacca
25201 acgccagttt ttaaggcact agctagagga ttagaataag atcctaattt
ttgaccactt
25261 aaaccaattg ctactaaaag cattaagcaa tcaagaattg tgacactagt
cattacggca
25321 accaaaactc gagggatatt cttttctgga ttattcattt gttttgcagc
aatcggaata
25381 aatgaaaagc cagtaaacaa ataaaagatt ggtgtaaatg catcgccaaa
atgtttgaag
25441 aatggtgtaa caccggttaa agccgcatgc ggaattacgg gagtgaagtt
tgatttttta
25501 atgaagaaag ctccgatgat aataaagaca attaaagtaa ttatctttgc
agctgctgaa
25561 atattattaa caatttttac ccaagatcta ccaaagaaat taataactga
aaataataaa
25621 attaagaaaa taatactacc aatatagaca gcattatttt tgaaaatcgg
cagaaaactc
25681 ttgaggacag taagtaaagc tactacttca gctgaaagag ttgtacaacc
taaaaaccaa
25741 gtgaaaatgc cgagttcata accagcaaag cgaccaaagg cattatagga
ataaagccat
25801 gctgctcctg attgtgtaaa acggcttgat aagtcagcat agcacaaagc
aatcatgctg
25861 acagtgacag cagtacatag aagaacaaga attgctgtta ggttcatgta
acgataaatt
25921 acagaaggta gcaggaaagt tcctgatcca atgaccgcat taatacctaa
gaaatatatt
25981 gatataaagg agagtttctt aggtacagaa ttattattag tcatgttttt
acctctctat
26041 ctagtaacgt tctcctttat ctattgtaat gctttgaaaa agtaaatcta
tacgaaattt
26101 aaagaaaagc taataaaatc aataacaagt ggttgactta ttaataataa
gtattactat
26161 taaattgtaa gtaaggatag ctaggtaagg agaaaattat gactgaaatt
attcaagttt
26221 gcactttagc taaataagaa tttgagatgc ttttattaga attgaaatta
tgatttttat
26281 aatgtgagga aaataaaaat gacaaaaatt gctattttag gtgctgctgg
tcaaattgct
26341 caattagttg aaccaatgct attaaagaag aatgatatta atatggtttt
atatctacgt
26401 catcctaata agttaaagca aattaatgac gctagagaaa aagtaattaa
gggagacgca
26461 agcaatttta atgagctaaa agaagctttg gcagatgtag atctcgttta
tgcaaattta
26521 gctggttcaa atattgaaga tcaggcaaaa actgtagtta aagcaatgaa
tgcaaacggt
26581 attaaacgtt tgatctgggt ctcttcctta ggaatctatg atgaagtgcc
tggtaaattt

26641 ggagaatgga ataaaaatat cttaggctca tatttaacaa cttatcgggc agctgctgat
26701 cagattacga caagtaatct agattacacc attattagac cagcttggct aactaataaa
26761 aatgaaatta gttatgaaaa gactgttggt gccacaactc catttagggg aactgaagta
26821 tcacgtttaa gtgtagcgga ttatattgct gatttaattg aaaatccaac caaggatgtt
26881 aaggcaaatg ttggattaga taaagcaggt actgatggtg acaagcctgc ttggtattaa
26941 aaattaaaat taatgaaacg taaaaagaac ggtcatgacc gttctttttg tgtattatat
27001 ctacaatata aatcctactt cttgattcta gttttgtacc atttaatccc gaagtaaata
27061 aataaaatta ataaaagggc aataattacg agcgtgtagt catcgaagat tctgactaca
27121 acttgccagt tttttcccat atagtgtcct aatccaacta aaacactgtt ccaaactaaa
27181 cttcccaaag tagttaaaat taaaaactta gtccagccaa catgggcaat acctgcagga
27241 atggagatta aacttctaac aactggaata cagcgaccat aaaaaattgc gccaattccg
27301 tgtttgtcaa accagttaat tgccttttgt gcatcatctt ttttgaaacc tagaagttta
27361 aatagtttag tttcaagtag tctctctaag cgcggtaagg ttagatttct actaattgag
27421 aataaaataa tggctccaat aactgaacct agagtagacg caaggataac tccaattaga
27481 gttaactttg ttccactaac taagaaacct cctagtgtca aaattacctc agagggaatt
27541 ggagggaaaa tattttcaac ggcaattaaa agaataatgg caatatagcc aaagctttcg
27601 ataaattgtg ttaaccaagt tgtcatctat atatttattg tctttctaaa attttgtcta
27661 ttttttcaaa gaatgaatct ttatctgctt ttgtaacttc agtaatttct tttccattag
27721 gatcaatgaa cattcgtcca cttgctaagc ctgatgtgat tacattagct tttacctgac
27781 gagtttcaac aagctcagga tagagtgctg acatagttgt taaaacgtcc cacaagtata
27841 gctctgcatt aggtatagag ataattagac tgtatcctaa accaactaag tcgatagctg
27901 gatattttct taaacttgcc cagtgctgcc gaagttcatc agttaatgga agttcttcag
27961 tactttcaag accaaccatt tgaatcttaa ggtctgaatc taaaacgcgt tttacagctt
28021 ctggatccca gaaagcattc cattcctgtg ttccgtctgc atcgacacta actacattgc
28081 catgtccatc taatgaacca cccatccagt aaactttatc aatctttgac tgaatcgaat
28141 catcttcatc taaagctctt gctaagtcag taattggtcc tgtcatgatt aaggtaactg
28201 gtccatctgc ctttttttatt ttatcaatca tgtctaaatg agcaggcaaa gcagcttctg

28261 gtgttttaat ttctccactt tcatttagaa tagggaagta attaaaggaa taagttgcca
28321 ttcgccattc ttttggaaat tgatgaattg cacgcgaatt tgagcgagct acttctagct
28381 tgtcgccgcg caagttaaat ttatctacca tttttcgaca agcttctact gctggatcaa
28441 tatatccgtc accgtcaatt gcgctaattc caattagttt gatatcaggt gcttgcaata
28501 gtaaaagaag tgagaccaaa tcatcaatgt ttccatcatg attaaaataa atatttttca
28561 tttatttgcg acctttcatt ttgtttactt aactatttta taaaaagata tggtataagg
28621 taaataaaaa agacatctta cttggtttta agaaggaacg tgataaaata ctatgggaat
28681 tttaatagga aggaaaaata attgatggca acggaacaca ttgaaaataa attaaagctt
28741 ttaccagata aaccaggttg ctatttaatg aaagatataa atggcaatgt gatttatgtg
28801 ggaaagtcta agaatttaaa gaaccgcgtt cgttcctatt ttaagagtaa gcaagttggt
28861 cggcgagctg aacttgttcg tgaaattcgt gattttgaca ttattacagt ttcatctgat
28921 aaagaatctt ttttactgga aattacttta attaaaaaat atcaaccata ctataacgtc
28981 caactaaaac aaggtacggg ttacccatat attgaaatta ctaacgaaag ggacccgcaa
29041 actcgtttaa catcaattgt tcataaagat catggctatt attttggacc gtatccaaat
29101 gtttatgcgg ctcaagcaac gcttaagttt atccaaaagg tttggccgct aagaagatgt
29161 tcaggtcatc agggacgtcc gtgtctttac tatcatatgg gacaatgtct cggtgcatgc
29221 tttaaagagg tcccaaagag cgcttatgac aagcaaataa gaaaaataaa gcgtttctta
29281 aacggcgata tttcacaagt taagcaagac ttaactgaaa aaatgactca ggcttcaatg
29341 gatttggagt ttgagcgtgc agctgaattt agagatcagt taaaatatat tgaacaaacc
29401 gttgaaaaac aaaaaattat ttctaatgat catacccaga gagatatttt taacttttac
29461 gttgataaat catggatttc gattcaagta tttttcttaa gacaggctaa attattacgt
29521 cgggaaacta gattatttcc attaacagat actaatgaac cacaggatgc atttgtatct
29581 tttattgcgc aattctatgg ccaaagaaat cgtgttttac ccaaagaagt tttggtccca
29641 gctggtattg ataatgaagc attagctgaa gttttaggcg taactgtaag aactccgcaa
29701 agaggtcaaa aacgtgcctt actagagatg gcacatgaca atgctaagtt gaagttagat
29761 gaaaaattcc gcttgttaga attaggtaat agaaaaacta aaggagcaca aaaagagatt
29821 tttgatgctt tgggtttacc atatggccat cgaattgaaa gtttcgacca ctctcatatc

29881 caaggagctg atcctgtttc tgccttggtt gtgtttactg atggtgaacc agataaacat
29941 gaatatcgga agtataaatt gaaaggcgaa gtagaacacc aaaatgcggc tgacgaagta
30001 ggaaatacac gtgaagtggt gagaaggcgt tatagtcgtc ttttgaaaga acacaagaaa
30061 atgcctgacc tgattttaat ggatggtggt caaattcagg ttgatgcttg cttagatgtg
30121 ttgagaaatg aattaaatgt caatattcct gtagcaggga tggttaaaga cgaccatcac
30181 cgcactaatc acttgatttt tggtgatcct actcaaggag aagaattaaa gctaattcct
30241 ttaaatccga aatcagaagg attctactta atgacaagaa ttcaggatga agttcaccgc
30301 tttgcaatta cttttcatag aagaactcat gctaaaaatg cactttcaag taagttagat
30361 gagataaaag ggattggtcc taagtctaga aataagcttt tacgaaagtt tggatcttta
30421 aagaaaatta aagaagcttc aatcgaagaa ttacgagatg cgggtttaac tcttccacaa
30481 gcacaaactg ttaaattgag tttgtaaaat aacgagaaaa gacctgattt aatagggctt
30541 gatatggtat catagttaag aaactttaag aatttttta attataactt gatatataaa
30601 aggaggcata cttaatgttt gtcgaccaaa caaaaataga tgtacaagca ggaaaaggtg
30661 gcgatggagc agttgctttt cgtcatgaaa aatatgttcc acttggcggt ccggctggtg
30721 gagacggcgg acgaggcggc agtattattt tagtcgctga ttccggtttg agaactttaa
30781 tggattttcg ctttagacgt aagtttaagg cagataatgg tgaaaatggc cgtatcaaat
30841 cccaatatgg tcgcggagct aaagatgtac gcttaaaagt tccaatggga acaagtgtct
30901 acgactttaa taccggcgaa cttttaggtg acttagttaa aaatggtcaa gagcttgtag
30961 ttgctcatgg cggaaagggt ggcagaggaa atattcactt tgctacccca accagaacag
31021 ctccagaaat tgctgaaaat ggtgagccag gagaatttcg aaccttacgc ttagaattaa
31081 aggtattggc agatgtagga ctagtaggat ttccatcagt tgggaagtca acacttctat
31141 ctgttgtaac taaggcaaag ccgaagattg cagcttatga atttactact ttgacaccaa
31201 atttaggaat ggtcgttttg ccagacggac gagacttttc aatggcagat ttaccaggat
31261 taattgaagg tgcatctaag ggcgttggac ttggaattca attcttacgg catgttgaac
31321 gtacaaaagt aattttgcac ttagtttcaa tggatccaaa taatggtaga gatgcctatg
31381 aagattatga aactattcgt aaggaattag caggctacac taaggactta acaagtaaga
31441 aagaacttat tgtagcaacg caaatggata tccctggtag cgaagaaaaa cttgctgaat

31501 ttaagaagaa attaggagat aagacggtat atcctatttc tagtgttacc catcagggtg
31561 ttagcgaact aatgggtaag acagcagatt tagttgaaga agttgcaaaa gaagaagctg
31621 aaaaaccagc agaaattaaa gttgccgaaa aagaatatgt ttacaaaaag cctgaagatg
31681 atggatttaa ggttgaacga actggtgagc atagctttat tgttactggc aataagttag
31741 agcgtcttgt tcaaagaact aatctagatc atactgatgg aattatgctt cttgctcgta
31801 aattaaagcg gatgggtgta gatgatgctt taagagaaaa gggagcagta aatggtgacg
31861 atgtatcaat agctgacttc acttttgaat ttgtagatta ataaaattta aaaaagttgg
31921 gagtaagact tttgaaaaga aagaaccgct ttattacagg ctatgccgga cttcgtgctt
31981 tagctgttat tggagtaatt ctatatcacc ttaatccaaa tcgatttatg ggtggatatt
32041 tgggtgtgcc gatattttta gtactatctg gatatttagt tacggaccac atgtttcatg
32101 cctatgaaga gcggggaaag tatgacttta agtcttttta tgtaagaaga attaagaaat
32161 tatatcccca aatgattgct cttttatggg gatgtagtgc ctatatctta ctttttcagc
32221 aaaatctttt agctaaatta aatcaaatag tagttgccaa cctattaaat gtttataatt
32281 tttggcaaat caagaatggc caaagttatt ttgagcgttt tgcctccaat gaatctccat
32341 tcgttcattt atggacgatg tcaattgaag ggcagtttta tattatttgg ccactagtag
32401 tttttctatt agttaaatta gctaaaaaga aaaaaataat tttttggatt attacagcct
32461 taactgtatt ttcagcttta gagatggcgt ttttatttaa gccaggtgtt gatacaagta
32521 gaatttacta tggaacagat acgagattct tttcattagg cctgggtgca atgttagccg
32581 tcttttggcc aacatggaag ttaaatgcca accttgaaaa aggcgataga cgactcttaa
32641 acgtaattgg agcaatagcc ttagtcggta ttttattgat gtctactagt ccgattttta
32701 acccacaaaa agcttttgct taccgtggtg ggatgttttt attctcttta gtaacaatga
32761 tttttgtagg ggtaattgct catcctggta gcagctggaa taagtggctg actaatcctg
32821 tatttagctg gattggttca cggtcatatg gaatttattt atatcagttc ccagtgatga
32881 tctttttga agataaggtt aaagatattg cagaccatgt aattttgtac catttaatcg
32941 aaattatatt aattttagta attgcagaat taagttatcg ttttattgaa aaaccatttg
33001 gacgaattga ttgggaaaaa ttgcgtcagt ttttatcaaa agccctaaat ttacaagcta
33061 accattatca atcaaaagct ttatttgcga ctggaatagt tgtctttatt ttaggtagtt

33121 ttggaattct taaggcacca actgtaaaag ccgaaaatcc taatcattca gaactagcaa
33181 ctcaaattaa atcaaaccga aacaagcaac tgaaaaagaa taaaaagtta attaaagaag
33241 cacagtctaa aaagaaaaag tcaacaactt caaaagcaga aattttagcg caagctaaaa
33301 aagcagcaac aactaaaccc gtgaataaag attttgaaaa atatggtatt tctcaagttg
33361 acttgcaatt agcacaaaaa attcaagtaa ctgcaattgg ggattctgta atggcaggct
33421 ctagtcaaaa cttgcaaaaa ctaatgccac atttaattgt tgatgcagct gtttctagac
33481 aacttggtga cacaattcca ctttttgaag agtataagaa aaaaggtgct ttaaataata
33541 atgtcttgat cggtttagga acaaatggtg cttttgcgcc taaagaactt gatcgtttaa
33601 tgcaaattat tggaccaaag cgacaagttt tttggattaa tacgcatgtt ccgacacgtg
33661 attggcaaga tcaagttaat ggtgaattgg atgcagcagc gaaaaaatac cctaacttaa
33721 ccattatcaa atggcatgag tttgccggga gtcatcaaga ttggttctat gatgatcata
33781 cgcacccaac tacagaagga tcaaagtttt atagtgccta tgtaactaaa acattggtta
33841 cgaaaggaaa atattagaaa tagggttttc atggaaatac aatttttagg aacaagtgct
33901 gggcagccgt caaagagcag gaatgtttct tgtaccgcac ttaaattatt agatgaatta
33961 aatgaagtat ggttatttga tgtaggcgaa gctacacaac atcaaatttt aaagactaat
34021 attagaccta gaaaggtaac gcgaattttt atttcgcata ctcatggtga tcatattttc
34081 ggtttgccag gttttctttc atctcgttct tttcaaggcg atggtggtcc attaacaatt
34141 tatggccctg ccggaattga acaatttgtg caaacttctt tgagagtttc aaaaactcgg
34201 gtatcatatc caattaaata cgttgtttta aaagaagacg gtttgatttt tgaaaacaat
34261 ctttttgccg tttacactgc tcgactagat caccgtgtac caagttttgg ttttcgtgtg
34321 gttgaaaagc cacgtcctgg agagttgtta atggataagg tggcagaata taatgtgcca
34381 aacggtccgc ttctcggtca actgaaagct ggaaagacga ttacgttgag tgatggtcaa
34441 aagttagatg gacgagattt ccttggtaaa gagcgtccag gccgagttgt tacgattatc
34501 tatgatacgc gtccaacgga aaatattggc gaattagctg atcatgcaga tgttctagtt
34561 catgaatcta cttttaatgg tgatgaagaa aaaatggctc atcgttattt ccattcaacc
34621 tgcttagatg ctgctcgtat agcgagagat catcatgtag gaaagcttta tttgactcat
34681 atttctgcgc gctatacagg aaaagcaggt aagcaactag agcatgaagc gagaaaaatt

34741 tttaaatata ctcgtttagc caatgattta gacagttttg aaattacatt gaggggataa
34801 taatatgagt aattcattga gggataaagt cgtagtagtt actggagctt caagtggtat
34861 tggacgctcg attgtgttag agagtgcaag tcgcggcgcc actgtcattt taatggcccg
34921 tcatcaagat aaattagaag aaattgctaa tgaagctcgt caactttcag gaaacgacgt
34981 atttgtattt ccaactgata ttagtaaagc tgatcaaatt gatcgtgcct ttaatgaaat
35041 tattagtcat gttgaccata ttgattatct agttaatgca gctggttttg gcgtctttaa
35101 agaattttta gagacttcac ctcaagtagt aactgaaatg ttccaaacta atgtcttagg
35161 cctaatgtac tttactcgtc ttgttgctcg agtaatgatt gaccaaaaag ctggccaaat
35221 tatcaacttc ggttcgattg ctggaattgt acctactact aaaactgcag catatagtgc
35281 aactaaggca gctattattc aattttcaaa tgttttacgt ttagaattaa agccatttgg
35341 tgttaaggtt atgacagtaa atcctggacc agtttacact aatttcttta atatcgcaga
35401 tgaaagtgga tcttatgcaa ataatgtaca acgttttatg ctcgatcctg atgatgtagc
35461 ttggcaagta gttcatttct ttggttctaa tcgtcgcgaa ttgaatttac cattaagtct
35521 agctacttta gcaaaacttt atcagatttt cccacgcatt ggtgattggg caagtttgaa
35581 gtttgctagt agaaagtagg agataataat gaataagcaa agaaatttag taattgggct
35641 aataattgtt ttagtgcttg taattttcgc ctgtttaaat actgaaccag tggcaattaa
35701 ttttggcttt ttccaaccta aaatgccgtt gataattgtt ttagttatta tgcttttact
35761 aggtgcttta gtttcacttc taattggaaa aggagaaaaa gtttctcctg atgtaaaaaa
35821 agcactagca aaacagaaga aagaattaga taataaatac cagagtgaaa ttgaatctaa
35881 agataaacag attgctgaat taaagaaagt taacaattct gaagctacaa aataagattg
35941 gacttgattt atcagctctt agattgtatt attgaaatac gtgagtattt agtgaatttg
36001 tttatttaaa ggttcgcaag tctcaattct ataaaaaagg agatctaact aatggcagtt
36061 cctgcaagaa aaacttctaa gcaaaagaaa cgttcacgtc gtggtcatat taaattaacc
36121 acaccagcaa tgcattacga tgctactact ggtgaatacc gtttaagcca ccgcgtttca
36181 ccaaagggct tttacaaagg ccgtcaagtt gctaacgaaa acaagcaaca aaataacgat
36241 taattaaaga caccttgggg tgtctttttt tatgtaattt ttgattaaag gaataggtat
36301 taatgaaatt agttatatta cactcaagtg atacgcatgg ctttttgatg ccaactgatt

36361 atcaaaataa agacaactat gatgctccaa tttctcttag tcgcgtaagt agcgtggtta
36421 aaagtgaaaa agaaaaatat ggtgcagaca atgtcctagt tactgatagc ggagattgtc
36481 tacaaggttc acccttagct agctatactc aaaagttgcc tgaagacgaa ggattgcgta
36541 aatttactga agcttataat gaagtgggat atgatacacg tgcattaggt aaccatgact
36601 ttaattatgg tttggattat ttatcgtact atgtcgataa caatcgtgct ccatttatca
36661 atgataatgt tttagatgca gatactaatg tacctttctt tggaaaagaa tatacaattg
36721 ttgaaaaagc tggtctaaag ataggtatta tgggaattac tactcagtat atctctcact
36781 gggagtcaga ggatcatatt aaaggcttaa agtttgtttc tggttatgaa gtacttgcta
36841 aattagctaa aaaattgcgt ccacaagtag atattctttg tgctctatat catggtggtt
36901 ttgaaagtga tccattgact cgtaaagcaa ctgagcctca tacaggcgaa aatgagggct
36961 ataagatttt aactgaaatt ccggaaattg atgtcttttt aactggacac caacatcgcc
37021 gcttgaactt agttactcgt aacacggcca ttgttcagcc aggctaccgc ggagaagcag
37081 taggtaaagt gatcattgat tttgataaag acacgaatgg taaagttaaa atcaatgata
37141 tgactaccga attgattgat agtaaagatt atgcaccaga tccagcaatt gaaaagatcg
37201 ttaagagtct tgatgaagaa acacaaaaat ggctagatca acctattgcg catttatctg
37261 aacctgcacc aattgatgat gctatcaaag gcagaattga gggagcacct tttatcaatc
37321 ttattcaaca aatgcaactt tggtttactg ggcagatgt ttcagcaaca gcaattatgt
37381 ctgaatctgc ccatggcttt tcaaagaatg taacaatgcg cgaagtatta ttaaactatc
37441 catatgcaaa tcaactctgc attgttaagt tgactggaaa agaattaaaa gaaattattg
37501 aatattcagc tggattctta gaaaaagata aagacggtaa aatcaaattt attgatcgtt
37561 ggattacacc taagccaatg ctttatcatt ttgatctttt ctacccagta aaatatgaag
37621 ctgatctttc aagaccagtt ggtcatcgat tgactaaagt aaccttaaat gggaaagatc
37681 ttgaagatga taaggtatac aagttagctg tcaataatta tcgtgcttta ggcggaggat
37741 tttatccagc ttacagcatg gataagattg aaaagatctt tgatcaggac tacgttcaaa
37801 tgtttagcga atacttaact catggtgata ttaaagtaga tactgctaaa aactacaagt
37861 tttattaatt aaggtactaa aaaagctgtt tcattacgaa acagcttttt aaaatattca
37921 aatttagttc aattaaagtt tgttgtactt actcttgtct ttgtaactct tggcagtaat

37981 ggcgggaata ataattgcga atagcaagcc aaagactgcg ccaataatca
aagattgcat
38041 aggattaaat tgcatctgag ttaaagatgc agcgataaaa ccgataactt
cgcaaaaaat
38101 gcatgaccaa accatagtaa taatatagcg cataatttct ccttctttcg
ctaggtatct
38161 tagcaaaaat aagagtttta cgcaagccaa gaagagagaa tttgctatac
ttaaattatt
38221 agaaagggtg agcatatggg agcagtgagt ctgcaaaacc ttagtagttt
tacattactg
38281 gagagtccaa ctaaagttaa agatttagct gaaaatgcaa agaaaaaagg
ttattctgca
38341 ctcgccttga cggatgtaaa tattacctat ggcttagtta atttttataa
agctgcaaaa
38401 gaaactggaa ttaagccttt attagggatg cagttaagaa tcaacggctt
ggttgaccag
38461 gcaaataagt atgatttaat tgtcattgct aaagacgatc aaggatataa
gaatattttg
38521 cgtttatcaa gtgcggtcaa tcttttaacc gaaaatggtg aaaaagaaaa
tgttttagag
38581 cttgaagaat taaaaaaata tctcggtcac ttagttataa ttacgcctag
taacatacat
38641 agcgaactaa agatgctgca agctaacaat cctaaaatgg gagctaatta
tatccgtaca
38701 ctgaaagatt ctgttcctac atcttcatca atttatttag gcgtttatgc
tgatcaagga
38761 cagcaagaat atataaatta tcttcgttca ttagcaacgc aatttgagtt
attgttaact
38821 gcggttgaag acggacaata tcttaatcgt aacgaacaat ttttaagaag
aactctgcaa
38881 gctattaaga gtaacaccca tttagaaaat gtggagcaat tagctaagca
agctggttcg
38941 cattacttaa aaaatagcga agaattgcag gttaattatc gaaaatttgg
gattgaagat
39001 gctttagaaa acgctgaaaa aataggtcaa atttgcagtg ctcagattac
gtttcaagat
39061 cctcaattgc caaagttcaa acaaaataag tttccaacat caaaagaata
tttgcattca
39121 ttagcacaaa atggacttgc taaaagattt aaaggaagga ttccggaaaa
ataccaagca
39181 agattagatt atgaactaaa agtaatcaat gagatgggat ttgatgatta
ttttcttatt
39241 gtgtgggatg tgatgaactt tgcccatagt gttcacatta caactggacc
tggacgaggc
39301 tcagctgctg gttcacttgt ttcatatgct ttaagaatta ctgaagtcga
tccacttgaa
39361 tataatttgt tatttgaacg tttcttaaat cccgctcgtc agcaaatgcc
agatattgac
39421 ttagatattc cagataatcg acgtgatgaa gtaataaaat atatgtttga
aaaatacgga
39481 atgaatcacg ccgcccaaat tttaaccttt ggtactttgg ctgctaagca
agttttaaaa
39541 gatgtttgtc gtgtttttgg actaaataaa gttgaaacct atcgctggtt
agatgcaatc

39601 ccgcatgcaa aaggaaaaat cactttagca gaagcatatc aaaaatcaaa agagttacag
39661 cttttagtta atacaaatgc atttagtaaa attctatttg cgactgctga gcatttagag
39721 aatttaccgc gtcattattc aattcatgct gccggtttag taatcactga tgattcatta
39781 gcagagattg tgggcttaca ggcgggacct ttaggaatac ctgtaacgca gcaaaccaaa
39841 ttaaatgttg agtccttagg attattgaaa attgactttt taggcttaag aaacttaact
39901 attttaggca atattattgc agcattaaag tcagaaggag ttgaaattga tcctaatcag
39961 attccgttaa atgatcaaga aacgcttgct ttatttcagc gtggtgacac agatgccgtc
40021 ttccaatttg aatctgatgg aattaagcgc gtcttagagc agcttcatcc tgacagtttt
40081 gaagacattg tcgctgtaaa tgctttatat cgcccaggtc caatgaataa tattggtcat
40141 ttcattaata gaaaacatgg taaggaaaaa gttcaatatc ctgatcctag cttaaaaaaa
40201 atcttaggac caacttatgg cgttttagtt tatcaagagc aagttatgca gacggctcaa
40261 gttttagctg gtttttcttt aggtgaagcc gatcttttaa ggcgagctat gtctaagaaa
40321 aacgctgatg tgatccaaaa agaacgagag aagtttattc aaggcgctgt aaaacttggc
40381 agacgaaaag agatagcaga gcaggtttat gattatattg ctcagtttgc taattatgga
40441 tttaaccgtt cgcatgcagt tgcatatagt aaaattgcct tttggcttgc ctattttaag
40501 gttcattatc ctggcgcttt ttatctttct cttttgaaca gtaatattgg caatcgtaat
40561 aagatcgctc aatacttgat gcaagcacaa gaagctggaa ttaagacttt gccaccagat
40621 attgagaaca gtaaggcgga tttttcgctt gaaaacggca aaattttagt tggcttgaag
40681 gcagttcgcg gattaagaag tgatttgctg aaacaaatat tagaaattaa gcgtccgatt
40741 aagtcaatga ctgatttctt atggaagatt gataataatt tgttgagtgc tgatgctatt
40801 gcaaacttaa ttaaagcagg tgcttttgat cgactagctc caaatcgaaa tgaactttta
40861 aaaataaata aagacctagt tgaaagtgta aagatggcag gttcaaattt atctttattt
40921 gaaactttgg agcccaaaat cgaagaagaa aaaatgccaa ctgctgctga aaaatcagcg
40981 atggaagtag aagcaatggg ctttagtact ggaattaacc caatcattgc tgttcaaaaa
41041 tatgctagaa agtataacgc aaaaagattg caggcttttg aaaacaatga acaggggatt
41101 gcagttggca aattaatgaa gataaagcaa atcaccacta aaaaaggtga taatatggct
41161 ttcgcggtct ttagcgatag tagtggcgat aaagatttta cgatctttcc tcaggtatgg

41221 gagaaagtag gagaaaactt aaagattggc gaaatatatt tattgcaggt gaagacgcaa
41281 agtgatcgct ttagtccgac aaagacgcag tttttacttt caaatgctcg caaagttaat
41341 tttaaagatt aatttaaaac ttttaaattc tagcaaaata gattctgata ggattgaaag
41401 cgaaaacatt tatttaacag actttattaa cgaaaaaaat aggattattt ttgaagacaa
41461 tgagttaaaa aaatggtacg ataaaaccga tgtgagtaaa acacagaaaa tcgtagaggt
41521 gaattcatga aacggattgg tattttaact agtggtggag atgcccctgg tatgaacgct
41581 gccattagag ctgttacacg tacagctttg gcaaacggta tcgaagtttg cggaattcgt
41641 tatggatatg ctggtttagt tgctggcgat attttccaaa tgacttctga aacagttgcg
41701 gacaagatta accgcggcgg tacctttctt tattcagctc gtttcccaga atttaaggaa
41761 gaagaagttc aacttaaggg aatcgaacaa ttaaagaagc acggaatcga tgctttagtt
41821 gtcatcggtg gtgatggttc ttaccatggt gcattgaagc ttacaagaca tggctataat
41881 gctattggtc ttcctggttc aatcgacaat gatattcctt acactgattt cacaattggc
41941 tttgacactg catgtaatac tgcaatggaa gctattgata aaattcgtga tactgcaaca
42001 agtcaccaac gtgtatttgt tgtaaatgtt atgggtcgtg attgtggaga tattgcaatg
42061 cacgttggtg ttgctactgg cgcagatgca attgttatcc cagaagaacc ttatgacatt
42121 aaggaaattg ctgaaaactt gaagcaagga tttgctaatg gcaagaagca tggaattgtt
42181 gttttagcag aaggtgttat ggacgctaat aaatttaagg atgaacttct taagtatggc
42241 gactttgatg ctcgtgctaa tatcttaggt cacatgcaac gtggtggaag cccaacaact
42301 cgtgatcgtg tattagcaag cgaaatgggt gcttatgctg ttaagttgtt acttgaaggt
42361 aaaggcggct tagctgttgg aattgaaaac aacaagcttt ctcaccatga tattcttgat
42421 ttgtttgatg caaaacacca cggtaattat gcgttatact cattaaacaa agacttagcc
42481 aagtagtttt tgattttgta ttataggaga ttttttcaag atgaagaaaa ctaaaattgt
42541 tagtacttta gggccagcct caaacgatgt tgagactatt actaaattag ctgaagcagg
42601 tgcaaacgta ttccgcttca acttctcaca cggtgaccac gaagaacatc gtgcacgtat
42661 gaacatggtt cgtgagattg aaaagaagac tggtaaactt cttggcattg ctcttgatac
42721 taagggtgcc gaaattagaa ctactgaaca agaaggcggc aagttcacta ttaacactgg
42781 tgacactatc cgtatttcta tggatgacac taagaagggt aacaaggaca tgatccacgt

42841 tacctacgat ggactttacg atgatactca tgttggtggt cacgtattaa tcgatgatgg
42901 tttagttgac cttttgatta aggaaaaaga tgacactaag agagaattag tttgtgaagc
42961 tcaaaacact ggtttaattg gttctaagaa gggtgttaac gctcctggtg ttgaaattcg
43021 cctcccaggt atcactgaaa aagatactaa cgatattaaa ttcggtttaa aggaaggtat
43081 caacttcatc tttgcttcat ttgttcgtaa agcacaagat gttcttgaca ttcgtcaatt
43141 gcttgaagaa gcacactgtg aatacgtaaa gatcttccct aagattgaat cacaagaagg
43201 tattgataac atcgatgaaa tcttaaaggt ttctgatggt ttaatggttg ctcgtggtga
43261 catgggtgtt gaaattccat ttatcaatgt tccatttgta caaaaggaat taatcagaag
43321 atgtaacgca ttaggtaagc cagttattac tgctactcaa atgcttgact caatgcaaga
43381 aaacccacgt ccaactcgtg ccgaagttac tgacgttgct aacgctgttc ttgatggtac
43441 tgacgcaact atgctttcag gtgaatctgc taatggtctt tacccagtta aagctgttaa
43501 ggctatggct gaaattgaca tgcgtactga aaagcaactt gctaagcgta acaagttagc
43561 tcttcaaaga tttgaagaat acaagggttc aaacgttact gaagctattg gtgaatcagt
43621 tgttagaact gctgaagaat tgggcgttaa gactattatt gcagcaactg aatctggtta
43681 cactgcacgt atgatttcta agtaccgtcc aaacgctgat atcattgcta tgactttcga
43741 cgaaaagatc gaacactcat taggtgttgt ttggggtgta aagccaatgt tggttgaaaa
43801 gcctaagact actgaagaaa tgtttgacaa ggcagcagaa cttgctaaag aaactggtct
43861 tgcaaaagac ggcgacttgg taattatcgt tgctggtgtt cctttgggtg aaactggtac
43921 tactaacttg atgaagttac aattgatcgg tacccaactt gttaaaggtt taggtgttgg
43981 tgaacaatca gttattggta aagctgtagt tgcaacttct gctgaagaag caaacagcaa
44041 agttcaagat ggtgacatct tagttgctaa gactactgat aaggattact taccagcaat
44101 caagaaggct tcaggtatgg tagttgaagc ttcaggttta actagccacg cagctgttgt
44161 tggtctttca cttggtatcc cagttgttgt tggtgctact gatgcagttg aaaagatcaa
44221 ggatggttct aagattactg ttgatgctcg tcgtggtgct gtttaccaag gtgaagcaac
44281 taacctttaa tctaaattaa ttagcttatt aaaatagtca gcgaaagctg gctattttat
44341 ttttgtcttt ttttccttat actatatagg tgacagacaa attagaagga ttgaggcgct
44401 gatatgttag gtgaaataag aaaaggaaaa gttactgatg aaaatcaaac tgctttttat

44461 gtacaggttg atggcgtaac ttttgaatta aagaaaatag aagttacaca agatgagcca
44521 atccatttag gtgacgaagt acagggcttt atctatgaaa ataaagataa aaagaaggaa
44581 atgacgcaat tttatccttt tgctcaaaaa gatcagtatg gttgggctac agtaactgaa
44641 gtgcgccgtg atttgggtgt atttttagat attggattaa atgataaaga cgttgttgtt
44701 tctttagacg atcttccact tgaaaaagat caatggccaa agaaagatga tcgtttgtta
44761 gtacgccttg agacagatga taaggaacgt atttgggcta agatggcaga agaagatgtt
44821 tttgaacagt tagctgccaa tttccctgcg catttggaaa ataaaaatat gtctggtact
44881 gtgtaccgta actatgaagt tggatctttt gtaattactg atcagtatta cttagccttt
44941 gtccataagt ctgaaatgtt ccgtcccttg cgcctagggc aaaaaattaa ggcacgcgta
45001 atcggggtta gtcaatatgg tagattgaat ttaagcgttc ttcctcgagg ctttgaggag
45061 attgatgatg atgctcaaat gattttagta agtttacgcc gtgaagcaac taagacctta
45121 cctttttatg ataaaagcga tgctcaagaa attaagaatc actttggtat ctctaagtca
45181 gcctttaaac gcgctttagg tcatttatta aaaaatagat taattactga agataaagat
45241 gcaggaacaa ttagcttggt agaaaaagat gaatcagaca cagacgatga aggataattt
45301 agaagattat ttacgtttca gtcaagtgga acgtggctta agtcctaata ccattatttc
45361 ttatcgaact gatttagaag aatatttaga ttatttacaa gatcaaaatg aaacaagttg
45421 ggaagttgac tatttagttg ttgacgcttt tcttgctacg caaaaagaca agggcaaggc
45481 aacaacctca attagtcgaa tgatctctag tttgagaaaa ttctatcaat ggctcttaag
45541 acaggatata attgaacgtg atccattaat aaaaatcgat tccccaaaaa gtgaacgaag
45601 attgccaacc gctttaagcg aagaagaagt agataaatta cttgcggctc ctgatacgaa
45661 tacgccatta ggaattagag atcgtgcgat gctagagtta ctttacgcaa ctggaatgcg
45721 ggtgagtgaa ttaataaatt tacgaactgg tgacattcat tctgatttaa aaattattag
45781 agttcttggt aaaggttcaa aagaacgact tgtaccaatt actgaagttg ctttatcgtg
45841 gcttgaaaag tatcaaaagg atgttaggga tgctcaagta ctaaaaagcg gtcaatttac
45901 tgatgtaatt tttctaaaca atcatggtca tcaactaacg agacaggctg tttggcagaa
45961 gataaaaaag tattgtcagc tggttggaat aacaaaaaat gttactcccc atactttgag
46021 acatactttc gctactcatt tgcttgaaaa cggggcagat ttgcgtgtag ttcaagaaat

46081 tttgggtcat agtgatatta caactacgca gatctatact aatctgacgc agaagcatat
46141 tttggaggtt tataatcaag cacatccacg tgcttaaata ttatgatcag caaatataaa
46201 aaagactatg aaaaaactgt aatgggatat ttgtcttatc ttccagattt taaaaatatt
46261 aagaacttac aagaagagat gaagttatat actagtgatt ctaatcaatt tgaagttctt
46321 atttataaag aaaagggtaa cgcaagaggt attattggca ttcaagaaga aaaagacttt
46381 ataattattc gctatctatc tcttgatcca acgatgcgtg attcagaaac caaacgcttg
46441 gtaatgaaag atctaaagca tctttatcct gataaaatga ttaccgcttt acctgactgg
46501 gcctttttat taagctattt aaaggaagat aagaccgatg aataatgtcg atgaattaac
46561 tttagatctt ccaaatttta cgggaccatt agatctactt ttgcacttaa ttagatcaca
46621 aaagatagat atttacgata ttccaattgc gaagattact ggacaatact tagctaattt
46681 ggctcgctgg cagactttag acttgcaaat tgctggcgag tattttgtga tggcatcaac
46741 tcttcttaga attaaatcgc aatatttact tcccaaaaat gactttgttg aagaagatca
46801 atatcaggaa gacccgcgtg ctgaactagt tgagcagctg gtccaatatt cagtttttca
46861 aagaattgca gaatacttta agaagcgaga tgaagaaatg ccaattactg tggctaaaga
46921 tccatctgtt tcgcctaaaa aagaagttga gcctcttccc ttaggagaaa ttacaagtga
46981 tgagctggct aatactttta aagtcgtttt ggagcgtttt aaattaagaa aaccacaagt
47041 gggacaaata gaagttcatg aaacttcaat tgaagaaatg acgacatttt taaaggataa
47101 acttcaccat agaaaaaagg ctagcttttt tgactgtatt aagaattttc aggatcttga
47161 tcaggtaatt ggtttgtttt tagcagtttt agaattatgt cgtgatcata aaattttagt
47221 aaagcaaaat agagactttg gtgacctaga attagagaaa gttgagacta atggcaagta
47281 aagaagcaca attagaagct ctcctttatg cagcaggggа tgatggtttg gaaacagata
47341 atttattaca attattagaa atttcaccat cggcattaca agagttagca aatcatctaa
47401 aagatcgact taaaaatgat gagaactctg gcttacaact gatctgcatt aatcatactt
47461 ataaattaac taccagtgcg gaatgcgggg atgttatttc taaatttttc caaaaagatt
47521 tatcgaagaa tttaagtcag tcagctcttg aaatactttc aattattgca tatcgtcaac
47581 cgataacacg ggttgaaatt gatgatttac gaggtgttaa ctcatcagga gcattgcaga
47641 ctttagtttg gcgtggactg attaaagtaa atggtaaaaa agacgtacct ggacatccta

47701 acttatatgt gactacagac tatttcttac agtattttaa ctatgaaagt ttggctgatt
47761 taccagtaat agaagagttt gaatcagatg acaatcctgt gaacttattt gatcaggatg
47821 agagaaatag caaagaaata aattttgatg aaggagagta aaatggctga aagattgcaa
47881 aaagtaattg cacaagccgg cgttgcgtca agacgtaagg ctgagaaaat gattacagct
47941 ggacaagtaa ctgttaatgg taaaagaatt actgagctgg gaacgaaagt tgaaccgagc
48001 gacagaattg aagtaaatgg agttccaatt gagaaagaaa cactccacac atatttgttt
48061 tacaaacctc gtggcgtaat ttcttcagta aaagatgata agggcagaaa gactgttgtc
48121 gattactttg aagatgtacc atatcgctta tatccagttg gtcgtcttga ttatgatact
48181 tcaggcttgt tattaatgac caatgatggt gaattagcta ataagttgat gcatcctcgt
48241 aatgaagtac caaaggttta tgtagccaaa attaaaggac ttctttctcc agaagatatt
48301 cactctctta cgcatggggt tcgaattgat ggacgtaaga gtgcgcctgc aaaattgaag
48361 attttgaaga cggatgaaaa gaaaaatact caaattgttc aattaacaat tcatgagggg
48421 cattatcacc aagttaagag gatgtttgaa gcagttcatc attttgttga aaaattatct
48481 cgtgaaaagt atgctttctt gactttaaaa tcactgactt caggtgaata tcgcgaatta
48541 acgcatgaag aagtctctcg gttaaatcac ttatctgact aacgctactt gttaagctat
48601 gataaaataa aactaataat ctataattgt ggaggatttt ggaatgaagg aaaataaatc
48661 tcaggacaat caagaaccta agggccctta caagcatttt gaacgtccga caacgtctag
48721 aagtgcgatg catcatcgcc accgttcaga gcctgcaccc gaaccaactc ctagtcgtcc
48781 agataagaac tctaagggta caaaccatag tcagttatgg gctgggttaa ttatagttgc
48841 aataattttg atcgccttga ttccgattgt ttcttcaaag ttgcactcta attctaataa
48901 cttggctgaa aagagtgtta aagtttcaaa gagttcttct aagtctcata aatctaagag
48961 taaaaagcct aagcgtagtt cttcgaagaa gagtaagtct aagagtagtg tgaagaaaga
49021 agagaagaaa tcgtcaagcc aatctcatga cgtaactcca gctgtaacta gtcagtctca
49081 aagtagtcaa actagccaaa gcagtatcca atcttatgct tctagtgcta gtcaaactac
49141 cactcaagat caaactcaac aagcaaacca aaattcatac caaaataatc aaaatacctg
49201 gcagaataac agtggaaaca gctcgtcaca aaatcaagct ccaagttctt acactgttca
49261 agaaggcgat tctttatcaa aaattgctcg tgaaaataac acttcagtgc atcatttaga

49321 gcagcttaat ggtttagaat cagctgatag tattagtatt ggtcaatcaa ttaaacttaa
49381 ataattaaaa aacattatta tttacaattt agttaagaag tattatcatt tacttgtaaa
49441 aagtaaataa aaggatgttg tcttcagggc agggtgaaat tcccgaccgg cggttagagt
49501 ccgcgagcta cgatgtagtt gaaccttagg taccgatagt taaagtctag atggaagaag
49561 acagactaaa aaactaaaca tgatttagat taataaggtc tgttgctgag gcaacagacc
49621 ttttgtatgt tatggaggaa tcatcatgca aagaaaaact agtttaagtc atgtcagact
49681 tgcttattgg cttgcctggg caatgattgg tgcattggca tttttaatta tgaaaattga
49741 agtcccaatt attccaggct ttgattattt gaagatggac ttttcggatt cacttgttgc
49801 cttgagcacc ttagtttttg gacctttggg cggaacaatg attgcactct ttaagtcatt
49861 attgagtctt tttatttctg gttttaatcc aatttcaatg atcggacaac tagctgcgtt
49921 tttagcaagt ttagcttata tcttgccatt ttatttcatc agtaaaaaac atgaggataa
49981 agtaaaatat caaatttttg gtttaattgt cggtacacta gctttaacgg tagtaatgtc
50041 actagcaaat tattttgttc taactccaat gtatatttct ttaatgggat ttaagttaaa
50101 ctcatcactt ttaacttacg ttgtttctgc tattattcct ttcaacttaa ttaagggttt
50161 gattaacagt attgttgttt tgattttagc gaaaacttta ttacctgttc tagagaagtt
50221 tgtaagacgt aatttctaaa atattttcta aaagaattat gtttagccag aagtcgagtg
50281 acttctggtt tttgttttat ggtagaattt actttgaatg aaaaagaaag gactcaattt
50341 tttaatgcaa gtagctattg atggtcctgc atcagcgggg aagagtacag ttgctaaaat
50401 tatcgcgcat aatttaggct atatttatat cgatactggt gcaatgtatc gagcatgtac
50461 attaattgca cacgaaaatc acgttgacta tggtgatgaa aaggcgatct tagacctggt
50521 tgacaagagc acaattgaat ttaagcaaga agatggagaa cagaaggttt atgtaaatgg
50581 caaggatgta tcaattgcca ttagaactcc agaaattact gagaatgttt ctcaagtgtc
50641 agccttgaag tcaattcgtg aaaagatggt tgaattacag cgtgaaatgg ctggtaaaca
50701 taatgttatt atggatggcc gtgatattgg gacgacagtt ttaccagatg ctgaggtaaa
50761 aatcttctta atcgctagtg tggcatcaag agctaagagg cgctttttag attttcaaga
50821 aaaaggaatt caccaagact tgaaagatat tgaacatgat attgaagttc gcgactataa
50881 agattctcat cgtgaaattt ctccattaaa aaaagcagcg gatgcaattg aattagatac

50941 aacaaacctt actattgatg aggttgtagc taaaataagc gagataatac aaaaaaagca
51001 aaaaaattaa acaaaagtgt ggaaaaaaga catcaagtac tttaaaataa agtatgatat
51061 tgggaggtaa ttatgacaga taatagtaat caattcttag atgctctaaa agaaatgcag
51121 ggagttgaag tcggcaacat tgttaacgtt gaagttttaa gtgttgaaga tggacaaatc
51181 gctgttggtg ttgaaaatgc aggtgttgaa ggtgtaatca ctaaacgtga attcactaat
51241 gaccgtaacg cagatttgaa ccaattggta aaacctggtg ataagtttga agctttagtt
51301 cttagaagag ctggtggcga taaagaaaac ggcgaattct tcttctcagt tactcgtttg
51361 aaggaaagag aagcttacaa ggaattagaa aaggtctttg aagaaggcaa gactgttgaa
51421 ggtactgtaa ctggtgctgt tcgcggtggt ttattagttg acgttggtac acgtggtttc
51481 ttaccagcat cacttatttc taaccgttac gtttctgact tgaagcctta cattggcaag
51541 aagatgaact tgaagatcac tgaaattgat cctaacaaga acagattaat tctttcaaga
51601 aaagacttaa tcgaaaagga acgtgaagaa gcattcgaaa acgttgcttc acaattagtt
51661 gtaggtgata ctgttgaagg taaagtatca cgtttaactg gttttggtgc ctttgttgac
51721 gttggtggtg tggacggttt agttcacatt tcagaaattt cttacaagca tgttgacaag
51781 ccaagtgatg tattgaaggc tggtcaagat gttaaggtta aagttatcgg tattgatgac
51841 gatagacaca gaatctccct gtcaattaag caaactttac catccccatt tgaagaagct
51901 actgaaggtt tacatgaagg cgacatcatt gaaggtgaag ttaagacttt aacttcattc
51961 ggtgcattca tcgaagtagc tgatggaatc caaggtttag tacacgtttc agaaattgct
52021 aacaagcacg ttgataagcc aagtgatgtt ttaaaggttg gtcaaactgt taaagttaaa
52081 gttttgagcg ttgacccaag tgacagaaga atctcattgt caatcaagca agctgatcca
52141 aatgctgcta agagtgaaaa ctcacgtcca cgtcgtcgtc aagattctgt agctgataag
52201 tacatgaatg acaatgacaa cggttttgct ttaggtgaca ttattggtga tcaacttaaa
52261 gataaggatt aatcgtcatt cgtctaaaaa agctgactcc ggtcagcttt ttttatttct
52321 tagataaata gaaaggggta gaaataatat gtcattaccc atcgttgcat tagttggacg
52381 accaaatgtt ggtaaatcaa ctatttttaa tagaattatt aattctcgcg tagctattgt
52441 cgaggataaa gctggcgtca ctagagatag aatttatgct cgtgctgaat ggatgggcca
52501 tgaatttatc ctaattgata ctggtggtat tactttagac tccggtgaaa tcgaagagca

52561 aattaaggct caagctgaaa ttgcgattga tgaagctgat gtaatcgtca tgttgggggga
52621 tgtgacccag cacatgacta acatggatga aacaattgct aagatgcttt atcgaactaa
52681 gaagccaatt attttagcag ttaacaaggc tgataatcct gaacaaagaa ctgatattta
52741 tgatttctat agtttaggtt tgggtgatcc aattcctgtt tctggtagtc acggaactgg
52801 tatgggtgat ttacttgatg caattgttgg tgaattcggc gataaggcca atcaacatga
52861 agatgattcg attcgcttta gtgtaattgg tcgtcctaat gtaggaaaat cttcactggt
52921 aaatgctatt ttaggtgaac aacgtgtaat tgtttctaac atcgaaggga caacgagaga
52981 cgccattgac acgactttta ctaatgatgg gcaaaagtat actattgtcg atactgccgg
53041 aattagacgc cgtggcaagg tctacgagaa gactgaaaag tattcagttc taagagcaat
53101 tagtgctatt gaagaaagtg atattacact tttagtttta gacgctagca caggcattcg
53161 tgaacaagat aagcacgttg ctggatatgc tcacgacgca ggccgaggag taattattgt
53221 cgtaaataag tgggatcttc ctaaaaaaga tagcacaagc atgaaagact ttgaaaatac
53281 aattagacag gagttccaat acttggacta cgctccaatt gttttcgttt cggcaaagac
53341 tggtcaaaga gttcctgata ttttgaaact agttaaagaa gttcacgata atcaaactcg
53401 tcgcattaag tctagtgtct tgaatgactt gctacttgag gcaaccagaa ttacgccaac
53461 gccacttgtt aacggaaaga gattaagaat ctactacatg acgcaagttg cagtaactcc
53521 gccgacattt gttgtttttg taaatgatcc agaattattg cacttctcat atcagagatt
53581 tttaatcaat caattgcgcc aaaactttga ttttgtagga acaccaatta aaatcttagc
53641 tagaaaacgt aaataagcct aaaatttcaa aaaaataggg atatatctcc gaaaaagctt
53701 gcaactaagc gtcttagtgt gctaatttta ttgcagggaa taaatgaata aatcattttt
53761 cttatttttt aaaggttttg tttaaaatac tattagttca tttcgggagg tgaagtggaa
53821 tggcaaacaa ggcagaatta gtttctgaag ttgctgctaa aactgactta accaagaaag
53881 aagttgctgc tgcagttgat gcaatcttta gctctattca agaagatctt gctaaaggtg
53941 aaaaagttca attgatcggt ttcggtactt ttgaagtacg tcaccgtgca gctcgtaagg
54001 gtcgtaaccc acaaactggg gctgaaatcg aaatcccagc ttctaaggtt cctgcattca
54061 agccaggtaa ggctcttaag gatgctgtta agtaattgtt cagttactag caaaataaaa
54121 acgtgctaag ttgtatagct tggcacgttt tttgtgtaca attattgtgt tagcacaaat

54181 gaaaagagtg aaaaaatgac ttattctgaa caactactag atgcaattca aaaccatgac
54241 ttttctgata atcatattct tcttaaaaaa gcacttgaaa atgatgatcc ggaagtttta
54301 gcttctttag ctgaaaactt aactgatctt ggttttactg atctttctaa ggaagtttac
54361 cgctatttga ttgctcagtt tccaagagaa gatttgttta aggtatactt agctgaaatt
54421 ttattaaatg atggcgatga agatgatggt ttgcagcttc tatatgatgt aaagccggaa
54481 agtgatgctt atatcgagag cttgcttgat ttagctgatt attaccaaag caatggtctg
54541 attgaaacag cacggcaaaa actcttagaa gctcataaat tagctcctga agaagatgca
54601 attaactttg gcttagctga acttgactac ttaagtggcg attatggtga agcattagga
54661 ctataccgag agcttgcgaa agaaaacaag acctttggcg aagtagtcct aagtcaaaga
54721 attgctgctt gccttgccaa attaggtgag tacgaagaag cggccaacga gattaagagt
54781 catgaaaatg atattttgag cattgatgca ttatatgaag caggtttaat tatgttatct
54841 gcgggcgata ataaggcagc aattaagtac ttagatcaag taatagaaac tcaacctgac
54901 tatgtaaatg cctatccgtt actcgctcaa gcttatgcgg cagaagataa taatgaagaa
54961 gtattgagaa ctgctcagac gggcctttct tataatgagc tagatgaggt cttatatagc
55021 ttaggtgcta aagcagctgc taacttaaat cagttagacg aagcagaacg cttgcttaag
55081 aaggggctag agattgctcc agacaatagt gacttacgtt tgcagttgtc taacttgtat
55141 ctccaccagc accaagatga agctaatatc gccttattta aggaccttga tgatgaagaa
55201 cttgaaccac aagctcactg gaatatggct atctcatatc aaagacttga agactatgat
55261 aaggctaaga gtgaattctt actggcatat ccagcctttc aaaagaatgc aagcttctta
55321 agacagatga ttaatttgtt ctatgagttg cgtgagatcc caacaactaa ggaattgatc
55381 aagaagtatt tgcaagttca acctgacgat attgatatgc aggatatgtt agatgaattg
55441 aatagtgaag aataatagag cgactattta gaaagtaatt gcttttgatc gtaaagaata
55501 cccctcaaag ataatttgag gggtattttt ttatagtaat ttatccaatt gtttgttaaa
55561 tcttctcaga cttagcttaa gtttaaaaat tttaaagata cttaattgta ttttttgtaa
55621 tttgtttata tttctctgca tattagcgat cgtgcgatta caattattac ggctatattt
55681 ttgacttaag tttttgatat aagctttggg taatttgact ccataaattt taatcataga
55741 tttatttagt gaaactaaga ttctgacaag tttgcgattg aattcttctt gataagtttt

55801 atttttgct ttagttaatt tggctcttat agtattaaag ttatttcttt
ttggatattg
55861 gtaagaatca gaatctagct tattttttag aaatttcttg aaagtcgtta
aatcttttt
55921 agtaggacct ttcatagtaa gacttactaa accatataat ttcaagaaag
tatcttgatt
55981 ttgaaaagct agagtatcaa tcattgatgt aacgttaaaa ggaatgagag
tagtattgtc
56041 atcatttgca gcatattttt cttgcttgcg ataagcacca acgaacatat
ctgccatttg
56101 aatgcctaat tcttgtttac tgtcgcatgt ttgataagta aaaaatacgt
tgttttcctc
56161 gcctagattt ttgataattt gagaaagtaa cttgcagcta aaatattcac
tttcaagctg
56221 agaatttaga ttagtttgat caaggtaaat atttaatttt aagttaacaa
caggattttg
56281 ctgacgtaga tcttcaataa ttctcttaat tgggtaaatg taagattgaa
gcattaatac
56341 ttgttgcata atagtattat caagagtggt atcgtgctta tgataagtag
ggcgttggta
56401 aatataagca ttggttagaa aaggcttaac aatatctaac gctaatcgat
tcatcgtgtc
56461 tgaaacattt acactctttt tctcttttt ataacgagaa tgataaattt
tttgaaaata
56521 tctattatat aaatctttta gttgaaaggt ggggatgact aaaaagcctg
aatagaactt
56581 ttcagttgta ttgtcagatt gagatttaaa agaaatgctt tcatcaataa
aaatatgaaa
56641 ttctttgata tgacggcgtg gttggaagag tttgtttaag atcatcattt
ttctccaagt
56701 aaatttgata gaagtattat atcgaacata agttctgaaa gaaagcgaac
aaggtaaaat
56761 atttttgaaa ttgtattttt ttatcatatt ttttattatg taaaactgct
gaacatatat
56821 tcttaatgaa cttgttgtta aatgggcatt cagggtaaaa tagaaacaga
gaatatgaag
56881 aatgaggatt gtaggatgtc taattttgag tttttaaata aagacgtttt
aactgagcaa
56941 tactatcaaa aagctgctga ggcagagatg agttatgcaa tggggcttta
ttccaacgtc
57001 ttggtttctg cacgaactat agcagaaaat ttggcacgag aagttgcaga
tgaaaattat
57061 ttagtagttg atgatcgcga aacatttgat aatgtattaa aacgcttaaa
gctaggtcaa
57121 tatattgata agtatgcttt gcagtttttt tatgacgtga aaaatgtcgg
taatactgca
57181 gctcatactt taacaactag taatcaggaa gaagcactaa cggcgttaaa
gagactctat
57241 tctttgtgcg tttggtttgt tggaaattat tatgatgaaa aagtcgatat
cactgaattt
57301 caagaaccaa aaaaagatgt tttgtatcaa acgactattc aacccatctc
aaatgccgag
57361 aagaatttga tttatattca aactgcagac aattcattgg gaaaattttc
tgtttatgaa

57421 ggaaatcaaa aggtaggaaa aactggtgtc ggtgatttag ctgaagataa
ttctgacaat
57481 agtccttatt tacgtgattg ggccaaaaaa agaattaagc agtacatgaa
cacatctggc
57541 gttcctgtaa atttagaatg ggctgaatta gcttaccgta aatcagatgg
tctatggttt
57601 tctgatcatg atgttcatcg tgtcttagaa agatccgggg ttaagcatag
tcacgatttg
57661 tctggtgatg aatggtttga aacagattta gaaacagcta aaaaagctat
taaagcagtt
57721 aaagaaggaa aagatagcct tgataagatt agtaatgtag aagaaaagat
tgttttacgc
57781 ccagagcagc aagaagctgt taataagact aaattaggct ttaaatcagg
acaaaagatg
57841 ctatggaatg caaagatgcg ttttggaaag actttgactg cacttaagct
tgttagagag
57901 caaaaatata aaaaagtgtt aattatgacg caccggccag ttgtagatgg
tggctggttt
57961 gacgacttta tcaagatcgg aatgcctgat gctgggtatg tctatggttc
taaaaataag
58021 ggacatcaaa atattaaaga acttgaagat cttaataaac catatgtata
ttttgctagt
58081 atccaagact taggtggctc tgaaatagta ggtggtaaag tctctgataa
aaatagagat
58141 ttatttgctg tagattggga tttagtaatt gttgatgagg ctcatgaagg
aacgcagact
58201 gaattaacac agaatatttt aaatttagta gttaagaaac atactaagga
attagattta
58261 tctggtactc cgtttaacat cttgtctgac tatgacgatg aacacgtctt
tacttgggat
58321 tatactatgg agcaagaagc taaagaaaat tggaataagg aacatccaca
tgaagataat
58381 ccatattctg cacttcctag ggtatcaatg tttacctttg aaatgaataa
acgtttcaat
58441 gatccccgct ttaccagaga aggattagga aagtatacat tcaacttcaa
agaatttttt
58501 agaacagata aagatggcaa atttgtgtat gaaagtgatg ttaagagatt
tttagataat
58561 attacgaacc ctggaacagc taattatcca ttttcaacta gagaatttag
aaacagatta
58621 cgtcatacct tatgggtttt acctggaatt aaggaagcta atgctttaga
agaattgttg
58681 aagaagcatc cagtttttgg aacggaatac aacatcatta atgtggttag
aaatgaccga
58741 tcggatactg aattgggtac tgataatgat gttgatgctg agagaaaaga
gatagcaaaa
58801 gcggataaag ccggcaagaa gactgtaacc cttactgtac ggaaactgac
gacaggagca
58861 actgttccag aatggactgg tgttttattc ttatctaata ctaatagcgc
tatgcagtat
58921 ttgcaggccg catttagagc acaaacgccg tattcatctc caaagtttgg
taagaaagaa
58981 aattgttata tttttgactt tgcacctgat cgtgctttaa cagtaatggc
agaatcaacg

59041 agtttagcaa caggtgttgg taaaattcaa actaaagaac aaagagctaa
gatggcaaaa
59101 ttgatgaact ttttaccaat tctcggtgag agtgatcaac aaatgaagcc
atataaagta
59161 gatagattgt tatctcaatt aaaacgagct tatgcagaaa aagcagttcg
tactgggttt
59221 gatgatgatt ctatttatag tgatgagctt ttattaatta aagatgctga
cttgaaagat
59281 tttaatgatc ttaagggaat tattggtaca acaaagaaag aaaaagctcc
aactaaaatt
59341 gatgttaacc atcaaggttt agatgaagaa gaatatgaca tggctgaaaa
ggctagaaag
59401 aaaaaaacga gagaaagatc tcaagaagag caagatgcca ttgataaagt
aaagaaatta
59461 aagaaacaac gtaaaacaat gatttctatt cttcgtggaa tttctatcag
aattcctatg
59521 atgatttatg gaatggatat taagtttgat gaaaatgttt caattaaaaa
atttgtagat
59581 aaaattgatg acgtatcttg ggaagaattt atgcctaagg gtatcaccaa
agctttattt
59641 aataaatttg ttaaatatta cgatgctgac gtatttattg aagcaggtaa
aattattaga
59701 cgacaagtta aggaattaga taaagcagat cctcttgagc gagttgaaaa
aattgctgaa
59761 attttcaata cctttaaaaa ccctgataaa gaaactgttc ttactccttg
gcgtgtcgtt
59821 aacatgcatt tgggcaaaac cattggcgga ctatcatttt ttgataaaga
ttatcaatat
59881 tcatacgaag atggtaatgt tgttagaaga tggatcgata cagaatacac
tgataagatt
59941 tttaatgaaa attcacaaat cttagaaatc aatttgaaaa caggcttata
tccgttgtat
60001 gtcgctactt caatttattg gaaagaattc aataagttaa atgatcaaac
tgcagggaaa
60061 tttggttttg aagacgaatt gatgatttgg caaagaattt taagagaaaa
tattttctta
60121 gttgccaaga caccaatggc gaaggaaatt gctagaagaa ctttggctgg
atatcacgat
60181 gattgggata ttaattcaga atatgtagag aacatagtgg aagactcaaa
agccgatgtt
60241 aataaagaag ctaagaagat tgaagggatg tttggaaata tgaaatttga
tgttgtaatt
60301 ggtaatccgc catatcaaca agaagctaaa ggtgcaagta caagagatga
accgatctac
60361 ccacagttta tggatttgtc atatcagtta tctaatttag taactcttat
tactccggca
60421 agatttcttt ttaatgctgg tcaaacttct aaggcttgga atgaaactat
gcttcatgat
60481 ccacatctga aagttgtatt ttacgaacaa aatagtagca aagtttttcc
aaatactgat
60541 attaagggcg gaatagctat tactttaaga gatgctacta aagattttgg
agaaattgga
60601 atatttacat ctcatcctga aatggaaagt attgttaata aagtatggaa
atataaacaa

60661 catagcttta gtaaaatggt atcttcatca ggcctttata gattttctaa tttagtgttt
60721 gaagagaata ctaaattaaa aaaatctgtt agttctggta ctggaaagaa aatcacctct
60781 aaaattatga gtgaaaataa tgatattttt agaaaagata gagtattaga tactgatgtt
60841 agtttaatag gtttggtgag aaataggcgt acacatagat ttattttaag aaaatatttg
60901 caagataatg attttattga taaatataaa gtattaatcc ctaaagctaa tggtagtggt
60961 aagcttggag aaattatcag tacaccaatt attggaaaac cctctatagg tgtaacggat
61021 acttttataa gcttaggacc tgttgattct ttagaggatg ctgaagcaat attaaaatat
61081 ataaagtcta aatttggaag agcattatta ggtgtaagaa aaattacaca agataatcct
61141 aaatctacat ggcaatatgt tccaatgcag aactttacat ctgattccga tattgattgg
61201 acaaagagta tatctgaaat tgatcagcaa ttgtacaaaa aatataatct ttcagatgat
61261 gagattaact ttattgaaac taaagtacag gagatggatt aatgctgaat attgtagatc
61321 aaatgtttca gtattgtgaa atacctgaag acggacaaca atattttttt aatgattcaa
61381 ttgaaaataa aaaaattata gtatcagcct ttaaacacaa ttcatttatg tctaaatata
61441 tctatatgcc gatattaagt ttgaaatttg tgtttaatac ggaaaagcat aaaagaattt
61501 atcactattt aaatcatgaa aatttaacat ctattttaaa gtgtcagtat ttttatgtag
61561 ggaatcaaca agaaatgaat gattctttag agggtaaata ttcttgggac ttagcatttg
61621 acttacttaa tgagaaggat aactcaaatc aaatacaaaa aatgtttgag gaaataaaaa
61681 ggagacgtcc ttttgatagt tatatttggt cgtttacttt agatgaaaat aatatggctt
61741 tgcaaaatta tggtgacctt gcagtagtaa tgaatccaca ggatacattt gaaagtttag
61801 ctagaaaata tactaattca gactttgaaa atatgaatac tgggaatgcg tatattttac
61861 ctttacatgt agtttatgat taccaaacgc aagtaaagta tttatcaaat accttagata
61921 tatggttgga ttctataaac agtggtaatt atggtgatta tcaagatgct tcattagcaa
61981 tgtatttata ttctttggta tttaagagcc caaaatatca tcaagaggaa gagattcgat
62041 atgttattaa taaaattcca gatattaatg gaaatggata tgacataaaa attaatggaa
62101 aaaagaaact agaagcacca tttctcccct ctttatgtag tgaaattata gttaatcaca
62161 aagctgatca cgtaaaaaat gaaagtattg atacaataca gaccaaagtg aaatctatga
62221 ttgagaatta tggctttaat aatactatcg taagaaaaac ggatctagaa tactaattat

62281 gtcagaaaaa ataattaaat catctgatcg agtaaaagat ataggtgaag
tctttacacc
62341 taaaaaaact gtaaatttta tgcttgatca acttgaaatt aaagaaaaaa
taaattcatt
62401 gactgcaact tttttagagc catcagcagg tgaaggtgcg tttttagttg
aaatattaag
62461 acgcaaacta aaatatgcaa agacacaaag cagttctgtt gaagaaatgc
aaaataatta
62521 tttgcaggtt ctatctactt tatatggtat tgaacttatg gaagataata
ttgaaatgtt
62581 agttatgaat atgaataatg tttttcgtga tatgtatttc aatacattta
aagcagagga
62641 tcagaatcta aaaatcttga aaagtgcgca gactattatt tcagcaaata
tgtctcaagg
62701 ggatgcattg acaagaaaga cagcaacagg aaagccaatt atctttagtg
aatggaaacc
62761 ggtaggaaag aataaagttc aaagaacaga gtatacattt gattctattg
ttgaaggtgg
62821 tggaccattg ggttctgtgc agaaacagta tgaacaaatg gatctttttg
ctgaagataa
62881 ttctgaaagt aatgatgaaa atgttcgaca ttatttgcct gttaaatggg
aagatattta
62941 taagagatta gttgaatagt gaatttattc ctaaaaggca tcgatactac
cacgtttggt
63001 gaaaaattac ttaaataaca cagtttaaaa cttagaaagt ttaatattaa
taaaggaaac
63061 aattgtgaaa gaaaaggctt atcaagcaag taataatata tggttatcat
cctttacgat
63121 acaatttctt ataaatttct attttcaaaa gtatgatagt gatacttatt
tgcgacctaa
63181 ttatcatccc ttattatata tagatatagc agaattagta atctcaatag
tattgtctat
63241 gggtaccgtt atctttgatt attttaaagg taaaactatt aatttaacaa
ttaaaaaatt
63301 ttatatggag tctgacattg aaaacgatat atttcgatta gtctgtattt
ttgaattatt
63361 aggttttatt tatattcttc ttcatgatat gtgctatttg aaattaataa
acagaacaac
63421 tttttacaat ttatttggat tatttttgat agtaattgta ctttactttt
attatttatc
63481 tgacaaaatt aaaaagatgg aatggagttc tttgagtggt aatagcaagg
ttagttggaa
63541 atacttaata caaaataatc attttgatca gtataaagga gaaataattc
tatctcaatt
63601 attaaaattg ttaagagtag aaaatgtaga aataaaagtg tcagcaaatg
attactttaa
63661 tatttataaa aaatttgttg aaaaattgca atttctttct aatgaaaagt
tgatagaatt
63721 acgtaactat ttacagatgc aaaatagaga tgattttaaa tggtcaattc
agaaaatttt
63781 gtctgctaat ttgggcaaag tatgtatttc attacttgtt tctatgcttt
caatttcatc
63841 tatagtgcaa ttagggtcaa atataaaaaa agaaatgata ctcaatggaa
tattatcgat

63901 tatgcattac tatatacttt tttacgtttc gcttttatta atcttttatt taattaaact
63961 actaatttat ttagttttta ttccacatca aagaaaggtt agaaatgaaa atatagaatc
64021 gtatttgtta tcagcaattc agactgtttt agatttacga tctaaaagat agtaatggag
64081 ttataggtat tttatcgtag taataaccac aacaaaaaga gcaagacttg atcaacaact
64141 gaatcaaatc ttgctctttt tcttatttat ttaattcttt aaagtggtcg tggattagga
64201 taaatccaat ccaagctacg aagcaaaggg tgaagacggc aattagacct gtatagtgct
64261 aaatttctaa atgggtgtgc ttaatatttg ctgtttcaac atagctaagt ccttgcatca
64321 tatatatggc tggagcgata taccagtaag cagccggata gtctgaactg atccggttct
64381 ttatatctgc ttttgaaatg tataaagcaa agatttgcat aaatacgtat acacgaagaa
64441 ctaaccaagg aaattagaga tagggatgtt aaagaatgta ccatcatctt cccaacctca
64501 gtttccgttt aaagtagaag cagttggatc taatacgaca tcccacatta ccatgataaa
64561 agttgctgtg aatggaatta gaaatgtttt gataccagat aatttctttt catattggtt
64621 gttaattaca agcgacaaaa tccaagtaaa gtaacccatt ccaaagtaag ctggcataat
64681 aacgattgga acatcccata ttcttggacc cgcaatatca tagtgataat tcccaaatgg
64741 gaaacctgta tgaatactca aaccttaaaa gaaattgctg acaacccagc agataataaa
64801 gaataagtaa taaattaaat aacacaatca taacttaacg ctgctaatta ttatacaaag
64861 ctgtagtcaa cacttataat attccttgta agcgataaca aaaggcgagg aatataatta
64921 tggaacaaga aacaaaagct ccagttatta actggaaagc aaattatgat gttgttgtat
64981 taggctttgg tggagcaggc gcaactgctg ctagatttgc agctgataac ggcgctaaag
65041 tattaattgc tgatgttgct ccttacggtc atgaaggcgg gaacactaga tattctgccc
65101 aactccttgg cacgggaact gactatgaca agttgaagga atactatctt ggcttaactc
65161 atcctatgga tttgccagaa gatatggttg aaacttatgt ccgcggtatg tgcaatatgc
65221 gggagtatgt aaagaagtac ttgggcgttg aaccagttag tttcatcaag gactttagca
65281 agataccaaa ggaataccgt atcggagtta ctcaagaata tcctgaacta aagggacatg
65341 aaacttatga ctataccttg gttcacaatg gctggttcga cgcagcactt tggaaacttt
65401 taagacaaaa agttctagat cgcaaagaca agattgatgt ctggtatgaa gcacgtgcta
65461 gtcacttgat tcaagatcca atttctaaag taatcaaggg cgttcaaatt gaaaaattag

65521 gtcaaaagat caatgtcttg gctaggcggg gcatcatttt gacgacgggt ggttttgaaa
65581 ataataagga aatgattcag aactatttag ctgcacctaa acttgcccca ttaggtacta
65641 tttacaataa gggtgacggt atttcgatgg cgcaagaagt cggcgctaag atgtggcaca
65701 tgtataatta cgagtcgctt ggcttgttgc atggtttatc atttgacacg ccagaaggcg
65761 agagatccaa gttagtcctc agctggccag agcttaacca tggcagcgtc tttatggtgg
65821 cggacgatgg caagcgttac tttaacgaag ctggtccaaa tcgtcacggc catattttcg
65881 atcatggtat ttggcgcgtt ccacgtagtc acgagaagcc atatattatt tttgatcaaa
65941 aacaatacga aaccattgaa gcagggcaga ttccatacga taagttcaat gaaaaattga
66001 ttaaggctga tacgcttgaa gagctggcta aaaagacagg cgttgatgca aacggcttaa
66061 aggaacaagt cgagatgttt aaccgttttg tagaaaatgg tcacgatgat caatttggtc
66121 gagatattaa atcaatggct aaatttgcgg aagatggtcc attttatgca attaagatgg
66181 catataacgt tttgaatacg cagggtggac cggaaagaaa cactaaagct gaaattcttg
66241 atgttaataa taagccaatt ccgcacttgt atggtgctgg cgaattgggt ggtattaatg
66301 caaaccagta tcaaggtggc ggtaacttag ctgaatgttt gatttttggt aaaattgccg
66361 gtgagcaagc agctaatgtc actgatgagg ttacagacga agcttctgat aagtacaacg
66421 gcattaatga attagtagat ggtgacaagg tcaaggacat taagcttgat aaagatcagt
66481 atctcggcga gtctaatgct ggtattggtg gaaagcttgt tgtgcgcgtt acttacgata
66541 acggaatcaa aaatgtcgag atagtacaaa atcacgaaag tgaagatgtc ggcaagcgag
66601 cactaaaggt tattccagag cgtattgtta aagaaaatac cccagatgtt gatgcagttt
66661 ctggtgcatc tgctacaagt cgcgcaatta gcgaagccgt tcgcaaggct ttgaagaatg
66721 tgaaggaata aattaattta caactacaat gcggttgtaa aaacggtcga aaagtttttc
66781 gaccgttttt tggtataatt taaattaaaa attgtggcgc atttaaagat ggatatttaa
66841 ctgcactaaa aataacatat aacaaaaaat gtcagggaaa aagaaatgat tgattataga
66901 actttaaagc atagtgataa tggaatgccg acatgggatg cttttcttgg tcctgtattg
66961 caggtggcaa gtactaaaag agaatggata gggaaagaat tggatcaagc agtacttgat
67021 gaagttgatt tacctgaaga acttgctaat ttaagatatg catctaaata tcacgattta
67081 atcgcatata atcgtgtcgg atgggcctta agtgagttaa aattatcggg tttacttaat

67141 tcacctagaa gaagtatcta taggataagt gaaacaggaa aagaagcatt agctaaatac
67201 ggattgaata ttacacgtga tttcgttcat tctcagtcag catatattga acaccaaaat
67261 aagttgaaaa ataaaaagga aattgataat gatatcactg aagatcaaac ttttgaatta
67321 actgaaaagc aaatcaaaga ttggtttgat aaacaaagta gtgatttatc tgaacaatta
67381 attgctaaat tacgcagtac agatccttat caatttgaac atatgatggt ccggctgtta
67441 aatgagatgg gatataaagg gaccaatgga caatccgtcg taactcagaa atctaatgac
67501 ggtggcattg acggaattat taaccaagat ccattgggct tgcgtacggt atacgtacaa
67561 gttaaaagat attcagaaaa gaatattgta ggtagtccag aaattaatag cttctttggt
67621 gcacttagac gtaaacaagc ggatcgtggt gtgtttatca ctacttcttc ttttactaat
67681 gatgcgaggg atgctgcaaa acagttaaat attgccttag ttgatggtga gatgctgaca
67741 aacttgatga ttcaatacaa agttggtgta cagattcgtc aaaattatga attatataat
67801 atagatgacg atttctttga agaatagaga taacttatat gaaatatgaa agttacttag
67861 aaatgcctag agaaccaaaa tcaaaggata aaaagatttg gcaagattat tttaaaaaat
67921 ttcaacaaag atttatccat aatgaaagag tttttatgct ctatcgaacg acatcttttg
67981 agcagaaaag tattaaaaaa gtaaataatg aatataagta tctgttagaa aatgaattaa
68041 ttatattatc aaaagcagaa gagaagtatt ttagattatt taaagagcca tataaaatta
68101 gtcaacaaat aaaaaaagaa aagctccaat actggatacc ttatttgaca agagtagatt
68161 ttgttaatat gggtgcatat atgggaaatg atgtatcact aatattaatg gataataggt
68221 atttggtaat agaaggtcgt cttgataagg actataaggt cataaaagct atttcaaagc
68281 aaaagttaat taaaagttta atcaatttgg agattggata ctggtctgaa gtttatcatt
68341 cttctgaagc catagtagaa gatggttcaa gttggatatt aaaatttaaa tgtataaaga
68401 ataagaaata caaagaattt gtattttatg gcgataattg ttatccatat aattttgatg
68461 atttcgcgca aattgcatat ataaaagatt ttgtatacta ataggacaaa tatttgagta
68521 aatcctaagt agtatgatgt aaaattacga gcttgaatta tagctaattt gaatgaagca
68581 acaacttttg ctctttaaag tattgaagat ccttgatatc ataggattct tattttaact
68641 ctcgcaaaaa acgcgaactt tcgcgaactc tcactttgcg cgcgaaagtt cgcgagagtt
68701 aaaaaatggc aacagattag tagaaatctg ttgccatttt aattagttta atagccaatc

68761 aattaaatta agtttttgga taccattata atttgctgtg cggttgatag tgtctaaagt
68821 taacagatat ttagggtaag aatcacgaat ttgctctagc gatctcaatt cgcgttttaa
68881 aactttttca tccatagtgg ttaaagcaac ttgataatag gaaagctcat tcatatcgtt
68941 aatcccgaca aaatctacgt catatttatt gctttctcca acataaactg ctttattgcg
69001 tctaagtaac tctaaataaa caatattttc aataatatgt ccaaaatctt cttgatggtc
69061 aggcaggaga atattcctaa ttcccatatc aacaccataa tatttactct gactatttag
69121 gagttgacga ccatggatat catatctttt tgcttcatac attaatagac tgtctagaat
69181 accgcctaag tatcgatcaa ccgttttgtt gctagaggaa tacccagcac ttttgagagt
69241 aatagttatc ttattaatag aaacctgact gccgattgaa gagtagaggt tttgaataac
69301 tcgctccaaa actggtgtgt ctttaatatt taaacgagaa acaatatcag ttaagacaat
69361 tgtagaataa acgccttgaa gatattcatt tctttctttt ggagtatcaa taaataaggt
69421 ataaggaaaa gaactttcaa gatatttatt gaacatgtca tcattattat taaacttatc
69481 gttctgttta tgccaagcaa caaattcaga aaaagataat ggcaagattt taagttctac
69541 atatctacca gtaagtagtg tagcaatttc actgcttaag aaatatgcat tagaaccagt
69601 taaatagata tcgacattat cttttaagaa gagtgaatca acaactcttt caaattcttt
69661 aacgtgttgg atttcgtcaa gaaaaatata attcatttga tcaggtatga ttttattctc
69721 aagataatca tataaatttt gagcattgcg aagattatca tatttcatat tttcaaaatt
69781 gatactaatg aattgttctt ctttgactcc ttcatgttcc tttaaataat tacgaaatag
69841 atagaagaga gtagattttc cagcacgacg tacaccagat actactttaa taatttgttt
69901 attttgccat ctttttaaga agtccaaata ttcaggacga ttaacatatt tttgcatttt
69961 ttctccatag taaacgattg gcatatataa agtatgtata tttataaata tattttagca
70021 aaagtttgga gttaactcca aactttgaac ttttttagca agtttttgga gttaactcca
70081 aattatctat attttttact tatttttgga gttaagtaca aaaaaagaaa cctaaccaca
70141 atggctaggt tatttcttac tatcttctaa agaaaatatg tctctttttt ctttcataag
70201 taaagccttg gcctaatgct tcatgtacat caattacgga aacaaaggca ttgggatctt
70261 cacgcaaaat taattctttg acctggctaa tttctctagg agaaaccaca caataaatca
70321 ttttcttagc ggagtgatca tagccacctt cggcatttaa aaatgtatag ccgcgatcta

70381 gctgcacatc aatcatctta gcaatttgtt catacttatc agaaataata aaaagtccac
70441 gtgctgcgta agcgcctgct tggattgaat cagtaattcg cgctaataca aaactagcaa
70501 gcaacgtgta aatcatgtgc ttaatgtcta ggtaagtgag ggagattgtc aaaacaaaag
70561 catcaagacc aagaagcgac ttcccaacag gtatacctaa ttttaaatct aatatcctag
70621 caataatatc ggttccgcca gtggtgccgc catatttaaa gacaatcccg atgccaaagc
70681 cagaaaaaag tcctgccaag acacctgcaa taaaaagatc atgttctaaa ttcaattggt
70741 ttataatagg aatgtgtaac caaaatgata aaaagaagga tagacaaagc gtgccccaaa
70801 tggtcaaagc aagtagccgt ttgcccataa aacgatatcc gattataatt aaggggatat
70861 ttagaagtaa cgttgataga ccaggattga tatggaacca atatctaagt aaaagcgcaa
70921 taccacttat tccaccatca gctagtttgt tagggatact gatggcatct aagctaaaag
70981 cataaatact acaaccaaga gttatgaaaa ataattgttt aaagagttgt ttattgaaaa
71041 tcttagtcat gttacttcct tctttcttac aaaactaagg aataattaag gggaataggg
71101 aatatatgaa gattaccaat ttaccggaag tttttaccgc cgcgcttcca gttttaaaac
71161 aaattaatga agcgggttat gaggcttatt ttgttggagg cagtgttaga gacttgctct
71221 taaatcgtca tattcatgat gttgatattg caacaagtgc ttatccgatg gaaattaagc
71281 aaatttttaa aaagacaatt gatacgggaa tcaaacatgg aacagtgaca gttctttatg
71341 aaggtgaaag ttacgagatt acaactttta gaacggaatc aggataccaa gacttccgca
71401 ggcccgatca cgtaactttt gttcagaact tatcagaaga cttgaagaga agagacttca
71461 cgattaatgc tctagccatg gatgttgctg gcaatgtcat tgatcacttc gatggcttag
71521 gcgatttaga caagcatcta atccgtgcag ttggtaaagc tgagaaccgt ttccacgaag
71581 atgctttaag aatgatgcgg gcagttcgtt ttatgagtca gttgcaattt acgcttgaac
71641 cagaaactga gcaagcaatt agcgataatc atgaattgct cagtaagatt tctgttgaac
71701 gaattcgcga cgagtttgtc aaaatgggga ttgctcccgg tagccaaaag gcttttcaga
71761 ttttcttaga tactggttta tctgaagaag ttccaggttt taaaggtaaa aaagagaatt
71821 tggctcttta tcctcaactc aattttagtc ctactactga agctaatcta tgggctttga
71881 tgattatttt actaaaatta ccaaatgaga agattccttc ttttatgcgg atgtggaaga
71941 attctaatgc aatggagcgt caagtagctg atattgtcgc tttctttgac ttaattagtt

72001 cacgtgctcc aaacaactat gatttatatc aagctggctt agaaactatc gcttcgacca
72061 ttgatttggc gcatatttta ggtcagccaa ttaacggcag tgctttggtt gatggttatg
72121 aagctttgcc aattaaaaat agccatgatc tagtgattga tggccacttt ttattaaaaa
72181 atggtattcc agctggacca agagtgggtc tactactaga agaaattaaa aaggcagtcc
72241 ttgaaggagt aattagcaac aacgaagcag caattactga atttctttct ttaaataatt
72301 aaaaatagct agcaaaaagg cgagcagtga aaaatcacta cttgcctttt taaacgtaaa
72361 ataaaattga aaaatacatc aagatcgatc ctagtaaaac aaacatgtgc caaatcacat
72421 gtatataagg aactccgcgc attgaaaata aaatagcccc gacagtataa gcaattccgc
72481 cccaaactag aagccaaaaa ccagttggtc caagaactgg ataaagggtg ctagaagcaa
72541 taataacaat ccatcccatt aatacgtata aaatcgtatc taaaataacg tgttttcctt
72601 gattgaaaat ataaaacaag atgccaaaga ttgctagagc taagataata ccgaacatta
72661 cccagccctt agcgccgccg attgcaacaa gagtataagg tgtataagtt ccagtaatta
72721 aaataaaaat tgaggagtga tcaaatatct ggaaaaccct tctagctcta gtaaaaatca
72781 aactgtggta gagtgttgaa aagagataga gaataatcaa gcttgaacca tagagagtaa
72841 aggcagttac tcttactgca cttcctgtat gggcagcttt tacaattaaa attactaatc
72901 cagctactgc taggcaaaaa ccaatcccgt gagtaatagc actaaaaata ttatttaaaa
72961 tgttgtatgt ttttgagcga ttttttggct cttgccacaa ttgtttaaaa gacattcatt
73021 gattccgcct tatatacttt tataatctat ttaataaatt ttaactcatt tttgtatttt
73081 tgatatactt gaggttgtac ttaataaatt ctaacataga ataataatta aagaagaata
73141 ggggatagga gctagcatgg ctaagattaa agtagtaact gattcctcag ttcaattaac
73201 tcctgaagaa attgataaat atggcattac cgtggttccg ttaaccatta ccattgatgg
73261 tcagacttac acagatggcg tcgacattag ccgcgaagaa tttgtgaaaa agatggatgc
73321 atcaaaagaa ttgcctaaaa ccagtcaacc atcaattgga gtttttgaaa aagtatttaa
73381 agatttgact gatgatggca gtcaagttgt tggtattttt cttgctcgct ctttaagtgg
73441 aacaattgag gctgctagac aagctgcgga tttgattggt aagagcaaag aagtaacttt
73501 aattgattct gacttaactg atcgagcaga agcataccaa gtactagctg cagctaaaga
73561 tgccaaagaa ggtaagagtc ttgaagaaat tgtcgctcac gttgagcaac ttaaagaaca

73621 tcaaaaactc tatatgatgg ttgtaaacct caataattta attaaaggag
gtcgtcttgg
73681 accgcttgcg ggtaagattg ctaccttatt aaatatccga attgaattgc
acatgccagg
73741 tggtcatctt gaagtcgcta aaaaaggacg cggtaagaaa ttttctaaaa
attgcgataa
73801 acgcgtttta aaagatatcg aagaacacaa gcaggaaatt aaagaagtag
caatttctta
73861 tgttgatact cctgaagata tgaaagtttg gactgaaaag attaaagaaa
tcaatcctga
73921 tatcaaggtt ttaactcgag taacgagtcc gattatttca actcacgcag
gtagcggagc
73981 atatgcagta ttctatacaa tggaggagta ttaatggctt atcgtgaaag
cttttatcgc
74041 tttttaatga cgcaaagaaa tccaggatcg gtagacgata ttgctcaatt
tgctaataat
74101 gctcaacacg atagttcttt tccaaaacaa gaagaagact atgaaaagtt
atcagactac
74161 ttagagttaa acgctggcta tttgccaagc atgagtgttt ttgacaaggc
atatcaatta
74221 tatttagaca atatgaactg aggctaatat ggcagaagta gaacccaata
attctcatca
74281 aaagaagact acaaaaaata aaaagaaaca tcttcccaaa tatgcccaat
ttttgttaac
74341 tggtatagct ggtgcagcga ttggtgcagg tttgaccttt ggaattatgg
aagttaagga
74401 attaaagtcg cctttttatc aagttgaaaa agtgtatgag caattacaag
gttcatatta
74461 taaaaaagtt tcatcgcaaa cattgcgcca aggggcaatt aacgggatgc
ttgatagtct
74521 taatgatcct ttttcagaag gattaagtgg cgcgaatcaa gaacaagtta
ataacatctt
74581 agaaggctca acttttggcg gagttggtat tcagatggca gtacgcaaca
ataaagttgt
74641 tgtggactct attgttgcta attctccagc ttcaaagagt actattaagc
caggtgatga
74701 aattcttgcc gttgacaata aaaaagtcag cgcagctcag ttcactaaag
tcgcttcctt
74761 agttagagga aaagttggca ccaaagtaac actcaaactc aagcgtgcta
attcaacctt
74821 taatgttact ttaaagcgcg ctaaaatttc gcagtctagc ttaactaaac
gtacagaagg
74881 taatgcgacc attattacaa ttacgcagtt tgacgtgaat actgctaagg
atttgaagag
74941 tgccttaaag tcaatcaata ctaaaaaata tcctaagtta attattgatt
tgcaggataa
75001 tccaggtggt gaaatgaatg ctgctctgaa atctgcatct tatttcttgc
caaacaagaa
75061 gattattatg caatacaagg accgcaagga aaaagaagta attcgctctg
ataagaatct
75121 ttcaggaggc tttcatactt cactcaagcc aattatttta attaatggaa
atacggctag
75181 tgcaagtgaa atctttacag ctgctttagt gcaaaatcat tgcggggttt
cagttggtca

75241 aacgagctat ggtaaaggaa ctgttcagca agtaggacaa actgaggatt cagagtataa
75301 atatacagtt gctaaatggt tgacgccaaa tggaacttgg attaatcaaa aggggcttaa
75361 gccaacttat ccagtttctg aatcaccttt agcaaaattg ccgcaatttc aaagtatgag
75421 tgttttgaag aagtccatga ctggtgttga tgtggctacc ttgcagcaat acttaactgc
75481 cttaggctat ttaccaaagc atgttactgg cgtttttgat gatgaaacca agaatgctgt
75541 aattaaattt caaaaagaac ataacttgac aacagatggt atagtaaatg ggcaagtaca
75601 agctcagctt tatctagcag tcgctcaaaa attacaagat gataatcctg ctttaaagaa
75661 agcgttgagc ttaaatctaa aggatatgga ggactaaaat ggcaactatt caatggtatc
75721 caggacatat gaacaaggct aaaaatcaac ttgaagacaa tttagacttg attgacgtct
75781 taattgaagt tttagatgcg cgtcttccag tttcttctcg taatccaatg attggacaat
75841 taaccaagaa gaaacctcat attattatct tgaataagtc agatttagct gacccaattc
75901 agactaaaaa atggactcgt tatttccaag atgagggcaa tttcgtcatc tcaatggatg
75961 ctcagcataa tactaatatg acgagtttgt ttaagattat taaattagct ggtaagaaaa
76021 agacagagaa gttgattgca aagggtgctt ctaatccgat gatccgcgta gcgattgctg
76081 gaattccaaa ttgcggcaaa tcgacgatta ttaaccggat ggttggaaga aatgcggcaa
76141 ttgtaggaga caagcctggt gtaactcgtg gtcaaagttg gttaaagacc aagacgaatg
76201 ttcaaattct agatacacca ggaattttat ggcctaaatt ctctgatcaa gaggtaggtt
76261 ataagttagc agcttgtggt gcaattaagg ccgatgtttt tcatcccgat gatgtcgcct
76321 tatttgtaat tgaatttttg aagcaaaact atcaaaaaga tcttgccaaa tttgcccaag
76381 caactagtga ggagcttgaa aatatgtcta atcctgattt attacttgct atgactaaca
76441 aatacggcat gcgggatgat tatgacaagt tttcactctt tatgcttcaa cgtttacgta
76501 aaggaaaatt gggaaggatt acgtttgatc ttagatgacc attactgaaa taaaaaactt
76561 gcttcaagga gaagtaagta gcgaacaatt agaagaatta cgtgctgatg aaagaaaagg
76621 cgtgcaaaag ctcttaatta gctacgaaaa aagacagctt aaacgtgcgc aagcacttgc
76681 tcagtttcaa aatcgttttt cttatgagaa agaattttgg caaaaaaatc aacttgttgc
76741 tggagttgac gaagtaggaa gaggaccact agctggacct gtagttacag ctgctgttat
76801 tttgccccat aattttgatt tgattgacgt taacgattca aagaaactat ctcctaaaaa

76861 gagaaaggcg ctttttcctc aaattctggc caaagcagtc agcgttagcg ttggcttagc
76921 taataatgat gtaattgatc ggattaacat ctatgaagct gaccgtctgg caatggcgca
76981 tgctgttcaa ggcttaaaag taaagccagc tgccttgtta gtcgatgcga tgaatgtacc
77041 gttgaatatt ccacaaatca aattgattca cggggatgct aagtctaatt caattgctgc
77101 tgctagtatt gtggctaaag ttttccgtga caatttgatg gatgcctatg gtgaagttta
77161 tccagagtat gattttaagc acaatgcagg atatggtaca cgtgagcaca tggaagcctt
77221 gaaaaagtat ggtccaacac caattcatcg tcgatctttt gcaccagtta gcgaatacga
77281 aaaataaata aaaaataaac cttcactttt gcgtacttga tagtggaggt tttattttat
77341 gaatttaaaa gaattttgct taagattaaa attgcaacat ggcgtcggta ctacaacttt
77401 gggtaaaatc gctgaaaatt ttacggctgg cgaagaagta acagttgaca aaatagagag
77461 tttatcatta aaatcaaata tccaaaatct tgttttagct gcgatgagaa atgataaatt
77521 taattcttgg attgaaagaa ttaaattgca gtgtgatgtt gtcactatct ttgattcaat
77581 ctatccagaa ggactccgcg agatgtacaa tgcaccgacg attcttttta ctagaggaga
77641 tttatccttg cttaaaaaag aaattacgac tatagttggc gcaagacaac ccacaaatta
77701 cagccggttt gttttaaaac agttaattcc gcagctaata gagcaagatt ttgtgattgc
77761 tagtggttta gctcgcggag ttgatgggat tgctcatcag gaaaccttga aaaataatgg
77821 taaaacaatt gccgtagttg gaaatggatt gaatcatttt tatccgcaag aaaataagga
77881 attgcaagaa gaaattgtgg caaaggggtt attaattagt gaatatttac cagatacacc
77941 cccgcgtcct tatcgctttc ctgaacgtaa tcgcattctt gcgggtttaa gtaaaaatat
78001 aattatcact gaagctagaa aaagatctgg ttctttaatt actgcaaata ttgctttaga
78061 agaaaatcgc aatatttttg cggttcctgg accagtctca agtcccttat ctgaggggcc
78121 taacgagcta attgctgctg gtgcatatcc actagttaat gctgatttta gaaacttgct
78181 ttagttgaca agagtgaaat ttttggtatt ttagaatttg gtagaaattg tgcaaggaag
78241 gggcatttta tgcctactaa acgaaaatat aaaaagaatt tagttattgt cgagtctcca
78301 cataaggcta agacaattga aaaatattta ggtagaaatt atcatgttat cgcctcaaaa
78361 ggtcatattc gcgatttgcc taagtcccaa atgggagttg acgtcgaaca cgattatgaa
78421 ccaaagtata tttctattcg tggaaaaggc gatacgatta aagaattaaa gagcgaagct

78481 aaaaaagcga agtatgttta tcttgcttct gaccccgatc gtgaaggaga agctattgct
78541 tggcacgtag ctcatgcttt aaacttagat cctaaagagc acaaccgcgt tgcttttaac
78601 gaaattacta aagatgcggt taaaaatgcc tttaagaatc ctagaaccat tgatatggat
78661 attgtcgatg ctcaacaagc acggcgtgtt cttgaccgct tggttggtta ttcaatcagc
78721 cctatcttgt ggcaaaaagt taagaagggc ctgtctgctg gacgtgttca atcaattgcc
78781 ttgaagttag taattgaccg tgaaaacgag attaagaatt ttaagccaga agaatactgg
78841 acaattgatg ctgattttga aaagggtaag gaaaaattca agggtgcctt ttatggcatt
78901 aagggtaaga agcaagactt accaaataat gaagcagttc aggatatctt aaaacagatt
78961 gataaacgta agaactttga agttaccaaa gtagttaaga aagaaagaag acgtcagcct
79021 gctgctcctt ttactacttc aactatgcag caagaagcca acaagcgtct aggatatcgt
79081 actcgtcgaa caatgagaat tgctcaatct ctttatgaag ggtcaacct tggtaaggga
79141 tcagttggtt tgatcactta tatgcgtact gattctaagc gtattgctaa tgttgctaag
79201 catgaagctt caaaatttat ccatgaagaa tatggtgcaa attatgcagc aatcaagccg
79261 caacacttta aaaacgatgc tgatgctcag gacgcccatg aagcaattcg tccaacttca
79321 gcttttagaa caccagcttc agttaaagaa tatctaacaa ctgaagaata tcgcctctac
79381 actttaattt ggtcaagatt tattgctagt caaatgacac cagccgttta cgacactgta
79441 agagcagata ttgaacaaaa tggtgtcacc tttagaacaa ctggatcaaa acttaagttt
79501 gctggcttta ctaaggttta cgataaccaa aaggaaaaga ataatgaatt gcctgagtta
79561 aacgaaggcg ataaggttaa gcttaagaag actgatgacc gtcagcactt tacccagcca
79621 cctgcaagat acactgaagc tagtttagtt agagctcttg aagaaaatgg cgttggtcgt
79681 ccatcaactt atgcaccaac aattgatacg attcaaaagc gttattacgt aaaacttgag
79741 ggaagatcaa ttgtgcctac tgaactaggt gaaattgttg ataaactaat cgaagaattt
79801 ttcccagata tagttaacgt tgactttact gctcaactag aagatgatct tgatggtgtt
79861 gaagtaggca aaaagaactg gattaaagta gtagatgaat actacaagcc attttctaaa
79921 gaattagaca aggctgatca acaaattgaa aaagttcaaa ttaaggatga accagctggc
79981 tttaattgcg acatttgtgg tgcaccgatg gtaattaaga tgggacgtta tggtaagttt
80041 tatgcttgct cacgcttccc ggattgccgt aatactaagc caattgttaa aaaggttggt

80101 gtaacttgtc ctaagtgcgg taagggagaa gttattgaaa agaagtctaa acgtaatcgc
80161 aagttctatg gttgctctcg ttatccagat tgtgattttg tatcttggga tcaaccaatt
80221 ggtcgtaatt gtccaaatga tggtcatttc ttagttcaaa agaagaacaa gaagggacta
80281 attattcttt gtccaaacgg cgattatcgt gaagaaccag aagaaaatta aattattata
80341 aaaatcaatg atatcattaa aacgaaagcg gtttaatgat atcattttta tatagacttg
80401 aaagagtcga gcagaaagga aagattgcat ggtaaaaaat gtaaccgtaa ttggcggagg
80461 ccttgcagga agtgaagctg catggcaatt ggctaagcgt ggcattgaag tagatttata
80521 tgaaatgcga ccaaagaaaa acacacctgc tcatgaaact gcaaattttg ccgaattagt
80581 atgtactaat tcaatgagat caaaccaact ttcaaacgcc gttggtttgt taaaagagga
80641 aatgcgtcag cttgattctt taattatgaa ggctgctgat gaaacagctg tgcctgcggg
80701 cggagcttta gcagttgatc gggataagtt tagttcagtg gttacaaaaa ctctaaagga
80761 cttgccgaac gttcatgttc atgaagaaga gattactgaa atcccaaaag atggaattac
80821 cattattgcg actggaccac tcacttcaga tacattggca gaacagatta aggaattttg
80881 tggtactgat tcacttcatt tctttgatgc ggctgcgcca attgtagctg caagctcaat
80941 tgatcgtgac attgtttata agaaatctcg ctatgacaag ggtgaagcag cttacttgaa
81001 ctgcccaatg actaaggaag aattctataa tttctataaa aacttagtta gtgcagaaac
81061 tgctacttta catggctttg aggataaaaa tgtctttgaa ggctgcatgc caattgaagt
81121 tatggcaaaa agaggagaaa aaacaatgct ctttgggcca cttaagcctg ttggcttaga
81181 agatcctaag acaggaaaga ctccatatgc ggttgtgcaa ctacggcaag ataatgcggc
81241 aagcacgatg tacaacattg ttggcttcca aactcattta aaatatggcg aacaaaagag
81301 agtcttttca atgattccag gtcttgaaaa tgcaaaattc gtccgttatg gtaaaatgca
81361 ccgcaacact tacattgcta gtcctgaagt tttaaatgca aattatgagg ctagaaaaca
81421 agccggccta ttttttgcgg gacaaatgac tggggtagaa ggttatgtag aaagtgcagg
81481 ctctgggtta gttgctggaa ttaatgcagc aagagaagcc ataggcgaag aaacattagt
81541 atttcctaag tcaacagctt taggatcgat ggctcattac atcacaacta ctagtgctaa
81601 gcacttccag ccaatgaatg catcttatgc attattacca aaactagatt ataaggttag
81661 aaataagcag gaaagacacc tggaaatttc taagcgagca ttgaaagatc ttgaagcctt

81721 taaggaagaa gagaagcttg attaataatg tctaaagaaa atgataagtt aatcaaacaa
81781 ttccaagatt acttgaacta tgaacgtaat tatagtaaaa atactgtaag ctcttactta
81841 aatgatctag atgaagcaaa gcaatttttt aaagaaaatg gtggctttag cggctgggat
81901 aaagttaaaa gtcgcgatgt tgaaattttc ttacaaaatt tagctacaca gaataggtct
81961 agaacaactc aagcgcgtaa gatgtcgagt ttgagatctt tttatcgctt tctcgttaag
82021 cgagaagtgt tagaaaatga tcccatgcag acaattagct taagattggg cgaaaagaaa
82081 ctaccacagt tttctacgaa aaaagagatg cgccaagtct ttgatagtct tgtggggcat
82141 gacagattgg tagtgagaaa tcgcgccatg tttgagcttt tttatgcgac tgggatgcgg
82201 ctaagtgaga tggcatcttt gaagctagat cagattgatt ttgatttaaa aattatctta
82261 gttcatggga agggtaataa agatcgctat gttcctttcg gaaaagatgc acttgatgct
82321 ttaaaggaat atcgtgatga tgttcgacca gctcttcttg gtcaaaatga agatttaggc
82381 tatgtatttt tgaataatcg cggacagaag ctaactgggc gcgggattga atacattatg
82441 cagcaagtgt tcattaaggc tggagttggc ggcaaggttc atccccacat gcttagacac
82501 tcttttgcaa ctgaaatgct caataatgga gcggacctga gaagtgtcca ggaattgttg
82561 ggacatgaat ctttatctac gacgcagatt tatactcatg ttacgatgaa gcatttacaa
82621 gcagattatc aaaaattttt cccaagaaaa gataagaaag acgaggcata atatatgaca
82681 acaatttgtt ctgttagata caacaatcaa accgccatcg caggtgatgg tcaagttact
82741 ttaggtgaaa aggtaatcgc taaggcaact gctaaaaaga tcagaagaat ttaccacgat
82801 caagtagtaa ttggctttgc cggtggagtt gcagatgcag ttagtttgca agatatgctt
82861 gaaggaaagc ttgaaactta ttcaggtgat ttacgtagag cagctgtaga aatggcacaa
82921 agctggagaa aagatccaac tttagctaaa ttgcaagcga tggtaattgc ctttaatgac
82981 aaagatttat tattgatttc tggtaatggt gaagttcttg aaccagacga agatgtggta
83041 gcaattggtt cgggtggtaa ctttgctcaa gcagccgcaa ttgcaatgac tcgccatagt
83101 agtggcatga gtgcgagtga aattgccaaa gaagcagtta agattgcttc tggaattgat
83161 gtctttactg atgaccacat tactactgat gaaatctagg tgaaatattt tgactgaaga
83221 aaaaactcca aaacaaattg tagaattact tgataaatat attattggcc aaaacgaagc
83281 taaaaaatct gttgctgtag ctttatataa tcgctaccgt cgtttgcagc tacctaagca

83341 gatgcaacaa gatattaccc caaagaatat gttaatggct ggtcctactg gtgttggtaa
83401 aactgaaatt gcacgtcgtt tagctaaaat tgttgacgca ccttttgtaa aagttgaagc
83461 gactaagttt actgaagtag gttacgttgg acgcgatgtt gaatcaatgg tgcgtgactt
83521 agttgaagaa gccgttcgca tggaagaaaa agaacaattc gaccgtgtta aagtacaagc
83581 aactaagaag gcaaataacc gcttagttaa gttaatcgtt ccaggcatta agcgtgaaaa
83641 ccgtgaaaat tcaatgcagc aaatgatgca gatgctttct ggcaacttca acatgaacca
83701 agctcaagat aatgaagaag taactgacga tattcgaaat gaacgtctta gcgtggctga
83761 tcaacttaat aagggcttac ttgaaaatcg tgaagtaaca attgaagttg aacaagcacc
83821 taaggttaat ccaatgggcg atatgatggg ccaaatggga attgatatgt cgagcttgat
83881 gggaaattta atgcctaaga agactgttaa gcgtaccttg aaggtatcgg atgcacgtga
83941 agttttaatc caagaagaat ctaagaagtt aattaattac gattctctct accaacgcgc
84001 aattgaacga acccagcaaa atggaattat ttttatcgat gaaattgata agattaccgc
84061 tggcaacaag aagacttctg gtgaagtttc tcgtgaaggt gtccaaagag atatcttgcc
84121 aattgttgaa ggatcaactg tttcaactaa gtatggacct gtttcaactg accacattct
84181 ctttattgca gctggtgcct ttgctgaaag taagccaagc gatttaattc cagaattgca
84241 aggtcgtttt ccaattcggg ttgagttaaa cgccttaact caagaagatt ttgttaagat
84301 cttgaaggat cctcaaaact cacttttgaa gcaatatatt gcacttctta aagctgacgg
84361 tattaagcta gtcttcactc aagaagcaat tgaccgtatt gctcaaatcg cctttgaagt
84421 aaatcaaggt actgataata ttggtgctcg tcgtttagca actatcttag aaaagttgct
84481 tgaagatgtc ctttacgagg gtccagatat gaatatggga gaaattacta ttactcagaa
84541 gtatgttgac caaaaattga gtgatataat tatcaataag gacttaacaa aatttatttt
84601 gtaattttag tagataaaga gaagttaaat tatggattat caattaaaaa ataatttttt
84661 aacagtaacg ctttctgatc agggtgctga aatccaaagc gttaaagatg taaacagtgg
84721 tcgagaatat atttggcaag ctgatcctaa aatttggggt cgtcatgcgc cagttttgtt
84781 ccctgtagtt ggtcgcttaa aggacgatca atatacttat gatggtaaga cttatcatat
84841 gggacaacat ggttttgcaa gagattcgaa gtttactgtt gaagaacaag acgataagca
84901 cattgttttt cttctgactt ctaatgaaga tactaaaaaa atgtatccat ttgattttga

84961 gttaagagta acttacagct tagtcaataa cttgttgtca gaacacttta cggtaactaa
85021 tacagatact aaggaaatga tctttggtat cggtggccac cctggcttta atttaccaac
85081 tgataatggt ttgactaaga atgattatta cttcaaattt gccccatcca tggatcatgt
85141 gaagattcca ttgaaggcac cttaccttga ctgggataat cgttcattgc ttgcaacaga
85201 ttcactgttt gaaattagtg atgaattatt taaggatgat gcttgggtct ttgaattaaa
85261 gcaacctaat aagatttcta ttagaactga taagagtgat tatcatatca acgttaaaat
85321 ggaaaatgcg ccatttgttg gtttatggtc acaatatcct aaatctggta attacatttg
85381 tattgaacca tggtggggta ttgcagacac cttagatagt gatggtcaat tagagcacaa
85441 gcgtggaatg aatcacattg ctccaaacga agtttgggag aatggtttta caatggcttt
85501 ccacgataag gctaagtaat taaagattta agactttggt tgagagatca aagtcttatt
85561 ttgtatatag gtgaaaaaat gagaatcgga tatctaaaga atttgggtag acatgaatta
85621 gaacaggcgt catctaagtg gcagacttta catgaaaatg aaaaaattga tttagaacca
85681 gttggctatg atgagattga agctaagatt caaaatggtg aagtagatgc agtcttagtt
85741 aattctagaa atgacattta tcaagcgctt gcaagctatt cagtaagtga gattgcctta
85801 cttgccttag ttcaggcagg taattttaat aagtatcagc aaactgttga attagatgag
85861 ttgaaaaatg tgccgtgtat tatggttgct tcagtagcag atgagaagag cgagcatcat
85921 tatctaaaag acattttgag aataaagaac gatctgattg cagttgaaac tttaggtgaa
85981 gcaattttga tggttgaatc tgggtcaggt tacttaataa tgaatgaaat gactgcgagt
86041 cacgtagatg atgatcaggt gcagaaatta tttttgctca gggatggtaa gcaattgcgt
86101 gagaagtatg tcttacttgc taagccagat gatgcaagag tagcagaatt tagcaagata
86161 atgaaagaaa gtattaatta aagtaaaata attatgtaaa gaattgcctt gattggtgta
86221 ttttaaaacg tccagtgtaa aaagctggac gtttttttcct gccttgcgat atacaagttc
86281 ccattctggt gcgaaatttg gtacaataga atgtatgttc tatagagagg gtgaaaagaa
86341 gtgctgacta agtcgcatcg gatttgcagt attgcaatcg tcgaattaag tttattgtta
86401 acgaatcgct tgttaattga tccagttaac aacattatta ttctagctgc aactgcaatc
86461 ggcgcttctt tacctgacat tgacgaatac aatagttcag caagtaagaa gtcagtaatt
86521 aacttcagcc tttttcttag acaccgcgga atcacacatt cttttcttgg ctgggcagtc

86581 ttttctggtg gtttatatta tttaatgagt aaatttatcc ctattcggat taataattta
86641 acttcgcaga attactggag tgcactttgg tttggtctag taattggcta tttttttgcat
86701 ttaattcaag atagctttag taaacaggga gtagagtggt tagctccatt ttataagaaa
86761 aagaaaaagt caccgtttca ttataaagtt ggtggctgtt ttgaaaaatt tttaactcct
86821 atgtcctatt tagctgtaat aacgataact ttttactgga tctggatttc cttaacacca
86881 gcgacgcaag tctttttgtt ttgaaaagtc attgcgcacc tgtaagataa ttagtggggg
86941 aagattccaa gagaggagtc ctagtatgtc ccaattatta gtagtagctg gtttaattag
87001 tcttttatat gttgtgatag atatgaaaag tcaggctatt ccagttgaaa atgacacagt
87061 ccacaaaggg cgataataga aagtaaagtg gccaagagtt aaaattgctc ttggtcattt
87121 ttaatttagc cagtatactg ataataatga aactattaaa ggatatgaat tatggattca
87181 aacgatttaa tttttgccaa attagaacat aagcgtctgc gttataactt gattgctgat
87241 ttatttttaa ttgttggtgg aatacttatt tttagacagc cagcgatgag tatgtcattt
87301 caatttctgg gttctttgat gattatcata gcaatgatct ttaccatact ttcttttaga
87361 atcagaagta gcaagcaata tcgcgccttg tcattattag ttaaaaatga gccaagcagt
87421 cttaaggaaa ttatgaattc gtttggcgca gggatgactt ttttagcttt gtttatggct
87481 attggcttca tggtaataat agtcatcttc tttttcttac ttagtcgatc atggattaag
87541 gcattactat tttttatcac ttttgaaaca gtgattgatc ttaaagactt tatgaataat
87601 ctcacaaaat tgaaggataa aagtacgatt taggctttta tcctttttta tatcagttaa
87661 aataattgtg atttaatgag catattaatt tacttttagc ttaaaatccg ataatatagt
87721 atctataaaa tttcgctttc tagagagaat caatttataa cactacaaaa ttgcagattc
87781 cgtaaatttt cctgggttgg aataggcaat taaactggag gtatttttta ttgataagta
87841 ttgttaaaag tagatcattc ggtctagcag cagtaatgac cttaatctgt tctgtcgctg
87901 gaatttttat tgctaaattg ccatatctca atttaatcgg tgctttagta attgcgttgc
87961 tattaggaat atctttacag gttttaccag taggggttag agaagaggca gcacctggaa
88021 ttggatttat ttctaataag ttcttgagac taggaattat tttactaggc tttaggctta
88081 atctaaccaa gttagctgat gctggcatta agactatttt agtggcaatg ttaggcgtta
88141 gcggtacgat tattttaact tattggttaa gtaaaaagtt tggtgcagaa gacgaattag

88201 cagctttatc tgcttgtggt actggtattt gcggagcagc tgctgtaatg
ggtgtttctc
88261 ctcaaattga aagccgcgat gaagaacgta aaagagaaaa cgaagttctt
gcggttgcag
88321 tagtttgtgt aatgggaacc gtctttacat tatttgaaat tgtgatcaag
ccaatgctgg
88381 gattgactga ttcacagttt ggaattgtag ctggtggatc tctacatgaa
attgctcacg
88441 ctgttgcagc tggaaatgca tttggcgaag ctagtttaga tagcgccttg
attatgaaac
88501 tttctcgtgt tttactttta gcgcctgttg ccctgattgt tggttactgg
tatcagcgaa
88561 gactagttaa agagagtgca gcggatcaca cgcaagcacc taaaaaattg
ccaattccat
88621 ggttcttagg tggtttcatc ttaactagtg ttttaggaac cttcttacca
tttccaccag
88681 tagttttgga tagtttggtt caagcagcat acgtattctt aggaatggca
atggctgcgc
88741 ttggaatttc agttaacttc aatgtgatct ttaagcgcgg tggtacagtt
tttggtgctg
88801 cggcaattag ttcaacttgt ttgatgattt tcatgattat catgagtaaa
ttattctttt
88861 aaatatagta aaaaggcgat tgcatcttaa atcttatgca atcgcttctt
tatatgttta
88921 taaaaaaatt aattagtttg gcttttttta aaacaagctt atttatcttg
attcatttag
88981 aataatttcc tttaatggat ttaaagccta aaaggatgat aattatggaa
attaataaat
89041 taaaaacttt tgtagaattg gcttctactt tgagtttttc aaaaactgca
gcaaatttgt
89101 ttataaacca aagtagtgtg tcaaaacaaa ttaaatcact tgaaagagaa
ctgaactgcg
89161 agctatttat caggtcaaag cgtcaagtaa gaatgacgga atacgcaaaa
attctttttc
89221 cagctgctca aaaaataatc aaattgaacg atcaagcttt gtctgatttg
agaaactgtc
89281 ttcaggatcg ggacgataag attattattg gtgttttacc gactttttgg
aattcttctt
89341 tttttgatca aataagtaac tttaaaagac agaacccaaa tttgagaata
attattaaag
89401 aaggtgaaat aaactattta acaaatttat tccagcaggg taaattggat
tttgcttttg
89461 taagaactac gtatggctat aaatccgatt ttgaggaatt tacagttgat
catgaatctt
89521 tctgtgcttg tctaaattat gaacatcctt tagctacaaa aaaatcttta
aaactagctg
89581 atttaaagaa tgaaaacttt gtcttctatt caggtgaaga gatgttatat
cacacagtat
89641 ttgacctttg ccgagaaagt gggtttgagc ctcaattttc tcttgccagc
cacaacataa
89701 aatcgatctt aaatctagtt aagaataatg aaggaattag tattctcatg
catgctcctg
89761 aaaaagcgaa agatgtaact tatgtaccga ttaagccagc tttttctacc
agattagttt

89821 gcgtgcgaca acctaaccaa gctactaaga tagccagcga tttttggaat tacttagtta
89881 aataatattc tcttttggaa ttgaactatc caaaaaaaga attgctaagc atagtgtttt
89941 ttatcaaaat agagccaatt agtattccat aaatatagtt ttatttatag aaaggatctt
90001 gattatgaaa ggaaaaagaa gagtaatttt gctaggagga atggtgctta cctctttaac
90061 cttacttact gcttgtcaaa caagcgccac caataataat tcttcttcag ctaatactag
90121 taaagttgtt gctaaagcaa ctaagtatca agtatctaat agcgacatta gcaatgctaa
90181 aaagctccta attaattcaa gtaaatggca ctataacgct aaaaacaagg tttactatca
90241 agtaaacgtt aaatacggta ctaagactaa gagttcttat gaatcaatgg gaatttatat
90301 tccagcagct tatgtaaatg ccaaaaaatc tggtactaat acctataaga tctcaattaa
90361 tactaataag aaagtaaatg gttatactgc gtcaactgcg ccaattgtaa tgccaattaa
90421 tacgccaggc tattccgcac agcaagcacc aacaggttat gaatctggtt cagccaagtt
90481 tactaaggca ggatttattt atgtccttgc tggttgccgt ggtttaaacc aaactgatag
90541 ttctaacgga tcttcaccat ggggtgttac tgatttgaag gcagctatta gaactttgcg
90601 tttgaatgaa aatagaatag ctggaaacac taatcgtatt ttcacttttg gccacagtgg
90661 tggtggtgct caaagtgcta ttgtaggtag ttccggaaat agcaaggctt acattaaata
90721 cttgaaggca atcggagcac cgcttgctac tacttctgga aaggctacaa gcgatgcaac
90781 tgaaggtgcg atgtcatggt gcccaattac tagtttagat actgctaatg aagcttacga
90841 atggaatatg ggacagtatt caactagtgg aacacgtgcc tctggcactt ttaccaaggc
90901 tttatcaaac gatatggcta aaaagtatgc tgaatacatt aataaacttg gtttaaaaga
90961 tagtaagaac caaaaactta ccttaaagaa gtcaagctca ggtatctata cttcagggac
91021 ttatgccgaa tatttaaagg cagaagtaga gcagtcgctt aataatttct tgaaggatac
91081 taagttccca tatacagcta cttcaatgaa tggtccaagt ggagctgcac aacaatctgg
91141 ttctaagcct tcaggtcaaa aaccgagtgg tgcaccaagc aataacatga agaatggtaa
91201 taatagtaaa ccaagcggca agaagccaag tggtagcatg ccaaatggtg gcgctggcgc
91261 accaggtggt tcacaaagta gtggtaaaac atataagacc gttcaaagtt atattaagtc
91321 tttgaataag aacggcactt gggtaaaata taacgctaaa actaatactg ccaaaatcac
91381 tagtttgaaa gcttttgtga ctcattgtaa agaagcctca aaggatgttg gagcttttga

91441 tggacttact cgtcaacaaa ccgaaaataa tttgtttgca acaactacga
gaaaaaatgg
91501 ccactttgat tctacgatga gtaatttact tagtgaaaat gcaagtaaat
atgctaaatt
91561 aaagaattac aaatctagtt atgctagtgc ctttgcatca gatttaaagc
aaaaagatgc
91621 tcaaggaaac acgatgacta caagattaaa catgtataat ccaatgtatt
acttgtctga
91681 ttactacaag ggctataata cgactaaagt agctaaatac tggaggatta
gaactggtat
91741 taatcagggc gatactgcat taactgttga aactaactta gctttagctt
taaagcaaga
91801 tagtcaggtt aaaagtgtta actttgctac ggtttggggt caaggacata
ctgaagctga
91861 aagaactgga aataacgaaa ccaactttat taagtgggtt aataagtgtt
tgaaaaatta
91921 gctgtgatat acatttataa aataccaata tagtaaaaag gcgattgcac
attattaatt
91981 gtgcaatcgt ctttttttaa tcatatttac tccattgatc ttgcaatttt
tcaattaaag
92041 gctggactcg atctttattt tgttttaagt aacaagcata aaacttctga
tgggctactt
92101 cgtcttttaa tggacgaacg ctacgattat taggtaaatt aagtccccat
tgtgagtcta
92161 aactcgttaa gtttgtcgta aagaaaggta aaacagagta ttgtaaaatc
tcgctgtatt
92221 cactggattc attttgaaaa atgattttgc tatctggaat ctcgtcttgg
taaatcttag
92281 tccaaaagcc aattggtgaa gggcttaaga ttgttttgcc cttaagcatc
ttgaaggata
92341 gttccttata ttgattaaga ttgcattcgg caggtaagtt aacagacatt
gattctgtgc
92401 ctaagtaagt tgaagtaatg tcgcggtctt caattggaaa gttaagcaaa
aggcaagtta
92461 cttgatggtc agctaataaa ttttcaaaat tagattcaac taaatcacta
acgatctgga
92521 tattctggtc atgtaaagat cgtaaaacga ttcccggccc tggtgcattc
acgccaacaa
92581 ttaaagtggt ttgatctttt tcaaattgct tgactcgctt agtaaaatca
agattatcat
92641 caattaactt tttagcttct cgggctgtat atttaccagt ctcgcttaag
tagagcttat
92701 ttggttttct aatgaataaa gtaacgccta aatcttcttc gagctttttc
atggaatgag
92761 tgaccgctgg ctgggttaga cctaatactt cggcagtctt agcaactgtg
ccgtatttgg
92821 caaaagtcac taattcttct aataaataat tttcaatcat aacttacctc
actataaatt
92881 acatttatcc tagcattaaa aactctgaat tttcaagtat agatcttctt
cgtataatca
92941 tttatgaaaa gtttgaaagg aagaagaatg acttatggca cgagtagtaa
ttttaactgg
93001 tagtcctcat tatgaaggtg cttcacaaaa attagctgat aattttgaaa
aaggtgtaaa

93061 agaagccggc aatcacattt atcgttatga tgcaggtctt caaggagata gtcagccgca
93121 ttttttacaa ttagagcatg cgcctggtat ggaagttggc attcctgata atgatgaggt
93181 agaaaaggaa gttattccag agttattaga tgcagatgtg gttgtcttgg tatcttcact
93241 ttattatttt gaaattaatg ctcagctgaa aacagtcatt gatcgctttt atgattataa
93301 tcatgaatta aaggataaaa aaatggtctt tatgatggct gggtatggta cgcaagaaga
93361 tatggatgca gttaagcttc atatgcagaa gttgagcgac tatatgcgct ggcaaatgat
93421 tgatcaaatt tatgccgatg attcatggaa taagcaaaaa ttagcgaaat ttgctcaagc
93481 ggcttataac ttaggtaaga gtattaaata aaacattgta tataggaaga aagagaaaaa
93541 taaaatgaaa tacgcagaat ttgttaaggc tggcgaaatg aaaattattg ataaaccatt
93601 gcctaagatt gaaaaagatg acgatgtaat tattaaggtc cttcgtactt gtgtttgcgg
93661 ctcagatttg tgggcatacc gtggattaga agagcaaggt catgaaaata gcggtcacga
93721 aattattgga attattgaag aaattggaaa aaatattact accgtcaaaa agggggactt
93781 tgtaattgct ccttttaccc atggttgtgg tcattgccgg gcatgtttag caggttttga
93841 cggtgtatgt atgaatcatg acttgagtga aaacttctct ggtggctatc aagcagaata
93901 tgtgcgtttc cagcatgcac aatggtcatt agtaaaggtt ccgggtaagc ctgaagatta
93961 tactgaagga atgcttaagt catttttagc cttagctgat gtaatggcaa ctggttatca
94021 tgcggctcgt gtagctaatg taaggcccgg agattcagta attgttttag gtgatggtgc
94081 agttggcttg tccgcaatta ttgcatcaaa attgcgtgga gctaagcaaa tcgtttctac
94141 gagtcgtcat ccagatcgtg aaaagttagc acgtgaattt ggtgcaactg acaatgtggc
94201 tgagcgagat gaggaaggag ttcaaaattt gatctccatt acgcgtggtg gtgcggatgc
94261 agtcttagaa tgtgtcggca gtaagttatc taacgaagaa gctgtgcaag ttggtcgtcc
94321 gggtgcaatt gttggccgtg ttggtttacc acacggagct gaattaaatc cgggtttgat
94381 tttttacaag aatatgagtg ttgccggcgg tccagcttca gtgactactt atgataagga
94441 agtattgctt aaggctgtct tagatggcaa gattaatcca ggcaaggtct tcactaagac
94501 atatgattta gatcagatta atgaagctta tcaagatatg gcagatagaa aagtaattaa
94561 gtcctacatt aaagttagtg attaaggaga aaaatatgaa taagtggaca aatgaagaaa
94621 tagctgcttt tgataaggct caaactttac acaatagtcc ttataatgat gataagacaa

94681 cttctgaaga agacaatcct gtctgggaag taactgtcga taatcaggtt
ttcattcgcg
94741 gtgctaaagg gattaaggat actaaatggt atcaatctgg aaccaaaaat
ggcggtcaaa
94801 ttgaagttgc tggtcaaaag tttgaagtga aataccaagc agttaataac
gcagaattaa
94861 ttaaggatgt taccgaagct tttaataaaa aatatcatgg tcagtatcca
attgacttga
94921 tggtatcaga tcctgtggct aaggcaacaa ttgcaattgt gaaaaagtag
aaaaaagaaa
94981 tatctcttat tgaaagtgat aaaatttcat gatattctaa tctcatcaat
tgaaattcat
95041 ctcttagaaa gaagggtcgc cgttgcgtct ttaatcttgt gaaaaaagca
tagttaaata
95101 agtactccat ttatgtagga aagactatgc acttttgaca agattaaagc
gaggcactat
95161 cttcttttaa tatcttaaaa gcagaaagag tctgtacttg tcataagtgc
aggctctttt
95221 tttgaggtga tttgatgagt agtattaaaa tttcgaactt atcatttaga
tatgaagata
95281 gtagcgataa tatttttaat aatttgaatt tggatttaga cagttcttgg
cgtctaggct
95341 tagttggcag aaatggacgc ggaaagacaa catttctgaa gctattaaat
gggaagttac
95401 tagggagagg caagatacaa actaggttga acttctctta ttatcctatt
tcaataaatg
95461 atcctaaaaa tattactttg tatgaactgc agaatcaggt gcaatttgag
cagtgggagt
95521 tagaacgtga gttgaattta ctaggtgctg atccgaattt gatttggcag
ccttttaata
95581 gcttgagcgg gggcgagcag acgaagatct tgctagcgct ttcgtttact
gatcagaatt
95641 cattccctcc gattgatgaa ccaactaatc acttagatga agcaagtcgt
aaggaagtta
95701 gcgattattt gcgtgtgcat catcaaggtt atattgtagt cagccatgat
cgggacttct
95761 tgaatcaggt gacagatcat atcttagcaa ttgagaatac agagattcac
ttgtatcaag
95821 gcaactttgc gacctatgaa gaagttaaag cagaaagaga tgaattcaac
cgggaaaaaa
95881 atcaaaaact agctggtgaa attggtaatc ttcatcgtca aaaggaacag
ttctaccatt
95941 gggcgcaaaa gatagaagct aaaaagaaac ttggtcaaaa aacgcagtac
attattaatc
96001 gaaggtctag aatcaataaa gctgccttgg gtcatgcatc ggcgaagatg
atgaagaagt
96061 caattaatgc tagaaataga atggataagc gaattgagga aaaagaagga
ttgatgctta
96121 atattgagga tgtgccaagt ctaacgatga attattgccc taattaccat
caagttctgc
96181 ttgaaacaga gaagttaaga ttaagcattc aagataagaa attgtttgct
gatctaaact
96241 tagtggtaag gaaccacggc gtggtttcgc ttgaaggtaa gaatggagct
ggtaagtcga

96301 cattgctaaa gttgttgctg gggaaagcaa gagaaactaa atatcaggga aagtatagtt
96361 tagcaagtgg attgacaata tcctatctac ctcaagactt cactatatat aaaggaagct
96421 taaaaaagtt tgctcaagag cagggtgtat tatatgaaga cttgcttaat atgttgaaga
96481 agatgggctt tccgcgttca aactttgaga cgcctattga agagatgagt atgggtcagc
96541 aaaagagagt tgcggtagca aaatcattag ttgaaaaagc agacttatat ttgtgggatg
96601 agccggctaa ttacttagat gtgtttaatc aagatcagtt gattaagtta ttgcaggaag
96661 ttaagccagc tatgttgttg attgaacatg ataagtactt tattgagcaa gtagcagatc
96721 aacggatcgt tatatcaaat taaactgcat ttaaaaagag gatgatttac agcattaatg
96781 taaaattatc ctctttttga gtataggatt aagttagaat tctagtcgca tttgtttgtt
96841 gctaaaaaat gacccaatga ttattgtgtg attcttttcg ctgactcgat aaaaaattgc
96901 atattttttg ccaatagtta ttttgtaagt tggtttatcc aattgatatt tttgatgtac
96961 attttgataa agataaggaa aatcagatag caactgtata ttatggtaaa ttaaagaaat
97021 atattttgtt atttcctcgt ctgaaagaag aagagtattc ttccagtaag aaatttcgtt
97081 agttagggat gtagtaaaat ggtcagaata aatgatagta tccatttatt ttccccagct
97141 gtatttgcca aacatggctt tcatttcttt ctctgtagca aaatttccag tttcaaagtc
97201 tttaaaacct tcttctatta atgcacgatc atgttctaaa tcatttttgc tcaagtcgat
97261 aattactcga tgattttttt tatcaactgt gtaggaaagt agctctccat catgaacttc
97321 ccagtccttt ggaaaaatac tgcctagaga attcccaatt tttctgactt ttagtttttt
97381 catgtttgat catcctttct tattgttcct ttagtataac atgttataac ttaaatgaga
97441 tgcgcaaact attaaaaaat ggatagacta tttttctttt tttacatttc ctaaaaaaac
97501 tactatttta tgcaaaaata aacgtcacat tcgcgtaata gatattgaga attgatttct
97561 taaactattt attaggagaa ctttttcgtg aaaaagcaga aaaagccaga tgtggcgaaa
97621 ttacttggca tcatagacga tctgatgttt cagaatgtga tgaaggatcc agtgaattgc
97681 cggatgtttt tgcatgaggt tttaccagat ttggatattc aaggccttac tgtgcgaacg
97741 caggaaagaa tagcatttaa taaggaagag aagttttctg tactggatgt gttgataaaa
97801 gacagtcagg gtagaaggta cgatatagag atgcaggtcg cgccacaaaa agacttggat
97861 aaacgagcac gctactacat gtataagatg atggaagatg gatttttgca tcagggtgaa

97921 gaatatggcg aattgacagc tgtctatgta atatttattt tgccgtttga tcctaaaggc
97981 aaaggactga agcgatatac tttcacttac tctgctagag aagatacatc tgttgaacta
98041 aacgacgaat ctgagataat atacttaaac agcaaaggaa agaaaggcag cgttagtcaa
98101 ggactagaag atttttattc gttgatggaa ggtaaaaata ctacaaatag tgaatttatc
98161 aaacgaatca aaaagacaat ggataattat cgtaaaacag aagaatggag tgaacatgtg
98221 atgaatacgg aacaaattaa agaaatggca cttgcacaag gtgtagaaca ggggttggag
98281 caaggaagaa gagaagcaag aatttttgat atacggaaaa tagttaagat tctaaagaga
98341 atgaatcaaa gcgatgagca aattttgcaa gagctaaaac aagattatag tgacgatttt
98401 tctgatgaag aattaaagaa atttttgaag tagaagcatt tttacttata aggaaagaaa
98461 aataaccaaa aaagcactga taaatcaatc tttaattaga ctggttatca gtgcttcttt
98521 tgtatattaa ataattttaa aataaaacct ttgctcagct tttattctgt gcctaaaaaa
98581 gattatagtt aattatatga agaaaactac aagttgattt tatgatggag aaaatagatg
98641 gctattttaa aactgcaaaa tgtagcttat caagtgggac agaagcagat tctacaaaat
98701 atatctttta aaatcgaaga tggtgctttt ttgacactga ttggaccgtc aggcgcggga
98761 aaaagtacaa ttctcaaatt aattgctaac ttaattagtc caacgagtgg aaagattttt
98821 tttagagaaa aagatattat gacaattgat cccgtaactt atcgcagaag tgtttcttac
98881 tgcttccagc agccatcttt atttggagaa aaagtagcgg acaatttatc ttttccatat
98941 gagattagaa aacaagcagt tgatcgagag cgtatattac ggttattgca tgatgtaagt
99001 ttagaagcag attacttaga taaggatatc actagtctgt caggcggtga gaaacaacgt
99061 attgctttga ttagaaattt gatttttta ccgaaagttt tgcttttaga cgaagtgacc
99121 acgggtttag atggagacaa taaggcgatt atacatcaat tgattgagcg agttcatcaa
99181 cagggtgtaa cagtgattca agttactcac gatcaaggtg aaatagacaa agctcagaat
99241 attattaaga taaaaaaagg tggcatcctt gaatgaaaaa tgtaatggta agcaattggt
99301 cactagtttt tgcttttgct ttggttttag tttcaattgc aatctctgct aaagaaaaac
99361 ttggtttaac caaagatatt ctaattagtg tgattcgcgc aattgtccag ttaatagtta
99421 tcggctatgt tttaaagttt atttttcatg taaataatta ttggttaact tttacatcaa
99481 ccttaattat tatttttaat gcgtcgtgga atgctaatca aagagaccct aatcctgata

99541 agaaatttta taattctgtg attgcggaag ctattggaac atacgccact
ttagcgatct
99601 taattctttc aggtgtaatt aaaccaattc caatgcaagt tattccgatt
actgggatga
99661 ttgcaggtaa cgaaatggta gctgttggct tgtgttataa gtcattaaat
gaaagttttc
99721 gcgatttacg gcagacgata ttagaaaagc tagccttagg tagtgatatt
aaaacagcct
99781 cgatgccaat tttaagaaga agcatcaaaa cagccatgca accaacaatt
gattcggcta
99841 agactgttgg cttggttaac ttaccgggga tgatgtcagg cttgattttt
gctggcgtta
99901 atccgattta tgcaattaag tatcaaatta tgattacttt tatgctttta
agcgcaacaa
99961 gtttaggggc tgtaattgca ggttacttag cttatcgaaa ttattttaat
gagagaatgc
100021 aattaaaata aataggccag acttgaatgt caataggcct atttttttgtg
aagaaatccg
100081 cttacttgaa agtcatttat tttgtatgta tacttgtaaa ttgtaagtaa
gaatatgaaa
100141 taaatggaag aggggaattt gtcgtgacag aagaaaagta ttacggtgct
aacgctattg
100201 tagatagctt aatcaatcat gatgtgaaat ttgtttttgg aattcctggg
gcaaaaatcg
100261 atcgcgtatt tgaattgctt gaacattcta agaattcgcg tacaccaaaa
tttattttga
100321 ctcgtcatga acaaaaacgca gcctttatgg ctgctggagt aggacgaatt
actggaaagc
100381 ccggcgtagt attaacaact tcaggtcctg gagcttcaaa tttagcaact
ggcctagtaa
100441 cagctacagc tgaaggagac ccagttttag caatttcagg ccaagttcaa
agaacagatc
100501 ttttgcgctt aactcaccaa agcatgaaaa acgcagcttt atttgaacca
attactaaat
100561 atagtgctga agttcaagag ccagaaaata tttctgaagt aattgccaat
gcttatcaag
100621 aagctgaagc tgctaagcag ggtgcaagtt ttgtttcagt tccgcaagac
gtaactgacg
100681 cagaagtaaa gactagcgtg attgcgccat tagaagcacc aaagcttgga
ccagcaagtc
100741 cgatcgaatc aactttgctt tctcaacgta ttaaggcagc taaattacca
gttcttttac
100801 ttggaatgag agcgtctgat ccagaaacga ccaatgcgat tcgacgttta
attgctgaaa
100861 ctcacttacc agtagttgaa accttccaag gagctggaat tattccacgt
gaattagagg
100921 acgacttctt tggtcgcgtg ggactattta gaaatcagcc aggagatcaa
ttgcttaaga
100981 aaagtgactt agtaattgca attggttatg atccgattga atatgagcct
cgtaactgga
101041 attctgaccg cagtgccaat attgttgtta ttgattccat gcgtgcagaa
attgataaaa
101101 actaccaacc tgaacgcgag ttagttggag atatttcaca gactcttgat
ttccttctac

101161 catatatgaa gggctattca atgcctgaag aatctaaaga ctacctagat
tctctgcgta
101221 agactttgca aaagcgcgat actccaccta ctgtaactaa agaccaaatt
aaaagtcatc
101281 ccctaagtat cattcaagct ttgcaagata gggtaactga tgatatgaca
gtgacagtag
101341 acgtgggtag tttctatatt tggatggcaa gacactttag aagctacgaa
cctagacatt
101401 tactttttag taatgggatg cagacgctag gagttgcact tccttgggca
atttcagctg
101461 ctttagttag accaaatact aagattgttt ctatgtcagg cgatggcggt
tttatgttct
101521 cttctcaaga tttagaaaca gcggttcgct taggcttaaa tattgttcat
atcatctgga
101581 atgatggtaa ttatgatatg gtaaagttcc aagaagaaat gaagtatggg
gattcagcgg
101641 ctgttaagtt tggaccaatt gattttgtta aatatgcaga aagttttggt
gccaaaggat
101701 accgcgttga aaaagcagct gatttaaata aggttttaga tcaagccttt
aaagaacaag
101761 gaccaacaat tgttgatgtg ccagttgatt atagtttcaa ccaaacatta
ggcaagacgt
101821 tgttagatga ccagattcat taactctgga aaggaaaata tcatggaaaa
atatgaaagc
101881 aaagtattcc agcatggcac tttagggatg ctggtgcctg gattatttga
tggaacaatg
101941 acaattgaag acctgttaaa gcatggtgat tggggcattg gcacagcaag
tggtcttgat
102001 ggtgaaatga ttgtgctaga ccatacaccc tatttggctc aaagtaatgg
agaaatcaga
102061 gttttaaaag ctgaagagaa agtgcctttt gcgacagttc attttgagca
gataaaagat
102121 aagtttactg ctagaaattt aactcaaaaa gagttagaag accatatttt
aaaaacttat
102181 ccttataaaa atgtattttt cgcagttaag attgtgggta agttttcagc
tgttaaaaca
102241 cgggtggttg agaagcaaac tagaccttat aaaactttgc ttgccgtagc
aaatgaacaa
102301 gccatatttg aaaaatatga tgtctctggg acagtaatag gttactttgc
accaaaaatg
102361 tttcaaggaa tggcagcacc gggatttcac ctgcattttt tagcagatga
taagtcaatt
102421 ggaggccacg tattaaactt tgatgtcagt gaagcaaaag tttatttgca
accatttact
102481 atgatcgacc agcatttgcc attagataat caagaatttt tagataaaga
tttagatata
102541 gcagatatgc atgatcaaat taaaaaagca gaaggctaaa caaaaagtgc
gtattatcaa
102601 cgtttttgag actaagataa tacgcatttt tttgttacaa tttttgattt
aatttttgaa
102661 cttgattttt tacttgatgg taaatacata gccaaataat taagtagcca
ataatgtaaa
102721 gaactgtaaa aacaaggaat ggtataattt gtaacttgat ccaatcatta
aggatgtaga

102781 aaagaagata atcaaggtag atgacaatag catcaatcgt tgttgcaagt ccaagtggaa
102841 ttctttcaac tttaaagact gcactgatgc ctccagcaat aaaagcaagt aagagacttg
102901 agataatatt tttatttaca tcttgaactg aaatagtctg aaaattaata gtaaaagaat
102961 aaatagagta aaagattaca agaattagag gaccaaagcc agccgcctgt agtccacttt
103021 taacaaagtt tttgataaag tctttcattt taattcagtt cctttctaat tttggcaaaa
103081 caacgtcttg aaacatagtc agttagtcca tttttcataa taatattgac tccagagttt
103141 ttggtttctt caaagtggtc aatatagttt cgattggcta tcgctgattt attgattttc
103201 caaaagttgg atggaagaag atcagtcagt tgatacagtc gctttctaat atgatattgc
103261 ttgtcattat tacagatggc tagaacctta ttttcaacaa ttgtaatcat gacaatatct
103321 gaaaaactga ccatagttaa atctttatca tcgtaagcta caattgtatt tgaattgcca
103381 tttgataaaa caaactcttt tagttgagct gaaaaatcac ttctttgatg tacataagct
103441 tccactattt ctgctttact tggttcaatg aataatttga atttcatttt taactccttt
103501 caatttttat tatagagatt agaaataaaa aaactttatt ttgcttgtta tcggttaaaa
103561 agatgcagta atcggtaaaa aataataaat taacgagtat taaataagat tttttgaaa
103621 aaatataaaa aaagaacaat gatcttctat tttgaagaac tcattgctct tttttcgca
103681 ttttgtttaa gaaattaaaa tatatacaag ctagttataa ataaatattt gacataaaga
103741 attgtaattt aatcaaacta tcttattgtg tttaacagga aacaaaaatg aagtaaccgg
103801 aataactaag tacagaatca tagctatttt tggaataaat aatgacagaa taatcaaaat
103861 aacatttaaa aatagggtaa cagcaacctt tctaatgaat gatctactga taataatatt
103921 cataatctgt gaatattttg ctgtcttttt tgcgccaaag tattgaatta gatattctaa
103981 aattttggca actaaaaata caattccgaa gttagcaact gcaaaagttg agctttcagc
104041 cataatccac ttagtcatta caggaagaag cgaaagatca gcaaggaaga agaaatctag
104101 tattgcaatc gtttttgttg gtttaaaaat atagtatgaa aatgctaagt gcaggtcata
104161 ccaaaagtcg gcgactacaa agaaagtaat taaaaagacg atgatattag caataaattg
104221 ttcataatgc acttcatgaa ttgctggctg aatttctaag acaataattg tgataataat
104281 cgccattacg ccatcattaa aagcttgtaa atgttcccag agtctttcag gttctttttt
104341 ttgagcctgt gctaattctt gccgaactgt ttttaacttt tctttttctt gatctgaaaa

104401 atcgttaaaa ttgtgttgat gtttatcttt tctcattttt atattcctat
tctgttaatc
104461 gatttcttcc cacttatctt gaatttgctc aattagtgaa aggagtcgct
ttttcttttt
104521 ctttttcttt tcacaacgat acaaacaaat taataacata atatataaag
tttattcaat
104581 atattcatga aaaaagttac aataggagtg aaaagtaata gttattttag
gaaaatgtga
104641 ttaatttgct taatatattt tcaaagaaaa gaaaaataat tatcaatttt
gtagattcag
104701 ataataaaaa tcaaatcatc actaacaaaa gctttttttgg taaaaagggc
aaacaaatca
104761 atattgattt aggatcaatt attgatgaac ttcaaaatca tggttatcaa
atcccatcta
104821 attttgacag gaagatggca ttaacttttg gaaaaaaaga taatattgtc
aatattaatg
104881 tgtcacatca aaagaagatt attgatgtta accatatcac taagaatttt
ccgcttaacc
104941 aagtaaagag aacgataaaa caaatagttc actattctgg agcagggagc
aggacaccca
105001 ttgataatgt taattcggtc gaattttatc gaaccttaga aatcgatgca
gttacaaaag
105061 aaatagtatc aaaaacggcc tggatttcaa atatcaatac ttttctaaaa
attggtgttc
105121 caaccttacc gggttatgtt cctgataaaa ctacggtagg tggtaaggag
atagtacccg
105181 atgatggaaa tcagaaatat tttgtaaaat ataagctaaa tgaacatccc
tcaacaaata
105241 aacaagcagc tattattcaa tatgtaaatt taacagcaaa taatgctatc
gttaaaacta
105301 ctaaattaga gggagaaccc aattttccta ttaattacga tattaataat
gatttaaatg
105361 acttaatagc taggggtttc aagctagttg acaatggttt taatggtaaa
ggaattcaat
105421 tctttggcaa taatgatagc tatattccag tttttattgt gacgcttaga
cgtgatgaaa
105481 taattgtaga ctcggcgcat ccctatcaaa aggtggatcc tgaagaatac
cagaaagaga
105541 taacttttac gattaaattc agtggtgcag gtaagcagac gccagaaaat
attattaaaa
105601 caaggcattt gcagcgaaaa gttttatttt cattagtttt ggataaaata
gttgaaaaca
105661 atacctatcc aactgaatgg aagaccgtta agaaagactt tcctgacaag
attcaagttc
105721 ccgtcgttaa aggatatcat agtaatcttt ctcaaataga aactgaaaat
ataactaata
105781 gcaaatcaga aatcaaggta tcttacatcc gcaataataa gataattcca
attgatgaag
105841 atggaaagct aatacctaca aaagaagctt tatatttagc aacagatccc
gatgatccga
105901 ccaagctatg tactaaacaa ataattcctc aaattccagg ctataaaagt
aattacaaaa
105961 taattacctt atctaattca aataaagata ttaaagttat ctacaaaaaa
attgcagctg

106021 atttttatac tgcttcgagt actaacgatc aaattgcaat tgtgaacttt attgatttag
106081 atcatgaagg gaaacagata acttcttcag gtccattaat tggcaagcct aatgaaaata
106141 tcacagattt atatagcaca tcgattccta tagcagggct tgaaaaagca ggttatcatg
106201 tcatttttaa taatttcgat ggaaataata agattcaaaa atttgatgga aatgatttaa
106261 caacacaagt ctttacagtg gggttaagta aaaattctga gcaaaatatg caattgcaac
106321 aattgaagag cagtgttaat gagcttatcg gtcaaaacag taataattta tcaaatccag
106381 ttgttgcgaa tttaatgaat gttcttaaat cattaataga tttaatttca attttgaata
106441 aagataataa gaacaaaaat taaaagaaag ctgaatctaa gaatgaataa aaagtgtcca
106501 aattgtggta aagaagtaaa tcttggggaa gctaaatgtc cattttgtgg ctatgatttt
106561 gtgaataata ttaatgctaa aattccaggc gatttgggta tgatgggcat aaagttaaat
106621 aatgtgccaa agaagaaaaa gaaaactaaa ccagaaaata aaaagattga aaaaaacatt
106681 aactcaaaag ttgaggtcgc aaaaacccaa gaattaaaac aagctagaaa aaataaagca
106741 gctaaaaaga ataaaggctg gttatagtta ttagcttgtc tagcattttt gacttttggc
106801 tatattggtt tttatgttgg aagcaatttt aattcaattc aacatagcac ttctagtgaa
106861 agtagttcta ataacagtca taatcatgtt aaaaaaagtg caacaagtaa aacttcttct
106921 aggcaacaag ataagcaaag taaatcagtt cagaagcaag atactaatac aaaattattt
106981 acctctatgc aagtccaacc tgcaataagc aatcaagaag aaatcaaaaa ctttttaaca
107041 ggcgtattta ctaaaccgca agcaaataag tttatttctg gtgaaaataa ttctgatttt
107101 cgtcaattac gtgacgtaac tttaagttgg gaaccgaatg atgttaatat caaaattaaa
107161 ttgttgaatg tttctcaaaa gggtaatgat tatactttaa attacctagt ttattataat
107221 tttattactg gaagaaaaca gattatggtt tataacaatg gtttgttagc acgcgatgat
107281 agcgcttttc acattgtgag attaggatct ggatgtctaa ttaaataaat aaaacataac
107341 ataacataac tttttatttg tgctatgatg aaattgtgaa tatattatat gtgcctaatt
107401 tctttataat tgatgctaaa tttaaatgag atggctttta tttttggagg aaaagttatg
107461 aattttaggg atatgaagag aaaagagcgt tggagtatta gaaagttaac tgtcggagca
107521 gcaagtgtat tattgggaac atactttgtt gcatcttctc aaggcgttgc taaagctgat
107581 accattaagg acaataatgt aaataacaat actgttaatg aacaaactag tgaaaagaaa

107641 atatcacagc caattacacc gaatactcaa gctgtagtta ttgcaaaaaa caatcctcaa
107701 aatagcgaag ataatactgc caatttacaa aataaggctg acctaattag acaaaataat
107761 caaaaaatca ttaataggct aactgtacaa aaaaatacta ctcaaaaggt tgaaaacagc
107821 actttttctg agataaaaac tagagaagca gctaatactc agccttttaa tattgcagaa
107881 aatagttcaa aatcggttgc tactaattct aataatagtc ttgatgaatc gacttttggc
107941 aaagtcgacg ttaatagttg ggatacagaa actgttaata atgggttaaa tattactggc
108001 taccatggca gtgatcaaga acatctagtt attccaaacg ctgctgattt tactaaggcc
108061 ggaaagttaa ctaatgagaa tgcagttatt acagtaaatg cagctaagtt caaaaagatt
108121 ttaaatacaa ttaagcctac tactgtagct atcagtaaga ctgataataa aaaagttcaa
108181 gtttcagata aaactgcttt tggtgggaat ttaactactg aaaatggagt tagcaccaat
108241 gatcctaatt tgaaacagat ggatcttacc aatttagata ccagcaattt gactgatatg
108301 agcggaatgt tttgcggagc atataaggtt aagtcattag gtaatttaag tggctgggat
108361 gtttctcatg ttaccaatat gcaacagatg tttgacgaga atacttatga tagtccagct
108421 ctagagtcat tagatggcat taacaagtgg aatgtaagca atgttcaaaa tatattgtgt
108481 ctttttagaa acgatattaa tttaaaagat atcgatattt ctggctggga tttaggtaaa
108541 gcaacgggag gttggacgac tggacaagtg tttgccagct ttactaatgc tagcaagaca
108601 gttattaatc ttaataatgt aaagttgcct tctgatttta attttgcaac cagttttgct
108661 aatggccaag ttgtaattac aaataatggt gatcttttac aattagatac tacttcgaag
108721 gttccattta aagataactt aacttttagg caatataata gttgggtaga gtatgccaat
108781 cctactgtca aaacctatag tcacagcata ttttatgatt caaaaggcag ttctgatgct
108841 aaaacgatcg ttcaagacgc aattgataca tttttacata ataattatgc aaattataaa
108901 aaaataacta ttcctaccga tgcagtatat ctttctacta agccgagtga tagcgcaact
108961 gctatagcaa atggaactta tgatatagct aaaactgaaa ctgaaactag tgatactact
109021 tatcaagtat ttcgtgatga tgaaattcca atgcagttaa aatatgttga gacggatact
109081 aataaagatc tcggaactgt tactgtcgtt ggtaatggaa taactattgt gacttctcat
109141 agtattggtc tgaatccatt gcccattact aatcttgctg actggctagg caacgatgat
109201 tatacaaagt cagatatgag tattactggg caaaaacact ttggctttga cgggattgca

109261 gagccggtta atgtagaaat acatttaact aagaaggtga ttacagagca acggacagtt
109321 actattcatc tatatgaaag agacagtaat ggcaatcctg ttaaagatag taatggaaat
109381 tatgtatctg tagctccaga tcttaaagca acttttaatt atcaacagga tgagtttgta
109441 acttctaagg gtaaagttgt taagaagagt aatatttggt tatgggatga aaaagcgcct
109501 aaaacgtatg atggtgcaaa ttctcaagaa gtaaatgtcg gatatgtaga gggagtagat
109561 ggcgtgactc ctataattag taatgtaaaa tgctggaaag ttccaattaa taaagaaggg
109621 acaagctaca tattagttcc aaatgatgga aattggtcga cagtcgaatt ttcaactcct
109681 gctgtagatg gttactatgc aaatccattt atagataata ttgtaaacgg ctatccattg
109741 ttaggtagac cagctaattt atttgtaaat cctaggttca ataactctgt tcgtcctggc
109801 acgtctgatg caagtgatag tagtattgct tatacaaatc aagatggtat ttatgaaaat
109861 aaaaatgaac atataatctg gtattttcct aaggaaaata cagcaacaat taagagaact
109921 attaattttg taaaaaatga tgcaagtcat actcaattac aagaaacaag tactcaggat
109981 gtaaatctta atggctcttt ggtagcagct aatccaaatg gtactggcaa agattacttc
110041 acaatatatc ctgcagaata taatgataca agtattgtta ctggcagccc aattgctgta
110101 ataaataata gtaataaacc tacatatagt tggaaagcta aagatggcca aacaagtgta
110161 aatactgata taaccggata tacagtttca gctgttaaaa ttgggaacgc agactattca
110221 acgaatgaaa atgacattaa atatgccaaa gaaattgccc aaaatactgc aacattggat
110281 tctccagcag atttgaatat ttcttttgta tatgcacctg ttagccttac tagtagttat
110341 caattcgttg atgatgataa taaagaacag ttaatagcac ctgctacgtc attttcaggt
110401 caaactggcg aaactgttga acttaatatt aagctaccag ataattatga attagcaaaa
110461 ggaagtacat taccaactag ttatactttt aagcctagcg acaacgaaca gttaaaaatt
110521 catttaattc atcaaaagaa agttttatcg gatgatacta gaacaattag acgtaaagta
110581 actattactg atcccgatgg taaagttgat cctaagtcaa caacacaaag tgtctcattt
110641 agtagaagcg atactttaga taaggtaact aatcaacata tttatggcaa atggaatacg
110701 aaacaacaag ctttaccagc aattatttta cctgtatttg ctggatatac gccatctggt
110761 aaggtggatg caattaacgt aactgctgat agtaataatc ctgctgatat taatattagt
110821 tacattgctg ataagcaaaa cagttcttat aaatttgttg atgataataa aaatgaggct

110881 acggttggta caagtcattc gatttctgga accactgatg aaacaattga tttaaccaaa
110941 gcaataaaag attcagttcc aactaattat gatttagttc cagatcaaaa tatgagttat
111001 atctttaaag ctagtgataa taagccactt ataattcatt tgactcatca aaagaaagtt
111061 ttatcggatg atactaggac aattagtcgt aaagtaacta ttactgatcc tgatggtaaa
111121 gttgatccta agtcaacgac acaaagtgtt acatttagta gaagcgatac tttagataag
111181 gtaactaata aacacattta tggtaagtgg aataagaatc aacaaacttt accagcaatt
111241 gctttgtcag tatttgctgg atatacacca tctgacaagg tggatgcaat taatgttacg
111301 gctgatagta ataatcccgc tgatattaat attagttaca ctgctgataa gcaaaacagt
111361 ttttatcagt ttgtagacga taatgaagag ggagctaagg ttggtacaag tcatccaatt
111421 tctggaacca ctgatgaaac gattgactta actaaagcaa taaaagattc agttccaact
111481 aattatgatt tagttccaga tcagaaaatg agttatacct ttaaggctag tgataataag
111541 ccacttataa ttcatttgac tcatcatatt agtgcagtaa ctaatgattc caacttaaca
111601 aagacggtaa gtcgtaccat caatgttaca aaaccagatg gaacaaaatt agcaccagtt
111661 gttcaaaggg taagctttct ccaaacagca actaaagacg atgttactgg aaaagttaca
111721 tacaataatg attggaaagc aaatggtaaa gatagtttta cagaatatga tgctccaaca
111781 gttgatggtt atactgctaa tccagttaaa atagaaaagc ttactcctaa tgttaatact
111841 aaagattcaa ctaaaaatat tgtttatgag gctggttctc aaagtataac tgtacgtttt
111901 agtgataaag ataataaaga ccaacaagtg ggtgaagcga tttctattaa tggtgttact
111961 ggtgaaaatg cagacttttc tactataact attcctacaa attatgtatt agctgaaggg
112021 gaaaaattac caactaatca taaatttacc gccaatgatc aaacagttta tacggttgaa
112081 ctaagacata aaactgaaga cgccaaaatt gatcctaatg ctaagctcag tcaaaaagta
112141 agtcgtacta ttttgattac caaacttgat ggtagtgtta ataagattat tcaagaacat
112201 acctttactc gcactggtac gaaagatatg gttaccggaa atattagcta tggaccatgg
112261 tcagaaaatg gtaagtataa ttttagtgat attacagcaa aagcaattgc aggatatgca
112321 attattggtt cagctccaga aattgtagta acactagaaa ctaaggactc gatagtcaat
112381 attagctatc ttgctaatgg acagactaat tattatcaat ttattgatga tgattaccaa
112441 gtaggagatc ctgtaattaa tcaaaaacat aatattgcag gtaaaactgg ggatagcatt

112501 aatttaaata ttacgattcc tgaaaattat gacttagcaa aaggaaacca tgtgccagcc
112561 acgtatactt tcaaggctaa taacaatcaa ccgattgtga ttcatttggt tcaccatttg
112621 agtgcaatta tcaatgatcc taaattaatg aagaaagtta ctcgtacaat taatgttaca
112681 aaaccagatg gaacaaaatt agcaccagtt gttcaaagcg taagttttat ccaaacagca
112741 actaaagacg atgtcactga aaaagttaca tatcataatg attggaaagt aaacggcata
112801 gatagttttg ctgaatacag tgctccagta tttgctgggt atacagctag tcaaactaag
112861 gttgaaaaag taattcctaa tataaatacg caaaattcaa cgataaatat tacttatcaa
112921 gctaatccac aaaagatatc tgttcgcttt attgatgatg acaatcaaaa gcaacaggtt
112981 ggtagtgcat tcattattaa cggcgttact gatggaaatg cagacttttc tactgtagca
113041 attcctgaaa actatgtatt agcagaagga tcaaagttac caactaatca taaatttacc
113101 gccaatgatc aaacagttta tacggttgaa ctaagacata aaactgaaga cgccaaaatt
113161 gatcctaatg ctaaacttag tcaaaaagtt agtcgtacca ttttgattac taatattgat
113221 ggacaagtta ataggattgt tcaggaacat acctttactc gtattggcaa aaaagatatg
113281 gttactggaa atattaatta tggctcttgg tctgaaaacg gaagttatac tttcgcagca
113341 attcctgtaa agaatattgc aggatatgca actagtggtt cagctccaga aattgtagta
113401 acaccagaaa ctaagaactc aacagtcaat attagctatt ttgctaatgg acaaacaagt
113461 tattatcaat ttattgatga tgattaccaa gtaggagatc ctgtaattaa tcaaaaacat
113521 aatattgcag gtaaaactgg ggatagcatt aatttaaata ttacgattcc tgaaaattat
113581 agcttggcaa aggaaagtac attaccaaca agttatacct ttaaagttag tgataataag
113641 cctcttgtaa ttcatttaat tcatcaaaaa gaagctttat cagatgatac tagaataatt
113701 agccgtaaag taattattac tgatccaaat ggtaaaattg atcctaagtc agtaacacaa
113761 agtgttacat ttaatagaag cgatacctta gataaggtaa ctaatcaaca tatttatggc
113821 agatggaata cgaaacaaca aattttacca acgattgctt tgccagtatt tgctggatat
113881 acaccatctg gaaaagtaaa tgaaattaat gtaacagctg atagtcataa tcctgctgac
113941 attaatatta gttacactgc tgataaacaa aacagttctt ataaatttgt tgatgataat
114001 gaaaagggag ctgatgttgg tacaagtcat ccagtttctg gaaccactga tgagatggtt
114061 gacttaactc aatcaataaa ggatttagtt ccaagtaatt acgatttagt tccagatcag

114121 aaaatgagtt atacctttaa ggctagtgat aacgatccac ttataattca
tttaactcat
114181 catattagta aggtagttaa tgatcctagc ttaactaaga cggttcgtcg
caccattttg
114241 attactaata tcaatggtaa agttgataaa actgttcagg aacacacatt
tacccggact
114301 ggtaagaaag atatggttac ggaaattatt aaatacggac catggtcaga
aaatggtaag
114361 tatacttttg ctgcaatacc ggtgaaggca attgaaggat atgcaattaa
tggatctgtt
114421 cctgaagttg tagtaacacc tgaaacaatg gactcaataa tcaatattgg
ctatgttgcg
114481 aatggccaaa caagttacta tcaatttatc gacgatgatt ctcaagccgg
agatcctgta
114541 attaatcaaa aacataatat tgcaggtaaa accggtgaaa ctattaagtt
aaatatttca
114601 attcctgaaa attatgattt agtaaaaggc aacagtgtac caactactta
tactttcacg
114661 gcagaggata ataaaccaat tgtgattcac ttagttcatc agattagtgc
agtgactgat
114721 gatactagct taactaagtt agttcgtcgt acgattttga ttactaatat
tgatggcaaa
114781 gttgatagag ctattcaaga gcatacattt actcggactg gaaagaaaga
catggttact
114841 ggaaatatta gctatggtct gtggtcagaa aatggtaaat atacctttgc
tggtattcca
114901 attaaagaaa tcgcaggata tgcaattaat ggttcagctc ctgaagttgt
agtaacaccg
114961 gaaacaaaag attcaacaat taatattagt taccttgcaa atggccaaac
aagttactat
115021 caatttatcg acgatgattc tcaagccgga gatcctgtaa ttaatcaaaa
gcatcctatt
115081 gcaggtaaga ccgatgaaac tattaagtta aatatttcga ttcctgaaaa
ttatagctta
115141 atagaaggtt ctgttttacc aacaagttat acatttgaat caggtaataa
accaattatt
115201 attcatttga ttcatcaaaa ggaaagtttg ccgaatgata ccaaaactat
ttctcgtaaa
115261 gtaacgatta ttgatcctga cggtaaagtt gattctaaat caacaacaca
aagtgtcaca
115321 ttcagtagaa acgatatttt agacaaggta actaataagc atatctatgg
taaatggaat
115381 gaagctcagc aaactttacc ttcaattgac ttaccaagtt ttaacggata
tacaccaacg
115441 ggtgaagtga ctgcagctga agtaacaact aatagtggtg atatagcaga
tattaatatt
115501 agttatgtac ctaacgagca aactagttat tatgaatttg tagatgatga
caaccatgaa
115561 agtcttattg ctccagctac tgtaatttca ggcaggaccg gagagactat
taaattaaat
115621 attgttacac ctaatggtta cgcattagcc aaagacgtca cgttgccacg
aagttataga
115681 tttaaagcta ctaacaacga accaattatt attcatttag ttcatcatat
gagcaaagta

115741 actaatgatc caaaattgac taaagtaatt actaggacaa ttaatgtaat
caatccagat
115801 gggactactg attctattgt tcaaacagtt cgatttgttc aaacagctac
aaaagatgat
115861 gttacaaata gtgtttcgta tatgaatgat tggaagaatg ttggtgcaga
gagttttgct
115921 gaatacaatg tgcctgaaat tgcaggttac acttcaagca aaactaaggt
agaaaaatta
115981 acaccgaaat ttggtgatca aaatgtaatt gtcaatattg actacattaa
aaagagccag
116041 ataaaccctg ttaagcctgt caagccaata gtaacgccaa ttgaaaaaga
taataaagaa
116101 caatccgcta acaagcagca aacagtaact gctgagccaa attctaagcc
caaagtaacc
116161 caagaaatca agcaacctgt ttctaagcct aataaaacta atggtaaaat
tcttgaaaga
116221 aaagacagac aaacatacaa gtttcaaggg cagacagtaa ctatgaagaa
agtggttaat
116281 gctaaagaag caggacatat taataagata aaggctaaaa aggcagcttt
gccacaaacc
116341 ggtaatgatg aagcaaaaac tacagctata atcgcttctg ggttagcagt
tgatttaagt
116401 ttgataggtt tagctggatt aaaaaagaaa agatctaatt aatagatatt
ttgaatttta
116461 taaaaagagg caaaataata atgttatcaa gaaataataa tcgttttaat
gaacttaaag
116521 aaataagtcc tcgttattca attcgcaagt ttactgtcgg agcagcttct
gttcttatag
116581 ggatgtcaat tttcggacta aattctcaaa cagctcgagc agacagtatt
aatgaaaatg
116641 atacaaataa acaaaatcct gctattgagc agaaatcaga caaagctcta
acagctacac
116701 cggcaagtga tattaaaaat gtggttgtaa ctgctaagaa tgtagatgca
caaaatcaag
116761 taccggctga aacctcaaat ggtaatacaa gcagtaaaca aaacacaatt
aatactaata
116821 aagaaaacag ccaacgtgct gaactacaaa tagaaaacac tagaaaagtt
gttgcagcaa
116881 acaaagaaga atctacacaa gatacaactg ctattcaaaa tcaagccaaa
ccagttgcaa
116941 aagaaaacta tagtgttata cagcatgatg tgattgctaa taacggaaat
acaccacatg
117001 atagtggtta cgttcagtta aatttaggat tacaaataaa gaatactaag
aacattaatc
117061 ctggtgatta tattgatatt gacttaggat taccattaca atctggacaa
caaaaaacat
117121 atagtgatgg tttagcagag aaaaatacac cagtaaccat taaagacaat
gcaggaaaga
117181 cggatacaat agggagtatt gcgacagtag gaaatattcc taacgaattt
taccagttaa
117241 gttttaatga ccatattcaa aaatatggta ccgtacttct taatcttgat
ttaaagcaat
117301 attctttaat tcagcgtgct attagtacgg tcggttattc tcatgaaaag
aatggtccaa

117361 ccagttatag tgctcaagat gatttagtta ttggtgatgg tgcttataag
cttacttctg
117421 gactaagcgt tcctgttaaa tacattccgc aagaaagtaa tggttcagtt
attcaacgtg
117481 ttggagaaaa tacttggatt caaggaagaa gtactggcaa aaattctaga
gtatggactg
117541 tttatcctga tggctcattt actgtaaacg ataaatctgc acttggatta
gatggaattg
117601 tatatttttc taagaatttt ggcaatagtg ctacagtaac tgtttatacc
ttaagtaaca
117661 atccttactt tgattctaat tatgcaagcg acgatgatat taaggaacag
attgaaagtg
117721 cttttgcaca gttaaagtca actaacaatt tagatcaaat tgctcaagat
aattcaaatg
117781 ttggttttag tataaataaa ataccagaaa atgatatttc aattgctgtt
acccataatg
117841 ataattccga aaaagcgtat agcacagtta tgagggctga tgggtcaaaa
tactcagggg
117901 atcaattaat gctctcacgt acatatcaca ttactgttaa tggagctaag
ttaggccaaa
117961 tatattcttt accaatttct tttgtttctg aaattactaa atctggtgtt
aatgtctcaa
118021 agccagctaa tattactaag ccagaacaag ataagataca aacttatcaa
gattcaaaca
118081 aatatgaagt acttaataat gatttaaata atgcttatcc tattttatat
aaagggatta
118141 tgattgataa tcctgggctg atgaacttta tgaagaataa tcttgctact
tggattgatg
118201 tgaaaaatga tgaagatcct agtgatgagt tgttaggccc tgcagcgtat
actcttaatg
118261 tatctgttgt aaatcaacca actaacttaa aaccgcaaaa tattgcaaat
ggcaattctt
118321 cttcaggaca acagttagat caaactattc ttgttgtttt ccaggattta
gatgaaaata
118381 acaaaaatat cttaagtaaa gatttaactg gattaagtgg agctgatgct
ggatattcaa
118441 ctgcaaatga tatcaaagct ctagaaaatc aacattatga attagttagt
gatgatacta
118501 acggtcgaaa tcttaaattt ggtaatcaag aacaagtatt ttatgtcaaa
tttagacatg
118561 ttttaaagaa agaaaaacaa gaaaataaga gcgtttctag aaatattact
tatgttgatg
118621 ataaaggaaa tcctgttaaa ggctcacctg atggcaaagc tagctatgta
caaagtgcaa
118681 gctttgttcg ttttccagtg aaagatttag tcactggtgt tgtcggctat
agtattaata
118741 atgatggtgc tattgatact caggatggta ctcatgcatg gaaagcaatt
ggtagtaatt
118801 attttgaaaa agtaatttca aaagatcctg catcattagg gtttgagcat
gttaactatg
118861 ctgttattcc agagaaaaca gtagatgcaa atactaaaga tcaagagatc
caagttattt
118921 atagtggtac tactaagaac cctgacaagc cgagtgaacc aagtcaacca
actaaaccta

118981 ataaaccaga agtgccgaat aagtctgaca agtcgaatga atcaagcaag
cctgataaaa
119041 caagtgatgt caagccaaca gaatctgtaa aattgattca tcaaacaaca
gcagatactt
119101 caaagaacca aagtgaatca attaaggaag aaaatttatc aaaaccaaag
gctgttttag
119161 ataagacaag tttttcacct ttggctagaa gaaatagtcc tttagtcaat
gagaaaacta
119221 acattcttga atcctcaaaa gattcaacta agactactaa aaatagaaca
gtaaattcaa
119281 gtaaagaatt gccacaaaca agtagtaaca ctcaacaatc aattaatgat
gaagtgattg
119341 gttctgtggc tttatcaata ggattaattg gtttagccgg tgttaagaag
agaaaaaatg
119401 caagataaac tacactaaag agaaaagaga ccgactttaa gtcagtccct
ttttcttttg
119461 tgagtgctaa aattaaacta taagatactt tttatttata tatagaggtt
aaagatggat
119521 gataagttta atcagaatga aaatatagat gaacatgtgg tggtcgatgc
aaatcatcct
119581 ggatatggtt ttaccgaaga aaaactctta aaaacaatta cgcaaactgt
tcattatgta
119641 ggggctgcta gccgaaatcc tcaagataat gtgacgaggg taagttttag
tcgtgtctat
119701 atcgttaata aaaaaagtgg caaagtaatt gaggatcgtg gctttcagcc
aaaagaaaag
119761 agcatgcaat taattggtac tccaacactt ccaggctata ttcctgatcg
agtagtagtt
119821 ggcggagaaa aggtaacacc taatgattta aataaagaat atatagttac
ttttaaactc
119881 aatatgaaac caactaatac aatgcaaaaa gcaaaaatcc ggtacgtgga
tctaactact
119941 aataatcaat tattaaccga agatactgtt tcgggtttag caaatacacc
aattaattat
120001 gatcctcaat ttaaaatcaa gcactttgaa gaacaaggtt ataagctagt
ggctaatggc
120061 tttaatcata acggagatgt gcaatttttt ggaagttcaa atgattactg
tccaattttt
120121 attatcactt ttatatctgc tgttgaagct gtaaacataa ataatcctgt
agactgggtt
120181 gatccatctt tatatcacag aaagagtaag ctgattgtta attttgaggg
atgcccgaat
120241 ccgccaaaga cagtcattca aacaattaac tggtttcgaa caattacagt
taataatata
120301 gtcaaaaaaa taattcctaa tggtgtttat gataaaactt ggcgtgcgga
tgaggaaaga
120361 tatcaagaag ttaaaattcc catagttcag ggatatcatg cagatttaga
tgtcattaat
120421 caaaaatctg taactttaga tgatcaaacc attacagttc aataccaccc
taatggtcat
120481 ttgattccag ttgatgaagc gggagctgct attcaaggtg caccaacgcc
tcaatttgaa
120541 tcggactcta atgatgcaag taaagttaaa aatgatcaaa atgtgccgaa
agtaagcggc

120601 tatcaagcaa ctttagctac agtaactcca gcaaatccag gacaagattt aactgtagtc
120661 tatgaaaaga taaacaataa tgacgatact ttatatatta atctaaatca cgatcatgaa
120721 tcagattctc aagctgcaat agatcatgaa aatgtagatc aacaaactga tttgacagat
120781 aatagcaaaa ttgttcaaac agcaattatt aattttatcg atatagatga tcatggtcac
120841 tctattactt cttccggaaa actttctggg cgcgttggtg attcaattaa tgatttatat
120901 agtactgaaa ttccattgaa agtattgaaa gagcaaggat atagggttgt atttgatgac
120961 tttaacgaaa atagtgaaag acaatacttt aaagaaagtg ttctacctca gatatttact
121021 attggattaa gtaaaaaatc aagcgataat caagaaagtt cagatctaga tgaagaatat
121081 gaaaaattac gtcaaacaag agagcagttt aagcaaataa agccaacaat tatggatagt
121141 tctgacgaat tgaattcttc gcaaaccgtt aataaactca ttgatattat tacggggtta
121201 ttaactttga tatttatgct tagtaaaggt aataaaaaat aaaagattat agatctatat
121261 aattaatttt gcatttcctt gcaagatttt attaaaaagc gcatatttta attatagatt
121321 ttaaaaatag aaggtaagtt aatgcttaaa aatattatct ttgattttgg aaatgtcatc
121381 atgaattata atcccgatga aattttgaat cattatgagt taagtccagc agaccacgat
121441 ttattgagaa agacaatttt tgaatctaaa gagtggagcg agattgatgc tggcaagatt
121501 acggaaaaag aggcaactga aatatttata gatcgtgttc ccgaccaatt aaaaagcaag
121561 gttaaacaaa ttatggcaac ttggccagaa aatgttgatt tctatgaacc agtttttaac
121621 tacatggaaa aattgcgtca agatggttat aagatctatg gtttgtctaa cacagggatg
121681 caatttgcta attttgttaa gaactctgaa atggggaatt atttcgatgg ttatgttttc
121741 tctgctcaag agaagttaat gaagccagat agaaggattt atgagaaatt aattgctcgg
121801 tatattttaa agccagaaga aagtttattt attgatgatt tgaaagcaaa cactgatgca
121861 gcaaaaaaat taggtatgca agcttttact tttaaaattg acaagctagg tgagttacaa
121921 gactatattg ctagtcatta attttaagta taaaaatttt tcaggtgaat aagaaatgaa
121981 gacaaatgat caaattgcaa attgggcgat ggacttacaa agtttggctc aagctggatt
122041 aagatatgga catgatgttt ttgatcgaga acgttacgaa gaaattcgtc gtattgctgg
122101 cgagatgatg tcagctagaa ctggaattcc ggaagagaaa cttaagactc tttttttagg
122161 tgatgaaggt tatcaaactc ccaaaattga tacacgagct gcaattttta aagataatag

122221 aatattgctt gttcgtgaaa aaaagacaca ggagtggtca ttgcctggtg gctggaatga
122281 ttatgatcaa actactgctc aaaattgcgt taaagaagct cgtgaagaag cgggaagaat
122341 agtagaaccg attaagatta ttgcagtgca agatcggaat catcataata agcctgttat
122401 tgcgactaat attacgaaaa tattttattt atgtaaggaa attagtggaa agtttacgcc
122461 aaatgatgaa acggatgcat gtgattattt taatttagat aatttaccta aactttcact
122521 tggacgaaat actaaagaac aaattgcaat gtgttttgat gcaaataatg atcctgactg
122581 ggatactaaa tttgaataag atcaataaaa tagctccaag aaatattgct tggggcgttt
122641 ttttatgcaa atataatagc cacattttgg taaagaatga atgtccttat cagaatgtgg
122701 ctttcgtatt ttatagtggt tgaatagttt gataataaag ttagtaaaaa atagcttagg
122761 ttgtttttg ttgcatgtta ggtataattt ttaaccactt agtcaaattt cattgaaaag
122821 catctatatg atagtaaaat taaattcaaa taagattagt taatgaataa taaaaagagg
122881 tatataaaat gtttttcaat aaaaatgacg aaactaaaga taaagttgtt gatgaagttt
122941 taagcagtga tagttttaat aatttgaatc ttgatttaat tgatcttgat cttaaagttg
123001 aaactggtaa taagtttgaa gttcactatt gtggatctga agatgaaaag ccgagcgttg
123061 agcttaaaga tgatacactt gaaattgaag aaccgagagt agagcgtaag cacggaaagt
123121 tttggagaaa agattttatt gaagtaaata tagttagtca aagcgatagt ggtgctttag
123181 ttgttacagt tcctgatggt catcaattag gtgcggtaaa agtttcttca gtaagtggtg
123241 atacagattt taagagttta aatcttgatt ctttagttat ctctgttgta agcggagatg
123301 taagtctaag aacagtacaa actgacgaag ttaaattaac cacaacctct ggagatattg
123361 atctcaaaga agttacagta aaacaaggaa aagcccaact agtaagtggc gatttcacac
123421 tgaaaaatag tttagtcctt gatcaattta aggcttcaac tacttcgggc gataacttag
123481 ttgaagatgt aaaagctgct caatatcaac taagtacttt aagtggagat aattcgttat
123541 tcggaaaaaa tgaagcagct gccgaaggag atagtacaaa taatgctggt actattattt
123601 taagtacttt atctggtgat aatacggtta agtaatttag ttgaaaagaa actggctgat
123661 cattgaagat ttaaaaaatg gtttattttt aaataatgaa tattgaataa gcaaaaaaca
123721 aatttataaa aaactgttga catttatgat gtttatatta aaatgattat taacaaattt
123781 aattaatctg aaaacaaaga gaaagaagag tagcataatc ttaattttca gcgaatctcg

123841 ttaggtgtga ggagataaat tataagatta agtgaaaatc actttcgagt tctgattctt
123901 aattttaagt gagaagaagt gggagcaccc attatagtgc ctacgtatcg tcaagtaatt
123961 gactgcacgt gaaaaggtat tactagtgat agcaatacga acaaagggtg gtaccacgct
124021 gaagcgtccc tcaactaagt aatttagttg aggggctttt ttgtattcag agatattttt
124081 tggagggaaa gaatatgatg acagatcata catcacgaaa attaacatgg aaggattatt
124141 tagtggtcgc ttccttgcta tttggtttat tctttggggc tggtaacttg attttcccat
124201 tgcacttggg acaacttgct ggagcaaatt ggggtacagc cgcagttgga tttttaatca
124261 cgggtgtatt actgccttta ttatccgttt tagctgttgc aattacgcat gctgatggcg
124321 tttatgatat cggaaaaccg ctaggtgctg gttttgcagt tgtatttatg gtcttgatcc
124381 atgctacaat tggtccttta tttggtacac cgagaactgc aactgtttca tttacagttg
124441 ggattgcacc atttgttcct aaaaatatgc aaggaacagc cttattaatc ttttctgctt
124501 tgtttttctt agcagctttt ggtttttctt accatcaaaa caacatctta tctaatgttg
124561 gtaaagtatt aaacccacta ttcttatctc tactattctt agtattcctt gtagcttttg
124621 ctcgtccatt aggtaaccca caaaccgcag ctgtaactgc tgcttacaag catggcgctt
124681 tagtgaacgg attcttagaa ggatacaaca caatggatgc tttggcagga ctagccttcg
124741 gtgttactgt tgttacagca gtacgcggaa tgggacaaaa gaatgctaaa agtgtttcaa
124801 aagttgttgc taagtctggt ttgttagctg tactagctat cggttttatc tacttattat
124861 tgatcttaat gggtgcaatg tcattaggcc gctttaaagt ttcagacaac ggtggtgttg
124921 catttaacca aatcgttaat gtttatggcg gtgtcttcgg tcaagctcta cttgcatttt
124981 tattaactgt tacttgcttg accactgctg ttggtttagt tgctgctttt gcgcaagact
125041 tccacaaaca cttcccacaa gttagctacc acgcttggct agcacttagc tgtttagctt
125101 cattcttagc tgctaacttt ggtttagata caatcattgc atggtcaact ccaatgctga
125161 tgttcttata cccattatca atggtattaa ttttattgtc tgtctgctca ccattgttta
125221 agactgatgg agttgtatat ttctttgtga tcgtctttac agttgttccg gccttgggcg
125281 atatggttgt agcctttcca agtgttgtca gtcaaagctc atttggttta gcagtagctt
125341 ctgtaagaaa ccatttaccc ttagctaaca tggggctttc atggcttgtg ccagctttag
125401 ttggtttggc aattggatta gttgttcatt ttgttaaaaa taaaaaatta acttcaactt

125461 tagaagaaga ttaattgaaa agatctgctg attaacttca gcagatcttt
tttatctaaa
125521 gcacaaagat gacgttgatc gaaaatttat cttagatttg atttcaaagg
aattaactga
125581 cttagcaact tatttttaga catttttggc aattatgtag tgttaaattt
gcgtatttct
125641 tcataaaata ttttatgagg taataattat gtcagaatat aaagttcata
taattaatac
125701 tggaaaagtt agaattgagc ctgaattagc atttggtgga gatcattgtt
caactgctaa
125761 ggctgccggc ttttcaatta aaagatcacc taaaatttgg ttaccagtaa
atgtgttttt
125821 agttgaaaca ccagataaat tagtcttgct tgatacaggt tgggaccatt
caatgagtcc
125881 gaatggcgtt tttgataaaa aagctcaaat aaagtcctta ggttcttcac
ttttatatca
125941 tgttaatcaa ggagtggttg caaaaaggat gacagcaagt gaatagttag
ctaagttagg
126001 aattaagcca agtgatattg actatgtcat tatttcacat cttgactgtg
atcatgctaa
126061 tggattacgt caattcaaag atgccaaaaa tattatggtg gcacggtcag
aatatgagta
126121 tgctaagaag catccctata ttcgttttaa gaaaaaatgg tgggacgatg
tagatttgaa
126181 tctgtatgat tggaatgata gtcaaggccc atttggtaga tcttatgatt
tatttggaga
126241 caagagtatt gaactcatta atatcccagg acacactgct ggattagttg
ctaccaagat
126301 cactaatagc gatggcaaat atttccttta ttatggagat ggtggctatg
gtgaaaaatc
126361 ttggaaggaa atggtaactt ctggtatttc aatggataaa agtgctcaaa
agaagtcgct
126421 tgagtggatt agaaaagaaa gtctagatcc aaattgtgta agatcattag
cttgtcacgg
126481 gattgaagaa aagcctgaag tatttgaatt ttaagtagta aattctagtt
aaacaagtcc
126541 ttatgcacat cattaaaatg catcccttta tatatatcag gggtgctatt
tttgatggcc
126601 caatttgaaa ttgtatttaa aactggtagc agcgtttttc ccttatttgt
taatgaatat
126661 tcgacgcgaa tcggcatttc attatattgt tttctttcaa tgatatcttg
ttgctgtaat
126721 tccttaagcg aatcagctaa tgctgcatca gaaatatttt ccatctgatc
ttttatttca
126781 ccatatctca tagacttttt gtgtcctaag atacacaaga gtcttggttt
ccattttccg
126841 ccaaagatat ctaatgcgta atcaacagta caatagtatt cttttggtaa
ttttctttga
126901 tacataattg acctcactca ataattcttt agtaactaaa gctcttttta
atatatataa
126961 atcctttcaa ctttatctta ctataaaaat gtaagttgat taaaacagaa
aggaaaattg
127021 atatggcagt aaaagcttat ttagaagtta cgatgcagat cccagcagaa
aatagaaagg

127081 cagctgcaaa ggtttatagt gattaccgta aaccattttt agaaacaatt ccaggcgctg
127141 aaagtaaggc attacttgtt cgcgatgaag atgttcaggt attacatggc tttgattcag
127201 tagaacatgc gcaagattat ctcaagagtg aaatgtttac taaacatgtt tttccaggct
127261 taaaacctac ttggactgct gaaccagaag ttagaatttt ttcagttgtt ggatagaata
127321 ctaaaaatat caaacgttct agtttgatat tttttattag gtaaagaaaa aatgaaaata
127381 actaaaatcg atctattata tagtgatcca gtagaagata attggcggcc gtctttttgt
127441 cgtatttata ctgacagggg tatttatggc gatggtgaag ttgctctatc atatggcgga
127501 gcaaaagaag ctgcttttat tgaaatgaaa gaattagctc ccatgttaat cgggatgaat
127561 cccttagagc atgaggtcat ctggcagaaa ttatacagaa atagtttctt tggtaaaaat
127621 gcaggtcctg taatttttgg cgctatttca gcttttgata ttgctttatg ggatattaag
127681 ggaaagtatt atcaagctcc actgtgggaa ttacttggtg gcaaacaacg agatcattta
127741 aaagcttatg ctagtcagct ccaaatggga tggggagagt caagaaagcc tgcagcttca
127801 gctcaagatt atgcaaataa tacaaaaatt gccttagaaa agggatttaa gactgttaaa
127861 attaactttt taacatttga cgagcgagga aagcaggttc catggcaaaa gcagactgct
127921 tttttatcga ataattattt aaatttagca gaagagcgaa taaaagcagt tcgtgatact
127981 ttaggaaaag atggcgaatt aattttagaa aaccacgctt taactgataa gttatcagct
128041 gttcagtatg gaaaaatggc gtcgaaatat aatattttgt attttgaaga accagtaaaa
128101 cctgatcctg atctattaag ttatgttcat caagaaacag gtctaaatat tgcttctggt
128161 gaacgaatgt attctcgttg ggactttaag cgtgcttttg atcaaaatag cattcaagtt
128221 gcgcagccag atatttgtac ggctggagga gtaactgaag ttaaaaagat atgcgatatg
128281 gcagatacaa gtgagcttgg tgtacaaatc catgtagccg gaagcaactt agcaactgca
128341 gtgtcactaa acttagaagc agcactccct aactttgtga ttcatgaata taacgtcaat
128401 actgagatgc caaaaatgct taagttaact aaatatgatt atgaacctaa caatggtgta
128461 ttcactattc ctaacaggcc aggaattggt aatgaaatat ctgattatgc ttttaagcat
128521 agtagggtag ttacggttaa gtgaaataga aatttctgta cttgatggtg gtttggttcc
128581 aactttatat taaaaaatgt ttatctaaaa tttaaaagta gaatttaggt caaaactgat
128641 ctgaattctg ctttttattt gaatttaaat agattaaatt aatgtttttt taattttgta

128701 ttgaggttta aagcttaaaa atataaaatt aatttaaaat ataattaaaa tacaaaacac
128761 ttggagaaaa agtatgagca ttaagcaact atttaaatca aataaaaaac agtttatttt
128821 gatttttttta atggtgatta ttggaatggc gagtgactca ctatctcaat atttaatgac
128881 gccagcgtat aatcatctac gaaacatgaa tttattgggc tttattctgt ttatgtgttt
128941 agctctaggc tgtgatggtg tcagaattat actaatatca agatcagatt atttgtatgg
129001 caaagacgta cagaattatt tacaccagat ccgtgccaga attagtcgct atttctttaa
129061 aaatgatatt aatcagactg caaaagtaca aaataatatg gttgcaaatc ttgatcaact
129121 aactactaaa tacttaactc cgattaaaaa tggctttatg tattttttag ctgtagtttt
129181 ctcaattgga attctatttt cttttaattg gattctagtt gttgtaactg tgattttaac
129241 tattatttca ttattgttac ctaaggcctt tgaaaaacta acttctacgg caacagttac
129301 tgtcacaaag aaaaatgaaa aattcctcaa tattttggat aaatgggtaa aaggtctaga
129361 tgaactaaga agatatgcaa gtttcgatat ttttactagc tcgattaata atggggcaaa
129421 agagtataag aaagccgcaa ttcatcaagg agcaacgatt gcaattgctg atatggctac
129481 agcgcttgta aatattggcg gtcaaatgtt gcttctaggt ttatgtgctt acttatattt
129541 gcaaggtaga attgcttttg gtgctgtaat tactactggc caatttagtt caacaattat
129601 gaatggtgtt gcagcatttg tggcccaatg gaatttaatt aaatcagcta aaggcttaaa
129661 tgaagaaatc cttgagctgg agcaacaggt tgaaataccc aaggatcaac atgatgatca
129721 aaaaattgcc aagatagaaa taaaagattt aaccttacaa tttaagaatg gcgaaaagat
129781 ttcatatcct aatttaactt tcaataaagg tgaaaaaatt ctaataactg gcgatagcgg
129841 aagtggaaag tctaccttat ttaaattaat cttaaataaa ctcactccaa gtaaaggtac
129901 aattcgtttt attgatcagg atggtaagga agtatattta aatccggata aaattggata
129961 tttggcacag gatagtaccc tctttccaga cacgcttgaa aataatatca ctatgtttaa
130021 tgctgagctt aataatcaag taaaacaagc aactaaaaaa gttgatttag ataaagatat
130081 tagcaacttc cctgaggggc tagctacaca agttgattta gataagaaca atttgtctgg
130141 tggtcaaaaa caaaaattag tcttagctag agcgttagtc cataattctc cctggctttt
130201 tattgacgag ggcactagtg caattgatag caaatctacg aagcatatct tgcaacatct
130261 tttaactaca gatggttctg taattatgat tgcacataac ttttctaacg aattagtttc

130321 aatgtttgat cgggtagtaa aactagatga tggggggcac taatcaatga
gctttaaaga
130381 ctttataaag acgaattact tcagatttat ttacatgaat atcttgtcag
taatttctgg
130441 tgctgctgtc attggtgcgg gatatgtaca aatgtacttt ttaaccgatg
ttaaaaacaa
130501 agattggcgt ggaattttgg ttactgtcct tttgataggg cttttgtata
ttggcactca
130561 ggggatgatc tactacattc aatttgaaat tcgaattcaa gaagaagaat
ataatcaaca
130621 gatccgcaat aaacttgcta gccattactt cttggatcaa agaaaccaca
aagttgctga
130681 tgtacaaaat agaatgacta atgaccttga aatggttaga actaatttct
tcgattggta
130741 cattatgatc ccattttatg gagctatctt tgtatcagcg ttagtagcat
tactatccat
130801 taactggatg atctttttag caagtattgt aattgatatt atttcttatt
ttattccaaa
130861 actaattcaa aagaagatgg agcaagctac aaccaatgtt tccaagcaaa
acaaatatta
130921 tcttgatgtt ttggcgaaat ggttttctgg ccttgaagaa ttaagaagat
actttgctgg
130981 tgcaaaacta tttaaaattg gtgagcaggc ttcaaataaa gtagagaacg
cacatattaa
131041 acaaacagct gctcagcaag aattaattgt tttaagtggt ttttgtaact
tagttggaca
131101 aatgttatta ctttcattaa ctgcattttt tattacaaaa aatgctatta
tctttggtgc
131161 tatcatgtct gttcaaaact ttgccgctaa tgtctcaata ggtttgcaac
aggtgattga
131221 aggtttaagt ttcatgaaat cttctaaaga gttgatggga gaaattgatc
atgattcttc
131281 tgtaattaat gtagataaag tagaagggga agaagcacca tttgccttta
ccactaagaa
131341 tttatcatta aactttccaa atggagaaaa gctaacattt ccagatattg
atgttaagcc
131401 aggggagaag atcttattaa gtggagattc tggtgctgga aaatctacgt
tgtttaagtt
131461 gattttaggt gatttaaagg caagtacagg caaagtcgaa tttgaaaatg
cagctggaga
131521 cacaataatc cctgatatgt cgagaattgg ttatatccca caagacccta
atttatttcc
131581 tggtacaatt aaagaaaata tcactatgtt taatgataaa ttggatagtc
gagttgaaga
131641 aactataaat gaagtaaact tcgataaaga cattaaaaaa tttaaaaatg
gaattaatga
131701 agaacttgat ttagacaaaa ttaatatttc aggtggtcaa agacaaaaga
ttgttttagc
131761 aagagcaaaa attcatgata gtgatattat cttgattgac gaaggaacta
gtgctattga
131821 tcaaaaagca acaatgaata ttttgggaaa actgcttaat agtaaagcaa
caattgtatt
131881 tattgctcat aactttaatg agcatatgcg tcagatgttt gatcgagaaa
tttatttaag

131941 taaagattaa attcttttag actgtctact tggtgcaaat ttagtattag aagcaaattt
132001 tggtatttga tttttgcatc ttgttttaga aaaattacca ctcatttaaa aagtgaatta
132061 tttaagtctt tttttctgta ttctaatagt gaattcagaa aggaaggctt tttatgttaa
132121 ttgaaactta taatttaact aaaaaatatg gtaaaaagtt ggctttgaat aaagtcaatc
132181 taaaagttga tcgtggtcaa ctagtggctt atctgggaac taacggagca ggaaaatcga
132241 caacgattaa tattttaact ggattgttaa aaccaaccag tggaacgata acttatgcta
132301 aagatttaaa gattggtgtt gtttttcaaa gtagtgttct tgatgatgtc ttaagtgtaa
132361 atgagaatct taatttaagg gcgcagatgt atcgtgatgt ttcaaaagca tggcttgagc
132421 agctaattga tttgattggt attcggagtt tccttcatca aaaatatggt actctttcgg
132481 gcggtcaaaa aagaagagta gatattgcta gagctttaat tgatcatcct gatattttgt
132541 ttttagatga accaactact ggtttggatt tacaaacgcg gatcgtaatc tggaatttac
132601 tgcagaagct tcagaaagaa cagggattaa cgatttttct gacaacgcac tatttggaag
132661 aagcagaaaa tgcagatcaa atgtatattt tagaaaatgg acaagtacta gctaatggat
132721 cggcttatga gattaaagat aaatatgccc caagtaaatt agtaatcacg ctaaaagaaa
132781 agcaacgatt aataactaca tatccagtta ttgaaagaaa taaagaaact gaaattgatg
132841 gacttgatcc tcatgaagta attacgctgc tgcatgagaa tcaagctagt ataaaatctt
132901 ttgaataccg acaaggttcc attgatgacg cctttattaa aatcgctgga aaggagctac
132961 aataatgttt gctttaatta aaagaaatac taaaatttac tttgctaata aaccaggcgt
133021 aattatgtca tgctttgggg cattgatttc atttgtaatt tatattggat ttttacagca
133081 aaatcttgtt aattcctggc aacatgttag tcacgcaaaa caattattag atctttggat
133141 gctaggcgga attgtatcaa ttgccggaat tactacatct tttcaagcac tgggtcaaat
133201 ggttaatgat aaagaaaatc gagcaattga tgacatgaaa ttaactgctc taactcctat
133261 tagtgaaagc atggcttatg ttgtatcagc tactattgtc agctttctga tgcaaattat
133321 tacatttgct gttatgacag tatatttttc agtagttgat aaagcagatg tagtgcacaa
133381 tgcatattta ccttgcttag gcttcatgct tttgggcgca cttggcgcaa cattattaaa
133441 tttaatcatt gtaatgttta tcaattcttc gactactttt agccgtttat cagctgtgat
133501 tggtgcggcg gcaggattta tggtagcaac ttatatgcca tacggaactt tatcaaaaac

133561 tgctcaaaat atagtaaaat tatttccaag tagttatgaa gcagcaagtt ttagaagtct
133621 gcttttgaat aaattaagtc agcaagatgt gccaactaga atgcgagatc aattaattga
133681 atatttagga attcatttta aatttggatc tcatcagcta aataatttcg ataatgcttt
133741 agtaatgatc gggatgattg tacttttagc agttattatt gttatattgg cacttttgct
133801 taatagaaag aagaaatagt acgtggatgt cgaatttcat tcaaatccta atctagaaga
133861 ggatcaaatc gaagttaaag tgatttctgc aagacaaacg actaacgtaa ataatctttt
133921 atcgtatata aagaattttg ataaaaaagt agattctctt ttacctgtga aaactgaaga
133981 tcgaatttta acaattaaga tagaagaaat tatcaaagta gaagtcgaaa agacagaatt
134041 aagtttttat acgactaatg ctgtgattaa aactaatggt cgtttgtatc agattctgga
134101 aaagttaaat aatgattttg tccaagtatc aaagcatgga attattaatt tgaattatct
134161 tgaatcactt gaagcaggct ttactggtaa tatgattgcc aagcttgctt ttaaacagaa
134221 gacagatgtt tcaagaagat acttacctga gttagaaaag aagttgggat tataagatga
134281 aaatatggca aaaagcttta gactattttg taacaggcat gggatttggc gcaattattt
134341 atttagtaat tttgggagta acttatgaaa atatcggcga tctaagagtc cccttaaaca
134401 gtgtattcat agtatttgta tgtagtggat taattggaga attgagtttc ttatttaaaa
134461 catccttacc atatttctgg gcattgattt tacatctgat aggaactttt gcattgtttt
134521 caataatgat acttttaaat cattggttaa ttaactggca aactttactt atttttgtta
134581 ttacatatgc aattatttgg ctcataatta gattaattca agaaaaagac attcgccgga
134641 ttaatcgtca aattaagaaa cgtaacggtt gagtgataaa atattattaa ataatagctg
134701 atagaaagtg gcgtagcaat gtatccagat aaatttaatt taactcaaga acaaaatcgg
134761 cgttttgcaa agaaaaattt agtaagatta gttttcacta cttcacgttt tgaaggctta
134821 aatactacat taccacagac tcaaactatt attgacggga tgagtgcagc aggcgtttca
134881 attgacgata ctaacgtgat tgttcaatta aaacgtggct ggcaatatgt aattagggaa
134941 gataagcctt tgtctttaaa tattgagcaa aatattaatt tgctagtagc acgctatgat
135001 tcattagatc ctggtagttt tcgtactgga aatgtaacag tagaattagg aaatagtaat
135061 ggcgagtggc aaccaccaga aattaatttg gaccaagagc gtaaattctt tgatgaactt
135121 atgcaaaagg atacgtccat tactgataaa gctatgactt taatgtatca caatatgaga

135181 aatcagcttt tttgggatgg aaataaaaga actgcaactt tagcagctaa taaattaatg
135241 attgacaatg gtgctggatt aattaatgtg ccattaaata agtggtacaa gtggaatgaa
135301 ttaatcagtg aattttactt aactggaaaa atggataagt taaaggattg gacttatgaa
135361 aacggaattc agggaattga gttttcgtct gatcctgggg tacctaagcc aactattgat
135421 ccaaaagatt atgaataatc tttatccata ctttaaacgc ataataacct attacaaata
135481 aatattttgc atgatgttat ttctattcta aaataaaagc aaagttagaa atgaggtaat
135541 aatatggcaa aaaatgaaga agaagtaaaa aatagtgttt ttggatttgg taattcaaat
135601 gaagcttttg caaaattctt taagggtaca agttacctca aggctttagc agaaagtaaa
135661 gaagctaaga ctgcagttca taatgtaagt tttgaaccag gttgtcgaaa caattggcat
135721 atgcacacag tgccgcaaat cttaatcgct gtagctggtg aaggctggta tcaagaagaa
135781 ggcaaagctc cacaaaagat gctgccagga gatgtggtaa ttgttaatcc aaatactaag
135841 cattggcatg gagctacaaa agattcctgg tttgctcatg ttgttgtgat ggtaggcgac
135901 agtaagaata catggcttga acctgtttct gatgaagaat atgataaatt agactgatta
135961 atcggaaaat tcaatatgta aattataatt aaaccgaaaa attcctcttt aaagaagata
136021 aaactttaca tgtctatctt taagaggatt tttttatttt tgaaagtatt gtttaactaa
136081 tgagaaatta ttactgttag ctgttattag atcaatagtt gtttgtgctg attttaaatt
136141 tgcttacaaa tataagtcta atcatctgga gtcaattgat attagaaaaa agaataaaat
136201 taaaaacaag ttagaataat attgactttg tttaatttat tatgtaatat acttactaaa
136261 taaatggtat gaataaccac taagaatagg aaattagctt tactttaact agtcagcgaa
136321 cccaagttag tgtgagtggg tatagggaag taaagtgaaa gtagcctagg agtgcttgaa
136381 gtgagagtaa ttgtaacttt aggagggtgc gcccgttata gcgtcaacgt atctagtttt
136441 tctatgtacg gggtaaggga ataatagtaa tattattcta aaataaggtg gtaacacgcg
136501 tgataacgtc cttataagct ttatgcttat agggacgttt ttttatacct agtattaata
136561 taatttttat cgaaaggaat attaaaatga agataaaaaa agcgagtatt aggcagtcaa
136621 tgattattgc atcacttatt ttcggaatgt tatttggagc gggaaatctt atttttccag
136681 ttcatttagg tcaattagca ggtagcaatt ggctcatagc gagtagtggc tttcttgttt
136741 ctggagtttt gctgccatta atggcgctat tggcaattag tattactagg tcaaatggaa

136801 tctatgatct tgcattgccg aatggtcgtt actatgcact tatcttttta atccttgttc
136861 acgctacttt aggaccacta tttgcaactc cccggacagc cactgttcct tatgaaatcg
136921 gaattgctcc ttatttatct aagtcagcta atacttgggg actattaatc tattcagcaa
136981 ttttctttag tttagtttat attttttcaa atcatgaagg taagattact actttaattg
137041 gtaaagtctt aaatcctttg tttttgttat tgctgtttct gatctttttg ttagcattta
137101 tacatccatt gggttctgga aatactgctg ctataactgc ttcatatgca aggcatgctt
137161 tttccaatgg atttttacaa ggttataata caatggacgg tttagcagca ttagcatttg
137221 gaattactat tattacagca attaaagaat taggtattaa aaagacaaga caggtttcaa
137281 ttgaaacagc caaaagtggt tttctaggaa ttgcaggagt tgctatcata tacttggctt
137341 taatttggtt gggagcaact agtttacatc aatttaaatt agcagcaaat ggtggaatta
137401 ctcttgctca gatttcacat cattacttag gaactgctgg agatgcgatt ttagcaactt
137461 tagcaaccat tacttgtatg acttcagcaa tgggattagt aattgcattt tcacaagatt
137521 tccatcgtcg ttttcctaaa atttcatatc gaacattttt gcgaattaat tgtggtcttt
137581 cattcctatt tgcgaatttg ggtcttgatc aaattattgc atggtcaaca ccaattttaa
137641 tgtttcttta tccactagca atcacattga ttattttagg attactttca ccaatattta
137701 agcatgaccg acttgtttac cagattacaa ctgcgctgac tctaattcct gcattctttg
137761 acatgttaaa tgcattaccg gctaacttac atgacagcca attattgcag acattacttg
137821 ggtttgctca acaatatttt ccatttttca gtttgggctt tggttggtta acttttggta
137881 ttgcgggctt aatccttgga ttgatttgtc atgtattaaa agtaagaaaa cgtttaggac
137941 tacttagcga atagtgtgct ttataatcaa atacgaagtt tacagtattg tgtaaaaaag
138001 gtaatacaat tattggagct attgcagatg gtgaccttgg tattgattat ctgtttagat
138061 caatttattg taatctcttt tcatttaagc ttagaatatg aagtgaaagt attgctttga
138121 tcaaaggaga tatctaatat gtgtacatca attttataca gtcctaaaga ccattatttt
138181 ggcagaaatc ttgattatga aattgcttat ggtcaaaaag tggttattac accaaaaaat
138241 tatgaattcg agtttaccga tttaccagct gaaaaatcac attatgcaat gattggtgta
138301 gctgctgtag cagacaatac tccattatat tgcgatgcaa ttaatgaaaa aggattggga
138361 gttgctggtt taagctttgc tggacaaggg aagtattttc caaatgcagc agataaaaag

138421 aatattgctt catttgaatt tatttcatat ttattagcta cttatgaaac tgttgatcaa
138481 gtcaaagaaa gcctaactaa tgcaaatatt tctaatgtga gttttgctaa aaataccccca
138541 gcttcagaac ttcactggct agtaggagat aaaactggta agagtattgt tgttgagtca
138601 gatgaaaagg gcttgcatgt ttataacaat ccagtaaatg ctttgacgaa cgcaccttta
138661 tttccagagc agttaactaa tttggcaaac tatgcttcag ttgttccagg cgaacctgat
138721 aataattttt tacccggtgt taatcttaag ttatatagtc gtagcttagg tacgcatcac
138781 ttaccaggcg ggatggattc agaatcacgt tttgttaagg tatgttttgc cttgaatcac
138841 gcaccaaaag atagtgatga ggtagaaaat gttactaatt tctttcatat tttagaatct
138901 gttgaacaag caaaagggat ggatcaagtt ggtcctaata gttttgaata tacgatgtat
138961 actagctgta tgaatctaga aaaaggaatt ttgtacttta attgctatga tgacagtaga
139021 attagtgctg ttgatatgaa caaagaagat ttggattcat cagacttagt tgtatatgat
139081 ttgtttaaga aacaagatat tagtttcata aattaatgaa acatttctta gttagaaatt
139141 attatcaaaa tcaataaaaa atcgcgccca taatcaaata atcgatatca aatgaactaa
139201 aatgatatcg attattttta attgagcacg attatttgta aaaactaaaa tattaattta
139261 attgttgcca tctctttttc aaggtaaagg caatatcttt aggttcacga tcgcgagtaa
139321 aaattccttt atcgttacca ccaacacgca tgattccttc actcgtctta aagtcagcaa
139381 agttccaaac taattcgcca caaataaatg gatatttctt aaatatatca aaatacattt
139441 gataatattc attttgatat tcttggctcc acatttcatc tggaaggcga tgagaagaag
139501 ataatgtatc agcgccaaac tcagtaaaga caaatggttt attaagcttt aagttttgcc
139561 agccgtctag ttcatcttcc agcatctttt tagcattaac gatttcagga ccaccagcaa
139621 cgtaccaacc ataataacgg tttaagcaga caaagtcaca aagtgggtga agcttatcca
139681 cttttggacc gctacccata actaaagttc cagtataagg acgattttgt ggatccaatt
139741 ttctcgcaaa ggcaaaaata tctttgaaat agtcatagga ttcttgagta gttgattctg
139801 gttcattgaa taaactccag gcaatcactg aagggtgacg ctggtcgcga tcgatcattt
139861 cttttatttc ttgttcatgg accttttttta attcaggcac agttttcgaa gcaaaaaagt
139921 gcgactgatt agaattagtt acattaagaa agttagttat tgaacgatta agaccaacag
139981 cgggtacttc atcaataatt aaaaagccat atttatcggc atattgatac cattcttcgg

140041 cgtaaggata gtgactgctt ctaaaacagt tagcgccaat ccatttcatg
cattcgtagt
140101 cgcgtttgat aatgctttcg ttaactgctt tgcctaaaac attaaaatct
tcgtgcttgc
140161 caaagccttt taaataaatt gggtgattat tgagcaagat tttatcattc
acaattttaa
140221 ctgtgcggat accaatttta tctgtgtatt cgtcaactgt ttttccgtct
tcgagcattt
140281 caatctttat tttgtatgaa tacggatcgt ttggactcca aaggtgcgga
ttcttaattg
140341 ttaaactact agtattttta gaagtagcac acgctacttc tttttgatta
tcgaaaagtg
140401 ttactttaaa ttcagcatta ttatttgcct cgatattgta ggtaattgtt
gccttattat
140461 ttgctaattg ataatttagt ttaaaattag tgatttggct ttgaggaagt
gctaagagcc
140521 agacattccg cataatgcca gaataattga agaaatcaaa ataaggttga
gcaagttttt
140581 gaccgttatc taagatttct tcggtgccgc aaggaatagc tttttcagat
aattcgttat
140641 tgactaaagc agttacacga ttagtttggt cgtaattaat ataatttgaa
atttttactt
140701 gaaatggtaa aaaaccacct tcatgttgac cgacttcatg tccattaata
aatacttttg
140761 cgcgatgagt aaccgaacca aaacggatat aaagttcttt cttctttaga
aatgaaggaa
140821 taaagaagtc tttttgatac caaaagtctc cagtatagta tttacggtct
cgcttagtag
140881 ttaattctgc aaacgtacct ggtacaggca tttcttcagg atcaggcaac
tctttattcc
140941 atccctcgtc aagaccaaca gagttaggat cagtttcaaa ttgccaagtg
ccattcatta
141001 aagtgttaaa ccgatattta ttttgaattg gatatagtgc agattccata
gatgattttc
141061 ctttctagta atgttttctc ttatctatat tttacgcttg gggagagtaa
atagatttga
141121 aaatggttta ataaattaaa tcccttgact gtaacctcgc gtcacagttt
attttattta
141181 tagagaataa ttaagaaagg aatgttgaga gtggccaatg ataaattatt
atcaatttct
141241 gaatttgcta aacttgcagg cactaccaga cgaactttga tattttatga
tcaaaaagat
141301 gtctttaagc caatgaagat cgaagaaact ggttatcgct actatagtta
tgagccactc
141361 tatcaaattg gttttatctt gggattacgt gatttaggat tttcggtaga
agaaattaaa
141421 gattatttgg gtggttctag cagggatgcc ttgaataaaa aattaatttc
tttaaagaat
141481 aagatcgaac ttcgtatcca aaatttaaag caactcctag ctattttgcg
tcaaaaagaa
141541 aatgacaact ttcaactaac tgatgtcgat ttttatgttg tgaaagaatg
ttttttacct
141601 gatcacgact tttggtgttt ggatcctaag gctgattgtt ctgaagcaga
aattgcctct

141661 tcatgtaatc agttttatca agaacttggt acaggggtta tggccaataa gaacttatct
141721 ggatttatca cagatttacc tcaggcaaaa gctaatacct atgccaattc cggttttaga
141781 attattaaaa aaatcataca atcaaaaagt caacatcccg attattagtt aaaatgatgg
141841 caattacttg attgttaagg tggaaaatac tggtgcagga attgaaaaag ggattagcta
141901 caattagaaa atataccaaa gaaaataatt taaaaattgg caatgacttg tggcagttca
141961 atattggctt aaacattgag aaacttggtt taataaaaaa tggtatttta gcatataaaa
142021 ttttaaaata aagaagaaat tgaagcttta gataggagtt taatttggaa ttaccaactc
142081 aaatagaaat taaaggtgca agagtccata atttaaaaaa tattgatgta aatattccac
142141 ttcataaatt tgttgctatt tcaggcttgt ctggttcagg taagtcttct ttagcaatgg
142201 gaattttata tgaagagggc tcaagacgat atttagatgc actgtctact tacatgcgtc
142261 ggcgaattaa acaaggtgca caagctgacg ttacttcggt taaacatatt ccgtctgctt
142321 tagctttgcg ccagcgtcca agtattccaa atgaacgagc aactgttggg acaacgaccg
142381 agacttttaa tgttttgcgt ttgatttttt caagattagg ttcgccagtt tgtcctaacg
142441 gtcaccgcgt agctccaagt ttagatatcg cggaagctat gtctaagtca ggtgaagaaa
142501 tgggacaaat tacttgtcca acatgcggtg ttaagtttta tgtaccaagc gctgaagatt
142561 ttgcttttaa ttctgatggt gcttgtcaag aatgtggtgg aactggaaaa gttcgtcagc
142621 ttgatgatag taaactaatt gctgatccta atttatcaat tgaggacgga gcagttgctt
142681 catggagctt gccaggaaga aactttatgc caagcgtagc tcaacatgct ggagtaagga
142741 ttgatattcc ttataaggat ttaacggata aagaaaaaga ttttgtctta aatggtcctg
142801 agaaaaaata taagatggac tttttgtctg gaactggtcg agttttccat gattttaatg
142861 cattgtatga aaatgctcat caggctgtgt tgcgctcagc taaaagcagt aaaagtgaac
142921 gtgcacaaaa gagaattgca gaatttttca cttattcaac ttgtccagtt tgtcatggta
142981 cgcgtcttcg tccagaactt ttgaagcagt tagttaatgg taaaaatatt tatcaagtcg
143041 agcaaatgca gctaggggat ttgccagctt ggagtaagga agtattagca aaattgcctg
143101 aagatatgca taaaatggct agctctttga tcaatgaatt tcttaataat ctgcagccgc
143161 tgcttgattt aggactagat tatttaacta tggagcgagc tggcaacact ttatcaactg
143221 gtgaacttca gagaattcaa ttagcgagaa cactacgtac agaaacaact ggcgttttat

143281 atgtcttaga tgaaccgtcg attggcttgc atccagctaa cgttgatggc ttaattaaag
143341 ttttgcgcaa attagttgat cagggtaatt ctttagtagt tgttgatcac aacgttgata
143401 ttattaaagc tgctgatgag attattgaaa ttggtccagg ttcaggtgaa caaggtggtc
143461 aaattttaga tcaagacagt cctgaagaaa ttaagaaaga taagcagtca ctaattgcac
143521 catatttaaa tggaaaagca catttaatgg ctagaaaagt tagcgatcag gttaattcag
143581 atcaaattag ttttgaagta gaagattact tcaacttgaa agatgttcaa gcaggtattc
143641 ctttgaatca attaactagt gtgacaggct tctctggagc tggaaaaact agtttgattt
143701 tagattcatt agttcctgca attaaggcac aagataaaag tgaaccacta cctaaacaaa
143761 ttaagaattt tgattgtcca attaataatg tggttagcgt ggatgcttca ccaattggca
143821 agagtacgcg ttcaacagtt gcaacttata cctcaattat ggataatttg cgtaaacttt
143881 ttgcaaaaca gtctttggct aaagaaaaac attatacgcc aagctatttt tcttacaaca
143941 ataaagaagg tgcttgtcca acttgtggcg gaacaggaat tgttacttta gatattcaat
144001 tcttgcctga tatgcagcaa acttgtccaa cttgtcatgg cgaccgctat aatcttggaa
144061 ttcaaaagat aaagtggcat gatatgtcaa ttgtggatgt tttgaatctt gatgttaacg
144121 aagcgattcc tgtttttaag cgtgtgccaa agattgaacg tgatttgcag ttgcttaaag
144181 aagttggttt agattacctg cacttaggtg aaagtacgcc aactttatct ggtggagaag
144241 cacagcgttt aaaattagtg actcacttaa gtcataagca agacgatact ttatttgtct
144301 ttgatgaacc aacaattggt ctgcatcccc tggatgttaa agtcctagtt gaagtcatgc
144361 aaaaattgct tgatcaaggt gcaactatta ttacgattac acatgacctg aatttagttg
144421 ttaatgcaga ttatatgcta gatctgggac cacgaggtgg tgaaaatggt ggtcgtattg
144481 tagcgcaagg ccaaccacta gaattaatta aagatccgca aagtttaact gtagagtatt
144541 taactaatta tctaaaacaa tttaacttag attaattatt tgatgtatgg atttagagta
144601 aaagtaaaga atttataaac atgcttgaat aataaagaat aattgtttaa tctattaacc
144661 gttaattgaa taaagaagca aatgaactgt taggtgagac tcctacgtaa atatacgctg
144721 atgcccagca acatctagag atgccaacgg gttaaacagg agttgtcgac ttaaggcttt
144781 tcccagtcag ctagttgaaa aacttcacgt tacgtagtgt taaaactgaa cgacgagtat
144841 aaatcgctaa attaagacca atgcagtttt tgcgttggtc tttttgtttg tttgccgatt

144901 ttttagaggg agagattact atgttaaaat tcaactctaa agatcgcgta gtaactaatg
145961 cgactttggc taaacaaatt gctagacaag aaaaaaatga agtcttaaag caattgcata
145021 caacaattaa tggattaaat cctgttcaag caaaacaacg cttggagcaa gatggcttaa
145081 atgaggtctc gaataaagaa catcatccaa aattacattt tttaatcgat gcttttatga
145141 cacctttttac tggtgtgttg ttatttttag caggtttatc ttttttaact aactatgttt
145201 ttgtgccagc tgatcaaaaa gatttgagta ccgtaattat catgattatg atggttctga
145261 tttcaggaat tactagtttt attcaaaatg taaaaacaag tgatgcggtt gactcattgc
145321 taaatatggt ttctgtaact acaaatgtta aacgagctgg cgaagataag gaattaccaa
145381 ctaaagatgt agtagttggt gatattatta atttatctgc tggagattta gtgccagcag
145441 atatgtattt gttaaagagt aaagacttat tttgttcagc gagctcatta aatggagagt
145501 caagtccagt tgaaaagatg gcagatcaga agccaaaaga aagtgataat tacttagatt
145561 atccaaatat cctttatgaa ggaacaaata ttgtttctgg ttctggaatg ggcgtcgttt
145621 ttgctacagg tgataaaacc gtttttggaa atctggcccg tactcttgct cagaataaaa
145681 acaaagagac aacttttgac attggaatta aaaatgtttc taaaatatta ctaatcatga
145741 cagctgtaat tgcaccacta gtgtttttga ttaatgattt aactaaaggc gattggctaa
145801 atgctttaat ttttgctatc gcaacggctg ttggtttaac gccagaaatg ttgccagtta
145861 tcgtgaccag caatttagtt aaggggtctg ttgaaatggc taagcatgaa accattgtta
145921 aaagaatgaa ttcaatccaa aactttggct cagctgatat tctttgcact gataagactg
145981 gtaccttaac acaagataaa gtggttttgg aacgacacta taatttaaac ttagaagaaa
146041 aaccaaaagt attagagctt tcatacttga atagttatta tcaaacaggg atgaaagatt
146101 taattgataa agcagttatt gatgctgctc aaggagaact agatacaaag aaaattaatc
146161 aaaactacca aaagattgat gaaatacctt ttgattttaa gagaagacgt atgagcgttg
146221 tcgttatgaa ccagcaacat gaacatattt tggtaactaa aggtgctgct gaagaaatgc
146281 ttgcttgttc taacagagta gaaatagatg gtgcaataag tccacttgat gaagatcaac
146341 gtcagaaaat tttaactgaa attgatgaga tgaatcagga tggtttacgt gttgttcttt
146401 taggatataa agaaaatcca gctcctgttg gtgaatttag cattgatgat gagaatgaat
146461 taatcttagt tggattttta gcatttttag atccaccaaa agacagtgct aaggcagcat

146521 tgactgcctt aaaagaagat ggcattacag tcaaaatttt aactggggat aatgaagcag
146581 ttacgagaaa tgtaggaaag caagtaggtt taaatattga tcaagtttat cagggaaaag
146641 accttgaaaa taagactgac gctgaattaa aacaaatggt gcaagcatgc gatgtttttg
146701 taaaattatc tccacaacaa aaagctcaga ttattgactt acttagacaa gatggtcata
146761 ccgtagctta tatgggagat ggaatcaatg atgctcctgc aatgaaagcg gcagatgttg
146821 ctatctcagt tgatacagcg gtggacattg ctaaaaagtc agcagatata attttgctcc
146881 acaaagattt aatgatttta gaaaagggag tacaaattgg ccgtcaagta tttggcaata
146941 cgatgaaata tattaaaatt acgctttctt ctaattttgg caatatttta tcaattttag
147001 tagcttcttc atttttgcca ttcttaccaa tgcttccaat tcaactttta attttagact
147061 tgatgtatgg aactagttgc ctatctattc catttgataa tatgaataag cattatttat
147121 cagagcctag aaagtggagt acaaagaagc taccgaagtt tatgttctac tttggtccaa
147181 cgtcctctat ttttgatatc ataacttttg ctttgctttt ctttgtggtt tgtccgcaag
147241 taactggaac tagttatgca gcggctagcg caacgcaaaa attaattttc tcagccatct
147301 tttgtacagg atggtttatt gaatcacttt ggacccaaga aatggttatt catgcactta
147361 gagatcccaa attaccattt attaagcaac atgctaccgc aattgttact tggtcaacaa
147421 tcgggatggc agtagtggga actgtcttac catttattac accaatagct aaagcaataa
147481 aatttggtcc aattccaatg tactttttag caattgtatt tcttttgtta atcttatata
147541 ttgctttaac tactttagta aagaagtggt atttgaaatc agaaaaatat ttgatttaat
147601 caacaagaaa tattttcttt aaagaatcat gggttaattc cttgatgcaa ccaatttcta
147661 acatgttaat gatgattttg gtaatcggta ttttaaccta tggtgcaatt cgtgtaatgc
147721 aaggcagtct tactatgggt gccctaattt catttttaat gtacttgttc caattaatgg
147781 gaccagtaat tatggttagc caattcttta acgaattagc caagacatca ggttcaactg
147841 aacgtattcg tgaaatgctc aatgaaccca ttgaagaatc tgctgatgat caaaaaattg
147901 atgttgctgg taaagaattg caattgcaaa atgtcagctt ctcttatgaa aaagataagc
147961 aaattttgca taatattaat gtagtggcta aaccaaatac ggttgtcgct tttactggac
148021 catctggtgg tggtaaatca acaattttct cattgattga acgcttctac aaaccaactt
148081 caggtaagat tacgattggt aatgaaaata ttgacaatat cgatttaact aattggcgtc

148141 atcaaattgg tttagttggt cagaattctg cggtaatgcc aggaacgatt catgaaaaat
148201 tagtttatgg attagatcat gaagtaaatg atgacgaact atggcgtgta ctaaagatgg
148261 catatgctga taaattcgta aaagaatcag aagatggttt agatactcaa attggtgaac
148321 gaggggtaaa actttctggt ggtcaaagac agagaattgc gattgctcgc gcattcttac
148381 gtgatcctaa gattttgatg cttgatgaag caactgcaag tcttgactca gaatctgaag
148441 caatggtgca aaaggcttta acaagtttaa tgaagaatag aatgacatta gtaattgcac
148501 accgtttaag tacgattgtt gatgcagata gaatctactt cattgatcat ggtacagttt
148561 ccggttctgg tacgcacgaa gaattagtta agtcaacacc gctttatgct gagtacgttc
148621 ataatcaatt taagaaataa tatatagaaa gaagctgatt ttatatctag gatttccaat
148681 tgttagtttt attgtttcag tgggacctaa gcctggtcct agaactgaga atgataaaag
148741 taaataaact aacttaaaga gttgcttgga aaaagcagct ctttttttgtg tataagttaa
148801 aaaatggatg aaattgggta aaagtttaac tatagttaaa ctttcgagat aaatggggca
148861 taatttgact tatagttaaa cttttgatat tcgaatgctt aataaaaata aataatcatt
148921 tttagtatat gattgattat agaaacaaac tgaaaaaata tattgaggta actatgaagc
148981 aattaacatt aaataatggc attaaaattc ctatttttgg ttttggaact tatgaaatta
149041 gtcctgataa aacaaagcaa gcagtattat cagcttttga agcaggttat cgcttaattg
149101 atacggcgca atattatcaa aatgaacaac aagttggcga agcagtgaaa gcaagtggtc
149161 taaatcgcga tgaagtcttt attaccacta agacaatgac tgatggttat gaagatacta
149221 agattggttt agatgaatca ttgagaagaa gtggactaga ttattttgac ctagttttaa
149281 ttcactggcc gatgggatat gatattgata cttggcatgc ccttgaggca gcatataaag
149341 caggaaagac gcgcgcaatt ggaattagta attttaatag tcgacaaact ttagatctaa
149401 ttcaccaaag tagtgtccgt ccaatggttg atcaaattga aactcattta cttttgcaac
149461 aatggaagat gcacgaattt ttagaaaaag aaaatattgt gcatgaaagt tatagtcccc
149521 ttggtaatgg tcaacaacat ttgatgggta atccagtttt aacagaaatt ggcgaaaaat
149581 atggcaagtc atcaattcaa gtaattctac gttatctagt ccaaaataat gttgtaacta
149641 ttccgcgttc gactaatcca gctcatatta aatcaaatat tgatatcttt gattttgaat
149701 tagatagtga cgatcttaag agattaaggg ttttagacga acgtaagcca attgatggtt

149761 ggcctgcaga aatgcgtgaa gatgaagatt attaagtcaa aattgaacta aaagttcaaa
149821 ataaaagcca cattttggta aagagatgaa cattcttacc agaatgtggt ttttattcag
149881 ttcaagtata ataatttaac cctgatttaa atgatggggt taataaatac tcaaatgttg
149941 ggagctgtac gagcgtatta gcttttcaac tagttattgt tgatgaggaa gatccattat
150001 atgaaattga gctagaaaat aagtttaacc tccctttatt tactggagaa agagaaatta
150061 gcatgctagc ctctggttta aaattaaagg cgaaaaataa tgttttgagt ttagttagtg
150121 atgaaggcgt gattgatgat acaattagca ttaacgatat aactaaagat aaacaatcat
150181 tcgttttaaa taaaatttag tttagtagtt aaatacccac tcactagaat tgaaagtttt
150241 gctttcaatt ctttttttgt agaagtgcac tataaatcat attcatacta ccaataatta
150301 ttttaatttt taatatattt tctgcagtaa tatattaatc aatgagaaat gtcattaagt
150361 taagacatga ctacttagct ttgtatttta agacattgat tcaatattga atcagcgttt
150421 tttccatgtt tttgattaac ttagttttgt taattctccg tttcgcattt taaagaaaaa
150481 agtagccatt tctttggctg aatagttggg gtggatagtc caaaaacgaa taattccaat
150541 taatgcagaa atgctgtaat aagaaaatag ttcttgtaag tcattatttg cttgtaaatg
150601 catcaatgaa tgtgacaatc cttccatttc taacaaatgg tccattaact tggttgagaa
150661 acttaggtca ccatgactgg aaatcagaat acgaattgtt actaagttgt ctttgaagac
150721 ctgtaaaaaa ttagttagat attcaatacc attatcagat tttaatgcta gttgatcgag
150781 cattttctga tgtggttttt ttaatgccgt aaaaagccta gcttcaatct gctcaataat
150841 tgcccattta tcgttgaaat ggcgataaat agtaacacga ttaatatttg ccatgtcagc
150901 taattcattc atcgtaattt tatttaaatc acttgaatcc attaatttta atagtgcaga
150961 ttctattatt tgatcagttt ttttgctttt cttatgatat ttattcaaaa tgcaacactc
151021 catctcattt tgtagttaaa ttatagtcct tttttctcta agattgaatc aacaattaag
151081 aagagaggta ataacccatg acagaaatct ttaagaaaca ttggtcagaa tataaatata
151141 gtaaaaacat gttgcaagat gcattaatca agcatgtgat ggatttaatg ccatatcgca
151201 tgagcgatga aggtgaagtc ttagaaatca ttatgaatat taaagaaagt gatgaacaaa
151261 gctggattaa tgcctggagt aatatggcag caaacttaca gaaccgtgcg cataagtttg
151321 aaaaagccgg caaactaatt agtgctggta gtagttatct gcgggcttct acttattggc

151381 gtgtatccct aatgtatttt ggtgattggc aggaccctag aatgcaagaa ttttctcaaa
151441 gaagtaaaga ctgttatgac cgtgctttag agttaaatgg ttatcctggt gaagcaataa
151501 gaattcctta tgaaaatact tatttaccag gtcattttta ccgctcgccg tttgctaaaa
151561 ataaaaaggc accattatta atcgtaattc caggacggga tacttgggca gatgatacaa
151621 tttgggttac tgacgcggca attaagcgcg gaattcatgc cctaactttt gatggacctg
151681 gacaagggat ggcattaaga ctgcaagact taaagtttag accagacttt gaaaatgttt
151741 taggaccagt tattgatttc gctcaaaaac aagaggaagt tgattcagaa caaatcggag
151801 caatgggtat gagctttggc ggctttttag tggcacgtgg tgcagctttt gatgatcggt
151861 taaagtttat aatttctgat cctggtaata tgaattgggg tggtatgatt gctaaacggt
151921 tagaaaaagc acttaagatg ccagcaatga ttcgacctaa tatgattgac tttgttttaa
151981 aagactatga atggaagcat ggagcaactg ataagactat aattaacgaa ttaaagaaat
152041 ttgataatac aaaaattgta caaaatatta aaactaaaac tttagtttta gatggttccg
152101 cagaggttac tcatggagca gcgaaaaagt tttatgatgc tttaacctgt cctaaggact
152161 acttactgtt tgatgaaaat tcaactgctc agcaacatac gcaaatgggt ggctatgcaa
152221 ctggaactga atatattatg aattggattg aagacaattt ttagttaata ggaaggagaa
152281 gctaatgaaa ctctttttaa ttggtgctac aggtagaaca ggatctgaaa tagttaaaca
152341 agccttaaca cgtaatgatg aattagttgc ttacgttcga aatccgtcaa agttaaatat
152401 aaatgatcct gagctaacag taattaaagg acaacttgat gatgttgcaa aaatggcttc
152461 tgaaatgaag ggatgtaacg ctgtattagt aacgttaggt aatccaattt ctaacagtag
152521 tggtaaatta tttagttttg caatacctga tattattaag gcaatggatc aagcaaaaat
152581 caaacgatta attagtcttt ctgcattagg tgttggtaca actttagcca ataccagtta
152641 tccttaccgt atgggagcca aaggattttt gaaaggtaat ttttctgacc atgaagcggg
152701 tgaaagtcaa cttaagaatt ctgatttaaa ctggactacc gttcatcctg gtccgttgtt
152761 taatggcaaa aagactgaaa atcctttagt tagggatgct gattcgggat acaagatgcc
152821 aggtgcacct cgaacttatc gtagtgatgt agcacaggtg atgttaagaa ttatcaagga
152881 tcgtaaaacc tttggtaaac agttaatcat gtgttcaaag caacataaat agccctcttt
152941 aatattaata cctattggta catttttaat tttagagggc gaaaaatcta cttttgatta

153001 aatgaaaata aggctttgcg ttaaaaactt attgtatatt tttttgtttt atgatactat
153061 gcttaataac atagcgaaaa aaatagtgat taagtttact acgcgtatac cttggaaaac
153121 tccaagcagt tatgttctta aatttaactg taaggagttt ttttcatgca caaaaatata
153181 tattattcat gggctagtaa cttttatcga ttatttattg gccaagggct agctagtatt
153241 atttcagaaa ctgtcaatta tagtttgctt ttttatctta cctcaatttc gaaaccagcc
153301 actattttat ctatttctga aataatttct ttattaccaa tcgcattatt tgctcccttc
153361 gctggcgtaa tggcagatag agttagtaag aaaaagatac ttattttttc tgatacaatc
153421 agtcttattg ttactctttt acttttttg caggcaatca atagccacaa taatctaacg
153481 attgatctaa ttctgatttc aaatttactt agatcaacta ctattagtat tcagaatccg
153541 actattcaat ctgctgtacc tgaacttgta cctgatgata agcttttaaa aataaaatggg
153601 caatatagtt cgcttcaagc acttaatcaa ttctttccgc cgattttagg tggaatatta
153661 tatggattgc tgtcagtaga ctatattctt ttactatcag gacttgcaaa cgtaataggt
153721 atttgtgccg taataattac atgctttccg aggaatgaag ttaataatgc aaatcaacga
153781 ggctttcacc ttctgttcga attaaaacaa ggtcttcata gtattattca agttactggc
153841 ctgaataggg tgattattta caaggctttt tccgtagcta ttattgtgcc atcaactgct
153901 ttatatccac ttatgactag tgaacatttt catggatctt taaaatttga tgcgccgtta
153961 gtggaagttt caattgcatt aggtatggca ttaagtggtc tgtgtatggg aaatttaaat
154021 aatttatcac atcgacttac acctgcaggt attggagtgc taggaatcgg attaagtttg
154081 tttatttctg gtatacttcc cggcaatcat tatggtttta taatctttat tattattaat
154141 tttattgctg ggttatgtat tccttggatt gatgcacctg ctcaaacatt gcttcaagat
154201 aagattgaac ataaaaactt aggaaaggtg attagttttt atttaatgat gatcggattg
154261 gccggacctg cgggtctaag tttaagtagt gtgataagta aatttatgtc tactgctgag
154321 atttttattg gatcaggaat actacttatt ttgatgcttt tagttgcttt atttgatagg
154381 aagcttagaa gtatttaagc tagttgaata tatgaattta actaactaca taatttattt
154441 ctcttctaga tatggaaact tacttacatt cattgtcttt ttacttttat tcagagatta
154501 ggagtaacca aataatcaaa ttagaaaaag cgagcaatgc attttaaaat tgaaaatcat
154561 tgctcgcttt tataaactag aaactttttt agatactttt aggtatagct atttcttgaa

154621 attttgatat ccggcttcgg taaatttatt taataatttt acatcatgag accatttctg
154681 atctttaaca aaatgatggg cacggttaac tgcaaatgaa ccatgaataa tataaaggaa
154741 aatattgttt tagctttctt tgtaggttta tttttaggag ccatttcaat ctttctggct
154801 attggtggtg gaccacttaa tgtatctcta ttcgtaataa tttttcactt cacaatgaag
154861 caatcttcgg tttattcaat tgcaacggtc tttttctcac aaattaccaa aatcatttcg
154921 attgttgcta gtgcacaata tcagatgttt gatatgaaaa tgattcctat gttgatcatt
154981 gcttcaatta ttggcggtta tattggtaca gtttggaacc agaagatttc atcagcaaaa
155041 ttggaaaatc tttatacagt tttcatgatt gctattacag caattactgg ctttaatgta
155101 attcatttta tataaaaaag caggtaataa acctgcttca attaactatc tatttttgtg
155161 gctctttaca aaacgcgtaa cagtaattat tcagctgttg cagcgaatat cagagcaaag
155221 atgaaggcaa tatgtaaacc ggacataaat aattgatttt gtcctactgg ataagtggtg
155281 atatttttac cagctttaaa actcattgag ctataaaagg ataatggttg atagacttac
155341 tccaagttcc atcccgaaat tacgaactaa ggaattaaca ctaccagcaa tccctaagaa
155401 ttgctggtca acggaagaca taataactga atttttggc gattggaaga gtcccattct
155461 gactccgtaa aataagaaag taactacgaa gagccaaagt ggtgaattta agttaaagaa
155521 agtcataaag gtaaaaatcg caattgcaat taaagcagca attcgtttgt ttttcatgaa
155581 attatctctt tcctactttt tacaagttac agatgaatgg taatatttac gtttacagat
155641 gtcaacatta atttgatttt tgtccattag ttttgagcaa aatatgatgg atattaaata
155701 ataagtgtta ccattaaaac tgtaaagcag aaactattgt gttaggagga ttttatggat
155761 aaacgtaaaa ttgtaattgt tggtgcttca catggtggtc atgaatcagc aattgagctg
155821 ttagataaat ataatgatgt agatgtaact gtttacgaag ctggcgactt tatttcattt
155881 atgtcctgtg gaatgcagtt gtacttagaa aataaagtta cagcagaaga tgacgttaga
155941 aactttgccc cagaagatgt tgaaaagaag ggcggtcatg tttatgctaa ccatgaagta
156001 actgctattc atcctgaaaa taagactgtt acagttaaag acttaactaa taattctgaa
156061 gaagaagtcc aatatgatag gttgattctt tcttcaggcg taactcctaa agttttacct
156121 gtgccaggta atgatctcaa gaatatctat ttgatgcgtg gacgtgactg ggcttctaag
156181 ttgatgagtg ctgctaacga tccagcaatt aaaaatgttg ctattgttgg tgctggttat

156241 atcggtactg aagctagcga agtatttgct aaagctggca agcatgtaac tttaatggat
156301 atgattgatc gcccattagg aacctatctt aattctgaat tactcgatgt tttggaacct
156361 acctttaaga aaaatatgga cttgaagatg ggtgtcaaga tcgaaggctt taacggcaat
156421 ggaaaagttg aaagtgttaa aactgatcaa ggtgatgtcc cagctgactt ggttgttgtt
156481 tcagcaggtg taatgcctaa tactgactgg ctaaagggcg tagttgactt agatcaaaga
156541 ggctggatta agactgatcc atatttgaga actaatgtaa aagatgttta tgcaattgga
156601 gatgcaattc tgccgctttc gattccagct ggtaaaccaa tgccaattgc cttagccact
156661 actactagaa gagaagctca atatgtagtt gatcatatct tcgaagataa gccagaccgt
156721 gccttcaaag gtgttatcgg tgcttcagct cttagtgtat ttgactatca ctttgctact
156781 gcaggattaa atcaattctc agctgctaag aacaagcttg attaccaaac tagtttctat
156841 gaagataaca tgcgcccagc ttatgttcca gaagcagata atcctaaggt atatgtcagc
156901 ttaaccttta atccatacac tcaccaaatt ctaggtggtg cagttctttc taagtatgat
156961 attactgctc aaggcaatgt tttagcttta gcaattagtc ataagatgcg tttggaagac
157021 ttggctgaac aagacttctt cttccaacca ggatttgacc gtcaatggag tttgcttaac
157081 ttagctgcac aacatgcctt aggtatggca agattctaaa tttaggcatt aataaagata
157141 agttatcaag aaaaactagc catgtaaaaa catggctagt ttttttgttg gatttagttg
157201 tgtttgggta aaatagttag taattacaat tgctcattga gggagatcat ttcaagtaaa
157261 ctttgggcag tagaagagcc ttcgttgcct gcttttaagc ccgccctttg caaagcttgt
157321 tcaatatttt ctgtagttaa aataccaaaa gtgactggaa ttttagcttg taaattcatc
157381 tgcataatcg cattagttac gttttgaata atcatggcat aatgatcagt ttcacccttg
157441 attactgcac cgagagtcat aattccagca tatttatcag atttaattag tttttgagca
157501 gtgaaggcta tttcaaatgc tcctggtacc cagatgacat caatttcatc gtcttggatt
157561 ccgaattgat gaagtgtttc tttagcaccg cgaaccaaat ttttagtaac aattttatta
157621 aatttagcta cgacaatcgc gatttttcca tgagtattag tgaagtttcc agtatattca
157681 gtcattattc agcctccttg agttctaata aatgatgaaa tctctttttc ttagtttgta
157741 ggtaaaaacg atcaacatca ttagatgcaa gttctaatgg aatacgttta ttaatcttaa
157801 taccgtaatc gtttaattgg tcaattttat caggattatt agttagcaaa ttgatttctt

157861 tgatcttcaa ttctttgaaa atcacagcac caaccaaagg attcttccaa gtttggaagc
157921 gtcctttttt agcttcttgt aaagctaaat tcatataata ttggtctttt tccatttaaa
157981 tcacttccta acagaacaaa aaaagaacct cgaagctaaa cttcgaggtt cagaaatagt
158041 atgcaagtta aaaaataatt caagtacgca aataagcgta gattaaaatt atccttcttc
158101 catctagact ataactatcg gtaccattaa gggttcaacc gcttgcgcgg gttgcggact
158161 ttaccgccgg tcgggaattt caccctgcct cgaagaacat ttatttaatt ctcattcggc
158221 ataaagctgc ttttcttttt tgtcaaatga tatttatagg gctcttttaa agaatttact
158281 gaaaagtaaa gcaacgattg tttttattgt acataacttc aatgaaggga tgcataaatt
158341 atttgatcgt gaaatccatt tggtaaaaga ataaacgaaa catcacaaat ttcagtggat
158401 tgatactgga gtctgtgatg tttctttatg tataaaattt gaaattaatt attgattttg
158461 attattctta tcaatttaac tgctaaaact caaactttct cataaagatt gggtgagttt
158521 ggtggtctag gtaactagcg gttttaggcg tgatttctaa aacacctaaa tctttttttgc
158581 tgcctcaaca tcgtcaacgt aaatttcatt taaatgagaa tttttgattg ctggtttgtt
158641 tagttaaaat atctaccata gtatgacctt ccttgtcctg aacgcctatc agcattatta
158701 tagaataaaa gactgacatt ttttgacagg aaagcttgca caactatgaa aaaatctgaa
158761 cgacttaatc aagaactact atacttaacc gataaaaaga tctttcattt aaaagaacta
158821 gaagaaaaat ttgcaatttc tgaacgaaca gctttgcgag atattaccga tctagagcag
158881 ttaggcttag ctttttatag tgaaactggg cgtagtggtg gttattacat tactaatagt
158941 aagttactat tgcccatccg ttttaatacg caagagatca acgctatctt tttcgcttta
159001 caggcaataa aaaatcttag tgaaacgcca tacagtaatg aatatcaaaa gattcagact
159061 aaattactaa agaatctacc tcaaaaaatta caaaaccaaa ttagtcttca aaaagaattt
159121 gttcactata ttagtcagcc aagcttgaat gaagtacctt tttttacttt gctacttaaa
159181 gcttgtctga ataatagcgt aattttagcg cgaaattttc agtttattaa agaagaacaa
159241 aaattgcagt tgcttgatat attctaccaa gcagggaatt ggttttgtca tgcttataat
159301 ttggatttaa aacgttggtt tgtcttgcgg cttgataaat ttgaaacagt aaagctacta
159361 aatactaaac aatcggtgat gaataaagag caattacttg atagcttccg gcaatatgaa
159421 cgggagcatt attgcatccc ttatcaatgt cagttaacca aaatcggtga acaaaaagtt

159481 gagtataatt catacccaat catgcaaatt gaccataagc atggaaaaat atatctgaat
159541 ggtaaatata acaaggaaga actacattat ttagtcgact atttgatatc tctgggttct
159601 gaagtaaaag tattaaagcc aaaagaatta aagaatagct atttggaaaa gttaaatgaa
159661 ataattcagc aatattaatt tgctaattct gacataggtt gtcaggatta aggaataaag
159721 taaaactgaa aggttttagg aggtagaatt tatgacaaag ataatgaacg aagatttttt
159781 agacgtttta gaagataaag gaccagcaac gattatttcc atcaatggca atccagctag
159841 tgtggttaat acttggtcac aatatattaa tgtggttgcg gatgatacta ttttgattcc
159901 ggctgctggg atgcattcaa ttgaacagga tttcaagaaa gattctaagc atgaattaac
159961 gattacgatt ggttcttata attatcaagg aaccgctggt atgggtcgcg ggtatcatat
160021 tcacggtagt ggtcaattta ttaatgaagg tgcatatttt gatcaaatga aagcacaatt
160081 tgattggatt cgagcagtat tagtggttaa aattcatgat gttgaacaga aaatatagta
160141 aaaagtttgg agattagctc caaactttta ttttttacgc tttttcactt aaatcttgat
160201 tatgcaaaag tactataatt gaaagaaaag atttaaagga ataatcaata acaataattg
160261 aactgaaaaa cgttaattat caagtgggaa aacgaactat tttaaataat ctatcttttg
160321 ggataaaaaa tggtgacttt ttaactttaa ttggtccatc gggagcggga aaaagtacaa
160381 ttctaaaatt aattgcgaac ttaatcagtc ccagttcggg cactattttt tatcatggta
160441 gcgatattca aaaattagac ccaattaagt atcgtcgaca agtaatgtac tgctttcagc
160501 agccggttct ttttggcaaa actgttaaag ataatttatc ttttccctat caaatcagaa
160561 ataaagaagt agatttagcc aggctagatt cattattaaa gattgtagat ttaccagctg
160621 aatttttaaa taaagaaata aattctttgt caggcggtga aaaacaaaga gttgctttaa
160681 ttcgaagtat tgtcttctta cccaaagtgc tattacttga tgaagttaca gcgggattag
160741 atggaaaaag taaagaaata gttcatcgtt tgattgaatc tgtccatcga caagggacaa
160801 ctattgtaca agtgactcat gatcagagtg aaattcaagt agctaaaaat gttttagaaa
160861 ttaaaagtag gggcttagta aatgaaaaaa gtagaggctt agtaaatgaa aaatgtaacg
160921 gttagcaact tatccttagt tttagctttt gcgctagtat tagtatcaat tgcaatttcc
160981 gcaaaagaaa agctgggatt aaccaaagat attttattag cgtaattcgg gcaatcgtgc
161041 aattagtgat tatcggtttt gttttaaaat ttattttca tatagatacc cttcggttaa

161101 cggctgttcc gataaatagt gtttttatcg tcttcatttg cagtggtttg attggcgaat
161161 taagttttct atttaaaacg gacttatcgt atttactagc atttattttt catcttattg
161221 gaacccttgc attattttca ataatgatgt ttttaaataa ttggttgatt aattggtaaa
161281 ctttattcat ttttatcttt gcttacgcga tgattcggtt agtcattaga ttgacgcagg
161341 aacgcgatat taggcggata aatcaagaag cgtaataaac aaaaagttaa attcaaataa
161401 tcttactaaa agtacgagca cttaattttc aatattaagt gctttttctt gatggttatc
161461 aatttttgta aaactcattt ctcctaaact aaagcatgta aaacaaagga gaatgtaaat
161521 ggtaaagatt caacaatttt ttgttaaaca tcaaaagata attttggcac ttaatactgt
161581 gctgatttta tttgcggaaa cagctaagtg gttattccat atcaatgctg cttatcaagt
161641 tttgatgcta atagtaggaa ttattggctt aattccaatt ttgttaacag ctatttcatc
161701 attaaaagta aaattagttt ctattgatgt cttagtatcg ttagcagtaa ttggtgctat
161761 tgttattggc gaatttaatg aagcggcgat tgttacctgg ctttttgcat taggtgatgt
161821 tctagaagaa cttaccctta aaaagactcg taaagctgtg tctgatttaa ctaaaatggc
161881 gccaaaaact gcgttggttt tgcaagatga cggagcaacg gaagaagagg acatagattt
161941 tattgatctt ggtgaaaaga ttttgattaa aaccggcgat caagtcccag ttgatggaaa
162001 aattgtcgca ggttcaggct atttaaatga agccaatgtt aatggtgagt caaaaccagc
162061 tgataaaaaa caaggtgatc aagtctatgc cggaacaatt ctagaagatg gtaccttgac
162121 ggtagaaact acagcagctg gagacgatac tacctttggt aaaattatcg aaatggtaga
162181 agaagctcaa gattctaagt ctcatactga aaaattaatc aatcgttttt ctaaatacta
162241 tactccagcg gtattagtga ttgcgattat cgtaggctta attactagag acttcaaatt
162301 aacgattaca gtaatggttc taggctgtcc cggtgctttg gttattggcg ttcccgtttc
162361 aactgtcgct ggtatcggta atggggctaa gaacggcgtg atgtttaaag gctcacaagt
162421 aatggatgcg gctcgtaaag ttgatgagat tgcctttgat aaaaccggta cgttaacagt
162481 tggccatcct gaggtaaata aaattgaagt tttgagtggt aataaggatg aaattattaa
162541 tcttgctgcc aatattgaat ctcaaagtaa tcacccgtta gccaaagcaa tggctaaact
162601 aagtgagcag aagattgatc aaatcgagac taagacagtt aagggcaacg ggatgatagc
162661 tgacttggat cgagaaaaat attatttagg gaatttagct ttaattgaga aaaatacctt

162721 tgctaattct aaattaactc aagtggttaa taaattaagt aatttgggaa actctgttgt
162781 agtcttagct aatagtaatc aaagtaaatt ggctgttttc ggcattaaag atcgtcttcg
162841 cccagaagca gaaactgcat taaggcaatt gaagcaacta ggtattaaga agttgattat
162901 gctttcaggt gataatcaag aaactgctca acaaattgcg gctgatttgc caattgatga
162961 aattcatggg caaatgttgc cagcagataa ggcacaattt gttaagaagg aacaagaaaa
163021 tggtcatcat gttgccttta tcggtgacgg catcaatgat agtccggcgt tagctaatgc
163081 cgatattgct attgcgatcg gtagcggtac ggacgttgca gtcgatgttt cagatattgt
163141 tttagttaaa aatgatttgc gtaaaattgc cgatgcttta tcaatttcta aacgaacggt
163201 tttaaatatg aatgaaaata ttgggattgc attgttaact gtgttgttat tgtttattgg
163261 attatttaca ggttatgtcg agatggctaa tggcatgttt attcatgaat tcagtatctt
163321 ggtcgtaatc ttaaacggca tgcgtttaat taaaaatcat cgaaaagttg acggccatca
163381 attttctgat aaagaaaaag atttagcatt aaacatgtaa ggaataagaa aggaagattt
163441 ttatgcaaaa agtaatgatg aagttaggtg gcatgacttg cccatcttgt ttaaccaaga
163501 ttgaaaaggc agttgaagat gtcgatggta cagatcaaat caaggtatta tttaatgccg
163561 gtaagttaaa gttcatgatg aatactgata aggctaatgt tgacgatgtt aaaacagcaa
163621 tcgaaaagat gggctatgaa gtcaagggtg taaaggcaaa ggagctagac taatgaaggc
163681 taaggaagca tacaaggcag aattaaagca aagtgatatt gatcatcata cgcctacagc
163741 gggtgcaatg gtggatcaca ttattgctaa tttattaatc catactttga aaatagatca
163801 agctaagttc ttcgccaagg ggtcggctag tttattttta gaacagcgtg ctgatgagtg
163861 gatcagatat gaacagaatg agtttaacca gttaaatcaa attttagtta ataacggcga
163921 aagtattcct accactactg atcaatttaa agaatatact atgcttgagg agaatggagc
163981 aagtaagtac gaaaatggtc aagatcaact tttcgcttta gtaaaagact ttgatacaca
164041 aattttattt attactaaag caattgcctt aactcaaaaa gaaacttggc cagaattgag
164101 tgctaatttg attgaactgc tagcctggat taaggaacaa atccgtcaat cgcaaaactt
164161 tttaggtcat gaattgcgtg aaagattata tactgaagaa gatgatgacg atgatgattt
164221 ttaactaaag gaagttttta gatggcagag ttatgtgtac atttagttcc attatttaat
164281 gcattaccaa taaatgatca aatgcaaata gaagaactag ttcaccatca aaattatcaa

164341 aatggtgaat tagtaatgga tcctacttca agtaataatt tagtaattgt tgctcatggt
164401 ggtgcccgcc tttatacttt agacgaaaat ggccgtgaaa atattaccca gattttaaag
164461 acgggtgatt atgcgggtga aaactggtta tttggtgaag caaatacgaa tacttatgtg
164521 gaagctacag aaaatagtga tatttgttta ctcaaccgaa atgatttttt agctttaatg
164581 aaaaagaagc cagaattaag tattcaactc ttagaacaaa atattattaa agtacgtgct
164641 atgcataagc aaattcaatt attaagtttg cctaaggtag aagatcgact attgagttat
164701 ttacaggctt atgcaaaaca agtgaataaa aattcattta ctttgcccct aaaaatgaaa
164761 gatttagcct tgtatttagg cactactccc gaaactttat cgcgtaaatt tgtactgtta
164821 gagaagcagg ggagcttaaa acgaaaacta cgtcatattg atttattata gttattaaag
164881 atctacgaac taactggttc gtggattttt tgattatcgg aaattaataa agagtagttt
164941 ttaaattcaa aaaggagatg cttctttaat aaaattttat taaaggaggg agacaatatg
165001 tcactaattt atattcgtca agcagccaaa aatgaccttg aacaaatcat gccgattatt
165061 gatgaggcaa aaaagttttt aaaagaagag aaaaatccgc aatggcaaag tggttatccc
165121 aatgttgaga ctattacagc cgatattaaa gacggggccg cttgggtctt aatcgttgat
165181 cagaaaattg ctgtttatac tgcaattacg gatggacctg atcctaatta caagcagatt
165241 gatggtaaat ggaagaatga tcttgatcct tatgtggcca ttcatcgcgt tgcaatttct
165301 gatgagtatc gcgggatgca ccttgctagc tatttaatga gcagtttgac ttcaatgcac
165361 tatgcggaaa gcataaggaa ttatcgcgtt gatacttttc gaactaacga cgtagtttgg
165421 catattgttg aagacgctgg ctttgtaaaa cgtggcaata ttaagaatat ggatccaatt
165481 gattcatacc gtgttgcttt tgaattaaat ttatagtttt agaatgaata gagggggctt
165541 aattgcatcc ctctttttta gtagcatcta attaaaggaa acaaaaatta tgaaaattgg
165601 atttatcggt atcggtgtaa tgggtacagg gattattaat aacttacgga aaaataatta
165661 tgatgtaact gtctataata gaacaaaagc tcatgcgcaa acagttttgg ataatggggc
165721 taaatgggcg gacaatccag ctgaactaac taaaaaagtt gatattttat tcactatggt
165781 tggctttcca aaagatgttg aagatattta ttttggtcag gatggtatct tgattgtcgc
165841 caaagctggt caatatgttg tcgatatgac cacgagtaaa cctagtctgg cacaaaagat
165901 tgatcaaatt ggtgaagaaa aaggcttaca tatcttagac gctccggttt ctgatgggga

165961 tattggcgcc aaaaatggaa ctttaacggt catgatcggt ggccataaag aagaccttaa
166021 tcatttgcat gatttattca taacctttgc tagcaaatta aattattttg gcagttatgg
166081 tagtggacaa catgctaaga tagctaatca aattatgatt gctggagaac catgactggc
166141 atgaccgaaa tgctggtata tgctaaagcg gcacatttaa atttagaaca aatttgtcaa
166201 accttgcagt ctggtgcagc agaaaatttt agtctagatt catatggtcc aaaaatattg
166261 cagggtgatt atacacctgg tttttttgct aaacattttt taaaagattt gcgtattgcc
166321 ttataagaag ctgataagat gcagttagat ctgcctgcga ctgaagaagc aaggcattta
166381 tatgaaatct tggttgacca aaagaaattg ggcaatgatg gaacacaagc tttgattaag
166441 ttgtggtgga agtaatgagt ttcagaacat cattagctga taaaatgcgg cagctgctgt
166501 ggtttcacta atttgttttg tactcacttt tatcttaaaa aatagaacta aaccagaatt
166561 atagtgagat caatgaaaat atataattac atcaataaag ttaaaaaact agatattctt
166621 ttgtcaaagg agattgctca aatattatta cgacagggcg ttccttttac taaatcacaa
166681 attgaagtag ttatgtattt atatcatcat caagatcaag ttgtgtatca gcgtgacttg
166741 ccagatattt tgggaattac gcgaccaatg gctaatgggt tagtaaaaca tctgattcag
166801 aaaaatgctg taaaattaat cgaagattca gatgattcaa gattaaagcg tattaaatta
166861 atgccagtag taatagcgga atattgattc tcgaactgct gcttttgcta aagaatttaa
166921 ccaaatggaa ggtaagatga ccaaaggctt tactcagaag cagcgttatg agttcgatca
166981 attgttggta agagctatta aaaatttaga agaaaagtag gaaaatgagc ttcatttttt
167041 ggtgtaaagc tcatttttct tttttatgat aatttttgat attaaaatga tattttaatt
167101 atctaaatca cgtatcatta ttaatggaaa gaggtgacaa aatgcaaaaa attgcttatt
167161 ataattctcc cgttggtcaa attttactag ccagtgatga gcaaggctta actggcttgt
167221 ggcttcgtag tgatcgcttt tatgctgata atttagctaa agatcatcaa cttggtaaaa
167281 ataaatattt agatgatgct attagatggc ttgatctcta ttttgaagga aagaatccta
167341 agtttttacc taaattacat ttaattggaa cagattttca aaatcaggtc tggttagcct
167401 tactagaaat tccttacggt gaagttacta cttataaaaa aattgcagaa aaagtagctg
167461 ggcgagcaga tcatttaccc attcgagcag tagggagtgc agtaggtaga aatcatattg
167521 ctttaattgt accttgccat cgtgtaattg gtagtaatgg caatcttacc ggttatagtg

167581 gggggattga caaaaaaatc caacttttgc agttggaagg aattgatatt agtaaatttt
167641 cgctgccggc taactataaa tgacgacttt gtttaagcgg tagttgcgct agtttaagaa
167701 ttttcattcg agtcgaggca aatgtagcaa ataaaggtaa tagatattca ttaattgtgt
167761 gattttgctt ggaaccaaat tgataatcag ctaaacgtgc acccgtactg accgcaacta
167821 gcacttcctt gttatcaagt gcgtgtcctt tactaccata agcccagcca tgttcaagaa
167881 ccaaatcaaa ccatttttc ataagtgggg gaacactgta ccagtacatt ggaaactgaa
167941 aaacaattcg gtcacttctt aatagggcat cttgttctgc tttaatgttg atattttcat
168001 ctgggtaagg ttcataaata ttcctaactt ctgttgttat atctttaatt agcgcttggt
168061 ttacactgga ctcttttaag tcggggtgaa atagaaaaat tgttgttttc attttgttat
168121 ctctcctaat tcaattttag tcagtataaa gtgtaacgtt gcgttatagt caagagagta
168181 attaaaaata gatttttaa agtagcatta ggatgatagt atttttaaga ttttgaatca
168241 aaaattaatg gtaaagatct ggatatatag acatctttat gtttgttata tttggttgag
168301 atatatgtct aagcatattt ttgatattgt tgacggcatt cattaaaagt ttaaaagatt
168361 caaattcaat ttaaatatta tgaaaattca gcttcaaaaa tatctacatt attggtactt
168421 atatgctcaa taactgcaaa acaaacgttt tcaacagaag aggactctgc ccaaatttga
168481 tgccacattt tggccacttc ttttcctcga aagccgaaag caccggcgcc ccaagctcct
168541 aggattaaat tatttacgtg attttcttca gcaattgttt ttacaaaata cattctattt
168601 tgtaaaactt cgcatgctat cctataagag atattatcaa agtttgcacg gttataacac
168661 ggtgcagcac aggttataac atcgaattta tttactaatg taccgtctaa attagtaaaa
168721 acaatatttg gactgtatat tgcacgattt tggtatagat gatgattaat gtgttcacga
168781 ttccattgat aaaaatcagt aaaattagtt aatactgaat ataaattaga ttcatgacat
168841 agcacttctt cttgagctcc tgaaccattt aagtaaccac ctccaggagt tagataatct
168901 gcaaaactta aaattgctgt ttttaaatta gggtaaatat ttttcaaatt gtttgcagca
168961 tgggctgtat tttcctcttt aaaagataaa tttgcataat gagtatattt tatatgtggt
169021 ttttgtaaaa aatcttttgg actataaatt ttactttgct taatacttaa attagtttca
169081 ttagtatatt tgttttccat tatttttaag tggcttttag ccattgcaat gcgtttagct
169141 acaattccca tatagaatct ccaaaattaa aaaagatatt tattgattta taaatattat

169201 ctttaatgtc cagcagtaat tttaattcca ataattgaaa caattaataa
aatgacaaaa
169261 atccatgtca tcatattcat ttgatcatga aataagaatg aaccaactag
tactgatcca
169321 acagctccaa tttctgtcca aactggatac gcgattccaa tggtaaatgt
ttcgttgcta
169381 taattaaccc tgcaaaacta gctatcatcc caataattgt tgcaattgcc
caaccaattt
169441 tagtaaatcc ttggctcaat ttcatcgtcg ttgaccagac aacttcgaaa
attcctgcaa
169501 ttactaaata aatccaagtc atataaattc ctccaaaaga aaagagcaat
tgtatatctc
169561 ttcaaaggcc taatgagata tataattact ctaattcttt tgactcaaga
tccaggaagt
169621 tatgatgata atggtgttaa acaaattaac gtgatcgatt ggttgttaaa
gagctgatac
169681 ataagatgtc tatatttaca tatatttccg tgtaaatata gacgtttttt
catctcttgc
169741 atttcaaaaa atcatatgct attattttac ttacaaaaag taagtaagtt
taggagctga
169801 taaaaatgaa tattgaatta ggtatctcat catttggtga aactacacaa
ttagaagaaa
169861 caggaaaagc aattagtcat gaccaaagaa tcagagattt attagaagaa
attattcttg
169921 cagataaatt gaatattgat gcttttgcaa tcggtgaaca tcaccgcaaa
gatttcgctg
169981 tttcagcacc ggaaattatt ttagcagcag ctgcttcgaa aactaagcaa
atccatctct
170041 caagtgctac tacaaatcta ccaacaatta atccaattag agtttacgaa
caatacgcaa
170101 caatggatgc aattgcacca ggtagagttg aaattatggc aggaagaggt
tcatttactg
170161 aggcatttga tctattcgga tatgatttag aaaattacga tgaacttttt
attgaaaaac
170221 tcgaaatgct gcttaaaatg aacaaagagg aagttcttga ttggccaaaa
gggaaattta
170281 cacctagagt tgatcataaa ggaatttacc cgcgtacaaa aaagccaatt
cctatttctg
170341 ttgctacggg tggaagcata gagtcgacta ttcgcactgc agagttaggt
ttgccaatcg
170401 tttatgcgat aattggcggt gatcctttgc actttaaacc actaattaag
ttgtacagaa
170461 gagttgcaga aaaagttggc aatgatttaa cgaagatgcc gattgctgct
cattcctggg
170521 gctggctgaa tcaagataaa gaaaaggcca ttaaagatta tttctatccc
acaaaattat
170581 tagttgatca aattagtatg gaaagaccac aatggaacgg gatgacatat
gaacaatatc
170641 ttgaagcggt cggagaaaat ggtgcaattt ttgttggtga tagcgaaact
gttgcaaata
170701 agattattaa agtaatggaa gaactaggct taacgcgatt ctatcttcat
ttaccaattg
170761 catcgatgcc tcatgaagat gtcatgaacg caattagaat ttatggagaa
gaagtagttc

170821 ctaaagttaa ggcgtatttt cgaaaaaaga ctgagcaaga ttgtcttaaa ttaaagtgga
170881 agaattaaat agagagtaat aatataggtt tcaagagtct agatgacttt tgaagccttt
170941 tttactttta gagataaacc taaggaggag tgatctatta tgattattcg caccgtaaca
171001 atggatgatt taaatgacat agtagaacta gaatctgacg catttaagat gactaaaaag
171061 caaactgaac aagatatgat tggtagaatt gagaattatc ctgatacttt tttagttgcg
171121 caagaagatg gaaaagtgat tggtcatatt tttggtcctg cttttaataa acgctatatt
171181 gaagatgaat tatattttaa aaatcatcct aatcaaaaaa aggatcgtta tcaaatggtc
171241 ttgagtttag cggttttgcc taaataccgc aaacaaggcg tagcgacaaa attaattgaa
171301 gcattagcgc aagaggccat aaagcaaaat cgtcaagcta tatctttaac ttgtttaccg
171361 aaattgatta aattttatga aaaacgtggt tttcataatg aaggcgagac taatgatgat
171421 attccagatc caactggtgt tactagtgta aacatggtaa gggctttata aaatgactaa
171481 attaactgaa gaaatgtatg caatttttga tcgtgacgaa ttttctttta aaaagctgaa
171541 agaggaatat tctgaagagg agctagcgca aataaaagca agttttaaag atgtttggca
171601 aacgtggaaa aaggtaaatc ttagtgttta tctaaaatta ccacaagata aatttgctaa
171661 agttcatgtc gaaagttgga caaatggttg gaacttacgc gatcactatt gggcagctta
171721 tcgtttaaat actttggctg aaaagagccc ctgcattggt gtaatgctcg ataaaaagca
171781 attgcaagtt tatttaatgt ttcaacatta taagagtgaa aaacgtggtg acagttctga
171841 acaatataat aaattattag ctgatattcc aacttgggca aagaatcgca aaattagtaa
171901 ctggtactta tgggacaaaa atgagatgga atttgtagat catttgtcct tgaatgatta
171961 tttaaaagac acacaaaagc aaactacatt taatgaagaa gccatcaaaa caagtttctt
172021 gctgggcaag tttgctttta gaaatcaaga tcaagttgaa aatatggaag aatttatttt
172081 ggatgggatt aagcaattat tgccgttata tgaaaaaata gagcgttaga taaatttgtc
172141 taaaaccagc aactgtgcta ataattaggg tgagatttaa tttactataa gtaagtgagt
172201 gaattttatg ccaataagaa agccattagc agatttaatt agacctaagg atttatcaca
172261 gtttgtaggt caaaaagatt taattgaaaa ggaacgtcct ctttatcaaa ttattaaaaa
172321 acatgtgcca atctcactgc ttctctgggg accaccagga acgggaaaga ctagtttagc
172381 tcaaattatt gctcgtgaat ttgactatcc attggcaact tttaacgctt caattgataa

172441 taaagctaag cttacacaga tcattaatac atatccttat caatcctttg ttttgttaat
172501 tgatgagatt caccggatga ccactacgct gcaagatttt ctgctgcctt atttagaaaa
172561 tggacgtatt cttttaattg gggcaactac ggaaaatcct attatgtcga ttgtgccagc
172621 agtgcgatca cggtgtcaga tttttgaatt caaagcgtta agtgatgaag atattgaaca
172681 agttttaatc cgtgctttaa aagaagtctt tcattttaaa gatgaaaata ttgaccgaaa
172741 agcagttaag attattacaa tttctgccga tggcgattta cgagttgcat taaatctttt
172801 agaaacgatt catgcagtta atgaggatct ttcagttgaa gaagtaaggc aatttgtaaa
172861 aggacaacat tttgcctatg accgaaaagc aactaagcac tatgattact tagctgctta
172921 ttccgattct atggccggtt ctgacacaga cgcagcttta tattatttag cagttttact
172981 taaaaatggt gatttacctt cagtagtcag gcgtctgcgt gagattccat atacttacat
173041 tggcttagct aatcctgagc aagctacaca gattgtgatt gcggctaatc aagcagaaaa
173101 aattgggatg cctaaggcaa aatatccttt gatgtttgca acaatgctga tgtgtttatc
173161 gccaaagtca ggtgttttcg atgaaatttg gcaaaaatta gacgaagata ctgatcatcc
173221 aagtaatcat ccaatgccgc gaggactccg cgatatgcac tataaacatt ctgaagaaat
173281 aactggtggt ggactgattg acaatccatt tgcgcagccc tttcagatcg caaagcaaaa
173341 ttatatgcct aaaggactcg aaggaaagcg gtattatttt gcaaaagaca acgcaagtga
173401 gaaaaagttt gaacagcagt atttgaagct gcatcaatat atttatggta aggattatga
173461 taaagaaggt aataactatg aaaattgatg tgtttgcaca tattttaacg ccggaatttt
173521 atcaagcaat gcttgatatt gatgcgacaa ttccgtaaaa gtattccttt attaagatag
173581 aaactttagt taatttagat gaaagaatag ccaagtggcc taataaaaat actaagcaag
173641 ttgtttcttt tgcaaatatt aatcagaaga ctttgttgac ggagacgagg taagtaagat
173701 tgcccaatat gctaatcaag aattagctgg tattgttgca aatcatccaa cgtattttga
173761 agctggagta gggatgctag ctatgaataa catccctgcc tcacttaaga ttctagagaa
173821 tattgagaaa agtccttatt tagtgggagc acaaattttc acacgtcatt taggaaaaag
173881 cattgctgat ctggaatttg aacctatttt attaaaagca gcagaactaa atcttccatt
173941 atggcttcat cctgtatttg ataatcgaaa gcctgacaat aatttggtat tttcttggga
174001 atatgaatta tcgcaagcaa tgctgcagct agttcaaagt gatattttca aaaaagcacc

174061 cgacttaaag attctggctc atcacgctgg cgcaatggtg ccgttcttta
gtggtagaat
174121 tgatcatatt ttagatcaag aacatgcaga gatgtttaag aacttttatg
tagacacagt
174181 cattttaggg aatacacctg ctttacaatt agcacttgat tattttggta
ttaatcatgt
174241 tttgtttgga actgatgcac catttgcggt tatgccagca ggtgctgata
agatagtcac
174301 tgatgcaatt gaaaatcttg atattttcga tttatatgat aagaagatct
ttgagacaaa
174361 ttaccaacat atgattaaat aacctgttcg tttatgcaag actgagtggc
taaagaactg
174421 ctcagtcttt ttattacaaa aattttataa tatatttctt ttttctttgt
tctgactaag
174481 tgctacatta aagatgaaga gtagagattg gaggatgtat catgtataac
tttgatcaag
174541 aaccagaacg acggcactta gtcgatgaat ctggcctaaa tacatttcta
agtaaactat
174601 atggtttaat ggctttagca gttttagtat ctgctgtaag ttcataccta
acagtgacta
174661 tttttgctag acagatgctt aatttaatga atcatcatcc ttgggcaatg
tggtttttac
174721 ttctcttacc aatagcttta actttagtca ttaattttaa cgctactaga
agtccagtaa
174781 tgagctttgt tttgctaatg ctcacagcaa ttgtatatgg tgtaactttt
gcctttattg
174841 ctggtgctta tactggcgaa gatatcgctt cagcctttgt agcttcagca
agtgtcttta
174901 ttgtaatggc tattctagga acagtaacaa aacgtgactt aagtagaatt
ggatcctatg
174961 catcagcagc cttaattggc ttaatagtag ccatgctcat caacctttc
ttaaaaaatc
175021 caacaataga ttatattttc tcttttattg cagttattat ttttactatt
ttaactgcct
175081 gggatgctca aagaatgaag aatatctatt tgcagtttga tggagagctt
tcaacaaatg
175141 gtttaacagt actaggagcg cttcaattat atttggattt tgttaacttg
tttctacagt
175201 tcctagatat ttttggttct aatgataata attagtcttt agcgttcaag
cttaataact
175261 tgaacgtttt tttattttta ataaatgttt ttgaaaatta aacgatttgt
cgttaaaata
175321 tcactaattg aaagaaagga aaaaataaaa atgacagcaa atgagaagat
tatcgcctta
175381 gttaagccag aatacttaaa gaaaatacca gctattttc gtaaacatgc
cactgaagga
175441 acttgtaaat tgattgctag agagcatccc gatttatata gtacttttga
aaaggaagtt
175501 gaaccaacag aggaagaaaa acaagaaatg gcaaatttag taaatggtat
ttttgaagag
175561 agaatgaaga agcataagat gttgtaaaaa tgtctttcca ttcgctagga
agtgaaaact
175621 atggaaattg gcacgatgtt aagaaataag cgaaaaatat taggactaac
gcaagatcag

175681 tttattcacg gaatcattag taaatctttt tattctaaag ttgaacgagg attaaatgaa
175741 attagcgcaa aggacttgtt tgcgatttta gaagcaaata atattaacct aaatgatttc
175801 ataaataatg caggttctac tagtaaaagc gatagattaa aagtggaaat tttagttgct
175861 tttaataata aaaataaaca tgaggtacaa gaattaaata aaaaaataca agtatcaaat
175921 tgtgatcaaa caatcaaact atcttcactt ttgatcaacg ctatattaaa taatcaatta
175981 gataaaataa atttaaatac tcgtaatgaa ataaaaaaga aattatttga tgtagaaaaa
176041 tggtggaata acgtttacac gcttcagttg ttttgtaatt caatgcttat ttttagtttt
176101 gacgatttaa ccttttatat gcagcaactc acaaagcaaa agaatataaa ttctttgtcc
176161 tttgatgtac aacggttgat tggttctatc tgtataaatt atttccataa ctgctataaa
176221 aatcatcaaa ctgattgtgc caaatggggc tatcagataa ttgatagttt agctaaactt
176281 cctgatttag ccatttatag gattattaaa aactattatc aaaatttttt tactggtaat
176341 caaaaagaag ctagtaaaat catagacttt tttaaagatt atgatcttaa agagatatac
176401 acggtcttac cttaacttat gcttaagtgt ttcaaatatg aaacatttaa gcattttttt
176461 attttccatt catactaata cttaaaataa attttaagaa gtgttacaga tgaacccaac
176521 atatctcaaa tcaaacctat taattggttt aaagtctaag aaaaatcaac ttctcattat
176581 actacttgtg ctgtttatta tcttttcttt atttatagtt gaaaaacaaa agataggaga
176641 cggagcaaaa agctgggaga attatggtca gtctcttcaa gcaaatgcaa attattttga
176701 tagcgaaatg ctaaaaagac cgacttataa aagcacatat gacaacttaa acaagcagtc
176761 acaagagcta gctagtctac aaaatagtca agtttttgta gatcctgttc aatatctgac
176821 tagttatcgc aacttattaa atactatgca agctggttat cacaaccact acgcaggcgc
176881 taaaacttta aacctaccat cacaattctt tatcaagcaa gaattgaata aaacagatta
176941 cctaattaag cataaaattc caatttttat gaataatgaa agttctgcga catatctaat
177001 ttatattttg tcggttttag gaactattat atttttttat ctttctttta tttcaagtga
177061 tagttggatt attaatttac gacatcagtc tatcttaaaa aatattcctt atcgttttaa
177121 agatgaagta tttggaaaaa ttattattaa ttctacatta gtttttttgc cgcttttatt
177181 agggatgata ctagcatata tatttgctgg cttacgtcat gggtttgtta ctttgaatta
177241 tccagtgatt ttttattcta ctagtatatt agctattcct ctatggctat attgcttttt

177301 attcttagta tatctttcag gcctaataat ctttactact agtttgagcc tatttttaaa
177361 tcagttaact aaaaatgttt atctaacgat ttttattgaa gttattcttt atgccttatt
177421 gtttttacct aaggctactc taaaatggct agtatttttg ccttttccat acctaaattt
177481 aactgattta ctaaatggca atttggccaa taaactaaat ttaaatagca ttaatttatt
177541 aacaggatat tgcgttttat taatatggtc gatattttta cttatcttat ttaaatattt
177601 ggcagcaggg aggaaaaagc aatgaagaag cagtattttt tatttcaatt aaaacgtttt
177661 ttcacaaacc ctaaaaatat tggcttattt gctttaactt ttattctttc tctatatttt
177721 ggattagtta gcgtgcctaa tcgtcaatta attgaaaagg tcgatcccta tgccattaac
177781 ggagagtatc gtagatatac attattctta aataaagcaa caactaaaat aaaggaaatg
177841 caagaatcta agaatactca tgaagtacct aatgagggat atgttaatgc aatcaaaacg
177901 tatccagata ttttaaattt tgataagcat cgaattaatg gaattaaggc aaacaactgg
177961 aaagaatatg taatttactc aagtaagtgg tacaaatacg ttgatcactt gatttttgta
178021 gatgaaaatc agcagttctt atatcccgtt cagtactatc agaatgataa ttaccgtgaa
178081 gatggacact ttgcttatca acgaacagct catttctatg atgcattagc taaaagcaaa
178141 cagccactaa ctaaaaatat tgttgaagag aaaacgactt tacaaatatt acaaaattct
178201 atttctggtt ggacaacatt aattctaatt ctttttgtag ttctttttgc agctgatatc
178261 gtaactaatg atcgcaaata ttattcagtg ttgaaaggta tcccattaag caaaaatact
178321 attctctgga ttaaaactgg ggtagttgaa ataggtgtac ttttcaacta cttactagct
178381 tttatcgtgg tcactttgtg tactgcacct cgatatggtt ttggttcatt taacttaagg
178441 acaacgtttt atactggaag actatatttt attcagccct ttaaatatcc aacgcttggt
178501 aattacttta ttcagtttgg aacatttgct attttaattg cttttctatt tattagactt
178561 actattttac tttcaattat ttttaaaaat gaatatgtag ccgaattatt agcaagcgtc
178621 ttcgcagtaa gtgggaaagt tctatatttc tctttaggta tgggatttgt ctacccaatt
178681 ttacaaagtt taccaatgac ttattttact gttggagaaa gcttgtccgg aaatttgtcc
178741 tatctaatgg attcaccagg atggggattt aattccggta tagttccact aatgttgtca
178801 attgtaatta ttgaaatttt tatgttaatt attagtaggt caaagagaat taaactgata
178861 aggagatgag attaaatgct tgaattaaaa aatattaaat tatcatttgg gaatcatata

178921 gtcttagata atttaaatta tagttttcaa agtggtaaga tttatggatt agttgcacca
179981 aatggcacag gaaaatccac tttaatgaat gtcatcttaa ataatttgaa accgcagaat
179041 ggatatattg cgtacgataa tttgcaatac aagaatgaga agacaattgt tagattacac
179101 aaaaaaattt gcgcttttcc tgatcaaagt gacttatttc cttttatgac tggtaaagat
179161 catttaaaac tttatgctaa tttatggaag aataatcctg aaagtattga aaagattatt
179221 tctactctta aaatggaaag ctacgtcagc cgaaaagttg aaacttattc attaggaatg
179281 aaacaacgtt tatgctttgc aatggtagtg gcagccaaca ctccagtgat gcttttagat
179341 gaagttatga atggccttga tcaacagaac gtccaattaa tttcacaagt tttattggat
179401 ttgcggtcgc aaaaaaagct aataattatg gcatcccact tattgcaaaa tctacaacaa
179461 tatgcggacc aagtcttatt tttaaaagat gggaagataa ttgaagactt taataacaag
179521 gcagaaacta agcagtactt agaatttaag agttcagctc tacttactga tattctgaac
179581 aatgttgaag ttcaaacttt accaaataaa actaatcttg tagcaattga taatataaat
179641 caagaaacat ttaatcaaat aacgaattat ttgattaaaa ataatatttc attttcagtt
179701 acgaaaatgt ctttagaaaa tctatttaat aaattctatg gttaatttat agctccttag
179761 ctagtggtcg attggcacat ccatttggta cgcaaactgt tagaagaaaa caagatcttt
179821 tttcagaaaa agataagtat ggtaaaactg tcgatattga tcgcccaatt attgatgaag
179881 tggaaaaaat agctcaaaat cacaatactt caatggcaac aattgcccta gcatggctta
179941 aaactaaggt tactgctccg attgttgggg ctacaaaggc acaccactta gatgctttag
180001 aagaagctat caagattaat ttaagtaaag aagaagtcca tgcgctcgag gcgccatata
180061 aagcgcatga cttagaaggc gtgatggccg acaatcgtga ccgtgaacat aactggacgc
180121 agtatttgac taatagcgct gaaaaatagt cccatatatt ttatttatta cttttctagc
180181 actaaaatga agttgttaga aaaaggagga cttatactat gacagaagtt ccaacaatta
180241 cattaaatga tggtaataaa atgcctcagt taggtttagg cgtgtttaga attccggacc
180301 atgaaaaagc aagacaaaca gtagaaatgg ctttagcaaa tgggtataga atgattgata
180361 ctgctgaggc atatgacaac caaattgcag tcggtgaagc tattgctgaa agtgcggtca
180421 accgggatga catcttttta actactaaga tttgggtttc aaatatgtcg tatgaaaagg
180481 ctacaaaggc aattgatcat gacttaaaag aattaggtac tgattacatc gatttggtat

180541 tacttcacca accctatggc gatacttttg gtgcttggga tgctttagca
gccgctaagc
180601 gcgcaggtaa aattcgctca attggtttat caaattttta tcctgatcaa
tatatgaact
180661 tggaattagc tcatgaagat aagccagcaa ttaatcaaat tgaaattagt
ccttggttcc
180721 agcaaactaa tgatgttaag ttcttccaag gaagagacgt agcagttgag
gcttgggcac
180781 aatttgcaaa tggtaagcac gacattttca ataataaagt tttgaaagaa
attgctgaga
180841 atcatcataa aactgttgga caagttatct tacgttggtt aactcaaaag
agcatcgtag
180901 ttattcccaa ggctgttcat gatgaacatc aaaaagagaa tattaatatc
tttgacttta
180961 aactaacaca agatgaaatg gcaaaaatta aggaactaga caagggcgaa
agtcaattct
181021 tcgaccaccg tgatccagct gctattgata acatctttgg tgaaagtttg
agacaacttc
181081 gtgattcaga ataattaggt caatctaata caaaagagac tagtttagtg
ctagtctctt
181141 ttactttgca attactaaat gataatcacc taaaaaggtg ctttcaaaat
cttttggaag
181201 tttatattgc tggttaaata taacaatcat cgcatgaaag ttctgataat
tacgcagtgt
181261 aaaatgatcg acattagctt caactaaatc ttttgccttt tgataagaaa
ctggaagttg
181321 gtaatctaaa caattaaata atcgtctagc atatttatca acgacgaact
cgcctttctg
181381 aataccatac attaagatta catctgctgt ttctggacca attcctttaa
tgctaagcag
181441 ttcagaacgt aactggtctt tagataattg gcgacattta tctaggtcaa
aatcatattt
181501 ctgaaagtag gttgccaatc ttttcagagt ggcagctttt ctagtataga
agccagcaga
181561 agcaatggcc ttttctaatt tatcatcggg catctttaag atgttctctg
gccagaaatt
181621 agtagcttgg tacagagttg ctaaggcctt atcaacattt ttccaattag
tattttgaat
181681 caaaattgtt gaccaaatta cttgccagtc gcttcgccca ggccaccagc
cattaggatc
181741 taggtggtcg tacattaagt catagagttc attcaaatcg atctttttca
ttattattcc
181801 tccactaact aattaattat cataatctta agcattcaat ttaacaaaaa
tatgatattt
181861 taggtactaa tagataaaat aaaaagataa agataagaaa ctaggttttg
ataatggaag
181921 aaaatactaa aaaaatgctc gccggtttac catatcgtcc agacactgag
gagttaagca
181981 gaatttcaac gaaagctcat cgtttaagta gggactataa tcaaaccgcc
gatgaagata
182041 aagaagttag aaaagcaatt attgatgaat tatttccaaa tcatgaaaca
ggagtgtatt
182101 tacaaggacc aattgaggtt gactatggaa aatttactaa actaggtaag
aatttttatg

182161 ccaattttaa cctaactatt ttagatactt gtcctgtaac gattggtgat aatgtaatgt
182221 gtggtccaaa tacttcatta gtaactccac ttcatccctt attgcctgag caacgtaatg
182281 cacgtcttca aaaagatggg aaaattgctg atatcgaata tggtgctcca attacaattg
182341 gcgataactg ttggctagca agtaacgtga ctgtctgtcc gggcgtaact attggtaaga
182401 actgtgttat cggagcaggg agcgtagtaa ctaaaaatat tcctgataat tcacttgttt
182461 taggagttcc aggtcgggta gtaagaaaaa ttactgaaaa agatcgctta gataattatc
182521 cttactaaaa atttgctata gaatcgtctt gcgaacactt gtttgaatga agtataataa
182581 ctatgaaaaa agactgttaa agcattaact ttagcagtct tttgttctta ctatttattt
182641 attttagatt tactattgag taatccatgg gatttttat accaatacca taatccaaaa
182701 ggaacgatat tttcttcatg attgattaat cgtttaatat tagaacggtg tcttacaaac
182761 aataagacca tcatgattcc agtaataata gtcagaaaga catcatgaaa ccagaaagaa
182821 caaataaaaa ttatcactat tgaaactaaa ctagataaac ttacgtatga agtaataaag
182881 agcattggta aaaatactac tgcacatagt ccaaagaatt ttagattgta gcctaggaat
182941 acacctgcac tagttgctac cgcttttcct cccttgaatt ttagaaagat tgagaaagta
183001 tgacctaaaa tagcgcaggc accggcaatt aataataaat actttggtcc ctttaaatgg
183061 aaaattaaag gcaaatcggt agcaagtgtt ccttttaata catcaataat taaaaccgca
183121 cttccagcta tcggtcccat tactctgaat gaattagttg ttccaatgtt tcctgatcca
183181 aaatttctta tatcttcatg aaaaaatatt tttccgacta acacacctgt aggaaaggac
183241 ccaattaagt atgctaaaat aaaaactagt aaataattca acgtagacat ttaattttca
183301 accccattta aaatctaaag ctattttaac agagattata atagctgtgg gattgatttt
183361 gattgtctgt taaaaatgga ggcatttatt tggctaaaac aaataaaacg accagttcat
183421 atgatgattc ttcgatccag attcttcatg gactagaggc agtaagaaaa cgtccaggaa
183481 tgtatatcgg ttcaactgat cgacatggtc taaatcaact agtttacgaa atcgttgata
183541 actcagttga tgaagcaatg gctggttatg gtaaagaaat taacgtaact attcataaag
183601 ataattcggt tacagttcag gactttggac gtggaatgcc aaccggaatg catgcatcag
183661 gaattccaac aattgaggtt attctgactg ttttgcacgc aggtggtaag tttactgaga
183721 aaaattatca aacctctgga ggcttgcacg gtgtcggctc ttctgtcgta aacgcattat

183781 cttcttatct aaaggttaga gtcgttagag acggacaggc ttatgaagaa gaatttgaaa
183841 atggtggaca tccgattggc actcttaagc atttgggtaa aactaaagat aagaatggta
183901 ccacgattac ttttaaacca gatacgacta tcttttcaac tactacctat aattatgaaa
183961 ctattcaaga acgaattcgt gaatcagcat ttcttttaaa gggtgttaaa tttactttaa
184021 ccgatgaacg cgaacccgaa catcatgatg aatttctcta tgaagatgga attaagtcat
184081 ttgtatctta cttaaatgaa gataaagata cgatgggaga tgtattctac ttttctggtc
184141 agcaaaatgg cattgaggta gaattcgctg gtcagtataa cgatgggtac tcagaaaact
184201 ttgtttcatt tgtaaacaat gttcgtactg ctgatggtgg tagtcatgag gctggtgcaa
184261 gaagtggatt tactaaagct tttaatgatt atgctaaaaa gcagggcttg ttaaaaaata
184321 aagataaggg tctagaaggt agtgattacc gtgaaggcct aagtgcagtc ttatctgtta
184381 agatccctga agaattatta gagtttgaag gacagaccaa gggtaaatta ggtactccac
184441 aagcaagaag tgttgttgat agcattgttt acgagcaact ttcttattac ttaatggaaa
184501 atggagaatt tgctcaaagt ttagttcaaa aagctcaaaa ggcaagggat gcgcgtgaag
184561 ctgctaaaaa ggccagagat gaaagtcgaa gcggcaaaaa gagacgaaag aaagaaattc
184621 tttctggaaa attaacccct gctcaatcac gtaatcctaa gaaaaatgaa ttattcctag
184681 tcgagggtga ctcagctgga ggatctgcaa aacaaggccg agatcgaaaa tttcaagcaa
184741 tcttaccatt gcgtggtaaa gtattgaata cacaaaaagc taaattgcaa gatatcttta
184801 agaacgaaga aattaacact atgattcata ctattggagc tggcgttggt tctgattttc
184861 aaattaaaga tgctaattac gataaaatca tcatcatgac agatgcggat actgatggtg
184921 ctcatattca aattttgttg ttaaccttct tttacagata tatgcgtcca atggttgaag
184981 cagggcgcgt ttatatcgca cttccaccac tttacaagct tcaaaaggga aacggtgcaa
185041 aggctaagat caaatatgcc tggactgatg aagaactaag tgaagactct aaagatatgg
185101 gtaaaggatt tgctttacag cggtttaaag gtttgggtga aatgaacgct gatcagttat
185161 gggaaactac catgaatcct gaaactcgta ctttaattag agttaagatt gatgatgcac
185221 aacttgctga aaagcgtgtg acaaccttga tgggagataa ggttgctccg agaagaaaat
185281 ggatcgacga aaatgttaaa ttccgcatgg gcgaagatgg ttcaattcta gaaacaagta
185341 gagattaaga ggttagattt ttaaatggta aaaactactg aaagaattag ggaattacct

185401 cttgaagaag ttatgggaga aagatttgga cgatattcaa aatatattat ccaagaacgt
185461 gctctccctg acattcgaga tggtttgaaa cctgttcaac gtcgtattct ttatgcaatg
185521 tataaagata ataatacata tgacaaacct tataaaaaag ctgctaaggc tgtcggaaat
185581 gtaatgggta actttcaccc tcatggagat agttctattt atggtgcgct agttcactta
185641 tctcaagact ggaagatgcg tgaacccttg atcgagatgc acggtaataa tggatcgatg
185701 gatggggatg gtccagcagc aatgcgttat acggaatctc gtttaagtaa aatttctaat
185761 ttgcttttac aggatattga taaagaaaca gttcaaatga ttttgaactt tgatgacaca
185821 gaatatgaac caactgtcct acctgcgcat tttcctaatt tattagttaa tggttcaact
185881 ggaatttcag ctggatatgc gacagaaatt ccgccacata acctagctga agttattgac
185941 gcagctgttt atctcttgaa acatcccgat gccaccacag atgatttaat ggaatttgtc
186001 aaaggaccag attttccaac tggtggtatt gtacttggaa caaaaggcat taaggaagct
186061 tatgaaactg gtcgtggaag aatacaagtt cgctctaaga ccactattca agatattaag
186121 ggacaccgtc aacaaatcgt agttactgaa gttccttttg gcgtcaacaa ggcgatgatg
186181 gtcaagaaga ttgatgaaat ccgtttaaat aaggaaattg atggaatttc cgaagtaaga
186241 gacgagactg accgtcatgg actttcaatc gttattgaat taaaaaaggg tgcagacagt
186301 caaaatattt tgaactatct ttttaaaaac actgatttgc aagtttcata taacttcaat
186361 atggttgcaa ttgaccgtat gactcccgtc caagttggct tgaaacgaat tttatcttca
186421 tatttgaccc ataaaaaaga ggttgttact aaaagaacta aatttgattt aaaaaaggca
186481 aagcagcgac tagaaatcgt tgaaggtttg attcatgcgc tggatatttt agatcaagtt
186541 attaagacaa ttagatcttc taaagataaa aaggatgcta aaaataattt aattgctaaa
186601 tatgaattta ctcctaatca agcagaagct attgtttctt tgcaactgta ccgtttaact
186661 aatactgatg ttaaccaact taagaaagaa caaacagaac taaataaaaa gattgacaaa
186721 ttcaatgaaa ttttaagcga caataaagtt ctagaaaaag ttgtagttaa agaattaaac
186781 gcggtcaaaa aagaatttgg ttctccaaga agaactgaaa tcacagctaa agcggctaaa
186841 attgaaatta acgaaaaagc tttagttgcc gatgaagacg ttcgtgtatt agtaagtaaa
186901 gatggctact taaaacgctc atccattcgt tccttccagt cgactgacac aagtgataat
186961 ggattacctg atggcgataa ggttattttt gaaaagacaa tgtcaacctt agataacctt

187021 tatattttta ccaataaagg aaaccttatt tatcgaccag tccatgaatt aatagaaaca
187081 aagtggaaag aaactggtca acacctttct caagaaatcg gacttgataa tgacgaagaa
187141 atcattcgag tatttgaaat taatgactta aaagcaagtt tgaacttttt aattgccaca
187201 aacgatggct atattaaaca agttacctta gctgatttgc agccaactag aacttataaa
187261 tctcgtgcga tcactgcaat taagcttaag agtaaagaca gtaaagtcgt aagaattgat
187321 aaagttaaac cagatagtaa gcaagaaatt accttaataa ctaatcaagc atatgcagtc
187381 agatttgata ttagtgaaat tcctacttcg ggttctaaag cagtaggggt taaatccgtt
187441 aaccttaaag acaatgatta tatcgtaaat tatgttcttg ccgatcctaa atatgttgac
187501 ttgattaata ttgggattat tactcaacgt ggggcattta agcagctaaa acttaactta
187561 ataaacaagg taactcgtgc taaacgaggt gtattagtac tacgtgaact taaaactaaa
187621 cctcatcgaa tcatggcaat tagtagctac ggcgagaatc atatcttgca tttaactact
187681 tctagtgatc gtcatatcga attaaataca tctgaatttc cattaggtga tcgatattca
187741 aatggttcat ttgttttaga tccaatgaca gatggaacac caatttcatt aacgctaggt
187801 caaccattag cagacggaag agatagaaca gaagacttat tttaaatgaa agctaagaat
187861 agcattttaa agctactctt agctttttta ttaagataaa taattataga aaaatatctt
187921 atattttatg taaatatttt cattatctag atacaggtgt tatgataaaa agtgtaggat
187981 gaataatagg ctttataaaa ggctatcgga aaataattat atgtatttta aatttatatc
188041 aattagatgt tattccaaat ttatataaga ggcattcaat catgaaaaca aatactgatg
188101 cagttctatc tgcgaaatct ttacgttact ttcttcaatt aattgataac atgagttaca
188161 ctcaagcagc tcaaatctta ggaataaccc aacctgcatt aactcaacaa attaagaaat
188221 tggaacgtgc agttggatct cccttatttg gtcaaatggg gaaaaagctc tatttaactg
188281 atgctggtct aaaaatggag gaaacagcca aggcactatt ttcaacagtt aataatgccg
188341 ttgatagtat ccagcaatat acaaaatcag atactgggac tatttcatta ggtgtattgt
188401 ccactattga agctagggtt atcgatcaat ttctaattga atttaaccag aaatatccag
188461 agataacctt acatgtcggc ttttacaata gaacagagct ttgggacaaa ttagataata
188521 atcaattaga cttagcagtt ctttatatgc cagatcacaa tgctactgta aaaaacatga
188581 agcaatatat ggctaaacaa atttatccag aatcagtagt atttttaact cataatccag

188641 aaacgaaatt ttataagtta gcacaacata agtgggtaac ttaccctaag
gattactact
188701 tgcctcaaat tgtgcgtcga ttttatgcta ctgaatttcc aaataatgag
ttgctaactc
188761 cagttacttt tactgctcct tatcaactaa taaaatttgc ggaacaaact
gattacgaca
188821 cttatgtttc aaagagcttc tataaggctc acaaggctca aatcacgctc
tctccattag
188881 agccaaagca tgaaacaaac tttgaaagtt gctttattta tcgtaaaaac
aagagtgaga
188941 tacctcgatt agttaatttc ttaaaagaat gggataaatt tattaatgaa
aaagattatg
189001 atagtcgatt agaagaggtt aaagttaata tctagtttta gaagcattgt
ttttataact
189061 aaaagcaatg ctttttagtg cgggattgat ttgcttaagt tcaaatttta
atctaaaata
189121 ttaagtagat ataattttaa aggagctcta tataatggca aaagaattga
tttttggtca
189181 ccaaaatcct gatacagatg caatcggaac tgcaattgca tactcatact
tacaaaataa
189241 attaggttac gatactgaag cagtcgcttt gggcgaagct aacgatgaaa
ctaagtatgc
189301 cttgaataaa ttcggtttta ctgcacctcg tgtaattaaa actgcttcaa
acgaagttga
189361 tgcagttatg cttgttgacc acaatgaacc tcaacaaagt gtttctgaca
ttgataaagt
189421 taaagtgact cacgtagtag atcaccaccg tatcatgaac tttaataccg
ctgatccttt
189481 atactaccgt gcagctccag ttggttgtac aagtacaatt atgtggcaga
tgtacaacga
189541 aaaagaaatt gaaattccac aagatattgc tggaattatg ctctcagcta
ttatttcaga
189601 tactttatta ttaaaatcac caactactac agatcaagac aaagaagctg
tagaagcttt
189661 agccaacatt gctggtgtta actacaaaga atatggtctt aaaatgctaa
aagctggtac
189721 caatattgct gataagtcag aagaagactt aatcgattta gacgctaaga
gctttgaatt
189781 aaatggcagc aacgttcgtg tagcacaaat taatgttgtt gacttgccag
aggctttaga
189841 gcgtaaagaa gctttcttaa aggctatgga tgaagcttca aagagtgaag
gctacgatat
189901 gttcatgctt ttgattacta atattcttga ttcagattca gaagctttag
tagtaggtag
189961 tgatgaatca aaagctaagt ttgaaaaggc atttaatact aaattgtcag
attcagaagt
190021 taagttacca ggtgttgttt caagaaagaa gcaagttgtt cctccattaa
ctaatgcttt
190081 tgaagcttaa gttattattt agttcaattt tagaaaagac aggtatgatc
tttcatactt
190141 gtttttttat ttaattagga aaaatgaaat cattacaagt attaaaagat
gtatttggct
190201 acacaacatt tcgcccagga caagaaaaag ttattgattt agttcttaaa
ggagaaaatg

190261 ttctagcagt aatgccaact ggtgcaggaa aatcaatgtg ctatcaaatt cccgccttag
190321 ttaattccgg tttgacttta gtaatttctc ccttaatttc gctaatgaaa gaccaaattg
190381 attccttaaa gcaaaatggt attaatgcgg cagccctaaa ttcggcgact cctcaagaag
190441 aagtaaatcc aattttaaga caagcatatg aaggcaaaat aaagcttctg tatgtgactc
190501 ctgagcgatt agcaatggat tattttcgct atcagctaaa cttcttagat atttcattag
190561 tagcagtcga tgaagcacat tgtatttcac agtggggaca tgattttcga ccagcgtatc
190621 gccaaataat ggatggcgtc aaatcaatta agagtaatcc gaatattttg gccttaactg
190681 caactgccac accctcagtt caaaaagaca tagcagaaca attgcagata cctaataaaa
190741 attatgttat tacttcattt gcaaggccaa atctaagttt caaggtagtg gataatccta
190801 aaaatactaa cttgtatctt ttagactata ttaaaaagca ccctaatgaa tctggaatta
190861 tatatgccag tacaagaaaa catgttgaag aattaactga ctatttagct caaaatggta
190921 ttctaacagc tagttaccat gctggtcttt ctaatgaaga gagatcagat gtacaagatg
190981 cttttcaatt cgataaagta caagtgatag ttgctactaa tgcttttgga gtggggattg
191041 acaaaagaaa tgtgcgcttt gtaattcatg ctaacagtac gccaaactta gaatcgtatt
191101 atcaagaagc tggcagagca ggtagagacg gtgagcgttg cgagggtatc atgatctatc
191161 atcctaaaga tatccggctc tatcgttggt ttattgaaca atctgattta gctgatgaat
191221 atcaaaaaat acagtatcaa aaactagcta tgattactaa atatgttaat accacagagt
191281 gtttacaaca gtttatcgtc agctattttg gacaaaggtg tgagccctgt ggtaagtgct
191341 ctaactgttt aggaaccttt attgaaaaag acattacgtc agaaagtaaa agcattattt
191401 caactattta tgaattagat ggtcgttttg gcaagactgt agttgctgat gtagtttcag
191461 gagcaaataa tcaacgcatg agagaaattg atgcagctca tttaaggcat tatggcagtt
191521 taaagattgg aaagagtagg gtcataaact taataaacta tctaattagc cataattatt
191581 tacaactgac tgatagtcaa tatccagtag ttcatgtgac taacttagga tgggacgttt
191641 tagataataa aaagcgtgta actcaaaaga tttctaaaaa aatagaaaaa ttaagtcaaa
191701 cctataatca aattccgaaa gaagagaatg acttatttgt tcgcttaaaa gaagttcgtt
191761 tagatttagc taaaaaacag gggataccag ctttttatat cttttcagat aagtctttaa
191821 gagaaatggc actgcaaaaa cctaagacac aagcagaatt tttaaatatt tcaggagtag

191881 gtcaagctaa gcttaaatcc tatggtcaaa ttatgctagc tgcaatcaaa aattacttga
191941 aagaagacgc agactaatga agtgtagtta aataaagtgt aaaatgaatt agaatagtat
192001 aaaattcagg aggattaata tgcaatatcc acaattagac ctagaaaatg caaaaggtcc
192061 aaaagcctta attcaaacaa atcatggaga tattaaaatt caattatttg aaaaagaagc
192121 tccaatgact gttgaaaatt ttgtccgttt agctaaaaaa ggctattatg atggcactac
192181 ttttcataga gtaattagtg atttcatgat ccagggcggt gatccaaagg gtgacggtac
192241 tggtggtgaa agtatctggg gacatccttt tgaagatgaa ttttcaaaca aattattcaa
192301 tgtacgtggt gcgctttcca tggctaattc tgggcctaat acaaatggca gccaattctt
192361 tattgttcaa aataaaaacg tacctaaacg catgattaaa gaaatggatg ctgcaggtta
192421 tcctaaagaa ttagttaagg catacaagca aggtggaaca ccatggcttg atggtcgcca
192481 tactgttttt ggtcaagtga tcaaaggtat ggatgttgtt gatgaaattg caaaggttcc
192541 acgtgataag acaaatgata agccaaaaga agatgtgatt attaagagta ttcatattga
192601 agattaaaca aattaaacat ggtctaaatc gatcatgttt ttaatttgtt ttcactataa
192661 cttggaggtt aatcatggga atattttcag atgaaaaata tcgtccatct gaatcgcgta
192721 ttttagctac agcattgact ttttctgcgg gatttattga tgcctatact tatattcaac
192781 ggggacatac tttatctgct gggcagacag gaaacgtcat cttctttgcg tcagcatttg
192841 ctgaccataa tattgcaggt atgttaaaca gggcaacaac gtttattgct tttaccttag
192901 gattactact tgttggactt tttcataagt atgtaaaaag taattattgg cgtgtatttt
192961 gtttatttcc aattctattt atttgcttgg gcgttggttt cgttcctaaa aacgtaccaa
193021 attattatgt agttccggta attgcgtttg gtttagcggt tcagaatgca tcatttagta
193081 aaattgaagg tatgggatat aataatgcat ttacaactgg caacttaaaa aaatctgtag
193141 ttgcttggag cgcatatttc tttggtgaag ataagtcaca gcataacgca gctgttaact
193201 atatgttctt ggttattgca tttgcacttg gtgcaattgt atcagcattg ctccaaaaga
193261 tctttgtttt aaaaactatt tggtttgctg ttttactttt aggagcaatc aatatcgttt
193321 atacgatctc tttaaataaa agaaagaaac tatctttttc taaaaatgaa taattaatag
193381 agaaactttg agaatgataa aactcaaagt ttttttactt ataaaagaga gtagcttagt
193441 tcatcttgac tatacttcgt gtaatatata ttatgtaaag taatatttaa aaatatatca

193501 tatttgtaat taatcacaat caacagccca accatcaatc aactaattat ctactattta
193561 tcaaaagtta ctttttaaaa atcattttta aatctactag catttcaatt agaacacatt
193621 aatcttttta cagtcagaaa ttctgcttac cccttatttc acaattaatc agctaaaaat
193681 actaagctaa agatttttca attaatcaga ttagatgatt attaaaaagc atttttcttta
193741 gtgttgagat gacgattcta cgccttttaa aatggttctt gattaatagt tgttaagttg
193801 ttagctataa gaactcataa attactggat gcgaaacttt tttgttcatg atacatttct
193861 ttcatctaaa tccagtaagc ttttaattag atcatgttag ttcggatatg ctagatacta
193921 agttgaatat ttcttaaaat tattatgaaa cattcaaaat aaaaggtaaa aagagataaa
193981 gcataatcat agtaaacctg gtcataataa tttttatcta tcaattgtta ttgcgggtat
194041 tttaatttat tttcttatta aaattaaagt ctagaattat caatatatac gtaatggtct
194101 ttcattaaaa ttattgtttt aatgatgcct attgaaggta acctagcaaa atacatataa
194161 ttcattgatg cgtcaagctt tcataaatac aaatttcgtc aaaactatta caaatttatt
194221 caaattctag tcaaaaaaat gaccaaaaag tgaccacgac tttctcttct actatgactg
194281 ctatctttac aaataatcga taatatgtta tatggacaaa aaaagccact ccagagaact
194341 taatctccag agtggctttg ttttcatcta ttcaaatctt ccccaaggat cattgccttg
194401 gcgacaaact aagtaaccgt attgaccatt tgcccgtggt tgtctaatcc agacatagcc
194461 accatgacga ctataagcat cgtatttaac acaatcgccc ttagttactg tgccaatgat
194521 atcactagtg gttctagcac catagcgaat attaatagtg ccgtttggat agaacttacc
194581 ttcttcctta taccaagtat caccaatatc atcaacccaa gattgtggtg ttgattgctt
194641 aacggctggc ttagatggtt gggctactgg cttaccgtca gtcttaagat caatcaagct
194701 gatgttaccg tcaacgttta atccgcgcca gttatcggta aattgccaga ttaatatccc
194761 gttcattgag ggaaagtaac cgaagtttgc ctcacttgtt gcaccagata caggataaga
194821 cgctacccat aagcagtttg gatattttgc taatacctta ccagtatcaa tcttagactt
194881 aagtaatgca acacctgagt ataacaatgg ttggtaacca gcacttacga tcgtatctaa
194941 gaaagctagg attgcagtag tattagcacc agcatctcca ccagtttcat taccactacc
195001 ttgttcataa tctaatgcta agtaagaacc tacattaagt ccaactgctt tagctgagtt
195061 aactgcataa ttaccttctt gaacggctac gttactattg ctactaaatc ttccatagtg

195121 ataagccatt ggcaacatac cattagcttt tgcagtagaa atttgagatt gtgccttatg
195181 attacgatag tctaagcctt ctgatacttt gacaattgca aacttagcac cagaataatt
195241 agctacatta gtatcttgat agctagatac gtctacaccg taatttcttt ttgctacttc
195301 catatatgtt acctcctact tcttaacttc ctttaattcg ccaacaatgg tcgtcttagg
195361 tttatcaaca gtatctgcaa ttgattgtgt ttgcttcata gcagtcacag ccttttcaac
195421 cattcctttt acgaaactca tagttgggtg tggtaggtgt gccatatcca aaagcaagaa
195481 caagccttga acgacaaagg ttaacttgtc ttcgccttcc ccgcccctct tttctgcttg
195541 gtagacaagt ggacttacag actgggcaac aatcttttcg gcctttgcta aaaggtcgcc
195601 ttgtgctact ttcttatcga ttgcaatttt gtgtttagcg tagacagaaa caacaattac
195661 tgctgtaact gaggttacta caagaactaa atctaagata tgtgaaaagc tcattattta
195721 gcctccttta aatctttttc tgttttttcg atcattttttg tttgtccttt aatttatctt
195781 gtaaagactt gatttctttc tttagattgt caacactcat atctgtttca tctggtggag
195841 cgtcatgttt agctttaacc tgataaagag tagcgattgt accaatcaag tatgcgattg
195901 ctaatatcag attgcttaga tcttgcaagc gtcccacccc ccctataaat aatgagcaac
195961 aagtaggatt agaataaaaa gaattgtctc gccaataacg ttgagaccca ttacccagta
196021 gttagacatg attacgtgta gcaatgacaa ggtagcaata gaaaagctga ttgcgccgca
196081 gcacactaag agcgtggcta taattttacg atttttttact ctgaaaattg tgcataaaaa
196141 aagcactagc cccactagta ctataaaagt atcaattctg acgtcgtttt cgatgtttga
196201 ccaatcaggc ggccagaaga aataatgttg gtcattatag agaaatattc caatacctgc
196261 aatccagaga ctgattaatg tttgtagaac tgttaagact aatttaaaag gatcagatct
196321 aattttttga taattttctc gcatgtttat cacctacttt taaattcgat ttctagcatt
196381 gcaaatgcca agtttccatg gtgttggtgt tcagagtgta ggagcttttt tagtttcttc
196441 atattctctc ctaactagcc gcccttgcgt actgtttatt tcttaggcga ctgcttttat
196501 ttaagctaat tcatttttaa cataaagagc agttgcggta actgaattgt ttgaatctgt
196561 aagaatactt ccaattacag cagcaaccca aacttgagct gcactgtttg tcttgttgat
196621 tacatagttt ccacaggtga aaccgttaga ttgagaacca atccagcatt tgaaagtata
196681 gcctgggacg gctggagcgg taatgtcata gcctatactg aaggccacct ttggatcagt

196741 aacttcactt gcttttgtag aagttaaaac tgaaacttct tcaaaatgta aagcgccatg
196801 taccgcaaac atattcccaa cagtacctaa agttgtacgc tttaatccat cttgtgaatt
196861 tccaatcaat acgctatctg tagtagctgg gttaccgttt tcacttaagt tcataaattt
196921 aatatctgcc atattatttc tcctcattaa ttgttaaaat atctattctt caaccatgca
196981 actctttgca tgtaccatct tccaatacgg ttgacgttat caactgacgt gttaggcgtg
197041 taaggccaca catcaagttc tgcatcaaat gctttgagtg gaatatgtct gacaaaatca
197101 acaaaagctg tatctactgc tgaatcagac ataactccat caactaaagc tttggtgcgc
197161 tttaagattt catctttata aaatttccaa agctgataca tcaagcgatt atcatttgta
197221 acattttcaa aagttacacc atgatctcgc caatcattag tatcagactt agctggcata
197281 tgctctaaga agtccggtgt acgtccaaag attgaatcta agtcatatgc tgcgaaatac
197341 cacttcttgc catcgaatgt ttgaagtaaa tagtttctga aaatgccatc atcgttatcg
197401 actaaaactg aaaagatgta atagtcaatt gcactgtcaa tatctagtaa tggactaact
197461 gccttattga agtcatccgc tgtatcgtaa ttagctaaaa cagctcggat taactcattg
197521 actgaatctt tgacccatgt agtatcttta gtgctgcaga attgcaattc catctggtcg
197581 tctttcaagt tggtttcttt caaaaaagct ccttgtggcg tccagatagt atcaataatt
197641 gcatatttgt tctcagactt cttaggcatt ttagccatcc aatcatcttt aggaatgttg
197701 aatgtatata ttccccaata ttgaccatta atataaacac caatcggaaa tccatctacc
197761 gctccataag taccaccaat tgatagttgt gggtctgttt cagcaattat gcggttgcct
197821 ttatcgtcaa ctaattggtc accgttcgtg ttgagaattc ccgtatcagc agtcctgtgc
197881 gtagctctga tacttcccca aagtctagca ccaaccacgt tgagcgcttg tgatggttca
197941 gtatagtttg ccttgattac gtacttgtga ttctttccgt atcctctgaa agcttcaaaa
198001 ctcttatcaa gatttaaagt gtaattcttc ttaggttgac caacgctaga agcaccctgc
198061 actttaaatt tttcaactgt gccagatact ccataagctg ggaaactata agtgacttcg
198121 ttctttaaag tcttagattt atctttcaga gataaaatat tagatcccca caggtaaaga
198181 actggcatac catacttttc agacttgaaa ctagtagcat tcttgattgc ctcccccgact
198241 gcttgaccat cggctggtaa tccagcttgc gtaagagtga catcagtcaa cggtagccac
198301 ttatagccac caatacggtt accgtagtca tctgtgatat ggttaccaaa gtcatcaacc

198361 agcaagatat ccatcagatg accagcaccc tctaagttct gaatatcaac gctattcttc
198421 ttaattccta atgaattaag tccaatatca ttggtattct gttcaatctt gccttcttgc
198481 tttaaaatct gttctttaag tggagctatt tcagagttat caatgccaat agcttgatat
198541 gcaccgcaat ctttccatgc tccatagaga taaacccact tgtgacccgt gtcagcagta
198601 atgaagatac catctgcacc attaggatag ttagtagtta actctccagc gttagcaact
198661 gcaataggat tggtcttcat ttgtgaaaga cgttcattta ttgcactgtt catcgtatcc
198721 attcctgttt gaaattcagg tctggttaca atgtcatagt tagcaatctg ttgttgcgtg
198781 cctgctaaat gttgagaaat attagcttgc tctgcacggt tagctttaat ttctccattt
198841 aaagcgtcta aggtttctct tgaagtttga agtgaagtat caatgttgtt cttggttgta
198901 actattaatt gatttacttt ctgagtacca tcagcaacca tttgatctgt tttaacttga
198961 tattcacgtt ctaagctatc aagctgcgaa ctgtaatact tataagtgtt accaattcta
199021 agatcatttc ctagcacttc gaacacgatg tttacactgg ttaagacgtt gccattacca
199081 tcttttaagc caaagtgtcc cttaaaaata ccttcttgag gaaacatctg atcttctaag
199141 atgtagtcca tcactccgcc atcatgtgca ttttccattg agccttgcca ctcacggtaa
199201 agagcatctg gagccatgat aatttcgttg gtatcaggat cttcgtaatg cttacctact
199261 gcaccttgga taaatggaat aaacccgtga acattcatta cacgtccttg gtcataccac
199321 ttaaaaggaa gaactttgcc gttatcgttc acacgaactt taaagaagtt gctaagtctt
199381 acataacctt cgccttcttt agcaatgtca gcaggaaagt aatacggttg tccgttattg
199441 ttaattgttg gaatattatt cattagtcaa gcttcacctc ctcagctatt tgactatgat
199501 tttcttgatt aggactaatt gtttgaattt ccgtagcacc atcaccgaaa acggcaagac
199561 ttaattttgc tacttgtgtt tctaaagctt taattcgttc ttcttttttc ttcttattaa
199621 catcattgct atcttgatca ccaatgatat cctgacattt ttcttctgta tttttgactt
199681 tgctttctaa ttcagcaaac ttcttattgc cggcttccca attccgatca agatgttctc
199741 tataggcggc atcttgtgtt ggtgagcctt ctttttcaaa aacgtcagcc atctactcac
199801 ttccttttaa ataaaaaaca gcccattttg gactgttcat actatttatt cgtatcattt
199861 tcatcttttc ctttatcaga tgtatcttta tccttttgac tatttaacag ttgttgcaat
199921 tcctgttgta gtttttggat gtcttgcgta ttttgctgtt gactctctcc ccagttggtt

199981 gtgaaagtat caaagccagc attagcttta tggactattt cagctagatt actcatctta
200041 agactcatac tggtgtacca ctcacgatat ttgtcttgtt ggttatgctg ataagtcatg
200101 taatcaataa ggttaaaagc taaattacca aaagtaattg tattaccttt gcttggatct
200161 tgcggatatg aagtaattga ttgcactctc gtctcaacat caattccttg cctggttcgc
200221 aaataaccat agtcaccaat actgacatca tgtagatact tagcaaactt cttaaagtta
200281 gcaccatcaa ctgtgtactg aacgcttgga taatcgtgga ctagcttttt aaccttatca
200341 tcaagcaatt gttgactcat cacgaaagac ttaccatcat cagtgaagtc ttctgtataa
200401 ggcgtggcat caattatagg ccagcccttt tctttaacca atggagaaat ataagttgag
200461 gttaaggtat tcacactaga attatctcca ctttgcttag tgatattaat gattcctgta
200521 gctctagtag ttattgcagt atcgtcttct tgaactgata tcttagtaca gttcacattg
200581 tcaacgaaaa cgaatttatc tttgctacca atctcttttg ctacatagca agtcttattt
200641 ttccaataat attcaacacc gtagctagtt gctgcagttt gtaaaagttc gtcagcatag
200701 ccgccaccta gcccgtcact aaatgtttta accgagtttt gattaacgtc tgaattaata
200761 actacttcaa agcctgaatc cttgaataga aagttcagag catctttgat tttaactggt
200821 ttagatgttg ttgacgtatc tgtactatca ccactcttag ttgtggtagt ctctacacca
200881 atcttatctt tgacatagtg ataatggaac attcgggcta tttgtattgc acttactgag
200941 tactgtatat agttgcctat atcatcagta ttgtaagtct gcaggacata atcttctcct
201001 ttttcaggaa cactaattat gctaaatggt gctaaaacat cgccatctac cttattggta
201061 tggagattat agaaattgaa actcaacgtg tctaagctac ccaaagttga cgttacttga
201121 acatcttgtc ctttaactat tgctactttg ccagcagcag tcttaattga tatcaagtaa
201181 tcatctccta atagtaaaac ctagtattga acttaattgt atagtcagta gcgccgtcaa
201241 cattaatatc attccaacct ttactgaaat ccagataggc atgattagtc ttagaatact
201301 gttctatgcc gttaatagtt ggaacaatac catcaattac aattgtgtct tgcttagtat
201361 aactaccaga caacttaaat acctgattag ttgtactgtt ggttatcgtc aaagcatctt
201421 tcacattacc gttaaaagta atctctacag gcaattcatc agctttcaaa tcgatgttag
201481 aagcattata aactctgcag ggacttgaat tgaaaacata tttcagttcc acaccatgcg
201541 gaatattaag acctaagccc catttgccac tttcataact gaaatcacta tctgctgtag

201601 ttgctactgt ttcagcaagt cccaacggac aactcaaatt aatggttgct gtagcttgcc
201661 agaagttttc tatttgtggg tatgaaatag ccccatcaac aacacatttc cagcgtaaat
201721 aaggaatacg attggtatag atgtaaaatg gttcatcgct gttaaagata cgctttagat
201781 ctagtctctt aagttccaga tctatcgtat cttctgcttg gatcattaaa gtcagcggaa
201841 tagtcatctt gtcaataacc acatcagtta gaacttggcc aactttgctt tgttgcacgt
201901 aagtgtgggc gtaatttacg cccggcggtt caaaagtaat aactctaaag cctagtttgt
201961 ctaagtcgta ttctgtaccg tctaatcttt taacaaaaat actagacaat tggtactgca
202021 cctccttgtt gcttaatagt aatgtcgtgt gcttgtaaca tcttaatcgt tggataagtt
202081 gctctcgcca tctctttgtc agaaactata aatttaatca ctgtattacc atcaattgct
202141 tggccttgat tattaacttt aacaatattt tgaccagcta cagtgttgct atcaggtgtt
202201 ccataaccag ctactgaact gtactttact tgttccataa ttctagctaa tgaagccgta
202261 ggactttcag gagctacttt agcacgttgc aatatagcct tatcaatcaa gtgatctgca
202321 gtcgattttt gtggattaat cacaaattca ggaccatctt cgccgacacc gataacttct
202381 ggtttatcga actcgccacc attaccgtgc cagctaccag agaatcttcg gtgaccagtt
202441 ggactccagc cagcaccaaa gtggatatca ttcttccaat tagagtcatt aaataaagct
202501 ctaagttggt tagttccacc cataatgctg ccacccatgg tataggcttt ataggtgcca
202561 cgcttatatt gcaaaagtcc aatagccggt cctgaaccat ctccatcagg atcagaaccc
202621 ggttgaactg ctttagggtt accgcctgat tcgcctttaa tcatagataa gatcttagct
202681 atatcagttc cggtaacgtc agcatgcatt tcagcagcta cttgtttaat agtttcgccc
202741 caacggctga caccgaagcc actcggatct gcactatcag caccaatacc aaataaaccg
202801 ttcagcttat caatcatgct ccagaaacct ttaagtcctg gcatcttttt gataaatgct
202861 tctagtccat tattagcttt agcattgcta ctagaacctt caccttttaa gccaggaacc
202921 cgtctataac taatattgct tgatggacca tctttaacgg ctgacatacc aatattagga
202981 tttgagcttg gactcattgc tgaccaatac ttaccaccgc cggcatagac accaacgtgg
203041 tcgctaccac cagggccaaa gaatactaag tcgcctggtt gtgggttaga aactggtgta
203101 gttgcgttgt attgagcacc cgaatagtga gggaaactct tgccaaaggc tttttgcaag
203161 gcatacatga ctaatccgga acagtcgaaa gcatcaggac cagaagcacc ccatacgtaa

203221 ggttttcctt taccatattt ttcgactgca cctagaagtc cgccttctgc accaccacca
203281 tcaagtttgc cagaaaccat tgaccagagt gttgaccaga aagcttgtac atttttgtta
203341 gcttgatcaa ataagccact agccagctcc ttcatggatc gtttaccgct attaaagcct
203401 ttgaagttga agatatcctt cacataatca attgggtgag caatgatctt agtagccatg
203461 ttgaataggt ctttcaagtg acctgccgtg ttaccaatcc atgaaccaat gccaccaagc
203521 cagcccatga aaccagtacc tttagcaaag tgagtaatgc ccatgccttg cataatcata
203581 gcggtctcag acgcattgag tatctcatca cccggcatca gcatagttgt ggtatttctg
203641 ccttgaaaaa caccaaattc gccggtagct ggtctgaata aagcttcttt gttgcctgtt
203701 tcagggctat caaagccatc attaaccatt gctaaggtag gttcagttat tgcacgtcta
203761 ggtccactaa acgcaccagt accactagcg aacttcttag ggatcttctt aattgtgtta
203821 cctttaccac caaaagcgta gataaccgtg ttaatgccac taatacctgc gttaacaata
203881 tcaatcaggc cacccatagc gtccttacct agctttttca tcgctgacca catatcacca
203941 aagatattct gaacgccttt agataagctt ttccaaccag ctttaaagtt aggaccaaag
204001 ttatctagcc agtttttcat atcagaacca aaccgtgaag ttctgtcctg catcttggac
204061 catgcggtac ctaagttctt tctagtatca gaccagccac gattccagtt cttgctaaag
204121 ttcttattaa agttgtcata ccatttatgg atattgttac cccaattact ggtattccgt
204181 ttcatacggt caaacgaatc aataagcgcc tgcttgctgt tcttccaacc gttatcccac
204241 ttctttttga actgttcact gaacttgtcg taccacttgt gagtattagt accccacttg
204301 ccgatgtttc tctgcatatt tctgaatgag tcagcaagtg ctttttttatt atttttccaa
204361 gccttgtccc acttcttgct gaagtctttc atccgagatt gagcatcttt aaagagagtg
204421 ccgaatagat tacctttctt aaaagccttt acatatctat tctcacttag ccctttaaca
204481 aagttgcccc agttcttaga tacctgcttt ccccagttac caataccagt ccacaatcct
204541 ttaaagaaat tgtgactgtt tttaagcatg ccatcaaagc ctttttttgag gttttggcct
204601 agactgtcga cccacttctt aaacttagga ttgttgtcat acagcaactt aattgcgccg
204661 gcaataggtg caaccatcag aagtgttaag ccggaccagt tcttcttgat ccagtcaata
204721 gctgctttca tccctttaga gatgtttttta ggcaagtcag taaagaactt agaggttgtt
204781 ttgctgatac tattccagct gtcagagatg tttttgccta aatcagagaa aaatttacca

204841 gtattcttct tggtatcatc ccagtcttta acaaatgcct tagtaaaggc ttgtacattc
205901 cgtaccatac catcaaagga tttatgtaca gtatccgctg tcttattaac ccattctcta
204961 aatggtttga tatgcttata agcttcatag aaagctaaac caagtgctgc aataactgct
205021 actgtagcca taaacggatt agctaagaaa gccagtttta gtaaatcaaa agcaccttta
205081 acaccgttga tagctttacc aagaacagga aacgccttag atataccagc aataatagtt
205141 ggtgcttttc tgagagtact aatagtgttg cctatgccga caatgcctag acgcatagta
205201 tccaatactt tgaaagttgt cttaccagca acaattgcag caataaggcc accgaatact
205261 ttccccagcc tttcaattga cgctctgtgt ttagtaactc catcaacagc cttaccagtg
205321 ctatccatat aaggaagcat ctcagctact ttagaagtaa ctttgccaat agcgttacta
205381 atgtcttgaa atacggttac gataccctca aatgttcctt tagcaaaaat accaatgaac
205441 tctccaaatt ccttgataag tggacgcaaa cgaaccatga cagcgttaat agattggctt
205501 agactttgga acgtttttgc tactcctgaa aaatcaagag aagatcgttg ctttccgata
205561 tttctaaagc cttcaacaat gccactaagt aggttagcaa atacgttaaa gacattcgcg
205621 ctaatcacac caacaaaacc agctatagct tgaagtgctg gagtaacggc cttaccaata
205681 tctctaaatg aattacctat atcactcaag tgagagcctt gaaaagcacc agctatatta
205741 gctctaaaag acattgcaaa ggtattaatt acagtcatca caccgttcat aatcccagca
205801 atggacttac caaagttttc aaaactcttc tcagcaccgc tagaagtaat ccagttactc
205861 atcttgccga gtaatggatt ctttagatct tgaaatggtt taacgatagc accagctaaa
205921 acaggaatac gcgcatgaat ggtacgttcc ataccgtcca tagtctgaga gaagttatca
205981 gtagcattct tatacttctt gcccatccct ataaggacag tattcattgc atcagctgat
206041 atcttaccgt tagacatcat gtcacgaact tgcgcggtag ttagcttact gttgtgagta
206101 accttacgct catattcaac cagttcttcc ataaacttag ggaatacgtt ctggatagac
206161 atcatgtctt gtgcattagc tttaccatta ccgatcatct gtgaccactg catagcgaag
206221 ttctttacag cgtcatcact ttgaccaaag gcgtcttgta aagtaagaac agctttagat
206281 aaatctcttg tcttgctaga tgagttagta acagcgtaga atttttggtt taagtcatta
206341 accatctcca cgctgttctg tgctgcaata gctaggtcat tagtctcttt aactaattct
206401 tttcctttac cagctgaacc ggttaaagtg ttccattgtg caagcatatc ttgctgcagt

206461 cgattgtaat gagtcccttc tgtaatcact ccaccgattg catttttaat
tgtgcctaaa
206521 gcagtttgag cagcattagc ggcaaagtta ccaatgaaag tacctttaat
aatctcatgc
206581 aacttagaaa aatgctttcc tgcattgtca gcattacgac tagcactgtc
agtagctttc
206641 ttagtgttat cagtactatc aagcttactg ttctttctat cgggtacttt
gtcaatctct
206701 tccttagcct gtcttgcctt ttctacagca tccttattat cagctttttg
ttcggtcttg
206761 gtttcttttg gaacagaatc aagattacgc ttagtttgat tagtcttttc
ctgaacatct
206821 ttattatcta agtttgcttt aatatcgaca ggcttgctaa gcttttcgtc
aacgtctttg
206881 ctggtttcat cagccttttg tttaacttta tcagttgact cttcaaagct
actatctagc
206941 tctttgcctg catccctacc aactgaattt aagatctgat ctattaactg
tgcatcggtt
207001 ttaaccttat caacggggat gtctatatca atagttatcc ttccatcggc
catttattaa
207061 cctcctttcg ctgtgttcat caaggaagca aaggcagatg ataaagaact
actgttaaag
207121 acatcttcct cagtcttagg tttcttcaac ttcaaagagt agtactgttg
aagttgcatt
207181 accctgcctc gttgttcatc tggtacatca gctaaattct tttgtctcag
ttcaattatt
207241 ctttggattg gtgtatcttc acccagtcca tcaaaaagag cacggaatac
acaccagtgc
207301 attttcccgc gctcttttaa aagatctatg tggtattgca tcataaatga
tgagtaaata
207361 gcgtctgcat cttgctcata tgagtaaata tgctgtggtg ctatattcat
gctccaatcc
207421 tttgaagagt taccttgata tgctgattta gtaatggttt cgttgattaa
cttgaaacta
207481 ctctcaataa aatcaggatc taaaggtatc tcttggtcgc caaagaataa
tttaagggct
207541 tttagttcct tgttttcact atcatcttct actagctcta agtacttgat
tacattatca
207601 aaagctaaat ttatttgata tgtttctccg tcaaacttga tagctgataa
aggcgtatca
207661 gttagactta acatagtttt aacctcttac tgaggtaggg gtagctaagt
gcttaagttt
207721 cttaagtttc ttttccttga cttcaacaga cttaactgct tcatcattta
aggcatttaa
207781 tacagcacct aaagcatcgg ttgaatagtt gtaatacttg taaagtcgct
taccttcccc
207841 attacccaag agtttatcta atgctgaaat agctgacgct ttcacagagt
tgaaactatt
207901 ctctacgtct ttcttttgaa cagccatatc tttatctgcc cagcttggat
catctaaagc
207961 tttaaggccg ttcatgactt caacagaagc tttggctaca gtcttttgaa
agttatcatc
208021 aaacaagatc ttataagttt tgccggccag tttaacatct accttgttat
ctactttaat

208081 tcgtttgtct aaatcaataa ttgacattag ctacctccta attttgcttt
ggtgtatcag
208141 cattagtgtg cgtttggagt agttgctggc gtcttatcgt caacagatgc
agtaaacaca
208201 cgtgattgat ctgattcagt cattgttaac ttgcctgtgg ttgtctttgg
tgctccattg
208261 aaagcaatag taagtgaaaa gttttgttgt gcgtctgctg caccaccagt
tggagttaca
208321 gcggttaaag tacatgctga aactactggc atgccgttgt taatccataa
cacacgagtt
208381 ttagcagctg atccgatagc atattgctta ccgtcaatgt agtcttgtgc
agggtcgcct
208441 aagtagcgaa cacctttaac agctaattgc atagacttac cagttacttc
ggtgtcatca
208501 tgcccatctc catcgtaata agctgtcttt tgtgtttttt cttgccatga
tggtgtaatt
208561 tcgctaatac cacgggctaa ccaagcccat ttaccagttg tgatatcgga
taaatcgtta
208621 tcattgtctg taatatcaat atataggcgg ttagcaacgt ttaacgctgc
accatctgta
208681 gggatttccg tccctttaat ctgaactaaa ttagtagcca ttctctaatc
tccttatttt
208741 gtgtaaacaa aaacagcaac gtctaataaa tacgtcactg ttccttgcat
atcctgttct
208801 gtttcactag gggcgctaga tacgtcaatc ttgtcgaaaa caaaattgct
actatcgctc
208861 tcaatcgctc ctggctgtaa atcatctaaa aataaactga tcttaaaaag
atcatcttta
208921 atctctctag cgctcttacc acgctctgta attgcataat tgtataccca
gtactctcta
208981 ccactaaaat cagttgaaag cttatgagag ccctgctcag gaactaaacc
aatagaatta
209041 tctggtgcta ggtatgcaat tttgattggc aattcagttc ctttgataat
tgattgtgct
209101 agagcttcct gtagatcaaa cttaatgtca ctcatgccat ttcactccgt
ttataaaagc
209161 ttctttaaca agagccatct ctgaaagatt gcccttaagt cttaagtccc
agcgtcttga
209221 tgttcctggc gtggtgtagt tatgaattct aaatggacca ccatattgat
tggtaataaa
209281 tccataaaac tgtgcccttg cataaggtgc aacatagtaa atgctagaac
catcagttgc
209341 tactttggca tggtcacgta aatctccact tttcttaggt acgtacttct
ccattgcctg
209401 ttgtgcatca ttagctatag ctaaacgccc acgtcttaac tgatcttcac
tgaacttgtg
209461 gttcatgata ttaagattcg tcctaacttt aatccccatg ctgtcacctc
aaatcattgt
209521 tagcttgtat tgataaagct tattactaaa cggctcatag tcttcattaa
tattggttaa
209581 cgtgtattct tgcccttcat agataatctt tgccttgttc tcaatatctt
gcttagatag
209641 cgagataaac ggctctgata taccttgata gagcactact gtaccgttag
ctacaatttg

209701 acgatcattg ttagtccctg agtagattgt tctaagatgt accacacagt tctgtatttc
209761 aacgccatcg tcatatgtag cttcattgta tagatcatct tcaaatttac gtttgattat
209821 gattgattgg ttacacattg attttggcgg ttttaacatg atgagatacc tctataaagc
209881 aagccagtat aaaacaaata ttctaaggct agattataaa caccgttagt tgctgaatcg
209941 gttggactag taccagttga aacacttgta ccatcaatag atacgctctt gatgtctttc
210001 tgtgccatct cataggtact agaggctcca atgtcactca tatactgaat ttgtaaagct
210061 aaagcttttt tgaaccaatt aactcggtta atgtcagtat cttcgtcaat agaatgtagt
210121 tcatagtaca tccctgttaa aggattaatc atactttcag ccgctttttc taacttatca
210181 aaatcagcat ctggaacaat accagttagc ttctgaaact catccttatt aagcaatgcc
210241 atttagaagc cccttgtta cttattagct atttctactt ggtgctgatg ctgcattttg
210301 ttgctttgaa gtatcagcag gtacaaacaa cggatcaatt tgaaacttgt attcaactaa
210361 acgtacgttt cttacgtcca tatcttttgg caaactccag gcttctttct tcttgaagtc
210421 gtctggggtt ggaaagttag gtttgttagg cacaaacgta ggactaattg aaatgccagc
210481 cggatgaata caaccaacac gtttttgaac tacactctca cgtccacctt gcttaagtgg
210541 ttcatctaca acttgagtac tgtttaataa agttgaataa cgtacggctc ccgtaccaaa
210601 taaataggct acagatgttg aattgctacc agatttttct acaggaatag agtcgtcaat
210661 tactacttgt tttccgttat aagtgttaat tggagttcca ccatttgaag gttgaatagt
210721 atcaattaag ttttgtgctt tcatcattgc ataagttgcg gagttaacag aaataccagt
210781 tagcatattt tctggttggt ctcccattaa tccaagtgct gcaataaatc cttttgcact
210841 aaattccgca tttgtaggtg acttagacgt taaatcaagg attttagcat tagccacatc
210901 gtcaacttgg aatgcacctt ctaacactgc taaaagcatt tgctcatcac tagtattcca
210961 aaagttggca aaacgactag caatttgttc ttgaataggt gcacctgaaa gcaatctaga
211021 gatttcagtt tcaccatagg ctttagtttg gtagaacttc atcccaattt gagagccaga
211081 tgttaggttt gatacgttaa tatcttgctc atcggtccag ttatcgggtg ttccatctaa
211141 gtcattgata tatggaatag taatctttgt ccctggttgt gttagcttat ttcctaaatc
211201 tgaatccgga gtcaagatac cggaattaac taagttgtta gtcttggtag ataagttttg
211261 tacccagtta ccaaagacag taggaataat catatctgat aaatgtgttt ctgccatgtt

211321 ttactacttc tttctaaata aatttgccca gccttgtgga tctttcttaa acatttctgt
211381 ttgttggtca agactcatgt tagctggatc ttttggtact tcggacgtgc catttcctga
211441 aacaactacg tgagtagccg gctttggttc ttcttttggc ttgtcctgtt tgaataaaaa
211501 gccgtaatct tgtttagcag catccacttg ttcttgaaag ccaattaaat tgccttttc
211561 atcaagtgat actttatcgg tgtcaataaa aggtaaaaca gccttgtttt caagtgctcc
211621 agctttacta agagccgttg aaatagcaaa ctcttttttt tgactagcta acttagaaga
211681 ccattctttg tctttttctt tgttagcctt ttcaaactca gctactttag ctttaagctc
211741 ttcattatcc tttgcttgac tacccaaggc tttaatttgc ttatctcggt ctgagatctg
211801 tgacttatag ccgtctactt gatcgtttaa gctagctagt ttacggtcat agtcatcacg
211861 taaggcattc gtttccttac cagcttgtgc cataacggcc ttgatctgtt cagcatttaa
211921 gccttgtttt tctaaaaaat cacgttccat atctagttct cctaacgttt aatttatacg
211981 agggacgacc tcgctcagag cataataaaa aagcagttta gcgacatact caggtcgatt
212041 taattaattt gttttgacta tctgttcacg atcatattga cgtgctaaga agtcattatc
212101 ttttacaatc tttcttaatt tagcttgata accccttatt gattgattta gctttctaac
212161 gttttcaggg tcttcatccc tctcagcaag ttcttttttg tatttaaggc gtctaatact
212221 acgttcataa tatctttgct gctgctgtat cttagcgttc ttgacggctt gcttaggatc
212281 ataatgcttt tgaaagttat gagatacacc cttgatgtaa ggataaagaa tgtggctaca
212341 gttaatcttt tctgtaatct tgtattacta caagtgtcgg actatatcat ctaaggcttt
212401 cgcctcgcat tgcacttcga ccggtagctt gtcttccggt ctactctact cgatttttac
212461 tctttcgata gtctctacac ctttttaata aattcccagc gataaccgcc agcacttttt
212521 ctatactttt tgtgtaagca aactgcacta atttgtcttg aatttatgcc agttgctctt
212581 tcagcttctc gcacaccatc aaaagtgtta attagtttgc catctttagt atattgacca
212641 actctctttt taagagaatc agtgtatttt tttaattgct ggcttttctt tgctaaatat
212701 tttggatccc atatttagca atatctttat gtaaattata aggtctttga tttattgcac
212761 tttttttaaa gtgatctata aatctttgat gaatatcagt tgactgacca atatatactt
212821 taccagttgc cttattaata attatataaa ttccaatagt tttattcatc gctgagaacc
212881 tcttaaactt ggcacgggat taacctaaat taggctttcc ccgttagctt taacaatatt

212941 aatctatata actattatat catttatgta gacaattaat accgttaaaa cacccctaag
213001 ctaagggttc acaatgttta tacagggcat taacgtctac cctgtgtgcc ggccggcttg
213061 ccgtatccat aatcgtaaat attagggtac gaaagatcag cctttggatc acttttagga
213121 acaatacaca ccactttgcc ctgaataggt gcacaagctg gtcttgctgc tggatgacta
213181 gacatagtag caagcacact tccgaactct ttcatagatt gcgctcttgc ttcgttaaat
213241 actcgatgtg ttgcagaagt aataacggtt cgtgtgtagc cttctagcgt ccattcatgt
213301 cctcctctat cagttagggc tgtgggtaga cctttgtcat accattgcag aatgttgtct
213361 tttagtgccc tgtcggctgt cttgttccca gtagatacat ctagcactgt cttgtctact
213421 atctgctgat aggtcttaag agcactattt ttttgaacat tagtagttag cagcgtttga
213481 ttaacatagt tattgacatt tctaaaggtt tgatcggcat aagcattaat aattgctact
213541 gtatcatcac tcacacctcg cattggttgc tttaatgctg tggataattc actgttcatt
213601 cttttagcaa ctgttaaacc ttggccttga ataagatcat aaatagcctg tttactatag
213661 ccaatgtttt tagcaataaa gtcaactact ttatcagtta atccacccat ttcagccagt
213721 gcctttgaac gccattctaa gatactttta tcatcaacac gtgtaagcat acccttgtgc
213781 tttttaaaag cgtcaatcag caagtaaaag gtatactgtt gcaagtaatc atagtaatca
213841 actatcttgc ttgcctgttt catcatcttg tttagttccg gatccatgct tatcagttcc
213901 cttgttctta gtatctggtg ctaatggatt gaattgctca aagtcagggg ctggtgtggc
213961 cttttctttt tgaagttgtt ccgcccagtt ttcagcctca tcatcgctca aattatagtt
214021 acgcttaaca aactctttaa caggcatagc accagcgtta agagctgata aatcattctt
214081 aaactgtgca tcttgatcta caaatatgcc gtcattgaag tcaggcgtga ttactaggct
214141 atctaagtca ccagtccatt taggctgttg atcgctccat aattctggtg tctgtagcag
214201 ttcagcaatc gcatagacta attggtctat ggtgtcctct atctgcgtta agtaacttga
214261 tctagtttga taagtcattg agttgcttga aacaacacca gtagccgtct gaatgccagt
214321 aggcgttgct gtgaatgtac cttcacttaa tccaatctca ttctcaaact catgtaagaa
214381 gtagctcatt gctgcttgat attgatctgt acgaatgtta atagtcaaat ccttaaaagc
214441 actgctgtca tctattctag cattaattgg tacaaataca tcatcatctg tgtcccaata
214501 catttgctga ttttccggaa taggattacc gttaatttga gtttgacgtc ttacccagct

214561 ttgagggatg gttacacgtc ttcggccggt tttaacatcc caataaaatc
cgtcctgtgt
214621 cacattgatg tcatctaaga tattccggca attatcgcat aatcctaagc
ctagtgggct
214681 agtaaaactc ttattgttgt caccaggatt tcgatagaag gcaaacagcg
gcttagttat
214741 atgcgtaaaa gtagcttgtt gtggcaagtc agcatactct tcaatcgaag
ttaaaggtac
214801 ttgagtgccc gtttcgcttt catcagttga ctcatacagt tcattagtaa
ttgtgtaagg
214861 tgtgtattcg tggccgttct catctgcttg tttattgccc cattgatgga
actctagcag
214921 tgtgtaatag tgtggctcgt cgttaacagt cttaacaatt ttacgagcta
atgcaatctc
214981 tctaacttct gtagtgttag cagtcaatgg atatacgctt aacgcgtcag
accagttcaa
215041 tttaatctta tcatcttcaa catacggcct aattgcactt gatcctaatg
ctatagcacg
215101 ctgtaaatta gtctcaaatg ttgtgtaaaa gcgactttcg cgaaagatct
catcaagttg
215161 tttttgtaaa tcattatcat tcaccttaat agaacactgc tcattgaaga
taattgacgc
215221 taggcgttta gaagcttttt gcgtcatatt gacggtgttc atcttacgct
ttcgttcatt
215281 accaagtacc caatacttaa cgtctacagg cttatcagaa taataatctt
tagccctgcg
215341 aatacgtgta tattcttctg ccggcactgc aattcttgga tcatctgtaa
tagctcctag
215401 tgatttaatc atccctaact tagcacctcc tttcctgaat aaactcttaa
tgctagccca
215461 tagtcccata tactcaccac cttatgctgc tagtccgagt agttgctcat
tatccacaat
215521 agcgtactta atggcatcgc aagagtgatc gtcttccttg ataacgcgcg
gattatcgct
215581 attaactgta gctggatccc actgatattt ttggtgttct tgtagaaata
tctcattgcc
215641 tggtgttttc aaaacatata aacgcccttg tgctagaaga tcctcaacac
ggtcaatcat
215701 tgctgcttca ctcaacttat gtaccttaga ccactgcgtg ttgtacattt
gccaatattg
215761 tgtatagata ccaccatcag cagagtcgat tgtctgatta taaggtagaa
cgccgtattt
215821 attggttatc tcattgataa agtcatgcac acgctcagcc tgttcagatg
ctgacagctt
215881 acgagcgtac ttagttggat catagtaaaa ggtatctagt acataaacgt
tgtacttgtt
215941 agttaaagca cacgctacac aagcggtagc agataccata aatccggtat
ccatgccgta
216001 aaagatttca gtgatatatt catcatctgg tatcttatcc acaaccttaa
acagattcat
216061 attatagaca ttagtaccca atccaacagc ttcaccaagg tatagccagc
ggtaatagtc
216121 cggatcatta cgcttatatg tctcaatcag gtctaattgc tgcttagtgg
taaagcctag

216181 cttgtcgtct aagtaggtgc ttgtgtctat gaagtaatca ggatcagttt ggcgctgtgt
216241 aatccattca ttcacccatg cgtaaggatt gcgcggtgga ttgtatgaaa tataaacctt
216301 tacctgctct gcaaaatcgg gtttttgtct aatgaacgta ggtaccgatt gatcgaatac
216361 atcaacactt tttaagttag caaactcttc aaaccaaacc gctatcacat tacccacaat
216421 gttggattta agtttcattg gatcattagc accgtagaag tagaatgttg atcctgtcct
216481 tatatgagta atcgttaaag gtgatacacg agttctgaac tcactttcaa cgtgcaactt
216541 attcatagcc catagaatct ggttataaac actgtcacgt aaatagcgtt gattttcacg
216601 tacacaaatg atgtttactg ttttgccttg cgcaatatac ttcatcattg tggtaacaag
216661 cttgaagctg attactgacg acttaaaaga accacggcca cccttataaa cttgataagg
216721 cttattacta ttccatgcgg tataaaagtg aggattaatc tcctggctta gttttactat
216781 tgccattgct atcaatctcc cttatatcgt ctacaatgat tgtcttattg cttggatcat
216841 cattttcttc aagcgcctta agctgtgctt cactaagttg tgcctctgca agtgtcttac
216901 gagcattagc ttcattaagt ttctggaatg tttggtcacg gtaaatgtcc ggacgcctat
216961 ttttaagcca gaaaataata gcagaagcgt ttggatccac ttctgttact gtcttgctta
217021 taggtatttt ttcataaacg tcaacattct ctgccgttgc aattgctatc tcttcccgtg
217081 tagcttgtgg atgatctagc ttataaatgt tagcaaactt agtacgttca gctttaagat
217141 taaactcatc tttcttgacc atcttatatt gtgtagtggt aagagtgtgt tttttcataa
217201 ctgaaatgaa agagtcttca atttcagcat caactatttc tttacctttt ttcagggcgt
217261 cactcatgtc actatatgta cttaaccatc tttgaaaggt ttgacgttta acaccaatct
217321 tcttggctat ctgttcattg gttaagccat ctctagccca tgatctaagc ttagtgagat
217381 tatcaggctc tagccatttt ttgtattcag catgtgccat ctaatccacc tcatcacttt
217441 aaccaccttt gctaccaaac gaacttcttt taactttaac gccacttctt cttagagcaa
217501 tctttactga tctaacactt cgttttgcac tagttaatgc tttagctaat ctattaccat
217561 tagctctcat gctttgacca ccagtaacac gattagccat tcttgtaatt cgtcatcgtc
217621 ccaagtagca agttcaccag atttattatc tgctaatcta taagcgttga cttgctcttt
217681 agtaagatta tcagcaacta caattggaac ttctttatag cccaatttct tagcagcctt
217741 gtaccgtgta tgacctgcta tgattaccct cccattatcg acaacaatgg gctgctgcca

```
   217801 accaaattcc ttaatagaat tagcaacagc gtcaactgcg tcatcgttat
ttctagggtt
   217861 attttcatag ggcttaatct tatcaattga aactgtttca acttttatgc
ttaactccta
   217921 aacttatcta ctaaattcca aatcaatcca caaatgatac caactaaaag
aattaatagt
   217981 atgccaccgt aataaattaa aaacgggctt aggactagcc accatgacca
tgtaataagg
   218041 ttcataattc tggctaccgc aaaaataatt gttaaaacga ttgctaaaaa
tttcataaat
   218101 atctccatca aaaaagagct agttttgcaa ctaactctta ccaattttta
aactgtgatg
   218161 tctatgtcct atttcactgg ttcccccagt tatagcaaag tgtggagtcg
aaccaccccg
   218221 cagccgtttt cacgacccgc tctgacctct aagctacagt gctaccaatc
aatttaattc
   218281 tataaattag gaacacacat tgttcgtacg ctatgtgtcc ttatggcaac
tggtggaatc
   218341 gaaccaccac taacttggta cttcatacca acgctctacc tactgagtta
agttgccatg
   218401 gtgttctgtt aaggtacaga gctaaaccag atgcaattct gtaaattaaa
atcagacaaa
   218461 caaaatatat tcgtgatctg aacaatgttt ttttacgcct gctttccgac
aggcaatggg
   218521 caggcgagga attgaacctc gctacataat tgtgaaagaa aattcctctt
ttctttctaa
   218581 atttcaaatt gtgccactct acccacggca gttgctgctg cggtccagcc
aacaactacc
   218641 cagctcttcc tacgctcgcc tcgaaaccgt tgagggtcgt ggcataggcg
tcatttacct
   218701 aggcaactat tatatacaat agccaataat ggactaccgc ctaggctatc
aagcaatcag
   218761 gattc
//
LOCUS       Contig_4              352766 bp    DNA     linear       18-
OCT-2022
DEFINITION  Genus species strain strain.
ACCESSION
VERSION
KEYWORDS    .
SOURCE      Genus species
  ORGANISM  Genus species
            Unclassified.
COMMENT     Annotated using prokka 1.12 from
            https://github.com/tseemann/prokka.
FEATURES             Location/Qualifiers
     source          1..352766
                     /organism="Genus species"
                     /mol_type="genomic DNA"
                     /strain="strain"
     CDS             complement(499..1008)
                     /locus_tag="OCPHGMPK_00917"
                     /inference="ab initio prediction:Prodigal:2.6"
                     /codon_start=1
```

```
                     /transl_table=11
                     /product="hypothetical protein"

/translation="MYQIDLGLQPNLRATAKRVDKFLTINFQSYLNLAGLHRNQLTSP

QLSFAPGSTNKNGVENNFIDEAQDDIDIADPVRKVCAAIYRTMDNCTDTALKPYRRIL

IGTYIDQLRIVDVAATVNLSTRSIDTKKINAQCEFADRWLYWKRYFGVDDLPDLRVFS
                     QKSKNSMAG"
     CDS             complement(1038..1205)
                     /locus_tag="OCPHGMPK_00918"
                     /inference="ab initio prediction:Prodigal:2.6"
                     /codon_start=1
                     /transl_table=11
                     /product="hypothetical protein"

/translation="MDETRNDLEVGNETAVMMYLNILKYAKHHCPEDEDPHEITDRIF
                     TDMLAANKASN"
     CDS             complement(1192..1410)
                     /locus_tag="OCPHGMPK_00919"
                     /inference="ab initio prediction:Prodigal:2.6"
                     /codon_start=1
                     /transl_table=11
                     /product="hypothetical protein"

/translation="MKLYGYEVNTCNYKCFKTEQLKNFRSMLKSNIKNFENIIEPTIE
                     EMIDEDKVEELLPLIEHEIKVRSNDGRN"
     CDS             complement(1407..1661)
                     /locus_tag="OCPHGMPK_00920"
                     /inference="ab initio prediction:Prodigal:2.6"
                     /codon_start=1
                     /transl_table=11
                     /product="hypothetical protein"

/translation="MAIELKIGTRGTREEVEDTYTRSFLEDNGLLKFDPRKFAVNCVW
                     GVHTKYGYMCSFSFDDILIYMGDGIWDLRVAKEDKERPWL"
     CDS             complement(1852..2073)
                     /locus_tag="OCPHGMPK_00921"
                     /inference="ab initio prediction:Prodigal:2.6"
                     /codon_start=1
                     /transl_table=11
                     /product="hypothetical protein"

/translation="MQKIDLSLVSKFVDASIANDKKLALKLAKKIAEQHNCSLSFELD
                     TLDWSANWLKSDERVATQAMVRELRQYEA"
     CDS             complement(2074..2535)
                     /locus_tag="OCPHGMPK_00922"
                     /inference="ab initio prediction:Prodigal:2.6"
                     /codon_start=1
                     /transl_table=11
                     /product="hypothetical protein"

/translation="MRVNFTIEGPPIGKARPRVTRTVTYTPAKTARYEDLVRYTAINS
```

FKGIFDKDEPLDVKIMAYFEVPKSLSKKRKALCLSNQELPTKKPDADNVGKIIMDGMN
PKMKRDKRLHKMVEVMRGVYHDDKQVTTLLVKKRYAKRARVDVRIKRDMGD"
CDS complement(2541..2843)
/locus_tag="OCPHGMPK_00923"
/inference="ab initio prediction:Prodigal:2.6"
/codon_start=1
/transl_table=11
/product="hypothetical protein"

/translation="MIFEIWSVNDIFEDGSMSQKYLNALAKAGFKLVKAKDELSDRIY

KIYIKNLSDLRLLHKIVNHDLIISFPESKPTTKDTMSVMCDSDDDPKIVIYDDWIE"
CDS complement(2936..3094)
/locus_tag="OCPHGMPK_00924"
/inference="ab initio prediction:Prodigal:2.6"
/codon_start=1
/transl_table=11
/product="hypothetical protein"

/translation="MIKTKIITRPKYENYEFEFDIERFSNQLNRKIIDIQYTSNNSNY
EAIITYEV"
CDS complement(3091..3390)
/locus_tag="OCPHGMPK_00925"
/inference="ab initio prediction:Prodigal:2.6"
/codon_start=1
/transl_table=11
/product="hypothetical protein"

/translation="MEVIDKRKENKDEEWKVGDVVCYYNDKKESANYGLIVGPTGENE

YYIAFLDDDAIRGLSIDGGFPEPNVSGTSSVNELISILKSNWKYVEKVNAKLVIE"
CDS complement(3395..3604)
/locus_tag="OCPHGMPK_00926"
/inference="ab initio prediction:Prodigal:2.6"
/codon_start=1
/transl_table=11
/product="hypothetical protein"

/translation="MLEENHNLEKIIEDAKQYRWYSVPDMYMVEILDTTGRSAGFVQS
IFTDKKEASEVAKVLHGVVREVVGG"
CDS complement(3604..4359)
/locus_tag="OCPHGMPK_00927"
/inference="ab initio prediction:Prodigal:2.6"
/codon_start=1
/transl_table=11
/product="hypothetical protein"

/translation="MNNELIKVTVKNDQQLVSARELYKGLGIKRRFSAWWEQNSNGFE

ENSDFTSVLISTEVQNNGGVQARELQDYALTIDMAKQLCLLSRTKKGKEYREYLIEIE

KKWNDPQNVVQRAMDILHSENLQLKLENKSLNRQLEESNKKASYLDVILGTTDAMVTT

QIAMDYGYTAVKFNKLLHALGIQHKVNGQWILYKAYMGKKYTTTKLHTYTDKHGKDHA
KPLTAWTQKGRRLIYDILKENNVLPLIEREDIA"
CDS complement(4352..4657)
/locus_tag="OCPHGMPK_00928"
/inference="ab initio prediction:Prodigal:2.6"
/codon_start=1
/transl_table=11
/product="hypothetical protein"

/translation="MQNRLKKLRLEKRLTLADVQVKTDIDFKTLENFEKGLENGIPNS

LAIWQKLANFLEVPIEYLMGLNDDSKTLTVNDLNPAKEDAYERITDMLCEDEDDEDE"
CDS complement(4663..5550)
/locus_tag="OCPHGMPK_00929"
/inference="ab initio prediction:Prodigal:2.6"
/codon_start=1
/transl_table=11
/product="hypothetical protein"

/translation="MAQRRMFSQRIVESARFLKMPPSTQCLYFHLGIHADDDGVVEGY

NVMKQTGSTEDDLKILAAKGLITVLNADLVTYINDWKENNRIRPDRKVDSIYRDLLIR

MLPDIELQQSKARADTGKKTGIKAIEGNGRPVDNQWTSNGPHRLGKDRLGKDSINNIV

DSSESPTQIYESEFEQLWSMYPKKQGKKAALRHYKTWRKRSKDNTFEAMKEKLERYLK

FLKIKQTPLEYTLNGSTWFNGRFDDELDMTPVKPRYNQQAKPVRRATNWDKVQQQQSQ
TVPQMTQEERNAIFREYGR"
CDS complement(5563..6354)
/locus_tag="OCPHGMPK_00930"
/inference="ab initio prediction:Prodigal:2.6"
/codon_start=1
/transl_table=11
/product="hypothetical protein"

/translation="MEIYGKEEDRAKWALHYAQVKANINQPKRTHKVTVSGKNKQGKP

YSYDYKYADLADIDKAVMDGIKKATDKEGNVTFSYFFDIQTTNTAVAVQTLLVDSSGF

TVKTNKIIFQNGKAYDAQATASLISYAKRYSLSGAFGIAADDDNDVQDQKTIYEPKIL

TKQELENYQVYYNGVQANLYDLYQEARDGIKDAQDWFKESHTPEDAQAIHQVAQIFKK
KHKKTDTSKTKEKQAVLDKIQQAKKDPFEDKKVVSDADSTVDRLF"
CDS complement(6356..7231)
/locus_tag="OCPHGMPK_00931"
/inference="ab initio prediction:Prodigal:2.6"
/codon_start=1
/transl_table=11
/product="hypothetical protein"

/translation="MTKELIKFENEDFPISYTKAEIDFQGYPALDAKVDAMTKDWDKY

```
VVTAESYPYDKKTRAELNRIRRALSDRRKVIVKESSQQITEFNTKVKGLDLKIKGVVD

HLSEGLKTFDEQARKDKHQQNLLRLGELAKEYGVALQELDYQDKWDNKTTSWTTIEED

ARQQFEAIVEKQKARKEAEQVIANKANEYTKPAMSANPYLQMLDYKSLPDVLTQMDND

HDYLIKQAKQQEENRKKAIEALEQHGDKYVDAKTGEVVDKVHSVTLKLTGTKEQLTAL
                     SNFIRDWGISYERVD"
     CDS             complement(7430..7585)
                     /locus_tag="OCPHGMPK_00932"
                     /inference="ab initio prediction:Prodigal:2.6"
                     /codon_start=1
                     /transl_table=11
                     /product="hypothetical protein"

/translation="MSFTKWINQNINKFLGTSFTERETEIMTIGATFTVIAVAYLLMY
                     NVVFPNI"
     CDS             complement(7582..7833)
                     /locus_tag="OCPHGMPK_00933"
                     /inference="ab initio prediction:Prodigal:2.6"
                     /codon_start=1
                     /transl_table=11
                     /product="hypothetical protein"

/translation="MSSKETWLTVKELCKKYDYSNAAYTKRRKKCLLSPFKDAIVYDG
                     QHTMIIEERWQAFLKDQSRKHWEEVFGTGVMRDRKVLER"
     CDS             complement(7830..8186)
                     /locus_tag="OCPHGMPK_00934"
                     /inference="ab initio prediction:Prodigal:2.6"
                     /codon_start=1
                     /transl_table=11
                     /product="hypothetical protein"

/translation="MQPLDISYLYLSDIGKSKHEIITRLQQLSLIDGNYQATGKGVDT

GWLGIKTTSVWNPATESMINDQATVLTALGIIYIPVLFEDKFYKDMSASQNLVERACD
                     GELLTIKELRKQGYLK"
     CDS             complement(8208..8426)
                     /locus_tag="OCPHGMPK_00935"
                     /inference="ab initio prediction:Prodigal:2.6"
                     /codon_start=1
                     /transl_table=11
                     /product="hypothetical protein"

/translation="MRQVEHSNLRKMELKTNEDNQPMNNWSRVIIETDEVNPKVLAIV
                     TNEEYELAKGLRIRLKSIPKELIFKRLP"
     CDS             complement(8439..9221)
                     /locus_tag="OCPHGMPK_00936"
                     /inference="ab initio prediction:Prodigal:2.6"
                     /codon_start=1
                     /transl_table=11
                     /product="hypothetical protein"
```

/translation="MNNLIKIQVKDDQQLVSARSLYKGLELKSRFNEWTSQNFKEFEN

GSDYTYAVTTAHVGNGATREITDYLLTIDMAKELCMMSKTSKGKEVRKYFIQVEKNWN

NPDMIMQRALNIASSRVKLLENKNKELEDVNAKQAEKIAKDADDVVFAKAIRYSHHAI

TIAELADILTQNGFVIGRNQLFQLLRQAKYLSHQRSTWNLPMTDKVKRGYFRITHRLT
RDDRPYSQVFVTPKGQRHIINKALRGKFDDIYQEVMVTTFDV"
CDS complement(9233..9445)
/locus_tag="OCPHGMPK_00937"
/inference="ab initio prediction:Prodigal:2.6"
/codon_start=1
/transl_table=11
/product="hypothetical protein"

/translation="MKFDLKQARKYSGFSQAKMADMLGIGLNTYRDYENGNTYMRVDT
AQKFARLVNIPMNNIIFYHETTDKIH"
CDS 9599..10219
/gene="lexA_1"
/locus_tag="OCPHGMPK_00938"
/EC_number="3.4.21.88"
/inference="ab initio prediction:Prodigal:2.6"
/inference="similar to AA sequence:UniProtKB:P9WHR7"
/codon_start=1
/transl_table=11
/product="LexA repressor"

/translation="MDNILGDNIKKYRKASSYTQKDLANLLKVKPTTVASWEQGRNKP

LMDKVTKLTNIFGVSLTDLVGSDQENEVTDYFSKKPRMVPLLGTIAMGAPITAEQNIE

KYIPEYMMDRYADDTLFALRCQGDSMYPLIPNGAIAIIRQQTDVENGEIAAVLINGEA
TLKKVLHVGKAVVLRPANPEYRDIILDKDHPGTILGKMIKYEMNFD"
CDS 10230..10826
/locus_tag="OCPHGMPK_00939"
/inference="ab initio prediction:Prodigal:2.6"
/codon_start=1
/transl_table=11
/product="hypothetical protein"

/translation="MNQRINPETGYPMSTDKQIKIKKEALSVTKAFHEENPHEAGIKL

LKELGLPDPRDERTILSDKIGQYIIGILGAEAELHYKMGLWNEAENEYLQIFYLSIYY

TDALRILYSKEHRYRDAVYILEFTMQTIQEFSQLFYKKDYDTLSLALEKAKNIANKKA
RIDTSRLSSEQKVVLTQTAGKNFKKIKNWMNEISEKKN"
CDS 10841..11572
/locus_tag="OCPHGMPK_00940"
/inference="ab initio prediction:Prodigal:2.6"
/codon_start=1
/transl_table=11
/product="hypothetical protein"

```
/translation="MGLFDSLSKKMEERSEKKKQEDAEYQEILNTFKNDGAKEFENYY

FDLKTNKILEARGVFKRSYRVINFEDVLSYSINKKEHNDSKTQTKRKHGIGRAVVGGV

LLGPVGAAVGGLTSKKETQTISKDFVDHLGVVVNLIDGTSFEIEYLDSTLKADDSLVT

GCIKKVNELATVLEAGIENAKRKQTETVATEPAKLEENNAQSAQPSSPASDPLDEIKK
                     LKGLLDIGAITQEEFDIKKKQLLDL"
     CDS             11586..12590
                     /locus_tag="OCPHGMPK_00941"
                     /inference="ab initio prediction:Prodigal:2.6"
                     /codon_start=1
                     /transl_table=11
                     /product="hypothetical protein"

/translation="MKQFQWLDDGLHKMFLDYLHRIYTERLSFYGIQNSDLNRFSWSE

NKKVLICGIKVYADIATQGEPSGFLVFAVPTNTLNKVLFLNLFNETKNPSFSRHYNEQ

EMLNWIIDSGLISKSTAKNIVPGSLKMIFSVFDDIKINYKDEPNFVSNLTEDWIRSWV

DKEYNSTLAAESTPTLVNYFPSTFKRYFIDKYHFEDMLKEINNDQFTDEFNQCLFAYE

HEKWFLCASGLGSCLEHLMYIILNNYAKKGYNILNRFPKDPTAKDYVNRFRQKPIGID

SRQARAINLFFMARNSVDHYSSGKTQRIFCDLLLDGISDVYNDYFGASMNAPLAPTQK
                     "
     CDS             complement(12593..12799)
                     /locus_tag="OCPHGMPK_00942"
                     /inference="ab initio prediction:Prodigal:2.6"
                     /codon_start=1
                     /transl_table=11
                     /product="hypothetical protein"

/translation="MSLTSNERKLLIKILEDYGYEHELWDLPQDNNGKYYSDNPDSPN
                     FEVPYDEPYMAQEANLIQSIINKI"
     CDS             13003..14217
                     /gene="xerD_2"
                     /locus_tag="OCPHGMPK_00943"
                     /inference="ab initio prediction:Prodigal:2.6"
                     /inference="similar to AA sequence:UniProtKB:P0A8P8"
                     /codon_start=1
                     /transl_table=11
                     /product="Tyrosine recombinase XerD"

/translation="MKDQKEYAVTMRTISKGKYQFRQKYPDPLLSTPDKIVLKVATVV

LTKKTSQAWQQARTILKNKIDAKMSVSRISSITLEQLKEHYFEYLDKQDKINKDSKYQ

SHYIYKSHINIFLREKDVDPSTIIENLNTPYFKRYFDKMLETKSWSYTNVRRAALWNM

FDFGLGYGYLNFNPLQNFRLRKLETPQSELDLNIEDKYLTDDEYHRLLAEFKKRGRND
```

```
YVDFIQFLYYMGLRVGEGGSLKTKDIVKVNDKYYANINGTLIKKHKGNSKRGEFKKKP

GAKSDSSNRQVYLPPEAVKIYKRHDLGRKFLFTKKTTKNAVEANSVNRYLKRIAPYAG

IDPDKLTSHIFRHTHVSKLAEQGVPLEMIKKRVGHSDSKITEEIYYHITGKARTDFEH
                     QIDAFSKNSKFE"
     CDS             14934..16004
                     /gene="xerS"
                     /locus_tag="OCPHGMPK_00944"
                     /inference="ab initio prediction:Prodigal:2.6"
                     /inference="similar to AA sequence:UniProtKB:Q7ZAK7"
                     /codon_start=1
                     /transl_table=11
                     /product="Tyrosine recombinase XerS"

/translation="METNKYLSLIKQELANMPDFVKEYNLGTTHSLTTTYQYLTEIRR

FFDWLRQNKLSTASSNKEIELVTLEDMQRNDVMLYIHHLKHTKNQQGRLNSPTTINRS

INALRSLYKFLTITSDNNHGEPYFDRNVMLKINSLNDTKTLNYRAHVLESHMYMGDLK

YQFLDFIENEYEHKCNKQSLPSFKKNHERDIAIIALILGTGIRVSECVSVNVRNINLK

DEMLDVIRKGGQKDSVPIADWTIPYLKKYQEIRADRYHADRDDIAFFLTRWHGKTRRM

TANAVEKMVNKYSAAFGKPLTPHKLRHTLASELYEVTKDQVLVAQQLGQKGTTATDLY
                     THVDQKKQKAALNKINRHKNQD"
     CDS             16073..16963
                     /gene="benM"
                     /locus_tag="OCPHGMPK_00945"
                     /inference="ab initio prediction:Prodigal:2.6"
                     /inference="similar to AA sequence:UniProtKB:O68014"
                     /codon_start=1
                     /transl_table=11
                     /product="HTH-type transcriptional regulator BenM"

/translation="MELRVLRYFLAVCEEKNISKAANSLHISQPSLSRQLKNLEEELG

VTLFYRGHQEITLTQEGYYLQEHAEEIISLANKTQQNLKHSKIISGELYIGAGESIAI

KRVMDIVNNIVKNYPQVKIHVFNGNSSMIEGKIERGILDFGITMGQYDTTQNFNSLTL

PENNEYGIIVSKDHPLAKQPYIVPEDLQKYPLIISKHTADSDNFRDWCTDPQKLNIVA

TFNLPDNLQYLVEKGLYCLLIYKDLLSLSPESNLCFIPLQPKIIDHNRLIWNSRVQLS
                     NVARLFLKLLRNSINNEKLV"
     CDS             complement(17026..18117)
                     /locus_tag="OCPHGMPK_00946"
                     /inference="ab initio prediction:Prodigal:2.6"
                     /codon_start=1
                     /transl_table=11
                     /product="hypothetical protein"
```

/translation="MKNKFLKILGLVAGVGFTLVIVFQVILAMNTSAIKNSTPATVNS

ATSNSSDKEIIAEALNTDFKSYKFKVKNSPGIEATRTWSGKYQNDPLKAQAKVDKTSM

WLNEDKVYQKMWDNTGSPKWKASSTMTPNKVVSQFDPAIILSGSKAIKNDMKLKKSGS

VYRVSYSGSSLRFWNKTHKKLIKKLKGNASGLDNEGALKRLKVEYTLKEKSGHLVLTS

FSFYLRFAYAGYNISQEGTYSEINAIKVSTPNALKSKSSIKKSHRVYHPKADDSFDIN

IDSENSHSSQSSQSSSSSSSQQSSSSQSSKSEEHNDAKQDSQNQSNATSQSADNKESN
QQSQNNNTNTNNQQGQNQNNQQPASGQSH"

```
     CDS             complement(18202..19089)
                     /locus_tag="OCPHGMPK_00947"
                     /inference="ab initio prediction:Prodigal:2.6"
                     /inference="similar to AA sequence:UniProtKB:P0A0N1"
                     /codon_start=1
                     /transl_table=11
                     /product="DegV domain-containing protein"
```

/translation="MKIALITDSTSDISPEEAKANDITVVPIPVIIGDKQYMDGIDIT

AEKLFELERDGAPFPKTSQPSPGTMLELCQKLKDEGYEAIIAIPLTSGISGFYNSLVN

LAHNHPEFNLYPYDPAMTVRSMGNLVLAAAKMIKNGWEPKEILAKLDEIRDTIDVLFV

VDDLNNLVRGGRLSNASAFIGTMLQIKPLLTFKKDDYQIVSFDKVRSMKRAVKKAENI

TIERINNSPIKDKIALTVYNSNDLEQATKVKDDFQAAFPNMTIKMFNFSSVIATYLGE
KSLGITWMADIDKMDFSKK"

```
     CDS             complement(19205..19615)
                     /locus_tag="OCPHGMPK_00948"
                     /inference="ab initio prediction:Prodigal:2.6"
                     /codon_start=1
                     /transl_table=11
                     /product="hypothetical protein"
```

/translation="MDVLIFNSVSESIKRAINKKCGLNLSQTRLLLFFDRHQNETLTM

GELAQALSISLSTLSRQIKQKKTAALIQVERSKKDSSKSLNLNAEGLAKAQELKDVLR
QIEAQVFSTWSQEKIQDFDSKMQIVVNNLMADEL"

```
     CDS             19804..21495
                     /locus_tag="OCPHGMPK_00949"
                     /inference="ab initio prediction:Prodigal:2.6"
                     /codon_start=1
                     /transl_table=11
                     /product="hypothetical protein"
```

/translation="MAFDGLFIHSLLQNLSPALIGGKLTKIYQPFEQDLVLNFRKDRT

NQRLLISANAQNPRFYITTDTITNPDVAPTFVMVLRKYLEGSILKKIEQVGVERIVNF

YFSNRNELGDEMQLILSVELMGRHSNVILYNADDNKIIDLLKRINPDENRARLLLPHA

SYELPPLLPGLNGFHLSLDHFNDLKKKYPEPIDFVKQFNGLDGDDKKEFTGYLEDDFS

YSSLQTFFNQFNRPKGYVLQTIKHKDRVYTYLPYHLELNLIYSNDDINETLDNFYREQ

ANREWVKQKSKKVENIVNNELKKLKKKIIKLRKQLDQAENSEGYRIKGELLNAYLHEV

KPGMESIDLPNYYENNQPLKIKLDPALSPARNAQKYFTRYQKLRNSIKHVNEQIKVAN

ENLDYFDSIQTAIDNADPQDIDAIRDELINQGYLKEQTHNRRRKKKISEKNLNTFKLS

DGKKVLVGKNNYQNDWLTLKKADKRDYWFHVKNMPGSHVILQDSSPSDEDIKEAAEIA

AYFSKGKTSGHVPVDYVQVKRIKKPNGAKPGFVIYTGQNSIEVTPDEHDFLSKKI"

```
     CDS             complement(21581..24745)
                     /gene="carB_1"
                     /locus_tag="OCPHGMPK_00950"
                     /EC_number="6.3.5.5"
                     /inference="ab initio prediction:Prodigal:2.6"
                     /inference="similar to AA sequence:UniProtKB:P63740"
                     /codon_start=1
                     /transl_table=11
                     /product="Carbamoyl-phosphate synthase large chain"
```

/translation="MPLHNEINKVLVIGAGPSIVGEVTELDILAKQALAAFEESSIQV

VLINPNPATVTTDPRPDVNVYLEPMTLPFVKRIIRMEKPDAIIPAFGGKPALRLTKEL

LESGILAQMNIELLTINSLALSLSTPHNFYTFLKANKIAVANQWILEKEEDLRRIVEH

AHFPLLLSKKQHYRPDNMISLSNLTQLEQYFLDEENDDHFNWQDYRLSEDLSNWEELI

FDIVRDNNGNFCFVGNTGSLEPVGINSSDSLLVTPILTRNNNQIQRLRNCCRKIANCL

NLHGALSIHFAVKQSGESFNYKVLSIKPRLTQTSLLSYRSGVYSIGYVTAKIALGYNL

NEITDPQSGISAAVDPVQDACFVKLPYWSFTEAGYNHYALNNKTTSGGQALGIGRNFE

SAFLKALQSTTGFSNNVTTFNKECAKSEEQLLQDLSHPNEMHLVTLLAALAKGISYHK

LHKLLHVHLVFLQKFNHILILLKKLQADELTPDLLLKVKKNGFSNRLIAEIIGREEKA

IADLCTKWSIKPSYIEIDGTAGLIHPNIKAVYSSYGIEDEVKPLANSKKCLLLGLKPF

QVSLTGEFDYMLYHAAKTLKEQGISPVIISNNPESVSAAYDVCDRVYFEPITLENILE

VAWKENITEVVTQFSGKQINQYRIQLLEHGLTILGQPNLKEILKEDHAEEVYQAGVNP

VPALQCDNEKNIFAFVKDNGFPALIGGTSNGKKQKSAVVFDLPALQRYIAENSLEHIT

VSKFIEGKKYEVTAISDGKNVTIPGIIEHFEQTGSHASDSIAVYRPQNLSTNDQRRLR

DSAISIATKLHLRGPVNLHFLLAHDQIYLLQMKSYSGHNVAFLTKALKRDITAITMKV

LLGSKLSELELPSDIWPSNNLIHVKMPVFSYLSYQGENTFDSKMKTSGSVIGRAKHLP

TALFKGYEGSDLMISTYGTVFISVKDSEKQNAIKIAARFHQLGFKLLATEGTANVLAE

EGITTGIIGKVQEGSNSLLEKIKQHRINLVINVTSLSDSASHDAIMIKDAALSTHIPV
FSSLQSAQDVLTVLETLAMTTQPL"
CDS complement(24749..25804)
/gene="carA_1"
/locus_tag="OCPHGMPK_00951"
/EC_number="6.3.5.5"
/inference="ab initio prediction:Prodigal:2.6"
/inference="similar to AA sequence:UniProtKB:P99147"
/codon_start=1
/transl_table=11
/product="Carbamoyl-phosphate synthase small chain"

/translation="MKRYLILEDGTAFSGHGFGAPITATGELAIQTSNFGYQEAISDP

ANFGKILAFTTPMIGGSGINAIDYESIDPSVRGIIANDIAFHISANPTFQDLNDFLKE

KRIPAIYGVDTRALVQKLKNKQVIKASIMDTNDAHAFDQIKALVLPKNKTAQISTTHP

YAAPNVGKTVAVIDLGLKHSLLRSLSLRKINSVVLPYNASIYDIINLRADAIVISNGP

GRVEEVAPFLKPVFDKFYGKLPILGIGLGFLAISNFLNLELLDLIPAYNGSNFPVIEQ

TNNRIWQTAMNIDQLVVPDSLSLNLSRKYFDLKSDLLAGFSIKDDKVLATAFNPEGSP
GNFDAASIYDQFLNMME"
CDS complement(25808..26722)
/gene="rluD_1"
/locus_tag="OCPHGMPK_00952"
/EC_number="5.4.99.23"
/inference="ab initio prediction:Prodigal:2.6"
/inference="similar to AA sequence:UniProtKB:P33643"
/codon_start=1
/transl_table=11
/product="Ribosomal large subunit pseudouridine
synthase
D"

/translation="MTENYQLVIEKEKGRLDKVIAEKIKDLSRTKIKELVNDKNILVN

NKPEKVSYKVQAGDLIDITVPPAKPLSLEAQNLNLDIVYEDADVIVVNKPQGMVVHPS

AGHPDHTLVNGLLYHTHELADSPEGFRPGIVHRIDKDTSGLLMVAKNDKARESLEKQL

AEKINKREYLAIVHGNFETKNGVIDAPIGRNPNNRKQMAVNPKGKAAVTHFTVLEQFK

DYSLVKCVLETGRTHQIRVHMKYIGHPLAGDLLYGPKKTLTGHGQFLHAETLGFYQPS
THKWLEFSAPLPAIFEKQLENLRRETKH"
CDS complement(26737..27201)
/gene="lspA"
/locus_tag="OCPHGMPK_00953"

/EC_number="3.4.23.36"
/inference="ab initio prediction:Prodigal:2.6"
/inference="similar to AA sequence:UniProtKB:Q9HVM5"
/codon_start=1
/transl_table=11
/product="Lipoprotein signal peptidase"

/translation="MKRAKQVLYLVISLLVVVADQWLKNYIVTNFKIGDEKTIIPGIL

SFTYLQNDGAAWNIFSGQMILFYLISIAAIVVVVYYLFNPKYKNWLFDTGLALVLGGI

VGNFIDRLHLKYVIDMLQLDFVQFNIFNIADSAITVGIVLVFIYLIFISEKD"
CDS complement(27202..28881)
/gene="fhs1"
/locus_tag="OCPHGMPK_00954"
/EC_number="6.3.4.3"
/inference="ab initio prediction:Prodigal:2.6"
/inference="similar to AA sequence:UniProtKB:Q5XC12"
/codon_start=1
/transl_table=11
/product="Formate--tetrahydrofolate ligase 1"

/translation="MNLKSDIEIAQNTKELPITEIAQKIDLQPEEIELYGNDKAKISW

KGINRIKKSQKLGKLILVTSISPTPAGEGKSTITIGLGDAISNQLHKNTLIALREPSM

GPVFGLKGGATGGGYAQIIPMEDINLHFTGDMHALTSAIDTLAALVDNYIYQDNSLEL

DPNRILLKRGIDVNDRTLRKITIGQGSRFNGIEHEASFAITVANELMAILCLATDIND

LKKRIGNILVGFSVKDEPVYVKDLGFEGAIAALLSTALKPNLVQTLEHTPAIVHGGPF

ANIAHGANSVIATNTALHLSDYVLTEAGFGADLGGQKFMDFVSNHLDKRPDAVVVVAT

VRALKYQAEESTDHLDKENIPALEKGFANLKRHMENMAHYGVPVMVLINKFASDTDQE

LSKLKELVQNDGFDCEVVSYHDEGSRGGIKAAEKVVELTGKASDFTPVYEPEDSVEDK

IAKIAHKIYHAKDIEYSDKAKKQLAEIKKMGKDQLPVIIAKTQYSFTDKKKILGAPED
FTLHVKDLALKNGAGFIVVATGSILDMPGLPKHPAALDIDVDNNGKISGLF"
CDS complement(28878..29222)
/locus_tag="OCPHGMPK_00955"
/inference="ab initio prediction:Prodigal:2.6"
/codon_start=1
/transl_table=11
/product="hypothetical protein"

/translation="MKKHFIFLGAAGVIYYSWLLALIFVAFIVGYESNKSISYPAIAL

GTIFAIIVIYTWLNSYFQKEASVYWLKLPYRQEIQLQDLKIKWQWKAFDIYEARGKLN
TYLLLALKRKKK"
CDS 29355..30278
/locus_tag="OCPHGMPK_00956"
/inference="ab initio prediction:Prodigal:2.6"

/inference="similar to AA sequence:UniProtKB:P0A0N1"
/codon_start=1
/transl_table=11
/product="DegV domain-containing protein"

/translation="MKIGVLTDSSSYLTKEQCEKYGIDVLPIPIIWDNKEYLDLIDIG

FHEFYKKLNSSTTLPTTSQPSIGTVKKYVEKYINEGYTDLIIITLSSGISSFYSNVLS

VANEETRINIHPFDCKITCAGEADAAILAGRLVKAEADINLIMHDLKDLRNTTDVRFM

VDNLNHLKRTGRLSNAASFVGSILKIKPILSMDVQNEGKISAIAKERQYKRAYNHIKK

DFSRLTSNMPYPIQLTIFHADDEEREAAWINDYQTSFPKIKVDQSIIGPVVGVHVGQH
TMAMIWCRDLDSYFDKNGKPLLNINSQVVED"
CDS complement(30337..31569)
/gene="cpdA_2"
/locus_tag="OCPHGMPK_00957"
/EC_number="3.1.4.53"
/inference="ab initio prediction:Prodigal:2.6"
/inference="protein motif:HAMAP:MF_00905"
/codon_start=1
/transl_table=11
/product="3',5'-cyclic adenosine monophosphate
phosphodiesterase CpdA"

/translation="MLTQDKKTSFWVISDTHLIADSLHDDGQAFSQMQKTSQGKDLYY

QETALSAFVRMAEEKKPAAIIVTGDVTFNGERVSAERFAEIFKPLTKTKLLVLPGNHD

IYDGWAREFHGKKQYYAGQISPRMWRNIFRTSYETAVSVDNSSLAYSVQLNPDYLLIL

ADSNDYGKEESSTAPATAGFLGKEQRKWIKEQLQYASQNNLRVIFCMHHNLYAHNPAV

NKGYVVDDYRELRKLLAQYNVKLVFSGHIHAQNIMSPQNSCPATEVVTACFCSNDQGY

GVVKVSPKEISYTCHHFKMKDYLTDKEKQNWTLTHFHDYLENIQLGTISAELMQEDLY

RNHDDLKSVRQMGRLFGEMNYYFFTGKNHIDFDELQKLKKSEIYQRLISENPQYELYL
QTLYDTSVHDNLHVKIKY"
CDS complement(31572..32696)
/gene="rlmL"
/locus_tag="OCPHGMPK_00958"
/EC_number="2.1.1.173"
/inference="ab initio prediction:Prodigal:2.6"
/inference="similar to AA sequence:UniProtKB:Q9K0V4"
/codon_start=1
/transl_table=11
/product="Ribosomal RNA large subunit
methyltransferase L"

/translation="MKEYTLYATMGAGFESVVAKELNNLGYETSTENGRVFFKGTQKD

VVKTNLWLRSADRIKILLKEFKAFSFDQLYDEVYSYDWAELLPVDAQFPVKGRSVRSK

```
LHSEPDVQSIVKKAIVDKLTDQYRRRGFLPESGAEYPLDIHIYKDVARLSLDTTGQSL

FKRGYRVEHGGAPLKENFAAGLIELTPFDGSHPFIDPMTGSGTIAIEAALKAKNIAPG

IWREFAFDNFDWFDKKIHPELVEEAKTKEKQEHAPILASDIDQSILEIAKVNAHNAGV

LQDIKFKQVAVKDFATDLENGVIIANPPYGKRLKDREAAEKIYEEMGQTLRPLSSFSQ
                     YYLTSDPQFEKFFGARATKKRKFYNGNLRTDYYQYWANKR"
     CDS             complement(33161..33598)
                     /gene="gpsB"
                     /locus_tag="OCPHGMPK_00959"
                     /inference="ab initio prediction:Prodigal:2.6"
                     /inference="similar to AA sequence:UniProtKB:Q8Y614"
                     /codon_start=1
                     /transl_table=11
                     /product="Cell cycle protein GpsB"

/translation="MASLNDIQLNPQSILKKQFRTKMKGYDSDEVDSYLDTIISDYST

FATIIEDLQAQISELKAQQKQAPTQSDAPKLDFKNEEVEDDVKTYTPHNIQKPIVNSA
                     NEEKEAPELTTNVAMIQRISTLERKVYNLEQRMNQQDRTYQAN"
     CDS             complement(33812..34381)
                     /locus_tag="OCPHGMPK_00960"
                     /inference="ab initio prediction:Prodigal:2.6"
                     /codon_start=1
                     /transl_table=11
                     /product="hypothetical protein"

/translation="MKRLWVTGYRSYELSIFSDKDPKLTVIKYTLTNYFKSLLEEGKI

DWIISGANLGIEQWALEAAIQLQNEYSIHTALMTPYLEFSKRWNENNQMKYQNLTEQV

DFTAATSNYPYMSPAQLKNYQSFMLGHTDRAVLIYDPEHPGKPKYDYEAIQKYQEGHD
                     YPVDIIDFYDLQESAEEYEENHRQENNFY"
     CDS             34450..35079
                     /gene="recU"
                     /locus_tag="OCPHGMPK_00961"
                     /EC_number="3.1.22.-"
                     /inference="ab initio prediction:Prodigal:2.6"
                     /inference="similar to AA sequence:UniProtKB:Q5KXY4"
                     /codon_start=1
                     /transl_table=11
                     /product="Holliday junction resolvase RecU"

/translation="MVKYPTGSLTHYTKDQTDLLHPRKQKHRKDVVFSDRGMNLEQQI

NESNKYYLLEDIAVVHKKPTPVQIVKVDYPKRSRAVIKEAYFRQASTTDYNGVYQGRY

LDFEAKETRNKRSFPLKNFHEHQIIHLERCLKQKGICFTIIRFVTLNRYFVTPASFVI
                     KAWKTPHKSSMTLQELIDNSIEIKSGFRPTLPYLDAIDNFIDRNKKNGR"
     CDS             35069..37447
                     /gene="ponA"
                     /locus_tag="OCPHGMPK_00962"
```

/inference="ab initio prediction:Prodigal:2.6"
/inference="similar to AA sequence:UniProtKB:Q04707"
/codon_start=1
/transl_table=11
/product="Penicillin-binding protein 1A"

/translation="MADNNQKPTNSRMANLHPEGKPRKRIWLQIIKWFFIVAMLIIVS

GVGLFAYYAKDAPSISQAQLQSGGTSSLYTRDGKFLLSLGSEKRTYVNNDEIPKELKN

AIVSVEDRRFYKEGIGLDPIRIVGSVLVNAKSKGVAAGGSTITQQLVKLSVFSTAASQ

RTLRRKAQEAWLSMKVEREFSKEQILEFYINKVYMNYGNYGMGTAADFYYGKDLKDLD

LAQTALLAGMPNAPVTYDPYVYPEKAKYRRDIVLRAMLKNDKITKSQYTQAVNEPITQ

GLQPKKNNATSELRKVDDPYIKEVISEVKSKGFNPYNDNLKITVNIDQDAQQKLYDLV

NNGSVPFTNDKMQVGATIIDPRTGHVIAIIGGRKLPSVQLGLNRAVQTGRSTGSTVKP

VLDYGPAIEYLNWPTSHILDDSKYVYPGTNIQLYDWDNKYEGKMTMRHALEQSRNVPA

VKTLSKVGVARASLFARKLGVNVPSDSGLSVAIGANASSLQMAGAFGAFANNGVYHKP

QFVSKIETPDGLTRNYDSSGTRVMKESTAYIMTDMLKGVLTKGSGTQARIKNLYEAGK

TGTVKYSDEELVKYPQYKNSPKDSWFVGYTKLYSMGIWTGYDNLKDGTPSGVGSSSAQ

LLYKQMMSYLMTDKANKDWTKPSTVVRRRIANGTQDKVVSPTASNSSWQLFVKGHAPK

NPYSEVTVDKRKDEDDDVDTRFDTEDDTSSKGQSHSQSSSSSSHAEQQSSSQKQSSTS

NNNNNSNNQNQNNNQQTENKSDNQNNNSQQNTNNQNNQNQQQHQGNNSNNNH"
CDS complement(37533..38162)
/gene="nth_2"
/locus_tag="OCPHGMPK_00963"
/EC_number="4.2.99.18"
/inference="ab initio prediction:Prodigal:2.6"
/inference="similar to AA sequence:UniProtKB:P0AB83"
/codon_start=1
/transl_table=11
/product="Endonuclease III"

/translation="MEKLLSDEEARLVLKRILSLYPDAKGELHWDTKFHLLCAVLMSA

QTTDKMVNKTTPKFFNDYPDSASLAQANIRDIEAHIRTIGLYRTKAKHLKETAQIITD

KFNGEIPQDKKTLMTLPGVGEKTANVVLAEGFKIPAIAVDTHVSRISKRFKIVGEKAT
PHEVEQRLEELLPKEEWIRTHHAMILFGRYTMPARTKDQDPYSFLPPLK"
CDS complement(38171..38815)
/gene="dnaD"
/locus_tag="OCPHGMPK_00964"
/inference="ab initio prediction:Prodigal:2.6"
/inference="similar to AA sequence:UniProtKB:P39787"

/codon_start=1
/transl_table=11
/product="DNA replication protein DnaD"

/translation="MASFAAIQNEGFTVISNSLLRYYPSLKISEIEAMLLLQLESFKQ

MNNFFPSNNDLSERMNLSPVEVSQLIQDLIDKELLELGQKRDSEGRITNFYDLKPLYQ

KLDTIIDERETNANDQVRAGELIEERQSVNPLQELVRQFEIEFGRLLSPIEKQEIAAW

LNIDHYNPEIVKLALREAILAQVYNFKYVDRILLNWQRHGLTTTDQIKNFLQRN"
CDS complement(38883..40181)
/gene="asnS"
/locus_tag="OCPHGMPK_00965"
/EC_number="6.1.1.22"
/inference="ab initio prediction:Prodigal:2.6"
/inference="similar to AA sequence:UniProtKB:P67572"
/codon_start=1
/transl_table=11
/product="Asparagine--tRNA ligase"

/translation="MTELISIKDSSKHVDQEVKMHVWLTDKRSSGKIIFLQLRDGTAF

FQGVVRKNDVSEEVFEAAKSLRQEASFYITGTVHEDKRSHFGYEIQISDLKIVSNNEG

YPIGNKEHGVDFLLDNRHLWLRSKRPFAIMQIRNTMFKATVDFFEKEGFIKFDAPIFM

HSAPEGTTQLFHVEYFNNDAYLSQSGQLYGEAGAMAYGKIFTFGPTFRAEESKGRRHM

TEFWMMEPEMAWMHQDESLDIQERYLAYMVKQVLENNEYELRILGRDPEKLRPTTEGN

FTRLPYDDAIKMLQEAGRDIKWGDDFGAPDEGYISEQFDRPVFIVNYPTTIKPFYMKK

NPDNPKEYLCADVIAPEGYGEIFGGSEREGNYEILKKQIEEAGLNLEDYQWYLDLRKF
GGVPHSGFGMGFERTIAWICKLDHIREAIPFPRLINRMQP"
CDS complement(40250..40744)
/locus_tag="OCPHGMPK_00966"
/inference="ab initio prediction:Prodigal:2.6"
/codon_start=1
/transl_table=11
/product="hypothetical protein"

/translation="MNKYVKKGLKITGIVVGVIAVIYLCLCFVFYFATKRASDQVKQI

NEIALEKTPITTITKNYHLSRSVESNSLAGTSKKGKKYYFIYLPKSKKAYLYQANKGK

SEEQILKIFNEAHPGHDNLKCQLGWYKGMPVWEVTYNKKNGNYGYVLYTFKNGKELAY
ADNL"
CDS complement(40737..43550)
/gene="dinG"
/locus_tag="OCPHGMPK_00967"
/EC_number="3.6.4.12"
/inference="ab initio prediction:Prodigal:2.6"
/inference="similar to AA sequence:UniProtKB:Q2FYH5"

/codon_start=1
/transl_table=11
/product="putative ATP-dependent helicase DinG"

/translation="MKKAFTDDVFAVVDLETTGTQRNQGDHIIQFGCAIIKKRKVVKT

YSFLINPHREIPQAVENLTHISNRDVAQARDFKYYAPKIRKILENTIFVAHNVNFDLP

FLNYELVNAGLEPLTGKAIDTVELAQIAFPTFPSYKLRDLTARLKIKHLNPHRADSDA

LVTAKLLLKVIKKLENLPQATLNTLTSLSKGLLRDTDYIFYEIGQVARQTKRPLDKDL

IQVKHLILKKQISSTRQIGTASKGEFPQSDDEKKKLFKKNLRFRRGQVSLINRLHEFV

YSDTDNSLVVEAPNGSGKTFSYLMAYAYELYSGRKLVVATPTKVLQEQILKQEIPQLL

RVTHLDLDAQIVKSSSHYLDLDGFYNSLYQTDNPIQQTLILQMGILIWLTETETGDLD

ELQLTNYQAPLFAAIEHPGDARIGTAFAEYDFWNLARSRQEQADILITNHAYLANHYM

DSIWGQNPFLVVDEAHRFVENVASSRNDSLQFESFWGMCSHLRNLLFYAEDSAKVRFG

NNIEFKIILDKLEKNTNDLIHSINKVQQALYDNRNFATSREDRIQNAVSLGFQGQDLF

RSVPQFKRLLSQMQDNIEIVRQETNQLLFLLYHQQKNLLTSDDVLIRDLQEEIDHLDY

YSEQNYLLLDQLSEPQKLDHEGFVLEISNEEDPLSTNLTWLMLDPEEEVQQLYQYFDK

KLFISATLAQQDDFSYTIKNLYLDPEKTLTYRAKPSFKVEKHLKIYALSDNDAPEDPN

NPEYEEFISNLLTQIKNYNHILVLFTNLDVIRDVFSKLSENNNLKDYEILAQGLTGSN

EKIAKRFGIAEKAVLLGANSFWEGIDFKHNGVDLAIVTRLPFESPDQPEVKLRTECLK

KQVGANKIFEVDTLPRAILRFRQGCGRLIRNERDHGIFVILDQRVWNKSYGEQFLAGL
PVSAKKVSKQQLLNVLRNSKQNE"
CDS complement(43575..47189)
/gene="addA"
/locus_tag="OCPHGMPK_00968"
/EC_number="3.1.-.-"
/inference="ab initio prediction:Prodigal:2.6"
/inference="similar to AA sequence:UniProtKB:P23478"
/codon_start=1
/transl_table=11
/product="ATP-dependent helicase/nuclease subunit A"

/translation="MTKFTKEQDQAINDSGKDILVSASAGSGKTTVLVERVLNKILSG

TPVSSLLIITFTKAAAREMKERIKQKISDQLEVEPDNQFLRNQLLDIDTANISTIDSF

CLDVIRRFYYVIDLDPQFSVLTDETQGELLKERALREIEADYLEEDNQDFQDFYDNFS

GDRDAEGARNLLLQLYNTVTTQPNYENFLDELPTCYEVGDNLIQSNLWQQQIKPLLLK

EVSDLKAEVEALLAEPEINGSDLAKVKENYDIFSNRLDSFLESLNKDQPYNEIRASLM

NCKFEKAVRKSKKWSDESIEVYQKSQDLKSDLNDQLKKIFASFFVVEEKEQVAVLQKS

EKIVKTIVAAEKKLIQKFGQLKREQNLIDYSDMEQFAFSILTTDTSNAHIAQEYYQEK

FNEILIDEYQDVNALQENIIKAIKKKGQNTLFMVGDVKQSIYGFRQARPDLFLSKYHT

YGKDNDSEKIILADNFRSTKRVTKTVNELFNPVLTTNFGGIDYKKEGRLQFGASYYPA

DLPTASEYIFTDKKQTQSAYEDQYGDEMDFSEVQMVIARIKQLKAENFQVWDRRTQLK

RPLEYSDIAIITRTRSDNLQVMQEFAKADLPLFVTDAQNYFQTFELIMIMNYLRLIDN

PQQDIPLVAVMRSPLFNFKEPELAQLRVKTPAGNFYTALTNFASVNSALGKKCREFLK

QLETLRSFAATHRISELIWSIYEKTHLLEIVTGLPNGQQRRVNLESLYERATSYESAG

FKGLYQFISFIERMRKNQKDLAQPLLSDKADNAVKLMTIHASKGLEFPVVFVMGLGHQ

YQTRDLSGNFTISQKELGLTIKEKNYRIDSLVKSLADVQKRQQMLEEEARILYVGLTR

AQQKLILVASVNEIENKQKKWLSELDQKKDTIPLVKKINAQSPLDFLGPKLEQIHDFD

QTIQNMTSALEEQDKLYYLKFDLDLEPEKVKSQNENSQEVNSKVNKVVQELYDFKYPF

EDATKTTAYQSVSEIKKAFNDPIDKELENSRLISSSNRYLQPIDETPTFLEEQKFTGA

EIGTAMHLVLQYYNYEGNKDLENLDQEIDQLVELGKLNSLMVPYLSKEALNWFVMSDF

AKEFWKQPDKLHRESQFSSLVNASELFSDFSDPSAKILVHGTVDGYFEAKDGLVLFDY

KTDFVDKTNEEQAIEKIKQKYTGQLRLYEQALNEMNNDKKVIGKYLILLDARKVVPVD
"

```
     CDS             complement(47190..50666)
                     /gene="addB"
                     /locus_tag="OCPHGMPK_00969"
                     /EC_number="3.1.-.-"
                     /inference="ab initio prediction:Prodigal:2.6"
                     /inference="similar to AA sequence:UniProtKB:P23477"
                     /codon_start=1
                     /transl_table=11
                     /product="ATP-dependent helicase/deoxyribonuclease
subunit
                     B"
```

/translation="MINVITGRQVDNLQNDIIDQAVKSYYQDKTHDVFIIVPNHIKFT

TEVRALSKLSVLTNKKQVAVNKLHILSFSRLAWYFLKDEAIKLPQILDDAASVMLLEQ

IVKDHQNELKLFQNKNQITSGALKQMYEAILSVRAGNIELENIDNEKLNEETSYKVHD

LRIIYDDFIERLSEKFATKDEMQLLLNEFLAKSNNLSSMEFYFSDFSHFSLQELTSVR

LISKKAKNTTLAFKTKVGKIDSKAEPGDYDYVVQRTIGQLEHFWQNQQLNYQTTEFPL

SQTNPSSLLNGVWTKTSSFDESLSKFLQPVKADSRYAEAYFVARTIYQQVALNHYRYQ

DFLVLAPNLSEYETYLTPILRQNNIPFFNDLQKEMKYHPLVVAVENLQQIFKRGFQTD

NVIALMKTQLFIPEWYKSVARYQNDVDLLENFVLAHGIKGSLWNKPLKKFVDAEVIAL

DKSEQEVEELERLRQYFISILSKFFEEIEAEKDPQAGVTIFWNFLIKNRVSKRLEAWR

KEANDAGDLQLAQQPEQVWSTLNDLLKDYLLVAKEFSLEQFFDLLISGFSEANFSQIP

STLDAVNISEMGMVQGQGYKQVFIIGATSSNLPQIEKIPGFFSSENLEQLNDGNNTSG

YLEDQQKINNLDQNYQFGNALSLASDRIYISYPVINTANERLEPSIFYKQLLRLTQAE

EFSQHDLPNSAGDLLTFMTNPEASLGYLTYLKSKQAVKVDSILEMTEQKIGEVAQNVL

EGSSFKNIPENLSPELAQELYGDRIETSVSQLETYYQNSFEYFLNYGLHLKKRFENEL

DVIQAGNYYHETFDYLVKKIKEKNLNFADLTDSKLTELLIEVREELKEKGRYRQLLND

PFNKYLFHKLDQTTSNVAHYWHSNVNKTTFRPQYSELSFGKNQKVTGLSYSWKDENNK

KKIVDLRGKMDRVDLAKVNDRVLGEVIDYKSSAKKFDLGLFANGISMQMISYLEVLKK

NNKFFAQGKNLDVLGAFYQNITSSLERLSSDKMILSNYQIKDLLKESTKKLMYNGILI

ADEEILDLIEPGMEKDRANSEIYSSVKRKVNGDISWPRNQSFTPDQLELLLAYNSYLI

KNAGSEILSGKIKLDPYTYGQQSSLTYSDFKDIFFFDAMLKENNYHKIKAIDKKTLLN
LIKEKLDLDGDE"

```
     CDS             50753..51670
                     /gene="galK_1"
                     /locus_tag="OCPHGMPK_00970"
                     /EC_number="2.7.1.6"
                     /inference="ab initio prediction:Prodigal:2.6"
                     /inference="protein motif:HAMAP:MF_00246"
                     /codon_start=1
                     /transl_table=11
                     /product="Galactokinase"
```

/translation="MNKKVEIKAHGKVILIGEHSVVYGYNALALPIQALNISTTVEET

AGLTWMDTNHYHGAFFDAPDEYDGIKYIVKTMLAKVADAPNVKITYSGEIPIERGLGS

SAVVALGTTKALSQFLNLNLDHDEIMKITNHAEMINHGKASGLDAATVSSDYLVFFNK

QDGPQQLSQKLGATLLIMDTGELGNTKVAVQAVKKQMDESDLKKKQITRLGELATATQ

ENWLKQNAKEIGKIFNEAQSILASFDLSTKKIDNICKIANENGALGTKLSGGGLGGIV
IALCPDQETAQKIAKKAQVNFDNYWIEEI"

```
     CDS             51670..52635
```

/gene="thrB_1"
/locus_tag="OCPHGMPK_00971"
/EC_number="2.7.1.39"
/inference="ab initio prediction:Prodigal:2.6"
/inference="protein motif:HAMAP:MF_00384"
/codon_start=1
/transl_table=11
/product="Homoserine kinase"

/translation="MKKTARAHTNIALIKYWGKANQALKTPLMSSLSMTLDAFYTDTT

FEHDSSQTEDTFILNGQKQTPEESKRVFNYIHLLQEKFGFNDHFTIKSTNHVPTSAGL

ASSASAFAALATSFAASYGLDLSRRELSKLARLGSGSATRSIYGGFVEWQKGIDDASS

YAVPIDENPDLDLSLLALEVDTKQKKISSTKGMKLAQTSPFYQPWLARNKQEIAELKQ

AIKEKDFTKIGKLSELSANEMHACNLTANEPFTYFEPETIKAIKLVENLRQQGIECYY
TIDAGPNVKILCTLRNRKEIISAVQKSLTNVKIVVASFGPGVTLL"
CDS 52670..53743
/locus_tag="OCPHGMPK_00972"
/EC_number="2.7.4.2"
/inference="ab initio prediction:Prodigal:2.6"
/inference="similar to AA sequence:UniProtKB:Q9KWG3"
/codon_start=1
/transl_table=11
/product="Phosphomevalonate kinase"

/translation="MITEQAPGKLYIAGEYAVLEQNCPAILVAVNEFVRVSIAKSTGT

SGLIHSKQYSQDSIHWIRKGNQMVIDNRDNPFEYILSAIKFTERFCLEQKVPMSLYDL

HVNSDLDSADGKKYGLGSSAAVTVATVKAILNFYGLHCTKDLIFKLSAISHYSVQGNG

SAGDIAASVYGGWLAYQTFDKAWLKQELATKSLSDVLNEAWPGLKIQLLTPPEGLNLV

IGWSQKPASTSQLVDKTNAKKKFIKTQYDTFLEQSRQCVLDMIKGFNEKNIALIQKQI

RLNRQLLKDFAALNHIAIEIPRLTKLIKIAEQFNGAAKTSGAGNGDCGIVIADEQTDI
EEMKNIWRENGIMPLNFLVHSIA"
CDS 53768..54793
/gene="fni"
/locus_tag="OCPHGMPK_00973"
/EC_number="5.3.3.2"
/inference="ab initio prediction:Prodigal:2.6"
/inference="similar to AA sequence:UniProtKB:P50740"
/codon_start=1
/transl_table=11
/product="Isopentenyl-diphosphate delta-isomerase"

/translation="MSQRSQRKEEHLALAKMFFNSNKNNDFKHIHLIRPALPESAVSK

ESIATEMFGQKISAPFFINAMTGGSDASYTINRRLAQAAAKENIPMALGSASILEKEI

DQIKSFEIARQENPDGLLFANVNPTTKPKVAQKIVQVLNANALQIHLNSVQEAVMPEG

DRDFHWLDNLKAIRQAVDVPIIIKEVGMGIDPESLRTLLINDFSIIDLGGSGGTNFAQ

IENERRKNQKLMFLEDIGLSTVKTLLAARTIPVNKTIIAAGGITNALDIFKSLVLGAQ

YVGIANYFLQFANQDTESLIVAIQNLKYELRLLTALFGLKNIAEADEVKYYLDTDLYN
FTRQIYN"
CDS complement(54790..56463)
/gene="yheI_5"
/locus_tag="OCPHGMPK_00974"
/EC_number="3.6.3.-"
/inference="ab initio prediction:Prodigal:2.6"
/inference="similar to AA sequence:UniProtKB:O07550"
/codon_start=1
/transl_table=11
/product="putative multidrug resistance ABC
transporter
ATP-binding/permease protein YheI"

/translation="MNFRELIKINPVRFCLFVIITVLTPITLIGNTALVQLETTIVLS

RNLRSFIIISAISLVVAALGDVFMYISQYLITYQAQDLNNSVRDKIVKHYFYDGKKHQ

VSEMQNRLTNDLQMVNENYVGAIFNLLYGSVMIISIIIYLIMLNWILLLTICLIVAIT

LVLPKLIEKPLQKANQLISDSNQVYLDTLNDWLAGLEQLRQFAAGAKLFSVSKLASKK

LEDANVKQTTYTKLLNAITGIASAIFGLIIFLLSGFLVKNGLLEISALLIVGNFRFYL

NQAINQISNARGHMKGIKKLVTEIDKSAEPVHSSSKDSSIAPAIIKTKSLGLKFSNGE

SLTYPDLQIKEGEKILLTGDSGAGKSTLFKLILGELKPSSGKIIFEDKNGNQIVPDLR

KIGYLPQDPVVFPASILDNITMFNDKLDNKVAEAVKEVNLSADLKKFDEGIDKKLNLD

KLNISGGQRQKIVLARAKVHDSDIILIDEGTSAIDQKATMDILNNLLKSKATIVFIAH
NFNKKMHDLFDREIHLVKDWKKKAFVKNAKAFFDAFKKWLQNAVIKESN"
CDS complement(56463..58043)
/gene="msbA_2"
/locus_tag="OCPHGMPK_00975"
/EC_number="3.6.3.-"
/inference="ab initio prediction:Prodigal:2.6"
/inference="similar to AA sequence:UniProtKB:Q9KQW9"
/codon_start=1
/transl_table=11
/product="Lipid A export ATP-binding/permease
protein
MsbA"

/translation="MTFKELIKSNIPRSILIIGLYILYAIAGALGEYLFKNSLNNILK

GNLNGYIYWTFIQAAMEIGAAILLPIATIAFTKQTQSYLHHIREDIMQHYYNSGNDEK

VSEMQNQLTGNLKMLTTDFATPWVSILSGVLEMFISIGILLKMNWSLVLVSAILLGIN

FLIPKIMEKKTAQAAKQVNVKNEKLLNAIEHWLGGLQELRRFSAYGRLRKQLHQASDD

YTKASKESCKYNTISYLLNGWANALAQVGLAFFAGILFINRSISFGEFAVAGSFGFAV

FSAIWEITSAITQIKSTKELRQEISELRRNEKRTVKASAYGVSVKNLKISYDQGETIS

YPDFTIKAGDKVLLTGDSGTGKSTLFAVLLGKLKAQSGTVTYLDKNNQPLENATVGYL

PQDPVVFPVSIKENITMFTKKLEQQVLNVVNKVQLSTDLAKMPAGINTVVDLKNENLS

GGQRQKVVLARSEIHHQPFVLMDEVTSAIDQKATEKIIDELLKTDQTVLMVAHNFTPE
LKAKFDHEIKLEKKGGHE"
CDS 58533..59360
/gene="yokD"
/locus_tag="OCPHGMPK_00976"
/EC_number="2.3.1.-"
/inference="ab initio prediction:Prodigal:2.6"
/inference="similar to AA sequence:UniProtKB:O32003"
/codon_start=1
/transl_table=11
/product="SPBc2 prophage-derived aminoglycoside
N(3')-acetyltransferase-like protein YokD"

/translation="MTQDIITTITTKDDFKNTFISAQLKTTDKVMVHTALSKFYYVPG

GPESLVQALEETINQGTIMMPSEVTTNCDPADWEYPPVKQDLIQLVRDNMPPYNPDTS

PSEGLGITPEYFRTLPDVVRSCHPYLPIAIWGKDKVQIANKQPLDMPYGLNSPLDYLY

QNDGKIIFLGTDYETCTMLHYAESTIGRKTETCSAATGIDKNGKTIWTDYQNIDMDSY

DDFNDLGLAFERNFPNEFNQVKLNNSFIKVIKVKPLINFARVWFKKKDKNNLQSLEL"
CDS complement(59417..60307)
/gene="glcU_2"
/locus_tag="OCPHGMPK_00977"
/inference="ab initio prediction:Prodigal:2.6"
/inference="similar to AA sequence:UniProtKB:P40419"
/codon_start=1
/transl_table=11
/product="putative glucose uptake protein GlcU"

/translation="MQGIALLVGLGPLIGWGLFPTIASKIGGKPVNQIIGATFGTFIF

AVIYNLMQGIALPTGSALIWSIISGIGWASAQILTFHSFTLIGSSGTMPITTAFQLLG

TSLWGVFALGDWPSTSDKLVGFLALALIVLGAWMTTWTEKKTDENSAKLRKAVIVLLI

GEIGYWAYSAAPQAAKIDGSKAFLPQAIGMCLVAIFYAIYLKVKDPKQTIALAQGISY

KQIISGFFFAFAALTYLISAQPDMNGLATGFILSQTSVVLATLTGIWFLHESKTKKEM
IVTLVGLAIIIGAATMTVIV"

```
     CDS             complement(60334..60729)
                     /gene="rbsD"
                     /locus_tag="OCPHGMPK_00978"
                     /EC_number="5.4.99.62"
                     /inference="ab initio prediction:Prodigal:2.6"
                     /inference="similar to AA sequence:UniProtKB:Q2G1A5"
                     /codon_start=1
                     /transl_table=11
                     /product="D-ribose pyranase"

/translation="MKKTGVMNSNISRVIADMGHMDWLGVGDAGTPVPAGTEKIDLSV

RPGLPSFIDVLKEVLTELEVQKMYIAEEIKTENPKQLEAIKKTLPNVEIKFIPHSELK
                     KDLKSSKAFIRTGEETPYSNVILESGVTF"
     CDS             complement(60840..61289)
                     /locus_tag="OCPHGMPK_00979"
                     /inference="ab initio prediction:Prodigal:2.6"
                     /codon_start=1
                     /transl_table=11
                     /product="hypothetical protein"

/translation="MDENIQTFWHQFCKKHNLNENTPVEAWAFGANQKDADELASLVD

KEIKTATTSAYELYGKDEKLPKVGEWSIILDGNAKPVCVIKDVCVEIIPYNLISQEYA
                     YHEGEGDRSYAYWRTVHDEFFTREYKERGKIFYSQAPMVCEVFEKVK"
     CDS             complement(61292..62056)
                     /gene="deoD"
                     /locus_tag="OCPHGMPK_00980"
                     /EC_number="2.4.2.1"
                     /inference="ab initio prediction:Prodigal:2.6"
                     /inference="protein motif:HAMAP:MF_01627"
                     /codon_start=1
                     /transl_table=11
                     /product="Purine nucleoside phosphorylase DeoD-type"

/translation="MNEGPFILDFDKNLHAVFEPNHDQEPFYFHPRLLYAFVPEKEIN

AFLDQHLHRTLGEFESCSFNPKIYEVQIDNEFFTLCQAPLGAPAAVQLLEWLIAYGVE

KVLTIGTAGALVDLPENAMLLPIRAIRDEGTSFHYMQPGQFVELDSSFLKEVEKALTE

LNVDYDEITTWTTDGFFRETPKKVAQFRQLGASTVEMECAALAACAQFRKIDFAQLLF
                     TADTLADMDNYDERDWGGESHRTGLSIGAQILTRIK"
     CDS             complement(62071..62535)
                     /gene="rppH"
                     /locus_tag="OCPHGMPK_00981"
                     /EC_number="3.6.1.-"
                     /inference="ab initio prediction:Prodigal:2.6"
                     /inference="similar to AA sequence:UniProtKB:P0A776"
                     /codon_start=1
                     /transl_table=11
                     /product="RNA pyrophosphohydrolase"

/translation="MTQDYIKTLRKKVGHEPIILNFAGGILANDQNEILLQKRSDFNE
```

```
WGLPGGALEFGESSQEACVREFLEETGLKVKVKSLLGVSTNFIQHYPNQDVAQAVTIV

FVVELVEKTSKEVSSETLDLKYFSKDKLPQIFNKQHKLFIDHYFNEDYPFID"
     CDS             complement(62538..63038)
                     /locus_tag="OCPHGMPK_00982"
                     /inference="ab initio prediction:Prodigal:2.6"
                     /codon_start=1
                     /transl_table=11
                     /product="hypothetical protein"

/translation="MTSKLIIIRGNSGSGKTTLAKEIHHQLPRNTLLIPQDTVRRKML

NVKDGGKTLALPLLENLLEYGYHHCSYTILEGILNAKWYADLFEKSQQLFGSQIYAYY

FDIPFEETIRRHKACHEYSFGEEKMRSWWNEKDYIGFIPEYAFTSQMHLDDEISIIMN
                     DIQVEE"
     CDS             63123..63392
                     /locus_tag="OCPHGMPK_00983"
                     /inference="ab initio prediction:Prodigal:2.6"
                     /codon_start=1
                     /transl_table=11
                     /product="hypothetical protein"

/translation="MDNMIVRNSSASTRVSKSVKDKAIKNLAKRGITLSEFLRFAVGK
                     AADDDIELLNFLDTPEALQGKSEVENGNIEKIGSLDDLDKWMDNL"
     CDS             63389..63736
                     /locus_tag="OCPHGMPK_00984"
                     /inference="ab initio prediction:Prodigal:2.6"
                     /codon_start=1
                     /transl_table=11
                     /product="hypothetical protein"

/translation="MKQVEVYFSPTYKKELKKLKKKNYPIKLITQCLKAIIAQDNKIL

KKIKHHSLQGSWKGFNEFHPGRLSSNSNLNYDQWIVIYTFSKSKLILTLVSTGNHEIL
                     TKHKPKTLLNNKP"
     CDS             complement(63783..64496)
                     /gene="fbiB"
                     /locus_tag="OCPHGMPK_00985"
                     /EC_number="6.3.2.31"
                     /inference="ab initio prediction:Prodigal:2.6"
                     /inference="similar to AA sequence:UniProtKB:A0QTG1"
                     /codon_start=1
                     /transl_table=11
                     /product="Coenzyme F420:L-glutamate ligase"

/translation="MITIEALHNFPKIERKCDLSAEIIKTIKKEEFNIRDNDILCIAS

KLISKSEGLFVDLNSINPSQLALRLHQQIPKKDPRIIQIIIDQTDDKNGKKLQISKNF

IGGWLPNGLFLTSAGVDKIDADTAVVLPKNCDEIAQRISDDIFDQLKVRVAIIITDSD

GRIDKKGATQVAIGLYGISGIRKSQYEDKTNVETICDMLAASAGLLMGQKGKMLPIVK
```

```
                     IRGIDYVFDKSATIRDAVN"
     CDS             complement(64499..64894)
                     /gene="cdd"
                     /locus_tag="OCPHGMPK_00986"
                     /EC_number="3.5.4.5"
                     /inference="ab initio prediction:Prodigal:2.6"
                     /inference="similar to AA sequence:UniProtKB:P19079"
                     /codon_start=1
                     /transl_table=11
                     /product="Cytidine deaminase"

/translation="MLDKTWKKMYEEAKNVQEFRQISDHLEAGGVAAAVLSNTGRIYT

GVCIDTACTLGVCAERNALFNMITCGENHFKRVLAIMSDGKNGAPCGACREFMAQLME
                     GHYQDVEVMLDYENEKIVTLGELTPDWWL"
     CDS             complement(64884..66077)
                     /locus_tag="OCPHGMPK_00987"
                     /inference="ab initio prediction:Prodigal:2.6"
                     /codon_start=1
                     /transl_table=11
                     /product="hypothetical protein"

/translation="MRLKSVSFKIAVLFLVFTAVFTLFRSSMSGIFSLFYAKNGILDA

NISSIKSFQNIGIMFGLLPAGYLADKVGRLKVLALSSLIIATSFFILILFRNFFFFSM

AELLYGIGLALNSGTLLAYVTDLQEQNKIAPSSKLMGMQVIVLNLTTLIGGNIGTWLF

GIKDTAPIWFAMLGLVLYPVFIWFMVSFLSFSDNRASNKKKDKYNIKKIISFFSKRNF

WILLLLNVGYDCGTQFILIYWSIIYVEKLGFNLSLVYTLFMCALILGSVLFSKISTFV

NSKAITLLNTGSMIGAFVLSGTIGNKYLLLFLFLLIELLMGMMSGQISATSNEAIYGE

SNKSTMLSTISFIAEILVSVSLFISNSIMKVFGDLKVMFFVSAVYFGVVLLTIPLIKE
                     KQNVR"
     CDS             complement(66105..66422)
                     /locus_tag="OCPHGMPK_00988"
                     /inference="ab initio prediction:Prodigal:2.6"
                     /codon_start=1
                     /transl_table=11
                     /product="hypothetical protein"

/translation="MNLSVKENVIKEIKEEAGIDITVERLIAIHDSNKHYKGMYPYGI

TTVFFLCKPTGGSFTENDETIASGYFALDNLPELSEDKGSKEQVEMCFKAYYNPNWQA
                     EFE"
     CDS             complement(66419..66751)
                     /locus_tag="OCPHGMPK_00989"
                     /inference="ab initio prediction:Prodigal:2.6"
                     /codon_start=1
                     /transl_table=11
                     /product="hypothetical protein"
```

```
/translation="MDKQEQKQIMKQDRLIDWAKRLQSLAQAGLTYGKDNFDLERYQE

IRDISAEMMAEKSGLPIEKVKELFCNEVGYQTPKLGTRAAIFKDDKKFCSFRKMMVLG
                     QSQVVGVK"
     CDS             complement(66721..66987)
                     /locus_tag="OCPHGMPK_00990"
                     /inference="ab initio prediction:Prodigal:2.6"
                     /codon_start=1
                     /transl_table=11
                     /product="hypothetical protein"

/translation="MKCLFKLMIKGVGIWFILLMLYFVINLFINFNVFQISNLFGVRL
                     TIDVSKGRVVTMSSVAPNFYISLLLFTLFYGGIAFWINKSRSKL"
     CDS             complement(66992..67474)
                     /gene="bltD"
                     /locus_tag="OCPHGMPK_00991"
                     /EC_number="2.3.1.57"
                     /inference="ab initio prediction:Prodigal:2.6"
                     /inference="similar to AA sequence:UniProtKB:P39909"
                     /codon_start=1
                     /transl_table=11
                     /product="Spermine/spermidine acetyltransferase"

/translation="MELKKVTNQNLWDVVNLKVKSDQKEYVADNATSLLEAYATQNEG

KKVETFAIYEKDNLVGFAMINFNVYHWEGAPKVARNNYCLWRFMIDQKYQDQGLGVDA

LAVLIKYIKTMPLGNGNKLYLSYVPSNFAAEQLYKAAGFVPNGEKIEEEIVLVLDLEK
                     "
     CDS             complement(67477..68019)
                     /gene="ydaF_1"
                     /locus_tag="OCPHGMPK_00992"
                     /EC_number="2.3.1.-"
                     /inference="ab initio prediction:Prodigal:2.6"
                     /inference="similar to AA sequence:UniProtKB:P96579"
                     /codon_start=1
                     /transl_table=11
                     /product="Putative ribosomal N-acetyltransferase
YdaF"

/translation="MRRLHNYPFVLHNFKTGKHKVELVLLETWQAKDLFDQLQKNLVE

FSKWLVWAKKIDSVQKEVDSIKQFQQKMVDGTAFNLVILVDKIPAGMIDLHDLNPESG

EVGYWLSNDFQHLGIMTESVEFLKKYAFEQLGLKYLILRTHPQNFASQNVAKRSGFEY
                     QGIDDNGHKKFVLRNNVGRR"
     CDS             complement(68480..68650)
                     /locus_tag="OCPHGMPK_00993"
                     /inference="ab initio prediction:Prodigal:2.6"
                     /codon_start=1
                     /transl_table=11
                     /product="hypothetical protein"
```

```
/translation="MEALKQAQIKDNYESFIELVADYEQDELDQQIDLLTRIEKERKF
                     AQSQTRLDKFDE"
     CDS             complement(68692..68901)
                     /locus_tag="OCPHGMPK_00994"
                     /inference="ab initio prediction:Prodigal:2.6"
                     /codon_start=1
                     /transl_table=11
                     /product="hypothetical protein"

/translation="MKAWKISGSIVLLLYAVISVFICIRKVDGAGVVQTPEMRNITLI
                     IWGVFGLIILISYLIWLAILKHSKK"
     CDS             complement(68908..69267)
                     /locus_tag="OCPHGMPK_00995"
                     /inference="ab initio prediction:Prodigal:2.6"
                     /codon_start=1
                     /transl_table=11
                     /product="hypothetical protein"

/translation="MTKVELTRFRIKKGKEAKAQEWMDFLNEHHADTVATMAGEKMYV

ESVFKEKNPDGYTYFYWYSVQGEGGNTVEESKSYIDKKHLEYWDECIDTEYKPVDMKL
                     EENLIAPAVEKVIEKDA"
     CDS             complement(69260..69562)
                     /locus_tag="OCPHGMPK_00996"
                     /inference="ab initio prediction:Prodigal:2.6"
                     /codon_start=1
                     /transl_table=11
                     /product="hypothetical protein"

/translation="MIIDTKQLQKAVIENKKKHNFNTTDVKFELLLLYGEVNELFQAW

LKEDQENINEELADVAIFLLGISEMLGSDLGEDIVKKMKINAKRKYVHGKKIISDD"
     CDS             complement(69569..70015)
                     /gene="yafP_2"
                     /locus_tag="OCPHGMPK_00997"
                     /EC_number="2.3.1.-"
                     /inference="ab initio prediction:Prodigal:2.6"
                     /inference="similar to AA sequence:UniProtKB:Q47158"
                     /codon_start=1
                     /transl_table=11
                     /product="putative N-acetyltransferase YafP"

/translation="MLIRKYENSDFDQLCHVMDRARMQELKTANMEQVFIQLRDAPYL

GYLLKCKMYVATKEEKLVGFVGLRPHELSFLYVDPTFQKHGVGNKLMAFALDQLVRPI
                     KLDVFTDNFVAKALYEKYGFKVVKTVVEKWSDEYPIEFAQDTMEMK"
     CDS             complement(70015..70350)
                     /locus_tag="OCPHGMPK_00998"
                     /inference="ab initio prediction:Prodigal:2.6"
                     /codon_start=1
                     /transl_table=11
                     /product="hypothetical protein"
```

```
/translation="MQENVMSFVNISPLFIAIIIGFVVSFNENTSVKVPAIVVIISTI

ISFLFPIFNLKSWVTYPVIISESAMFVLATMLLSQKMKKWLAWILGLIVGFVWAIVLL
                     ILLGVTFNI"
     CDS             70489..72066
                     /gene="ndvA"
                     /locus_tag="OCPHGMPK_00999"
                     /EC_number="7.5.2.3"
                     /inference="ab initio prediction:Prodigal:2.6"
                     /inference="similar to AA sequence:UniProtKB:Q2YQ73"
                     /codon_start=1
                     /transl_table=11
                     /product="Beta-(1-->2)glucan export ATP-
binding/permease
                     protein NdvA"

/translation="MSLKFANKKKLALYVFLACISACGNVVIAYVTKIMLNSAQYHRG

SLKRLILIAALGAALLIVIMFLNFAYRYLRSDITRDINLKLKEKTITYLIAQQNDSQK

DGLNLMTNDLKQIESLKITNELMIISEIVAFAISIFVGLINSWLLTIIFVIATIIPGF

IQKFFIKDIQNKSAIWEKQNAEYTQATVDAINGSKTAMLYDAQVPVISYVIKQAKQME

NALRALNYTQGAISTLIITIADIFSFIIPFLVGAILMFNGQIGAGTLVMIVQLSNDFI

NPITMILDQLNQIRSTKPIWDKIQKAINFNNKNENAANPSFNQLSVEKLTYTVDDKAI

LKNVSFTVNANEKVLLMAPSGFGKTTLLRLLIGQLQPTSGSIIIDQQNVTGNWQKSHD

YFGYINQKPFMFDRSLHFNLTLGKKYSEEDLNEAIKLAGLTDLVKEKGLDYQVGQNGN

NLSGGQIQRIEIARSILAKRPILLADEATSALDNELSLQIHQVLLKNSNFAVIEVAHK
                     ISHQEKEMFDRIIDLSK"
     CDS             complement(72113..72595)
                     /locus_tag="OCPHGMPK_01000"
                     /inference="ab initio prediction:Prodigal:2.6"
                     /codon_start=1
                     /transl_table=11
                     /product="hypothetical protein"

/translation="MNAIFKRRAVREYANEKIDEEKIQKLIDAFQAAPCALHQTEVME

LSVITDPELLKKIEDNTNNSCYNAPLVFMINTKKDSQFGERDASVAAENVMVEAVDLG

LASVYVMGGVIALNKFPEVQKELGMDEGYETSVLLPIGYPADNGKPEDRSNRYKIVRK
                     "
     CDS             complement(72677..74284)
                     /locus_tag="OCPHGMPK_01001"
                     /inference="ab initio prediction:Prodigal:2.6"
                     /inference="similar to AA sequence:UniProtKB:Q9WYC4"
                     /codon_start=1
                     /transl_table=11
```

/product="putative ABC transporter ATP-binding protein"

/translation="MNYLFHYLRKRPGAVGIVIVLTIIASALRVTHALINVNIFNALI

KLEFHGFFYWVIIDIAVFAVLSVFLVLMQIQMAKTVQLLSLDLRKDIIRQLSGKSIEE

FNKQDTGVYASWLTNDVTTIENDGFYNILQSVQIITDPLFSMIALAKFSWTFIPIIAL

VSFLTVFLPQLIHKRLASASLSTTKANEKLLNVINDSLRGFNTFSIFGVEKQLEARVT

EAALFLLDKKVKQAKCQSIANNIAGFSNILGQVGIQAWTGYLALEKIISIGVIGSSGN

LAYNVFNSLAVIAPIWTEMTALKPIFDKYHLTEKKNYKEEGQDCTENKFDSLEVINLQ

KKYQDREVFAHPINLCILKNQKVGIYGKSGSGKTTFMKIISGQIRSYTGEIQLNKVEL

NRLKYSALQKILLYVEQTPYLFNDTVRYNLTLGEHFTDNEIIDALKKANLWETISKLP

DGLNTEINENGNNFSGGQKQRLALTRGLLRKRRLFLLDESTSNLDKKTALKVENTFLG
LEDATVIFVSHQLHNENKSAFDQIIEI"
CDS complement(74376..75194)
/locus_tag="OCPHGMPK_01002"
/inference="ab initio prediction:Prodigal:2.6"
/codon_start=1
/transl_table=11
/product="hypothetical protein"

/translation="MTIGELLKQTRQSLGLTQTEMAAGIVSTSYYSKVERNIHQISAD

ELIAILNRHHIDNAAFFAHLSSSSSQKKRDFIKEIGDAYEKKDKQTLLAIKKDIDQLP

DSKQKKYYQLQIELVLNVYLTKADDIPDELKNNLKKFVFQDNNWNENSLQLFRETIRV

YDIDELTFLVNAILVKYQQPAELPIKTQEILGGICINFLDKCYEHNTPELIKQPLQYI

SKLSNASELLLVKILKNYYQAVFNKNIDEVKTISGVLEKAGWKDFVSVLPHLSN"
CDS 75348..76223
/gene="hflK"
/locus_tag="OCPHGMPK_01003"
/inference="ab initio prediction:Prodigal:2.6"
/inference="similar to AA sequence:UniProtKB:P0ABC7"
/codon_start=1
/transl_table=11
/product="Modulator of FtsH protease HflK"

/translation="MLMRILLIVLVILLLVLLALSFHIVPQNYEGLVETLGKYSRTVK

AGFVMIFPGVQRIRKVSLALQPLEISKYRIITKDNAEITTSLTLNYLVTDSYKYFYNN

TDSVESMVQLIRGHLRDIIGRMELNEALGSTSQINAQLADAIGDLTDIYGIRVVRVNV

DELLPSPEIQKAMDKQLTADREKTAAIARAEGEARNIELTTKAKNDALVATAKANAEA

IKTQADADAYRIKKLQESLDKAGEGYFRNQSLDSFNQLAQGPNNLIVVDKDEISNLGK
IPAAKKIWDQSKNED"
CDS 76290..77096
/locus_tag="OCPHGMPK_01004"
/inference="ab initio prediction:Prodigal:2.6"
/codon_start=1
/transl_table=11
/product="hypothetical protein"

/translation="MEINRKELRQTARKQLKGNWLWVIGLLIIPGIVNLFLEYITNYV

WTGSFNTYNLSLVSWWQGLGWSIFTIITGLIATMIAWGVQYSTLAFRDTGEKPNVFKA

IFSSFTNGYFSKTFLTSLLTTLFTFFWGLLLIVPGIIKSFSYAMTPYIMKDMIDSKHE

MTATEAISESRKIMKGNKTTLFIIWLTFNIWYFIITLIGIVIAFLYLKPFNKPLADLI
FQALIAYLIAIILIAIINFLLSLYLQPYYRQTVANFYRSLAGDKYLKKGK"
CDS complement(77163..77738)
/locus_tag="OCPHGMPK_01005"
/inference="ab initio prediction:Prodigal:2.6"
/codon_start=1
/transl_table=11
/product="hypothetical protein"

/translation="MKNANEIVIGKPVKISHVLIGAAAIVILSLFVTGFGYLPWWVFL

IVLILGIFITLPTCFNSYWRINNEKVKITSYSNNDAIKLMQLLGLHKKDEQVIKLANI

ENAEIIYKKNIRISPVDFNPDYLNLYLDTKDGQKYTLSLGNIDYQKLDTIIQFLKENQ
IELIDKQGIVQLLRENKNLFTHFHNKKWTAV"
CDS 77822..78367
/gene="ydaF_2"
/locus_tag="OCPHGMPK_01006"
/EC_number="2.3.1.-"
/inference="ab initio prediction:Prodigal:2.6"
/inference="similar to AA sequence:UniProtKB:P96579"
/codon_start=1
/transl_table=11
/product="Putative ribosomal N-acetyltransferase
YdaF"

/translation="MKKISLTQFTETSLKEIWKQGFSSDHPEWTKFNAPYFNDYQKFT

NFESFKESTIAKYLLSEDCRCIIVNNQPIGMVSKDWIDEQTRWLEIGIVIYDSSKWSG

GYGTTALRIWINYIFQNTDKLEHIGLTTWSGNPSMMRVAEKLGFQKEAQIRKVRYYNG
KYYDSMKYGILRDEWKNVGKN"
CDS 78339..79067
/locus_tag="OCPHGMPK_01007"
/inference="ab initio prediction:Prodigal:2.6"
/codon_start=1
/transl_table=11
/product="hypothetical protein"

/translation="MNGKMSVKIKKIKDSQYIVWCDEKDIGTITTYHNEFHNKYLYLK

FNLTKYPTYFPFSKIKQIEGQSLQVMTSSPNTNLIHLLLQNGFKCKRQCYTPEVTKKD

LKIKLNSNYSIHPFNANSKNYSALCHLLYQYYQEVHQAVSPLTVSENTFVKEVPTKTG

YYSTNKDGKIENFVFTEKNEIAYICSFSEKSCDLFIQAVLSKMFNKYSSIFFEADDTD
WAATKLLDCFNVDKENSFNTYIYI"
CDS complement(79105..81375)
/gene="yhcR"
/locus_tag="OCPHGMPK_01008"
/EC_number="3.1.31.-"
/inference="ab initio prediction:Prodigal:2.6"
/inference="similar to AA sequence:UniProtKB:P54602"
/codon_start=1
/transl_table=11
/product="Endonuclease YhcR"

/translation="MKNHRRVLIGSLASAALMSLFLVNNGSVKADEVVQPNQVQQSGQ

VKVTTNQDNAKKPNNDQWANPANYKNDIPVQVLGINDLHGNIDTTGKAYVGRGMHYNA

GNAARLSSYLNNAENDFKDENPEGNTFRVEAGDMVGASPATSSLLQDEPTMKSLKAMD

VNIGTLGNHEFDEGLGEFYRIVEGKAPKTGQFNQEEQNYPHENSNLQIVISNVVNKKD

QTVPFDWQPYLTKEVKAGDKSSKVGFIGVVTADMPKLTFAKNLKDYQILDPAETIAKY

DHILQNQGVNAIVVLAHTGVSTYKGKTSGEAVDILKKLYKIDPNNSVDLYVAAHSHQY

ANGTVGRTKIVQANKFSMAYDDAIGYIDPQTNDFVNGSLVTHVYPVMSPKEDKLTKAD

PKVEAIIKDAENRTSKITNSVIGKTTPGTEISASLNDNKENAVGDLVVDAQLFEAKKK

GIQADFAITNGGGVRSNLNIESDGSIKWKSAQAVQPFNNQIQVFNLTGKQIYDVLNSQ

YVKDGERYYLVSGMRYAYTDTDDPSHPQKVAVMLDGNGKQIDLNKTYRVITSNYLVDS

TAEFKGAKKVADVGIDTDLFVDYFKDQTAAGKLILAPELNRKFYLTKPQFAELLNKLK

QEAQASKQNTVNNKLDEKNSQPANKTDKLVNRVDETKVSEPERSKKTAAAAKVVYVIY

KAKKDENTLKVVNESVGKSSSLAMFTRGKNFNRNESKKASLPKTGTENVSWISWLGLA
LTSLVSVFGLGIKKRK"
CDS 81624..82124
/locus_tag="OCPHGMPK_01009"
/inference="ab initio prediction:Prodigal:2.6"
/codon_start=1
/transl_table=11
/product="hypothetical protein"

/translation="MKIGLIQASSQKQKNGILEHYLNATINSDDQLINFGIYPNSSIN

LSYVQVSLAIALLINSKSLDFIVTGCTSGQGMMLACNTFPNIQCGYLPTPQDAFLFSH

INNGNVVSFPLGLNWGWSGEINFSETMKALFKQPWGTGYPPSQASRKVKNTTEVKKFN
QLNKNR"
CDS 82121..82267
/locus_tag="OCPHGMPK_01010"
/inference="ab initio prediction:Prodigal:2.6"
/codon_start=1
/transl_table=11
/product="hypothetical protein"

/translation="MTSILPSVDSDLLVPVLRYEPVYNFIIQNGKDTELVNLIKKLKH
SYFN"
CDS complement(82310..83071)
/locus_tag="OCPHGMPK_01011"
/EC_number="3.5.1.-"
/inference="ab initio prediction:Prodigal:2.6"
/inference="similar to AA sequence:UniProtKB:P59745"
/codon_start=1
/transl_table=11
/product="Carbohydrate deacetylase"

/translation="MKQILIRADDLGYSRAVNYGIYDAIHNGIINNVGVMVNMPATEH

GLDLLKNEDIDFGMHTDITNGKPILPPDQVPLLVDEHGNFKRSKIYRDNYGKQDFTNL

DEVIAEIDAQYHQFLELIGKKPDYFEGHAVMSDNFIKGLHIVAEKYDLPILDFAFDNQ

PVSFKKETRFSPYMESMKSNYDPEKVFLTMLKHADEEVIPMMICHPGYLDQYILNTSS
LTIPRTQEVDFAMDPKIRKIIEKEQIHLVRYSECK"
CDS complement(83073..83540)
/locus_tag="OCPHGMPK_01012"
/inference="ab initio prediction:Prodigal:2.6"
/codon_start=1
/transl_table=11
/product="hypothetical protein"

/translation="MHLVIISTMTLLFAQGGHDNFATLGAVAASMACAGMALGAALRI

KGKKNKTLVWGYFISNIIGGVTEPSLYGLALRYKRPFLGMMIGGFAGALYAGITHVTA

YVMVPVANFLALTAYVGGSTANIVNGIISGVIALIVAAVATYIIGFGKEEEIN"
CDS complement(83598..84497)
/gene="bglF_3"
/locus_tag="OCPHGMPK_01013"
/inference="ab initio prediction:Prodigal:2.6"
/inference="similar to AA sequence:UniProtKB:P08722"
/codon_start=1
/transl_table=11
/product="PTS system beta-glucoside-specific EIIBCA
component"

/translation="MSKDNYNNLAREILQAVGGINNISSVTHCMTRLRFTLKDSSIPK

DDEVKKIEGVLGVVRAGGQYQIIIGQTVPKVYETLLKLGNGKIAGSAPIDENLDANAQ

KEKMTFKRGFNIVLNKVAGSLTPTIPILITAAMFKMLAAICGPTMLNITPTSSDLYKL

FTLVGDAGFYFFPVFLGYTSAKQFKTSPMLGMLMGAILLDPNLVKIVTAGKPFTVYGI

PMALTNYGNTLIPILLSVWIMSYVERFFKKYVPTSLSTVFVPTLTIAVMIPITLCVLG
PLGGFLGDYICNGLLAFGKLGGV"
CDS complement(84490..85326)
/gene="bglG"
/locus_tag="OCPHGMPK_01014"
/inference="ab initio prediction:Prodigal:2.6"
/inference="similar to AA sequence:UniProtKB:P11989"
/codon_start=1
/transl_table=11
/product="Cryptic beta-glucoside bgl operon
antiterminator"

/translation="MRVEKKINNNVAVCTDGNGQELIALGRGIGFPKTPYELTDLSKI

DMTFYRVSSQTVQMLTTISEDVIMVSSKIVQLAQTKLQNQFSTNVVFSLADHISFAIE

RIKKNEIFDFSLSYDIQHLYPKEYDVGLKALQYVKEDLKIIFPKAEATAIAMHFINAR

EVSSQNISKSNTDNTLNLIIGTIEDQFNIKIDKKSFAFNRFKIHLQYFLKRIESNKQI

HDEISVQLIQDMMENNYQIFKCGKEIVAQLNEKYRTEITDDEMFYLMIYIQRIMRGVK
NE"
CDS complement(85339..85818)
/gene="crr"
/locus_tag="OCPHGMPK_01015"
/EC_number="2.7.1.199"
/inference="ab initio prediction:Prodigal:2.6"
/inference="similar to AA sequence:UniProtKB:P45618"
/codon_start=1
/transl_table=11
/product="PTS system glucose-specific EIIA
component"

/translation="MFSLFKKKEKTNIVAPISGELIELEKVKDPVFAKKMMGDGFAIV

PSVQNSTFDVYAPTDAEIVSLPSTKHAVGLRNREGQEILIHIGIDTVDLKGNGFTSYV

SQDDYVHQGEKLISVDDNVLKQNKLDDTVMVILVKGYKKNLLTAYGTQVDQETNLIE"
CDS complement(86060..86260)
/locus_tag="OCPHGMPK_01016"
/inference="ab initio prediction:Prodigal:2.6"
/codon_start=1
/transl_table=11
/product="hypothetical protein"

/translation="MGLTFNNPNNLQQMLDKFATVPDPKKTPKRPGEGLDKPKKNGGQ
TPNVINEEKEHEVKSKKKKDKK"

```
     CDS             complement(86424..87068)
                     /gene="yodC"
                     /locus_tag="OCPHGMPK_01017"
                     /EC_number="1.-.-.-"
                     /inference="ab initio prediction:Prodigal:2.6"
                     /inference="similar to AA sequence:UniProtKB:P81102"
                     /codon_start=1
                     /transl_table=11
                     /product="Putative NAD(P)H nitroreductase YodC"

/translation="MTQFENNNFGDVLLNRHSVRHFDKDVKIPREELREMIKETITAP

SACNLQAWQFIVVDTDEGKKKLHEFYMPFNNPQIDTSSAIIQIFGNTLAFKKYRALWD

HMYEEKRISKEELDKVYNTFLPLYEHADKTMLTADAMVDSSLAAMQLMLVARAHGYET

NAMAGYDAKKAASVMGLDPEQYVPVMGIAIGKPDASAEELKSVRYPVDDVLKFE"
     CDS             complement(87187..87462)
                     /locus_tag="OCPHGMPK_01018"
                     /inference="ab initio prediction:Prodigal:2.6"
                     /codon_start=1
                     /transl_table=11
                     /product="hypothetical protein"

/translation="MKEQRKKDKTFLVVGRIASIISVLMYVSYIAQIISNFQGQKGNP
                     IQPLVAAVNATLWVIYGWINPVERDWPIIIANAPGVILGLIAGITCF"
     CDS             complement(87482..88492)
                     /gene="btr_2"
                     /locus_tag="OCPHGMPK_01019"
                     /inference="ab initio prediction:Prodigal:2.6"
                     /inference="similar to AA sequence:UniProtKB:P40408"
                     /codon_start=1
                     /transl_table=11
                     /product="HTH-type transcriptional activator Btr"

/translation="MNTEILELLHQNREYKDWKKVENKLKPLIVDYINGEPVYGFYET

LKDKSLNKNGQLISISTQPVDSFVPYHIHNYVEMMVVLLGNCTIRTASNTIHMKQDEI

IVMGRDTIHKVDEIDSGTIVVNIALKPAAFSLNDLDFLTHKEGVQSISSMLFSLLASN

NSKVAFNLFHTNHESHVVNTVYDIISEYYQPDSFSNQIIKMEVLELFLRLIRIASHSP

NLVNAKKRQNNNEIDLLTLLLYIEKNYKFITLDNMAAYFGFNANYLSAYLKKRTGYTF

IKLVHIQRINVAAEYLTFSNAPIDNISTEVGYENPSYFYKVFKKYIGCSPSDYRKKYQ
                     KN"
     CDS             88682..90388
                     /gene="nag3"
                     /locus_tag="OCPHGMPK_01020"
                     /EC_number="3.2.1.21"
                     /inference="ab initio prediction:Prodigal:2.6"
                     /inference="similar to AA sequence:UniProtKB:Q7WUL3"
                     /codon_start=1
```

/transl_table=11
/product="Beta-N-acetylglucosaminidase/beta-glucosidase"

/translation="MMVDLSAKPYYLNKDQIKYVENTVKNMSVNEKVGQLFFVIGQDE

KQVNITEFIKKYQPGGMMYRPDQAEKIKREIATAQTTSKIPMFFAANLESGGNGIVTE

GTWFGNPLQMAATDDSHSAYELGNVSGEEAAQVGNNMSFAPIVDIDNNFRNPIMNTRT

FGSDKNRVIRMADAQIKGLKDNKIIPVIKHFPGDGVDDRDQHLVSSVNSLSADDWMNS

YGEIYHHLINAGIPSIMIAHIMQPAWERKLDPGIEDKDLHPASSSKLLINGLLRNVLK

FNGLAITDATPMIGYNAAMSRKDLLPATINAGIDMILFNKNINEDYRFIHEAIDNGTL

SMKRVDEAVTRILGTKLAQGIMNTNGELTLLPPKEINLHSAEHQKLAEKVAKKSVTLV

KDRDQLLPLTPKKYPHVRLVVLGDSDDGGFKEGGHVTQAFKDALEKEGFKVTLFDRKN

LDFHEMFEEGVEDMKKKFDLAFYVANVETASNQTTTRLDWIHLMAADSPWYMKSIPTV

FISTANPYHLYDIPNVSTFINAYTGNQASIDAIMRKVTGKEKFEGKSPVDPFCGDFTT
RL"
CDS 90453..90578
/locus_tag="OCPHGMPK_01021"
/inference="ab initio prediction:Prodigal:2.6"
/codon_start=1
/transl_table=11
/product="hypothetical protein"

/translation="MNYQQQFNKLKNMFAHNPMFQNMDHKQNKAPKIHLQAFRNR"
CDS complement(90737..92023)
/locus_tag="OCPHGMPK_01022"
/inference="ab initio prediction:Prodigal:2.6"
/codon_start=1
/transl_table=11
/product="hypothetical protein"

/translation="MITVKGKYLYKNNKRFFYLADTCWGAFTSVSMPDWKYYLNMRKH

EGFNAIQINILRQYDSTPPIDGREPFPITYNEDGTYQYDYTQFNDAYFDNAEMMLAEM

MKRNMLPVLVLLWGNFVPNTWMNDPHGFQANTSKPQTISFDRVKPYITYVVKRFKKFN

PIYFVSGDVGFDKNEQQNPEIEEKYYQEVIDAAKSIDPNGIYTFHINGESSTLPDNLA

RKASFFSYQSGHGPSGEEAAEKIPKKLRKESYQGPIMDVEPCYEGMTQFGVLPTHRFS

AYDVRRTAWKAVLSGADAGLGYGAFGIWPWNDNYRKGQKVTGFLKPYDWRKCLNFRGA

KDLAFLKESVLQYGADGLKPIALENKEVYAAENEKYVLIYNPVANPVDLSELKVKDSS
CRIIDLNTLRMLEGTICNSTVSMEPVIEDELIVIEK"
CDS complement(92094..92759)

/gene="yvdM_2"
/locus_tag="OCPHGMPK_01023"
/EC_number="5.4.2.6"
/inference="ab initio prediction:Prodigal:2.6"
/inference="similar to AA sequence:UniProtKB:O06995"
/codon_start=1
/transl_table=11
/product="Beta-phosphoglucomutase"

/translation="MLKGLLFDLDGVLTNSAKFHLAAWNNLANELGITLTDDQLDSLR

GISRIDSLNLILKYGGQENKYSEAEKEQFAAEKNAKFVKQVETMTAKDILPGISKFLV

DAKKQNLKMIIASASKNAPRILTRLGIMDEFDGIVDPATLHNGKPDPEIYIKAQQIAG

LQSNEVISFEDAEAGVAAIKAAGQFAVGIGNKELLKEADYIVSKTSDLKLSEIETVFQ
KRK"
CDS complement(92767..94296)
/locus_tag="OCPHGMPK_01024"
/EC_number="3.2.1.55"
/inference="ab initio prediction:Prodigal:2.6"
/inference="similar to AA sequence:UniProtKB:A7LXT8"
/codon_start=1
/transl_table=11
/product="Non-reducing end alpha-L-
arabinofuranosidase
BoGH43A"

/translation="MIKSYQNPIIRGMHPDPSVVRVEDTYYLANSTFEYYPGVTVMTS

KDLLNWQTLGGVAKLPAQADLRKSKSNEGIFAANIRYHNGYFYIITTNFAEFKTFIIR

GKLNSDGIIEWEKSRVEINVPGIDPDVYFENDRTYVQFTGYIDDKGTKAIRQVEIDLA

TGEILDGPRILTYGTGGRDVEGPHIFKKDGWYYLLVAEGGTGEGHMITIFRSKELWGS

YESAPTNPIFTNRDHANEPIQNVGHGDLFTDSNGNWWLVCLGTRPANVGFKQITNLGR

ETLLYPVEWENGWPVINKGVPTEEVNLVDFPKHAAALDNKQKVTEFIDDFNEDKLRPE

WLTLRDALGNNLTINDSCLILRGNDLTIGDLGTAGFIGLRQTEHEEVFEVKINDKKSN

LNAGSFGIASLIDAENHAAIMIRKDNHGFDVYRDQQIADIKLNEKIGHLNKMPASFKL

INTKETKTFGVDDGEKEVSFTTNAIHLSNQAIAALNTGDIAGLYARNGAIMVVDKVKR
G"
CDS complement(94299..97085)
/locus_tag="OCPHGMPK_01025"
/inference="ab initio prediction:Prodigal:2.6"
/codon_start=1
/transl_table=11
/product="hypothetical protein"

/translation="MKISNIQVNHMNKPMGFNLSSLRIDFKIEDTNETNLKKQLIISS

NDQVVYDSNLITYNNNYFNVPFNLEPRKHYDVKVIVSGKEKVEGTTFFETGKINEKWT

AKWIANKNKDLQNTLFKKKFSVKDSVKSARLYITGLGLYEAYLNDPKIGNEYLTPGST

AYDKWVEVQTYDITNQLKEDNEILISTGDGWYKGNIGFDGGQINIYGDQQMAIAELHI

EYEDGHEEVVNTDASWQTTSGQITKSGIYYGEDYDATIEIKDWQNVVVLDKDTSVLKD

RLSLPITVHENLKPVKLIHTPKGEEVLDFGQNHAGLLEFYNREPKGTKIILQVGEILQ

EGNFYRDNLRKARAAFTYISDGKEGWVRPHFTYYGFRYVKVTGNTKPINIDNFRAPVL

YSDMRETGDIETDNKLVNRLLKNIQWGQKSNFIDIPTDCPQRDERLGWTGDADIFSNT

AALNMNVFEFFKKYAKDMAVEQEDHNGMLTMYAPKMGTDDGGAAIWGDAATFIPWNMY

QAYGDSAILKQNYSDMKAWVDWITKHTKVPNLWIGCFQFGDWLALDGEVPGLPTGKTN

EDYIASVYYYASIKIVAETAKVLGHSKDAKKYQKQAEKILQAIRDEYITKNGRLAIDT

QTAYALALYFNIVAKDKKARVAKDLVTRLHKDNDHLKTGFVGTPFINQVLSQNGYHKL

AMKIFMQEDFPSWLYAVKLGATTVWERWNSVEADGSMNPEGMNSLNHYSIGAVMEWVY

RYVLGIREHKNGYQRIEFAPMFDYRLKHVKGHYDSSYGRFNVEYQIETDESHTIKVNL

DVPYGTVVKADLPRAKRVNVNGEEKDNGFELTVGHYEINYIPTHDYIERYTEETPVKV

IMNDKELVLKIDEVSGALKMFENNPSSLNGPLSGMSLAKINEIIPTFNISNEEFEKIN
SILTSTPIESERKQ"
CDS complement(97098..98279)
/locus_tag="OCPHGMPK_01026"
/inference="ab initio prediction:Prodigal:2.6"
/codon_start=1
/transl_table=11
/product="hypothetical protein"

/translation="MNKKKIPLAIGILSMNLLLMSASVVGAAIAAIAKSFPKEPISKV

QMLSTIPQLGQVIATLIFTWLTYIMTRKNISLVAILFVIVGGLIPAFYNSNLNIILAC

MIAIGFGTGLISNVGPVLLQEHFEGETRATVMGWSVGVNSVGMMAFTAIGSALGSTDW

RNLFWIYAVGVVILLVTYFMVPQDTRLKREKNSSGKASIWATLKNVNKLAYIYLLITF

FTSIAMTMFMANQSIVLAGKGMGTGYTGLVTALGNIGGIITAALLGKIRKFTKNYTIA

YGFIAFALSFICVLFFNNIVMHIIGNMFSGMGIVLINATIPFQLSNLADRSEFPLVIS

MNTLISSIAGALAPVLLAALHLTVGSNQFIAGIILSVAITVLIFVTRLGSIVGKATEN
K"
CDS complement(98459..100849)
/locus_tag="OCPHGMPK_01027"

/EC_number="3.2.1.23"
/inference="ab initio prediction:Prodigal:2.6"
/inference="similar to AA sequence:UniProtKB:A7LXS9"
/codon_start=1
/transl_table=11
/product="Beta-galactosidase BoGH2A"

/translation="MRKIIDLNTNWSFHFGEIRKPNFLAKKSYAFGGLTEPLGDEEGE

LLPLGAGGKHFLNLISGGHPEIGLHNLAGTEFIDSLGQEWKNVNLPDDWKRREPYKND

PSILMQGSKPDGIAYYRHTFKLPDNLDENKKYILHFDGIMGSSDVWFNGAYLGHNDSG

YVSIDLDVSDLIRFAQEGLNVILVRNDTTTGSEGWWYEGAGIYKKVWLEVTDLAYIER

DSFFLRTLTLDNKKVQVQVEFCVYNEKDKSINVRPRLSINNKEFFFDQVDIAAHQKSN

FVRKINIYNPYAWSPEHPYLYSVKLTVLDDELTKEVGLKTVAYDTNGFILNGERYQLH

GVCEHQDFGGVGVALNQDIIDYKIKTLKKMGVNALRSSHHFASQELLDACDRLGIILM

DENRLLETTPWRLADLRKMVKKSRSHVSIAFWSIANEEVVGNTNYAIRSVKRLTETIR

MLDPDKLLVSAELLNPEGIVNDEYLKYFDIFGVNYPESGVMGNGAELIHESHPDLPMM

STENASYFSTRGIYQDDGDKCECSNFGSMYSMVLPGKRKPDDPGVGGTAHPEKVMEYL

KSHHYMSGVFVWTGFDYYGEPSPFGWPGISSQFGICDLGGLPKDYYYYYQANWTKKPM

IHIMPHWNKKGLEIINGQTKVRVFSNAEEIELFINGRSQGRKEVKNYQVNWDVVYESG

KLTAKAYIGNKVVSSDEQITSGKPTKIKIINRYHGKNNTIFELQAIDDDRNFVPTANN

SISLECEDGQIIGLTNGDPSDVSPYSLSEIKLFSGKAIVIIEHKEHTSPKLQLKIK"
CDS 100968..102074
/gene="tetD"
/locus_tag="OCPHGMPK_01028"
/inference="ab initio prediction:Prodigal:2.6"
/inference="similar to AA sequence:UniProtKB:P28816"
/codon_start=1
/transl_table=11
/product="Transposon Tn10 TetD protein"

/translation="MSEKIYMAYNKIQDYLNSYFAKHKIEASMFDAIHYLYNQRDFSY

KSTELNWPEEKCSNLDDLYQVLKNIAIEVTPIIRNSTANRLIKNVSEHSFFNKRQDAN

ILLQFQHDKNQLHKHDYFEINLVLKGMLIATLNEEKRMLKKGDFLIISPNTIHQINVG

SDSIVVCITIRKSTFDKAFFSLIQNDDPISNFFKYNLYSAKQNYLLFSIDINYQLLET

IQNIFIQAYSTSSQANIICCSYISILLSYGLQGLTTSTLSVQNNTLTNKITTILNWIK

KDSATIELNQIAKKLGYDKAYLGKLIIKNTNHSFNYLRNYYRIHNSCQLLQFTDYSIE

KISIKTGYSSPNHYERCFRQLMKTTPTKYRSNKK"
     CDS   complement(102121..103653)
                     /locus_tag="OCPHGMPK_01029"
                     /inference="ab initio prediction:Prodigal:2.6"
                     /codon_start=1
                     /transl_table=11
                     /product="hypothetical protein"

/translation="MQTVEKWKKYELTLNGPSDGNPFRDVELTATFDRNNHQITVDGF

YDGNGVYKVRYMPEVEGEYTVTTHSNVPELDGKTDTFTVVSASADNHGPVRVAGKTHF

AYTDGSSYIPFGTTAYAWTVQPKEVQEETLATLKENKFNKIRMLVFPKSYSYNEEEPA

IYPFEGFLRYKRGANDWIFDAKETGFDYTRPNPKYFQNLEDNIEKLDQLGIEADLILF

SPYDRWGFARMGLENNLWYLKYVIARLASYKNVWWALANEYDLMDLVGQIPLKDWDRI

GQFVHEKDPSQHLESIHNFYDPPQHKDTIKNWYDHTKPWITHLSVQSDNVFLIPKWIE

EYQKPVVDDECRYEGNIEFGWGDNTAKGMMDNFWRVILRGGGCTHGETYIDKPHTKRP

IWWAHGGKLYGESQKKINFLYNLLKDNGFDWVKPQATSGPTWELAAGSTPDEKNWLIY

FGENQPEFEVLSFLPKGKKYRAKLINAWDETIEDFDQEVQNDQYIHLPRKDYQALLLT
                     EE"
     CDS   complement(103672..104898)
                     /locus_tag="OCPHGMPK_01030"
                     /inference="ab initio prediction:Prodigal:2.6"
                     /codon_start=1
                     /transl_table=11
                     /product="hypothetical protein"

/translation="MNVEEIKRSNKHNWKLKVGILGISLFLQVAGSNLAAIPLIAKSF

PNQSLTSIQALFTLPSFTIMLFILFSNAVIKWVGKRNTVIIGMIATIIGGLIPFFINN

FAIIVASRLLVGAGIGLFTSLAVSLIGDCFSGEEQKTLIGIQGAMGTLGNSSMTFVAG

LLLGIKWQATFLYFLVIIPFLILFMMGYTSKMEKETTIEKSGSKKINQTSKHAKIPAS

IYVAFVMLFLYFSAFMVEATASALVIQQNHLTNQGMLSTEIAIAGLAGAVISMAYARI

FKILKNFTPVVAVALGSVGFIVCSQASNMTIFFIGLLLMMISSLIIPYVYDTILSEVD

SSISNLIISIAQICNNLGAFASPYVIALLSGMTGLSTAVDQMRISAGILGVIAIVFVI
                     IAVSRSGKKASAKVAK"
     CDS   105053..106015
                     /gene="cytR_2"
                     /locus_tag="OCPHGMPK_01031"
                     /inference="ab initio prediction:Prodigal:2.6"
                     /inference="similar to AA sequence:UniProtKB:P0ACN7"
                     /codon_start=1
                     /transl_table=11

/product="HTH-type transcriptional repressor CytR"

/translation="MTTIRDIAKLANVSVATVSRIINNSGKVSASTKEKVNKIINETN

YHPNQVARTLYQKRSKMIGIIIPFIENVFYARVINGVQSVLQPAGYTCLVSFNVGSDS

QKYQTAIDNFLQNNIDGIISSAFDLHENYYHIPFVMYDSANIDDNIVRIASNNIKGGI

KVVDLINNNRLKKVLIQHLPLKLPTVNERISSIIKELNKKQLNYTLQEINESDTYNIA

AEKALKNIQKYDAIITINDIYAAYILKLAKKMNLKVPEDFQLVGYDNNELSEFTTPTL
STIDQQPELIGKTAAKRLLDMIHGKKSTENSLIDIKAVKRESTK"
CDS complement(106081..106989)
/gene="axeA1_2"
/locus_tag="OCPHGMPK_01032"
/EC_number="3.1.1.72"
/inference="ab initio prediction:Prodigal:2.6"
/inference="similar to AA sequence:UniProtKB:D5EV35"
/codon_start=1
/transl_table=11
/product="Acetylxylan esterase"

/translation="MKYFKKNIGENGAEATFYLQEPNKEIDINRKSPVMIVVPGGAYM

WTSWREEEPIALEFLGKGFSCIVAQYATEGLSFYDEKHDYSKEPVSAFPNPLVDLAKI

IAYVRENAEEWNLDSNSINVVGFSAGGNLTAQLGVYWNTDWLEKLVGKPRKKYQPNSI

CLSYGAPHMAKTPEKDRYRQTKKMGGSNMIDYATLGNDQSEERVRLVNMVDTVSQDVP

PTFIWHTMEDPYVPVASALHFASQLEEKMVPFELHIYQKGKHGLALADLRTDSKKDRS
QSNEQAATWKRLYLNWLKENKLINLF"
CDS complement(107002..109194)
/gene="bglF_4"
/locus_tag="OCPHGMPK_01033"
/inference="ab initio prediction:Prodigal:2.6"
/inference="similar to AA sequence:UniProtKB:P08722"
/codon_start=1
/transl_table=11
/product="PTS system beta-glucoside-specific EIIBCA
component"

/translation="MNSNYQEVAQKILVAVGGAENIDEVFNCIARLRFYLRDLNKINY

QELGIIPEVSESKFEDGQLQILLGQDVEKYYQALTNLMKQTDANRGVKKMSKADEKNK

KLAEQIVKLIGGKDNVISLVHCVTRLRFKLKDEKKANDDAIKQLKGVMGVAHAGGQYQ

VIIGNNVADVYDQVMPLLGLSSEEEVTTDDEKDGNIFSRLVALISSLFMPLLGVMTGA

GMLKGLLVLLNVLGWVKQGTGTYMIWNAAADSLFYFLPVLLGFSAAKTFHVNKYLGAV

TGGALVYPTMVAAATAHKAITFFGLPVNLMNYSQTMLPIVFAIWGMSWFEKGTKKVVP

KVVQNLFVPLLDLMVIVPLTYLIVGPVMQTLSQWLSSASLWLYGLVPVVAGFIIGGIW

QAAVIVGLHWAFIPVLMNNLMTNHFDPINGIMYCTLFGQVGAALAMGLKAKDKNFKEI

AIPAAVSGFFGVTEPIIYGVTLPHKKSFVFASIGSAFGGAIAAAFHAGMYTMPGGGIF

GIPAFINPKGIDASFIAFVVSLIVAFAVAFILTFIWGDKVVPATPNKKFDVKKVEFKD

QGIVSPIQGAAVELKNVNDPVFSAGTIGKGLAVQPNNNEVRAPFDGEVISVFPTKHAI

GLKSENGVELLIHLGINTVNLKGKYFDAKVAAGQKVKQGDLLETFDVDQIKAAGYDTV
VPIIVTNTNNFDDIIVEKKDGDSVKFGDQILIATVDQEVEIPENATQA"
CDS complement(109200..110042)
/gene="licT_2"
/locus_tag="OCPHGMPK_01034"
/inference="ab initio prediction:Prodigal:2.6"
/inference="similar to AA sequence:UniProtKB:P39805"
/codon_start=1
/transl_table=11
/product="Transcription antiterminator LicT"

/translation="MLLIKRVLNNNAVVATDKHKQTLVALGNGIAFHKKAGEIISEDK

VEKIFYPQNEKDTNSMSEMLASIDPKYIELSDQIISETIASSGKKLSDDIYLSLPDHL

QFAVERLKKGMPIQNRLTIETMQTYPDEFQLGKRVLRYLEKTENLKFPEDEATNIAMH

LITAEEGNSLEHTAGTIELINRFLEIISEMLKMEINSKSVAYYRLVTHLKFFAQRISK

KQTQESNVDPELYNMVIHRYHKEYLISQRIAGIVMQKFNYKVSQDEIMYLTIHIHHIA
VADR"
CDS complement(110213..111508)
/locus_tag="OCPHGMPK_01035"
/inference="ab initio prediction:Prodigal:2.6"
/codon_start=1
/transl_table=11
/product="hypothetical protein"

/translation="MLTTKGKYLYKDGKKFFYLSDTCWGAFTSIEMSDWRYYLDTRKS

EGFNTIQINILRQWDSSKPLAGREPFAIKEHNDGSYEYDFTKLNESYFDNAVKMLEEM

QKRDMVPALVLLWSNFIPDNWIASYHAAKNSLMPFDQIKPYVTYVVNKFKKFHPIWFV

SGDVGFTDNEKQDRKKAIEYYREVLNTAKEIDPEGIYTFHINGESHDLPEEFVKQVSF

YSYQSGHGYAGQKTAYTIPLVLRKKQKYDGPIIDTELCYEGLTQMHSPEPKRYSAFDV

RKAAWRAVLSGADAGLGYGSFGIWPWKDSSHPEQKLEQNFNVQLVPYDWRTCLTFRGA

KDLGFLKSILDEYALSGLNSLNSNEDNAIRAAESENYVLIYLPTAGTLDFSKFGFNVN
ECKVIDLQKRTILEGKVENITLQMLPILEDELVIISKKH"
CDS complement(111639..112451)

/locus_tag="OCPHGMPK_01036"
/inference="ab initio prediction:Prodigal:2.6"
/codon_start=1
/transl_table=11
/product="hypothetical protein"

/translation="MYKLIDEKDPRIEETKDVYRKWADIKIKDEDFTKVAALKLAMQD

KMREYGCNAGTIQCWTELQREIGILPYASEALLQTEGMPVTCETDINGAISEILAESA

TLGNERALFADVNCRHPENENGELLQHLGTFAFQTAKNRPVLPPSHFVFDYPGSAAFE

VKDGTYTLVRFDGDHGKYSMLIGNAKTCDGPYEQGTFAWFEFKDLNRFEAKYVYGPYI

HHMAGVRADVVPILAEACKYINVAPDYYDPIKDKVAAYWYGDLDTILDDDQK"
CDS complement(112460..113092)
/locus_tag="OCPHGMPK_01037"
/inference="ab initio prediction:Prodigal:2.6"
/codon_start=1
/transl_table=11
/product="hypothetical protein"

/translation="MTNKNSLKQIKLGFAPTRRNIFSATAAIEFTNKTREKLDELGVN

YVDIKDVNDDGLLYDDEGLEKITEKFKEAKIDGLFIANENFGTEYEVARLAAKLNVPV

LLWGPRDEAPAPDGSRRRDTQCGLFAIGKVLRHFNVPFTYIRNSDIDDPSFSRGVIDF
IKVCNVVKTFKNTRILQIGTRPFDFWSVIVNEGELLENLVSNFLQFRWAN"
CDS complement(113193..114212)
/gene="galR"
/locus_tag="OCPHGMPK_01038"
/inference="ab initio prediction:Prodigal:2.6"
/inference="similar to AA sequence:UniProtKB:P03024"
/codon_start=1
/transl_table=11
/product="HTH-type transcriptional regulator GalR"

/translation="MKLKMSDIAKKAGVSKAAVSFALNGKPGVSEETRKKIFKVIEEL

GYEPLRKHKKGGVRKLTSISLVIIKDTTGMMSRSYASLPFFDSLISHLSENVGGFGGQ

VQIITLNIRNLKKDLEESEALNRSKAAIILATDLNKNQVLFLNSKIKNAIFIDNYFED

VDADFVSIDNFQGGYNAGKFIVDSGYTKIGYVASNRIITNFLKRREGFRAALKEKNIE

VPNEFVFSLNPIELKGTLPEFFYSGQEYPHAIFCEDDYMALRLVKELTRIGTKIPEKV

AIMGFDDIFEDTMINPELTTIHVPIEQIVRQAINQLQEKVSNSNWLSQKSFVSTKLIV
RESLK"
CDS 114345..115178
/gene="fas2"
/locus_tag="OCPHGMPK_01039"
/inference="ab initio prediction:Prodigal:2.6"
/inference="similar to AA sequence:UniProtKB:P46374"

/codon_start=1
/transl_table=11
/product="Ferredoxin fas2"

/translation="MNSLDLKKKAVQLRKRTWQLIYNHKNGHTGSDLSCADILVALYY

DVLNQTPETFEDKNADTYIQSKGHAVEIWYEILADKGYIDQEDLDKRYSTFNSPYIGH

PTTDVKGMEFHTGSLGHGLGLGVGVALAAKMDHSKKRTFVLMGDGEQAEGSIWEAAMA

ASNYSLDNLTAIVDHNDLQITGTTDSVMRSNPISNKYQAFGWDTIEVNGNDIDALVNI

LNKPNNPGKPRVIIANTVKGKGITVAENRADWHHKIPSEAEYQEGLRELDAQLEGLNN
D"
CDS 115171..116112
/gene="dxs_2"
/locus_tag="OCPHGMPK_01040"
/EC_number="2.2.1.7"
/inference="ab initio prediction:Prodigal:2.6"
/inference="similar to AA sequence:UniProtKB:Q9RUB5"
/codon_start=1
/transl_table=11
/product="1-deoxy-D-xylulose-5-phosphate synthase"

/translation="MTNEKKLTANAVIANTLDKATKTDPDLLVVTCDSRGSAGLVNFT

NAHPDRTVEMGIAEQNAVTVAAGMAHAGKHPFVFSPAAFLAMRSIEQVKVDVAFNQTN

VKLIGISGGNSYTWLGTTHHSLNDVAITRAIPNLEVYQPCDQYQTRALFKYLLKSQHP

AYVRVGKRKLDNVYHQDFDFIPGKAKVIRKGHDICLISVGEMLYFTLQAAKNLAKAGI

DAEVVDLSSIKPLDTEMLDQLAQQFDQIVTVEEHDVINGIGSAVASEVAKFGHAKLTI
LGFPDEPAIQGTQDEVFHYYGLDSEGIEKSVKKVLKK"
CDS 116122..117573
/gene="glpK"
/locus_tag="OCPHGMPK_01041"
/EC_number="2.7.1.30"
/inference="ab initio prediction:Prodigal:2.6"
/inference="similar to AA sequence:UniProtKB:O86033"
/codon_start=1
/transl_table=11
/product="Glycerol kinase"

/translation="MTEKYVLAIDQSTQGTKAILVDHQNQIFCKTSLAHKQIINEDGW

VSHDLNEIRANLITLFKQVLKKVTPDQIESLAITNQRESAAAWSRKTGEPLCKTIVWQ

DNRAEKLISRISYPELTDKVKNSTGLALSPYFTGAKWGWMLLNEPRVVQAQANNDLCL

GTMDTWLVFQLTNGQSFKTEPSNACRTQLMNIKTGNWDQELVEIFGIDINDLPDIVDS

NSNFGETDLFGLLPHKIPILSVLGDSQAALFAHGCFETGDFKVTFGTGSSVMLNIGKK

LPNNFNNQLNTSLAWSINGQKSYVLEGNINYAGACVTWLKDKLHLIQDPAETANLALT

ANPNDHTLLIPAFAGLGAPYWLPDMKAAFVNMTATTGKKELVRATLNSLVYQINDILH

AFHQLSPKVNDEIHTDGGMIKNKYLMQYLSNITQKNVKIANISELSALGSAMNAMHHF
DGMQYSKTYTPRINKNTAKDYCNEWTTWIQKLA"

```
     CDS             complement(117632..118495)
                     /gene="thrB_2"
                     /locus_tag="OCPHGMPK_01042"
                     /EC_number="2.7.1.39"
                     /inference="ab initio prediction:Prodigal:2.6"
                     /inference="similar to AA sequence:UniProtKB:Q8Y4A6"
                     /codon_start=1
                     /transl_table=11
                     /product="Homoserine kinase"
```

/translation="MKIFVPATSANMGPGFDCLGTAVSMFLELDVLETSDKWFVEHDM

NEISHDENNLIVKTALELAPRLTPHHLSVKSQIPLSRGLGSSSTAIVAGIELANQLAN

LNLSEQDKCRIAAKIEGHPDNVMPAILGGMVVASKIENQYYFQELPLIPFDFVAYIPS

YELDTKASRNALPEKLAFKNATYESSILGTLTASLALQDYETAKKMIEADEFHEPYRQ

KLVPELVKIREIAHQHEAFATYLSGAGSTVMTIIEHSRTEKFIESLREVGLDDRIEQL
KASSQGVFVEE"

```
     CDS             complement(118505..119731)
                     /gene="hom"
                     /locus_tag="OCPHGMPK_01043"
                     /EC_number="1.1.1.3"
                     /inference="ab initio prediction:Prodigal:2.6"
                     /inference="similar to AA sequence:UniProtKB:P19582"
                     /codon_start=1
                     /transl_table=11
                     /product="Homoserine dehydrogenase"
```

/translation="MKTIEVAMLGLGTVGSGVIDILKHSKEKIRQITNTEFKTSKILV

RNIDKYRSLYPDLNLTTNFEQISQDQDIKVVIEVMGGLHPAKEYVSELLKQGRTVITA

NKDLVASFGPELMAIANEHQGRLFYEASVAGGIPILRVLNQNYVGDQIKQISGIVNGT

SNYILTQMADNNWSYEQALSEAQKLGFAEADPTNDVSGKDAAYKLIIILGRLAFGINLK

LDQIKMMGITEIGQEELAIIKKFGYTLKLIAKLETQNSTYYPVVMPMLVSENSMLGQT

KNEFNGIEIESYAIGKSFYYGPGAGKLPTANSVINDLVAVAKKEAPFKTDWNSVSVSV

KDIFTRQYLFKVDSSQNKVVANLMDRYQIEDADFLNDEQYGYYLSGNITVDQKAKLFN
ALNEKNIKVYSMYEVY"

```
     CDS             119832..121319
                     /gene="thrC"
                     /locus_tag="OCPHGMPK_01044"
```

/EC_number="4.2.3.1"
/inference="ab initio prediction:Prodigal:2.6"
/inference="similar to AA sequence:UniProtKB:P23669"
/codon_start=1
/transl_table=11
/product="Threonine synthase"

/translation="MKYRSTRANLNYQLDAPAAIIQGLSPEGGLFVPTTFPKPIFDLE

QFKNMSYQEMAKKVLATFFDDFSDDQLSQIVDEAYGNQWDDKEIVKIAKHANNYYLEL

FHGPTLAFKDIALQILPRLMHTAIKIKQNDSDIIILTATSGDTGGAAMSGFKGQEHTH

VIVFYPYGGVSPVQLRQMLGLKGDNLTAIAIKGNFDDAQTNVKKIFNNQALVKKLKLN

KLQFSSANSMNIGRLVPQIVYYFYSYVQLVKNGEITAGQEINFSVPTGNFGDILAGYY

AKKLGLPIHKLICASDQNNVLTDFFKTGTYDRQRKFYLTNSPAMDILVSSNLERLLFD

LYNEDSNELAQLMNELSTKGKYTVSPIVKEKLDQLFAAGFATEDEVEDEINRIFIADQ

YTLDPHTAVASFVTKKYQENSLDQAPTVIVSTASPYKFPETVFHALTGKEVKEKGVPA
IKELNSFLQEDLSGNVNNLIKTGNYQEQIIKASEMENTLLNILDI"
CDS complement(121350..122408)
/gene="asd"
/locus_tag="OCPHGMPK_01045"
/EC_number="1.2.1.11"
/inference="ab initio prediction:Prodigal:2.6"
/inference="similar to AA sequence:UniProtKB:Q04797"
/codon_start=1
/transl_table=11
/product="Aspartate-semialdehyde dehydrogenase"

/translation="MKKEFTVAILGATGLVGQRMIKQLEQSTIPVKKVKFLASKRSAG

KKLKFHNQEVEVEETKPESFSGVDIILSSAGGAVSKKFLPEAVKRGSVCIDNTSAFRM

DPDVPLVVPEVNGEEIKNHHGIIANPNCSTIQMVMALKPMMDKFGLKRVIVSTYQAAS

GAGHAALEELFKEAKDYMAGRDIKEDAQVLPTAGDKKHYPLAFNLLPQIDVFEDDGYS

HEEWKMIHETKKILLNDMNAADIKVTATCVRVPVEIAHGESVYFELADKNATVSDLEN

VLKDFPGVQVQNDFRKQEYPQPLNAVGSNDTFVGRIRPDLETPGAFNMWVVADNLLKG
AAGNTVQIAEKLVANDWL"
CDS 122497..123852
/gene="yclM"
/locus_tag="OCPHGMPK_01046"
/EC_number="2.7.2.4"
/inference="ab initio prediction:Prodigal:2.6"
/inference="similar to AA sequence:UniProtKB:P94417"
/codon_start=1
/transl_table=11
/product="Aspartokinase 3"

/translation="MKVAKFGGSSVASGEKIAQVINILNNDNKRQVVVVSAPGKRNKT

DTKVTDLLIKYANLTLRKQNCEKIQAQIYSRYAEIGEYFNLSMQGLNEIKALINDLPT

HTFPNRDYLMAAFKAHGEFLNAKLLTMIMKKQGFKVRLLTPRQAGLVVTGSPNDASVN

PETYVNLRNLELDSAEKIIFPGFYGISPSGNIATFSRGGSDITGAILARGFNAELYEN

FTDVDAIFSANPAIVSHPEPISKMTYREMRELSYAGFSVFHDEALIPAIQAQIPVNVK

NTQHPERPGTMIVPETNFKAQHTITGIAGGKNFAALYLHKYLLNKGAGFTLKILQILD

QHKVAYEHMPSGIDDITIIFKKEDLNAELIDQICNEIQVSLNPDQMQWIDDYAITMVV

GEGMRDKLSLCASILYPLGQKNISIQMINQGASQISIMIGTRRCDAEEVIRTIYQTFF
N"

```
     CDS             complement(123910..125853)
                     /gene="pepO_3"
                     /locus_tag="OCPHGMPK_01047"
                     /EC_number="3.4.24.-"
                     /inference="ab initio prediction:Prodigal:2.6"
                     /inference="similar to AA sequence:UniProtKB:Q07744"
                     /codon_start=1
                     /transl_table=11
                     /product="Neutral endopeptidase"
```

/translation="MRRYFSVRGGAGDLTKPDVNTRPQDNLYLAVNSEWLSKAKIPAD

RTSTGINLILDMRIEDKLMKDFAEFANGKKLLPDVANFEKAINYYKLAADFDKRNKDQ

AEPIKKDLSRLTSLKNFEEFNQKAAQLISSGYELPFDIYVSEDMKDTDHNALYATGPQ

LFLPDTTAYQVPDAEKLLAVLEKQTVKLLTMAGISEEQAKTWAKNGIEFDKKLSKILK

STEEWADEVAMYNPASLSDFEAKFDQFDINSFLKQLLPEMPEKVIVAEPRYFNHINDF

LSESEFDEFKSWMILKFINKSATYLSQDFREAAFPFRQAVYGVPELPSQDKHAYRLAN

AAFDEVIGIYYGKTYLGDEAKADVISMIKKMLKVYEQRIQENTWLSEPTKKQAIIKLK

ALKLKVGYPEKNRALFDNLVVDPNKTLYENHVLINQERIKDILQRLNKKPDKSIWEMP

GNLNNACYDPYKNDLTFPAGILQTPFYDAKQSRAANYGGISATIGHEVSHAFDNNGAQ

FDEKGNMKNWWTKEDFAEFNKRTKAVADIFDGLQYGPVKLNGKQVVSENIADLSGLSC

AIAANKAEGGEMKDLFKTYAKSWMQKQRPESIKAEVQSDVHAPQPTRVNIPAQNQEEF
YEAYNVTPEDGMWLDPENRITIW"

```
     CDS             complement(125911..126180)
                     /locus_tag="OCPHGMPK_01048"
                     /inference="ab initio prediction:Prodigal:2.6"
                     /codon_start=1
                     /transl_table=11
```

/product="hypothetical protein"

/translation="MENLDSERSLYIEAITQEVSKILAKEEKIPLENAEHNFIHSRTY
NYLAYSNDPFIEDGPEDFVDLYHNEQKYHRLVSTTQLLVEQENKN"
CDS complement(126337..127860)
/locus_tag="OCPHGMPK_01049"
/inference="ab initio prediction:Prodigal:2.6"
/codon_start=1
/transl_table=11
/product="hypothetical protein"

/translation="MKLTKYTTIPFLAAILAGVAVEATPHQQVDIVQASTTKNQGASK

SEKPGKPGGKKTGTPPSGNPPQKPNGQTGKGGPNTQSYDYSGTLKATVNVKTGSKTVG

KKTISNSQSDQNTALVQKNGKLTLDGTTLKKTGASANDDNANFYGIDSVILANGKNAV

ATVNKAKILSNATGANGIFATNSGTVNVKNSTITTTGKANSRGLDATYGGKINADQIK

ISTQGDHSAGVATDRGGGVIKVKNSKITTSGSGSPLVYSTGNISLKNVKGTASGSQIA

GMEGYNKIFLNNSKLTSTSNKISGSDPIKNGVIIYQSTSGDADTSSGKSATFKAKDST

LKTKISSGAMFYVTNTTGKITLENTKLDFNSQKVNLLNVTGNKSNNWGTKGKNGGHIT

LTATKQKLSGNITVDSISSANVKLLKNSVYTGKTSIVANSYASSKAKTPLTMNISNSS

KWVVTGNSTVTNLSVAKGGKVVDAKGRKVTIVENGKTVQKGSSPYKITVKGAFSEKN"
CDS complement(128070..128444)
/locus_tag="OCPHGMPK_01050"
/inference="ab initio prediction:Prodigal:2.6"
/codon_start=1
/transl_table=11
/product="hypothetical protein"

/translation="MNNQDLLVNLIGGNIPHVGGVVTFDQKAKEETVVKFASHDGRLH

KDIFLAQRLAEKIEKKLPGNLVINAGVHIDGISKEQIDDSFVMTDEIAQKILAWVQEE
TSSFKAPKYTTHLKRDASGKLI"
CDS complement(128501..129064)
/gene="ecdB"
/locus_tag="OCPHGMPK_01051"
/EC_number="2.5.1.129"
/inference="ab initio prediction:Prodigal:2.6"
/inference="similar to AA sequence:UniProtKB:P69772"
/codon_start=1
/transl_table=11
/product="putative UbiX-like flavin
prenyltransferase"

/translation="MKKIIVGISGASGSIYGIDLLEKLSKATNVETHLVMSNWAKENI

KLETDYSVAEVENLADFVYDNRNLGAAIASGSFRIDGMVIAPASMKTVAGISIGFDED

LIMRAASVMLKEQRKLILVPRETPLSAIHLENLTKLAKLGVHIIPPIPSFYDHPKTIQ
DIIDHQTMKVLDSLDIEADFAKRWDGE"
CDS complement(129077..130561)
/gene="bsdC"
/locus_tag="OCPHGMPK_01052"
/EC_number="4.1.1.-"
/inference="ab initio prediction:Prodigal:2.6"
/inference="similar to AA sequence:UniProtKB:P94405"
/codon_start=1
/transl_table=11
/product="Phenolic acid decarboxylase subunit C"

/translation="MTNLPKDPWDLRKVLREIKDNPEDYHETDIEIDPDAEISGVYRY

VGAGGTVERPTKEGPAMMFNNLKGFPNTRVLIGLMASRKRDGRILHHDYKTLGRFLKD

AVENPVHPIKVDKKDAPAQEVVHLASDPDFDIRKLVPAPTNTEYDAGPYITCGLVKGS

NPDKTMTDVTIHRMVLEDKDTLGMYIMPGGRHIGHFQKEFEKLNKPMPITINIGLDPA

VTIGATFEPPTTPLGYDELDVAGALRQEPVQLVDGVSVDESALARSEYVLEGYIMPNE

TIKEDINTDSGFAMPEFPGYNGPANPAVNVVKITAVTHRKDNPIMQSVIGPSEEHVSM

AGIPTEASILELVDKAIPGKVVNVYNPPAGGGKLMTIMQIHKDNPADEGIQRQAAILA

FGAFKELKTVWLVDEDVDIFDMNDVVWTMNTRFQADQDFVCIPGMRNHPLDPSERPQY
DPKSIRVKGMSSKTIVDGTVPFDMKDQFIRAKFKEIPDWEKYLK"
CDS complement(130626..131522)
/gene="gltC_2"
/locus_tag="OCPHGMPK_01053"
/inference="ab initio prediction:Prodigal:2.6"
/inference="similar to AA sequence:UniProtKB:P20668"
/codon_start=1
/transl_table=11
/product="HTH-type transcriptional regulator GltC"

/translation="MEINKLRAFVDLAKTLSFSETAENLYTSQSTISKQIKSLEKELG

QELFERNNKNVALSEYGKLILPNAEKIVLLSDWISKETSEFKNNNSGQIRLGIIPTFA

NYDIFKQTMVYQKLKPDISLALQEIETNQLINTLKQKQVDAAFIRTMMPEKLDFEKII

IAQESFTVCLNKEHPFANKNEIKLSDLKDETFIMLAKNSLLYEPVINLCRKAGFDPKI

SFVSDRMSSILQMVENKQGVAILMHPQTKKSEVSFVSIKPTLTSTLLFIRNKGNHSKI
ENDFWNYLKQFEMPNWNNCDNR"
CDS 131635..132366
/gene="ydfK"
/locus_tag="OCPHGMPK_01054"
/inference="ab initio prediction:Prodigal:2.6"
/inference="similar to AA sequence:UniProtKB:P96689"
/codon_start=1

```
                     /transl_table=11
                     /product="putative membrane protein YdfK"

/translation="MPIGVIVNASSVLLGGIFGGFFGNKLSEDFKEKLNMIFGACSMG

MGIFSIAPMKYMPAVIFAVILGTIVGLAIHLGKIFNQGALLMQRPITRMIPTENLSIS

RDEFMSLMVTATVLFCASGTGIYGSLDEGMSGDITILVSKSILDFFTAAIFAASLGYA

VSIIAIPQFIIFFILFLLARFILPFTTPSMISDFKACGGFLMLATGFRMVKLKMFPIA
                     EMIPAMILVMPLSWLWSDVILKLIH"
     CDS             complement(132411..134981)
                     /locus_tag="OCPHGMPK_01055"
                     /inference="ab initio prediction:Prodigal:2.6"
                     /codon_start=1
                     /transl_table=11
                     /product="hypothetical protein"

/translation="MSKKVLWKDAVNSLTNSWGRFIGIMLLMAVSAFAFIGLKMAGPD

MRYTAQRYYRNINLADLTVSSNYGLDNRDLQRLRNHAKNATLDLGYLQDTTVNNSKIS

LRVLSETKSVSTNKIVSGKLPNKNNQIAISYLLKDKYRLGEWIKLDNHNNLKTNRFKI

VGFIRPSEYTDRSNIGQTTVGTGQLSGIAVVKKSAFNSSNYSIARLRFNNTKNLDPYS

SDYQRLVDDKKKILTKALDKNGRTKEIERDKNFASAQNQLDQAKQQVQLAENNGVNVA

KQKAQLEKQESKLNKQKRQIKQLGNVSYYVNDRKNDPGYDTYQSNSEKIQLITDIFPV

FLFAVAALVSFSTMTRFIEEERQNIGVLRALGYSKSDTSLKFIVYSLTAALTGVLIGA

IGGYWLLPQIIFNSYTANLTLTNFQTLFSWKYLFLTIFIAVLCTTGAALIQLFFVLRA

KTSELLLPKPPKNGSRIFLERIKPLWQRLGFNYKVTLRNIFRYKVKMIMTILGIAGCT

GLLMMGFGIRDSLAGIGQKQYSELIKYDLIAVDKKDLSGNQSRKLEQKLDSKQVNKYL

PVHFENITKKIDGTNQDISIIVPKEDKNINNYINLRNRQSGQKINLNNHGVVISEKLA

NLLNLKSGDRLTLNTNNGKKVKLPISRICEMYMGHYVLMNSDIYEKYFDQKMSANAQL

IELKSGVKTDDFANSLMKTEAVNAINLNTSNKQIIDSLIQSMDKVMFLLIGLAALLAI

VVIFTLTTTNLEERMREISTLKVLGFYNKEASLYIYRETIILSIFGILFGFLIGNWLH

SFIIDNLAPMNAMFRPGILMSNYLLSAIIPLAITAIMAIFVNRKIKDVNMLEALKSLD
                     "
     CDS             complement(134991..135692)
                     /locus_tag="OCPHGMPK_01056"
                     /inference="ab initio prediction:Prodigal:2.6"
                     /inference="similar to AA sequence:UniProtKB:P9WQK1"
                     /codon_start=1
                     /transl_table=11
```

```
                     /product="putative ABC transporter ATP-binding
protein"

/translation="MAFIELRNCSKKYGTGATTVWANKDLSFDIEQGELVIILGSSGA

GKSTLLNILGGMENVTKGEVIVNGNNIANYSAKELTKYRRNKIGFVFQFYNLIPNLTA

KENVELANELVANPLDAEVALKEVGLEKRINNFPSQLSGGEQQRVAIARAIAKNPALL

LCDEPTGALDYQTGKKVLKILQDRSLKKNSTVIIVTHNAALAPIADKVIRIQDGKIKQ
                     VENNAHPQDIEKIEW"
     CDS             complement(135798..135956)
                     /locus_tag="OCPHGMPK_01057"
                     /inference="ab initio prediction:Prodigal:2.6"
                     /codon_start=1
                     /transl_table=11
                     /product="hypothetical protein"

/translation="MKSRDNLSKLITFLVLCGFLILAVKRGNIIESLIFSVLIFIFIA
                     VKFIFNKK"
     CDS             complement(135953..136624)
                     /locus_tag="OCPHGMPK_01058"
                     /inference="ab initio prediction:Prodigal:2.6"
                     /codon_start=1
                     /transl_table=11
                     /product="hypothetical protein"

/translation="MKEISKYQQLRKKLIENSNPEIAVQMESYMRNKFKFYGLKSPER

RKSYHDLIKLEKANKKIDWDFLDQAWADEHREAQYFVCDYLIALEKYLTFEDIDHIFN

YVKSKQWWDTIDSLIKPIGNIGLRDDRVTDLMLAWSKDDDFWVRRVAIEHQLLHKDKM

NVKLLNAILENNLGSSEFFINKAIGWALRDYSKTNPDWVKNFISKHHAEMASLSIKEG
                     SKYLS"
     CDS             136783..139521
                     /gene="ppc"
                     /locus_tag="OCPHGMPK_01059"
                     /EC_number="4.1.1.31"
                     /inference="ab initio prediction:Prodigal:2.6"
                     /inference="similar to AA sequence:UniProtKB:A0QWX4"
                     /codon_start=1
                     /transl_table=11
                     /product="Phosphoenolpyruvate carboxylase"

/translation="MSIKKLENSSDHAMVAEEVKILTNLLNESTRQLSGDVVFNKIQD

LIKISAQKDYDALEKQIASLTNQEMMVVARYFATLPLLVNISEDVELASEVNLLNNTD

QDYLGKLEDTIDLVAQKENAREILKHVNVVPVLTAHPTQVQRKTVLELTDQIHGLLRS

YREVKNGTINQAEWTEKLRAYIEILMQTDIIRSHKLQVSNEITNVLAYYPKALIPAIT

KFTARYQELAKKHNLDLKGATPITMGMWIGGDRDGNPYVTADTLKLSAGLQSQVIFEY
```

YIKKLNKLYRTISMSTSYMKTSSAVEKLSELSNDDSPFRTNEPYRRAFYYIESRLLHT

EYQLLGTADKNSFVKKRDLENLDKIPVYKNPQDFKADLITIKESLEQDHDQAVIKSFF

TELLEAIDIFGFHLATIDMRQDSSVNEACVAELLKSAGICDNYSDLPEKEKVQVLLKE

LNDDPRNLHANNKPKSDLLQKELKIYKTARQLKDRIGEDVIKQHIISHTESVSDLLEQ

AIMLKEYDLLDNQSARIQVVPLFETVEDLENSREIMKEFLNLDIVKKWLASQNNYQEI

MLGYSDSNKDGGYLASCWNLYKAQKDLTAMGKKLGVNITFMHGRGGTVGRGGGPSYEA

ITAQPFGSINDRIRMTEQGEIIQNKYGNQDTAYYNLEMLASATIDRIVSKQIVSEDDI

GGFRDSMDKIVLDSNKVYRKLVFETPAFLDYFLQATPIKQISNLNIGSRPAARKKITD

FSGLRAIPWVFSWSQSRIMFPGWYGVGSAFKHFIDADSHNLETLQKMYQGWPFFHSLL

SNVDMVLSKSNMDIAKQYADLCEDEDTKQVFDTIYQEWELTKEVILQIEGNKELLADN

PSLQMSLNYRMPYFNILNYIQIEMIKRDRVDDIAGVYESILPITINGVTSGLRNSG"

```
     CDS             139552..140097
                     /gene="pncA"
                     /locus_tag="OCPHGMPK_01060"
                     /EC_number="3.5.1.19"
                     /inference="ab initio prediction:Prodigal:2.6"
                     /inference="similar to AA sequence:UniProtKB:I6XD65"
                     /codon_start=1
                     /transl_table=11
                     /product="Nicotinamidase/pyrazinamidase"
```

/translation="MTKALLIIDYTNDFIADNGSLTCGKPAQDLEDYLIELADKFYEN

GDYVIFPTDGHTGDKFSPEYKLFPPHNIVGTPGQELYGKLKDWYESHKSSERIYKFNK

NRYSSFQNTNLDNYLRERKIDDLWLTGVCTDICVLHTAVSAYNLNYGITIPTKGVTTF

```
                     TEHGQEWALDHFKNSLGAKVI"
     CDS             complement(140211..140747)
                     /gene="pyrR_1"
                     /locus_tag="OCPHGMPK_01061"
                     /inference="ab initio prediction:Prodigal:2.6"
                     /inference="similar to AA sequence:UniProtKB:F2MMP6"
                     /codon_start=1
                     /transl_table=11
                     /product="Bifunctional protein pyrR"
```

/translation="MAKEIWDKLAMKRALVRITYEIIERNKGVEDLVLVGIKTRGVYL

ARRMRDRLKEVEKVDVPVGELDITPYRDDRKNDFSQEKIPDLEPMDLDITNKNVVLVD

DVLYTGRTIRAAMDALIANGRPKSISVAVLVDRGHRELPIKADFVGKNIPTSLNEQVS

```
                     VNVSEIDGVDSIELINLK"
     CDS             complement(140775..143963)
```

/gene="carB_2"
/locus_tag="OCPHGMPK_01062"
/EC_number="6.3.5.5"
/inference="ab initio prediction:Prodigal:2.6"
/inference="similar to AA sequence:UniProtKB:O32771"
/codon_start=1
/transl_table=11
/product="Carbamoyl-phosphate synthase large chain"

/translation="MPKRTDIHKIMVIGSGPIIIGQAAEFDYSGTQACLALREEGYEV

VLVNSNPATIMTDTTIADKVYIEPLTVESISRIIRQEYPDAILPTLGGQVGLNMALAL

AKTGILDELNIELLGTKLSSIEQAEDREKFKELCKKLGEPVPPSRTVNTVEDALAFGD

EIGYPIIVRPAFTMGGTGGGICHSHEELAEIAKNGLELSPVTECLIEKSIAGYKEIEF

EVMRDHDDNAMIVCCMENFDPVGIHTGDSIVFSPSQTLSDKEYQMLRDCSLRLIRALK

IEGGCNVQLALDPNSFDYDVIEVNPRVSRSSALASKATGYPIAKMAAKIAIGMTLDEI

KNPVTGTTYAEFEPALDYVVCKIPRWPFDKFSKADRTLGTQMKATGEVMAIGRTAEEA

MQKAVRSLEIDEKDLYSEKAHRASDEEIEQKLVRAQDDRLFYLAEAFRRGYSLEDVHE

LTKINFYFLDIVSHMVEMEKTIKENKDDLETLRLAKKYGFSDPTIAALWDEEADQVRD

LRKKQNIIPVYKMVDTCAAEFESKTPYFYSTYDGENESHKSGKKSVIVIGSGPIRIGQ

GVEFDYATVHCVKALQKMGYEAIVINSNPETVSTDFSISDKLYFEPLTLEDVLNVCDL

EKPEGVIVQFGGQTSINLAAGLEKHGVKILGTTVKDLNRAEDRELFDQIIKKLKLNQP

KGLTATTHDGVIKAAEELGYPVLVRPSYVLGGKAMEIVYNKSELEEYLHDHVDIAADH

PILVDDYLDGRECDVDAICDGKDVLLPGIMEHIEHAGVHSGDSMAVYPPQTFTDDVKE

KIMDVTRKLALTLNCVGIMNIQFIVRNGEVYVIEVNPRASRTVPFLSKITGIEMAQVA

TRVIMGESLAEQGYSDGLAPEPEMISVKAPVFSFSKLADVDSYLGPEMKSTGEVMGSD

HTFAKALYKAFAGAKMQLPENGNVLLTIEDRDKDKILPIAKRFARIGYRIFATKGTAD

FLKNNGLHVDLVTKVHENENADDNILNELRDGKIDLVINTMGHDIEKNSDGFIIRQMA
IQQNVPLLTALDTADALLTSLENRSFATDALK"
CDS complement(143956..145044)
/gene="carA_2"
/locus_tag="OCPHGMPK_01063"
/EC_number="6.3.5.5"
/inference="ab initio prediction:Prodigal:2.6"
/inference="similar to AA sequence:UniProtKB:P99147"
/codon_start=1
/transl_table=11
/product="Carbamoyl-phosphate synthase small chain"

/translation="MKRYLILEDGSIYEGEAFGADCESSGEVVFTTGMTGYQEAITDQ

SYADQILVFTNPLIGNYGITLADYESLEPQIKGVVCHQVARHPDNWRMQTTLPKFLER

LNIPGLQGIDTRELVKKLRIHGTLKGKITDSKEDATKIAQELKEQNITQGVISRVSTK

NSYPVPGSKRNIVVVDFGIKHSILRELAERDCNCIVLPYTASADEILSLNPDGVLLSN

GPGDPEEMVEAAKMVQEVEKHVPLFGICMGHQVFALANGAKTYKMKFGHRGFNHPVRE

IATGNIGFTSQNHGYAVDPASIDKENLMITHVEVNDGTVEGLRHKKYPAFSVQFHPDA
TPGPHDEDSLFDDFMSMIDQRKEEERHA"

CDS complement(145041..146318)
/gene="pyrC"
/locus_tag="OCPHGMPK_01064"
/EC_number="3.5.2.3"
/inference="ab initio prediction:Prodigal:2.6"
/inference="similar to AA sequence:UniProtKB:P65906"
/codon_start=1
/transl_table=11
/product="Dihydroorotase"

/translation="MATVIKNGTVYQNGRLIKADVLIEGKKIKAIGIDLDAENVIDAQ

GMLVSPGLVDVHVHYRDPGQTYKEDIKTGSQAAARGGFTTVGAMPNVTPVPNTPELMK

KMVEENQSKGVVHIFQYGPITNDETTDIIPDYAALKKAGAFALSNDGHGVQTAQTMYL

AMQKAKENNLIIATHAQDDSLFNKGIVNEGITAERLDLPPVTELAETTQIARDLLLAQ

KTGVHYHICHVSTKTSVELVRLAKARGINVTCEVAPHHILLTDNDIPKDNGYFKMNPP

LRNKEDQAALLVGLLDGTIDLIATDHAPHAKKEKQVGMKDAAFGITGSETAFSTLYTK

FVKEEKVLRLEQLLALLSDKPAKVFGIKNAGVLEPGKNADIAIFDIEHKTEIKEADFK
SKGVNTPFTGQKVYGETVMTLVDGEVVYQRGTK"

CDS complement(146318..147274)
/gene="pyrB"
/locus_tag="OCPHGMPK_01065"
/EC_number="2.1.3.2"
/inference="ab initio prediction:Prodigal:2.6"
/inference="similar to AA sequence:UniProtKB:P05654"
/codon_start=1
/transl_table=11
/product="Aspartate carbamoyltransferase"

/translation="MEKLDLVSLPHFVSVENLSVEEVEALIKRAEYFKNGGATPRLTQ

PVYISNMFFEDSSRTHTSFEMAERKLGLTVIPFDPAHSSVNKGETLYDTSLIMDAVGV

NIEVIRHSQNEYYQDLIHPKKNQYLNIGVINAGDGSGQHPSQCLLDMMTIHEHFGHFK

GLKVAIVGDITNSRVAKSNMELLTRLGAEVYFSGPEYWYSSEYDKYGKYEKLDKLVPE

```
MDVMMLLRVQHERHSGDPNEKSFDAKAYHEEYGINHNRYQELKPDTIIMHPGPINHDV
                     ELSGDLVESDKCMFTRQMQNGVFMRMAMIEAVLRGRRLGGLK"
     CDS             complement(147421..147963)
                     /gene="pyrR_2"
                     /locus_tag="OCPHGMPK_01066"
                     /inference="ab initio prediction:Prodigal:2.6"
                     /inference="similar to AA sequence:UniProtKB:F2MMP6"
                     /codon_start=1
                     /transl_table=11
                     /product="Bifunctional protein pyrR"

/translation="MAKEIWDALAMKRALTRITYEIIEQNKGTDNLVLIGIKTRGVYL

AKRIHDRIQKLEDVDVPVDELDITLYRDDRHDASLKQDPVINSNKISVDINDKKVILI

DDVIYTGRTIRAAMDALMDDGRPSSIKVAVLVDRGHRELPIRADFVGKNIPTATDEQV
                     AVNMVEKDGKDSVELKSLPK"
     CDS             complement(148133..149056)
                     /gene="pyrDB"
                     /locus_tag="OCPHGMPK_01067"
                     /EC_number="1.3.1.14"
                     /inference="ab initio prediction:Prodigal:2.6"
                     /inference="similar to AA sequence:UniProtKB:P54322"
                     /codon_start=1
                     /transl_table=11
                     /product="Dihydroorotate dehydrogenase B (NAD(+)),
                     catalytic subunit"

/translation="MINTHVKLPGLDLKNPVMPASGTFGFGDVPAAKKFDLNDLGAMV

IKTTTPHATTGNPQPQIAVLDTGVLNSVGLTNPGVDAVIKDKLAPLRQSYPALPIMAS

VGGEDEAGYVEVAQKLSDSGLVNALEINVSCPNVNQGGMSFGVHPDVVESLTKKIKAV

TNVPIYVKLTPNVTDIIQIAKAAEKGGADGLSLINTLLGMDIDIKTRKPVLGHNVGGL

SGEAVKPVALRMVHQVRQSTSLPIIGMGGISSAQDVIKFMLAGANAVAVGTAHFKDSI
                     ASKHIADELPNELEKLGIENINDLVNQVEFN"
     CDS             149308..150015
                     /gene="pyrF"
                     /locus_tag="OCPHGMPK_01068"
                     /EC_number="4.1.1.23"
                     /inference="ab initio prediction:Prodigal:2.6"
                     /inference="similar to AA sequence:UniProtKB:Q5FJB3"
                     /codon_start=1
                     /transl_table=11
                     /product="Orotidine 5'-phosphate decarboxylase"

/translation="MTRPVIVALDLDNEKKLNALLPKLGKPENVFIKIGMELFFNEGP

AIVRNLSEQGYQIFLDLKMSDIPNTVYNGAKALAQLGITYTTVHALGGSQMIKAAKEG

LIAGTPVGKNIPKLLAVTELTSISDDILHEEQNCNLSMKQQVLSLAETAKKAGADGVI
```

```
CSPLEVKNLRQNVGDDFLYVTPGIRPAGNAKDDQSRVATPAQAKEWGSTAIVVGRPIT
                     LATNPEAAYEAIKKEFN"
     CDS             150017..150655
                     /gene="pyrE"
                     /locus_tag="OCPHGMPK_01069"
                     /EC_number="2.4.2.10"
                     /inference="ab initio prediction:Prodigal:2.6"
                     /inference="similar to AA sequence:UniProtKB:P0CB78"
                     /codon_start=1
                     /transl_table=11
                     /product="Orotate phosphoribosyltransferase"

/translation="MHKDQIISQLIQEKIITISPDKPFTYASGMLSPIYTDLRLTVSY

PELRDMIASDLANLIAKEFPEATIVGGVATAGIPHAALVAEKLHLPMIYVRPKPKDHG

KGRQIEGRFSENDKIVLIDDLITTGGSVLNAVKATEKDGGHVAGVSSIFTYYLPDAKE

DFKKANVKYAPLLSYPELLKKENESGHITSEQYDILKTWHEDPWAWGKKFNN"
     CDS             150905..152500
                     /locus_tag="OCPHGMPK_01070"
                     /inference="ab initio prediction:Prodigal:2.6"
                     /codon_start=1
                     /transl_table=11
                     /product="hypothetical protein"

/translation="MDTNNILSYIKWRGDISLSARPFDEVDALVIATFSYIHLDGIVP

DSNKEVSIKDVAQKYFNSSNQNLDHYKYQDLLKLMANSVRFGDAKLSYFADVLTDRIQ

FSAIKISLDNDTNFISFRGTDDTLVGWKEDFEISFRTTGAQKYALKYLTNILKTTNQV

YSLAGHSKGGNLAEYAAVNLPDDLKENIDTIYTFDSPGLSTQVDGVTDKLKRYVPEFS

IIGRLFEPENITPTILVSDRSKLAQHDPMSWEVSGSHFITRNHRNPTSKIYNQIINQW

IGEANLQEREALTNDLFSAFAASGATKITELNKNGFGGFGAILFSLTDSSRRTRFVLG

SLWETIWRSIKATHLEKLFINTNSIIGWVLILLGIVDLTIPDYAYRAFGGLVALFCVG

WSGYHIVETANSHLLPKPKRFFIISYLIVFGLAIAIISNNRLLNFLAHYVLGIFLIGF

AYVRLRSVIVKNKKNGIFRNIIDVLESLIAFAAGIIVIINPNYFSRQAVISLGILLII
                     YGFFQLVMELFKQRKLRMPSKHR"
     CDS             complement(152541..153362)
                     /locus_tag="OCPHGMPK_01071"
                     /EC_number="3.1.3.-"
                     /inference="ab initio prediction:Prodigal:2.6"
                     /inference="similar to AA sequence:UniProtKB:Q5XD45"
                     /codon_start=1
                     /transl_table=11
                     /product="Putative phosphatase"
```

```
/translation="MNEIKVIAIDVDGTLLNSQKQLTRGVKNAILMARKAGIKIVIAT

GRPLSGVKQILHELNLANQNDEYVVCFGGGVVETTSGNVLFEKKLTYDNYLDLETIAL

KLGLHFHASAPDRIYTADRDIGDFTLYEANLVNLGISYRTPAEMKDVPIIKCMYVDEQ

KYLDKKIADHTLFAHLDDKITFTKTAPFYYEANPKGVSKGNALKILCQKLNLTNENIM

AIGDEENDLSMIKYAGIGVAMENAVTSVKDNAQMVTKDCDHDGVAAVIEKVLQEK"
     CDS             complement(153367..154395)
                     /locus_tag="OCPHGMPK_01072"
                     /inference="ab initio prediction:Prodigal:2.6"
                     /codon_start=1
                     /transl_table=11
                     /product="hypothetical protein"

/translation="MILSFSIVLILFLLALATFVWVLTHERRSLWSGMTLVWTIITFG

LLTAVSLMIAAEVFPITHQFVLLVFAVILIALMVLVFAFIAVLIVMFIYNGIKILIRE

GNRWTNFLSLAMGIGIIAYLFLFPVVGRLTRNNFGTYLYLFVNLTIIYLIFIMMMYTL

TSWINLINFKIKKLNYIVVLGAGLIGKKVTPLLAARINRGIEVYRNNPGSKIIMSGGQ

GPGEEIPEAAAMAKYAEEKGISKQDIIVEDKSKTTRQNLAFSHKLMKADSRFAIVTNS

YHVYRALVLAKRMGLDCIGYGAKTKWYFTLNAFVREFIAYLTITWRLQLSVVGCIGLV
                     VLLGALLR"
     CDS             154525..154851
                     /locus_tag="OCPHGMPK_01073"
                     /inference="ab initio prediction:Prodigal:2.6"
                     /codon_start=1
                     /transl_table=11
                     /product="hypothetical protein"

/translation="MTEQLLVTTTENIPNQKYEIIGEVFGVTTQSKNAIRDFGAGLKS

IVGGEIKAYTSMLTESRDQAINRLRQNAADLGADAVVMMRFDSGSIAGDMQSVVAYGT
                     AVKFID"
     CDS             complement(154881..156272)
                     /gene="rsmF"
                     /locus_tag="OCPHGMPK_01074"
                     /EC_number="2.1.1.-"
                     /inference="ab initio prediction:Prodigal:2.6"
                     /inference="similar to AA sequence:UniProtKB:Q5SII2"
                     /codon_start=1
                     /transl_table=11
                     /product="Ribosomal RNA small subunit
methyltransferase F"

/translation="MSLPVEFIKKYQNLLGEEESNKFFAALDSSSKKGFRLNPLKNNY

HDVQYSLADPIEFTSDGYYGGISGRDSEWTSGYVYSQDPSAMYPAVALNVKPGEKVLD
```

LCAAPGGKSTALASFLENKGLLVANEISKSRAKDLRENLERWGATNVVVTNESPERLS

KKLPHFFDKILVDAPCSGEGMFRKDPAAMQYWSPEYVLTCQARQKEILTQAMKMLKSG

GELVYSTCTYSPEEDEEIVAWLVENYHLKIEPLKLYPGMSMGKSDWSKSNLPELKDTV

RLWFQNGVGEGQFIAHLRSTEAVEDASGKVKKRKKKHQKKSSITRLTKDEITTVAKVL

DNFVLPDSLENWQKTVLQSNGHVFVPAVDPELLNELHILNNGVELGLLKKKRFEPSHQ

LAEVLGQKEQKQLLNLKNETDYEHYLHGETIKVNSELRGFVLVAYQNHIFSFGKIGND
QILKNFYPKGLRK"
CDS complement(156272..157249)
/locus_tag="OCPHGMPK_01075"
/inference="ab initio prediction:Prodigal:2.6"
/codon_start=1
/transl_table=11
/product="hypothetical protein"

/translation="MKIKRLILLIISFVAFLGLGMILSRQQSQRADQVLNNNGLSSTY

YVFKSKKKHNIKQLLNYVNKNFAHDKVQIHFESDYIPDRVLIWANYNLKSQPLAKGSS

RYFTKSDFDGSIPFAVISADTKSKVVTLQNNHYLKENNDYVTIIGELKRNNESQNGQA

VYYLSTGINQPTAKESITNYKIVVDGLNKNEANSMHAYLKGNMQTKDFSKTYNRQHGI

SPTKKLIAAILCVVIALVVAAIAAVLAENPVNLKFVPGKIWAHLLADSGLRFFLANGI
VVLLTSLGLQTWKFYSNHSQLYILFLVVFAMQIITYVLMLLAIKIKKEK"
CDS complement(157249..157977)
/locus_tag="OCPHGMPK_01076"
/inference="ab initio prediction:Prodigal:2.6"
/codon_start=1
/transl_table=11
/product="hypothetical protein"

/translation="MKQYQIKLNQLDRELNYLPLHRQVDIINNIIANIQQKKILPKSP

NSLGFLPDSLDIMIDKIGNKDKAQEANNLLNNFRSFLSREYGVWSLPNLETARLIKQE

YHVKSSLEIMAGNAYWSKALSQVGIKATASDSFSWAKSSTTGEAPIFATENLDALSAI

KKHPEVDLIICSWAPNFGEDDLKILDLYRSLDHQPVLLFIGEKNGATNSTSFWQKANC
RANTKINRSFQSFDFINEKVFEIK"
CDS 158068..158640
/gene="spsB_1"
/locus_tag="OCPHGMPK_01077"
/EC_number="3.4.21.89"
/inference="ab initio prediction:Prodigal:2.6"
/inference="similar to AA sequence:UniProtKB:P0A068"
/codon_start=1
/transl_table=11
/product="Signal peptidase IB"

```
/translation="MSKHKTESAESLGHWLLQVFILAIVIFGLYIFMFRVLLANETIT

GPSMQPTFENNDRIIAVRHSKLSRGDIVILRAPDQPGALYIKRIIGVPGDSIKSKNDV

MYINGKPIKEPYLTEYKKKLAKGQLYTNNFSLQQLYHVKRVPKDCYFVMGDHRNVSKD
                     SRMLGFIKKKDIVGEVKLRYFPFDQIQWFK"
     CDS             158668..158853
                     /locus_tag="OCPHGMPK_01078"
                     /inference="ab initio prediction:Prodigal:2.6"
                     /codon_start=1
                     /transl_table=11
                     /product="hypothetical protein"

/translation="MSPEELSDAIIEFEVKNNVNDTALAFSSHLSVEKLHAMKTGEAK
                     YTMEEAEQVLDYIQANS"
     CDS             complement(158925..159761)
                     /locus_tag="OCPHGMPK_01079"
                     /inference="ab initio prediction:Prodigal:2.6"
                     /codon_start=1
                     /transl_table=11
                     /product="hypothetical protein"

/translation="MTSFSSIFKELTKNKVRLVRRLVFLQIVVGILLGFWALFYSAFE

GESKAYFLISFITITPLFDVAYLFLSSRKNEQVYASQTWRLAPIKESTLLLANLFSAI

VDGLYLVVIQLAMILIAGLPIIGTNEFWKTTFKNFGSIGEGKLWQDIHITDLISVIVL

LILIGIFIYLAVSLINFTGKIVSDFLPEKISKLVYFLVVVSLTCIGLIFLINVYWFVM

DSVTGFLQNGMFLTTLGKDAIDVRSDLTGNYLTMAVVLAVDIILIAINVVLLRKYHEA
                     KI"
     CDS             complement(159771..160589)
                     /locus_tag="OCPHGMPK_01080"
                     /inference="ab initio prediction:Prodigal:2.6"
                     /codon_start=1
                     /transl_table=11
                     /product="hypothetical protein"

/translation="MTTFKALFNQMGKKKRRSVYLLALLQLVAASVFALWALFSNGGA

FSNQDKPIAWFVMVFTFAPLADMAYVVLSAWQNEKEYSSQTWRLVPVSSSKFYLANIL

SAIVNGLYLVLIQIGMGVVTFLPALISNDVRTNIWEINQAISKSSHAGDFWQKLSDSF

PIGEMLSALLAFLLFAILVYSIVTTLNLSSRTITDFLPDKHSKLIRFVIMIVLIFIFI

VLTNNLGSLLNQLRWGETSNGLVAFAEGNLMMALIDIILGSINIWLLKDFHEGK"
     CDS             complement(160592..161299)
                     /gene="natA_2"
                     /locus_tag="OCPHGMPK_01081"
                     /inference="ab initio prediction:Prodigal:2.6"
                     /inference="similar to AA sequence:UniProtKB:P46903"
```

/codon_start=1
/transl_table=11
/product="ABC transporter ATP-binding protein NatA"

/translation="MSKLLEVKGLTYRKNLKTILEDVNLDVDSGKIIALLGENGAGKT

TLMRVIVGIAKHYQGEVLLNGDGSEVYRKANIAMTDNLKGLSDSTKVGEVEEFYQKLF

VDFDSSKFEQLRSFMKLNNDMKLGQLSRGMREKLEIALTLSREAKLYLLDEPFSGIDP

MARKRIINSILLWKSPDSTIVISDHFVNEITTILDEVVIVKDKKIASHLSADDIRAKG
HSIEEYYESLYADEGAE"
CDS complement(161301..161669)
/gene="ytrA"
/locus_tag="OCPHGMPK_01082"
/inference="ab initio prediction:Prodigal:2.6"
/inference="similar to AA sequence:UniProtKB:O34712"
/codon_start=1
/transl_table=11
/product="HTH-type transcriptional repressor YtrA"

/translation="MQFQDNIPIYVQIEKYLYRQIALGKIKPGEKVPSVRQLAVDLTV

NVNTVQRALNQMNTEGILEVRRGLGSFVTTDTELILQKRRELIKDTMSEFLESTAQLG
LTPEKTLEELKKFIEEKKGD"
CDS complement(161983..163230)
/gene="pepT_1"
/locus_tag="OCPHGMPK_01083"
/EC_number="3.4.11.4"
/inference="ab initio prediction:Prodigal:2.6"
/inference="similar to AA sequence:UniProtKB:Q9L4G1"
/codon_start=1
/transl_table=11
/product="Peptidase T"

/translation="MTEYPNLLPRFLKYVKVNSRSDEHSDRFPSTEREENFQKNVIMK

DLEELGLKDVHYNQKSGCVIATIPSNIDYEVPTFGLLAHCDTADFNSVDVKPQITENY

DGKSKIQLGDSEFYLDPEVFPHLKNYQGQTIISASGDTLLGGDDKCGISELMTFAEYL

MEHPEEKHGEIKLAFTPDEEIGTGAEHFDVEEFGADFAYTVDGEAPGKLDWGTFSAAQ

FGLDIQGVNVHPAVAKGQMINAIQVGIDFHNQLPEHDRPEHTDGREGFFHLMNFDGTV

DSAHMDYIIRDFERDGLERRKNLVKDIVKKMNDEFGSERIKLKMWDQYYNMADELEKH

MEIVDLARDAYKAEGLTVNEDPVRGGTDGSQLTYMGLPCPNLFAGEENMHGRYEYTVL
ESMWKAVDVLRKINQLNVERNKK"
CDS complement(163241..164038)
/locus_tag="OCPHGMPK_01084"
/inference="ab initio prediction:Prodigal:2.6"
/inference="similar to AA sequence:UniProtKB:Q97PK0"
/codon_start=1

/transl_table=11
/product="GTP cyclohydrolase 1 type 2"

/translation="MTKVADIVNRLRADFPEEIASEGDPVGMQIGSMDAEVTKVMTTL

DVRPQVVDEAIEKGVDLIISHHPVMFRPAKNLDFSDPQNAMYGKLIANGITVYSIHTN

SDKAQNGSADWQAEELELRDVERFALDADGIAIGRKGKLPKSMTAKEFAYYVKDKMQI

PMARLITADNDKIISTVAFICGDGGKFWRQALIDGVDAFITGDVYYHVGHDMISAGLT
VVDPGHYTEKLFKYKVGERLEKWNKNYNWNVPVVVSEVSTNPFQDLF"
CDS complement(164035..164718)
/gene="trmK"
/locus_tag="OCPHGMPK_01085"
/EC_number="2.1.1.217"
/inference="ab initio prediction:Prodigal:2.6"
/inference="similar to AA sequence:UniProtKB:P54471"
/codon_start=1
/transl_table=11
/product="tRNA (adenine(22)-N(1))-methyltransferase"

/translation="MLEERLAQIGKMVDPESRLADIGTDHAYLPIALVKEGKIDFAIA

SDVAAGPLNNAKQDIEQAGLENKIETRLGSGLETLKAEDRIDTVVIAGMGGKLMTNLL

ETAYQEGKKYPTLILEANIGESLVRKWLMDHQYEIVAEKIIEVAGHIYEIIKAEIKTN

KHDLSEQELAFGPFLLKEKNPVFIKKWTNQLKYYQNLKANLNKAKNKDEDKINKINDL
IKMIEEVLK"
CDS complement(164842..165963)
/gene="sigA"
/locus_tag="OCPHGMPK_01086"
/inference="ab initio prediction:Prodigal:2.6"
/inference="similar to AA sequence:UniProtKB:P0A0J0"
/codon_start=1
/transl_table=11
/product="RNA polymerase sigma factor SigA"

/translation="MAESKTTKKEATMTLDKKVKEVVKEVKKDKQITEKEFTAQLIKP

YNLQGKAVDQLVQEFEDNGISIVDEKGEPSKLALKKQKDVEKAELKDMSAPSSVRMND

PVRMYLKEIGRVSLLNADQEIALAKRIEAGDEEAKQELAEANLRLVVSIAKRYVGRGM

SFLDLIQEGNMGLMKAVDKFDYRLGFKFSTYATWWIRQAITRAIADQARTIRIPVHMV

ETINKLIRIQRQLLQDLGREPTPEEIGAEMDMPTDKVREILKIAQEPVSLETPIGEED

DSHLGDFIEDKDATSPEQHASYELLKEQLEEVLDTLTDREENVLRLRFGLDDGRTRTL
EEVGKVFGVTRERIRQIEAKALRKLRHPSRSNQLKDFLD"
CDS complement(165979..167793)
/gene="dnaG"
/locus_tag="OCPHGMPK_01087"
/EC_number="2.7.7.-"

/inference="ab initio prediction:Prodigal:2.6"
/inference="similar to AA sequence:UniProtKB:Q9X4D0"
/codon_start=1
/transl_table=11
/product="DNA primase"

/translation="MAGRIPEQFIDEVRNSVDIVDVISQYVSLEKKGKDYLGLCPFHQ

EKTPSFTVSEGKQFFKCFGCGKGGNVFKFLMYQDHLTFPESVKKVAELAHLQMPEAYG

EAAKPLSPLKKMYRQATEFYQHILLTTRVGERALKYARKRELTDDLLKHFKVGYAPDS

DNILLTYLKQKDYTDDDLRESGLFVESQDGQLFDRFRDRLMFPLGDESGYTVGFSGRR

ISNDKTIAKYMNSPETKIFNKSKLLFHFAEAKKAARSEKHLILYEGYMDVIAAYKAGI

QSGVASMGTSLTTEQVYMLRRITPNILVNYDGDPPGIHAAERATKLFGQVQGFNLGII

VLPENLDPDEYVKKYGKEKYRAEVAGALSSTDFLLERLANQYNLHNDREKLAYITAAV

QMIAGLNDPVAADLYLDKLARQTGVTRESLKVTFVRERRKLQRARNHQQAYQASTLME

NVSESAEPKEAQAGTDSETRNPALDRLLYLFIHSDEARDYLLSQQFLFPDERYAKLAE

FWLKYHQTHDEAEVKGFIDFIPEELQGIIINMEMIPMPPDYSKRELDDQLRALEKSKI
DEQLNDLLNQLKEAQRKQDNSLELEIAQKVIALRRKKF"
CDS complement(167817..169889)
/gene="glyS"
/locus_tag="OCPHGMPK_01088"
/EC_number="6.1.1.14"
/inference="ab initio prediction:Prodigal:2.6"
/inference="similar to AA sequence:UniProtKB:P00961"
/codon_start=1
/transl_table=11
/product="Glycine--tRNA ligase beta subunit"

/translation="MTKDYLFEIGTEEMPAHVVSRSVKQLADRTKKYLKENGLSFKDI

KTYSTPRRLTILVEDLAEKQDDIDEVKKGPAKKIAQDKDGNWTKAAQGFARGQGMTTD

DIYFEELKGTEYAYVHVQKEGKKASDILMGMSDIIKAMTFPTKMRWDSNDFEFVRPIH

WMVSLLGSEVVPVKLLDVVAGRKTQGHRFLGDSVVLANADDYEEALKSQYVIADADER

KGMILNQIQELVAQHNWKVNIDKGLLEEVTNLVEYPTVFAGSFDEKYLNIPDEVLITS

MKDNQRYFEVYDENGKLINHFIAVRNGNSEYLDNVIAGNEKVLVARLDDAQFFYDEDK

KYPLAHFVDKLKNVSFHDKIGSVAEHMARVQIIGDYLGKKFNISETEMKDFDRVSDIY

KFDLVTSMVGEFAELQGVMGMHYARLTGEDENVSVAIKESYMPTSAEGDLPSTTVGSL

LSIADKLDTIISFFGAGMIPSSSNDPYALRRNAYGIVRIVLNEGWSLPVKDVLPELIQ

LLSGKTAAKLPKDAEAENEIADFIRDRVKQQLQVEKYDYDVIDAVLASSQQDPIQILA

AAKTLQMHHDDADFKPVVESLTRITNILKKAKYRNAAKVDESLFQDVSEEELNAGVNA

LEENTDLSIAELYKGFVELQPVINNYFESNMILDKDEKVKNNRLAQLLKVNNLADRMG
DLSKLVIK"
CDS complement(169882..170799)
/gene="glyQ"
/locus_tag="OCPHGMPK_01089"
/EC_number="6.1.1.14"
/inference="ab initio prediction:Prodigal:2.6"
/inference="similar to AA sequence:UniProtKB:O67081"
/codon_start=1
/transl_table=11
/product="Glycine--tRNA ligase alpha subunit"

/translation="MSEKLNIQNMIFKLEQFWASKGCMIMPSYDEQKGAGTMSPYTFL

RAVGPEPWAACYVEPSRRPADGRYGDNPNRLYQHHQFQVVIKPAPKDIQQYYLDSLRV

LGIEPLEHDIRFVEDNWANPSMGCAGVGWEVWLDGMEVSQFTYFQVVGELDVKPTMSE

ITYGVERLASYIQDVNSVFDLEWGDGVKYRDIFKQPEYEHSKYAFEESDQDQLLKFFD

IYEATAKRLLSQNLIHPAYDYILKCSHTFNLLDARGAVSVTERAGYLSRIRNLAHLAA
KGFVEEREKRGFPLLKHQEETKKAGQNND"
CDS complement(171094..171846)
/gene="recO"
/locus_tag="OCPHGMPK_01090"
/inference="ab initio prediction:Prodigal:2.6"
/inference="similar to AA sequence:UniProtKB:P42095"
/codon_start=1
/transl_table=11
/product="DNA repair protein RecO"

/translation="MVRELCEVQGLIFKRKKYKEADVLAKIMTKDHGIFTIDVRGALR

PKSRLGAATLNFSYGKYIVNTNWKGISTLRTFKDVKQLDQLYLDLTKNAYSSYVLDLL

DHAFVEYKEIGSFYDLIMKALLKINSGEDAAIITQMVQLKLLNAYGVAPQLDRCLICG

KVQGTFDYSLELGGIICSDHFNSVSQRMHLDPKVVALIRTLALVDIDRLGKVKINPQL
KKNSGKVIDRMYANYLDLNLKTKKFLDELALF"
CDS complement(171847..172755)
/gene="era"
/locus_tag="OCPHGMPK_01091"
/inference="ab initio prediction:Prodigal:2.6"
/inference="similar to AA sequence:UniProtKB:P37214"
/codon_start=1
/transl_table=11
/product="GTPase Era"

/translation="MDEKKDYKSGFVALIGRPNVGKSTLLNFLVGQKVAIMSPQPQTT

RNKISGIYTDDQEQIVFIDTPGIHKPKNKLDDFMDKSSYSALDEVDVVLFMVEPEPAG

KGDQYIAELLKKIKKPVFLVINKIDKVHPDKLLSIIDSYKNLGDFAEIVPISASQGNN

VSELIKTIAKYLPEGPQFYDADQLTDRPEYFIVAELIREQVLKLTHEEVPHATAVVVD

RMRDHEGGKLQIEATIYVERPGQKGIIIGKKGQMLKQIGIAARQEIEALLGEKVNLRL
WVKVQKNWRSDPVFLKSIGYNAKELR"
CDS complement(172782..173309)
/gene="ybeY"
/locus_tag="OCPHGMPK_01092"
/EC_number="3.1.-.-"
/inference="ab initio prediction:Prodigal:2.6"
/inference="similar to AA sequence:UniProtKB:P71335"
/codon_start=1
/transl_table=11
/product="Endoribonuclease YbeY"

/translation="MNNLDISFNDEVNFLDNDDKDWIPWITNLLLSAKKEINKENAQE

MSINFVSSKKIHEINKKYRGKDRPTDVISFAIEDGLDEDFMSAFNDDPDFVEDIGDLF

LCPEVIKRHSVEYETGFNREFGYTLVHGYLHLNGFDHIEDDEAKVMFGIQGKVLREYG
LPLHPDQENHGKQIH"
CDS complement(173313..174272)
/locus_tag="OCPHGMPK_01093"
/inference="ab initio prediction:Prodigal:2.6"
/inference="similar to AA sequence:UniProtKB:P9WIA3"
/codon_start=1
/transl_table=11
/product="PhoH-like protein"

/translation="MGQTIEATFTPTKPETIMNLVGINDANLRLIEESYDVQVTDTGS

EIEVTGEESLVKKIMQIFTALDKIVTRGVTITATDVVSAIKMADKGTLEFFGELYNKI

LIRDAKGRPVRVKNMGQKRYIEAIQKYDVVFGIGPAGTGKTFLAVVMAIAAFKKGEVS

RIILTRPAVEAGESLGFLPGDLKEKVDPYLRPIYDSLYAILGVETTNRLMERGVIEVA

PLAYMRGRTLDDAFVILDEAQNTTQAQMKMFLTRLGFNSKMVVNGDQTQIDLPGKAKS
GLLQAEHILANIEQVKFINFTFNDVVRHPVVAKIVRAYEKEGH"
CDS complement(174303..174746)
/locus_tag="OCPHGMPK_01094"
/inference="ab initio prediction:Prodigal:2.6"
/codon_start=1
/transl_table=11
/product="hypothetical protein"

/translation="MSLNDTLMQDMKSAMKAKDKEALTTIRSLKAAVMNYKIKVGHDL

TSDDELTVLSSALKQRKESLEEFTKAGRDDLIAQTKKEMQLIEKYMPKQMSKEELEQT
VEETIKEVGASSKKDFGKVMQALMPKIKGKADGKAASSIVGKKLN"

```
     CDS             complement(174850..175026)
                     /gene="rpsU"
                     /locus_tag="OCPHGMPK_01095"
                     /inference="ab initio prediction:Prodigal:2.6"
                     /inference="similar to AA sequence:UniProtKB:P23829"
                     /codon_start=1
                     /transl_table=11
                     /product="30S ribosomal protein S21"

/translation="MAKTIVHENESIDDALRRFKRSVSRSGTLQEYRKREFYEKPSVK
                     RKLKSEAARKRRHY"
     CDS             175178..176020
                     /gene="yqfL"
                     /locus_tag="OCPHGMPK_01096"
                     /EC_number="2.7.11.32"
                     /inference="ab initio prediction:Prodigal:2.6"
                     /inference="similar to AA sequence:UniProtKB:P54470"
                     /codon_start=1
                     /transl_table=11
                     /product="Putative pyruvate, phosphate dikinase
regulatory
                     protein"

/translation="MTEEKKENQKIVNLIIISDSVGDTAFNMVQAGAVQYPDVKFNYR

RYPFITNREKLEKVFDEITEFENVLIAFTLIHEDEQLAAIKFAREHNMKYVDLLSGVI

ENIHSLTGEEPKHEIGAVHHMGQNYFDRISAMEFAVMYDDGKDPKGFLEADVVLLGVS

RTSKTPLSLFLANKNLKVANLPLVPQTHIPDEIYKVDPKKIIGLTNDPSVLNEIRRQR

MIAYGLNPDTTYSNMDSINAELDAANKLYKKLGCYVINVAHRSIEETAALIMEHLGID
                     DYAK"
     CDS             complement(176092..176970)
                     /locus_tag="OCPHGMPK_01097"
                     /inference="ab initio prediction:Prodigal:2.6"
                     /codon_start=1
                     /transl_table=11
                     /product="hypothetical protein"

/translation="MSQLEKLSRRHYLISKLSAAFLYALAVAVALNFFWGPGKIYASG

ITGFAQIIQSVSERFMPFTLSTSVMYFVLNIPLFILGWCKIGHRFTIFTIIAVLLGSI

MMRVIAPIKITYDPIICALFGGVINGVGTGIALKSGISTGGLDVLGIILRKKTGKSFG

QINIFFNLIIVICAGFVFGWTRALYTALNIFVNGRVIDAVYTQHQKMQVFIVTEHPKH

IIEGIQRKMHRGITIFHDVEGAYSHTEKTVLMTIIDRYDMFDIYNIVIQSDPYAFMSV
                     SEVDKVYGRFKEQKPV"
     CDS             complement(177066..177623)
                     /gene="msrA"
                     /locus_tag="OCPHGMPK_01098"
                     /EC_number="1.8.4.11"
```

```
                     /inference="ab initio prediction:Prodigal:2.6"
                     /inference="similar to AA sequence:UniProtKB:P54154"
                     /codon_start=1
                     /transl_table=11
                     /product="Peptide methionine sulfoxide reductase
MsrA"

/translation="MKVAKPLDNTKESHYDTAIFAGGCFWCMVEPFDTYPGIISVVSG

YTGGHVKNPSYEEVCTGTTGHVEAVKITFDPKIISYQKLVNIYWQVTDPTDAMGQFQD

RGSQYQPIIFYNSPKQKKIAEESKKQLEDSHKFDQQIVTKIEPAEPFYPAEEYHQDFY
                     KKDPLRYQIEEAGGRSQFIEKYWKK"
     CDS             complement(177772..178530)
                     /locus_tag="OCPHGMPK_01099"
                     /inference="ab initio prediction:Prodigal:2.6"
                     /codon_start=1
                     /transl_table=11
                     /product="hypothetical protein"

/translation="MNFEEELYNLDVPEEHIPELLKFGKNNPEFLTKAIKAEGNAKKY

SVAQTKMEPSDLINNQIAFLGSSVTYGAGALGESFVDYLSKKDNICAFKDAVSGTTLV

DNDSNSYIGRIGRLPVLEKLTTFVLQLSTNDAIKGKEDLGEIATDDNFDAQTVTGAIE

YILNYVKKNWNCPVLIFSNPKFDSEFYGQMVERVKELQSKWGFDFLNMWDDDSFNYSE
                     KDRELYMLDPIHPTRAGYKLSWLPVIEKELLEIK"
     CDS             complement(178533..178964)
                     /gene="msrB"
                     /locus_tag="OCPHGMPK_01100"
                     /EC_number="1.8.4.12"
                     /inference="ab initio prediction:Prodigal:2.6"
                     /inference="similar to AA sequence:UniProtKB:E6ESW1"
                     /codon_start=1
                     /transl_table=11
                     /product="Peptide methionine sulfoxide reductase
MsrB"

/translation="MFNKEEALKKLNSEQYKVTQHAATEYPFTGKYDNFNQKGIYVDV

VSGEPLFSSEDKYDAGCGWPSFTKPIEKLVYHRDQSHGMERTEVKSPVADSHLGHVFI
                     DGPVDQGGLRYCINSAALKFIPYDKLEEEGYKEYKKLFKGR"
     CDS             179071..180255
                     /gene="patA_2"
                     /locus_tag="OCPHGMPK_01101"
                     /EC_number="2.6.1.-"
                     /inference="ab initio prediction:Prodigal:2.6"
                     /inference="similar to AA sequence:UniProtKB:P16524"
                     /codon_start=1
                     /transl_table=11
                     /product="Putative N-acetyl-LL-diaminopimelate
                     aminotransferase"
```

/translation="MPKLANDLSLTKNTRLNSLGPSKIRAFDAKASQIPGIIKLTIGE

PDLNTPDHVKEAAIADIKANDSHYAPQAGKPELLEAISKYLERSLDVKYDPKTEICVT

VGATGALNDVFMSILNPGDKILVPTPVWALYFQLIKLTGAIPVQMDTSKDGFILTPEH

LETVLQNEGKGAKAIILTDPSNPTGRVYPEKTLKALAEIITKYHLFSVTDEIYGELVY

DNNVHHSLSQYIPERNILISGLSKAYAMTGWRLGYIAAPADIMKTIQKVNAFLVTSVT

DNVQAAAIEALNNGQADPLESRKIYEDRLKFMKAGLEKLGFEMSTPQGAFYIFAKIPD

AFGTDDEAFANELATKAKVGVTPGRYFGDGGQGYVRMSYASSTEQLKEALKRIAEFVE
NI"

```
     CDS             complement(180432..182285)
                     /gene="aspS"
                     /locus_tag="OCPHGMPK_01102"
                     /EC_number="6.1.1.12"
                     /inference="ab initio prediction:Prodigal:2.6"
                     /inference="similar to AA sequence:UniProtKB:P67015"
                     /codon_start=1
                     /transl_table=11
                     /product="Aspartate--tRNA ligase"
```

/translation="MMNMEKRTDYAGNITSKYEGQEVTLYGWVQRVRNLGNLVFIDLR

DREGIVQVVVNKDSGKELMDIADSLNNEDVIEVKGKVVKRSSVNPDMKTGEVEVDATE

IDILNKSKVPPFEIKDDINAAEQTRLKYRYLDLRRPTLQKAIMLRSKILKATHEYFDE

HGFIDIETPILGKSSPEGARDYLVPSRIYPGSFYALPQSPQLFKQLLMGAGFDKYYQL

ARCFRDEDLRGDRQPEFTQIDMETSFTDEKQVQDYTEGLLKKIMKDVMGIDLKTPIKR

ITWNEAMNKYGSDKPDTRYEMFIHDLSPIFKDSDFKVFSGAIADGGYVKGIAVKNGAK

QYSRKKIEEKQDYIKRYHAKGLAWVKYEDGEFSGPVSRFLTDENKEALKKEFDLEGGE

LVVFVADKWKVVTDSLDHLRREFAKETGIIPENVYDFVWVVDWPLFEYDEGLGRWIAA

HHPFTMPDDEGIKLLDTDPHKAHARSYDIVMNGDEMGGGSIRIHKRSIQEKMFKALGF

TKKRAYEQFGYLLDALDMGFPPHAGLAIGLDRFAMMLAQKDNIRDVTAFPKNASASEP
MMHAPAPVADQQLDDIGIKVEDKYEDSVKEIEARLEKEAQEDADNNSTWDE"

```
     CDS             complement(182291..183577)
                     /gene="hisS"
                     /locus_tag="OCPHGMPK_01103"
                     /EC_number="6.1.1.21"
                     /inference="ab initio prediction:Prodigal:2.6"
                     /inference="similar to AA sequence:UniProtKB:P30053"
                     /codon_start=1
                     /transl_table=11
                     /product="Histidine--tRNA ligase"
```

/translation="MKVQRPKGTVDILPADSGSWEKVETIARNFFKRANYREIRTPSF

ENYEVFSRSSGESSDVVEKEMYDFNDKGGRHIALRPEGTAGVVRAYVENKLYGPDVVK

PFNVYYIDNTFRYERPQAGRQREFHQIGVESFGSNSPLADVETIMMGHDLLAELGVKN

YELHINTLGNAQVRKDYHDALVNYFTPLKDQLSEDSQRRLSMNPLRILDSKADEDKQF

LPDAPRIVDYLDEDSKKNFETITDMLEQLGINYVLDDDLVRGLDYYTGVIFEFMVEDK

NLWESATTILGGGRYNHLVEEFDGPETPAVGFGIGEERLMLVLKEQNPALFKDEGIDF

FITNIGEGTEMKAVEIARSLRKQGFKAQYDVDQKKLKQQFRKADRVHAKFVITLGAKE
LENGVLNIKRLADGKTLDLSLEDLNDMKSVMEKIED"
CDS complement(183858..184295)
/gene="dtd"
/locus_tag="OCPHGMPK_01104"
/EC_number="3.1.1.96"
/inference="ab initio prediction:Prodigal:2.6"
/inference="similar to AA sequence:UniProtKB:O66742"
/codon_start=1
/transl_table=11
/product="D-aminoacyl-tRNA deacylase"

/translation="MRVVVQRVNKAQVTIDNEVVGKIKRGFLLLVGLREGDELDQVKK

AADKIAKMRIFEDEDGKTNLSLKDVNGEILSVSQFTLLANTKKGNRPSFVEAMRPPKS
KDLWENFNQELENRGFHVETGEFGADMQVSLENDGPFTIVLDI"
CDS complement(184295..186547)
/gene="relA"
/locus_tag="OCPHGMPK_01105"
/EC_number="2.7.6.5"
/inference="ab initio prediction:Prodigal:2.6"
/inference="similar to AA sequence:UniProtKB:O54408"
/codon_start=1
/transl_table=11
/product="GTP pyrophosphokinase"

/translation="MSKYREMTHEEVLAECKKYMNDEHLAFVESAYKFAKNAHEGQFR

VSGQPYIIHPTQVAGTLATLRLDPDTVAAGYLHDTVEDTPVTNDDIKEKFGKDVAFIV

DGVTKLSKIQYKSKSHEEYLADNHRKMLIAMAKDLRVIMVKLADRLHNMHTLDHLRPD

KQRRIADETLDIYAPLADRLGIGTIKWELEDMSLHYLNPQQYYRIVNLMQSKRSEREG

YIADAIKTLKQTLDSLGIEYEIYGRPKHIYSIYKKMVNKHKDFSEIYDLLAVRVIVKS

VRDCYAVLGAVHTKWKPMPGRFKDYIAVPKVNGYQSLHTTIIGPGGKPLEIQIRTEAM

HQVAEYGVAAHWAYKEGVKGEVEQTDANKKLNMFQEILELQNETKDSHEFMKSVKTDI

FSDRVYVFTPKGDVYELPKGSVPLDFAYMVHSEVGAHSVGARINGKIVPLDYKLKNGD

VVEMLTQTSARPSRDWIKLVKTSRARNKIKRFFKAEDREENIEKGQNFIEQELLNRGL

APKEFMDKEHIQKVINHFNYHNEEEMFAAVGYGEISAATVANRLTEDLRKKAEDEKQR

QLEEKIMNAGQQSSTEEDDSSDAPADIMRVKHNNGVMVQGVSDLMLHLAKCCNPVPGD

PIIGYVTKGRGVTIHRADCPNITEEAKKQGRLIDVAWENIAKDKDKENYNADIEIYGF

NRSRLLSDVINALNSKTKNIVNISGKVDSNNMAHIYATVSVRDAAHLEDILSRMRDVP
NVYEAKRSIN"
CDS complement(186565..186945)
/locus_tag="OCPHGMPK_01106"
/inference="ab initio prediction:Prodigal:2.6"
/codon_start=1
/transl_table=11
/product="hypothetical protein"

/translation="MKKNSEEKKNFNFKAWWSKINEKSFMPLVWLTLLGVIVWIICPL

VHLSRVWRIGLVFFIFNSYLSYQIGRIMQIRKLSYWWLLLFPVVFAIAVLIHFAKYNF
LLCLVYLSFELFGLWHDDFYKEKK"
CDS complement(186976..187923)
/gene="prmA"
/locus_tag="OCPHGMPK_01107"
/EC_number="2.1.1.-"
/inference="ab initio prediction:Prodigal:2.6"
/inference="similar to AA sequence:UniProtKB:Q04AV7"
/codon_start=1
/transl_table=11
/product="Ribosomal protein L11 methyltransferase"

/translation="MKLLAVKVECSHELEDGLSFFMQDELDAQGIESRKRSDFIAEGQ

KHDSSLVELDDIENLPKDLELTAYFDYENADKKDLIKKIMDKIAEMKSYGLDTGEVSI

STKEVADEDWNTAWQKYYHVIDFSRHLAIVPEWEDYYPAFSDQKLIRLDPGLAFGTGG

HTTTQLVLLAMERALVKPMSVLDVGTGSGILAIAASKLGASHVLGTDISDEAVTAAKE

NIALNDVDNIDVRKANLLKDIDDKYDLIVANILADILLELIPDLDSHLNKEGKVIFSG
IDYLQLPKIEKSLAENGFEIKMKMQEGRWIGLLIARKPD"
CDS 187985..188488
/gene="ybaK"
/locus_tag="OCPHGMPK_01108"
/EC_number="4.2.-.-"
/inference="ab initio prediction:Prodigal:2.6"
/inference="similar to AA sequence:UniProtKB:P0AAR3"
/codon_start=1
/transl_table=11
/product="Cys-tRNA(Pro)/Cys-tRNA(Cys) deacylase
YbaK"

/translation="MSKKNKKKKLNKTLVEKMLDQQKIPYKQLSFNTIQKGDVKQMDT

SILDDEEALVYKTLVCEGNKNSPLVGVVPVTEHLSMKKLAKASDNKKCELLPLKKLVA

TTGYVHGANTPIGIYQKHHFPIYLDSSMKEHDQIAVSSGEVGRSIMINPDDLQKITEA
TFVDLLE"
CDS complement(188540..190231)
/locus_tag="OCPHGMPK_01109"
/inference="ab initio prediction:Prodigal:2.6"
/codon_start=1
/transl_table=11
/product="hypothetical protein"

/translation="MKNSDIKKYKSKVIDYLIPIILSLVIATILIWTILSTRSGLTLD

DTAFHFHRFYDTNQQIKNHNFSYFQMNYGMGESGRIVNALYGPFFAYIMGFLLLFCSS

WLRFQVVITYLIFLVGGLGVYHLSRKVKMTRTVSSVITALFLTTGYIAYWLRSNAFNS

WGAVLIPFVLIQGINLLNNYKKRFSWISLGVVMAIVAQVHLLSTFFSILALIPFFIYG

LILSENKRQMWFDVFKAVVLFVVLTANVWGAFLLLYPTNQMASPIDFSPVLTSVNLSL

AGTSTMWTSITEVTLLLFIVQIIYVAFNFKSSRINTFVTLEGLVFFYLSSNLFPWQFV

KDNFPAVTGYLQFPNRFTVVAYPLLFVGVGLTITELIKKHGKKLGLLASGLAIVIVLF

NVRADFNEINNNISYNKGITNSSREMQEKGLEAYINKIDINTPDYLPVQKKIKSADVT

GMLMNMAPQRKNFSKKVLSGGRLEIKWNGKTAKSTTLPVFIYHQSELTVNGKKVNPKL

NKIGMPIVKSKQGQNTAILSFKTPTWFTILLYISILGWVVMLIYGVFRLVKIVKK"
CDS complement(190312..192738)
/gene="ctpE"
/locus_tag="OCPHGMPK_01110"
/EC_number="3.6.3.-"
/inference="ab initio prediction:Prodigal:2.6"
/inference="similar to AA sequence:UniProtKB:P9WPT1"
/codon_start=1
/transl_table=11
/product="putative cation-transporting ATPase E"

/translation="MKDNFKQPIGLTSAEVDQRTKEGKVNRAVNDQAKTAWQIIKENT

FTYFNLIFLVLSILLVIVGEYKDLTFLPVIIANMIIGIVQELRAKRVLDKLNVMNTTK

IAVIRDGKEEVIPIEHLVLGDVIVLQTGDQIPADGQVISGTLRVNEALLTGEADEIEK

DVGTELMSGSFVVSGKAYARLEKVGKNSYVSKLTLEAKAMNSKEGSEMVRSINKLIKW

VGIIIIPCGIALFIVARYVNHLNLADSVVSMEAAVIGMIPEGLYLLTTIALALAAMRL

ARSQVMLHDMKSVETLARVNVLCVDKTGTITEPKMEVEKIIPVNTNKDDLAKNISVFA

QNMPHDNATIKAVANAFNQKVNIQVKKIIPFTSVNKYSGAVVGESTLLMGAPEFVLRG

QFKDYEKEINKYTSQGYRVLAFAKYPEALEDEKQKLSKEVDLLGYILISNPIRKEARN

TFKYFERQGVNVKVISGDNPLTVARVAKQAGIKDADKLIDATEIGEGDYEEVVRKYNV

FGRVKPDQKKKLIKALQKNGNTVAMTGDGVNDILAMKSADCSIAMASGNSAAVQASQV

VLLDSDFARMPKVVDEGRKVVNNIERSASLFLVKNIFSFLMAIFSLVFAMSYPLRPAQ

IALISAFTIGLPSFLLALENNYSRIRGKFIPNVLSRAIPGGITDMLAVSILVVADMVL

ALGRQEVATTATMLLVAVGMMVLYHISRPLNKFRWTVLGGSFLALVLAVIFFHNLFAL

SRISATAVFLLLVLFFAEITIFRFLSKFADGVREVLVVYDQKGKDLTLNDIKQAYRKG

```
                     RNLVNMPIRK"
     CDS             complement(192753..193769)
                     /gene="ansA"
                     /locus_tag="OCPHGMPK_01111"
                     /EC_number="3.5.1.1"
                     /inference="ab initio prediction:Prodigal:2.6"
                     /inference="similar to AA sequence:UniProtKB:P26900"
                     /codon_start=1
                     /transl_table=11
                     /product="L-asparaginase 1"
```

/translation="MQKKILLLSTGGTIASVVSDAGLVPKESGEQLINMLGELPYDIT

VEDILQLDSSNIQPEEWKLIAEKIFENRNKYDGIVVSHGTDTMAYTASMLAFMLQNIN

IPVVLTGSQVPINVVLSDAPDNLRLAFAAAASCCPNIYLAFDRKVMLGCRSVKVRTIN

FDAFESVNVPPVAKVTSDGLVFSEQNLPKNTKECTLNTEINPHVSLVKLFPGFDPKLL

FAMVESGCKGIVIEAYGLGGMNYIRRNMVGAVGELIRYGIPVIASSQCLYERSDLTKY

EVGREALLEGAISARDMTSESAITKLMWGLGQGWDVHQIAEFFNTDVVGEVTIEKDDT

```
                     LRVD"
     CDS             complement(193870..195246)
                     /gene="yjjP_1"
                     /locus_tag="OCPHGMPK_01112"
                     /inference="ab initio prediction:Prodigal:2.6"
                     /inference="similar to AA sequence:UniProtKB:P0ADD5"
                     /codon_start=1
                     /transl_table=11
                     /product="Inner membrane protein YjjP"
```

/translation="MKNKENKIERVNQLHHKHTLSHHHHMRIRWQEFFTSDNITPAKN

ATLAERSVIVGHIGMMLLSYGTGAWRVRSAMNTIARCLDMTCSTDIGLVSIEYTCVDA

DGKSYTQALSLPTTGVNTTKLDRMENFVNQFKEDDGNWTIGQIHNRLEKISTMKSRYS

PWQVGLAAGLACGGFIFLLGGGIFEVICAFFGASCGNYVRRKMADRHLTILANITVAV

AVACAVYVGSFYLIRLFYPVSLHHLDGYIGAMLFVIPGFPFITSGLDLSKLDMRSGLE

RMAYAIMIIVVATAVGWVMALILNLHPQNFEKLGLTPVVLTLLRLVASFCGVFGFSIM

FNSKISMATVAGLSGAVANTLRLSLVNWSNVPPAVAAFIGAFVAGMLASIIRRKIGFP

RIAITVPSIVIMVPGLYMYRAMFNLGLTSLNAGALWMVQALMIVICLPLGLIAARILM
DKDWRHAG"
CDS complement(195366..196292)
/gene="ldh_2"
/locus_tag="OCPHGMPK_01113"
/EC_number="1.1.1.27"
/inference="ab initio prediction:Prodigal:2.6"
/inference="similar to AA sequence:UniProtKB:P56511"
/codon_start=1
/transl_table=11
/product="L-lactate dehydrogenase"

/translation="MSRRKVFLVGDGRVGSTFANDLLQNVKIDELVICDVVKKITEGD

ALDLEDLAPFVGQCTVKSGDYSDAKDADIAVITAGAARKPGMTRLDLVNTNAKILESI

VKPIVDSGFNGIFVVSANPVDILTTLTQKLSGFPKNKVIGTGTSLDTARLRVALSHKT

GVNVDHIDAYVLGEHGDTSFENFDEAIIDHKPLRSYKELDEQTLVELETDVRKKGGKI

IANKGATFYGVAMCLTQICKAILENNARVMPLSAPMTGEYGIHDLYLGSPAVVTANGI
SDVIELHLSEDEKKKMTYSATKMKEVVDGIEL"
CDS complement(196373..197746)
/locus_tag="OCPHGMPK_01114"
/EC_number="1.3.5.4"
/inference="ab initio prediction:Prodigal:2.6"
/inference="similar to AA sequence:UniProtKB:P83223"
/codon_start=1
/transl_table=11
/product="Fumarate reductase flavoprotein subunit"

/translation="MAYKFPTQTVDNLKEEYDAIIVGSGGTGLAAAIQAHQYGLNVAI

LEKNSFLGGNTMRASSGMNAAESIVQARLGINDSMQKFYDETLKGGGYMNDREMLHYF

VEHAPLAIAWLEDLGIKVDDLTITGGMSKKRTHRPSSMAPIGAFLVNNLLKLADERHI

PIFIDSKVTNLLQDKDKKITGVEVNKEHQVRAKAVLLATGGFGAAKDLIAKYRPDLRD

YKTTNQAGATGDGLKLADKVGAQLEQLELVQVHPTVQQDTDHAYLIGEAVRGEGAILV

DRDGERFINELSTRKIVSNAITETPEHSAYLIFDQGVRDRVKAIEFYDSINLVVHGST

IEELAENIKVPAANLKKTLQSWNEAVKKNSDTEFGRTTGMASLDHAPYYAIHIAPAIH

YTMGGIHIAPDTRVYDTNGNFIAGLYAAGEVSGGLHGNNRIGGNSIAETIIFGREAGK
TMAKLIK"

```
     CDS             complement(197761..199167)
                     /gene="fumC"
                     /locus_tag="OCPHGMPK_01115"
                     /EC_number="4.2.1.2"
                     /inference="ab initio prediction:Prodigal:2.6"
                     /inference="similar to AA sequence:UniProtKB:P64173"
                     /codon_start=1
                     /transl_table=11
                     /product="Fumarate hydratase class II"

/translation="MVKKGYRVESDTLGPVEIPEKALWGPQTQRSKDNFHTGALMPIG

IIRALLQIKLAAAQANIEAGTETEEKGRAIIEAIHQLLDLNNEELQPYFPLHVYQTGS

GTQTNMNVNEVVANLANQNHPGLNVLPNDDVNMGQSSNDTFPTAMNLVATQALDDLKP

SVKHLIKELKVKQDEYWTTVKVGRTHLQDAVPLTFGQELSGYIAALNHDLAYINELEE

TLYELPIGGTAVGTGLNAAPGMAEDIAERLSRKYGHQFDVKTNKFFGLANHSGLNVVH

GALKALAADMFKIAQDIRFLGSGPRAGLGELNLPANEPGSSIMPGKVNPTQAEAVTMA

CLRVFGNDTTITMAASQGNFEMNVFKPVMIAAFLESANVLSGTISSFANKMIHGLTVN

KERMAKDVTDSLMTVTALSPHIGYHEAATIAQTAAKENTTLRTAALKSGLVTKEQYDK
                     WMKPITMTNAMNTKPIDD"
     CDS             complement(199306..200007)
                     /gene="metP"
                     /locus_tag="OCPHGMPK_01116"
                     /inference="ab initio prediction:Prodigal:2.6"
                     /inference="similar to AA sequence:UniProtKB:O32168"
                     /codon_start=1
                     /transl_table=11
                     /product="Methionine import system permease protein
MetP"

/translation="MISFLTKNFPNVVALGWGGDAGWGTAIFQTLFMTFWSAIFGGIL

GLVFGLLLVLTKPKGILANKVVYWIVDKLVSIFRAVPFIILLAFVAPVTQKIVGTQIG

MKAALVPLTLGVFPFFARQVQVALESVDNGKIEAAEALGASTKDIIFDVYLRESRSEL

ARVSTVTLISLIGLTAMAGAVGAGGLGNTAISYGYNRFNNDVTLVATLLVLLLVLIVQ
                     VVGDLVAKRLNHQER"
     CDS             complement(200000..201049)
                     /gene="metN"
                     /locus_tag="OCPHGMPK_01117"
                     /EC_number="3.6.3.-"
                     /inference="ab initio prediction:Prodigal:2.6"
                     /inference="similar to AA sequence:UniProtKB:O32169"
                     /codon_start=1
                     /transl_table=11
                     /product="Methionine import ATP-binding protein
MetN"
```

/translation="MTAQINLKDITVKFGENKAVNNVNLEINKGDIYGVIGFSGAGKS

TLVRTINLLQYPSAGSVEVNGTILFKESKLQVSKKQLQEKRRKIGMIFQNFNLLNEET

VIENVAFALQHTHLSDKELEEKSLHLLELVGLKDKSDFYPAQLSGGQRQRVAIARALA

NDPDILISDEATSALDPKTTNQILDLLYDLNKKLGLTIVLITHEMDAVKRVANKIAVM

ETGKIIEKGDLREVFLHPQKQLTRQFVGGALQAQHILNTYNFDKLADNAELYQLVYSI

NDVTKSVVADLDVSLNTKASILYGNVEVLSGEPIGTLAILLNLDEQKQKEAIKFLESK
                     NVVVTKLDKGVLTND"
     CDS   complement(201066..201920)
                     /gene="metQ"
                     /locus_tag="OCPHGMPK_01118"
                     /inference="ab initio prediction:Prodigal:2.6"
                     /inference="similar to AA sequence:UniProtKB:P31728"
                     /codon_start=1
                     /transl_table=11
                     /product="putative D-methionine-binding lipoprotein
MetQ"

/translation="MHKKRIRNRIIWSVVAVLIVIAGWFSFGPTSTNTKDQKEVVVGV

VGQTKQDAEIWKSVAETAKEDYGIKIKIKNFTDYNQPNKALQNGDIDLNAFQHYTFLN

AWNKANHGSLVAIGNTYIAPIRLYSKKYKNINELPQGATIAVPNDASNESRALYVLKN

AGLIKLRKGVKLATVADITSNPKGLKIKEVSAEQTARVIDDVDASIVNNTYAVPAKLG

DKQTIYIEPLNKDSEQWINIIVANKKDKNNKIYKDLVKAYQTEKTKKLSEKLYGKTEI
                     AAWGLKLK"
     CDS   202325..203053
                     /locus_tag="OCPHGMPK_01119"
                     /inference="ab initio prediction:Prodigal:2.6"
                     /codon_start=1
                     /transl_table=11
                     /product="hypothetical protein"

/translation="MNGESKMKNKVVKIIMWILFWFYLIPYYLVKKFLFKKAAHPIFW

ANCVTIGLFFVLGLVTSPSSHNTSQQSTQKTTLTKKKKEGPETVTKTITKKVGTKELN

KEKARAKVLTQEEKNKQAEYNKLKNQLDDYQAKEEKQKQEAEQERKAAEAQAQKEAAA

KKKEIEEAQKKAKQNQETNNNGSSSQSQGDLYTANQGTIVGNRNSKIYHVPGQAGYHM
                     HSANAVYFNSEQDAINAGYRKAKR"
     CDS   203053..203904
                     /gene="endA_1"
                     /locus_tag="OCPHGMPK_01120"
                     /EC_number="3.1.30.-"
                     /inference="ab initio prediction:Prodigal:2.6"
                     /inference="similar to AA sequence:UniProtKB:P0A3S3"

/codon_start=1
/transl_table=11
/product="DNA-entry nuclease"

/translation="MINKKITQFLAAALLFSLGAGQTYTSQISSASAIKIVKKYKYDY

SDQKEASRLKNKNHKVEKKISSLDKKIADLQKQLKHYTNPNAVESLSKQKLSTLNYEG

KSVVNVNNNNPDFSKEDLDTSQGAWQKYGDLDSQNRVTAANALLNKSLMPKTKREALH

VNPTGWHNKKINHHWLFNRSHLIGYQLTGQNNNWKNLMTGTRHLNDPDMLMYENEVAT

YLKSSPTNYVRYRVTPIFRNNELLARGVEMEGQSINSNDVHFHVYIFNVQDGVTLDYN
TGNSKIS"
CDS 204206..204523
/locus_tag="OCPHGMPK_01121"
/inference="ab initio prediction:Prodigal:2.6"
/codon_start=1
/transl_table=11
/product="hypothetical protein"

/translation="MTQEDLAEFSSLSVNFISKIERTGNQNISIQKLDAIAKALQTSV

ITLIDESSKTPKSQTFVNKLSNDRVFLDKLTNDMKKLPVAQEEALAKSFITIINTFAK
EDK"
CDS complement(204585..205565)
/gene="cbh_3"
/locus_tag="OCPHGMPK_01122"
/EC_number="3.5.1.24"
/inference="ab initio prediction:Prodigal:2.6"
/inference="similar to AA sequence:UniProtKB:P54965"
/codon_start=1
/transl_table=11
/product="Choloylglycine hydrolase"

/translation="MCTSIIYDSNGQHYFGRNLDLEISFGEHPVITPRNYVFKYRKLP

NRKATYAMIGMAIVKDNYPLYFDAANEKGLGIAGLNFDGPCHYFPESAEKENVTPFEL

IPYLLSQYASVDEVKDALENVNLVDINFSKKLQLSPLHWLIADKTGKSIVVESTVSGL

HVYDNPVHVLTNNPEFPGQLTNLANYANISPAQPKNTVVPDADINLYSRGLGTHHLPG

GMDSASRFVKVAFVRAHAPEGKDEASSITNYFHILHSVEQPKGTDEVGPNSYEYTIYS
DGTNLETGTFYYTNYENNQINAIKLSNENLDSDKLIDYELLEKQHINYQN"
CDS complement(205665..207287)
/gene="ybiT"
/locus_tag="OCPHGMPK_01123"
/inference="ab initio prediction:Prodigal:2.6"
/inference="similar to AA sequence:UniProtKB:P0A9U3"
/codon_start=1
/transl_table=11
/product="putative ABC transporter ATP-binding
protein

YbiT"

/translation="MITVSDLSLNLSGRTLYEDVNLKFTPGNCYGVIGANGAGKSTFL

KLLEGKIEPTTGNISIDANERMSSLNQDHFAFEDCTVLDTVIQGHKELYQVMKDKDEL

YSKPDFSDEDGVKAAELESKFAELDGWNAEADASKLLQSLGIPEDLHQSKMSELPEAD

KVKVLLAQALFGNPDILLLDEPTNGLDVHTVNWLEDFLADYPNIVIVVSHDRHFLNQV

CTQMCDVDYGKIQLYMGNYDFWYESSKLASELAANQNAKKEEKIKELQEFIARFSANA

SKSKQATSRKKQLEKITLDDIKPSSRKYPYVKFEMHRDLGNDLLKVEDVSYSVDGEKI

LDHVSFIVRPGDKVAFLSRNTLATTALMDIIAGKIKPETGTVTWGQTTAFNYMSRDLN

ANFNNDELTILDWLRQYATKEQDDNTFLRGFLGRMLFSGEEIEKKIKVLSGGEKVRCQ

LSRMMLEPANVLVMDDPTNHLDLESITSLNDALKDYQGSILFTSHDHEFIQTIADHIV
EVGPKGIVSRADTSYDEFLNSPTIQEQVENIY"
CDS complement(207340..207621)
/locus_tag="OCPHGMPK_01124"
/inference="ab initio prediction:Prodigal:2.6"
/codon_start=1
/transl_table=11
/product="hypothetical protein"

/translation="MKKKIKSFDNKTVLTIGRIGSVLSVLMYVSYIPQIMNNLQGNYG
NPIQPLVAAINCFIWVLYALLREKKDWPLFVANFPGILFGLATFVTSLH"
CDS complement(207737..209023)
/gene="eno2_1"
/locus_tag="OCPHGMPK_01125"
/EC_number="4.2.1.11"
/inference="ab initio prediction:Prodigal:2.6"
/inference="similar to AA sequence:UniProtKB:Q042F4"
/codon_start=1
/transl_table=11
/product="Enolase 2"

/translation="MLKSVIENVHALEIFDSRGNPTVEVFVTLSNGVVGKAEVPSGAS

TGENEAVELRDGGSRLGGKGVMNAVNNVNTEINDALKGLDPHDQPNIDATMIALDGTP

NKGRLGANAILGVSMATACAAAKDNHQPLYRYLGGTDLEMPQTFHNVINGGEHADNGI

DIQEFMITPVAKTSFRDGFEKIVNVYHTLKKVLEDMGYETGLGDEGGFAPNMKNSEEA

LKALHESIIKAGYKPGEDIAIACDCAASYFYNKEDGKYHLEGKVLTDEELADYYDKLL

DEFPELISMEDPYDENDVEGMVKFTESHKDRIQIVLDDFICTNPKLLNKAIHEGAGNA

SLIKLNQIGTVTETLETIRLSRKNGYNTMISHRSGETGDTFIADFAVAVNGGQLKSGA
PARSERVEKYNRLLEIEEELGKGERLAFFPDNVDLD"
CDS complement(209201..209785)

```
                     /locus_tag="OCPHGMPK_01126"
                     /inference="ab initio prediction:Prodigal:2.6"
                     /codon_start=1
                     /transl_table=11
                     /product="hypothetical protein"

/translation="MKLHCLIQLMISAIILLGLIFNIRNSRLFNFYDHLLHHKLVRNR

DSKIWKIITILNEPKVIVVWDFLLAGLLVLLNHPWLAVWSLGTLAVTDAVGIFLKHSV

KRQRPLSMKTYRDGYSFPSGHVLSATIMSLMLWQIFGHTMGIWLLIILIIAWGLVVIS
                     RLNMRAHYLSDVLGATSLALFCFTIAQQLLGLAF"
     tRNA            complement(209940..210027)
                     /locus_tag="OCPHGMPK_01127"
                     /product="tRNA-Ser"
                     /inference="COORDINATES:profile:Aragorn:1.2"
                     /note="tRNA-Ser(cga)"
     CDS             complement(210306..211154)
                     /gene="catM"
                     /locus_tag="OCPHGMPK_01128"
                     /inference="ab initio prediction:Prodigal:2.6"
                     /inference="similar to AA sequence:UniProtKB:P07774"
                     /codon_start=1
                     /transl_table=11
                     /product="HTH-type transcriptional regulator CatM"

/translation="MNLKQLKYFLVVAEERQITSAAKKLFIAQPPLSYQLKQLEKEIG

AQLFIRNAHGMELTEEGKAFQKYAEKIVDLSLTATSQIRQIKTGELGKIRIGVISSCG

GVVPNKQFKKLIEYYPNVSFEIHEANTFGIIEQLQDGIIDLGIVRTPFNLEGLNFKDF

HQEPMVAVTNNNDFDRGKIEHLSQLKGKPIILYRRFQEIFNRSFRHQGIRPFYAVTCD

DARTAIHWADEGLGIALVPQSIAQTYAKSSIVSIKHAHWVTHLNIVWRKDRQVTPLME
                     KIIEMF"
     CDS             211278..213545
                     /gene="zosA_3"
                     /locus_tag="OCPHGMPK_01129"
                     /EC_number="3.6.3.-"
                     /inference="ab initio prediction:Prodigal:2.6"
                     /inference="similar to AA sequence:UniProtKB:O31688"
                     /codon_start=1
                     /transl_table=11
                     /product="Zinc-transporting ATPase"

/translation="MENKDLNGLTDAQAKERLKKDGLNEVPEPEFNFFKEFMSKLWNL

SAWILEAALILECILGKWIQSLFVLLMLLFAAFNGATQKKKSRRVLNTISHELTPTVS

VKRNGKWKQLNSKFLAEGDLISLKRGDVLAADVKIVQGQITVDESSITGESKAIKKSV

GKTAYAGTTVVDGDALATVVATGSNSRSGKTINLINNSAAPGHLQQLLTRIIYYLCLL
```

DGVLTLILVIAALIRGQNVIEMLPFLAMMFIASIPVAMPSTFALSNSFEATRLSKEGV

LTSDLTGIQDAANLNLLLLDKTGTITENKTAVSQWDNLSKLQNKEVLALVGAAIDKRS

PSIIDTAINDYLTEKGITPDIPNSFTPFTSNTGYSMAEVKNYNIKLGSFKQLSLIDKN

ADQEAKNIDFTAGRSVAVLINDKLSGVFILRDKVRSDSKKALEELKKRGIKPIMLTGD

NRRTAEAVAKEVGLTGKVISIHDFNDRTDVNDLAGIADVLPEDKLKMVKLFQEDGYIV

GMTGDGVNDAPALKQAEVGIAVSNAADVAKRSGKMVLLEDGLTPIVKILDAGHRVYQR

MTTWSLTKLSRTAELTMLLTFGYLCFNYIPMALNAMVIYTIMNNMVTMMIGTDNTHIT

YKPESWNMLKLAKIAFSLAAGWTVIGFGFVWYLVSHNYAQGTVSTMVYVYLVLSAMLI

VLITRTRKFFWQSAPSKSVAIVQIIDVLLTFTLALLGLAMTKINFTNLGLTIIVALIA
AIVIDLFYQPIMKNK"

```
     CDS             complement(213632..214975)
                     /locus_tag="OCPHGMPK_01130"
                     /inference="ab initio prediction:Prodigal:2.6"
                     /codon_start=1
                     /transl_table=11
                     /product="hypothetical protein"
```

/translation="MNENDKVAGLPELAKSVIWMINFGYLGIQIAFTLETSQMSRIFQ

TLGADPTKLGWFFILPPLAGLVVQPAIGSLSDKTWAPKLGGRRLPYLLIGMIFAVIMM

LLLPNIGSFGLGYGSVEALVFGAIAIAILDVSANMAMQPFKMMIGDMVNDEQKSYAYG

IQSMLSNSGAVIAAFFPFLLTILGVANTAKKGVVPQSVVISFYVGAVILVITSLLTVT

KVHEYDPKTYARYHGINEEDNKKDGNWFVLLKKAPKVFWEVSLVQLFCWFSFQYLSTY

ATGAIAANVWKAFDPASAGYQLAGNWFGVLTAVQSIAAVIWSYILAKVPNNHHKLGYG

ISLLFGAVGYGSIFFIKSQNLLILSFVLIGISWAAMNTYPLTLVSNALSGKHMGTYLG

LFNCSICLPQIIASLLSFILFPFLKCSMPAMMLTAGISGILAAISVLFIKETYRN"

```
     CDS             complement(215142..215651)
                     /gene="dfrA"
                     /locus_tag="OCPHGMPK_01131"
                     /EC_number="1.5.1.3"
                     /inference="ab initio prediction:Prodigal:2.6"
                     /inference="similar to AA sequence:UniProtKB:P11045"
                     /codon_start=1
                     /transl_table=11
                     /product="Dihydrofolate reductase"
```

/translation="MIRFIWAEDEDGHIGYQGTLPWHLPADLRHFKDLTSNHVIVMGR

RTFESFPGLLPKRQHIILSTSPTLQRKYQDNVHVQIFSQLEELKNWIKHHSNQTIDII

```
GGRRVFEEFMDEVDILEKTKIHHVFKGDTLMPEINYGDFKLVNSEIHQADEKNKFAYD
                     FLEYKRIEK"
     CDS             complement(215681..216637)
                     /gene="thyA"
                     /locus_tag="OCPHGMPK_01132"
                     /EC_number="2.1.1.45"
                     /inference="ab initio prediction:Prodigal:2.6"
                     /inference="similar to AA sequence:UniProtKB:P00469"
                     /codon_start=1
                     /transl_table=11
                     /product="Thymidylate synthase"

/translation="MATLEQPYLDLLNKIMTEGHDKEDRTGTGTRSLFGAQMRFDLSD

GFPILTTKRVSFGLIKSELLWFLRGDTNIRFLLEHNNHIWDEWAFKNWVESDEYHGPD

MTNFGLRSQKDAKFKKVYQEEMKRFGERILADQDFAAKFGNLGDVYGAQWRHWQKREG

GFIDQIQNVIDQIKKTPYSRRLIVSAWNPEDVPNSALPPCHVLFQFYVNDGRLSVQLY

QRSGDMFLGVPFNIASYSLLVNLIAQETGLKPGEFIHTLGDAHIYRNHFDQVKELLTR
                     KPYDSPKLWLNPDKKKIEDFEMSDIKLVNYKHHGTIKAPVAV"
     CDS             216773..218260
                     /gene="cls_2"
                     /locus_tag="OCPHGMPK_01133"
                     /EC_number="2.7.8.-"
                     /inference="ab initio prediction:Prodigal:2.6"
                     /inference="similar to AA sequence:UniProtKB:P63801"
                     /codon_start=1
                     /transl_table=11
                     /product="Cardiolipin synthase"

/translation="MILTWDIIRRIIEILWLINVGLAIWTVFRSHRDIASTWAWLLIL

SILPYVGFILYLFTGRQLSHDDIFSIKAEQQKFRDQFLNEQNKLLKLHDLLPNKDQNP

RARRLVELNLNNDDALLTFNNEVETFIDGKVLFQNLIKNIEQAKSSINIEFYTFYDDQ

LGNQVLKALEKAANRGVKVRVLYDASGSRGTKPSFFNKLRQLGGKAQPFISTAGNRFF

TTPRANYHLHRKLVIIDNQIGYIGGFNIGDQYVDRSKKFGHWRDTHLRVTGQAALLME

IRFAMDWNTSCRKSHLSTYSVDNLIENFRLNVVQAKNLVPMQIVSSGPDNSNFGIRRA

YEEIIAQAQNYVYIQTPYLIPGDSILEALIIAAKSGVDVRIMIPSMPDHPFVYRATEY

YAKYLVNNGVKVYKYDNGFIHAKTIVSGSNIASVGSANQDFRSYQLNFEVNAFTYNPA
                     LTTELKGIFEKDLKESTLLTKKYFNDQSHWRKFKQYFSRLLSSIL"
     CDS             complement(218325..219833)
                     /gene="yjeM"
                     /locus_tag="OCPHGMPK_01134"
                     /inference="ab initio prediction:Prodigal:2.6"
                     /inference="similar to AA sequence:UniProtKB:P39282"
```

```
                     /codon_start=1
                     /transl_table=11
                     /product="Inner membrane transporter YjeM"

/translation="MKKDKSVKIGLKSLVLMIFSAIFGFSNSLTAYYQMGYASIVWYI

ITAILFFLPSALIFAEYGAAFKGVKGGIFSWLKGSVSEKTAFIGTFIWLAAWVVWLVS

STQFFLVSVSTAISGHDTTQSWRFLSLSSTQLLGILEVIFLVVVTFFAAKGVDKIAAV

SNVGGFFTLAITIGFTLVSILVFFLNHGHLAEPLTTQSLVHSPNPAFQSPIAVVSFIV

YALFAYGGLETSAGVIDSVDKPEKTFPKALITAMIWMTALYVLNILMCGVAANWSSEL

SGKNVDLANVEYVLINNLGVETGKAFGLSHSASLTLGATFSRFAGLADVLAGISAAFL

MVYSPVKSFIEGCDPKLLPKNLVKLNKHGMPERSMWVQAVIVSVIILFISFGGNAAGQ

FYTILMDMMNVSSSAPYLFLIGAYPFFKMKQGIDRPFVFIEGKKRVWTVTLVVWLVVA

IGIIFTCVEPLFTGDYATSFWTAIGPVAFGIIAWIYYSYQERKSVTVVDEEE"
     CDS             complement(219960..220487)
                     /gene="apt"
                     /locus_tag="OCPHGMPK_01135"
                     /EC_number="2.4.2.7"
                     /inference="ab initio prediction:Prodigal:2.6"
                     /inference="similar to AA sequence:UniProtKB:P68779"
                     /codon_start=1
                     /transl_table=11
                     /product="Adenine phosphoribosyltransferase"

/translation="MAIDFKEHIASVKDFPNKGIIFRDITPILQDGKLYRAATHELAE

YAKSRGAEVIVGPEARGFIVGCPVATELGIGFVPARKPHKLPREVESASYDLEYGSNV

LEMHKDAIKPGQKVVICDDLMATAGTLHATKELIENLGGEVVGAAFYIELTDLKGREQ
                     FPDLDIYSLVKYHGA"
     CDS             complement(220622..222892)
                     /gene="recJ"
                     /locus_tag="OCPHGMPK_01136"
                     /EC_number="3.1.-.-"
                     /inference="ab initio prediction:Prodigal:2.6"
                     /inference="similar to AA sequence:UniProtKB:P21893"
                     /codon_start=1
                     /transl_table=11
                     /product="Single-stranded-DNA-specific exonuclease
RecJ"

/translation="MKWQQRQASSLDQELIEDYGLTDIQAKLFALRGINTAEKLDFWL

NADENDLADPFLMHDMEKSINRINQAIDNGEKITIYGDYDADGITATSIMMDTLEILG

ADVHFFIPDRFKDGYGPSMSRYQEIVEDGTKLIITVDNGVTGVEEVKYAQEHGVDVIL
```

TDHHTFQDQKPAAFSTVHCNYPGQKYPFDDYCGAGVAYTMCRALMQDTMPELLDLAMI

GTIGDMVKVTGEGHIIVKRGLDILNQTERPGLRALIKQAGLTMGQITATDVGFNIAPR

LNAVGRLADASLAVELLLSDDEEQAEEIAAKIEELNNERKELTAEVYEKCLAQVEEKG

WQHRNTLVLYDPDFHEGVLGLVANKVVEKLHKPTLVLTKDENGELKGSGRSSEGFNLF

DALEPMKDDFLDKFGGHDFACGLSLKVDKLEELRSRFEKSFKPSTDLEVKDFDFELNL

REVTPDTLTEINLVGPFGTGNPEPVFSISEPHIKSLFKMGKDKNHVKFTAEKNNGKLT

IVGFNKGFLNQNLLPFIKELYVTLALNSWKNVSSVQGMLTGIEYGAPKLAVFDQVIDM

RQEDYVMGFADKYLIFDKNTISWARNGLGIPEEKISLARDYHGNSEVVALLDTPRNQV

ELNTALENKYHQLYLRFLFDRLPITNLPSRDAFGKTLKYIYAHPNLTVEDYQVVSAYL

GLDYQAVLFILRVFFELRFVSFIDGKIIGNKRPESKKLTASKYFTSVASQIKFKNQLK
TMPSDQLISYVKQYLK"

```
     CDS             complement(222987..223676)
                     /locus_tag="OCPHGMPK_01137"
                     /inference="ab initio prediction:Prodigal:2.6"
                     /codon_start=1
                     /transl_table=11
                     /product="hypothetical protein"
```

/translation="MAKKTRNTSKKAWIVRIAAIILLIIGLGMIFNSQIRDFMVRQNQ

TTALKNLNKDTVKKNQAKKGMFDFSKVEEIDFGKVTKSRVNNTADAIGAIAIPSVKMY

LPIMKGLSNDAMSTGGGTMRPDQVMGKGNYPLAGHYMTAKGVLFSPLEDTKLGEKVYL

TNLDKIYIYRIYMKKVVDPTAVWLVDNTKQNIVTLITCADGGINRWAIRGKLIAEKPA
TDKNLQVFKLK"

```
     CDS             complement(223839..225677)
                     /gene="lepA"
                     /locus_tag="OCPHGMPK_01138"
                     /EC_number="3.6.5.-"
                     /inference="ab initio prediction:Prodigal:2.6"
                     /inference="similar to AA sequence:UniProtKB:P65272"
                     /codon_start=1
                     /transl_table=11
                     /product="Elongation factor 4"
```

/translation="MDIKKLQDYQKHIRNFSIVAHIDHGKSTIADRILELTDTVSERQ

MKNQLLDDMPLERQRGITIKLNSVEVKYHAKDGETYIFHLIDTPGHVDFSYEVSRSLA

ACEGALLVVDATQGVQAQTLANTYLAIDDDLEILPVINKIDLPSADPEMCKNEIEEMI

GLDASDAVEVSGKTGQGIPELLEEIVKKVPAPTGDLNAPLKALIFDSKYDDYRGVVLS

VRIEEGTVKPGDKIRIMNTGKEFEVTEVGVSSPHPVKKDMLIAGDVGYLTANIKSVRE

TRVGDTITSAENPTEKPLPGYRQIPPMVYSGMYPVDNQKYDDLKEALQKLQLNDAALE

FEPETSQALGFGFRCGFLGLLHMDVVQERLEQEFGMDLIMTAPSVDYHAIMNDGSTKL

IDNPADLPDAGEYKEVQEPYVKAEVMVPNDFVGPVMELCQRKRGEFVTMDYLDKYRVN

VIYNMPLAEIIYDFFDELKSSTKGYASLDYEITGYRATDLVKIDMLLNKEPIDALSFI

AHREEAQNRARQMTTMLKKLIPRQNFEVDIQGAIGAKIISRATIKPYRKDVTWKIHTG
DPDRRAKLLEKQRRGKKRMKAVGKVEVPQDAFMAVLKMNDDDIKGK"
CDS complement(225817..226983)
/gene="dnaJ"
/locus_tag="OCPHGMPK_01139"
/inference="ab initio prediction:Prodigal:2.6"
/inference="similar to AA sequence:UniProtKB:O87778"
/codon_start=1
/transl_table=11
/product="Chaperone protein DnaJ"

/translation="MAQRDYYDVLGVDKNASESDINKAYRRLAKKYHPDLNHEPGAEE

KYKEVNEAYEVLHDKQKRAQYDQFGQAGVNGQAGFGGQGYGGFGGQGGYSSQGFGDFG

DIFGDIFGSAFGGGRSRVDPTAPQKGQDLDYTMTIDFMDAIKGKKTDITYTRSEVCPT

CDGSGAEKGTHPITCDKCHGTGVMTVTRQTPLGVIQQQTTCDKCGGRGTIIKHPCQTC

HGKGTVDKKQTLEVKVPAGIDNGQQIRLSGQGEAGKNGGPYGDLYIVFRVKPSKEFRR

NGTTIYSDAPISFAQAALGDKIRVNTVHGPVDLTIPAGTQPNTNFKLRGQGVPKINGT

GNGDQEVTVKVVIPKKINDKQKEALVDYVKAGGGNISPQEKNFFERLKDKLNGE"
CDS complement(227063..228934)
/gene="dnaK"
/locus_tag="OCPHGMPK_01140"
/inference="ab initio prediction:Prodigal:2.6"
/inference="similar to AA sequence:UniProtKB:O87777"
/codon_start=1
/transl_table=11
/product="Chaperone protein DnaK"

/translation="MSKVIGIDLGTTNSAVAVLEGKEPKIITNPEGNRTTPSVVAFKN

GEIQVGEVAKRQAITNPNTIVSIKSHMGEEGYKVKVGDKEYTPQEISAFILQYIKKFS

EDYLGEKVTDAVITVPAYFNDAQRQATKDAGKIAGLNVQRIINEPTASALAYGLDKDE

NDEKVLVYDLGGGTFDVSILQLGDGVFQVLSTNGDTHLGGDDFDKRIMDWLIQNFKEE

NGVDLSNDKMALQRLKDAAEKAKKDLSGVSSTHISLPFISAGEAGPLHLEADLTRAKF

```
DELTDDLVQKTKIAFDNALSDAGLSVSDIDKVILNGGSTRIPAVQKAVKEWAGKEPDH

SINPDEAVALGAAIQGGVISGDVKDIVLLDVTPLSLGIETMGGVFTKLIDRNTTIPTS

KSQIFSTAADNQPAVDVHVLQGERPMAADDKTLGRFELTDIPPAPRGVPQIQVTFDID

KNGIVNVSAKDMGTGKEQKITIKSSSGLSDEEIKKMQKDAEEHAEEDKKRKEEVDLRN

EVDQLIFTTEKTLKETKGKVPETETKNVQDALDALKKAQKDNNLDEMKEKKEALSKAA

QDLAVKLYQQNGGAQGAAGQAGPQGAQGGQPNNDNGSSNGQGGSTVDGDFHKVDPDK"
     CDS             complement(228952..229530)
                     /gene="grpE"
                     /locus_tag="OCPHGMPK_01141"
                     /inference="ab initio prediction:Prodigal:2.6"
                     /inference="protein motif:HAMAP:MF_01151"
                     /codon_start=1
                     /transl_table=11
                     /product="Protein GrpE"

/translation="MSKEEFPHEKDLKDEVTPDKAPKKDPKATPKEEVKENPAKDYEK

EIAELRAKNKDLEDKYLRSEAEIQNMQARYAKERAQLIKYESQSLAKEVLPAMDNLER

ALAVKADDEAAKQLQKGVQMTLDSLVKSMKDQGITEIKAEGETFDPALHQAVQTVAAE
                     NDDQKDHVVKVLQKGYQYKDRTLRPAMVVVAQ"
     CDS             complement(229542..230600)
                     /gene="hrcA"
                     /locus_tag="OCPHGMPK_01142"
                     /inference="ab initio prediction:Prodigal:2.6"
                     /inference="similar to AA sequence:UniProtKB:P30727"
                     /codon_start=1
                     /transl_table=11
                     /product="Heat-inducible transcription repressor
HrcA"

/translation="MLTERQELILKTIIMDFTQSHEPVGSKTVMNQLPVKVSSATVRN

EMAALEEKGLLEKTHSSSGRIPSTAGYRYYLDHLINPVKIPASVYNRIIYQLDQPFQQ

VNEIVQEAAKILSDLTNYTAFAAGPETRSVKVTGFRIVPLSSHQVMAILVTDDGNVKN

QIYTLPHHTNGEEIEKAVRLINDQLVGKPLSSVNEVLLKRIADHLVAGGSAPEILDLL

QDVIKDAASEQMYVDGQINLLSNYESDDLAKVKSLYKLIDQNDAISSLIGFNPKDEIK

NDSKSKVQVKLGSELQSDLLEDYSLLTAQYSVGKYGKGTIALLGPTNMPYSQMIGLLE
                     YFRNELAKKLLDYYGRFK"
     CDS             complement(230741..231367)
                     /locus_tag="OCPHGMPK_01143"
                     /inference="ab initio prediction:Prodigal:2.6"
                     /codon_start=1
                     /transl_table=11
```

/product="hypothetical protein"

/translation="MAFDYKKEYRNLYHPKKQPEIIKVPKMNYIAVSGSGDPNQEDGT

YHKALELLYGLAYTIKMSKKNEHKIPGYFDYVVPPLEGLWWSKDKQKIDYAHKENFAW

ISMIRLPDFVTEKEFNWAVKTATEKKKQDFSQVKFFTYDEGLCAQVMHIGSYDDEPAT
IEEIHRFVKKENYQIDIQNPRYHHEIYLSDPRRTKTERRKTVIRLPIK"
CDS complement(231391..232896)
/gene="ettA_2"
/locus_tag="OCPHGMPK_01144"
/inference="ab initio prediction:Prodigal:2.6"
/inference="similar to AA sequence:UniProtKB:P9WQK3"
/codon_start=1
/transl_table=11
/product="Energy-dependent translational throttle
protein
EttA"

/translation="MSNIKISNLSFKYSDSSENIFNNLNLDLDSSWKLGLVGRNGRGK

TTFLNMLRGKLQGTGAIKANLEFNYFPLYVENEEQLTLYALEEQVQFDRWELERELNL

MQVDANLIWQPFNTLSGGEQTKILLALSFINKDAFPLIDEPTNHLDEESRKQVVRYLQ

KHSQGYIAVSHGRDFLNQVTNHVLAIENTEIHLYQGNYASYEDTKEKRDKFNREKNEK

LRGQIKTLNASRQRIKGYSLQSENNKKASAHKNEIHADINKGFFGHKAAKIMKRSKNI

ERRMNKDIQDRKGLIRNVESMPELEMNFQPNYHSTLLETRHLDLKVQDKKLFKDLNLI

VKKRGIVSLEGKNGSGKSTFLKSVLNKNIDVTYQGILSLTNGLKISYLPQNFVEYSGT

LAEFVQKEHLSYEKMLNMLRKMGFPRTSFETRIEEMSMGQQKRVAIAKSLVEEADLYL
WDEPANYLDVFNQDQLIDVLKKVKPAMLLVEHDEYFISQVASKRIKLKVDD"
CDS complement(233157..234095)
/gene="ribF"
/locus_tag="OCPHGMPK_01145"
/inference="ab initio prediction:Prodigal:2.6"
/inference="similar to AA sequence:UniProtKB:Q59263"
/codon_start=1
/transl_table=11
/product="Riboflavin biosynthesis protein RibF"

/translation="MKVITLDYPISAPITNQKVILTLGFFDGVHIGHQKLIKDAKLIA

EEKKLPLMVMTFDKHPKEIYKNDHKFVYLETAHEKEQKMEKLGVDYLVIIKFTKEFSQ

LKPQDFVDQVIMKLKADTVVVGFDYTYGPKDIANVENLPKFARGRFQIVVEPKQSIDK

IKVGSTYIRKAIQGGNVELAALLGQPYETSGIIVHGFRRGHKIGFPTANLDISDAKV

LPAEGVYATRAKINGKWHDAMTSVGYNETFKTNHGLTIETNIFNFDEEAYGEPLTLAW
YKFIRSNKKFSGIEELSRQLDQDKRNIKQYFYDLEK"

```
     CDS             complement(234108..235001)
                     /gene="truB"
                     /locus_tag="OCPHGMPK_01146"
                     /EC_number="5.4.99.25"
                     /inference="ab initio prediction:Prodigal:2.6"
                     /inference="similar to AA sequence:UniProtKB:Q97QJ3"
                     /codon_start=1
                     /transl_table=11
                     /product="tRNA pseudouridine synthase B"

/translation="MLNGIVVVNKPRGVTSSDCVYKLRKILQIRKIGHAGTLDPEVNG

VLPIAIGQATKLIELMHEKPKSYIGSGMFGRSTDSYDLDGKTIAEEKIITPFTKSEII

AGMKQLTGKLEQVPPIYSAVRVNGKRLYEYARENIPVERPKRKVNVYSYELTKDPEYD

PLEKTESFDFAIRCSKGTYVRSLVNDLGEKLGVPAVMTSLTRTSSSGYDLNQAVDLET

IEAEVDTPEKWLQPIDSFFAELPQLQLSPDQFKRVSNGASINLNTSYAKVALVYNGHI
                     KAIYQRQGKIYRPEMMLLKNE"
     CDS             complement(235042..235413)
                     /gene="rbfA"
                     /locus_tag="OCPHGMPK_01147"
                     /inference="ab initio prediction:Prodigal:2.6"
                     /inference="similar to AA sequence:UniProtKB:P65967"
                     /codon_start=1
                     /transl_table=11
                     /product="Ribosome-binding factor A"

/translation="MKHRIGRVEGEILRELTKILRKDIRDPRLSDITITAVECTNDLS

YATIYYSLLTEDPAKEKEVAEGLDKAKGMMRHLLGQTLTVYKVPELIFKRDTSVAYGS
                     KIDNLINQVKKQDQERENKNK"
     CDS             complement(235444..238089)
                     /gene="infB"
                     /locus_tag="OCPHGMPK_01148"
                     /inference="ab initio prediction:Prodigal:2.6"
                     /inference="similar to AA sequence:UniProtKB:P04766"
                     /codon_start=1
                     /transl_table=11
                     /product="Translation initiation factor IF-2"

/translation="MAKKRIYEVAKELGIENKIVVKKAQDLGFDVKSHMSSLDDKQVS

KLVDSFKSANTTKPSTEKDSKNSSRKEKTKIKVSVGAIRRRDNRNDHDNRHSNNKRRN

NKIKKQQNDRRAERNKPQAEAKPAARDLLNKFKKKQRAEASELNAQTEASRRKWHQEQ

NPQRSKVKKVENTRKPKEEKLEGAAAVKARVQASQKPVGPKIIKPSPARNKAKRPTVK

KVEPIAPVVPAPQKEEAKPTRKKDFTRKKREVPDYERERSEHSDKARRRRNKKNKRIN

QSKEVKKQPTQRKERPLPETLVYEEGMNAQDLGKLLHREPAEIVKKLFMLGVMTNQNQ
```

SLDKDTIELLAAEYGIEAQEKVHEDISDIDTLYTKEMEESKASKHQEKRPPVVTIMGH

VDHGKTTLLDRLRHTNVSEHEAGGITQKIGAYQVRIDDRLITFLDTPGHAAFSNMRAR

GAEITDIVVLVVAADDGVMPQTIEAIDHAKSAGVPIIVAVNKIDKPGANPDHVMEQLM

KYGLVPEDWGGDTIFVKISAKTGKNVEELLQMILLQADVMELKADPDQKAIGTVIEAR

LDKGRGSVADVLVQQGTLKVGDPIVVGDTFGRVRVMTNDKGRRVKKATPSAPVEITGL

NDVPEAADKLVVFKDEKTARSVGEQRAKNALEKQRENVQHVTLDNLFDTMKKENMKEV

DIVLKADVQGSAEALQQSLEKIEVEGVRVNIIHSGVGAINESDVTLAGASNAFIVGFN

VRPTNTAKSQADAEGVDIRLYNIIYKVMDDVEAAMKGMLEPTYEEKVTGNLTVRETWK

VSKIGTIAGAFVDNGYVTRDSGIRVIRDGIVKYDGKVASLKRFKDDVKEVKQGFDCGI
TIENFNDIKVDDQLEAYEMQEVPVK"
CDS complement(238094..238405)
/gene="rplGA"
/locus_tag="OCPHGMPK_01149"
/inference="ab initio prediction:Prodigal:2.6"
/inference="similar to AA sequence:UniProtKB:P32729"
/codon_start=1
/transl_table=11
/product="putative ribosomal protein YlxQ"

/translation="MQNKQKTLNLLGLVQKAGKLATGFDAVKISLSKNQAKLIFIGSD

VSQNTKDKLAFLMRKKDVKLVDVFSSAEITQALGKERKLVSVTDSGFSKAMIKNLNEG
V"
CDS complement(238408..238704)
/locus_tag="OCPHGMPK_01150"
/inference="ab initio prediction:Prodigal:2.6"
/codon_start=1
/transl_table=11
/product="hypothetical protein"

/translation="MKKRKIPMRKDLLTNTMQPKKELVRVVVDKDKNISVDPTGKKSG

RGAYVSLEPDKIAEAQKNQVLEKSLGVKVSADFYDDLYAYVDHQKARKELFGDK"
CDS complement(238715..239938)
/gene="nusA"
/locus_tag="OCPHGMPK_01151"
/inference="ab initio prediction:Prodigal:2.6"
/inference="similar to AA sequence:UniProtKB:P32727"
/codon_start=1
/transl_table=11
/product="Transcription termination/antitermination
protein NusA"

/translation="MSKEMVEAFATLEKEKGIKQEIIVDAIKAALVAAYKKNYNQAQN

VEVVFDEKKGNFKVNAIKTVVDEVQDSRLEVSLKDALEINRAYEVGDEIRFEVTPKDF

GRLAAQTAKQVIMQRLREAEREHIISEYSQYKDEIVTGTVERRDNRFVYVKIGNVEAV

MPHNDQLPGENYNPQDKVRVLVTRVGSDSKGAQITVSRTAPDLVKRLFEQEVPEVYDG

TVEIVSIAREAGDRTKIAVKSNDPDIDPVGTLVGPKGARVQNIVNELGGENIDVVKYE

ENPSDFIANALNPAEVIAVQFSGDDDDKNALVIVPDYQLSLAIGKRGQNVRLAARLTG

YKIDIKPESQVEFVDSEGDDVETDQDIDDSNESSNEETTESTAEQLEEDSSDDFPNGE
DVDSALDDDADKDEE"

```
     CDS             complement(239958..240434)
                     /gene="rimP"
                     /locus_tag="OCPHGMPK_01152"
                     /inference="ab initio prediction:Prodigal:2.6"
                     /inference="similar to AA sequence:UniProtKB:Q97S61"
                     /codon_start=1
                     /transl_table=11
                     /product="Ribosome maturation factor RimP"
```

/translation="MTKVTELVADVVTPLAEARGDELVDVEYVKEKKQYYLRIYVDRR

PGGIDIEEIANLSELVSEKLGELDPDPFPEPYILELSSPGLERPIKNEKDWERAKGSY

IHVSLYQKIDGEKTYEGTLKDLNQDQIVLEVKIKTRRKEITIPRKTIASSRFAVEF"

```
     CDS             complement(240542..244840)
                     /gene="polC_1"
                     /locus_tag="OCPHGMPK_01153"
                     /EC_number="2.7.7.7"
                     /inference="ab initio prediction:Prodigal:2.6"
                     /inference="similar to AA sequence:UniProtKB:P13267"
                     /codon_start=1
                     /transl_table=11
                     /product="DNA polymerase III PolC-type"
```

/translation="MANKNELFKKLLDQIKFPDKFEDNDIVQNGEIENVDVYANERKW

QIHVFFDTPLDFETYRSLNELIHQTFEPFVNVELLVRTKDGNSKNLPAYWTYAIQNST

NLKPAVREFLQGQAPHLEKEKWLIPTQNNVVNGLLSEQVLAELNSEFRRYGFFNIKFT

TKVDETNIDENLKSLEQVQAQHESAMQEFYEKQPAEPKKKMPIKSTRMGSNKVDKKAK

FIQIQDLEDGSGNVAIEGHIFNTDIHELKSGSVIFTGEITDYTDSISFKKFVSDKDQI

DYLKGIKPGVWARMQGYAADDQYQHDVVFNIRNLELIEHKGREEKYEGEEKRVELHLH

TNMSQLDATSTASDYIKTAKKFGQKAIAITDHADVQAFPEAYHTGASEKMKILYGYEA

NMIDDHALLVLNPTEMDYRDREYVIFDVETTGLSSVYDTIIEIGAVKMKNGEVLERFD

EFINPHHPLSDTTINLTSITDEMVGAADDEKDVIKKFKDFYGDRPLCGHNVQFDVGFV

NAALRRAGLDEISQPVVDTLEVSRLLHPEQTRHTLDSLAKKYNVVLEHHHRANQDAEA

TGYLMFKLLDAFNARFHEADLGKMNDYAKYGQVYKRAKPSHMSVLALNQEGLKNMYKL

VSLASTKYFYRIPRTPKSELRKYHKGLLFGSGCWQGDVFISMMQKGYDEAREKARFYD

YLEVQPPASYQTLIDDDLIKDEAELHEIIQNIYKLGKELNKPVVATGDSHYVEPHEAI

YRKILLAAQKSNPNRNKNLPDLHFYSTQEMLDAFRFLGEDVAKEIVIENTNKLADQIE

EIAPIKSGLYPPHIENADQEMKDLTYNKAYELYGKPLPKIVEDRIELELNSIISNGYA

VIYLISQRLVAKSNKDGYLVGSRGSVGSSLVATMSGITEVNPLAPHYRCPKCKYSKFF

ENGEYGSGYDLPDKNCPKCGTPLVKDGQDIPFATFLGFHGDKVPDIDLNFSGDYQPVA

HNYIRVMFGPDNSFRAGTIATVADKTAYGYVKHYEDENELHLRNAELDRLAAGASGVK

RTTGQHPAGIVVVPDNMDIYDFTPVQYPADDLSAAWLTTHFDFHSIHDNILKFDILGH

DDPTMIRMLQDLSGIDPLTIPPDDPGVMSLFSSPEILGVTPEQIQSKTGTLGVPEFGT

KFVRGMLEETKPTTFSELLQISGLSHGTDVWLGNAEDLINNGTCKLKNVIGCRDNIMM

DLIHWGVKPEVAFFTMESVRHGKGISDENMKILKKNDKIPDWYIPSCLKIKYMFPKAH

ATAYILMALRIAWFKVYYPVIYYASYFSVRADLFDLVAMSHGKNTVKAAMKEIQDKGM

DVSAKDKSLLTILEIANECLERGIKIKMVDVNESEAMDFKIIDDHTILAPFNAVPGLG

DNAAKQIVAARAEQKFLSKEDLATRGKVSQTIMEYFENNGVLEGMPDQNQLSLF"

```
     CDS             complement(244846..246543)
                     /gene="proS"
                     /locus_tag="OCPHGMPK_01154"
                     /EC_number="6.1.1.15"
                     /inference="ab initio prediction:Prodigal:2.6"
                     /inference="similar to AA sequence:UniProtKB:Q831W7"
                     /codon_start=1
                     /transl_table=11
                     /product="Proline--tRNA ligase"
```

/translation="MRQSKFFMPTLKEAPSDAVAKSHQLMLRGGYIRQVTAGVYAYLP

LGYRVLRKAENIIEEEMNNINVPEMIMPHLLPATLWQESGRYKKYGAEMFKLQDRHGR

ESLLGPTHEETFTEIVAKNLKSYKQMPLALYQIQTKFRDENRPRFGLLRGREFVMLDG

YSFAATREQLDEQFDDQKGAYLKIFNRAGVTVHPVIADSGTMGGKNSTEFQAPAAIGE

DTIATNEKGTYAANLEMAKSIDTFKQEPEDAKELTKVATPGMDTIDKLAEFLKVPATR

IVKSILYIADDQKVLVLIRGDKEINEVKLGHVLDADEVRTANADELVEITGSEKGGVG

PIGADWADKIVADETVKDLYNVVVGANETDYQYQNANLNRDFKVDEFADIRTANEGEP

DPVDHLPLKFTTSIEVGHIFKLGTYYTKTMGADFLDNNGKAKPVIMGSYGIGVTRMLS

AAVEQHLTKNGIAWPKEIAPFAIHLIQMKMKDETQTELAEKLEKELSTKYDVLYDDRN

ERPGVKFNDADLVGAPLRITIGRKAKDGIVEVKRPTDEKATEISISDLDAMITKELG"

```
     CDS             complement(246560..247816)
                     /gene="rasP"
                     /locus_tag="OCPHGMPK_01155"
                     /EC_number="3.4.24.-"
                     /inference="ab initio prediction:Prodigal:2.6"
                     /inference="similar to AA sequence:UniProtKB:O31754"
                     /codon_start=1
                     /transl_table=11
                     /product="Regulator of sigma-W protease RasP"
```

/translation="MKGILIFLVVFGILVFVHEFGHFIVAKKCGILVREFSIGMGPKL

FQKMRAKTTYTIRWLPLGGYVRLAGPDDAAKIDPGTTVVLQLDKQNKVKRIDASGSQM

PIEGIPVQVNSADLVDDLIIQGYENGNEDQLKTYAVDHDATIIEQNGTELLIAPRDTQ

FQEASVGKKLATNFAGPFMNILLGFIVFIIWSLAAPGAPTTTVGSTIANQPAQVAGIK

ANDQIIAINDKKISNFNQIASELAKSKGKTVEVTVKRENKVKDFSVKPKARKIDGQRI

YQLGFYGKPDNSLGAKLKRGWDTSISTTGLIFNAVGNLFRHFSLNKLSGPVGIYSQTV

QVSNMGFTYLLAFLAMISINLGIVNLIPIPGLDGGKLLLNLIQLIIGKPIPEDKEAIV

```
                     DVIGFVILLLLLIVAVTGNDIYRYFIK"
     CDS             complement(247829..248620)
                     /gene="cdsA"
                     /locus_tag="OCPHGMPK_01156"
                     /EC_number="2.7.7.41"
                     /inference="ab initio prediction:Prodigal:2.6"
                     /inference="similar to AA sequence:UniProtKB:P0ABG1"
                     /codon_start=1
                     /transl_table=11
                     /product="Phosphatidate cytidylyltransferase"
```

/translation="MKQRVITAIVALILFIPIVLMGGIWIDVLACAFAAVGISEIFIM

KKQIIVSWDFILALLATLTMAVPDSFFKFLPAFLNKYDVFYIFVMLMLVRTVLSKNRV

TFDDAGVYTIAALYIGTGFHFMAAIRNAHLGLAILGYVFAVVWSTDIAAYMVGRKIGK

HKLWPVISPNKTWEGSIGAVICAVIVAAVYVALVPTGRNNVLMIILAFFLSIIGQMGD

```
                     LVESAYKRFYGVKDSGKILPGHGGILDRFDSMLFVLPVVAFMGIL"
     CDS             complement(248646..249365)
                     /gene="uppS"
                     /locus_tag="OCPHGMPK_01157"
                     /EC_number="2.5.1.31"
```

```
                     /inference="ab initio prediction:Prodigal:2.6"
                     /inference="similar to AA sequence:UniProtKB:Q88VJ8"
                     /codon_start=1
                     /transl_table=11
                     /product="Ditrans,polycis-undecaprenyl-diphosphate
                     synthase ((2E,6E)-farnesyl-diphosphate specific)"

/translation="MSQSKKSLNHLAIIMDGNGRWAKKRHLPRFVGHRHGMDNIRNIA

LAANKMGIKVLTLYAFSTENWARPTDEVNYLMRLPIDFFDKFMPELMENNVRVNIMGF

LDELPEKTYLVTQKAMSDTANNTGMILNFAFNYGSRREITAGVQKIAHQVKAGEINPN

DIDEKMVSDHLLTHSLTPYDDPDLLIRTSGEERLSNFLLWQMAYTEFSFSNKLWPDFD
                     KNDLEKLVKDYYGRNRRFGKL"
     CDS             complement(249384..249941)
                     /gene="frr"
                     /locus_tag="OCPHGMPK_01158"
                     /inference="ab initio prediction:Prodigal:2.6"
                     /inference="similar to AA sequence:UniProtKB:Q5XDH3"
                     /codon_start=1
                     /transl_table=11
                     /product="Ribosome-recycling factor"

/translation="MANEVIEKAKDNMKKSIAVFQKELGGIRAGVANASLLEGIKVDY

YGVPTPLTQMSSVSIPEARVLMVTPYDKSSLDDIEHAILASDLGITPANDGTVIRIVI

PQLTGERRQEIAKQVGKLAEKGKIAVRNVRRDAMDTLKRQEKNGDITEDEQRSLEKQV
                     QKVTDDATKEIDKLADQKSQEITQG"
     CDS             complement(249941..250666)
                     /gene="pyrH"
                     /locus_tag="OCPHGMPK_01159"
                     /EC_number="2.7.4.22"
                     /inference="ab initio prediction:Prodigal:2.6"
                     /inference="similar to AA sequence:UniProtKB:Q831V1"
                     /codon_start=1
                     /transl_table=11
                     /product="Uridylate kinase"

/translation="MSQIKYKRIILKVSGEALAGEKGTGINPEVIKHLAEEIKSVHDM

GVEIGIVCGGGNMWRGETGANLGMERAQADYMGMLATIMNGLALQDGLENLGVPTRVQ

TSIEMRQIAEPYIRRKAMRHLEKGRVVIFGGGTGNPYFSTDTTAALRAAEIDADVILM

AKNGVDGVYSADPKVDPNAKKYSELTQLDLISKNLKVMDSTASSLSMDNNIPLVVFNV
                     NKPGNIKKVVMGENIGTVIKGDK"
     CDS             complement(250776..251801)
                     /gene="tsf"
                     /locus_tag="OCPHGMPK_01160"
                     /inference="ab initio prediction:Prodigal:2.6"
                     /inference="similar to AA sequence:UniProtKB:P0A3B7"
                     /codon_start=1
```

/transl_table=11
/product="Elongation factor Ts"

/translation="MAKITAQLVKELRERTGAGVMDAKKALVEVDGDMDKAVQYLRDK

GMAKAAKKADRVAAEGLTGVYVDGNVAAITEVNSETDFVSSNDKFVKLVNAATKTIAE

GKPANMEAAEELKMADGTTLAQSFVDATATIGEKIVLRRFALEEKTDDQEFGAYQHNG

GQIGVITVLEGADAATAKHLAMHIAAMSPKVISPEELDDEFIADQLAVMNHKIDQDNE

SRALVNKKPLPHLVYGSEKQLSDEILAKAKEDIKAELKEEGKPEKIWDKIIPGKMQRF

IDDNTQVDKQFAVLSQNYIMDDSKTVGEFLKEKGAKLVAFQRYEVGEGIEKKQEDFAA
EVREQMK"
CDS complement(251839..252624)
/gene="rpsB"
/locus_tag="OCPHGMPK_01161"
/inference="ab initio prediction:Prodigal:2.6"
/inference="similar to AA sequence:UniProtKB:P21464"
/codon_start=1
/transl_table=11
/product="30S ribosomal protein S2"

/translation="MTVVTMKQLLEAGVHFGHQTRRWDPKMAPYIFTQRNGIYIIDLQ

KTIKMLDDAYNYVKAVAQDGGVFLFVGTKKQAQDAVKEEATRAGQYYVNQRWLGGTLT

NWTTIQSRVKRLKQLKEMSEDGTFDVLPKKEVALLTKEMEKLERFLGGIEDMPRIPDV

MFVVDPKKEKIAVHEANILGIPVVAMVDTNTDPDPIDVVIPANDDAIRAIRLISGAMA
DAIIEGKQGQDDSEDVEKEMADKAAAENGDEESIEEVVEKSED"
CDS complement(252778..253806)
/gene="yfiC"
/locus_tag="OCPHGMPK_01162"
/EC_number="2.1.1.223"
/inference="ab initio prediction:Prodigal:2.6"
/inference="protein motif:HAMAP:MF_01872"
/codon_start=1
/transl_table=11
/product="tRNA1(Val) (adenine(37)-N6)-
methyltransferase"

/translation="MDLLKPNERIDYMYNDDLRIIQEKDAFSFSLDTLLLGYFAQIKI

HDNYKVVDLCSGNAAASIYMSYFNRAHYDAVEIQADMADQARRSIELNKLENRIEVHC

LNALEAPKKLGKDKYDMVVVNPPYFKVPKGHVINPDEKKALARHELAINLEQIIKVSS

DMLKMKGKMFMVHRPERLGEIMHYCLENHLSVKWVQPFVSKRNADANLVVVEAIRNTA

SDGLTLRDAIVVHNPDGSFTPEIEQIISENKKQGKESKKESYYFYCLLCNDGSFYGGF

TNDLKHRLKMHNEGKGAKYTKSRRPVKMIYHEEFDDKRLALKREYWFKHHSRKWKEDF

```
                     LKEHNVKF"
     CDS             253875..254489
                     /gene="plsC"
                     /locus_tag="OCPHGMPK_01163"
                     /EC_number="2.3.1.-"
                     /inference="ab initio prediction:Prodigal:2.6"
                     /inference="similar to AA sequence:UniProtKB:Q8DNY1"
                     /codon_start=1
                     /transl_table=11
                     /product="1-acyl-sn-glycerol-3-phosphate
acyltransferase"

/translation="MFYKIIRPIARFIVWVLNGHLHVHHKDRIPKGNYILVAPHRTWW

EPILFALAASPMEFMFMAKKELFKNPVLRFILVHAHAFSVDRDNPGPSAIKIPVKGLR

KSDLSLIIFPSGTRHSEELKSGAFVIAKMANKPLVPVVYQGPLTFKGLLKRQPLEVCF
                     GDPIYIPRKEKVNKETTPALYHQLEEAWDKLDKEQNPSFHYIAK"
     CDS             254556..255737
                     /gene="cfa"
                     /locus_tag="OCPHGMPK_01164"
                     /EC_number="2.1.1.79"
                     /inference="ab initio prediction:Prodigal:2.6"
                     /inference="similar to AA sequence:UniProtKB:P0A9H7"
                     /codon_start=1
                     /transl_table=11
                     /product="Cyclopropane-fatty-acyl-phospholipid
synthase"

/translation="MLEKPLYKMMLSHSFNIPVKVTYWDGKSEVYGNGTPEVEVIFNK

KIPFKDITSNASLALGEAYMNKDLEIKGSIQQLIEAAYESSQSFMRASKFRKFLPKQK

HTEKQSQEDIQSHYDIGNDFYKLWLDPTMTYSCAYFTNGNTDDLEAAQIAKVHHILQK

LDPKPGKTLLDIGCGWGTLMLTAAKEYGLKVTGVTLSQEQFDLVNKKIKDMGLEDQAE

VLLEDYRELGNRDFDYVTSVGMFEHVGSENLGEYFKDVAKYLKPHGIALIHGITRQQG

GATNAWINKYIFPGGYIPGLVEIVSRIEEANLQIADMEMLRRHYQRTLEIWDKNFNDH

RSEVEGMLGERFARMWDMYLQACAASFESGNIDVIQYLLTKGPSGKSLPMTRQYMLND
                     R"
     CDS             complement(255791..257584)
                     /gene="yheH"
                     /locus_tag="OCPHGMPK_01165"
                     /EC_number="3.6.3.-"
                     /inference="ab initio prediction:Prodigal:2.6"
                     /inference="similar to AA sequence:UniProtKB:O07549"
                     /codon_start=1
                     /transl_table=11
                     /product="putative multidrug resistance ABC
transporter
                     ATP-binding/permease protein YheH"
```

/translation="MDNNEETKSVWSKAIPLKEQIHIFKRVLKYVKPFKVEMGIAVFG

AFLVSVINMLLPRGLQFFLDNYLIKQKATVQIIIWAGLLYGLGTIIKAVLQFIYQYLY

ALGAEKTLESVRKDLYRKLHSLGMRYFDQTPAGSIVSRVTNDTMTLSDFLGVLCQVVI

GVFSLVTAFVAMYVTNKFAALIVLLFLPILGFVFWIYTQKSSKLYRGFRERLSRINTN

LNESIEGVSLIQQFKQEKRMTNHFENENGTLMKTRFNMIRVNSLLLSPMTSLLYSLAL

ALVLMYFGFPLQKTFVPAGIVYAFSQYVQQFFNPISTMMDRMTFFQDGIVAGKRIFRI

MDETEYEPKQEDDKNAVISKGKIEFKNVSFSYDGKHEILHNVSFVVNPGETLGIVGHT

GSGKSSIINVMMRFYEFGSGEVLIDDIDIRKFSKKELRKKLGLVLQEPFMFYGDISSN

IRLYNNQITDQQIKKAAQAVQADSFIEKMPGKYHAKVIEGGSELSQGQRQLISFARTL

VTDPKILVLDEATANVDTETENLIQDGLKKLRQGRTTLAIAHRLSTIADADQIIVLDK
GRIVEQGTHTELLEKKGYYYNLYKLQQNSDK"
CDS complement(257574..259340)
/gene="yheI_6"
/locus_tag="OCPHGMPK_01166"
/EC_number="3.6.3.-"
/inference="ab initio prediction:Prodigal:2.6"
/inference="similar to AA sequence:UniProtKB:O07550"
/codon_start=1
/transl_table=11
/product="putative multidrug resistance ABC
transporter
ATP-binding/permease protein YheI"

/translation="MNIFSKLGWFFKQEKKRYIIGISFLALTSIANLVPPRILGLMAD

QLDKGSISWSEYAALILAVVAAAIVLYLLRYFWRKQIWGGAAELERQMRSRLFKHFMV

MDRTFYQRHRTGDLMAHATNDINSIQNVAGDGVLTLVDSLVTGGTTMIAMIIFTDFRL

TVIALLPLPFLALGAWKLGDKLHYSFDKSQAAFSRLNNKTQESVSGIKVLKAFGESTQ

DSTAFNKMVDETIQINKKVFKWDSMFDPLGTLIIGATYVITIIYGGLLVTNHTLSVGQ

LVSFIAYISNMVWPMFAIGYLFNILERGSASYDRVERLLYERPQITDENADQSLTVKD

IQGDLKYDIRSFAYPDEKEIPVLQNIEFTLKPGQTLGLVGKVGAGKTTIIELLLREFD

HYQGKITLGGHDIRNIPLKLLLNEISYVPQNNFLFSTSIEKNIAFSDQDATKNKIESA

AQKSDLHDDIMQMPAGYQTLVGENGVSLSGGQKQRLSIARALLKESPILILDDALSAV

DAKTETAILNSLRSERKGKKTLIAAHRLTSVMDADLIIVLKNGKIVERGKHADLLAQD
GWYAEMWRKQELRAKVGEIDGQ"
CDS complement(259418..259639)

```
                     /locus_tag="OCPHGMPK_01167"
                     /inference="ab initio prediction:Prodigal:2.6"
                     /codon_start=1
                     /transl_table=11
                     /product="hypothetical protein"

/translation="MNLGLAIFLIIIALLIGLVGGFYGARAYMKKYFQDNPPISEDMI
                     AAMMAQMGQKPSAKKLNQVMNMMKHQQQK"
     CDS             complement(259712..259960)
                     /locus_tag="OCPHGMPK_01168"
                     /inference="ab initio prediction:Prodigal:2.6"
                     /codon_start=1
                     /transl_table=11
                     /product="hypothetical protein"

/translation="MDKKEEEKLIKRINELTKISRERELTPDEVEERKKLRSAFLENF
                     RAGFRQQLEDTVVIDENGKEVTSEKAKEAQRRKGLRKD"
     CDS             260099..260722
                     /gene="lexA_2"
                     /locus_tag="OCPHGMPK_01169"
                     /EC_number="3.4.21.88"
                     /inference="ab initio prediction:Prodigal:2.6"
                     /inference="similar to AA sequence:UniProtKB:P31080"
                     /codon_start=1
                     /transl_table=11
                     /product="LexA repressor"

/translation="MTEPHANKQLEILRFIYDTVEERAFPPTVREICSAVDLSSTSTV

HGHLARLEKKGYILKDATKPRAIEVTEKGREALGIKPKDIPIVGVVTAGQPILAVQDI

DEYFPLPPDLENDAGELFMLRVHGESMINAGILNGDHVIVRKQSSANNGEIVVAMTED
                     NEATVKRFFKEDGYYRLQPENDTMDPIILPVVQILGKVVGLYRNNID"
     CDS             complement(260757..261407)
                     /locus_tag="OCPHGMPK_01170"
                     /inference="ab initio prediction:Prodigal:2.6"
                     /codon_start=1
                     /transl_table=11
                     /product="hypothetical protein"

/translation="MRYPDKLLVEVKERSRGMKLEGINFGAGPVHPKLMIIGEAPGRE

EIESLIPFHGASGKELMKSLASIGLKREDVYITSAVRSRPYSVKRVFSKKENKEVIKY

PNRKPTKKEILAHAPLLDFELQYAQPKIIVTVGTTALSRLLDHKYEISKVHGKIIKNT

SILELNSKKDGYVWSKEKYTIIPQYHPAAVFYNRKLADTIAQDWLNIKPLIKLVKK"
     CDS             complement(261391..261819)
                     /gene="mutT4"
                     /locus_tag="OCPHGMPK_01171"
                     /EC_number="3.6.1.-"
                     /inference="ab initio prediction:Prodigal:2.6"
                     /inference="similar to AA sequence:UniProtKB:P9WIX7"
                     /codon_start=1
```

```
                     /transl_table=11
                     /product="Putative mutator protein MutT4"

/translation="MKTVHEYSAGSIIYRINKNEIEFLLVQSMLNRTWGFPKGHLEAG

ENNVQAAKREVYEEVGLRPNYDFNFKESLTYKITRDRLKTVTLFLSEFIPTQKIKLQK
                     SEIGASKWVNLEEASKCLHYEELNELLKKAQDYIKNEISR"
     CDS             261992..262633
                     /gene="spsB_2"
                     /locus_tag="OCPHGMPK_01172"
                     /EC_number="3.4.21.89"
                     /inference="ab initio prediction:Prodigal:2.6"
                     /inference="similar to AA sequence:UniProtKB:Q5HHB9"
                     /codon_start=1
                     /transl_table=11
                     /product="Signal peptidase IB"

/translation="MKKQEQTESWGQWILQVLILVAIFFGIFFVLNKFVFANLTVSGI

SMQPTFENNDRVIALRHAKIKQGDIVIVDAPDEPGALYIKRVIGLPGDTVVSKNNQIY

INGKKINQPWLKAGQKLIDNGEDGVSGTKYTNTQNFTLSSLAKTQNYRQFYTAKQLKE

MQKTNKVPANTYFVMGDHRSVSKDSRYIGTIPRSKIVGVVKMRYWPLNHITFY"
     CDS             complement(262728..263078)
                     /gene="rplS"
                     /locus_tag="OCPHGMPK_01173"
                     /inference="ab initio prediction:Prodigal:2.6"
                     /inference="similar to AA sequence:UniProtKB:O31742"
                     /codon_start=1
                     /transl_table=11
                     /product="50S ribosomal protein L19"

/translation="MDPLIQELTKEQLRDDMPDFRAGDTVRVHVRVVEGTHERIQMFE

GVVIKRKGAGISATYTVRKMSSGIGVERTFPVNDPRVAKVEVLRHGRVRRAKLYYLRD
                     RHGKAARIAEKRRG"
     CDS             complement(263192..263914)
                     /gene="trmD"
                     /locus_tag="OCPHGMPK_01174"
                     /EC_number="2.1.1.228"
                     /inference="ab initio prediction:Prodigal:2.6"
                     /inference="similar to AA sequence:UniProtKB:Q6GHJ5"
                     /codon_start=1
                     /transl_table=11
                     /product="tRNA (guanine-N(1)-)-methyltransferase"

/translation="MKINVLTLFPDMFTPLQVSMLGRGLEDKKWELNLVNFRDFTTDV

HHHVDDTPYGGGAGMVLQIMPIKKALDSLTNKGKVIITAPQGKTFNEKMAQNWAKEEN

LTFICGHYEGFDQRVYDLADETVSIGDYVLTGGELPTMSMIDATVRLLPGILGNAASP

VEESFSHGLLEYPQYTRPADFEGEKVPEVLTSGNHQKIAEWRHKEALRATYLHRPDML
```

```
                     AERELTNEEKKMLEEIKLEKEN"
     CDS             complement(263904..264425)
                     /gene="rimM"
                     /locus_tag="OCPHGMPK_01175"
                     /inference="ab initio prediction:Prodigal:2.6"
                     /inference="similar to AA sequence:UniProtKB:P66656"
                     /codon_start=1
                     /transl_table=11
                     /product="Ribosome maturation factor RimM"

/translation="MTEYFEVGKILSPHGLTGEVKVNATTDFPEERLANGSRLFIKNS

DQYQELIVEATRRHKQFYLVKFEGIDDIDQAEKVRGKELYVAEADQQELPEGSYYFKD

ILNCPVYDAKTGEKLGVLANIETPGANDIWEIKPEHGKSFWIPNIESVVNNVDLANKR
                     IEVTLLEGLRDEN"
     CDS             complement(264494..264766)
                     /gene="rpsP"
                     /locus_tag="OCPHGMPK_01176"
                     /inference="ab initio prediction:Prodigal:2.6"
                     /inference="similar to AA sequence:UniProtKB:P66444"
                     /codon_start=1
                     /transl_table=11
                     /product="30S ribosomal protein S16"

/translation="MSVKIRMRRMGSKRKPFYRIVVADSRMPRDGRFIEEVGYYNPLT
                     NPDEVKLEEDKIFEWLEKGAQPSDTVRSLLSKAGLMTRYHDAKYGK"
     CDS             complement(264853..266283)
                     /gene="ffh"
                     /locus_tag="OCPHGMPK_01177"
                     /inference="ab initio prediction:Prodigal:2.6"
                     /inference="similar to AA sequence:UniProtKB:P37105"
                     /codon_start=1
                     /transl_table=11
                     /product="Signal recognition particle protein"

/translation="MAFENLSERLQKALKNLTGKGKISEADINDASREIRLALLEADV

NFKVVKDFIKKIKKEALGKEVQESLNPGQQIIKIVDDELTKMMGEEAVSLNKSQHIPT

IIMMVGLQGTGKTTTVGKLANYLMKNEKARPLLIAGDIYRPAAIDQLKQIGQQLNVPV

YSEDNQDVAEIVKHGLEEADKNKNDYVLIDTAGRLEIDEQLMDELKRVTAVAHPDNTL

LVVDAMTGQAATQVAEGFNNDLDLTGIILTKLDGDTRGGAALSIRAVTGLPIIFTGQG

EKLTDLSTFHPDRMASRILGMGDMLTLIEKAQQDYDAKEAEKMAEKMRENTFDFNDFI

DQMDQVQKMGPLDQIIKMIPGLGNNPALKNIQIPEKQISHIKAIVYSMTPEERENPDI

LNPSRRRRIAAGSGRSIAEVNRMIKQFKQSKEMMQKMTKGDMGEFANLPGMNSPMGKM
                     AMRSMNKRFKKNKKKLMKKIKRYRSK"
     CDS             complement(266285..266626)
                     /locus_tag="OCPHGMPK_01178"
```

```
                     /inference="ab initio prediction:Prodigal:2.6"
                     /codon_start=1
                     /transl_table=11
                     /product="hypothetical protein"

/translation="MDELEKNERLGDLFAYYGPLLTKGQQDYFEDYYYNDLSLGEIAD

NHHVSRQAVYDNLKRSSKALEKYEDKLHMKRDYIRIEEKITEAVYALEHGKINRAQIE
                     LLKLLKQMGVE"
     CDS             complement(266767..268191)
                     /gene="pepDA_2"
                     /locus_tag="OCPHGMPK_01179"
                     /EC_number="3.4.-.-"
                     /inference="ab initio prediction:Prodigal:2.6"
                     /inference="similar to AA sequence:UniProtKB:Q48558"
                     /codon_start=1
                     /transl_table=11
                     /product="Dipeptidase A"

/translation="MKHLSACTTILVGKKASIDGSVMISRNDDTAGAITPQKFIIEPA

AHGEKGRKIKSWLNKFEMDLPEDAQRVPAVPNVDYKKLGYYDESGINQKNVAMSCTES

TYGNERTLAFDPLVEDGLDEDCMQTVVLPYIDSARAGVKRLGSLIKKYGSPAGNSVLF

GDKDEVWYMEIVTGHHWVAQRIPDDCYAATGNRVAIQQVNFDDPDNFMWSEGIQEFVE

KNHLNPDHEGWNFRHIFGTYTEQDRHYNTSRQWYIQKLFNPEIEQDPEDPDIPFIRKA

SKKLAKEDIEFALGSHYQDTPFDPFGHGTEEEKHRYRPIGLNRTQNSHILQIRSDVPE

EMAGIMWLCIGGPTFTPYIPFFANMNETDPSFNNTSMTYNKEDAWWYYKSLAALVESH

YPQFVQLDTKYLEELNRYYRGRVEEIIENAKDLSGEELTNYLTRENQKTVAHTKKDSE
                     ELMGQMFTDAIKMSKLTFKMDPNL"
     CDS             complement(268231..269562)
                     /gene="ftsY"
                     /locus_tag="OCPHGMPK_01180"
                     /inference="ab initio prediction:Prodigal:2.6"
                     /inference="similar to AA sequence:UniProtKB:P51835"
                     /codon_start=1
                     /transl_table=11
                     /product="Signal recognition particle receptor FtsY"

/translation="MGLFDKIKKSLFGHKDEDQEEKEEHKVDEETTEEAQDSEVNTEA

EEPENESDLALKETNKPDEETEDSDETPGWSDAGSAFEESQNEVEEKTTKAEDTSLEE

SKSEEYEDKISVSEPETDLAENTEEESAEKEDEQERYDKGLEKTNHSFGARLNAFFAK

FRTVDEDFFDDLEDLLIESDVGFETAEELTDSLRDEAKLQNAKSHDDLKQVIVEKLVD

IYDKGGEGKDSKLADDPEAQPNVYLFVGVNGAGKTTTIGKLAKRIKDSGKSVMMVAAD
```

TFRAGAVEQLVEWGRRDGVEVVTGPEKADPASVVYDGVKKAKDRGIDFLLVDTAGRLQ

NKVNLMSELDKIKRTIKKILPDQPNEVLLVLDGSTGQNALLQAKDFDKTTKLTGLVLT
KLDGSSKGGVVLAIRNEMDLPVKLVGLGEKVDDLANFDAEKFAIGLFQGLI"
CDS complement(269565..273125)
/gene="smc_2"
/locus_tag="OCPHGMPK_01181"
/inference="ab initio prediction:Prodigal:2.6"
/inference="similar to AA sequence:UniProtKB:P51834"
/codon_start=1
/transl_table=11
/product="Chromosome partition protein Smc"

/translation="MPLQQLVLNGFKSFADKTTIRFNNGITGIVGPNGSGKSNITEAI

RWVMGEGSAKSLRGENMKDVIFAGSQMRAPMNHAEVELIFDNRDHQLASDEKEVIVTR

KILRNGESDYLLNHHPVRLKDVRTLFIESGMSSDSLGIISQGKVDEILNSKPQQRRGI

FEEAAGVLHFKQQKETALKQLDKTNANLIRINDLVKELEGRIEPLHEQSSLAKEYKFQ

KEQLDHKLKQLLGLEIESLNEEKKDVAKKANTNQEILDKLDNEVKQSQADLEEKRKQS

NKRHAEKDEKQQELLSLTQKLAALNTDLQMHQQSREYDVATQKEYNAQAEELKERQKH

LSKQLTANEEDLKSQNQVLAEFLKKQKELKQELKQGPEQLNNQLEEVRSDYIQTLQDQ

TSNNNEIVYLKNELTRSQKSSNNRQQEVEEQLAAAQKVLAELKKQGHDLVLKRQQLNE

TISSLDQKITQKSKLKDQNEQTYLSVRNKLQQISAQVEGLKRIRDRHEGYYYGVKYVL

NHQSDFHGIVGVIGELISFPAELEAALSTALGGGVQDLVTIDQNSARDAINLLKQTRS

GRATFLPLDGLRHNEIATSTLNSLQSIEGFKGVAADLVTAKTKVNISNAISYLLGNVL

VVDTIDTALRVQRRIGRYYRIVTLDGDIISPGGSMTGGTRNIRNNSPLATNAEIDKLT

NQIKVGKEKFAKLEATLNDLNHKLSDLQTDLETKNTELNSLNQKISEQAIKYENEEKE

VKRLNQLNDLQQKAELEKKQEEAELTSRLEKEQAKKDQLEKLAQTQRSKMDQLKSDLN

DFDEAYQKLQAKLSNLNSDLAVVKNKLENLNAKKAELEEQLVNTNSRLKDIDKKIKAL

ALSQNGQSETEIEEQVAELSKKKKQMQQALAEINQDLGKFDAQINNLDQVATRNYNLR

KNTAAEQEEYSAKLGELKSQINQKLGTLSEEYSLTFEAALQLSKGQNTTELRKKLERE

VHLHKMSLADIGEVNLNAIEEYEDVKTRYDFLNGQQTDLLKARKDIEESMSKLDDEVK

NRFSTTFHQIEKSFAKIFPIMFDGGHARLELTDPKNLLETGIEIIAQPPGKKSQKLTL

LSGGERALTAITLLFAMLQVNPVPFCILDEVEAALDETNVDRFAQFLNHYDMKTQFIV
ITHRRGTMQKADNLYGIVMQESGISKVLSVSLNEIKKEVN"

```
     CDS             complement(273140..273820)
                     /gene="rnc"
                     /locus_tag="OCPHGMPK_01182"
                     /EC_number="3.1.26.3"
                     /inference="ab initio prediction:Prodigal:2.6"
                     /inference="similar to AA sequence:UniProtKB:P66670"
                     /codon_start=1
                     /transl_table=11
                     /product="Ribonuclease 3"

/translation="MVSVAFKNNLKEKYGIKFNNENLLEDAFTHSSYANEHPGRKDYE

KLEFLGDAVLQLAVSDYLYRHFPRLNEGELTRMRSNIVRTEGFSEFAIECGFPAEINL

GKGEEKAGARKRKTLLEDVFEAFNGALFLDQGMPAVQHFLHLTVYPLIAEGDFNASRD

YKTELQERLQVNGPVKIEYQVIAEDESKPSFKVQLLVNDKKISEGQGRNKKAAEQQAA
                     KAALDKNK"
     CDS             complement(273924..275681)
                     /gene="appA_1"
                     /locus_tag="OCPHGMPK_01183"
                     /inference="ab initio prediction:Prodigal:2.6"
                     /inference="similar to AA sequence:UniProtKB:P42061"
                     /codon_start=1
                     /transl_table=11
                     /product="Oligopeptide-binding protein AppA"

/translation="MKKGKLISTLTLLSGVALTLAACGNNNKDTSHPNFKEYTPKKAI

KNGGSVSVAVVTDTPFTGIFNDELSTNNTDSEVMQYGDESLFATNDTYKFVKGGAADI

KINKNAKTATITINPKVKWSDGQPLVAKDYEYAYEIIANKATHSQRYTSSLTDLEGLE

EYHEGKSDTISGIEMPEGENGRTVVLHFKEMKPGMNQSGNGYIWEAAAPYHYLKDVPF

DKLISSNKVRKNPLFYGPYKVSKVVRGQSVSWVPNEHYYKGKPHLNKITASVITPASV

AQSIKSNKFDVTQVSNSQWPNIKGTKGVNFIANIPLSYSYLGFKVGKWDSAKGENVMN

KDAKMNNRALRQAIAYGMNVDQVYKRYSYGLSFRIPTLIPKQFGDYFDKDVKGYTYNI

KKGNELLDKAGYKKKGTYRVQPNGKPLTIRLAAMTGSKVQEPIIQNYIQQWKKLGLNV

KLTSGRLMEMNSFYDKVQNDSKDVDMFIGAWSLSSEPSPQDLYGAKAPFNYSRFVTKE

NTDLLNDIDSQKAFNNSYRVKKFHQWQAYMDKEAYVVPVSNSYSIYAVNNKLTGYSLT
                     PSKSMGGGFPNWYYVGYAK"
     CDS             complement(275807..277561)
                     /gene="appA_2"
                     /locus_tag="OCPHGMPK_01184"
                     /inference="ab initio prediction:Prodigal:2.6"
                     /inference="similar to AA sequence:UniProtKB:P42061"
                     /codon_start=1
                     /transl_table=11
```

/product="Oligopeptide-binding protein AppA"

/translation="MKKAKLFGSLTLLSGVALTLAACGNNSNSKVDNPTKSFKEATPK

KAVKKGGTVSVALETDTPITGVFLNELSDTQNDSDAMAPGNEALFDTNDTYQINDKGP

ATLKLDNKNKTATITVKKGVKWSDGKQVTAKDIEYSYEIIANKATKSSRYTEPLQNIV

GLSEYHDGKSNTISGIEMPDGENGRTVVLHFKEMKPGMKYSGNGYFWEAAAPYHYLKD

VPFNKLQSSDQVRKNPLFFGPYKMSKVVRGQAVTFVPNKYYWRGTPKLDKVTIQVLNP

NSASQAIKSHKYDIAGVVNSQWKNVANTNNVNWIANIPLAYSYLGFKVGKWDGAKGEN

VMNKDAKMNNKALRQAIAYGMNVSAVTKRYTNGLTFHIPTLIPKQFGQYYDKNVKGYD

YNIKKGNEILDKAGYKKKGKYRVQPNGKPLTIRLAAMTGNSTQEPIIQNYIQQWKKLG

LNVKLTGGRLMEMNSFYDKVQNDSKDVDMFMGAWSLSSEPSPNDLYSVKAPYNFTRFV

TAKNTKLLQEMDSEKSFNTNYRVKKFHEWQKYMNDEAYVVPVSNSYNVNAVNSKITGY
SLKPSAANSLWYNVGIAK"
CDS complement(277874..278788)
/locus_tag="OCPHGMPK_01185"
/inference="ab initio prediction:Prodigal:2.6"
/inference="similar to AA sequence:UniProtKB:P9WFZ9"
/codon_start=1
/transl_table=11
/product="Putative peptide transport permease
protein"

/translation="MAKDKNTSKKDQNAKTSLPPSGFKIIVREVWRDKVAFASFCIIV

AILLFTFVGSLFLNKGQVTEINIAEAYYGWGESGHVFGTDDGGRDILKLLMMGGRNSI

LIGLSVTVLCEGVGLLVGLFSGYFGGVTDAVIMRIVDFIQILPQFPIIIVLTTVIPNY

NAGTLVLLISMFGWTSTARYFRAFVLSQREREYVLASKTSGSSNLTIIFREVLPNITS

ALVIDVVLMIAGNIGIETTLSFLGYGLPTTTPSLGTLIGFANDPVNVTTRPWLWMPAT
FLLLIISLSINYVGRALQRAGDARQREN"
CDS complement(278805..279767)
/gene="gsiC"
/locus_tag="OCPHGMPK_01186"
/inference="ab initio prediction:Prodigal:2.6"
/inference="similar to AA sequence:UniProtKB:P75798"
/codon_start=1
/transl_table=11
/product="Glutathione transport system permease
protein
GsiC"

/translation="MWKTVLRRILIMIPQLLILSLLVFILAKMMPGDPFTGMINPNSD

PKEIARLRQEYGLNDPVWVQYTRWLGNMFHGDLGQSYIQKVPVTSLIWDRAVNTFWLS

LMTVVLTYLIAIPLGVTAGRHQDEWQDHGVQIFNYITYAVPPFVFYILGIWLFGFTLG

WFPISGSVSADVNPGTLAYFWSRFYHLILPSILCALISTTAIVQYLRTGIVDNKVEDY

VRTARSKGVPENVVFNKHILRNSLLPIAAFFGNTITGLLSGSMVIESVFSYPGMGKLF
LDSIGQRDYTTLTALILLFGVLTLIGNLLSDIIMSIIDPRIRIK"
CDS complement(279770..280738)
/gene="oppF"
/locus_tag="OCPHGMPK_01187"
/inference="ab initio prediction:Prodigal:2.6"
/inference="similar to AA sequence:UniProtKB:P24137"
/codon_start=1
/transl_table=11
/product="Oligopeptide transport ATP-binding protein
OppF"

/translation="MADEIIEIKNLKVHYPIRSGFWNRVTGYVKAVDGINLSIKEGET

YGLIGESGSGKSTTGKAIVGVEKVTSGQILYKGVDVTKASNRKRLNYNKDVQMIFQDS

MSSLNPRKRIEDIIAEPIRNFENLTTDEERKRVQELLDIVGMPSDAIYKYPHEFSGGQ

RQRIGVARAVATHPKLIVADEPTSALDLSVQAQVLNFMKHIQQEYNIAYLFISHDLGV

VKHMSEDIAIMHRGRFVEIGKRDQIYKNPMHIYTKRLLSAIPVVDVEHREQHKEERQR
VEKEFLQDQDKWYEKDGRVLPLQKVGDRHFVALPKEMIKQELKEGE"
CDS complement(280745..281755)
/gene="oppD"
/locus_tag="OCPHGMPK_01188"
/inference="ab initio prediction:Prodigal:2.6"
/inference="similar to AA sequence:UniProtKB:P24136"
/codon_start=1
/transl_table=11
/product="Oligopeptide transport ATP-binding protein
OppD"

/translation="MEKQSDLLLDIEHLHTAYRLHGKFYDAADDINLTLKRNEILAVV

GESGCGKSTIASSIIGLYDHKNTRVTGDILYNELNLVNLNESLFNKIRGNNIGMIFQD

PLASLNPLMKVGDQVAETLYYHTDMDEKARHARVIELFNQVGMPRPEEMYEMYPHELS

GGLRQRVVIAIAIACKPEIIIADEPTTALDVTIQAQILDLLKEIQKESHSGIILITHD

LGVVAETADQVAVMYAGQIVEKADVKTIFENPLHPYTRSLLHSMPQTDDESQDLHVIH

GTVPSLVNMPRTGDRFAARIPWIPASAHEENPKIHEVEPGHFVRCTCWQSFHFQDEEK
KG"
CDS complement(281913..282155)
/gene="acpP"
/locus_tag="OCPHGMPK_01189"

```
                     /inference="ab initio prediction:Prodigal:2.6"
                     /inference="similar to AA sequence:UniProtKB:Q2FZ51"
                     /codon_start=1
                     /transl_table=11
                     /product="Acyl carrier protein"

/translation="MTEEEIFNKIADMISERFSIDRDKITKDLNFQNDLDADSIDFVE
                     LVMDLEDTFGAEIPDDDAEKLQTVGEAVEYIKNHQN"
     CDS             complement(282211..283212)
                     /gene="plsX"
                     /locus_tag="OCPHGMPK_01190"
                     /EC_number="2.3.1.-"
                     /inference="ab initio prediction:Prodigal:2.6"
                     /inference="similar to AA sequence:UniProtKB:Q82ZE8"
                     /codon_start=1
                     /transl_table=11
                     /product="Phosphate acyltransferase"

/translation="MKKIAIDAMGGENAPQAIIDAVLKVKPKLKDTKFILFGDVEKIN

QLIPDEQKERIEFIATSEVIVDSDEPVRAIRRKKDSSMVVAANYVKAGKADALFSLGN

TGALLACGIFIIGRIKGVERPALMPTLPSAKSEQGFNIIDVGANAQSKPEYLVQWAQM

ANFYAQKIRNIQNPTVALLNNGAEDDKGDPLHQEAYKLLKETKLNFIGNVEGNDLMEG

KADVIVTDGFTGNATLKAIEGTASVILRLLKDSLLNNGLRPKVGALLAKPGLTALKKR

FDTARYGGAVLLGVNAPVVKTHGRSNIRPIYYTLLQIDRMLSQDLVGEYKKYFSESR"
     CDS             complement(283224..285263)
                     /gene="recG"
                     /locus_tag="OCPHGMPK_01191"
                     /EC_number="3.6.4.12"
                     /inference="ab initio prediction:Prodigal:2.6"
                     /inference="similar to AA sequence:UniProtKB:P64325"
                     /codon_start=1
                     /transl_table=11
                     /product="ATP-dependent DNA helicase RecG"

/translation="MNNKQSKLFEPVTELKGVGAKTATALAGLGINTIYDLLFYFPFR

YDDLETIPLDQIEDGQKVLLKGIVVTDPFVSRFGYRKTRLSFKMKIDHDIIMVNFFNQ

PWLKDKVESGKEVAVYGKYQVARQSLSGFKLVAEKKDSGFAPIYSVNRHLKQNKLQKL

IDLALEEVLPEVGETIPAVLREKYRLLSDQTLVEKMHHPKNGNEAKIARRSAIFREFF

LFQVQLAQLLSQRDEDVPGIEKRYDLAAVKELIQAIPFELSDDQKKVVNEIFADLHSP

RQMRRLLQGDVGSGKTVVAVFAIYAAITAGFQAALMVPTEILAQQHFTKVDELLRPLG

VRVALLTGDTRELEKREIYRELADGTINVVIGTHALIQKDVHFKNLGLVIIDEQHRFG

VNQRNTLIKKGVAPDVLAMTATPIPRTLALTVYGDMAVSEIRHLPKGRKPVVSSWATS
```

SKLKEVLELMRSQLDKGFQIYVVTPLISESEKSDLKNAEDLQARLAHYFKDENVVLLH

GQMKGNQKNEIMDSFAAGKIDILVTTSVIEVGVDVPNANMMVIFNADRFGLSQLHQLR

GRIGRGQTQSFCVFVSDPKTEIGKKRMKIITSTSNGFKLAEEDLKLRGEGDVFGKAQS

GLPQFQVGDVVNDYNILVTAQKEARALIKADPNLTSSENKFLLEVLKYKKDLNNN"

```
     CDS             complement(285267..286931)
                     /locus_tag="OCPHGMPK_01192"
                     /inference="ab initio prediction:Prodigal:2.6"
                     /codon_start=1
                     /transl_table=11
                     /product="hypothetical protein"
```

/translation="MVLTEINSDQFRDMVRAATHRMGKNAEFVNKLNVFPVPDGDTGT

NMNLTIESGAKAVNENLSESVGDLTESLSKGMLMGARGNSGVITSQLFRGFYKATEGK

KTLTAQDLADAFANGVSTAYKAVMKPVEGTILTVARVAAEEGKNKANETDDVSEVMRA

VVDGAKKALKTTPDLLPVLKQVGVVDSGGQGLVFIYQGFLEGILGEKDNDDYQLDEGE

MDELINAAHHQAESAQVQLSTSDIKNGYCTEIMVDLTADVPNKEKFDLDQFREHLSNL

GDSLLAVSDGEVAKVHVHTEHPGDVFTYGKKFGQLGKIKIDNMRIQHETILENNEEQE

ESVDFAVIAVASGHGIRELFKSEGVNRIISGGQTMNPSTKDIMDAITKSGAKKAIILP

NNGNIIMAAKQAAEVSDIPVGIVPTKTISQGLTAMLSFDPDASVEENVEAMSEDLDTV

KSGEVTKAIRDTKIDDIQVKKDDYLGIVDGTIKVDNPDLVDTTVSMIEKMLDEDSEII

```
                     TIMYGSDATREEADAVVKKLEASHDDLEFEIHDGGQPVYYFLVSVE"
     CDS             complement(286953..287315)
                     /locus_tag="OCPHGMPK_01193"
                     /inference="ab initio prediction:Prodigal:2.6"
                     /codon_start=1
                     /transl_table=11
                     /product="hypothetical protein"
```

/translation="MAVKIKTKYGLIDISNSVIATVVGGAATSNYGVVGMASKNALRD

GAYTILNRENYRRGVVIKTNDNQIVVDVYIIVGYGLKISEVSHNVQDSVKYNLENLLG

```
                     IKTKSVNVIVQGVKILDD"
     CDS             287487..287672
                     /gene="rpmB"
                     /locus_tag="OCPHGMPK_01194"
                     /inference="ab initio prediction:Prodigal:2.6"
                     /inference="similar to AA sequence:UniProtKB:P23374"
                     /codon_start=1
                     /transl_table=11
                     /product="50S ribosomal protein L28"
```

/translation="MAKDIITGRKTVFGNKRSKALNSVRRSWKPNLQKVRILVDGKPK

```
                     RVWVSARALKSGKVKRA"
     CDS             complement(287722..288390)
                     /gene="thiN"
                     /locus_tag="OCPHGMPK_01195"
                     /EC_number="2.7.6.2"
                     /inference="ab initio prediction:Prodigal:2.6"
                     /inference="similar to AA sequence:UniProtKB:O34664"
                     /codon_start=1
                     /transl_table=11
                     /product="Thiamine pyrophosphokinase"

/translation="MKAIALLGGPKEEWPQNLADKIKLAQKKGALIMASDRGSLFLLE

LGIVPDVALGDYDSLTNHERQLVERKVKDMRYSNPIKDFTDSEMLYYAAFIDYKVDDL

TVYGATGGRLDHFFVNMYAVLKAPFASFKEQIKFVDKQNIVRFFGKGRHLISYNSDYK

YLGIGALTEVKDFSIKNAKYDLAPVDLKVPTMYSSNEYLRQQAVKISCKFGVLIVINS
                     RDKK"
     CDS             complement(288387..289037)
                     /gene="rpe"
                     /locus_tag="OCPHGMPK_01196"
                     /EC_number="5.1.3.1"
                     /inference="ab initio prediction:Prodigal:2.6"
                     /inference="similar to AA sequence:UniProtKB:P0AG07"
                     /codon_start=1
                     /transl_table=11
                     /product="Ribulose-phosphate 3-epimerase"

/translation="MIAPSILNADNMHLSRDIKAAIESGIERFHIDIMDGHFVPNLSY

GPELVKDFKRSFPFTKAEIHLMSNNLDTTLPLFVEAGCDILEFHYEATNKPDYWLNYL

KDHNVRCGMAINPSTPVNKLKPFLNKLDQVLLMTVKPGFGGQKFEEVSVDRLTELQDM

IKNENLDLPIEVDGGIDRETLKLTRDAGAKIFVAGSYIFKNGSIEDQVMKLKKEDK"
     CDS             complement(289046..289939)
                     /gene="rsgA"
                     /locus_tag="OCPHGMPK_01197"
                     /EC_number="3.6.1.-"
                     /inference="ab initio prediction:Prodigal:2.6"
                     /inference="similar to AA sequence:UniProtKB:P67682"
                     /codon_start=1
                     /transl_table=11
                     /product="Small ribosomal subunit biogenesis GTPase
RsgA"

/translation="MNKAQGTIVSAISGYYDVEIENKVVRTRARGVFRDRKQKPLVGD

RVVVQLDDQGMNYLIEILPRTNEIGRPAVANVSRVLLVISAVEPDFSLELLDRYLTFF

AWKNVGVVIYLSKSDITPNEKLKAIKCKLDYYQKIGYSVFEDAEELERQLPTMIQKDQ

IWTLAGQSGAGKSTLLNKLEREANQATGAISTALNRGKHTTRQVKLFKYASGFIADTP
```

GFSAIDLFKIKVDELGNYFYDLKDASVKCKFRRCQHIKEPGCEVKKLIEEERIAKSRY
DSYLKIRQEISENRMPEYLKK"
CDS complement(289939..291927)
/gene="prkC"
/locus_tag="OCPHGMPK_01198"
/EC_number="2.7.11.1"
/inference="ab initio prediction:Prodigal:2.6"
/inference="similar to AA sequence:UniProtKB:O34507"
/codon_start=1
/transl_table=11
/product="Serine/threonine-protein kinase PrkC"

/translation="MEKGHLLGGRYKIISVLGEGGMANVYLAEDIILQRKVAVKVLRL

NLQKDPQTIQRFQREALSTSELSHPHIVSILDVGTEGDCHYLVMDYVDGSDLEEYIQR

NNPIPLPKVIDIMDQILSAVALAHKHNVIHRDLKPQNILMDKKGNIKIVDFGIAIALN

QSTMTQTNTAMGSVHYMSPEQARGSMATKQSDIYSLGIILYKLLTGTVPFTGENAVAV

ALKHFQEKTPSLRAKNPSIPQALENVVFKATAKDPRDRYKSVLDMKKDLDTCLDPKRK

DEPVYKPQHDPAADETIVIPPLEGSVHNKDHETQEKSEPKEEKKNFLDNLKGHKWWWI

GALIAFCVIMVILFFALGRKDMVDIPNLNKMTQTEAKNSLNSSGLELGKISYEYSDTV

PKGKVIRTKPPIGSQIKDGQKVNLIISKGPHLVEMPNVIGESYTVAKKQLVKLGFSVA

KTEEYSSNPKNQVNAQDVEPGQQINPDKATVTLLVSKGPSSASSTRSRRHTIKLRDIV

GYSLKGAQDYARENHLTLKVEEEDSDDKNDGTVLRQSPESGTDVSSGSTLTVIVAKSK

KTDANSSSNSSTTTTTVTKSYTINYPADSSQSNSQKTPDHIQVYVSDDDHSINNIYRD
MNITSEQSFTVPFKLTKKNGHIKILRNGNTILDEDVNK"
CDS complement(291930..292682)
/locus_tag="OCPHGMPK_01199"
/EC_number="3.1.3.16"
/inference="ab initio prediction:Prodigal:2.6"
/inference="similar to AA sequence:UniProtKB:Q5XAP6"
/codon_start=1
/transl_table=11
/product="Putative protein phosphatase 2C-type"

/translation="MIKTAFASSIGRVRETNQDFVKVFKNQNNIIMGIVCDGMGGHQG

GDVASTMAVSHLGHNFEKTDFTDPDLAQKWLTVQLRLENETILNTADRFADLNGMGTT

VVLAIAFTEKILIAHLGDSRCYLYGGNEFKQITEDHSLVHELVKMGQITEQQARNHPQ

KNIITETLGVSSTVNPEFDVLEVHAGDIFLLCTDGLTNSLTDPQIQQILATKNLSLKE
RCNKLINEANRLGGGDNITVCLLAYVEKDGDK"
CDS complement(292689..294005)
/gene="rsmB"

```
                     /locus_tag="OCPHGMPK_01200"
                     /EC_number="2.1.1.176"
                     /inference="ab initio prediction:Prodigal:2.6"
                     /inference="similar to AA sequence:UniProtKB:P36929"
                     /codon_start=1
                     /transl_table=11
                     /product="Ribosomal RNA small subunit
methyltransferase B"

/translation="MSNARTVALETLIKVFNQKSYSNIALNNELVKHELKPADKALAT

RIVYGTIQYKIFLEYQLKPLIKTKLRDKFLMPLLLMSAYQYFFLEKVPANAIFDEANK

LAKKFSKKNSGSYKLVNGILRALERQGKVLPSSDNLVDYLSIKESFPKWLVEYLLDHF

GEDQTKEILVRSNQPAANSIRMAAVKGKFEEIKTALRKDGFDFKESCLTGHNLNLSKG

GVAQTSLFKDGKITIQDAAASLAVDAFNFTGNEQVLDACSAPGGKTVQIAEKLTNGSV

IALDIHENKLRLVKNAAKRLHVSDKVKTKALDARKAQEYFTAGQFDKILVDAPCSGLG

LIRRKPEIRYEKSLDDIKNLARIQLSILENISGLLAPSGELVYSTCTISLEEDEGVVN
                     QFLKLHPEFDLVPVQVGKLPKQNMVRIFPSVDGSDGFFIAKLKKRG"
     CDS             complement(293998..294942)
                     /gene="fmt"
                     /locus_tag="OCPHGMPK_01201"
                     /EC_number="2.1.2.9"
                     /inference="ab initio prediction:Prodigal:2.6"
                     /inference="similar to AA sequence:UniProtKB:P99127"
                     /codon_start=1
                     /transl_table=11
                     /product="Methionyl-tRNA formyltransferase"

/translation="MSSVIFLGTPNFGSVVLQGLIDQGYDVKAVVTQPDKRVGRKQVV

HQSAVKQTALKHNLPVYQPAKLSGSDELAELMKIEPDFIVTAAYGQFLPTKFLKSAKI

APVNVHGSLLPKYRGGAPIQYSVLNGDKETGITIMEMVKKMDAGDIFSQKALPIEADD

TSGTLFDKLSILGRDLLLETLPKFIDGTITRTPQNEAKVVFSPNISKEQEQIKLSMTA

EQANNLIRALNPDPGAYLMLDGKRFKIWKAKPLAEKTSFPAGTLVTNKKRFVISMANG
                     SELELLEVQPTGKKKMNIKDYLNGQGSHFTSGEKIIDE"
     CDS             complement(294963..297359)
                     /gene="priA"
                     /locus_tag="OCPHGMPK_01202"
                     /EC_number="3.6.4.-"
                     /inference="ab initio prediction:Prodigal:2.6"
                     /inference="similar to AA sequence:UniProtKB:P17888"
                     /codon_start=1
                     /transl_table=11
                     /product="Primosomal protein N'"

/translation="MIAQVIVDVAAKQTDRVFEYHIPQELTDVAVGSRVVVPFGRRKV
```

QGFVVGISKTTSYSGKLKDLLVVVDELPPLTPELVALSKTLADKIFAYRITILQSMLP

RVMKANYRKILVPITEEAKKLKIFKGEPLDLNEVSNLNTIVEIRKLIKNNQAKIEYLV

ENKAKKKQAAIYLLTKTKVEYQEILKGLRKTAKKQQALLDNIVKKYHEYPKLGSKIVS

ELDVSSSVLRTAVKKGWLVKKEQEIYRDPLKDFESRKQKNQIKLNSEQTHALNSIGKS

IQKKEAKTFLLEGITGSGKTEVYLHAISIALEQKRNALMLVPEISLTPQMVKQVKERF

GDNVAVLHSGLSEGEKYDEWRRIRRGEAQVVVGARSAIFAPLKNIGLIVIDEEHESSY

KQNDTPRYHARDVALLRSKFHKCPVVLGSATPSLASRARAQKGRYELLLLTKRANQKS

LPKVDLIDLKTVEFAGAQFDLSIPLVDAIKEKIAKKEQVILLLNRRGFASFMLCRNCG

FVLKCPNCDISLTLHKDTQQMQCHYCGHKEPIPRKCPKCQSEKIRFLGTGTQKVQEEL

EKLLPEAKILRMDVDTTRRKGSYKKILDSFGDHKADILLGTQMIAKGLDFPNVTLVGV

INADTALSLPDFNSAEKTFDLLTQVSGRAGRAEKTGRVMIQTYNPDNYAIKLAQSQDY

ESFYQKEMQVRFQGNYPPFFYTTLITITSKNEQSAAKEAFVIKRKLMRHLHAPTIILG

PTPSSVAKIKNQYYYQILVKYKYEPKLHDLLHQIQDDAQLAQKRGLNIYIDNEPERIM
                     "
     CDS   complement(297414..297632)
                     /gene="rpoZ"
                     /locus_tag="OCPHGMPK_01203"
                     /EC_number="2.7.7.6"
                     /inference="ab initio prediction:Prodigal:2.6"
                     /inference="similar to AA sequence:UniProtKB:Q5XAP2"
                     /codon_start=1
                     /transl_table=11
                     /product="DNA-directed RNA polymerase subunit omega"

/translation="MKITYPSIDKLLSRVNSRYSLSVLAAKRAHEIQAGAPLALKHYK
                     SDKAVGKALEEIAAGKVTIDPEHREDIG"
     CDS   complement(297636..298250)
                     /gene="gmk_1"
                     /locus_tag="OCPHGMPK_01204"
                     /EC_number="2.7.4.8"
                     /inference="ab initio prediction:Prodigal:2.6"
                     /inference="similar to AA sequence:UniProtKB:Q8Y672"
                     /codon_start=1
                     /transl_table=11
                     /product="Guanylate kinase"

/translation="MAHQGLLLVLSGPSGVGKGTVKSAMVKQKAFSFEYSVSMTTRKP

RPGEVDGKDYYFVSEDRFQEAIRDNELLEYNEYVGNHYGTPLAPVQKMLKEGKDVLLE

IDVNGAKQVRKLMPDGVFIFLTPPDLHELKHRIVNRGTDSDKVIAMRMKQARKEILMM

```
                     EDYDYAVVNDTVANAVDHIKSIVEAEHVRVPRVINDYRNMVKED"
     CDS             298410..298703
                     /locus_tag="OCPHGMPK_01205"
                     /inference="ab initio prediction:Prodigal:2.6"
                     /codon_start=1
                     /transl_table=11
                     /product="hypothetical protein"

/translation="MNLLKSLNNYSKKIYNYFFTYNINKLTTFAQDPGKIIRILEHAL

FHKEYILLTYQNGKTEIGQLIKRTSAGRFVLDSYDNNIIRIIDLNDIFNVETA"
     CDS             complement(298765..300444)
                     /gene="recN"
                     /locus_tag="OCPHGMPK_01206"
                     /inference="ab initio prediction:Prodigal:2.6"
                     /inference="similar to AA sequence:UniProtKB:P05824"
                     /codon_start=1
                     /transl_table=11
                     /product="DNA repair protein RecN"

/translation="MLVELDIKNFAIIKTLKVRFQEKMTVLIGETGAGKSIIIDAVSL

LLGSRAQNEMIRSGEKKAVITGLFVLSEQKELIEKLCEKYGLPFEDDQLIISRELTHK

GRNVVRINGQLTTINVLREIGKNLVDIHGQNDQQILMDPDRQIDLIDNYAKPKFKDEL

EDYAADFERWRHLTSQLRKLREDAQEIAQKQDILEFQNNELESADLTDPDEDEKLEEE

FNELNNYQKIADTANYFMQLYDDDEHGLATLLGDAQNAAEDLSNYGKKFENFATSFND

GVYSLNDARSELSSLMDQMDFDEERYQYVSSRLDTLNTLKKKYGPTLEDVFKFAQKVK

NELGKFETGDLDEEKIQKQLADVENKLSRKAKRLHKQREQTARELEEKIKKELADLYM

EKARFSIRINETDSFTKLGTDQVIFMIAPNPGESLMPLVKIVSGGEQSRLILALKAIF

SRVEPVGTMIFDEIDTGVSGRVSAAIGKKMHAIGQEKQVITITHSPQVAAAGDQRYSV
                     VKQVKDGATYTQVGPLTQEEAITAIAQMMAGSDVTDAAKRNAADLMESFKK"
     CDS             complement(300455..301267)
                     /gene="tlyA"
                     /locus_tag="OCPHGMPK_01207"
                     /EC_number="2.1.1.226"
                     /inference="ab initio prediction:Prodigal:2.6"
                     /inference="similar to AA sequence:UniProtKB:P9WJ62"
                     /codon_start=1
                     /transl_table=11
                     /product="16S/23S rRNA (cytidine-2'-O)-
methyltransferase
                     TlyA"

/translation="MAKKRADVLLFEQGLFNSRSQAQRAIMAGLVNDHNHQRIDKSGE

KFPEGEIFHIKDDGKKYVSRGGFKLEKALQVFNINLKDKICLDIGASTGGFTDVALQN
```

```
GAKLVYALDVGYNQLAWKLRDNPQVIVMEKQNFRYSKPADFTDGLPDFAMTDVSFISL

DLIMPPMYEILKDQCDAVCLIKPQFEAGPENVGKHGIVHDHDVHANVIKHTMQMAQKI

GFNILGVDYSPIKGGKGNIEFLIHLGKDLANPGQDLWQGSPVDVVQRAVSGL"
     CDS             complement(301271..302137)
                     /locus_tag="OCPHGMPK_01208"
                     /EC_number="2.5.1.10"
                     /inference="ab initio prediction:Prodigal:2.6"
                     /inference="similar to AA sequence:UniProtKB:Q08291"
                     /codon_start=1
                     /transl_table=11
                     /product="Farnesyl diphosphate synthase"

/translation="MNLKDFQKIYTPQIDKFLEDHLASEVDNPIFSKIMSYSVMAGGK

RLRPLLFLATLETLNYKISESELRIACGIELIHTYSLIHDDLPAMDNDDYRRGKLTSH

KKWGEAEAILAGDALLPLGIQWITEGSNSAALAIIISQAVGPNGMVGGQYLDIDSTNN

DLVKENVKVIDQMEWLKTGCLIEASVKMAAVYAGASTDQVDRLDNFSKQFGRSYQIYD

DLVDVVETEQEAGKATHKDADEGKNNTLTLLGIEASRKQLLKMIETGKEAVKIFKQSL
                     LGDFFNLYQKVL"
     CDS             complement(302138..302383)
                     /gene="xseB"
                     /locus_tag="OCPHGMPK_01209"
                     /EC_number="3.1.11.6"
                     /inference="ab initio prediction:Prodigal:2.6"
                     /inference="similar to AA sequence:UniProtKB:P0A8G9"
                     /codon_start=1
                     /transl_table=11
                     /product="Exodeoxyribonuclease 7 small subunit"

/translation="MATKKNNFEEQLNELQEIVNKLESGNVPLEDALNEFQAGVKLSR
                     ELEKKLNDAEQTVAKLVDKDGNEKTLDPQNASAPEEE"
     CDS             complement(302364..303734)
                     /gene="xseA"
                     /locus_tag="OCPHGMPK_01210"
                     /EC_number="3.1.11.6"
                     /inference="ab initio prediction:Prodigal:2.6"
                     /inference="similar to AA sequence:UniProtKB:P04994"
                     /codon_start=1
                     /transl_table=11
                     /product="Exodeoxyribonuclease 7 large subunit"

/translation="MADNPVYLSVSDLNFYISQKFKNDPYLHKVFLQGELSNFRFRMH

SHQYFSLKDDKSKINVVMFRSFFEKLKFKPEEGMKVYVSGYVDVYGPQGSYQFYAQTM

EPAGLGALYEQLRQLQEKLEKEGLFNEDHKKRLPLFPDRIAVVTSASGAVIHDIMVTA

NRRFPHAEIDLYPAKVQGDEAADTIVASLKQIQAQGDKYDVVIIGRGGGSLEDLWPFN
```

EEKVVRQIYAMQMPVISSVGHETDTTLADLVADARAATPTAAAEYATPNLADVLTKIV

QLRARLYAAMQANIRAKRQILDRLRNAPVLQEPTRIYDQQIQQVDMLTHRLNQAMSNQ

LQHDRSGVRLLQERLKALNPGRRLEQLERERTFIVANLFSTMTTYLKDQRNKLNRTMQ

QLDDISPLKTISRGYVYTTDQEGNTVTSVDKLEIDEKLKLHFKDGQVQVNVENIRREK
DGNQEK"
CDS complement(303724..304572)
/gene="folD"
/locus_tag="OCPHGMPK_01211"
/inference="ab initio prediction:Prodigal:2.6"
/inference="similar to AA sequence:UniProtKB:Q7A697"
/codon_start=1
/transl_table=11
/product="Bifunctional protein FolD protein"

/translation="MKKLLEGKTPANEITENLKKEIKNLKNDGINPTLCVIEVGDDPA

SKIYLRVKRNLAKKVGINEVGLHFPADTSQAELLGKIKKLNQDPNINGIMVQLPVPPQ

INPRAIFETIAPEKDADGFSPLNLGRLWEGQSDIIPATVRSILTLIDYYGIKMAGKNT

VIIGRSVIVGKPLAAVLLERDATVTIAHSKTNNLADLTRNADVIISDVGKAHLVTEDM

VKDGAVLIDVGMNRENGKLMGDVDFDTVAPKAEAITPVPGGVGPLTVASLMKQAVILT
RKQHGR"
CDS complement(304620..305021)
/gene="nusB"
/locus_tag="OCPHGMPK_01212"
/inference="ab initio prediction:Prodigal:2.6"
/inference="similar to AA sequence:UniProtKB:P65582"
/codon_start=1
/transl_table=11
/product="N utilization substance protein B"

/translation="MTQHEMRRIAMQAIYLANQGNLTDAEEVCQKALKALELKSFPDY

SAEIVNGVLANKKELDEQLSKYLKKGWRLNRINEIDLAILEVALYEMQNSKAIEPVAA
LNEALNLCDEFSSKESKKFINGLLANFIKKD"
CDS complement(305022..305468)
/locus_tag="OCPHGMPK_01213"
/inference="ab initio prediction:Prodigal:2.6"
/inference="similar to AA sequence:UniProtKB:P99157"
/codon_start=1
/transl_table=11
/product="Alkaline shock protein 23"

/translation="MADNSTILLSSNDKGDETRVDLGVLEVILGIAARKVDGVSEMRG

TLKTGINTLFGRSNQGKGVSLSVEDDKLTADVYAYLDYGVNVPKVCVELQKNLTLQLK
QMTDLDLTQINVHVVGLVSETEETESEVKADTEALFPDDKEDEAKD"
CDS complement(305486..306055)

```
                     /gene="efp_1"
                     /locus_tag="OCPHGMPK_01214"
                     /inference="ab initio prediction:Prodigal:2.6"
                     /inference="similar to AA sequence:UniProtKB:P99066"
                     /codon_start=1
                     /transl_table=11
                     /product="Elongation factor P"

/translation="MTMISVNEFKNGLTIEYNNDLWRIVEFQHVKPGKGSAFVRSKLK

SLRTGAVQEYTFRSTAKVNTADIQTKAMQYLYNDGTSFVFMDTTTYEQLEIPEAQVER

ESKFLKENMVVNVIMHDGETLGVDLPNTVDLEVAETEPNIKGDTSSGGGKPATMETGL
                     VVNVPFFINQGDVLTINTADGTYVSRANK"
     CDS             complement(306140..307249)
                     /gene="ypdF"
                     /locus_tag="OCPHGMPK_01215"
                     /EC_number="3.4.11.-"
                     /inference="ab initio prediction:Prodigal:2.6"
                     /inference="similar to AA sequence:UniProtKB:P76524"
                     /codon_start=1
                     /transl_table=11
                     /product="Aminopeptidase YpdF"

/translation="MSSNQELLTLINNRIDKTTKLIKEEQADALIIFNQANYRFLTNF

SGEEAELILTAEGDRVLLSDSRFKDQIRHQAPGEMKVVMQTMDVVKEIAKQLKQLDVK

NVLVEGEFISATQFEALKEACPDLNFVLSAELVEKVRNVKDDLELATLQKAIDISVQS

FKEILPLIKPGVSERAIGAKLDYLFKMNGGDGPSFETIIASGYRGSWAHGVASDKKIQ

QGELIVIDFGSFYHGYTADITRTVALGQVEPELKKIYYIVLEAQKRGIAAAIAGNTGK

DIDQAARNYIKEQCYGEYFGHGIGHGIGLEVHELCTPAMPYGKEVMKNNMAITVEPGI
                     YLPDRGGVRIEDDVLIKDDHPYVMSQLPKDELLIL"
     CDS             complement(307322..307621)
                     /gene="rpmA"
                     /locus_tag="OCPHGMPK_01216"
                     /inference="ab initio prediction:Prodigal:2.6"
                     /inference="similar to AA sequence:UniProtKB:P05657"
                     /codon_start=1
                     /transl_table=11
                     /product="50S ribosomal protein L27"

/translation="MMINNLEALKLFAHHKGGGSTANGRNSAGRRLGAKRADGQKINA

GSIIFRQRGTKIHPGKNVGIGGDDTLFALTSGVVKFERLGRDRKQVSVYPVEEAK"
     CDS             complement(307641..307952)
                     /gene="rplU"
                     /locus_tag="OCPHGMPK_01217"
                     /inference="ab initio prediction:Prodigal:2.6"
                     /inference="similar to AA sequence:UniProtKB:P26908"
                     /codon_start=1
```

```
                     /transl_table=11
                     /product="50S ribosomal protein L21"

/translation="MYAVIKTGGKQYKVAKGESVFVEKLEVKPGDEVTFDEVILVNDG

KSTKIGTPVVEGAKVVAKVEKQGKEKKVVTFKYKPKKHSHTKYGHRQPYTKVTIESIE
                     A"
     CDS             308169..308525
                     /gene="mgsR"
                     /locus_tag="OCPHGMPK_01218"
                     /inference="ab initio prediction:Prodigal:2.6"
                     /inference="similar to AA sequence:UniProtKB:P54503"
                     /codon_start=1
                     /transl_table=11
                     /product="Regulatory protein MgsR"

/translation="MLKFYGYKRCSTSKKAQKWLDDHQVVYQYQDLVEKTPKQQDLLK

WLTDFQDRGLRYFFNTSGQVYRQMHLKDQINKMTLEEAAKLLSQNGKLIKRPLVTDGK
                     KLTCGFKEDIYEKTWL"
     CDS             complement(308520..309359)
                     /locus_tag="OCPHGMPK_01219"
                     /inference="ab initio prediction:Prodigal:2.6"
                     /codon_start=1
                     /transl_table=11
                     /product="hypothetical protein"

/translation="MTKNIKKKSWMRWLVFLAGLEIIAISINFFYGPINIAAGGSTGI

SILVDAVWGINRSITVLVVNILMLILAAIFLGKKTTKNVALGSLLLPVLMGLTPSFKI

TNNQLLAVIYGGALMGLGVSLLYRVNASSGGTTIPPMILKKYFYINPAIGLLAIDMVI

IFLNIFVDGWNAFLLAALSQVVTAMTMNYAEAGFDRKYQVRVMSNEHFEELKTMLLDN

YNGLTIYDVVGGYSDDDKKQLLLVVDTREYGPLINKIHEIDSDAFIITDTVARVHGGQ
                     WGL"
     CDS             complement(309437..310759)
                     /locus_tag="OCPHGMPK_01220"
                     /inference="ab initio prediction:Prodigal:2.6"
                     /codon_start=1
                     /transl_table=11
                     /product="hypothetical protein"

/translation="MFDYRNNLKRILNNTAGDDKSLQNSLNAIKVVLGMTGEAESTSV

NNKMDDKVIAQINTFLQGITTLIERPGDKNAKKIALGALHKLTFDHNIATNFGKKLGL

LDQAIQASEETSTAVNRKIFEDAVKPKKERNRDYRKGRRYTVIRALKGADLINNNGEV

VFEANEGQIHDLNLKSGDIVEALPLANSLNEAEVLRVVGYRKLRSRDYDKIADFKYGV

VQGSPGNLSVSRNIHGEKLVINHKPVIVALDSSYYQNGNIHLEDGAIVDLAWYTGDVR
```

```
LKKDPASAVQIRWIYETEQPKRVSKTKKAKTKVVASTQVLAKLDMNLHYQRVGIAVGD

NQNEAILEGIVNRYNGIPIPIDAFEGKKKVIESQIKDLDIVILVTAFAAHDSTWNISE
                     FASKYHVKFAVSSSKGYQAFERALYRAENGMPAYEGNQQLEYKMLKNN"
     CDS             310886..310999
                     /locus_tag="OCPHGMPK_01221"
                     /inference="ab initio prediction:Prodigal:2.6"
                     /codon_start=1
                     /transl_table=11
                     /product="hypothetical protein"
                     /translation="MTKYTVKLSEEDYQMLKDCHSKNPSIMKALEEAKKED"
     CDS             complement(311125..311304)
                     /locus_tag="OCPHGMPK_01222"
                     /EC_number="5.3.2.-"
                     /inference="ab initio prediction:Prodigal:2.6"
                     /inference="similar to AA sequence:UniProtKB:P99132"
                     /codon_start=1
                     /transl_table=11
                     /product="putative tautomerase.1"

/translation="MPFVHVELIKGRSDEQLTQMMKDITEAVHKNTGAPKEHIHVIIN
                     ELDKHTYGQGGEWRA"
     CDS             complement(311354..311911)
                     /locus_tag="OCPHGMPK_01223"
                     /inference="ab initio prediction:Prodigal:2.6"
                     /codon_start=1
                     /transl_table=11
                     /product="hypothetical protein"

/translation="MRTRENSLVSIIMTGLFAAIIYLGVWVLRIPVPALVGRPFIHFG

NTLTAVAILYLGYRNGMLAGIIGLGGFDLLNGYAATSWLTMLEVVVVASVLTAVYKGM

HYQDSKKNIIILGIIAGITKIFTTYCVSIVEALMVGTSLNVALVSSFVSLPATVINSI
                     STAICTPILYFAVRDGARRVLRKSV"
     CDS             complement(311887..312744)
                     /gene="pdxK"
                     /locus_tag="OCPHGMPK_01224"
                     /EC_number="2.7.1.35"
                     /inference="ab initio prediction:Prodigal:2.6"
                     /inference="protein motif:HAMAP:MF_01638"
                     /codon_start=1
                     /transl_table=11
                     /product="Pyridoxine kinase"

/translation="MKEKLLISQDLSCLGQVSLSVALPILGACGYQPDVLPTAILSTH

TGGFGDNTFLNLSSEMSKIIAHWQDQKIDFENLYLGYLGKEAIDFWIDHISNFKVSKV

LLDPAMADSGKLYRGLNLDYVAKMRVLANFATILTPNLTEACLLLDKKYRSFSIDEIE

ELALELKKKFNVNEVLITGIQMQDKIKIAGVSVRDTAFVIETDKVKRSFFGTGDMFAS
```

```
SLLAAILSGYSLKESSQIAAIFVKMTIKATDSTRDKRLGPNYAGALSWLMKKVEEKKV
                     RKCEQGKIR"
     CDS             complement(312741..313067)
                     /locus_tag="OCPHGMPK_01225"
                     /inference="ab initio prediction:Prodigal:2.6"
                     /codon_start=1
                     /transl_table=11
                     /product="hypothetical protein"

/translation="MIGNYITVARSKYIKGRARLTVGRIEKIRIRKSGAADWHWSRNQ

FITAEHLLNLKDSYNYLRHDYCWYNRLAIKMALIYWHNKLLQIKLNSKRYAVKKKRLK
                     LERTLK"
     CDS             complement(313244..313696)
                     /gene="yafP_3"
                     /locus_tag="OCPHGMPK_01226"
                     /EC_number="2.3.1.-"
                     /inference="ab initio prediction:Prodigal:2.6"
                     /inference="similar to AA sequence:UniProtKB:Q47158"
                     /codon_start=1
                     /transl_table=11
                     /product="putative N-acetyltransferase YafP"

/translation="MNKIKIREFQKKDFNDLCVIHDQARKQELKAANLEAAFKPLKIA

AIEEDLFSYNIYVATIDEKIVAFVAFSDDELAWLYVDPSFQKQGIGSKLIEFSLTKMK
                     RPTYLEVLTGNPAKSLYLHKGFKFLKHESGKMPGNETFHVEVDLLIYK"
     CDS             complement(313702..315462)
                     /locus_tag="OCPHGMPK_01227"
                     /inference="ab initio prediction:Prodigal:2.6"
                     /codon_start=1
                     /transl_table=11
                     /product="hypothetical protein"

/translation="MLVTLTIIFTKPALADVDYNITNVDVTAHVNANGSLLMERRITY

KFDDDAHGVFYQQNLTDNQELKNQQVRVISGKNSQPVIQSDSHANNTYELSRSDHGYR

FKVWHNVTDGDKFTIIYTYEITNAIINWKDTAELNFKIIGDGWDTDLDNVRVGIFFPS

SVKDLKAWAHGDLSGQITVNPEKGSIIMTAANVSGNEGIEVHSLFPTAVTSQNKNIRD

QNHKKYVLNQEAKFAHEANERRQRSRMLGLGALVLSGLFSLSIIIRSFTLKKSGVKPK

KEKQLPRNYDVPTVSPVMAEILDLDDKPSSRSLTAYLMELAVKKRIEIEPIKVRRKTY

YKISLIDKDLMGEVPLIKYLFNKVGDGKSFTTYELKKQRSSKLSKLFANWQKKEKQAA

INAGFFDKKVEHKKETNLVLMIILLVLSGAGIIAVFFSSEGTAGLFIVATIMLGLAIL

ATIYSNKKLSPYTAKGAESTNQVRGFKKMLDEIGNFKMREVGELPLWEEIMPYAVALG

VSKKVLKQLKIEFADEIDDTNILFWGPFYSSGRDGFANNFNSSFNSGANLNSSASGGS
```

```
                     GGFSGGSSGGFGGGSGGGAF"
     CDS             complement(315601..316785)
                     /gene="narT"
                     /locus_tag="OCPHGMPK_01228"
                     /inference="ab initio prediction:Prodigal:2.6"
                     /inference="similar to AA sequence:UniProtKB:O33854"
                     /codon_start=1
                     /transl_table=11
                     /product="putative nitrate transporter NarT"

/translation="MKEKNPQDHVIFKISLLSISIFLMMAPAIAPALPLMYHAFPGID

KAGVEMLSTIPNIGIVIGLLISPFLIKLMGEKPTIITGLVITLLAGTFPMYATAYTPI

LISRFLIGAGIGLFNSLAVSLIPQFYSSNEEKLATMVGYQNVMGSLGAALASFLISWL

LTISWHAAFAIYFLVIPVLILFILFVPLPSSRQKKTDKARKTKEKQTINGKVILIAIL

MFFIFLFYMPMSFKIPALVVQEKLGTVSEVSALTGVLNLVGIPIGASFGFFFKKLHDK

IFPLGFGLVAIGFFIIAFASNFVMLSLGCLILGIGFGLGVPYMYNWLDWSAPANSVNL

ATTIVLVLVNVGCAISPMIINAVTSSAKMGLLISAIFFACFSIYAIIHYLRVHKKQSA
                     TA"
     CDS             complement(316812..317714)
                     /gene="nlhH_1"
                     /locus_tag="OCPHGMPK_01229"
                     /EC_number="3.1.1.1"
                     /inference="ab initio prediction:Prodigal:2.6"
                     /inference="similar to AA sequence:UniProtKB:P9WK87"
                     /codon_start=1
                     /transl_table=11
                     /product="Carboxylesterase NlhH"

/translation="MMKDQEILNAVKEMRISWKENDDKRDSGLPHDIPEVTRIDDLQY

GEDPKWNLLDLYLPKNVKGKIPVIINIHGGGWVYGTKETYQFYGLGMAKRGFAFVNPN

YKLGPEVKFPKELDQVNEYIHWVADHADEYNLDKNNVFLIGDSAGGQMAEQYTAILTN

PEYREKFGYTLTDLKFRAVALNSPATFIKDPGMIGDGTLAYFDPEVMKSAKNQDLIDV

EKYITEDFLPTFISTANEDFIHDCAVRLDGFLRAKGIEVIQKSWGDEKHPEPHVFLIN
                     QKDELAKEANDEEAEFFRKHLVKD"
     CDS             complement(317806..318684)
                     /gene="soxS"
                     /locus_tag="OCPHGMPK_01230"
                     /inference="ab initio prediction:Prodigal:2.6"
                     /inference="similar to AA sequence:UniProtKB:P0A9E2"
                     /codon_start=1
                     /transl_table=11
                     /product="Regulatory protein SoxS"

/translation="MTYGKKIKQHEIVIPTDPLPVWYFIFHDHNSEKYIAPHWHRGIE
```

LSYVENGRIDDFLINKKHYSSKSGTILVVNTQEIHSIHDFKNTNSLALSIIFPYDYIT

NLYPDIAHQVIKINDPATFTDKQKLAYLHLQGLLSQFIKVYFLKIPTKYLEQQRLIDE

ILCLLLANFTEDKKDQEQISERKIYTINRLQYITQYVNNHYQEELNLALLAEKCNISK

EYLARFFKKEMELTVETYINNVRAEHAYGELKNSKKNLTQIAISCGFSSIRTMNRAFK
KLYGKSASEMKKKLIK"
CDS complement(318684..319589)
/gene="bglK_2"
/locus_tag="OCPHGMPK_01231"
/EC_number="2.7.1.85"
/inference="ab initio prediction:Prodigal:2.6"
/inference="similar to AA sequence:UniProtKB:Q93LQ8"
/codon_start=1
/transl_table=11
/product="Beta-glucoside kinase"

/translation="MKKNYLSIDIGGTNVKYAELNNAGNIIEQGKIKTSHDKEQFLKN

IDQIVEKYVKKEIKGIAFCAPGKIAHTKIHFGGALPFLDGIDFAVRYKKYDIPVTVIN

DGKASVLAENWLGSLKDMQNCAAITLGTGVGGGIIVNGKLLNGAHFQAGELSFLQLNM

KEPGFDGFAGGYASAVLMIRNVNEAIENDDETDGLAAFEAINNGNEKAKKIFDEYCKR

IAAIIIDIQAVVDLDAIAIGGGISAQPIVVQGINQAYDKVLADNELIRKTFTRPKIVE
AKFKNEANLYGALYNLFIHVNGEKL"
CDS complement(319728..321044)
/gene="pepE_3"
/locus_tag="OCPHGMPK_01232"
/EC_number="3.4.22.-"
/inference="ab initio prediction:Prodigal:2.6"
/inference="similar to AA sequence:UniProtKB:P94870"
/codon_start=1
/transl_table=11
/product="Aminopeptidase E"

/translation="MGALSEQELAAFSADFNKNDKNKVAFRAARRNGLFEASFNDEVS

KRLNHTFSTELDIGGVTDQKHSGRCWEFATLNVLRHHFGKKYHVKDFTFSQAYNFFWD

KIERANAFYDSMIRLADKPVDDREVETWFDFAGQDGGLWQMAINLVKKYGVVPSYAMP

ENATSNNTTALIDSLARKERKDALVLRKLVQEGKLEEAEKAKKEFLNEVYRMAAVALG

EPPKKFDLEYRDDDKKYHLEKDLTPRQFAEKYLKDFNWDDYVVLLNSPNYDYNKRYHQ

GLYDNVAGGQPITGLNVPIEVLARAAAAQLKDGRAVIFGNDVLKQMERKTGFLDTDLY

KTDDLFSVDTQMSKADRLATGEGSATHDMTLVGVDEDNGEIRKWKVENSWGDKYGHKG
FYEMSQKWFEEYVYDVVVDKKYLSEDLVKLWEEPAVDLKPWAHIGL"
CDS complement(321214..321579)

```
                     /locus_tag="OCPHGMPK_01233"
                     /inference="ab initio prediction:Prodigal:2.6"
                     /codon_start=1
                     /transl_table=11
                     /product="hypothetical protein"

/translation="MKKLDTNKLTEKQADLFKNNLVYIATVDENGNPQVGPKGSMTVL

DPSHMQYLEKTKGAAYENIKRGSKVALVAADVPSHTAVQVLATAHVHEDDEYAKKVVS
                     GTDTPNAFVVVLDIDEIFE"
     CDS             322278..322607
                     /locus_tag="OCPHGMPK_01234"
                     /inference="ab initio prediction:Prodigal:2.6"
                     /codon_start=1
                     /transl_table=11
                     /product="hypothetical protein"

/translation="MKMSKSKLLYVTLASSLAILSTACGAKKTDSSNQPIKFSQAVPH

KTIKKGGTLTYALENESPFTGIFSAEIADTSPDSEAAAPGDEALFDINDHYQFTNKGA
                     ASLKTKS"
     CDS             322630..324033
                     /gene="appA_3"
                     /locus_tag="OCPHGMPK_01235"
                     /inference="ab initio prediction:Prodigal:2.6"
                     /inference="similar to AA sequence:UniProtKB:P42061"
                     /codon_start=1
                     /transl_table=11
                     /product="Oligopeptide-binding protein AppA"

/translation="MKKKVRWSDGKPVTAKDLEYAYEILANPKVQTTQYTSSLENIKG

MAEYHRGKANNISGLEMPDGPNGKKLILHFKELKPAMMNSANGFFWEHAAPYHYLKHV

PFDKLVSSPQVRKHPLYFGPYKMDKTVQGQSTSWSRNPYYWRGKPNFEHIYMSNITNS

NVSQAIKSEKFDVANVLSSQWEQVKNTKGVNFVGKKTLSYDYLAFKVGKWDAKLGKNV

ENPHAKMNNPALRKAMAYAMNVDVINKRFYHGLEFRINSLIPSQFTPYYDKNIPTYSY

NLDKANKLLDKAGYKKAPGALYRSQPNGKPLVINLAVRGSGENSEAIWRNYIQQWKKA

GLNVKFLGGRPMEFNRWVAAVKASDPKIDVLEGAWGAAGDPSPSVFYGEKMPYNFARF

VSPTNTKLLNEIDSAKSFDKKYRVQKFHEWQRWMYNKAYVIPTSGAYSVTAVNSKVTG
                     WSLKPSANVWYEAGFSK"
     CDS             complement(324119..324556)
                     /gene="hmrR_2"
                     /locus_tag="OCPHGMPK_01236"
                     /inference="ab initio prediction:Prodigal:2.6"
                     /inference="similar to AA sequence:UniProtKB:Q9X5X4"
                     /codon_start=1
                     /transl_table=11
                     /product="HTH-type transcriptional regulator HmrR"
```

/translation="MESKKILHNVFQNIEVGIGEVSKVVGVSQRQLRYWEEKGYIKPV

SDDNSGVRRYNLSTLYLIVFIKEQLDKGFTLAAAFERSKDIRVKSKIVRTFFEHTFND
                     VKITNPDKGYGEIDLGELRTDDGKMYHFKGVVDERGRYVKIDE"
     CDS   324729..326201
                     /gene="emrB"
                     /locus_tag="OCPHGMPK_01237"
                     /inference="ab initio prediction:Prodigal:2.6"
                     /inference="similar to AA sequence:UniProtKB:P0AEJ0"
                     /codon_start=1
                     /transl_table=11
                     /product="Multidrug export protein EmrB"

/translation="MDQQPTDIHGKAYNRNLLVLVLIIGSFCTVLNGTLLATALPSIM

RSFHISTATAEWLSTAFLLVNGVMIPVSAWLINRFGSRKMYLTAMSVFFIGTVTAAVA

PNFGTLLTGRIIQGLGVGVTMPLLQTIMLSIFPANKRGAAMGTVGIVIGLAPAIGPTL

SGWIVDNLSWRYLFSIIAPIAGIVIILAFFLIKDVLPTKKEKIDVWSVTTSTIGFGSL

LYGFSEAGNKGWTNPEILGFIGVGIIFVILFGMRQLKMADPFLDITVFKHFEFSLAAA

LSGITNLAMVGIEMVLPLYIQNLRGVSAFHSGLMLLPGALMIGIMSPITGRLFDKYGA

RKMAITGMTLLTLGTIPFVFLTAESSYTMIIVLYAIRMVGVALVMMNVTTSGMNSLPL

NKISHGTAVNNTFRQVLSSIGTAILVSILTTTTNNHMPAKELLHTLPLQYKNQAINAT
                     LDGFRASFAMSILFALVALVLSFFLKKGNRARENQEEVNG"
     CDS   326201..326761
                     /locus_tag="OCPHGMPK_01238"
                     /inference="ab initio prediction:Prodigal:2.6"
                     /codon_start=1
                     /transl_table=11
                     /product="hypothetical protein"

/translation="MIIVILILAVILFIYFNVIPGKGHSLVSWISLIITLLCVLGIVE

HDYNHWGLKTETQTSTQNLTSSVNPNLPILLYQPLGNGTEKVYLYKTNNNQKKPKAIK

LDKVSTKVKQGKEASLKIRTTRYVYKDNFSRIMFSAFSHNNELKHREYIFTLPSNWKV
                     ISTKDMQKLQKQMQEKMQSQKAASLH"
     CDS   complement(326804..327469)
                     /gene="nfnB_2"
                     /locus_tag="OCPHGMPK_01239"
                     /EC_number="1.-.-.-"
                     /inference="ab initio prediction:Prodigal:2.6"
                     /inference="similar to AA sequence:UniProtKB:A0R6D0"
                     /codon_start=1
                     /transl_table=11
                     /product="Nitroreductase NfnB"

/translation="MMNVEEAILTRHAVRLFNPDDKISRESLEKIVELAQHAPSWVDS

```
QPWKVYIATSNTLAAVKKRHAENVKNGVKANPDWPTTHREDWAKFPRENMLKHNEATA

RFWENPELSSMTRQDLQTRLYDAPAICYLTIPKDSNQWSDYDLGAFGQTLMLAARGMG

IDSMPAYEIVKFPESVKEIMNIPDSEWLAMGIALGYADMATRVNEYRAPRVPLDEMLK
                     IKD"
     CDS             327743..328873
                     /locus_tag="OCPHGMPK_01240"
                     /inference="ab initio prediction:Prodigal:2.6"
                     /codon_start=1
                     /transl_table=11
                     /product="hypothetical protein"

/translation="MKKYQFLSFRIADIGDYLKVFACTAVMAQPIMALVMDIKQPTHT

QDVFGFLYNLIKYTAPAFIFGILYTTIRTYDLKKHFSYRKYFRSNWSNLFVPTIWWTS

IYLLEMPWEQQVNKYYNFPTFCWQFINGNAAPHLWYNTMMLQFIILMPLFWAISRYVG

KNTKRGIIIAIVTFILYFTWLGFYDTYVFHGIHQNDWYLLDRVFISFFIYGVYGVLAW

QLRDYVNAFLTNFWWLLSIIFIICFIWTNIELQDFGHPINFNNAPYYKPSMTLYSLAV

IALIAAFCLHQVKKNSQTSLKVFHFLAIYAYRAYLSNVFWNQLVWRGLNMSFHAHYHP
                     FLTFFGTWIFTWCLSFASAYLLHLCWSWVKENKLSRISFLFN"
     CDS             complement(328920..329507)
                     /gene="vbhT"
                     /locus_tag="OCPHGMPK_01241"
                     /EC_number="2.7.7.-"
                     /inference="ab initio prediction:Prodigal:2.6"
                     /inference="similar to AA sequence:UniProtKB:E6Z0R3"
                     /codon_start=1
                     /transl_table=11
                     /product="Adenosine monophosphate-protein
transferase
                     VbhT"

/translation="MIEERKDTEWYRRKFLYPNGTLKNKLNIKDAAELAQKEYLGSAV

RALAFLRKNKKITSVEDLKKIHKIMFGWLYDWAGQIRDYELIKGDTEFLEYTRIKFGI

EEIDEKMRQLASQKELANTDYAFLLDRLNYLHPFREGNGRSSKLFLQAFAANHGQVID
                     YPRSNKELIIAQNNADINQIARLIKVKSKSKRIKC"
     CDS             complement(329500..329919)
                     /locus_tag="OCPHGMPK_01242"
                     /inference="ab initio prediction:Prodigal:2.6"
                     /codon_start=1
                     /transl_table=11
                     /product="hypothetical protein"

/translation="MLSKISILLTNHQLTLKDISQNQHLAEQDLRKALDENPDNWSSS

VLSALSSSLNMRPGDLLELLDPVYSLKINDHSQMIQGIYIEDPEIYEQIRFVVESEHL
```

```
                     EGWQPDEEDILRLLDEALNPSPTFQTEISQIWGGEDD"
     CDS             complement(330055..331866)
                     /gene="glmS"
                     /locus_tag="OCPHGMPK_01243"
                     /EC_number="2.6.1.16"
                     /inference="ab initio prediction:Prodigal:2.6"
                     /inference="similar to AA sequence:UniProtKB:P0CI73"
                     /codon_start=1
                     /transl_table=11
                     /product="Glutamine--fructose-6-phosphate
aminotransferase
                     [isomerizing]"

/translation="MCGIVGVVGKPARDIILNGLTNLEYRGYDSAGMYLNDLNGNEYL

TKAVGRISNLKEKLTPDEQGLVGIGHTRWATHGKPTVDNAHPHFDETKRFYLVHNGVI

ENYVELKEKYLQGVKFHSNTDTEVVVQLIGKIAREKNLDGFSAFKEALKLVKGSYAFL

LVDNTEPDHVFIAKNKSPMMLGLGDGFNIIASDAISVLDQTKTFVDLQDGDVGDITKD

SYTIETVDGKKVERKPHVLDIDPNAASKGTYEFYMLKEIDEQPSVMRKISQTYFDENG

DVKVEPQIIDALSKADRIYIYAAGTSYHAGLVGKTLLEHYTGIPIEVGLASEAGYHFP

MLSKKPFFIFLTQSGETADSRVVLKEVNKRNIPSLTITNVEGSTLSREADYTMLLEAG

PEIAVASTKAYTAQIAVQAVLAKALGEALNNQDAKDWNLKHDLAIAAEGMQQLVDSKE

SLKEIADKYLIKSRNAFYIGRGIDYAVALEGALKLKEVSYIQTEGFAAAELKHGTISL

IEKGTPVIALINDPATADLTRGNIQEVVSRGANIITIVGKDFAREGDDVVLPEINYYM
                     SALLTVVPAQLLAYYASKDKGLDVDKPRNLAKSVTVE"
     tRNA            complement(332047..332121)
                     /locus_tag="OCPHGMPK_01244"
                     /product="tRNA-Arg"
                     /inference="COORDINATES:profile:Aragorn:1.2"
                     /note="tRNA-Arg(tct)"
     CDS             complement(332208..332903)
                     /locus_tag="OCPHGMPK_01245"
                     /inference="ab initio prediction:Prodigal:2.6"
                     /codon_start=1
                     /transl_table=11
                     /product="hypothetical protein"

/translation="MKIIKCVFKWLGIAICLFLIFAFMQVPILESTPVYSIDSQQQFI

EAFKHVNMFHNIFMIIIFTIITFIMEWYVCKWLNNKLDFSKKLSRKNLILSLGAGVFC

FILQLLTMFSVFVNTKPDVFTETLKTGLAVPLILSLTLIGPTLEELLFQAGIQKGIFK

RLNPWIAIILTAIIFAGAHNVTLNWSFLNRFLSGVAFGYVYQKTDDIKMAILSHSISN
                     LLPLIICCMQAWS"
     CDS             complement(332949..333719)
```

```
                     /locus_tag="OCPHGMPK_01246"
                     /inference="ab initio prediction:Prodigal:2.6"
                     /inference="similar to AA sequence:UniProtKB:Q5XAQ1"
                     /codon_start=1
                     /transl_table=11
                     /product="Putative bifunctional
                     phosphatase/peptidyl-prolyl cis-trans isomerase"

/translation="MIKTIFFDVDGTLVSHKTDSVPKSTRKALKLLRENGIKLVLATG

RDMSQLKKLPVKDIKFDGYLTMNGQLCLDENGNELFGNPIDEKDTEKMIELIKEEAVP

ITMIGKEGPYINFVTDQVVAAQKAVSSKVAPVGKYHGEKVYQFIAYGKRDDLKKLVDN

LPNCKLSWWNEYAVDIISKDGGKLAGVEKYLKLQGQTLTDAVAFGDGENDLDMLKAVQ
                     LGIAMGNGEEQVKQIADYVTSDINEDGIYNACKHYKLI"
     CDS             complement(333807..336341)
                     /gene="pepN"
                     /locus_tag="OCPHGMPK_01247"
                     /EC_number="3.4.11.2"
                     /inference="ab initio prediction:Prodigal:2.6"
                     /inference="similar to AA sequence:UniProtKB:P37896"
                     /codon_start=1
                     /transl_table=11
                     /product="Aminopeptidase N"

/translation="MAEVKRFYETFHPDHYDIYLDISREKKSFHGKTIVVGDAQEELV

KLNQKYLKITSVRVDQKKADFDYNDKEEVVNIKAGKVGKMKIEVDFEGKLTDSMMGIY

PSYYEVDGEKKQLVGTQFETTFARQAFPCVDEPEAKATFALAIKFDEKPGETIISNQP

EEKFKDGVHYFKPTVRMSSYLVAFVFGDMQKKLTKTKSGVEIGVFATKAHKAKELDFA

LDIAKRSIEFYEDFYETPYPLEHSYQVALPDFSAGAMENWGCVTYREAYLLLDPDNTS

LDMKQLVATVIAHELAHQWFGDLVTMKWWDNLWLNESFANMMEYVAIDALEPNWKIWE

MFQTSEVPAALQRDATDGVQSVHVMVNDPAEIDALFDSAIVYAKGSRMLVMVRALLGD

KALREGLKNYFAAHKYSNATGDDLWNALGKASGLDIGAIMHSWLEQPGYPVVNAKVED

GKLVLTQKQFFIGEGKEVGRKWQIPLNANYAAVPKIMKDEELVVGDYKNLLANNGTPF

RLNVGNNSDFIVKYDQTLLDDILSHVDELDAVDKLQLLQDFRLLAEGGQMSYADIVPL

LPKFADSTSPIVNNALYRIMATLRNFVTPGSDEEKQLKKLYDLLSEKQVKRLGLLPKV

GESNDDNLTRPYVVAASLYADNDDTINGLHAIYSENSDNLEGISADIRSAVLVNEVKN

FGNMTLFDKLLKKYQETSDASLKQDLCAGITSTKMPEIIDAIVDDFENAQVVKPQDLR

AWYRNVLANPFGQEQAWNWIRLEWPWLEATVGGDMEFATFITVTANIFHTEERLKQFK
                     DFFEPKINTPGLTREIKMDTKVIETKVNLVKKEQAAVNAAIAKAVA"
```

```
     CDS             336536..337444
                     /locus_tag="OCPHGMPK_01248"
                     /inference="ab initio prediction:Prodigal:2.6"
                     /codon_start=1
                     /transl_table=11
                     /product="hypothetical protein"

/translation="MKKKVKIFILVCLLFIGVSVTTTNTNFSQPAFASMGSTGGGHST

GGSSGGGTSYTSSDSSDSNNSSIGGIVGGFAILVLIFFSSYIIAALFDFIFKIRLRFE

YDSNYLIKNSGDNNYKLFYPNQRFDKYQKLLHRLHIKFTKPDPKDDLTAYKEVYIQAQ

FLYSQLLREKYVNSKYQVKSLKQYLDRNYYKAMVKEIELKRNAGTVDETVINSVIFEE

VAHLDKSLLLAKLTVLGKDKEIQFNQDFDASFSRNKWTDYVLFGLEDNRNTKIINLVY
                     GEHFHLNGEDFNDYQSGNEYTENKIN"
     CDS             337567..339951
                     /gene="pepX"
                     /locus_tag="OCPHGMPK_01249"
                     /EC_number="3.4.14.11"
                     /inference="ab initio prediction:Prodigal:2.6"
                     /inference="similar to AA sequence:UniProtKB:Q59485"
                     /codon_start=1
                     /transl_table=11
                     /product="Xaa-Pro dipeptidyl-peptidase"

/translation="MKYNQYGYLQTDFDQMIKELESINFLPDNWKENSFSDLLATLVE

NSVAEAKTYGAKEAKLAEFAVSENETLKDFLAKDPASISRNSFYNIALQLLGYHVGYD

YDLKDPLARMKANALPYTEKEEIDRNELIKVFYRLLNTRAKNGQTFIDNMAGKGYFTQ

FYGNNKFMYFNGKSLPVFDMSKVIREVVYVESDLDTDEDGKPDLLQVTVFRPYQSNNF

KFPALYTADPYFGGIIANEKRNHNVDVNLEDATKSTYPKYEAMPTAQAKKPASEDQAA

TEEAVHKAAYSLNEYLLARGFANVYAGAIGTRGSDGLRITGAPEETESAKEIIEWLHG

DRIAYTDRTRKHETKASWCNGNIGMTGRSYLGTLQIAIATTGVAGLKTVVSEAAISSW

YDYYREHGLVIAPEACQGEDLDMLAETCQSNLWDAGDYLKIKPKFDAMQKRLLEKSDR

ETGQYSDFWEARNYRHHTDNIKCSWISVHGLNDWNVKPKNVYKVWQKVSKLPIAHKLF

LHQGPHYNMNNLLSIDFSDLMNLWFCHELLGIKNNAYKQWEDVMIQDNLQPDIWHEEP

TWNNELGKKEIYFPDKKGKLLKDGGEDNDKLSFTDQGGKVFKEAKISEGKWQYDFISG

EEKWLDQQLRFVTDEFIHPVTLVGRPKIKMSLATSLPKGQISVALVDLGSRKRLTPIP

KSFNDQIQELGYRFGTEVMKEFVPDKETKAKLITKGHMNVQNYADMKHAEKIEAGKFY

DLEFELQPTFYRLPAGARLGLIIYSTDQGMTKRPLEDETYTVDLDKTQLIFHEM"
```

```
     CDS             complement(340081..341355)
                     /gene="tcrY"
                     /locus_tag="OCPHGMPK_01250"
                     /EC_number="2.7.13.3"
                     /inference="ab initio prediction:Prodigal:2.6"
                     /inference="similar to AA sequence:UniProtKB:O69729"
                     /codon_start=1
                     /transl_table=11
                     /product="putative sensor histidine kinase TcrY"

/translation="MIQKFRWKFIGMSITALFVVLVITLGTLLGISYTQSHNEVDRVL

TTLINNQGQLTPHNAKPVFGNQKDPINKNFLAGEYNPEAIFQYRYFTVASTKNNTPKI

INDANVYDVAQKKIINTSKQVFKDKVTSGSVSIGNNQYAYRVGKDQKDNQFIVYLNES

LIYHRFSLLFRVSVVLGLIALIVFALILILVSKKAIGPIITTYHKQREFITNAGHELK

TPLAIISANTEMEEMLGNNSEWNKSTKEQVDRLTRLINRLIALARTGETGEVVLNKVN

FSEIVKKNVTSFKSVMQKNDLKYNAIIMPDLYVKAERNILSELTNILLDNARKYCDKD

GEVKVSLTKGKLGTNAILRVANTYKEGKGKDYSHFFERFYREDESHNSKKSGFGIGLA
                     MAQEIVQTFHGKIHVSHKDEMIVFTVILRLAK"
     CDS             complement(341365..342030)
                     /gene="tcrA"
                     /locus_tag="OCPHGMPK_01251"
                     /inference="ab initio prediction:Prodigal:2.6"
                     /inference="similar to AA sequence:UniProtKB:O07776"
                     /codon_start=1
                     /transl_table=11
                     /product="Transcriptional regulatory protein TcrA"

/translation="MKILLAEDEEQLSRVLVAAMKSVNYDVDAVFNGRDAVELAKKNS

YDVIILDIMMPIMDGITALKQIRKSGDKTYVLMLTAKAEIDDRVTGLDSGADDYLTKP

FSLKELLARLRSKERRDDQYTPNEVEVGDLALNVSEQELVSHNSIRLGSKETQLLNYL

MLNEGKEFSTSDLLAHVWKDEDDANEEVVWIYISYLRQKLQSIQSSVQIVGEKGGSFS
                     LIR"
     CDS             complement(342035..342646)
                     /gene="ywaC"
                     /locus_tag="OCPHGMPK_01252"
                     /EC_number="2.7.6.5"
                     /inference="ab initio prediction:Prodigal:2.6"
                     /inference="similar to AA sequence:UniProtKB:P39583"
                     /codon_start=1
                     /transl_table=11
                     /product="GTP pyrophosphokinase YwaC"

/translation="MDIYGGYAPVLDKLLNQMLEYFRKENRIHQEQTGDSLYEHLIGR

VKRPESMIEKCQRKELPVTPESAMKKIRDSVGIRVVCNFIDDIYTCIDLIQDWDDVVI
```

VKQKDYITNAKPNGYRSYHMILDVERPEKDIEGNTPGHYYVEVQLRTIAMDTWASLEH
EMKYKHKIKNPEMIGKELKRVADELASCDVSMQTLRHLIREED"
CDS complement(343240..343650)
/locus_tag="OCPHGMPK_01253"
/inference="ab initio prediction:Prodigal:2.6"
/codon_start=1
/transl_table=11
/product="hypothetical protein"

/translation="MTKYTVKSELTGTPWQIENRTKKHKFIVDESASGKNVAPNPVEY

LVGAVNSCISISAGMIANVHHLDVKNFKVTNQAITTKLGHGKSVVSEMLITVSFDSSM
SKEEKQDFLAHTLHVSTVYQTVSQSIKTYVELED"
CDS complement(343664..345211)
/gene="opuBB"
/locus_tag="OCPHGMPK_01254"
/inference="ab initio prediction:Prodigal:2.6"
/inference="similar to AA sequence:UniProtKB:Q45461"
/codon_start=1
/transl_table=11
/product="Choline transport system permease protein
OpuBB"

/translation="MIKSLWQMLITEHDQIWATTLVHIKISLIALLIAMVIAIPLAFI

LKGHKKAAEFTLQIAGIIQTIPSLAILGLLLPFVGIGTTPAIIALVLYAIMPIFQNTY

SGLTDIPDNLEEAATAFGLTKWKKLQRLEIPMAMPMIFAGVRIALVMIIGTATLAALI

GGGGLGTYIYVGINSNNNTEVLMGAILSALLALVFSGVLKFIAKNNKRLKWGSIIIAG

ILLIWGGSSLYSHFTSTSKNVAEPKQTITIAGKMGSEPAILINMYKDLIEKNDPNTKV

VLKPNFGGTTFLFKALQTDKVDIYPEFTGTVLQTLVKTGKKTGNDPNQVYVEARSDLK

KQFDMEYLKPMAYQNGYALATTQKFAKDNHLTKMSDLNRVNKKVHAAFDPDFTSLKDG

YPGLKKIYKLDFASIKSTESSIRYHAIANNQANIVDGYTTDAEIKKYNLVMLDDDKKF

FPPYQGAPLMKAKFAERNPRIVKALNKLAGKITTQEMQEMNYQVNVKHEKPAEVAHDY
LVKHGLI"
CDS complement(345204..346163)
/gene="opuBA"
/locus_tag="OCPHGMPK_01255"
/inference="ab initio prediction:Prodigal:2.6"
/inference="similar to AA sequence:UniProtKB:Q45460"
/codon_start=1
/transl_table=11
/product="Choline transport ATP-binding protein
OpuBA"

/translation="MIEYKNISKKYQDQEILKDVSFTVKKGDIFVLVGPSGSGKTTLL

```
KMFNRLIEPTKGEILLDDKSIKDYDLRKLREKTGYVLQTASLFPNLTVGENITIVLEQ

EGVSRKERRKKQEELLKSVGLDPKLYCDRMPNELSGGEQQRVGIIRALAANPEVVLMD

EPFSALDPVVRKQLQDLLLNLHDQLNKTVIFVTHDMQEAIRLGNTIGVLHKGVLEQVG

KPREILRHPATKFVREFFAGNRINQKLDLETLLKSELGVDPRFYPKVETLVRYHVYSV
                     QDLIKDCSNHPDSLFIAETEFGEFLIEPKRVWNFFLKWVAGND"
     CDS             complement(346291..346707)
                     /locus_tag="OCPHGMPK_01256"
                     /inference="ab initio prediction:Prodigal:2.6"
                     /codon_start=1
                     /transl_table=11
                     /product="hypothetical protein"

/translation="MKRKNPLVLDTKQHAIILNASGSIGEVTPRLQGFFDLIHDKIST

KDEFIEKLNREVKKFGTDPGRRKELMDYQMRLDDEREFGKEQGKQKEQISSIKRLIKM
                     ARQFKASDDFILKQLMEDYGDNFSKSELQEFIKKNK"
     CDS             complement(346704..347123)
                     /locus_tag="OCPHGMPK_01257"
                     /inference="ab initio prediction:Prodigal:2.6"
                     /codon_start=1
                     /transl_table=11
                     /product="hypothetical protein"

/translation="MRKNENELVAKITDDPVFSWVMEQDDNCCKLLQAILPELNITRV

EFETQKRLKFHPEKRGTIPDILAYDQEGRVYDIELQVLQNKFLAQRIRYYFSMIDQNL
                     LMSGGKYRDLPETYVIFILPNDPYDHDEKTTNLPFSS"
     CDS             complement(347243..348151)
                     /gene="gtaB"
                     /locus_tag="OCPHGMPK_01258"
                     /EC_number="2.7.7.9"
                     /inference="ab initio prediction:Prodigal:2.6"
                     /inference="similar to AA sequence:UniProtKB:Q05852"
                     /codon_start=1
                     /transl_table=11
                     /product="UTP--glucose-1-phosphate
uridylyltransferase"

/translation="MKVRKAVIPAAGLGTRFLPATKAMPKEMVPIVDKPTIQFIVEEA

KKSGIEDILIVTGKNKRSIEDHFDANPELEQDLEEKGKTELLHLTQSITNLGVNLYYT

RQPHPAGLGDAILRARSFVGDEPFAVMLGDDLMDDKVPLTKQLIDRYDKTHASTIAVM

PVPHEEVSKYGVIDPDNEIEPGLINVKAFVEKPDVDKAPSDYAIIGRYLLTPEIFDIL

AHQKPGRGGEIQLTDAIDTMNKTQRVFAHVFNGERHDVGNKEGYLETSIEYGLSHPQI
                     KDDLRKYIIAMGQKLQNEDKKTNKKK"
     CDS             complement(348210..349133)
                     /locus_tag="OCPHGMPK_01259"
```

/inference="ab initio prediction:Prodigal:2.6"
/codon_start=1
/transl_table=11
/product="hypothetical protein"

/translation="MKSFLNQTRNRTANFIQLLSKHISQGEINQNSIIIAYYVLLSIF

PIIIIIGNVLPLFSIDTKPIARYLELIFPSQVSSLIMPIVNALLKKHSSGFISLGIVV

AIWSLSCLVNSIRLGANKIYGVDKQEKGKSWWNYLLARTFTFALSVILVVGFSIIIFI

FTFGRQIMEFLAPIFNLSLWWVYKLENYRWPIIILMTVIVNLYINYVIPNIRVQKRVV

WPGVWFTVISWSALSYFFGLYLRQFGTRWQNYGIVGSFIIFMLWLNVGSFLLLLGICI
NAAGDELKGRKIEYSQSPILRIFRKNKELPK"
CDS complement(349149..349973)
/gene="map"
/locus_tag="OCPHGMPK_01260"
/EC_number="3.4.11.18"
/inference="ab initio prediction:Prodigal:2.6"
/inference="similar to AA sequence:UniProtKB:P19994"
/codon_start=1
/transl_table=11
/product="Methionine aminopeptidase 1"

/translation="MITIKSKRELQGMQKSGRVLAAMFEGLRDVIKPGISTWEIEEFA

QDFMKKHGGRLSEQGFEGYKYGTCISVNDEIAHAIPRKDKILKEGDLVSVDVTCNVDG

YETDSCTTYGVGKISAEDQKLMDVTKKAMYMGIDQAVVGNRIGDIGSVIQNYVENENG

YGDVRELVGHGIQPSIHEDPEVPHWGKAGHGLRLREGMTITVEPMVEAGGDWRIEQKT

VSDPNDDWVYYATPDGSKSAQFEHTFAITPDGPKILTLQKPYDGYEKYLPHFLDED"
CDS 350160..350606
/locus_tag="OCPHGMPK_01261"
/inference="ab initio prediction:Prodigal:2.6"
/inference="similar to AA sequence:UniProtKB:P00323"
/codon_start=1
/transl_table=11
/product="Flavodoxin"

/translation="MKAQIVFASMTGNDEDMAEILEENLQDAGFDVENIDVSFADASS

YLDADLCVMVTYTYGEGVMTDELKDFYDQLVTLDLSGKKFAVMGSGDKTYKDHYCENV
DDFEKAFIKCGAVEVAKPVKIENAVDDEDIDLIDQATEEIVEAFND"
CDS 350599..351108
/locus_tag="OCPHGMPK_01262"
/inference="ab initio prediction:Prodigal:2.6"
/codon_start=1
/transl_table=11
/product="hypothetical protein"

/translation="MTKKHHKRKGQKITQAQLRELGSKLVTRHRNFYVYVIFGFLAAV

INIACFLLFHNVWKIPVIYANTVAFIISNLASFSFNKHGVFIQNVDKQHGVVYQLCLF

FIYRIISLIPDNLIMLVGISWLHWNALFVKIVDQILVGIFNYLTTKSIFLNTSNKFAK
RFKDHFNKK"
CDS complement(351146..352288)
/gene="mnaA"
/locus_tag="OCPHGMPK_01263"
/EC_number="5.1.3.14"
/inference="ab initio prediction:Prodigal:2.6"
/inference="similar to AA sequence:UniProtKB:P39131"
/codon_start=1
/transl_table=11
/product="UDP-N-acetylglucosamine 2-epimerase"

/translation="MKKIKVMTVFGTRPEAIKMAPLVLKLKQDERFEEITVVSAQHRE

MLDQVLDIFKIKPDYDFNIMHKNQTLEDITSKVLIDMAKVIKTERPDIVLVHGDTTTS

FAAGLATFYEQTTLGHVEAGLRTWNKYSPFPEEMNRQMTDDLADLYFAPTELSKKNLI

KENHPSDNIYVTGNTAIDALEQTIKKDYHHDVLDEIKPGNRAILVTMHRRENQGEPMR

RVFKVMKQVVDSYDDVEIIYPVHLSPRVQEVAKEVLGGDPRIHLVKPLDVVDFHNLAK

RSYFIMTDSGGVQEEAPSLGKPVLVLRDTTERPEGVEAGTLKLVGTEVNKVHDEMIRL
LEDKNAYDEMANAKNPYGDGKASDRIMDAIAYYFDKEHNQKPADFR"
ORIGIN
1 cctacgctcg cctcgaaacc gttgagggtc gtggcatagg cgtcatttac
ctaggcaact
61 attatataca atagccaata atggactacc gcctaggcta tcaagcaatc
aggattcgaa
121 cctgagtcag ataaataaga aaaagtaaaa aataaaagaa ttgtaagcta
aaaactaaaa
181 ataaaagtca tttagaggaa tgtttatcgc ctgagtttag tcaggctata
accgccagtc
241 gaattgaacg actgctaacg tctaccaaga acggttactt ttaatctgtt
acttaccgaa
301 caaattaaga tcttgtatta atttcgaaag gagatattct ttcaaacgta
tcgcacgtag
361 tactgtcaag gttaagtcga aagatcgtaa ccaccacgtc taatctttcg
acaataacaa
421 tttatcatga aatgcacgca agcagaacgc aaggtttacg caagctttac
gcacgactaa
481 tttttccgaa tcttaatttt atccggccat actgttttta ctttttttggc
tgaacactcg
541 taggtcgggt aaatcatcaa cgccgaaata tcttttccag taaagccaac
gatcagcgaa
601 ctcgcattgt gcgttaatct tcttagtatc aattgaccta gtcgacagat
taactgtagc
661 tgctacatca acgatacgta gctggtcaat gtatgttcca attaagatac
gtctgtaagg
721 ctttaaagct gtatcagtgc aattatccat cgtccgataa atcgctgcac
agacctttct

781 cacaggatca gcaatatcaa tgtcgtcttg tgcttcgtca atgaaattat tctcaacacc
841 attcttattt gttgatcccg gtgcaaatga taattgaggg cttgtaagtt gattacggtg
901 taagccagct aaattaagat agctctgaaa attaatagtc aagaacttat ccacccttt
961 agctgtagct cttaaattcg gctgtaatcc taagtcaatt tgatacacac ttacactccc
1021 tctcatcttc tagttgttta gttacttgct ttattcgctg ctaacatatc agtaaatatt
1081 cgatccgtga tctcatgagg atcttcatct tcggggcaat gatgcttagc atacttcaag
1141 atgttcaagt acatcatgac tgctgtttca ttgcctactt ctaaatcgtt cctagtttcg
1201 tccatcattc gacctcactt tgatttcatg ctcaatcaaa ggcagcagct cctcaacttt
1261 atcttcgtca atcatttctt cgattgttgg ctctataata ttttcaaaat tcttgatgtt
1321 tgatttaagc attgatctaa aattcttaag ttgttctgtc ttgaaacatt tgtaattgca
1381 agtattaact tcgtatccat acaatttcat agccaaggcc tctctttatc ttctttagct
1441 actcttaagt cccagattcc atcacccatg tatatcaaaa tgtcatcgaa actgaaagaa
1501 cacatatagc cgtatttagt atgaactccc caaacgcaat tgactgcgaa ctttcttgga
1561 tcaaacttca ataaaccgtt atcttctaaa aaacttctag tatatgtgtc ctcaacctct
1621 tctctcgttc ctctagtccc aatcttcaac tcaattgcca taattatctc tccatttctt
1681 gtaatactct tcaacttctt tgtcatagtc tactggctca agtgaaccgt cttcttgtac
1741 gtgataccac ttgccttttt gatactttaa atcgttgctc ataggcaaaa caccacaaac
1801 ccgacagcac ccagacaaat cagcatccct gcaatgaata tcaattccgc actatgcttc
1861 atactgtctt aactcccgaa ccatagcttg cgttgctact ctttcgtcac tttttagcca
1921 atttgcgctc cagtctaaag tgtcaagctc aaaactcaaa gaacagttat gctgctcagc
1981 aatctttttc gctaacttta aagctaattt cttatcgtta gcgatgcttg catctacaaa
2041 tttgcttacc aaactcaaat caatttttttg catttaatca cccatatctc tcttaatcct
2101 tacgtcaact cttgcacgtt tggcatacct ctttttgact agcaaagttg ttacttgctt
2161 gtcatcgtga taaacacctc tcataacttc aaccattttg tgaagtcgtt tatcacgctt
2221 catttttggg ttcatgccgt ccattatgat ttttcctacg ttatcagcat caggtttctt
2281 agttggcagt tcttggttag ataaacataa agccttacgt ttcttgctta aactcttcgg
2341 gacttcaaaa tatgccatga tcttaacgtc taacggctca tctttatcaa atatgccttt

2401 gaagctatta atcgctgtat acctaactaa atcttcatat cgtgccgtct tagctggcgt
2461 gtaagttacc gttctagtaa ctctcggtct cgccttacca attggcggtc cttcaatcgt
2521 aaagttaact ctcataaaac ttattcaatc caatcgtcat aaattacaat ttttgggtca
2581 tcatcactat cacacatcac gctcatagta tctttggtcg ttggcttcga ttcaggaaac
2641 gaaatgatta aatcatgatt tactatcttg tgtaaaaggc gtaaatctga aagattttta
2701 atgtaaatct tatatatccg atcacttaat tcgtcctttg ctttcaccaa cttaaaaccg
2761 gctttagcaa gtgcatttaa atatttttga gacatagaac catcttcaaa tatgtcattt
2821 acagaccata tttcaaaaat catgtttata aactccccat atacaaaatc aatatcccta
2881 gcagaattaa aaatgctgct gcaaaaatcc aatctgacat gctacttatc ctcaattaaa
2941 cttcgtaagt aataattgct tcgtagtttg aattatttga tgtgtattga atatctataa
3001 tttttctatt taattgatta gaaaaacgtt ctatatcaaa ttcaaactca taattttcgt
3061 attttggtct agtaataatc tttgttttaa tcattcaatc accagttttg cgtttacttt
3121 ttcaacgtat ttccaattag atttcaatat tgaaattaat tcattaacac tacttgttcc
3181 gctaacattt ggttcaggaa aacctccatc tattgataat ccacgtattg catcatcatc
3241 aagaaaagca atgtaatatt cattctcgcc tgttggtcct acaattaatc cataattagc
3301 gctttctttt ttatcgttgt aataacaaac tacatctccg actttccact cttcatcttt
3361 attttcttta cgcttatcta taacttccat ttgcttaacc tccaactact tctctaacca
3421 ctccatgaag caccttagca acctcactgg cttctttctt gtctgtgaag atagactgaa
3481 caaatccagc tgaacgtcct gtcgtgtcta aaatttcaac catgtacata tcaggaactg
3541 agtaccagcg atattgttta gcgtcttcga tgatcttctc taagttgtga ttttcttcaa
3601 gcattacgca atgtcctctc tttcaatcaa cggtaggacg ttgttttctt tcaaaatatc
3661 gtagattaag cgtctaccct tttgagtcca agcagtaagt ggcttagcat gatccttacc
3721 gtgcttgtcg gtgtatgtgt gaagtttcgt tgtggtgtac ttcttgccca tgtatgcctt
3781 atacaagatc cactgcccat tgactttatg ctgaatgcct agtgcatgta gcagtttgtt
3841 aaacttaaca gctgtataac cgtaatccat agcaatttga gtagtaacca ttgcgtcagt
3901 agtaccaaga atgacatcta agtaactagc tttcttgttg ctctcttcaa gttggcggtt
3961 taagctcttg ttttccagct taagttgcaa gttttcactg tgcagaatat ccatagcacg

4021 ttgaacaaca ttctgtggat cattccactt cttttcgatt tcgattagat actcacggta
4081 ttctttaccc tttttggttc tgcttaaaag acacagctgc ttagccatat caattgtgag
4141 tgcgtaatct tgaagctctc tagcttgcac accaccgttg ttttgaacct ccgtacttat
4201 aagtacactg gtaaaatctg aattttcttc aaaaccattg ctgttctgtt cccaccaagc
4261 ggaaaatctt cgcttgatac ctagtccttt atatagctct cttgcactaa ctagctgttg
4321 gtcattcttg accgttactt tgattaattc gttattcatc ttcatcgtcc tcatcttcgc
4381 atagcatatc cgtaatacgc tcgtaagcat cttctttggc tgggttcaag tcgttaacag
4441 ttaatgtctt gctatcgtcg tttaatccca ttaggtactc aattggaact tctaaaaagt
4501 tagctaactt ttgccaaatt gctaaagagt taggtattcc attttccaat ccttttttcga
4561 aattttctaa agttttaaag tcaatgtcgg ttttgacttg tacatcggca agagttaatc
4621 tcttttccaa tctcaatttc tttagtctat tttgcatggt ggttacctcc cgtactctct
4681 aaaaattgcg ttacgttctt cttgcgtcat ttgcggtact gtctgcgact gttgttgctg
4741 aaccttatcc cagtttgtag cccttcgaac tggcttagct tgctgattat atctaggttt
4801 aacaggtgtc atatccagtt catcatcaaa tcggccatta aaccaggtag atccattaag
4861 tgtgtactct agcggtgttt gtttaatttt aagaaactta agataacgct ctagtttttc
4921 tttcattgct tcaaaagtgt tatccttgct acgctttctc cacgtcttat agtgtctaag
4981 agcagccttt ttaccttgtt tcttaggata cattgaccat aattgttcaa attctgactc
5041 atatatttgt gtcggagatt cggaagaatc gactatatta tttatactat ccttacctaa
5101 cctatcctta cctaacctat gcggtccatt tgacgtccat tggttgtcca ctggacgtcc
5161 attaccttca attgctttaa tgccagtctt tttaccagta tcagcccttg cttttgattg
5221 ttgaagttca atatcaggta gcattcttat aagcaagtcc cgataaattg aatcaacttt
5281 cctatcagga cgaatacgat tattttcttt ccagtcattt atataagtaa caagatcagc
5341 atttaaaact gttataagcc ctttagctgc cagaattttt aaatcatctt cagtagagcc
5401 tgtttgtttc attacgttat aaccttcaac aactccatca tcatcagcat gtatccctaa
5461 atgaaaataa agacattgag ttgaaggagg cattttaaga aatcttgcac tttcaacaat
5521 acgttgtgag aacattctac gttgtgccaa ttaattcacc acctaaaata atctatctac
5581 tgtagagtca gcgtcagata ccaccttctt gtcttcaaat ggatctttct tggcttgttg

5641 aatcttatca agaacagctt gtttttcttt tgtctttgaa gtatcagttt tcttatgctt
5701 ctttttaaag atttgtgcta cttgatggat agcttgtgca tcttctggtg tatgtgactc
5761 tttaaaccaa tcctgcgcgt ctttgatacc atctcttgct tcttgatata agtcatacaa
5821 gttagcttga acgccattgt aatacacctg ataattttca agttcctgct tggttaagat
5881 tttaggttca tagatagtct tttgatcttg tacgtcatta tcatcgtctg cagcaattcc
5941 aaaggcacca ctaagtgaat acctttttgc ataactaata agacttgcgg ttgcttgcgc
6001 atcatatgct ttgccgtttt gaaaaatgat cttatttgtt ttgacagtaa agcctgagga
6061 atcaactaga agcgtttgga cggctactgc agtgttggtt gtttgaatat cgaagaaata
6121 actaaaagtt acattacctt ctttatctgt ggctttttta atgccatcca taactgcttt
6181 gtcaatgtcc gctaaatcag catatttata gtcataactg taaggctttc cttgcttatt
6241 tttgcctgaa acagtaacct tatgggttcg tttgggttga ttaatatttg ctttaacctg
6301 agcataatga agtgcccatt tagctctgtc ttcttctttg ccatagattt ccatattagt
6361 ccaccctttc gtagctaatg ccccagtctc gaatgaagtt agacagggca gttagctgtt
6421 ctttagtacc agtaagtttt aaagtaacac tatgtacctt atcaaccact tcgccagtct
6481 tggcatcaac gtacttgtcg ccatgctgtt caagcgcttc aatagccttc ttgcggttct
6541 cttcttgctg tttagcctgc ttaattaaat aatcatggtc attatccatt tgagttagta
6601 cgtcaggcag cgacttataa tcaagcatct gcaaatatgg attagcgctc atagccggct
6661 tagtatattc attagcctta ttagcaataa cttgctcagc ttcttttcta gctttttgct
6721 tttcgacgat cgcttcaaac tgctgtctag cgtcttcttc aatagtggtc caactagtag
6781 tcttgttgtc ccacttatct tggtagtcaa gctcttgtaa ggctacacca tattcttttg
6841 ctaattcgcc taagcgaagt aagttttgct gatgcttgtc tttacgggct tgctcgtcaa
6901 aagtcttaag tccctcacta aggtggtcaa ccacaccctt aatcttcaga tcaaggcctt
6961 ttaccttagt attaaattcg gtaatttgtt gactactttc cttaacgatt actttgcggc
7021 ggtcacttaa agctcgtcta attcggttaa gctctgctct agtcttcttg tcgtaaggat
7081 aagactctgc agttactaca tacttatccc aatccttggt cattgcatca actttggcat
7141 ctaaagctgg atatccttgg aaatctattt ctgcctttgt atagctaata ggaaaatctt
7201 cgttttcaaa tttgattaat tcttttgtca tctttttact ccatattgtt agaattctgc

7261 tagaataaag ttagttgttt tgtagactca ctggttgtac agtgggtctt
ttcttttgct
7321 ctctcaaatg ctaattcgta ttcatcttgt ctttggtcgg tggctaaata
attgatagca
7381 tttgaagcaa gcactaatct ttcttgcaat tttgacacgg tattacctcc
taaatattag
7441 gaaacacaac gttatacatg agtaagtagg ctactgcaat cacagtgaac
gtagcaccta
7501 tggtcataat ttctgtttca cgttcagtaa aactagtgcc taaaaactta
ttgatatttt
7561 ggttaatcca ttttgtaaaa ctcatctttc taacaccttt ctatctctca
taactcctgt
7621 accgaatacc tcttcccaat gctttctcga ttgatctttt aggaaggctt
gccaacgttc
7681 ttcgataatc atggtatgtt ggccgtcata cacaattgca tccttaaatg
gacttaataa
7741 gcacttctta cgccgtttag tatatgcagc atttgaataa tcgtatttct
tgcatagttc
7801 tttaacagtt agccaagttt ccttgctact cactttaaat acccctgctt
tcttaactct
7861 ttgatggtta ataattcacc atcgcaagcc ctttctacta gattttgact
tgctgacatg
7921 tctttgtaga acttatcttc aaaaagaact ggaatgtaga taatacccaa
tgcagttaga
7981 acggttgctt gatcgttaat catgctttcg gtagctggat tccaaacact
agtcgtttta
8041 atacctagcc agcctgtatc cacacccttg ccggttgctt gataattacc
atcaatcaaa
8101 gacagctgct gcagtctagt gatgatttca tgtttacttt ttccgatatc
acttaaatat
8161 aagtaactaa tatctagtgg ttgcatttct tatctcctct caatcaatta
aggtaaacgt
8221 ttaaaaatta actctttagg aatagactta agtcgaattc ttaatccttt
tgccagctcg
8281 tattcttcgt tagtaacaat tgctaatact ttgggattga cctcatcagt
ttcgatgatt
8341 actcttgacc aattattcat aggctgatta tcttcattag ttttcaattc
catttttctt
8401 aaattactgt gttctacttg tctcatgcta tctccttact aaacatcaaa
tgtagttacc
8461 ataacttctt gataaatatc atcgaatttt ccacgtaaag ccttattaat
gatgtgtctc
8521 tgtccttttg gcgttacaaa cacttgtgaa tatggacgat catctcttgt
aagtctgtgg
8581 gttattctga aatatcctct tttaactttg tcagtcattg gtaagttcca
agtacttctt
8641 tgatgtgata aatacttagc ctgtcttaac aattgaaata gttggttacg
gccgatcaca
8701 aagccattct gtgttaaaat gtcggccaat tcagcaatgg taattgcatg
gtggctatat
8761 ctgatagcct ttgcgaatac tacatcgtca gcatctttag cgatcttctc
tgcttgttta
8821 gcgttgacgt cttcaagttc cttattctta ttttcaagta gctttacgcg
actgcttgca

8881 atgtttaatg cacgctgcat aatcatatcc ggattattcc agttcttttc tacttgaata
8941 aagtatttac gaacttcttt cccttttgat gtttttgaca tcatacaaag ttctttggcc
9001 atatcaattg tcaataagta atctgtaatt tctctggtag cgccatttcc aacatgtgca
9061 gttgtaactg catatgtata atctgagcca ttttcaaact ctttaaaatt ttgagacgtc
9121 cactcattaa atctgctttt tagctctaat cctttatata aagatctggc agatacaagt
9181 tgttggtcat cttttacttg aatttttatt aaattattca ttaattacct ctctagtgta
9241 ttttatccgt agtttcatga taaaaaataa tattattcat tggaatatta actaagcgag
9301 caaatttttg agctgtatct actctcatat aagtatttcc attctcatag tctcgatagg
9361 tatttaatcc aattcctaac atatcagcca ttttagcttg actaaaaccg ctatatttac
9421 gtgcttgctt taaatcaaat ttcattattt cacctctttc gtccctcgac acttaatatt
9481 aaactacgga taaaatgtat tgtcaacatt tttatgtata ttttatccat atttttgtgt
9541 taaataactt attttctgtg tataataagc gtagttgata gctataggtg gtgaaatcat
9601 ggataatata ttaggagata acataaaaaa atacagaaaa gctagttctt acactcaaaa
9661 agatttagct aatttactaa aagtaaaacc cactactgtt gcctcctggg agcaaggcag
9721 aaataagcct ttgatggata aagtaacaaa attaaccaat atttttggag tttcccttac
9781 tgatcttgtc ggtagcgatc aagaaaatga agttactgat tacttttcca aaaaaccaag
9841 aatggttcct ctactaggaa ctatagcgat gggagcgcca attactgcag aacagaatat
[truncated: 439,810 more chars]
